# DPP4 Regulates DHCR24-Mediated Cholesterol Biosynthesis to Promote Methotrexate Resistance in Gestational Trophoblastic Neoplastic Cells

**DOI:** 10.3389/fonc.2021.704024

**Published:** 2021-12-02

**Authors:** Weijie Yuan, Wenjing Yong, Jing Zhu, Dazun Shi

**Affiliations:** ^1^ Department of Gastrointestinal Surgery, Xiangya Hospital, Central South University, Changsha, China; ^2^ The Hunan Provincial Key Laboratory of Precision Diagnosis and Treatment for Gastrointestinal Tumor, Changsha, China; ^3^ Department of Obstetrics, Xiangya Hospital, Central South University, Changsha, China; ^4^ Department of Gynecology, Xiangya Hospital, Central South University, Changsha, China

**Keywords:** gestational trophoblastic neoplasia, chemoresistance, methotrexate, DPP4, cholesterol, DHCR24

## Abstract

Metabolic reprogramming could promote cellular adaptation in response to chemotherapeutic drugs in cancer cells. Herein, we aimed to characterize the metabolomic profiles regulated by Dipeptidyl Peptidase 4 (DPP4) in methotrexate (MTX)-resistant gestational trophoblastic neoplastic (GTN) cells. A total of eighty metabolites were found to be commonly altered in DPP4-depleted JAR/MTX and JEG3/MTX cells. Cholesterol biosynthesis-related metabolites were markedly impacted by DPP4 knockdown in MTX-resistant sublines. Manipulation of DPP4 expression remarkably affected the level of cellular cholesterol in GTN cells. Our analysis also identified 24-Dehydrocholesterol Reductase (DHCR24) as a potential downstream effector of DPP4. Manipulation of DHCR24 expression affected cellular cholesterol level, reactive oxygen species (ROS) accumulation, and chemosensitivity to MTX in GTN cell models. In addition, over-expression of DHCR24 could markedly restore cellular cholesterol level and rescue cell survival in DPP4-depleted MTX-resistant GTN cells. Highly correlated expression of DPP4 and DHCR24 was observed in clinical GTN specimens. Further, DPP4 inhibitor sitagliptin effectively inhibited cholesterol biosynthesis, reduced DHCR24 expression and enhanced MTX-induced cytotoxicity *in vitro* and *in vivo*. In conclusion, our findings suggested that DPP4 might regulate DHCR24-mediated cholesterol biosynthesis to promote methotrexate resistance in GTN cells. Targeting DPP4/DHCR24 signaling might help to sensitize MTX-resistant GTN to MTX treatment.

## Introduction

Gestational trophoblastic neoplasia (GTN), including epithelioid trophoblastic tumor, placental site trophoblastic tumor, choriocarcinoma, and invasive mole, is one of the curable cancers due to its intrinsic sensitivity to chemotherapeutic drugs (1). Although methotrexate has been used for the treatment of GTN for decades, a considerable fraction (25%-50%) of low-risk GTN patients fails to respond to single-agent methotrexate (2, 3). In addition, while most patients with GTN could be cured by MTX-containing regimens, there is a subset of patients with high FIGO risk scores of >13 for whom the mortality rate is 38.4% (4). A number of genes have been potentially implicated in the development of MTX resistance in GTN, including dihydrofolate reductase (DHFR) (5), ATP-binding-cassette (ABC) transporter MRP1 (6), CD105 (7), Type I interferons (8), and SLAMF1 (9). Exploring the diverse mechanisms underlying the acquisition of MTX resistance might allow the identification of potential targets for GTN treatment.

Metabolic reprogramming could promote cellular adaption, proliferation, and survival in response to chemotherapeutic drugs in cancer cells (10). Activation of cholesterol biosynthesis pathway has been shown to contribute to the development of chemoresistance in cancer cells by maintaining lipid rafts (11), ABC transporter activity (12), reactive oxygen species (ROS) homeostasis (13–15), and mitochondrial integrity (16). Some cholesterol synthases (CYP51A1, HMGCR, HMGCS1, etc.) have been shown to regulate drug resistance in various cancers. Howell et al. showed that cholesterol synthase CYP51A1 was significantly upregulated in the lapatinib-resistant lung cancer cells; CYP51A1 inhibitor ketoconazole reduced mitochondrial cholesterol and thereby overcome lapatinib resistance (17). In prostate cancer cells, HMGCR was identified as a potential target to enhance the efficacy of enzalutamide-based therapy (18). In lung cancer cells, over-expression of HMGCS1 was shown to be involved in resistance to Abraxane (19). In addition, cholesterol biosynthesis inhibitors including Statins and Bisphosphonates could sensitize cancer cells to anti-cancer drugs (20). However, the role of cholesterol biosynthesis pathway in the development of MTX resistance in GTN still remains largely unknown.

DPP4 is a membrane-bound dipeptidyl peptidase that cleaves various substrates including incretin hormones (GIP, GLP1), cytokines, and growth factors. DPP4 could regulate glucose and lipid metabolism through degrading GIP/GLP1 and thus reducing insulin secretion in islet β cells. Although DPP4 is widely expressed in different cell types, its expression is much higher in adipocytes, fibroblasts and hepatocytes, especially in a variety of disease states including obesity and diabetes (21). Silencing expression of DPP4 in hepatocytes suppresses inflammation of visceral adipose tissue and insulin resistance in obese mice (22). The tumor-promoting role of DPP4 has also been reported, as aberrant expression of DPP4 is frequently observed in hepatocellular, colorectal, and breast cancer (23–25). Recently, we showed that DPP4 is a potential regulator of MTX resistance in GTN cells (26). However, the metabolic function of DPP4 in the development of resistance to MTX in GTN cells still awaits further investigation. In the present study, we aimed to characterize DPP4-regulated metabolomics profiles in MTX-resistant GTN cell models through mass spectrometry-based untargeted metabolomics approach. We showed that DPP4 might regulate DHCR24-mediated cholesterol biosynthesis to promote methotrexate resistance in GTN cells, which may provide novel insights into the molecular mechanisms of drug resistance in GTN.

## Material and Methods

### Reagents and Cell Lines

Primary antibodies were provided by following sources: DPP4, DHCR24, BrdU, and β-Actin (Abcam, Cambridge, MA, USA); Methotrexate (MTX) and DPP4 inhibitor Sitagliptin were obtained from Selleckchem (Houston, TX, USA). pCMV3 plasmids encoding empty vector (EV) or DCHR24 were obtained from Sinobiological Inc. (Beijing, China). Lentiviral shRNA plasmids encoding scramble control, shDHCR24, and shDPP4 were provided by Genecopoeia Inc. Human GTN cell lines JEG3 JAR, and BEWO were provided by American Type Culture Collection (Manassas, VA, USA). These GTN cell lines were cultured in Dulbecco’s modified Eagle’s medium supplemented with 10% fetal bovine serum (Hyclone, Logan, UT), 4 mM glutamine, 100 U/mL penicillin, and 100 µg/mL streptomycin.

### Establishment of MTX-Resistant GTN Sublines

The MTX-resistant GTN sublines JEG3/MTX and JAR/MTX were generated by exposing parental cell lines (JEG3, JAR) to stepwise elevated concentrations of MTX as we previously described (26, 27). Briefly, JEG3 and JAR were plated at 3 × 10^6^ cells per 100 mm culture dish and incubated overnight, then were induced by MTX starting from low concentration (10% IC_50_, 0.1 μg/ml). After incubation with MTX for 48 hours, we discarded the MTX-containing medium and continued to culture without MTX until the cells exhibited normal growth characteristics and cell density reached ~70%. These GTN cells were then passaged and the above operation was repeated. According to the status of cell growth, we gradually increased the drug concentration of intermittent induction (ultimately 20 μg/ml). The MTX-resistant JEG3 and JAR sublines (JEG3/MTX, JAR/MTX) were obtained after 20 rounds of intermittent and repeated exposure to MTX in 8 months. All the drug resistance assays were performed after these sublines had been grown in a drug-free medium for 1 month.

### Untargeted Metabolomics Analysis

GTN cells (1×10^7^ cells) were extracted using pre-cooled methanol and the resulting extract was analyzed by LC-MS/MS analysis (positive and negative modes) as described previously (28). Peak extraction, alignment and retention time correction were performed by XCMS program. The identification information of metabolites was obtained by searching the laboratory’s self-built database and integrating the public database and metDNA. The identified metabolites were subjected to further statistical analysis to screen for metabolites affected by DPP4 knockdown in MTX-resistant GTN sublines. The selection criteria for differentially changed metabolites were set as described by Lu et al. (29). Briefly, partial least-squares discriminant analysis (PLS-DA) was performed to investigate the metabolic changes and screen for potential metabolites (VIP value). The unpaired student t test with Welch’s correction was applied to determine the significance of each metabolite. Differentially changed metabolites were selected with the following criteria: VIP value > 1, │Log_2_(FC)│ ≥ 1 and P < 0.05.

### KEGG Pathway Analysis

MetaboAnalyst 5.0 was used to analyze KEGG pathway enrichment of DPP4-regulated metabolites (https://www.metaboanalyst.ca/) (30). P-values are calculated based on accumulative hypergeometric distribution.

### Cholesterol Measurement

Amplex red cholesterol assay kit (Invitrogen) was used to analyze the cellular cholesterol level (31). Briefly, GTN cells (10^5^ cells) were lysed and protein concentration was determined and diluted to 1 μg/μl for further experiments. The lipids were extracted with a mixture of chloroform/isopropanol, and were resuspended in reaction buffer and cholesterol was measured according to user’s manual. Hydrogen peroxide (H_2_O_2_) was used as a positive control.

### Filipin Fluorescence Staining of Free Cholesterol

Filipin fluorescence staining was conducted on GTN cells according to the manufacturer’s instructions (Abcam). Briefly, GTN cells were seeded onto coverslips in 24-well plates (5×10^4^ cells/well) and grown for 48 hours. U18666A was used as a positive control. GTN cells were then fixed with 4% paraformaldehyde for 30 min at room temperature, following by PBS washes and incubation with Filipin solution (50 μg/ml) for 1 h at room temperature. The coverslips were washed and mounted onto a slide. The Filipin-cholesterol staining was visualized using an Olympus BX43 fluorescence microscope (excitation 340 nm; emission 430 nm).

### BrdU Incorporation Assay

GTN cells were seeded onto coverslips in 24-well plates (2×10^4^ cells/well) and incubated overnight. After MTX treatment (10 μM for MTX-resistant sublines; 3 μM for parental cell lines) for 96 hours, GTN cells were labeled with BrdU (25 μg/ml) for 1 hour. Cells were then fixed and the DNA was denatured with 2N HCL solution. BrdU mouse antibody (dilution 1:200) was added to detect the incorporated BrdU. After incubation with Alexa 488-labeled secondary antibody, the coverslips were washed and mounted onto a slide. The incorporated BrdU was visualized using an Olympus BX43 fluorescence microscope. The percentage of BrdU-labelled cells in each section was determined in four randomly selected fields (10× objective). Data are presented as mean ± SD from four independent experiments.

### TUNEL Assay

Terminal deoxynucleotidyl transferase-mediated deoxyuridine triphosphate nick end labeling (TUNEL) assay was conducted to assess the level of apoptosis in GTN cells. Briefly, GTN cells were seeded onto coverslips in 24-well plates (2×10^4^ cells/well) and incubated overnight before addition of MTX. After MTX treatment (10 μM for MTX-resistant sublines; 3 μM for parental cell lines) for 96 hours, GTN cells grown on coverslips were fixed with 4% paraformaldehyde and then permeabilized with 0.1% Triton-X 100 in PBS. After being washed by PBS, cells were incubated with reaction mixture for 60 min at 37°C. Coverslips were then mounted and analyzed under an Olympus BX43 fluorescent microscope. The percentage of cells identified by TUNEL in each section was determined in four randomly selected fields (10× objective). Data are presented as mean ± SD from four independent experiments.

### DPP4 Enzymatic Activity Assay

DPP4 Activity Assay Kit (Abcam) was used to analyze the cellular/tissue DPP4 enzymatic activity. Briefly, GTN cells were seeded into 100 mm culture dishes (2×10^6^ cells/dish) and incubated overnight. After treatment with various concentrations of sitagliptin (100, 250, 500, 1000 nM) for 24 hours, GTN cells were exacted with DPP4 assay buffer and were centrifuged to remove any insoluble material. Supernatant was collected and diluted, mixed with substrate (H-Gly-Pro-AMC) buffer, and then incubated at 37°C for 30 min. The purified DPP4 was used as a positive control. The release of AMC was measured with An F97Pro fluorospectrometer (excitation: 360 nm; emission: 460 nm).

### RNA Sequencing

The mRNA was extracted from MTX-resistant GTN cells with or without DPP4 knockdown. The mRNA libraries were then constructed and RNA sequencing was performed on an Illumina NovaSeq6000 platform (HaploX, Shenzhen, China). The expression level of protein-coding genes was calculated as Fragments Per Kilobase of exon model per Million mapped fragments (FPKM) value.

### Immunohistochemistry (IHC)

Archival paraffin-embedded tissues of patients diagnosed with GTN between 2010 and 2020 were used for IHC analysis. MTX-sensitive GTN (n=12, chemonaive patients underwent abdominal surgery*/*hysterectomy and diagnosed with GTN*;* these patients achieved complete remission after treated with single-agent MTX). MTX-resistant GTN tissues (n=23) were obtained by surgery from patients who developed resistance to single-agent or combined MTX regimens. IHC procedure was conducted as we described previously (26). The immunostaining with tumor cell positivity ≥30% was regarded as high expression (26).

### Animal Studies

Immunocompromised nude mice were obtained from the breeding facility at the animal center of Central South University. All animal studies were performed in accordance with institutional ethical guidelines for experimental animal care. For xenograft study, JAR/MTX cells (1×10^6^) were subcutaneously inoculated into the flank of each mouse. Inoculated tumors were allowed to establish for 11 days (tumor volume ~50mm^3^) before initiation of treatments (n= 4). Sitagliptin solution (100 mg/kg per day for 9 days) was administered by oral gavage (32). MTX was administered at a dose of 5 mg/kg every other day until day 20 by intraperitoneal injection (33). Phosphate buffered saline (PBS) was used as negative control. In order to determine tumor volume by external caliper, the greatest longitudinal diameter (a) and the greatest transverse diameter (b) were determined. Tumor volume based on caliper measurements was calculated by the modified ellipsoidal formula: tumor volume (mm^3^) = a×b^2^/2. Mice were sacrificed 20 days after cell inoculation, and the subcutaneous xenografts were removed, washed by PBS, and weighted. The expression of DHCR24 and Ki-67 in xenograft tissues was evaluated by immunohistochemistry. The immunopositivity of Ki-67 was calculated from 6 randomly selected fields (10× objective) for each section. Data are presented as mean ± SD from four mice for each experimental group.

### Gene Expression Omnibus (GEO) Datasets

GEO expression dataset GSE75328 (Transcriptional profiling of primary human white preadipocytes in response to knockdown of DPP4) were downloaded from National Center for Biotechnology Information (NCBI) website (https://www.ncbi.nlm.nih.gov/geo/query/acc.cgi?acc=GSE75328) (34).

### Statistical Analysis

SPSS 16.0 (SPSS Inc, Chicago, IL, USA) was used for statistical analysis. Error bars throughout the figures indicate standard deviation. The Student’s t test (two tailed unpaired) was used to compare means of two groups. Differences among more than two groups were analyzed using one-way ANOVA or two-way ANOVA followed by Dunn’s or Tukey’s multiple comparisons test. Fisher’s exact test was used to analyze the association between DPP4 expression and MTX resistance in GTN specimens. P<0.05 was considered significant in all of the tests.

### Supplementary Methods

Experimental procedures regarding soft agar assay, cell viability analysis, reactive oxygen species (ROS) measurement, cell cycle analysis, and western blotting could be found in supplementary files.

## Results

### DPP4 Regulates Cholesterol Biosynthesis in MTX-Resistant GTN Cells

**Figure 1:**
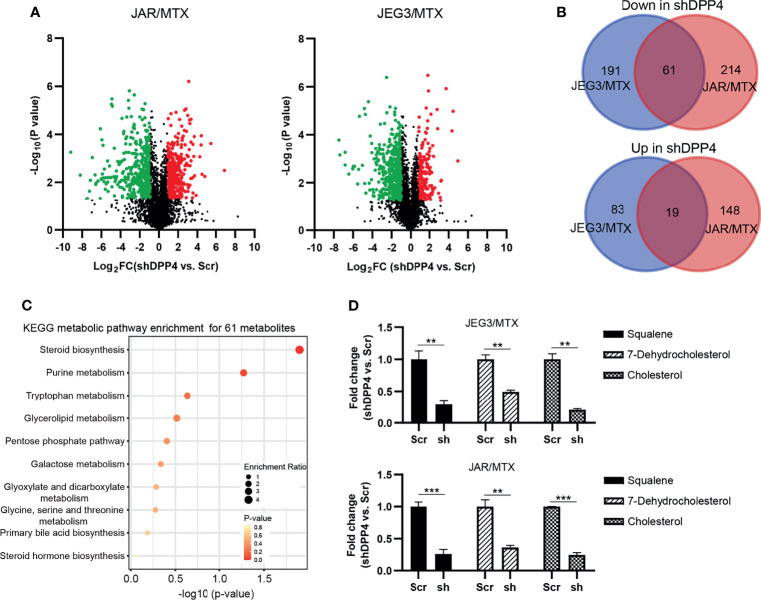
DPP4 is a metabolic regulator of cholesterol biosynthesis in GTN cells. **(A)** Volcano plots showing the differential alteration of metabolites associated with DPP4 knockdown in MTX-resistant GTN sublines. **(B)** Venn diagram of up- or down-regulated metabolites associated with DPP4 knockdown across MTX-resistant GTN sublines. **(C)** KEGG analysis on 61 metabolites was conducted to show the enrichment of metabolic pathways. The top 10 pathways that arise with low p-values and with high enrichment ratio are indicated in the figure. **(D)** Metabolites involved in steroid biosynthesis were greatly impacted by DPP4 knockdown. n=3, **P<0.01; ***P<0.001.

In our previous study, we established MTX-resistant JEG3/MTX and JAR/MTX sublines in order to identify potential regulators associated with MTX resistance in GTN cells (16). Higher IC_50_ values were observed in these sublines than in parental cell lines (**Supplementary Figure S1A**). The colony formation of these MTX-resistant sublines was much higher than that of the parental cell lines after MTX treatment (**Supplementary Figure S1B**). Hence, untargeted metabolomics analysis was conducted on these MTX-resistant GTN sublines (JAR/MTX, JEG3/MTX) with or without DPP4 knockdown (three biological replicates for each group). Collectively, we detected 1965/2570 unique metabolites under negative/positive mode across all these samples. Selecting criteria (VIP value>1, shDPP4 vs. Scr, P<0.05, │Log2FC│≥1) was set to identify differentially altered metabolites following DPP4 depletion in JAR/MTX and JEG3/MTX cells. A number of 354 and 442 metabolites were significantly changed following DPP4 knockdown in JEG3/MTX and JAR/MTX cells, respectively (**Figure 1A** and **Tables S1, S2**). A total of eighty metabolites were commonly changed in both of JAR/MTX and JEG3/MTX cells following DPP4 knockdown, with 61 down-regulated and 19 up-regulated metabolites in shDPP4 group, respectively (**Figure 1B**). KEGG metabolic pathway analysis showed that metabolites associated with steroid biosynthesis were significantly enriched for these 61 down-regulated metabolites (P=0.0127; **Figure 1C**). Consistently, the level of the metabolites involved in steroid/cholesterol biosynthesis (Squalene, Cholesterol, and 7-dehydrocholesterol) was reduced in shDPP4 group compared to Scr control in JAR/MTX and JEG3/MTX cells (**Figure 1D**). There were no significant changes in triglyceride or fatty acid levels following DPP4 knockdown in MTX-resistant GTN cells.

### DPP4 Might Regulate Cholesterol Biosynthesis Through Up-Regulating DHCR24 Expression in GTN Cells

**Figure 2:**
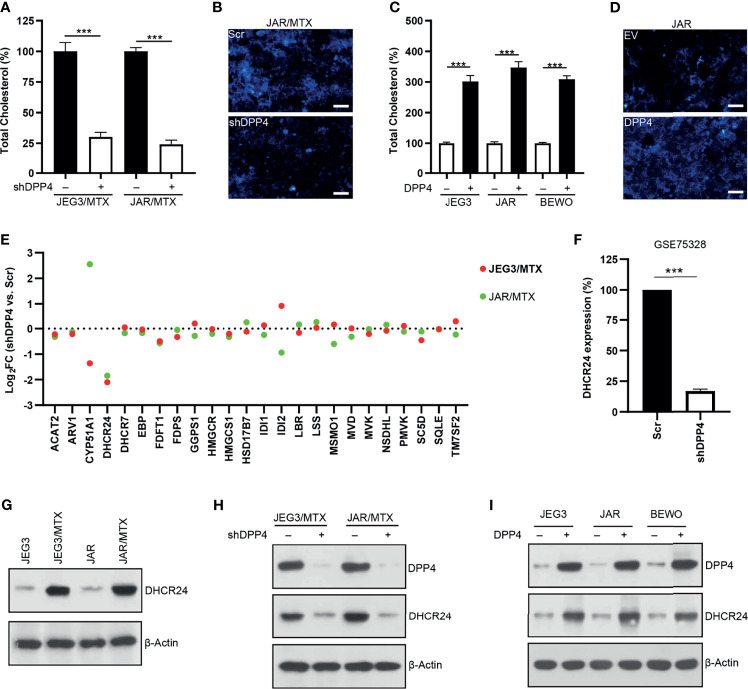
DPP4 might regulate cholesterol biosynthesis through up-regulating DHCR24 expression in GTN cells. **(A)** Knockdown of DPP4 reduced the level of cellular cholesterol in JAR/MTX and JEG3/MTX cells. n=4, ***P<0.001. **(B)** Knockdown of DPP4 reduced Filipin-cholesterol fluorescence in JAR/MTX cells. Bars: 50 μm. **(C)** Over-expression of DPP4 increased cellular cholesterol level in JAR, JEG3 and BEWO cells. n=4, ***P<0.001. **(D)** Overexpression of DPP4 increased Filipin-cholesterol fluorescence in JAR cells. Bars: 50 μm. **(E)** Altered expression of cholesterol biosynthesis-related enzymes following DPP4 knockdown in MTX-resistant GTN sublines. **(F)** Analysis on GSE75328 revealed dramatic decline of DHCR24 expression following DPP4 knockdown in primary human preadipocytes. n=4, ***P<0.001. **(G)** Expression of DHCR24 in parental GTN cells and their MTX-resistant sublines. **(H)** Knockdown of DPP4 reduced the expression of DHCR24 in JAR/MTX and JEG3/MTX cells. **(I)** Over-expression of DPP4 increased DHCR24 expression in JAR, JEG3 and BEWO cells.

Amplex red cholesterol assay was conducted to measure the amount of total cholesterol in GTN cells. The level of cellular cholesterol in JAR/MTX and JEG3/MTX cells was greatly elevated compared to parental JAR and JEG3 cells (**Supplementary Figure S2**). The level of cellular cholesterol was markedly reduced following DPP4 Knockdown in JAR/MTX and JEG3/MTX cells (**Figure 2A**). Filipin fluorescence staining showed that knockdown of DPP4 attenuated Filipin-cholesterol staining intensity in JAR/MTX cells (**Figure 2B**). In addition, over-expression of DPP4 increased cellular cholesterol level in JAR, JEG3 and BEWO cells (**Figure 2C**). Consistently, Filipin-cholesterol staining intensity was greatly increased in JAR cells with over-expression of DPP4 (**Figure 2D**). Therefore, DPP4 might regulate cholesterol biosynthesis in GTN cells.

RNA sequencing was conducted to analyze the expression of cholesterol biosynthesis-related enzymes in MTX-resistant GTN cells with or without DPP4 knockdown. 24-dehydrocholesterol reductase (DHCR24), one of the twenty-four cholesterol biosynthesis-related enzymes, was found to be markedly down-regulated in JAR/MTX and JEG3/MTX cells following DPP4 knockdown (**Figure 2E** and **Tables S3, S4**). Similarly, analysis on GEO dataset GSE75328 showed that DHCR24 mRNA expression was also reduced in primary human white preadipocytes following DPP4 knockdown (**Figure 2F**). The protein expression of DHCR24 in GTN cells was analyzed by Western blotting. JAR/MTX and JEG3/MTX cells exhibited remarkably higher DHCR24 expression than parental JAR and JEG3 cells, respectively (**Figure 2G**). Knockdown of DPP4 reduced the expression of DHCR24 in JAR/MTX and JEG3/MTX cells (**Figure 2H**). In addition, transfection of DPP4 plasmid increased DHCR24 expression compared to EV group in JAR, JEG3 and BEWO cells (**Figure 2I**). These findings suggested that DPP4 might regulate cholesterol biosynthesis through up-regulating DHCR24.

### Knockdown of DHCR24 Sensitizes MTX-Resistant GTN Sublines to MTX Treatment

**Figure 3:**
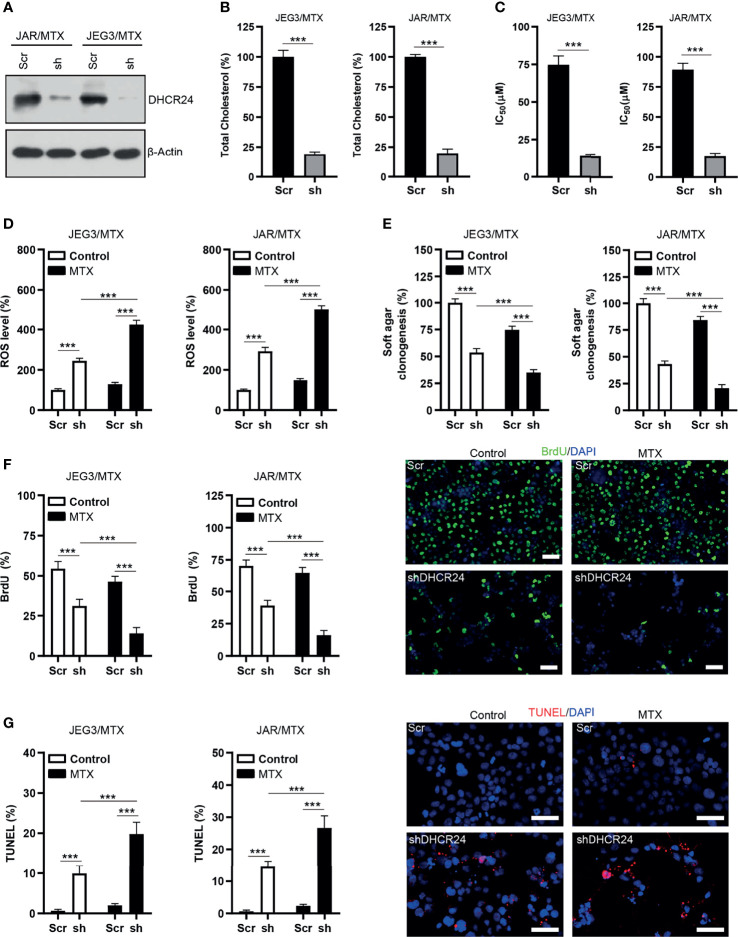
Knockdown of DHCR24 expression influences cellular cholesterol level, ROS accumulation and chemoresistance in MTX-resistant GTN cells. **(A)** DHCR24 expression was effectively attenuated by shRNA lentiviruses in JAR/MTX and JEG3/MTX cells. **(B)** Knockdown of DHCR24 reduced cellular cholesterol level in MTX-resistant GTN sublines. n=4, ***P<0.001. **(C)** Knockdown of DHCR24 increased the chemosensitivity to MTX in JEG3/MTX and JAR/MTX cells. n=4, *P<0.001. **(D)** Knockdown of DHCR24 increased the accumulation of ROS after MTX treatment (10 μM) for 96 hours in JEG3/MTX and JAR/MTX cells. The ROS level in Scr without MTX treatment was regarded as 100%. n=4, ***P<0.001. **(E)** Knockdown of DHCR24 impaired clonogenesis after MTX treatment (10 μM) in JEG3/MTX and JAR/MTX cells. The colony formation in Scr group without MTX treatment was regarded as 100%. n=4, ***P<0.001. **(F)** Knockdown of DHCR24 reduced BrdU incorporation after MTX treatment (10 μM) for 96 hours in JEG3/MTX and JAR/MTX cells. n=4, ***P<0.001. Micrographs showed the result of JAR/MTX cells. Bars: 100 μm. **(G)** Knockdown of DHCR24 increased TUNEL-labeled cells after MTX treatment (10 μM) for 96 hours in JEG3/MTX and JAR/MTX cells. n=4, ***P<0.001. Micrographs showed the result of JAR/MTX cells. Bars: 100 μm.

The protein expression of DHCR24 was remarkably reduced by DHCR24-specific shRNAs in JAR/MTX and JEG3/MTX cells **(** **Figure 3A**). The level of cellular cholesterol was greatly reduced by DHCR24 knockdown in JAR/MTX and JEG3/MTX cells (**Figure 3B**). The cytotoxic effect of MTX on DHCR24-depleted JAR/MTX and JEG3/MTX cells was evaluated by CCK8 assay. DHCR24 knockdown increased chemosensitivity to MTX in JAR/MTX and JEG3/MTX cells (**Figure 3C**). Knockdown of DHCR24 markedly increased ROS accumulation in JAR/MTX and JEG3/MTX cells (**Figure 3D**). Compared with Scr group, increased ROS level were shown in shDHCR24 group after MTX treatment (**Figure 3D**). Moreover, soft agar clonogenesis and BrdU incorporation of DHCR24-depleted GTN cells decreased compared to Scr control (**Figures 3E, F**). DHCR24 knockdown further reduced soft agar clonogenesis and BrdU incorporation compared to Scr control after MTX treatment (**Figures 3E, F**). Further, knockdown of DHCR24 considerably increased TUNEL labeling in JAR/MTX and JEG3/MTX cells (**Figure 3G**). Compared with Scr group, increased TUNEL labeling was shown in shDHCR24 group after MTX treatment (**Figure 3G**).

### Over-Expression of DHCR24 Promotes MTX Resistance in MTX-Sensitive GTN Cells

**Figure 4:**
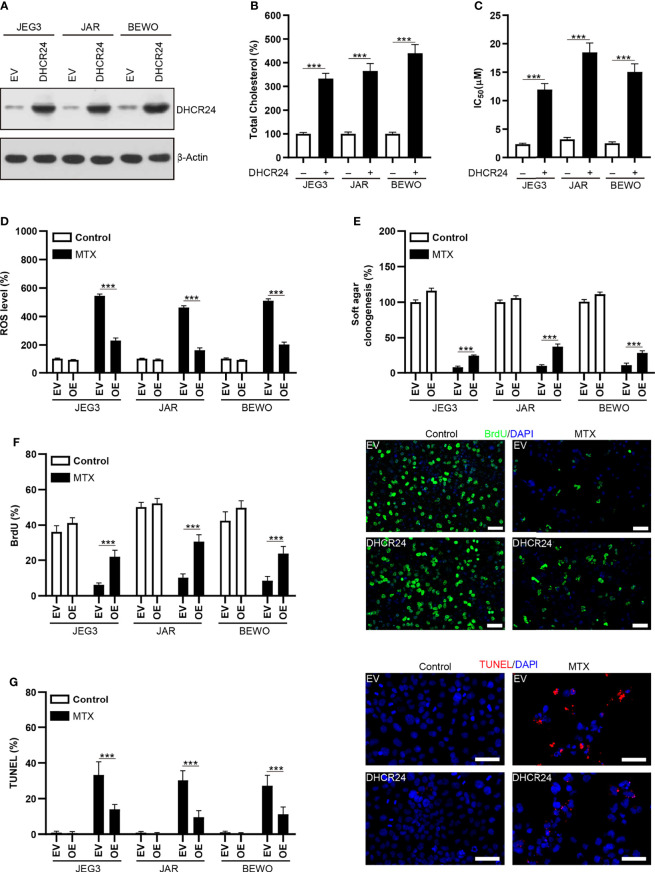
Over-expression of DHCR24 expression influences cellular cholesterol level, ROS accumulation and chemosensitivity to MTX in GTN cells. **(A)** Overexpression of DHCR24 in GTN cell lines. **(B)** Overexpression of DHCR24 increased cellular cholesterol level in GTN cells. n=4, ***P<0.001. **(C)** Over-expression of DHCR24 increased the chemoresistance to MTX in GTN cell lines. n=4, ***P<0.001. **(D)** Over-expression of DHCR24 attenuated ROS accumulation following MTX treatment (3 μM) in GTN cells. The ROS level or caspase-3 activity in EV of each cell line without MTX treatment was regarded as 100%, respectively. n=4, ***P<0.001. **(E)** Over-expression of DHCR24 rescued soft agar clonogenesis after MTX treatment (3 μM) in GTN cell lines. The soft agar colony in EV of each cell line without MTX treatment was regarded as 100%, respectively. n=4, ***P<0.001. **(F)** Over-expression of DHCR24 increased BrdU incorporation after MTX treatment (3 μM) for 96 hours in JEG3, JAR, and BEWO cells. n=4, ***P<0.001. Micrographs showed the result of JAR cells. Bars: 100 μm. **(G)** Over-expression of DHCR24 reduced TUNEL-labeled cells after MTX treatment (3 μM) for 96 hours in JEG3, JAR, and BEWO cells. n=4, ***P<0.001. Micrographs showed the result of JAR. Bars: 100 μm.

DHCR24 plasmid was transfected into MTX-sensitive JAR, JEG3 and BEWO cells to assess the prosurvival effect of DHCR24 in GTN cells. The level of DHCR24 was greatly increased compared to empty vector (EV) control in these GTN cell lines (**Figure 4A**). Over-expression of DHCR24 increased cellular cholesterol level in JAR, JEG3 and BEWO cells (**Figure 4B**). The cytotoxic effect of MTX on DHCR24-expressing GTN cells was evaluated in JAR JEG3, and BEWO cells. DHCR24 group exhibited markedly higher IC_50_ value than EV group after MTX treatment (**Figure 4C**). In addition, DHCR24 group exhibited less ROS level after MTX treatment EV group in these GTN cells (**Figure 4D**). Over-expression of DHCR24 also significantly promoted soft agar clonogenesis and BrdU incorporation compared with EV after MTX treatment in JAR BEWO, and JEG3 cells (**Figures 4E, F**). Similar with BrdU analysis, cell cycle analysis showed that MTX treatment strongly reduced the proportion of JAR cells in the S and G2/M phases in the EV group. However, overexpression of DHCR24 increased the percentage of JAR cells in the S and G2/M phases after MTX treatment, as compared with EV (**Supplementary Figure S3**). Further, Compared with EV group, reduced TUNEL labeling was observed in DHCR24 group after MTX treatment in JAR JEG3, and BEWO cells (**Figure 4G**). These results suggested that over-expression of DHCR24 could promote chemoresistance to MTX in MTX-sensitive GTN cells.

### Over-Expression of DHCR24 Rescues the Effect of DPP4 Knockdown on MTX- Resistant GTN Cells

**Figure 5:**
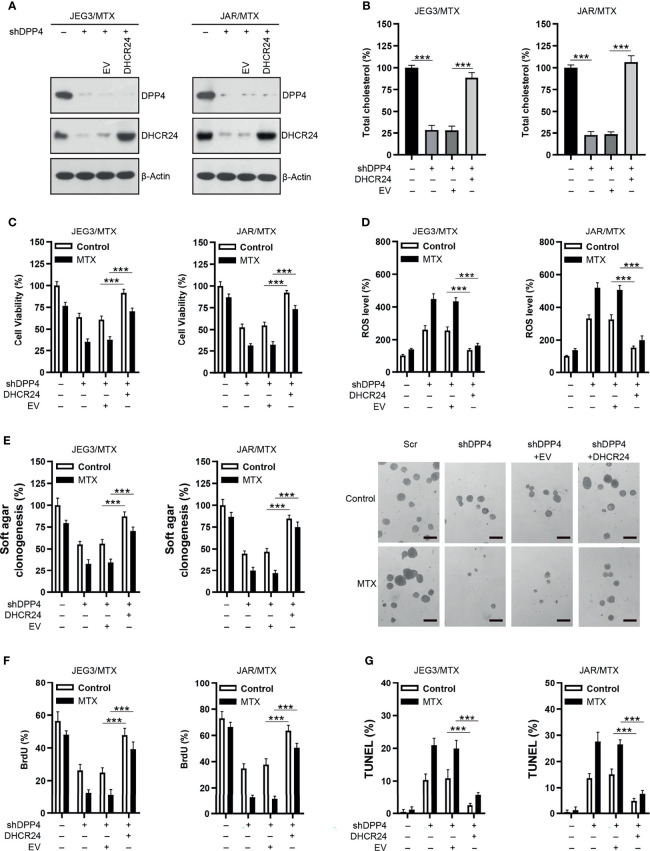
Over-expression of DHCR24 rescues the effect of DPP4 knockdown on MTX-resistant GTN cells. **(A)** Over-expression of DHCR24 in DPP4-depleted JEG3/MTX and JAR/MTX cells. **(B)** DHCR24 expression effectively restored cellular cholesterol level in DPP4-depleted JEG3/MTX and JAR/MTX cells. The cellular cholesterol level in Scr was regarded as 100%. n=4, ***P<0.001. **(C)** DHCR24 expression promoted MTX resistance in DPP4-depleted JEG3/MTX and JAR/MTX cells. n=4, ***P<0.001. **(D)** DHCR24 expression attenuated ROS accumulation and caspase-3 activity following MTX treatment in DPP4-depleted JEG3/MTX and JAR/MTX cells. The ROS level in Scr without MTX treatment was regarded as 100%. n=4, ***P<0.001. **(E)** DHCR24 expression promoted soft agar clonogenesis following MTX treatment in DPP4-depleted JEG3/MTX and JAR/MTX cells. The colony formation in Scr without MTX treatment was regarded as 100%. n=4, ***P<0.001. Micrographs showed the result of JAR/MTX cells. Bars: 200 μm. **(F)**, DHCR24 expression promoted BrdU incorporation following MTX treatment in DPP4-depleted JEG3/MTX and JAR/MTX cells. n=4, ***P<0.001. **(G)** DHCR24 expression reduced TUNEL labeling following MTX treatment in DPP4-depleted JEG3/MTX and JAR/MTX cells. n=4, ***P<0.001.

Rescue experiments were conducted in order to confirm the role of DHCR24 as an important downstream effector of DPP4 in regulating MTX resistance in GTN cells. DHCR24 expression was markedly increased following transfection of DHCR24 plasmid into DPP4-depleted JAR/MTX and JEG3/MTX cells (**Figure 5A**). Cellular cholesterol level was effectively restored by DHCR24 expression in DPP4-depleted JAR/MTX and JEG3/MTX cells (**Figure 5B**). The cellular function of DHCR24 was analyzed in DPP4-depleted JAR/MTX and JEG3/MTX cells. Higher cell viability was seen in DHCR24 group than that in EV group without or with MTX treatment in DPP4-depleted JAR/MTX and JEG3/MTX cells (**Figure 5C**). Additionally, ROS accumulation induced by DPP4 knockdown was significantly reduced by DHCR24 compared to EV control without or with MTX treatment in MTX-resistant GTN cells (**Figure 5D**). Moreover, compared with EV control, overexpression of DPP4 rescued soft agar clonogenesis and BrdU incorporation attenuated by DPP4 knockdown without or with MTX treatment in MTX-resistant GTN cells (**Figures 5E, F**). Further, Compared with EV group, reduced TUNEL labeling was observed in DHCR24 group without or with MTX treatment in DPP4-depleted MTX-resistant GTN cells (**Figure 5G**).

### DPP4/DHCR24 Signaling Is Highly Activated in MTX-Resistant GTN Specimens

**Figure 6:**
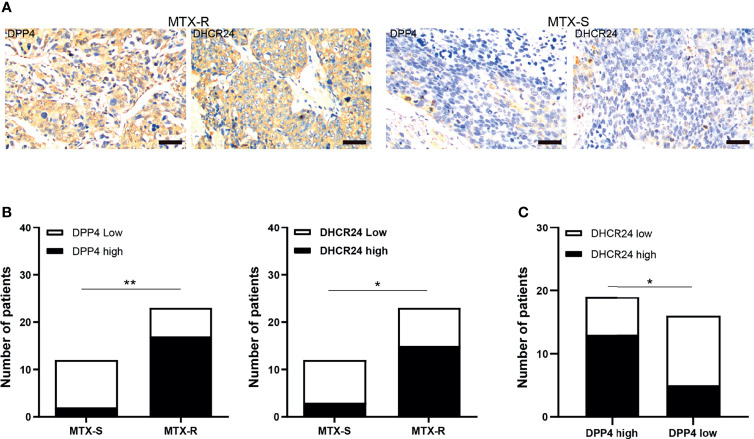
DPP4/DHCR24 signaling is highly activated in MTX-resistant GTN specimens. **(A)** Immunostaining of DPP4 and DHCR24 in clinical GTN samples. Representative IHC staining micrographs were shown for MTX-resistant (left panel) or MTX-sensitive (right panel) GTNs. Bars: 100 µm. **(B)** DPP4 and DHCR24 were highly expressed in MTX-resistant clinical GTN samples. *P<0.05, as compared with MTX-sensitive GTNs. **(C)** High expression of DPP4 was significantly correlated with high expression of DHCR24 in GTNs. *P < 0.05, **P < 0.01, as compared with DPP4-high GTNs.

The expression of DPP4 and DHCR24 in GTN specimens was evaluated by IHC. MTX-resistant GTNs (MTX-R) exhibited higher expression of DPP4 and DHCR24 than MTX-sensitive GTNs (MTX-S) (**Figures 6A, B**). The expression of DPP4 was also highly correlated with that of DHCR24 in these GTN specimens (**Figure 6C**).

### DPP4 Inhibitor Sitagliptin Potently Reduces DHCR24 Expression and Attenuated MTX Resistance in GTN Cells

**Figure 7:**
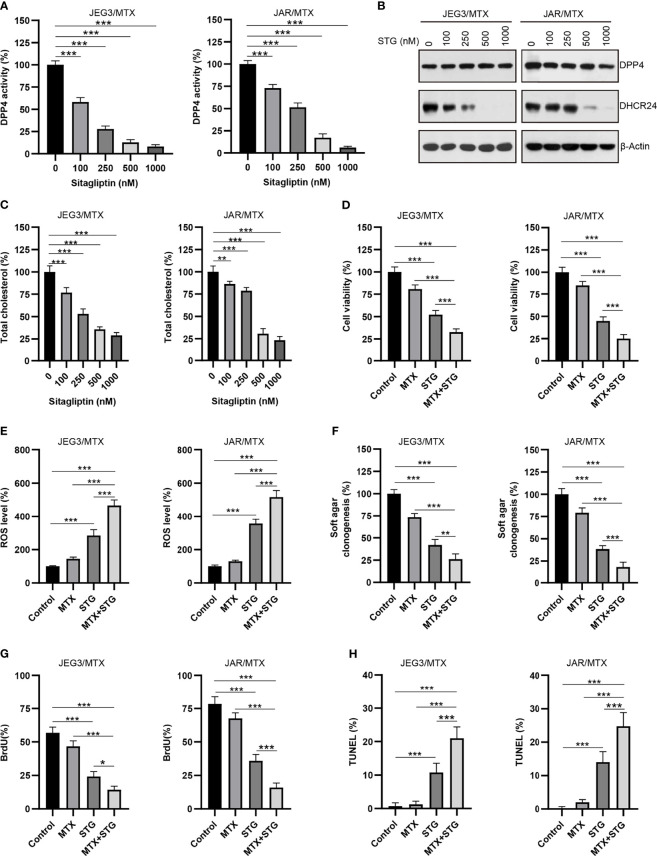
The effect of DPP4 inhibitor sitagliptin on DHCR24 expression, cellular cholesterol level and MTX chemosensitivity in MTX-resistant GTN sublines. **(A)** Dose effect of sitagliptin on DPP4 enzymatic activity in JEG3/MTX and JAR/MTX cells. Cells were treated with vehicle control (DMSO) or escalating doses of sitagliptin for 24 hours. The DPP4 enzymatic activity in vehicle control was regarded as 100%. n=4, ***P<0.001. **(B)** Dose effect of sitagliptin (STG) on DHCR24 expression in JEG3/MTX and JAR/MTX cells. **(C)** Dose effect of sitagliptin on cellular cholesterol level in JEG3/MTX and JAR/MTX cells. The cellular cholesterol level in vehicle control was regarded as 100%. n=4, **P<0.01; ***P<0.001. **(D)** Sitagliptin alone or combined with MTX reduced cell viability in JEG3/MTX and JAR/MTX cells. Cells were treated with sitagliptin alone (1 μM) or in combination with MTX (10 μM) for 96 hours. The cell viability in vehicle control was regarded as 100%. n=4, ***P<0.001. **(E)** Sitagliptin alone or combined with MTX increased ROS accumulation in JEG3/MTX and JAR/MTX cells. The ROS level in vehicle control without MTX treatment was regarded as 100%. n=4, ***P<0.001. **(F)** Sitagliptin alone or combined with MTX impaired clonogenesis in JEG3/MTX and JAR/MTX cells. The colony formation in vehicle control was regarded as 100%. n=4, **P<0.01; ***P<0.001. **(G)** Sitagliptin alone or combined with MTX reduced BrdU incorporation in JEG3/MTX and JAR/MTX cells. n=4, *P<0.05; ***P<0.001. **(H)** Sitagliptin alone or combined with MTX increased TUNEL labeling in JEG3/MTX and JAR/MTX cells. n=4, ***P<0.001.

Sitagliptin (STG), a classical DPP4 inhibitor, has been widely used for type 2 diabetes mellitus treatment (35). Plasma concentration of sitagliptin at 0.1~1 μM could be clinically achievable after administration of single oral dose of sitagliptin (200 mg) (36). As shown in **Figure 7A**, the DPP4 enzymatic activity was considerably diminished after sitagliptin treatment (0.1~1 μM) in JAR/MTX and JEG3/MTX cells. Sitagliptin treatment at 0.5 and 1 μM could significantly reduce DHCR24 expression and cellular cholesterol level in JAR/MTX and JEG3/MTX cells (**Figures 7B, C**). The cytotoxic effect of sitagliptin on GTN cells was also evaluated. Sitagliptin exhibited mild inhibitory effect on the cell viability of JEG3 and JAR cells compared to their MTX-resistant sublines (**Supplementary Figure S4**). Co-treatment with sitagliptin increased MTX-induced cytotoxicity compared to MTX or sitagliptin alone in JAR/MTX and JEG3/MTX cells (**Figure 7D**). In addition, sitagliptin combined with MTX considerably induced ROS accumulation, inhibited soft agar clonogenesis, attenuated BrdU incorporation, and increased TUNEL labeling compared with either drug alone in JAR/MTX and JEG3/MTX cells (**Figures 7E–H**).

### Sitagliptin Reduces DHCR24 Expression and Attenuates MTX Resistance in JAR/MTX Xenograft Model

**Figure 8:**
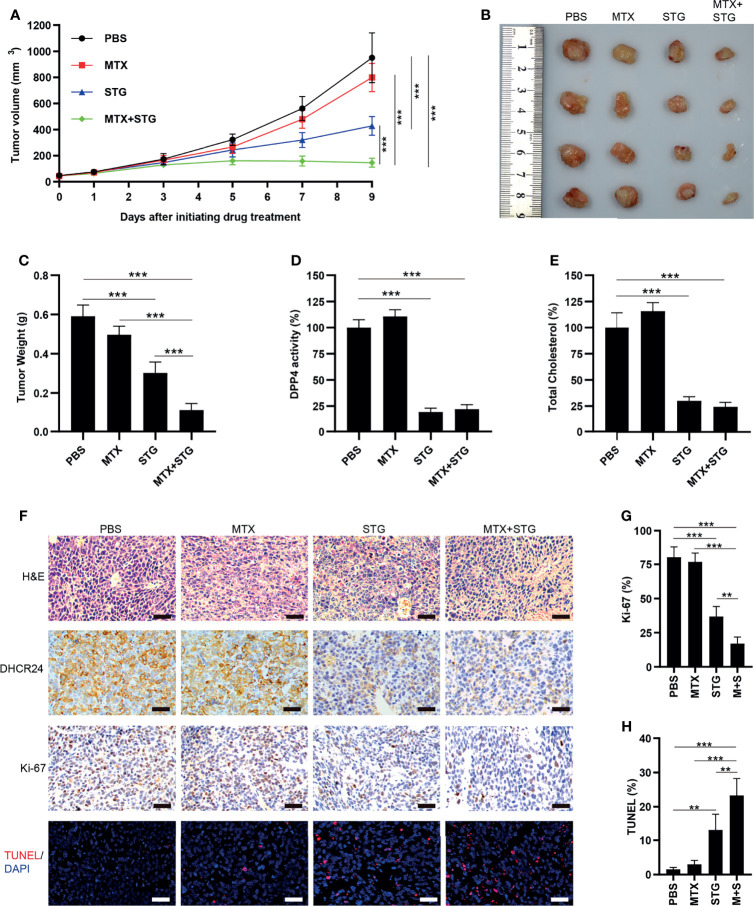
The effect of sitagliptin on cholesterol biosynthesis, DHCR24 expression and tumor growth in JAR/MTX xenograft model. **(A)** Sitagliptin alone or sitagliptin +MTX effectively suppressed the growth of subcutaneous JAR/MTX xenografts *in vivo*. n=4, ***P<0.001. **(B, C)** Sitagliptin alone or sitagliptin+MTX markedly reduced the weight of JAR/MTX xenografts. n=4, ***P<0.001. **(D, E)** Sitagliptin alone or sitagliptin+MTX markedly reduced DPP4 enzymatic activity and cholesterol level in JAR/MTX xenograft tissues. ***P<0.001. **(F)** Immunostaining of the JAR/MTX xenografts after drug treatment. The tissue sections were immunostained with primary antibodies (DHCR24 and Ki-67) or labeled by TUNEL. Bars: 100 μm. **(G, H)** The positivity of Ki-67 and TUNEL labeling in each group. n=4, **P<0.01; ***P<0.001.

The effect of sitagliptin was further evaluated in JAR/MTX xenograft model. No mice died after sitagliptin treatment. As depicted in **Figure 8A**, sitagliptin alone or sitagliptin +MTX effectively suppressed tumor growth *in vivo*, as compared with PBS control or MTX. The tumor weight in sitagliptin +MTX group was further reduced compared to either drug alone (**Figures 8B, C**). The DPP4 enzymatic activity and cellular cholesterol level were greatly reduced by sitagliptin or sitagliptin+MTX treatment (**Figures 8D, E**). Hematoxylin/Eosin staining revealed severe necrosis within the xenografts following sitagliptin+MTX treatment (**Figure 8F** and **Supplementary Figure S5**). sitagliptin or sitagliptin+MTX treatment also strongly reduced DHCR24 expression in xenograft tissues (**Figure 8F** and **Supplementary Figure S5**). Ki67 positivity was greatly decreased after sitagliptin or sitagliptin+MTX treatment, as compared with PBS or MTX group (**Figures 8F, G** and **Supplementary Figure S5**). The combination of sitagliptin with MTX further reduced Ki-67 positivity in JAR/MTX tumors, as compared with sitagliptin or MTX alone (**Figures 8F, G** and **Supplementary Figure S5**). In contrast to PBS or MTX group, treatment with sitagliptin or sitagliptin+MTX significantly increased the TUNEL labeling in JAR/MTX tumors (**Figures 8F, H** and **Supplementary Figure S5**). Sitagliptin+MTX exhibited the highest TUNEL labeling in JAR/MTX tumors, as compared with sitagliptin or MTX alone (**Figures 8F, H** and **Supplementary Figure S5**). Taken together, these findings confirmed the *in vivo* efficacy of sitagliptin alone or sitagliptin+MTX combination for the treatment of MTX-resistant GTN.

## Discussion

DPP4 plays an important role in the pathogenesis of type 2 diabetes, obesity and cardiovascular diseases (21, 37, 38). Increased DPP4 activity and levels have been shown to be closely associated with these metabolic diseases (21, 37, 38). Recently, DPP4 has been proposed as a new adipokine, which might play an important role in lipid metabolism in adipose tissues (39). Sato et al. showed that DPP4 is crucial for regulating the expression of enzymes related to steroid metabolism (40). Our metabolomics analysis revealed that steroid/cholesterol biosynthesis-related metabolites were markedly impacted by DPP4 depletion in MTX-resistant GTN sublines. Consistently, manipulation of DPP4 expression remarkably affected the level of cellular cholesterol in GTN cells. Therefore, our findings confirmed the metabolic role of DPP4 as a potential regulator of cholesterol biosynthesis, which might mechanistically contribute to MTX resistance in GTN cells. Although DPP4 is known to promote lipid biosynthesis (triglyceride, fatty acid, cholesterol, etc) in adipocytes (34), our metabolomics analysis revealed that the biosynthesis of cholesterol, rather than other lipids (Triglyceride, fatty acid) was greatly affected by DPP4 knockdown in MTX-resistant GTN cells. Therefore, DPP4 might exert distinct cellular functions associated with differential metabolic pathway activation in specific cell types.

ROS plays a pivotal role in mediating cytotoxicity induced by chemotherapeutic drugs through inducing DNA damage, attenuating membrane fluidity, and disrupting mitochondria function in cancer cells (41). Neutralization of ROS is a key underlying mechanism of drug resistance in cancer chemotherapy (41). Cholesterol has been shown to exert a critical function in antagonizing the cytotoxic effect of anti-cancer drugs in cancer cells (16, 42). The high cholesterol level not only attenuates the accumulation of ROS but also renders cancer cells more resistant to anti-cancer drug treatment (16, 42). DHCR24, a pivotal enzyme of terminal step of cholesterol biosynthesis, could exert its prosurvival effect through attenuating oxidative stress-induced apoptosis in different cell models (43–45). Recently, we showed that ROS induced by MTX is greatly neutralized by DPP4 in MTX-resistant GTN cells (26), despite its mechanistic association with DHCR24-mediated cholesterol biosynthesis still remains largely unknown. In the present study, our gene expression analysis identified cholesterol biosynthesis-related enzyme DHCR24 as a downstream effector of DPP4 signaling in MTX-resistant GTN cells. Cellular function analysis revealed that manipulation of DHCR24 expression could also regulate cholesterol biosynthesis, ROS accumulation and chemosensitivity to MTX in GTN cells. More importantly, over-expression of DHCR24 could apparently restore cellular cholesterol level and chemoresistance to MTX in DPP4-depleted MTX-resistant GTN cells. Therefore, DHCR24 might serve as a potential downstream effector of DPP4 function in promoting MTX resistance in GTN cells. However, the downstream signaling of DPP4 leading to the upregulation of DHCR24 in GTN cells still need to be characterized in future study.

Metabolic reprogramming is a hallmark of both diabetes and cancer, which is presented with similar abnormalities in glucose, fatty acid, and cholesterol metabolisms (46). DPP4 has been identified as a potential regulator that links enhanced cancer risk with metabolic diseases such as hyperglycemia and obesity (47). DPP4 inhibitors, such as sitagliptin, linagliptin, vildagliptin, and saxagliptin, have good efficacy, safety and tolerability in the treatment of type II diabetes (48). Therefore, the repurposing actions of DPP4 inhibitors may serve as an alternative intervention in cancer treatment. Some preclinical studies showed that DPP4 inhibitors could suppress the growth of a number of cancer cell lines (49–51). As a proof of concept, we treated MTX-resistant GTN cells with a classical DPP4 inhibitor sitagliptin *in vitro*. Our data suggested that sitagliptin potently attenuated DPP4 enzymatic activity, inhibited DHCR24 expression and cholesterol biosynthesis, and enhanced MTX-induced cytotoxicity on MTX-resistant GTN cells. The effect of sitagliptin on MTX-resistant GTN cells was also evaluated *in vivo*. In line with *in vitro* findings, sitagliptin treatment (alone or combined with MTX) effectively reduced DPP4 enzymatic activity, inhibited cholesterol biosynthesis and DHCR24 expression, and suppressed tumor growth in JAR/MTX xenograft model. Impressively, combined MTX and sitagliptin treatment further decreased Ki67 positivity and induced apoptosis compared to either drug alone in transplanted tumors. Therefore, our study may identify DPP4 inhibitor sitagliptin as a potential agent to treat MTX-resistant GTN.

In conclusion, our findings suggested that DPP4 might regulate DHCR24-mediated cholesterol biosynthesis to promote methotrexate resistance in GTN cells. Further, DPP4 inhibitor sitagliptin could effectively disrupt DPP4/DHCR24 signaling and enhance MTX-induced cytotoxicity in MTX-resistant GTN cells *in vitro* and *in vivo*. Considering the well-tolerated long-term safety of sitagliptin in the treatment of Type 2 Diabetes Mellitus (52), the use of sitagliptin for the treatment of GTN might not cause severe side effect. Therefore, our findings might have potential translational value in designing DPP4-targeting experimental therapeutics to treat MTX-resistant GTN.

## Data Availability Statement

The datasets presented in this study can be found in online repositories. The names of the repository/repositories and accession number(s) can be found in the article/**Supplementary Material**.

## Ethics Statement

The studies involving human participants were reviewed and approved by Xiangya Hospital, Central South University. The patients/participants provided their written informed consent to participate in this study. The animal study was reviewed and approved by Xiangya hospital, Central South University.

## Author Contributions

DS designed the study. WYu, WYo, and JZ performed the experiments. WYu, WYo, and JZ analyzed the data. DS wrote the manuscript. All authors have read and approved the final version of the manuscript.

## Funding

This work was supported by The Natural Science Foundation of Hunan Province (Grant Number: 2018JJ6065) and National Natural Science Foundation of China (Grant Number: 81201904).

## Conflict of Interest

The authors declare that the research was conducted in the absence of any commercial or financial relationships that could be construed as a potential conflict of interest.

## Publisher’s Note

All claims expressed in this article are solely those of the authors and do not necessarily represent those of their affiliated organizations, or those of the publisher, the editors and the reviewers. Any product that may be evaluated in this article, or claim that may be made by its manufacturer, is not guaranteed or endorsed by the publisher.
