# Supplementary material for: DPP4 Regulates DHCR24-Mediated Cholesterol Biosynthesis to Promote Methotrexate Resistance in Gestational Trophoblastic Neoplastic Cells
Source: Front Oncol. 2021 Dec 2;11:704024. doi: 10.3389/fonc.2021.704024 (PMC8675944; doi:10.3389/fonc.2021.704024)
Supplement: Supplementary file 1 [file DataSheet_1.pdf]

Supplementary Figures

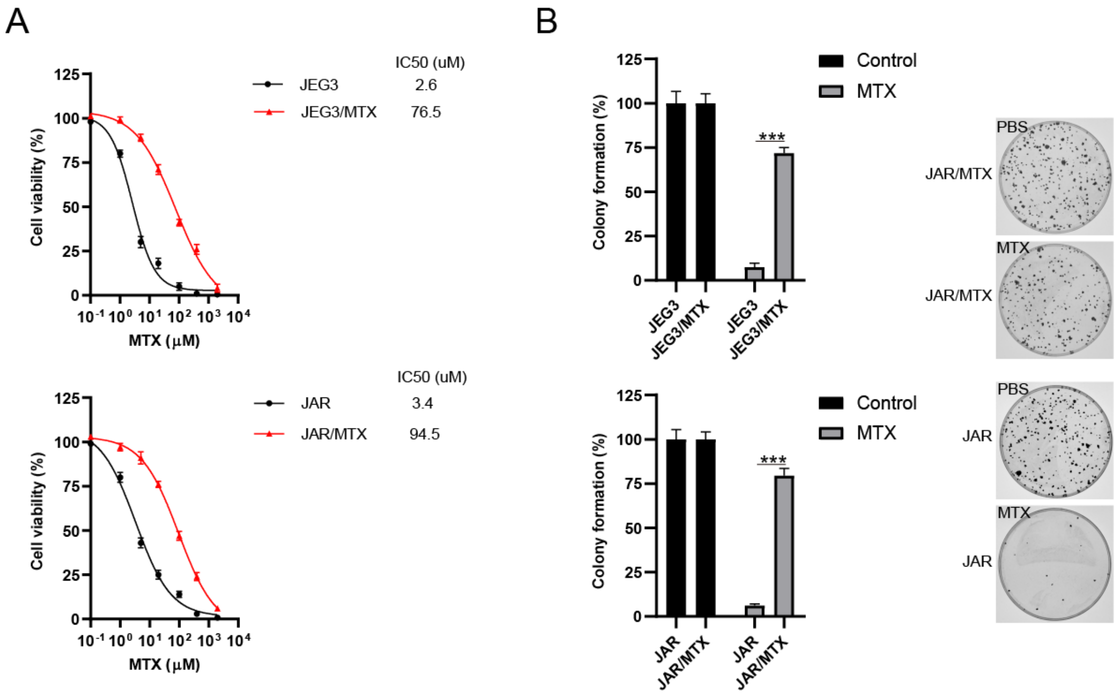

Figure S1: A. IC$_{50}$ values of MTX-resistant sublines and parental GTN cell lines. **B**. Colony formation of MTX-resistant sublines and parental GTN cell lines with or without MTX treatment (10 μM) for 12 days.

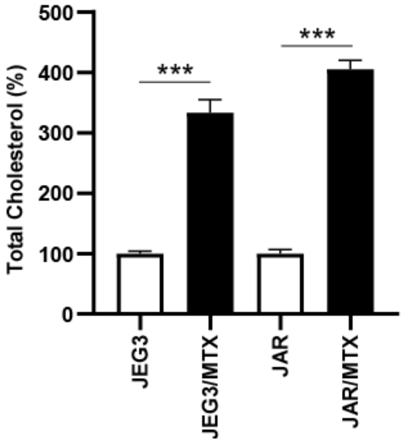

Figure S2. The level of cellular cholesterol in JAR/MTX and JEG3/MTX cells was greatly elevated compared to parental JAR and JEG3 cells

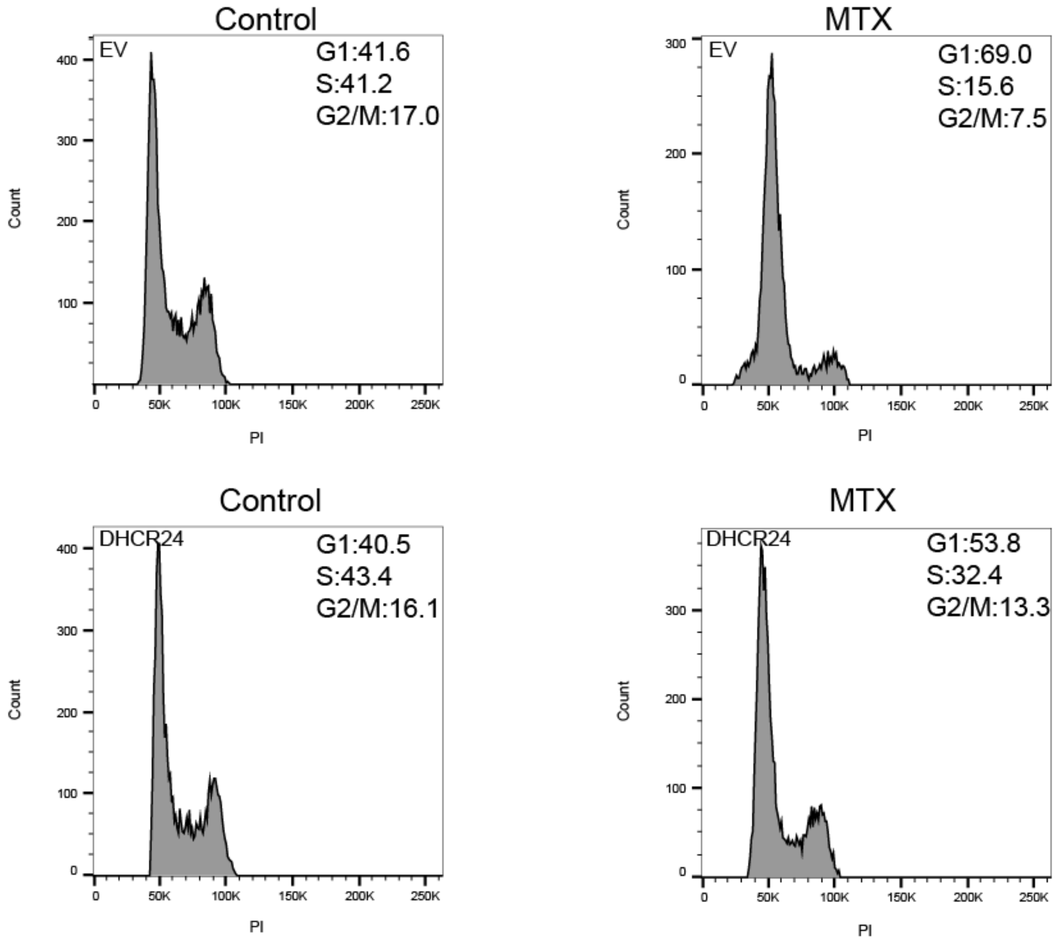

Figure S3. Cell cycle analysis of EV or DHCR24-expressing JAR cells after MTX treatment (3 μM) for 96 hours.

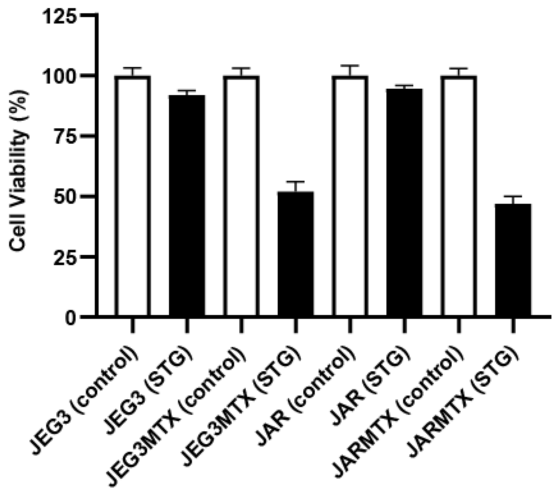

Figure S4: Differential response of GTN cell lines and their MTX-resistant sublines to sitagliptin treatment

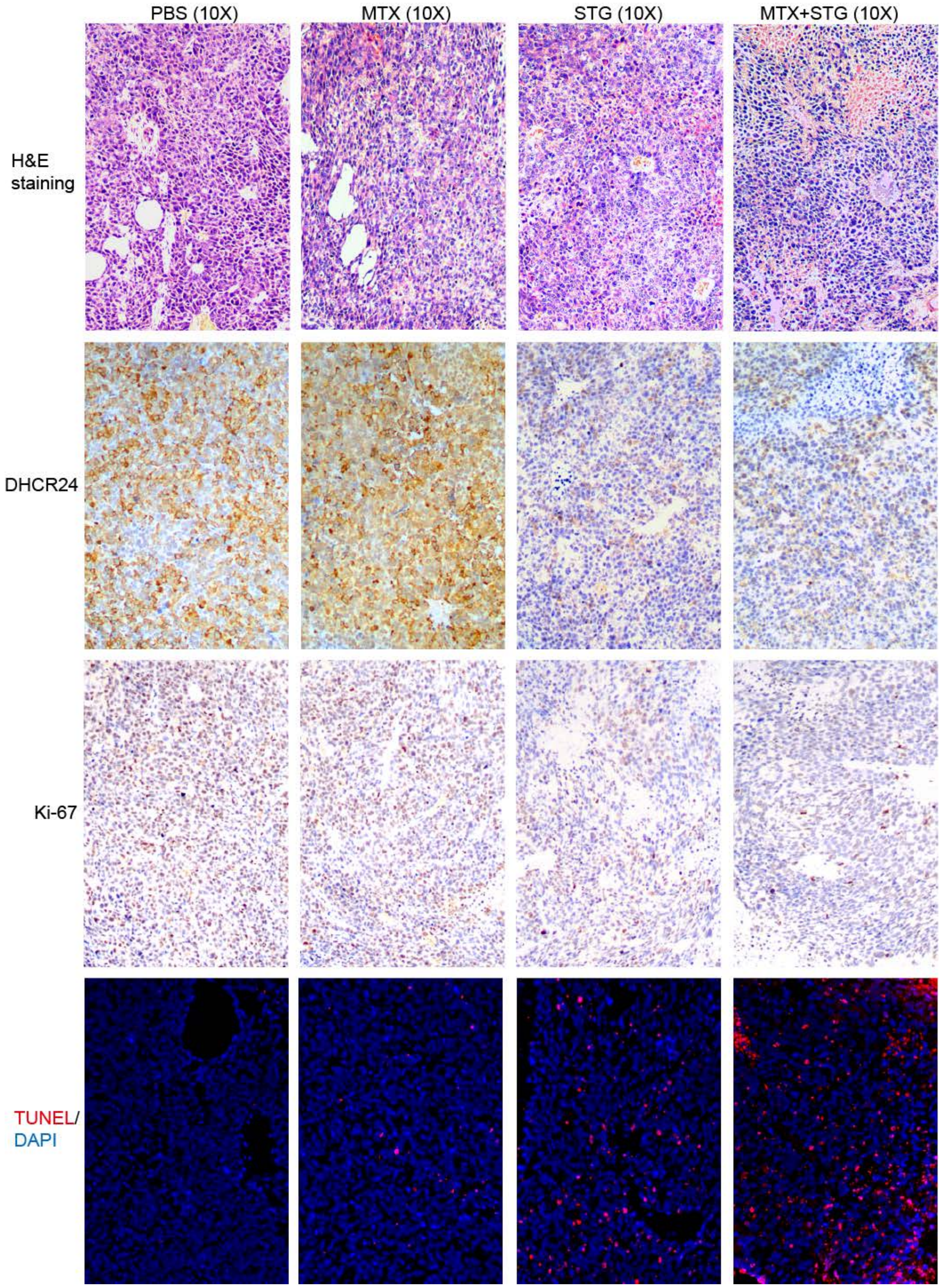

Figure S5: Immunostaining of the JAR/MTX xenografts after drug treatment. The tissue sections were immunostained with primary antibodies (DHCR24 and Ki-67) or labeled by TUNEL (10x objective magnification).

Supplementary methods

Soft agar assay

Soft agar assay was used to examine the *in vitro* clonogenesis of GTN cells after drug treatment. Briefly, the 2 mL culture medium with 0.5% agar was first plated into each well of a 6 cm culture dish. After the agar solidified, each well received another 2 mL of 0.35% agar in culture medium containing 1×10^5 cells with or without MTX. After 10~12 days, colonies were fixed by 4% paraformaldehyde and counted.

Cell viability analysis

GTN cells were seeded in a 96-well culture plates in triplicate (2×10^3 cells/well). Cell viability was monitored by CCK-8 viability assay at 96 hours after various concentrations of MTX treatment (1, 5, 20, 100, 400, 2000 μM). The optical density (OD) (absorbance) was measured at 450 nm using a Multiskan MK3 microplate reader (Thermo Fisher Scientific, Waltham, MA, USA). IC_{50} values were calculated in SPSS software as described previously [1] .

Reactive oxygen species (ROS) measurement

The level of Intracellular ROS was detected by DCFDA fluorescence assay [1]. Briefly, GTN cells were seeded into 96-well plates (2×10^3 cells/well) and incubated overnight before addition of MTX (10 μM for MTX-resistant sublines; 3 μM for parental cell lines). After MTX treatment for 96 hours, GTN cells were incubated with 25μM DCFDA for 30 min before ROS measurement. An F97Pro fluorospectrometer (Lengguang Technology, Shanghai, China) was used to measure the DCFDA fluorescence.

Western blotting

Western blot was conducted as we described previously [1]. Protein lysates (25 μg) was separated by 10% SDS-PAGE and transferred to Hybond-P PVDF membranes (Amersham Biosciences). Blots were blocked with 5 % non-fat dry milk in Tris-buffered saline buffer for 2 hours at room temperature and then incubated with diluted antibodies against various proteins for 2 hours at room temperature, followed by incubation with horseradish peroxidase-conjugated goat-anti-mouse antibody

(Abcam, Cambridge, MA) for 1 hour at room temperature. The signal was visualized with an enhanced chemiluminescence detection reagent (Abcam, Cambridge, MA).

Cell cycle analysis

To identify shifts in the proportion of cell cycles, Briefly, JAR cells were seeded into 6-well plates (1×10^5 cells/well) and incubated overnight. After MTX treatment (3 μM) for 96 hours, JAR cells were fixed in 70% ethanol overnight, and incubated with staining buffer containing propidium iodide and RNAase for 30 min. After incubation, the cells were analyzed by BD FACSCanto™.

References

[1] Jun F, Peng Z, Zhang Y and Shi D. Quantitative proteomic analysis identifies novel regulators of methotrexate resistance in choriocarcinoma. Gynecol Oncol 2020; 157: 268-279.

Table S1: Differentially regulated metabolites in shDPP4 vs. scr comparison in JEG3/MTX

Index	Compounds	Formula	RT(min)	cpd_ID	shDPP4-1	shDPP4-2	shDPP4-3	Scr-1	Scr-2	Scr-3	VIP value	p_value	-Log10P	Fold_Change	Log2FC
N1085	Rimsulfuron	C14H17N5O7S	2.537	C10952	21437.2	21338.61	22270.28	1774.77	2090.77	1060.82	1.462	0.0000	5.915	13.204	3.723
N1894	dTDP-4-oxo-2,3,6-trideoxy-D-	C16H24N2O13	3.181	C18032	7125.78	8893.98	6759.41	743.5	1023.08	647.99	1.435	0.0079	2.104	9.434	3.238
P1692	Quercetin-3-(6"-malonyl)-Gluc	C24H22O15	3.204	C12638	9376.14	9241.3	16072.21	713.79	1129.49	2142.86	1.316	0.0412	1.385	8.703	3.121
N0866	Thiobenzamide	C7H7NS	3.185	C16281	12175.31	17193.38	12771.12	2892.93	2167.03	2106.69	1.410	0.0160	1.795	5.880	2.556
P1368	(5-Phenyl-1,2,4-triazol-3-yl)ur	C9H9N5O	2.298	C15383	150376.73	153707.25	141907.18	28166.15	27303.91	27957.11	1.444	0.0008	3.099	5.346	2.418
P0450	Phytosphingosine	C18H39NO3	4.521	C12144	3941.8	6224.94	5448.43	728.49	1230.66	1086.27	1.370	0.0205	1.688	5.127	2.358
N0527	Magnocurarine	C19H24NO3	4.731	C16991	13646.34	8060.85	8543.5	1013.43	435.51	4701.55	1.279	0.0259	1.587	4.918	2.298
P0784	Creatine; LC-ESI-QTOF; MS2	C4H9N3O2	0.852	C00300	26263.96	32823.84	20800.69	6277.69	7084.92	3081.73	1.367	0.0172	1.765	4.858	2.280
N0479	p-Bromophenylacetic acid	C8H7BrO2	0.934	C03076	13839.73	11934.82	7968.26	3030.82	2312.69	1715.49	1.359	0.0309	1.510	4.780	2.257
N1892	dTDP-6-deoxy-beta-L-talose;d	C16H26N2O15	3.044	C03187	10964.27	10566.93	13757.54	3352.6	1534.51	2617.8	1.422	0.0037	2.430	4.702	2.233
N0935	Quercetin 3-sulfate	C15H10O10S	1.252	C00616	3396.48	3681.62	5538.61	1339.62	773.06	715.71	1.346	0.0314	1.503	4.461	2.157
P1836	Ceftibuten	C15H14N4O6S	3.370	C08117	27409.9	34580.7	30138.54	5510.74	9117.31	7274.35	1.416	0.0023	2.635	4.206	2.073
N1835	Cefprozil	C18H19N3O5S	3.360	C06888	34587.64	35409.97	31033.19	7008.56	8089.42	9595.02	1.452	0.0004	3.430	4.091	2.033
P1204	Niflumic acid;NFA	C13H9F3N2O2	1.292	C13698	12045.77	22819.2	20876.09	5065.77	6150.98	2545.3	1.291	0.0412	1.385	4.050	2.018
N1154	alpha,alpha'-Diethyl-4,4'-bis(2-	C24H24O2	9.450	C15066	7937.9	11003.09	10538.33	2129.72	2386.36	2811.64	1.414	0.0134	1.874	4.023	2.008
P1471	Vitamin K1 epoxide;(2,3-Epox	C31H46O3	7.641	C05849	14334.96	15680.95	17759.28	4500.31	3819.18	3565.68	1.424	0.0043	2.370	4.020	2.007
N0870	Thifensulfuron-methyl;Thifens	C12H13N5O6S	1.233	C10957	3652.83	3738.29	3299.65	835.86	513.87	1387.82	1.429	0.0026	2.582	3.905	1.965
N1137	Acesulfame-K	C4H5NO4S	0.834	D08836	25850.2	24438.83	22457.1	3937.07	6589.06	8944.84	1.436	0.0010	3.007	3.736	1.902
P0362	Phytpl, (E,7R,11R)-3,7,11,15-	C20H40O	8.953	C01389	164969.58	158539.02	162967.69	40340.21	45773.17	44636.35	1.445	0.0000	5.825	3.721	1.896
N0224	(S)-Malate	C4H6O5	1.216	C00149	22251.93	15704.73	26406.76	4786.1	5719.1	6922.89	1.358	0.0333	1.478	3.693	1.885
P1673	Taurine	C2H7NO3S	0.828	C00245	13232.16	15720.71	8778.31	4427.05	3871.83	2154.78	1.311	0.0355	1.449	3.609	1.852
N0413	Taurine	C2H7NO3S	0.834	C00245	21202.11	22579.25	21895.11	6671.72	4672.48	6929.14	1.455	0.0002	3.639	3.594	1.846
N0989	Plocamene D	C10H13Cl3	0.950	C17105	13102.2	10837.37	8629.73	3234.78	3768.12	2241.15	1.381	0.0178	1.750	3.523	1.817
P0464	Mesaconitine	C33H45NO11	3.378	C08698	21624.84	21605.94	18321.92	6402.24	6243.24	4882.49	1.429	0.0018	2.748	3.512	1.812
P1060	Amdinocillin	C15H23N3O3S	11.101	D02888	27403.04	24990.29	26980.4	8152.26	6294.77	8635.11	1.439	0.0001	4.266	3.439	1.782
P0782	Creatine; LC-ESI-QTOF; MS2	C4H9N3O2	0.836	C00300	27511.5	33553.11	27487.68	8180.05	11317.48	7275.81	1.408	0.0022	2.662	3.307	1.726
N1351	1,2,3-Trichloropropane	C3H5Cl3	1.349	C14400	7576.97	14435.63	11624.51	2938.42	4593.96	2656.84	1.291	0.0491	1.309	3.301	1.723
N1766	cis-2-Chloro-4-carboxymethyle	C6H3ClO4	0.925	C04706	10493.73	9820.69	8462.4	2279.04	3585.68	3016.77	1.431	0.0016	2.810	3.240	1.696
N0442	3-Oxo-5beta-cholanate;3-Oxo-	C24H38O3	8.951	C03070	47458.16	45859.65	45952.5	15040.44	15601.19	13267.92	1.461	0.0000	5.112	3.172	1.665
P0926	Tomatidine	C27H45NO2	8.953	C10826	24434.2	24962.45	26317.96	7015.71	9443.89	7465.5	1.436	0.0001	4.067	3.165	1.662
N1945	Dephosphoryl CoA	C21H35N7O13	1.233	C00882	30775.1	36432.83	37807.88	15355.5	14345.47	3757.92	1.378	0.0096	2.020	3.139	1.650
P2214	Isoamylamine	C5H13N	0.835	C02640	13371.56	11301.1	13017.44	4731.82	3674.59	3630.44	1.428	0.0011	2.970	3.131	1.647
N1919	Cypermethrin	C22H19Cl2NO	1.706	C10984	110478.63	132986.33	166826.08	31897.22	39129.82	60702.4	1.360	0.0148	1.828	3.115	1.639
N1044	Taurine	C2H7NO3S	1.108	C00245	160075.58	128512.99	127500.19	39609.5	52240.8	43190.32	1.423	0.0072	2.143	3.081	1.623
N0922	Prenalterol	C12H19NO3	6.043	C07533	71781.05	53309.69	56150.29	8491.58	26966.65	23645.9	1.362	0.0073	2.137	3.066	1.617
P1649	Sodium molybdate;Sodium mo	Na2MoO4	2.340	C15455	22925.77	18650.94	17088.5	6316.71	6142.69	7114.66	1.398	0.0153	1.815	2.997	1.584
N0227	Adenosine monophosphate (A	C10H14N5O7P	1.224	C00020	25246.12	32642.74	29060.05	9783.37	14012.14	5662.98	1.387	0.0042	2.378	2.952	1.561
N0211	.beta.-Nicotinamide adenine di	C21H27N7O14	1.233	C00003	53309.26	65493.17	75087.84	26546.7	17144.51	22609.28	1.392	0.0112	1.951	2.924	1.548
N1315	Atrazine-desethyl-desisopropyl	C3H4ClN5	0.834	C06560	62799.57	58621.09	56355.91	19857.12	22704.98	18297.33	1.454	0.0002	3.794	2.921	1.547
N0222	Adenosine monophosphate (A	C10H14N5O7P	0.925	C00020	89678.58	108248.17	135397.58	64563.37	6548.82	43114.31	1.260	0.0299	1.524	2.918	1.545
P2523	3,5-Dinitro-4-hydroxyphenylpy	C9H6N2O8	3.046	C04286	12903.74	13642.45	10414.55	3130.38	5681.42	3856.6	1.382	0.0034	2.462	2.918	1.545
N1293	Arotinoid acid;TTNPB;Ro 13-	C24H28O2	4.731	C15634	71963.05	39755.81	48812.2	14425.92	8790.82	32615.26	1.207	0.0478	1.321	2.875	1.524
N1644	Fenchlorazole-ethyl	C12H8Cl5N3O	1.108	C18831	7918.19	6545.64	11071.86	2791.58	2130.08	4061.58	1.296	0.0387	1.412	2.843	1.507
P0922	Tomatidine	C27H45NO2	8.953	C10826	118835.89	125658.2	132085.63	42072.56	46189.48	45119.9	1.438	0.0010	3.021	2.823	1.497
N1426	1-(sn-Glycero-3-phospho)-1D-	C9H19O11P	1.233	C01225	127295.08	144229.99	149621.4	58222.75	53797.32	43277.62	1.440	0.0008	3.088	2.712	1.439
N0503	Nitrofen	C12H7Cl2NO3	1.376	C11065	9598.54	11213.65	8509	3811.04	3894.8	3194.12	1.413	0.0113	1.948	2.690	1.428
P2404	gamma-Coniceine	C8H15N	1.226	C10138	51052.31	44451.36	46766.4	16159.63	19277.02	17739.1	1.432	0.0011	2.964	2.675	1.420
P2393	4'-Hydroxy-2,2',3,3',4,5,5'-hept	C12H3Cl7O	2.074	C14726	17068.6	13421.96	18346.04	5912.82	5436.21	6938.63	1.385	0.0143	1.845	2.670	1.417
N1226	Benfuresate	C12H16O4S	0.842	C18452	11418.58	11258.7	11034.18	5248.58	2888.85	4587.49	1.433	0.0086	2.063	2.649	1.406
N0973	Pesticide4_Chlorantraniliprole	C18H14BrCl2N	1.822	C18454	40958.64	31715.15	36590.31	14213.23	15705.72	12165.76	1.419	0.0073	2.137	2.596	1.376
N1528	3'-Phosphoadenylylselenate;3'-	C10H15N5O13	1.374	C05696	9853.23	9653.91	6371.39	3487.1	3575.32	2994.47	1.342	0.0399	1.399	2.573	1.364
N1518	3'-O-(4-Benzoyl)benzoyl ATP;	C24H24N5O15	1.233	C13744	6417.71	7776.62	9313.59	2278.72	3729.53	3157.2	1.364	0.0151	1.821	2.565	1.359
P1207	Nicotinic Acid Adenine Dinuc	C21H27N7O14	0.918	C00003	25414.15	16759.24	21659.96	8562.11	9116.19	7292.7	1.348	0.0307	1.512	2.556	1.354
P0940	2-Hydroxy-2-ethylsuccinic aci	C6H10O5	0.910	C02488	13702.51	9156.02	11994.15	4803.88	5756.18	3172.41	1.330	0.0169	1.772	2.538	1.344
P0374	Propionylcarnitine	C10H19NO4	1.276	C03017	459580.02	431701.11	450173.01	164507.7	205212.1	167358.08	1.436	0.0002	3.673	2.498	1.321
N1956	dCDP;2'-Deoxycytidine diphos	C9H15N3O10P	0.917	C00705	70021.52	78505.72	72413.6	35285.1	27670.63	27336.15	1.443	0.0003	3.560	2.447	1.291
P1887	Dihydroneopterin triphosphate	C9H16N5O13P	0.719	C04895	19710.81	17937.31	19801.14	12035.4	7941.99	3509.39	1.326	0.0377	1.424	2.446	1.290
N1728	Glycerone phosphate; LC-ESI-	C3H7O6P	0.925	C00111	28556.31	30051.31	24771.42	11464.16	11301.39	11434.56	1.436	0.0090	2.045	2.438	1.286
P1510	Topotecan	C23H23N3O5	0.828	C11158	22390.32	21702.41	20806.5	9023.22	11341.12	6386.2	1.408	0.0076	2.119	2.426	1.279
P1190	Penbutolol	C18H29NO2	2.365	C07416	15704.75	14259.74	12307.15	4476.69	6259.04	6760.78	1.388	0.0035	2.459	2.416	1.273
P2444	4-Acetamido-4'-isothiocyanosti	C17H14N2O7S	0.720	C13702	24356.3	25033.18	24638.19	10021.18	12187.36	8639	1.430	0.0040	2.399	2.400	1.263
N0800	UMP;Uridylic acid;Uridine mo	C9H13N2O9P	0.900	C00105	282378.25	324876.3	409463.35	160111.91	111309.4	152299.77	1.355	0.0217	1.663	2.399	1.263
N1391	(5-L-Glutamyl)-L-glutamine	C10H17N3O6	0.858	C05283	35024.4	41017.25	41023	20077.37	12836.32	16110.74	1.417	0.0014	2.840	2.388	1.256
N0925	Potassium sulfate	K2O4S	0.834	C13192	18581.32	15366.21	13322.68	6894.62	9318.07	3690.33	1.315	0.0152	1.818	2.375	1.248
P1912	Cytosine arabinoside;Cytarabi	C9H13N3O5	1.110	C02961	53197.29	39180.05	37221.1	16019.26	21550.18	18145.66	1.331	0.0298	1.525	2.326	1.218
P1006	Adenosine 5'-monophosphate	C10H14N5O7P	1.218	C00020	50776.94	52982.42	55087.28	23521.67	26391.88	18616.92	1.425	0.0012	2.934	2.318	1.213
P1507	Tralomethrin	C22H19Br4NO	2.423	C18413	26003.52	28455.45	28821.36	13603.03	10311.61	12078.12	1.428	0.0003	3.565	2.314	1.210
N0016	2-Aminoethanesulfonic Acid	C2H7NO3S	0.826	C00245	422649.27	414983.7	406341.97	179651.51	181147.05	180860.85	1.462	0.0004	3.442	2.297	1.199
N1079	S-(2,2-Dichloro-1-hydroxy)eth	C12H19Cl2N3	1.222	C14861	134423.94	154917.36	173575.18	78092.94	60881.95	64215.4	1.406	0.0074	2.131	2.278	1.188
P1280	N-Methylhexanamide	C7H15NO	6.660	C02722	34830.78	27223.06	26077.67	15334.34	10974.7	12444.48	1.363	0.0142	1.847	2.274	1.185
P0993	Arcaine	C6H16N6	1.276	C03047	112255.82	155449.67	170143.4	75318.41	55548.12	61671.91	1.318	0.0318	1.497	2.274	1.185
P0220	Adenosine-3-monophosphate	C10H14N5O7P	1.218	C01367	601805.97	666988.96	637817.85	292964.81	325949.06	222723.94	1.417	0.0014	2.863	2.265	1.180
N1641	Fenoxanil	C15H18Cl2N2	1.223	C18519	8137.36	9531.19	6626.1	3507.78	4266.58	3015.18	1.355	0.0200	1.699	2.252	1.171
N0012	Uridine 5-Monophosphate	C9H13N2O9P	1.224	C00105	331096.48	384605.07	413667.7	188276.38	141263.32	174844.38	1.413	0.0040	2.401	2.239	1.163
P1869	Dephosphoryl CoA	C21H35N7O13	1.288	C00882	16737.62	13550.27	15103.19	7458.53	7034.7	5858.66	1.407	0.0040	2.401	2.230	1.157
P0459	Methenolone	C20H30O2	4.949	D08193	10810.16	10812.75	9121.8	5343.13	4623.03	3824.32	1.406	0.0017	2.757	2.229	1.157
N1786	Copal-8-ol diphosphate;(13E)-	C20H38O8P2	3.900	C20270	24918.02	24624.06	27854.49	16520.13	8842.87	9474.46	1.369	0.0172	1.765	2.222	1.152
P1760	Cholesteryl-beta-D-glucoside	C33H56O6	2.801	C03855	39345.4	40851.5	34456.18	16778.76	26068.33	9005.81	1.290	0.0374	1.427	2.211	1.145
N1392	(4-Cyano-2,6-diiodophenyl) oc	C15H17I2NO2	1.483	C18740	146118.32	153932.03	163741.35	70649.06	74577.21	68368.8	1.452	0.0015	2.814	2.171	1.119
N0246	Uridine monophosphate (UMP	C9H13N2O9P	0.900	C00105	37315.66	37513.98	45269.6	18597.07	18558.37	18561.61	1.423	0.0146	1.837	2.156	1.108
N1738	Heroin;Diamorphine;Diacetyln	C21H23NO5	9.026	C06534	26324.91	23843.11	23509.24	10473.39	11590.71	12117.08	1.447	0.0008	3.077	2.155	1.108
N0904	Prothiofos	C11H15Cl2O2P	1.798	C18405	28147.48	31264.4	30298.54	8850.73	15679.16	17600.33	1.380	0.0181	1.742	2.129	1.090
P2078	Brugine	C12H19NO2S2	0.910	C10849	25473.11	21230.13	22774.1	8444.58	13195.56	11080.61	1.383	0.0028	2.553	2.123	1.086
N0015	Adenosine 5'-Monophosphate	C10H14N5O7P	1.224	C00020	842114.19	1003056.39	939613.03	463753.34	447165.69	400660.76	1.433	0.0039	2.410	2.123	1.086
N1424	(Z)-But-1-ene-1,2,4-tricarboxy	C7H8O6	0.974	C04002	1564.56	1708.34	2362.18	1135.78	504.3	1014.29	1.238	0.0362	1.442	2.123	1.086
N1516	2,3,9,10-Tetrahydroxyberbine	C17H17NO4	1.611	C12330	23774.25	26695.8	23019.28	14185.29	6103.53	14606.62	1.325	0.0297	1.527	2.106	1.074
N1501	2,3-Dibromo-1-propanol;2,3-D	C3H6Br2O	0.792	C19224	29200.68	26609.92	22825.81	11502.28	17164.82	8771.56	1.336	0.0134	1.874	2.100	1.071
P1742	Acrolein	C3H4O	0.844	C01471	21018.32	19329.38	18020.13	7664.9	8885.94	11353.41	1.394	0.0022	2.651	2.092	1.065
N0869	Thifluzamide	C13H6Br2F6N	0.834	C18505	22674.12	24497.4	21154.26	11238.34	13670.72	8094.27	1.394	0.0063	2.198	2.070	1.050
N1950	delta-3,4,5,6-Tetrachlorocyclo	C6H6Cl4	1.789	C06989	17832.2	16442.41	15154.77	8590.32	5740.43	9664.52	1.388	0.0059	2.227	2.060	1.043
P0580	Octanoylcarnitine	C15H29NO4	4.558	C02838	36338.15	42420.43	34586.17	17426.35	17465.93	20251.2	1.398	0.0076	2.120	2.055	1.039
P0783	Creatine; LC-ESI-QTOF; MS2	C4H9N3O2	0.844	C00300	29457.77	25181.65	29613.52	13916.53	9427.11	17672.77	1.350	0.0110	1.957	2.054	1.039
P0956	BETAINE	C5H11NO2	0.841	C00719	19072.02	29666.15	24405.43	15891.51	13246.19	6694.96	1.208	0.0393	1.405	2.041	1.029
P2008	A 80987	C37H43N5O6	2.755	C15661	30322.56	27875.18	24266.56	13102.2	18715.58	8658.81	1.302	0.0217	1.663	2.037	1.027
N0221	Adenosine monophosphate (A	C10H14N5O7P	0.917	C00020	494446.26	565469.51	707623.41	314888.75	292275.29	261192.06	1.345	0.0346	1.461	2.036	1.025
N1087	Rifampin	C43H58N4O12	2.948	C06688	11427.25	12141.3	11283.64	4430.12	7403.97	5333.83	1.398	0.0153	1.816	2.030	1.022
P0378	Pymetrozin	C10H11N5O	1.800	C18590	239102.61	220586.03	230606.39	113718.36	117561.41	110407.1	1.440	0.0006	3.191	2.020	1.015
P2307	Mirasan	C13H21ClN2	0.902	C11716	1245826.81	1044686.09	1137252.89	489606.16	672641.87	541641.67	1.393	0.0020	2.701	2.012	1.008
N1065	Silver sulfadiazine	C10H9AgN4O2	1.789	D00433	24102.31	24408.23	27071.8	10993.53	15409.36	11269.65	1.414	0.0031	2.511	2.006	1.005
P1822	Ceftriaxone	C18H18N8O7S	0.716	C06683	11406.43	11984	10395.79	4070.23	7825.38	4944.93	1.322	0.0250	1.601	2.006	1.004
P2041	Albizziin	C4H9N3O3	3.790	C08264	16223.59	21542.96	17515.6	8932.4	11875.65	6765.45	1.305	0.0135	1.870	2.005	1.004
P0284	Carnitine	C7H15NO3	0.844	C00318	504205.49	637204.28	586192.2	360172.24	242966.76	259114.03	1.359	0.0057	2.243	2.004	1.003
N0645	Molybdopterin guanine dinucl	C20H24N10O1	0.892	C19928	1912.6	4563.54	5038.11	6441.76	7971.6	8717.2	1.250	0.0364	1.439	0.498	-1.006
P1386	Tiocarbazil;Thiocarbazil	C16H25NOS	3.205	C18898	28016.28	35779.1	31816.55	58218.35	72758.96	61397.98	1.388	0.0076	2.120	0.497	-1.009
P0835	Solanidine	C27H43NO	8.480	C06543	5399.01	6647.37	5892.56	10328.58	13675.65	12132.58	1.375	0.0152	1.817	0.496	-1.010
P1648	Sodium stibogluconate;Antimo	C12H20O17Sb2	0.720	C07638	12060.38	13410	16020.05	29131.59	27380.19	27103.08	1.421	0.0016	2.808	0.496	-1.011

P0482	N-Acetyl-DL-leucine	C8H15NO3	1.409	D07350	8655.21	7858.21	7640.92	16760.63	16994.74	15017.77	1.425	0.0015	2.830	0.495	-1.014
P1761	7-Dehydrocholesterol	C27H44O	10.931	C01164	20207.09	21312.96	22377.76	40424.52	46380.28	43057.6	1.431	0.0003	3.557	0.492	-1.023
P0409	Vindesine	C43H55N5O7	3.287	D06304	26787.49	21434.9	31472.65	59121.22	49226.52	54313.71	1.385	0.0025	2.610	0.490	-1.029
P0846	Solanidine	C27H43NO	8.480	C06543	25653.96	21944.19	22249.15	43979.43	48175.55	50421.03	1.424	0.0010	3.014	0.490	-1.029
P2415	Fluoxymesterone	C20H29FO3	0.914	D00327	2850.33	6259.84	4865.44	9923.21	11625.63	6986.32	1.193	0.0495	1.306	0.490	-1.030
P2327	2-Methylsuccinic Acid	C5H8O4	1.750	C08645	7056.75	4614.16	7543.8	14012.74	13728.63	11676.05	1.364	0.0051	2.294	0.487	-1.037
P1917	Cuauhtemone	C15H24O3	1.501	C09645	3017.21	2900.91	2802.08	6454.99	5519.48	6001.69	1.420	0.0055	2.258	0.485	-1.044
P1322	N,N-Dimethyl-5-methoxytrypta	C13H18N2O	2.680	C08309	55103.45	48301.56	56014.66	101631.85	111531.69	116954.93	1.426	0.0014	2.865	0.483	-1.050
P0321	Quinoline	C9H7N	1.409	C06413	16950.39	14535.59	14573.11	27746.69	35661.98	32029.15	1.391	0.0116	1.935	0.483	-1.051
P0884	D-Pantothenic Acid	C9H17NO5	2.024	C00864	7957.03	6723.18	10003.86	17931.26	14042.5	19226.79	1.335	0.0133	1.876	0.482	-1.053
P1593	pinolidoxin	C18H26O6	2.747	C08503	14865.26	9822.29	18335.58	28912.46	31452.95	28914.89	1.371	0.0165	1.782	0.482	-1.053
P0276	Doxylamine	C17H22N2O	0.861	D07878	19327.33	19107.87	22521.39	37633.51	49068.04	40302.09	1.379	0.0165	1.783	0.480	-1.059
N1900	Doripenem	C15H24N4O6S	0.734	D03895	8649.01	9640.32	11528.52	23020.68	17964.59	21518.87	1.396	0.0068	2.164	0.477	-1.068
N1741	Haloxyfop	C15H11ClF3NC	1.636	C04871	3923.75	6609.47	5852.34	11490.55	12266	10628.13	1.398	0.0059	2.230	0.477	-1.069
N0851	Tiocarbazil;Thiocarbazil	C16H25NOS	3.218	C18898	6015.59	8727.05	6286.96	12344.1	15224.65	16627.82	1.362	0.0099	2.003	0.476	-1.072
P2225	Imolamine	C14H20N4O	2.124	D07159	10636.96	12190.67	13395.34	24023.57	26734.1	25474.01	1.430	0.0003	3.547	0.475	-1.073
P1231	17Z-hexacosenoic acid	C26H50O2	7.832	C17278	1800.99	833.18	1358.76	2644.03	3018.67	2756.02	1.339	0.0222	1.653	0.474	-1.076
P1789	CITCO;6-(4-Chlorophenyl)imi	C19H12Cl3N3C	0.720	C15639	13439.83	10860.36	16019.12	29562.73	28026.71	27476.47	1.413	0.0042	2.381	0.474	-1.077
N1018	Sodium ortho-phenylphenate	C12H9O.Na	2.113	C19298	15198.18	15354.74	15575.09	31414.62	32715.65	33617.38	1.459	0.0010	2.981	0.472	-1.083
N1658	Hypoxanthine;Purine-6-ol	C5H4N4O	1.233	C00262	117505.24	98070.11	140669.23	247403.75	231897.09	282349.19	1.407	0.0025	2.599	0.468	-1.096
N1023	Sodium folinate	C20H22N7O7.N	0.809	C17731	23847.02	21644.6	26189.85	56093.83	47133.76	51094.65	1.432	0.0026	2.581	0.464	-1.106
P0181	HYPOXANTHINE	C5H4N4O	0.924	C00262	330528.47	292358.44	564755.71	1073960.94	824630.53	658366.69	1.209	0.0422	1.374	0.464	-1.106
P0955	"(8S,9S,10R,13S,14S,17S)-17	C21H30O2	8.693	C00410	28927.4	27603.98	26168.37	58357.76	62769.06	57990.92	1.439	0.0003	3.472	0.462	-1.115
P0850	gamma-L-Glutamyl-L-cysteinyl	C11H19N3O6S	1.609	C04544	734.84	1629.7	1978.72	2379.66	3063.06	3985.33	1.194	0.0491	1.309	0.461	-1.118
P0028	Progesterone	C21H30O2	8.690	C00410	103341.25	99865.25	100009.97	212385.8	226042.8	221309.44	1.444	0.0005	3.280	0.460	-1.122
P2479	3-Hydroxyhexobarbital	C12H16N2O4	1.434	C03068	12558.67	14364.74	14296.55	29680.84	29166.73	31084.48	1.440	0.0000	4.406	0.458	-1.125
P2221	Integerressine	C33H38N4O4	2.918	C10006	22657.01	20122.01	26115.38	53664.98	50760.02	47092.3	1.420	0.0005	3.345	0.455	-1.137
P2542	3beta-Hydroxy-5alpha-cholest-	C28H46O3	9.726	C04746	4608.34	5155.02	4623.62	9453.56	11648.96	10749.06	1.414	0.0079	2.102	0.452	-1.147
P2443	dTDP-4-oxo-alpha-D-xylose;d	C15H22N2O15I	0.720	C21351	18312.73	16605.18	20934.87	39932.24	41280.65	42757.83	1.435	0.0003	3.534	0.451	-1.150
N0062	cis-5,8,11,14-Eicosatetraenoic	C20H32O2	11.062	C00219	32450.79	25076.08	33195.18	67212.36	61974.6	73226.01	1.428	0.0011	2.970	0.448	-1.158
P0338	Serotonin	C10H12N2O	1.413	C00780	5818.05	5925.79	5516.78	12473.76	14548.35	11513.1	1.403	0.0143	1.846	0.448	-1.159
P1972	Alpha-Tocotrienol	C29H44O2	8.581	C14153	591.51	387.52	327.42	881.68	1131.53	908.04	1.335	0.0088	2.057	0.447	-1.161
P1483	(-)-Chimonanthine	C22H26N4	1.850	C09133	7857.98	7659.51	12109.16	20456.76	22391.4	19055.34	1.386	0.0045	2.348	0.446	-1.164
P2450	Ethambutol	C10H24N2O2	1.692	C06984	28844.08	26275.33	29585.85	60365.24	62650.89	66916.35	1.437	0.0005	3.304	0.446	-1.165
P1165	10-Formyl-7,8-dihydrofolic aci	C20H21N7O7	0.819	C03204	1204.84	2105.35	1085.08	3159.26	2814.08	3933.74	1.294	0.0164	1.784	0.444	-1.173
P1408	Tetrachlorobisphenol A;3,3',5,5	C15H12Cl4O2	0.720	C14528	16986.32	15440.77	18078.02	38260.54	37266.91	38552.5	1.442	0.0002	3.806	0.443	-1.176
N0217	(S)-Malate	C4H6O5	0.917	C00149	7340.73	8028.91	10289.77	19084.98	15584.51	23364.63	1.336	0.0279	1.554	0.442	-1.177
N1898	Drazoxolon	C10H8ClN3O2	1.208	C18929	26395.08	21492.46	24740.88	54113.65	58019.63	52150.36	1.447	0.0002	3.688	0.442	-1.178
P1457	UDP-L-Ara4FN;Uridine 5'-dip	C15H23N3O16I	2.755	C16154	5117.47	7207.66	10314.67	18300.58	16233.84	17061.19	1.372	0.0139	1.858	0.439	-1.188
N0651	Miglustat	C10H21NO4	9.093	D05032	18135.48	18985.07	15175.63	39498.83	36269.24	43574.42	1.432	0.0023	2.633	0.438	-1.190
P1833	Ceftizoxime sodium	C13H12N5O5S	0.728	C08118	4569.71	3994.13	5376.68	10681.96	9267.91	11897.6	1.390	0.0059	2.229	0.438	-1.192
N0857	Threonic acid	C4H8O5	0.842	C01620	108563.41	82520.94	75407.13	205125.52	178550.72	227486.06	1.402	0.0038	2.415	0.436	-1.197
P2332	Luteolin;3',4',5,7-Tetrahydroxy	C15H10O6	0.727	C01514	9118.04	10077.51	11903.24	24721.38	21962.89	24795.39	1.422	0.0004	3.350	0.435	-1.201
N1307	Bamethane	C12H19NO2	2.304	D07490	5506.87	5229.68	7713.36	17663.43	13524.61	11269.89	1.305	0.0356	1.449	0.435	-1.202
N0435	Terpendole E	C28H39NO3	9.093	C20536	78416.52	80458.01	65011.38	175023.08	161483.41	180878.67	1.447	0.0002	3.625	0.433	-1.208
P2304	Molybdoenzyme molybdenum	C10H12MoN5C	0.720	C18237	24616.87	24642.83	30055.11	60975.68	59516.27	62960.09	1.437	0.0004	3.434	0.432	-1.210
P2478	Hexahomomethionine	C11H23NO2S	1.791	C17233	23396.17	23007.06	28562.03	56439.57	56949.64	60235.65	1.436	0.0003	3.587	0.432	-1.212
N1123	Adrenorphin	C44H69N15O9	3.152	C16108	6625.46	10493.52	7239.69	20289.62	17866.04	18530.55	1.418	0.0033	2.481	0.430	-1.219
N0398	6-Hydroxynicotinic Acid	C6H5NO3	1.253	C01020	2538.31	2142.44	2215.79	5408.48	5201.08	5552.48	1.456	0.0001	4.289	0.427	-1.229
P2178	2,6-Dibromohydroquinone	C6H4Br2O2	2.539	C16248	4076.46	6891.18	4283.49	12704.68	13054.94	9993.79	1.348	0.0068	2.168	0.427	-1.229
P1861	Diatrizoate	C11H9I3N2O4	2.755	D02240	6038.25	9635.92	13952.87	21417.82	26781.49	21255.85	1.330	0.0117	1.930	0.427	-1.229
P1335	1,2,3,4,6,7,8,9-Octachlorodibe	C12Cl8O2	2.522	C18180	9986.31	9275.4	17730.55	33825.24	25228.53	27978.07	1.319	0.0110	1.960	0.425	-1.234
P0706	1-Stearoyl-2-hydroxy-sn-glycer	C23H48NO7P	9.910	C21484	26234.35	27702.9	24146.13	64146.96	54745.54	65019.45	1.418	0.0050	2.302	0.425	-1.236
N0992	Piperazine adipate	C6H10O4.C4H1	1.798	C13811	36021.41	43369.76	46732.41	94991.87	94940.85	108005.54	1.439	0.0007	3.156	0.423	-1.240
P2228	Imazamox	C15H19N3O4	2.090	C18598	9812.99	9919.18	14723.18	27606.14	26355.11	27438.84	1.416	0.0077	2.112	0.423	-1.240
N0254	9-Riburonosyladenine	C10H11N5O5	1.228	C11501	4486.74	3654.5	4531.43	9626.35	11465.25	8977.39	1.410	0.0088	2.054	0.421	-1.247
P1591	Physostigmine;Eserine	C15H21N3O2	3.194	C06535	9284.38	9489.56	11923.27	25765.89	22244.9	25955.24	1.416	0.0010	2.987	0.415	-1.269
P2425	Dolichotheline	C10H17N3O	1.690	C16696	19308.35	19129	26004.45	50365.83	50995.91	54774.12	1.427	0.0009	3.055	0.413	-1.277
P1219	1D-myo-Inositol 1,3,4,5-tetraki	C6H16O18P4	0.720	C01272	16923.06	16826.69	19786.78	43723.71	40694.35	45926.04	1.432	0.0004	3.424	0.411	-1.284
N1246	Benzoic acid	C7H6O2	1.523	C00180	11465.79	8543.65	9031.57	20850.39	28313.23	21574.94	1.373	0.0178	1.748	0.411	-1.284
P1193	Phenkapton	C11H15Cl2O2F	0.720	C19010	40137.57	41544.29	47937.66	100705.44	101225.75	113857.69	1.430	0.0009	3.064	0.410	-1.285
P0457	Methohexital	C14H18N2O3	2.872	C07844	6567.94	9864.02	12483.7	23335.61	23516.13	23763.16	1.407	0.0145	1.840	0.409	-1.288
P0169	Hecogenin	C27H42O4	9.286	C08902	72063.58	71070.85	67487.69	168930.46	176704.92	169811.57	1.445	0.0000	4.511	0.409	-1.291
P2532	3,4,3',4'-Tetrahydrospirilloxant	C42H64O2	2.635	C15888	4095.26	1567.26	6055.29	8206.56	10914.99	9653.78	1.281	0.0283	1.547	0.407	-1.296
P1902	4alpha-Methyl-5alpha-cholest-	C28H46O	7.799	C04453	5022.56	3220.34	5082.64	10361.12	10553.96	12282.18	1.401	0.0016	2.808	0.401	-1.317
P2445	Epivoacorine	C43H52N4O6	2.260	C09156	4633.47	3107.94	6044.46	14459.24	8548.06	11364.44	1.257	0.0382	1.418	0.401	-1.318
P1230	N8-Acetylspermidine	C9H21N3O	0.728	C01029	5510.68	6554.27	8180.74	14044.55	17535.49	18904.71	1.381	0.0082	2.088	0.401	-1.318
N1854	Chivosazole C	C47H67NO12	2.529	C15730	5648.21	4703.63	7634.96	17006.93	12971.84	15005.55	1.393	0.0045	2.350	0.400	-1.322
P0498	Lauric acid	C12H24O2	9.095	C02679	13376.35	14708.11	10121.92	33251.84	30718.82	32406.74	1.426	0.0009	3.025	0.396	-1.335
P2224	3-Aminopropane-1,2-diol;(R)-3	C3H9NO2	1.546	C06057	5698.47	2791.25	5271.72	13431.66	8490.89	12933.03	1.280	0.0270	1.568	0.395	-1.341
N0985	Ponceau MX;Acid Red 26	C18H14N2O7S	0.734	C19294	26549.47	22140.66	26529.89	65477.83	58051.02	67563.43	1.444	0.0013	2.875	0.394	-1.345
P1680	Sulfoacetate;Sulfoacetic acid	C2H4O5S	0.727	C14179	12171.77	11504.25	13988.19	31252.68	29128.79	35352.52	1.418	0.0038	2.426	0.393	-1.346
P1443	Usambarensine	C29H28N4	3.014	C09249	6505.55	3890.32	7926.98	16979.43	13691.83	16017.29	1.373	0.0039	2.405	0.392	-1.349
P1696	1-Linoleoylglycerophosphocho	C26H51NO7P	8.438	C04100	4272.78	4680.69	3892.83	9684.9	11529	11577.08	1.422	0.0043	2.371	0.392	-1.352
P0342	Spermine	C10H26N4	2.016	C00750	27023.78	21571.02	36912.45	79791.19	58730.32	79804.51	1.351	0.0094	2.027	0.392	-1.352
P1531	Tropinone	C8H13NO	1.796	C00783	4697.11	5869.37	7087.74	15769.34	16199.25	14245.87	1.422	0.0005	3.276	0.382	-1.388
P1475	Vindesine	C43H55N5O7	3.287	D06304	6085.86	4439.16	10177.26	18808.53	16060.68	19652.3	1.361	0.0082	2.088	0.380	-1.397
P2463	Ecgonine methyl ester	C10H17NO3	1.350	C12448	98976.71	84253.24	115429.54	257494.35	244191.91	285258.17	1.423	0.0006	3.201	0.380	-1.398
P1734	Saphenamycin	C23H18N2O5	2.115	C12311	3437.3	7710.76	9152.5	16563.18	19110.81	17900.36	1.376	0.0126	1.899	0.379	-1.400
N0300	N.epsilon.-Acetyl-L-lysine	C8H16N2O3	1.440	C02727	72220.29	78975.41	81002.98	203359.21	185441.57	224108.71	1.441	0.0055	2.261	0.379	-1.400
P1660	1-(beta-D-Ribofuranosyl)-1,4-c	C11H16N2O5	1.472	C15497	1008.61	935.02	1625.31	4121.01	2429.83	2888.47	1.254	0.0442	1.355	0.378	-1.403
N1629	Flavanone;2,3-Dihydroflavone	C15H12O2	0.921	C00766	7290.82	4857.67	7278.66	18839.72	12705.53	19946.96	1.337	0.0302	1.520	0.377	-1.406
P1936	D and C Red No. 9;C.I. Pigmen	(C17H12ClN2C	3.014	C19382	5522.54	3769.91	7028.6	15922.27	13180.73	14266.18	1.392	0.0021	2.684	0.376	-1.410
N0554	Menatetrenone	C31H40O2	9.375	D00100	10629.61	12426.6	9357.52	27939.72	27622.14	30876.93	1.448	0.0002	3.660	0.375	-1.415
N1587	Gentioflavin	C10H11NO3	0.801	C09962	4485.98	5042.54	3590.36	12061.92	10122.83	12825.37	1.422	0.0039	2.409	0.375	-1.416
N0945	Pyridine;Azabenzene	C5H5N	0.917	C00747	18329.31	14219.95	18310.08	46363.33	40531.82	49620.71	1.433	0.0025	2.606	0.373	-1.424
N1942	Dehydrofalcarinone	C17H20O	1.698	C08445	3728.87	3817.44	6670.67	13298.06	11819.73	13129.82	1.413	0.0057	2.246	0.372	-1.428
N0835	Vindesine	C43H55N5O7	3.297	D06304	4320.18	3106.69	6779.25	13199.56	11703.98	13361.77	1.401	0.0076	2.119	0.371	-1.430
P1244	Orthophosphate;Phosphate;Pho	H3PO4	0.728	C00009	9287.79	9638.82	10258.77	26732.49	25228.84	27552.14	1.441	0.0004	3.414	0.367	-1.446
P1971	AM-251	C22H21Cl2IN4	2.793	C20220	3835.86	4545.95	7349.36	13754.13	16339.03	12813.93	1.376	0.0038	2.417	0.367	-1.448
P2209	L-Capreomycidine;(2S,3R)-Ca	C6H12N4O2	1.417	C18472	3761.71	2299.37	4330.02	6414.13	11496.14	10467.95	1.273	0.0464	1.333	0.366	-1.449
P2378	Farrerol	C17H16O5	1.393	C09734	8269.31	4728.87	11003.3	19060.28	17728.8	28858.26	1.260	0.0391	1.408	0.366	-1.452
N1034	Sipeimine	C27H43NO3	9.666	C10808	4915.69	5706.98	4295.05	15379.9	11558.97	14219.1	1.413	0.0095	2.023	0.362	-1.464
P1983	7-Hydroxy-1,2,3,6,8-pentachlo	C12H3Cl5O2	1.817	C15244	4159.57	3050.27	9790.91	18694.42	12224.37	16084.51	1.258	0.0240	1.620	0.362	-1.467
P1005	(24R,24(1)R)-Fucosterol epoxi	C29H48O2	8.695	C03910	8381.94	9237.96	10077.45	25952.43	24234.23	26559.38	1.439	0.0001	4.022	0.361	-1.470
N1522	3-Chloro-D-alanine	C3H6ClNO2	11.959	C02634	448234.37	447610.02	435968.8	1168716.24	1366229.72	1160602.1	1.442	0.0070	2.152	0.360	-1.472
P1694	S-Decyl GSH;S-Decyl glutathi	C20H37N3O6S	1.999	C11303	14132.69	5313.72	21267.95	42969.17	29370.88	40904.1	1.281	0.0184	1.736	0.360	-1.476
N1941	D-(+)-Glyceric acid; LC-ESI-Q	C3H6O4	0.875	C00258	16749.37	16711.05	18949.67	46570	47646.44	52432.9	1.452	0.0010	2.999	0.357	-1.484
P0713	1-Stearoyl-2-hydroxy-sn-glycer	C23H48NO7P	9.917	C21484	5801.49	4181.22	4217.11	13756.7	11713.67	14330.78	1.409	0.0016	2.802	0.357	-1.487
P0763	1-Stearoyl-2-hydroxy-sn-glycer	C23H48NO7P	9.377	C21484	34242.89	40966.29	31883.18	100500.38	91663.25	109052.77	1.424	0.0013	2.886	0.356	-1.492
N1592	GDP-4-acetamido-4,6-dideoxy	C18H28N6O15I	1.241	C20672	12373.05	2483.82	13882.23	30163.64	26366.13	24619.04	1.333	0.0244	1.612	0.354	-1.498
N0418	Fructose 1,6-diphosphate	C6H14O12P2	1.214	C00354	13699.18	10287.29	41655.78	75278.99	64924.58	46045.85	1.224	0.0385	1.414	0.352	-1.505
P1078	Phylloquinol	C31H48O2	8.206	C03313	20191.25	18516.32	21575.21	56700.54	61240.11	53340.38	1.434	0.0014	2.863	0.352	-1.507
P2065	Calcidiol;25-Hydroxyvitamin I	C27H44O2	7.267	C01561	28995.2	27177.77	26863.2	74071.85	85201.1	78460.97	1.300	0.001	2.870	0.349	-1.518
P0896	Solanidine	C27H43NO	10.266	C06543	27090.05	30499.51	28559.44	87722.24	72511.68	86479.44	1.420	0.0064	2.191	0.349	-1.518
P2024	Adenophostin B	C18H28N5O19I	0.721	C13722	4088.37	4244.19	5791.02	13895.07	12635.53	14332.05	1.427	0.0003	3.544	0.346	-1.533
N0668	N-Acetylaspartylglutamic acid	C11H16N2O8	1.557	C12270	3214.02	1819.35	2858.99	7286.9	6037.4	9513.29	1.339	0.0259	1.586	0.346	-1.533
N1955	Decaline;Decalin	C26H31NO5	2.529	C10589	12445.59	12828.25	18444.21	47718.74	36635.92	42168.19	1.413	0.0037	2.430	0.346	-1.533
P1706	Rimantadine	C12H21N	0.802	C07236	16361.84	10055.72	15958	39112.01	42523.97	41378.86	1.427	0.0015	2.828	0.344	-1.538

N1088	Ricinoleic acid	C18H34O3	7.531	C08365	4594.67	3451.91	5149.12	12496.98	13285.4	12550.78	1.450	0.0007	3.164	0.344	-1.539
N0103	Nitrilotriacetic acid	C6H9NO6	2.653	C14695	67641	72418.86	54079.12	177261.73	197191.61	193207.43	1.451	0.0001	3.930	0.342	-1.548
N1134	Acetylphosphate	C2H5O5P	1.208	C00227	19080.7	15366.88	19203.62	65425.42	44981.47	47032.51	1.367	0.0297	1.527	0.341	-1.553
P1976	Antimony trioxide	Sb2O3	2.397	C19192	5153.2	4898.04	5512.44	13426.78	15729.19	16538.29	1.425	0.0069	2.163	0.341	-1.554
P1278	N-Methylindolo[3,2-b]-5alpha-	C34H51N	8.704	C15294	19546.12	16827.88	17505.44	51460.06	57054.81	50415.19	1.436	0.0012	2.939	0.339	-1.561
P1828	CETRIMONIUM	C19H41N	3.190	D07664	6965.57	3988.87	5143.22	16440.95	12979.3	18189.72	1.369	0.0081	2.092	0.338	-1.564
N1632	Feruloylputrescine;Subaphyllin	C14H20N2O3	2.682	C10497	10053.76	11027.79	8531.1	29044.17	30022.13	30117.2	1.459	0.0002	3.686	0.332	-1.591
P1592	Pipecuronium	C35H62N4O4	11.431	C07554	27398.73	28212.38	27232.99	87664.85	83309.53	79312.31	1.440	0.0016	2.791	0.331	-1.595
N0567	L-tyrosine methyl ester 4-sulfat	C10H13NO6S	2.246	C04201	2351.49	4643.74	4621.69	10124.04	12529.22	12684.4	1.408	0.0022	2.654	0.329	-1.605
N0303	Pyruvaldehyde	C3H4O2	1.233	C00546	5323.18	2121.05	11726.93	21647.3	16966.02	19762.88	1.319	0.0272	1.566	0.328	-1.606
N0913	Progabide	C17H16ClFN2C	5.013	D05621	3555.7	3255.22	3642.06	10306.94	10982.84	11269.18	1.458	0.0004	3.450	0.321	-1.639
N1007	Phenol	C6H6O	3.592	C00146	2023.06	1053.44	2580.4	4183.75	7499.21	5967.45	1.291	0.0358	1.446	0.320	-1.642
N1667	HLo7	C15H17N5O4.2	0.734	C07596	8267.77	6334.01	12232.84	28726.98	26729.97	28563.16	1.436	0.0039	2.407	0.319	-1.647
N1846	Chlorfenapyr	C15H11BrClF3	0.735	C18455	1132.23	1426.03	1330.26	4576.21	3868.35	3882.07	1.442	0.0031	2.509	0.315	-1.664
P2262	Diosgenin;(3beta,25R)-Spirost	C27H42O3	9.153	C08898	4614.04	7015.49	5180.09	14663.74	19600.83	19443.34	1.395	0.0079	2.100	0.313	-1.676
N0247	Glycodeoxycholic acid	C26H43NO5	9.284	C05464	6582.89	7357.72	5840.7	20690.4	21284.98	21283.85	1.461	0.0001	3.860	0.313	-1.677
P2538	3alpha,7alpha,12alpha,26-Tetra	C27H48O4	8.712	C05446	327393.98	295703.69	322637.4	1017509.41	1010329.24	1000640.78	1.445	0.0000	4.951	0.312	-1.679
P2184	L-Citronellol	C10H20O	3.977	C09849	1182.09	1712.14	1377.85	4672.24	5122.4	3907.04	1.405	0.0054	2.265	0.312	-1.681
P1934	Dasytrichone	C18H16O4	1.283	C10035	11565.8	4960.31	12694.6	34101.14	23425.21	37508.08	1.319	0.0182	1.739	0.307	-1.701
P1143	N-Acetyl-L-phenylalanyl-3,5-d	C20H20I2N2O5	0.727	C04418	15559.79	17849.05	19843.43	60357.82	52575.41	60650.74	1.431	0.0011	2.955	0.307	-1.705
P0372	Propamocarb	C9H20N2O2	0.909	C18885	6201.83	6177.56	8073.08	20652.49	24577.08	22383.85	1.430	0.0010	2.999	0.302	-1.725
N1422	1-Hydroxy-2-methyl-2-butenyl	C5H12O8P2	1.245	C11811	4644.45	5120.12	4773.63	16638.05	14249.59	17284.78	1.444	0.0058	2.238	0.302	-1.728
N1142	9-Methylthiononanaldoxime	C10H21NOS	1.806	C17253	6034.61	3683.35	2186.88	12435.03	14637.65	13383.17	1.412	0.0042	2.374	0.294	-1.765
P1641	Telaprevir	C36H53N7O6	3.154	D09012	3422.32	3362.35	3927.25	11918.15	10916.01	13609.19	1.422	0.0061	2.212	0.294	-1.766
P0559	Niacin (Nicotinic acid)	C6H5NO2	1.019	C00253	101296.54	67266.09	88019.26	242906.61	360648.26	273164.02	1.367	0.0214	1.669	0.293	-1.773
N1863	Dimethomorph	C21H22ClNO4	0.826	C18583	10700.29	5232	7862.07	31446.61	29133.04	21249.21	1.377	0.0116	1.937	0.291	-1.782
N1910	Dinex	C12H14N2O5	2.662	C19038	10311.56	8073.67	9359.13	27557.47	34210.37	33659.21	1.435	0.0053	2.276	0.291	-1.782
P2570	3-Oxovalproic acid	C8H14O3	1.276	C16652	19989.59	15636.13	18437.2	59536.47	66654.2	59769.63	1.437	0.0004	3.394	0.291	-1.782
P0168	Harpagoside	C24H30O11	1.401	C09783	3811.6	2772.23	7826.33	16907.36	13863.64	19079.98	1.359	0.0054	2.265	0.289	-1.791
N1619	Epidihydrocholesterin;Epichole	C27H48O	8.681	C12978	3427.86	1801.86	2308.53	8889.93	9268.69	7965.78	1.435	0.0007	3.162	0.289	-1.793
P1665	Squalene;Spinacene;Supraene	C30H50	9.344	C00751	2590.1	2252.39	3293.15	9251.64	8305.79	10738.59	1.429	0.0058	2.233	0.288	-1.798
N1231	Boc-Asn;N-alpha-Boc-L-aspara	C9H16N2O5	0.892	C01410	7149.63	6889.86	7858.11	23768.6	19836.86	33393.24	1.341	0.0442	1.354	0.284	-1.814
N1058	SUCCINYLSULFATHIAZOL	C13H13N3O5S	0.842	C11745	1014.14	1491.42	2408.92	4668.23	5258.38	7604.92	1.325	0.0271	1.566	0.280	-1.835
N0856	Ticagrelor	C23H28F2N6O	0.958	D09017	416.28	914.5	957.02	3300.3	2458.94	2403.42	1.384	0.0080	2.095	0.280	-1.835
N0993	Pinocembrin 7-rhamnosylgluco	C27H32O13	0.817	C09828	12724.78	11018.78	472.39	31343.96	29614.33	25749.98	1.359	0.0195	1.711	0.279	-1.840
P1442	UTP;Uridine 5'-triphosphate;U	C9H15N2O15P	0.728	C00075	5507.71	3267.69	4650.94	15957.98	16415.94	15925.17	1.436	0.0020	2.700	0.278	-1.847
P1257	Norspermine;N,N'-Bis(3-amino	C9H24N4	8.164	C21004	21020.62	19253.82	20024.07	51607.42	80988.84	85746.75	1.347	0.0384	1.415	0.276	-1.856
P2014	AK-toxin I	C23H27NO6	0.938	C16787	8815.91	1896.87	5314.98	18877.41	26786.78	13434.18	1.236	0.0462	1.336	0.271	-1.883
P0934	alpha-Isopropylmalate;(2S)-2-I	C7H12O5	1.717	C02504	78938.25	75789.96	75193.65	274847.45	296069.69	287709.4	1.445	0.0006	3.221	0.268	-1.901
N0994	Physovenine	C14H18N2O3	2.936	C09232	5469.15	3090.18	6513.21	24020.41	13833.42	18769.2	1.336	0.0314	1.503	0.266	-1.909
P0771	SOLASODINE	C27H43NO2	9.913	C10822	3777.75	3349.68	2267.26	13496.27	8718.45	13163.43	1.351	0.0230	1.639	0.266	-1.913
P0219	Adenosine	C10H13N5O4	2.656	C00212	5692.91	6540.57	4103.71	19183.21	23415.68	19019.7	1.416	0.0028	2.551	0.265	-1.915
N1712	Glutathione disulfide;GSSG;O	C20H32N6O12S	0.925	C00127	1530.45	967.88	1653.65	4473.08	5618.01	5740.12	1.424	0.0033	2.483	0.262	-1.931
P1637	Pithecolobine	C22H46N4O	8.563	C10611	70448.82	61562.6	65544.78	263071.61	246219.47	258328.99	1.443	0.0001	4.220	0.257	-1.958
N0523	Maleamic acid	C4H5NO3	1.258	C01596	3463.81	7163.72	3115.68	16140.68	18349.74	19072.03	1.425	0.0018	2.739	0.257	-1.963
P1658	Sipeimine	C27H43NO3	9.502	C10808	3392.81	3383.93	2474.88	12166.96	10430.49	13581.16	1.413	0.0057	2.243	0.256	-1.967
P1524	(1R)-Hydroxy-(2R)-glutathiony	C20H25N3O7S	11.955	C14791	14301.8	28276.38	38501.01	133003.3	87800.3	96584.32	1.342	0.0152	1.818	0.255	-1.969
P0824	Solanidine	C27H43NO	10.266	C06543	7656.45	4096.81	4211.35	21782.72	19076.85	21898.62	1.418	0.0006	3.213	0.254	-1.975
N0259	Glycodeoxycholic acid	C26H43NO5	9.284	C05464	2105.61	1291.8	2058.01	5755.85	8163.14	7630.48	1.405	0.0107	1.969	0.253	-1.982
P2555	Gomisin H	C23H30O7	1.908	C17820	177.59	53436.67	69292.99	154363.25	168228.41	163924.54	1.369	0.0249	1.604	0.253	-1.985
P1623	Primary fluorescent chlorophyl	C35H40N4O7	1.953	C18098	3039.58	2477.57	6083.47	18438.74	12115.99	15375.11	1.351	0.0099	2.006	0.253	-1.985
P1580	Pseudopelletierine	C9H15NO	0.786	C10865	2657.76	3500.07	1791.88	11175.97	10524.13	10156.55	1.430	0.0005	3.301	0.250	-2.003
P0307	D-Ala-D-Ala	C6H12N2O3	1.661	C00993	29874.82	35125.11	36614.92	126755.42	146232.82	138627.25	1.439	0.0011	2.942	0.247	-2.018
P1975	Anthranilate;Anthranilic acid;o	C7H7NO2	2.660	C00108	3849.89	7301.75	3830.64	21046.88	22701.41	17114.06	1.399	0.0025	2.596	0.246	-2.022
N0599	Methazole	C9H6Cl2N2O3	0.734	C19123	4442.51	2871.82	4653.95	16696.99	15240.71	16945.28	1.452	0.0001	4.030	0.245	-2.030
N1748	Guanosine 3'-diphosphate 5'-tri	C10H18N5O20P	0.736	C04494	1851.07	2010.18	2481.43	10361.79	7432.15	8188.33	1.412	0.0143	1.845	0.244	-2.034
P1974	alpha-TOCHOPHEROL	C29H50O2	8.563	C02477	9804.46	9279.15	9057.83	37756.02	37894.08	40074.52	1.445	0.0003	3.581	0.243	-2.040
N0622	N-tert-Butyloxycarbonyl-deace	C23H44N6O5	10.264	C11604	4283.62	3799.43	1926.69	14253.5	11909.37	15876.33	1.420	0.0028	2.546	0.238	-2.070
P0373	Propanil	C9H9Cl2NO	1.617	C14229	42452.12	32519.69	36522.29	175357.07	125997.69	169408.83	1.395	0.0141	1.851	0.237	-2.078
P2036	Alloxanthine;Oxipurinol;Oxyp	C5H4N4O2	1.650	C07599	13762.02	11961.79	14521.77	56550.27	61571.02	54255.58	1.439	0.0009	3.043	0.233	-2.099
N0269	S-Ribosyl-L-homocysteine;S-D	C9H17NO6S	1.258	C03539	3266.74	4032.49	652.62	11239.17	10918.37	12423.11	1.420	0.0056	2.254	0.230	-2.121
P1715	Quercetin 3,3'-bissulfate	C15H10O13S2	2.589	C01155	3674.79	959.14	5542.17	17469.78	10810.65	16415.97	1.326	0.0137	1.863	0.228	-2.135
N1270	Bumetanide	C17H20N2O5S	0.975	D00247	717.85	204.46	153.44	1851.69	1097.32	1792.71	1.313	0.0182	1.741	0.227	-2.140
N1155	alpha-Ribazole;N1-(alpha-D-R	C14H18N2O4	2.022	C05775	3803.45	2287.96	4829.65	16972.11	12738.63	18889.15	1.398	0.0112	1.951	0.225	-2.154
P1303	2-Amino-5-chlorophenol	C6H6ClNO	1.717	C20669	92413.62	69513.8	86911.62	382266.24	360783.03	376133.73	1.443	0.0000	5.158	0.222	-2.169
N0664	N-Acetyl-ala-ala-ala-methyleste	C12H21N3O5	1.237	C11329	2242.54	2969.08	3040.98	13334.75	13156.34	10725.17	1.440	0.0044	2.356	0.222	-2.173
P1732	1-Hexadecyl hexadecanoate	C32H64O2	6.029	C13821	1161.67	35386.99	47386.14	117516.99	129531.11	133533.24	1.391	0.0119	1.925	0.221	-2.181
P2456	Epidihydrocholesterin;Epichole	C27H48O	7.769	C12978	1380.16	62.44	172.47	2570.82	2385.69	2541.52	1.322	0.0411	1.386	0.215	-2.215
P2503	IAARh123;[125I]Iodoaryl azid	C28H19IN5O5	0.720	C11599	4542.03	3509.09	4195.99	19539.77	16789.96	20528.39	1.428	0.0035	2.458	0.215	-2.215
P0316	Schizandrin A	C24H32O6	3.165	C16951	1887.49	705.29	3344.12	11327.38	6178.06	10682.22	1.298	0.0283	1.549	0.211	-2.247
P0868	3-Dehydrosphinganine;3-Dehy	C18H37NO2	7.051	C02934	7946.61	5361.74	6794.25	34761.72	28402.57	32385.28	1.426	0.0020	2.697	0.210	-2.249
P1215	1D-myo-Inositol 1,3,4,5,6-pent	C6H17O21P5	0.728	C01284	4651.64	5333.17	4476.2	24030.81	21435.78	24586.95	1.437	0.0015	2.811	0.206	-2.276
P1944	D-Fructose 1,6-bisphosphate	C6H14O12P2	1.210	C00354	5983.55	6006.61	10242.39	30565.7	31783.21	45827.62	1.366	0.0211	1.675	0.206	-2.283
P0805	Cholesterol	C27H46O	9.286	C00187	14636.61	12858.83	12442.8	62234.54	61893.58	71797.29	1.445	0.0003	3.504	0.204	-2.294
P0564	N,N-Dimethylsphingosine	C20H41NO2	6.993	C13914	5075.43	5464	2728.37	25404.95	24197.85	18194.4	1.396	0.0079	2.105	0.196	-2.353
N1642	Fenothiocarb sulfoxide	C13H19NO3S	1.985	C11085	1224.3	1699.57	1375.23	6587.05	8472.34	7032.8	1.435	0.0065	2.189	0.195	-2.361
P1843	Cefonicid sodium	C18H16N6O8S	0.728	C08105	2424.65	2493.21	5423.65	18970.9	15767.01	18410.18	1.419	0.0005	3.285	0.195	-2.362
N0019	Xanthosine	C10H12N4O6	1.656	C01762	23403.1	21840.98	24727.59	123687.8	133736.26	118242.38	1.457	0.0015	2.832	0.186	-2.425
P0970	N5-Phenyl-L-glutamine;gamma	C11H14N2O3	1.958	C20843	2134.71	866.9	660.15	4395.71	8225.07	7224.31	1.320	0.0290	1.537	0.185	-2.438
N0362	Fructose 1,6-diphosphate	C6H14O12P2	1.199	C00354	7331.82	9572.54	10314.07	59905.56	39325.03	49984.03	1.405	0.0188	1.725	0.182	-2.455
P2144	betulin	C30H50O2	8.339	C08618	1024.26	549.85	1629.15	7178.05	5658.2	5117.37	1.389	0.0060	2.224	0.178	-2.487
N0974	Perfluorooctylsulfonyl fluoride	C8F18O2S	0.735	C18143	466.3	1805.43	1504.05	6620.8	6709.28	7945.84	1.435	0.0006	3.227	0.177	-2.494
N1335	1D-myo-Inositol 1,3,4-trisphos	C6H15O15P3	0.744	C01243	722.8	630.74	1079.57	4189.43	4413.53	5154.84	1.443	0.0017	2.764	0.177	-2.499
N0365	Fructose 1,6-diphosphate	C6H14O12P2	1.216	C00354	11367	17015.65	25886.6	111593.26	81823.15	117322.01	1.412	0.0088	2.054	0.175	-2.517
N0392	Fructose 1,6-diphosphate	C6H14O12P2	1.216	C00354	3837.74	3450.29	9379.64	34070.62	25033.38	36721.56	1.400	0.0066	2.177	0.174	-2.523
N0833	Voglibose	C10H21NO7	0.925	D01665	9826.81	2809.35	3777.84	29263.96	29488.35	41992.79	1.387	0.0095	2.023	0.163	-2.618
P0101	Gibberellin A4	C19H24O5	3.669	C11864	536.57	958.84	549.24	3268.41	4827.41	4457.3	1.398	0.0122	1.915	0.163	-2.618
P1683	Sulfanilic acid; LC-ESI-ITFT;	C6H7NO3S	1.224	C06335	4520.56	3710.58	5787.98	23910.52	35817.12	26665.91	1.389	0.0192	1.717	0.162	-2.624
P0888	D-Octopine;N2-(D-1-Carboxye	C9H18N4O4	1.133	C04137	1394.46	2059.1	10965.8	26182.12	34987.86	30875.38	1.387	0.0033	2.479	0.157	-2.674
P1516	(+)-.gamma.-Tocopherol	C28H48O2	8.164	C02483	996.19	2244.84	1350.51	6901.14	10962.47	12490.28	1.351	0.0307	1.513	0.151	-2.725
N0673	MTIC	C5H8N6O	1.656	C16250	2981.1	5704.47	6111.73	30984.58	33595.93	33313.8	1.457	0.0000	4.465	0.151	-2.726
P2161	Laurylaldehyde	C12H24O	6.526	C02278	6915.82	6174.07	6721.49	42507.52	42518.21	47068.96	1.442	0.0014	2.869	0.150	-2.737
P0995	2-Hydroxy-6-ketononatrienedic	C9H8O6	1.617	C12624	2331.75	2128.2	3190.98	18205.06	14525.1	19285.28	1.417	0.0070	2.152	0.147	-2.765
P0324	Rubrofusarin	C15H12O5	1.949	C09047	22251.09	23748.67	22481.06	149973.7	165810.89	154300.19	1.444	0.0011	2.946	0.146	-2.779
P2405	Fluoxetine	C17H18F3NO	1.268	D00326	363.79	257.7	5196.51	13524.49	14810.72	12486.85	1.387	0.0101	1.997	0.143	-2.811
P2507	Hydroxyzine 2HCl	C21H29Cl3N2C	12.078	D00672	607.28	582.45	1146.22	5327.21	6254.46	4888.49	1.423	0.0024	2.621	0.142	-2.818
N1954	D-(+)-Raffinose; LC-ESI-QTO	C18H32O16	0.830	C00492	3014.71	2522.53	4216.11	23685.6	20714.41	24376.99	1.452	0.0008	3.085	0.142	-2.818
N0069	Lumichrome	C12H10N4O2	4.423	C01727	1619.93	1188.84	1511.7	9707.07	11436.5	9368.37	1.447	0.0041	2.392	0.142	-2.820
P1723	Sepiolite	Mg4(OH)2(Si2C	1.949	C19522	760.89	448.4	1678.62	8334.86	4911.27	7219.74	1.354	0.0186	1.730	0.141	-2.825
P1363	Tiludronate	C7H9ClO6P2S	0.728	C08141	1298.39	1157.95	566.99	6312.71	8405.32	7047.94	1.418	0.0048	2.322	0.139	-2.848
P1851	Dicofol;Kelthane	C14H9Cl5O	0.728	C14301	2325.96	1344.53	1166.49	10739.23	11224.41	12987.01	1.430	0.0009	3.032	0.138	-2.853
P1159	N-Dodecane	C12H26	5.622	C08374	1565.43	1546.42	1087.95	12301.95	10259.52	8016.21	1.387	0.0180	1.746	0.137	-2.864
P1407	Tetradecanol	C14H30O	6.037	D05097	68031.98	69340.04	67578.75	474513.28	499725	520476.55	1.445	0.0009	3.027	0.137	-2.867
N0094	N-Acetylaspartylglutamic acid	C11H16N2O8	1.258	C12270	59601.29	15842.13	19857.69	241412.12	245121.92	216632.57	1.444	0.0006	3.204	0.136	-2.883
P1160	13-beta-D-Glucosyloxydocosan	C28H54O8	0.913	C04103	9370.68	1725.53	9191.34	41561.78	69881.5	38377.98	1.309	0.0427	1.369	0.135	-2.885
N0921	Primary fluorescent chlorophyl	C35H40N4O7	2.932	C18098	2139.6	1381.73	2494.16	16449.6	10417.58	19288.56	1.363	0.0344	1.463	0.130	-2.940
P2138	BITHIONOL	C12H6Cl4O2S	1.772	C07967	1034.12	361.19	1511.6	10078.49	5017.73	8178.36	1.315	0.0385	1.414	0.125	-3.001
P0560	NG,NG-Dimethyl-L-arginine	C8H18N4O2	1.961	C03626	10075.3	19803.62	30874.13	184191.46	131123.63	175956.43	1.403	0.0101	2.152	0.124	-3.015
P2505	3-Fluoro-cis,cis-muconate	C6H5FO4	1.717	C16474	2536.16	3643.44	1163.43	15116.29	19034.65	25929.44	1.361	0.0260	1.585	0.122	-3.032

P0347	Tacrine	C13H14N2	4.425	C01453	1701.2	1090.84	682.23	10018.69	10670.94	10581.57	1.442	0.0000	4.440	0.111	-3.170
P0411	Xanthosine	C10H12N4O6	1.650	C01762	2851.84	5873.09	3957.09	37500.13	42639.03	34509.52	1.432	0.0020	2.701	0.111	-3.176
N1858	Cellobionate;Cellobionic acid;4	C12H22O12	0.834	C20888	15934.12	354.89	13999.86	89518.01	90839.2	93867.27	1.452	0.0022	2.662	0.110	-3.178
P1245	Octane	C8H18	6.253	C01387	2119.61	3198.4	2152.47	21325.37	24400.99	22163.68	1.441	0.0006	3.202	0.110	-3.184
N1594	Flufenacet	C14H13F4N3O2	2.130	C18731	6760.41	8100.32	6418.87	48316.54	90934.59	61597.83	1.347	0.0413	1.384	0.106	-3.239
P1909	Cyclohexylamine;Cyclohexana	C6H13N	6.037	C00571	3230.09	3717.27	3534.68	32901.71	32751.9	34955.75	1.445	0.0004	3.446	0.104	-3.263
P2254	Istamycin Y0	C14H30N4O4	2.165	C17985	6465.43	99.29	226.87	20436.95	23868.46	21376.21	1.406	0.0040	2.395	0.103	-3.274
P1793	Chlorodifluoromethane	CHClF2	1.720	C19361	1775.6	1828.19	2403.56	21061.3	20259.02	17560.83	1.435	0.0027	2.563	0.102	-3.293
P0798	Guanosine-3',5'-cyclic monoph	C10H12N5O7P	2.131	C00942	41416.82	51144.91	43071.17	411438.07	538565.51	421950.22	1.421	0.0093	2.030	0.099	-3.338
N1084	Robenidine	C15H13Cl2N5	1.216	D08486	469.69	1252	761	8873.3	9474.82	7972.48	1.452	0.0005	3.300	0.094	-3.406
P1492	Tridemorph	C19H39NO	7.050	C11285	1431.97	1163.61	1678.99	15146.88	15223.88	16691.62	1.443	0.0005	3.263	0.091	-3.461
N0330	Xanthosine	C10H12N4O6	1.656	C01762	1899.05	631.22	818.08	11748	14469.31	13001.71	1.447	0.0009	3.027	0.085	-3.550
P0791	Guanosine-3',5'-cyclic monoph	C10H12N5O7P	2.132	C00942	6120.77	5714.16	4808.06	54559.04	83227.49	61355.99	1.395	0.0194	1.711	0.084	-3.581
N1145	Agroclavine	C16H18N2	1.914	C09023	3347.48	247.02	93.31	14547.55	16150.01	14531	1.442	0.0014	2.842	0.082	-3.616
P1629	Ponceau MX;Acid Red 26	C18H14N2O7S2	0.728	C19294	1144.83	631.79	2071.98	17231.29	14586.75	16191.76	1.435	0.0004	3.421	0.080	-3.641
P2498	3-Dehydrosphinganine	C18H37NO2	6.660	C02934	144568.01	142090.4	138835.73	2022804.38	1802885.57	1707215.49	1.436	0.0030	2.524	0.077	-3.701
P2150	6-Deoxocastasterone	C28H50O4	9.078	C15802	3489.56	27899.79	1798.55	151427.45	150300.7	161466.68	1.435	0.0010	2.991	0.072	-3.803
N0636	NAc-DNP-Cys;N-Acetyl 2,4-d	C11H11N3O7S	2.130	C11605	732.92	823.71	264.33	6084.36	11467.25	7910.7	1.356	0.0366	1.437	0.072	-3.806
P1371	(E)-4,8-Dimethyl-1,3,7-nonatri	C11H18	6.752	C21795	22339.86	22204.99	23058.78	232342.06	355686.85	398849.83	1.381	0.0255	1.594	0.069	-3.868
P1462	(-)-Solenopsin A	C17H35N	6.818	C16782	1157.78	968.63	536.83	9028.74	13710.77	17330.97	1.354	0.0345	1.462	0.066	-3.911
P1745	cis-.DELTA.2-11-Methyldodec	C13H24O2	8.945	C18206	754.37	606.8	852.7	9118.07	9156.19	15254.62	1.350	0.0360	1.444	0.066	-3.921
P1316	N1,N12-Diacetylspermine	C14H30N4O2	0.819	C03413	2345.97	2682.37	2290.92	39686.01	42113.99	40719.84	1.446	0.0002	3.635	0.060	-4.065
N0582	Levosimendan	C14H12N6O	1.150	D04720	2249.81	2048.58	1024.19	30429.04	25477.02	34487.43	1.440	0.0074	2.134	0.059	-4.086
P2223	Isopentenyl phosphate	C5H11O4P	1.492	C20345	2113.22	2089.78	640.24	28110.38	31057.86	30784.46	1.443	0.0001	3.944	0.054	-4.215
P1886	Didecyldimethylammonium ch	C22H48N.Cl	6.802	C15436	1288.49	521.39	1295.72	41623.7	40816.38	42792.01	1.446	0.0000	4.765	0.025	-5.334
P2267	2-Hydroxyfelbamate	C11H14N2O5	1.704	C16582	271.39	193.4	852.6	20727.33	23750.19	16281.97	1.413	0.0112	1.950	0.022	-5.527
P0249	Dinitramine	C11H13F3N4O4	2.248	C18785	499.05	626.54	149.42	25806.69	25314.97	22089.98	1.439	0.0021	2.683	0.017	-5.843
N1138	acanthoside B	C28H36O13	0.817	C10890	1794.15	944.06	2401.1	117407.92	108866.36	99956.05	1.457	0.0021	2.682	0.016	-5.988
P0428	terpinolene	C10H16	6.843	C06075	184.39	228.57	191.66	21327.16	22715.74	24677.19	1.442	0.0018	2.739	0.009	-6.829
P0074	Phenethyl alcohol	C8H10O	2.149	C05853	494.91	420.47	388.64	70588.49	75507.32	69327.52	1.445	0.0007	3.158	0.006	-7.368

Table S2 Differentially regulated metabolites in shDPP4 vs. scr comparison in JAR/MTX

Index	Compounds	Formula	RT(min)	cpd_ID	shDPP4-1	shDPP4-2	shDPP4-3	Scr-1	Scr-2	Scr-3	VIP value	p_value	-log10(P)	Fold_Change	Log2FC
N0385	Maleate	C4H4O4	0.827	C01384	23823.74	28364.07	28400.15	158.87	141.28	382.38	1.438	0.0032	2.499	118.072	6.884
N0676	N-(Phosphonomethyl)glyc	C3H8NO5P	0.850	C01705	34647.64	38302.72	36504.48	911.53	127.26	1438.5	1.445	0.0002	3.613	44.183	5.465
P1732	1-Hexadecyl hexadecanoat	C32H64O2	6.029	C13821	17853.33	15131.94	13877.07	554.96	769.66	379.64	1.487	0.0057	2.247	27.497	4.781
N0605	N-Methylethanolaminium	C3H10NO4P	0.843	C01210	14921.72	15508.96	14508.92	892.65	681.2	559.04	1.447	0.0001	3.942	21.070	4.397
N0609	N-Hydroxy-L-valine	C5H11NO3	0.842	C20313	157630.41	174459.46	185545.55	5544.39	17319.78	1928.08	1.438	0.0003	3.551	20.879	4.384
P0378	Pymetrozin	C10H11N5O	1.800	C18590	41804.02	46609.57	40962.34	1358.57	2262.11	3178.11	1.499	0.0009	3.065	19.029	4.250
P2552	3-Methyl-1,2-didehydro-2,	C31H52	6.927	C20411	7690.37	6974.06	6005.34	422.71	532.16	623.93	1.488	0.0053	2.275	13.092	3.711
P1988	7-Methyl-3-methylene-1,6	C10H16	5.971	C06074	45845.64	51478.61	46152.7	2819.09	4520.31	4266.78	1.500	0.0008	3.081	12.362	3.628
P2459	ent-Fisetinidol-(4beta->8)-	C45H38O16	11.230	C10225	41263.14	72678.71	61828.71	4499.17	4127.49	6426.97	1.423	0.0277	1.557	11.676	3.546
P0867	3-Dehydrosphinganine;3-I	C18H37NO2	6.669	C02934	46545.54	42280.86	45404.36	5832.36	4950.96	2444.94	1.501	0.0000	4.623	10.147	3.343
N0047	4-Hydroxy-L-glutamic aci	C5H9NO5	1.789	C03079	29353.3	28369.7	25060.35	2597.65	2743.99	3421.49	1.440	0.0020	2.692	9.447	3.240
P0450	Phytosphingosine	C18H39NO3	4.521	C12144	7630.64	5244.11	5454.52	1006.62	748.54	219.83	1.445	0.0131	1.882	9.281	3.214
N1629	Flavanone;2,3-Dihydroflav	C15H12O2	0.921	C00766	3697.25	3681.39	4225.1	255.84	487.07	564.41	1.438	0.0004	3.361	8.876	3.150
N1085	Rimsulfuron	C14H17N5O7S2	2.537	C10952	22815.61	22332.94	20293.68	2370.27	2516.25	3057.29	1.443	0.0008	3.073	8.238	3.042
P0817	3-Ketosphingosine	C18H35NO2	6.220	C06121	18478.56	20363.43	17602.4	2533.68	2202.13	2129.93	1.498	0.0020	2.700	8.221	3.039
P0616	4-Deoxytetronic acid	C4H6O2	0.844	C01770	62877.37	67586.16	41237.89	9090.2	6661.81	5594.78	1.432	0.0237	1.625	8.043	3.008
P0789	3-Ketosphingosine	C18H35NO2	6.220	C06121	5105.55	6456.94	3916.24	731.74	619.13	596.7	1.432	0.0252	1.599	7.948	2.991
N1151	Altretamine	C9H18N6	10.023	D02841	32135.54	29457.48	29825.34	3937.73	3830.8	4306.05	1.445	0.0007	3.128	7.571	2.921
P1905	Cypridina luciferin	C22H27N7O	1.384	C02825	6107.64	6469.38	8018.57	521.54	1911.3	371.2	1.458	0.0017	2.774	7.345	2.877
N0211	.beta.-Nicotinamide adenir	C21H27N7O14P2	1.233	C00003	30506.92	18485.8	17733.27	3000.39	3037.95	3359.03	1.331	0.0437	1.359	7.100	2.828
P2344	2-Methylcholine	C6H16NO	0.844	C02224	141700.75	143479.3	148388.54	25980.39	19113.96	16709.62	1.503	0.0000	5.056	7.015	2.810
P0943	(4S,5R)-4,5,6-Trihydroxy-	C6H11NO5	0.819	C20845	104740.77	106095.39	97903.3	15921.2	13956.1	16828.73	1.503	0.0003	3.560	6.610	2.725
N0935	Quercetin 3-sulfate	C15H10O10S	1.252	C00616	17367.82	16303.37	19070.16	2828.97	2326.64	2886.72	1.439	0.0020	2.702	6.558	2.713
P2042	9alpha-Fluoro-11beta-hydı	C22H31FO3	2.190	C14630	16553.26	16183.76	13281.93	2086.91	1937.25	3096.18	1.484	0.0030	2.522	6.463	2.692
P1874	Dihydrostreptomycine	C21H41N7O12	1.259	C01023	5297.11	6799.2	5771.66	741.56	662.9	1411.16	1.476	0.0020	2.706	6.346	2.666
P1473	Violacene	C10H13BrCl4	0.736	C17107	14873.96	16961.53	15507.84	2219.51	2432.68	2830.54	1.498	0.0011	2.969	6.327	2.662
P1626	Pregnan-21-ol;5-beta-Preg	C21H36O	6.277	C00892	34470.19	35831.01	33392.29	5569.67	4523.93	6373.35	1.503	0.0000	5.004	6.297	2.655
P0022	Phosphocholine	C5H15NO4P	0.846	C00588	1853177.5	1943496.85	2052706.88	382983.7	294388.79	261287.64	1.500	0.0001	4.161	6.232	2.640
N1391	(5-L-Glutamyl)-L-glutamir	C10H17N3O6	0.858	C05283	24334.71	25922.3	27836.28	546.82	10253.79	1964.75	1.389	0.0120	1.919	6.118	2.613
P1841	Cefotetan	C17H17N7O8S4	1.310	C06886	12263.08	19264.11	13893.7	3180.15	1574.81	2842.47	1.423	0.0224	1.651	5.978	2.580
P0748	3-ketosphingosine	C18H35NO2	6.220	C06121	37873.83	28108.42	33781.66	5672.7	7518.87	3521.04	1.470	0.0046	2.338	5.969	2.578
N0378	3-Propylmalate	C7H12O5	1.789	C02123	5022.32	3952.21	3414.83	543.83	494.56	1054.72	1.390	0.0106	1.976	5.919	2.565
N1896	dTDP-3-amino-3,4,6-tride	C16H27N3O13P2	1.216	C11910	19809.56	22445.63	22332.94	5296.84	2497.68	3477.3	1.435	0.0001	3.927	5.730	2.519
P2496	Gliclazide	C15H21N3O3S	0.915	D01599	18433.37	17731.55	13726.57	2940.31	2954.37	2875.76	1.472	0.0112	1.950	5.689	2.508
P0713	1-Stearoyl-2-hydroxy-sn-g	C23H48NO7P	9.917	C21484	6907.71	6913.36	5757.22	1045.04	1598.21	973.16	1.486	0.0012	2.928	5.414	2.437
N1424	(Z)-But-1-ene-1,2,4-tricarl	C7H8O6	0.974	C04002	9934.74	9052.12	6585.41	1282.79	1443.54	2018.06	1.390	0.0165	1.783	5.390	2.430
N1739	Hepcinat	C22H29FN3O9P	0.950	D10366	55287.35	33317.39	52160.33	7357.33	14364.15	4450.5	1.350	0.0184	1.735	5.378	2.427
N0377	3-Propylmalate	C7H12O5	1.789	C02123	52458.94	53851.75	48835.48	7780.46	11041.64	11071.96	1.442	0.0000	4.336	5.190	2.376
N0554	Menatetrenone	C31H40O2	9.375	D00100	5302.34	3653.12	4801.88	1114.57	1249.18	304.98	1.385	0.0056	2.252	5.155	2.366
N0265	.beta.-Nicotinamide adenir	C21H27N7O14P2	1.237	C00003	34369.22	40399.87	39138.09	1660.02	1359.71	19752.04	1.334	0.0293	1.533	5.002	2.323
P0004	Sphingosine	C18H37NO2	6.669	C00319	205446.36	200104.89	218759.64	39684.2	41610.48	43976.34	1.502	0.0007	3.167	4.984	2.317
P1629	Ponceau MX;Acid Red 26	C18H14N2O7S2.2	0.728	C19294	62945.76	77071.88	78707.31	18623.95	17315.79	8601.02	1.476	0.0014	2.868	4.911	2.296
N0129	Myristoleic acid	C14H26O2	10.023	C08322	213870.67	189176.41	198864.18	38911.4	42779.91	42793.69	1.442	0.0015	2.814	4.835	2.274
P2057	Caldopentamine;N-(3-Am	C12H31N5	5.936	C21100	25963.36	25263.7	36464.63	5599.74	6389.16	6891.74	1.435	0.0231	1.637	4.645	2.216
N0246	Uridine monophosphate (U	C9H13N2O9P	0.900	C00105	31471.67	18130.48	19421.69	3953.21	6825.98	4359.86	1.306	0.0464	1.334	4.559	2.189
P1835	Ceftizoxime	C13H13N5O5S2	0.728	C06890	15245.54	17194.14	18098.02	3849.76	3383.06	3881.24	1.493	0.0031	2.505	4.547	2.185
P2214	Isoamylamine	C5H13N	0.835	C02640	7342.42	11126.86	10890.18	2425.25	2751.58	1350.27	1.426	0.0165	1.782	4.498	2.169
P0874	DOPAMINE	C8H11NO2	1.800	C03758	6492.06	5278.29	3714.38	1456.67	738.27	1281.53	1.390	0.0310	1.509	4.454	2.155
P2222	Indole-3-ethanol;Tryptoph	C10H11NO	0.820	C00955	96991.24	98795.02	101361.09	23315.5	21329.11	22620.37	1.504	0.0000	4.615	4.418	2.143
N0973	Pesticide4 Chlorantranilip	C18H14BrCl2N5O	1.822	C18454	47000.35	36647.11	57707.12	9958.12	9871.48	13121.86	1.370	0.0243	1.615	4.290	2.101
P0498	Lauric acid	C12H24O2	9.095	C02679	11668.89	8969.43	9107.98	3182.78	2031.2	1765.25	1.458	0.0049	2.312	4.262	2.092
N1202	8-Hydroxy-2,3,4-trichloro	C12H5Cl3O2	1.162	C15236	17005.41	20971.01	22087.41	7382.1	4101.4	2778.51	1.398	0.0019	2.727	4.211	2.074
N1335	1D-myo-Inositol 1,3,4-tris	C6H15O15P3	0.744	C01243	20611.5	24689.66	25330.89	4778.32	8609.59	3385.09	1.407	0.0011	2.943	4.211	2.074
P2569	3-Oxoglycyrrhetinate	C30H44O4	2.115	C02943	18742.63	16618.23	16376.62	5002.99	2071.1	5394.51	1.477	0.0009	3.064	4.149	2.053
P0869	3-Dehydrosphinganine;3-I	C18H37NO2	6.669	C02934	105958.94	103899.79	115503.94	31685.54	25576.68	22691.37	1.497	0.0001	4.037	4.069	2.025
P0343	Sphinganine	C18H39NO2	6.669	C00836	11797.88	18143.13	12059.02	3267.39	5337.42	1740.05	1.379	0.0206	1.685	4.060	2.021
N1899	Doxefazepam	C17H14ClFN2O3	0.843	C19413	13517.1	15125.02	16144.85	350.88	5567.77	5485.9	1.369	0.0123	1.910	3.927	1.973
P1972	Alpha-Tocotrienol	C29H44O2	8.581	C14153	13306.26	12763.65	14335.81	2991.39	2854.82	4466.73	1.491	0.0001	3.846	3.918	1.970
N1065	Silver sulfadiazine	C10H9AgN4O2S	1.789	D00433	1725.67	1908.3	1394.35	587.29	312.78	384.34	1.397	0.0048	2.319	3.915	1.969
P1851	Dicofol;Kelthane	C14H9Cl5O	0.728	C14301	42277.96	53788.14	52628.61	13441.6	13387.69	11347	1.476	0.0080	2.098	3.895	1.962
P0308	D-erythro-N-stearoylsphin	C18H37NO2	6.669	C00319	565595.04	533644	564241.08	108695.12	146360.93	174585.77	1.497	0.0003	3.576	3.872	1.953
P2043	8-(3,3-Dimethylallyl)spath	C20H22O4	1.225	C09002	9478.95	10142.27	11441.17	2764.2	2520.48	2739.18	1.488	0.0049	2.306	3.871	1.953
P2113	Bendiocarb;2,2-Dimethyl-	C11H13NO4	1.534	C14433	10414.14	10588.1	9270.71	1863.75	2885.04	3183.74	1.487	0.0002	3.686	3.816	1.932
P0771	SOLASODINE	C27H43NO2	9.913	C10822	4639.44	2403.08	3903.39	1465.98	1191.69	319.63	1.315	0.0369	1.432	3.676	1.878
N1302	Anthocyanidin with a 3-hy	C15H11O2	0.966	C21381	24620.31	18452.98	21415.65	4294.82	9251.42	4084.58	1.382	0.0032	2.501	3.658	1.871
N0245	L-Histidine;(S)-alpha-Ami	C6H9N3O2	0.742	C00135	26435.63	26799.4	26453.95	6076.89	7381.39	8501.83	1.444	0.0010	2.996	3.629	1.859
P0559	Niacin (Nicotinic acid)	C6H5NO2	1.019	C00253	82734.01	83310.34	81223.09	18922.98	18165.08	33314.87	1.484	0.0063	2.204	3.512	1.812
N1592	GDP-4-acetamido-4,6-dide	C18H28N6O15P2	1.241	C20672	5636.67	6996.21	6612.31	2137.82	455.71	2924.92	1.360	0.0106	1.973	3.487	1.802
N0062	cis-5,8,11,14-Eicosatetrae	C20H32O2	11.062	C00219	20481.29	18297.07	23400.25	5427.22	4617.54	7938.04	1.407	0.0020	2.695	3.458	1.790
N0384	Maleate	C4H4O4	0.834	C01384	594317.1	618654.33	628810.84	187782.73	164390.16	182302.64	1.446	0.0000	4.915	3.446	1.785
N1001	Phorate	C7H17O2PS3	1.516	C18690	54004.73	77919.15	67461.81	26525.59	30040.97	1559.24	1.305	0.0161	1.793	3.430	1.778
P2064	Calcipotriol	C27H40O3	9.348	D01125	19327.32	17090.44	17053.67	2600.98	8254.95	4732.37	1.447	0.0076	2.120	3.430	1.778
N0651	Miglustat	C10H21NO4	9.093	D05032	14280.71	9710.47	9091	2818.3	4893.93	2107.29	1.311	0.0247	1.607	3.369	1.752
P1329	N-tert-Butyloxycarbonyl-d	C23H44N6O5	2.115	C11604	174986.11	186063.64	177620.3	54902.15	53910.82	54216.78	1.503	0.0007	3.184	3.304	1.724
N0305	(S)-Lactate;L-Lactate;L-La	C3H6O3	1.237	C00186	111546.98	111496.99	108915.92	28393.77	28775.76	43697.2	1.435	0.0034	2.464	3.291	1.719
N1665	Homogentisate;Homogenti	C8H8O4	1.540	C00544	45837.59	45348.57	40039.84	13364.13	12846.65	13908.59	1.438	0.0030	2.519	3.271	1.710
P0813	L-Glutathione (reduced for	C10H17N3O6S	0.919	C00051	14163.22	13023.97	10458.4	5994.95	1764.76	3808.33	1.409	0.0063	2.201	3.254	1.702
P0962	beta-Citryl-L-glutamate;be	C11H15NO10	0.958	C20775	21389.48	14005.69	12951.69	8328.29	2823.51	4006.06	1.310	0.0321	1.493	3.190	1.673
P0257	Dicyclomine	C19H35NO2	6.037	C06951	4937.99	4758.98	5805.12	1260.56	1630.33	1992.36	1.470	0.0015	2.813	3.175	1.667
P2017	Aerophobin 2	C16H19Br2N5O4	0.720	C17163	23913.73	28423.87	30044.88	7287.69	9090.56	10194.61	1.471	0.0035	2.459	3.100	1.632
N1510	2-Oxoadipate;2-Oxoadipic	C6H8O5	0.850	C00322	3782.4	2461.57	4332.56	1332.16	598.42	1504.79	1.284	0.0323	1.490	3.079	1.622
P2467	DuP 697	C17H12BrFO2S2	0.719	C11704	18689.11	22618.4	22952.58	7184.8	7370.21	6614.89	1.478	0.0076	2.116	3.035	1.602
P2225	Imolamine	C14H20N4O	2.124	D07159	42849.12	40612.34	36659.98	13614.94	12549.9	13759.53	1.491	0.0033	2.477	3.009	1.589
N1894	dTDP-4-oxo-2,3,6-trideox	C16H24N2O13P2	3.181	C18032	11179.36	12373.99	11399.65	3889.48	3084.55	4647.36	1.432	0.0002	3.636	3.008	1.589
N0236	N-Acetylaspartic acid	C6H9NO5	1.229	C01042	15387.91	15775.49	14174.4	5293.27	4893.59	5212.03	1.441	0.0014	2.862	2.944	1.558
P0110	Galacto-N-biose	C14H25NO11	0.856	C07278	8510.66	8982.62	6617.79	3433.52	2339.55	2566.05	1.441	0.0086	2.066	2.891	1.532
P1198	10-Hendecenoic acid	C11H20O2	0.911	C13910	36937.05	40969.02	46734.22	12030.36	19351.05	11920.71	1.451	0.0021	2.674	2.878	1.525
P1244	Orthophosphate;Phosphate	H3PO4	0.728	C00009	73110.38	82230.28	82285.35	28754.06	31185.95	23715.43	1.490	0.0003	3.530	2.841	1.506
N0258	N-Acetylaspartic acid	C6H9NO5	0.940	C01042	19937.25	22598.15	17379.6	6577.17	5668.77	8912.89	1.396	0.0035	2.452	2.832	1.502
N1543	Kalbreclasine	C20H23NO12	1.914	C08529	12808.4	15283.92	13273.3	5677.3	4524.87	4476.2	1.422	0.0019	2.720	2.818	1.495
P0621	7,8-dihydro-L-Biopterin	C9H13N5O3	0.961	C02953	85827.56	101944.15	80787.2	37930.1	18352.43	40053.07	1.430	0.0037	2.430	2.788	1.479
P0896	Solanidine	C27H43NO	10.266	C06543	10060.4	6915.26	10212.82	2252.39	5008.21	2543.57	1.359	0.0151	1.821	2.773	1.472
P1918	Cryptopleurine	C24H27NO3	2.473	C10586	22347.41	21333.72	19085.44	8161.19	8300.13	6285.22	1.484	0.0007	3.178	2.759	1.464
N1835	Cefprozil	C18H19N3O5S	3.360	C06888	18237.8	26680.95	32252.67	8728.09	12232.63	7285.37	1.276	0.0443	1.354	2.732	1.450
N1528	3'-Phosphoadenylylselenat	C10H15N5O13P2S	1.374	C05696	17326.27	14893.24	14627.3	4237.92	6910.28	6004.66	1.410	0.0011	2.965	2.731	1.450
N1748	Guanosine 3'-diphosphate	C10H18N5O20P5	0.736	C04494	23339.67	26082	26711.04	9971.85	11226.42	7062.51	1.419	0.0007	3.172	2.694	1.430
N1079	S-(2,2-Dichloro-1-hydroxy	C12H19Cl2N3O7S	1.222	C14861	216381.62	211547.2	237085.29	46487.79	104422.72	97481.97	1.391	0.0083	2.081	2.677	1.421
P0706	1-Stearoyl-2-hydroxy-sn-g	C23H48NO7P	9.910	C21484	31771.7	30840.53	31436.06	12790.55	10483.74	11957.95	1.501	0.0003	3.566	2.669	1.417
P1215	1D-myo-Inositol 1,3,4,5,6-	C6H17O21P5	0.728	C01284	57162.21	68596.14	66334.39	23650.38	28020.69	20336.71	1.475	0.0014	2.858	2.668	1.416
N0675	N-[(7,8-Dihydropterin-6-y	C18H23N6O8P	0.940	C20562	14370.36	15977.76	15739.14	5339.95	5677.04	6408.18	1.436	0.0003	3.583	2.645	1.403
N0866	Thiobenzamide	C7H7NS	3.185	C16281	24109.8	23867.89	21148.22	9615.17	7221.99	9416.29	1.428	0.0004	3.413	2.633	1.397
P0894	L-Glutamic acid; LC-ESI-I	C5H9NO4	0.820	C00025	10234.52	12055.22	12952.65	3821.53	5464.61	4130.7	1.457	0.0030	2.526	2.627	1.393
N0435	Terpendole E	C28H39NO3	9.093	C20536	57917.16	54128.37	51483.43	19242.54	23696.26	19869.57	1.435	0.0002	3.658	2.604	1.381

P0928	N-Acetylaspartic acid	C6H9NO5	0.944	C01042	33600.67	37795.37	38411.93	11923.58	21769.5	8716.99	1.411	0.0187	1.729	2.589	1.373
P2236	L-Aspartate;L-Aspartic aci	C4H7NO4	0.936	C00049	196878.58	204761.67	134835.69	54707.55	108882.46	45314.08	1.324	0.0217	1.664	2.568	1.361
N0974	Perfluorooctylsulfonyl fluo	C8F18O2S	0.735	C18143	15049.15	17625.58	18494.47	5957.39	9579.37	4411.71	1.363	0.0072	2.145	2.565	1.359
P0738	3-ketosphingosine	C18H35NO2	6.220	C06121	86269.27	70855.69	67511.89	43080.17	26325.26	18804.83	1.397	0.0088	2.057	2.547	1.349
P1912	Cytosine arabinoside;Cytar	C9H13N3O5	1.110	C02961	87069.59	107876.04	74501.36	40079.91	25858.44	41144.45	1.396	0.0162	1.791	2.516	1.331
P1143	N-Acetyl-L-phenylalanyl-3	C20H20I2N2O5	0.727	C04418	128323.69	158810.27	159479.74	62296.11	66627.53	49180.86	1.458	0.0046	2.339	2.508	1.326
P1843	Cefonicid sodium	C18H16N6O8S3.2	0.728	C08105	38197.8	47841.61	47806.95	17703.66	20339.03	15588.44	1.457	0.0065	2.184	2.496	1.319
P2416	Ethylhexyl salicylate	C15H22O3	1.234	D05226	14746.37	15861.76	13249.84	5200.93	6084.06	6309.32	1.479	0.0026	2.591	2.493	1.318
P2104	Capsi-amide;N-(13-Methy	C17H35NO	6.045	C17515	7351.3	6358.31	6150.94	2437.63	2573.12	3074.94	1.472	0.0025	2.603	2.456	1.296
P1233	Nitrazepam	C15H11N3O3	0.921	C07487	22399.73	21178.41	17195.74	11361.47	3467.16	10178.69	1.352	0.0208	1.683	2.430	1.281
P0931	N-Acetylaspartic acid	C6H9NO5	0.940	C01042	52709.53	65198.86	55630.78	23574.2	36159.57	11701.97	1.364	0.0230	1.638	2.429	1.281
N1846	Chlorfenapyr	C15H11BrClF3N2	0.735	C18455	9431.71	12634.56	10955.47	3826.38	4534.05	5255.77	1.380	0.0099	2.005	2.425	1.278
P0835	Solanidine	C27H43NO	8.480	C06543	7158.97	5423.73	5673.51	2424.98	2775.06	2342.2	1.437	0.0176	1.754	2.421	1.275
N0995	Phthalate	C11H19N3O6	1.241	C21016	23274.1	21635.4	21669.44	9146.39	8888.31	9481.16	1.443	0.0007	3.143	2.420	1.275
P1876	Dimethylarsinous acid	C2H7AsO	13.831	C20300	50103.48	35524.12	45333.57	20741.41	22501.24	10964.81	1.381	0.0110	1.959	2.416	1.273
N0023	N-Acetylaspartate	C6H9NO5	1.224	C01042	763337.31	766092.41	728956.25	331161.56	312300.05	298728.27	1.445	0.0000	4.858	2.397	1.261
N0856	Ticagrelor	C23H28F2N6O4S	0.958	D09017	82254.71	71570.95	53714	36190.53	26459.81	24106.13	1.322	0.0255	1.593	2.392	1.258
P1827	Cetylpyridinium chloride	C21H38N.Cl.H2O	2.365	C11307	6353.46	3866.72	5966.42	1589.13	2338.92	2882.62	1.318	0.0380	1.421	2.377	1.249
P0831	L-Glutathione (reduced fo	C10H17N3O6S	1.251	C00051	6196.12	5857.81	4392.05	2969.46	2602.39	1351.9	1.367	0.0131	1.882	2.375	1.248
N0209	Adenosine	C10H13N5O4	1.739	C00212	13363.18	12173.5	12047.35	5893.19	7055.5	2987.6	1.366	0.0182	1.740	2.358	1.238
P0932	N-Acetylaspartic acid	C6H9NO5	0.936	C01042	153277.65	138982.21	129695.65	63402.45	53955.25	61696.01	1.481	0.0027	2.576	2.357	1.237
P2503	IAARh123;[125I]Iodoaryl	C28H19IN5O5	0.720	C11599	32614.48	40936.46	45829.3	17455.16	19409.05	13837	1.414	0.0158	1.800	2.355	1.235
P1737	Salicifoline	C12H20NO2	1.119	C17063	16243.92	11990.8	13596.9	4850.63	9207.47	3714.23	1.337	0.0213	1.672	2.354	1.235
P1363	Tiludronate	C7H9ClO6P2S	0.728	C08141	18631.2	23881.78	22586.3	8142.89	11372.8	8251.29	1.440	0.0045	2.352	2.344	1.229
P1492	Tridemorph	C19H39NO	7.050	C11285	14088.87	15871.19	18504.57	5488.67	6611.48	8602.41	1.424	0.0057	2.242	2.341	1.227
P0763	1-Stearoyl-2-hydroxy-sn-g	C23H48NO7P	9.377	C21484	22240.24	19983.84	21101	10215.42	10708.43	6314.24	1.460	0.0053	2.279	2.325	1.217
P0882	Hercynine;Nalpha,Nalpha,	C9H16N3O2	2.472	C05575	6474.44	5406.84	4784.37	2595.59	2064.37	2512.59	1.430	0.0158	1.803	2.324	1.216
P2210	LNT	C26H45NO21	0.828	C06371	30391.99	36323.86	28263.98	14222.13	13555.38	13234.49	1.454	0.0164	1.785	2.316	1.212
P2454	Ergothioneine	C9H16N3O2S	1.717	C05570	38356.28	41780.92	37091.04	16564.83	14264.99	19850.09	1.477	0.0005	3.263	2.313	1.210
P2402	GDP-4-acetamido-4,6-dide	C18H28N6O15P2	1.301	C20672	4063.21	5295.78	3414.12	2582.95	1847.78	1132.61	1.306	0.0286	1.543	2.296	1.199
P1680	Sulfoacetate;Sulfoacetic ac	C2H4O5S	0.727	C14179	67325.71	77628.22	80462.79	34881	34165.08	29208.59	1.475	0.0032	2.490	2.294	1.198
N0214	(S)-Malate	C4H6O5	0.934	C00149	1266758.87	1204758.08	1017563.27	493996.16	523321.76	510065.34	1.412	0.0121	1.919	2.284	1.192
N0599	Methazole	C9H6Cl2N2O3	0.734	C19123	33488.34	37389.75	38717.37	16345.83	17116.94	14707.46	1.427	0.0018	2.744	2.275	1.186
N1410	(S)-3-Sulfonatolactate	C3H6O6S	0.734	C11499	23455.36	28752.1	28384.78	12046.57	12736.54	10663.43	1.407	0.0070	2.156	2.274	1.185
N0637	NAC-Diketide	C15H17NO4S	0.934	C12089	317332.74	290950.58	252022.79	123415.15	129626.42	126848.22	1.410	0.0131	1.881	2.265	1.179
N1744	Halacrinate	C12H7BrClNO2	4.390	C18941	6339.46	7622.47	6360.45	1161.47	3417.7	4464.4	1.258	0.0446	1.351	2.247	1.168
P2109	Benzeneacetic acid, 3-[3-[	C33H31ClF3NO3	1.285	C15631	10760.69	15290.02	12516.77	5472.57	7862.51	3900.73	1.352	0.0159	1.799	2.238	1.162
P0533	Pantothenic Acid	C9H17NO5	3.354	C00864	5067.92	6223.34	5211.07	1819.24	1818.56	3741.87	1.352	0.0232	1.634	2.236	1.161
P1216	2-(Hydroxymethyl)-3-(ace	C8H11NO6	0.957	C04690	14478.18	13179.95	12954.08	7112.41	5840.07	5217.61	1.478	0.0006	3.231	2.235	1.160
N1198	6'-Dehydro-6'-oxoparomar	C12H23N3O7	1.631	C17583	119903.32	121427.36	113963.9	53424.11	52328.87	53641.93	1.445	0.0009	3.050	2.229	1.156
N0082	ostruthin	C19H22O3	11.893	C09281	127262.3	135581.7	120451.85	43455.99	83748.87	45350.72	1.347	0.0239	1.621	2.221	1.151
N0387	2-(Hydroxymethyl)-3-(ace	C8H11NO6	1.258	C04690	8570.87	11998.78	9752.99	6494.31	3446.93	3817.36	1.294	0.0166	1.780	2.204	1.140
P1692	Quercetin-3-(6''-malonyl)-	C24H22O15	3.204	C12638	11502.7	16405.84	16293.04	8463.73	6245.66	5376.32	1.367	0.0203	1.692	2.201	1.138
P1245	Octane	C8H18	6.253	C01387	5725.73	5421.95	5357.51	1821.13	2111.02	3570.14	1.410	0.0268	1.572	2.200	1.138
P1193	Phenkapton	C11H15Cl2O2PS3	0.720	C19010	212523.48	246088.55	246020.14	109047.02	115739.36	96183.93	1.478	0.0021	2.686	2.195	1.134
P0919	γ-L-glutamic acid-L-cystei	C8H14N2O5S	0.919	C00669	33952.77	32377.83	26189.84	13707.52	15380.88	13218.5	1.445	0.0143	1.846	2.187	1.129
N1952	Delphinidin 3-O-(6-caffeo	C30H27O15	0.978	C16367	13200.91	14827.17	17256.84	6314.38	7270.34	7183.16	1.386	0.0154	1.813	2.181	1.125
P2332	Luteolin;3',4',5,7-Tetrahyd	C15H10O6	0.727	C01514	48051.5	57281.93	54994.92	25303.73	27664.15	20705.26	1.465	0.0016	2.798	2.176	1.122
N0985	Ponceau MX;Acid Red 26	C18H14N2O7S2.2	0.734	C19294	130338.9	149411.8	155474.63	70613.14	72469.16	58151.28	1.412	0.0022	2.659	2.163	1.113
P1165	10-Formyl-7,8-dihydrofoli	C20H21N7O7	0.819	C03204	20068.58	19064.42	17402.62	9598.53	6590.73	9987.12	1.456	0.0023	2.643	2.160	1.111
P1886	Didecyldimethylammoniun	C22H48N.Cl	6.802	C15436	14494.89	11966.58	13156.43	6551.58	6086.67	5796.87	1.472	0.0066	2.181	2.149	1.104
N0380	Glutathione	C10H17N3O6S	1.224	C00051	182301.14	172042.05	172571.66	106272.21	92486.35	47190.58	1.352	0.0308	1.511	2.142	1.099
P1368	(5-Phenyl-1,2,4-triazol-3-y	C9H9N5O	2.298	C15383	269651.75	259965.24	237533.63	123676.56	118351.33	120557.17	1.490	0.0041	2.386	2.116	1.081
N0654	Metofluthrin	C18H20F4O3	3.127	C13487	4286.77	3074.6	4383.49	1947.95	2154.42	1464.49	1.319	0.0235	1.630	2.110	1.077
N1418	1-O,2-O,6-O-Trigalloyl-be	C27H24O18	0.925	C04360	41944.11	47083.42	53485	23189.59	19987.94	24596.33	1.390	0.0092	2.038	2.103	1.072
N1120	9-Hydroxybenzo[a]pyrene-	C20H12O2	0.966	C14854	1301739.26	1225469.81	1055503.03	681943.08	525160.51	507597.85	1.391	0.0031	2.508	2.089	1.063
N0865	Thiobinupharidine	C30H42N2O2S	7.979	C09990	3573.92	3736.62	3830.75	1231.81	2428.03	1677.15	1.357	0.0266	1.575	2.088	1.062
N0940	Pyrophosphoric acid	H4O7P2	1.216	C00013	12423.79	8835.06	10776.62	7140.26	3739.59	4498.81	1.284	0.0191	1.718	2.083	1.059
P0846	Solanidine	C27H43NO	8.480	C06543	27017.96	26370.66	25513.7	11406.41	12544.09	14006.72	1.493	0.0004	3.413	2.079	1.056
N0557	Meclofenamate sodium;Me	C14H10Cl2NO2.N	0.734	C02996	16238.39	19053.85	17728.74	8812.11	8926.88	7841.61	1.421	0.0030	2.520	2.073	1.052
P1810	5-Oxoproline	C5H7NO3	0.944	C02237	76834.27	78912.78	74345.66	40514.22	46117.5	24734.92	1.431	0.0215	1.668	2.066	1.047
P1371	(E)-4,8-Dimethyl-1,3,7-no	C11H18	6.752	C21795	75123.14	111636.97	100876.89	60125.98	39723.77	41215.16	1.337	0.0260	1.585	2.039	1.028
N0806	Thiadiazin;Milneb	C12H22N4S4	0.834	C18628	19162.62	18481.26	23173.9	9434.37	11549.97	8937.33	1.377	0.0078	2.109	2.033	1.023
N0389	Glutathione	C10H17N3O6S	1.224	C00051	43537.11	49007.99	43515.56	25723.55	22794.05	18876.91	1.410	0.0011	2.963	2.019	1.014
P0580	Octanoylcarnitine	C15H29NO4	4.558	C02838	11907.79	10160.12	11306.01	23826.89	20779.95	22313.04	1.481	0.0012	2.932	0.499	-1.004
N0413	Taurine	C2H7NO3S	0.834	C00245	12163.56	9806.58	14137.8	22796.18	24644.82	25589.54	1.408	0.0022	2.658	0.494	-1.016
N1743	Halofenozide	C18H19ClN2O2	6.085	C18791	22253.37	19032.11	18445.95	40124.18	39429.98	41307.68	1.436	0.0008	3.098	0.494	-1.017
P1254	O-Ureido-L-serine;(2S)-2-	C4H9N3O4	1.812	C20639	6450.99	9543.67	5840.87	14472.58	14557.82	15259.79	1.437	0.0188	1.725	0.493	-1.020
N1840	Chloroform	CHCl3	1.141	C13827	8694.25	7479.64	6794.98	12325.73	16223.88	18228.77	1.317	0.0346	1.461	0.491	-1.026
N0639	N7-Methylguanosine 5'-di	C11H18N5O11P2	4.797	C20183	5851.66	4857.66	5040.27	10700.52	10375.54	11028.45	1.435	0.0003	3.464	0.491	-1.027
N0484	Oxidized Photinus luciferi	C10H6N2O2S2	1.120	C03797	24098.57	17219.57	9873.65	34822.53	35875.6	34180.34	1.312	0.0470	1.328	0.488	-1.035
P1696	1-Linoleoylglycerophosphc	C26H51NO7P	8.438	C04100	1666.06	1637.04	973.41	3596.06	2706.75	2495.19	1.323	0.0261	1.583	0.486	-1.041
P1750	5-Hydroxy-L-Tryptophan	C11H12N2O3	2.157	C00643	46119.52	45404.71	40480.48	92787.7	99393.9	79584.73	1.455	0.0101	1.995	0.486	-1.042
P1028	IAA-94;Indanyloxyacetic a	C17H18Cl2O4	4.828	C13703	4071.46	5621.17	6543.92	10979.55	12845.27	9621.83	1.391	0.0097	2.014	0.485	-1.043
N0088	Psilocybin	C12H17N2O4P	2.155	C07576	52932.17	53700.28	47169.07	102956.12	116441.15	97646.12	1.414	0.0053	2.276	0.485	-1.044
P1531	Tropinone	C8H13NO	1.796	C00783	1457.93	721.73	647.63	2219.7	2152.84	1462.25	1.227	0.0476	1.322	0.485	-1.045
N1912	Diacetyl;Biacetyl;Dimethy	C4H6O2	1.224	C00741	16437.72	15611.95	15357.51	35151.17	32445.05	30435.67	1.428	0.0045	2.342	0.484	-1.048
N0193	1-METHYLXANTHINE	C6H6N4O2	9.716	C16358	15.97	7.32	18.95	22.7	35.52	29.7	1.205	0.0404	1.393	0.480	-1.058
P0278	D-Glucose	C6H12O6	0.904	C00031	33024.13	41801.72	46038.17	81533.83	82464.01	88180.55	1.477	0.0019	2.728	0.479	-1.061
N0587	Lipoic acid, reduced	C8H16O2S2	1.375	C02147	6433.43	6716.9	3703.42	10869.02	11501.64	12891.29	1.358	0.0092	2.035	0.478	-1.065
P0861	Erythrulose 1-phosphate;L	C4H9O7P	0.711	C03394	442151.3	441580.27	446787.89	879041.97	895231.29	1013094.65	1.484	0.0074	2.130	0.477	-1.067
P0860	Erythrulose 1-phosphate;L	C4H9O7P	0.711	C03394	29926.24	29022.81	29012.65	58230.15	59476.28	67360.86	1.483	0.0072	2.143	0.475	-1.073
P2401	GDP-D-mannuronate;GDI	C16H23N5O17P2	0.720	C00976	15829.19	17840.12	18948.91	35321.69	37010.31	38707.55	1.492	0.0001	3.875	0.474	-1.077
P1974	alpha-TOCHOPHEROL	C29H50O2	8.563	C02477	889.66	526.38	636.49	1433.52	1602.87	1296.22	1.412	0.0061	2.215	0.474	-1.078
N1812	Cefmetazole	C15H17N7O5S3	0.726	C08103	48644.05	47230.24	49383.46	99672.83	97878.57	109689	1.435	0.0037	2.428	0.473	-1.081
P1192	1,3-Benzenedisulfonic acic	C14H14N3O12PS2	0.720	C13745	7810.58	9404.34	7140.08	15673.03	19006.06	16948.85	1.457	0.0024	2.614	0.472	-1.084
P2370	Fenuron	C9H12N2O	0.902	C11224	22963.29	21380.39	24589.23	46042.3	55285.18	46050.7	1.461	0.0089	2.051	0.468	-1.096
P2078	Brugine	C12H19NO2S2	0.910	C10849	1500.29	2927.45	2315.71	4105.42	5910.3	4415.5	1.323	0.0241	1.617	0.467	-1.098
N1048	sweroside	C16H22O9	1.864	C17071	43414.57	39611.94	37536.03	90992.34	79663.55	89062.84	1.427	0.0015	2.834	0.464	-1.107
N1195	5'-monophosphate-2'-deox	C10H14N5O6P	4.797	C00360	31661.08	28905.07	21467.26	55832.1	56054.04	65162.2	1.397	0.0019	2.732	0.463	-1.110
P2168	2,3-Dioxo-6-nitro-7-sulfar	C12H8N4O6S	1.368	C13667	9202.19	6086.81	8003.41	18864.74	14857.99	16897.42	1.433	0.0041	2.384	0.460	-1.120
N0555	Meloxicam	C14H13N3O4S2	0.925	C08169	56972.13	49068.91	19100.06	98881.66	77433.45	95651.72	1.269	0.0313	1.505	0.460	-1.120
N1194	5'-Phosphoribosyl-N-form	C8H15N2O9P	1.166	C04376	14535.08	13278.88	10900.74	21746.18	30438.36	33126.73	1.315	0.0360	1.444	0.454	-1.140
P1763	Chlorpropamide	C10H13ClN2O3S	1.372	D00271	9406.92	6668.79	7249.4	15338.05	19344.58	16774.03	1.439	0.0040	2.393	0.453	-1.141
N0525	Maitotoxin	C164H256O68S2.2	0.842	C16854	9678.23	10537.74	4971.75	16558.91	19248.29	20248.06	1.345	0.0113	1.947	0.449	-1.154
N0412	FMN	C17H21N4O9P	3.185	C00061	8569.52	8532.77	5985.34	17552.47	15319.97	18600.05	1.396	0.0020	2.709	0.449	-1.157
P1804	Casimiroin	C12H11NO4	1.409	C10654	13047.65	13772.65	12265.66	26482.4	26178.67	34674.01	1.423	0.0263	1.580	0.448	-1.160
N1777	Chlorsulfuron	C12H12ClN5O4S	0.834	C05071	7810.08	4523.28	4195.32	10681.27	12608.8	13644.86	1.332	0.0110	1.957	0.448	-1.160
N1161	aldicarb	C7H14N2O2S	1.424	C11015	39467.53	32294.18	30083.36	75357.22	78743.62	73510.2	1.431	0.0009	3.066	0.447	-1.160
P0653	2(5H)-Furanone	C4H4O2	0.902	C17601	56294.17	63258.95	61833.14	137581.3	148782.76	120441.05	1.467	0.0085	2.073	0.446	-1.165
P0648	2-QUINOLINECARBOX	C10H7NO2	1.409	C06325	233782.02	231641.43	231288.93	535629.55	520971.86	524162.62	1.505	0.0002	3.815	0.441	-1.182
N1494	2,3,5-Trichloro-cis,cis-mu	C6H3Cl3O4	1.374	C12833	7742.11	10968.13	7702.62	22148.72	17146.6	20694.56	1.375	0.0049	2.310	0.440	-1.183
N1350	1,2,3,4-Tetrachlorobenzen	C6H2Cl4	0.859	C18236	9129.34	7458.41	5562.12	14369.51	17923.79	18102.66	1.372	0.0045	2.350	0.440	-1.186
P2307	Mirasan	C13H21ClN2	0.902	C11716	161951.58	171866.1	170407.02	385121.25	408964.6	353372.22	1.487	0.0044	2.358	0.439	-1.186
N1125	Adenylyl-molybdopterin	C20H26N10O12P2	0.896	C19848	6088.15	7702.62	6419.06	17864.31	13005.2	15168.72	1.368	0.0170	1.770	0.439	-1.188

P0804	L-Methionine;Methionine;	C5H11NO2S	1.409	C00073	32128.16	33994.67	36191.58	81171.39	75555.02	76902.67	1.499	0.0001	4.155	0.438	-1.191
P0684	1-(3-Pyridyl)-1-butanone-4	C9H9NO3	1.409	C19569	94546.74	93507.33	89057.8	216067.97	203526.72	213992.64	1.502	0.0002	3.712	0.437	-1.193
P1069	Adenosine 5-O-(3-thiodipl	C10H15N5O9P2S	1.376	C13743	18631.98	14855.64	15211.6	36170.17	40199.13	35307.54	1.480	0.0005	3.284	0.436	-1.197
P2420	4-Carboxy-4'-sulfoazobenz	C13H10N2O5S	0.711	C06673	121128.84	113999.59	120276.26	257331.77	258796.76	299754.79	1.482	0.0069	2.159	0.436	-1.199
N0479	p-Bromophenylacetic acid	C8H7BrO2	0.934	C03076	992.97	1511.55	512.97	2287.95	2074.38	2598.27	1.297	0.0269	1.571	0.434	-1.206
N1461	3-Hydroxybutyrate	C4H8O3	1.631	C01089	9022.01	9296.86	11820.57	23570.21	25605.32	20399.16	1.399	0.0038	2.421	0.433	-1.207
P0242	Amastatin	C21H38N4O8	3.038	C01552	32645.34	32057.83	28362.65	60300.62	85788.81	69284.24	1.411	0.0287	1.542	0.432	-1.211
N1090	Scymnol;5beta-Scymnol;5	C27H48O6	7.996	C16260	3707.43	2710.15	3528.16	7538.39	7094.55	8525.37	1.409	0.0016	2.794	0.429	-1.219
N0112	S-Lactoylglutathione	C13H21N3O8S	1.856	C03451	222742.52	242523.33	224748.21	546610.3	506412.71	558398.73	1.439	0.0008	3.102	0.428	-1.224
N0027	Nicotinic Acid Adenine Di	C21H27N7O14P2	1.224	C00003	156248.8	147984.91	142140.69	320727.59	350479.24	374956.35	1.430	0.0040	2.402	0.427	-1.229
P0744	2-Aminoethanethiol	C2H7NS	1.467	C01678	4537.73	5437.22	5902.87	13859.66	12768.2	10601.2	1.444	0.0090	2.044	0.426	-1.229
P0292	Cinobufagin	C26H34O6	3.428	C16931	1306.91	1646.6	1794.06	4428.51	2695.24	4055.93	1.342	0.0470	1.328	0.425	-1.236
N1433	.beta.-Nicotinamide adenin	C21H28N7O17P3	0.925	C00006	7624.77	7442.82	2015.56	14130.91	12635.82	13581.11	1.300	0.0455	1.342	0.423	-1.240
N0760	Tetramethylrosamine	C24H24ClN2O	2.562	C11622	10880.7	13830.58	7934.4	24861.91	26126.88	26330.72	1.409	0.0090	2.046	0.422	-1.244
N0913	Progabide	C17H16ClFN2O2	5.013	D05621	1803.4	1958.95	2784.13	4736.23	4752.43	6099.03	1.364	0.0076	2.119	0.420	-1.252
P2065	Calcidiol;25-Hydroxyvitan	C27H44O2	7.267	C01561	8023.47	9983.2	8240.35	18473.76	22021.31	22143.13	1.457	0.003	2.489	0.419	-1.255
P0948	(R)-S-Lactoylglutathione;S	C13H21N3O8S	1.875	C03451	8099.02	7169.21	6379.44	17942.91	16116.46	17734.67	1.489	0.0002	3.662	0.418	-1.259
P0338	Serotonin	C10H12N2O	1.413	C00780	22115.16	21116.84	19352.92	52415.32	47721.51	50396.3	1.497	0.0002	3.686	0.416	-1.266
N0633	Nafamostat	C19H17N5O2	0.825	D08240	32145.21	31355.08	34227.51	70845.35	86278.25	77965.46	1.421	0.0076	2.119	0.416	-1.266
N1278	Anhydrorhodovibrin	C41H58O	1.233	C15877	7139.7	10817.4	11940.71	25040.68	25619.82	21273.24	1.394	0.0022	2.663	0.416	-1.267
P2239	L-alpha-Acetyl-N,N-dinor	C21H27NO2	2.572	C16662	8646.46	15046.66	13048.5	32770.26	27261.46	29170.53	1.448	0.0024	2.628	0.412	-1.280
N0337	4-Amino-4-deoxychorisma	C10H11NO5	2.151	C11355	7957.29	7741.07	5715.94	17191.37	18587.51	16366.69	1.423	0.0005	3.331	0.411	-1.284
P2533	3,5-Dichloro-L-tyrosine	C9H9Cl2NO3	1.467	C03347	27814.04	19155.21	27050.35	49212.71	75407.2	55790.33	1.362	0.0343	1.464	0.410	-1.285
P2215	Isazofos	C9H17ClN3O3PS	0.715	C19001	12094.97	10952.52	8514.8	24677.39	24143.43	29009.75	1.464	0.0020	2.708	0.406	-1.302
P2048	9-Methylthiononanaldoxim	C10H21NOS	1.800	C17253	2187.52	2653.83	1617.88	6268.34	5877.93	3800.61	1.333	0.0399	1.399	0.405	-1.304
P1976	Antimony trioxide	Sb2O3	2.397	C19192	2063.99	3134.65	4958.74	6922.08	9468.89	8724.01	1.371	0.0120	1.919	0.404	-1.306
P0547	Phenaceturic acid	C10H11NO3	1.418	C05598	76211.31	73775.47	68115.86	181792.26	179215.77	180776.34	1.504	0.0002	3.779	0.403	-1.313
N1833	Cefoxitin sodium	C16H16N3O7S2.N	0.938	C08106	46214.23	47001.08	13008.73	74673.13	86409.32	103667.91	1.279	0.0224	1.650	0.401	-1.318
P2209	L-Capreomycidine;(2S,3R	C6H12N4O2	1.417	C18472	11456.04	11677.58	7547.86	26806.1	24070.86	25603.5	1.475	0.0016	2.792	0.401	-1.318
N1129	Adenophostin B	C18H28N5O19P3	0.726	C13722	16566.61	24624.71	25065.77	52358.74	54875.69	58685.21	1.421	0.0011	2.961	0.399	-1.324
N1816	Cefacetrile sodium;Cephac	C13H12N3O6S.Na	0.892	C12691	13458.68	14552.65	11645.52	37349.61	30484.44	31759.62	1.411	0.0051	2.294	0.398	-1.328
P2560	Glycerol 3-phosphate	C3H9O6P	0.861	C00093	482.55	424.29	973.23	1556.06	1658.46	1518.46	1.409	0.0265	1.577	0.397	-1.332
N0280	Salicylic acid	C7H6O3	3.596	C00805	4083.59	3010.41	2628.59	6737.69	8566.94	9185.02	1.368	0.0083	2.081	0.397	-1.333
P0957	N6-(delta2-Isopentenyl)-a	C15H22N5O7P	1.866	C04713	4927.32	5551.78	7525.28	15501.37	11365.36	18876.93	1.349	0.0386	1.413	0.394	-1.345
N0915	Reactive blue 2	C29H20ClN7O11S	0.888	C13746	7457.12	9668.19	6634.98	22516.44	18031.16	20479.39	1.403	0.0022	2.652	0.389	-1.361
P0321	Quinoline	C9H7N	1.409	C06413	62233.54	57865.36	58825.23	158721.49	149721.77	154267.06	1.503	0.0001	4.163	0.387	-1.371
P1458	UDP-glucose;UDPglucose	C15H24N2O17P2	1.216	C00029	4136.37	5649.85	6157.95	15853.95	15351.32	10093.89	1.366	0.0340	1.469	0.386	-1.373
P0482	N-Acetyl-DL-leucine	C8H15NO3	1.409	D07350	31728.94	31361.31	28717.15	82557.28	76454.58	78943.17	1.500	0.0001	3.872	0.386	-1.374
N0576	L-Aspartate 4-semialdehy	C4H7NO3	0.826	C00441	5361.38	5334.4	2481.03	10972.37	12509.82	10714.21	1.380	0.0064	2.195	0.385	-1.376
N1465	4-Hydroxycyclophospham	C7H15Cl2N2O3P	0.726	C07643	176296.55	166950.45	181456.45	440665.06	447645.78	487815.16	1.440	0.0014	2.859	0.381	-1.391
P2075	Butoconazole	C19H17Cl3N2S	0.720	C08065	114851.02	111970.84	119474.86	282895.33	290932.38	337534.06	1.482	0.0073	2.136	0.380	-1.396
P1259	Norfloxacin	C16H18FN3O3	0.720	C06687	13637.21	12626.13	14779.28	35222.02	34302.79	39537.17	1.489	0.0020	2.705	0.376	-1.410
P1474	Vinyl chloride;Chloroethyl	C2H3Cl	1.364	C06793	5143.92	3908.83	3525.74	14117.55	9649.23	9924.94	1.380	0.0294	1.531	0.373	-1.421
P0739	3-Indoleacetic acid	C10H9NO2	1.409	C00954	35508.05	32631.98	30716.34	95302.73	86220.05	87679.4	1.496	0.0004	3.365	0.367	-1.445
N0816	Tyr	C9H11NO3	0.925	C00082	4139.98	2531.07	8350.94	14136.34	15518.38	11725.14	1.312	0.0183	1.738	0.363	-1.462
P0374	Propionylcarnitine	C10H19NO4	1.276	C03017	88726.34	93821.36	86689.28	245683.11	243509.66	253427.89	1.504	0.0000	5.273	0.363	-1.464
P1761	7-Dehydrocholesterol	C34H48O2	10.931	C01164	5614.52	6703.49	6444.55	17018.89	19271.3	15616.07	1.529	0.0051	2.293	0.361	-1.468
P1848	Dibasic sodium phosphate	Na2HPO4	0.773	C13558	7984.77	5070.65	3329.36	13937.64	13665.69	17851.58	1.401	0.0072	2.143	0.360	-1.472
N1315	Atrazine-desethyl-desisopr	C3H4ClN5	0.834	C06560	20061.21	19371.07	16728.2	50952.02	54225.81	51350.45	1.443	0.0000	4.682	0.359	-1.479
P1734	Saphenamycin	C23H18N2O5	2.115	C12311	14572.02	10364.28	5939.16	29590.79	27779.5	28847.6	1.451	0.0146	1.835	0.358	-1.482
N0759	Tetraphenylphosphonium	C24H20P	1.864	C11152	4479.38	3214.42	2084.93	8456.42	10392.2	8546.4	1.379	0.0034	2.470	0.357	-1.486
N0247	Glycodeoxycholic acid	C26H43NO5	9.284	C05464	16184.2	16315.44	13196.22	44754.96	41352.28	42450.67	1.440	0.0000	4.373	0.355	-1.492
P0918	(R)-S-Lactoylglutathione;S	C13H21N3O8S	1.862	C03451	3760.76	6282.93	5022.29	16251.23	12337.12	13840.73	1.443	0.0044	2.353	0.355	-1.494
N1621	Faropenem sodium;SUN 5	C12H14NO5S.Na	0.817	C13310	11937.67	16957.47	7685.25	32650.08	33583.09	37878.18	1.396	0.0041	2.389	0.351	-1.509
P0783	Creatine; LC-ESI-QTOF;	C4H9N3O2	0.844	C00300	9690.91	9498.66	1756.95	18901.88	18506.91	22463.26	1.375	0.0227	1.644	0.350	-1.515
N1838	Chlorothalonil;Tetrachloro	C8Cl4N2	0.934	C11037	4230.16	3512.79	6378.3	14753.07	13773.91	11913.3	1.397	0.0019	2.731	0.349	-1.518
P1229	NAD+	[C21H28N7O14P2	0.720	C00003	5429.22	4956.42	6472.77	15555.14	15740.96	17693.49	1.489	0.0005	3.337	0.344	-1.539
N1501	2,3-Dibromo-1-propanol;2	C3H6Br2O	0.792	C19224	5157.72	4607.17	2944.29	9838.3	11097.03	16048.44	1.298	0.0388	1.411	0.344	-1.541
P1459	UDP-2-acetamido-3-amin	C17H26N4O17P2	0.720	C20359	8026.98	5777.75	9272.31	21654.96	22167.3	23501.35	1.488	0.0009	3.027	0.343	-1.545
N1801	Cleviprex (Clevidipine)	C21H23Cl2NO6	1.847	D08892	5487.92	4152.1	2734.27	12994.68	10631.52	12683.76	1.396	0.0019	2.728	0.341	-1.553
N0264	.beta.-Nicotinamide adenin	C21H27N7O14P2	1.233	C00003	6444.68	7187.52	7026.93	18947.53	19063.65	22627.56	1.425	0.0066	2.180	0.341	-1.553
P1484	(-)-Hygroline	C8H17NO	2.730	C10152	2576.35	2568.12	2808.73	7552.35	6931.24	9119.56	1.461	0.0142	1.848	0.337	-1.569
P2475	Hexetidine	C21H45N3	2.605	D07068	806.68	1109.3	1101.37	2901.15	2716.61	3380.25	1.471	0.0031	2.512	0.335	-1.576
N1694	Iopodic acid	C12H13I3N2O2	0.830	C18093	3692.44	2631.58	1278.63	6856.29	7476.86	8404.25	1.375	0.0061	2.214	0.334	-1.580
N0636	NAc-DNP-Cys;N-Acetyl 2	C11H11N3O7S	2.130	C11605	4910.21	4740.31	149.15	12052.02	8316.46	9247.39	1.249	0.0307	1.513	0.331	-1.596
N0628	NCGC00384822-01![(2R,	C27H24O18	0.817	C04360	5293.46	5314.9	1688.41	12378.25	12604.79	12337.17	1.390	0.0198	1.702	0.329	-1.602
N0259	Glycodeoxycholic acid	C26H43NO5	9.284	C05464	4446.89	4915.63	4898.1	15487.11	13622.57	14213.37	1.438	0.0018	2.748	0.329	-1.603
P0651	2(5H)-Furanone	C4H4O2	2.730	C17601	22675.95	20030.85	13074.53	59045.45	55811.76	55375.94	1.486	0.0021	2.680	0.328	-1.610
P0169	Hecogenin	C27H42O4	9.286	C08902	128867.28	127914.85	121440.22	385253.85	386434.26	383335.8	1.505	0.0000	5.022	0.327	-1.611
P1156	13-(2-Methylcrotonoyl)oxy	C20H30N2O3	1.401	C04170	2768.94	5384.3	5128.72	13979.43	12459.28	14735.49	1.468	0.0012	2.931	0.323	-1.632
N1903	Docetaxel	C43H53NO14	1.681	C11231	13283.68	220.49	15636.69	27841.81	24821.02	37847.94	1.238	0.0317	1.499	0.322	-1.635
N0402	5,10-Methylene-THF	C20H23N7O6	2.379	C00143	245.8	1318.9	306.98	1810.23	1726.65	2277	1.249	0.0447	1.350	0.322	-1.635
P1917	Cuauhtemone	C15H24O3	1.501	C09645	7080.5	301.07	7301.26	16083.15	14283.53	15407.87	1.371	0.0400	1.398	0.321	-1.640
N0565	Lansoprazole	C16H14F3N3O2S	6.085	D00355	3284.77	3035.43	3378.84	8559.41	10663.9	11402.71	1.407	0.0136	1.867	0.317	-1.659
P0967	2-Dehydro-3-deoxy-L-aral	C5H8O5	0.711	C00684	6258.63	1792.23	5062.56	10957.72	14243.46	16314.08	1.383	0.0105	1.978	0.316	-1.663
N0862	Thiouracil	C4H4N2OS	1.021	C19304	10524.57	16849.96	5403.31	21717.07	41040.84	41015.02	1.237	0.0470	1.327	0.316	-1.663
N1220	6-Carboxyhex-2-enoyl-Co	C28H44N7O19P3	0.892	C06723	3158.18	3526.25	2909.01	11725.6	8842.49	10187.89	1.407	0.0109	1.962	0.312	-1.681
P0859	Erythrulose 1-phosphate;L	C4H9O7P	0.711	C03394	3783.96	5057.85	5062.63	13571.2	14227.77	16866.97	1.474	0.0039	2.404	0.311	-1.684
N1032	Slotoxin	C177H281N47O5(	0.925	C20066	1502.71	2660.42	6973.8	13382.89	9749.8	12658.96	1.303	0.0195	1.710	0.311	-1.684
N1257	Calcium sulfate;Gypsum	CaSO4	10.904	C13194	660601.25	681499.69	651925.35	2457990.95	1656980.36	2304642.78	1.373	0.0265	1.577	0.311	-1.687
N1615	Erbon	C11H9Cl5O3	2.138	C19072	298.53	14970.31	14452.75	33114.87	31547.88	31205.5	1.328	0.0426	1.371	0.310	-1.690
N0137	ADENINE	C5H5N5	1.739	C00147	107580.15	63991.51	58247.29	233914.52	278086.69	247542.77	1.411	0.0011	2.957	0.303	-1.725
P1300	Pantetheine 4'-phosphate;4	C11H23N2O7PS	1.258	C01134	2004.48	2003.94	2369.51	6639.92	6612.23	7844.61	1.485	0.0040	2.403	0.302	-1.726
P1510	Topotecan	C23H23N3O5	0.828	C11158	2925.36	5097.56	4779.21	13210.97	13974.74	15846.58	1.476	0.0007	3.161	0.298	-1.749
N0671	N-Acetyl-D-glucosamine 6	C8H16NO9P	0.859	C00357	11374.95	10847.5	9268.51	34616.46	35555.77	36508.57	1.445	0.0000	5.048	0.295	-1.760
P2112	Bensultap	C17H21NO4S4	0.721	C18563	1162.5	624.3	1872.05	3802.48	4942.18	3735.35	1.415	0.0054	2.271	0.293	-1.770
P0782	Creatine; LC-ESI-QTOF;	C4H9N3O2	0.836	C00300	7316.3	6174.33	2022.51	19003.57	15297.57	18942.83	1.434	0.0043	2.371	0.291	-1.779
N1752	Glycodeoxycholic acid	C26H43NO5	7.065	C05464	4515.8	5217.4	4475.29	15779.89	15844.2	17356.3	1.442	0.0004	3.436	0.290	-1.785
N1253	C.I. Direct blue 15	C34H28N6O16S4.	0.726	C19213	3767.26	2354.45	4305.53	10979.27	11275.15	13897.86	1.404	0.0028	2.545	0.288	-1.794
N0908	Propanoyl phosphate;Prop	C3H7O5P	1.382	C02876	4472.98	4171.63	2454.92	16097.07	8485.9	14114.59	1.286	0.0477	1.321	0.287	-1.802
N0936	Quercetin 3-O-sophoroside	C27H30O17	0.925	C12667	2927.9	2677.67	3756.67	9468.79	11903.53	11510.86	1.418	0.0036	2.442	0.285	-1.812
N1677	Isofenphos	C15H24NO4PS	1.856	C11002	3176	3935.97	3696.45	10725.69	11065.99	16242.43	1.348	0.0348	1.458	0.284	-1.815
N1308	Aztreonam;Monobactam	C13H17N5O8S2	0.709	C06840	1908.11	4196.82	8540.7	19606.43	14626.32	17530.32	1.349	0.0086	2.066	0.283	-1.821
N0507	Nithiamide	C5H5N3O3S	0.958	D05176	19357.29	18824.76	3608.46	44469.98	49034.64	57192.6	1.366	0.0062	2.207	0.277	-1.850
N0989	Plocamene D	C10H13Cl3	0.950	C17105	1187.29	1237.08	136.09	3191.29	2567.69	3539.99	1.340	0.0091	2.040	0.275	-1.861
P0806	5-Methylbarbiturate;5-Met	C5H6N2O3	1.418	C05281	9350.67	6492.75	7684.04	28553.32	28265.4	28644.14	1.501	0.0014	2.869	0.275	-1.861
P2379	Fluoroacetyl-CoA;Fluoroa	C23H37FN7O17P3	0.720	C19775	3703.35	3391.14	5552.84	15207.63	16159.78	15031.5	1.492	0.0007	3.179	0.273	-1.875
P2303	Molybdopterin guanine dir	C20H24N10O13P2	0.720	C19928	16981.19	15650.45	18303.51	57599.94	58390.18	72305.02	1.474	0.0093	2.031	0.271	-1.886
P1706	Rimantadine	C12H21N	0.802	C07236	1180.93	2579.32	1766.5	5174.77	7603.25	7784.41	1.412	0.0140	1.853	0.269	-1.896
P2167	L-Tyrosine methyl ester	C10H13NO3	0.919	C03404	3898.29	1064.36	5376.65	11482.75	16610.03	11040.57	1.369	0.0149	1.826	0.264	-1.920
N1790	Coenzyme II (β-NADP)	C21H29N7O17P3	1.233	C00006	2066.16	6963.42	3252.48	16103.11	12969.96	17698.2	1.367	0.0048	2.318	0.263	-1.929
P0249	Dinitramine	C11H13F3N4O4	2.248	C18785	19242.95	196.94	17583.33	48443.25	43108.81	49454.39	1.413	0.0212	1.674	0.263	-1.929
P1665	Squalene;Spinacene;Supra	C30H50	9.344	C00751	108.26	189.5	162.56	609.63	540.17	614.43	1.424	0.0002	3.691	0.261	-1.938
N1246	Benzoic acid	C7H6O2	1.523	C00180	6626.05	9767.79	9628.9	31098.03	33055.77	35607.13	1.436	0.0002	3.765	0.261	-1.939
N0249	N-Acetyl-D-mannosamine	C8H16NO9P	1.216	C04257	2042.22	6972.34	3400.79	15116.22	18415.64	14134.46	1.375	0.0041	2.388	0.260	-1.941

P1709	Reactive blue 2	C29H20ClN7O11S	0.720	C13746	2332.37	1186.81	1121.32	5451.02	5214.6	7184.61	1.430	0.0066	2.183	0.260	-1.944
P0347	Tacrine	C13H14N2	4.425	C01453	1059.69	914.14	435.97	3795.5	2835.27	2748.35	1.428	0.0079	2.101	0.257	-1.961
N0679	N'-Phosphoguanidinoethyl	C4H13N3O7P2	0.726	C04503	5234.66	3589.93	4935.78	16835.64	17437.37	19316.33	1.435	0.0003	3.557	0.257	-1.961
N1735	Hexachloroacetone	C3Cl6O	0.846	C19122	3820.99	4484.9	177.49	7915.18	11870.47	13560.12	1.287	0.0197	1.706	0.254	-1.975
N1614	Erythritol;Erythrol;Phycito	C4H10O4	1.424	C00503	2910.06	3379.96	3108.76	13634.55	10752.74	12816.81	1.423	0.0073	2.137	0.253	-1.985
P2501	Ibudilast	C14H18N2O	2.730	D01385	15092.51	17629.68	6785.18	54037.09	51015.51	51676.78	1.483	0.0043	2.363	0.252	-1.988
P1632	Plumieride	C21H26O12	2.356	C09797	41024.29	40813.48	44092.03	175376.15	149192.51	175231.42	1.491	0.0044	2.360	0.252	-1.989
N1017	Solanine	C45H73NO15	12.271	C10820	1230.7	2293.66	4184.76	10444.31	10809.23	9400.05	1.405	0.0047	2.332	0.251	-1.991
N0410	Propionic acid	C3H6O2	1.332	C00163	6094.03	5921.86	4882.41	22573.99	21669.26	23373.9	1.444	0.0000	4.723	0.250	-2.001
N1941	D-(+)-Glyceric acid; LC-E	C3H6O4	0.875	C00258	4157.28	3556.21	2391.83	15685.83	10646.5	14346.06	1.381	0.0136	1.866	0.248	-2.009
P0805	Cholesterol	C27H46O	9.286	C00187	31142.38	30256.72	23467.72	114995.41	115039.13	115592.71	1.502	0.0001	3.971	0.246	-2.026
P2485	hexachlorophene	C13H6Cl6O2	2.356	C08039	5233.68	737.51	1474.25	12554.06	9056.41	8885.58	1.357	0.0145	1.838	0.244	-2.034
P1624	1,2-Bis(4-hydroxy-3-meth	C16H16O4	2.356	C04547	2883.55	3108.81	2251.51	12451.88	12079.66	10346.79	1.486	0.0020	2.695	0.236	-2.081
P1002	7alpha-Hydroxy-3-oxo-4-c	C27H42O4	7.491	C17337	21033.08	16593.99	17657.28	66721.54	78518.4	89987.05	1.469	0.0101	1.997	0.235	-2.089
N0667	N-Acetylgalactosaminate	C8H13NO7	2.354	C03408	4581.48	3019.4	6301.52	18804.15	17155.18	23441.6	1.396	0.0058	2.235	0.234	-2.095
P2291	Myxalamid S	C25H41NO4	6.797	C12157	5898.83	5403.17	215.56	20562.21	12793.76	16449.69	1.371	0.0127	1.898	0.231	-2.112
N0099	BUCLADESINE	C18H24N5O8P	2.354	D07546	111399.8	114332.3	104505.98	492009.62	426271.79	514156.76	1.433	0.0047	2.327	0.231	-2.117
P2079	Callystatin A	C29H44O4	7.699	C16891	48975.02	37475.6	36988.81	147049.59	183735.12	206786.92	1.459	0.0123	1.911	0.230	-2.123
P0569	N-Octanoyl-L-homoserine	C12H21NO3	7.491	C21199	63805.51	52238.7	52022.21	214014.03	238808.19	280329.43	1.475	0.0083	2.079	0.229	-2.125
N0069	Lumichrome	C12H10N4O2	4.423	C01727	741.13	1013.74	574.86	2956.63	4034.63	3205.99	1.400	0.0079	2.102	0.228	-2.130
N0013	N-Acetylglucosamine 1-Pl	C8H16NO9P	1.166	C04501	34177.92	31611.89	27096.11	137620.72	150401.14	118783.66	1.425	0.0056	2.250	0.228	-2.131
N1638	Fentin hydroxide;Fentin	C18H16OSn	0.917	C18729	4386.88	2571.25	1386.7	13363.34	9735.4	13491.17	1.378	0.0047	2.326	0.228	-2.132
N0661	Monobasic calcium phospl	Ca(H2PO4)2	0.859	C13556	3305.91	3941.7	2438.14	10811.58	15267.06	17640.39	1.363	0.0253	1.596	0.222	-2.174
N0582	Levosimendan	C14H12N6O	1.150	D04720	3336.86	5899.09	2632.12	17822.88	17085.62	18758.99	1.430	0.0013	2.898	0.221	-2.177
N0584	Lonafarnib	C27H31Br2ClN4O	0.726	D04768	1338.08	1609.78	4270.88	10897.84	11041.61	11676.92	1.415	0.0079	2.101	0.215	-2.219
N1301	Antimony trioxide	Sb2O3	0.855	C19192	2106.51	1760.26	446.06	5493.25	6281.82	8443.52	1.352	0.0118	1.929	0.213	-2.229
N1360	2-Hydroxymethylclavam	C6H9NO3	2.720	C17357	5919.14	4622.71	3695.25	22252.04	23204.53	21899.39	1.443	0.0001	4.052	0.211	-2.242
P1822	Ceftriaxone	C18H18N8O7S3	0.716	C06683	1412.75	805.21	660.33	4070.97	5058.18	5224.05	1.470	0.0018	2.755	0.201	-2.318
N1271	BUCLADESINE	C18H24N5O8P	2.354	D07546	12130.71	10768.31	15968.91	68858.75	59117.86	70595.53	1.433	0.0014	2.869	0.196	-2.353
N1625	Florasulam	C12H8F3N5O3S	0.726	C18850	1537.65	1115.15	2098.31	8307.01	9384.03	7345.25	1.422	0.0023	2.642	0.190	-2.398
N0869	Thifluzamide	C13H6Br2F6N2O2	0.834	C18505	4056.13	2377.48	2068.24	13328.41	14756.83	17081.24	1.419	0.0019	2.732	0.188	-2.409
P1757	Chromafenozide	C24H30N2O3	2.356	C18515	6105.49	10170.78	6186.27	41106.06	36372.43	42075.03	1.492	0.0002	3.707	0.188	-2.412
P2444	4-Acetamido-4'-isothiocya	C17H14N2O7S3	0.720	C13702	1964.57	794.95	2420.21	7690.2	9210.62	10677.01	1.457	0.0041	2.390	0.188	-2.413
N0984	Phenobenzuron	C16H14Cl2N2O2	1.615	C19090	5561.43	6240.03	3725.12	27824.22	27567.53	27472.42	1.445	0.0009	3.036	0.187	-2.416
N0807	UDP-2,3-diacetamido-2,3-	C19H28N4O18P2	1.224	C19725	1616.99	1785.51	616.15	8304.65	8814.47	4573.74	1.309	0.0399	1.399	0.185	-2.432
P1190	Penbutolol	C18H29NO2	2.365	C07416	2515.82	4111.87	4338.44	20759.26	17075.76	22272.47	1.477	0.0042	2.375	0.182	-2.454
N0976	Pentanoate;Valerate;Pentai	C5H10O2	2.737	C00803	5766.47	3551.4	3108.35	23290.36	22296.82	23054.39	1.441	0.0006	3.193	0.181	-2.466
N0441	Aspidospermatine	C21H26N2O2	10.547	C09041	5178.85	2695.5	2333.9	20166.24	16342.6	20982.27	1.415	0.0016	2.790	0.178	-2.494
N0852	Timepidium bromide;Time	C17H22NOS2.Br	1.158	C12768	2007.06	2500.62	1652.41	13430.27	11376.87	9941.14	1.414	0.0082	2.088	0.177	-2.496
P1887	Dihydroneopterin triphosp	C9H16N5O13P3	0.719	C04895	796.71	727.73	1428.85	4804.24	6807.16	5557.95	1.456	0.0080	2.098	0.172	-2.539
N0669	N-Acetyl-L-Leucine	C8H15NO3	3.626	C02710	4396.79	3943.92	3895.94	20806.47	31118.86	19610.49	1.359	0.0323	1.491	0.171	-2.547
P2513	Hydroflumethiazide	C8H8F3N3O4S2	0.720	C07763	3191.81	2370.35	4015.02	16812.06	18600.81	20865.33	1.487	0.0021	2.677	0.170	-2.555
N1858	Cellobionate;Cellobionic a	C12H22O12	0.834	C20888	23267.33	716.58	780.05	48750.45	49404.08	47557.86	1.355	0.0324	1.490	0.170	-2.557
N1137	Acesulfame-K	C4H5NO4S	0.834	D08836	4016.87	3577.89	2949.66	21032.46	20303.01	21169.36	1.446	0.0000	5.636	0.169	-2.567
P2268	Methotrexate	C20H22N8O5	1.314	C01937	8639.17	1167.32	1428.11	24003.92	19796.79	22827.98	1.443	0.0069	2.162	0.169	-2.568
P1686	Sulfadimethoxine sodium	C12H13N4O4S.Na	1.210	C18386	1769.03	2976.42	2548.04	14373.75	15104.76	14578.51	1.502	0.0000	4.416	0.166	-2.595
N1508	2-Oxophytanate;2-Oxophy	C20H38O3	12.258	C02117	326.54	226.74	624.37	2551.12	2784.11	1815.43	1.380	0.0115	1.939	0.165	-2.602
P0947	7alpha-Hydroxy-3-oxo-4-c	C27H42O4	7.499	C17337	2341.19	2528.03	2639.6	13403.55	15045.89	17382.35	1.482	0.0078	2.108	0.164	-2.610
P0942	N-Acetyl-D-glucosamine 6	C8H16NO9P	1.168	C00357	2041.87	1947.97	2285.64	11022.2	13876.86	13427.73	1.485	0.0063	2.202	0.164	-2.611
P2223	Isopentenyl phosphate	C5H11O4P	1.492	C20345	5451.38	177.9	262.77	12547.32	11055.27	12930.62	1.417	0.0197	1.706	0.161	-2.632
N1233	Bis(glycerophospho)-glyce	C9H22O13P2	1.216	C03853	2164.89	3528.14	2097.95	18941.84	13098.08	16747.02	1.401	0.0108	1.965	0.160	-2.647
N0328	Pyruvic acid	C3H4O3	1.233	C00022	16813.57	21398.34	10199.11	107378.54	99484.56	101059.47	1.441	0.0001	4.277	0.157	-2.669
N1031	Sodium 3-ethyl-7-isopropy	C15H17O3S.Na	1.515	C13414	898.41	1927.84	3031.99	8627.27	12995.43	16100.73	1.335	0.0314	1.504	0.155	-2.687
N0925	Potassium sulfate	K2O4S	0.834	C13192	1563.74	1636.5	700.02	6244.81	8411.38	10785.56	1.357	0.0268	1.572	0.153	-2.706
P1773	CoA;Coenzyme A;CoA-SI	C21H36N7O16P3S	0.720	C00010	2619.19	1189.73	1310.92	10182.95	11693.71	11587.72	1.491	0.0001	3.827	0.153	-2.708
N1606	Ethyl loflazepate	C18H14ClFN2O3	0.726	D01293	610.64	845.46	809.98	3270.14	6229.3	5505.72	1.337	0.0405	1.393	0.151	-2.727
P0991	Adenosine	C10H13N5O4	1.742	C00212	111707.56	58373.8	40307.76	490977.97	620973.98	288494.66	1.344	0.0488	1.312	0.150	-2.735
N1954	D-(+)-Raffinose; LC-ESI-(	C18H32O16	0.830	C00492	570.64	682.02	265.03	3016.66	4061.32	3400.71	1.415	0.0046	2.340	0.145	-2.788
N0655	Methylselenocysteine Se-o	C4H9NO3Se	0.950	C18905	783.11	1183.86	1397.75	7307.6	7696.7	8575.5	1.438	0.0006	3.189	0.143	-2.809
N1254	Capecitabine;(1-(5-Deoxy-	C15H22FN3O6	2.446	C12650	2772.49	4873.41	2272.26	22565.58	24430.44	23528.49	1.442	0.0001	4.092	0.141	-2.830
P1656	Slaframine;(-)-Slaframine	C10H18N2O2	1.276	C06185	3148.56	3810.27	3983.67	24012.36	35007.59	19987.45	1.398	0.0366	1.436	0.138	-2.852
N0356	Methyl farnesoate;Methyl (	C16H26O2	9.998	C16503	206.07	630.75	255.48	1631.79	3318.86	2959.1	1.314	0.0400	1.398	0.138	-2.856
P1407	Tetradecanol	C14H30O	6.037	D05097	15936.3	15731.69	14262.66	113372.28	115006.94	110669.96	1.505	0.0000	4.737	0.135	-2.884
P2036	Alloxanthine;Oxipurinol;C	C5H4N4O2	1.650	C07599	605.13	1601.02	13300.53	37375.41	38767.22	39437.36	1.462	0.0131	1.882	0.134	-2.898
N0481	Oxalureate;Oxaluric acid;N	C3H4N2O4	0.958	C00802	1140.43	1543.05	20958.3	68586.76	55613.85	55384.12	1.386	0.0043	2.362	0.132	-2.925
N0325	Pyruvic acid	C3H4O3	0.958	C00022	16250.24	20049.15	11195.39	127439.16	130619.12	104651.5	1.429	0.0033	2.487	0.131	-2.933
P0803	L-Methionine;Methionine;	C5H11NO2S	0.919	C00073	12095.03	4526.31	10976.19	83596.27	83721.66	43911.48	1.376	0.0401	1.396	0.131	-2.936
P0074	Phenethyl alcohol	C8H10O	2.149	C05853	495.57	1071.37	831.88	6732.7	5832.74	6598	1.495	0.0003	3.580	0.125	-2.998
N1472	4-Methyl-2-oxopentanoate	C6H10O3	2.990	C00233	1775.95	2034.35	2375.28	19880.86	12965.3	17285.51	1.395	0.0178	1.749	0.123	-3.019
N0993	Pinocembrin 7-rhamnosyl	C27H32O13	0.817	C09828	1149.5	1672.69	2739.36	11527.22	19145	15593.06	1.376	0.0214	1.669	0.120	-3.056
P0372	Propamocarb	C9H20N2O2	0.909	C18885	7067.74	10803.03	8765.88	73345.17	76847.09	75185.05	1.504	0.0000	5.807	0.118	-3.081
N1088	Ricinoleic acid	C18H34O3	7.531	C08365	794.41	974.95	861.42	10234.27	6241.2	5892.82	1.332	0.0418	1.378	0.118	-3.088
P0970	N5-Phenyl-L-glutamine;ga	C11H14N2O3	1.958	C20843	276.09	302.75	4327.75	15219.78	11690.71	14899.28	1.448	0.0024	2.612	0.117	-3.091
P0934	alpha-Isopropylmalate;(2S	C7H12O5	1.717	C02504	75618.42	83084.4	71292.59	645810.46	656728.55	702636.19	1.503	0.0006	3.239	0.115	-3.124
N1717	Glucoraphanin;4-Methylsu	C12H23NO10S3	1.490	C08419	105.85	101.31	3170.83	10047.8	9950.13	9721.64	1.411	0.0127	1.896	0.114	-3.137
P1660	1-(beta-D-Ribofuranosyl)-	C11H16N2O5	1.472	C15497	1167.77	1435.24	807.92	12215.1	11106.7	8227.02	1.457	0.0141	1.852	0.108	-3.209
P0990	Adenosine	C10H13N5O4	1.742	C00212	12369.64	3179.5	1555.6	52845.45	70151.99	35480.29	1.372	0.0318	1.498	0.108	-3.212
N1399	(15S)-15-Hydroxy-5,8,11-	C20H32O3	7.157	C04742	49.01	2589.39	140.18	9763.15	6305.71	9811.08	1.358	0.0075	2.123	0.107	-3.219
P0373	Propanil	C9H9Cl2NO	1.617	C14229	24004.64	29484.42	24713.8	205268.31	284090.24	297704.41	1.463	0.0143	1.845	0.099	-3.331
P0411	Xanthosine	C10H12N4O6	1.650	C01762	3547.86	2267.53	1329.4	24698.31	25272.6	23046.95	1.500	0.0000	4.721	0.098	-3.353
N0121	Mevalonic acid	C6H12O4	2.986	C00418	48332.06	98479.65	61589.36	751824.42	636306.65	796486.18	1.432	0.0027	2.562	0.095	-3.390
P0941	N-Acetyl-D-glucosamine 6	C8H16NO9P	1.160	C00357	1599.98	1131.92	447.9	10693.16	12435.16	10541.83	1.491	0.0006	3.247	0.094	-3.404
N1867	Dihydroergocristine mesyl	C35H41N5O5.CH4	2.138	C13168	122.69	9782.22	144.8	30855.84	47466.34	32026.16	1.358	0.0101	1.995	0.091	-3.457
P1268	N6,N6,N6-Trimethyl-L-lys	C9H20N2O2	1.218	C03793	2635.92	2773.52	633.36	24570.45	20456.09	21455.71	1.490	0.0006	3.224	0.091	-3.460
P1303	2-Amino-5-chlorophenol	C6H6ClNO	1.717	C20669	66764.91	86182.92	75151.24	807300.3	831121.84	872746.55	1.504	0.0003	3.602	0.091	-3.461
P0140	Isoscopoletin	C10H8O4	2.656	C18079	9047.89	2555.14	1697	52329.51	45583.3	48709.04	1.492	0.0002	3.818	0.091	-3.463
N1384	2-Ketobutyric acid	C4H6O3	0.950	C00109	4548.08	2026.49	2461.3	37581.37	29366.21	40747.71	1.416	0.0079	2.102	0.084	-3.575
N0673	MTIC	C5H8N6O	1.656	C16250	2203.29	1424.22	1129.37	20912.99	18871.58	17741.83	1.439	0.0011	2.951	0.083	-3.596
N1138	acanthoside B	C28H36O13	0.817	C10890	4345.01	3728.67	8109.29	53415.26	74184.26	70020.07	1.417	0.0085	2.073	0.082	-3.610
N0019	Xanthosine	C10H12N4O6	1.656	C01762	11299.57	1991.75	4313.84	79323.43	75919.8	73031.12	1.441	0.0001	4.026	0.077	-3.697
P0800	GR 89696	C19H25Cl2N3O3	1.384	C00387	1029.75	201.89	276.54	6832.81	9619.37	4597.05	1.369	0.0424	1.372	0.072	-3.803
P1609	Philanthotoxin 74	C24H42N4O3	7.697	C20052	323.54	643.96	1174.61	6439.31	10687.49	12922.59	1.394	0.0374	1.427	0.071	-3.810
P1316	N1,N12-Diacetylspermine	C14H30N4O2	0.819	C03413	2666.6	952.77	971.41	21463.54	22581.9	22677.98	1.502	0.0000	4.667	0.069	-3.861
N0875	TOPIRAMATE	C12H21NO8S	2.354	C07502	1797.32	748.19	627.87	13213.24	15139.88	18494.65	1.415	0.0082	2.088	0.068	-3.884
P2002	alpha-Keto-gamma-(methy	C5H8O3S	1.717	C01180	953.85	620.2	1471.66	15130.95	16052.21	18001.7	1.496	0.0016	2.804	0.062	-4.013
P1909	Cyclohexylamine;Cyclohe	C6H13N	6.037	C00571	297.02	631.37	562.83	8273.14	7556.3	8888.12	1.498	0.0014	2.856	0.060	-4.051
P1226	Ozagrel	C13H12N2O2	1.733	D08327	1801.89	575.07	1134.79	24442.41	16455.61	17806.04	1.452	0.0160	1.796	0.060	-4.063
P1793	Chlorodifluoromethane	CHClF2	1.720	C19361	2285.41	2379.73	2928.03	39866.34	42066.72	45912.24	1.500	0.0017	2.762	0.059	-4.074
N1940	D-(-)-Erythrose	C4H8O4	1.224	C01796	890.89	2037.98	2334.74	33889.8	24493.77	33303.71	1.416	0.0098	2.010	0.057	-4.123
P2505	3-Fluoro-cis,cis-muconate	C6H5FO4	1.717	C16474	1123.72	3189.08	2682.61	41430.2	47869.77	48205.14	1.498	0.0014	2.867	0.051	-4.297
P0995	2-Hydroxy-6-ketononatrie	C9H8O6	1.617	C12624	796.48	2746.36	570.07	20665.65	29930.87	30713.78	1.459	0.0124	1.906	0.051	-4.305
N0330	Xanthosine	C10H12N4O6	1.656	C01762	481.7	163.9	87.47	5133.9	5977.45	4390.93	1.422	0.0058	2.238	0.047	-4.402
N0103	Nitrilotriacetic acid	C6H9NO6	2.653	C14695	8557.93	4852.66	6039.18	155924.17	120206.82	157923.2	1.425	0.0074	2.131	0.045	-4.480
P1011	7alpha-Hydroxy-3-oxo-4-c	C27H42O4	7.524	C17337	16731.09	12977.15	11893.91	275783.71	331420.23	383557.25	1.478	0.0094	2.026	0.042	-4.574
N1239	Betonicine	C7H13NO3	2.786	C08269	375.57	497.45	905.54	14238.48	12099.18	16146.45	1.427	0.0065	2.185	0.042	-4.578

P0428	terpinolene	C10H16	6.843	C06075	55.35	96.93	83.78	1727.82	2700.63	1472.52	1.398	0.0370	1.432	0.040	-4.644
P1230	N8-Acetylspermidine	C9H21N3O	0.728	C01029	753.15	518.22	1161.8	19280.93	21346.8	20727.65	1.502	0.0004	3.415	0.040	-4.656
N1640	Fenson	C12H9ClO3S	0.975	C19030	661.68	230.36	118.52	9523.71	9876.64	9769.27	1.447	0.0000	5.175	0.035	-4.851
P1975	Anthranilate;Anthranilic ac	C7H7NO2	2.660	C00108	1691.91	1361.17	907.53	42040.64	29771.13	45424.79	1.461	0.0153	1.815	0.034	-4.888
N0620	N-Nitrosomorpholine	C4H8N2O2	3.576	C19283	210.45	337.19	184.51	7516.92	7664.73	7352.96	1.448	0.0000	5.250	0.032	-4.944
P0219	Adenosine	C10H13N5O4	2.656	C00212	1519.74	782.1	756.45	39099.3	35663.41	33069.77	1.497	0.0021	2.678	0.028	-5.140
N0665	N-Acetylimidazole;1-Acet	C5H6N2O	0.950	C02560	74.61	161.27	390.32	10002.38	5745.49	8927.24	1.379	0.0240	1.620	0.025	-5.300
P1602	Phylloquinone;Vitamin K1	C31H46O2	7.799	C02059	250.89	490.22	536.08	15887.54	15620.56	19798.49	1.487	0.0063	2.200	0.025	-5.328
N0948	Pyocyanine;Pyocyanin;5-M	C13H10N2O	1.166	C01748	250.49	95.35	99.98	5655.96	7199.2	5221.1	1.419	0.0099	2.006	0.025	-5.341
P0562	Nandrolone	C18H26O2	3.960	C07254	1365.15	425.19	783.95	34154.74	32928.96	37738.91	1.500	0.0013	2.887	0.025	-5.348
P0522	Phylloquinone	C31H46O2	7.524	C02059	338.43	353.65	363.63	12693.2	17294.92	20764.03	1.450	0.0193	1.714	0.021	-5.587
P0888	D-Octopine;N2-(D-1-Cart	C9H18N4O4	1.133	C04137	637.86	674.23	424.44	32400.74	26217.55	25324.49	1.485	0.0065	2.189	0.021	-5.595
P0998	7alpha-Hydroxy-3-oxo-4-c	C27H42O4	7.524	C17337	3765.45	735.13	1355.16	86376.03	103190.75	121623.71	1.477	0.0094	2.027	0.019	-5.732
P1933	Dazomet	C5H10N2S2	1.717	C18457	212.77	1151.75	463.62	33809.9	33351.29	36334.8	1.503	0.0003	3.537	0.018	-5.823
N1914	Creatine	C4H9N3O2	2.994	C00300	145.02	233.69	89.1	9193.53	7473.81	9876.72	1.429	0.0065	2.184	0.018	-5.826
N1910	Dinex	C12H14N2O5	2.662	C19038	1429.17	404.45	517.24	48360.4	40105.2	50502.25	1.434	0.0045	2.350	0.017	-5.885
P0798	Guanosine-3',5'-cyclic mor	C10H12N5O7P	2.131	C00942	11264.32	5958.12	7483.56	615596.82	465954.4	498215.63	1.482	0.0076	2.122	0.016	-5.999
P1001	L-Tryptophan	C11H12N2O2	2.115	C00078	3063.27	7142.22	7013.25	221716.73	467878.07	508821.6	1.372	0.0481	1.318	0.014	-6.121
P0839	Palmitoylcarnitine	C23H45NO4	7.138	C02990	319.09	317.59	430.91	27074.88	25763.04	34452.87	1.481	0.0087	2.059	0.012	-6.353
N1594	Flufenacet	C14H13F4N3O2S	2.130	C18731	866.28	706.8	995.39	95642.07	70802.26	71676.7	1.417	0.0106	1.976	0.011	-6.535
P0791	Guanosine-3',5'-cyclic mor	C10H12N5O7P	2.132	C00942	583.98	1008.36	847.19	95454.79	69443.39	69787.75	1.469	0.0121	1.916	0.010	-6.588
N0061	cis-5,8,11,14,17-Eicosape	C20H30O2	10.547	C06428	3241.32	3271.06	910.74	265878.01	214313.7	267453.46	1.434	0.0049	2.310	0.010	-6.654
P1892	Dihydroaceanthrylene;1,2-	C16H12	1.821	C19401	210.97	1117.71	525.77	60422.73	125077.72	140552.87	1.373	0.0479	1.320	0.006	-7.458
P0324	Rubrofusarin	C15H12O5	1.949	C09047	344.69	868.71	392.2	316944.25	299820.31	324849.46	1.504	0.0006	3.258	0.002	-9.196

Table S3: RNA-seq results of JEG3/MTX cells with or without DPP4 knockdown

GeneName	Fold change	Log2FC	Pvalue	Scr-1	Scr-2	Scr-3	shDPP4-1	shDPP4-2	shDPP4-3
HTR4	0	#NUM!	0.4324683	2	0	2	0	0	0
SNORD121A	1.25	0.3219281	0.8475902	0	0	4	0	2	3
HTR7	0.875	-0.192645	0.9521745	7	0	1	4	0	3
EXOC3-AS1	0.8880866	-0.171228	0.6927549	89	90	98	82	96	68
DHDH	1.8571429	0.8930848	0.2882719	2	14	5	20	6	13
BNC2-AS1	6	2.5849625	0.1459873	0	0	2	7	1	4
DHFR	0.8022343	-0.317904	0.0235281	3353	2850	3733	2349	2849	2773
AC007066.2	1.096	0.1322478	0.5622549	81	63	106	58	116	100
AC097499.1	#DIV/0!	#DIV/0!	0.8030358	0	0	0	0	0	1
RN7SL560P	0	#NUM!	0.4374838	0	4	0	0	0	0
AL390037.1	0	#NUM!	0.824326	0	0	1	0	0	0
MYBPC1	0.8333333	-0.263034	0.9336839	4	0	2	2	0	3
MYBPC2	0.5714286	-0.807355	0.6858519	4	2	1	0	0	4
TMEFF1	2.8461538	1.5090136	0.13472	2	8	3	3	13	21
TMEFF2	#DIV/0!	#DIV/0!	0.6554062	0	0	0	0	2	0
RPS7P11	0.942288	-0.08576	0.8579872	618	613	727	594	678	573
AL353613.1	1	0	0.9856639	0	4	1	1	4	0
RPS7P10	1.1818182	0.2410081	0.3751491	50	45	70	88	61	46
AC064836.1	0	#NUM!	0.824326	1	0	0	0	0	0
AC064836.3	0.8688525	-0.202817	0.7382243	35	43	44	38	41	27
RNU6-652P	2	1	0.8324799	1	0	0	0	2	0
MBD3	1.0047195	0.0067927	0.6694158	507	663	737	684	695	537
MBD4	1.1129286	0.154361	0.2090415	1219	695	1265	1102	1256	1180
MBD5	1.0821918	0.1139562	0.4623712	473	370	471	298	533	591
MBD6	1.0431973	0.061012	0.3998295	870	1014	1056	1046	1035	986
MBD1	0.8101604	-0.30372	0.0570498	1247	1288	1579	1067	1172	1094
MBD2	1.0567515	0.0796361	0.2921442	789	802	964	870	966	864
AC006237.1	1	0	0.9617562	0	0	2	0	0	2
FBXO27	1.2795276	0.3556112	0.1463596	59	73	122	108	122	95
FBXO28	0.8760475	-0.190919	0.3228597	1009	809	1046	688	928	893
FBXO25	1.0924639	0.1275856	0.2167525	624	507	740	574	782	688
FBXO24	0.25	-2	0.0384349	18	12	18	0	3	9
FBXO21	0.8227047	-0.281553	0.0875412	2089	2063	2481	1345	2000	2112
MCHR2-AS1	1	0	0.9667581	4	0	2	3	3	0
FBXO22	0.9503644	-0.073447	0.898255	2191	2390	2692	2160	2442	2310
MYBPHL	0.8	-0.321928	0.890112	4	0	11	4	7	1
AC073592.10	0	#NUM!	0.824326	0	1	0	0	0	0
AC044839.4	0.9090909	-0.137504	0.9490469	6	7	9	7	5	8
FBXO18	1.0788555	0.1095017	0.1664883	1807	1821	2104	1801	2121	2262
FBXO16	0.1	-3.321928	0.0174184	11	6	13	0	3	0
FBXO17	#DIV/0!	#DIV/0!	0.011062	0	0	0	3	6	7
FBXO15	1.9090909	0.9328858	0.3715358	4	5	2	8	8	5
FBXO10	0.9255663	-0.111592	0.8304827	102	104	103	94	101	91
FBXO11	1.1594944	0.2134958	0.1348388	1257	986	1475	943	1626	1742
AC092794.1	0	#NUM!	0.3958203	2	2	0	0	0	0
MINPP1	1.3946785	0.4799326	0.0009801	481	394	478	534	753	600
Z97192.1	0	#NUM!	0.1915466	1	0	7	0	0	0
Z97192.2	0	#NUM!	0.824326	1	0	0	0	0	0
Z97192.3	0	#NUM!	0.6840298	0	0	2	0	0	0
FBXO47	0.8	-0.321928	0.8603706	3	2	0	0	1	3
FBXO48	1.3130081	0.3928758	0.1024874	81	71	94	97	102	124
FBXO45	0.9218898	-0.117334	0.6606295	1219	843	1113	726	1057	1144

FBXO46	0.9802074	-0.028841	0.8081756	863	999	1321	965	1229	926
FBXO43	0.2222222	-2.169925	0.2177025	4	0	14	0	3	1
FBXO44	1.0378549	0.0536047	0.6450359	71	97	149	132	122	75
FBXO41	0.4530154	-1.142368	1.31E-06	204	251	258	102	118	103
FBXO42	0.9547276	-0.066839	0.9328536	735	850	911	713	824	846
GRAMD4P3	0.8604651	-0.216811	0.7871918	14	12	17	1	27	9
FBXO40	0	#NUM!	0.4521054	1	0	3	0	0	0
GRAMD4P4	1	0	0.9890559	0	0	1	0	1	0
GRAMD4P2	0	#NUM!	0.2290435	7	0	0	0	0	0
GRAMD4P8	0.875	-0.192645	0.950071	2	0	6	0	4	3
MBIP	1.0512821	0.0721498	0.4092897	935	647	992	755	963	988
Z73965.1	0.6666667	-0.584963	0.6268946	10	2	18	0	10	10
AC073636.1	0	#NUM!	0.6385096	0	2	0	0	0	0
FBXO38	1.1428571	0.1926451	0.6984877	13	9	20	12	22	14
FBXO39	#DIV/0!	#DIV/0!	0.8030358	0	0	0	0	0	1
HTR1D	3.5	1.8073549	0.4195205	0	1	1	0	2	5
FBXO36	1.1878261	0.2483236	0.1293393	172	162	241	214	213	256
FBXO34	0.9563679	-0.064362	0.9505683	877	689	978	656	869	908
FBXO32	0.6153846	-0.70044	0.478474	11	4	24	6	11	7
FBXO33	1.1739879	0.2314176	0.0446701	755	694	873	735	956	1035
FBXO30	0.9241928	-0.113734	0.7640231	712	514	911	437	736	802
FAM90A1	0.875	-0.192645	0.7643992	32	35	53	18	34	53
FBXO31	0.9620833	-0.055766	0.9876071	642	848	910	650	831	828
AGAP1-IT1	2.14	1.0976108	0.0258037	16	12	22	30	49	28
HTR2B	0	#NUM!	0.6840298	0	0	2	0	0	0
HTR2C	0.8003597	-0.32128	0.2563304	220	149	187	100	177	168
HCFC1-AS1	#DIV/0!	#DIV/0!	0.8030358	0	0	0	0	1	0
AL160006.1	0.8580645	-0.220842	0.4107874	200	212	208	160	189	183
VPS13C	0.8666424	-0.206491	0.4845929	1132	545	1075	486	916	983
DHPS	0.8310149	-0.267054	0.220691	545	628	768	551	575	487
VPS13D	0.8808681	-0.183002	0.3620449	1422	1347	1470	894	1310	1530
VPS13A	1.4341987	0.520245	0.1963324	944	407	883	622	1149	1433
VPS13B	0.9331713	-0.099786	0.7905542	605	379	662	304	655	577
CD24P4	0.4081633	-1.292782	0.1052252	14	14	21	6	6	8
CD24P1	#DIV/0!	#DIV/0!	0.8030358	0	0	0	1	0	0
AC106774.2	2	1	0.7782384	0	0	1	2	0	0
IL2RG	0.75	-0.415037	0.5793897	7	28	21	7	25	10
AL078581.1	6	2.5849625	0.3547041	0	0	1	0	3	3
AL078581.3	3.3333333	1.7369656	0.3636243	1	0	2	2	8	0
GPKOW	0.9555328	-0.065623	0.9981718	1052	1360	1501	1255	1299	1185
AC093382.1	1	0	0.9745101	2	7	0	0	0	9
RD62P1-PAR	0.25	-2	0.4566761	0	0	8	0	1	1
IL2RA	#DIV/0!	#DIV/0!	0.6411933	0	0	0	0	0	2
IL2RB	3.2835821	1.7152705	1.75E-05	15	25	27	53	96	71
PEX26	0.8495968	-0.23515	0.1536185	1499	1666	1795	1144	1503	1567
IL36A	#DIV/0!	#DIV/0!	0.6411933	0	0	0	0	0	2
IL36B	2.6666667	1.4150375	0.2214312	4	0	5	4	14	6
AC098828.3	0.2	-2.321928	0.4605013	5	0	0	0	0	1
AC098828.2	1.6666667	0.7369656	0.7098196	0	0	3	2	3	0
CRISPLD1	0.8812416	-0.182391	0.3995604	459	515	508	329	532	445
CRISPLD2	1.0663215	0.0926424	0.2046947	2679	2985	3413	2759	3470	3450
DHX8	0.7992293	-0.323319	0.0216215	2469	2508	2808	1723	2220	2279
DHX9	0.9555632	-0.065577	0.8962688	13144	10562	14078	9466	13343	13296
AC116533.1	0.9532164	-0.069124	0.9715761	630	623	799	635	739	582

AC080013.1	0.4285714	-1.222392	0.3438551	7	2	12	1	3	5
MC2R	0	#NUM!	0.5781912	0	0	3	0	0	0
AC080013.2	0	#NUM!	0.6385096	0	2	0	0	0	0
AC080013.3	0.6666667	-0.584963	0.6979564	5	3	16	7	9	0
AC080013.4	0.7380952	-0.438121	0.6248322	15	10	17	0	23	8
AC080013.5	0.1666667	-2.584963	0.3630032	4	0	2	0	1	0
AC080013.6	0.7272727	-0.459432	0.7576599	4	4	3	2	6	0
SLC10A4	0.6666667	-0.584963	0.7646954	5	0	7	3	1	4
SLC10A5	0.7	-0.514573	0.7738244	6	0	4	1	6	0
SLC10A6	0	#NUM!	0.6436235	2	0	0	0	0	0
SLC10A7	1.1530984	0.2055157	0.1468832	558	478	610	443	723	732
SLC10A1	5.5	2.4594316	0.1757001	1	1	0	7	3	1
SLC10A2	0	#NUM!	0.5337954	0	3	0	0	0	0
SLC10A3	0.8885536	-0.170469	0.5147087	1398	1693	1880	1588	1575	1254
IL36G	#DIV/0!	#DIV/0!	0.8030358	0	0	0	0	1	0
PEX5L	4	2	0.5810589	0	0	1	0	4	0
MC1R	1.0496454	0.069902	0.7792434	38	59	44	43	57	48
AL021368.2	1.1506849	0.2024929	0.646986	35	5	33	23	30	31
OR3A4P	#DIV/0!	#DIV/0!	0.8030358	0	0	0	0	0	1
ZHX2	#DIV/0!	#DIV/0!	0.5415473	0	0	0	0	2	1
ZHX3	0.7940651	-0.332671	0.0458996	900	939	958	601	796	824
CACYBP	1.0995236	0.1368785	0.0836602	4811	4622	5679	4994	6405	5217
ZHX1	1.2102473	0.2753019	0.1304271	440	233	459	338	525	507
RNF144A	1.1571146	0.2105318	0.1082779	938	1095	1003	904	1290	1319
MC3R	#DIV/0!	#DIV/0!	0.8030358	0	0	0	0	0	1
RNF144B	0.7055215	-0.503238	0.002407	1652	1520	1881	830	1343	1392
AP005057.1	1.2307692	0.2995603	0.7614841	3	6	4	5	7	4
PEX16	0.84243	-0.247371	0.2562357	659	711	737	632	584	559
PEX19	0.9769737	-0.033608	0.841213	895	971	1174	909	1099	962
PEX10	0.9526112	-0.070041	0.976703	297	357	380	340	327	318
PEX12	0.7857143	-0.347923	0.1747642	182	240	292	154	216	191
PEX13	1.0299662	0.0425969	0.4449879	1331	1226	1581	1167	1520	1575
PEX14	1.065995	0.0922006	0.2887758	554	650	781	648	740	728
AL033523.1	0	#NUM!	0.824326	1	0	0	0	0	0
RN7SL559P	0	#NUM!	0.0419516	13	0	1	0	0	0
INPPL1	0.7752164	-0.367329	0.0116021	3739	4177	4794	3218	3339	3296
IGF1R	0.7188604	-0.476216	0.0022803	2811	2963	3001	1580	2231	2497
PPIP5K1	1.0578556	0.0811427	0.324932	622	560	702	619	731	643
PPIP5K2	1.0816149	0.113187	0.4677019	1902	958	1747	1061	1920	2002
MIR6124	0	#NUM!	0.824326	1	0	0	0	0	0
SNORA59A	3	1.5849625	0.6529844	0	0	1	0	1	2
RN7SL558P	0.4444444	-1.169925	0.4827206	2	6	1	1	2	1
DHDDS	1.0025478	0.003671	0.62662	2020	2456	2589	2283	2481	2319
AC006238.1	6	2.5849625	0.3391613	0	0	1	6	0	0
RBMS2P1	0.5238095	-0.932886	0.430049	13	4	4	4	6	1
AL390038.1	#DIV/0!	#DIV/0!	0.8030358	0	0	0	0	1	0
HTRA3	0.0833333	-3.584963	0.1287608	0	4	8	0	0	1
HTRA4	1.2967033	0.3748484	0.3365407	36	17	38	33	45	40
HTRA1	2	1	0.7703774	2	0	0	0	4	0
HTRA2	1.1932921	0.2549473	0.0345923	668	797	801	850	999	855
RNU7-3P	#DIV/0!	#DIV/0!	0.1664889	0	0	0	0	7	1
AL391845.1	#DIV/0!	#DIV/0!	0.8030358	0	0	0	1	0	0
AL391845.2	#DIV/0!	#DIV/0!	0.8030358	0	0	0	0	1	0
HULC	11	3.4594316	0.1109113	0	1	0	8	3	0

HULC	11	3.4594316	0.1109113	0	1	0	8	3	0
ZNF350-AS1	0.6470588	-0.628031	0.6833829	7	0	10	2	8	1
CHCHD10	0.9665308	-0.049112	0.8363167	1845	2349	2678	2804	2018	1820
IGF2R	0.6732356	-0.570817	0.0011919	9069	11010	10215	4538	7334	8523
AL355916.1	0.8518519	-0.231326	0.7881574	34	12	35	25	27	17
AL355916.2	1.65	0.722466	0.3965202	4	8	8	7	4	22
AC008385.1	0	#NUM!	0.4460243	4	0	0	0	0	0
AP000842.3	0.7619048	-0.392317	0.720286	12	3	6	0	12	4
AL353759.1	0.5454545	-0.874469	0.4204439	11	5	6	2	5	5
AP003973.2	#DIV/0!	#DIV/0!	0.4785195	0	0	0	1	1	1
AC034206.1	0.5	-1	0.428795	2	13	7	3	8	0
ZIC2	1.0866142	0.1198398	0.2934702	277	255	357	311	314	341
ZIC1	0.7947368	-0.331451	0.1321798	327	272	351	208	305	242
HUNK	1.1031066	0.1415722	0.1464827	2663	3028	3290	2579	3625	3703
IGHV2OR16-5	#DIV/0!	#DIV/0!	0.6554062	0	0	0	0	2	0
RNU6-645P	#DIV/0!	#DIV/0!	0.8030358	0	0	0	0	0	1
CLVS1	2.3333333	1.2223924	0.4216646	4	2	0	5	8	1
AL138885.2	0	#NUM!	0.5075476	0	0	4	0	0	0
ZIC4	0.7261538	-0.461653	0.1817079	129	79	117	53	83	100
ZIC5	1.0291971	0.0415193	0.6915224	147	114	150	132	168	123
RNU6-647P	0	#NUM!	0.824326	1	0	0	0	0	0
AL034351.1	#DIV/0!	#DIV/0!	0.8030358	0	0	0	0	1	0
MNX1-AS1	1.0801688	0.1112568	0.5671952	51	91	95	112	70	74
MIR6133	0	#NUM!	0.4374838	0	4	0	0	0	0
MNX1-AS2	1.5185185	0.6026645	0.4121716	9	13	5	13	14	14
RPL36AP13	0.375	-1.415037	0.6184693	0	0	8	2	1	0
RPL36AP15	0.2727273	-1.874469	0.365522	10	0	1	0	0	3
SCN11A	0.5	-1	0.8652929	0	0	2	0	0	1
RPL36AP21	1	0	0.9890559	1	0	0	0	0	1
SSTR5-AS1	0.6666667	-0.584963	0.8272397	3	0	0	0	1	1
RPL36AP29	0	#NUM!	0.6410557	1	1	0	0	0	0
RPL36AP26	0	#NUM!	0.824326	0	1	0	0	0	0
HUS1	0.8795518	-0.18516	0.4756709	416	472	540	427	417	412
RN7SL574P	0.3333333	-1.584963	0.3610357	4	5	3	0	0	4
SRFBP1	0.9180328	-0.123382	0.7140062	505	294	482	294	463	419
SNORA50A	1.125	0.169925	0.8971695	4	0	4	0	1	8
SNORA50A	1.125	0.169925	0.8971695	4	0	4	0	1	8
SNORA50B	0	#NUM!	0.6436235	2	0	0	0	0	0
SNORA50B	0	#NUM!	0.6436235	2	0	0	0	0	0
RRP7A	0.877632	-0.188312	0.3658654	3682	4457	4969	3892	4227	3385
C3orf18	1.0666667	0.0931094	0.8656827	19	11	15	6	14	28
MCAM	0.922655	-0.116137	0.6824158	562	553	708	475	610	597
AC083875.1	#DIV/0!	#DIV/0!	0.8030358	0	0	0	0	0	1
C3orf14	0.7878289	-0.344046	0.1018577	373	374	469	319	338	301
MCAT	0.905	-0.14401	0.6813949	397	495	508	464	461	342
NF559-ZNF17	0	#NUM!	0.109327	2	7	0	0	0	0
MOCS2	1.0895522	0.1237354	0.1701593	1159	976	1215	1132	1341	1177
MOCS3	0.8581508	-0.220697	0.3000589	795	905	993	796	820	695
FGD5-AS1	0.8332897	-0.26311	0.0920191	2492	2306	2838	1616	2423	2324
MCEE	1.0982067	0.1351496	0.222242	376	367	428	406	449	431
AL024498.1	1.4444444	0.5305147	0.6649577	2	5	2	5	6	2
RRP12	0.9043437	-0.145057	0.562108	1153	1501	1559	1236	1282	1292
AL024508.2	1	0	0.9256879	10	5	10	8	2	15
RRP15	1.0343811	0.0487679	0.4781474	1106	730	1218	934	1149	1076

ZIK1	3	1.5849625	0.6206259	1	0	0	3	0	0
CT45A10	0.3541667	-1.4975	0.0013262	81	56	55	19	15	34
MOCS1	1.0404294	0.0571791	0.3533043	1315	1450	1613	1383	1606	1566
TRBV12-5	0	#NUM!	0.5378194	0	2	1	0	0	0
MINOS1	1.3960396	0.4813399	0.2192081	24	18	59	35	46	60
RRP36	0.8870519	-0.17291	0.403872	2024	2221	2590	2038	2069	1956
PAIP2	1.1425334	0.1922364	0.0357943	1399	1304	1710	1447	1840	1755
ZIM2	0.2	-2.321928	0.03339	10	15	10	0	5	2
PAIP1	1.0224487	0.0320285	0.5030746	2651	2172	2928	2183	3080	2662
AC010359.1	1	0	0.9398567	1	3	3	4	1	2
MOCOS	0.658363	-0.603045	0.0962581	84	110	87	69	53	63
RRP1B	0.965991	-0.049918	0.9840531	1794	1905	2270	1563	2090	2113
AP002990.1	0.5892857	-0.762961	0.1790855	39	19	54	18	29	19
HERC2P10	0.3333333	-1.584963	0.1694452	6	12	6	2	5	1
AL161719.1	7.5	2.9068906	0.0833671	0	0	2	7	5	3
RNASEH2CP1	0	#NUM!	0.4460243	4	0	0	0	0	0
RBMS1P1	0.7165354	-0.48089	0.3281258	40	37	50	26	28	37
MT1DP	#DIV/0!	#DIV/0!	0.8030358	0	0	0	0	1	0
AC007161.1	0	#NUM!	0.5781912	0	0	3	0	0	0
DIO1	3	1.5849625	0.3538722	0	0	3	5	2	2
DIO2	0.522293	-0.937069	0.0506358	54	46	57	11	45	26
FAM91A1	0.8936306	-0.16225	0.5022217	1279	805	1056	643	1066	1097
AC092803.2	0.7189189	-0.476099	0.2473187	68	52	65	43	53	37
TBCAP1	1.4444444	0.5305147	0.711607	1	7	1	2	3	8
AC067969.1	0	#NUM!	0.355562	5	0	0	0	0	0
AC092803.1	0.96	-0.058894	0.9278526	12	11	2	4	9	11
OR7E62P	1.1304348	0.1768778	0.6059708	46	54	38	40	66	50
GLUD1P2	1.4516129	0.5376568	0.3899028	6	10	15	17	18	10
MCL1	0.924729	-0.112897	0.5814249	11045	10581	12690	8514	11360	11859
AC108097.1	1.3548387	0.4381211	0.4802397	10	4	17	12	12	18
GLUD1P3	1.0347222	0.0492435	0.7942606	45	54	45	47	41	61
GLUD1P8	3.6666667	1.8744691	0.2831071	1	0	2	4	0	7
OSCP1	0.7235772	-0.466781	0.3240179	41	39	43	22	37	30
MCM7	0.7776393	-0.362827	0.0128226	8262	9222	10601	7278	7619	6943
COL14A1	0	#NUM!	0.2762615	2	0	4	0	0	0
MCM8	1.0177448	0.0253759	0.6230819	1614	1059	1779	1154	1695	1682
MCM9	0.6604651	-0.598446	0.0097664	410	261	404	174	264	272
AC026355.1	0.8235294	-0.280108	0.8836678	8	0	9	4	4	6
AC026355.3	0	#NUM!	0.824326	0	0	1	0	0	0
CEP170B	1.0268652	0.0382468	0.4219959	1796	1969	2414	1812	2312	2221
AC025766.1	0.8125	-0.29956	0.9359567	6	0	10	7	0	6
GGPS1	1.1016407	0.1396537	0.1908086	832	641	1026	780	1090	883
DIS3	0.996614	-0.004893	0.8381328	1819	904	1707	1069	1860	1486
MCM3	0.8181852	-0.289501	0.0374527	7759	8654	10147	6721	7829	7181
MCM4	0.7391861	-0.435991	0.0012581	6172	6703	7446	4068	5293	5660
MCM5	0.804111	-0.314533	0.0510241	7210	8895	9874	6909	7422	6559
MCM6	1.030036	0.0426948	0.4091422	4115	3521	4849	3510	4747	4603
MCM2	0.7951482	-0.330704	0.0250758	5998	7287	7120	5042	5864	5319
RPL36AP33	0.5	-1	0.81753	2	0	0	0	0	1
IL21R	2.1	1.0703893	0.4154181	9	1	0	5	10	6
RPL36AP43	0.6	-0.736966	0.8431819	0	0	5	1	0	2
RPL36AP40	#DIV/0!	#DIV/0!	0.8030358	0	0	0	0	1	0
ITGA9-AS1	1.3870968	0.4720684	0.4515916	11	9	11	14	16	13
RPL36AP45	0	#NUM!	0.824326	1	0	0	0	0	0

MAB21L3	0.7517241	-0.411725	0.2531977	118	67	105	57	77	84
LIFR-AS1	0.7311828	-0.451696	0.4173544	33	24	36	15	27	26
MAB21L2	0	#NUM!	0.6436235	2	0	0	0	0	0
IL1RN	#DIV/0!	#DIV/0!	0.0358725	0	0	0	1	10	2
IL23R	2.25	1.169925	0.4909437	0	3	1	3	1	5
CALR4P	1.0769231	0.1069152	0.896511	2	3	8	2	10	2
SCAANT1	0	#NUM!	0.6631647	1	0	1	0	0	0
IL1R1	1.2671139	0.3415462	0.0017652	1934	1654	2226	1972	2702	2693
AC078923.1	0.3636364	-1.459432	0.4123684	3	7	1	0	0	4
IL1R2	0.8277778	-0.272685	0.5614701	51	55	74	44	47	58
AF201337.1	0.6666667	-0.584963	0.6642594	3	7	11	6	2	6
IL23A	1.7692308	0.8231222	0.1384438	11	8	20	32	18	19
AC082651.1	0	#NUM!	0.5781912	0	0	3	0	0	0
SH2D2A	0.4485294	-1.156726	0.0228662	33	38	65	16	28	17
AC082651.3	0.2857143	-1.807355	0.4466716	7	0	0	0	1	1
UBA6-AS1	0.9882075	-0.017114	0.848437	169	101	154	155	150	114
OTUD4P1	#DIV/0!	#DIV/0!	0.6554062	0	0	0	0	2	0
AZIN1-AS1	0.7692308	-0.378512	0.8981799	3	2	8	7	0	3
OR7E59P	0	#NUM!	0.6385096	0	2	0	0	0	0
AC011676.1	0.5625	-0.830075	0.5085798	7	7	2	3	5	1
AC011676.3	0	#NUM!	0.5075476	0	0	4	0	0	0
AC011676.5	1	0	0.9890559	1	0	0	0	0	1
SH2D1B	1.4285714	0.5145732	0.7101615	3	0	4	2	6	2
AL451048.1	1.5882353	0.6674247	0.4885996	8	0	9	5	6	16
AC098829.1	2	1	0.834705	0	0	1	0	2	0
MCUB	0.9180193	-0.123404	0.6249788	1112	1108	1415	1032	1197	1108
MRPS31P4	1.7	0.7655347	0.3728851	8	3	9	4	9	21
SIPA1L2	0.8263689	-0.275142	0.1835149	466	399	523	286	457	404
MRPS31P5	0.5540541	-0.851901	0.2647634	31	8	35	3	17	21
SIPA1L3	0.8498448	-0.234729	0.2114641	1138	1303	1102	765	1115	1131
SIPA1L1	1.1124077	0.1536856	0.1190159	4380	4318	4842	3873	5415	5774
COX6CP14	0	#NUM!	0.824326	0	0	1	0	0	0
COX6CP10	0	#NUM!	0.4374838	0	4	0	0	0	0
AC130651.2	1	0	0.9806644	2	2	0	1	3	0
MT1P1	1.375	0.4594316	0.7177504	3	4	1	7	0	4
C1QTNF12	2.3333333	1.2223924	0.1497667	0	6	12	21	11	10
AC242426.3	0.55	-0.862496	0.1013645	40	25	55	15	29	22
AMER1	0.7358949	-0.442428	0.0262968	1233	1578	1301	697	1049	1280
AL359636.1	0	#NUM!	0.824326	0	1	0	0	0	0
AL359636.2	0.1666667	-2.584963	0.3797882	2	0	4	1	0	0
RPS3AP5	1.2428571	0.3136605	0.4454626	14	23	33	29	33	25
RPS3AP6	1.1832983	0.2428138	0.0218037	1780	1830	2102	2340	2387	2032
RSF1-IT1	3	1.5849625	0.2741442	0	4	1	5	6	4
RPS3AP2	1.3333333	0.4150375	0.8492451	0	3	0	3	0	1
RPS3AP3	0	#NUM!	0.824326	0	0	1	0	0	0
AC027338.1	2.6666667	1.4150375	0.4958369	0	0	3	0	5	3
QPCT	0.2	-2.321928	0.1465306	3	9	3	0	1	2
SIPA1	0.7691883	-0.378591	0.0744738	510	683	618	484	462	447
AL390039.1	1.1481481	0.1993088	0.7867138	14	5	8	8	16	7
AC108681.1	0.2857143	-1.807355	0.3813473	2	4	1	0	1	1
C3orf67	0.8	-0.321928	0.4972669	81	45	54	53	44	47
C3orf62	0.8997555	-0.152395	0.6972247	138	116	155	110	154	104
AC095050.1	0	#NUM!	0.6436235	2	0	0	0	0	0
C3orf70	0	#NUM!	0.4185478	3	0	1	0	0	0

SPECC1L	1.0663317	0.0926562	0.2879157	643	596	751	666	800	656
TIMELESS	0.6843993	-0.54709	2.36E-05	3101	3320	3617	1892	2621	2357
CAMSAP1	1.1116208	0.1526647	0.2046708	1301	1340	1283	1105	1671	1586
CAMSAP2	1.1315985	0.1783622	0.2252594	5862	3561	5372	3768	6241	6733
C3orf80	5	2.3219281	0.4606115	1	0	0	0	0	5
NPC1L1	0.3333333	-1.584963	0.6378737	1	1	1	0	1	0
AMELY	0.7391304	-0.436099	0.6311407	22	5	19	12	12	10
RWDD4P1	1.3333333	0.4150375	0.3263264	17	17	32	30	31	27
PNPT1	1.3840777	0.4689249	6.61E-05	1804	1393	1953	1991	2716	2421
RWDD4P2	1.4151625	0.5009677	0.0417064	104	72	101	105	133	154
CAMSAP3	0.9176471	-0.123989	0.7886656	222	223	320	229	269	204
AC024075.1	0.4528302	-1.142958	0.1246684	19	14	20	5	11	8
AC024075.2	0.8172043	-0.291231	0.5446974	51	73	62	60	56	36
AC024075.3	0.4166667	-1.263034	0.2223272	9	8	19	0	8	7
DNAJC3-AS1	1.1601563	0.2143191	0.4139795	117	57	82	85	101	111
AC104986.2	1.4210526	0.50696	0.5200225	4	7	8	10	4	13
AC104986.1	2	1	0.8186965	0	0	1	0	0	2
C3orf20	4	2	0.1850932	0	0	4	3	3	10
AL117329.1	0.7826087	-0.353637	0.649288	23	10	13	5	12	19
RNU6-658P	0.75	-0.415037	0.8157078	5	0	7	0	5	4
C3orf38	0.8886054	-0.170385	0.3797585	775	737	840	564	791	735
C3orf33	0.7047619	-0.504792	0.1531074	68	117	130	62	78	82
C3orf30	0	#NUM!	0.824326	0	0	1	0	0	0
C1QTNF9B	0.3333333	-1.584963	0.6659102	3	0	0	1	0	0
C3orf35	1.0909091	0.1255309	0.8412529	2	3	6	8	4	0
MIR1289-1	0.3333333	-1.584963	0.5282528	2	0	4	0	1	1
C3orf49	0.3333333	-1.584963	0.2090617	20	2	8	2	0	8
AC010598.1	2.3333333	1.2223924	0.4920492	1	0	2	3	3	1
AC034205.2	#DIV/0!	#DIV/0!	0.6554062	0	0	0	0	2	0
SNORA18	#DIV/0!	#DIV/0!	0.8030358	0	0	0	0	1	0
SNORA18	#DIV/0!	#DIV/0!	0.8030358	0	0	0	0	1	0
SNORA18	#DIV/0!	#DIV/0!	0.8030358	0	0	0	0	1	0
SNORA18	#DIV/0!	#DIV/0!	0.8030358	0	0	0	0	1	0
SNORA18	#DIV/0!	#DIV/0!	0.8030358	0	0	0	0	1	0
SNORA18	#DIV/0!	#DIV/0!	0.8030358	0	0	0	0	1	0
SNORA18	#DIV/0!	#DIV/0!	0.8030358	0	0	0	0	1	0
SNORA18	#DIV/0!	#DIV/0!	0.8030358	0	0	0	0	1	0
SNORA12	0.3157895	-1.662965	0.2238307	6	5	8	0	5	1
SNORA12	0.3157895	-1.662965	0.2238307	6	5	8	0	5	1
SNORA12	0.3157895	-1.662965	0.2238307	6	5	8	0	5	1
SNORA13	0.7777778	-0.36257	0.8552437	5	1	12	5	2	7
AC010608.2	0	#NUM!	0.5622741	0	1	2	0	0	0
C3orf52	1.005196	0.0074769	0.6832525	630	726	761	618	772	738
C3orf58	0.7767549	-0.364469	0.0484475	949	718	940	519	729	777
AC034195.1	#DIV/0!	#DIV/0!	0.8030358	0	0	0	0	1	0
MT-ND6	0.7165253	-0.48091	2.71E-05	25726	26244	29868	17739	20976	19924
RALGAPA1P1	0.8099174	-0.304153	0.5929038	39	43	39	38	24	36
MDC1	0.7586249	-0.398541	0.0044874	2456	2725	3022	1740	2190	2293
SNORA40	0.5	-1	0.6960303	2	0	6	3	1	0
SNORA40	0.5	-1	0.6960303	2	0	6	3	1	0
SNORA40	0.5	-1	0.6960303	2	0	6	3	1	0
SNORA40	0.5	-1	0.6960303	2	0	6	3	1	0
SNORA40	0.5	-1	0.6960303	2	0	6	3	1	0
SNORA40	0.5	-1	0.6960303	2	0	6	3	1	0

SNORA40	0.5	-1	0.6960303	2	0	6	3	1	0
SNORA40	0.5	-1	0.6960303	2	0	6	3	1	0
SNORA40	0.5	-1	0.6960303	2	0	6	3	1	0
SNORA40	0.5	-1	0.6960303	2	0	6	3	1	0
SNORA40	0.5	-1	0.6960303	2	0	6	3	1	0
SNORA40	0.5	-1	0.6960303	2	0	6	3	1	0
SNORA40	0.5	-1	0.6960303	2	0	6	3	1	0
SNORA40	0.5	-1	0.6960303	2	0	6	3	1	0
SNORA40	0.5	-1	0.6960303	2	0	6	3	1	0
SNORA40	0.5	-1	0.6960303	2	0	6	3	1	0
SNORA40	0.5	-1	0.6960303	2	0	6	3	1	0
SNORA40	0.5	-1	0.6960303	2	0	6	3	1	0
SNORA40	0.5	-1	0.6960303	2	0	6	3	1	0
SNORA40	0.5	-1	0.6960303	2	0	6	3	1	0
MT-ND4	0.8384012	-0.254287	0.0502512	185579	181067	208185	132971	180772	168196
AC009127.2	1.6666667	0.7369656	0.6156163	0	4	2	5	4	1
MT-ND5	0.6863053	-0.543078	0.0005103	98176	102397	106034	48655	77524	84247
AC009127.3	#DIV/0!	#DIV/0!	0.8030358	0	0	0	0	0	1
AL157935.1	1.8275862	0.8699395	0.0670223	46	12	29	37	76	46
MT-ND2	0.8201472	-0.286045	0.030682	68246	72471	79869	48881	69178	62854
SNORA3B	5	2.3219281	0.1591466	1	0	2	14	1	0
MT-ND3	1.0193103	0.0275933	0.410659	11924	11724	14570	12493	14234	12229
MT-ND1	0.9723494	-0.040453	0.9204896	40308	45452	48993	39028	49760	42239
AC008632.1	#DIV/0!	#DIV/0!	0.2533058	0	0	0	0	7	0
IL4I1	2.4285714	1.2801079	0.3331234	2	3	2	1	8	8
AC023090.2	#DIV/0!	#DIV/0!	0.242062	0	0	0	6	0	0
RN7SL587P	0	#NUM!	0.824326	1	0	0	0	0	0
SNORA36	0	#NUM!	0.2324194	3	3	0	0	0	0
SNORA36	0	#NUM!	0.2324194	3	3	0	0	0	0
SNORA37	0	#NUM!	0.824326	0	0	1	0	0	0
RNU6-678P	0.4	-1.321928	0.5773956	3	1	1	1	1	0
SNORA32	0	#NUM!	0.6385096	0	2	0	0	0	0
SNORA32	0	#NUM!	0.6385096	0	2	0	0	0	0
SNORA32	0	#NUM!	0.6385096	0	2	0	0	0	0
SNORA32	0	#NUM!	0.6385096	0	2	0	0	0	0
SNORA33	1.2043011	0.2681961	0.4722065	28	25	40	25	38	49
SNORA33	1.2043011	0.2681961	0.4722065	28	25	40	25	38	49
SNORA33	1.2043011	0.2681961	0.4722065	28	25	40	25	38	49
SNORA33	1.2043011	0.2681961	0.4722065	28	25	40	25	38	49
SNORA30	#DIV/0!	#DIV/0!	0.5070017	0	0	0	3	0	0
SNORA30	#DIV/0!	#DIV/0!	0.5070017	0	0	0	3	0	0
SNORA31	2	1	0.7311087	1	1	0	0	0	4
SNORA31	2	1	0.7311087	1	1	0	0	0	4
SNORA31	2	1	0.7311087	1	1	0	0	0	4
SNORA31	2	1	0.7311087	1	1	0	0	0	4
SNORA31	2	1	0.7311087	1	1	0	0	0	4
SNORA31	2	1	0.7311087	1	1	0	0	0	4
SNORA31	2	1	0.7311087	1	1	0	0	0	4
SNORA31	2	1	0.7311087	1	1	0	0	0	4
SNORA31	2	1	0.7311087	1	1	0	0	0	4
SNORA31	2	1	0.7311087	1	1	0	0	0	4
SNORA31	2	1	0.7311087	1	1	0	0	0	4
SNORA31	2	1	0.7311087	1	1	0	0	0	4
SNORA31	2	1	0.7311087	1	1	0	0	0	4

SNORA31	2	1	0.7311087	1	1	0	0	0	4
SNORA31	2	1	0.7311087	1	1	0	0	0	4
SNORA31	2	1	0.7311087	1	1	0	0	0	4
SNORA31	2	1	0.7311087	1	1	0	0	0	4
SNORA31	2	1	0.7311087	1	1	0	0	0	4
SNORA31	2	1	0.7311087	1	1	0	0	0	4
SNORA31	2	1	0.7311087	1	1	0	0	0	4
SNORA31	2	1	0.7311087	1	1	0	0	0	4
SNORA31	2	1	0.7311087	1	1	0	0	0	4
SNORA31	2	1	0.7311087	1	1	0	0	0	4
SNORA31	2	1	0.7311087	1	1	0	0	0	4
SNORA31	2	1	0.7311087	1	1	0	0	0	4
SNORA31	2	1	0.7311087	1	1	0	0	0	4
SNORA31	2	1	0.7311087	1	1	0	0	0	4
SNORA2A	0.4	-1.321928	0.4331599	7	2	1	0	3	1
SNORA2B	0.3333333	-1.584963	0.7290749	0	0	3	0	0	1
AR	0.9878049	-0.017702	0.8672282	118	86	124	98	100	126
SNORA27	0	#NUM!	0.824326	0	0	1	0	0	0
SNORA27	0	#NUM!	0.824326	0	0	1	0	0	0
SNORA27	0	#NUM!	0.824326	0	0	1	0	0	0
SNORA27	0	#NUM!	0.824326	0	0	1	0	0	0
SNORA27	0	#NUM!	0.824326	0	0	1	0	0	0
SNORA28	#DIV/0!	#DIV/0!	0.3196487	0	0	0	0	1	4
SNORA25	#DIV/0!	#DIV/0!	0.8030358	0	0	0	0	1	0
SNORA25	#DIV/0!	#DIV/0!	0.8030358	0	0	0	0	1	0
SNORA25	#DIV/0!	#DIV/0!	0.8030358	0	0	0	0	1	0
SNORA25	#DIV/0!	#DIV/0!	0.8030358	0	0	0	0	1	0
SNORA25	#DIV/0!	#DIV/0!	0.8030358	0	0	0	0	1	0
SNORA25	#DIV/0!	#DIV/0!	0.8030358	0	0	0	0	1	0
SNORA25	#DIV/0!	#DIV/0!	0.8030358	0	0	0	0	1	0
SNORA25	#DIV/0!	#DIV/0!	0.8030358	0	0	0	0	1	0
SNORA25	#DIV/0!	#DIV/0!	0.8030358	0	0	0	0	1	0
SNORA25	#DIV/0!	#DIV/0!	0.8030358	0	0	0	0	1	0
SNORA25	#DIV/0!	#DIV/0!	0.8030358	0	0	0	0	1	0
SNORA25	#DIV/0!	#DIV/0!	0.8030358	0	0	0	0	1	0
SNORA25	#DIV/0!	#DIV/0!	0.8030358	0	0	0	0	1	0
SNORA25	#DIV/0!	#DIV/0!	0.8030358	0	0	0	0	1	0
SNORA25	#DIV/0!	#DIV/0!	0.8030358	0	0	0	0	1	0
SNORA25	#DIV/0!	#DIV/0!	0.8030358	0	0	0	0	1	0
SNORA25	#DIV/0!	#DIV/0!	0.8030358	0	0	0	0	1	0
SNORA25	#DIV/0!	#DIV/0!	0.8030358	0	0	0	0	1	0
SNORA25	#DIV/0!	#DIV/0!	0.8030358	0	0	0	0	1	0
RN7SL586P	#DIV/0!	#DIV/0!	0.6411933	0	0	0	0	0	2
SNORA26	1.3	0.3785116	0.7565836	1	0	9	0	5	8
SNORA26	1.3	0.3785116	0.7565836	1	0	9	0	5	8
SNORA26	1.3	0.3785116	0.7565836	1	0	9	0	5	8
SNORA26	1.3	0.3785116	0.7565836	1	0	9	0	5	8
SNORA26	1.3	0.3785116	0.7565836	1	0	9	0	5	8
SNORA26	1.3	0.3785116	0.7565836	1	0	9	0	5	8
SNORA26	1.3	0.3785116	0.7565836	1	0	9	0	5	8
SNORA26	1.3	0.3785116	0.7565836	1	0	9	0	5	8
SNORA26	1.3	0.3785116	0.7565836	1	0	9	0	5	8
SNORA26	1.3	0.3785116	0.7565836	1	0	9	0	5	8
SNORA26	1.3	0.3785116	0.7565836	1	0	9	0	5	8

SNORA23	#DIV/0!	#DIV/0!	0.8030358	0	0	0	1	0	0
SNORA22	1.4615385	0.5474878	0.5701517	5	0	8	8	7	4
SNORA22	1.4615385	0.5474878	0.5701517	5	0	8	8	7	4
CGGBP1	0.9196728	-0.120807	0.6073655	1801	1358	1609	1283	1586	1516
GRID2IP	0.8043478	-0.314109	0.5510937	63	32	43	40	41	30
SNORA20	1	0	0.9890559	1	0	0	0	0	1
SNORA20	1	0	0.9890559	1	0	0	0	0	1
SNORA20	1	0	0.9890559	1	0	0	0	0	1
SNORA20	1	0	0.9890559	1	0	0	0	0	1
SNORA60	0.7380952	-0.438121	0.5986551	16	10	16	9	10	12
AL138789.1	1.5714286	0.6520767	0.539546	1	4	9	1	8	13
C2	0.64	-0.643856	0.6474164	1	9	15	1	1	14
SNORA5C	1.5185185	0.6026645	0.3576841	9	6	12	17	8	16
C3	0.7243213	-0.465298	0.0085917	1023	1379	1171	710	931	947
DYNC2LI1	1.1612903	0.2157287	0.2359545	172	112	212	172	228	176
SNORA5A	2	1	0.7861378	1	0	0	1	1	0
C5	1.6533333	0.7253776	0.0764845	24	20	31	48	34	42
C9	1.2	0.2630344	0.8801854	0	3	2	2	4	0
SNORA58	6.5	2.7004397	0.1613502	1	0	1	0	5	8
SNORA58	6.5	2.7004397	0.1613502	1	0	1	0	5	8
SNORA57	0	#NUM!	0.824326	0	0	1	0	0	0
SNORA57	0	#NUM!	0.824326	0	0	1	0	0	0
SNORA57	0	#NUM!	0.824326	0	0	1	0	0	0
SNORA57	0	#NUM!	0.824326	0	0	1	0	0	0
SNORA54	0	#NUM!	0.6840298	0	0	2	0	0	0
SNORA55	1.2142857	0.2801079	0.6475389	12	5	11	13	11	10
SNORA53	0.1	-3.321928	0.2068076	1	0	9	1	0	0
SNORA51	0.3333333	-1.584963	0.4229593	2	4	3	0	0	3
SNORA51	0.3333333	-1.584963	0.4229593	2	4	3	0	0	3
SNORA51	0.3333333	-1.584963	0.4229593	2	4	3	0	0	3
SNORA51	0.3333333	-1.584963	0.4229593	2	4	3	0	0	3
SNORA51	0.3333333	-1.584963	0.4229593	2	4	3	0	0	3
SNORA51	0.3333333	-1.584963	0.4229593	2	4	3	0	0	3
SNORA51	0.3333333	-1.584963	0.4229593	2	4	3	0	0	3
SNORA51	0.3333333	-1.584963	0.4229593	2	4	3	0	0	3
SNORA51	0.3333333	-1.584963	0.4229593	2	4	3	0	0	3
SNORA51	0.3333333	-1.584963	0.4229593	2	4	3	0	0	3
SNORA51	0.3333333	-1.584963	0.4229593	2	4	3	0	0	3
SNORA51	0.3333333	-1.584963	0.4229593	2	4	3	0	0	3
SNORA51	0.3333333	-1.584963	0.4229593	2	4	3	0	0	3
PES1P2	1	0	0.9890559	1	0	0	0	0	1
SEMA6A-AS1	0.75	-0.415037	0.4446838	64	26	50	16	33	56
SEMA6A-AS2	1.0833333	0.1154772	0.8975075	4	7	1	7	2	4
CP	0	#NUM!	0.5075476	0	0	4	0	0	0
CS	0.7984881	-0.324657	0.0182797	4081	4601	4811	3253	3919	3602
SNORA47	0	#NUM!	0.5075476	0	0	4	0	0	0
AC243591.1	#DIV/0!	#DIV/0!	0.8030358	0	0	0	0	1	0
SNORA46	1.2888889	0.3661279	0.6099556	9	20	16	12	40	6
SNORA46	1.2888889	0.3661279	0.6099556	9	20	16	12	40	6
SNORA43	#DIV/0!	#DIV/0!	0.8030358	0	0	0	0	0	1
SNORA43	#DIV/0!	#DIV/0!	0.8030358	0	0	0	0	0	1
SNORA43	#DIV/0!	#DIV/0!	0.8030358	0	0	0	0	0	1
SNORA43	#DIV/0!	#DIV/0!	0.8030358	0	0	0	0	0	1
SNORA43	#DIV/0!	#DIV/0!	0.8030358	0	0	0	0	0	1

LRSAM1	1.2780424	0.3539357	0.0063016	602	765	942	984	1003	964
SNORA81	1.3125	0.3923174	0.7579145	4	10	2	1	8	12
SNORA81	1.3125	0.3923174	0.7579145	4	10	2	1	8	12
SNORA81	1.3125	0.3923174	0.7579145	4	10	2	1	8	12
SNORA81	1.3125	0.3923174	0.7579145	4	10	2	1	8	12
QPRT	0.8996579	-0.152552	0.5193357	4824	6198	6807	5365	5725	4950
ECSIT	0.9332182	-0.099714	0.8368119	462	650	625	568	559	494
SNORA7B	0	#NUM!	0.4460243	4	0	0	0	0	0
AC002519.1	0.5	-1	0.6906809	3	1	0	0	2	0
AC103760.1	7	2.8073549	0.1468707	0	1	1	0	10	4
SNORA77	0.6666667	-0.584963	0.4308564	25	16	19	15	7	18
SNORA77	0.6666667	-0.584963	0.4308564	25	16	19	15	7	18
SNORA77	0.6666667	-0.584963	0.4308564	25	16	19	15	7	18
RNU6-674P	0	#NUM!	0.824326	0	1	0	0	0	0
SNORA75	0.5757576	-0.796467	0.4233281	10	10	13	2	3	14
SNORA75	0.5757576	-0.796467	0.4233281	10	10	13	2	3	14
SNORA75	0.5757576	-0.796467	0.4233281	10	10	13	2	3	14
SNORA75	0.5757576	-0.796467	0.4233281	10	10	13	2	3	14
SNORA75	0.5757576	-0.796467	0.4233281	10	10	13	2	3	14
SNORA75	0.5757576	-0.796467	0.4233281	10	10	13	2	3	14
SNORA75	0.5757576	-0.796467	0.4233281	10	10	13	2	3	14
SNORA72	0.125	-3	0.2704008	8	0	0	1	0	0
SNORA72	0.125	-3	0.2704008	8	0	0	1	0	0
SNORA72	0.125	-3	0.2704008	8	0	0	1	0	0
SNORA72	0.125	-3	0.2704008	8	0	0	1	0	0
SNORA72	0.125	-3	0.2704008	8	0	0	1	0	0
SNORA72	0.125	-3	0.2704008	8	0	0	1	0	0
SNORA72	0.125	-3	0.2704008	8	0	0	1	0	0
SNORA72	0.125	-3	0.2704008	8	0	0	1	0	0
SNORA73	#DIV/0!	#DIV/0!	0.8030358	0	0	0	0	1	0
SNORA73	#DIV/0!	#DIV/0!	0.8030358	0	0	0	0	1	0
SNORA73	#DIV/0!	#DIV/0!	0.8030358	0	0	0	0	1	0
SNORA73	#DIV/0!	#DIV/0!	0.8030358	0	0	0	0	1	0
SNORA70	0.4444444	-1.169925	0.5690977	1	3	5	0	0	4
SNORA70	0.4444444	-1.169925	0.5690977	1	3	5	0	0	4
SNORA70	0.4444444	-1.169925	0.5690977	1	3	5	0	0	4
SNORA70	0.4444444	-1.169925	0.5690977	1	3	5	0	0	4
SNORA70	0.4444444	-1.169925	0.5690977	1	3	5	0	0	4
SNORA70	0.4444444	-1.169925	0.5690977	1	3	5	0	0	4
SNORA70	0.4444444	-1.169925	0.5690977	1	3	5	0	0	4
SNORA70	0.4444444	-1.169925	0.5690977	1	3	5	0	0	4
SNORA70	0.4444444	-1.169925	0.5690977	1	3	5	0	0	4
SNORA70	0.4444444	-1.169925	0.5690977	1	3	5	0	0	4
SNORA70	0.4444444	-1.169925	0.5690977	1	3	5	0	0	4
SNORA70	0.4444444	-1.169925	0.5690977	1	3	5	0	0	4
SNORA70	0.4444444	-1.169925	0.5690977	1	3	5	0	0	4
SNORA70	0.4444444	-1.169925	0.5690977	1	3	5	0	0	4
SNORA70	0.4444444	-1.169925	0.5690977	1	3	5	0	0	4
SNORA70	0.4444444	-1.169925	0.5690977	1	3	5	0	0	4
SNORA70	0.4444444	-1.169925	0.5690977	1	3	5	0	0	4
SNORA70	0.4444444	-1.169925	0.5690977	1	3	5	0	0	4
SNORA70	0.4444444	-1.169925	0.5690977	1	3	5	0	0	4
SNORA70	0.4444444	-1.169925	0.5690977	1	3	5	0	0	4
SNORA70	0.4444444	-1.169925	0.5690977	1	3	5	0	0	4

SNORA70	0.4444444	-1.169925	0.5690977	1	3	5	0	0	4
SNORA70	0.4444444	-1.169925	0.5690977	1	3	5	0	0	4
SNORA70	0.4444444	-1.169925	0.5690977	1	3	5	0	0	4
SNORA70	0.4444444	-1.169925	0.5690977	1	3	5	0	0	4
SNORA70	0.4444444	-1.169925	0.5690977	1	3	5	0	0	4
SNORA70	0.4444444	-1.169925	0.5690977	1	3	5	0	0	4
ACTL6B	0	#NUM!	0.824326	0	0	1	0	0	0
ACTL6A	0.8252656	-0.27707	0.0600623	1910	1860	2159	1320	1847	1726
SIRT4	1.1586538	0.2124496	0.4097835	55	83	70	86	86	69
SIRT5	0.7535771	-0.408173	0.0978784	212	215	202	129	165	180
SIRT6	1.0563978	0.0791532	0.4115219	445	569	635	632	625	485
SIRT7	0.9564823	-0.06419	0.9461186	937	1071	1301	1147	1071	947
F3	1.2174595	0.2838738	0.0042827	1208	1173	1445	1353	1722	1583
SIRT1	1.1891295	0.2499058	0.1224476	853	586	824	603	974	1114
SIRT2	0.9083918	-0.138613	0.5852781	873	1027	1091	889	1025	803
F5	0.5762712	-0.79518	0.1593489	29	31	58	26	17	25
SIRT3	0.8968144	-0.157119	0.517216	445	464	535	392	506	397
F8	1.1304348	0.1768778	0.738006	5	4	14	11	9	6
TGIF2LY	0	#NUM!	0.824326	1	0	0	0	0	0
FLJ22447	0.9310345	-0.103093	0.976646	27	24	36	33	22	26
SNORA67	0	#NUM!	0.4460243	4	0	0	0	0	0
SNORA67	0	#NUM!	0.4460243	4	0	0	0	0	0
SNORA67	0	#NUM!	0.4460243	4	0	0	0	0	0
SNORA67	0	#NUM!	0.4460243	4	0	0	0	0	0
SNORA67	0	#NUM!	0.4460243	4	0	0	0	0	0
SNORA67	0	#NUM!	0.4460243	4	0	0	0	0	0
SNORA67	0	#NUM!	0.4460243	4	0	0	0	0	0
SNORA67	0	#NUM!	0.4460243	4	0	0	0	0	0
SNORA65	0.6031746	-0.729352	0.341004	14	18	31	16	8	14
SNORA66	1.0625	0.0874628	0.7661392	10	14	24	20	14	17
MDFI	0.704918	-0.504473	0.488876	17	16	28	12	12	19
SNORA63	0	#NUM!	0.3963337	1	3	0	0	0	0
SNORA63	0	#NUM!	0.3963337	1	3	0	0	0	0
SNORA63	0	#NUM!	0.3963337	1	3	0	0	0	0
SNORA63	0	#NUM!	0.3963337	1	3	0	0	0	0
SNORA63	0	#NUM!	0.3963337	1	3	0	0	0	0
SNORA63	0	#NUM!	0.3963337	1	3	0	0	0	0
SNORA63	0	#NUM!	0.3963337	1	3	0	0	0	0
SNORA63	0	#NUM!	0.3963337	1	3	0	0	0	0
SNORA63	0	#NUM!	0.3963337	1	3	0	0	0	0
SNORA64	0	#NUM!	0.0769374	4	1	5	0	0	0
SNORA64	0	#NUM!	0.0769374	4	1	5	0	0	0
SNORA64	0	#NUM!	0.0769374	4	1	5	0	0	0
SNORA64	0	#NUM!	0.0769374	4	1	5	0	0	0
SNORA64	0	#NUM!	0.0769374	4	1	5	0	0	0
FH	0.9377019	-0.092799	0.7949122	3784	3586	4396	3604	4050	3379
AC020604.1	1	0	0.9890559	1	0	0	0	1	0
HLA-DMA	0.9810298	-0.027631	0.8946701	117	120	132	125	129	108
SNORA9B	0.2857143	-1.807355	0.1849329	6	5	10	1	5	0
HLA-DMB	0.1764706	-2.5025	0.1220953	7	1	9	1	1	1
GC	0.75	-0.415037	0.8724887	4	0	0	0	0	3
GK	1.0327869	0.0465426	0.7452578	87	66	91	65	93	94
MDH1	1.107459	0.1472532	0.0516601	7052	6970	8461	7701	9196	8002
MDH2	0.8523176	-0.230537	0.2244813	7878	9026	10215	8126	8009	6979

IRNPUL2-BSC	3	1.5849625	0.4088458	0	0	3	9	0	0
AC020594.1	0	#NUM!	0.041058	1	3	9	0	0	0
GGT3P	0	#NUM!	0.824326	1	0	0	0	0	0
HR	0.4615385	-1.115477	0.3206453	5	9	12	4	7	1
HPGDS	2.6666667	1.4150375	0.4671465	2	0	1	2	0	6
GGT4P	0	#NUM!	0.276989	0	6	0	0	0	0
HLA-DOA	#DIV/0!	#DIV/0!	0.4121728	0	0	0	0	1	3
HLA-DOB	1.7142857	0.7776076	0.5630714	0	2	5	4	7	1
IK	1.0841185	0.1165225	0.1440951	5250	4306	6576	5749	6264	5476
AC122719.1	#DIV/0!	#DIV/0!	0.8030358	0	0	0	0	1	0
AC122719.2	2	1	0.8186965	0	0	1	0	0	2
VPS33A	0.8878049	-0.171685	0.4563277	621	623	806	579	643	598
VPS33B	0.7325008	-0.449098	0.0064598	839	1030	1174	683	820	726
AL132765.1	3	1.5849625	0.5179806	0	0	2	0	5	1
AL132765.2	3	1.5849625	0.6253252	0	0	1	3	0	0
PIGBOS1	0.6944444	-0.526069	0.1721485	76	103	109	79	76	45
AL512408.1	1.8666667	0.9004643	0.4645514	9	4	2	0	13	15
NSMAF	0.8593433	-0.218693	0.3041338	839	658	970	505	842	773
ECSCR	0	#NUM!	0.6385096	0	2	0	0	0	0
AC127024.6	0	#NUM!	0.6840298	0	0	2	0	0	0
AC127024.4	0.8823529	-0.180572	0.9056019	13	0	4	5	9	1
AC127024.5	2.6	1.3785116	0.1740668	2	1	7	7	12	7
AC127024.2	2.5	1.3219281	0.6749786	2	0	0	0	5	0
AC127024.3	0.8	-0.321928	0.9219351	2	0	3	0	0	4
AC127024.8	1.0526316	0.0740006	0.9426087	4	7	8	0	9	11
MDN1	0.7827663	-0.353346	0.079759	3720	3312	3726	1746	2894	3781
KL	3.25	1.7004397	0.3597164	2	0	2	0	12	1
NOP53-AS1	0.65	-0.621488	0.6073574	5	5	10	2	4	7
KY	0.6	-0.736966	0.7762998	0	5	0	2	0	1
AL162400.2	#DIV/0!	#DIV/0!	0.8030358	0	0	0	1	0	0
SNORA80C	0	#NUM!	0.6385096	0	2	0	0	0	0
ZC3HC1	0.9873354	-0.018388	0.8271851	587	674	713	593	763	593
SNORA80D	3.8	1.9259994	0.1639367	3	2	0	6	10	3
SNORA80E	0.3333333	-1.584963	0.5299124	5	0	1	0	0	2
MDM2	0.7526908	-0.409871	0.0312277	6246	4311	5981	2699	4774	4975
HLA-DRA	#DIV/0!	#DIV/0!	0.8030358	0	0	0	0	0	1
MDM4	0.6899169	-0.535505	0.0005847	5945	4265	6521	3063	4248	4232
AC092802.1	1.5	0.5849625	0.8049212	2	0	2	0	6	0
AC092802.2	0.952381	-0.070389	0.9989055	7	1	13	3	13	4
MDM1	1.0411985	0.0582451	0.5640173	374	270	424	274	405	433
SNORA80B	#DIV/0!	#DIV/0!	0.0365748	0	0	0	0	7	7
SH2D4A	1.033522	0.0475691	0.4087478	711	678	908	747	837	790
SH2D4B	2.6666667	1.4150375	0.4645867	0	3	0	5	1	2
AL354836.1	0.6508876	-0.61952	0.1340063	51	55	63	32	39	39
MAGOH2P	0	#NUM!	0.5643268	1	0	2	0	0	0
MB	0	#NUM!	0.1824345	5	2	0	0	0	0
MDP1	0.2	-2.321928	0.4406739	0	3	2	0	1	0
SH2D3A	1.0232153	0.0331098	0.4762395	977	1150	1319	1170	1187	1169
SH2D3C	0.7333333	-0.447459	0.7828679	7	3	5	7	3	1
VPS37C	0.8885191	-0.170525	0.3845623	1660	2091	2259	1603	1901	1836
VPS37D	0.8435374	-0.245476	0.654219	36	58	53	46	41	37
VPS37A	1.1196944	0.163105	0.1061193	758	733	865	823	990	825
VPS37B	0.9810671	-0.027576	0.8325192	956	1260	1270	1151	1233	1036
RNFT2	1.0984081	0.1354142	0.3236483	195	232	264	277	255	227

COL15A1	0.25	-2	0.3109026	1	7	4	3	0	0
AC026356.1	1.1891892	0.2499783	0.5749247	27	13	34	17	42	29
AC026356.2	1	0	0.9593139	7	3	3	6	4	3
RNFT1	1.0931174	0.1284484	0.4230074	301	201	239	223	300	287
AC093635.1	3.7142857	1.8930848	0.1290161	6	0	1	8	14	4
AC022021.1	0.7692308	-0.378512	0.8337833	3	7	3	7	3	0
MAGOH3P	0.6666667	-0.584963	0.8296186	0	2	1	0	1	1
PC	1.0052576	0.0075653	0.6412853	560	638	704	636	651	625
AC007064.2	1.25	0.3219281	0.8527774	0	0	4	0	3	2
AL049543.1	2	1	0.4775664	1	4	1	5	5	2
PABPC1L	1.1318315	0.1786591	0.1143844	817	589	991	880	909	924
DHFR2	0.7651007	-0.386279	0.1166012	336	234	324	169	235	280
TXNDC11	0.7965057	-0.328243	0.0435026	875	996	1048	654	849	822
TXNDC12	1.0710176	0.0989822	0.201719	1926	2017	2140	1956	2334	2225
TXNDC15	0.7785924	-0.36106	0.0344058	788	981	959	604	783	737
TXNDC16	1.2266783	0.2947569	0.124146	492	256	399	351	549	507
TXNDC17	1.1119714	0.1531197	0.1559493	505	552	622	625	647	595
AL162151.2	0.859375	-0.21864	0.748622	27	19	18	12	17	26
AP002414.4	#DIV/0!	#DIV/0!	0.6554062	0	0	0	0	2	0
AP002414.2	0.5	-1	0.7988788	1	1	0	0	0	1
TIMD4	1.1454545	0.1959202	0.6978507	6	27	22	18	26	19
MDS2	0.25	-2	0.2497879	1	8	3	0	1	2
RNF11P2	#DIV/0!	#DIV/0!	0.5509701	0	0	0	0	3	0
KIF9-AS1	0.9393939	-0.090198	0.9847632	22	16	28	25	29	8
MST1R	1.6923077	0.7589919	0.204266	5	23	24	14	38	36
MST1L	0	#NUM!	0.824326	0	0	1	0	0	0
OSGEP	0.9740555	-0.037924	0.8879532	1302	1539	1553	1415	1515	1350
AC016629.3	#DIV/0!	#DIV/0!	0.1261023	0	0	0	0	7	2
AP000354.1	#DIV/0!	#DIV/0!	0.1226305	0	0	0	5	0	3
TF	0	#NUM!	0.6840298	0	0	2	0	0	0
TG	0.5625	-0.830075	0.5383654	3	5	8	1	7	1
AC022167.2	0.4375	-1.192645	0.0299097	46	30	52	6	28	22
TH	1.0268456	0.0382193	0.7465131	48	37	64	66	53	34
AC022167.4	0.25	-2	0.5705817	1	0	3	0	1	0
U1	1.5263158	0.6100535	0.4545999	5	7	7	8	13	8
U1	1.5263158	0.6100535	0.4545999	5	7	7	8	13	8
U1	1.5263158	0.6100535	0.4545999	5	7	7	8	13	8
U1	1.5263158	0.6100535	0.4545999	5	7	7	8	13	8
U1	1.5263158	0.6100535	0.4545999	5	7	7	8	13	8
U1	1.5263158	0.6100535	0.4545999	5	7	7	8	13	8
U1	1.5263158	0.6100535	0.4545999	5	7	7	8	13	8
U1	1.5263158	0.6100535	0.4545999	5	7	7	8	13	8
U1	1.5263158	0.6100535	0.4545999	5	7	7	8	13	8
U1	1.5263158	0.6100535	0.4545999	5	7	7	8	13	8
U1	1.5263158	0.6100535	0.4545999	5	7	7	8	13	8
U1	1.5263158	0.6100535	0.4545999	5	7	7	8	13	8
U1	1.5263158	0.6100535	0.4545999	5	7	7	8	13	8
U1	1.5263158	0.6100535	0.4545999	5	7	7	8	13	8
U1	1.5263158	0.6100535	0.4545999	5	7	7	8	13	8
U1	1.5263158	0.6100535	0.4545999	5	7	7	8	13	8
U2	0.25	-2	0.3210354	10	0	2	0	0	3
U2	0.25	-2	0.3210354	10	0	2	0	0	3
U2	0.25	-2	0.3210354	10	0	2	0	0	3
U2	0.25	-2	0.3210354	10	0	2	0	0	3

U2	0.25	-2	0.3210354	10	0	2	0	0	3
U2	0.25	-2	0.3210354	10	0	2	0	0	3
U2	0.25	-2	0.3210354	10	0	2	0	0	3
U2	0.25	-2	0.3210354	10	0	2	0	0	3
U2	0.25	-2	0.3210354	10	0	2	0	0	3
U2	0.25	-2	0.3210354	10	0	2	0	0	3
U2	0.25	-2	0.3210354	10	0	2	0	0	3
U2	0.25	-2	0.3210354	10	0	2	0	0	3
U2	0.25	-2	0.3210354	10	0	2	0	0	3
U2	0.25	-2	0.3210354	10	0	2	0	0	3
U2	0.25	-2	0.3210354	10	0	2	0	0	3
U2	0.25	-2	0.3210354	10	0	2	0	0	3
U2	0.25	-2	0.3210354	10	0	2	0	0	3
U2	0.25	-2	0.3210354	10	0	2	0	0	3
U2	0.25	-2	0.3210354	10	0	2	0	0	3
U2	0.25	-2	0.3210354	10	0	2	0	0	3
U2	0.25	-2	0.3210354	10	0	2	0	0	3
U3	0.9444444	-0.082462	0.9707452	9	2	7	4	9	4
U3	0.9444444	-0.082462	0.9707452	9	2	7	4	9	4
U3	0.9444444	-0.082462	0.9707452	9	2	7	4	9	4
U3	0.9444444	-0.082462	0.9707452	9	2	7	4	9	4
U3	0.9444444	-0.082462	0.9707452	9	2	7	4	9	4
U3	0.9444444	-0.082462	0.9707452	9	2	7	4	9	4
U3	0.9444444	-0.082462	0.9707452	9	2	7	4	9	4
U3	0.9444444	-0.082462	0.9707452	9	2	7	4	9	4
U3	0.9444444	-0.082462	0.9707452	9	2	7	4	9	4
U3	0.9444444	-0.082462	0.9707452	9	2	7	4	9	4
U3	0.9444444	-0.082462	0.9707452	9	2	7	4	9	4
U3	0.9444444	-0.082462	0.9707452	9	2	7	4	9	4
U3	0.9444444	-0.082462	0.9707452	9	2	7	4	9	4
U3	0.9444444	-0.082462	0.9707452	9	2	7	4	9	4
U3	0.9444444	-0.082462	0.9707452	9	2	7	4	9	4
U3	0.9444444	-0.082462	0.9707452	9	2	7	4	9	4
U3	0.9444444	-0.082462	0.9707452	9	2	7	4	9	4
U3	0.9444444	-0.082462	0.9707452	9	2	7	4	9	4
U3	0.9444444	-0.082462	0.9707452	9	2	7	4	9	4
U3	0.9444444	-0.082462	0.9707452	9	2	7	4	9	4
U3	0.9444444	-0.082462	0.9707452	9	2	7	4	9	4
U3	0.9444444	-0.082462	0.9707452	9	2	7	4	9	4
U3	0.9444444	-0.082462	0.9707452	9	2	7	4	9	4
U3	0.9444444	-0.082462	0.9707452	9	2	7	4	9	4
U3	0.9444444	-0.082462	0.9707452	9	2	7	4	9	4
U3	0.9444444	-0.082462	0.9707452	9	2	7	4	9	4
U3	0.9444444	-0.082462	0.9707452	9	2	7	4	9	4
U3	0.9444444	-0.082462	0.9707452	9	2	7	4	9	4
U3	0.9444444	-0.082462	0.9707452	9	2	7	4	9	4
U3	0.9444444	-0.082462	0.9707452	9	2	7	4	9	4
U3	0.9444444	-0.082462	0.9707452	9	2	7	4	9	4
U3	0.9444444	-0.082462	0.9707452	9	2	7	4	9	4
U3	0.9444444	-0.082462	0.9707452	9	2	7	4	9	4
U3	0.9444444	-0.082462	0.9707452	9	2	7	4	9	4
U3	0.9444444	-0.082462	0.9707452	9	2	7	4	9	4
U3	0.9444444	-0.082462	0.9707452	9	2	7	4	9	4
U3	0.9444444	-0.082462	0.9707452	9	2	7	4	9	4
U3	0.9444444	-0.082462	0.9707452	9	2	7	4	9	4

U3	0.9444444	-0.082462	0.9707452	9	2	7	4	9	4
U3	0.9444444	-0.082462	0.9707452	9	2	7	4	9	4
U3	0.9444444	-0.082462	0.9707452	9	2	7	4	9	4
U3	0.9444444	-0.082462	0.9707452	9	2	7	4	9	4
U3	0.9444444	-0.082462	0.9707452	9	2	7	4	9	4
U3	0.9444444	-0.082462	0.9707452	9	2	7	4	9	4
U3	0.9444444	-0.082462	0.9707452	9	2	7	4	9	4
U3	0.9444444	-0.082462	0.9707452	9	2	7	4	9	4
U3	0.9444444	-0.082462	0.9707452	9	2	7	4	9	4
U3	0.9444444	-0.082462	0.9707452	9	2	7	4	9	4
U3	0.9444444	-0.082462	0.9707452	9	2	7	4	9	4
U3	0.9444444	-0.082462	0.9707452	9	2	7	4	9	4
U3	0.9444444	-0.082462	0.9707452	9	2	7	4	9	4
U3	0.9444444	-0.082462	0.9707452	9	2	7	4	9	4
AC018635.2	0	#NUM!	0.824326	0	0	1	0	0	0
U4	0.9230769	-0.115477	0.9887307	0	8	5	6	2	4
U4	0.9230769	-0.115477	0.9887307	0	8	5	6	2	4
U4	0.9230769	-0.115477	0.9887307	0	8	5	6	2	4
U4	0.9230769	-0.115477	0.9887307	0	8	5	6	2	4
U4	0.9230769	-0.115477	0.9887307	0	8	5	6	2	4
U4	0.9230769	-0.115477	0.9887307	0	8	5	6	2	4
U4	0.9230769	-0.115477	0.9887307	0	8	5	6	2	4
U6	0.4117647	-1.280108	0.3458935	5	3	9	2	3	2
U6	0.4117647	-1.280108	0.3458935	5	3	9	2	3	2
U6	0.4117647	-1.280108	0.3458935	5	3	9	2	3	2
U6	0.4117647	-1.280108	0.3458935	5	3	9	2	3	2
U6	0.4117647	-1.280108	0.3458935	5	3	9	2	3	2
U6	0.4117647	-1.280108	0.3458935	5	3	9	2	3	2
U6	0.4117647	-1.280108	0.3458935	5	3	9	2	3	2
U6	0.4117647	-1.280108	0.3458935	5	3	9	2	3	2
U6	0.4117647	-1.280108	0.3458935	5	3	9	2	3	2
U6	0.4117647	-1.280108	0.3458935	5	3	9	2	3	2
U6	0.4117647	-1.280108	0.3458935	5	3	9	2	3	2
U6	0.4117647	-1.280108	0.3458935	5	3	9	2	3	2
U6	0.4117647	-1.280108	0.3458935	5	3	9	2	3	2
U6	0.4117647	-1.280108	0.3458935	5	3	9	2	3	2
U6	0.4117647	-1.280108	0.3458935	5	3	9	2	3	2
U6	0.4117647	-1.280108	0.3458935	5	3	9	2	3	2
U6	0.4117647	-1.280108	0.3458935	5	3	9	2	3	2
U6	0.4117647	-1.280108	0.3458935	5	3	9	2	3	2
U6	0.4117647	-1.280108	0.3458935	5	3	9	2	3	2
U6	0.4117647	-1.280108	0.3458935	5	3	9	2	3	2
U6	0.4117647	-1.280108	0.3458935	5	3	9	2	3	2
U6	0.4117647	-1.280108	0.3458935	5	3	9	2	3	2
U6	0.4117647	-1.280108	0.3458935	5	3	9	2	3	2
U6	0.4117647	-1.280108	0.3458935	5	3	9	2	3	2
U6	0.4117647	-1.280108	0.3458935	5	3	9	2	3	2
U6	0.4117647	-1.280108	0.3458935	5	3	9	2	3	2
U6	0.4117647	-1.280108	0.3458935	5	3	9	2	3	2
U6	0.4117647	-1.280108	0.3458935	5	3	9	2	3	2
U6	0.4117647	-1.280108	0.3458935	5	3	9	2	3	2
U6	0.4117647	-1.280108	0.3458935	5	3	9	2	3	2
U6	0.4117647	-1.280108	0.3458935	5	3	9	2	3	2
U6	0.4117647	-1.280108	0.3458935	5	3	9	2	3	2

U6	0.4117647	-1.280108	0.3458935	5	3	9	2	3	2
U6	0.4117647	-1.280108	0.3458935	5	3	9	2	3	2
U6	0.4117647	-1.280108	0.3458935	5	3	9	2	3	2
U6	0.4117647	-1.280108	0.3458935	5	3	9	2	3	2
U6	0.4117647	-1.280108	0.3458935	5	3	9	2	3	2
U8	0.3333333	-1.584963	0.4399555	2	2	5	2	0	1
U8	0.3333333	-1.584963	0.4399555	2	2	5	2	0	1
U8	0.3333333	-1.584963	0.4399555	2	2	5	2	0	1
U8	0.3333333	-1.584963	0.4399555	2	2	5	2	0	1
U8	0.3333333	-1.584963	0.4399555	2	2	5	2	0	1
U8	0.3333333	-1.584963	0.4399555	2	2	5	2	0	1
U8	0.3333333	-1.584963	0.4399555	2	2	5	2	0	1
U8	0.3333333	-1.584963	0.4399555	2	2	5	2	0	1
U8	0.3333333	-1.584963	0.4399555	2	2	5	2	0	1
U8	0.3333333	-1.584963	0.4399555	2	2	5	2	0	1
U8	0.3333333	-1.584963	0.4399555	2	2	5	2	0	1
U8	0.3333333	-1.584963	0.4399555	2	2	5	2	0	1
U8	0.3333333	-1.584963	0.4399555	2	2	5	2	0	1
U8	0.3333333	-1.584963	0.4399555	2	2	5	2	0	1
U8	0.3333333	-1.584963	0.4399555	2	2	5	2	0	1
U8	0.3333333	-1.584963	0.4399555	2	2	5	2	0	1
U8	0.3333333	-1.584963	0.4399555	2	2	5	2	0	1
U8	0.3333333	-1.584963	0.4399555	2	2	5	2	0	1
U8	0.3333333	-1.584963	0.4399555	2	2	5	2	0	1
U8	0.3333333	-1.584963	0.4399555	2	2	5	2	0	1
U8	0.3333333	-1.584963	0.4399555	2	2	5	2	0	1
CLYBL	0.6183432	-0.69352	0.0221317	117	104	117	60	74	75
AC009950.1	9.5	3.2479275	0.0455092	2	0	0	8	7	4
HYAL1	1.2424242	0.3131579	0.5203397	25	24	17	23	36	23
HYAL2	0.8293868	-0.269883	0.2978682	473	566	608	526	452	388
HYAL3	1.0813008	0.1127679	0.723254	32	61	30	58	42	33
AC023830.2	0	#NUM!	0.6436235	2	0	0	0	0	0
HYAL4	1.3384615	0.4205757	0.1620725	67	43	85	58	87	116
SSSCA1	1.0396359	0.0560784	0.4241164	927	1129	1350	1356	1140	1045
AC023830.1	0	#NUM!	0.824326	0	0	1	0	0	0
MCCD1P1	#DIV/0!	#DIV/0!	0.8030358	0	0	0	0	1	0
'HUMPD3-AS	0.8717454	-0.198021	0.5313003	398	243	396	304	308	292
AC092801.1	0	#NUM!	0.824326	0	1	0	0	0	0
AC116535.1	0.25	-2	0.1794173	6	4	10	0	5	0
ALS2CL	0.1538462	-2.70044	0.1589064	4	2	7	0	2	0
PLEKHG4B	2	1	0.0054662	30	26	66	59	80	105
MIR99AHG	0.5238095	-0.932886	0.4663814	6	3	12	2	2	7
XG	0.7586207	-0.398549	0.6857986	25	9	24	24	9	11
GLB1L	1.1290323	0.1750867	0.4553851	112	67	100	82	131	102
THEMIS2	1.41	0.4956952	0.0823902	43	77	80	83	103	96
AC106047.1	1.8	0.8479969	0.6327937	1	4	0	4	0	5
AC004520.1	0.5067751	-0.980583	0.0042968	132	77	160	61	57	69
AL450063.1	0.5	-1	0.8638172	0	0	2	1	0	0
AC034198.2	0	#NUM!	0.6385096	0	2	0	0	0	0
AC118344.1	0.5194805	-0.944858	0.1565361	28	20	29	6	10	24
AC118344.2	0.825	-0.277534	0.6793337	53	12	55	15	44	40
DKC1	0.8721746	-0.197311	0.191706	4394	4305	5414	3424	4675	4210
AC079610.2	1.0181818	0.0259952	0.8545697	12	16	27	17	18	21

BX119904.2	#DIV/0!	#DIV/0!	0.8030358	0	0	0	0	1	0
RPL18P10	0.5	-1	0.7031599	1	1	2	0	2	0
AC003682.1	0	#NUM!	0.1372033	3	0	6	0	0	0
HCAR2	0	#NUM!	0.5781912	0	0	3	0	0	0
TRGC1	4	2	0.5223508	1	0	0	1	3	0
AL117328.1	0.9	-0.152003	0.9438059	2	5	3	4	4	1
AL117328.2	0.4166667	-1.263034	0.2964548	2	15	7	0	7	3
HCAR1	1.5	0.5849625	0.7729581	0	0	2	2	0	1
PBX2P1	0.4444444	-1.169925	0.522068	5	2	2	1	0	3
AC016723.1	#DIV/0!	#DIV/0!	0.2808669	0	0	0	3	0	2
AC005014.2	0.2142857	-2.222392	0.2315559	11	0	3	1	0	2
ACTL10	0.4615385	-1.115477	0.5086184	5	3	5	0	0	6
AC005014.1	0	#NUM!	0.6436235	2	0	0	0	0	0
MEA1	0.8748655	-0.192867	0.444788	762	971	1056	854	900	686
SNORA79B	0.9565217	-0.06413	0.9110427	8	11	4	0	13	9
AL451047.1	#DIV/0!	#DIV/0!	0.1472932	0	0	0	1	0	7
AC098826.2	1	0	0.9890559	0	1	0	0	0	1
AC011043.1	1.0191693	0.0273938	0.6338946	385	375	492	326	476	474
AC011043.2	0.6666667	-0.584963	0.8317553	2	1	0	1	1	0
AKR1B1P6	#DIV/0!	#DIV/0!	0.8030358	0	0	0	1	0	0
AKR1B1P2	0	#NUM!	0.824326	0	0	1	0	0	0
AKR1B1P1	#DIV/0!	#DIV/0!	0.8030358	0	0	0	1	0	0
AP001432.1	1.4615385	0.5474878	0.5794313	8	3	2	12	3	4
ABCA12	1.170008	0.2265184	0.1407312	945	592	957	688	1060	1170
ABCA13	0.8315789	-0.266075	0.3097936	195	221	249	128	215	210
ADNP2	0.9048174	-0.144301	0.5128297	864	780	930	611	821	897
SNORA73B	1.6759259	0.7449584	0.1062494	24	55	29	34	92	55
MIR6088	#DIV/0!	#DIV/0!	0.4501902	0	0	0	0	0	4
MED1	0.9107489	-0.134875	0.5992083	2139	1919	2138	1022	2030	2591
MECR	0.9929852	-0.010156	0.6878861	1141	1251	1457	1333	1341	1148
AP003733.1	0	#NUM!	0.1771179	0	2	6	0	0	0
MED8	1.0743679	0.1034881	0.2831912	566	676	775	771	725	671
MED9	0.7683459	-0.380172	0.025635	771	824	926	622	637	678
AP003733.4	0.2045455	-2.289507	0.0327647	15	3	26	2	2	5
MED4	1.0485532	0.0684001	0.3831982	694	545	800	622	769	747
MED6	1.1227679	0.1670597	0.0778695	841	815	1032	951	1086	981
DKK3	2	1	0.8147319	1	0	0	0	0	2
MED7	1.1145889	0.1565116	0.149761	592	488	805	650	778	673
AC012360.2	2	1	0.4454645	2	2	3	8	6	0
AC012360.3	0.9763033	-0.034599	0.9486342	153	112	157	106	149	157
AC012360.1	2	1	0.5027621	0	2	3	7	0	3
SNORA74A	#DIV/0!	#DIV/0!	0.8030358	0	0	0	1	0	0
SNORA74B	0	#NUM!	0.5387425	3	0	0	0	0	0
RNU6-689P	0	#NUM!	0.0654744	6	5	0	0	0	0
SNORA74D	#DIV/0!	#DIV/0!	0.8030358	0	0	0	0	0	1
GPLD1	1.5384615	0.6214884	0.0469546	60	38	71	59	96	105
MIR6078	0.2857143	-1.807355	0.3990211	3	3	1	0	2	0
MEG3	#DIV/0!	#DIV/0!	0.8030358	0	0	0	1	0	0
OSER1	0.9636324	-0.053445	0.9983208	1352	1530	1710	1315	1716	1394
AC009126.1	0.8153846	-0.294447	0.6545348	29	21	15	16	17	20
VPS26A	1.3299501	0.4113722	0.0034045	2174	1424	2018	1960	2905	2604
VPS26B	0.7756628	-0.366498	0.029077	889	928	1125	753	780	749
MEFV	8	3	0.1884256	0	0	1	2	3	3
SNORD111B	1	0	0.9480217	0	0	2	1	1	0

AC113404.3	1.6052632	0.6828098	0.2288477	14	9	15	26	13	22
AC113404.1	#DIV/0!	#DIV/0!	0.8030358	0	0	0	1	0	0
AC131971.1	0.2692308	-1.893085	0.1226561	7	6	13	0	3	4
MIR3936HG	0.96	-0.058894	0.9737659	8	15	27	14	23	11
FRMPD2B	1.05	0.0703893	0.9341183	2	10	8	1	6	14
MEI1	1.5714286	0.6520767	0.6044382	0	3	4	5	3	3
MEI4	0.4545455	-1.137504	0.5874825	0	5	6	4	1	0
ZC3H10	0.7477064	-0.419456	0.1770175	125	127	184	111	123	92
GCFC2	0.984127	-0.023084	0.829427	485	234	541	372	470	398
SNORA70F	4	2	0.5412414	0	0	1	0	3	1
SNORA70G	#DIV/0!	#DIV/0!	0.5371397	0	0	0	0	0	3
AL807757.1	0	#NUM!	0.6631647	1	0	1	0	0	0
AL807757.2	1.2	0.2630344	0.8842018	0	0	5	0	6	0
AL390036.1	0	#NUM!	0.824326	0	0	1	0	0	0
LIMD1-AS1	1.6	0.6780719	0.500916	11	3	1	15	4	5
SNORA71A	0.6296296	-0.667425	0.4096821	20	15	19	12	4	18
SNORA71B	0.6293706	-0.668018	0.1893995	45	35	63	27	46	17
SNORA71C	1.0123457	0.017702	0.8581087	22	28	31	28	22	32
SNORA71D	1	0	0.9832201	0	3	0	0	0	3
AC083873.1	3.8181818	1.9328858	0.0247435	4	4	3	19	13	10
MSRB2	1.1544622	0.207221	0.1855009	229	298	347	394	337	278
MSRB3	2	1	0.3673077	8	1	3	3	11	10
MSRB1	1.1125107	0.1538193	0.1495622	1058	1240	1195	1314	1347	1225
COL16A1	0.7491525	-0.416669	0.2792523	81	82	132	81	74	66
MEN1	0.9815011	-0.026938	0.7917483	1616	1942	2118	1813	1949	1809
RNF43	0.7843915	-0.350354	0.1746933	248	196	312	177	241	175
AL589765.7	0	#NUM!	0.824326	1	0	0	0	0	0
RNF44	0.8828407	-0.179775	0.2918371	4057	4758	5294	3788	4474	4194
OR7E83P	0.6875	-0.540568	0.5980826	12	8	12	11	7	4
AL589765.6	0	#NUM!	0.4324683	2	0	2	0	0	0
AL589765.4	1.1891892	0.2499783	0.6557678	11	13	13	14	18	12
RNF40	0.7877593	-0.344173	0.0292383	2003	2411	2432	1437	1928	2028
RNF41	1.0204669	0.0292294	0.5938238	926	1147	1054	975	1106	1110
AC009220.3	1.1666667	0.2223924	0.797658	0	0	6	5	1	1
AC100778.2	1.3333333	0.4150375	0.7039043	10	3	2	5	10	5
AC009220.2	#DIV/0!	#DIV/0!	0.4155775	0	0	0	0	2	2
AC100778.4	2	1	0.8186965	0	0	1	0	0	2
MELK	1.2203931	0.2873459	0.0043494	3471	2822	3730	3465	4599	4168
AL589765.1	0	#NUM!	0.4217812	0	0	5	0	0	0
AL159987.1	0.6241611	-0.68001	0.0328097	134	131	182	114	83	82
OARD1	0.9459732	-0.080129	0.8810257	936	960	1084	930	1057	832
AC122718.1	1.0163934	0.023459	0.8287064	59	24	39	53	34	37
OR7E85P	0.7037037	-0.50696	0.7000271	9	1	17	6	9	4
AC122718.2	0	#NUM!	0.6385096	0	2	0	0	0	0
PALB2	0.8485804	-0.236877	0.3222994	620	382	583	368	497	480
RNF10	1.0310176	0.0440689	0.3518194	3748	4072	4818	4083	4724	4223
RNF11	1.1352372	0.1829938	0.0518008	1405	1292	1540	1380	1797	1633
RNF13	1.0502536	0.0707377	0.3862782	740	531	898	692	836	750
RNF14	0.8095495	-0.304809	0.0451461	1212	1145	1350	838	1142	1021
TREM2	0.6666667	-0.584963	0.7662923	0	3	6	0	5	1
TREM1	2.7268293	1.4472244	2.16E-09	56	65	84	173	209	177
FKBP9P1	0.5172414	-0.95109	0.3709356	8	5	16	5	5	5
RNF17	0.9090909	-0.137504	0.9695244	11	7	15	14	8	8
OR7E87P	7	2.8073549	0.254944	0	0	1	5	2	0

MEPE	#DIV/0!	#DIV/0!	0.8030358	0	0	0	1	0	0
RANGAP1	0.9666126	-0.04899	0.9129282	7633	9289	10184	8644	9322	8235
IL3RA	0.4830508	-1.049753	0.0596823	37	22	59	16	25	16
SSX2IP	1.0319767	0.0454105	0.6929725	557	338	481	329	585	506
RNF31	0.8664207	-0.20686	0.2962181	758	866	1086	634	881	833
RNU6-680P	0	#NUM!	0.4185478	3	0	1	0	0	0
AC006330.1	0.5833333	-0.777608	0.3244312	10	24	14	2	14	12
RNF32	0.768595	-0.379704	0.4431267	52	31	38	18	39	36
SMARCD1	0.8437868	-0.24505	0.1228642	2185	2579	2873	1944	2290	2210
SMARCD2	0.911151	-0.134238	0.521893	3827	4666	4878	3784	4406	3993
RNF34	0.9071182	-0.140637	0.5176243	1362	1701	1868	1290	1620	1563
SMARCD3	0.6027397	-0.730393	0.1642352	50	35	61	39	32	17
RNF38	1.1462483	0.1969196	0.1273556	2170	1829	2025	1649	2652	2604
AC106871.1	#DIV/0!	#DIV/0!	0.6554062	0	0	0	0	2	0
ITLN1	0.7777778	-0.36257	0.638105	14	15	16	8	13	14
ITLN2	1	0	0.9890559	0	1	0	0	1	0
MESD	0.9139258	-0.129851	0.4981348	3544	3991	4292	3208	3927	3674
RNF39	0.9622642	-0.055495	0.931452	44	32	30	14	35	53
MEST	0.5932203	-0.75336	0.3183767	12	24	23	16	11	8
RNF20	0.9718847	-0.041143	0.9110135	2509	1883	2686	1947	2524	2408
SMARCE1	1.0559579	0.0785523	0.3960912	462	462	595	424	577	603
ILF3-AS1	0.8151515	-0.29486	0.2690139	423	385	512	419	346	311
RNF24	0.6612666	-0.596696	0.0003579	624	623	790	359	488	500
RNF25	1.0656545	0.0917398	0.3118462	659	843	935	845	946	806
RNF26	0.843805	-0.245019	0.1668454	1715	2186	2354	1425	1867	1986
C2orf69P1	0	#NUM!	0.5781912	0	0	3	0	0	0
PNRC1	1.0242248	0.0345324	0.4591259	912	979	1205	950	1109	1112
PNRC2	1.1646267	0.2198676	0.030192	3742	2982	3979	3385	4601	4479
TRMT61A	0.7517326	-0.411709	0.031085	670	810	973	622	673	549
TRMT61B	1.2866242	0.3635907	0.0125182	312	345	442	402	468	544
AL031710.2	3.1666667	1.662965	0.257031	0	6	0	11	6	2
AC005253.1	0.65	-0.621488	0.2652269	24	43	33	9	35	21
MIR320B2	1.3333333	0.4150375	0.8157784	0	2	1	4	0	0
AFG3L2	0.7530049	-0.409269	0.0046349	1268	1382	1593	922	1205	1068
COX7A2L	1.033468	0.0474937	0.3852279	4659	5658	6266	5355	6324	5459
AL390860.1	#DIV/0!	#DIV/0!	0.8030358	0	0	0	1	0	0
GPM6A	0.5862069	-0.770518	0.4685581	8	5	16	3	5	9
C17orf105	1	0	0.921736	2	0	5	3	1	3
HIST1H3J	0.8444444	-0.243926	0.784825	14	13	18	12	17	9
AC018730.2	0.1538462	-2.70044	0.1506884	7	6	0	0	1	1
BRI3BPP1	0	#NUM!	0.4374838	0	4	0	0	0	0
C17orf107	0	#NUM!	0.0651467	2	0	11	0	0	0
AC022417.1	0.625	-0.678072	0.72348	1	3	4	0	1	4
C17orf100	1	0	0.8317726	54	56	70	68	62	50
IFNL3P1	1.3421053	0.4244978	0.4783434	7	14	17	11	20	20
HIST1H3A	#DIV/0!	#DIV/0!	0.8030358	0	0	0	0	0	1
HIST1H3H	0.8763441	-0.190431	0.7443097	53	49	84	46	51	66
HIST1H3C	0.375	-1.415037	0.5417273	0	3	5	2	1	0
GPM6B	1.1923077	0.2537566	0.7137977	9	7	10	9	16	6
HIST1H3E	#DIV/0!	#DIV/0!	0.2905913	0	0	0	2	0	3
C17orf113	0.8766234	-0.189971	0.7228197	85	110	113	115	88	67
HIST1H4K	1.1891892	0.2499783	0.6205574	6	6	25	15	18	11
TREX2	0.8888889	-0.169925	0.9001972	3	5	1	0	1	7
IGFL2	0.1666667	-2.584963	0.3217314	4	1	1	0	1	0

IGFL1	3	1.5849625	0.6253252	0	0	1	3	0	0
CDAN1	0.9146251	-0.128748	0.7239961	409	443	495	432	386	414
HIST1H4B	#DIV/0!	#DIV/0!	0.4737294	0	0	0	0	4	0
IGFL4	0.2962963	-1.754888	0.1936535	5	1	21	2	2	4
HIST1H4H	0.9913793	-0.012491	0.7500811	178	155	247	217	174	184
HIST1H4I	1.0300245	0.0426787	0.5250727	517	491	624	581	643	457
HIST1H4J	0.88	-0.184425	0.8310185	3	19	3	5	13	4
HIST1H4D	1.3076923	0.3870231	0.7688841	9	2	2	2	11	4
ITM2A	1.0325203	0.0461702	0.6445216	276	288	297	230	353	306
HIST1H4E	0.8769231	-0.189478	0.843427	35	15	15	25	11	21
ITM2B	0.9521376	-0.070758	0.9210583	8047	8221	9953	7757	9010	8199
RAB40AL	0.3611111	-1.469485	0.1310019	12	6	18	3	6	4
ITM2C	1.3919978	0.477157	0.0001783	1068	1334	1297	1690	1732	1727
QRFP	0.4888889	-1.032421	0.1922201	17	14	14	6	11	5
EPM2AIP1	0.7659479	-0.384682	0.0797842	818	540	868	383	652	670
AC007314.1	#DIV/0!	#DIV/0!	0.5371397	0	0	0	0	0	3
AC024563.2	1	0	0.9890559	0	0	1	1	0	0
GNPNAT1	0.7695035	-0.378	0.0459702	912	894	1014	476	837	857
STK24P1	1	0	0.9239532	0	0	2	2	0	0
AC024563.1	0.2	-2.321928	0.1428304	7	4	4	0	2	1
DLAT	0.8345845	-0.26087	0.0690501	3621	3519	4050	2487	3506	3346
RSPRY1	1.0724477	0.1009073	0.2785869	2276	1711	2321	1796	2471	2498
AVPR1A	#DIV/0!	#DIV/0!	0.6482162	0	0	0	0	1	1
IGFN1	#DIV/0!	#DIV/0!	0.8030358	0	0	0	0	1	0
MSS51	0.8151261	-0.294905	0.6866917	40	16	63	31	38	28
MKLN1-AS	0.35	-1.514573	0.2325301	6	8	6	0	3	4
PALD1	0	#NUM!	0.5781912	0	0	3	0	0	0
RPUSD4	0.8581245	-0.220741	0.2514638	788	737	949	678	758	687
RPUSD1	0.8678029	-0.204561	0.4126311	616	763	792	676	657	551
RPUSD2	0.8451413	-0.242736	0.2137177	715	917	951	682	781	720
RPUSD3	0.8190203	-0.288029	0.1584565	1341	1667	1871	1422	1410	1164
CYP3A51P	0	#NUM!	0.6436235	2	0	0	0	0	0
NCR3LG1	0.4642857	-1.106915	0.3080852	7	14	7	0	4	9
AL359878.1	0.9772727	-0.033167	0.9646403	47	27	58	22	43	64
AL359878.2	0	#NUM!	0.6436235	2	0	0	0	0	0
PDZK1IP1	0	#NUM!	0.4521054	1	0	3	0	0	0
UBE2E1-AS1	1.1764706	0.2344653	0.7226592	2	4	11	8	5	7
PDXDC2P	0	#NUM!	0.3626892	1	0	4	0	0	0
DLC1	0.9681242	-0.046736	0.9775324	1635	1502	1757	1138	1671	1929
AC092800.1	0.8333333	-0.263034	0.8910539	1	4	1	0	0	5
AC104984.6	0	#NUM!	0.824326	1	0	0	0	0	0
AC055764.2	0.3333333	-1.584963	0.7307796	0	0	3	0	1	0
AC104984.1	#DIV/0!	#DIV/0!	0.6099294	0	0	0	2	0	0
AC104984.2	1.4	0.4854268	0.8085063	1	0	4	0	7	0
SDR9C7	0.5625	-0.830075	0.5218702	7	8	1	0	3	6
TNFRSF14-AS	0.2857143	-1.807355	0.5057867	0	1	6	0	0	2
NUSAP1	1.0843355	0.1168112	0.1829582	4560	3512	4817	3834	5270	4872
SIRPA	1.3121099	0.3918885	0.0439556	277	297	227	366	386	299
TBCCD1	0.8541549	-0.22743	0.2773455	516	581	672	483	556	472
PAM16	0.7013889	-0.511714	0.3473795	49	31	64	9	48	44
GLTPD2	0.9674797	-0.047697	0.9915064	53	22	48	23	48	48
RGS9BP	1.4285714	0.5145732	0.4267237	25	6	11	20	9	31
ELMO1	0.9788294	-0.030871	0.8709621	1215	1380	1420	1205	1445	1280
ELMO2	0.6694301	-0.578995	3.24E-05	1451	1539	1835	894	1163	1173

ELMO3	0.9265574	-0.110048	0.7453212	918	981	1151	949	1030	847
RELL1	0.9491979	-0.075219	0.8597282	265	237	246	182	260	268
RELL2	1.1456311	0.1961425	0.6507319	31	21	51	14	64	40
AP000593.3	0.3571429	-1.485427	0.3367959	4	3	7	0	2	3
AP000593.1	2	1	0.8186965	0	0	1	0	0	2
AL137077.2	3.2857143	1.716207	0.032716	4	4	6	22	16	8
RSL24D1P11	0	#NUM!	0.4460243	4	0	0	0	0	0
AL137077.1	2	1	0.6529872	0	0	2	2	0	2
KCNC4-AS1	0.25	-2	0.5705817	1	0	3	0	1	0
AC034207.1	0.1666667	-2.584963	0.3479817	1	1	4	1	0	0
ZC3H18	0.9014238	-0.149723	0.4253173	4463	5445	5684	4232	5086	4737
ZC3H13	1.0632538	0.088486	0.3996038	2936	1804	3054	2082	3201	3004
ZC3H14	1.0568655	0.0797917	0.3454448	2538	1849	2823	2012	2912	2696
ZC3H15	1.225994	0.2939519	0.0334988	3762	2189	3682	3394	4630	3786
AL442128.2	1	0	0.9020352	8	3	13	8	2	14
AC145212.1	0.7857143	-0.347923	0.7680208	11	2	1	1	9	1
DLG1	0.9423543	-0.085659	0.8224375	2290	1493	2410	1322	2243	2271
DLG2	1.0569948	0.0799683	0.5021749	195	140	244	181	228	203
DLG3	0.9179668	-0.123486	0.5845902	1756	1945	2260	1670	1987	1815
DLG4	0.9466667	-0.079072	0.9695811	13	30	32	19	27	25
DLG5	1.1784409	0.2368794	0.0659493	4256	4400	4749	3642	5808	6347
CLASRP	0.9441417	-0.082925	0.9285818	1484	1686	1968	1687	1593	1571
HIST1H1T	0.2	-2.321928	0.4376609	4	0	1	0	1	0
AC003681.1	0.549763	-0.863118	0.0838359	60	66	85	11	34	71
ZC3H7A	0.9276932	-0.10828	0.6801065	2421	1820	2591	1650	2387	2301
ZC3H7B	0.833095	-0.263447	0.095224	2992	3633	3866	2566	3013	3161
HIST1H1D	#DIV/0!	#DIV/0!	0.6554062	0	0	0	0	2	0
HIST1H1E	0.8666667	-0.206451	0.7979582	21	17	37	10	24	31
HIST1H1B	0.55	-0.862496	0.4600536	7	6	7	4	3	4
HIST1H1C	0.2903226	-1.784271	0.1026835	10	13	8	0	3	6
AC026700.1	#DIV/0!	#DIV/0!	0.6411933	0	0	0	0	0	2
BBOX1-AS1	0.9356061	-0.096027	0.9660838	56	93	115	93	88	66
AC016722.2	1.7272727	0.7884959	0.4407056	7	1	14	0	17	21
AC016722.3	2.25	1.169925	0.5539601	2	1	1	0	0	9
PALLD	0.9227864	-0.115931	0.5637657	4460	4267	5040	3562	4521	4621
CHMP3	0.952296	-0.070518	0.8944095	647	753	843	564	758	814
AC016722.1	#DIV/0!	#DIV/0!	0.8030358	0	0	0	1	0	0
CHMP6	1.2365145	0.3062792	0.2631756	89	81	71	124	103	71
ECT2L	1.3333333	0.4150375	0.7535929	6	6	0	5	1	10
CHMP7	0.9684256	-0.046287	0.9966765	679	832	801	628	807	804
CHMP5	1.3014207	0.3800874	0.0002071	2005	1795	2394	2326	3095	2640
PCNPP5	0.75	-0.415037	0.9271823	0	1	3	2	1	0
SNORA68B	0.8333333	-0.263034	0.9497566	0	0	6	0	4	1
RN7SL589P	#DIV/0!	#DIV/0!	0.5371397	0	0	0	0	0	3
PALM3	1.306319	0.3855072	0.0031738	1515	1579	1891	2482	2123	1907
DLL1	1.2641509	0.3381687	0.3308446	30	29	47	56	49	29
PCNPP1	0.2592593	-1.947533	0.0863235	7	11	9	3	2	2
DLL3	1.4909091	0.5761923	0.2323885	19	12	24	32	31	19
DLL4	1.05	0.0703893	0.8584159	6	8	6	13	2	6
IGFBPL1	#DIV/0!	#DIV/0!	0.8030358	0	0	0	0	0	1
CLASP1	0.9177829	-0.123775	0.5756557	1470	1285	1575	1026	1445	1503
CLASP2	0.8743097	-0.193784	0.4082083	1700	1121	1706	843	1570	1545
AC025279.1	0	#NUM!	0.824326	1	0	0	0	0	0
DLK2	1.0440252	0.0621565	0.7031861	37	42	80	50	65	51

AL033527.5	0.8235294	-0.280108	0.8348137	7	2	8	2	8	4
FLJ12825	0.5833333	-0.777608	0.5805694	7	4	1	0	4	3
NOC2L	0.9736256	-0.038561	0.8533069	4132	5130	5563	4652	4966	4816
AL033527.2	2	1	0.7433853	2	0	0	0	3	1
AC092798.1	1	0	0.9890559	1	0	0	0	0	1
AC079905.1	0.5	-1	0.812391	0	2	0	0	1	0
AC012613.2	#DIV/0!	#DIV/0!	0.6554062	0	0	0	0	2	0
RABL3	0.8544084	-0.227002	0.2728807	596	486	642	437	508	528
RABL6	1.0990944	0.1363152	0.1352545	2680	3230	3586	3611	3566	3260
CDCA2	1.0568278	0.0797404	0.3297457	1244	895	1398	1097	1375	1266
CDCA3	0.9423631	-0.085645	0.8533235	1464	1587	1807	1491	1570	1517
CDCA4	0.8988912	-0.153782	0.4410789	3315	3838	4211	3261	3481	3473
CDCA5	0.8606726	-0.216464	0.2153378	2955	3363	3881	2851	3206	2721
CDCA7	1.0074892	0.0107644	0.6203504	3361	3160	3894	2768	4028	3697
CDCA8	0.9785531	-0.031278	0.8779274	1978	2247	2396	1858	2403	2218
AC026358.1	0.2727273	-1.874469	0.2030001	4	7	11	0	0	6
AC021087.2	1.6190476	0.6951454	0.3669485	7	2	12	11	6	17
AC021087.3	0.7777778	-0.36257	0.6510326	20	14	20	15	10	17
AC021087.1	0.5333333	-0.906891	0.5259032	9	1	5	0	4	4
AL135905.2	0.5795455	-0.787006	0.159081	31	32	25	15	18	18
FUCA1	0.9436842	-0.083624	0.8592903	570	606	724	531	628	634
FUCA2	0.7443817	-0.425886	0.0034673	2158	2588	2863	1762	2055	1847
CARS-AS1	#DIV/0!	#DIV/0!	0.2106225	0	0	0	4	0	2
MAGOHB	0.9662958	-0.049463	0.9236705	935	938	1183	961	1131	861
AC008536.1	0.3766234	-1.408806	0.0651632	28	12	37	2	9	18
AC008536.2	1.375	0.4594316	0.7774672	2	4	2	0	6	5
AC008536.3	0.9818182	-0.026472	0.919146	12	14	29	13	6	35
AL358794.1	0	#NUM!	0.6410557	1	1	0	0	0	0
PANX1	0.8659004	-0.207727	0.3058527	728	708	913	653	706	675
PANX2	1.0264262	0.0376299	0.5282438	625	838	921	935	786	726
TUBB2BP1	#DIV/0!	#DIV/0!	0.8030358	0	0	0	1	0	0
PANX3	2.0555556	1.0395284	0.217805	9	6	3	10	10	17
AC211469.1	0	#NUM!	0.5781912	0	0	3	0	0	0
OR7E91P	1.2422907	0.3130029	0.1027145	122	135	197	154	226	184
MTCYBP35	0	#NUM!	0.824326	0	0	1	0	0	0
SLC7A4	0.8143415	-0.296294	0.1421993	2151	2509	2773	2247	1950	1856
SLC7A5	0.948449	-0.076358	0.8465833	17266	21380	21605	17464	20522	19159
SLC7A6	0.8032407	-0.316096	0.1378843	520	635	573	310	525	553
SLC7A7	1.2179063	0.2844032	0.0068368	5456	6630	6656	6988	8580	7258
SLC7A8	1.1368626	0.185058	0.0313366	9830	11078	11884	10902	13370	13008
SLC7A9	2	1	0.5797731	0	0	4	3	5	0
NUP62CL	1.0818278	0.1134709	0.3999193	373	231	337	304	378	336
CACUL1	1.1936255	0.2553503	0.0293303	1690	1502	1828	1492	2284	2216
ELOF1	1.0417969	0.059074	0.4285312	738	835	987	1012	900	755
AC003101.2	#DIV/0!	#DIV/0!	0.6099294	0	0	0	2	0	0
DLST	0.9730853	-0.039362	0.8828666	5439	6247	7077	5347	6487	6424
NOC4L	0.9023425	-0.148253	0.6395124	888	995	1148	1002	857	876
SLC7A1	0.782882	-0.353133	0.0855633	591	780	697	382	592	645
SLC7A2	1.0398486	0.0563735	0.6151758	3471	2839	3201	2132	3540	4218
MFN1	1.1265005	0.1718479	0.1304726	1141	854	1254	958	1394	1308
MFN2	0.9427827	-0.085003	0.7506611	4428	4915	5233	3838	4999	4905
P4HTM	0	#NUM!	0.824326	0	0	1	0	0	0
NOC3L	1.2721193	0.347234	0.0468367	794	391	759	681	888	904
AC004076.1	1	0	0.9890559	1	0	0	1	0	0

AC004076.2	1.1818182	0.2410081	0.7965327	5	3	3	6	1	6
RABIF	0.8801408	-0.184194	0.293085	1372	1475	1700	1205	1424	1373
AL121936.1	0.6666667	-0.584963	0.9244858	0	0	6	4	0	0
OR7E96P	2	1	0.6526252	2	1	0	1	0	5
ADORA1	0.8333333	-0.263034	0.9533086	0	1	5	1	1	3
AC144522.1	0	#NUM!	0.6385096	0	2	0	0	0	0
AC008870.3	1.25	0.3219281	0.8340417	3	0	1	4	1	0
AC008870.4	4.3333333	2.1154772	0.1014665	0	0	6	3	12	11
MFNG	#DIV/0!	#DIV/0!	0.6411933	0	0	0	0	0	2
IL6ST	1.1764706	0.2344653	0.4547271	3227	1620	2837	1828	3570	3642
CHODL	0.6672414	-0.583719	0.0352821	226	151	203	126	157	104
COL17A1	0.4151752	-1.268208	8.26E-19	2023	2305	2209	750	965	999
DLX1	0.2307692	-2.115477	0.2462237	1	3	9	0	1	2
DLX2	0.6764706	-0.563901	0.6056406	8	12	14	13	3	7
DLX3	0.928964	-0.106305	0.6335551	5204	5762	6321	4801	6053	5205
DLX4	1.1267134	0.1721206	0.0942981	1001	1115	1167	1195	1354	1150
DLX5	1.2383347	0.3084013	0.0062879	984	1196	1399	1422	1567	1443
DLX6	1.1541576	0.2068402	0.044336	911	999	1301	1207	1232	1267
MTCYBP21	1.3529412	0.4360991	0.5939309	4	6	7	10	2	11
AL356259.1	#DIV/0!	#DIV/0!	0.8030358	0	0	0	0	0	1
CORO1A	0.5325444	-0.909026	0.0554255	34	49	86	25	37	28
CORO1B	0.8118142	-0.300778	0.1229502	2092	2862	2884	2212	2228	1923
CORO1C	0.9983605	-0.002367	0.6690192	9441	9600	10846	8252	10878	10708
MTCYBP28	0.25	-2	0.5554205	4	0	0	0	1	0
OR7E99P	0.8409091	-0.249978	0.7587109	24	37	27	35	13	26
SLC14A1	1.1935484	0.2552571	0.673376	9	6	16	7	11	19
SLC14A2	0	#NUM!	0.5337954	0	3	0	0	0	0
ELOA2	1.2	0.2630344	0.8271627	1	0	4	2	0	4
CORO2A	1.3229386	0.4037461	0.0010045	2663	3198	3150	3149	4249	4523
MTCYBP13	0	#NUM!	0.824326	0	1	0	0	0	0
MTCYBP18	2	1	0.753159	0	2	0	0	0	4
AC012363.1	3	1.5849625	0.6732949	0	0	1	0	0	3
AC012363.2	0.3333333	-1.584963	0.6659102	3	0	0	1	0	0
AC108206.1	0	#NUM!	0.824326	0	0	1	0	0	0
OR10AE3P	#DIV/0!	#DIV/0!	0.8030358	0	0	0	0	1	0
AC106872.2	0	#NUM!	0.6385096	0	2	0	0	0	0
TOB1-AS1	1.65	0.722466	0.3792927	8	7	5	13	15	5
AC090505.1	1.25	0.3219281	0.5255343	9	20	27	14	21	35
AC106872.5	#DIV/0!	#DIV/0!	0.4737294	0	0	0	0	4	0
CDC73	1.0657396	0.0918549	0.3728466	1730	1231	1785	1249	1937	1872
AC106872.7	1.7916667	0.8413023	0.1043926	9	12	27	23	30	33
AP002807.1	0.6721311	-0.573185	0.4983467	18	16	27	23	9	9
AL118506.1	0.7693837	-0.378225	0.1647655	167	153	183	92	143	152
AL365273.1	1.3317422	0.4133149	0.035387	124	142	153	187	198	173
AC092892.1	0	#NUM!	0.6840298	0	0	2	0	0	0
AC084125.2	1.2068966	0.271302	0.7003709	0	14	15	11	8	16
SLC5A11	1.1204819	0.1641194	0.5499555	40	51	75	40	64	82
SLC5A12	1.1780822	0.2364402	0.3454687	74	63	82	67	106	85
SLC5A10	0.9295775	-0.105353	0.9157684	18	30	23	23	29	14
AC044781.1	#DIV/0!	#DIV/0!	0.4242335	0	0	0	0	3	1
EHBP1	1.3340798	0.415845	0.0031554	1761	1135	2016	1743	2478	2332
AC008870.2	1.0175439	0.025091	0.9133771	15	22	20	13	21	24
CDC42	1.0905783	0.1250934	0.083384	13591	14327	16331	14145	17806	16306
AC032044.1	1.2222222	0.2895066	0.7729529	2	2	5	3	2	6

AC032044.2	#DIV/0!	#DIV/0!	0.6554062	0	0	0	0	2	0
CDC45	0.9095456	-0.136782	0.6525274	643	769	987	693	772	717
GADD45GIP1	1.0300353	0.0426938	0.4975902	1254	1564	1710	1739	1662	1263
AC092902.1	#DIV/0!	#DIV/0!	0.0608992	0	0	0	4	5	1
FGF7P6	#DIV/0!	#DIV/0!	0.6411933	0	0	0	0	0	2
CDC5L	1.0416304	0.0588434	0.5622143	2263	1161	2317	1489	2406	2085
AC092902.5	1.6	0.6780719	0.5326247	2	2	6	3	7	6
AC092902.2	1.8188976	0.8630644	0.0174733	49	21	57	56	96	79
AC092902.3	#DIV/0!	#DIV/0!	0.3096126	0	0	0	5	0	0
PCBP2-OT1	#DIV/0!	#DIV/0!	0.6554062	0	0	0	0	2	0
AC022165.1	0.5	-1	0.4628449	5	6	11	5	0	6
FLJ46284	2.5	1.3219281	0.4586277	3	1	0	6	4	0
AC027281.2	0.4761905	-1.070389	0.4139773	9	6	6	0	10	0
CDC20	0.9824622	-0.025526	0.7833394	7381	9038	9468	8488	9014	7931
CDC23	0.9410514	-0.087655	0.7331208	4339	4071	4754	3351	4496	4541
HYKK	0.7477477	-0.419376	0.5040093	28	31	52	35	29	19
CDC26	1.2847222	0.3614565	0.1315824	100	97	91	118	145	107
CDC27	1.0676933	0.0944974	0.40545	2209	1472	2228	1486	2474	2349
AC084855.2	#DIV/0!	#DIV/0!	0.4155775	0	0	0	0	2	2
ATP6V1E1	0.8518778	-0.231282	0.1485176	5521	5806	7205	5121	5619	5047
ATP6V1E2	0.8333333	-0.263034	0.8175551	8	5	5	2	5	8
ATP6V1G1	1.1254968	0.1705619	0.0365161	3954	4233	4897	4555	5335	4836
ATP6V1G2	0.5	-1	0.4230064	4	9	5	0	6	3
AL442125.1	0.8192771	-0.287577	0.5795779	62	33	71	32	44	60
AL442125.2	0.9	-0.152003	0.9082049	11	1	8	0	8	10
CDC40	1.0689655	0.0962153	0.3267004	739	527	851	630	812	821
CDC34	1.0987365	0.1358455	0.1914611	1170	1484	1620	1728	1530	1438
CDC37	1.1157325	0.1579912	0.1111445	3181	3651	4444	4409	4613	3559
TRIB3	0.8448506	-0.243232	0.2488533	1205	1686	1595	1291	1337	1162
TRIB1	1.1443439	0.1945207	0.0679968	649	738	823	772	916	841
TRIB2	#DIV/0!	#DIV/0!	0.6411933	0	0	0	0	0	2
AC016573.1	0	#NUM!	0.824326	0	0	1	0	0	0
AC005154.5	0.25	-2	0.5141485	3	1	0	1	0	0
AC005154.1	0.8393782	-0.252607	0.231709	457	377	517	310	415	409
CDKN2AIP	1.0274348	0.0390469	0.6052497	687	693	807	542	895	810
AC005154.2	0	#NUM!	0.6840298	0	0	2	0	0	0
MIR6508	#DIV/0!	#DIV/0!	0.6554062	0	0	0	0	2	0
ATP6V1C1	1.1325479	0.179572	0.1107737	1224	880	1291	1067	1470	1308
ATP6V1C2	1.6967663	0.7627879	7.95E-08	448	566	625	918	944	919
AC093535.1	1.3030303	0.3818706	0.5508112	12	9	12	9	13	21
AC093535.2	0.9354839	-0.096215	0.9882027	18	17	27	21	14	23
FTH1P5	0	#NUM!	0.824326	0	1	0	0	0	0
FTH1P7	0.661385	-0.596438	0.0062157	476	599	629	425	393	309
FTH1P8	0.7525151	-0.410208	0.1438718	157	160	180	128	141	105
FTH1P1	1	0	0.9879501	0	1	3	0	4	0
FTH1P2	1.1266667	0.1720607	0.535106	43	56	51	54	63	52
FTH1P3	0.7	-0.514573	0.6403796	4	17	9	11	8	2
FTH1P4	0	#NUM!	0.3451105	2	0	3	0	0	0
CDC16	0.8823624	-0.180557	0.3594605	1949	1840	2425	1800	1921	1762
AC106779.1	1.4444444	0.5305147	0.6268034	4	1	4	5	3	5
SLC2A4RG	1.0138429	0.0198341	0.5266371	941	1070	1312	1165	1094	1110
EIF4BP2	2.5	1.3219281	0.5545371	0	0	2	2	0	3
EIF4BP3	1.1457859	0.1963375	0.2432475	128	125	186	152	173	178
RN7SL515P	#DIV/0!	#DIV/0!	0.8030358	0	0	0	1	0	0

SNORA14A	1	0	0.992244	1	1	1	0	0	3
EIF4BP6	1.0097087	0.0139392	0.639005	334	395	507	374	444	430
SNORA14B	0.25	-2	0.2014184	5	1	14	2	3	0
EIF4BP7	0.9182879	-0.122981	0.7744656	207	230	334	193	257	258
EIF4BP5	#DIV/0!	#DIV/0!	0.8030358	0	0	0	0	1	0
BANF1P2	0.6666667	-0.584963	0.8031653	5	1	0	3	1	0
BANF1P3	0.5714286	-0.807355	0.5286839	7	4	10	6	5	1
AC004816.1	1.25	0.3219281	0.1653748	76	76	104	118	91	111
ZNF587B	0.8349217	-0.260287	0.1862206	1055	773	1110	643	876	934
CLSTN2-AS1	0.2222222	-2.169925	0.3548417	0	2	7	1	1	0
AL512504.2	0	#NUM!	0.824326	1	0	0	0	0	0
AC092068.3	#DIV/0!	#DIV/0!	0.3981469	0	0	0	4	0	0
PLAC9P1	0.4	-1.321928	0.6095056	0	5	0	0	2	0
DMC1	1.6136364	0.6903155	0.2196964	11	14	19	40	9	22
AL449423.1	0.3333333	-1.584963	0.6582214	0	3	0	1	0	0
AL009178.2	1.6	0.6780719	0.4910806	6	11	3	3	10	19
SNORA16B	2.5	1.3219281	0.5527374	0	0	2	3	2	0
RN7SL513P	0.2307692	-2.115477	0.2365792	5	2	6	0	3	0
AC026691.1	0.7307692	-0.452512	0.6968937	10	3	13	5	6	8
AL009178.1	#DIV/0!	#DIV/0!	0.2256364	0	0	0	3	3	0
NFIL3	1.1480357	0.1991675	0.0373921	1676	1711	1882	1848	2282	1919
ATP6V1G3	#DIV/0!	#DIV/0!	0.8030358	0	0	0	1	0	0
ZNF585B	0.8695055	-0.201733	0.5328876	294	159	275	176	235	222
ZNF585A	0.8885794	-0.170427	0.6606103	158	80	121	72	128	119
OFCC1	1.0428571	0.0605415	0.8789995	27	22	21	15	33	25
POLR3DP1	2	1	0.7736272	0	1	0	2	0	0
AL138881.1	1.6666667	0.7369656	0.775104	6	0	0	0	10	0
AL356019.2	0.7017544	-0.510962	0.3416189	24	43	47	26	28	26
HYPK	1.9032258	0.9284467	0.1418256	6	14	11	18	22	19
AL031714.1	0.5873606	-0.767682	0.0054455	185	128	225	81	121	114
AP000254.1	1.1276596	0.1733316	0.7030557	16	12	19	15	11	27
SRP19	0.9192463	-0.121477	0.7924221	271	189	283	228	240	215
AMHR2	3.0555556	1.6114347	0.0237318	7	5	6	20	15	20
PCDH11Y	0.3157895	-1.662965	0.0933243	10	7	21	2	4	6
PCDH11X	1.2678571	0.3423922	0.5279662	21	10	25	10	30	31
WASHC2C	0.9937374	-0.009063	0.709011	1681	1351	1918	1385	1795	1739
SRP14	0.9840854	-0.023145	0.7716333	7881	9581	10374	8785	10069	8539
WASHC2A	1.0763739	0.1061793	0.1617754	1431	1431	1760	1456	1814	1705
AC037479.1	#DIV/0!	#DIV/0!	0.6411933	0	0	0	0	0	2
SNORA11B	0	#NUM!	0.6840298	0	0	2	0	0	0
SPOCD1	1.1456311	0.1961425	0.5666482	23	38	42	41	51	26
AC098824.1	0	#NUM!	0.355562	5	0	0	0	0	0
AC008537.2	0.4736842	-1.078003	0.4089303	3	7	9	0	4	5
ENTPD1-AS1	0.9279039	-0.107953	0.7567324	249	239	261	178	239	278
AC008537.3	0.4166667	-1.263034	0.3237423	9	3	12	0	9	1
AC008537.4	0.2	-2.321928	0.2936733	8	0	2	0	0	2
AC079416.1	1	0	0.9952471	6	1	3	0	2	8
SNORA11F	1	0	0.9367583	12	1	4	10	4	3
AL122035.2	0.8	-0.321928	0.7048487	14	27	19	24	13	11
RAB43P1	1.3666667	0.4506614	0.5031817	10	12	8	18	5	18
MIR194-2HG	4	2	0.5223508	1	0	0	1	3	0
AL122035.1	1.4347826	0.5208322	0.5111214	4	10	9	6	9	18
FP325331.1	0.6	-0.736966	0.6434653	3	5	2	3	2	1
AC087359.1	0	#NUM!	0.1278975	1	0	9	0	0	0

DMKN	1.2471042	0.3185821	0.0041075	1128	1209	1548	1632	1701	1512
AC007551.1	#DIV/0!	#DIV/0!	0.6411933	0	0	0	0	0	2
ATP6V1B2	1.036833	0.0521835	0.3858831	3990	4549	4710	3961	5008	4768
ATP6V1B1	0.9311792	-0.102869	0.826446	4885	6391	6829	6075	5822	4962
REP15	6	2.5849625	0.3128068	0	0	1	5	1	0
AL390763.1	0	#NUM!	0.6385096	0	2	0	0	0	0
AC068792.1	#DIV/0!	#DIV/0!	0.1219606	0	0	0	0	6	3
AL138787.1	#DIV/0!	#DIV/0!	0.8030358	0	0	0	0	1	0
AL138787.2	6	2.5849625	0.1583041	0	2	0	7	2	3
SPOCK2	0.48	-1.058894	0.1987248	11	28	11	9	4	11
SPOCK3	2	1	0.6621205	1	3	0	0	0	8
SPOCK1	0.5	-1	0.7087338	3	0	1	0	2	0
SALL4P1	1	0	0.9890559	1	0	0	1	0	0
SALL4P5	#DIV/0!	#DIV/0!	0.6411933	0	0	0	0	0	2
BCDIN3D-AS1	0.8676471	-0.20482	0.7800376	28	14	26	13	24	22
AC110994.2	2.3333333	1.2223924	0.533278	1	2	0	2	3	2
DMP1	2	1	0.6666199	2	0	1	0	5	1
ANKRD20A1	1	0	0.949421	1	0	3	1	1	2
ANKRD20A2	#DIV/0!	#DIV/0!	0.6554062	0	0	0	0	2	0
AC127029.1	0	#NUM!	0.6436235	2	0	0	0	0	0
AC023389.1	0.25	-2	0.6138887	0	0	4	1	0	0
AC023389.2	0	#NUM!	0.6840298	0	0	2	0	0	0
AC020593.1	#DIV/0!	#DIV/0!	0.8030358	0	0	0	1	0	0
AC092807.2	#DIV/0!	#DIV/0!	0.8030358	0	0	0	0	0	1
AC092807.3	1.25	0.3219281	0.6999793	6	6	4	11	6	3
ADPRM	0.9314079	-0.102515	0.9107265	70	79	128	72	106	80
PRR7-AS1	0.8	-0.321928	0.7168921	19	11	15	15	12	9
SEH1L	0.8298878	-0.269012	0.1023033	1087	1010	1289	752	1094	964
TPTE2P5	1	0	0.9922023	2	0	0	0	0	2
TPTE2P6	#DIV/0!	#DIV/0!	0.2488658	0	0	0	3	1	1
TPTE2P3	0.7142857	-0.485427	0.7222842	4	4	13	5	5	5
AL161421.1	0.6428571	-0.63743	0.5043542	15	9	4	4	5	9
TPTE2P4	2.5714286	1.3625701	0.2952905	2	5	0	10	4	4
TPTE2P1	0.5714286	-0.807355	0.3728268	12	8	15	3	12	5
TPTE2P2	2.1724138	1.1192989	0.1023457	14	4	11	12	31	20
DMPK	1.0803167	0.1114544	0.3836776	264	258	362	422	261	272
SNAI3	1.3571429	0.4405726	0.7159155	5	6	3	1	10	8
ATG10-AS1	#DIV/0!	#DIV/0!	0.8030358	0	0	0	0	0	1
ANKRD20A3	0	#NUM!	0.5387425	3	0	0	0	0	0
ANKRD20A4	2	1	0.8186965	0	0	1	0	0	2
SNAI1	1.2553714	0.3281142	0.0157978	449	561	619	698	718	629
SNAI2	1.1	0.1375035	0.9095981	2	3	5	0	5	6
AL133499.1	1	0	0.9813808	3	3	0	0	0	6
YJEFN3	1.0806202	0.1118595	0.4186591	175	143	327	231	271	195
GK-AS1	1.5	0.5849625	0.6611818	2	0	6	2	0	10
PEBP1P2	0	#NUM!	0.824326	0	0	1	0	0	0
TIMP2	1.1127493	0.1541286	0.0862083	4367	5310	5516	5369	5999	5538
TIMP3	2.1690544	1.1170663	2.77E-07	123	122	104	227	265	265
TIMP1	0.6733267	-0.570621	0.0389501	282	375	344	285	204	185
TEAD1	1.0694818	0.0969119	0.4481619	7477	4897	7329	4533	7606	8933
TEAD2	0.8709526	-0.199334	0.348348	627	798	706	577	680	599
TIMP4	1.5030303	0.5878741	0.0487428	48	48	69	94	91	63
TEAD3	1.088478	0.1223123	0.1281546	4549	5090	5992	5707	6029	5278
TEAD4	0.9193495	-0.121315	0.6758105	1749	2110	2167	1906	1911	1723

AC110754.1	#DIV/0!	#DIV/0!	0.8030358	0	0	0	0	0	1
GCHFR	0.6071429	-0.719892	0.5452978	4	6	18	0	10	7
TMSB15A	0.4	-1.321928	0.1488444	11	15	14	1	5	10
TMSB15B	1.5	0.5849625	0.5908587	4	2	4	5	7	3
TMSB15B	1.5	0.5849625	0.5908587	4	2	4	5	7	3
CACTIN	0.7781666	-0.361849	0.0910162	383	526	520	329	364	419
MGLL	0.984619	-0.022362	0.7830275	1193	1470	1563	1322	1419	1420
SOCS2-AS1	0.593985	-0.751502	0.1599505	41	37	55	35	29	15
TIMM9	1.1197691	0.1632013	0.1461091	661	574	844	802	864	662
AL157938.1	0	#NUM!	0.824326	1	0	0	0	0	0
AL157938.2	1.25	0.3219281	0.880716	2	1	1	0	2	3
AL157938.3	0.3333333	-1.584963	0.6210806	0	0	6	0	0	2
GPNMB	1.8142857	0.8594017	0.1237011	35	18	17	19	74	34
AC107222.1	0	#NUM!	0.6436235	2	0	0	0	0	0
DMTN	0.84375	-0.245112	0.7622304	10	19	3	3	8	16
MGMT	1.2348391	0.3043231	0.0370447	857	1214	1161	1509	1313	1169
AP003481.1	0	#NUM!	0.5337954	0	3	0	0	0	0
SLC8B1	0.7743957	-0.368857	0.105165	351	359	407	295	337	233
AC079906.1	0.2222222	-2.169925	0.3044743	2	1	6	0	1	1
SLC8A3	3.3333333	1.7369656	0.3876388	0	3	0	4	0	6
AP002892.2	0	#NUM!	0.3626892	1	0	4	0	0	0
RAB3B	1.0554963	0.0779215	0.4815719	330	285	322	279	341	369
RAB3C	0.6666667	-0.584963	0.5635376	1	22	10	6	9	7
RAB3A	1.544	0.6266728	0.0648769	38	37	50	48	84	61
RAB3D	0.8857143	-0.175087	0.7041422	105	90	120	92	81	106
AC003102.1	0.8252427	-0.27711	0.6481319	33	26	44	26	33	26
IL5RA	0	#NUM!	0.824326	0	0	1	0	0	0
SLC8A1	1.6060606	0.6835263	0.0188216	61	49	88	75	141	102
SLC8A2	0.8441558	-0.244419	0.7796694	20	18	39	23	25	17
RAB43	1.37	0.4541759	0.2582219	34	24	42	46	66	25
RAB44	0	#NUM!	0.6436235	2	0	0	0	0	0
RAB41	1.375	0.4594316	0.5415929	8	9	7	10	15	8
RAB42	0.2631579	-1.925999	0.1925489	7	12	0	0	3	2
RAB2A	0.9770297	-0.033526	0.8088582	2584	2472	3172	2446	2942	2651
TRGV3	0	#NUM!	0.2646708	3	0	3	0	0	0
RAB2B	1.0310284	0.044084	0.4652507	719	658	879	650	866	810
TRGV4	0	#NUM!	0.0526254	3	9	0	0	0	0
TRGV1	0.2857143	-1.807355	0.5833528	0	0	7	2	0	0
AC003092.1	0	#NUM!	0.2245369	2	1	3	0	0	0
CHMP4BP1	3.3636364	1.7500217	0.0742215	1	4	6	22	13	2
TRGV2	0.8571429	-0.222392	0.9684186	0	0	7	5	1	0
TRGV9	0.3857143	-1.374396	0.046142	18	20	32	6	9	12
TRGV8	#DIV/0!	#DIV/0!	0.4737294	0	0	0	0	4	0
TRGV5	3	1.5849625	0.6253252	0	0	1	3	0	0
LBHD1	0.7383721	-0.43758	0.2915388	66	50	56	34	45	48
RAB30	0.7509934	-0.413128	0.0898542	248	261	246	160	177	230
RAB31	0.9184777	-0.122683	0.5413207	2790	2872	3403	2356	2987	2983
RAB36	0.9147727	-0.128515	0.7046779	215	255	234	206	226	212
RAB37	0.4285714	-1.222392	0.5788228	0	7	0	1	2	0
DMWD	0.9221884	-0.116866	0.6915123	476	587	582	452	511	554
EIF4E1B	3.027027	1.5979016	0.003273	16	5	16	36	47	29
RAB34	0.8235294	-0.280108	0.7174308	11	23	17	12	23	7
RAB35	0.8290534	-0.270463	0.0860259	2645	3190	3472	2399	2803	2514
RAB38	1.2861272	0.3630333	0.021859	376	524	484	496	716	568

RAB5B	0.9627818	-0.054719	0.9794602	3559	3668	4219	3168	4108	3744
AL354685.1	#DIV/0!	#DIV/0!	0.8030358	0	0	0	0	0	1
RAB5C	0.9369565	-0.093946	0.8908091	954	1052	1214	1116	1040	861
AC005393.1	1.1923077	0.2537566	0.6546671	6	2	18	13	4	14
RAB6A	1.0346553	0.0491502	0.349708	5352	4621	6215	4730	6238	5781
RAB6B	1.0750354	0.1043842	0.315831	605	750	764	703	890	685
NIFK-AS1	1.0994475	0.1367787	0.4142464	108	119	135	133	136	129
AC092651.1	#DIV/0!	#DIV/0!	0.1541435	0	0	0	0	5	3
AC123788.1	0	#NUM!	0.0723894	2	3	5	0	0	0
RAB4A	0.9213778	-0.118135	0.6527885	788	909	974	763	927	771
RAB4B	0.7586207	-0.398549	0.8035328	9	4	16	14	4	4
P4HA1	1.2040912	0.2679447	0.0167723	2205	1966	2233	2018	2972	2721
P4HA2	0.7294568	-0.455106	0.0006004	2525	2832	3259	1826	2298	2161
P4HA3	1.2857143	0.3625701	0.6697648	5	2	7	8	1	9
RAB5A	0.973535	-0.038695	0.9394487	1652	1358	1751	1231	1719	1685
ADPGK	0.8367185	-0.257186	0.1100387	1122	1237	1432	923	1165	1084
MIR6728	#DIV/0!	#DIV/0!	0.6554062	0	0	0	0	2	0
URAHP	1.6818182	0.7500217	0.3091755	9	5	8	12	9	16
SLC13A3	0.8209046	-0.284713	0.1893142	489	473	674	446	477	420
SLC13A4	1.6793169	0.7478745	3.77E-05	156	157	214	295	312	278
CHPF2	0.7449777	-0.424731	0.0254878	576	596	620	441	417	477
AC012362.1	0.5	-1	0.8289598	1	0	1	0	1	0
AC011773.3	#DIV/0!	#DIV/0!	0.8030358	0	0	0	0	1	0
AC011773.1	0.375	-1.415037	0.4636888	2	5	1	0	3	0
RAB1A	1.0818425	0.1134905	0.1302242	11433	9861	13028	10418	14088	12625
RAB1B	0.9318138	-0.101886	0.7905463	1384	1751	2042	1551	1814	1459
RAB1C	1.375	0.4594316	0.677361	2	1	5	4	4	3
AL033528.2	3.25	1.7004397	0.2941125	0	3	1	9	0	4
AL033528.1	0	#NUM!	0.824326	0	1	0	0	0	0
FBXL20	0.8779612	-0.187771	0.4548325	406	426	561	290	444	489
RAB21	0.9553226	-0.06594	0.895998	2075	1886	2239	1651	2127	2145
AL133353.1	6	2.5849625	0.3657034	1	0	0	0	5	1
RAB20	1.2751705	0.3506901	0.0035879	685	832	976	960	1188	1031
MSTO1	0.7315564	-0.450959	0.0080471	602	711	788	465	564	508
RAB25	1.0631542	0.0883509	0.3224852	1609	2048	2075	2233	2048	1813
RAB26	1	0	0.9979358	2	4	0	2	1	3
RAB23	1.0843938	0.1168888	0.3890314	570	369	554	397	581	641
RAB24	1.0704698	0.0982441	0.5353278	80	91	127	137	75	107
RAB29	0.9855156	-0.021049	0.8413987	521	578	627	481	614	606
RAB28	1.0514648	0.0724005	0.4121498	425	353	485	397	494	437
SLC13A5	0.6666667	-0.584963	0.8914428	2	0	1	2	0	0
AL118505.1	1.3571429	0.4405726	0.454647	14	9	19	11	29	17
AL161911.1	0.8923885	-0.164256	0.581672	225	235	302	174	244	262
RNGTT	0.9972949	-0.003908	0.8056033	810	607	801	547	777	888
AL161911.2	1.4	0.4854268	0.7373827	3	1	1	4	1	2
FBXL19	0.9246154	-0.113075	0.7749784	892	1067	1291	1007	985	1013
FBXL17	1.0049628	0.0071421	0.8700977	246	279	281	169	342	299
FBXL18	0.6985314	-0.517603	0.0045205	1037	1292	1280	589	920	1012
FBXL15	1.4927007	0.5779249	0.0031494	142	180	226	282	309	227
FBXL16	1.1633987	0.2183456	0.215463	184	302	279	246	321	323
FBXL13	0.3636364	-1.459432	0.2595114	4	8	10	2	6	0
FBXL14	0.8407852	-0.250191	0.3179331	255	320	342	205	258	308
FBXL12	0.8278092	-0.27263	0.199622	693	785	996	703	668	677
RAB10	1.058327	0.0817854	0.2440976	5112	4876	5768	4548	6061	6066

RAB14	1.0412383	0.0583003	0.3883128	5722	5342	5959	4750	6565	6410
VAPA	0.997282	-0.003927	0.6204572	4039	3834	4636	3843	4568	4064
PSAT1	1.4057496	0.4913397	1.52E-07	4231	4298	5211	5681	6961	6673
RAB15	0.9777622	-0.032444	0.8675702	1698	1972	2041	1703	1996	1885
VAPB	0.8758078	-0.191314	0.2244513	2856	2957	3471	2401	2857	2873
RAB12	0.8804862	-0.183628	0.4963376	452	450	579	451	472	381
RAB13	0.9212865	-0.118278	0.7087958	711	792	860	741	785	651
RAB18	1.1853806	0.2453503	0.0406517	2493	1728	2414	2145	2882	2838
RAB19	0.7636364	-0.389042	0.686804	25	11	19	24	9	9
RAB17	1.3992689	0.4846732	0.0002072	755	810	897	1198	1218	1029
TNRC6A	0.956044	-0.064851	0.951088	1912	1303	2245	1184	1868	2168
TNRC6B	0.9108527	-0.13471	0.5901031	1227	1013	1372	783	1162	1345
PANK4	1.0798437	0.1108225	0.2322636	542	530	719	568	723	643
PANK2	0.837535	-0.255779	0.1484301	833	1002	1021	698	851	843
PANK3	0.9629817	-0.05442	0.9058466	3079	2181	2628	1741	2758	3097
CALML5	0.5625	-0.830075	0.5245866	5	5	6	0	5	4
VARS	0.8321412	-0.2651	0.1099103	4871	5975	6210	4631	4771	4791
CALML6	1.5833333	0.662965	0.389346	9	6	9	12	21	5
PANK1	1.2912713	0.3687922	0.0290644	307	367	380	319	536	506
MIR548XHG	#DIV/0!	#DIV/0!	0.5371397	0	0	0	0	0	3
CALML3	1.5	0.5849625	0.8233996	1	0	1	0	1	2
CALML4	0.4186047	-1.25634	0.1724304	11	8	24	6	9	3
LONP1	1.0072476	0.0104183	0.5636791	3459	4162	4659	4348	4186	3835
LONP2	0.8377835	-0.255351	0.1027805	1793	1755	1920	1261	1614	1706
AC091729.2	#DIV/0!	#DIV/0!	0.6411933	0	0	0	0	0	2
AC091729.3	0.9333333	-0.099536	0.8992924	36	49	50	34	46	46
SPDYE7P	#DIV/0!	#DIV/0!	0.8030358	0	0	0	0	1	0
AC091729.1	0.7674419	-0.381871	0.7255732	7	14	22	15	7	11
GRK5-IT1	0	#NUM!	0.1789363	2	0	6	0	0	0
ATP6V0E1	1.0167226	0.0239261	0.4924198	6048	7043	7779	7220	7508	6491
XXYLT1-AS2	0	#NUM!	0.6840298	0	0	2	0	0	0
AC093536.1	1	0	0.9774058	0	2	0	0	1	1
TINF2	0.9857602	-0.020691	0.7514255	621	682	874	708	781	657
DNA2	1.1222857	0.16644	0.2646697	968	602	1055	665	1076	1205
ATP6V0D1	0.9709342	-0.042555	0.8928049	3358	3981	4393	3696	4256	3439
ATP6V0D2	#DIV/0!	#DIV/0!	0.4121728	0	0	0	0	1	3
MYL12A	1.0728037	0.1013861	0.1750843	1333	1381	1805	1474	1744	1630
MYL12B	0.9925973	-0.01072	0.649339	5877	5843	7192	6295	6783	5694
SLC2A3P4	0.1666667	-2.584963	0.4002565	0	1	5	0	1	0
PTBP1P	2	1	0.7741804	1	0	0	2	0	0
ATP6V0A2	0.7057701	-0.50273	0.0034638	683	702	712	428	495	557
ATP6V0A4	1.191358	0.252607	0.3951257	39	42	81	44	83	66
TNRC18	0.7534539	-0.408409	0.0031896	10032	11803	12836	7602	8854	9667
AC135352.1	1.25	0.3219281	0.8767903	7	0	1	0	6	4
ATP6V0A1	0.7911253	-0.338022	0.0330771	1185	1256	1638	891	1186	1150
VASP	1.05599	0.0785962	0.2647876	5613	6848	7614	7126	7358	6715
VAT1	1.105983	0.1453292	0.1119568	6643	8368	8606	8598	9039	8483
TINCR	0.7127407	-0.488551	0.001818	2082	2637	2863	1775	1885	1744
VASN	0.8973561	-0.156247	0.6212046	338	430	518	380	398	376
FUBP3	0.9316918	-0.102075	0.6411843	5095	5330	5942	4001	5586	5662
FUBP1	0.9848332	-0.022049	0.8442756	7747	7240	8881	5950	8708	8848
ATP6V1G1P6	0	#NUM!	0.5781912	0	0	3	0	0	0
PANO1	1.6666667	0.7369656	0.5976742	1	1	4	3	0	7
ATP6V1G1P4	2	1	0.807847	1	0	0	0	1	1

COL18A1	1.3963964	0.4817085	0.2697457	36	47	28	55	74	26
DNER	0	#NUM!	0.824326	1	0	0	0	0	0
RAB8A	0.9556434	-0.065456	0.9907302	683	725	869	695	732	749
RALGPS1	0.859434	-0.218541	0.3213041	355	346	359	245	344	322
RAB8B	1.3953975	0.4806761	0.000597	1241	891	1214	1198	1726	1745
RALGPS2	0.9224102	-0.11652	0.6733197	921	650	852	500	827	908
VAV3	1.703259	0.7682978	0.0007234	237	148	198	217	372	404
RAB6C	0.969697	-0.044394	0.9413812	39	18	42	30	24	42
GPBAR1	0.25	-2	0.2828526	5	1	6	2	1	0
RENBP	0.6586227	-0.602476	0.0020811	485	491	723	368	417	334
VAV1	1.1653364	0.2207464	0.1146782	244	266	367	333	375	314
VAV2	0.9302502	-0.104309	0.657822	4396	5002	5111	4112	4750	4635
CDCP1	0.8628	-0.212902	0.2971156	767	862	871	529	755	873
EIF1AXP1	1.0784314	0.1089344	0.4588863	197	135	229	158	222	225
RAB7B	0.3931034	-1.347019	0.0072558	61	41	43	16	14	27
RAB7A	0.880832	-0.183061	0.2449814	11026	12623	13945	9942	12071	11101
FBP2P1	0.8846154	-0.176878	0.9852399	7	2	17	16	6	1
ZNF2	0.9188119	-0.122159	0.8007304	151	134	220	99	166	199
ZNF3	0.7877313	-0.344225	0.0279613	971	954	1156	752	890	785
ZNF8	0.688755	-0.537937	0.0163326	319	316	361	165	230	291
ZNF7	0.8637201	-0.211364	0.3756002	320	358	408	265	393	280
DUOX1	1.0789474	0.1096245	0.7082783	16	22	38	26	32	24
AL359538.1	0	#NUM!	0.3626892	1	0	4	0	0	0
ATP6V0E2	0.9057592	-0.142801	0.684981	362	438	537	416	426	369
DUOX2	1.2	0.2630344	0.8389988	3	0	7	0	8	4
AC084364.1	#DIV/0!	#DIV/0!	0.8030358	0	0	0	0	0	1
RN7SL524P	#DIV/0!	#DIV/0!	0.6482162	0	0	0	0	1	1
PLAC8L1	0.75	-0.415037	0.7654019	5	6	1	0	1	8
VAX2	0.8070175	-0.309328	0.6481488	17	20	20	7	26	13
AC097493.4	1.2857143	0.3625701	0.824973	4	3	0	3	4	2
CAPZA1	1.0977124	0.1345001	0.173774	6611	4937	6812	5264	7714	7176
CAPZA2	0.9949775	-0.007264	0.7533306	2097	1618	2059	1600	2105	2040
SMC2-AS1	1.5	0.5849625	0.3772616	22	2	18	13	22	28
RAB9A	0.9489381	-0.075614	0.9331155	661	743	809	712	717	671
AC097493.3	#DIV/0!	#DIV/0!	0.4737294	0	0	0	0	4	0
RAB9B	0.9467213	-0.078988	0.893565	89	74	81	54	85	92
AL391988.1	0.5454545	-0.874469	0.5687625	1	6	4	1	3	2
NANOS1	0.7886598	-0.342525	0.3877121	69	58	67	34	62	57
NANOS2	#DIV/0!	#DIV/0!	0.3981469	0	0	0	4	0	0
NANOS3	8	3	0.232169	0	0	1	8	0	0
SPDYE16	0.2222222	-2.169925	0.35539	3	0	6	0	2	0
AL121782.1	1	0	0.9716937	0	3	0	3	0	0
SPDYE18	1.8636364	0.8981204	0.0895432	11	17	16	23	26	33
AC241584.1	1.0714286	0.0995357	0.9337976	9	3	2	3	6	6
AC241584.2	0.9677419	-0.047306	0.9748384	23	17	22	19	14	27
AL009031.1	0	#NUM!	0.824326	0	1	0	0	0	0
AP000253.1	1.5	0.5849625	0.627348	4	1	5	6	9	0
TRHDE	0.8196721	-0.286881	0.6966767	19	17	25	3	22	25
CPEB2-AS1	0.6666667	-0.584963	0.9030659	0	0	3	0	0	2
RN7SL521P	0.0714286	-3.807355	0.0543048	6	4	4	1	0	0
AL513331.1	#DIV/0!	#DIV/0!	0.288876	0	0	0	3	2	0
TCEA1P4	0.59375	-0.752072	0.4925106	9	3	20	6	8	5
TCEA1P2	0.7320144	-0.450056	0.1306134	160	195	201	160	131	116
TCEA1P3	#DIV/0!	#DIV/0!	0.8030358	0	0	0	0	1	0

TCEA1P1	5	2.3219281	0.4102632	1	0	0	2	3	0
SPDYE2B	0.8333333	-0.263034	0.9193714	1	1	10	0	4	6
AC100771.1	0	#NUM!	0.824326	0	0	1	0	0	0
AC100771.2	0.35	-1.514573	0.2180747	7	9	4	3	2	2
HAPLN3	0.875	-0.192645	0.8572781	16	7	1	8	6	7
HAPLN1	3.4269572	1.7769282	2.36E-33	450	333	456	1136	1589	1521
AC239868.2	0.5941423	-0.75112	0.0174747	149	150	179	109	67	108
AC239868.3	0.5	-1	0.7689504	0	0	4	0	1	1
RNU6-610P	1.1818182	0.2410081	0.7506588	2	0	9	13	0	0
TMPRSS11A	0.5	-1	0.8188512	2	0	0	0	1	0
TMPRSS11E	0.2727273	-1.874469	0.1484823	8	13	1	2	1	3
TMPRSS11F	#DIV/0!	#DIV/0!	0.6554062	0	0	0	0	2	0
DNM1	1.0823529	0.114171	0.7179951	26	28	31	28	36	28
DNM2	1.0263597	0.0375364	0.4714278	4532	5565	5495	4873	5595	5535
CETN4P	1.3333333	0.4150375	0.7542812	4	4	1	4	6	2
DNM3	0.7333333	-0.447459	0.5163991	16	24	20	15	18	11
DNLZ	#DIV/0!	#DIV/0!	0.3096126	0	0	0	5	0	0
AC022905.1	0	#NUM!	0.824326	1	0	0	0	0	0
FP325330.3	1.25	0.3219281	0.7994963	0	1	3	5	0	0
RNF113A	1.1262077	0.171473	0.1953391	404	574	678	697	629	539
AC073389.3	0.6034483	-0.728698	0.2951648	15	19	24	9	13	13
AC073389.1	0.6888889	-0.537657	0.4062409	11	41	38	8	31	23
AL513477.2	0.4444444	-1.169925	0.5695478	4	1	4	3	0	1
AL513477.1	1.4219653	0.5078863	0.1064759	67	59	47	96	80	70
AC007552.2	0	#NUM!	0.824326	0	0	1	0	0	0
AC133540.1	0.3255814	-1.61891	0.0829884	5	21	17	2	5	7
MRPL48P1	#DIV/0!	#DIV/0!	0.2587603	0	0	0	2	2	1
SNORA3	0.6666667	-0.584963	0.8128492	1	2	0	0	2	0
SNORA3	0.6666667	-0.584963	0.8128492	1	2	0	0	2	0
SNORA3	0.6666667	-0.584963	0.8128492	1	2	0	0	2	0
SNORA3	0.6666667	-0.584963	0.8128492	1	2	0	0	2	0
SNORA9	2	1	0.658	0	0	3	0	0	6
SNORA9	2	1	0.658	0	0	3	0	0	6
SNORA9	2	1	0.658	0	0	3	0	0	6
SNORA9	2	1	0.658	0	0	3	0	0	6
SNORA7	1.6	0.6780719	0.5855421	4	9	2	1	16	7
SNORA7	1.6	0.6780719	0.5855421	4	9	2	1	16	7
SNORA7	1.6	0.6780719	0.5855421	4	9	2	1	16	7
SNORA7	1.6	0.6780719	0.5855421	4	9	2	1	16	7
SNORA7	1.6	0.6780719	0.5855421	4	9	2	1	16	7
AC022893.1	1.9230769	0.9434165	0.1677907	8	6	12	13	11	26
SNORA1	0	#NUM!	0.6385096	0	2	0	0	0	0
SNORA1	0	#NUM!	0.6385096	0	2	0	0	0	0
SNORA1	0	#NUM!	0.6385096	0	2	0	0	0	0
SNORA1	0	#NUM!	0.6385096	0	2	0	0	0	0
SNORA2	1.2121212	0.277534	0.6443922	8	0	25	16	17	7
SNORA2	1.2121212	0.277534	0.6443922	8	0	25	16	17	7
SNORA2	1.2121212	0.277534	0.6443922	8	0	25	16	17	7
AC068790.9	0	#NUM!	0.824326	1	0	0	0	0	0
ARF4-AS1	6	2.5849625	0.3063573	1	0	0	5	0	1
AL080317.1	1.1041667	0.142958	0.4729862	73	59	108	111	88	66
AC068790.1	#DIV/0!	#DIV/0!	0.4737294	0	0	0	0	4	0
AC068790.2	0.6666667	-0.584963	0.6003306	7	11	3	4	2	8
AC068790.3	0.1428571	-2.807355	0.0176291	14	11	10	1	0	4

AC068790.4	0.5	-1	0.8638172	0	0	2	1	0	0
AC068790.5	0.6333333	-0.658963	0.5435011	5	6	19	4	8	7
AC068790.6	0.25	-2	0.15979	2	8	10	0	2	3
GIGYF2	0.9473135	-0.078086	0.7901152	3367	2861	3357	2159	3338	3583
AC068790.7	0.3333333	-1.584963	0.5786506	3	0	3	2	0	0
AL024497.2	0.6666667	-0.584963	0.8829054	3	0	0	2	0	0
AL024507.1	0	#NUM!	0.824326	0	1	0	0	0	0
AL024507.2	1.0729927	0.1016403	0.6427539	48	38	51	67	41	39
SNORD8	#DIV/0!	#DIV/0!	0.8030358	0	0	0	1	0	0
GPR17	0.4285714	-1.222392	0.5909805	4	0	3	0	3	0
SNORD9	2.5	1.3219281	0.532215	0	0	2	2	1	2
SNORD6	0.5192308	-0.945552	0.2305992	19	9	24	9	9	9
AL080317.2	1.125	0.169925	0.6542705	22	13	29	29	30	13
AL080317.3	1.010989	0.0157673	0.8755252	27	16	48	16	39	37
GPR18	0.5	-1	0.5678064	1	1	12	2	3	2
GPR19	0.7653061	-0.385891	0.2324132	114	132	146	105	88	107
GPR27	#DIV/0!	#DIV/0!	0.8030358	0	0	0	0	1	0
AC005740.4	0	#NUM!	0.4460243	4	0	0	0	0	0
AC005740.2	2.6666667	1.4150375	0.3171791	2	3	1	6	0	10
AC005740.3	2	1	0.8324799	1	0	0	0	2	0
GPR22	0	#NUM!	0.6410557	1	1	0	0	0	0
GPR20	0.5	-1	0.8188512	2	0	0	0	1	0
MAFA-AS1	0.8461538	-0.241008	0.8656788	4	7	2	5	5	1
SH3BGRL2	0.8240896	-0.279127	0.078409	1813	1577	1965	1173	1774	1466
SH3BGRL3	1.547619	0.6300504	0.0106343	95	63	136	159	162	134
AC126283.1	1	0	0.9456107	0	0	5	0	1	4
GPR39	0.7647059	-0.387023	0.8947073	8	3	6	13	0	0
GPR37	0.9182796	-0.122995	0.662129	800	914	1076	719	841	1002
SEMA3F-AS1	0.7142857	-0.485427	0.6022256	5	15	15	7	12	6
GPR35	0	#NUM!	0.824326	0	1	0	0	0	0
GPR32	0.8863175	-0.174104	0.4512019	739	922	802	436	864	883
AC026704.1	2	1	0.8324799	1	0	0	0	2	0
AC079907.1	0.8275862	-0.273018	0.8252372	10	5	14	8	13	3
RPS18P12	1.2727273	0.3479233	0.6653206	5	10	7	7	13	8
VN1R1	1.171875	0.2288187	0.6056258	33	12	19	36	22	17
VN1R2	#DIV/0!	#DIV/0!	0.4737294	0	0	0	0	4	0
VN1R5	#DIV/0!	#DIV/0!	0.6099294	0	0	0	2	0	0
AL929601.1	0.5	-1	0.6623132	0	2	6	0	4	0
AL929601.2	2.75	1.4594316	0.325014	1	0	3	6	2	3
AL929601.3	0.6	-0.736966	0.7381678	4	1	0	1	2	0
GPR45	0.225	-2.152003	0.0022201	24	19	37	5	7	6
AL929601.4	0.8478261	-0.23816	0.7406859	22	13	11	8	21	10
SRPX2	0.7142857	-0.485427	0.7000643	5	5	4	0	7	3
MICALL1	0.8946599	-0.160589	0.3949524	4468	5393	5869	4377	4967	4729
MICALL2	1.5786618	0.6587022	0.000906	186	154	213	340	277	256
SRSF10	1.006665	0.0095836	0.6134306	5483	4277	6144	4323	5806	5881
SRSF11	1.2191071	0.2858249	0.0219223	4809	3180	5137	4256	6049	5697
SRSF12	2.5	1.3219281	0.5999264	0	2	0	2	0	3
GPR52	#DIV/0!	#DIV/0!	0.2905913	0	0	0	2	0	3
GPR50	0	#NUM!	0.3482063	0	0	6	0	0	0
AC090159.1	6	2.5849625	0.3854518	1	0	0	0	0	6
IGKJ3	0	#NUM!	0.5643268	1	0	2	0	0	0
IGKJ4	#DIV/0!	#DIV/0!	0.6554062	0	0	0	0	2	0
IGKJ1	2.2580645	1.1750867	0.0589275	11	6	14	14	31	25

IGKJ5	0	#NUM!	0.824326	1	0	0	0	0	0
AC090503.2	0	#NUM!	0.6436235	2	0	0	0	0	0
GPR68	0.8	-0.321928	0.8894814	0	2	8	1	6	1
GPR63	0.5126984	-0.963818	0.0006022	237	226	167	92	96	135
GPR61	1.0967742	0.1332665	0.762442	6	8	17	13	13	8
AL359385.1	1.5	0.5849625	0.8194094	0	0	2	0	0	3
SLC9C1	0.5	-1	0.4247239	11	2	5	1	5	3
SLC9C2	1.2564103	0.3293076	0.6510835	9	18	12	5	22	22
CARD8-AS1	0.8947368	-0.160465	0.913922	10	3	6	4	4	9
AC108448.2	#DIV/0!	#DIV/0!	0.6099294	0	0	0	2	0	0
AC092650.1	5	2.3219281	0.4088931	0	1	0	5	0	0
GPR78	0.7601351	-0.395672	0.0470978	388	371	425	265	299	336
AL512506.1	2.25	1.169925	0.5166203	2	2	0	4	0	5
GPR75	0.8086124	-0.30648	0.5144747	78	47	84	28	55	86
AL512506.2	#DIV/0!	#DIV/0!	0.6554062	0	0	0	0	2	0
SLC9B1	1.5714286	0.6520767	0.725312	4	3	0	0	3	8
SLC9A2	2.4285714	1.2801079	0.1526263	8	2	4	9	12	13
SLC9A3	0.5	-1	0.4900166	4	6	2	0	4	2
SLC9A4	2	1	0.5260495	7	0	2	0	13	5
SLC9A5	1.1428571	0.1926451	0.6194849	22	27	28	23	26	39
SLC9A6	0.9473336	-0.078056	0.8202468	1063	890	1066	655	1020	1185
SLC9A7	0.93241	-0.100964	0.7269179	594	562	649	453	604	626
SLC9A8	0.789206	-0.341526	0.029073	1185	1023	1331	767	1059	967
SLC9A9	0.3636364	-1.459432	0.3316054	2	7	13	6	0	2
GPR87	0.4556738	-1.133927	3.48E-05	186	161	217	98	84	75
GPR85	0.2068966	-2.273018	0.0649043	9	14	6	1	0	5
GPR83	1.4545455	0.5405684	0.6668487	3	1	7	0	9	7
GPR84	2	1	0.7741804	1	0	0	2	0	0
AC120024.1	0.1666667	-2.584963	0.3136028	1	4	1	1	0	0
GPR82	4	2	0.5412414	0	0	1	0	3	1
SLC9A1	0.9782084	-0.031786	0.8400275	3415	4221	4387	3672	4084	4005
AC053513.1	0.9411765	-0.087463	0.963447	6	3	8	1	7	8
PRKAG2-AS1	2.1111111	1.0780025	0.3952829	4	5	0	5	7	7
NFKB1	0.9846154	-0.022368	0.8051456	672	757	846	697	829	714
NFKB2	1.0681284	0.0950851	0.2455674	741	808	1005	881	972	875
NFE2L3	1.8523123	0.8893273	3.51E-16	2652	2524	2868	4090	5544	5266
NFE2L1	0.9599769	-0.058928	0.9666343	2504	2965	3201	2420	2920	2983
NFE2L2	1.1546899	0.2075055	0.0841063	1602	1081	1784	1447	2030	1681
AC126773.4	2.25	1.169925	0.4748536	0	0	4	3	4	2
AC126773.2	0.4782609	-1.06413	0.4213949	10	2	11	7	2	2
AC126773.3	0	#NUM!	0.6436235	2	0	0	0	0	0
AL049641.1	0.6666667	-0.584963	0.8754808	0	3	0	2	0	0
AC126773.1	#DIV/0!	#DIV/0!	0.8030358	0	0	0	0	1	0
VBP1	1.0975104	0.1342346	0.1984787	1048	796	1048	882	1168	1124
AC012615.6	1.0666667	0.0931094	0.7876561	31	24	20	27	16	37
AC012615.4	0.7619048	-0.392317	0.7623608	3	8	10	0	4	12
GIGYF1	0.9746193	-0.037089	0.8004052	2389	2315	3176	2361	2689	2630
AC012615.1	0.8035714	-0.315502	0.6887607	9	26	21	11	29	5
GTF3C1	0.8027258	-0.317021	0.0401744	2974	3707	3518	2283	3005	2899
GTF3C2	0.9902318	-0.014162	0.7450231	1969	2407	2483	2115	2396	2281
GTF3C3	1.0131699	0.0188762	0.6548147	1932	1430	2105	1374	2174	1991
GTF3C4	0.9727861	-0.039805	0.9940736	2858	3053	3202	2160	3280	3425
GTF3C5	1.0582615	0.0816962	0.2452548	2827	3235	3567	3337	3595	3258
GTF3C6	1.0501995	0.0706634	0.4191357	906	960	1142	980	1320	859

EIF4EP1	0.8	-0.321928	0.9603181	3	0	2	4	0	0
EIF4EP2	1.0663717	0.0927104	0.4348514	271	275	358	245	386	333
AC024560.3	0.5	-1	0.5741211	3	1	6	0	3	2
MICALCL	0.7561983	-0.403163	0.3236502	80	70	92	35	55	93
AC024560.2	0.72	-0.473931	0.5363388	21	16	13	15	11	10
SLC12A2	0.8395396	-0.25233	0.2663829	517	400	560	300	444	496
SLC12A3	0.4400318	-1.18432	1.56E-09	346	418	495	165	212	177
SLC12A4	0.7552931	-0.404891	0.004117	2724	3146	3529	2227	2475	2397
SLC12A5	0.4117647	-1.280108	0.3737168	10	4	3	0	7	0
SEC62-AS1	0	#NUM!	0.6631647	1	0	1	0	0	0
CYP2A7P2	0	#NUM!	0.824326	1	0	0	0	0	0
SLC12A6	0.9343066	-0.098032	0.7963676	294	291	374	188	335	373
SLC12A7	0.8794034	-0.185403	0.3433891	919	1089	1143	835	955	981
SLC12A8	0.4677419	-1.096215	0.1370874	23	16	23	14	7	8
SLC12A9	0.9218999	-0.117318	0.6832178	3588	4183	4777	3867	4155	3546
PAQR8	1.1298077	0.1760772	0.3906512	145	108	163	110	210	150
PAQR6	0.7320574	-0.449971	0.3235364	66	48	95	56	60	37
PAQR7	0.8615876	-0.214931	0.3272783	707	858	942	733	735	692
ANTXRLP1	#DIV/0!	#DIV/0!	0.6554062	0	0	0	0	2	0
PAQR4	0.8583433	-0.220373	0.5296468	226	314	293	289	229	197
PAQR5	0.6938776	-0.527247	0.5072215	18	13	18	8	7	19
PAQR3	1.2009646	0.2641937	0.0939391	900	603	985	675	1099	1214
Z94160.1	0.125	-3	0.0867203	1	11	4	0	1	1
AF146191.1	0	#NUM!	0.4521054	1	0	3	0	0	0
AC016727.1	0.7545455	-0.40632	0.5597285	21	28	61	8	27	48
AL807752.4	#DIV/0!	#DIV/0!	0.6099294	0	0	0	2	0	0
AL807752.5	1.0540541	0.0759489	0.8185386	7	9	21	13	18	8
AL807752.1	1.625	0.7004397	0.4112191	1	6	9	14	5	7
AC015983.2	0	#NUM!	0.6436235	2	0	0	0	0	0
SNORA38B	0.3333333	-1.584963	0.6833782	1	0	2	0	0	1
AC015983.1	3	1.5849625	0.4940935	1	0	1	0	1	5
MIA3	1.0087735	0.0126023	0.7001399	2059	1311	2101	1405	2145	1969
CFL1P8	#DIV/0!	#DIV/0!	0.8030358	0	0	0	1	0	0
CFL1P3	#DIV/0!	#DIV/0!	0.8030358	0	0	0	1	0	0
CFL1P1	1.6666667	0.7369656	0.2092232	11	17	14	17	16	37
CFL1P4	0	#NUM!	0.824326	0	1	0	0	0	0
CFL1P5	0.5	-1	0.2600416	12	9	17	6	6	7
AC090502.1	0.4545455	-1.137504	0.5177804	1	4	6	0	1	4
AC090502.3	#DIV/0!	#DIV/0!	0.8030358	0	0	0	0	1	0
AC106875.1	0.52	-0.943416	0.2808916	15	8	27	12	8	6
COL19A1	0.4	-1.321928	0.5091578	0	8	2	0	0	4
MICD	0	#NUM!	0.5387425	3	0	0	0	0	0
MICE	0.6071429	-0.719892	0.079532	76	54	66	37	33	49
MICA	0.90625	-0.142019	0.9360395	5	12	15	10	12	7
MICB	0.8	-0.321928	0.337023	125	203	157	136	110	142
AL139039.2	0.25	-2	0.5155155	1	3	0	0	1	0
AL139039.3	0.4444444	-1.169925	0.5603255	1	4	4	3	1	0
AL139039.1	0	#NUM!	0.6436235	2	0	0	0	0	0
ERCC6L2	1.1096319	0.1500812	0.3360778	460	309	508	313	534	570
AC130650.1	2	1	0.8324799	1	0	0	0	2	0
MIB2	1.1013431	0.139264	0.2305955	663	868	926	1002	939	765
AC130650.2	0.6346154	-0.656046	0.4908288	21	3	28	8	7	18
DOHH	0.9598174	-0.059168	0.9833478	283	423	389	350	402	299
MIAT	1.8571429	0.8930848	0.5079601	1	2	4	3	9	1

AC107952.2	0.6666667	-0.584963	0.8914428	2	0	1	2	0	0
MIB1	1.3148467	0.3948946	0.0482741	1214	625	1064	795	1439	1583
AC138932.1	0.8101852	-0.303676	0.4744804	84	49	83	38	64	73
AC138932.2	0.6451613	-0.632268	0.5871162	11	9	11	6	0	14
AC138932.3	1.4285714	0.5145732	0.6707045	0	2	5	4	3	3
AC138932.5	1.8333333	0.8744691	0.6029233	1	2	3	1	10	0
AL031320.2	0.4166667	-1.263034	0.4366041	9	2	1	3	1	1
MIDN	1.1488927	0.2002441	0.0380972	2716	3166	3420	3514	3792	3381
AL096828.3	1.1333333	0.1805722	0.7770213	3	4	8	9	5	3
RN7SL535P	0.7142857	-0.485427	0.8215964	2	2	3	0	0	5
AL136100.1	0	#NUM!	0.6840298	0	0	2	0	0	0
AC090888.3	#DIV/0!	#DIV/0!	0.8030358	0	0	0	0	1	0
MICG	0.4666667	-1.099536	0.1864415	7	15	38	7	14	7
MID1	1.0291221	0.0414141	0.5788094	811	638	886	589	903	911
DOK1	0.9534884	-0.068713	0.9903645	96	84	121	92	109	86
DOK2	0.6666667	-0.584963	0.8056378	6	2	4	8	0	0
DOK3	1.1971831	0.2596438	0.2851159	76	59	78	87	83	85
DOK4	0.7490218	-0.41692	0.0298137	512	613	664	448	483	409
DOK5	#DIV/0!	#DIV/0!	0.3109758	0	0	0	0	6	0
DOK6	#DIV/0!	#DIV/0!	0.4501902	0	0	0	0	0	4
DOK7	2.5263158	1.337035	0.0861779	5	3	11	32	7	9
TMED10	1.0380484	0.0538736	0.3707608	13232	13336	14669	11658	15828	15320
SNORA36C	#DIV/0!	#DIV/0!	0.6411933	0	0	0	0	0	2
SEL1L	0.9443863	-0.082551	0.8130746	3953	2653	3913	2262	3736	3936
MIG7	0.5	-1	0.6368154	0	6	2	0	0	4
PSEN2	0.7049632	-0.50438	0.0126903	329	333	426	235	255	277
PSEN1	1.021148	0.030192	0.5726158	2758	2855	2993	2311	3132	3345
AL138785.1	#DIV/0!	#DIV/0!	0.6099294	0	0	0	2	0	0
AC011287.1	0.8709677	-0.199309	0.908485	10	3	18	8	11	8
AC011287.2	0.4864865	-1.039528	0.2764784	8	9	20	3	5	10
PRSS29P	0.2941176	-1.765535	0.119358	4	19	11	2	8	0
TTC3-AS1	0.6666667	-0.584963	0.8466488	3	0	0	0	0	2
AP000350.5	#DIV/0!	#DIV/0!	0.8030358	0	0	0	0	0	1
AP000350.6	0.5302594	-0.91523	0.0144535	136	100	111	24	78	82
AP000350.7	1	0	0.9890559	0	0	1	0	1	0
DOLK	1.0155895	0.0223174	0.5600101	939	1025	1115	1056	1125	946
AP000350.2	1	0	0.927665	3	0	6	3	2	4
AL356986.1	#DIV/0!	#DIV/0!	0.4501902	0	0	0	0	0	4
AL031716.1	1.2941176	0.3719688	0.8300577	13	4	0	0	12	10
AC241585.1	0.6027397	-0.730393	0.2459651	16	36	21	9	18	17
AC241585.2	1.1190476	0.1622714	0.5092249	46	56	66	81	62	45
AC241585.3	0.902834	-0.147467	0.7875261	54	106	87	72	69	82
AC241585.4	0.3333333	-1.584963	0.5297083	0	3	6	3	0	0
ADNP-AS1	0.8870968	-0.172837	0.7929353	20	24	18	9	21	25
SNED1	0.5409836	-0.886343	0.2168415	13	34	14	8	9	16
AC099066.1	4.5	2.169925	0.2858319	1	0	1	1	7	1
AC099066.3	0	#NUM!	0.824326	1	0	0	0	0	0
B4GALT1-AS1	0.6666667	-0.584963	0.5601706	14	7	9	8	10	2
AP000590.1	#DIV/0!	#DIV/0!	0.5509701	0	0	0	0	3	0
AL691447.3	1.0714286	0.0995357	0.9250064	2	8	4	4	9	2
AC139272.1	0.7285714	-0.456858	0.4963351	40	14	16	13	15	23
LMBRD1	0.9827774	-0.025063	0.8332185	1548	1374	1665	1283	1684	1541
VCAN	1.2	0.2630344	0.6994394	29	3	13	11	22	21
LMBRD2	1.0871254	0.1205184	0.4660101	783	383	636	462	693	804

RNU6-621P	0	#NUM!	0.4324683	2	0	2	0	0	0
RN7SL530P	0	#NUM!	0.824326	1	0	0	0	0	0
AC068946.1	2.6666667	1.4150375	0.4349683	2	1	0	4	2	2
POLHP1	#DIV/0!	#DIV/0!	0.8030358	0	0	0	0	0	1
AC105235.1	7	2.8073549	0.2798517	0	0	1	7	0	0
MBTPS1	0.8274001	-0.273343	0.0528516	3631	3695	4163	2644	3375	3487
MIIP	1.1407907	0.1900342	0.0987305	1391	1310	1447	1749	1610	1373
MAPK14	0.9423792	-0.08562	0.7483942	2370	2461	2701	1930	2595	2573
MAPK15	1.3125	0.3923174	0.5901995	19	3	10	9	16	17
MAPK12	1.1427343	0.19249	0.1628131	342	351	470	526	417	386
MAPK13	0.4461538	-1.164387	0.1277454	32	7	26	7	15	7
MAPK10	2.7333333	1.4506614	0.0139783	8	8	14	36	29	17
MAPK11	1.1904762	0.2515388	0.6617731	10	21	11	31	15	4
CHPT1	0.76	-0.395929	0.0106768	1258	1453	1414	955	1139	1041
RNU6-623P	0.5	-1	0.6694314	6	0	0	0	3	0
MBTPS2	0.99908	-0.001328	0.9057698	444	324	319	253	378	455
SERPINE2	0.4454776	-1.166575	3.29E-09	377	364	442	133	194	200
SERPINE3	0.375	-1.415037	0.5368174	3	0	5	0	3	0
SERPINE1	1.5460849	0.6286196	9.14E-08	1037	1163	1261	1638	2018	1695
AL391840.1	1	0	0.9621224	1	0	1	1	0	1
DUS4L	0.9728774	-0.03967	0.9277771	279	224	345	219	321	285
RNU6-642P	0	#NUM!	0.6385096	0	2	0	0	0	0
TRIQK	1.1665072	0.2221952	0.1795102	413	261	371	330	503	386
SERPINF1	1.4452998	0.5313688	3.61E-05	751	817	900	1171	1338	1058
SERPINF2	1.1666667	0.2223924	0.8835593	1	2	3	0	2	5
AC141002.1	1	0	0.9969191	2	3	3	0	3	5
SERPING1	0.8968368	-0.157083	0.5392351	814	927	1041	859	881	755
YME1L1	1.2442625	0.3152909	0.0053688	6466	4837	6649	6017	8138	8182
DUS3L	0.8540618	-0.227588	0.3050458	408	459	524	384	416	388
FP325332.1	1.125	0.169925	0.8814551	1	0	7	0	6	3
AL133355.1	0.6470588	-0.628031	0.6198808	9	2	6	1	4	6
AP003385.5	0	#NUM!	0.6385096	0	2	0	0	0	0
FAM160B1	1.0115023	0.0164996	0.7660851	1097	795	977	673	1008	1221
FAM160B2	0.6816269	-0.552946	0.0005123	866	908	1078	625	728	591
RAD21	1.0063037	0.0090658	0.6938155	4544	3288	4383	3227	4580	4485
SERPINH1	0.8140739	-0.296768	0.1107684	2046	2454	2648	2017	2131	1671
SIVA1	1.0969916	0.1335525	0.1835732	1724	2120	2239	2439	2207	2027
TRIP4	1.0558952	0.0784666	0.3289488	722	595	973	713	873	832
FAM160A1	0.9270588	-0.109267	0.7283268	264	291	295	161	299	328
FAM160A2	1.3378886	0.419958	0.0006987	819	883	1225	1329	1332	1255
THUMPD3P1	1	0	0.9890559	0	0	1	0	0	1
TRIP6	0.8694834	-0.20177	0.3512967	3288	3921	4483	3601	3448	3117
RAD17	1.2137291	0.2794465	0.0404767	972	655	1068	856	1262	1153
DUS1L	0.9210437	-0.118659	0.7811845	1641	2051	2172	2049	1936	1416
SERPINI1	1.3861386	0.4710715	0.260905	24	17	60	33	71	36
RAD18	0.9813084	-0.027221	0.9037737	948	575	938	605	938	872
SRP54	1.1625691	0.2173164	0.0291365	4335	3286	4866	4292	5481	4744
SERPINA6	0	#NUM!	0.824326	0	0	1	0	0	0
SERPINA5	0.6103286	-0.712342	0.086032	45	78	90	32	43	55
FCRLA	#DIV/0!	#DIV/0!	0.6411933	0	0	0	0	0	2
TRIM8	1.2679918	0.3425454	0.025799	389	551	519	583	713	554
FCRLB	0.3108108	-1.685891	0.0214908	16	17	41	4	10	9
TRIM9	1.0873181	0.1207741	0.4476762	127	144	210	135	167	221
C14orf177	#DIV/0!	#DIV/0!	0.3096126	0	0	0	5	0	0

TRIM6	0.9972214	-0.004014	0.6956004	1107	952	1180	1011	1212	1007
TRIM7	0.6363636	-0.652077	0.5840655	13	5	4	5	1	8
TRIM4	0.9874214	-0.018262	0.7949109	229	127	280	208	210	210
TRIM5	0.8899043	-0.168278	0.4355115	730	659	909	541	774	730
TRIM2	0.7517241	-0.411725	0.2127719	174	106	155	57	143	127
TRIM3	0.8747346	-0.193083	0.4285547	312	280	350	236	308	280
RACK1	0.9881929	-0.017135	0.7128529	69238	82042	90608	82487	85439	71106
AC021086.1	0.8461538	-0.241008	0.841176	9	2	2	0	5	6
SERPINB3	0	#NUM!	0.5337954	0	3	0	0	0	0
MIOX	14	3.8073549	0.0625043	0	0	1	8	0	6
SERPINB1	0.9257306	-0.111336	0.7198805	2067	1971	2600	2013	2193	1939
SERPINB2	1.4761905	0.5618789	0.4377012	7	4	10	13	7	11
FCRL5	0	#NUM!	0.824326	1	0	0	0	0	0
MIOS	1.0599078	0.0839388	0.4396239	1356	796	1320	1008	1490	1182
FCRL3	0	#NUM!	0.6385096	0	2	0	0	0	0
ITPK1	0.9746377	-0.037062	0.8948243	2450	2947	2883	2584	2828	2658
SERPINB9	1.2804005	0.3565952	9.54E-05	12998	13581	15468	15267	19943	18627
SERPINB7	0.6666667	-0.584963	0.7095543	0	9	3	3	4	1
SERPINB8	1.5714286	0.6520767	0.2532062	13	23	13	16	29	32
SERPINB5	0	#NUM!	0.6436235	2	0	0	0	0	0
SERPINB6	1.6712088	0.740892	9.00E-07	642	797	836	1383	1320	1099
ITPKB	1.625	0.7004397	0.3100764	6	8	10	17	12	10
ITPKC	0.9886986	-0.016397	0.7293947	816	981	1123	1054	986	847
ITPKA	1.8488372	0.8866182	0.0156528	38	89	45	134	103	81
SERPINC1	1.5846154	0.6641327	0.1720961	29	8	28	30	40	33
SLC22A20P	0.5068493	-0.980371	0.1637093	23	11	39	10	15	12
AC009268.2	0	#NUM!	0.4374838	0	4	0	0	0	0
CHRNG	0.4285714	-1.222392	0.5545532	4	3	0	0	3	0
SRP72	1.1892968	0.2501089	0.0468269	4296	2808	4444	3583	5262	4889
CHRNE	1.2962963	0.3743955	0.5712834	8	2	17	11	5	19
QTRT1P1	0	#NUM!	0.255947	4	0	2	0	0	0
MISP	0.5722222	-0.805353	0.0582864	45	73	62	23	37	43
SERPIND1	0.6363636	-0.652077	0.7286196	4	0	7	0	3	4
C14orf180	0.6521739	-0.616671	0.4269076	18	17	11	12	9	9
GTF2H1	1.0319005	0.0453039	0.4169375	2577	1927	2894	2239	2715	2680
GTF2H2	0.9377365	-0.092746	0.8046472	1113	730	1064	773	1013	940
SRP68	0.8762049	-0.19066	0.2171644	5013	5350	6132	4438	5171	4844
GTF2H3	0.8506534	-0.233357	0.1989659	1099	881	1234	767	1011	956
GTF2H4	#DIV/0!	#DIV/0!	0.5371397	0	0	0	0	0	3
GTF2H5	1.0797546	0.1107035	0.2764009	622	662	835	764	895	629
MIR4527HG	#DIV/0!	#DIV/0!	0.8030358	0	0	0	1	0	0
SEMA5A	1.439047	0.5251137	0.0010307	1142	1023	1067	1047	1735	1869
AC084851.3	#DIV/0!	#DIV/0!	0.8030358	0	0	0	1	0	0
CHRM3	0.8925687	-0.163965	0.5057511	372	402	464	308	386	411
SEMA5B	0.25	-2	0.3800775	6	0	2	1	0	1
CHRM1	#DIV/0!	#DIV/0!	0.8030358	0	0	0	0	1	0
AC025470.2	#DIV/0!	#DIV/0!	0.2116454	0	0	0	5	1	0
CHRM4	0.9298246	-0.10497	0.9220164	12	22	23	10	18	25
CHRM5	1.5517241	0.6338721	0.2097538	21	12	25	44	12	34
FABP12	1	0	0.9890559	0	0	1	1	0	0
TECTA	0.7777778	-0.36257	0.7502632	11	5	20	10	13	5
SEMA6B	0.3984375	-1.327575	0.0091817	42	41	45	17	13	21
TECTB	1	0	0.9904495	3	0	0	0	0	3
SEMA6C	1.1650943	0.2204468	0.1873986	207	223	206	218	274	249

SEMA6A	1.2605798	0.3340874	0.0019083	4084	3810	4559	4093	5670	5935
SEMA6D	1.9274194	0.9466705	0.0002148	101	68	79	135	167	176
MITF	1.4692797	0.555109	0.0016749	324	264	356	318	558	511
GTF2F1	0.9331989	-0.099743	0.7287343	2087	2565	2788	2103	2581	2259
GTF2F2	1.1551909	0.2081312	0.0714202	941	697	1165	1039	1189	1010
AC025470.1	#DIV/0!	#DIV/0!	0.1402064	0	0	0	0	10	0
X1B-SULT1A	0.1666667	-2.584963	0.1598694	8	2	2	0	1	1
SEMA7A	1.1499653	0.2015903	0.0371135	2521	3005	3116	2982	3475	3481
ITPR1	0.8502415	-0.234055	0.4363504	172	239	210	92	220	216
RHOBTB3	1.2	0.2630344	0.5269876	43	23	24	35	25	48
ITPR2	1.1619601	0.2165606	0.2300503	491	320	393	330	490	579
ITPR3	3.962963	1.9865795	0.0009502	11	10	6	20	49	38
GTF2E1	0.9027119	-0.147663	0.4772678	1020	892	1038	743	938	982
GTF2E2	1.0423313	0.0598139	0.4280688	536	491	603	561	616	522
RHOBTB1	3.875	1.9541963	1.16E-12	45	46	61	162	268	159
RHOBTB2	0.5833333	-0.777608	0.0085381	110	167	167	84	97	78
AC009518.2	#DIV/0!	#DIV/0!	0.8030358	0	0	0	1	0	0
NSFL1C	1.0819672	0.1136568	0.1916011	2686	3339	3735	3426	3940	3194
AC108051.1	#DIV/0!	#DIV/0!	0.8030358	0	0	0	0	0	1
RAD9B	0.52	-0.943416	0.4347273	9	5	11	8	2	3
RAD9A	0.7644578	-0.387491	0.0375928	515	476	669	394	456	419
LMBR1L	0.9230541	-0.115513	0.6845971	969	1089	1308	969	1144	994
HYDIN	1.0666667	0.0931094	0.7718387	46	29	45	23	56	49
ALMS1P1	#DIV/0!	#DIV/0!	0.3096126	0	0	0	5	0	0
PAPD4	1.1329949	0.1801414	0.1391976	1049	687	1219	939	1170	1239
NIPSNAP1	0.6659529	-0.586508	0.0011693	546	612	710	418	449	377
NIPSNAP2	0.8690537	-0.202483	0.451032	645	478	832	545	599	555
BTBD7P1	8	3	0.2977675	0	0	1	0	8	0
PTP4A1	0.8770608	-0.189251	0.2073637	12235	11454	13614	8721	12288	11708
PTP4A2	1.0274845	0.0391166	0.3743859	3855	3588	4491	3681	4568	4013
PTP4A3	0.3529412	-1.5025	0.2739262	7	5	5	0	2	4
PAPD7	0.980954	-0.027743	0.7996841	1821	1698	2204	1610	2040	1964
PAPD5	1.1098858	0.1504112	0.3056748	991	623	925	650	1110	1058
GTF2A1	0.9755985	-0.035641	0.9835287	1618	1207	1519	876	1624	1738
GTF2A2	1.0323516	0.0459344	0.3744796	2519	2806	3299	2958	3161	2784
AC006378.1	0.8846154	-0.176878	0.8844558	11	8	7	9	13	1
AC006378.2	0.875	-0.192645	0.9182265	0	6	2	2	2	3
SEMA3C	0.553719	-0.852774	0.0864066	47	44	30	14	30	23
SEMA3D	3.5	1.8073549	0.0001228	10	13	23	35	56	70
SEMA3A	1.4591281	0.5451065	0.026695	313	133	288	233	400	438
SEMA3B	0.7647059	-0.387023	0.7245055	6	13	15	11	13	2
SEMA3G	1.5	0.5849625	0.8252133	4	0	0	0	4	2
SEMA3E	0.6730769	-0.571157	0.3000607	49	22	33	20	23	27
SEMA3F	0.591716	-0.757023	0.0886944	43	49	77	33	36	31
DPCD	1.2806268	0.3568501	0.0730035	176	248	278	295	386	218
EEF2KMT	0.7626614	-0.390885	0.0968948	308	321	378	280	250	238
PLA2G12AP1	0.5294118	-0.917538	0.3611339	9	17	8	5	1	12
SEMA4A	0.6666667	-0.584963	0.8411882	3	0	3	3	1	0
SEMA4D	1.0431484	0.0609444	0.4221934	1244	1314	1660	1128	1532	1740
SEMA4B	0.7395316	-0.435316	0.008698	1171	1419	1637	1027	1051	1048
ADRM1	0.9593091	-0.059932	0.9293121	987	1089	1340	1127	1033	1117
SEMA4C	0.9588505	-0.060622	0.9717211	8741	10213	11180	8781	10247	9866
MIR181A2HG	1.8888889	0.9175378	0.0113893	22	36	50	80	67	57
SEMA4F	0.7160494	-0.481869	0.0172316	392	509	557	328	404	312

SEMA4G	1.4727273	0.5584903	0.0652096	44	64	57	69	84	90
TRIT1	1.2555106	0.3282742	0.010033	742	595	886	865	1093	833
RAD52	1.0757576	0.105353	0.4440793	251	206	335	199	330	323
PTP4A2P1	0.6666667	-0.584963	0.5532809	9	8	10	4	11	3
RAD50	1.1483871	0.199609	0.5747972	63	26	66	29	75	74
PTP4A2P2	#DIV/0!	#DIV/0!	0.6554062	0	0	0	0	2	0
RAD51	0.850927	-0.232893	0.1901244	1585	1743	1958	1496	1537	1465
MIR1972-2	0	#NUM!	0.824326	0	0	1	0	0	0
DPF1	1.2519685	0.3241983	0.121561	116	133	132	178	140	159
DPF2	0.8929549	-0.163341	0.4339879	1636	2064	2148	1655	1821	1746
DPF3	1.5721567	0.652745	9.33E-09	1211	1060	1457	1650	2157	2054
AC098479.1	0	#NUM!	0.5337954	0	3	0	0	0	0
AL355032.1	1.6875	0.7548875	0.409051	4	6	6	6	15	6
HIST1H2BPS2	#DIV/0!	#DIV/0!	0.6099294	0	0	0	2	0	0
TIPIN	1.1516393	0.203689	0.0635271	725	650	821	702	969	858
AL138880.1	0	#NUM!	0.6436235	2	0	0	0	0	0
AL138880.2	#DIV/0!	#DIV/0!	0.6411933	0	0	0	0	0	2
SLC11A1	1.5925926	0.6713773	0.4307679	13	13	1	23	5	15
SLC11A2	0.8771259	-0.189144	0.244605	4794	4479	5368	3313	4744	4785
MIR181A1HG	0	#NUM!	0.3963337	1	3	0	0	0	0
TCERG1L	1.2194245	0.2862004	0.2450313	91	81	106	81	106	152
PAPPA	0.4444444	-1.169925	0.567416	6	0	3	1	0	3
WDPCP	1.0055249	0.0079488	0.7921737	121	103	138	108	138	118
AL078587.1	1.3870968	0.4720684	0.4516724	11	6	14	17	6	20
AC135506.1	0.7272727	-0.459432	0.5707564	7	20	28	8	23	9
DPH1	0.7590157	-0.397798	0.0173245	727	822	808	517	676	596
DPH2	0.9179375	-0.123532	0.7192397	792	873	1089	865	895	768
DPH3	1.0175219	0.0250598	0.519177	781	680	936	787	876	776
DPH5	1.0240515	0.0342883	0.5019332	932	983	1037	967	1022	1034
NAALAD2	0.9573092	-0.062943	0.9731947	557	335	654	421	507	552
DPH6	0.8030708	-0.316401	0.1233603	484	416	598	377	455	371
DPH7	1.0328515	0.0466329	0.4539818	641	702	940	756	900	702
SRPK2	0.9281484	-0.107573	0.678461	938	720	875	574	893	884
SRPK3	2	1	0.1850991	10	8	5	12	22	12
CR383656.5	0.7222222	-0.469485	0.7226115	9	1	8	1	5	7
CR383656.2	1.1111111	0.1520031	0.6333491	24	24	33	31	27	32
CR383656.3	0	#NUM!	0.5387425	3	0	0	0	0	0
CR383656.1	#DIV/0!	#DIV/0!	0.6317974	0	0	0	1	1	0
SRPK1	0.8532889	-0.228894	0.1894491	3036	2488	3187	1823	2793	2817
NODAL	4	2	0.3524358	2	0	0	7	0	1
AC007216.1	0	#NUM!	0.824326	0	0	1	0	0	0
AC007216.2	4	2	0.2218438	1	0	2	2	3	7
U4atac	#DIV/0!	#DIV/0!	0.5371397	0	0	0	0	0	3
AC007216.3	0.8571429	-0.222392	0.9650284	0	1	13	3	7	2
AC007216.4	1.5833333	0.662965	0.535285	1	6	5	17	2	0
RNU6-1203P	0.3333333	-1.584963	0.6715904	3	0	0	0	1	0
AP000255.1	0.1875	-2.415037	0.1263264	6	4	6	0	1	2
AC023481.1	0	#NUM!	0.824326	0	0	1	0	0	0
PAPLN	1.0909091	0.1255309	0.5881179	72	107	96	70	103	127
AC004817.2	2.6666667	1.4150375	0.4771236	1	0	2	0	4	4
AC004817.3	2.3333333	1.2223924	0.2552511	1	2	6	9	9	3
FKBP4P6	3	1.5849625	0.6684577	1	0	0	0	0	3
FKBP4P2	#DIV/0!	#DIV/0!	0.8030358	0	0	0	1	0	0
RNU6-1201P	0	#NUM!	0.3482063	0	0	6	0	0	0

AC112719.2	0.1	-3.321928	0.1959934	2	0	8	0	0	1
SDCBP2-AS1	0.9782609	-0.031709	0.9953006	49	38	51	21	45	69
C1GALT1	1.232756	0.3018872	0.0232525	2392	1568	2506	2063	2954	2954
PRSS30P	0.5634518	-0.827636	0.0549601	68	66	63	48	29	34
RAC1P2	0.9673913	-0.047829	0.9451634	21	43	28	17	42	30
SNORA22B	0.8	-0.321928	0.8941574	4	0	1	0	0	4
RNU6-1207P	0.25	-2	0.5554205	4	0	0	0	1	0
C14orf132	1.3050847	0.3841435	0.4602766	9	30	20	24	19	34
RNU6-638P	#DIV/0!	#DIV/0!	0.6411933	0	0	0	0	0	2
RN7SL546P	0	#NUM!	0.5387425	3	0	0	0	0	0
DPM1	1.1430899	0.1929389	0.036347	2196	1988	2567	2503	2828	2386
DPM2	1.1482759	0.1994693	0.075755	892	1038	1260	1305	1263	1095
DPM3	1.1532125	0.2056584	0.2088831	180	216	211	242	243	215
AC068707.1	0	#NUM!	0.824326	1	0	0	0	0	0
TIPRL	0.9156683	-0.127103	0.557012	1419	1234	1604	1099	1467	1332
SNORA22C	#DIV/0!	#DIV/0!	0.5509701	0	0	0	0	3	0
PSMB8-AS1	0.2857143	-1.807355	0.4327048	2	1	4	0	2	0
RNU6-1208P	0	#NUM!	0.824326	1	0	0	0	0	0
TEDC1	1.0031056	0.0044735	0.6353817	512	612	808	726	664	548
TEDC2	1.024026	0.0342523	0.5662442	457	527	556	584	531	462
AL096829.2	1.25	0.3219281	0.8925511	2	2	0	1	3	1
SNORA24B	0	#NUM!	0.1789363	2	0	6	0	0	0
AC022809.1	0.6470588	-0.628031	0.6554756	3	7	7	6	4	1
ADRB2	0.3333333	-1.584963	0.6628865	2	0	1	0	0	1
AC022413.1	1.7	0.7655347	0.4906896	5	0	5	6	4	7
ATE1-AS1	1.6666667	0.7369656	0.760483	0	3	0	1	2	2
DPP3	0.8462923	-0.240772	0.2437309	760	1052	966	753	831	767
AC064834.1	2.4285714	1.2801079	0.2996993	3	2	2	4	9	4
DPP4	0.3286266	-1.605479	2.71E-11	4005	4159	3238	1198	1079	1470
DPP6	0.1818182	-2.459432	0.0828415	12	4	6	1	0	3
RNU6-1205P	0	#NUM!	0.5781912	0	0	3	0	0	0
DPP7	0.9656652	-0.050405	0.9652262	177	116	173	152	181	117
DPP8	0.817755	-0.290259	0.1861552	844	568	931	477	695	744
DPP9	0.826029	-0.275736	0.1061131	1529	1776	1943	1398	1430	1507
RSPH10B2	1.6666667	0.7369656	0.6800281	0	0	3	4	1	0
MIR644A	#DIV/0!	#DIV/0!	0.4501902	0	0	0	0	0	4
RNU6-1206P	2	1	0.8324799	1	0	0	0	2	0
C14orf144	0	#NUM!	0.5075476	0	0	4	0	0	0
SRPRA	0.8128633	-0.298915	0.0295278	4679	5248	5901	3897	4627	4342
SRPRB	0.8574645	-0.221851	0.235904	2041	2348	2718	2029	2217	1848
RADIL	0.7297297	-0.454566	0.6201343	9	13	15	10	8	9
SERTAD4-AS1	1.4285714	0.5145732	0.2477771	17	15	31	26	33	31
MT-TL1	#DIV/0!	#DIV/0!	0.8030358	0	0	0	0	0	1
IQCJ-SCHIP1	1.0714286	0.0995357	0.5556704	193	254	197	181	241	268
AL109628.1	0	#NUM!	0.6436235	2	0	0	0	0	0
OR3D1P	2	1	0.6537304	1	0	1	4	0	0
BZW1P2	1.1290323	0.1750867	0.8385689	7	16	8	5	19	11
RNU6-636P	#DIV/0!	#DIV/0!	0.8030358	0	0	0	0	1	0
REPS2	0.6555556	-0.60921	0.3117723	35	30	25	16	35	8
C14orf119	1.0620221	0.0868138	0.2401286	1417	1478	1813	1663	1798	1539
RN7SL541P	#DIV/0!	#DIV/0!	0.242062	0	0	0	6	0	0
RNU6-1209P	0.6666667	-0.584963	0.87348	1	0	2	0	0	2
REPS1	0.915506	-0.127359	0.5515965	2081	1971	2410	1821	2064	2031
RWDD1	1.0257473	0.0366753	0.4388821	1042	976	1361	1175	1223	1068

RWDD3	0.9642857	-0.052467	0.9839406	42	17	53	22	44	42
RWDD4	1.1834171	0.2429586	0.0745283	420	345	429	413	552	448
DPRX	1.2270531	0.2951977	0.1330308	117	120	177	155	208	145
XPNPEP1	1.1094074	0.1497893	0.0927459	1859	2125	2277	2095	2566	2285
XPNPEP2	0	#NUM!	0.824326	1	0	0	0	0	0
XPNPEP3	0.8784559	-0.186958	0.3475388	641	576	700	465	642	577
CENPS-CORT	0.6666667	-0.584963	0.8466488	3	0	0	0	0	2
MIR6499	1	0	0.9756864	2	0	0	0	2	0
RNU6-635P	#DIV/0!	#DIV/0!	0.6554062	0	0	0	0	2	0
ZPR1	0.7187443	-0.476449	0.0005426	1656	1840	1983	1152	1431	1355
ITSN2	1.1329733	0.1801139	0.1804679	1074	703	1186	828	1210	1319
ITSN1	1.1678257	0.2238249	0.0432521	5239	4126	5734	4462	6563	6608
AC114763.2	0	#NUM!	0.284725	6	0	0	0	0	0
RN7SKP287	2.5	1.3219281	0.586765	2	0	0	1	1	3
AC114763.1	0.8372093	-0.25634	0.7514198	19	13	11	7	9	20
RN7SKP285	0	#NUM!	0.824326	1	0	0	0	0	0
RNU6-218P	0.5	-1	0.7988788	1	1	0	0	0	1
NOL8P1	0	#NUM!	0.824326	1	0	0	0	0	0
IGHV3-32	0	#NUM!	0.6410557	1	1	0	0	0	0
AC073342.1	2	1	0.8186965	0	0	1	0	0	2
IGHV3-35	#DIV/0!	#DIV/0!	0.8030358	0	0	0	0	0	1
RN7SKP271	0	#NUM!	0.6385096	0	2	0	0	0	0
AC131009.4	3	1.5849625	0.5010442	1	0	1	0	4	2
AC131009.3	0.4	-1.321928	0.105081	12	19	19	5	4	11
AC131009.1	0.6785714	-0.559427	0.7782988	5	2	21	15	2	2
RN7SKP276	0.2727273	-1.874469	0.2288882	10	4	8	6	0	0
MTHFD1	0.8725497	-0.196691	0.2201408	4628	4974	5345	3478	4658	4906
MTHFD2	1.1256069	0.1707031	0.0671993	1986	2008	2391	1975	2505	2707
RN7SKP272	0	#NUM!	0.5781912	0	0	3	0	0	0
DUSP8P4	#DIV/0!	#DIV/0!	0.5371397	0	0	0	0	0	3
DUSP8P5	0.7272727	-0.459432	0.3660545	41	31	60	24	45	27
DUSP8P3	0.9411765	-0.087463	0.9744241	5	1	11	5	5	6
RN7SKP260	0.1666667	-2.584963	0.3723105	3	0	3	0	1	0
ITGB1-DT	2	1	0.834705	0	0	1	0	2	0
RN7SKP268	0	#NUM!	0.824326	0	0	1	0	0	0
NPBWR1	#DIV/0!	#DIV/0!	0.8030358	0	0	0	0	0	1
LTA4H	1.0558696	0.0784317	0.2349051	1899	1820	2295	2045	2284	2021
RN7SKP269	1.25	0.3219281	0.814525	5	2	1	4	5	1
HEXIM1	1.1674779	0.2233952	0.0097272	4100	4287	5173	4995	5702	5134
HEXIM2	0.4756098	-1.07215	0.1146812	17	24	41	6	11	22
AC106872.11	#DIV/0!	#DIV/0!	0.6411933	0	0	0	0	0	2
AC106872.12	0	#NUM!	0.4374838	0	4	0	0	0	0
ENTPD3-AS1	0.971831	-0.041223	0.9765585	31	63	48	43	39	56
AC092118.1	0.5	-1	0.3199563	9	8	11	4	4	6
AC092118.2	0.6666667	-0.584963	0.790353	1	1	4	0	3	1
RN7SKP252	#DIV/0!	#DIV/0!	0.8030358	0	0	0	0	0	1
AC245060.5	2.6470588	1.4043903	0.0804868	2	4	11	10	13	22
AC245060.6	0.827957	-0.272372	0.7940331	22	12	59	24	23	30
DPYS	#DIV/0!	#DIV/0!	0.5070017	0	0	0	3	0	0
TRG-AS1	0.3421053	-1.547488	0.0948611	8	15	15	4	4	5
RN7SKP243	2.5	1.3219281	0.655915	0	2	0	0	0	5
RN7SKP249	0.5	-1	0.7145709	4	0	0	0	2	0
AL365277.1	1.7230769	0.7849871	0.0820207	21	14	30	32	49	31
CHST8	0.556962	-0.844349	0.1845382	28	25	26	19	10	15

DUSP4	0.7904617	-0.339233	0.0733272	631	642	698	532	539	487
AL365277.2	0.6938776	-0.527247	0.4846293	17	18	14	7	8	19
CHST9	#DIV/0!	#DIV/0!	0.288876	0	0	0	3	2	0
DUSP5	3.06	1.6135317	1.65E-29	550	625	725	2053	2023	1738
DUSP2	0	#NUM!	0.6840298	0	0	2	0	0	0
CHST7	0.92	-0.120294	0.965694	10	3	12	7	9	7
DUSP3	0.7035367	-0.507302	0.0001171	2161	2330	2606	1528	1766	1699
DUSP1	0.7390746	-0.436208	0.0563135	226	252	300	181	210	184
DUSP8	1.4381443	0.5242085	0.1130894	45	95	54	95	100	84
DUSP9	0.6666667	-0.584963	0.0381791	150	126	144	75	117	88
DUSP6	0.4	-1.321928	0.7006344	0	0	5	0	0	2
DUSP7	0.8440367	-0.244622	0.3488787	220	252	291	170	284	190
SFR1P1	#DIV/0!	#DIV/0!	0.4242335	0	0	0	0	3	1
RN7SKP230	0	#NUM!	0.824326	1	0	0	0	0	0
DPYD	1.4285714	0.5145732	0.7274331	1	4	2	1	3	6
CHST4	1.4444444	0.5305147	0.4188159	6	10	11	17	12	10
CHST2	1.6666667	0.7369656	0.6207325	0	0	6	1	4	5
CHST3	1.0909091	0.1255309	0.8780768	1	4	6	2	3	7
AC135803.1	0	#NUM!	0.5387425	3	0	0	0	0	0
RNU6-216P	0.3636364	-1.459432	0.3692597	3	6	2	2	1	1
RN7SKP229	0	#NUM!	0.4374838	0	4	0	0	0	0
PASD1	#DIV/0!	#DIV/0!	0.5070017	0	0	0	3	0	0
RRM2P2	1	0	0.9890559	0	0	1	1	0	0
RN7SKP219	0	#NUM!	0.824326	0	0	1	0	0	0
RRM2P3	#DIV/0!	#DIV/0!	0.8030358	0	0	0	1	0	0
RN7SKP209	#DIV/0!	#DIV/0!	0.247607	0	0	0	0	2	4
RN7SKP208	#DIV/0!	#DIV/0!	0.1677595	0	0	0	3	0	4
IFNA14	0	#NUM!	0.5337954	0	3	0	0	0	0
STK4-AS1	0.7894737	-0.341037	0.7421908	13	11	14	13	5	12
AC012617.1	1.1168831	0.1594782	0.6320173	24	18	35	26	29	31
AC092598.1	0	#NUM!	0.4460243	4	0	0	0	0	0
SRSF2	0.9234975	-0.11482	0.6202688	6297	5891	7380	5706	6533	5832
SRSF3	1.0046937	0.0067558	0.5303023	17503	17393	20497	16763	20118	18772
AC106730.1	2	1	0.4932653	4	1	1	8	4	0
SRSF4	1.0147789	0.0211654	0.4702749	2586	2756	3319	2663	3178	2948
SRSF5	0.8851093	-0.176072	0.3817698	10826	8828	13360	9227	10705	9289
SRSF6	0.9092042	-0.137324	0.4264055	7822	7280	8963	6369	7930	7581
SRSF7	0.9899812	-0.014527	0.654718	6974	7072	8811	6962	8142	7524
SRSF8	0.7640033	-0.388349	0.0059535	2260	2379	2645	1756	1879	1930
SRSF9	0.9142739	-0.129302	0.6333591	2746	3288	3613	2991	2955	2874
AP003680.1	1	0	0.9890559	1	0	0	0	0	1
LOXHD1	0	#NUM!	0.1909345	0	6	1	0	0	0
AC027045.2	0	#NUM!	0.6840298	0	0	2	0	0	0
AC027045.3	0.4285714	-1.222392	0.5713418	2	5	0	0	0	3
AC099554.1	#DIV/0!	#DIV/0!	0.2164877	0	0	0	4	2	0
CLPTM1L	0.8753939	-0.191996	0.270906	4367	5041	5190	4140	4509	4130
AP001439.1	0.1428571	-2.807355	0.3221946	2	0	5	0	0	1
AC004130.1	0	#NUM!	0.4460243	4	0	0	0	0	0
AP001043.1	0.6111111	-0.710493	0.5861942	2	10	6	2	9	0
AP001043.2	0	#NUM!	0.4374838	0	4	0	0	0	0
IGHV3-74	0.7333333	-0.447459	0.837273	5	0	10	2	0	9
VPS9D1	1.1601307	0.2142874	0.292814	89	100	117	137	108	110
TPMTP2	0	#NUM!	0.4374838	0	4	0	0	0	0
AC099804.1	0	#NUM!	0.824326	0	0	1	0	0	0

AL359979.2	1	0	0.9630562	0	0	4	0	3	1
IGHV3-41	0	#NUM!	0.2883218	0	0	7	0	0	0
IGHV3-42	0	#NUM!	0.5337954	0	3	0	0	0	0
IGHV3-43	0.825	-0.277534	0.7356175	26	10	4	6	8	19
SRSF1	0.9904885	-0.013788	0.7410331	7959	7861	8887	6893	8954	8625
IGHV3-47	0.9117647	-0.133267	0.958819	12	8	14	13	7	11
MTHFSD	0.8349136	-0.260301	0.1993059	515	580	698	470	504	523
AC099794.1	0	#NUM!	0.824326	0	1	0	0	0	0
E2F1	1.0175926	0.0251601	0.5546847	953	1084	1203	1235	1122	940
E2F2	0.9083333	-0.138706	0.551267	598	608	594	480	569	586
E2F3	0.8379158	-0.255123	0.1389767	1733	1496	1972	1079	1653	1626
E2F4	0.8849233	-0.176376	0.3452521	6221	6634	8131	6102	6584	5885
E2F5	1.01373	0.0196734	0.7471362	304	222	348	213	382	291
E2F6	0.8407295	-0.250286	0.2121609	544	536	565	440	464	479
E2F7	0.6636989	-0.591399	0.0003442	1650	1524	1768	776	1188	1316
E2F8	1.5751025	0.6554457	7.90E-06	878	857	948	993	1599	1634
AC112220.2	0.7012987	-0.511899	0.4387121	27	21	29	17	11	26
AC112220.3	1	0	0.9813095	2	3	1	2	0	4
AC112220.4	0.5764706	-0.794681	0.1773799	21	39	25	12	16	21
AC078777.1	0.5	-1	0.8143938	2	0	0	1	0	0
AP001189.4	0	#NUM!	0.5781912	0	0	3	0	0	0
AP001189.5	1.3636364	0.447459	0.5174313	5	11	17	6	22	17
AP001189.3	0	#NUM!	0.6385096	0	2	0	0	0	0
AP001189.1	0.2	-2.321928	0.4376609	4	0	1	0	1	0
ICAM2	1	0	0.9773797	5	0	0	0	2	3
LYPLA1P3	0.3846154	-1.378512	0.3560922	6	5	2	0	2	3
ICAM5	1.5066079	0.591304	0.0446113	62	97	68	114	138	90
IKZF2	1.0694789	0.096908	0.6502045	131	112	160	73	171	187
IKZF3	6	2.5849625	0.3547041	0	0	1	0	3	3
AP001189.6	0	#NUM!	0.5075476	0	0	4	0	0	0
IKZF4	0.9393305	-0.090295	0.8672236	172	140	166	130	152	167
AP000704.1	1.6666667	0.7369656	0.5361469	10	1	1	6	9	5
ICAM1	0.8885246	-0.170516	0.6276978	206	171	233	179	217	146
IKZF5	1.2987862	0.3771639	0.0362622	394	333	344	321	546	524
SRD5A3-AS1	0.7586207	-0.398549	0.7693806	12	7	10	15	5	2
AC084757.1	0.3846154	-1.378512	0.3700789	7	6	0	1	3	1
AC084757.2	1	0	0.9591042	1	3	0	0	4	0
AC084757.4	0	#NUM!	0.824326	0	1	0	0	0	0
PI4KAP1	1.1603427	0.214551	0.1007185	526	403	705	657	682	557
PI4KAP2	1.088705	0.1226131	0.1191666	2233	1916	2739	2283	2717	2499
AC127164.1	0.5714286	-0.807355	0.6357059	2	2	10	3	1	4
GAREM2	0	#NUM!	0.355562	5	0	0	0	0	0
GAREM1	0.9344609	-0.097794	0.8209069	179	131	163	100	156	186
TMEM63A	0.8593318	-0.218713	0.2044394	1387	1656	1656	1079	1586	1373
AC066595.1	0	#NUM!	0.6436235	2	0	0	0	0	0
TMEM63B	0.9145357	-0.128889	0.5859149	1351	1540	1567	1324	1451	1302
TMEM63C	1.1428571	0.1926451	0.8234381	2	1	4	6	2	0
AC105233.5	1.16	0.2141248	0.7152824	8	7	10	12	8	9
AC092451.3	0	#NUM!	0.824326	1	0	0	0	0	0
AC116337.1	#DIV/0!	#DIV/0!	0.5509701	0	0	0	0	3	0
AC136777.1	#DIV/0!	#DIV/0!	0.8030358	0	0	0	0	0	1
AC008737.1	0	#NUM!	0.0654134	4	7	0	0	0	0
GTF3AP6	2	1	0.834705	0	0	1	0	2	0
AC008737.3	1.5	0.5849625	0.8505665	1	0	1	0	3	0

AP002852.2	6.5	2.7004397	0.1306749	0	0	2	4	7	2
MON1A	0.664437	-0.589796	0.0201414	283	305	309	236	200	160
MON1B	0.7774803	-0.363122	0.0127081	1791	1989	2197	1250	1745	1652
PQLC2L	1.6666667	0.7369656	0.6104485	0	3	3	4	5	1
UIMC1	0.9275947	-0.108434	0.6349653	1812	1749	2143	1508	1981	1802
UFL1-AS1	0	#NUM!	0.1527178	2	4	1	0	0	0
AC110792.3	0	#NUM!	0.2883218	0	0	7	0	0	0
RPL7L1P11	0	#NUM!	0.824326	0	1	0	0	0	0
AC110792.4	0.5	-1	0.7932724	0	0	4	0	0	2
AC234772.2	0.8372093	-0.25634	0.7579506	9	27	7	14	14	8
AC234772.3	0.5	-1	0.8188512	2	0	0	0	1	0
KIAA1324L	0.987965	-0.017468	0.8472902	636	465	727	443	715	648
AC093677.1	#DIV/0!	#DIV/0!	0.5070017	0	0	0	3	0	0
AC093677.2	2.6	1.3785116	0.3178482	1	1	3	6	6	1
ELF3-AS1	0.5739645	-0.800967	0.126991	53	38	78	38	45	14
WWP1P1	0	#NUM!	0.824326	0	1	0	0	0	0
AC007907.1	#DIV/0!	#DIV/0!	0.4737294	0	0	0	0	4	0
AC022558.1	0.5	-1	0.8002255	1	1	0	0	1	0
AC022558.3	0.1666667	-2.584963	0.3064266	0	0	12	0	2	0
ACOT11	0.8571429	-0.222392	0.6678297	37	73	72	47	52	57
THOC7-AS1	0.2	-2.321928	0.436139	4	0	1	0	0	1
ACOT12	#DIV/0!	#DIV/0!	0.6411933	0	0	0	0	0	2
ACOT13	0.8556648	-0.224882	0.3116943	555	624	754	555	593	506
CENPCP1	2.5	1.3219281	0.6338788	0	0	2	0	5	0
AL359094.1	#DIV/0!	#DIV/0!	0.3721261	0	0	0	3	1	0
TRBV5-4	#DIV/0!	#DIV/0!	0.8030358	0	0	0	0	0	1
TRBV5-2	0	#NUM!	0.3042138	3	2	0	0	0	0
SUGT1P1	0.9324324	-0.100929	0.9436166	18	28	28	22	20	27
SUGT1P2	0.6	-0.736966	0.6742816	3	2	5	0	6	0
SUGT1P3	2	1	0.2427245	6	3	15	2	16	30
AC125257.1	0.9234375	-0.114914	0.814099	245	163	232	199	219	173
UVSSA	0.9957983	-0.006075	0.8308089	365	397	428	297	379	509
MIR1244-2	1.1963824	0.2586786	0.1859785	123	119	145	151	182	130
MIR1244-3	#DIV/0!	#DIV/0!	0.8030358	0	0	0	0	1	0
AC245060.2	1.2666667	0.3410369	0.7910374	11	2	2	1	5	13
AC245060.4	1.0334967	0.0475338	0.5416671	387	363	474	336	489	440
ZNF192P1	0.777027	-0.363963	0.4750027	53	30	65	24	42	49
LTB4R	0.8626482	-0.213156	0.5179922	305	249	458	283	290	300
LCMT1-AS2	0.742918	-0.428725	0.0044238	1443	1391	1861	919	1350	1219
LCMT1-AS1	1.5625	0.6438562	0.5440498	5	9	2	4	13	8
AP003096.1	0.3636364	-1.459432	0.3932445	1	9	1	1	3	0
MKL2	0.7514381	-0.412274	0.045594	1157	905	1241	519	924	1039
MKKS	0.9708933	-0.042615	0.9122927	1031	846	1112	866	1036	1000
XIAP-AS1	0	#NUM!	0.6436235	2	0	0	0	0	0
MKL1	0.7796531	-0.359096	0.0364839	1238	1567	1461	1071	1156	1099
RN7SKP191	0	#NUM!	0.824326	0	0	1	0	0	0
RN7SL118P	0	#NUM!	0.4496863	0	1	3	0	0	0
AC073488.4	0	#NUM!	0.824326	0	1	0	0	0	0
AC073488.5	0.25	-2	0.5415866	0	4	0	1	0	0
AC073488.7	0.2222222	-2.169925	0.1328992	7	5	6	0	3	1
AC073488.1	1.5714286	0.6520767	0.6475947	3	3	1	2	4	5
AC113935.1	1.0689655	0.0962153	0.3974291	220	221	313	232	299	275
AC073488.2	0.6666667	-0.584963	0.9030659	0	0	3	0	0	2
AC073488.3	0	#NUM!	0.824326	1	0	0	0	0	0

IFIT1B	#DIV/0!	#DIV/0!	0.6554062	0	0	0	0	2	0
B3GNTL1P1	0	#NUM!	0.824326	1	0	0	0	0	0
RN7SKP180	0	#NUM!	0.824326	0	0	1	0	0	0
NT5C2	0.9251856	-0.112185	0.7207869	647	487	617	364	543	713
RN7SKP186	#DIV/0!	#DIV/0!	0.8030358	0	0	0	0	1	0
TRDMT1	0	#NUM!	0.4460243	4	0	0	0	0	0
URGCP	0.7380224	-0.438263	0.0614306	281	304	396	253	228	243
YY1AP1	1.0214258	0.0305844	0.4264813	1526	1574	2034	1659	1817	1768
RN7SKP179	#DIV/0!	#DIV/0!	0.6411933	0	0	0	0	0	2
MIR5692C2	#DIV/0!	#DIV/0!	0.6554062	0	0	0	0	2	0
RN7SKP173	0.625	-0.678072	0.5969232	9	6	1	4	2	4
EIF4HP1	0.7967033	-0.327886	0.2423049	162	201	183	111	142	182
DQX1	0.5851852	-0.773035	0.1420211	36	33	66	30	22	27
EIF4HP2	0.8142857	-0.296393	0.6081301	55	21	64	24	57	33
WDR83	1	0	0.8575068	53	83	76	68	58	86
WDR89	1.2558993	0.3287208	0.0533454	728	369	810	689	915	791
WDR87	2	1	0.834705	0	0	1	0	2	0
WDR88	0.5285714	-0.91983	0.0754894	42	42	56	8	41	25
WDR92	0.8935698	-0.162348	0.637604	168	125	158	115	164	124
WDR93	0.3333333	-1.584963	0.7290749	0	0	3	0	0	1
WDR90	1.0731707	0.1018796	0.9049703	16	21	4	12	19	13
AL391219.1	#DIV/0!	#DIV/0!	0.6554062	0	0	0	0	2	0
WDR91	1.0574949	0.0806507	0.4408894	276	333	365	353	376	301
AC098820.3	0.9230769	-0.115477	0.9249351	9	1	3	2	8	2
RN7SL138P	1	0	0.9589987	6	6	7	5	7	7
WDR74	0.8740773	-0.194167	0.3169034	1275	1545	1786	1239	1404	1383
WDR75	1.1555594	0.2085914	0.0919959	2233	1399	2115	1961	2526	2154
AC098820.1	0.7910448	-0.338169	0.5950323	24	25	18	16	18	19
WDR72	1.0428157	0.0604842	0.5439316	560	348	470	468	523	446
WDR73	0.6265823	-0.674424	0.049285	99	103	114	78	58	62
HCFC2	0.8949153	-0.160177	0.6252263	221	151	218	115	208	205
WDR78	0.826087	-0.275634	0.8011979	8	12	26	9	10	19
WDR76	0.8090871	-0.305633	0.1243473	782	665	952	495	664	782
HCFC1	0.740513	-0.433403	0.0054227	5180	6490	6381	3458	4742	5167
WDR77	0.9314079	-0.102515	0.8077235	841	924	1005	915	896	769
WDR81	0.7865572	-0.346376	0.0497761	1113	1421	1364	836	1045	1185
WDR82	0.8116585	-0.301055	0.0481825	3834	4200	4369	2551	3757	3759
PARD3	1.0477656	0.067316	0.2531337	2978	2809	3634	2855	3580	3436
WDR63	0.5135135	-0.961526	0.0223079	66	48	71	23	31	41
WDR64	1.4827586	0.5682838	0.50063	15	5	9	4	27	12
WDR61	0.8232915	-0.280525	0.1888253	743	780	950	736	682	618
WDR62	0.8079113	-0.307731	0.0788537	962	1155	1220	876	946	874
WDR66	0.7409091	-0.432632	0.2530555	62	73	85	45	69	49
BLOC1S4	1.0714664	0.0995866	0.2711193	1152	1282	1344	1373	1485	1190
BLOC1S5	1.0010799	0.0015571	0.8111117	323	260	343	229	377	321
BLOC1S6	1.0212076	0.0302762	0.6516589	1564	1020	1424	1041	1512	1540
WDR70	0.9830317	-0.02469	0.7474558	1381	1281	1758	1356	1591	1398
BLOC1S1	0.6875	-0.540568	0.5163118	13	10	25	9	16	8
BLOC1S2	0.9638407	-0.053133	0.9504763	1335	1172	1586	1216	1487	1242
BLOC1S3	0.747101	-0.420625	0.0251958	507	635	669	430	458	465
WDR59	0.8927997	-0.163592	0.431441	1588	1432	1952	1394	1519	1526
WDR53	0.9777954	-0.032395	0.8395367	391	374	496	381	412	440
WDR54	1.1682044	0.2242927	0.1113205	386	473	550	543	657	446
WDR55	0.8263771	-0.275128	0.0518346	2528	2734	2980	2003	2491	2317

WDR60	1.047619	0.0671142	0.5488241	306	270	285	261	289	352
MKS1	0.9859056	-0.020479	0.8127735	384	514	521	488	494	417
WDR5B	0.8694915	-0.201756	0.5134911	198	204	188	177	166	170
LIX1-AS1	3.5	1.8073549	0.4710672	1	1	0	0	0	7
SGO1-AS1	0.1428571	-2.807355	0.1032232	11	0	10	2	0	1
WDR49	#DIV/0!	#DIV/0!	0.8030358	0	0	0	0	0	1
WDR47	1	0	0.7998937	332	249	314	245	327	323
WDR48	1.0544594	0.0765035	0.3907265	1406	1015	1380	1088	1493	1427
WDR41	0.9360406	-0.095357	0.7567872	1306	1108	1526	978	1367	1343
WDR45	1.1056447	0.1448879	0.1485161	1055	1358	1449	1330	1573	1367
WDR46	0.8416409	-0.248723	0.173244	1951	2292	2558	1940	2001	1783
WDR43	0.9130533	-0.131229	0.5478092	4778	3598	4908	2988	4605	4536
WDR44	1.0603448	0.0845335	0.5743694	534	355	503	304	579	593
WDR38	0.75	-0.415037	0.8857596	3	0	1	1	0	2
WDR36	0.9693835	-0.044861	0.9839671	1885	1135	1814	1170	1769	1747
WDR37	0.9931193	-0.009961	0.7232172	731	864	1021	765	879	954
WDR31	1.0508475	0.0715533	0.7007875	68	95	73	81	77	90
WDR34	1.2220228	0.2892712	0.014818	4003	4875	5053	6185	5721	5118
WDR35	#DIV/0!	#DIV/0!	0.0203621	0	0	0	1	10	4
WDR33	0.9684955	-0.046183	0.9965914	3244	3358	3714	2719	3725	3547
AC092117.2	0.2	-2.321928	0.4741744	0	1	4	0	0	1
AC092117.1	0.9444444	-0.082462	0.9979814	9	14	13	13	3	18
AL590556.1	1	0	0.9890559	1	0	0	0	0	1
AL590556.2	1.3333333	0.4150375	0.8450489	1	0	2	0	2	2
AL359092.1	0	#NUM!	0.6631647	1	0	1	0	0	0
WDR27	0.9679952	-0.046928	0.9367022	603	415	638	463	553	587
MKRN3-AS1	0.6285714	-0.669851	0.480999	17	14	4	8	13	1
WDR25	0.7943262	-0.332196	0.2585652	138	199	227	143	153	152
WDR26	1.1487313	0.2000414	0.0546169	2712	2081	2892	2395	3335	3098
TSEN15P2	0	#NUM!	0.824326	1	0	0	0	0	0
TRBV7-2	0	#NUM!	0.824326	0	1	0	0	0	0
TRBV7-3	0	#NUM!	0.824326	0	1	0	0	0	0
WDR20	1.0577465	0.0809939	0.3170581	903	815	1122	814	1072	1118
WDR24	1.1675774	0.2235182	0.099426	303	340	455	456	429	397
POLRMTP1	1.1724138	0.2294818	0.7233265	7	14	8	15	13	6
RNU6-228P	7	2.8073549	0.3457479	0	1	0	0	7	0
WDR17	0.6687898	-0.580375	0.0922576	128	82	104	65	62	83
DUTP1	0	#NUM!	0.824326	0	0	1	0	0	0
DUTP2	0.75	-0.415037	0.8636771	1	1	2	0	3	0
WDR18	1.1102925	0.1509398	0.1374904	1261	1578	1640	1703	1659	1611
WDR19	1.0309973	0.0440406	0.6462953	266	153	323	194	288	283
AP001282.1	#DIV/0!	#DIV/0!	0.8030358	0	0	0	0	1	0
AP001282.2	4	2	0.478626	1	0	0	1	1	2
WDR12	1.0375171	0.0531351	0.3409885	3095	2793	3601	2766	3672	3407
WDR13	0.9912892	-0.012622	0.6998755	644	743	909	863	739	674
DUTP6	0.75	-0.415037	0.9117941	1	0	3	0	0	3
WDR11	1.1689269	0.2251847	0.11525	1189	853	1350	899	1568	1498
AC026801.2	0.3333333	-1.584963	0.6622061	0	3	0	0	0	1
AC026801.3	1.2560976	0.3289485	0.4051024	25	22	35	26	31	46
RERGL	3.6666667	1.8744691	0.3788694	3	0	0	0	1	10
AC034102.5	1.5	0.5849625	0.6781139	0	3	3	3	1	5
AC034102.6	2.25	1.169925	0.4492185	1	2	1	6	2	1
PARK7	1.0436031	0.0615732	0.3049289	6014	6867	8012	7239	7839	6726
AC034102.1	1.5277778	0.6114347	0.3577634	17	6	13	15	30	10

AL022345.1	0	#NUM!	0.4496863	0	1	3	0	0	0
AL022345.2	0	#NUM!	0.2883218	0	0	7	0	0	0
AC099795.1	0	#NUM!	0.3052077	1	4	0	0	0	0
AC099795.2	0.5714286	-0.807355	0.7315239	0	7	0	3	0	1
PTTG4P	0	#NUM!	0.824326	1	0	0	0	0	0
LARGE1	0.6814701	-0.553278	0.0054751	356	446	504	234	357	299
LARGE2	0.6971122	-0.520537	0.0007511	2640	3056	3238	2120	2242	1866
IFNAR2	1.218107	0.2846409	0.286509	88	74	81	67	109	120
ACTBP2	1.6923077	0.7589919	0.4520849	4	7	2	12	7	3
RN7SL130P	0.6410256	-0.641546	0.5296433	6	15	18	13	4	8
AC084026.2	2.5	1.3219281	0.553222	0	1	1	5	0	0
AC084026.3	0	#NUM!	0.5781912	0	0	3	0	0	0
ACTBP1	#DIV/0!	#DIV/0!	0.5509701	0	0	0	0	3	0
ACTBP9	0.6666667	-0.584963	0.8035772	1	2	3	0	0	4
ADTRP	0.8846154	-0.176878	0.9455911	3	7	16	7	6	10
IFNAR1	0.6755043	-0.565963	0.0038546	635	443	657	342	469	361
DRC7	0.7402135	-0.433987	0.274142	76	79	126	67	56	85
PARM1	0.9001189	-0.151813	0.5254943	516	523	643	436	528	550
SLC38A10	0.8646266	-0.209851	0.2751645	3422	4094	4510	3528	3478	3392
SLC38A11	0	#NUM!	0.4460243	4	0	0	0	0	0
MAP3K20	1.1969618	0.2593771	0.1118966	712	472	725	512	886	887
MAP3K21	0.818408	-0.289108	0.3892142	168	94	140	71	135	123
TTLL13P	#DIV/0!	#DIV/0!	0.2758481	0	0	0	4	0	1
DRD1	#DIV/0!	#DIV/0!	0.1486377	0	0	0	2	6	0
DRD2	0.1818182	-2.459432	0.2087854	2	7	2	0	0	2
AC108449.2	0.8028169	-0.316857	0.6359574	34	15	22	9	18	30
DRD4	0.8629442	-0.212661	0.8202575	60	60	77	95	40	35
UVRAG	0.8956186	-0.159044	0.4864069	824	704	800	588	679	818
CHTOP	0.8481305	-0.237642	0.1456641	2000	1974	2525	1763	1956	1793
IQCA1L	#DIV/0!	#DIV/0!	0.1700664	0	0	0	0	9	0
MAP3K15	0.1666667	-2.584963	0.3204962	4	1	1	0	0	1
MAP3K19	0	#NUM!	0.4374838	0	4	0	0	0	0
MAP3K10	0.9520451	-0.070898	0.9963944	194	250	265	249	224	202
DRC1	1.3076923	0.3870231	0.7026408	7	2	4	4	6	7
MAP3K13	0.8241379	-0.279042	0.4629488	99	78	113	55	78	106
MAP3K14	0.5767344	-0.794021	0.0002487	368	556	503	255	257	311
DRC3	1.1616162	0.2161334	0.5062269	24	25	50	46	20	49
MAP3K11	0.872418	-0.196909	0.299579	1172	1473	1470	1075	1246	1269
PTTG3P	#DIV/0!	#DIV/0!	0.8030358	0	0	0	0	0	1
MAP3K12	0.986755	-0.019236	0.9529417	94	84	124	50	124	124
AL031658.1	0.2941176	-1.765535	0.2414145	3	4	10	0	4	1
AL031658.2	0.3636364	-1.459432	0.3701415	4	3	4	1	1	2
AC024619.3	0	#NUM!	0.5387425	3	0	0	0	0	0
AC024619.4	0	#NUM!	0.5337954	0	3	0	0	0	0
CKAP2L	1.4837736	0.569271	0.0025763	528	279	518	506	702	758
TIRAP	0.6392157	-0.645625	0.0795691	73	83	99	50	45	68
ACTBL2	0.5909091	-0.758992	0.5989338	1	16	5	10	1	2
AL136099.1	1.3461538	0.4288433	0.3784805	13	9	30	20	23	27
NSRP1	1.2255929	0.2934799	0.070557	697	400	716	573	888	761
HCG18	0.7970027	-0.327343	0.1004727	495	417	556	375	406	389
AL157786.1	0	#NUM!	0.2117164	3	0	4	0	0	0
HCG15	0.6626016	-0.593786	0.1094434	64	80	102	37	74	52
FLJ42102	#DIV/0!	#DIV/0!	0.8030358	0	0	0	0	0	1
HCG14	0.4	-1.321928	0.4792718	2	0	13	0	2	4

HCG11	0.8884211	-0.170685	0.615236	185	131	159	101	144	177
AL356356.1	1.0344828	0.0489096	0.8019359	18	4	36	18	14	28
AL157400.1	0	#NUM!	0.6840298	0	0	2	0	0	0
HCG27	2.5714286	1.3625701	0.4308045	0	7	0	6	12	0
WDR97	0.8333333	-0.263034	0.858981	3	3	6	0	7	3
AC092597.1	#DIV/0!	#DIV/0!	0.5338029	0	0	0	0	1	2
AC107956.1	0.7	-0.514573	0.6672224	7	12	1	5	9	0
AF001548.2	0.4285714	-1.222392	0.5709381	1	6	0	2	1	0
AL157400.2	6	2.5849625	0.3651604	0	1	0	0	5	1
DRG1	0.9212935	-0.118267	0.6483471	2489	2755	3167	2527	2804	2418
AL157400.3	0.375	-1.415037	0.5275168	6	0	2	0	0	3
DRG2	0.8850374	-0.17619	0.4214552	1105	1342	1563	1145	1227	1177
HCG25	0.5333333	-0.906891	0.5158367	5	6	4	3	5	0
AL157400.4	5.3333333	2.4150375	0.1159669	2	0	1	3	6	7
HCG20	2	1	0.8319548	0	1	0	0	2	0
IGKV2OR2-7D	2	1	0.807847	1	0	0	0	1	1
IFITM3	4.5538865	2.1870983	1.96E-49	1122	1430	1642	6869	6723	5507
VEZT	1.2727273	0.3479233	0.0083078	1375	952	1424	1267	1800	1707
IFITM1	3.4536585	1.7881255	4.90E-32	345	369	516	1450	1551	1247
IFITM2	1.4637681	0.549687	0.2112235	28	17	24	30	35	36
PARS2	0.7581522	-0.399441	0.0632156	314	370	420	273	275	289
MLC1	#DIV/0!	#DIV/0!	0.6099294	0	0	0	2	0	0
HS6ST1	1.0673575	0.0940435	0.2714199	686	776	854	774	857	841
HS6ST2	0.8616352	-0.214851	0.6357528	59	49	51	21	58	58
HS6ST3	1.0625	0.0874628	0.9161516	15	13	4	7	6	21
PART1	1	0	0.9592498	3	0	4	2	4	1
HLA-J	0.9166667	-0.125531	0.975815	0	5	7	2	3	6
SRRM2	0.8044866	-0.31386	0.0525577	15190	16786	17995	9774	14228	16199
HLA-K	0	#NUM!	0.824326	0	1	0	0	0	0
SRRM3	0.9314815	-0.102401	0.8578454	309	384	387	375	347	284
HLA-H	0.0666667	-3.906891	0.0445182	4	8	3	0	1	0
IGFL1P1	2.2	1.1375035	0.4984942	1	0	4	0	6	5
SRRM4	0	#NUM!	0.0850695	3	4	2	0	0	0
IGFL1P2	#DIV/0!	#DIV/0!	0.8030358	0	0	0	0	1	0
SRRM5	0.962963	-0.054448	0.9925523	7	3	17	2	21	3
HLA-L	#DIV/0!	#DIV/0!	0.8030358	0	0	0	0	1	0
HLA-B	1.5135135	0.5979016	0.3090391	4	9	24	25	17	14
HLA-C	1.4044913	0.4900477	0.0001356	3009	3908	4082	5442	5306	4700
HLA-A	#DIV/0!	#DIV/0!	0.8030358	0	0	0	0	1	0
HCG4B	0	#NUM!	0.824326	0	0	1	0	0	0
HLA-F	1.2727273	0.3479233	0.707765	15	1	6	5	11	12
HLA-G	1.6891947	0.7563356	1.97E-07	686	798	987	1476	1521	1177
HLA-E	1.1406706	0.1898822	0.0465574	3194	3787	3995	3958	4607	3955
SRRM1	0.9138004	-0.130049	0.5017676	5348	4935	6260	3937	5619	5561
AL392046.1	2.8571429	1.5145732	0.2414712	1	4	2	2	13	5
AL392046.2	1.5248619	0.6086786	0.0425535	65	42	74	87	72	117
MTHFD2L	1.1186441	0.1617511	0.4459948	122	58	115	123	91	116
DUX4L35	0	#NUM!	0.4521054	1	0	3	0	0	0
DUX4L37	1.3333333	0.4150375	0.8337618	0	6	0	2	1	5
HLA-Z	0	#NUM!	0.824326	1	0	0	0	0	0
CHTF8	0.7545385	-0.406334	0.0066849	3031	3702	3733	2480	2663	2754
MLEC	0.7667027	-0.383261	0.0035509	8477	9769	10447	6313	7902	7784
HLA-P	1	0	0.9794552	6	8	5	1	12	6
HLA-V	0.9131737	-0.131039	0.7704336	105	89	140	59	134	112

HLA-W	#DIV/0!	#DIV/0!	0.5070017	0	0	0	3	0	0
DUX4L27	0.125	-3	0.2442934	6	0	2	0	1	0
TMEM74B	3.625	1.857981	0.03073	0	5	11	11	32	15
PARVA	1.0348703	0.04945	0.3363949	4059	4126	4806	3875	4919	4650
PARVB	7	2.8073549	0.3235909	0	1	0	0	0	7
AC005593.1	0	#NUM!	0.0253293	5	0	12	0	0	0
DUX4L33	#DIV/0!	#DIV/0!	0.8030358	0	0	0	0	0	1
AC234771.4	0.6666667	-0.584963	0.7676391	2	2	2	0	1	3
AC006435.2	1.3888889	0.4739312	0.4290661	14	8	14	13	20	17
PARP6	0.9745958	-0.037124	0.9574284	160	105	168	112	181	129
AC068993.1	1	0	0.8969329	1	0	4	5	0	0
PARP3	0.6464646	-0.629357	0.2444564	46	25	28	15	27	22
PARP4	0.8584855	-0.220134	0.2618151	1370	1070	1284	756	1210	1231
PARP1	0.7514695	-0.412214	0.0010803	10096	11319	12440	7330	9136	8975
PARP2	1.04614	0.0650759	0.3378046	1483	1248	1712	1337	1777	1534
PARP9	5.2631579	2.3959287	3.20E-26	87	74	67	296	465	439
MLF1	1.5121951	0.5966443	0.0087198	96	121	111	150	192	154
MLF2	0.8764716	-0.190221	0.3345064	5253	6259	6751	5451	5602	4954
PARP8	1.0733871	0.1021705	0.292349	854	657	969	741	1053	868
DUX4L52	#DIV/0!	#DIV/0!	0.6099294	0	0	0	2	0	0
DUX4L50	1.030303	0.0430687	0.8570358	9	12	12	18	9	7
AMIGO1	0.61875	-0.692571	0.0363724	90	123	107	43	75	80
ICA1L	1.4151735	0.5009789	6.59E-05	867	682	1074	1094	1329	1289
AMIGO2	1.1721154	0.2291146	0.1014259	363	321	356	345	438	436
PSMC1P11	2.5	1.3219281	0.6099065	0	0	2	0	0	5
PSMC1P12	0.5	-1	0.8241474	0	1	1	0	0	1
DRP2	1.4642857	0.5501971	0.4176414	10	5	13	19	17	5
MTHFD1L	0.816789	-0.291965	0.0538258	1863	2127	2276	1577	1852	1689
AL645933.2	0	#NUM!	0.6840298	0	0	2	0	0	0
AC011632.1	0.9	-0.152003	0.9666335	4	10	6	12	3	3
MLH1	0.76356	-0.389187	0.0051927	2002	1743	2210	1342	1631	1574
MLH3	1.0603942	0.0846006	0.4675061	628	343	602	477	624	567
H2AFZP1	4	2	0.4791321	0	1	0	3	0	1
H2AFZP3	4	2	0.1495049	1	1	2	12	3	1
H2AFZP4	#DIV/0!	#DIV/0!	0.8030358	0	0	0	0	1	0
DOCK10	0.6	-0.736966	0.7624539	2	1	2	0	0	3
DOCK11	1.1609567	0.2153142	0.1796233	626	318	603	509	639	648
pRNA	0.6666667	-0.584963	0.779637	4	2	0	0	0	4
pRNA	0.6666667	-0.584963	0.779637	4	2	0	0	0	4
pRNA	0.6666667	-0.584963	0.779637	4	2	0	0	0	4
pRNA	0.6666667	-0.584963	0.779637	4	2	0	0	0	4
pRNA	0.6666667	-0.584963	0.779637	4	2	0	0	0	4
pRNA	0.6666667	-0.584963	0.779637	4	2	0	0	0	4
pRNA	0.6666667	-0.584963	0.779637	4	2	0	0	0	4
pRNA	0.6666667	-0.584963	0.779637	4	2	0	0	0	4
pRNA	0.6666667	-0.584963	0.779637	4	2	0	0	0	4
pRNA	0.6666667	-0.584963	0.779637	4	2	0	0	0	4
pRNA	0.6666667	-0.584963	0.779637	4	2	0	0	0	4
pRNA	0.6666667	-0.584963	0.779637	4	2	0	0	0	4
pRNA	0.6666667	-0.584963	0.779637	4	2	0	0	0	4
POLR3GP1	0.2727273	-1.874469	0.3428665	8	3	0	0	0	3
MRPL45P2	0.9015748	-0.149481	0.7460207	77	91	86	73	78	78
MRPL45P1	2	1	0.6836413	0	0	3	0	6	0
PSG11	2.8	1.4854268	0.3609584	2	3	0	1	2	11

ZNF587P1	0	#NUM!	0.1796811	2	5	0	0	0	0
CUZD1	0	#NUM!	0.6385096	0	2	0	0	0	0
CDH23	1.5555556	0.6374299	0.3264534	7	6	14	17	9	16
CDH24	0.800084	-0.321777	0.0685518	757	737	887	617	630	658
CDH26	0.4	-1.321928	0.2713182	6	10	4	3	2	3
AP003097.2	#DIV/0!	#DIV/0!	0.5371397	0	0	0	0	0	3
AP001775.2	0	#NUM!	0.4460243	4	0	0	0	0	0
AP003097.1	0	#NUM!	0.5337954	0	3	0	0	0	0
MLKL	1.5714286	0.6520767	0.4900505	3	7	4	8	8	6
GPSM1	1.0863192	0.1194481	0.2756024	358	406	464	467	445	422
GPSM2	1.0379851	0.0537858	0.5791963	885	631	906	583	967	964
AC089999.3	1	0	0.9669567	7	6	4	6	5	6
CMAHP	1.2984293	0.3767675	0.0928822	123	104	155	111	184	201
FHAD1	0.8666667	-0.206451	0.8335323	25	8	12	14	10	15
MFSD8	1.1589862	0.2128634	0.1644302	475	314	513	372	597	540
MFSD9	1.0201613	0.0287973	0.5911727	557	563	616	506	612	653
MFSD6	1.3406114	0.4228911	0.0737249	188	106	164	143	216	255
MFSD7	1	0	0.948249	2	0	4	1	0	5
MFSD5	0.8460138	-0.241247	0.2424826	812	1009	926	785	786	753
MFSD3	1.0410742	0.058073	0.6144434	192	225	216	230	256	173
MFSD1	1.0420054	0.0593628	0.3463688	1175	1165	1350	1248	1352	1245
GPSM3	0.7958333	-0.329462	0.3013358	143	164	173	146	124	112
AC093772.1	#DIV/0!	#DIV/0!	0.5509701	0	0	0	0	3	0
HGSNAT	0.940865	-0.08794	0.7824195	684	743	839	582	819	731
AC008736.1	1.7837838	0.8349408	0.1854293	2	14	21	29	16	21
AP000943.1	0.8550725	-0.225881	0.6564009	42	51	45	17	39	62
AC007992.2	#DIV/0!	#DIV/0!	0.4737294	0	0	0	0	4	0
AP000943.2	0.7883817	-0.343034	0.3750671	99	77	65	34	70	86
AP000943.4	0	#NUM!	0.6436235	2	0	0	0	0	0
DDAH1	1.0843373	0.1168137	0.8191602	34	31	18	16	26	48
DDAH2	1.2741047	0.3494838	0.0160886	401	491	560	675	606	569
CDH10	0.2769231	-1.852443	0.0205901	24	11	30	2	6	10
CDH11	#DIV/0!	#DIV/0!	0.5509701	0	0	0	0	3	0
CDH12	0.4307692	-1.215013	0.0050368	74	64	57	14	41	29
CDH13	#DIV/0!	#DIV/0!	0.3981469	0	0	0	4	0	0
AKNAD1	0.7777778	-0.36257	0.7432911	10	7	10	7	13	1
CDH15	7.6666667	2.9385995	0.0425555	2	1	0	3	12	8
CDH16	2	1	0.7741804	1	0	0	2	0	0
CDH18	1.0927835	0.1280076	0.3286336	451	287	426	456	445	371
AC091133.5	2	1	0.6878446	0	0	2	0	1	3
AC091133.3	#DIV/0!	#DIV/0!	0.8030358	0	0	0	0	1	0
AC091133.4	1.4137931	0.499571	0.2982754	19	8	31	24	27	31
CHSY1	1.0217216	0.0310022	0.7020139	425	394	424	307	451	512
CHSY3	1.5965909	0.6749947	0.0198682	59	51	66	69	103	109
AC091133.2	0.6216216	-0.685891	0.4367192	9	25	3	7	9	7
RN7SKP292	#DIV/0!	#DIV/0!	0.8030358	0	0	0	0	0	1
RN7SKP293	#DIV/0!	#DIV/0!	0.8030358	0	0	0	1	0	0
RN7SL128P	1.25	0.3219281	0.8598391	1	1	2	0	1	4
AC073487.1	1.8333333	0.8744691	0.4630621	5	5	2	3	16	3
MLPH	0.9347826	-0.097297	0.9662403	17	14	15	17	11	15
RN7SKP296	0.7	-0.514573	0.8567552	0	0	10	0	2	5
RN7SKP297	0	#NUM!	0.824326	1	0	0	0	0	0
PAXBP1	1.1781552	0.2365296	0.161628	1181	692	1130	771	1425	1342
USP6NL	1.2404632	0.310879	0.0295752	1085	802	1049	883	1370	1389

AP002026.1	1.5437788	0.6264661	0.0211745	86	49	82	102	115	118
ZDBF2	1.1237085	0.1682679	0.5521102	1522	696	1460	922	1519	1692
STARD3	1.0183844	0.0262822	0.5726536	1023	1307	1260	1189	1254	1213
STARD4	1.0771285	0.1071904	0.4007823	1202	797	996	815	1197	1214
STARD5	1.3402778	0.422532	0.1821161	47	30	67	64	77	52
STARD6	1.1111111	0.1520031	0.9364401	4	3	2	1	8	1
STARD7	0.9718679	-0.041168	0.9298528	6768	7689	8435	6345	8241	7662
STARD8	1	0	0.9031964	18	34	39	25	37	29
STARD9	0.5240964	-0.932096	0.0306091	45	55	66	25	29	33
DNTTIP1	0.9129979	-0.131317	0.6804347	1083	1366	1367	1257	1169	1058
DNTTIP2	1.3279144	0.4091622	0.0203826	1857	853	1965	2030	2341	1837
[MEM246-AS	0	#NUM!	0.1789363	2	0	6	0	0	0
CLNS1A	0.95068	-0.072968	0.938201	3735	4025	5034	3814	4396	3953
LAPTM4BP2	#DIV/0!	#DIV/0!	0.8030358	0	0	0	0	1	0
CDIP1	0.7459016	-0.422943	0.42336	53	31	38	34	28	29
PRKXP1	1.7857143	0.8365013	0.4360172	10	0	4	13	10	2
AC009563.1	2	1	0.5889039	5	0	0	6	4	0
AC034105.3	#DIV/0!	#DIV/0!	0.8030358	0	0	0	0	1	0
GUCD1	0.8239247	-0.279416	0.1135376	1707	2093	2152	1635	1739	1530
PPARGC1A	1.1025641	0.1408625	0.5249916	172	100	157	102	212	159
PPARGC1B	0.7167235	-0.480511	0.0136078	1515	1668	1798	763	1246	1561
APCDD1L	#DIV/0!	#DIV/0!	0.8030358	0	0	0	0	0	1
AC096664.1	0.1666667	-2.584963	0.3177349	1	4	1	0	1	0
AC096664.2	0	#NUM!	0.824326	1	0	0	0	0	0
PCDHGA10	0.8321739	-0.265043	0.2808341	324	412	414	213	338	406
PCDHGA11	1.1137441	0.1554178	0.5345581	76	43	92	53	85	97
PCDHGA12	0.755102	-0.405256	0.4443857	42	31	25	12	38	24
AC099062.1	0	#NUM!	0.6436235	2	0	0	0	0	0
SEPT14P24	#DIV/0!	#DIV/0!	0.6554062	0	0	0	0	2	0
FLVCR1	0.8260073	-0.275774	0.1885473	737	690	757	378	708	718
FLVCR2	1.0942314	0.1299178	0.1640159	945	1118	1248	1190	1282	1151
NSUN4	0.8926692	-0.163802	0.4318389	852	998	1001	773	874	898
NSUN5	0.8431163	-0.246196	0.274285	1546	1951	2125	1742	1633	1365
NSUN2	0.7844762	-0.350198	0.0052422	6558	6814	7744	4698	6075	5792
NSUN3	0.7752809	-0.367209	0.2668072	108	98	150	77	92	107
NSUN6	1.2762376	0.351897	0.0149142	347	279	384	386	436	467
NSUN7	1.0883392	0.1221283	0.4008332	198	159	209	172	230	214
DYNLL1	1.0509893	0.071748	0.3179953	3113	3436	3963	4018	3798	3232
DYNLL2	0.8111088	-0.302033	0.0383031	20423	25391	25391	16385	20849	20521
SEPT14P12	1.1071429	0.1468414	0.8922867	18	6	4	5	18	8
RN7SL146P	0	#NUM!	0.824326	0	1	0	0	0	0
AC103564.3	0	#NUM!	0.6840298	0	0	2	0	0	0
AC026458.1	0	#NUM!	0.824326	0	0	1	0	0	0
RN7SL145P	3.75	1.9068906	0.2574882	3	1	0	4	0	11
AL512604.2	0	#NUM!	0.824326	0	1	0	0	0	0
AL512604.3	0	#NUM!	0.6631647	1	0	1	0	0	0
AC098869.2	0.5172414	-0.95109	0.4171074	12	4	13	0	3	12
GNG5P2	#DIV/0!	#DIV/0!	0.4737294	0	0	0	0	4	0
IQCB2P	0	#NUM!	0.5781912	0	0	3	0	0	0
CXCL17	7.5	2.9068906	0.0933912	0	2	0	7	5	3
CXCL16	0.9318182	-0.10188	0.9659214	20	27	41	27	28	27
COX4I1P2	1.8	0.8479969	0.6069077	0	0	5	1	6	2
EXOC5P1	0.6	-0.736966	0.7269658	3	1	1	0	1	2
CR769775.4	3	1.5849625	0.6896151	1	0	0	0	3	0

PRPF4B	0.9859844	-0.020363	0.8591604	2400	1179	2557	1442	2321	2287
GVINP1	0.7222222	-0.469485	0.7145137	7	4	7	5	6	2
DSC1	0.9836066	-0.023847	0.941697	45	10	6	8	25	27
DSC2	0.9634534	-0.053713	0.9692009	771	382	735	398	726	695
DSC3	0.7010024	-0.512509	0.0091157	2175	1629	1982	871	1454	1731
TROAP	0.9928175	-0.0104	0.703185	982	919	1162	927	1120	994
GLIS3	0.8454106	-0.242276	0.5417997	74	66	67	33	85	57
PDSS1P2	2.7	1.4329594	0.1930987	6	0	4	11	13	3
GLIS1	#DIV/0!	#DIV/0!	0.6099294	0	0	0	2	0	0
GLIS2	0.9228111	-0.115893	0.704615	277	328	263	239	291	271
DSEL	1.2222222	0.2895066	0.1910518	180	102	150	142	213	173
AC021237.1	0	#NUM!	0.824326	0	0	1	0	0	0
E4F1	0.9002338	-0.151628	0.5194878	738	843	985	727	813	770
AICDA	0.3333333	-1.584963	0.4062209	2	7	0	0	1	2
CXCL10	2.8125	1.4918531	0.0977855	1	11	4	29	9	7
CXCL11	#DIV/0!	#DIV/0!	0.0412138	0	0	0	7	1	3
AC133644.2	#DIV/0!	#DIV/0!	0.3831122	0	0	0	0	5	0
ARHGAP11A	1.1374264	0.1857732	0.1630713	2243	1421	2281	1647	2619	2496
ARHGAP11B	1.0803238	0.1114638	0.4019512	587	373	646	416	606	713
AC016576.1	0	#NUM!	0.6840298	0	0	2	0	0	0
AC015987.1	2	1	0.8186965	0	0	1	0	0	2
NT5C3AP1	0.6	-0.736966	0.7604905	1	1	3	1	1	1
.AMTOR5-AS	0.8532609	-0.228941	0.6645312	61	38	85	24	67	66
DSG1	2.25	1.169925	0.341291	4	1	3	3	11	4
DSG2	0.8876237	-0.17198	0.4093068	5548	4285	5624	3006	5231	5483
DSG3	1.75	0.8073549	0.6834697	4	0	0	5	2	0
AC125494.1	0.3809524	-1.392317	0.3052399	5	4	12	5	2	1
AC125494.2	1.0714286	0.0995357	0.829911	3	1	10	7	0	8
COPS8P2	1	0	0.9645753	0	8	1	1	7	1
CTHRC1	1.6666667	0.7369656	0.7011525	2	0	1	5	0	0
MMAA	1.1515152	0.2035334	0.4380243	60	46	59	62	60	68
MMAB	0.7933818	-0.333913	0.1992302	350	396	493	377	330	276
DHODH	0.8363636	-0.257798	0.1424906	863	879	1063	742	804	800
AC099552.1	0.1818182	-2.459432	0.1984645	4	3	4	1	0	1
AC099552.4	1.1	0.1375035	0.9005066	1	7	2	4	0	7
PTGES3P2	0.5	-1	0.8652929	0	0	2	0	0	1
PTGES3P3	1	0	0.9966253	2	0	1	0	1	2
PTGES3P4	#DIV/0!	#DIV/0!	0.8030358	0	0	0	1	0	0
PTGES3P1	0.9887892	-0.016265	0.9010878	139	142	165	110	172	159
AP000550.1	#DIV/0!	#DIV/0!	0.6099294	0	0	0	2	0	0
AP000550.4	2.4705882	1.3048546	0.1086188	2	2	13	17	15	10
AC099791.2	#DIV/0!	#DIV/0!	0.3981469	0	0	0	4	0	0
TMEM88B	#DIV/0!	#DIV/0!	0.5509701	0	0	0	0	3	0
AC004039.1	0.5	-1	0.524048	3	2	11	0	2	6
TMEM87A	0.9231446	-0.115371	0.6183708	1124	1053	1232	932	1160	1055
TMEM87B	0.9032022	-0.146879	0.4877534	1388	1142	1561	945	1427	1323
AL157788.1	#DIV/0!	#DIV/0!	0.1958503	0	0	0	0	4	3
DSN1	1.0274488	0.0390665	0.3965813	1725	1532	2062	1692	1980	1793
PCAT19	0.2	-2.321928	0.4539573	2	0	3	1	0	0
AC068896.1	0.7692308	-0.378512	0.818151	0	6	7	1	4	5
TMEM86A	1.2680412	0.3426017	0.3547634	18	36	43	47	30	46
TMEM86B	0.9444444	-0.082462	0.9828374	5	5	8	7	2	8
ZNF680P1	0	#NUM!	0.824326	0	0	1	0	0	0
GLIPR1L1	0	#NUM!	0.1701088	3	0	5	0	0	0

PCAT29	5.5	2.4594316	0.1954188	2	0	0	5	1	5
AC136285.1	#DIV/0!	#DIV/0!	0.6411933	0	0	0	0	0	2
AC136285.2	0.5	-1	0.8652929	0	0	2	0	1	0
REV3L	1.0216867	0.0309529	0.6758386	772	489	814	501	857	762
UTP14A	0.8048714	-0.31317	0.0275642	2172	2390	2746	1771	2159	1952
AC079858.1	#DIV/0!	#DIV/0!	0.8030358	0	0	0	0	1	0
UTP14C	1.0213108	0.030422	0.6763626	929	561	997	592	1014	934
AL157392.4	0	#NUM!	0.1111285	3	0	7	0	0	0
AL157392.2	0	#NUM!	0.0833862	4	6	0	0	0	0
AL157392.3	0.78125	-0.356144	0.3096113	257	110	241	128	148	199
AC090198.1	1.1153846	0.1575413	0.789423	14	21	17	7	19	32
PGRMC1	0.8551331	-0.225779	0.1078139	5077	4792	5911	3794	5021	4679
AC137894.1	0	#NUM!	0.2325162	2	4	0	0	0	0
PGRMC2	1.2191901	0.2859231	0.0047839	1807	1875	1998	2034	2598	2293
DSPP	#DIV/0!	#DIV/0!	0.8030358	0	0	0	0	1	0
SAP18P3	#DIV/0!	#DIV/0!	0.5415473	0	0	0	0	2	1
SAP18P2	0.5714286	-0.807355	0.6991413	1	5	1	3	1	0
AC009229.4	0	#NUM!	0.824326	0	0	1	0	0	0
AC009229.2	3.5	1.8073549	0.5234342	2	0	0	0	7	0
MIR1910	0.75	-0.415037	0.9340195	0	1	3	2	0	1
SNIP1	0.7559623	-0.403614	0.022618	542	607	654	365	502	496
AC104393.1	0	#NUM!	0.4496863	0	1	3	0	0	0
PIN4P1	1.5	0.5849625	0.742109	3	0	1	2	2	2
AL034549.2	0.762963	-0.390315	0.4866908	49	42	44	39	51	13
AL034549.1	0.7435897	-0.427421	0.7013257	14	2	23	8	13	8
AP002360.2	1.0725594	0.1010575	0.4831719	151	302	305	308	258	247
AP002360.3	1.5	0.5849625	0.8194094	0	0	2	0	0	3
AP002360.1	0.8883929	-0.17073	0.7100166	73	66	85	58	80	61
MIATNB	0.8153846	-0.294447	0.7367324	17	32	16	29	11	13
AP002360.4	1.44	0.5260688	0.4665859	8	10	7	15	14	7
AL390961.3	1	0	0.978278	0	0	2	0	2	0
AL390961.1	0.1428571	-2.807355	0.0019327	14	22	20	1	3	4
AL390961.2	2.3333333	1.2223924	0.4358393	2	0	4	0	8	6
SLC52A2	0.9003295	-0.151475	0.62588	650	897	881	789	764	633
SLC52A3	1.1761658	0.2340915	0.278899	99	153	134	134	182	138
DSTN	1.0249717	0.035584	0.3553016	8649	8334	10368	8552	10183	9299
SLC52A1	0.4545455	-1.137504	0.036355	36	36	60	23	27	10
AL512844.1	0.8	-0.321928	0.8946757	1	1	3	0	2	2
AL512844.2	0.3333333	-1.584963	0.6587914	2	0	1	1	0	0
AC091132.4	1.6666667	0.7369656	0.6866863	0	0	3	5	0	0
PSIP1	1.3040481	0.3829971	0.0017859	4533	3089	4853	4562	6159	5547
AC018737.2	0.5	-1	0.8143938	2	0	0	1	0	0
PSMA6P1	1.2972973	0.3755091	0.291338	28	45	38	49	52	43
AC091132.1	0.75	-0.415037	0.8897197	0	2	2	0	0	3
DYNLT1	0.8275626	-0.27306	0.0979781	1309	1434	1647	1165	1315	1153
DYNLT3	1.302999	0.3818359	0.0131677	342	269	356	411	473	376
MMP2	1.7516779	0.8087375	0.0255343	22	57	70	61	101	99
MMP3	0	#NUM!	0.5387425	3	0	0	0	0	0
AF279873.1	0.25	-2	0.5132079	2	2	0	0	0	1
MMP9	1	0	0.9962929	6	1	0	2	2	3
CDHR2	0.25	-2	0.196679	6	4	6	1	0	3
CDHR3	1.5714286	0.6520767	0.310037	4	9	22	13	27	15
CDHR5	0.7076923	-0.498806	0.4811811	12	35	18	3	18	25
\ADACL2-AS	1	0	0.9890559	1	0	0	0	1	0

AC105137.3	0.4	-1.321928	0.5141719	0	7	3	0	0	4
BSN-AS2	3.8333333	1.9385995	0.0909426	1	1	4	7	6	10
EPS8L1	1.0089564	0.0128639	0.5270708	2198	2356	2815	2549	2508	2378
EPS8L2	0.6443327	-0.634122	8.52E-06	4823	5471	6213	3576	3849	3211
EPS8L3	0	#NUM!	0.6385096	0	2	0	0	0	0
AC008735.1	0.4411765	-1.180572	0.302441	2	12	20	8	3	4
YTHDF3-AS1	1.0151515	0.0216951	0.9557614	18	36	12	20	25	22
AC008735.2	0.8787879	-0.186413	0.8654708	15	7	11	7	7	15
API5P1	#DIV/0!	#DIV/0!	0.5371397	0	0	0	0	0	3
AC008735.4	1.3285714	0.4098758	0.3444803	30	16	24	33	31	29
API5P2	0	#NUM!	0.824326	1	0	0	0	0	0
MIR1915	0.8125	-0.29956	0.8463885	13	1	2	7	4	2
TLNRD1	0.8028169	-0.316857	0.1273285	744	968	915	745	714	650
DENND5B-AS	0.8636364	-0.211504	0.8805666	9	14	21	15	7	16
CDK20	1.0402414	0.0569184	0.6483043	111	198	188	177	193	147
IAH1	0.3965517	-1.334419	0.0676866	15	22	21	5	8	10
MSRB1P1	#DIV/0!	#DIV/0!	0.8030358	0	0	0	0	1	0
CDKN2B-AS1	0.7428571	-0.428843	0.656027	18	3	14	5	15	6
AC099063.4	#DIV/0!	#DIV/0!	0.6482162	0	0	0	0	1	1
AC018738.1	0.9528302	-0.069709	0.9174645	17	36	53	49	23	29
PSMG3-AS1	1.0422535	0.0597062	0.8596937	16	36	19	25	26	23
CDK17	1.0061183	0.0087999	0.7404103	1092	709	1141	715	1120	1125
CDK18	0.6950092	-0.524896	0.0577634	184	188	169	122	157	97
CDK19	1.2223595	0.2896686	0.0613201	624	416	579	510	698	771
FHDC1	1.3657905	0.4497362	5.09E-05	2512	2435	2776	2796	3828	3924
CDK10	1.0115962	0.0166335	0.5535501	838	932	1162	1038	1028	900
CDK12	0.8868702	-0.173205	0.3845769	3514	3379	3785	2158	3385	3927
AC026803.1	2	1	0.7782384	0	0	1	2	0	0
CDK13	0.8963945	-0.157794	0.3931395	1577	1525	1724	1092	1658	1576
AC026803.2	0.4468085	-1.162271	0.150329	10	18	19	7	7	7
CDK14	0.9392396	-0.090435	0.7582118	1048	836	1062	644	1062	1061
CDK16	1.0288751	0.0410678	0.3963108	4104	4818	5381	4509	5349	4858
AC009562.1	0	#NUM!	0.824326	0	0	1	0	0	0
GCNT1	1.4178545	0.5037095	4.09E-05	865	745	1056	1022	1402	1356
GRAMD4	0.9268745	-0.109554	0.6585851	1619	1767	2002	1407	1718	1869
PATE2	2	1	0.7349626	0	2	0	1	3	0
GCNT2	0.8948127	-0.160342	0.5837601	220	248	226	192	217	212
GCNT3	1	0	0.926424	5	0	16	2	9	10
DTD1	0.9915819	-0.012196	0.7217162	813	968	1070	970	987	870
GCNT7	0.25	-2	0.0799111	10	20	2	2	1	5
DTD2	0.7845862	-0.349996	0.0620147	494	519	570	314	474	454
SAAL1	1.0506934	0.0713418	0.2894324	1349	1269	1564	1440	1562	1392
TRMT1	0.8343517	-0.261273	0.1843905	457	473	543	378	451	400
TOLLIP-AS1	0.3461538	-1.530515	0.061701	14	14	24	6	4	8
TRMT5	1.2405012	0.3109231	0.0142842	900	665	909	901	1187	981
TRMT6	0.8756674	-0.191545	0.3242801	1747	1395	2102	1288	1773	1531
AC115989.1	0	#NUM!	0.5781912	0	0	3	0	0	0
AC115989.2	1	0	0.9890559	0	1	0	0	0	1
AC012462.1	0	#NUM!	0.824326	0	0	1	0	0	0
AC009812.3	0.5111111	-0.968291	0.1519895	27	12	51	14	15	17
AC012462.3	#DIV/0!	#DIV/0!	0.8030358	0	0	0	1	0	0
AC009812.1	0.6666667	-0.584963	0.6755833	3	4	17	5	3	8
AC009812.4	0.7783784	-0.361456	0.4636163	62	42	81	53	56	35
RN7SL155P	0	#NUM!	0.824326	0	0	1	0	0	0

RN7SL154P	0	#NUM!	0.824326	0	0	1	0	0	0
AC092115.3	0.6470588	-0.628031	0.7211481	2	7	8	9	1	1
AC092115.1	1.0909091	0.1255309	0.9286464	4	5	2	2	6	4
IAPP	#DIV/0!	#DIV/0!	0.8030358	0	0	0	0	1	0
MALAT1	0.8221429	-0.282539	0.5867421	2026	920	2654	1176	1864	1564
MALAT1	0.8221429	-0.282539	0.5867421	2026	920	2654	1176	1864	1564
WDTC1	1.0095207	0.0136705	0.6552871	888	1064	1094	873	1030	1172
IARS	1.1242221	0.168927	0.0900553	8484	6952	8827	7086	10014	10177
AL512353.1	0	#NUM!	0.6385096	0	2	0	0	0	0
AL512353.2	2	1	0.8147319	1	0	0	0	0	2
AL512353.3	#DIV/0!	#DIV/0!	0.5415473	0	0	0	0	2	1
PATL1	0.7625369	-0.391121	0.0078693	2163	2241	2376	1355	1882	1933
PATL2	1.028777	0.0409303	0.8308277	27	49	63	26	49	68
AL137918.1	0.3333333	-1.584963	0.6805352	0	1	2	0	0	1
MND1	1.2114904	0.276783	0.0845334	474	283	444	454	557	444
MNDA	#DIV/0!	#DIV/0!	0.4737294	0	0	0	0	4	0
METAP1	1.2302158	0.2989114	0.0021189	3384	3060	3842	3492	4574	4588
METAP2	1.1387838	0.1874938	0.1083269	4189	2721	4453	3595	5137	4208
AC110801.1	#DIV/0!	#DIV/0!	0.3096126	0	0	0	5	0	0
CCDC13-AS1	0.8571429	-0.222392	0.9279667	3	1	3	0	0	6
UBAP2L	0.7452794	-0.424147	0.0005917	6350	6708	7702	4389	5598	5485
PIMREG	0.9456961	-0.080551	0.9154309	498	632	601	584	548	505
AL096869.3	0	#NUM!	0.824326	0	0	1	0	0	0
AP002851.1	0.75	-0.415037	0.8719866	0	3	1	0	0	3
AL096869.1	3.25	1.7004397	0.2439154	2	0	2	6	4	3
AC012368.1	0.3636364	-1.459432	0.2322898	10	5	7	0	5	3
AL096869.2	0	#NUM!	0.6631647	1	0	1	0	0	0
DUXAP8	1.0331551	0.0470568	0.6776092	334	272	329	218	346	402
DUXAP8	1.0331551	0.0470568	0.6776092	334	272	329	218	346	402
RNU6-240P	0	#NUM!	0.6436235	2	0	0	0	0	0
DUXAP9	0.8636364	-0.211504	0.5681335	127	101	146	52	136	135
AC007750.1	0.6666667	-0.584963	0.7729333	0	4	2	0	2	2
AP000705.1	1.2727273	0.3479233	0.7796991	6	0	5	1	6	7
AP000705.2	4	2	0.5246085	1	0	0	0	1	3
PRUNEP1	0	#NUM!	0.824326	1	0	0	0	0	0
PATZ1	0.8896396	-0.168707	0.4212849	678	716	826	606	737	632
SNHG1	1.1152272	0.1573377	0.0604318	2791	2600	3652	3090	3778	3217
DTNA	0.7110482	-0.491981	0.187053	162	89	102	39	97	115
DTNB	0.4545455	-1.137504	0.3675052	6	8	8	5	5	0
SNHG8	1.3512563	0.4343013	0.0023013	514	688	788	933	907	849
KTI12	0.8497409	-0.234905	0.4596638	207	270	295	244	231	181
SNHG9	1.6470588	0.7198921	0.4444322	8	2	7	3	10	15
SNHG6	1.2186848	0.285325	0.0163596	554	559	803	742	838	755
SNHG7	1.1590727	0.2129711	0.0720665	790	724	988	1051	1022	827
SNHG4	1.0524345	0.0737304	0.7665361	96	76	95	46	117	118
SNHG5	1.3258692	0.4069384	0.0025854	525	543	629	756	842	652
SNHG3	1.3312649	0.4127977	8.97E-05	1275	1248	1667	1763	1971	1844
DUXAP1	3	1.5849625	0.1927409	5	1	4	8	22	0
UBAP1L	0.8169014	-0.291766	0.6701657	36	24	82	11	52	53
AP000695.2	1	0	0.9683603	0	4	0	4	0	0
AC097491.1	#DIV/0!	#DIV/0!	0.4501902	0	0	0	0	0	4
FMR1NB	0	#NUM!	0.5075476	0	0	4	0	0	0
PRPF19	0.8359801	-0.258459	0.1091337	7186	8519	9231	6825	7476	6545
AP006621.2	0.6730769	-0.571157	0.4328577	23	11	18	8	15	12

AP006621.3	1.2035714	0.2673218	0.2406643	109	68	103	113	116	108
AP006621.1	0	#NUM!	0.824326	0	0	1	0	0	0
AP006621.4	0.25	-2	0.4710071	0	0	8	1	1	0
AP006621.5	0.8859649	-0.174679	0.7401789	60	64	104	53	76	73
AMPD2	0.9947781	-0.007553	0.7676876	325	371	453	322	424	397
AMPD3	1.0780669	0.1084467	0.6317917	95	99	75	87	114	89
AC084024.2	0	#NUM!	0.5643268	1	0	2	0	0	0
PRPF18	1.0930851	0.1284057	0.5443921	132	89	155	99	201	111
LYPLA2P2	#DIV/0!	#DIV/0!	0.8030358	0	0	0	0	0	1
LYPLA2P1	1.3333333	0.4150375	0.8762725	0	3	0	1	0	3
Z82205.1	#DIV/0!	#DIV/0!	0.8030358	0	0	0	0	0	1
AC005845.1	0	#NUM!	0.4185478	3	0	1	0	0	0
MAGI1-IT1	2.6666667	1.4150375	0.4915624	0	0	3	0	4	4
AC012618.2	1.75	0.8073549	0.6209551	5	1	2	0	12	2
AC012618.3	#DIV/0!	#DIV/0!	0.3109758	0	0	0	0	6	0
AC012618.1	0.2857143	-1.807355	0.437802	0	7	0	0	1	1
ZDHHC11B	4.5	2.169925	0.2798846	0	0	2	1	5	3
PRPF39	1.254389	0.3269848	0.0598286	1187	564	1154	976	1408	1260
PRPF31	1.010083	0.0144739	0.5833668	915	1090	1367	1321	1148	937
NEUROG2	1.25	0.3219281	0.7342232	2	1	5	6	1	3
CYP51A1-AS1	0.5	-1	0.8652929	0	0	2	0	1	0
NANOGNBP3	1.75	0.8073549	0.5409485	3	0	5	7	7	0
AC008734.1	0	#NUM!	0.6631647	1	0	1	0	0	0
TRNT1	0.8820059	-0.18114	0.4888997	744	487	803	519	727	548
AL133410.1	0.7777778	-0.36257	0.840271	4	1	4	0	2	5
RAI14	1.0591181	0.0828635	0.4020274	3497	2235	3453	2503	3749	3476
AC007906.2	0.7272727	-0.459432	0.3499852	47	44	52	34	25	45
LTBP4	0.9869828	-0.018903	0.7596104	582	750	819	742	692	689
DTX1	0.260274	-1.941897	0.0067622	16	29	28	7	6	6
AC009228.1	0	#NUM!	0.824326	1	0	0	0	0	0
DTX2	1.1840592	0.2437412	0.0868508	382	370	465	556	458	427
LTBP2	1	0	0.9559581	15	25	23	12	26	25
AC007906.1	0.6	-0.736966	0.8104482	2	0	3	2	0	1
DTX3	#DIV/0!	#DIV/0!	0.207136	0	0	0	0	8	0
LTBP3	1.1126943	0.1540573	0.3051871	245	194	333	361	280	218
DTX4	0.8459256	-0.241397	0.1319573	4684	5579	5580	3484	4947	4971
LTBP1	0.7550688	-0.40532	0.0132492	2111	2312	2334	1224	1895	1983
AC018832.1	0	#NUM!	0.824326	1	0	0	0	0	0
AC107072.1	#DIV/0!	#DIV/0!	0.8030358	0	0	0	0	0	1
AC010900.1	0.4705882	-1.087463	0.3921589	7	4	6	1	3	4
AC107072.2	2.5	1.3219281	0.5527374	0	0	2	3	2	0
AC005355.1	0.2	-2.321928	0.315708	10	0	0	0	2	0
AC005355.2	4	2	0.4419238	0	2	0	0	0	8
HSPE1P7	0.25	-2	0.5622508	0	1	3	1	0	0
HSPE1P8	#DIV/0!	#DIV/0!	0.6482162	0	0	0	0	1	1
GS1-124K5.4	0.6734694	-0.570316	0.3194029	34	31	33	26	16	24
HSPE1P2	0.7653061	-0.385891	0.6106843	21	22	55	28	19	28
AC122710.1	1.122449	0.1666499	0.7833717	8	27	14	12	23	20
AD000671.3	#DIV/0!	#DIV/0!	0.092722	0	0	0	7	0	2
AC122710.2	2	1	0.753159	0	2	0	0	0	4
HSPE1P6	0	#NUM!	0.824326	0	0	1	0	0	0
HSPE1P3	2.2941176	1.1979394	0.1623853	3	8	6	26	3	10
HSPE1P4	0.5	-1	0.6010971	0	5	7	4	1	1
AC090543.2	2	1	0.8324799	1	0	0	0	2	0

SENP6	1.0870871	0.1204675	0.3174302	1644	960	1725	1236	1868	1602
SENP7	1.1122449	0.1534745	0.4902447	356	153	373	182	436	363
SENP5	0.8136752	-0.297475	0.1106666	1268	947	1295	722	1035	1099
SENP8	1.0888889	0.1228567	0.6631423	53	44	38	49	43	55
FLOT1	0.9389157	-0.090932	0.898415	2544	3181	3590	3147	3034	2565
FLOT2	1.0717856	0.1000164	0.199599	4316	5184	5503	5289	5567	5224
SEPHS2P1	0	#NUM!	0.824326	1	0	0	0	0	0
GLIDR	6	2.5849625	0.2917671	0	1	0	2	2	2
AL139824.2	#DIV/0!	#DIV/0!	0.8030358	0	0	0	0	0	1
SENP2	0.9620945	-0.055749	0.9603289	1707	1817	2148	1452	2077	1928
SENP3	0.8816425	-0.181734	0.4775005	443	597	616	468	483	509
SENP1	0.9248489	-0.11271	0.6712533	934	747	967	672	879	898
GCOM2	1.0769231	0.1069152	0.8628056	4	4	5	6	2	6
TRNP1	0.72	-0.473931	0.6297757	9	9	7	2	6	10
MNS1	2.005102	1.0036757	0.0005322	63	40	93	149	115	129
GCOM1	0.6875	-0.540568	0.6902079	3	7	6	4	2	5
LTC4S	0.4634146	-1.109624	0.2231559	22	4	15	4	9	6
CDIPT	1.0229406	0.0327223	0.5283127	1386	1670	1739	1801	1706	1398
AC073343.2	0.8461538	-0.241008	0.932689	7	0	6	6	4	1
AC073343.1	7	2.8073549	0.2600307	0	0	1	3	0	4
AL590065.1	#DIV/0!	#DIV/0!	0.6411933	0	0	0	0	0	2
AC015691.1	5	2.3219281	0.4368246	0	0	1	0	3	2
SLC4A1AP	1.052	0.0731347	0.3126697	1563	1279	1908	1394	1803	1800
SYNGAP1	1.070122	0.0977752	0.5859629	116	107	105	124	84	143
MNX1	1.0126716	0.0181664	0.6641299	218	330	399	350	345	264
AC008972.2	0.5592105	-0.838537	0.0549532	46	56	50	21	34	30
AC008972.1	0.6415094	-0.640458	0.4265093	14	18	21	16	7	11
SHANK2-AS1	0	#NUM!	0.6604275	0	1	1	0	0	0
SHANK2-AS2	7	2.8073549	0.3457479	0	1	0	0	7	0
SDR42E1	15	3.9068906	0.0429341	1	0	0	5	1	9
SDR42E2	0.5	-1	0.8276497	1	0	1	0	0	1
IL31RA	3.8181818	1.9328858	0.0300563	6	2	3	11	19	12
AIF1L	1.0811401	0.1125534	0.1715756	4286	5270	5566	4803	5995	5551
DUBR	1.121372	0.165265	0.3824146	137	110	132	116	147	162
AC104297.1	0.6666667	-0.584963	0.7556168	3	3	0	0	1	3
MIR1268A	0	#NUM!	0.824326	0	1	0	0	0	0
RNU6-698P	0.2916667	-1.777608	0.1689107	4	11	9	4	3	0
AC107959.1	#DIV/0!	#DIV/0!	0.8030358	0	0	0	0	1	0
AC107959.2	0.6666667	-0.584963	0.8290852	1	2	0	1	1	0
AC107959.3	#DIV/0!	#DIV/0!	0.5338029	0	0	0	0	1	2
RAB3IL1	0.9715475	-0.041644	0.8983822	409	488	544	478	518	404
REG1CP	0	#NUM!	0.0109221	2	10	5	0	0	0
L2HGDH	0.9466328	-0.079123	0.7954129	750	821	790	590	825	820
ZDHHC20P1	0.5	-1	0.537342	12	0	4	0	8	0
ZDHHC20P4	0	#NUM!	0.2218177	2	0	5	0	0	0
NEURL1B	0.6481481	-0.625604	0.3865387	11	29	14	8	19	8
AC124276.1	0.5918367	-0.756729	0.3892136	8	26	15	7	21	1
DAB2IP	0.8892626	-0.169319	0.4255992	1191	1335	1637	1180	1290	1232
AL606834.2	1.3928571	0.4780473	0.3141832	47	34	31	20	78	58
ERVV-2	2.1861241	1.1283753	1.17E-23	917	942	1139	1955	2423	2176
AL606834.3	0.8275862	-0.273018	0.767219	8	13	8	5	8	11
ERVV-1	2.6611446	1.4120469	4.51E-17	215	170	279	551	655	561
CDKN3	0.9446715	-0.082115	0.8489713	1069	902	1210	885	1131	989
EXOC3L1	0.8215385	-0.2836	0.4129201	86	112	127	79	98	90

EXOC3L2	0.5	-1	0.8276497	1	0	1	0	0	1
EXOC3L4	0.5789474	-0.788496	0.5600543	3	5	11	0	4	7
MFSD13A	1.0273415	0.0389158	0.5090965	478	536	705	639	626	501
MFSD13B	0.8888889	-0.169925	0.925066	0	6	3	0	1	7
AC005840.1	1.3333333	0.4150375	0.8127785	0	0	3	1	0	3
AC231759.1	0.625	-0.678072	0.7529328	1	1	6	1	4	0
IBSP	0	#NUM!	0.3473206	0	5	0	0	0	0
AC005840.3	0.6785714	-0.559427	0.6699908	11	1	16	8	5	6
AC005840.5	3.25	1.7004397	0.3393351	0	0	4	0	3	10
PSKH1	0.8596491	-0.21818	0.2733108	692	875	884	610	726	771
CDKL2	0.5	-1	0.7214899	0	4	0	0	0	2
CDKL3	1.295082	0.3730434	0.4117368	18	19	24	31	14	34
CDKL4	1.2	0.2630344	0.8214561	1	0	4	2	1	3
CDKL5	0.9637681	-0.053242	0.9210042	234	140	178	96	197	239
POLRMT	0.9350881	-0.096826	0.8414161	1381	1807	1865	1650	1558	1517
CDKL1	0.4166667	-1.263034	0.2752291	9	7	8	0	4	6
KHDRBS1	1.019201	0.0274386	0.4347771	8846	9221	10369	8607	10458	9917
KHDRBS3	0.7747253	-0.368243	0.2529346	115	135	114	94	112	76
AGAP11	8.5	3.0874628	0.0982171	0	0	2	0	6	11
AL139082.1	1.6	0.6780719	0.6321097	1	0	4	3	1	4
MOBP	#DIV/0!	#DIV/0!	0.6554062	0	0	0	0	2	0
TMPRSS12	1.2857143	0.3625701	0.7490799	1	3	3	5	2	2
TMPRSS13	0.7025882	-0.509249	0.0008217	1860	2280	2235	1412	1660	1407
TMPRSS15	0	#NUM!	0.5200113	2	1	0	0	0	0
MOB4	1.2932019	0.3709475	0.0088351	718	467	742	772	889	831
IBTK	0.9986777	-0.001909	0.7980405	1196	710	1119	764	1134	1123
MOB2	1.1688852	0.2251332	0.1180501	308	394	500	531	446	428
TMEM51-AS1	2.1311475	1.0916305	0.0307021	19	23	19	19	58	53
RNU6-695P	0.1666667	-2.584963	0.4507335	0	0	6	0	0	1
SP140L	1.0953646	0.1314112	0.1530077	1172	982	1233	1084	1370	1256
CR786580.1	0	#NUM!	0.6436235	2	0	0	0	0	0
AC097059.1	#DIV/0!	#DIV/0!	0.4098001	0	0	0	1	3	0
MBL1P	1.4166667	0.5025003	0.6714011	0	9	3	7	7	3
AC097059.2	0.8333333	-0.263034	0.8575958	5	2	5	0	5	5
AC245452.2	1.5714286	0.6520767	0.5187298	0	4	10	6	12	4
AC245452.1	0.6826347	-0.550814	0.1965636	64	46	57	37	43	34
MMS22L	0.978295	-0.031659	0.907518	1357	969	1498	911	1413	1417
GTPBP10	0.9580493	-0.061828	0.935765	701	495	711	481	732	614
ELOCP2	0.7142857	-0.485427	0.5065482	35	14	35	27	23	10
AC008149.1	#DIV/0!	#DIV/0!	0.5509701	0	0	0	0	3	0
MFSD14A	0.5882353	-0.765535	0.5682809	4	6	7	2	8	0
MFSD14B	1.0678049	0.094648	0.2820342	3336	3286	3628	2808	4260	3877
MFSD14C	1.1358867	0.1838189	0.0837904	613	594	699	695	767	703
HNRNPRP1	0.0588235	-4.087463	0.0312355	5	5	7	0	1	0
SMARCB1	0.8933126	-0.162763	0.4844265	1968	2368	2722	2130	2141	2034
AC005746.2	2.3333333	1.2223924	0.5495839	0	3	0	3	1	3
AC005746.3	0.1666667	-2.584963	0.3219086	3	1	2	1	0	0
AC005746.1	0.3333333	-1.584963	0.2334644	4	8	6	2	1	3
ELOBP4	0	#NUM!	0.2359908	1	0	6	0	0	0
ELOBP2	0	#NUM!	0.5781912	0	0	3	0	0	0
IFNL4P1	0.0714286	-3.807355	0.0571828	6	7	1	0	0	1
PTGFRN	2.3333333	1.2223924	0.2914246	7	1	1	11	3	7
SMARCC1	0.8824731	-0.180376	0.28851	8250	7624	9099	5521	8076	8441
SMARCC2	0.674904	-0.567246	0.0004906	2372	2693	2745	1258	1905	2108

TALDO1	1.0006631	0.0009564	0.6272113	9053	11448	12674	11230	12049	9918
TRAF3IP2-AS1	1.124031	0.1686818	0.5219111	36	39	54	53	45	47
MOGS	0.9826735	-0.025216	0.7544649	3181	3466	4088	3302	3806	3441
AL157394.1	0	#NUM!	0.824326	1	0	0	0	0	0
AC023632.6	1.0147059	0.0210616	0.9115418	33	14	21	20	29	20
AL132821.1	1.6666667	0.7369656	0.7533214	0	2	1	1	4	0
AC023632.5	0	#NUM!	0.824326	0	0	1	0	0	0
AC023632.2	0.5714286	-0.807355	0.7098946	1	3	3	2	0	2
AC023632.1	0.5	-1	0.812391	0	2	0	0	1	0
AP002754.1	1	0	0.9885287	9	0	0	1	1	7
AP002754.2	0	#NUM!	0.3026413	2	3	0	0	0	0
SERPINB9P1	1.3	0.3785116	0.6543989	5	11	4	9	6	11
RANBP17	0.7800329	-0.358393	0.0922659	632	436	755	398	548	476
SMARCA5	1.432146	0.5181786	0.0001519	5095	3222	5271	5380	7498	6582
SMARCA1	1.7456911	0.8037983	8.50E-07	2229	1287	2402	2631	3984	3716
SMARCA2	1.1547339	0.2075604	0.0571469	5470	5025	6056	4704	7070	7338
SMARCA4	0.8702135	-0.200559	0.2246082	4223	4956	5106	3813	4285	4333
ECHDC1	1.0079811	0.0114686	0.6030722	1116	901	1366	1015	1329	1066
RANBP10	0.8443629	-0.244065	0.1319283	1944	2232	2095	1522	1905	1868
ECHDC2	0.8263473	-0.27518	0.1866885	1216	1079	1713	1099	1225	988
ECHDC3	0.7863578	-0.346742	0.0895072	454	542	558	414	418	390
NOL10	1.0417259	0.0589758	0.385107	1446	1075	1697	1341	1650	1403
NOL11	0.9248292	-0.112741	0.6601952	2631	1994	2838	1954	2585	2363
NOL12	0.5597484	-0.83715	0.083632	51	47	61	33	41	15
SUCLG2-AS1	1.25	0.3219281	0.8339332	6	5	1	1	9	5
ICA1	0.7214485	-0.471032	0.1325581	129	101	129	80	107	72
MFSD1P1	0.2857143	-1.807355	0.4669419	1	6	0	2	0	0
DUS2	0.8714547	-0.198502	0.3563024	588	769	829	527	679	699
AC090152.1	0.6339869	-0.657475	0.1798248	48	78	27	22	31	44
AC011731.1	0	#NUM!	0.3320541	3	0	2	0	0	0
AC122713.2	3	1.5849625	0.5018318	1	1	0	2	4	0
AC008243.1	4	2	0.3261268	0	0	2	1	3	4
HSD17B3	0	#NUM!	0.5075476	0	0	4	0	0	0
HSD17B4	0.8482337	-0.237466	0.0969207	3932	3626	4388	2823	3694	3616
HSD17B6	0.6938776	-0.527247	0.0101029	327	293	360	178	268	234
HSD17B7	0.8800214	-0.184389	0.4193217	578	581	708	542	587	514
HSD17B8	1.2439024	0.3148733	0.2093057	118	121	89	113	134	161
RANBP3L	1.4	0.4854268	0.7338848	0	0	5	2	3	2
HSD17B1	1.2834076	0.3599794	0.0351651	473	621	702	949	787	569
HSD17B2	0	#NUM!	0.824326	1	0	0	0	0	0
CHRNA10	1.4512195	0.5372658	0.2510648	13	37	32	51	48	20
MON2	0.8747492	-0.193059	0.3964644	1349	924	1216	651	1195	1206
AC093579.1	0	#NUM!	0.6840298	0	0	2	0	0	0
NOL4L	0.6787879	-0.558967	0.1055339	82	127	121	60	107	57
AC113191.1	0.9097744	-0.136419	0.8030914	38	43	52	18	59	44
AL353719.1	1.2	0.2630344	0.8943342	1	4	0	2	2	2
ICE1	1.1021333	0.1402988	0.3371867	2953	1729	2818	1812	3140	3314
ICE2	1.010322	0.0148152	0.7438967	1002	528	892	647	958	842
LOXL3	1.1049724	0.1440103	0.5366888	61	43	77	56	76	68
LOXL4	0.2738095	-1.868755	0.0051481	35	25	24	8	5	10
LOXL1	0.5	-1	0.7019715	2	2	0	0	0	2
LOXL2	0.4038728	-1.308027	2.30E-11	383	515	548	166	220	198
TEKT2	0.5526316	-0.85561	0.3219284	8	16	14	3	8	10
TEKT3	2.6666667	1.4150375	0.3128097	1	1	4	6	10	0

DUXA	0.5	-1	0.81106	0	2	0	0	0	1
DUXB	5	2.3219281	0.3971277	1	0	0	2	0	3
TEKT5	1.4098361	0.4955274	0.1325596	37	36	49	52	68	52
AC073346.1	0.7924528	-0.335603	0.6644651	15	13	25	6	22	14
MORC3	1.3261759	0.4072721	0.0192755	737	395	824	673	976	945
MORC4	0.8381623	-0.254699	0.0736703	3993	3684	4403	2761	3730	3634
SHARPIN	0.9628339	-0.054641	0.9361731	481	585	656	597	598	463
AL133215.2	1.8842105	0.9139602	0.0123694	27	28	40	46	66	67
AL133215.3	0	#NUM!	0.6436235	2	0	0	0	0	0
VILL	1.3846154	0.4694853	0.4728909	4	9	13	19	9	8
MORC2	0.8602879	-0.217109	0.1631666	2206	2382	2498	1714	2200	2182
QSER1	0.9878493	-0.017637	0.9108966	2077	1107	1754	1108	1786	1984
AC005256.1	0	#NUM!	0.5781912	0	0	3	0	0	0
AL133215.1	0	#NUM!	0.824326	0	0	1	0	0	0
AC141586.5	0.2727273	-1.874469	0.3952749	2	0	9	0	3	0
AC141586.4	0.7142857	-0.485427	0.8597794	2	0	12	6	2	2
AC141586.1	0.9896194	-0.015054	0.8779026	86	114	89	117	88	81
AC141586.2	0.9230769	-0.115477	0.9631492	11	6	9	9	5	10
CR392039.3	0.445122	-1.167727	0.0137743	70	31	63	19	28	26
USMG5P1	1	0	0.9890559	1	0	0	0	0	1
CR392039.4	5	2.3219281	0.4650112	0	0	1	0	0	5
CR392039.1	0	#NUM!	0.1165453	4	2	2	0	0	0
VIL1	0.6	-0.736966	0.8417868	0	0	5	0	0	3
ACTG1P22	#DIV/0!	#DIV/0!	0.5070017	0	0	0	3	0	0
ACTG1P24	0.9090909	-0.137504	0.9477246	9	2	11	0	2	18
CDC42EP3P1	0.3333333	-1.584963	0.7307796	0	0	3	0	1	0
ACTG1P20	0.84	-0.251539	0.821497	7	10	8	1	3	17
RB1CC1	1.1564048	0.2096465	0.221809	543	264	606	427	642	565
AL355377.2	2.5	1.3219281	0.5413975	0	0	2	4	1	0
DSCR4-IT1	#DIV/0!	#DIV/0!	0.8030358	0	0	0	0	0	1
AC079612.1	0	#NUM!	0.824326	0	0	1	0	0	0
AC004918.1	0.4	-1.321928	0.5170014	5	0	5	1	0	3
AC004918.3	0.0526316	-4.247928	0.0264119	2	6	11	0	1	0
RAD51-AS1	0.79	-0.340075	0.2586114	177	131	192	126	122	147
CDKN2A-AS1	1	0	0.9890559	1	0	0	0	0	1
FO681548.1	2	1	0.6677248	2	1	0	0	2	4
AC093673.1	0.9358974	-0.095578	0.9600485	43	61	52	64	40	42
SEPHS1P1	#DIV/0!	#DIV/0!	0.8030358	0	0	0	1	0	0
SEPHS1P4	0.5	-1	0.8210345	0	1	1	1	0	0
SEPHS1P6	0	#NUM!	0.2117164	3	0	4	0	0	0
AC011481.3	14	3.8073549	0.0124845	0	0	2	6	3	19
PPP3CB-AS1	0.5272727	-0.923379	0.2127062	15	17	23	11	9	9
AC011481.1	0.7894737	-0.341037	0.7825895	4	5	10	1	10	4
AC011481.2	0	#NUM!	0.6385096	0	2	0	0	0	0
ACTG1P14	1.2222222	0.2895066	0.8051987	15	1	2	10	12	0
ACTG1P10	0.25	-2	0.2669758	14	2	0	0	0	4
ACTG1P15	0.6	-0.736966	0.7715764	0	2	3	0	3	0
MORF4	0.4	-1.321928	0.610796	0	4	1	0	0	2
ACTG1P16	0.5	-1	0.8276497	1	0	1	0	0	1
TMEM183AP1	0	#NUM!	0.824326	0	0	1	0	0	0
ACTG1P17	1.1666667	0.2223924	0.799895	1	0	5	5	1	1
GOT2P2	0.5	-1	0.8652929	0	0	2	0	1	0
GOT2P3	6	2.5849625	0.3507552	1	0	0	0	3	3
DHRS4L2	#DIV/0!	#DIV/0!	0.2545643	0	0	0	0	4	2

Z92544.2	0.8947368	-0.160465	0.9735893	1	2	16	3	11	3
FNDC3B	0.9132375	-0.130938	0.5382798	4257	3728	4117	2448	4138	4466
FNDC3A	1.2892237	0.3665026	0.0459731	1271	720	1062	877	1452	1607
ERVW-1	1.7931894	0.8425279	7.35E-13	768	752	888	1245	1616	1457
RNPC3	1.0334672	0.0474926	0.6391026	290	143	314	217	290	265
FLRT2	0.6086957	-0.716207	0.5502761	2	14	7	7	5	2
FLRT3	0.971831	-0.041223	0.9984591	29	13	29	12	29	28
FLRT1	2.7647059	1.467126	0.1184525	0	13	4	21	18	8
AC103957.2	0.6153846	-0.70044	0.4550124	13	10	29	16	5	11
ICOS	0	#NUM!	0.824326	0	0	1	0	0	0
STARD13	0.775	-0.367732	0.0626842	458	405	497	262	399	393
ICMT	0.9891405	-0.015753	0.7980754	5405	6593	6419	5286	6532	6399
STARD10	1.1474994	0.1984934	0.0837051	2362	2872	3444	3702	3488	2768
NEXN-AS1	0	#NUM!	0.5436524	2	0	1	0	0	0
AC103563.4	0	#NUM!	0.4217812	0	0	5	0	0	0
FNDC10	0.9729064	-0.039627	0.8800108	114	112	180	133	145	117
FNDC11	#DIV/0!	#DIV/0!	0.4501902	0	0	0	0	0	4
AC103563.3	0.3529412	-1.5025	0.3301737	11	1	5	1	0	5
VSIG10	0.6928591	-0.529366	0.0002536	1087	1145	1395	703	929	881
AIFM3	0.125	-3	0.2829921	0	1	7	1	0	0
AIFM1	0.8532476	-0.228964	0.1766063	2237	2587	2951	2094	2254	2286
AIFM2	2.1111111	1.0780025	0.4427488	0	0	9	3	0	16
AC010653.1	0	#NUM!	0.6840298	0	0	2	0	0	0
COL10A1	0.0714286	-3.807355	0.0579021	4	4	6	0	0	1
AL512605.1	0	#NUM!	0.5387425	3	0	0	0	0	0
AC010653.3	1.75	0.8073549	0.6234885	0	0	4	5	2	0
AC009320.1	#DIV/0!	#DIV/0!	0.3981469	0	0	0	4	0	0
AC027288.3	1.6	0.6780719	0.4353246	1	2	12	12	3	9
AL390866.1	#DIV/0!	#DIV/0!	0.5338029	0	0	0	0	1	2
HEATR5A	1.0252594	0.0359889	0.6277369	856	514	847	587	809	877
HEATR5B	1.0657248	0.091835	0.460724	1234	833	1189	793	1243	1434
LUADT1	0	#NUM!	0.6436235	2	0	0	0	0	0
AC027288.1	0	#NUM!	0.824326	0	0	1	0	0	0
AC027288.2	#DIV/0!	#DIV/0!	0.6099294	0	0	0	2	0	0
AC098868.1	0	#NUM!	0.5172237	1	2	0	0	0	0
AC009716.1	0	#NUM!	0.5075476	0	0	4	0	0	0
DHRSX	0.7171183	-0.479717	0.0114547	440	455	618	339	405	341
AC096719.1	0.6923077	-0.530515	0.8546155	1	1	11	6	0	3
AC008731.1	6	2.5849625	0.3849176	0	1	0	0	0	6
DHRS1	1.047619	0.0671142	0.4045816	431	435	562	531	509	456
DHRS2	1.0127119	0.0182238	0.6196415	410	576	666	608	577	488
DHRS3	0.9497258	-0.074417	0.9395219	327	376	391	350	346	343
DHRS4	1	0	0.9617562	0	0	2	0	0	2
DHRS7	1.0662936	0.0926047	0.245559	1361	1536	1749	1574	1864	1516
DHRS9	#DIV/0!	#DIV/0!	0.263214	0	0	0	0	5	1
AP006623.1	0.4597701	-1.121015	0.0930346	35	26	26	11	5	24
CYSLTR1	#DIV/0!	#DIV/0!	0.6411933	0	0	0	0	0	2
CYSLTR2	#DIV/0!	#DIV/0!	0.3109758	0	0	0	0	6	0
AC010809.2	2	1	0.5603448	5	0	0	4	2	4
AC010809.3	0	#NUM!	0.824326	0	0	1	0	0	0
MPC1	0.7881811	-0.343401	0.1227176	405	394	504	325	427	275
MPC2	1.2073955	0.2718983	0.1138154	175	171	276	289	235	227
AC010809.1	1	0	0.9013847	12	2	19	9	10	14
MB21D2	0.7586806	-0.398436	0.0293957	495	583	650	389	507	415

AC063950.1	0.875	-0.192645	0.9386562	5	0	3	0	0	7
AC007655.1	#DIV/0!	#DIV/0!	0.242062	0	0	0	6	0	0
STAMBP	0.9062552	-0.142011	0.4370733	1860	1935	2200	1565	2034	1834
PCDHGB9P	0.4642857	-1.106915	0.1536374	12	30	14	9	5	12
DVL1	1.1461308	0.1967717	0.0461481	1652	1919	2102	2122	2334	2046
DVL2	0.8208245	-0.284854	0.1046832	1120	1286	1378	1029	1058	1019
DVL3	0.8382735	-0.254507	0.0676893	8898	9669	10717	6960	8684	8904
MPDZ	1.2119904	0.2773783	0.0865961	1538	1056	1576	1070	1919	2065
AC234775.2	0	#NUM!	0.824326	1	0	0	0	0	0
AC234775.3	0.8888889	-0.169925	0.9064223	1	5	3	0	4	4
ADAMTS4	0.2	-2.321928	0.5227064	0	0	5	1	0	0
ADAMTS5	0.8181818	-0.289507	0.7225788	9	18	28	5	26	14
ADAMTS2	0.6666667	-0.584963	0.8754808	0	3	0	2	0	0
ADAMTS3	1.3362416	0.4181809	0.0137434	506	363	621	454	761	776
ADAMTS1	1.3489905	0.4318802	0.0002821	2318	2282	2681	2463	3619	3740
DDX11L16	0.4444444	-1.169925	0.560104	1	8	0	3	1	0
RNPEP	0.792454	-0.335601	0.0304127	18144	21570	22836	16727	17152	15689
ADAMTS8	0.1666667	-2.584963	0.4507335	0	0	6	0	0	1
AC034244.2	0.4	-1.321928	0.6857659	0	0	5	0	2	0
ADAMTS9	0.7925697	-0.33539	0.1449649	355	287	327	176	306	286
ADAMTS6	1.0296053	0.0420913	0.7214396	113	70	121	85	113	115
ADAMTS7	0.6785714	-0.559427	0.012098	242	296	358	165	248	195
DDX11L10	2.3333333	1.2223924	0.5428285	2	1	0	1	2	4
TRPC4	1.6666667	0.7369656	0.7294872	0	2	1	1	3	1
TRPC1	1.0646154	0.0903323	0.6106776	118	91	116	87	120	139
TRPC2	1	0	0.9798245	6	5	1	6	4	2
PCDHGB8P	0.3703704	-1.432959	0.1184775	33	16	5	0	9	11
AL157884.1	#DIV/0!	#DIV/0!	0.8030358	0	0	0	0	0	1
POLR1A	0.7442363	-0.426167	0.0099032	3702	4627	4640	2339	3512	3801
POLR1B	0.9220885	-0.117023	0.586205	1981	1943	2224	1405	2113	2151
POLR1C	0.9133618	-0.130742	0.6866122	497	527	615	516	537	444
POLR1D	0.9963336	-0.005299	0.6479959	3208	3549	3880	3563	3674	3361
POLR1E	1.018849	0.0269402	0.522267	1260	1258	1461	1140	1469	1445
NUFIP1	0.931367	-0.102578	0.7323413	606	534	623	454	567	621
NUFIP2	0.8960217	-0.158394	0.4661595	7733	5627	7654	4274	6957	7598
POLR2A	0.7808533	-0.356877	0.0232321	7249	9046	8597	4980	7093	7364
AC114760.2	0.3571429	-1.485427	0.3367959	4	3	7	0	2	3
POLR2B	1.0703448	0.0980757	0.3480097	3353	2638	3434	2393	3821	3874
POLR2C	0.7990431	-0.323655	0.0302086	1823	2079	2159	1471	1802	1570
POLR2D	0.9053222	-0.143497	0.4793309	2107	2408	2794	1944	2308	2365
POLR2E	0.9849769	-0.021838	0.7481268	3339	4064	4512	4168	4174	3394
POLR2F	0.8	-0.321928	0.9107417	10	0	0	7	0	1
POLR2G	0.8682189	-0.203869	0.3220016	1195	1513	1587	1235	1334	1160
CU459211.1	10	3.3219281	0.1213352	0	0	1	2	3	5
POLR2H	1.0527316	0.0741376	0.3125115	1838	2035	2442	2241	2491	1916
POLR2I	1.4444444	0.5305147	0.3190836	12	17	16	32	18	15
POLR2J	1.0034686	0.0049955	0.6102366	825	926	1132	1048	966	879
POLR2K	1.0600726	0.0841631	0.2604945	1117	1047	1415	1069	1417	1308
KLRA1P	1.4516129	0.5376568	0.2809195	26	7	29	25	37	28
POLR2L	0.9469981	-0.078567	0.999891	1755	2090	2551	2297	2147	1613
POLR2M	0.9527594	-0.069816	0.9071885	814	636	815	642	831	685
TRPA1	0.1764706	-2.5025	0.0288464	12	5	17	2	3	1
AP000356.1	0	#NUM!	0.824326	0	0	1	0	0	0
AP000356.2	1.0526316	0.0740006	0.8422476	1	8	10	11	3	6

POLR3A	1.0100371	0.0144083	0.6431785	1346	1577	1660	1255	1693	1681
POLR3B	0.8653614	-0.208625	0.258446	1491	1168	1478	915	1385	1280
POLR3C	0.7525376	-0.410164	0.0137988	811	943	1103	583	772	795
POLR3D	0.8519382	-0.231179	0.1930657	1004	1156	1271	802	1031	1090
POLR3E	0.8211057	-0.28436	0.0601983	1713	1811	2228	1450	1673	1600
POLR3F	0.9096902	-0.136553	0.6804611	555	356	606	397	517	466
POLR3G	1.2113503	0.2766161	0.2226133	190	108	213	130	232	257
POLR3H	0.9108527	-0.13471	0.6316872	847	962	1029	884	847	854
POLR3K	0.7348571	-0.444464	0.0467164	266	304	305	213	219	211
KLHDC7A	1.005988	0.0086131	0.8576388	45	59	63	46	51	71
KLHDC7B	0.8	-0.321928	0.8877973	1	3	1	2	2	0
AC016924.1	0.75	-0.415037	0.6543404	12	11	17	9	6	15
AL606489.2	0	#NUM!	0.6840298	0	0	2	0	0	0
AL606489.1	0.9090909	-0.137504	0.8719885	25	5	3	5	8	17
AL391704.1	0	#NUM!	0.824326	1	0	0	0	0	0
ZW10	0.9592014	-0.060094	0.9904115	1146	918	1392	895	1231	1189
TRPM1	0.7777778	-0.36257	0.8534788	1	2	6	0	7	0
TRPM2	3.0526316	1.6100535	0.024049	5	9	5	21	21	16
TRPM7	1.1046741	0.1436208	0.327837	1857	939	1761	1230	1971	1833
TRPM8	0	#NUM!	0.6840298	0	0	2	0	0	0
TRPM5	#DIV/0!	#DIV/0!	0.1024606	0	0	0	3	0	6
TRPM6	0.8132022	-0.298314	0.2481629	250	216	246	121	246	212
TRPM4	0.9004199	-0.15133	0.6292352	478	556	633	520	464	517
AC104640.1	#DIV/0!	#DIV/0!	0.5509701	0	0	0	0	3	0
KLHDC8B	0.8803264	-0.18389	0.5429393	310	350	443	333	313	325
AL022100.1	0.3333333	-1.584963	0.4118679	2	2	5	0	1	2
AC010319.4	1.3076923	0.3870231	0.5952539	2	14	10	12	9	13
AL022100.2	2	1	0.8147319	1	0	0	0	0	2
AC046158.2	0.3333333	-1.584963	0.7290749	0	0	3	0	0	1
AC046158.3	0	#NUM!	0.2919745	1	0	5	0	0	0
KLHDC8A	0	#NUM!	0.1179813	0	0	12	0	0	0
AC010319.1	1	0	0.9222636	4	4	9	6	5	6
RNPS1	0.9468085	-0.078855	0.8734191	9427	10355	11708	9619	10598	9598
AC046158.1	0	#NUM!	0.5781912	0	0	3	0	0	0
AGAP7P	0.7826087	-0.353637	0.9043208	2	6	15	14	1	3
AC012464.1	0.5	-1	0.7055109	0	2	4	0	0	3
AC012464.2	1	0	0.9555235	0	1	2	1	0	2
AL360270.3	0.4	-1.321928	0.3883716	5	6	4	4	0	2
AL360270.1	2.0606061	1.0430687	0.0784395	10	7	16	19	29	20
AL360270.2	1.106383	0.1458509	0.8232218	15	20	12	9	26	17
AC090403.1	0	#NUM!	0.6631647	1	0	1	0	0	0
ISPD-AS1	0.6	-0.736966	0.6385773	7	3	0	0	5	1
AC113192.1	#DIV/0!	#DIV/0!	0.6411933	0	0	0	0	0	2
VN1R54P	1.25	0.3219281	0.8749486	0	2	2	0	0	5
AL592466.1	0.375	-1.415037	0.4573733	2	4	2	1	2	0
MPND	0.9723926	-0.040389	0.8991122	86	97	143	101	109	107
OR7E12P	0.9230769	-0.115477	0.7955774	155	108	127	35	118	207
PTGER4	0.9726776	-0.039966	0.9836346	73	47	63	50	70	58
PTGER1	0	#NUM!	0.3473206	0	5	0	0	0	0
KRT7-AS	0.5932203	-0.75336	0.0888124	44	63	70	41	34	30
VN1R51P	2	1	0.7074237	2	0	0	2	2	0
TWISTNB	0.9941401	-0.008479	0.7631737	1451	956	1518	1062	1445	1395
CR382287.1	#DIV/0!	#DIV/0!	0.6554062	0	0	0	0	2	0
MPP1	0.9839767	-0.023304	0.740909	3418	3455	4111	3406	3899	3503

MPP2	0.672975	-0.571375	0.0042795	360	490	471	249	329	311
MPP3	0.3846154	-1.378512	0.0016377	50	74	71	25	19	31
MPP4	#DIV/0!	#DIV/0!	0.5082165	0	0	0	1	0	2
PTGES2	0.8599857	-0.217615	0.288652	1592	1808	2178	1656	1650	1491
PTGES3	1.0388968	0.0550523	0.2692589	17228	16983	20138	17493	20445	18525
PKDREJ	0.8148148	-0.295456	0.7532927	11	10	6	4	6	12
MPP5	1.0354167	0.0502114	0.5868785	1487	978	1375	965	1463	1548
AC092944.1	0.2727273	-1.874469	0.1484659	3	13	6	0	3	3
MPP6	0.9753309	-0.036036	0.949249	638	419	605	436	652	533
MPP7	1.3233112	0.4041524	0.0027565	1837	1405	1880	1649	2658	2471
KBTBD3	1.0689655	0.0962153	0.6771917	94	62	76	60	75	113
KBTBD4	0.890785	-0.166851	0.6736861	84	127	82	80	91	90
KBTBD6	0.8637795	-0.211265	0.3509829	410	427	433	245	436	416
KBTBD2	0.9625525	-0.055063	0.9763861	918	690	1009	616	917	986
KBTBD7	0.8892544	-0.169332	0.5418536	354	269	289	185	283	343
KBTBD8	0.7203008	-0.473329	0.1221952	289	133	243	108	183	188
BACE1-AS	1.125	0.169925	0.6445945	22	26	24	41	25	15
AC018638.8	0.5689655	-0.813587	0.128881	49	37	30	12	19	35
AC091231.1	0	#NUM!	0.3414317	0	2	3	0	0	0
AC122129.1	0.5652174	-0.823122	0.4573145	6	10	7	0	9	4
AC018638.1	0.5	-1	0.6175651	0	1	11	1	2	3
AC018638.2	0.7361111	-0.442005	0.1909044	85	116	159	81	102	82
AC018638.4	0.8681481	-0.203987	0.5271751	231	161	283	138	218	230
AC018638.5	1.1797235	0.2384488	0.3687183	93	57	67	81	84	91
AC018638.6	0	#NUM!	0.824326	0	0	1	0	0	0
AC018638.7	0.7777778	-0.36257	0.8812871	2	0	7	0	3	4
AL133456.1	0	#NUM!	0.5387425	3	0	0	0	0	0
AC097641.3	0.3714286	-1.428843	0.1576529	12	11	12	0	5	8
AC097641.1	0	#NUM!	0.0885691	8	2	0	0	0	0
IDI1	1.0546496	0.0767638	0.3008447	1556	1258	1724	1362	1771	1653
IDI2	2	1	0.6647467	4	0	0	0	5	3
AC018878.1	#DIV/0!	#DIV/0!	0.8030358	0	0	0	0	0	1
KLHDC10	0.9891827	-0.015691	0.935869	302	260	270	189	304	330
MPST	1.0919028	0.1268445	0.2172764	1292	1645	2003	2053	1761	1580
IDH1	0.7406579	-0.433121	0.0008514	3826	3785	4458	2404	3372	3163
IDH2	0.7759291	-0.366003	0.0222903	6240	7760	7877	5720	5978	5277
IL21R-AS1	0	#NUM!	0.824326	0	0	1	0	0	0
TRPV2	1.4446529	0.5307229	0.0012277	334	379	353	473	618	449
TRPV3	0.9509658	-0.072535	0.8927985	252	191	230	174	246	220
TRPV1	1.0495495	0.0697703	0.7228861	47	94	81	86	96	51
TRPV6	0.7308198	-0.452412	0.0056155	696	721	864	463	589	615
AL451044.1	0.6	-0.736966	0.8158703	5	0	0	3	0	0
TRPV4	#DIV/0!	#DIV/0!	0.1900534	0	0	0	2	2	2
AC004917.1	#DIV/0!	#DIV/0!	0.6554062	0	0	0	0	2	0
LMO7-AS1	0.5	-1	0.6607233	3	0	3	0	2	1
AC073585.1	0	#NUM!	0.824326	0	1	0	0	0	0
AC019070.1	#DIV/0!	#DIV/0!	0.5371397	0	0	0	0	0	3
8-Sep	0.6250655	-0.677921	2.63E-08	7041	7634	8209	3975	5271	5058
9-Sep	1.0109766	0.0157496	0.527475	6623	7896	8530	7351	8181	7770
6-Sep	1.1262432	0.1715184	0.0427643	5028	5820	6547	5922	6963	6706
7-Sep	1.2176743	0.2841282	0.0513738	4577	2649	4452	3686	5840	4694
TRPS1	0.0204082	-5.61471	0.0001705	13	19	17	0	0	1
AL133216.1	0.9	-0.152003	0.9305987	6	4	0	4	1	4
AL133216.2	0.9127106	-0.131771	0.7335822	198	186	269	169	255	172

1-Sep	0.7954545	-0.330149	0.7194769	5	19	20	10	15	10
TRPT1	1.1560102	0.2091542	0.2302277	248	252	282	397	283	224
4-Sep	0.5	-1	0.7778439	0	0	4	0	2	0
5-Sep	0.5	-1	0.4379874	8	3	9	5	2	3
2-Sep	1.1946485	0.2565862	0.0077664	11848	9813	12909	11318	15496	14485
3-Sep	0.6788432	-0.55885	0.0033623	411	408	495	224	336	332
AP000845.1	0.375	-1.415037	0.2634757	17	1	6	1	2	6
PFDN1	1.2237741	0.2913373	0.009796	1971	2082	2534	2846	2873	2342
PFDN2	0.9117384	-0.133308	0.5942858	1300	1474	1690	1311	1555	1204
PFDN4	1.1929157	0.2544921	0.0208126	1131	997	1288	1397	1427	1251
PFDN5	0.9217094	-0.117616	0.7687608	3858	5062	5705	4848	4763	3869
PFDN6	0.9875204	-0.018118	0.709985	1882	2236	2613	2398	2263	1986
AC108058.1	1.025641	0.0365259	0.9185415	16	10	13	8	14	18
AC009226.1	1.875	0.9068906	0.4847559	4	0	4	2	4	9
AC019226.2	#DIV/0!	#DIV/0!	0.6554062	0	0	0	0	2	0
AC254629.1	0	#NUM!	0.6436235	2	0	0	0	0	0
AC019226.1	1	0	0.9443813	1	0	1	2	0	0
AL359091.3	0.9375	-0.093109	0.8752533	13	12	7	0	25	5
AL359091.4	0.6829268	-0.550197	0.5353192	5	22	14	10	8	10
EHMT2	0.8082626	-0.307104	0.048697	2314	2841	2736	1865	2261	2252
TMEM94	0.9017355	-0.149224	0.3997891	2519	2787	2876	2172	2734	2472
EHMT1	0.9546067	-0.067022	0.9056336	2905	3270	3474	2705	3173	3333
AL109766.1	#DIV/0!	#DIV/0!	0.8030358	0	0	0	0	0	1
TMEM97	1.1256138	0.1707119	0.0398409	5265	5788	6461	5730	7223	6761
TMEM91	1.1956522	0.2577978	0.4668749	24	33	35	48	35	27
TMEM92	1.2120536	0.2774535	0.1693917	112	127	209	200	207	136
PSMD8	0.9389502	-0.090879	0.8607035	3845	4902	5389	4537	4723	4013
PSMD9	0.7819549	-0.354843	0.5503711	37	47	49	47	39	18
PSMD6	0.9461561	-0.07985	0.8789417	3237	3147	4035	3086	3561	3211
PSMD7	0.9488905	-0.075687	0.9062695	2164	2073	2748	1987	2359	2282
PSMD4	0.9181636	-0.123177	0.6429464	6362	7321	8251	6793	7133	6213
PSMD5	1.0144757	0.0207344	0.5601807	1633	1559	1920	1406	1832	1948
TMEM88	2	1	0.0964561	21	5	17	16	39	31
PSMD2	0.8561762	-0.22402	0.1475555	7827	9419	10093	7067	8383	7957
PSMD3	0.8984878	-0.154429	0.4716288	4185	5296	5729	4411	4923	4332
PSMD1	1.1413205	0.190704	0.0263509	4765	4011	5355	4794	5737	5597
TMEM8A	1.1657492	0.2212575	0.0872344	494	529	612	630	746	530
TMEM8B	0.9003165	-0.151496	0.7668875	196	167	269	208	177	184
TMEM80	0.9906542	-0.013547	0.8046368	333	377	360	389	357	314
TMEM81	0.990099	-0.014355	0.9374776	38	20	43	25	50	25
TMEM82	0.7142857	-0.485427	0.7962637	3	3	1	1	0	4
AC005019.1	#DIV/0!	#DIV/0!	0.8030358	0	0	0	0	0	1
PSME3	0.8121725	-0.300142	0.028937	6635	7682	8390	5317	6733	6392
TMEM78	0	#NUM!	0.4374838	0	4	0	0	0	0
AC005019.2	#DIV/0!	#DIV/0!	0.5070017	0	0	0	3	0	0
PSME4	1.0821017	0.1138361	0.2907241	4520	2995	4799	3322	5056	4947
TMEM79	0.8072289	-0.30895	0.2989317	137	212	232	142	162	165
PSME1	1.2400633	0.3104138	0.005877	1971	2094	2250	2739	2672	2420
PSME2	1.1194881	0.1628391	0.0952919	2677	3023	3598	3674	3686	3049
IDNK	1.4331797	0.5192195	0.0396536	68	66	83	95	114	102
RHOXF1P1	4.2727273	2.0951572	5.47E-05	16	9	8	43	40	58
RHOXF1P2	0.8571429	-0.222392	0.9018786	0	4	3	0	4	2
RHOXF1P3	0.7931034	-0.334419	0.7461096	4	12	13	4	9	10
AC010904.2	3	1.5849625	0.5460985	0	2	0	0	3	3

PSMF1	0.9229932	-0.115608	0.6858896	2030	2550	2770	2198	2403	2183
AC026410.1	0	#NUM!	0.5387425	3	0	0	0	0	0
AC026410.3	1	0	0.8984449	35	47	47	38	51	40
TMEM98	2	1	0.7475522	2	0	0	0	0	4
TMEM99	1.1425178	0.1922167	0.1250704	332	397	534	462	478	503
HIF1A-AS1	0.5	-1	0.7214899	0	4	0	0	0	2
HIF1A-AS2	1.6666667	0.7369656	0.7816572	1	2	0	0	0	5
PSMG3	1.0041012	0.0059046	0.6646743	446	470	547	502	523	444
PSMG4	0.8221154	-0.282587	0.2381374	537	529	806	537	538	464
AC010894.2	0.972973	-0.039528	0.9916476	15	8	14	10	18	8
PSMG1	1.0826972	0.1146298	0.1438833	2261	2246	2567	2439	2797	2423
AC010894.3	0.910299	-0.135588	0.9320764	55	101	145	118	91	65
PSMG2	1.0536263	0.0753632	0.2940509	1318	1367	1548	1518	1493	1449
AC010894.4	0.4	-1.321928	0.6568958	0	5	0	2	0	0
AC006333.2	0.7096774	-0.494765	0.1234542	101	94	115	52	94	74
AC010894.5	0.6363636	-0.652077	0.7198052	5	1	5	1	0	6
TMEM9B	0.9202052	-0.119973	0.6852648	1013	1127	1369	1040	1228	961
EFCAB3	#DIV/0!	#DIV/0!	0.8030358	0	0	0	0	0	1
TMEM51	1.4053691	0.4909491	0.0023719	214	250	281	335	379	333
EFCAB5	0.75	-0.415037	0.8892729	0	1	3	0	3	0
TMEM52	1.3517915	0.4348727	0.0929278	76	130	101	156	143	116
TMEM53	0.870801	-0.199585	0.5731436	113	137	137	110	116	111
EFCAB7	1.300578	0.379153	0.0923652	121	90	135	134	192	124
EFCAB9	0.3333333	-1.584963	0.6622061	0	3	0	0	0	1
TMEM47	0.241573	-2.049469	1.27E-05	54	56	68	13	12	18
SNORD13P3	#DIV/0!	#DIV/0!	0.6099294	0	0	0	2	0	0
TRMT112	0.9618005	-0.05619	0.9334116	2346	2822	3052	2875	2764	2267
TMEM43	0.9498015	-0.074302	0.9323672	1125	1041	1360	1049	1164	1136
TMEM44	1.1042584	0.1430779	0.3365359	217	194	270	237	315	200
EFCAB2	0.6739726	-0.569238	0.0156086	251	230	249	129	214	149
AC022217.3	0	#NUM!	0.824326	1	0	0	0	0	0
TMEM40	1.2490706	0.3208551	0.0391066	228	230	349	340	360	308
TMEM42	0.804878	-0.313158	0.7125451	21	9	11	4	8	21
AC000068.2	0.2	-2.321928	0.2535292	1	5	4	0	2	0
PSMA5	0.9279079	-0.107947	0.7457422	3761	4309	4955	4095	4339	3652
PSMA6	1.1270492	0.1725505	0.4388755	69	83	92	74	104	97
TMEM37	0.5721212	-0.805607	0.002206	238	302	285	190	138	144
MBLAC1	1.40625	0.4918531	0.4233186	14	7	11	20	12	13
PSMA3	1.0685646	0.0956742	0.1756268	4826	4341	5972	4985	6161	5031
MBLAC2	0.7360825	-0.442061	0.0969562	157	142	186	97	138	122
PSMA4	1.0643369	0.0899549	0.1785756	4999	4432	6050	5198	6049	5230
PSMA1	0.9425587	-0.085346	0.9973854	118	101	164	131	108	122
PSMA2	1.4666667	0.552541	0.0658078	39	57	69	94	73	75
TMEM33	0.9296387	-0.105258	0.6755327	2571	2066	2725	1881	2481	2482
KIF1BP	1.3667462	0.4507454	0.0012573	423	341	493	497	602	619
SMTNL2	0.8201754	-0.285996	0.3370299	145	128	183	94	146	134
AC000068.1	0.6190476	-0.691878	0.6354689	6	2	13	5	1	7
SMTNL1	2.9	1.5360529	0.1295305	4	0	6	15	8	6
GOT1L1	0	#NUM!	0.5622741	0	1	2	0	0	0
DMAC1	1.1959934	0.2582094	0.0196501	1322	1673	1847	1853	2109	1829
DMAC2	0.8700368	-0.200852	0.3033601	1389	1759	1738	1373	1497	1381
IDUA	#DIV/0!	#DIV/0!	0.6554062	0	0	0	0	2	0
AC024614.1	0	#NUM!	0.5337954	0	3	0	0	0	0
COL11A1	0.2727273	-1.874469	0.3242633	6	5	0	0	3	0

ZNF518B	1.038282	0.0541983	0.6404919	421	234	416	261	400	451
COL11A2	1.6086957	0.6858914	0.4250034	0	6	17	6	16	15
TMEM72	0.2857143	-1.807355	0.4466716	7	0	0	0	1	1
ZNF518A	1.256734	0.3296793	0.139894	531	211	446	348	596	549
TMEM74	0.9173228	-0.124499	0.8299685	68	76	110	56	88	89
TMEM75	0.6666667	-0.584963	0.7166693	6	5	1	4	0	4
PSMA7	0.9868878	-0.019042	0.7225724	4468	5855	6684	6165	5846	4773
TMEM70	0.8300546	-0.268722	0.1632729	552	589	689	469	586	464
PSMA8	0	#NUM!	0.824326	1	0	0	0	0	0
TMEM71	0.8	-0.321928	0.904823	0	5	0	3	1	0
PSMB6	0.9978153	-0.003155	0.6534399	3300	4128	4473	4129	4168	3578
TMEM69	1.0712739	0.0993274	0.2073667	1523	1576	1980	1534	2115	1792
PSMB7	1.1210427	0.1648413	0.0991287	6058	8153	8690	8607	8963	8103
PSMB4	0.9853679	-0.021266	0.7222969	5555	6447	7339	6745	6566	5747
PSMB5	1.0312838	0.0444414	0.4176002	3999	4758	5116	4948	4993	4366
PSMB2	1.0503984	0.0709366	0.2720156	4839	5554	6175	5716	6164	5523
TMEM65	0.7769517	-0.364103	0.365808	107	53	109	43	87	79
PSMB3	0.9328428	-0.100294	0.8034977	3655	4398	4857	4163	4295	3585
TMEM67	0.969697	-0.044394	0.9337026	131	113	119	67	165	120
PSMB1	1.0077826	0.0111845	0.5511872	6871	7669	8717	7976	8439	7023
TMEM68	0.8905156	-0.167287	0.4913781	563	416	592	383	527	489
AC010654.1	1.4411765	0.527247	0.3867512	13	11	10	17	15	17
TRMT10A	1.2874317	0.3644959	0.0246923	321	220	374	335	454	389
TRMT10B	0.8970793	-0.156693	0.6563209	249	181	289	159	218	268
TRMT10C	1.0249688	0.03558	0.5770345	1170	701	1333	923	1292	1069
IORMAD2-AS	#DIV/0!	#DIV/0!	0.8030358	0	0	0	0	0	1
TMEM62	0.7595276	-0.396826	0.0402155	530	660	673	376	481	558
TMEM64	1.0224829	0.0320767	0.7795573	338	370	315	238	393	415
PSMB8	1.4716981	0.5574818	0.2996039	18	16	19	22	41	15
PSMB9	1.2897959	0.3671428	0.1413093	66	80	99	129	88	99
TMEM60	0.9904348	-0.013866	0.8192324	335	387	428	333	443	363
TMEM59	1.1007621	0.1385027	0.0760756	2545	2467	3255	2776	3364	2960
PSMC5	0.9634836	-0.053668	0.882918	1982	2191	2728	2419	2303	1927
PSMC6	1.1828805	0.2423043	0.0276903	2420	1776	2907	2423	3173	2806
PSMC3	0.9117112	-0.133351	0.6006858	5924	7272	7690	6491	6562	5989
TMEM54	1.2266402	0.2947121	0.0343161	579	675	758	932	833	703
PSMC4	0.8908706	-0.166712	0.4469476	3198	4056	4072	3420	3590	3080
PSMC1	0.8263736	-0.275134	0.3400512	132	160	163	98	149	129
TMEM56	1.0697674	0.0972972	0.5695012	103	96	102	113	87	122
PSMC2	1.0729376	0.1015662	0.1439721	3774	3539	4615	3959	4757	4082
TMEM57	1.1161826	0.158573	0.1761834	609	454	624	542	711	630
AC015845.2	0	#NUM!	0.824326	0	0	1	0	0	0
AL359091.1	4	2	0.502311	1	0	0	4	0	0
NOMO3	0.8519934	-0.231086	0.3030798	536	609	686	343	579	638
AC103810.5	0	#NUM!	0.3912131	1	1	2	0	0	0
AC103810.3	1.6	0.6780719	0.5299424	13	4	3	2	21	9
AC103810.1	0	#NUM!	0.3728105	1	2	1	0	0	0
AC103810.2	0.7	-0.514573	0.7717077	3	6	1	4	0	3
SESN3	0.3793103	-1.398549	0.0011126	108	72	52	29	26	33
Z73429.1	0	#NUM!	0.824326	0	0	1	0	0	0
SESN1	0.8283119	-0.271754	0.0567527	2928	2665	3103	2050	2585	2568
SESN2	1.0055818	0.0080304	0.6035066	2708	3236	3372	2993	3346	3029
NOMO1	0.8360336	-0.258367	0.091095	3848	4649	4835	3365	4124	3657
NOMO2	0.7796865	-0.359034	0.0243132	1663	2085	2248	1270	1690	1715

UBALD1	0.9374208	-0.093231	0.9113779	1079	1317	1551	1365	1301	1034
UBALD2	1.2914187	0.3689568	0.0097953	1080	1408	1544	1982	1767	1458
AL596087.1	0.7065217	-0.501194	0.3938903	30	27	35	25	24	16
AP003486.1	0.8516129	-0.23173	0.6417506	63	40	52	42	49	41
SQSTM1	0.6163183	-0.698253	9.20E-08	38699	47898	50746	26604	29899	28144
PTOV1-AS1	0.9249548	-0.112545	0.7480925	358	347	401	325	411	287
PTOV1-AS2	1.0363636	0.0515303	0.8023038	12	11	32	17	18	22
TMEM31	1.1551724	0.2081082	0.6238567	15	19	24	21	27	19
TMEM25	2.3513514	1.2334901	0.0372377	14	13	10	34	39	14
TMEM27	0.5263158	-0.925999	0.2839708	16	14	8	4	13	3
TMEM17	1.2652439	0.3394155	0.14783	96	90	142	92	170	153
TMEM11	1.021377	0.0305155	0.4618618	1024	1137	1441	1160	1296	1223
TMEM18	1.0193336	0.0276263	0.5557496	724	803	904	797	943	738
TMEM19	1.0498534	0.0701878	0.5005597	232	203	247	242	230	244
ZNF512B	0.8806705	-0.183326	0.2941252	2000	2292	2211	1631	2017	2079
AL356599.1	0.4736842	-1.078003	0.3107697	22	4	12	11	2	5
AL353072.1	0	#NUM!	0.5378194	0	2	1	0	0	0
AC005842.1	0	#NUM!	0.1487666	7	1	0	0	0	0
AC007318.1	1.1407613	0.1899969	0.0645842	748	749	1025	903	1070	904
AC007318.2	0.3125	-1.678072	0.2903107	3	11	2	0	0	5
USP27X	0.8548523	-0.226253	0.3284478	356	347	482	294	391	328
AC009955.2	#DIV/0!	#DIV/0!	0.4501902	0	0	0	0	0	4
AC009955.3	0.4285714	-1.222392	0.6702892	0	0	7	0	3	0
AC009955.1	0	#NUM!	0.6631647	1	0	1	0	0	0
AC009955.4	0	#NUM!	0.2219629	0	7	0	0	0	0
AC073109.1	0	#NUM!	0.4460243	4	0	0	0	0	0
AC004034.1	0.7333333	-0.447459	0.8162815	4	0	11	2	9	0
AP002907.1	0.5789474	-0.788496	0.5254862	12	2	5	0	7	4
GDI2P2	0	#NUM!	0.6410557	1	1	0	0	0	0
RNVU1-3	0.4444444	-1.169925	0.6363299	0	0	9	0	3	1
ERRFI1	1.1005674	0.1382475	0.1026965	7929	7566	9354	7200	10223	9925
RNVU1-4	0	#NUM!	0.276989	0	6	0	0	0	0
RNVU1-6	0.25	-2	0.5682537	0	1	3	0	1	0
AC133785.1	0.4857143	-1.04182	0.2963051	3	16	16	6	7	4
RNVU1-1	1	0	0.9890559	1	0	0	0	1	0
AADACL2	#DIV/0!	#DIV/0!	0.6099294	0	0	0	2	0	0
AADACL3	0	#NUM!	0.824326	0	1	0	0	0	0
AC107075.1	1	0	0.9154088	4	1	10	5	6	4
KIF21A	1.4779759	0.5636227	0.0046804	1582	785	1447	1206	2273	2158
KIF21B	0.7926577	-0.33523	0.0860743	511	687	736	416	596	521
ST6GAL1	1.2696524	0.3444335	0.0059522	902	1097	1194	1159	1326	1569
RSAD2	5.8076923	2.537965	1.02E-06	10	7	9	37	50	64
SFMBT1	0.8158609	-0.293605	0.1485472	665	493	683	376	573	553
SFMBT2	1.1137958	0.1554847	0.2760798	432	415	436	350	507	572
RSAD1	1.0305958	0.0434786	0.5206876	335	385	522	382	481	417
AC092943.1	0	#NUM!	0.824326	1	0	0	0	0	0
AC019227.1	0	#NUM!	0.5781912	0	0	3	0	0	0
AC104785.1	0.2222222	-2.169925	0.3816127	4	0	5	2	0	0
KIF20A	0.8509608	-0.232835	0.1011281	6056	6577	6986	4704	6112	5879
KIF20B	1.851229	0.8884834	2.69E-05	521	277	748	710	1209	943
AC124861.1	0.9142857	-0.129283	0.9164135	16	21	33	19	27	18
TGOLN2	0.8850152	-0.176226	0.2951451	9591	9767	10559	6767	9495	10215
AC008147.4	0.5454545	-0.874469	0.6045901	6	2	3	2	0	4
AC008147.1	1.3333333	0.4150375	0.8441278	0	0	3	0	3	1

AC008147.2	1.4444444	0.5305147	0.5066718	5	3	10	8	11	7
AC008147.3	1	0	0.978278	0	0	2	0	2	0
MRPL40P1	1	0	0.9866059	5	3	6	0	13	1
OR7E22P	0.5909091	-0.758992	0.4984718	4	11	7	5	4	4
PTGDR2	0.5714286	-0.807355	0.5697382	12	1	1	0	5	3
EDA2R	0.7107362	-0.492614	0.0019587	1098	957	1205	638	791	888
LARP1B	1.2476281	0.3191879	0.0152686	746	527	835	787	996	847
CDC42-IT1	1	0	0.9756864	2	0	0	0	2	0
AC025822.1	0.8333333	-0.263034	0.9538463	0	3	3	3	0	2
AL590068.1	#DIV/0!	#DIV/0!	0.3096126	0	0	0	5	0	0
PSMA2P1	0	#NUM!	0.5643268	1	0	2	0	0	0
PSMA2P3	#DIV/0!	#DIV/0!	0.5082165	0	0	0	1	0	2
HNRNPUP1	2.8	1.4854268	0.2691077	2	2	1	6	3	5
VN1R42P	0.9	-0.152003	0.9134968	2	4	4	0	6	3
SAGE1	0.4749164	-1.074255	3.61E-05	200	185	213	72	129	83
AP000692.1	0.5	-1	0.4162863	10	3	7	1	8	1
AP000692.2	0.8181818	-0.289507	0.8155393	12	1	9	0	9	9
AC073348.1	2	1	0.814195	0	1	0	0	0	2
RAMP2	#DIV/0!	#DIV/0!	0.8030358	0	0	0	0	1	0
CAMK2N1	1.8727273	0.9051408	0.052807	19	14	22	40	35	28
CAMK2N2	3.1428571	1.6520767	0.2075408	5	2	0	3	7	12
XDC2P-NPIPB	1.1273885	0.1729848	0.609666	68	39	50	35	72	70
KIF26A	1.3992416	0.4846451	0.0003928	540	666	640	820	899	864
KIF26B	0.6666667	-0.584963	0.1874217	38	64	60	22	48	38
RAMP1	0.1111111	-3.169925	0.0048461	5	15	25	2	2	1
KIF28P	0.6595745	-0.600393	0.4981988	28	4	15	11	9	11
AL691403.2	6.5	2.7004397	0.1791369	2	0	0	5	8	0
OR7E29P	0.75	-0.415037	0.8602864	3	1	0	0	0	3
OSTF1	1.2669269	0.3413333	0.000658	2481	2590	3126	3416	3671	3298
AL691403.1	0	#NUM!	0.6840298	0	0	2	0	0	0
NPSR1-AS1	#DIV/0!	#DIV/0!	0.8030358	0	0	0	0	0	1
HNRNPUL1	0.8503869	-0.233809	0.1141615	5496	6357	6755	4666	5645	5513
HNRNPUL2	0.9639682	-0.052943	0.9924079	671	701	765	631	738	691
AC005498.2	2.8571429	1.5145732	0.2104998	1	5	1	10	6	4
AC005498.3	0.7594937	-0.39689	0.5445429	38	19	22	24	20	16
CALHM1	#DIV/0!	#DIV/0!	0.8030358	0	0	0	0	0	1
CALHM2	1.6318681	0.7065245	0.0149127	42	68	72	90	114	93
AC091230.1	0	#NUM!	0.355562	5	0	0	0	0	0
CALHM3	1.4736842	0.5594274	0.5210086	7	4	8	10	16	2
CALHM5	0	#NUM!	0.5337954	0	3	0	0	0	0
AC018639.1	0	#NUM!	0.824326	1	0	0	0	0	0
HSP90AB2P	0.1428571	-2.807355	0.0327267	3	10	15	1	2	1
AC114271.1	3	1.5849625	0.6684577	1	0	0	0	0	3
SLC44A3-AS1	0.8692308	-0.202189	0.6996426	36	50	44	24	37	52
AL157396.1	0.25	-2	0.5498031	4	0	0	1	0	0
HSP90AB4P	0	#NUM!	0.824326	0	1	0	0	0	0
AC012467.1	#DIV/0!	#DIV/0!	0.5509701	0	0	0	0	3	0
AC012467.2	1.1616162	0.2161334	0.4757982	87	44	67	55	101	74
HMMR-AS1	4.5	2.169925	0.1359401	3	0	1	6	10	2
Z97832.2	1.0588235	0.0824622	0.8181474	4	3	10	11	2	5
HSP90AB3P	0.7978437	-0.325822	0.2982278	115	114	142	90	101	105
AC008481.1	0	#NUM!	0.6436235	2	0	0	0	0	0
AC008481.2	0.6666667	-0.584963	0.7530383	4	1	1	0	2	2
AC233699.1	0.8367347	-0.257158	0.6364532	31	32	35	16	38	28

TRRAP	0.6651989	-0.588142	0.0005893	4268	4652	4479	2024	3191	3698
COL12A1	0.8850006	-0.17625	0.4078376	2890	2696	3101	1629	2785	3274
RN7SKP160	0.1666667	-2.584963	0.3526309	1	1	4	0	1	0
RN7SKP161	0.6	-0.736966	0.7578088	3	0	2	0	1	2
ZXDA	0.8601824	-0.217286	0.5524126	87	112	130	61	111	111
ZXDB	0.619403	-0.69105	0.0001782	426	437	477	220	303	307
RN7SKP163	#DIV/0!	#DIV/0!	0.6099294	0	0	0	2	0	0
ZXDC	0.911811	-0.133193	0.5889142	927	1008	1240	896	1077	922
IER5	0.9365449	-0.09458	0.8411226	1079	1362	1420	1258	1322	1036
NFRKB	0.752512	-0.410214	0.0091734	1928	2045	2496	1267	1681	1920
IER2	0.990736	-0.013427	0.7342858	559	617	767	693	708	524
IER3	1.0472879	0.0666581	0.5080369	197	224	298	256	285	212
AC078778.1	0.9338843	-0.098684	0.9850912	50	17	54	43	42	28
AC078778.2	0.2222222	-2.169925	0.0696211	10	12	5	0	4	2
AL590652.1	0.7575758	-0.400538	0.6901341	4	12	17	3	11	11
RN7SKP150	0.3333333	-1.584963	0.5274789	9	0	0	3	0	0
AC092119.2	0.655914	-0.608421	0.3400513	25	19	49	8	27	26
AC092119.3	0	#NUM!	0.280545	3	1	1	0	0	0
RN7SKP153	0	#NUM!	0.824326	0	0	1	0	0	0
HPRT1	0.9138795	-0.129924	0.5695192	1944	1873	2372	1784	2108	1764
CNTN4-AS2	0.0833333	-3.584963	0.0979997	10	1	1	0	0	1
POM121	0.7110232	-0.492031	0.0024921	1649	1909	2003	994	1387	1573
NOLC1	1.0267556	0.0380928	0.3758861	7393	7602	9000	7069	8953	8615
CHST11	0.9013855	-0.149784	0.5941624	442	325	460	329	421	356
CHST12	0.9213781	-0.118135	0.7066655	358	389	385	324	346	373
CHST10	0.9341672	-0.098247	0.8188799	402	534	583	436	496	487
RN7SL113P	0	#NUM!	0.5337954	0	3	0	0	0	0
CHST15	0.1403509	-2.83289	0.004951	9	39	9	5	1	2
CHST13	0	#NUM!	0.5387425	3	0	0	0	0	0
AC007319.1	0.75	-0.415037	0.7558361	3	9	0	0	3	6
CHST14	0.8661806	-0.20726	0.3168649	1247	1541	1464	1250	1232	1201
SERF2	1.01525	0.021835	0.5802313	1040	1442	1518	1476	1378	1207
AL133551.1	0.45	-1.152003	0.3272955	11	7	2	1	4	4
MCTS2P	0.742268	-0.429988	0.2224829	105	73	113	64	70	82
MRC2	1.3	0.3785116	0.7963916	2	8	0	4	4	5
AC009464.1	0	#NUM!	0.6436235	2	0	0	0	0	0
MRC1	2.0625	1.0443941	0.0916365	10	6	16	14	22	30
AC022706.1	0	#NUM!	0.5781912	0	0	3	0	0	0
ANKRD26P3	3	1.5849625	0.6896151	1	0	0	0	3	0
IFNGR1	1.0833856	0.1155468	0.2187598	1114	861	1215	974	1299	1183
RBM15-AS1	0.78125	-0.356144	0.6878549	9	16	7	4	6	15
IFNGR2	0.9928826	-0.010305	0.8457104	327	422	375	337	417	362
ANKRD26P1	8	3	0.2194181	1	0	0	2	0	6
AC132812.1	0.5140187	-0.960107	0.1421157	49	9	49	14	20	21
MRAS	0.5588445	-0.839481	4.45E-08	802	902	1100	450	594	523
HCLS1	0.3579109	-1.482328	4.06E-26	1803	2241	2466	691	866	773
RN7SKP118	#DIV/0!	#DIV/0!	0.6099294	0	0	0	2	0	0
RN7SKP116	0	#NUM!	0.824326	1	0	0	0	0	0
SF3B4	0.79698	-0.327385	0.0282089	3602	4094	4357	3132	3330	3144
SF3B5	0.9006681	-0.150933	0.561765	2702	3494	3982	3098	3339	2730
SF3B2	0.8847898	-0.176593	0.3299382	7838	9276	10670	7746	8640	8197
SF3B3	0.8134903	-0.297803	0.0351976	12630	14598	14743	9842	11945	12356
SF3B6	1.0966671	0.1331257	0.1237355	2127	2304	2800	2497	3044	2389
NOTCH2NL	0.969697	-0.044394	0.9705926	9	3	21	3	14	15

AC087318.1	#DIV/0!	#DIV/0!	0.6411933	0	0	0	0	0	2
RN7SKP106	1.25	0.3219281	0.8613012	0	1	3	0	3	2
SF3B1	1.1739845	0.2314134	0.0850012	11708	7355	11342	8759	13470	13466
SF3A3	0.9620621	-0.055798	0.9663655	5462	5468	6625	5263	6054	5572
SF3A1	0.7856318	-0.348075	0.0118181	4044	4759	5047	3198	3976	3707
SF3A2	0.8897431	-0.168539	0.4577948	1660	2034	2029	1754	1717	1621
NOTCH2P1	0	#NUM!	0.5075476	0	0	4	0	0	0
MORN1	1.0926573	0.127841	0.3812344	156	194	222	176	231	218
AC008875.1	2.1428571	1.0995357	0.3786876	0	2	5	12	2	1
AL451042.1	0	#NUM!	0.1212581	7	0	2	0	0	0
MORN4	2	1	0.7782384	0	0	1	2	0	0
AL451042.2	1	0	0.9792582	0	2	6	0	7	1
MORN2	1.1910828	0.2522737	0.1702168	122	171	178	178	193	190
MORN3	1.3764321	0.4609334	0.0010285	346	413	463	498	627	557
HYLS1	0.7976812	-0.326116	0.1279714	518	461	746	346	550	480
ITGA2B	0.9785203	-0.031326	0.8446081	376	385	496	372	435	423
AC010655.4	0	#NUM!	0.5337954	0	3	0	0	0	0
AC010655.1	3	1.5849625	0.6103938	0	0	1	1	1	1
AC010655.2	0	#NUM!	0.3482063	0	0	6	0	0	0
MREG	0.8841343	-0.177663	0.4282415	466	526	527	416	479	448
AP005059.1	0	#NUM!	0.824326	0	1	0	0	0	0
CACNB1	1.6363636	0.7104934	0.5824657	7	0	4	1	4	13
CACNB2	1.125	0.169925	0.5880416	66	48	70	37	99	71
CACNB3	0.9635258	-0.053605	0.9594261	84	129	116	81	130	106
AC092849.1	0.7272727	-0.459432	0.7520729	6	2	14	4	3	9
CACNB4	1	0	0.8981116	0	0	4	4	0	0
AL136097.1	0	#NUM!	0.6385096	0	2	0	0	0	0
AL583839.1	0.7692308	-0.378512	0.4594965	28	45	44	20	44	26
AL583839.2	0.2	-2.321928	0.4595179	2	0	3	0	1	0
MRI1	0.7493689	-0.416252	0.0145901	810	917	1046	688	700	690
SAFB2	0.9985307	-0.002121	0.6170314	2643	2867	3338	2770	3210	2855
AC069148.1	0.0909091	-3.459432	0.1245365	6	5	0	0	0	1
DCST1-AS1	1.0294118	0.0418202	0.8123335	17	6	45	18	31	21
GCSAM	0.852459	-0.230298	0.6674943	42	38	42	31	35	38
AP002992.1	0	#NUM!	0.6436235	2	0	0	0	0	0
ZZEF1	0.7088738	-0.496399	0.0034092	2089	2301	2394	1203	1591	2015
AL359736.1	#DIV/0!	#DIV/0!	0.8030358	0	0	0	0	0	1
SNAI3-AS1	0.75	-0.415037	0.4580028	20	43	37	17	37	21
ITGA10	0.75	-0.415037	0.8790046	2	0	2	0	1	2
ITGA11	0.3333333	-1.584963	0.4535909	6	0	3	0	3	0
SRXN1	0.4271845	-1.227069	0.075497	18	52	33	4	9	31
DUSP19	0.7672956	-0.382146	0.3794282	61	46	52	28	44	50
AC125603.1	0	#NUM!	0.824326	0	1	0	0	0	0
AC125603.2	5	2.3219281	0.4102632	1	0	0	2	3	0
DUSP18	1.0729927	0.1016403	0.67056	49	38	50	44	49	54
DUSP15	1.1578947	0.2115041	0.821092	2	16	1	14	4	4
DUSP16	0.6170513	-0.696538	0.0005092	676	695	752	271	510	529
DUSP13	1.3670087	0.4510224	0.0013326	377	425	465	570	566	596
DUSP14	0.8538973	-0.227866	0.1964089	2472	2815	2988	2360	2509	2197
DUSP11	1.080389	0.1115508	0.2453853	1042	995	1048	971	1280	1082
DUSP12	0.9464128	-0.079459	0.9239585	707	684	867	711	763	663
DUSP10	0.7534884	-0.408343	0.3128236	57	69	89	53	62	47
REXO1	0.846	-0.24127	0.26275	760	1099	1141	781	851	906
DUSP28	0.7982833	-0.325027	0.443128	85	81	67	75	55	56

REXO2	0.9572939	-0.062966	0.9720025	1407	1471	1852	1540	1684	1304
DUSP27	0.972973	-0.039528	0.9630969	37	11	26	26	27	19
REXO4	1.098236	0.1351881	0.1251298	1909	2208	2459	2395	2556	2271
REXO5	0.9562682	-0.064513	0.9935099	232	188	266	209	217	230
DUSP22	0.8692085	-0.202226	0.336894	626	682	764	585	607	609
DUSP23	0.3421053	-1.547488	0.095487	9	17	12	2	4	7
AC063952.1	0	#NUM!	0.2883218	0	0	7	0	0	0
AC063952.2	2.3333333	1.2223924	0.3803738	1	0	5	10	4	0
BRCC3P1	0.1212121	-3.044394	0.0166504	3	13	17	1	1	2
MRLN	#DIV/0!	#DIV/0!	0.6099294	0	0	0	2	0	0
MRM1	0.8587065	-0.219763	0.4308378	546	664	800	648	558	520
MRM2	0.7184517	-0.477037	0.0038827	1161	1146	1465	937	910	863
MRM3	0.9524577	-0.070273	0.9516956	319	414	508	465	404	313
CACNG6	1.1914894	0.2527661	0.5683295	11	11	25	38	10	8
DCAF15	0.9317912	-0.101921	0.8106249	1039	1113	1220	1121	1063	958
CACNG7	0.5833333	-0.777608	0.5381952	2	12	10	5	9	0
DCAF16	0.934181	-0.098226	0.7267411	1840	1535	1973	1175	1798	2023
CACNG8	0.5	-1	0.3167531	8	8	18	7	7	3
DCAF17	1.0128806	0.0184641	0.7275381	979	610	973	607	933	1055
CACNG1	0	#NUM!	0.1381401	3	5	0	0	0	0
DCAF10	1.2611872	0.3347824	0.0081408	791	656	743	799	1013	950
CACNG2	#DIV/0!	#DIV/0!	0.8030358	0	0	0	1	0	0
DCAF11	0.8762368	-0.190607	0.2973695	1732	2140	2091	1582	1820	1823
DCAF12	1.1549843	0.2078732	0.0408037	1641	1841	1925	1740	2338	2167
CACNG4	0.2971429	-1.750771	0.0004911	43	85	47	20	20	12
DCAF13	0.9313875	-0.102547	0.8073681	1619	1163	1809	1366	1574	1336
DDHD2	0.8848827	-0.176442	0.4304968	795	624	883	521	743	773
DDHD1	0.8312546	-0.266638	0.2985338	500	363	500	281	348	504
SERHL	1.1111111	0.1520031	0.7793993	16	23	6	26	10	14
HSP90AA4P	0.5	-1	0.8254608	0	1	1	0	1	0
HPSE2	0.7366548	-0.440939	0.2048332	101	94	86	56	63	88
AC020978.10	1.047619	0.0671142	0.8706495	8	2	11	6	7	9
RIMKLBP1	0.4	-1.321928	0.6517676	4	0	1	2	0	0
RIMKLBP2	0.5151515	-0.956931	0.3452067	12	7	14	1	14	2
AL353718.1	0.6904762	-0.534336	0.5933581	15	8	19	0	8	21
AC092692.1	1.0555556	0.0780025	0.8326819	1	8	9	14	3	2
TELO2	0.8944578	-0.160915	0.4975983	1181	1455	1514	1261	1313	1138
IGSF8	1.2389439	0.3091109	0.0209242	457	505	553	658	637	582
IGSF5	1.5	0.5849625	0.1852728	32	29	19	34	44	42
IGSF6	0.0833333	-3.584963	0.0931553	2	4	6	0	1	0
GRPEL2-AS1	0	#NUM!	0.3626892	1	0	4	0	0	0
IGSF3	0.9576864	-0.062375	0.858411	4486	5333	5188	3592	5385	5395
IGSF1	1.4	0.4854268	0.2607248	37	25	28	25	49	52
IGSF9	0.7403315	-0.433757	0.036582	321	378	387	244	315	245
AC073107.2	0.2195122	-2.187627	0.0650855	3	16	22	0	1	8
CR382285.1	1.1578947	0.2115041	0.6603339	12	10	16	18	14	12
AC073107.1	1.4897959	0.5751147	0.3803965	18	2	29	9	25	39
PITPNM2-AS1	2	1	0.6856375	0	0	2	1	3	0
HSP90AA5P	0	#NUM!	0.824326	0	1	0	0	0	0
CENPBD1	0.8713043	-0.198751	0.5380503	195	151	229	154	179	168
MRS2	0.9922031	-0.011293	0.7147192	1806	1602	2107	1533	2002	1937
AC019322.4	#DIV/0!	#DIV/0!	0.4501902	0	0	0	0	0	4
AC003685.2	0	#NUM!	0.6385096	0	2	0	0	0	0
ZNF101P1	1	0	0.9890559	0	0	1	0	0	1

SERP1	0.9588332	-0.060648	0.9765399	4860	4929	6049	4469	5502	5215
KIF13A	0.9132026	-0.130993	0.5784998	1364	1067	1394	787	1368	1338
KIF13B	0.7895772	-0.340848	0.1126681	340	338	339	197	319	287
RPARP-AS1	1.0321317	0.045627	0.5089522	377	424	475	457	425	435
MAPRE3	0.7679181	-0.380976	0.3403774	92	78	123	77	100	48
MAPRE1	0.9225827	-0.11625	0.5361702	11868	12585	14143	9967	13141	12500
MAPRE2	0.7766323	-0.364696	0.1117336	317	286	270	182	238	258
MRRF	1.1161638	0.1585488	0.0645975	2282	2115	2464	2400	2763	2495
IFI6	4.5127917	2.1740202	1.66E-49	2175	2803	3035	13213	12341	10607
OR7E39P	2.25	1.169925	0.4672203	1	0	3	2	4	3
MIR222HG	1.4340426	0.5200878	0.0857535	73	32	130	108	113	116
AC073347.1	#DIV/0!	#DIV/0!	0.2106225	0	0	0	4	0	2
ENSAP2	1.0440529	0.0621948	0.7152557	126	195	133	145	188	141
OR7E38P	1.5323741	0.6157685	0.0493526	37	46	56	72	79	62
MTND2P26	0	#NUM!	0.5387425	3	0	0	0	0	0
MTND2P22	0	#NUM!	0.5075476	0	0	4	0	0	0
EFTUD2	0.8062743	-0.310657	0.0235844	4920	5628	6187	4049	4919	4525
AP000941.1	0.3478261	-1.523562	0.2644941	2	10	11	3	0	5
PTCHD3P2	0	#NUM!	0.5075476	0	0	4	0	0	0
PTCHD3P3	3	1.5849625	0.6253252	0	0	1	3	0	0
OXA1L	0.9308599	-0.103364	0.6922907	5759	6273	7132	5637	6474	5728
KIF16B	0.9848243	-0.022062	0.8704711	405	362	485	308	468	457
AC100827.2	0	#NUM!	0.5622741	0	1	2	0	0	0
AC100827.4	0.2	-2.321928	0.5267773	0	0	5	0	0	1
AL157407.1	0.6842105	-0.547488	0.7384697	2	8	9	9	1	3
MTND2P40	0.5454545	-0.874469	0.5624916	7	2	2	0	4	2
BX322784.1	#DIV/0!	#DIV/0!	0.5230806	0	0	0	1	2	0
ADIPOR1	0.9802917	-0.028717	0.8069407	13599	15485	16785	13896	16287	14782
ADIPOR2	0.8729309	-0.196061	0.2071125	2463	2607	2965	2004	2545	2465
PSORS1C1	0.4285714	-1.222392	0.5763484	2	2	3	2	0	1
PSORS1C2	3.5	1.8073549	0.3989014	1	1	0	1	1	5
AL049780.1	1.0555556	0.0780025	0.9367243	9	6	3	4	7	8
AL049780.2	0.1111111	-3.169925	0.2451795	1	0	8	0	1	0
AL049780.3	1	0	0.9904495	3	0	0	0	0	3
DMAP1	0.9962145	-0.005472	0.648672	951	971	1248	1186	1035	937
AC002128.1	1.326087	0.4071754	0.4650703	16	10	20	23	30	8
AC002128.2	0.8571429	-0.222392	0.8974271	6	4	4	5	2	5
KIF18A	1.3725794	0.4568896	0.0181637	490	252	549	479	748	545
RALYL	#DIV/0!	#DIV/0!	0.6411933	0	0	0	0	0	2
KIF18B	0.8689362	-0.202678	0.2137195	1805	1888	2182	1465	1788	1852
MTND2P28	0.7763105	-0.365294	0.0202695	2473	2334	2690	1474	2045	2301
AL031123.3	1	0	0.9423867	18	16	17	13	17	21
AL031123.1	1.2307692	0.2995603	0.753991	1	6	6	4	2	10
AL031123.2	0.9324324	-0.100929	0.8868848	22	25	27	12	36	21
MUM1L1	1.1472868	0.1982261	0.3900997	105	60	93	102	107	87
ARAP2	0.6443662	-0.634047	0.077099	93	92	99	54	47	82
ARAP3	0.7894737	-0.341037	0.7650534	8	10	1	6	8	1
ARAP1	0.8312113	-0.266713	0.1161921	1581	1921	1996	1383	1527	1660
IFNG	#DIV/0!	#DIV/0!	0.6411933	0	0	0	0	0	2
IFNE	1	0	0.9263385	4	0	4	5	2	1
IFNK	0	#NUM!	0.4521054	1	0	3	0	0	0
HSP90AA2P	1.194332	0.2562039	0.4015981	98	73	76	55	123	117
AC002368.1	0	#NUM!	0.3320541	3	0	2	0	0	0
AL135999.1	0.2105263	-2.247928	0.168352	1	3	15	0	2	2

AL135999.2	#DIV/0!	#DIV/0!	0.5509701	0	0	0	0	3	0
RN7SL104P	0	#NUM!	0.6385096	0	2	0	0	0	0
AC021723.1	1	0	0.9890559	0	1	0	0	0	1
AC108010.1	0.5263158	-0.925999	0.3812337	9	7	22	3	2	15
PTPDC1	0.9423729	-0.08563	0.8619226	277	264	344	210	296	328
COMTD1	1.40618	0.4917813	0.0035631	659	812	762	1258	1043	839
CAPNS1	1.1085087	0.1486201	0.1272543	2382	3010	3446	3356	3490	2951
CAPNS2	2.6666667	1.4150375	0.4617643	1	2	0	6	2	0
AL354740.1	4	2	0.3515508	1	1	0	1	6	1
SP2-AS1	1.1212121	0.1650592	0.7661409	11	7	15	7	12	18
AC019069.1	1	0	0.8123065	77	118	128	136	125	62
AP000317.1	1	0	0.948212	2	15	7	13	10	1
UNC5A	2	1	0.656079	4	0	0	0	2	6
UNC5B	0.35	-1.514573	5.98E-08	190	219	211	44	79	94
AP000317.2	0.6666667	-0.584963	0.8411168	0	5	1	4	0	0
UNC5C	0.3043478	-1.716207	0.2019589	2	13	8	5	1	1
UNC5D	0	#NUM!	0.824326	1	0	0	0	0	0
RNU6-1170P	0	#NUM!	0.5075476	0	0	4	0	0	0
DDIT3	1.0640625	0.0895829	0.4727177	171	225	244	243	241	197
DDIT4	2.0665597	1.0472311	3.26E-13	354	430	463	738	982	857
UNC50	1.0111474	0.0159933	0.5367401	1141	1140	1397	1188	1344	1187
AC074033.1	1.1748755	0.2325079	0.0393774	990	1145	1479	1459	1509	1278
FAXDC2	0.616	-0.698998	0.0737785	79	69	102	37	42	75
AL031651.1	#DIV/0!	#DIV/0!	0.5230806	0	0	0	1	2	0
IGF2-AS	0.7142857	-0.485427	0.4981226	32	18	27	27	17	11
KCNQ1OT1	1.2924126	0.3700667	0.0638473	416	271	486	310	547	659
L1CAM	0.4230373	-1.241143	3.09E-16	1343	1762	1748	596	749	708
AC008109.1	1.056338	0.0790716	0.8273479	15	33	23	21	29	25
MOV10	0.8608422	-0.216179	0.1987894	2561	2859	3462	2394	2663	2589
AC007614.3	0.5	-1	0.7600834	3	0	1	2	0	0
HYOU1	0.6258406	-0.676133	9.08E-07	8180	9760	9869	4683	6171	6550
DZIP1	0.6	-0.736966	0.7798361	1	1	3	1	0	2
AL121749.1	#DIV/0!	#DIV/0!	0.6099294	0	0	0	2	0	0
DZIP3	1.3095615	0.3890839	0.0269619	742	395	756	644	1012	823
AC011472.5	#DIV/0!	#DIV/0!	0.8030358	0	0	0	0	0	1
MIR130A	0	#NUM!	0.5781912	0	0	3	0	0	0
MIR130B	0.8888889	-0.169925	0.8117282	44	52	39	46	23	51
MIR130B	0.8888889	-0.169925	0.8117282	44	52	39	46	23	51
AC011472.1	0.625	-0.678072	0.6853652	2	5	1	1	4	0
AC011472.2	0	#NUM!	0.5781912	0	0	3	0	0	0
AC011472.4	2	1	0.7782384	0	0	1	2	0	0
AC010883.1	0.5	-1	0.81753	2	0	0	0	0	1
AL357632.1	#DIV/0!	#DIV/0!	0.6251462	0	0	0	1	0	1
AC073934.1	0	#NUM!	0.4374838	0	4	0	0	0	0
MIR1307	0	#NUM!	0.5781912	0	0	3	0	0	0
AL354980.1	#DIV/0!	#DIV/0!	0.8030358	0	0	0	0	1	0
TRUB1	1.1567568	0.2100855	0.275214	696	405	564	400	745	781
TRUB2	0.9762353	-0.034699	0.8608377	1741	1951	2157	1832	2162	1716
EMX2OS	5	2.3219281	0.1580403	3	0	0	11	2	2
AC026412.3	#DIV/0!	#DIV/0!	0.6554062	0	0	0	0	2	0
AC005697.2	#DIV/0!	#DIV/0!	0.6411933	0	0	0	0	0	2
AC093084.1	0	#NUM!	0.5781912	0	0	3	0	0	0
AC026412.1	2.4285714	1.2801079	0.2973497	4	1	2	6	2	9
AC026412.2	1	0	0.9890559	0	1	0	0	1	0

AC130710.1	1.5	0.5849625	0.8594511	0	4	0	0	6	0
BMP8A	0.7083333	-0.4975	0.7159694	5	2	17	0	14	3
BMP8B	0.5714286	-0.807355	0.5655436	11	0	17	8	6	2
AC022695.2	#DIV/0!	#DIV/0!	0.8030358	0	0	0	1	0	0
ARCN1	0.8420693	-0.247989	0.1052139	6517	6474	7518	4368	6363	6539
MTCH1	0.8246025	-0.278229	0.0659973	6187	6977	7777	5542	6382	5344
MTCH2	0.8336352	-0.262512	0.1034207	2333	2584	2813	2097	2338	2009
MSI1	0.6808511	-0.554589	0.4758922	14	21	12	1	19	12
MSI2	1.0171571	0.0245426	0.5861113	3168	2677	3364	2416	3404	3547
RNU6-1178P	0.7142857	-0.485427	0.7560171	8	0	6	0	5	5
AL137028.1	#DIV/0!	#DIV/0!	0.3981469	0	0	0	4	0	0
AL513283.1	0	#NUM!	0.824326	1	0	0	0	0	0
MSH6	0.9801735	-0.028891	0.8755967	4451	3670	4791	3315	4685	4656
VMAC	0.9166667	-0.125531	0.8387909	25	39	32	19	38	31
AL137028.2	0	#NUM!	0.824326	1	0	0	0	0	0
MSH2	1.2719656	0.3470596	0.0126229	3209	2065	3319	2746	4202	3982
MSH3	1.0499806	0.0703627	0.460252	906	704	971	670	1075	965
MSH4	7	2.8073549	0.2945726	1	0	0	0	5	2
MSH5	0.9414414	-0.087057	0.9442694	62	58	102	43	69	97
AC010643.1	1.4	0.4854268	0.5056478	7	8	15	9	27	6
FAM222A-AS1	0	#NUM!	0.6840298	0	0	2	0	0	0
LINC00161	0	#NUM!	0.824326	1	0	0	0	0	0
RAP1B	1.1498095	0.2013949	0.0471405	2753	2338	3046	2452	3587	3317
RAP1A	1.1065428	0.1460592	0.0906532	1541	1525	1993	1632	2152	1814
NOP14	0.8853746	-0.17564	0.3337139	1262	1327	1616	1049	1348	1326
NOP16	0.8814666	-0.182022	0.4638289	1107	1336	1539	1236	1251	1023
AL160281.1	#DIV/0!	#DIV/0!	0.3981469	0	0	0	4	0	0
RFXANK	0.9630303	-0.054347	0.9112784	977	1041	1282	1120	1117	941
AC079322.1	3	1.5849625	0.6253252	0	0	1	3	0	0
G6PC3	0.8019843	-0.318354	0.1267557	1398	1615	1825	1432	1393	1055
NOP10	1.0613873	0.0859511	0.2900354	2010	2295	2716	2594	2763	2095
AC004232.3	6.25	2.6438562	0.1077591	4	0	0	0	11	14
MTCL1	0.6985816	-0.5175	0.1294815	105	77	100	53	66	78
DYSF	1.0846477	0.1172266	0.2362351	547	649	706	615	730	718
AC010789.1	1.1851852	0.2451125	0.489239	32	32	44	28	55	45
AL121603.2	0.7922078	-0.336049	0.5279669	40	48	66	46	49	27
MSLN	0.4	-1.321928	0.6181966	5	0	0	0	2	0
BICD1	0.7374179	-0.439446	0.0406968	315	257	342	189	243	242
BIRC6-AS2	0	#NUM!	0.824326	0	0	1	0	0	0
BICD2	1.1396624	0.1886065	0.0622126	2856	3115	3330	2815	3810	3975
BMP15	1	0	0.9800085	5	4	2	1	7	3
UNC80	0.4	-1.321928	0.6178933	2	2	1	2	0	0
MSL2	0.8960703	-0.158316	0.4731699	1286	1102	1480	826	1267	1373
IGBP1-AS1	0.8095238	-0.304855	0.8050485	14	0	7	2	13	2
MSL3	0.9221636	-0.116905	0.7450571	535	384	597	402	489	507
AC091271.1	0.9262295	-0.110558	0.8675212	27	49	46	27	67	19
MSL1	0.8284091	-0.271585	0.0732968	2546	2393	2981	1760	2341	2460
AL451085.1	0	#NUM!	0.5200113	2	1	0	0	0	0
HIF1AP1	#DIV/0!	#DIV/0!	0.3927105	0	0	0	1	0	3
BMP2K	1.1983356	0.2610321	0.1558113	279	208	234	220	303	341
AC008443.7	0.6582915	-0.603202	0.1299217	60	63	76	25	58	48
Z82190.1	0	#NUM!	0.4460243	4	0	0	0	0	0
AC008443.5	0.45	-1.152003	0.3243255	9	9	2	2	3	4
AC008443.6	0	#NUM!	0.6604275	0	1	1	0	0	0

AC008443.3	0.7834395	-0.352106	0.4792076	52	56	49	10	50	63
AC008443.4	0	#NUM!	0.5378194	0	2	1	0	0	0
AC008443.1	1.0365854	0.0518389	0.7517044	63	34	67	50	41	79
LINC00116	0.7002551	-0.514048	0.0384757	203	259	322	187	191	171
AL121989.1	0	#NUM!	0.824326	1	0	0	0	0	0
LINC00112	#DIV/0!	#DIV/0!	0.8030358	0	0	0	0	1	0
LINC00113	0.25	-2	0.3800775	6	0	2	1	0	1
LINC00115	1.7179487	0.780687	0.1804105	9	8	22	16	31	20
NOP56	0.9672749	-0.048002	0.8589893	6872	7458	9291	7766	8045	7037
NOP58	1.1880546	0.2486011	0.060389	4386	2605	5181	4567	5545	4349
PRKCZ-AS1	1.3408072	0.4231018	0.1143574	87	53	83	114	99	86
MIR135B	0	#NUM!	0.824326	0	0	1	0	0	0
BICC1	1	0	0.9890559	1	0	0	0	1	0
UROC1	#DIV/0!	#DIV/0!	0.8030358	0	0	0	0	1	0
LINC00106	0.9107143	-0.13493	0.9553213	11	15	30	17	15	19
EGFEM1P	0	#NUM!	0.4374838	0	4	0	0	0	0
LINC00102	0.6	-0.736966	0.7160907	2	3	0	0	2	1
NOP53	0.9697426	-0.044326	0.8749665	9698	12224	13276	11744	12118	10271
UNC79	0.7818182	-0.355095	0.6494571	24	13	18	14	10	19
MT1X	0.75	-0.415037	0.8230763	0	7	1	3	2	1
IGLV8-61	0	#NUM!	0.6385096	0	2	0	0	0	0
RCBTB1	0.8453119	-0.242444	0.2000311	930	782	1029	585	850	882
RCBTB2	0.6666667	-0.584963	0.87348	1	0	2	0	0	2
MT2A	1.7813268	0.8329522	0.0024128	107	158	142	310	274	141
TNFSF10	1.4562808	0.5422886	4.72E-06	1078	873	1297	1394	1726	1610
AP001636.3	0	#NUM!	0.355562	5	0	0	0	0	0
LINC00092	#DIV/0!	#DIV/0!	0.8030358	0	0	0	0	0	1
CYP39A1	0.7833698	-0.352235	0.3253302	186	95	176	128	120	110
MT1A	1	0	0.9756864	2	0	0	0	2	0
IDH1-AS1	1.4736842	0.5594274	0.3196127	9	12	17	19	21	16
MT1F	1.3732535	0.457598	0.0195242	142	162	197	244	258	186
MT1G	0	#NUM!	0.824326	1	0	0	0	0	0
MTCP1	1.3387097	0.4208431	0.4048076	29	10	23	25	16	42
AC004233.4	0.6428571	-0.63743	0.6441853	4	6	4	4	4	1
AC004233.2	0.4761905	-1.070389	0.3526858	11	7	3	4	3	3
AC004233.3	1.347561	0.4303506	0.1265404	48	49	67	73	71	77
AC004233.1	#DIV/0!	#DIV/0!	0.0751161	0	0	0	1	0	10
MYCBP2	1.1210863	0.1648973	0.3117045	1249	714	1167	757	1318	1434
AC040174.2	#DIV/0!	#DIV/0!	0.8030358	0	0	0	0	1	0
STK38L	0.9354184	-0.096316	0.7667965	1064	562	836	476	919	908
APOBEC3C	0.3539025	-1.498576	5.10E-29	2066	2252	2780	812	895	805
TNFSF18	0.6666667	-0.584963	0.8253677	0	1	5	0	4	0
APOBEC3D	0.9565217	-0.06413	0.9930006	9	8	6	8	4	10
MSR1	0.85	-0.234465	0.8631035	8	2	10	0	8	9
APOBEC3F	1.2201258	0.2870299	0.1972535	79	113	126	113	121	154
MIR3648-1	0.4375	-1.192645	0.3633032	4	7	5	2	2	3
AFDN-DT	1.3653137	0.4492324	0.0528683	83	71	117	109	136	125
APOBEC3G	0.6527778	-0.615336	0.3920311	13	27	32	9	32	6
TNFSF14	0.5740741	-0.800691	0.2751005	12	24	18	10	9	12
APOBEC3H	0.7298137	-0.4544	0.1716921	88	100	134	66	100	69
DDIAS	0.8934949	-0.162469	0.6122452	622	331	615	385	519	497
TNFSF12	1.4	0.4854268	0.3137369	27	11	22	27	32	25
TNFSF13	0.5365854	-0.89812	0.3201211	9	9	23	3	9	10
IGF2	1.0195496	0.0279319	0.424677	4583	4904	5654	4810	5491	5136

MSRA	0.9438202	-0.083416	0.9655133	20	32	37	47	30	7
ITGBL1	0.6857143	-0.544321	0.3997144	18	23	29	10	26	12
APOBEC3A	0	#NUM!	0.4374838	0	4	0	0	0	0
LDHAL6A	0.5833333	-0.777608	0.5985294	2	7	3	0	2	5
APOBEC3B	0.7590028	-0.397823	0.0823399	282	409	392	264	293	265
LDHAL6B	0.25	-2	0.6199202	0	0	4	0	1	0
AL161756.3	1.4117647	0.4974997	0.6023211	9	1	7	4	4	16
AL161756.1	0.9756098	-0.035624	0.9695875	6	18	17	13	16	11
MSTN	0.5	-1	0.7145709	4	0	0	0	2	0
AL161756.2	#DIV/0!	#DIV/0!	0.4242335	0	0	0	0	3	1
DMBX1	2.3333333	1.2223924	0.5352888	2	1	0	5	2	0
VMO1	0.53125	-0.912537	0.4827182	3	3	26	1	4	12
RAC2	1.2105263	0.2756344	0.8431501	3	12	4	1	17	5
RAC3	1.1766449	0.234679	0.1209206	464	581	642	801	673	511
RAC1	1.0570602	0.0800575	0.176207	17183	17254	20838	18518	20828	19083
AL390719.1	1.0357143	0.0506261	0.8133785	28	21	35	28	38	21
MST1	0.9863014	-0.0199	0.8878094	17	20	36	25	19	28
RAP2C	1.019877	0.0283951	0.6366516	2289	1803	2247	1591	2484	2390
RAP2A	1.188706	0.249392	0.0513749	1255	922	1347	1080	1468	1641
RAP2B	0.9310775	-0.103027	0.7253826	2470	2518	3021	2391	2606	2460
AC009271.1	0.3333333	-1.584963	0.6659102	3	0	0	1	0	0
IGKC	1.5550847	0.6369932	0.0099902	76	63	97	112	139	116
MSX1	1.0323383	0.0459158	0.6455008	128	124	150	154	145	116
RAE1	0.8049347	-0.313056	0.0391174	2407	2684	3096	2128	2282	2180
SLC25A5P5	0.6392405	-0.645569	0.1784532	33	52	73	34	39	28
SLC25A5P3	#DIV/0!	#DIV/0!	0.6099294	0	0	0	2	0	0
SLC25A5P2	2	1	0.807847	1	0	0	0	1	1
IGIP	1.1860465	0.2461606	0.246708	164	98	254	148	258	206
ELK2BP	2.8333333	1.5025003	0.0967773	6	1	5	14	9	11
SLC25A5P7	0.4	-1.321928	0.604695	0	4	1	1	0	1
VMP1	0.9596799	-0.059375	0.9686859	4033	4077	4638	3761	4515	3958
TACC3	0.9685783	-0.046059	0.8731	5197	6127	6880	6072	6118	5442
RAD1	0.9449369	-0.08171	0.8320605	1193	1161	1369	1054	1250	1214
TACC1	1.0832192	0.1153253	0.3615275	3586	3021	3619	2440	3918	4719
TACC2	1.3897186	0.4747927	0.0002959	1041	1226	1215	1264	1769	1806
TACO1	0.8018018	-0.318682	0.082157	859	1011	1127	808	812	783
RAG2	#DIV/0!	#DIV/0!	0.6554062	0	0	0	0	2	0
RAG1	1.352381	0.4355016	0.2998653	26	30	49	22	46	74
MSX2	1.1475799	0.1985946	0.0202698	9456	10394	11306	11172	12812	11770
PSPC1	1.034898	0.0494885	0.3231338	4410	4614	5676	4383	5577	5253
RAF1	0.8364486	-0.257651	0.0642027	3174	3163	3507	2355	3007	2872
AC092745.1	0	#NUM!	0.824326	1	0	0	0	0	0
AC092745.2	0.6666667	-0.584963	0.8466488	3	0	0	0	0	2
AC007364.1	#DIV/0!	#DIV/0!	0.6554062	0	0	0	0	2	0
RAI1	1.028777	0.0409303	0.8495634	38	44	57	28	71	44
IL36RN	1.0283881	0.0403848	0.4199799	1257	1287	1507	1251	1519	1396
TLX1NB	3.6666667	1.8744691	0.3570334	3	0	0	11	0	0
AC040914.1	0	#NUM!	0.824326	1	0	0	0	0	0
AC131934.1	0	#NUM!	0.6840298	0	0	2	0	0	0
AC025574.1	#DIV/0!	#DIV/0!	0.6411933	0	0	0	0	0	2
AC084032.1	0	#NUM!	0.2038809	4	0	3	0	0	0
AC091178.1	1	0	0.9278662	6	17	0	0	16	7
EIF3LP3	2	1	0.658	0	0	3	0	0	6
EIF3LP2	6	2.5849625	0.3030898	0	0	1	2	3	1

SCAMP1-AS1	0.9050132	-0.143989	0.7684382	136	104	139	117	152	74
RALA	0.9840583	-0.023184	0.725331	1549	1540	1992	1580	1730	1690
RALB	0.9738239	-0.038267	0.8390265	3305	3480	4141	3284	3688	3668
AC017015.2	0.25	-2	0.3414811	1	3	8	3	0	0
MTA1	0.9236288	-0.114615	0.6998801	1302	1378	1641	1316	1372	1303
OXCT1	#DIV/0!	#DIV/0!	0.263214	0	0	0	0	5	1
DROSHA	0.9155466	-0.127295	0.5131927	3339	2987	3644	2370	3440	3318
OXCT2	0.375	-1.415037	0.624337	0	0	8	0	0	3
RALY	0.9484899	-0.076296	0.9516186	6134	7556	8461	7263	7345	6402
MTA2	0.830736	-0.267538	0.1043037	3607	4359	4996	3474	3790	3504
MTA3	1.1034514	0.1420231	0.0641197	3386	3313	4369	3602	4511	4100
HSD3B7	1.25	0.3219281	0.8311002	2	3	3	0	4	6
HSD3B1	1.3990461	0.4844435	3.95E-05	752	808	956	993	1256	1271
ACTBP11	0.2222222	-2.169925	0.3360621	4	1	4	2	0	0
ACTBP12	0	#NUM!	0.4374838	0	4	0	0	0	0
AC012456.2	7	2.8073549	0.2923354	0	0	1	0	1	6
GOLGA5P1	1.1538462	0.2064509	0.8245534	5	2	6	4	9	2
TNIP1	1.0488122	0.0687564	0.3103907	2434	2871	3156	3010	3044	2820
AP001970.1	1.35	0.4329594	0.6229636	3	6	11	2	11	14
TNIP2	1.1320803	0.1789763	0.0761679	2199	2615	2954	3052	3094	2648
TNIP3	0.2941176	-1.765535	0.2496696	11	5	1	0	5	0
AC024361.2	3.5	1.8073549	0.4382657	0	0	2	0	5	2
IPO13	1.0150781	0.0215907	0.5643271	517	599	741	584	635	666
AC024361.3	0.8181818	-0.289507	0.8261262	6	2	3	0	8	1
IPO11	0.9680328	-0.046872	0.9785918	1358	1019	1283	910	1340	1293
AC024361.1	2.25	1.169925	0.5911365	3	1	0	0	8	1
CAPN15	0.8534258	-0.228662	0.2177109	1312	1578	1722	1284	1399	1253
MTBP	0.8429003	-0.246566	0.3984538	256	162	244	142	197	219
HOMER3	0.7037037	-0.50696	0.5771849	15	20	46	32	11	14
MBNL1	0.8613652	-0.215303	0.4315144	597	313	511	321	464	439
MBNL2	1.0017441	0.002514	0.8071354	1882	1042	1663	1097	1691	1807
AC138392.1	0.9123823	-0.13229	0.6205287	478	416	487	260	492	508
AP4B1-AS1	0.2	-2.321928	0.2634847	3	2	5	0	0	2
TAAR1	#DIV/0!	#DIV/0!	0.3246733	0	0	0	0	3	2
AL391152.1	0.9651163	-0.051225	0.9967355	30	21	35	20	33	30
MTAP	1.0761527	0.1058827	0.2056848	1927	1734	2130	1698	2224	2310
AL592528.1	#DIV/0!	#DIV/0!	0.8030358	0	0	0	0	1	0
MTDH	1.0027994	0.004033	0.7718218	1220	865	1130	814	1205	1205
HOMER1	1.0075042	0.0107859	0.7901641	1541	1145	1445	927	1590	1645
HOMER2	0.6521739	-0.616671	0.3648445	17	39	13	3	27	15
AC074032.1	1.4444444	0.5305147	0.6533646	2	3	4	2	4	7
AL049844.1	#DIV/0!	#DIV/0!	0.5509701	0	0	0	0	3	0
AL049844.2	0.7916667	-0.337035	0.6666215	12	15	21	7	19	12
AL121594.3	0.5	-1	0.7382045	2	1	1	2	0	0
CDKN1C	1.6470588	0.7198921	0.4544902	0	8	9	6	12	10
SETD4	1.1501632	0.2018386	0.2014692	336	272	311	376	400	281
SETD5	0.8474011	-0.238883	0.1490588	5068	4617	5610	3107	4931	4923
CDKN1A	0.7676902	-0.381404	0.0078844	6332	7538	7950	5331	6134	5286
RARG	0.8634361	-0.211839	0.6046572	123	157	174	137	101	154
SETD2	1.0434262	0.0613286	0.5413827	2194	1480	2175	1410	2420	2273
AC245128.3	1.25	0.3219281	0.9083873	4	0	0	0	1	4
CDKN1B	0.9558011	-0.065218	0.9236829	1930	1968	2256	1747	2127	2008
SETD3	0.9329238	-0.100169	0.6661288	6904	6713	8194	6008	7566	6774
SETD9	1.0644444	0.0901007	0.6008695	179	150	121	183	149	147

SETD6	0.781477	-0.355725	0.0507974	511	516	625	354	458	479
SETD7	0.9074316	-0.140139	0.5321574	1323	1183	1329	778	1280	1422
AC005696.4	0	#NUM!	0.1905915	0	1	7	0	0	0
AC005696.3	#DIV/0!	#DIV/0!	0.6411933	0	0	0	0	0	2
AL512599.1	0	#NUM!	0.3049182	1	1	3	0	0	0
AC005696.1	0	#NUM!	0.1289326	1	3	4	0	0	0
MTG1	1.2932692	0.3710226	0.1432685	67	58	83	79	95	95
MTG2	0.8433374	-0.245818	0.1801368	1563	2014	2104	1528	1624	1639
RARS	1.0497031	0.0699813	0.2851572	6988	5521	7530	6410	7868	6757
AC087641.1	4.5	2.169925	0.1373185	0	0	4	14	3	1
LRRC75A-AS1	1.0761996	0.1059457	0.1879107	6543	7989	8434	8002	8930	7784
MTF1	1.0361744	0.0512669	0.5129357	665	582	771	527	790	774
MTF2	1.1418238	0.1913401	0.1108763	1422	990	1459	1176	1653	1591
RARA	0.8018779	-0.318545	0.1509142	314	383	368	275	312	267
RARB	0.3333333	-1.584963	0.544375	0	1	5	0	1	1
OR8A3P	#DIV/0!	#DIV/0!	0.8030358	0	0	0	0	0	1
TOMM20P1	1	0	0.9890559	0	0	1	0	1	0
TOMM20P2	#DIV/0!	#DIV/0!	0.8030358	0	0	0	0	1	0
ARRDC1-AS1	1.0201058	0.0287188	0.5454183	598	609	683	663	651	614
OLFML3	0.7857143	-0.347923	0.7192594	10	23	9	18	11	4
TOMM20P4	0.98125	-0.027307	0.9750454	59	60	41	55	58	44
OLFML1	1.0190833	0.027272	0.4998866	1757	1974	2138	1777	2153	2051
AC010884.1	0.125	-3	0.2737675	8	0	0	0	1	0
MBNL3	1.4368504	0.5229098	0.0008337	9378	5657	9209	8272	13282	13281
CDKN2D	0.817734	-0.290296	0.5348451	47	71	85	46	84	36
CDKN2B	0.8378378	-0.255257	0.7380467	14	23	37	8	24	30
CDKN2C	0.984127	-0.023084	0.9383304	60	65	64	58	73	55
CDKN2A	1.0071367	0.0102595	0.6210848	1136	1643	1845	1690	1612	1355
OR8A2P	1.6666667	0.7369656	0.620272	1	3	2	2	6	2
SNCAIP	4.4	2.1375035	0.0165002	4	4	2	10	16	18
OSTM1	0.7959841	-0.329189	0.0398168	2203	1849	2472	1302	2038	1853
AC107079.1	2.5	1.3219281	0.6099065	0	0	2	0	0	5
SETP4	#DIV/0!	#DIV/0!	0.6099294	0	0	0	2	0	0
SETP2	#DIV/0!	#DIV/0!	0.2887809	0	0	0	0	0	6
SETP3	1	0	0.9356597	0	0	3	1	0	2
SETP8	1.25	0.3219281	0.9443313	0	4	0	0	5	0
SETP9	#DIV/0!	#DIV/0!	0.8030358	0	0	0	0	0	1
AC004471.1	1.7142857	0.7776076	0.424485	2	2	10	3	6	15
WWTR1-IT1	0	#NUM!	0.6840298	0	0	2	0	0	0
LARP4P	1	0	0.9890559	0	1	0	1	0	0
AL031316.1	1	0	0.9890559	1	0	0	1	0	0
LARP4B	1.0061744	0.0088803	0.661926	3890	3996	4261	3272	4477	4473
AC024267.3	1.1	0.1375035	0.8725746	8	4	8	1	4	17
AC024267.4	0.1666667	-2.584963	0.4507335	0	0	6	0	0	1
AC024267.5	0.3333333	-1.584963	0.60684	0	0	6	0	2	0
MTM1	1.0100629	0.0144451	0.6959407	272	210	313	229	283	291
AL133299.1	11	3.4594316	0.0904896	1	0	0	6	1	4
LINC00032	0.5555556	-0.847997	0.690814	4	0	5	3	1	1
AC004471.2	1	0	0.9890559	1	0	0	0	1	0
BMPR2	1.1133104	0.1548559	0.2716453	1269	1099	1118	911	1400	1570
MTO1	1.0740312	0.1030359	0.2837569	577	472	680	539	708	610
BX890604.1	1.0104839	0.0150463	0.6446214	378	367	495	352	433	468
SERAC1	1.01566	0.0224175	0.8319047	148	143	156	92	170	192
AC100821.1	1.3333333	0.4150375	0.7882135	0	0	6	0	4	4

AC098864.1	1.0870185	0.1203766	0.351469	201	222	278	230	240	292
AC100821.2	1	0	0.9837228	2	0	0	0	1	1
AVPR2	0.85	-0.234465	0.8033463	8	9	3	3	10	4
LANCL1	1.0300668	0.0427379	0.6077734	1569	1309	1612	1063	1705	1857
LANCL2	0.8360656	-0.258312	0.2307157	363	417	501	313	376	382
MTPN	0.8627901	-0.212919	0.2625799	2459	2408	2545	1422	2387	2586
AL008733.1	0	#NUM!	0.284725	6	0	0	0	0	0
AC004231.1	3	1.5849625	0.2364317	1	1	3	3	6	6
MIR148A	#DIV/0!	#DIV/0!	0.6554062	0	0	0	0	2	0
MIR148B	#DIV/0!	#DIV/0!	0.5371397	0	0	0	0	0	3
AC008588.1	#DIV/0!	#DIV/0!	0.288876	0	0	0	3	2	0
MTOR	0.8621726	-0.213951	0.1879868	2557	2772	2993	1917	2552	2706
GLOD4	0.8411542	-0.249558	0.1032808	3012	3402	3602	2644	2974	2807
GLOD5	0.3333333	-1.584963	0.474059	3	1	2	0	1	1
OLFM5P	0.6666667	-0.584963	0.6197231	12	4	5	6	3	5
MTRR	0.9456265	-0.080658	0.7958512	1827	1471	1778	1278	1763	1759
WDSUB1	1.1210407	0.1648387	0.1352067	601	537	630	575	745	662
ZMYND8	1.0496427	0.0698983	0.2865395	5251	4543	5878	4558	5852	6040
MKI67	1.1859482	0.246041	0.0663781	8366	6888	8871	6452	10337	11822
KIAA0930	0.6950236	-0.524866	0.0011947	1041	1210	1346	837	841	822
AL596094.1	1.2222222	0.2895066	0.7440593	4	2	12	1	10	11
ABCA9-AS1	4	2	0.2683413	0	3	0	4	1	7
AC104938.1	0	#NUM!	0.6840298	0	0	2	0	0	0
RPL37P18	#DIV/0!	#DIV/0!	0.5371397	0	0	0	0	0	3
RPL37P23	1	0	0.892289	8	2	8	12	3	3
RPS2P55	1.1527778	0.2051144	0.6396283	13	39	20	39	22	22
AC002076.1	0	#NUM!	0.824326	0	0	1	0	0	0
AC002076.2	0	#NUM!	0.6436235	2	0	0	0	0	0
MTTP	#DIV/0!	#DIV/0!	0.1433497	0	0	0	2	3	2
RPS2P45	1.1111111	0.1520031	0.889564	2	0	7	0	6	4
RPS2P46	0.9733666	-0.038945	0.8479817	2197	2440	2572	2594	2358	2065
VNN1	6	2.5849625	0.139986	1	1	0	6	3	3
ZZZ3	1.1204143	0.1640323	0.2586657	882	532	903	625	1049	922
AC069431.1	0.25	-2	0.5375562	3	0	1	0	1	0
VNN2	2.6521739	1.4071754	0.0684384	10	5	8	7	38	16
RPS2P36	0	#NUM!	0.1842387	1	6	0	0	0	0
RBAK	0.9307292	-0.103567	0.8625189	756	347	817	475	685	627
AC073349.2	0.4666667	-1.099536	0.4469652	10	1	4	0	5	2
RPS2P32	0.7096774	-0.494765	0.5975654	17	6	8	7	10	5
RANP3	0	#NUM!	0.824326	0	0	1	0	0	0
AC023043.1	0.6666667	-0.584963	0.8272397	3	0	0	0	1	1
RANP4	0	#NUM!	0.355562	5	0	0	0	0	0
TENM1	1.1359223	0.1838642	0.6282632	36	19	48	21	57	39
RANP1	1.1232877	0.1677274	0.6707858	19	30	24	30	33	19
TENM2	2.5	1.3219281	0.633036	2	0	0	0	3	2
AC105036.2	1.5	0.5849625	0.8552295	1	1	0	0	0	3
TENM3	1.0627258	0.0877694	0.4245144	4204	3707	4269	2947	4666	5331
AC023043.4	0.25	-2	0.5318227	3	0	1	1	0	0
AC105036.3	0	#NUM!	0.026465	9	1	4	0	0	0
TENM4	0.8333333	-0.263034	0.803204	1	25	10	6	5	19
RANP8	0.6666667	-0.584963	0.8238707	1	1	1	0	2	0
RPL13P12	1.0504386	0.0709918	0.4750614	241	314	357	358	310	290
RNU6V	1.6666667	0.7369656	0.7845573	1	2	0	0	4	1
RPS2P28	0	#NUM!	0.824326	0	0	1	0	0	0

RPS2P25	2.5	1.3219281	0.6334128	0	2	0	0	2	3
SLC25A6P5	1.3055556	0.3846639	0.5030084	11	9	16	13	12	22
SLC25A6P2	0	#NUM!	0.824326	0	0	1	0	0	0
SLC25A6P3	0	#NUM!	0.6840298	0	0	2	0	0	0
AC006141.1	0	#NUM!	0.824326	0	0	1	0	0	0
AVPI1	1.630888	0.7056577	8.13E-06	335	450	510	697	789	626
AL133153.1	0.2307692	-2.115477	0.2891144	8	0	5	0	0	3
AL133153.2	0	#NUM!	0.1496384	0	7	1	0	0	0
RBFA	0.760274	-0.395409	0.2744744	103	71	118	72	79	71
RPL37P10	0	#NUM!	0.6840298	0	0	2	0	0	0
MTX1	0.9623041	-0.055435	0.9433701	614	824	923	756	789	727
MTX2	0.982169	-0.025957	0.7875798	1259	1321	1514	1254	1385	1382
AL353662.2	0.1818182	-2.459432	0.2496928	1	1	9	1	1	0
MTX3	0	#NUM!	0.5387425	3	0	0	0	0	0
GOLGA6L9	0.8121547	-0.300174	0.6259546	62	23	96	38	70	39
BMPER	#DIV/0!	#DIV/0!	0.6411933	0	0	0	0	0	2
GOLGA6L6	#DIV/0!	#DIV/0!	0.8030358	0	0	0	1	0	0
GOLGA6L7	0.2391304	-2.06413	0.04133	9	17	20	6	5	0
GOLGA6L4	#DIV/0!	#DIV/0!	0.8030358	0	0	0	0	1	0
HCG9P5	10	3.3219281	0.1600848	1	0	0	0	3	7
AL391058.1	1.4848485	0.5703157	0.0830779	34	48	50	53	75	68
GOLGA6L2	0.4166667	-1.263034	0.396315	3	6	3	1	3	1
GOLGA6L3	0	#NUM!	0.6840298	0	0	2	0	0	0
CDPF1	0.883612	-0.178515	0.4773448	405	508	582	413	474	434
GOLGA6L1	#DIV/0!	#DIV/0!	0.5070017	0	0	0	3	0	0
RSBN1	0.9358717	-0.095617	0.8351836	365	241	392	214	334	386
AC011471.4	1	0	0.8977569	1	0	7	4	0	4
AC025575.1	#DIV/0!	#DIV/0!	0.8030358	0	0	0	0	1	0
AC025575.2	0.8368201	-0.257011	0.5297801	72	65	102	45	88	67
ZMAT1	0	#NUM!	0.0988206	2	2	5	0	0	0
AC005695.1	0.2222222	-2.169925	0.3109026	1	4	4	0	0	2
ZMAT2	0.9826887	-0.025194	0.7471563	5184	5610	6709	5513	6305	5382
ZMAT3	0.700105	-0.514357	0.0092619	2267	1483	1962	877	1546	1576
ZMAT5	0.7296037	-0.454815	0.1565646	93	158	178	76	124	113
MPIG6B	1.6304348	0.7052567	0.2245947	24	10	12	40	21	14
AC007365.1	0.8181818	-0.289507	0.8283562	6	2	3	0	6	3
ST7-OT4	#DIV/0!	#DIV/0!	0.136155	0	0	0	3	2	2
AC090589.1	5	2.3219281	0.4145588	0	0	1	2	3	0
AC090589.3	1.3333333	0.4150375	0.4399042	6	16	20	22	15	19
RBM3	0.6173057	-0.695943	1.55E-08	4402	4410	5403	2799	3156	2820
RBM4	0.7664671	-0.383704	0.2671823	138	82	114	56	121	79
RBM7	1.0218341	0.0311609	0.6241765	862	587	841	670	952	718
RBM5	0.8707281	-0.199706	0.2905641	1549	1274	1888	1153	1576	1373
RBM6	0.8563202	-0.223778	0.2106614	2045	2000	2901	1788	2144	2016
RBL2	0.9506399	-0.073029	0.8823694	589	422	630	364	613	583
RBL1	0.9700738	-0.043834	0.9614545	965	649	959	671	927	898
RBKS	1.2511628	0.3232695	0.2378587	70	78	67	100	100	69
TSGA10IP	#DIV/0!	#DIV/0!	0.8030358	0	0	0	0	0	1
BICRA	0.9864576	-0.019671	0.8165395	911	936	959	898	1023	847
MUC1	1.5352113	0.6184372	0.1610043	17	27	27	51	28	30
MUC2	0.3333333	-1.584963	0.7307796	0	0	3	0	1	0
POC1A	0.8300836	-0.268672	0.1378998	1329	1560	1778	1280	1418	1176
POC1B	0.9126595	-0.131851	0.6572364	375	276	368	245	342	343
MUC4	0.7245509	-0.464841	0.2910095	54	54	59	37	33	51

MUC6	4	2	0.4849955	1	0	0	3	1	0
NOC2LP1	0.9252336	-0.11211	0.7031157	768	882	1025	763	934	778
NOC2LP2	2.3636364	1.2410081	0.1994785	1	4	6	9	8	9
CAPN13	0.5714286	-0.807355	0.6973127	4	0	3	0	2	2
CAPN14	3	1.5849625	0.2694704	4	0	1	5	6	4
CAPN11	1	0	0.9357122	1	1	4	2	0	4
CAPN12	1.137457	0.1858121	0.3565659	78	100	113	116	115	100
CAPN10	1.1271571	0.1726886	0.1344997	659	728	815	914	811	757
RBMX	0.9918951	-0.011741	0.6446507	10516	9100	12340	9626	11418	10653
AL139423.1	#DIV/0!	#DIV/0!	0.8030358	0	0	0	0	0	1
RBPJ	1.1665908	0.2222985	0.0870243	3317	2194	3271	2573	3719	3953
ZFYVE28	1.6	0.6780719	0.7016658	2	3	0	3	4	1
ZFYVE26	0.9178284	-0.123704	0.5936553	1765	2153	2179	1435	1930	2231
RNU2-7P	0	#NUM!	0.824326	0	0	1	0	0	0
ZFYVE27	1.0464094	0.0654474	0.4348258	579	725	743	674	802	666
UBASH3B	0.6289414	-0.669002	0.0001408	578	616	582	315	401	401
ARHGEF38-IT	0	#NUM!	0.6436235	2	0	0	0	0	0
AL445309.1	0.3571429	-1.485427	0.3619061	7	4	3	0	0	5
AC007998.5	0	#NUM!	0.5075476	0	0	4	0	0	0
AC007998.3	1.6	0.6780719	0.6362255	2	0	3	6	0	2
ZFYVE21	1.0925203	0.1276601	0.1467537	1742	2072	2336	2205	2415	2099
AC127455.1	1.1818182	0.2410081	0.7837895	2	3	6	4	4	5
RBP5	0.8333333	-0.263034	0.8655088	5	1	0	0	5	0
ZFYVE16	1.1293353	0.1754738	0.6148007	1254	477	1037	670	1199	1257
RBP2	0.5116279	-0.966833	0.2421688	21	14	8	2	13	7
RBP3	0	#NUM!	0.824326	1	0	0	0	0	0
ZFYVE19	0.904376	-0.145005	0.6014376	543	582	726	545	623	506
RBP1	0.8965517	-0.157541	0.9540701	12	5	12	13	6	7
RNU6-1191P	#DIV/0!	#DIV/0!	0.0563836	0	0	0	3	3	4
CEP85L	0.951567	-0.071623	0.8764459	165	74	112	61	157	116
AL357874.1	0	#NUM!	0.6385096	0	2	0	0	0	0
AL357874.2	0.8181818	-0.289507	0.9056163	11	0	0	7	1	1
RNU7-71P	#DIV/0!	#DIV/0!	0.5509701	0	0	0	0	3	0
AC087392.1	#DIV/0!	#DIV/0!	0.1506188	0	0	0	8	0	0
AC087392.2	#DIV/0!	#DIV/0!	0.0560939	0	0	0	1	1	9
AC087392.3	0.5698925	-0.811238	0.1694965	23	35	35	6	26	21
AC087392.4	0.4193548	-1.253757	0.197565	16	10	5	4	5	4
AC087392.5	0.3846154	-1.378512	0.4091408	9	1	3	0	5	0
AC005699.1	#DIV/0!	#DIV/0!	0.8030358	0	0	0	0	1	0
MACROD2-IT	0	#NUM!	0.824326	0	1	0	0	0	0
LINC00315	1.7142857	0.7776076	0.5692697	4	2	1	9	3	0
LINC00310	0.3529412	-1.5025	0.3052165	9	1	7	0	4	2
LINC00311	0	#NUM!	0.6385096	0	2	0	0	0	0
AC138393.3	0.2413793	-2.050626	0.1329245	20	0	9	1	1	5
EDEM3	0.9521902	-0.070678	0.8802728	1597	918	1480	805	1426	1573
AC138393.1	0.9731544	-0.039259	0.918317	103	53	142	69	98	123
AC138393.2	0	#NUM!	0.6631647	1	0	1	0	0	0
AC093826.2	2	1	0.6581292	0	0	2	1	2	1
EDEM1	1.0362875	0.0514243	0.4811946	2527	2093	2545	1911	2719	2795
EDEM2	0.936333	-0.094906	0.8443331	2181	2659	2872	2537	2515	2169
AL512310.7	1.2272727	0.2954559	0.7033464	4	11	7	10	12	5
AL512310.6	#DIV/0!	#DIV/0!	0.1307332	0	0	0	3	0	5
AL512310.3	1.875	0.9068906	0.4689886	5	2	1	7	6	2
AL512310.4	5	2.3219281	0.4606115	1	0	0	0	0	5

AL512310.2	2	1	0.7094398	0	1	1	0	0	4
LINC00308	0.7777778	-0.36257	0.5314016	32	30	28	7	28	35
LINC00304	1	0	0.9239532	0	0	2	2	0	0
LINC00301	0	#NUM!	0.824326	0	1	0	0	0	0
AC103858.1	#DIV/0!	#DIV/0!	0.0734026	0	0	0	8	2	0
AC103858.2	1.7142857	0.7776076	0.5498002	0	2	5	3	5	4
RNU7-77P	#DIV/0!	#DIV/0!	0.1513955	0	0	0	0	4	4
PP7080	0.7879548	-0.343815	0.1551544	241	272	284	202	248	178
AL356801.1	0.7272727	-0.459432	0.7885532	2	3	6	0	2	6
GOLGA2P8	1.3461538	0.4288433	0.5481517	11	6	9	9	15	11
GOLGA2P6	0	#NUM!	0.5075476	0	0	4	0	0	0
GOLGA2P7	1.0666667	0.0931094	0.8254625	32	14	14	20	17	27
RBSN	0.7553812	-0.404723	0.0123058	1433	1509	1518	846	1240	1283
GOLGA2P5	0.8445532	-0.24374	0.4333593	271	182	364	167	260	263
ADSSL1	0.4833333	-1.04891	0.0483733	31	37	52	10	30	18
AC074035.1	#DIV/0!	#DIV/0!	0.8030358	0	0	0	0	1	0
DSCAML1	0.625	-0.678072	0.7189466	1	6	1	3	2	0
AC007362.1	0.8	-0.321928	0.9693107	1	1	3	4	0	0
RNU7-75P	0.5	-1	0.7326105	1	0	3	0	1	1
GLRA1	3	1.5849625	0.6684577	1	0	0	0	0	3
GLRA2	5	2.3219281	0.4606115	1	0	0	0	0	5
GLRA3	2.3333333	1.2223924	0.5590223	0	0	3	0	1	6
DYNLL1P1	#DIV/0!	#DIV/0!	0.6482162	0	0	0	0	1	1
GHET1	1.3478261	0.4306344	0.433351	13	21	12	24	20	18
GLRA4	0.3076923	-1.70044	0.2913141	1	6	6	1	2	1
AL359541.1	0.6923077	-0.530515	0.7285647	2	6	5	4	2	3
ASPRV1	0.5454545	-0.874469	0.2958566	9	15	20	8	7	9
AC027243.2	#DIV/0!	#DIV/0!	0.3831122	0	0	0	0	5	0
AC027243.1	2.6666667	1.4150375	0.4355249	1	0	2	1	3	4
SERBP1	1.0412722	0.0583473	0.2798327	19329	16998	21242	17559	21583	20803
ETFBKMT	0.7945205	-0.331844	0.4532286	49	55	42	32	47	37
AL021918.1	0.2	-2.321928	0.453612	0	5	0	0	1	0
AL121601.1	2	1	0.6954115	0	3	0	0	1	5
AL121601.2	3	1.5849625	0.6896151	1	0	0	0	3	0
MUM1	0.876819	-0.189649	0.2858395	943	949	1063	722	993	876
SMCHD1	1.0492466	0.0693538	0.5949302	1119	608	994	639	1129	1087
LINC00379	2.5	1.3219281	0.6101576	2	0	0	1	0	4
AREL1	0.7543692	-0.406657	0.0109593	1873	2168	2253	1177	1762	1809
LINC00375	0	#NUM!	0.824326	0	1	0	0	0	0
LINC00370	0	#NUM!	0.6436235	2	0	0	0	0	0
PPP1R16A	1.2542955	0.3268773	0.0837563	182	203	197	284	220	226
MUL1	0.8580974	-0.220787	0.3079587	836	1094	1213	888	985	824
RBX1	0.9836173	-0.023831	0.7706229	1804	2052	2248	2102	2129	1773
LINC00365	1.4	0.4854268	0.7474855	0	0	5	3	4	0
TUBGCP2	1.1783091	0.2367181	0.0375519	1676	2026	2153	2387	2485	2027
AL121987.2	0.3947368	-1.341037	0.1975643	7	20	11	0	3	12
LINC00397	0	#NUM!	0.1231141	6	0	3	0	0	0
BICRAL	0.8197802	-0.286691	0.244235	243	341	326	181	278	287
LINC00393	#DIV/0!	#DIV/0!	0.5509701	0	0	0	0	3	0
TUBGCP5	1.1706811	0.2273481	0.0616285	1755	1172	1889	1545	2134	1959
FGFR1OP	1.1223242	0.1664894	0.2988267	214	152	288	223	304	207
TUBGCP6	0.8876286	-0.171972	0.3670252	1171	1150	1470	985	1180	1200
TUBGCP3	0.8935558	-0.16237	0.3499124	1716	1624	1874	1298	1707	1654
TUBGCP4	0.8319914	-0.265359	0.2025625	605	526	732	379	562	609

MOXD1	7	2.8073549	0.119908	0	2	0	4	7	3
LINC00342	1.6214689	0.6973014	0.0822788	62	11	104	113	88	86
PPP1R13B	0.6555556	-0.60921	0.0117954	208	214	208	121	137	155
PPP1R13L	0.851258	-0.232332	0.1711356	3438	3943	4344	3303	3486	3192
LINC00337	1.2742382	0.349635	0.1229621	113	97	151	202	139	119
LINC00339	0.7768987	-0.364202	0.142205	195	186	251	139	185	167
LINC00332	0	#NUM!	0.6436235	2	0	0	0	0	0
LINC00334	1	0	0.978278	0	0	2	0	2	0
LINC00330	0.25	-2	0.547139	0	4	0	0	1	0
PPP1R12A	1.2071474	0.2716018	0.1076436	2528	1355	2441	1709	2992	2933
TMED10P2	0.1428571	-2.807355	0.2915418	1	1	5	0	0	1
AC026904.1	0	#NUM!	0.5387425	3	0	0	0	0	0
PPP1R12B	0.8939406	-0.161749	0.4414277	4412	3424	4591	2602	4052	4455
PPP1R12C	1.0575529	0.0807298	0.2866034	1960	2291	2647	2561	2540	2194
LINC00324	0.1578947	-2.662965	0.024432	5	13	20	1	5	0
PPP1R15A	0.9689729	-0.045472	0.8646782	1549	1836	2223	1875	1971	1588
PPP1R15B	1.056777	0.079671	0.3345892	11406	9687	12336	8877	12881	13569
LINC00364	0	#NUM!	0.6840298	0	0	2	0	0	0
KATNB1	1.0614919	0.0860934	0.2945465	825	996	1155	1080	1049	1030
FURIN	1.1039843	0.1427197	0.1002578	5496	6845	7537	6470	7946	7529
KATNA1	0.9102725	-0.13563	0.6158005	774	694	917	704	755	712
LINC00355	0.8639456	-0.210988	0.6689048	33	59	55	23	59	45
PPP1R14A	1.5	0.5849625	0.6525389	5	5	0	7	1	7
PPP1R14B	0.9864999	-0.019609	0.7219073	2409	3038	3590	3403	3075	2437
PPP1R14C	1.6111111	0.688056	0.4895243	11	6	1	8	13	8
LINC00348	3	1.5849625	0.6896151	1	0	0	0	3	0
CDR2L	0.9463415	-0.079567	0.9835019	58	72	75	89	53	52
PPP1R14D	0.8571429	-0.222392	0.9159412	4	0	3	1	4	1
LINC00346	0.7759563	-0.365953	0.4201047	80	59	44	51	39	52
MIR106B	#DIV/0!	#DIV/0!	0.3246733	0	0	0	0	3	2
POF1B	0.3130435	-1.675565	0.0001176	82	50	98	18	20	34
DHX57	1.1758721	0.2337311	0.0457278	1237	906	1297	1122	1446	1477
DHX58	5.1875	2.3750394	5.30E-07	8	9	15	54	58	54
OXER1	0	#NUM!	0.5075476	0	0	4	0	0	0
RCC1	0.9342171	-0.09817	0.7210075	2920	3420	3617	2883	3452	2967
AC113608.1	0	#NUM!	0.6436235	2	0	0	0	0	0
AC113608.2	2	1	0.8147319	1	0	0	0	0	2
AC104687.2	1.6666667	0.7369656	0.7286512	1	0	2	0	2	3
MUSK	1	0	0.9756864	2	0	0	0	2	0
AC083836.1	0	#NUM!	0.6840298	0	0	2	0	0	0
DHX40	1.0941567	0.1298193	0.2314587	1369	1110	1440	1071	1578	1639
MXRA8	1.2903383	0.3677493	0.0007747	6596	7532	8487	10084	10171	8926
MXRA7	0.7115033	-0.491058	0.0073527	932	1152	1202	818	841	679
PEX11G	0.785	-0.349235	0.5397361	48	75	77	78	47	32
MXRA5	0.4808429	-1.056362	1.64E-05	324	350	370	107	161	234
MMADHC	1.2009229	0.2641435	0.0030954	3192	3222	3988	3825	4622	4045
FKBP1AP1	0	#NUM!	0.4217812	0	0	5	0	0	0
FKBP1AP3	#DIV/0!	#DIV/0!	0.8030358	0	0	0	0	0	1
PAXIP1	0.7926063	-0.335324	0.0644364	874	803	1028	507	837	800
PEX11A	1.0821256	0.113868	0.6278943	83	55	69	64	92	68
PEX11B	0.8437118	-0.245178	0.3700126	234	290	295	237	260	194
PTTG1IP	1.0188704	0.0269706	0.4842253	6772	7800	8162	6939	8258	7966
DHX30	0.862675	-0.213111	0.2083773	2857	3299	3631	2667	3148	2628
DHX32	1.1701202	0.2266568	0.0351683	1334	1166	1409	1234	1748	1592

DHX33	0.7135696	-0.486874	0.0033064	1882	1817	2086	981	1473	1674
DHX34	0.9061099	-0.142242	0.5557174	1086	1125	1357	1045	1072	1116
DHX35	0.8929961	-0.163274	0.469821	629	673	754	585	684	567
DHX36	1.1439532	0.1940281	0.1793805	1027	604	1106	787	1115	1229
DHX37	0.6993638	-0.515885	0.0019596	1284	1803	1786	1013	1206	1189
DHX38	0.8260995	-0.275612	0.0739444	2953	3590	3825	2483	3012	3070
RCE1	0.8880466	-0.171293	0.3843927	1032	1181	1217	951	1118	977
AC073050.1	0	#NUM!	0.824326	1	0	0	0	0	0
RCC2	1.0147868	0.0211767	0.543411	8389	9602	10345	7975	10562	10218
DHX29	0.9645276	-0.052106	0.9948613	2218	1172	2220	1366	2124	1921
AC104447.1	0.42	-1.251539	0.1108879	22	11	17	4	10	7
AP001001.1	1.2857143	0.3625701	0.7394836	5	4	5	1	6	11
DHX15	0.9778175	-0.032363	0.9056926	6655	5079	6749	4782	6734	6557
DHX16	0.8816531	-0.181717	0.3464848	1400	1521	1870	1337	1519	1368
FLJ42969	0.8846154	-0.176878	0.7640274	31	34	13	20	27	22
LINC00299	0	#NUM!	0.1445379	1	7	0	0	0	0
AL590714.1	0	#NUM!	0.824326	0	1	0	0	0	0
LINC00294	0.8328912	-0.2638	0.4385995	127	101	149	96	106	112
AL513534.1	1.4129353	0.4986954	0.0287901	245	112	246	258	351	243
AL135791.1	3.4285714	1.7776076	0.1355122	1	5	1	5	7	12
NORAD	0.8466541	-0.240155	0.1911466	8031	6651	7614	4231	7057	7589
MAD2L2	1.0241814	0.0344712	0.5674626	556	713	716	694	733	606
MAD2L1	1.1203204	0.1639114	0.0624928	2034	1856	2227	2025	2587	2241
BRWD1-IT1	0	#NUM!	0.824326	0	0	1	0	0	0
AC084030.1	5.5	2.4594316	0.0690691	0	0	4	10	8	4
TERB1	0	#NUM!	0.824326	0	0	1	0	0	0
HS1BP3-IT1	0	#NUM!	0.5643268	1	0	2	0	0	0
TAF5L	0.9015444	-0.14953	0.4190192	2868	2407	3013	1953	2926	2593
TOMM22P2	0	#NUM!	0.824326	0	1	0	0	0	0
PSPHP1	#DIV/0!	#DIV/0!	0.5509701	0	0	0	0	3	0
NFXL1	1.1414276	0.1908394	0.1698293	567	357	575	473	620	618
GATA6-AS1	1.3753943	0.4598453	0.0580423	109	88	120	161	172	103
GULP1	0.8586006	-0.219941	0.3812498	1101	634	1009	510	905	941
RCL1	0.9756965	-0.035496	0.86799	1043	1130	1201	1056	1124	1112
TAF4B	0.8865546	-0.173719	0.554493	339	233	380	196	347	301
AC025430.1	0.0952381	-3.392317	0.0355659	13	3	5	0	1	1
IFT74-AS1	#DIV/0!	#DIV/0!	0.5070017	0	0	0	3	0	0
DNAH17-AS1	1	0	0.9890559	1	0	0	0	0	1
RCC2P6	2.1666667	1.1154772	0.512134	4	0	2	0	1	12
GLRX3	1.1909468	0.252109	0.0044308	4074	4202	4979	4951	5751	5084
GLRX5	0.9501183	-0.073821	0.9141201	3954	5287	5133	4556	4802	4299
GLRX2	0.8540773	-0.227562	0.4221927	203	219	277	193	224	180
RCN3	1.3363229	0.4182686	0.1702883	76	86	61	98	129	71
RCN1	0.9818727	-0.026392	0.7761861	7753	7405	8839	7177	8667	7718
PSRC1	0.9491035	-0.075363	0.9317601	1040	1139	1279	1112	1159	1011
RCN2	0.9938617	-0.008883	0.6522748	2376	2106	2849	2161	2612	2513
TAF1L	3.75	1.9068906	0.1739167	2	1	1	5	6	4
TAF1D	1.0218268	0.0311507	0.5311875	3113	1964	3353	2642	3186	2786
TAF1B	1.156365	0.2095968	0.071558	1955	1330	2151	1751	2405	2130
TNKS2	1.3964071	0.4817196	0.0013019	4286	3301	4214	3699	6103	6677
TAF1C	0.9403272	-0.088765	0.8875489	1345	1194	1617	1322	1355	1231
TAF1A	0.9016083	-0.149427	0.6208403	393	256	408	233	385	335
TAF15	0.8272432	-0.273617	0.0512532	7734	8527	9874	6739	7620	7261
TAF12	1.3207612	0.4013697	0.0005651	858	959	1073	1221	1396	1200

TAF13	1.0891441	0.1231949	0.1701802	1179	1135	1343	1088	1480	1415
TAF10	0.9442724	-0.082725	0.9675037	1243	1559	1720	1660	1516	1094
TAF11	1.1749522	0.2326021	0.056134	627	699	766	875	794	789
RAPH1	1.1644325	0.219627	0.3033344	376	376	367	239	468	596
AC093827.3	11	3.4594316	0.138227	1	0	0	2	9	0
KHD1-EIF4EI	0.6	-0.736966	0.1818418	46	26	48	13	20	39
ALG9-IT1	0	#NUM!	0.824326	0	0	1	0	0	0
AC007613.1	1.0526316	0.0740006	0.9552603	10	4	5	2	13	5
A1BG	0	#NUM!	0.5337954	0	3	0	0	0	0
AC131206.1	0.7285319	-0.456936	0.0670563	265	188	269	138	225	163
TMEM192	0.8267631	-0.274454	0.1246366	828	896	1041	585	833	868
A1CF	1	0	0.9737288	3	3	0	4	0	2
TMEM198	1.96	0.9708537	0.0433061	15	13	22	38	30	30
TMEM199	0.9402697	-0.088853	0.8782277	143	185	191	149	182	157
POLR2KP1	1.3333333	0.4150375	0.7401334	2	0	4	3	2	3
TMEM181	0.9100461	-0.135988	0.5812383	1839	1466	2120	1163	1801	1973
TMEM182	0.9936709	-0.00916	0.8333516	297	325	326	264	330	348
AC087393.1	1.5	0.5849625	0.8536377	0	1	1	0	3	0
TMEM186	0.8293578	-0.269933	0.249133	331	389	370	302	335	267
TMEM187	0.6650485	-0.588468	0.0625085	127	115	170	95	76	103
TMEM189	1.1086028	0.1487426	0.1084237	1450	1606	1861	1825	1962	1664
TMSB4X	1.056989	0.0799603	0.2515146	5952	6552	7921	7179	8031	6379
TMSB4Y	1.3973941	0.482739	0.0001207	573	563	706	829	949	796
GPRASP1	0.5542169	-0.851477	0.1686727	30	16	37	12	18	16
LDLRAP1	0.8840494	-0.177801	0.4374755	487	595	617	469	537	496
GPRASP2	0.8780488	-0.187627	0.7465026	34	26	22	14	28	30
TACR2	0.8636364	-0.211504	0.8910091	4	10	8	9	7	3
TACR3	1.3902439	0.475338	0.3144357	39	6	37	37	35	42
TMEM171	2	1	0.5508767	5	0	0	6	2	2
TACR1	0.9758454	-0.035275	0.9826979	56	85	66	56	63	83
TMEM173	0.8888889	-0.169925	0.9774972	1	3	5	4	2	2
AP005019.1	#DIV/0!	#DIV/0!	0.4737294	0	0	0	0	4	0
TMEM174	1.1428571	0.1926451	0.8903971	3	0	4	0	0	8
TMEM175	1.0053996	0.007769	0.6661638	308	257	361	360	282	289
TMEM177	0.9682422	-0.04656	0.9212505	414	454	486	461	451	399
TMEM179	0.7804878	-0.357552	0.5533471	19	35	28	19	25	20
AC060814.4	#DIV/0!	#DIV/0!	0.4737294	0	0	0	0	4	0
AL139174.1	#DIV/0!	#DIV/0!	0.6554062	0	0	0	0	2	0
AC011473.4	0	#NUM!	0.3473206	0	5	0	0	0	0
TMEM160	0.9518797	-0.071149	0.9984389	365	505	460	517	413	336
TMEM163	1.9333333	0.9510904	0.3562098	7	5	3	2	10	17
TMEM164	0.8630595	-0.212468	0.2780108	839	1105	1050	696	940	948
TMEM165	0.8520672	-0.230961	0.1569783	1400	1499	1745	1214	1462	1281
TMEM168	0.8618517	-0.214488	0.2878897	747	585	731	473	651	654
TMEM169	1.5555556	0.6374299	0.6136841	5	3	1	8	0	6
ENPP7P7	0.3333333	-1.584963	0.6337384	1	2	0	1	0	0
ENPP7P8	0.5454545	-0.874469	0.5732641	3	7	1	0	1	5
ENPP7P9	#DIV/0!	#DIV/0!	0.4501902	0	0	0	0	0	4
ENPP7P2	2	1	0.7049414	0	0	2	0	0	4
ZDHHC8P1	0.6739659	-0.569252	0.0513265	116	131	164	75	93	109
ENPP7P4	0.7777778	-0.36257	0.882427	1	1	7	2	4	1
ENPP7P5	#DIV/0!	#DIV/0!	0.6099294	0	0	0	2	0	0
TMEM154	1.3975232	0.4828723	0.0065412	601	381	633	518	839	900
TMEM155	6	2.5849625	0.3114557	0	0	1	4	0	2

TMEM158	3.4444444	1.7842713	0.0141468	7	2	9	14	22	26
TMEM159	0.8578089	-0.221272	0.5462482	131	151	147	127	160	81
LINC00265	0.6338028	-0.657894	0.0144325	203	238	269	105	136	209
AC068987.3	0.5454545	-0.874469	0.0246825	109	65	90	30	45	69
AC068987.5	0.5148515	-0.957772	0.1110694	24	25	52	14	14	24
AC073488.11	2	1	0.8118689	0	0	1	0	1	1
AC068987.1	0.5798319	-0.786293	0.1151464	39	41	39	24	20	25
TMSB10	1.087543	0.1210724	0.195375	26233	33205	37383	38522	36574	30201
BPIFB9P	0	#NUM!	0.824326	0	1	0	0	0	0
MAD1L1	0.8378611	-0.255217	0.2671723	596	761	962	650	687	606
AC025181.1	#DIV/0!	#DIV/0!	0.6554062	0	0	0	0	2	0
LINC00284	#DIV/0!	#DIV/0!	0.6554062	0	0	0	0	2	0
AC025181.2	0.672956	-0.571416	0.2323293	39	52	68	42	35	30
CALCRL	2.1304348	1.0911479	0.0315914	10	16	20	25	34	39
SSBP3-AS1	0.8852459	-0.17585	0.7542644	51	60	72	34	43	85
ILDR1	0.1818182	-2.459432	0.1030477	15	1	6	1	0	3
ILDR2	1.5025641	0.5874265	0.0367789	51	52	92	86	88	119
AP000892.3	0.5	-1	0.8638172	0	0	2	1	0	0
AC010886.1	0	#NUM!	0.824326	1	0	0	0	0	0
LINC00278	0.8363636	-0.257798	0.7188951	19	15	21	7	23	16
LINC00276	0.5	-1	0.7533949	0	3	1	2	0	0
LINC00271	1.8	0.8479969	0.5828367	3	0	2	3	4	2
MIR1205	#DIV/0!	#DIV/0!	0.8030358	0	0	0	1	0	0
MIR1206	0	#NUM!	0.5781912	0	0	3	0	0	0
AP000902.1	0	#NUM!	0.6385096	0	2	0	0	0	0
TADA3	0.8985737	-0.154291	0.6243164	751	799	974	830	822	616
TADA1	0.9080717	-0.139122	0.583895	585	509	690	438	581	601
AP000808.1	#DIV/0!	#DIV/0!	0.0187335	0	0	0	1	7	7
AC055822.1	0.8823529	-0.180572	0.9855225	2	6	26	13	12	5
AP000808.2	0.1875	-2.415037	0.1635856	1	10	5	0	0	3
LINC00221	1.1139053	0.1556266	0.309219	240	189	247	199	294	260
LINC00216	1.1538462	0.2064509	0.8627932	4	4	5	0	2	13
MIR1276	0	#NUM!	0.824326	0	0	1	0	0	0
HIF1AN	1.0164896	0.0235954	0.5907329	2382	2877	2928	2279	2944	3099
LINC00211	#DIV/0!	#DIV/0!	0.8030358	0	0	0	0	0	1
MIR1270	1.125	0.169925	0.9357213	7	0	1	0	2	7
LINC00205	1.8372881	0.8775779	0.0001373	95	82	118	182	208	152
NMD3P1	0.5	-1	0.6580309	0	4	2	0	3	0
AC092159.2	0.25	-2	0.3893309	3	0	9	3	0	0
PKHD1L1	1.3333333	0.4150375	0.8272938	1	5	0	2	4	2
AC092159.4	0	#NUM!	0.5075476	0	0	4	0	0	0
LINC00200	1.0113208	0.0162406	0.721242	989	1127	1064	827	1147	1242
MIR1262	4	2	0.5412414	0	0	1	0	3	1
LINC00240	1.1061947	0.1456053	0.6314321	52	23	38	49	41	35
LINC00242	1.4285714	0.5145732	0.7009309	0	3	4	2	2	6
LINC00243	0.7857143	-0.347923	0.7779736	5	6	3	1	3	7
AL139424.2	1.2	0.2630344	0.8592395	0	0	5	1	5	0
AL139424.3	0.25	-2	0.5554205	4	0	0	0	1	0
LINC00239	0.7419355	-0.430634	0.7080934	6	9	16	8	4	11
LINC00235	0.5705128	-0.809669	0.1145177	40	68	48	43	24	22
CDRT4	0.3870968	-1.369234	0.2701836	6	4	21	3	9	0
MIR1249	0.25	-2	0.5455026	0	4	0	0	0	1
AC011379.1	2	1	0.8186965	0	0	1	0	0	2
AC011379.2	1.3888889	0.4739312	0.5847114	6	0	12	9	12	4

LINC00226	#DIV/0!	#DIV/0!	0.5509701	0	0	0	0	3	0
LINC00222	0.9090909	-0.137504	0.9296984	8	7	7	5	3	12
MMACHC	1.0934449	0.1288805	0.337749	220	246	251	245	261	278
CDRT1	#DIV/0!	#DIV/0!	0.5082165	0	0	0	1	0	2
ERVK13-1	0.8358209	-0.258734	0.42741	172	132	165	107	115	170
VIPR1	1.1943128	0.2561807	0.2943012	74	62	75	89	79	84
C21orf2	1.2362205	0.3059361	0.4120955	35	60	32	50	55	52
RAPGEFL1	1	0	0.918396	31	54	43	34	43	51
TRMT44	1.0519006	0.0729984	0.3875236	450	390	528	447	474	518
MIR1180	1.1	0.1375035	0.8052859	2	0	8	11	0	0
AC008440.3	1	0	0.9988411	1	0	1	0	2	0
AC008440.1	2.4285714	1.2801079	0.314559	3	3	1	9	7	1
DOPEY1	0.9738863	-0.038175	0.9643796	270	155	226	114	268	252
DOPEY2	1.2333795	0.3026168	0.0466919	896	1067	925	877	1268	1417
CARHSP1	1.1458662	0.1964387	0.0627142	2022	2680	2894	2703	3243	2758
AC073195.1	0.4864865	-1.039528	0.3211792	10	3	24	2	11	5
WTH3DI	2.8333333	1.5025003	0.2722253	0	5	1	12	2	3
TRMT2A	0.9847932	-0.022107	0.7625922	941	1109	1238	1127	1122	989
TRMT2B	0.7018167	-0.510834	0.0017639	723	876	933	511	672	594
AC007029.1	0.1111111	-3.169925	0.2243571	0	9	0	0	0	1
HIGD1AP16	0	#NUM!	0.824326	1	0	0	0	0	0
AP001000.1	1.0884956	0.1223355	0.7121491	27	34	52	21	57	45
VPS8	0.8305947	-0.267783	0.2068659	903	652	1001	561	723	839
ITPR1-AS1	1.0526316	0.0740006	0.7481849	15	20	41	28	31	21
TRMT1L	1.0845565	0.1171052	0.3152618	1146	830	1158	830	1288	1281
DDIT4L	0.8414211	-0.2491	0.2582426	330	369	455	269	367	335
IFNLR1	0.9699571	-0.044007	0.9170669	68	80	85	91	59	76
TRMT12	0.8338591	-0.262124	0.255548	292	310	349	253	275	265
TRMT13	1.0631939	0.0884047	0.4959467	454	300	417	310	455	480
TRMT11	1.1629486	0.2177873	0.1276881	378	293	360	338	426	435
LINC00184	0	#NUM!	0.824326	0	0	1	0	0	0
URB1-AS1	0.8474026	-0.238881	0.5405673	129	90	89	89	110	62
LINC00174	0.8409475	-0.249912	0.4621245	200	129	262	144	180	173
AL158070.1	#DIV/0!	#DIV/0!	0.8030358	0	0	0	1	0	0
RDH5	0.4074074	-1.295456	0.2546349	4	12	11	0	7	4
LINC00167	0	#NUM!	0.1138391	3	4	1	0	0	0
RAPSN	0	#NUM!	0.6385096	0	2	0	0	0	0
LILRA6	0	#NUM!	0.06794	3	3	4	0	0	0
AC104446.1	0.6842105	-0.547488	0.7590091	8	0	11	6	7	0
PPP1R2P3	1.75	0.8073549	0.6029084	1	1	2	5	0	2
LILRA2	0	#NUM!	0.1172014	0	7	2	0	0	0
LILRA4	0	#NUM!	0.4005425	3	1	0	0	0	0
LILRB1	0	#NUM!	0.5436524	2	0	1	0	0	0
LILRB3	0.68	-0.556393	0.4672097	16	16	18	12	16	6
AL355472.1	1.4827586	0.5682838	0.3913111	5	9	15	10	16	17
AL355472.2	#DIV/0!	#DIV/0!	0.8030358	0	0	0	0	1	0
SPANXA2-OT	#DIV/0!	#DIV/0!	0.8030358	0	0	0	1	0	0
AMY2B	0.21875	-2.192645	0.0670994	8	4	20	1	5	1
AC090587.1	0	#NUM!	0.5387425	3	0	0	0	0	0
AC090587.2	#DIV/0!	#DIV/0!	0.8030358	0	0	0	1	0	0
TMEM140	0.6033058	-0.729039	0.0062566	251	243	232	83	167	188
TMEM141	0.9566396	-0.063953	0.9522886	110	123	136	151	108	94
TMEM143	0.8767705	-0.189729	0.6059752	194	242	270	232	188	199
RDM1	0.9983526	-0.002379	0.8000793	142	220	245	194	242	170

TMEM144	0.75	-0.415037	0.2735508	231	85	244	114	137	169
TMEM145	#DIV/0!	#DIV/0!	0.8030358	0	0	0	1	0	0
TMEM147	0.9254844	-0.111719	0.7688268	764	847	1073	817	965	702
TMEM14A	0.9928775	-0.010312	0.805746	384	495	525	400	538	456
TMEM14B	1.0090209	0.0129561	0.5490553	1387	1400	1758	1521	1674	1391
TMEM14C	0.8530285	-0.229334	0.1968695	2800	3462	3842	2788	3040	2791
AMY1B	#DIV/0!	#DIV/0!	0.5070017	0	0	0	3	0	0
TMEM130	1.45	0.5360529	0.326895	15	10	15	24	16	18
TMEM131	0.9642214	-0.052564	0.9429096	2703	2265	2802	1812	2760	2920
TMEM133	0.9251969	-0.112168	0.8482402	95	56	103	55	101	79
TMEM134	0.7577808	-0.400148	0.149813	197	263	279	208	168	184
TMEM135	0.8534212	-0.22867	0.1974824	1103	969	1114	698	1027	994
TMEM136	0.6758893	-0.565141	0.1435336	67	88	98	67	57	47
TMEM138	0.8821717	-0.180869	0.3582629	829	845	923	707	876	708
TMEM139	1.2152589	0.2812636	0.2509541	117	151	99	129	171	146
AL161909.1	0.4761905	-1.070389	0.3546744	14	5	2	2	4	4
DBF4P1	2	1	0.6442044	0	1	1	2	1	1
AC005551.1	0	#NUM!	0.824326	0	0	1	0	0	0
TMEM121	1.5384615	0.6214884	0.5378276	1	3	9	3	10	7
TMEM123	1.0885722	0.1224371	0.2824076	5332	3634	5350	3784	5713	6087
AC093330.1	0	#NUM!	0.824326	1	0	0	0	0	0
TMEM125	1.3693467	0.4534878	0.0070987	209	259	328	350	421	319
AC093330.2	0	#NUM!	0.6436235	2	0	0	0	0	0
PODXL	0.1578947	-2.662965	2.14E-05	49	46	38	12	6	3
TMEM127	0.9390347	-0.09075	0.7564469	2115	2403	2568	1987	2355	2312
TMEM128	0.8368892	-0.256891	0.2271327	455	463	535	407	423	386
TMEM129	1.0432034	0.0610204	0.4155108	547	626	725	630	736	614
TMEM110	0.7586207	-0.398549	0.7005267	10	6	13	0	17	5
TMEM115	0.8303571	-0.268196	0.2435626	746	859	971	782	775	582
TMEM116	0.9424626	-0.085493	0.8792895	235	312	322	252	292	275
TMEM117	0.7222222	-0.469485	0.7896546	7	2	9	8	1	4
OC1B-GALNT	0	#NUM!	0.824326	0	0	1	0	0	0
FRMD6-AS2	1	0	0.9890559	0	0	1	0	0	1
FRMD6-AS1	0.5652174	-0.823122	0.151245	30	24	38	10	26	16
ELAVL1	0.9551885	-0.066143	0.9675672	3309	3512	4136	3334	3726	3406
ELAVL2	0.9534884	-0.068713	0.9015994	55	39	35	22	48	53
TMEM100	1.5	0.5849625	0.8233996	1	0	1	0	1	2
TMEM101	1.0465439	0.0656329	0.461434	865	1067	947	1063	1042	908
TMEM102	0.9361702	-0.095157	0.7983026	270	267	215	224	254	226
MIR512-1	0.3333333	-1.584963	0.7307796	0	0	3	0	1	0
TMEM104	0.7606557	-0.394684	0.0604907	349	419	452	298	356	274
TMEM105	0.6086957	-0.716207	0.461875	13	9	24	16	7	5
TMEM107	0.7300178	-0.453997	0.0865455	169	167	227	137	144	130
TMEM108	1.1060606	0.1454304	0.5905999	37	80	81	53	86	80
TMEM109	1.0686689	0.0958149	0.2336491	1858	2208	2327	2197	2466	2169
AL391056.1	1.3	0.3785116	0.6873666	10	2	8	2	11	13
POLDIP2	0.874581	-0.193336	0.2785718	2955	3536	3651	2828	3237	2805
POLDIP3	0.8771794	-0.189056	0.2546349	4175	4897	5152	3818	4412	4247
SLC22A18AS	0.2222222	-2.169925	0.3790136	1	0	8	1	0	1
SBF1P1	0	#NUM!	0.824326	0	1	0	0	0	0
AC048351.1	#DIV/0!	#DIV/0!	0.6411933	0	0	0	0	0	2
PRMT5-AS1	1.1818182	0.2410081	0.8408649	0	3	8	0	7	6
AC011726.3	#DIV/0!	#DIV/0!	0.8030358	0	0	0	0	1	0
TESK1	0.9895068	-0.015218	0.7904638	317	262	374	281	333	329

TESK2	1.2337938	0.3031013	0.0645328	293	347	301	326	447	388
C3AR1	0	#NUM!	0.824326	0	0	1	0	0	0
EBLN3P	1.2411915	0.3117258	0.0330293	1162	803	1157	944	1401	1530
CAPN10-AS1	0.8686441	-0.203163	0.5997293	165	134	173	156	140	114
AC131097.3	0.6	-0.736966	0.7905399	0	1	4	0	3	0
AC131097.4	7	2.8073549	0.2256169	0	0	1	4	1	2
AC131097.1	0	#NUM!	0.3320541	3	0	2	0	0	0
AC131097.2	#DIV/0!	#DIV/0!	0.249172	0	0	0	0	1	5
ISM1-AS1	#DIV/0!	#DIV/0!	0.6411933	0	0	0	0	0	2
AC011330.1	0.7826087	-0.353637	0.7509097	3	11	9	1	6	11
AC011330.2	1.4090909	0.4947647	0.5137212	6	1	15	11	14	6
AF127577.6	#DIV/0!	#DIV/0!	0.6554062	0	0	0	0	2	0
AF127577.2	#DIV/0!	#DIV/0!	0.288876	0	0	0	3	2	0
AF127577.3	2.5	1.3219281	0.5527374	0	0	2	3	2	0
AF127577.4	0.7073171	-0.499571	0.5714288	22	4	15	5	12	12
AF127577.5	#DIV/0!	#DIV/0!	0.0742837	0	0	0	7	3	0
TPRG1L	1.364117	0.4479674	8.15E-05	892	861	1084	1249	1294	1327
GAS2L1P2	1.3483146	0.4311572	0.2808881	38	25	26	42	35	43
CIAO1	0.9397972	-0.089579	0.820727	3241	3748	4057	3438	3727	3216
UBR5-AS1	0.9497487	-0.074382	0.9627523	58	52	89	44	78	67
GAS2L1P1	0.85	-0.234465	0.8708125	9	5	6	8	6	3
NDUFA3P1	0	#NUM!	0.6385096	0	2	0	0	0	0
NDUFA3P4	#DIV/0!	#DIV/0!	0.8030358	0	0	0	1	0	0
AC000120.1	0.4444444	-1.169925	0.4549213	3	3	12	3	0	5
AL139421.1	4	2	0.5362768	1	0	0	0	3	1
CR381653.1	0.8611111	-0.215729	0.8659806	8	11	17	12	14	5
HM13-IT1	0.5466667	-0.871267	0.1538597	30	22	23	12	17	12
CR381653.2	0.3661972	-1.449307	0.0433927	27	15	29	5	16	5
TCP11L1	0.8241758	-0.278976	0.2188776	283	321	306	224	264	262
TCP11L2	1.0964706	0.1328671	0.4913765	139	134	152	104	190	172
AC025578.1	#DIV/0!	#DIV/0!	0.8030358	0	0	0	0	0	1
AC005609.5	0.875	-0.192645	0.979301	2	0	6	1	0	6
AC007996.1	0.693913	-0.527173	0.0406938	177	204	194	113	128	158
CDC20B	0.5897436	-0.76184	0.3946679	9	23	7	8	13	2
AC005609.1	0.6666667	-0.584963	0.8793857	1	1	1	2	0	0
SAMD5	1.1388889	0.187627	0.7640645	17	8	11	9	18	14
SAMD8	1.1017203	0.139758	0.2941199	1456	1098	1457	986	1686	1747
SAMD9	2.7184211	1.4427689	1.02E-08	157	69	154	258	430	345
SAMD1	0.9688557	-0.045646	0.8913024	1619	1923	2077	1914	1926	1604
SAMD3	0.25	-2	0.4449435	3	0	5	2	0	0
AC093726.1	0.6428571	-0.63743	0.335482	25	22	23	19	12	14
AC093726.2	15	3.9068906	0.0657822	1	0	0	0	8	7
TMEM270	0	#NUM!	0.824326	1	0	0	0	0	0
TMEM272	0.3220339	-1.634716	0.0329525	17	18	24	8	5	6
AP005018.2	1	0	0.9890559	1	0	0	1	0	0
AL583832.1	0.9411765	-0.087463	0.9879949	2	4	11	0	5	11
RN7SL168P	0	#NUM!	0.824326	0	1	0	0	0	0
TRAV8-7	0	#NUM!	0.1560297	3	2	2	0	0	0
AL358852.1	0.9	-0.152003	0.9286712	4	4	2	1	0	8
CEP250	0.975442	-0.035872	0.8960548	2182	2369	2575	1980	2324	2647
CYP2T1P	0.9174312	-0.124328	0.9739435	38	35	36	61	33	6
CDC25C	0.9532832	-0.069023	0.9109806	937	1007	1117	860	1014	1044
CDC25A	0.734512	-0.445142	0.0009059	2126	2308	2523	1476	1927	1707
CDC25B	0.9024015	-0.148159	0.5152665	1803	2283	2410	1893	2140	1829

LPAL2	0.4210526	-1.247928	0.3128602	14	6	18	14	1	1
AP001381.1	0	#NUM!	0.824326	1	0	0	0	0	0
AL713922.2	0.0625	-4	0.0609702	9	0	7	0	0	1
AC105429.1	0.625	-0.678072	0.4216642	28	4	32	14	16	10
RN7SL165P	0	#NUM!	0.824326	0	1	0	0	0	0
CYP2T3P	#DIV/0!	#DIV/0!	0.4242335	0	0	0	0	3	1
AC008105.1	1.375	0.4594316	0.7895081	6	0	2	0	5	6
RNU7-54P	0	#NUM!	0.3451105	2	0	3	0	0	0
AC008105.2	9	3.169925	0.1426005	0	1	0	7	1	1
AC008105.3	0	#NUM!	0.824326	0	0	1	0	0	0
AC007610.2	0.7333333	-0.447459	0.7431599	6	7	2	6	4	1
AC007610.4	#DIV/0!	#DIV/0!	0.6554062	0	0	0	0	2	0
AC004134.1	1.5	0.5849625	0.8395779	0	0	2	0	3	0
AC011476.5	0.5	-1	0.8638172	0	0	2	1	0	0
AC011476.1	3.5	1.8073549	0.3839137	0	0	2	6	1	0
AC011476.2	0.8589744	-0.219313	0.7620484	29	24	25	23	17	27
AC011476.3	0.7582418	-0.39927	0.3375852	57	58	67	25	58	55
AC092354.2	0.6666667	-0.584963	0.6557418	1	9	5	2	6	2
AC012065.2	1	0	0.9890559	1	0	0	0	1	0
AC012065.5	1.5333333	0.6166714	0.2439675	10	12	23	34	21	14
TAGAP	0.3157895	-1.662965	0.2463496	7	12	0	0	4	2
CEP290	1.3063492	0.3855406	0.0499028	237	161	232	274	336	213
CEP295	0.8963367	-0.157887	0.6444572	496	268	519	224	451	475
AL139327.2	0.7317073	-0.450661	0.6138915	12	15	14	14	9	7
AC010501.1	0.6792453	-0.557995	0.4552751	23	16	14	2	26	8
AP000553.4	0.4576271	-1.127756	0.1201739	13	26	20	7	14	6
AP000553.5	0.8181818	-0.289507	0.9083991	2	3	6	5	3	1
AP000553.6	1.3333333	0.4150375	0.5569611	9	2	16	8	17	11
PBXIP1	1.0315705	0.0448424	0.4394554	1788	2194	2258	1996	2318	2123
AP000553.1	1	0	0.9099396	6	9	14	11	10	8
AP000553.2	0.5833333	-0.777608	0.6437049	0	6	6	0	6	1
AP000553.3	0.5	-1	0.6866009	0	2	4	0	3	0
KCTD20	0.8668298	-0.206179	0.2358625	3811	3233	3987	2387	3489	3686
KCTD21	1.066335	0.0926607	0.4796198	215	164	224	181	241	221
MARCKSL1	1.2103966	0.2754798	0.0110201	1249	1494	1720	1769	1902	1731
REC8	2.5280899	1.3380478	0.0011994	41	12	36	89	61	75
RNU7-59P	#DIV/0!	#DIV/0!	0.8030358	0	0	0	0	1	0
AC010491.1	0.4166667	-1.263034	0.0847202	30	8	58	9	22	9
RECK	0.9977778	-0.00321	0.8112769	145	144	161	147	163	139
RN7SL160P	1	0	0.9890559	0	0	1	0	0	1
DMGDH	0.4782609	-1.06413	0.3741189	3	9	11	2	2	7
MCCC1-AS1	0.6785714	-0.559427	0.5982247	22	8	26	25	5	8
KCTD19	1.1338583	0.1812403	0.5617502	32	42	53	32	52	60
AC092989.1	0.4705882	-1.087463	0.4178246	6	4	7	4	2	2
KCTD10	0.9940516	-0.008607	0.7146829	2296	2531	2738	2216	2808	2496
KCTD13	0.7752212	-0.36732	0.2419754	191	155	219	172	142	124
KCTD14	0	#NUM!	0.824326	0	1	0	0	0	0
KCTD11	0.9344828	-0.09776	0.820549	277	298	295	272	300	241
KCTD12	0.8525684	-0.230113	0.1700673	2567	2303	2625	1616	2320	2454
MGARP	0.2	-2.321928	0.4391014	0	3	2	0	0	1
KCTD17	0.6781609	-0.5603	0.3572085	35	29	23	23	13	23
KCTD18	0.9826772	-0.025211	0.8490046	446	365	459	375	425	448
KCTD15	1	0	0.8010561	135	133	150	133	126	159
KCTD16	0.9444444	-0.082462	0.9504038	8	2	8	11	1	5

AC006042.1	0.2307692	-2.115477	0.3217746	7	0	6	3	0	0
LPAR2	0.9808061	-0.02796	0.8959933	145	191	185	197	204	110
AC006042.2	0	#NUM!	0.824326	1	0	0	0	0	0
LPAR3	1.2592593	0.3325753	0.5805096	6	7	14	17	4	13
AC006042.3	0.6666667	-0.584963	0.7397059	8	0	1	1	1	4
MBTD1	1.0992934	0.1365765	0.3203817	1084	653	952	744	1086	1126
PP12613	0.3333333	-1.584963	0.4940354	3	3	0	0	2	0
CEP192	0.857631	-0.221571	0.4105876	653	446	657	282	564	660
STAT5A	4	2	0.5101708	0	0	1	1	0	3
STAT5B	0.8215339	-0.283608	0.116922	628	710	696	469	645	557
REG4	0.5	-1	0.81106	0	2	0	0	0	1
AL158071.1	1	0	0.9940505	1	1	1	0	2	1
NTAN1	0.8421053	-0.247928	0.3800219	238	226	353	220	246	222
AL158071.2	1.0668258	0.0933246	0.508961	130	123	166	152	172	123
AL158071.3	0.75	-0.415037	0.9354852	0	0	4	0	0	3
AL158071.4	1.0701754	0.0978473	0.8640168	29	16	12	12	22	27
AIMP1	1.1463808	0.1970864	0.1004169	1560	1046	1732	1625	1894	1454
AIMP2	0.8042585	-0.314269	0.0689373	2576	3080	3596	2509	2678	2254
AC006042.4	#DIV/0!	#DIV/0!	0.6411933	0	0	0	0	0	2
LPAR5	0.8333333	-0.263034	0.731356	14	13	9	2	20	8
LPAR6	1.2941176	0.3719688	0.6802679	6	1	10	3	11	8
KYAT3	1.1306002	0.1770888	0.0939311	824	661	881	836	972	867
KYAT1	1.097076	0.1336635	0.2772481	254	236	365	338	305	295
IL20	#DIV/0!	#DIV/0!	0.6554062	0	0	0	0	2	0
IL25	#DIV/0!	#DIV/0!	0.8030358	0	0	0	1	0	0
AC005702.1	0.3333333	-1.584963	0.6574028	0	2	1	0	0	1
IL27	0.53125	-0.912537	0.1447847	27	27	42	8	12	31
AC005702.3	8	3	0.2345573	1	0	0	0	3	5
SPTAN1	0.8178403	-0.290109	0.0509136	6342	7171	7249	4564	6042	6374
OXGR1	0.6554622	-0.609416	0.2628899	51	20	48	15	35	28
IL11	0.4545455	-1.137504	0.3779545	14	4	4	7	2	1
IL15	0.8255814	-0.276518	0.7364154	17	21	48	21	36	14
PABPC4	1.0714661	0.0995862	0.1363626	7986	8647	9995	8707	10194	9630
PABPC5	#DIV/0!	#DIV/0!	0.4737294	0	0	0	0	4	0
IL18	0.808933	-0.305908	0.2101927	250	244	312	215	222	215
IL16	1.6666667	0.7369656	0.3629034	2	4	12	10	12	8
IL1A	1.5555556	0.6374299	0.6336748	8	0	1	12	0	2
AC090686.1	1	0	0.987967	2	1	0	0	0	3
FAM45BP	1.7323944	0.7927674	0.0671953	21	21	29	33	56	34
PABPC3	1.5	0.5849625	0.6758619	5	3	2	1	11	3
PABPC1	1.0740097	0.103007	0.1150795	25821	23644	29876	25029	31217	28967
TAGLN	0.8663793	-0.206929	0.7105528	64	63	105	65	56	80
RELA	0.8474324	-0.23883	0.1651215	1168	1389	1474	1047	1216	1153
RELB	0.5646552	-0.824558	0.0279336	66	65	101	35	47	49
REM1	0	#NUM!	0.824326	1	0	0	0	0	0
RELN	2	1	0.834705	0	0	1	0	2	0
AL121985.1	0.375	-1.415037	0.5347127	4	0	4	2	1	0
MGAT5	0.6080017	-0.717853	9.34E-07	2905	3121	3122	1393	2097	2072
MGAT3	1.1357884	0.1836941	0.0679099	1791	2134	2239	1944	2600	2457
MGAT1	0.8584191	-0.220246	0.2403844	3705	4713	4790	3794	3972	3572
MGAT2	2	1	0.639194	2	2	0	0	5	3
RELT	0.8774617	-0.188592	0.6765076	120	144	193	150	130	121
REM2	1.2777778	0.353637	0.6895751	5	9	4	7	4	12
IL32	1.1235955	0.1681228	0.5060217	45	40	93	56	88	56

IL34	1.75	0.8073549	0.6717089	3	1	0	3	0	4
VEGFB	1.0424322	0.0599535	0.4243149	681	758	847	817	871	695
VEGFC	0.8	-0.321928	0.8311924	8	0	2	0	5	3
VEGFD	0	#NUM!	0.4217812	0	0	5	0	0	0
AL442663.2	#DIV/0!	#DIV/0!	0.6411933	0	0	0	0	0	2
AL442663.3	0.1578947	-2.662965	0.0838158	2	13	4	0	1	2
AC022210.1	0	#NUM!	0.824326	0	0	1	0	0	0
AL442663.4	1.0882353	0.1219905	0.8411896	13	6	15	7	23	7
VEGFA	1.1676393	0.2235947	0.0086901	3985	3787	4737	4300	5113	5193
ar response to v	#DIV/0!	#DIV/0!							
in sprouting ar	#VALUE!	#VALUE!	ıembrane;G03	hsa05163	ıtions	Humathelial growth factor A			
AL442663.1	0	#NUM!	0.824326	0	0	1	0	0	0
TMEM260	0.9104367	-0.135369	0.6161303	502	368	481	283	480	467
TMEM262	4.3333333	2.1154772	0.2244976	0	0	3	1	8	4
TMEM263	1.0967103	0.1331824	0.2422341	883	679	961	699	1088	980
TMEM266	0.893617	-0.162271	0.826561	36	23	35	23	26	35
TMEM267	0.9293564	-0.105696	0.9096069	215	137	285	190	219	183
TMEM268	0.6072235	-0.719701	0.0240396	144	134	165	113	84	72
TMEM269	0.3191489	-1.647698	0.093814	7	27	13	0	4	11
OR13Z2P	2	1	0.7736272	0	1	0	2	0	0
IL6R	1.0661765	0.0924462	0.7388803	47	43	46	38	66	41
IL4R	0.7698976	-0.377262	0.0821247	358	363	548	275	389	313
TMEM250	1.0606876	0.0849998	0.2724899	1858	2309	2523	2394	2389	2313
TMEM251	1.1025641	0.1408625	0.4917534	83	86	104	106	123	72
TMEM253	0.2142857	-2.222392	0.1703072	4	5	5	0	2	1
TMEM254	1.6638452	0.7345212	2.52E-05	214	303	310	464	472	440
TMEM256	0.9259259	-0.111031	0.9473743	9	28	17	20	10	20
TMEM258	1.0061491	0.0088441	0.6700378	360	422	519	485	482	342
AF228730.6	#DIV/0!	#DIV/0!	0.3096126	0	0	0	5	0	0
TMEM259	0.9168451	-0.12525	0.6200001	1618	1853	2133	1651	1813	1674
AF228730.7	1	0	0.9904495	3	0	0	0	0	3
OR13Z3P	0	#NUM!	0.6840298	0	0	2	0	0	0
AL356317.1	0	#NUM!	0.5337954	0	3	0	0	0	0
C8orf44	0.8184358	-0.289059	0.5175308	138	82	138	124	92	77
TMEM240	1.5	0.5849625	0.761078	1	0	3	0	4	2
TMEM241	0.9894578	-0.01529	0.8497386	409	453	466	364	494	456
TMEM242	0.9180328	-0.123382	0.7588877	499	477	610	522	491	443
C8orf48	1.0769231	0.1069152	0.7692716	11	12	16	26	1	15
TMEM243	0.9474627	-0.077859	0.9704534	506	503	666	549	550	488
C8orf49	0	#NUM!	0.2394948	0	0	8	0	0	0
H2BFM	0.9411765	-0.087463	0.9920489	4	4	9	5	4	7
C8orf46	4	2	0.1692129	1	0	4	8	12	0
SNTG2	#DIV/0!	#DIV/0!	0.6411933	0	0	0	0	0	2
TMEM245	0.9590508	-0.060321	0.8744695	5710	4951	5774	3560	5843	6359
TMEM246	#DIV/0!	#DIV/0!	0.8030358	0	0	0	1	0	0
TMEM247	#DIV/0!	#DIV/0!	0.3196487	0	0	0	0	1	4
TMEM247	#DIV/0!	#DIV/0!	0.3196487	0	0	0	0	1	4
TMEM248	0.8456436	-0.241878	0.1167288	2672	2869	3354	2361	2596	2565
TMEM249	1.0833333	0.1154772	0.9164454	1	10	1	6	3	4
AL928711.1	#DIV/0!	#DIV/0!	0.1923616	0	0	0	0	2	5
GPX1P1	1.2608696	0.334419	0.6305427	4	9	10	14	11	4
GPX1P2	0	#NUM!	0.6840298	0	0	2	0	0	0
HAUS6P1	3.2	1.6780719	0.2412301	1	2	2	15	1	0
HAUS6P3	0.8333333	-0.263034	0.8863124	5	0	1	0	2	3

H2BFS	1.4482759	0.5343364	0.4349435	3	15	11	20	9	13
H2BFS	1.4482759	0.5343364	0.4349435	3	15	11	20	9	13
C8orf33	0.7530997	-0.409087	0.0108141	1040	1206	1464	870	970	954
C8orf34	0.4615385	-1.115477	0.4598883	6	4	3	0	1	5
C8orf31	0.8	-0.321928	0.537606	25	57	58	28	45	39
TJAP1	0.892297	-0.164404	0.4536705	825	945	1099	807	900	853
TMEM230	1.0779709	0.1083182	0.1425098	2574	2752	3190	2772	3400	3008
TMEM231	0.7977528	-0.325986	0.5975172	23	42	24	23	17	31
C8orf37	1.0687285	0.0958954	0.6295058	78	103	110	71	115	125
TMEM232	2.6666667	1.4150375	0.097283	7	6	2	18	12	10
TMEM234	1.0801688	0.1112568	0.5903198	58	91	88	75	83	98
TMEM236	0.5302013	-0.915388	0.1078663	64	21	64	8	36	35
TMEM237	1.1647564	0.2200283	0.0758594	486	378	532	497	588	541
TMEM238	1.25	0.3219281	0.7994963	0	1	3	5	0	0
TMEM239	0.875	-0.192645	0.9574174	0	0	8	5	0	2
IL7R	#DIV/0!	#DIV/0!	0.1307332	0	0	0	3	0	5
MXD1	1.4820568	0.5676007	1.81E-07	1217	1099	1418	1600	1996	1938
MXD3	1.3203971	0.4009719	0.0065473	317	347	444	512	507	444
MXD4	1.2077039	0.2722668	0.0791161	348	482	494	610	515	474
RERE	0.8895973	-0.168776	0.3476381	2775	3000	3165	1950	3009	2994
RERG	0.814842	-0.295408	0.1276795	430	424	507	301	431	377
TMEM220	0.88	-0.184425	0.8165219	14	10	1	3	12	7
TMEM221	0.9375	-0.093109	0.9895374	6	3	7	5	5	5
TMEM222	0.9001988	-0.151684	0.565312	779	804	932	787	796	681
AC002467.1	1.2465753	0.3179701	0.4326005	24	17	32	25	30	36
TMEM223	0.9046529	-0.144564	0.8275571	364	417	530	521	358	307
GRM7-AS3	0.1666667	-2.584963	0.3802332	0	2	4	0	0	1
SNTA1	1.0332963	0.0472541	0.5203636	250	280	371	311	296	324
C8orf59	1.1500816	0.2017362	0.2286599	224	186	203	256	237	212
C8orf58	1.0567227	0.0795968	0.5242814	145	139	192	182	187	134
TMEM213	0.5	-1	0.8652929	0	0	2	0	1	0
RER1	1.059689	0.0836409	0.2599916	2462	2740	3158	2989	3229	2641
TMEM214	0.8513537	-0.232169	0.1403502	3334	3907	4209	3002	3594	3152
TMEM216	0.9166667	-0.125531	0.9285673	7	0	5	0	10	1
TMEM217	1.6363636	0.7104934	0.4893171	2	2	7	4	8	6
TMEM218	0.9062937	-0.141949	0.6977439	230	231	254	227	216	205
TMEM219	1.1501532	0.2018261	0.1279845	539	632	787	860	790	602
IL9R	0	#NUM!	0.2762615	2	0	4	0	0	0
C8orf82	0.8222477	-0.282355	0.3692913	237	296	339	276	270	171
RETN	0	#NUM!	0.824326	1	0	0	0	0	0
C8orf88	0.9444444	-0.082462	0.9529118	6	6	6	3	8	6
C8orf86	#DIV/0!	#DIV/0!	0.8030358	0	0	0	0	0	1
TMEM201	0.8456067	-0.241941	0.2302574	651	914	825	567	755	699
PABPN1	1.0627433	0.0877931	0.2226827	1904	2012	2507	2326	2332	2168
TMEM203	1.0555834	0.0780405	0.2950032	1187	1313	1494	1454	1384	1378
TMEM204	0.7777778	-0.36257	0.848391	0	4	5	1	4	2
MKNK1	1.0023116	0.0033311	0.6789587	599	749	815	685	793	690
TMEM205	0.6478873	-0.626185	0.2336601	43	44	55	35	17	40
MKNK2	1.0896568	0.1238738	0.1666493	2497	2994	3075	3084	3344	2906
MXI1	1.2490775	0.320863	0.0694419	181	142	219	188	254	235
TMEM206	0.9146707	-0.128676	0.7329296	223	185	260	184	231	196
TMEM207	0.75	-0.415037	0.90322	2	2	0	3	0	0
TMEM208	0.8769543	-0.189427	0.507508	1098	1121	1235	1177	1084	768
TMEM209	0.8517647	-0.231473	0.2096169	1207	938	1255	782	1051	1063

UNGP3	2	1	0.7300836	1	0	1	0	4	0
ZNF300P1	0.375	-1.415037	0.5734621	5	0	3	3	0	0
C8orf76	1.4117647	0.4974997	0.4036462	11	8	15	27	6	15
REST	1.1742764	0.231772	0.1634636	1869	1167	1870	1189	2299	2273
SNTB2	0.7268079	-0.460354	0.0097713	917	796	1025	466	769	755
AC242376.2	0.38125	-1.391191	0.0049291	65	41	54	14	15	32
EMC10	1.0129366	0.0185439	0.6624192	372	545	629	464	606	496
EMBP1	1.5	0.5849625	0.8194094	0	0	2	0	0	3
NYNRIN	0.4620253	-1.113956	0.028872	43	73	42	34	22	17
DSCAM-AS1	#DIV/0!	#DIV/0!	0.8030358	0	0	0	0	1	0
REV1	1.0571243	0.080145	0.3787998	1079	793	1209	855	1167	1235
AC005358.1	0.5	-1	0.81753	2	0	0	0	0	1
AL008635.1	0	#NUM!	0.0494419	4	6	1	0	0	0
PLEKHS1	0	#NUM!	0.6436235	2	0	0	0	0	0
AL354983.1	1	0	0.9890559	0	1	0	1	0	0
H3F3BP1	1.5	0.5849625	0.8505665	1	0	1	0	3	0
AL512791.2	1	0	0.9095169	23	20	21	22	15	27
AL512791.1	1.3608247	0.4444813	0.2489494	33	30	34	33	57	42
SPOUT1	1.0294301	0.0418458	0.4061323	1351	1537	1937	1671	1692	1604
TAF7L	0.6666667	-0.584963	0.856923	1	0	2	0	2	0
PLEKHN1	0.9973262	-0.003863	0.8296255	84	126	164	116	163	94
AC074276.1	#DIV/0!	#DIV/0!	0.8030358	0	0	0	0	0	1
TERF1	1.0674747	0.0942019	0.3639525	891	624	960	730	1080	832
TERF2	0.8665016	-0.206726	0.2563182	1267	1562	1613	1149	1343	1357
TAF6L	0.9641148	-0.052723	0.9144988	245	247	344	295	307	204
AEBP2	1.200671	0.2638409	0.0224048	2261	1725	2273	1996	2791	2728
U91328.4	0.75	-0.415037	0.8770867	8	0	0	5	0	1
PLEKHO1	1.0833333	0.1154772	0.8497112	5	10	9	6	6	14
U91328.2	0.9227053	-0.116058	0.8589699	61	79	67	54	45	92
PLEKHO2	0.8877783	-0.171729	0.3884715	1875	2330	2398	1855	2011	1996
CDC123	1.1772996	0.2354815	0.0116664	2007	2122	2470	2357	2918	2494
NDUFA4L2	1.7777778	0.830075	0.5274588	5	0	4	8	8	0
AC004623.1	0.1666667	-2.584963	0.4003623	1	0	5	0	0	1
TAF9B	1.0004103	0.0005919	0.8175827	887	623	927	543	989	906
RUNX1T1	2.5	1.3219281	0.5441496	1	0	1	1	1	3
YTHDF1	0.7856955	-0.347958	0.007007	4592	4965	5627	3481	4433	4016
YTHDF2	1.172539	0.2296359	0.0065955	4097	4047	4798	4470	5508	5197
YTHDF3	0.9775712	-0.032726	0.9459892	1841	1296	1812	1151	1772	1915
MTRNR2L12	1	0	0.9724435	7	6	4	4	1	12
MKLN1	0.8535381	-0.228473	0.3181681	692	473	658	368	587	601
TMEM39A	0.9314542	-0.102443	0.6811798	978	1009	1135	813	1100	995
TMEM39B	0.9178241	-0.12371	0.7442889	235	316	313	259	266	268
CEP126	1.2537313	0.3262282	0.2554494	56	62	83	62	119	71
MTFMT	0.8579828	-0.220979	0.2797756	692	659	860	619	677	601
AL445490.1	3.7906977	1.9224634	4.16E-11	38	31	60	148	144	197
CEP120	1.1226923	0.1669626	0.2482843	995	618	987	690	1162	1067
CDC14C	0.9310345	-0.103093	0.909308	20	17	21	8	18	28
VIRMA	0.9813465	-0.027165	0.9000883	1262	891	1278	834	1232	1301
CDC14A	1.0049875	0.0071776	0.8375992	177	90	134	125	157	121
CDC14B	1.0623222	0.0872215	0.3344148	2710	2386	2991	2109	3258	3224
CEP128	1.1631356	0.2180193	0.1762873	346	228	370	275	416	407
AC240565.1	0.15625	-2.678072	0.0230895	14	9	9	0	2	3
SALL1	#DIV/0!	#DIV/0!	0.8030358	0	0	0	1	0	0
SALL2	1.206422	0.2707347	0.1911401	131	171	134	179	172	175

SALL3	#DIV/0!	#DIV/0!	0.1486377	0	0	0	2	6	0
AC240565.2	0.8333333	-0.263034	0.9630677	0	0	6	1	3	1
SALL4	0.9825591	-0.025384	0.8305678	1863	2167	2277	1806	2173	2218
PLEKHJ1	1.0157593	0.0225586	0.581173	604	695	795	773	750	604
RARS2	0.9365251	-0.09461	0.7889031	1579	1304	1733	1348	1552	1423
CEP135	1.1600454	0.2141813	0.2252422	327	198	356	253	437	332
SNRPN	1	0	0.9750081	0	3	1	0	4	0
CEP131	1.1149134	0.1569316	0.1626598	703	725	939	1006	864	769
VRK1	1.2000956	0.2631494	0.0249079	1480	1068	1635	1442	1936	1642
VRK2	0.930798	-0.10346	0.7959501	581	389	634	377	532	584
VRK3	0.9003861	-0.151384	0.4901295	1124	1270	1491	1067	1370	1061
ILF2	0.8311116	-0.266886	0.0706209	11639	13703	15016	10373	12463	10706
ILF3	0.7706892	-0.375779	0.0029216	12621	13874	15278	9133	11641	11420
LTB4R2	0.615942	-0.699134	0.1370079	46	54	38	16	31	38
SNRPG	1.1207252	0.1644326	0.0880763	3412	3936	4290	4340	4857	3846
SNRPE	1.0422588	0.0597136	0.3185443	8997	9253	11093	10458	11119	9006
RNU6-269P	0	#NUM!	0.824326	1	0	0	0	0	0
SNRPF	0.9730838	-0.039364	0.823025	1616	1814	2180	1907	1896	1656
MTCO3P11	0	#NUM!	0.0857641	7	3	0	0	0	0
SNRPC	0.8811032	-0.182617	0.3803644	3971	5091	5623	4251	4752	3936
MTCO3P12	1.8	0.8479969	0.6069077	0	0	5	1	6	2
AC079465.1	0	#NUM!	0.5387425	3	0	0	0	0	0
SNRPA	1.06411	0.0896473	0.3114313	2452	2831	3296	3526	3139	2464
SNRPB	1.0003461	0.0004992	0.6586148	3204	4127	4226	4175	4012	3374
CEP104	1.1383388	0.1869299	0.0703043	910	1071	1402	1166	1313	1372
FAM114A1	0.8957952	-0.158759	0.6991806	162	143	242	137	171	182
FAM114A2	0.95703	-0.063364	0.9473098	1108	826	1231	781	1117	1131
AEBP1	1.5	0.5849625	0.8395779	0	0	2	0	3	0
KIAA0556	0.7103971	-0.493302	0.0031541	713	767	761	461	550	581
DDRGK1	0.9036145	-0.146221	0.637462	795	1065	1128	948	869	883
MTCO3P29	2	1	0.6885184	3	0	0	0	1	5
MTCO3P22	0	#NUM!	0.824326	1	0	0	0	0	0
CEP112	1.2269939	0.295128	0.3701981	68	25	70	62	88	50
LPP-AS2	0	#NUM!	0.0583382	5	1	5	0	0	0
AL445646.1	0	#NUM!	0.5387425	3	0	0	0	0	0
PLEKHM1	0.8953875	-0.159416	0.486395	604	718	781	539	643	701
PLEKHM2	1.0064675	0.0093006	0.6172835	941	1106	1200	1101	1160	1007
ADORA2A-AS	#DIV/0!	#DIV/0!	0.0952959	0	0	0	5	0	4
PLEKHM3	1.006483	0.0093228	0.7793311	589	606	656	434	660	769
MTCO3P40	#DIV/0!	#DIV/0!	0.5509701	0	0	0	0	3	0
PLEKHF2	0.9515478	-0.071652	0.8741063	1003	851	1118	719	1054	1055
MTCO3P43	0.5	-1	0.8652929	0	0	2	0	1	0
PLEKHF1	1.0702128	0.0978976	0.2780829	1302	1590	1808	1800	1793	1437
CEP164	0.8798343	-0.184696	0.5026913	228	228	268	193	229	215
AC107031.1	0	#NUM!	0.4217812	0	0	5	0	0	0
CEP170	1.1873368	0.2477292	0.2279058	631	306	595	372	759	688
MTCO3P45	0	#NUM!	0.5781912	0	0	3	0	0	0
PLEKHG3	0.956825	-0.063673	0.8893235	2719	3121	3193	2363	3088	3192
RFC5	0.8392061	-0.252903	0.1159806	1397	1500	1587	1133	1291	1339
PLEKHG4	0.9040959	-0.145452	0.6985798	296	326	379	330	328	247
PLEKHG1	0.8644537	-0.210139	0.3531655	817	644	708	406	692	777
RFC3	0.93826	-0.09194	0.7515846	3293	2673	3574	2441	3258	3252
PLEKHG2	0.97118	-0.042189	0.9192691	539	600	700	521	633	632
RFC4	1.0727654	0.1013347	0.2243953	1835	1571	2311	2116	2232	1785

AL445250.1	1.3356643	0.4175575	0.2379241	69	24	50	76	53	62
PLEKHG7	0	#NUM!	0.0321694	5	2	6	0	0	0
RFC1	1.0146101	0.0209254	0.6717592	2126	1251	2304	1425	2240	2099
RFC2	0.895082	-0.159908	0.4830715	1770	2227	2408	1902	2010	1821
PLEKHG5	0.8318386	-0.265625	0.3254768	273	295	324	254	213	275
PLEKHG6	1.0566976	0.0795626	0.2831952	1725	2049	2258	2155	2091	2128
AC093821.1	0.8699187	-0.201048	0.7088524	25	64	34	30	50	27
AC007611.1	5.125	2.357552	0.0370537	0	6	2	3	13	25
CYP4A22-AS1	1.5217391	0.6057211	0.4827543	15	1	7	8	9	18
MTFR1	0.9596691	-0.059391	0.9700845	672	572	690	565	682	609
MTFR2	0.9372659	-0.09347	0.874754	651	592	893	626	725	651
AC025183.3	5	2.3219281	0.4088931	0	1	0	5	0	0
Z86062.1	0	#NUM!	0.824326	1	0	0	0	0	0
AC025183.1	1.5	0.5849625	0.3461539	10	19	11	14	17	29
PLEKHH2	0.9927536	-0.010492	0.928685	37	51	50	35	51	51
PLEKHH3	0.9979065	-0.003023	0.7240483	861	1001	1004	946	1056	858
AL390955.2	0.7142857	-0.485427	0.8351178	0	2	5	0	5	0
MTFP1	1.0338983	0.0480943	0.8257545	14	19	26	23	24	14
AP006284.1	0.9090909	-0.137504	0.9391106	4	2	5	1	5	4
RFFL	0.7621247	-0.391901	0.0478371	405	465	429	301	350	339
PLEKHH1	1.1231574	0.1675601	0.1591771	697	626	780	637	767	958
AC073052.1	0.1	-3.321928	0.1719952	0	5	5	0	0	1
SLC25A1P5	#DIV/0!	#DIV/0!	0.5082165	0	0	0	1	0	2
CEP152	1.1082803	0.1483227	0.3136309	408	218	473	356	459	403
AC009078.1	0	#NUM!	0.6385096	0	2	0	0	0	0
AC009078.2	1	0	0.9890559	0	0	1	1	0	0
AC009078.3	0.4318182	-1.211504	0.1601031	15	13	16	5	3	11
SNX29P1	0	#NUM!	0.824326	0	0	1	0	0	0
AP000708.1	1.6666667	0.7369656	0.7964	3	0	0	0	4	1
KIAA0586	1.150518	0.2022835	0.1621962	629	406	606	489	730	669
CEP162	1.4097421	0.4954313	0.0210269	245	130	323	301	404	279
RN7SL172P	0	#NUM!	0.2218177	2	0	5	0	0	0
LINC002481	1.2368421	0.3066613	0.448214	20	30	26	34	31	29
AL022157.1	0.7916667	-0.337035	0.7902927	2	12	10	6	4	9
DZIP1L	1.4305556	0.5165755	0.3181652	19	28	25	14	35	54
VRTN	0	#NUM!	0.355562	5	0	0	0	0	0
AF235103.1	0.6666667	-0.584963	0.8466488	3	0	0	0	0	2
RN7SL170P	0.75	-0.415037	0.848523	2	2	4	0	0	6
PGAM1P5	1.3333333	0.4150375	0.7148188	3	12	0	12	4	4
SPTBN4	1.0952381	0.1312445	0.8293331	13	29	21	10	40	19
SPTBN5	2.0681818	1.048363	0.0008238	37	37	58	73	111	89
PGAM1P8	1	0	0.8992569	6	1	11	7	3	8
PGAM1P9	#DIV/0!	#DIV/0!	0.3981469	0	0	0	4	0	0
FUT10	0.7421227	-0.43027	0.0271543	399	370	437	246	313	336
PGAM1P6	0	#NUM!	0.4324683	2	0	2	0	0	0
FUT11	1.2209507	0.288005	0.0311636	377	315	444	412	481	494
PGAM1P7	1.6363636	0.7104934	0.4961963	2	0	9	6	7	5
AP001046.1	0	#NUM!	0.6840298	0	0	2	0	0	0
SPTBN1	0.8925339	-0.164021	0.3968056	8109	8120	8188	4910	7985	8898
SPTBN2	0.8586387	-0.219877	0.5042763	119	161	102	80	157	91
SOSTDC1	3.5	1.8073549	0.4618526	0	0	2	0	0	7
CBFA2T2	0.7736189	-0.370305	0.0095024	1402	1448	1585	995	1250	1186
CBFA2T3	1.8904594	0.9187368	9.49E-05	70	90	123	180	189	166
MIR17HG	0.8508475	-0.233028	0.521018	492	208	480	330	363	311

AC096711.1	1	0	0.9602092	4	0	0	4	0	0
RFNG	1.2815166	0.3578522	0.0237778	304	333	418	516	449	387
KIAA0513	0.9207843	-0.119065	0.727956	361	437	477	373	399	402
AC096711.2	#DIV/0!	#DIV/0!	0.6554062	0	0	0	0	2	0
AC096711.3	0.9166667	-0.125531	0.9643764	14	6	16	14	14	5
AC090541.1	0	#NUM!	0.824326	1	0	0	0	0	0
Z83818.2	0	#NUM!	0.6385096	0	2	0	0	0	0
TMEM38A	1	0	0.940181	3	18	15	11	14	11
TMEM38B	1.1921032	0.2535091	0.0095297	2510	2207	2805	2468	3377	3122
AC022211.2	0.8148148	-0.295456	0.5616479	41	46	21	22	32	34
AC022211.3	1.047619	0.0671142	0.840827	12	10	20	10	15	19
AC022211.4	4	2	0.5239404	0	1	0	0	1	3
NOSIP	0.9574103	-0.062791	0.9460311	1714	1936	2173	2074	1814	1687
AC022211.1	0.4782609	-1.06413	0.3184324	9	8	6	2	5	4
MYCL	1.2186992	0.2853421	0.0298618	366	401	463	477	491	531
MYCN	1.14439	0.1945788	0.0207117	6262	6514	7634	7499	8377	7481
EEF1E1P1	1.3454545	0.4280937	0.4386651	26	15	14	16	31	27
AL360181.1	1	0	0.9890559	0	1	0	0	1	0
AL360181.2	2.2962963	1.1993088	0.096469	14	8	5	20	9	33
AL360181.3	1	0	0.9890559	0	1	0	0	0	1
AL360181.4	0	#NUM!	0.4374838	0	4	0	0	0	0
TCEAL3-AS1	3	1.5849625	0.6048817	0	1	0	1	1	1
AC012313.8	2.3888889	1.2563398	0.1029433	6	5	7	25	9	9
AC012313.9	0	#NUM!	0.2359908	1	0	6	0	0	0
MEGF10	#DIV/0!	#DIV/0!	0.3981469	0	0	0	4	0	0
MEGF11	0.64	-0.643856	0.4105426	16	11	23	9	10	13
MIR1827	#DIV/0!	#DIV/0!	0.8030358	0	0	0	0	1	0
H2AFJ	1.0957935	0.1319759	0.2253819	2803	3498	3731	4270	3789	2934
AC012313.1	0.8556188	-0.22496	0.2815799	443	474	489	372	423	408
AC012313.2	0.5106383	-0.969626	0.2018646	17	17	13	5	9	10
AC012313.3	0.2413793	-2.050626	0.0655206	12	7	10	1	4	2
AC012313.4	0.5555556	-0.847997	0.4054869	9	9	27	2	22	1
AC012313.5	1	0	0.9637713	6	9	8	5	7	11
AC012313.6	0.5833333	-0.777608	0.6995806	1	4	7	5	0	2
RPLP1P13	0.8333333	-0.263034	0.9492372	0	0	6	0	5	0
H2AFY	0.895166	-0.159773	0.3466139	8214	8954	10056	7582	8747	8041
H2AFZ	1.2059552	0.2701763	0.0107015	4900	5779	6684	7127	7527	6285
H2AFX	0.8713411	-0.198691	0.3482664	5021	6304	6474	5465	5336	4708
MTDHP1	3.2857143	1.716207	0.2396898	5	0	2	0	13	10
H2AFV	1.0167388	0.0239491	0.4896651	3681	4131	4435	4022	4410	4020
MTDHP4	0	#NUM!	0.824326	1	0	0	0	0	0
H3F3AP1	#DIV/0!	#DIV/0!	0.8030358	0	0	0	0	1	0
AL160286.3	1	0	0.9756864	2	0	0	0	2	0
MYH1	0	#NUM!	0.824326	0	1	0	0	0	0
MYH2	0	#NUM!	0.824326	1	0	0	0	0	0
MYH3	1.9655172	0.974909	0.1341921	6	5	18	15	24	18
H3F3AP5	0	#NUM!	0.824326	0	0	1	0	0	0
RASA3	0.5179856	-0.949016	0.0493842	55	31	53	22	24	26
H3F3AP6	1.2411765	0.3117083	0.2601293	59	50	61	71	62	78
RASA4	0.2	-2.321928	0.4184479	1	4	0	0	1	0
RASA1	1.1594107	0.2133917	0.1475109	1675	1123	1750	1175	2012	2086
AL160286.1	#DIV/0!	#DIV/0!	0.4501902	0	0	0	0	0	4
H3F3AP4	1.1491024	0.2005074	0.0183336	3699	3765	4568	4427	4925	4474
RASA2	0.8162428	-0.29293	0.2512284	492	277	450	242	399	354

RFT1	0.7171028	-0.479748	0.0046499	762	926	949	603	640	648
MYH8	4	2	0.5294903	0	0	1	0	1	3
MYH9	0.8364758	-0.257604	0.0877961	43999	53838	53370	35777	44633	46071
MYH6	5	2.3219281	0.3869708	1	0	0	3	0	2
MYH7	0	#NUM!	0.824326	0	0	1	0	0	0
BRAT1	0.8692217	-0.202204	0.3674471	746	844	941	762	754	684
CIART	2.7714286	1.4706298	0.0098314	5	9	21	24	37	36
RASD2	0.05	-4.321928	0.0173386	4	9	7	0	1	0
RASD1	0	#NUM!	0.5643268	1	0	2	0	0	0
MKNK1-AS1	0.5128205	-0.963474	0.3584772	6	5	28	5	6	9
TMEM30A	0.958622	-0.060966	0.881907	3806	3099	3777	2481	3882	3877
TMEM30B	1	0	0.9890559	1	0	0	0	0	1
ZNF571-AS1	0.7685185	-0.379848	0.4371401	44	74	98	54	41	71
OPA1-AS1	1.6666667	0.7369656	0.7035687	2	4	0	0	2	8
AL357140.2	3.375	1.7548875	0.0910945	2	2	4	7	6	14
AL357140.4	0.5	-1	0.8652929	0	0	2	0	0	1
FZD10	0.932133	-0.101392	0.8032475	192	263	267	189	253	231
GHITM	1.2338253	0.3031382	0.0033371	2540	2765	2825	2880	3754	3397
MYLK	1.1423488	0.1920032	0.4601956	101	58	122	62	114	145
FO538757.2	0.5483871	-0.866733	0.40752	15	6	10	2	2	13
FO538757.1	0.8754579	-0.19189	0.6728913	174	147	225	190	147	141
RFX2	0.970964	-0.04251	0.9296692	242	296	323	265	304	267
RFX3	1.4255319	0.5115003	0.0076275	246	206	253	227	404	374
RFX1	0.9155329	-0.127316	0.6513727	473	654	637	485	587	543
MYL4	1.8245614	0.8675497	0.0800642	9	22	26	36	43	25
MYL5	1.4453125	0.5313815	0.0424999	88	80	88	121	157	92
MYL6	1.0453037	0.0639221	0.3995379	1337	1764	2020	2010	1793	1550
RFX8	8	3	0.2574152	0	0	1	0	7	1
MYL7	1.5	0.5849625	0.8192369	0	0	2	0	2	1
MYL1	0	#NUM!	0.824326	1	0	0	0	0	0
RFX7	0.8120457	-0.300367	0.1798563	706	508	712	381	523	660
RFX4	#DIV/0!	#DIV/0!	0.5371397	0	0	0	0	0	3
MYL3	0.6315789	-0.662965	0.5551898	3	13	3	2	5	5
RFX5	0.8318452	-0.265613	0.2369015	444	380	520	276	392	450
MYL9	1.0615744	0.0862055	0.3890411	349	447	487	418	500	444
OR8B1P	#DIV/0!	#DIV/0!	0.4242335	0	0	0	0	3	1
HCG4P7	0	#NUM!	0.355562	5	0	0	0	0	0
HCG4P3	1	0	0.9742162	0	0	3	0	3	0
OR1AC1P	0	#NUM!	0.824326	0	1	0	0	0	0
HNRNPLP1	1	0	0.9890559	0	1	0	1	0	0
MYNN	1.0104012	0.0149282	0.7420766	292	145	236	246	221	213
RNU6-91P	#DIV/0!	#DIV/0!	0.6554062	0	0	0	0	2	0
GABARAP	0.625	-0.678072	0.6053467	8	5	3	5	4	1
HSD11B1	1	0	0.9890559	0	0	1	0	1	0
HSD11B2	3.0116279	1.5905435	1.50E-10	58	73	127	242	315	220
AP002364.1	0.3243243	-1.624491	0.108329	8	14	15	5	1	6
MYO6	1.014004	0.0200633	0.6605519	1259	767	1473	886	1315	1347
OR8B12	1.6666667	0.7369656	0.7561327	0	3	0	2	0	3
RNU6-90P	#DIV/0!	#DIV/0!	0.8030358	0	0	0	1	0	0
AL162586.1	1.4444444	0.5305147	0.1362428	44	23	50	47	68	54
AC002563.1	0.3333333	-1.584963	0.6805352	0	1	2	0	0	1
SNUPN	0.7289562	-0.456096	0.0672576	182	198	214	136	166	131
HIST1H1PS1	1.1	0.1375035	0.9241059	2	6	2	1	2	8
MYMX	0.5783133	-0.790077	0.2005826	37	19	27	16	22	10

HNRNPLP2	1.0833333	0.1154772	0.5109683	94	89	141	88	128	135
AC025434.1	0.9090909	-0.137504	0.9797262	0	0	11	1	2	7
DGAT2L7P	0	#NUM!	0.824326	1	0	0	0	0	0
OR8B7P	0.5	-1	0.81753	2	0	0	0	0	1
AL138976.2	1.3684211	0.4525122	0.4515055	8	16	14	13	20	19
MYPN	0.7647059	-0.387023	0.681258	17	9	25	16	10	13
VSIR	1.6166884	0.6930416	9.13E-07	927	977	1164	1857	1620	1483
CICP5	12	3.5849625	0.0185948	0	1	1	5	4	15
CICP3	0.1111111	-3.169925	0.0817149	1	7	10	0	0	2
CICP4	#DIV/0!	#DIV/0!	0.3096126	0	0	0	5	0	0
CICP9	0.4375	-1.192645	0.3709327	5	9	2	0	5	2
CICP7	0.5	-1	0.8652929	0	0	2	0	1	0
CICP8	1.25	0.3219281	0.8749798	0	0	4	0	5	0
NDFIP1P1	0	#NUM!	0.5387425	3	0	0	0	0	0
MYOF	0.966572	-0.049051	0.9452798	6712	6356	7364	4592	7311	7846
SNRPD2P1	0.3333333	-1.584963	0.6394405	1	2	0	0	1	0
MYOT	0.9166667	-0.125531	0.8874462	15	12	21	5	25	14
AC091429.1	6.5	2.7004397	0.1414732	0	2	0	3	5	5
MYRF	0.8395062	-0.252387	0.6582675	44	51	67	53	40	43
OR8B5P	1	0	0.9890559	1	0	0	0	1	0
CTBP1-AS	0.8846154	-0.176878	0.8118697	30	25	23	26	28	15
AC010983.1	2.25	1.169925	0.593452	4	0	0	0	1	8
EBAG9P1	0	#NUM!	0.824326	0	1	0	0	0	0
KIAA0895	0.7950139	-0.330948	0.334803	157	93	111	63	102	122
CXCR2P1	#DIV/0!	#DIV/0!	0.6411933	0	0	0	0	0	2
MYT1	0.375	-1.415037	0.4983528	0	8	0	0	2	1
SAP30	1.4798762	0.5654765	0.0029607	161	238	247	329	341	286
AL050331.3	1	0	0.9890559	0	0	1	0	0	1
RN7SL189P	#DIV/0!	#DIV/0!	0.8030358	0	0	0	0	0	1
RNU6-98P	0	#NUM!	0.6436235	2	0	0	0	0	0
GABPB1-AS1	1.0665962	0.0930141	0.4317689	318	192	436	313	370	326
snoU2_19	0	#NUM!	0.824326	0	0	1	0	0	0
snoU2_19	0	#NUM!	0.824326	0	0	1	0	0	0
snoU2_19	0	#NUM!	0.824326	0	0	1	0	0	0
snoU2_19	0	#NUM!	0.824326	0	0	1	0	0	0
RGCC	1.3125	0.3923174	0.5746682	11	10	11	6	17	19
AC068888.2	0.2	-2.321928	0.1639569	9	4	2	0	3	0
AC068888.1	0.8636364	-0.211504	0.8143843	30	19	39	28	19	29
AC079466.1	0	#NUM!	0.2452252	0	5	1	0	0	0
CCNT2-AS1	0.4	-1.321928	0.2103604	9	6	15	4	6	2
AL356805.1	0	#NUM!	0.824326	1	0	0	0	0	0
EDIL3	1.9705882	0.9786263	0.0117101	34	17	51	47	73	81
CTNNBL1	1.0228733	0.0326275	0.445451	2535	3023	3623	3110	3350	2931
AL445647.1	0.3023256	-1.725825	0.0113986	42	21	23	7	15	4
DDOST	0.9939548	-0.008748	0.6678959	8681	10451	11471	9687	10895	9836
NT5C3A	1.0252774	0.0360143	0.5678767	600	425	597	499	575	589
NT5C3B	0.9125243	-0.132065	0.6204695	1019	1311	1271	1095	1174	1017
YRDCP2	0	#NUM!	0.824326	0	0	1	0	0	0
SRRM2-AS1	0.7655172	-0.385493	0.4306249	48	43	54	40	32	39
AC107032.1	1	0	0.913945	0	2	6	5	3	0
AC107032.2	0.5454545	-0.874469	0.4727376	7	15	0	3	2	7
SAP18	0.9621182	-0.055714	0.952335	4442	4793	5627	4644	5150	4505
SUB1P1	1.1428571	0.1926451	0.8800312	1	4	2	4	4	0
SUB1P2	#DIV/0!	#DIV/0!	0.4501902	0	0	0	0	0	4

AC092112.1	1	0	0.9074736	4	0	13	6	9	2
IMMT	0.9986482	-0.001952	0.6132187	5489	5271	6254	5039	6183	5769
NOVA1	0.1925926	-2.376376	0.0001099	43	35	57	15	5	6
NT5C1A	#DIV/0!	#DIV/0!	0.6411933	0	0	0	0	0	2
NT5C1B	#DIV/0!	#DIV/0!	0.6411933	0	0	0	0	0	2
TMEM41A	0.841623	-0.248754	0.1420079	915	1033	1108	736	928	908
NT5CP2	2.6666667	1.4150375	0.5371629	0	1	2	0	8	0
PLEKHB2	0.9145827	-0.128815	0.4874304	6736	7393	7904	5286	7457	7408
TMEM41B	0.9538889	-0.068107	0.8630558	2306	2259	2635	1833	2576	2459
NT5CP1	2	1	0.8072994	0	1	0	0	1	1
PLEKHB1	1.1944444	0.2563398	0.738508	8	18	10	5	19	19
AC126474.1	1.5	0.5849625	0.5410936	8	0	6	12	4	5
AC126474.2	0.7971014	-0.327165	0.527178	60	26	52	25	42	43
AC010889.1	0.9302326	-0.104337	0.9709847	20	1	22	7	14	19
IMP3	1.0764706	0.1063089	0.4704873	262	276	312	398	331	186
IMP4	0.8681107	-0.204049	0.2577823	2307	2700	2795	2176	2535	2062
TRIM60P17	0	#NUM!	0.824326	1	0	0	0	0	0
AC092111.1	#DIV/0!	#DIV/0!	0.8030358	0	0	0	0	0	1
TRIM60P18	1.1395349	0.1884451	0.6964482	23	4	16	24	14	11
TRIM60P19	#DIV/0!	#DIV/0!	0.5509701	0	0	0	0	3	0
AC010503.4	0.7358491	-0.442518	0.2407505	67	76	69	48	61	47
PLEKHD1	#DIV/0!	#DIV/0!	0.5371397	0	0	0	0	0	3
BRCC3	1.0975376	0.1342703	0.2927171	1223	770	1134	878	1336	1218
AL121983.2	0.1818182	-2.459432	0.1901018	4	4	3	1	1	0
RN7SL181P	1.0625	0.0874628	0.8982244	1	7	8	4	9	4
XRCC6	0.9009615	-0.150463	0.401042	19275	21891	24152	18330	20836	19683
XRCC4	1.0203785	0.0291043	0.6490244	515	336	523	423	572	407
XRCC5	1.0766687	0.1065743	0.1306218	19063	16576	21503	17572	22815	21136
XRCC2	0.8869801	-0.173026	0.4363375	800	639	773	462	729	771
EAF1	0.9182288	-0.123074	0.5708135	1043	992	1059	815	1025	1001
XRCC3	1.0181269	0.0259174	0.5679469	548	646	792	836	624	562
EAF2	1.275	0.3504972	0.0932672	124	100	136	124	163	172
PNMA8A	#DIV/0!	#DIV/0!	0.5509701	0	0	0	0	3	0
XRCC1	1.0248668	0.0354364	0.5533774	561	786	905	869	812	627
PNMA8B	#DIV/0!	#DIV/0!	0.5070017	0	0	0	3	0	0
RGL3	1.2567568	0.3297054	0.0594992	169	156	267	260	252	232
RGL4	0.3275862	-1.610053	0.0951468	25	7	26	0	4	15
RGL1	0.9197366	-0.120707	0.5724551	2045	1998	2336	1504	2121	2242
KIAA0825	0.6285714	-0.669851	0.6282693	12	0	23	0	6	16
RGL2	1.0215818	0.0308047	0.4706857	2180	2271	2870	2692	2645	2142
VSX2	6	2.5849625	0.2916217	0	0	1	3	2	1
BARHL1	0	#NUM!	0.0271755	5	5	3	0	0	0
BARHL2	0.3333333	-1.584963	0.4763383	2	3	1	0	2	0
BRCA1	0.9790922	-0.030483	0.9044955	1346	835	1454	862	1387	1310
BRCA2	1.1663468	0.2219968	0.2285464	651	300	612	443	717	663
PNMA6E	0.375	-1.415037	0.2407494	4	13	7	1	1	7
ARIH2	0.7852119	-0.348846	0.014379	3004	3495	4023	2333	3159	2770
QSOX1	1.1898996	0.2508399	0.0103456	6286	7576	8355	8512	9440	8484
ARIH1	1.0394706	0.0558489	0.4385149	1526	1191	1514	1225	1634	1539
QSOX2	0.8544742	-0.226891	0.2094006	1265	1460	1611	981	1290	1434
NBPF4	1.5238095	0.6076826	0.2898439	16	9	17	16	15	33
NXPE3	0.6529032	-0.615059	0.0134372	261	238	276	111	174	221
VKORC1	1.21875	0.2854022	0.6362695	11	10	11	16	16	7
NXPE4	0	#NUM!	0.824326	1	0	0	0	0	0

NBPF3	0.9671733	-0.048154	0.9751667	514	549	582	402	575	614
NBPF8	0.8597237	-0.218055	0.4474282	361	234	346	261	267	281
NBPF9	0.7832253	-0.352501	0.0355363	804	806	858	507	762	664
NBPF6	0.6097561	-0.713696	0.3806682	12	22	7	2	13	10
NXPE1	0	#NUM!	0.6436235	2	0	0	0	0	0
NXPE2	0	#NUM!	0.824326	0	0	1	0	0	0
RGMB	0.8926554	-0.163825	0.5797862	324	348	390	326	349	273
NBPF1	1.070122	0.0977752	0.4475837	297	352	335	278	395	380
PNMA6A	0	#NUM!	0.824326	0	1	0	0	0	0
RGMA	0.8401015	-0.251364	0.2114865	508	518	550	427	473	424
MZB1	0	#NUM!	0.6631647	1	0	1	0	0	0
AC092747.4	0.920765	-0.119095	0.843586	142	67	157	85	162	90
AC092747.2	0	#NUM!	0.6410557	1	1	0	0	0	0
AC069236.1	0	#NUM!	0.824326	0	0	1	0	0	0
AL365226.1	0	#NUM!	0.824326	0	1	0	0	0	0
AL365226.2	0.8333333	-0.263034	0.9462128	2	0	4	2	2	1
AC011239.2	0	#NUM!	0.824326	0	0	1	0	0	0
PLEKHA1	1.2554714	0.3282291	0.013402	829	676	871	752	1121	1110
PLEKHA2	0.7047619	-0.504792	0.0423719	293	332	320	152	210	304
PLEKHA5	0.9467377	-0.078963	0.834669	536	398	568	314	636	472
PLEKHA6	1.1750958	0.2327783	0.0125358	9379	10336	11091	10148	12951	13101
PLEKHA3	0.9493088	-0.075051	0.8985296	367	334	384	317	379	334
AC008739.2	0	#NUM!	0.824326	1	0	0	0	0	0
PLEKHA4	1.2763158	0.3519853	0.0184976	289	295	404	404	490	367
AC008739.5	1	0	0.9890559	0	1	0	0	0	1
PLEKHA7	1.0272597	0.0388009	0.5453748	850	924	1014	764	1123	977
PLEKHA8	0.8731151	-0.195756	0.3430937	2525	2089	2747	1431	2359	2637
RGP1	0.9671408	-0.048202	0.9772118	1154	1480	1444	1016	1357	1571
DPY19L1	0.9362984	-0.09496	0.7449402	1204	985	1296	776	1226	1261
DPY19L2	#DIV/0!	#DIV/0!	0.8030358	0	0	0	0	0	1
DPY19L3	0.8965794	-0.157497	0.5079702	1310	1006	1426	781	1210	1364
DPY19L4	0.9194167	-0.121209	0.6739634	456	376	471	229	491	478
STK17A	0.9149296	-0.128267	0.6317843	1103	1045	1402	1038	1170	1040
STK17B	1.2088566	0.2736431	0.0896631	612	340	561	513	665	651
NPHP3-AS1	1	0	0.9699945	2	0	0	2	0	0
Z69890.1	0	#NUM!	0.0493849	5	5	1	0	0	0
FHOD3	0.9243243	-0.113529	0.8255551	59	68	58	37	55	79
LENG8-AS1	0.8025316	-0.31737	0.3370087	134	96	165	77	120	120
FHOD1	0.9255987	-0.111541	0.6684898	2397	2639	2773	2371	2544	2313
WWTR1-AS1	0.6	-0.736966	0.4783185	3	13	14	2	6	10
RGS5	1.0896226	0.1238286	0.6155369	85	54	73	55	86	90
RGS5	1.0896226	0.1238286	0.6155369	85	54	73	55	86	90
RGS2	1.3496732	0.4326101	0.0823986	110	87	109	163	153	97
RGS3	1.4762785	0.5619649	6.89E-05	476	568	579	804	843	749
ARID2	0.812828	-0.298978	0.2242314	1413	799	1218	536	1115	1137
RGS8	1.2105263	0.2756344	0.6823429	4	6	9	11	4	8
RGS9	0.831068	-0.266962	0.3764461	149	219	147	133	160	135
RGS6	0.4285714	-1.222392	0.5817415	2	1	4	1	0	2
RGS7	0.7142857	-0.485427	0.7990703	6	1	0	2	3	0
GPANK1	0.8810241	-0.182747	0.4625974	686	927	1043	768	817	755
MZF1	0.9233577	-0.115039	0.86667	222	271	329	291	264	204
NXPH3	1.3076308	0.3869553	0.000561	756	803	944	981	1203	1089
YTHDC1	1.1229914	0.1673469	0.0940528	3426	2660	3809	2949	4243	3920
NXPH4	0.4623824	-1.112841	5.78E-06	176	211	251	80	113	102

YTHDC2	0.8964753	-0.157664	0.5663534	1652	1010	1849	1055	1545	1444
AC010409.1	0	#NUM!	0.4521054	1	0	3	0	0	0
AL589826.2	2	1	0.7741804	1	0	0	2	0	0
MIR515-2	3	1.5849625	0.6732949	0	0	1	0	0	3
AC009171.2	1	0	0.9483038	4	5	9	6	11	1
NXPH1	0	#NUM!	0.1675055	0	3	5	0	0	0
AP003108.5	0.6153846	-0.70044	0.5440634	9	7	10	6	1	9
AP003108.1	0	#NUM!	0.5337954	0	3	0	0	0	0
AP003108.2	0.8688525	-0.202817	0.8758086	21	13	27	22	9	22
CACFD1	0.8955386	-0.159172	0.6563649	204	333	382	245	302	276
VTA1	0.9607567	-0.057757	0.9760849	2255	1669	2472	1615	2267	2263
AP003108.4	0	#NUM!	0.6436235	2	0	0	0	0	0
AC007993.2	0	#NUM!	0.824326	1	0	0	0	0	0
TMEM45B	1.0468085	0.0659976	0.6070788	135	158	177	141	200	151
TRIAP1	0.8253615	-0.276902	0.0841891	2161	2385	2646	1935	2158	1843
AP001630.1	0.4444444	-1.169925	0.3682749	6	5	7	4	2	2
AL136531.2	0	#NUM!	0.5337954	0	3	0	0	0	0
AL136531.1	2.2857143	1.1926451	0.3059839	1	2	4	7	4	5
EAPP	1.0799512	0.1109661	0.1386352	3102	3058	3671	3251	4027	3339
TMEM45A	1.1574697	0.2109744	0.1439127	214	233	296	258	327	275
AC105760.1	#DIV/0!	#DIV/0!	0.5371397	0	0	0	0	0	3
AC105760.2	0.6838235	-0.548304	0.249602	55	31	50	25	35	33
AL596247.1	2.6666667	1.4150375	0.4696794	0	1	2	1	0	7
AC087500.1	0.88	-0.184425	0.9154294	11	4	10	9	6	7
AC087500.2	4	2	0.5760976	1	0	0	0	4	0
AC104109.2	1.4210526	0.50696	0.5802924	4	5	10	2	15	10
AC104109.4	1.4285714	0.5145732	0.7223637	4	3	0	5	2	3
NCF4-AS1	1	0	0.9804455	0	4	1	2	0	3
BOK-AS1	0	#NUM!	0.6840298	0	0	2	0	0	0
CEP170P1	1.6	0.6780719	0.7041501	2	1	2	0	5	3
AC090204.1	0.240458	-2.056143	4.18E-07	93	76	93	22	15	26
Z74021.1	0.8222222	-0.2824	0.8065093	8	24	13	22	7	8
HNRNPKP1	0.5	-1	0.6640745	1	0	7	0	3	1
HNRNPKP2	1.5714286	0.6520767	0.609281	4	1	2	5	1	5
ARIH2P1	0	#NUM!	0.824326	0	1	0	0	0	0
ARIH2OS	0.3974359	-1.331206	0.0079131	48	36	72	26	18	18
AC087741.3	0.721519	-0.470891	0.4193707	23	31	25	11	30	16
AC087741.1	0.9753086	-0.036069	0.9988888	82	65	96	40	96	101
AC087741.2	#DIV/0!	#DIV/0!	0.6554062	0	0	0	0	2	0
AC019068.1	0.90625	-0.142019	0.8972606	16	5	11	6	12	11
HNRNPKP4	0.8758414	-0.191258	0.4027772	418	469	450	261	483	427
SNU13	0.966581	-0.049037	0.8783372	3803	4524	5288	4638	4669	3853
POU5F1B	4	2	0.5246085	1	0	0	0	1	3
INE1	1.3170732	0.3973355	0.4713231	16	13	12	25	16	13
EZH2P1	#DIV/0!	#DIV/0!	0.8030358	0	0	0	0	0	1
AC084816.1	#DIV/0!	#DIV/0!	0.6411933	0	0	0	0	0	2
AL645939.5	2.5	1.3219281	0.5958871	2	0	0	5	0	0
KIAA0319L	0.9414063	-0.087111	0.7582799	1517	1620	1727	1297	1642	1640
NXNL2	1.5510204	0.6332177	0.0412978	41	45	61	71	64	93
AL645939.4	#DIV/0!	#DIV/0!	0.8030358	0	0	0	1	0	0
MTHFR	0.7914508	-0.337428	0.1621692	281	248	243	196	224	191
MTHFS	0.5517241	-0.857981	0.4454878	4	7	18	1	6	9
GLUD1	1.1213396	0.1652233	0.0392819	3641	3473	4292	3680	4662	4448
GLUD2	0.6491228	-0.623437	0.4439834	18	13	26	2	11	24

RNU7-41P	#DIV/0!	#DIV/0!	0.6317974	0	0	0	1	1	0
ING1	1.2584551	0.3316538	0.0075835	717	804	874	985	1134	895
ING4	0.912704	-0.131781	0.6121741	433	454	522	377	483	426
ING5	1.0186335	0.0266351	0.5195661	869	1020	1170	1088	1046	982
ING2	1.1773113	0.2354958	0.0414493	664	622	953	829	991	816
ING3	0.9958159	-0.006049	0.7228022	301	258	397	295	314	343
TNNC1	0.9365079	-0.094637	0.942634	99	127	152	131	120	103
ATPIF1	1.0879487	0.1216105	0.1752748	2313	2790	3311	3208	3196	2750
TNNC2	0.6451613	-0.632268	0.51359	8	14	9	9	5	6
RNU7-40P	0	#NUM!	0.6436235	2	0	0	0	0	0
FLJ42393	#DIV/0!	#DIV/0!	0.4737294	0	0	0	0	4	0
INF2	1.0678048	0.094648	0.1961698	10875	12544	13717	13332	13434	12888
AC004865.2	1.6	0.6780719	0.6596042	2	0	3	3	0	5
AL138831.1	0.75	-0.415037	0.8904058	1	0	3	0	2	1
AL138831.2	#DIV/0!	#DIV/0!	0.0417137	0	0	0	8	1	2
UBQLN4P1	0.7710843	-0.375039	0.5419231	29	19	35	15	30	19
AL138831.3	0.5625	-0.830075	0.2427066	27	12	25	9	20	7
RHBG	0.4924242	-1.022026	0.0360714	51	40	41	23	19	23
MZT1	1.0883392	0.1221283	0.1764511	1168	994	1234	1129	1389	1178
HYI-AS1	0.8	-0.321928	0.8801673	3	2	0	1	0	3
AC245028.1	0	#NUM!	0.824326	1	0	0	0	0	0
RHAG	#DIV/0!	#DIV/0!	0.5509701	0	0	0	0	3	0
NOTUM	1.5816327	0.6614146	0.0703651	34	22	42	50	54	51
INHA	0.3157895	-1.662965	0.2183376	7	11	1	0	4	2
MIR516B2	0.4	-1.321928	0.610796	0	4	1	0	0	2
AC092017.1	0.7916667	-0.337035	0.7162378	16	8	24	12	14	12
AC092017.2	1	0	0.9806644	2	2	0	1	3	0
AC104837.1	#DIV/0!	#DIV/0!	0.4909737	0	0	0	2	0	1
MAN1B1-AS1	0.609375	-0.714598	0.3208853	19	18	27	15	8	16
AL136040.1	1.25	0.3219281	0.8595692	0	0	4	0	4	1
AL136040.2	0.6046512	-0.725825	0.185373	20	60	49	12	32	34
STK32A	0.5	-1	0.8241474	0	1	1	0	0	1
STK32B	0	#NUM!	0.824326	1	0	0	0	0	0
STK32C	0.9868421	-0.019109	0.970068	25	33	18	15	39	21
INIP	1.120777	0.1644993	0.107872	1606	1431	1699	1425	1859	2024
RALGAPB	0.9293194	-0.105754	0.6591327	2667	2281	2692	1604	2662	2834
CEBPB-AS1	0.4814815	-1.054448	0.1546523	14	19	21	4	11	11
RHCG	0	#NUM!	0.6436235	2	0	0	0	0	0
RHCE	1.1	0.1375035	0.8281851	4	0	6	7	2	2
ITPRIPL2	0.6859791	-0.543763	0.0008217	1396	1339	1580	701	1121	1138
ITPRIPL1	4.9428571	2.3053452	1.15E-06	12	13	10	43	59	71
MIR516A1	0	#NUM!	0.824326	1	0	0	0	0	0
CEP350	1.1003236	0.1379279	0.3273807	950	523	999	670	1060	990
RHEB	0.9789896	-0.030635	0.8167849	1853	2086	2296	1976	2189	1939
OR8C1P	0	#NUM!	0.4460243	4	0	0	0	0	0
GOLGA8CP	#DIV/0!	#DIV/0!	0.5070017	0	0	0	3	0	0
INMT	1	0	0.9312859	5	3	5	6	0	7
RNU7-49P	0	#NUM!	0.2038809	4	0	3	0	0	0
DUX4L9	3	1.5849625	0.6944949	0	0	1	0	3	0
AC011477.6	0	#NUM!	0.6436235	2	0	0	0	0	0
AC011477.7	3	1.5849625	0.6206259	1	0	0	3	0	0
AC011477.1	0.5652174	-0.823122	0.4826509	7	7	9	7	4	2
AC011477.2	0.5844156	-0.774933	0.2107505	27	18	32	10	17	18
AC011477.3	0.84375	-0.245112	0.868939	8	14	10	17	6	4

AC012066.1	0.5625	-0.830075	0.6304778	2	4	10	0	0	9
RN7SL2	0.9709091	-0.042592	0.8544053	63	74	138	103	86	78
RN7SL3	1.1818182	0.2410081	0.78235	4	0	7	6	0	7
GOLGA8EP	#DIV/0!	#DIV/0!	0.4909737	0	0	0	2	0	1
RNU7-48P	0.1818182	-2.459432	0.2173108	6	1	4	0	2	0
2RX5-TAX1BI	1.2025316	0.2660749	0.6044711	35	28	16	27	47	21
TNNI1	0.8055556	-0.311944	0.5009991	51	46	47	32	55	29
TNNI2	0.6808511	-0.554589	0.3612502	42	16	36	21	26	17
TNNI3	1.1333333	0.1805722	0.8852211	7	4	4	2	12	3
CTNNAL1	1.1781356	0.2365056	0.0438112	2873	1886	3190	2818	3490	3057
AL589182.1	1.1666667	0.2223924	0.8436889	10	0	2	5	1	8
AL589182.2	0.75	-0.415037	0.9019013	1	3	0	3	0	0
AL138737.1	0.7619048	-0.392317	0.7428681	11	2	8	0	8	8
GOLGA8DP	#DIV/0!	#DIV/0!	0.3721261	0	0	0	3	1	0
RNU7-47P	0.2	-2.321928	0.4605013	5	0	0	0	0	1
LX1A-SULT1A	0	#NUM!	0.824326	0	0	1	0	0	0
AC099508.2	0	#NUM!	0.824326	1	0	0	0	0	0
AC006041.1	#DIV/0!	#DIV/0!	0.6411933	0	0	0	0	0	2
TMEM52B	1.5652174	0.646363	0.2434092	11	13	22	13	27	32
UBA52P7	0	#NUM!	0.824326	1	0	0	0	0	0
UBA52P8	6	2.5849625	0.224159	0	2	0	0	5	7
EBF1	#DIV/0!	#DIV/0!	0.6099294	0	0	0	2	0	0
UBA52P5	4	2	0.5760976	1	0	0	0	4	0
BOLA3-AS1	1.409396	0.495077	0.1080749	37	54	58	71	63	76
EBF2	0	#NUM!	0.5172237	1	2	0	0	0	0
UBA52P6	1.2	0.2630344	0.8261487	2	5	3	2	4	6
AC008738.2	0.8	-0.321928	0.8327302	2	4	4	0	8	0
EBF3	2.0714286	1.0506261	0.2769669	8	2	4	6	15	8
UBA52P3	1	0	0.9890559	0	0	1	0	0	1
EBF4	1.5	0.5849625	0.6623976	4	2	4	0	7	8
AC008738.6	1.6428571	0.716207	0.4295035	3	2	9	6	9	8
ASS1P12	1.4615385	0.5474878	0.5862188	8	10	8	0	17	21
INTU	0.8	-0.321928	0.6883996	18	23	14	16	7	21
EBI3	0.3135819	-1.673086	1.31E-13	407	515	580	186	159	126
FSIP2-AS1	1	0	0.9451127	5	0	9	2	6	6
GOLGA8IP	0.7462687	-0.422233	0.5553396	16	18	33	14	23	13
AC136632.1	0.969697	-0.044394	0.9714921	9	12	12	11	6	15
TMEM50A	1.0142982	0.0204818	0.491333	1784	1738	2213	1719	2153	1945
INSR	0.6976301	-0.519466	0.0006427	1484	1538	1704	833	1214	1250
TMEM50B	0.9818529	-0.026421	0.8582101	338	300	409	287	382	359
CIDEA	1	0	0.9756864	2	0	0	0	2	0
AC010504.1	1.6470588	0.7198921	0.4724953	5	4	8	2	20	6
CIDEC	1.1304348	0.1768778	0.903682	3	17	3	6	19	1
TH2LCRR	1	0	0.9237926	1	0	3	3	1	0
RN7SL192P	0.1875	-2.415037	0.1459353	2	11	3	0	3	0
INVS	1.0148057	0.0212035	0.6267302	489	542	590	489	523	633
RNU6-282P	1	0	0.9833455	9	0	2	0	5	6
AL049597.1	0	#NUM!	0.824326	0	1	0	0	0	0
AL049597.2	0.6	-0.736966	0.5392548	2	9	9	1	6	5
C5orf64-AS1	0	#NUM!	0.6385096	0	2	0	0	0	0
RHOG	0.959464	-0.059699	0.9437022	938	913	1134	1041	1023	800
RHOH	0.5	-1	0.3191577	7	13	8	4	8	2
RHOF	1.8888889	0.9175378	0.3171581	10	3	5	7	4	23
RHOC	0.8763944	-0.190348	0.4818791	1490	1940	2128	1813	1694	1364

RHOD	0.6751295	-0.566764	0.0051879	517	715	698	454	459	390
ZNF503-AS2	1.2321429	0.3011695	0.540135	10	24	22	20	28	21
RHOA	0.8871258	-0.172789	0.3045052	19361	22130	24051	18217	21276	18651
RHOB	1.0390444	0.0552572	0.3599353	2600	3073	3368	3166	3243	2985
TNNT1	1.3031152	0.3819647	0.0043515	3313	4410	4764	5987	5671	4614
TNNT2	0.6171779	-0.696242	0.0042732	255	235	325	188	159	156
RHOJ	2	1	0.7741804	1	0	0	2	0	0
TNNT3	0.7257185	-0.462518	0.009441	1116	1228	1692	997	1030	902
RHOU	1.072732	0.1012897	0.2071314	8885	8001	9801	7513	10683	10432
RHOV	0.6284501	-0.67013	0.0333442	122	173	176	115	107	74
RHOQ	0.96633	-0.049412	0.9148479	110	52	135	97	105	85
AL161757.4	#DIV/0!	#DIV/0!	0.8030358	0	0	0	0	0	1
SMIM2-IT1	0	#NUM!	0.4460243	4	0	0	0	0	0
AL161757.2	0	#NUM!	0.6410557	1	1	0	0	0	0
KIAA0753	0.9661934	-0.049616	0.997461	516	392	571	356	563	510
AL133297.1	3	1.5849625	0.6732949	0	0	1	0	0	3
AL133297.2	0	#NUM!	0.1824345	5	2	0	0	0	0
SRSF9P1	1.8333333	0.8744691	0.3896986	0	5	7	15	3	4
SSC4D	1.125	0.169925	0.7552406	17	8	23	12	34	8
CMPK1	1.0070531	0.0101398	0.6005524	3025	2597	3452	2508	3503	3127
CMPK2	3.3943662	1.7631422	1.43E-09	50	53	39	126	197	159
BTF3L4	1.0139417	0.0199747	0.5180471	2492	2523	2875	2329	2963	2708
TMEM59L	0.7058824	-0.5025	0.837787	4	3	10	11	1	0
OR8B9P	0	#NUM!	0.6436235	2	0	0	0	0	0
AC005703.4	0	#NUM!	0.6840298	0	0	2	0	0	0
AC024896.1	0.7789731	-0.360355	0.0660868	623	613	809	544	581	468
EBPL	1.1302206	0.1766043	0.0755933	1283	1385	1594	1641	1713	1463
SUMO2P3	0.7948718	-0.331206	0.7633565	12	11	16	16	9	6
SUMO2P6	1.1	0.1375035	0.8175214	4	5	11	7	11	4
AC087501.1	0	#NUM!	0.5781912	0	0	3	0	0	0
AC087501.3	#DIV/0!	#DIV/0!	0.8030358	0	0	0	0	1	0
AC087501.4	0	#NUM!	0.3473206	0	5	0	0	0	0
AC087501.5	1.3376623	0.419714	0.3389907	30	28	19	44	30	29
SUMO2P1	1.2883436	0.3655174	0.210051	40	55	68	61	66	83
FAM8A6P	#DIV/0!	#DIV/0!	0.5070017	0	0	0	3	0	0
AC012555.1	5	2.3219281	0.3910149	0	0	1	3	0	2
BTF3P2	#DIV/0!	#DIV/0!	0.8030358	0	0	0	0	1	0
GOLGA8VP	1.475	0.560715	0.3210428	14	13	13	25	21	13
BTF3P5	1	0	0.9890559	0	1	0	1	0	0
BTF3P6	0	#NUM!	0.6410557	1	1	0	0	0	0
BTF3P7	#DIV/0!	#DIV/0!	0.3721261	0	0	0	3	1	0
MTIF2	1.1481632	0.1993277	0.1087143	1829	1141	1957	1577	2170	1910
MTIF3	1.0693642	0.0967532	0.2902912	758	603	888	794	897	714
AC063948.1	0.4915254	-1.024662	0.1920255	21	16	22	1	11	17
NT5DC1	0.99563	-0.006318	0.732052	927	759	1060	765	1068	901
NT5DC4	1.5833333	0.662965	0.2075133	10	19	19	21	30	25
NT5DC2	1.2272727	0.2954559	0.7604031	5	12	5	3	5	19
NT5DC3	0.6371127	-0.650379	1.76E-05	1061	882	1091	577	696	660
GOLGA8UP	0.2941176	-1.765535	0.2829379	11	1	5	0	0	5
PIKFYVE	1.1381926	0.1867447	0.2513735	1153	738	1141	747	1177	1527
AC107918.4	0.7692308	-0.378512	0.818344	2	6	5	5	4	1
SSBP1	0.9270899	-0.109219	0.6929254	2873	2958	3619	2819	3146	2796
SSBP2	0.8756906	-0.191507	0.4768313	258	223	243	153	288	193
SSBP3	0.9925992	-0.010717	0.6857576	6426	7619	8250	7068	7935	7127

SSBP4	1.315187	0.395268	0.002479	1053	1185	1344	1708	1651	1352
NUP107	1.048856	0.0688166	0.3805886	2615	1866	2949	2093	2948	2752
TEX41	2.1111111	1.0780025	0.3130343	1	3	5	7	6	6
TEX43	0	#NUM!	0.824326	0	0	1	0	0	0
TEX45	5.3333333	2.4150375	0.2420193	0	0	3	0	16	0
TEX46	0.75	-0.415037	0.8674673	2	1	1	0	0	3
AC025040.2	0	#NUM!	0.6385096	0	2	0	0	0	0
FGFR1OP2	1.1706308	0.2272862	0.0777141	652	474	808	682	887	695
CSE1L-AS1	0.5	-1	0.8652929	0	0	2	0	0	1
AL049647.1	0	#NUM!	0.824326	0	0	1	0	0	0
TEX50	#DIV/0!	#DIV/0!	0.6411933	0	0	0	0	0	2
TEX52	0.625	-0.678072	0.7550635	3	0	5	0	5	0
AL445649.1	0	#NUM!	0.5387425	3	0	0	0	0	0
ASPHD1	1.5714286	0.6520767	0.5945613	1	1	5	3	4	4
ASPHD2	1.2040816	0.2679332	0.3740909	38	54	55	66	67	44
AL606491.1	0	#NUM!	0.2883218	0	0	7	0	0	0
SAR1A	1.2376701	0.3076269	0.0007879	3399	3561	4354	4282	5147	4574
SAR1B	1.054208	0.0761596	0.3353793	1843	1559	2040	1543	2279	1915
TEX22	0.6666667	-0.584963	0.5205959	12	8	19	5	7	14
TEX26	#DIV/0!	#DIV/0!	0.1171991	0	0	0	0	3	6
TEX29	1.4615385	0.5474878	0.6072937	2	4	7	8	11	0
LOH12CR2	1.0714286	0.0995357	0.8803061	15	21	6	16	20	9
LUARIS	0.75	-0.415037	0.7872044	3	3	6	0	4	5
TEX30	1.110998	0.1518562	0.2186486	310	312	360	348	405	338
NCAPH2	1.0801718	0.1112608	0.214559	2387	2764	3231	3279	3171	2604
TEX35	7	2.8073549	0.307745	0	0	1	0	6	1
TEX37	0.7460858	-0.422587	0.0232967	477	447	545	345	365	386
TEX38	0	#NUM!	0.6385096	0	2	0	0	0	0
AC025280.1	#DIV/0!	#DIV/0!	0.5509701	0	0	0	0	3	0
AC025280.2	2.1666667	1.1154772	0.4453942	4	0	2	2	6	5
NCAPG2	0.8975057	-0.156007	0.4287836	1561	1264	1585	1033	1485	1440
SNX12	0.8364992	-0.257564	0.0959061	3217	3894	4155	2704	3351	3369
AC111200.2	1.4615385	0.5474878	0.2164476	15	18	32	33	37	25
SNX13	1.0857684	0.1187164	0.3768315	1452	853	1391	949	1588	1476
SNX10	1.4410744	0.5271448	0.000259	2047	1352	2074	2061	3225	2601
SNX11	1.1315053	0.1782434	0.1392538	503	627	657	708	669	645
Z82244.2	0.1428571	-2.807355	0.3837182	0	0	7	1	0	0
AC013731.1	0.2	-2.321928	0.4595179	2	0	3	0	1	0
SLC18A2	1.3333333	0.4150375	0.8879992	1	2	0	0	0	4
TEX10	1.1030203	0.1414593	0.1075115	3915	3785	4385	3552	4957	4821
TEX11	1.0199005	0.0284284	0.7815879	80	54	67	75	57	73
TEX12	0.1111111	-3.169925	0.0566489	4	10	4	1	0	1
TEX14	1.3508772	0.4338965	0.2459629	42	20	52	36	64	54
PABPC5-AS1	#DIV/0!	#DIV/0!	0.8030358	0	0	0	1	0	0
TEX15	1.8695652	0.9027028	0.20339	8	4	11	13	17	13
TEX19	0.8072289	-0.30895	0.4826328	63	80	106	65	54	82
SLC18B1	1.3913043	0.476438	0.5429054	8	6	9	5	13	14
SNX32	0.875	-0.192645	0.9554319	0	5	3	3	0	4
SNX33	0.9962162	-0.005469	0.8106618	545	697	608	574	695	574
SNX30	1.0353208	0.0500778	0.5412167	2325	1939	2361	1646	2503	2710
RIDA	1.2305389	0.2992903	0.0500891	311	260	431	433	447	353
SNX29	0.6922661	-0.530601	0.0075357	556	625	668	300	444	536
SNX27	0.8765044	-0.190167	0.2381349	2506	2337	2801	1803	2475	2422
SNX25	2.0753341	1.0533436	5.75E-10	278	217	328	440	611	657

AL359881.1	0.625	-0.678072	0.7876127	5	1	2	5	0	0
HOMER2P1	1.1904762	0.2515388	0.7785486	15	3	3	11	11	3
HOMER2P2	0	#NUM!	0.824326	0	1	0	0	0	0
AL359881.3	#DIV/0!	#DIV/0!	0.6554062	0	0	0	0	2	0
RIC3	1.25	0.3219281	0.8500295	1	0	3	0	1	4
RIC1	1.0498747	0.0702171	0.5713964	1506	1103	1381	898	1625	1666
AC022509.2	0	#NUM!	0.5378194	0	2	1	0	0	0
AC022509.3	0.7777778	-0.36257	0.656093	25	6	14	7	20	8
SNX24	0.9172729	-0.124577	0.5888912	1886	1676	2349	1483	2021	1918
SNX21	0.7652174	-0.386058	0.2325975	109	105	131	89	97	78
AC074375.1	0	#NUM!	0.824326	0	1	0	0	0	0
SNX22	0.8074074	-0.308631	0.5420089	50	32	53	31	43	35
AP000753.3	#DIV/0!	#DIV/0!	0.2327526	0	0	0	0	0	7
SNX18	0.886947	-0.17308	0.2939746	5516	5059	5789	3816	5296	5402
SNX19	0.7453855	-0.423941	0.0041565	1843	1804	2150	1105	1603	1613
SNX16	1.0892857	0.1233824	0.5790326	163	112	117	100	148	179
SNX17	0.836053	-0.258334	0.1103743	3020	3559	3839	2812	3114	2784
SNX14	1.1329007	0.1800214	0.1018103	1238	954	1352	1069	1534	1412
SNX15	0.7931034	-0.334419	0.7768521	17	7	5	13	4	6
NCAPD2	0.8237683	-0.27969	0.0478869	11491	13205	14213	8774	11687	11591
NCAPD3	0.7067444	-0.500739	0.0006066	2147	1983	2557	1234	1686	1806
AC003986.2	3	1.5849625	0.4621988	0	0	2	3	0	3
AC003986.3	0	#NUM!	0.6385096	0	2	0	0	0	0
AC090246.1	0.5	-1	0.6450488	6	0	2	0	0	4
RNU6-77P	0	#NUM!	0.5387425	3	0	0	0	0	0
RIF1	1.1904422	0.2514976	0.1432358	2241	1074	2293	1571	2685	2420
AC007956.1	0.5	-1	0.8188512	2	0	0	0	1	0
CTNNBIP1	1.028013	0.0398585	0.5624621	438	549	548	595	523	460
PAX8-AS1	0.9496843	-0.07448	0.9474265	1558	1546	1964	1601	1796	1416
ARL8A	0.5580925	-0.841424	2.84E-06	998	1092	1370	701	694	536
ARL8B	0.9524674	-0.070258	0.8723228	4812	4107	5408	3719	5199	4728
AC018926.1	#DIV/0!	#DIV/0!	0.8030358	0	0	0	0	0	1
PKMYT1	0.8833901	-0.178877	0.4230864	1966	2241	2542	2079	1972	1911
AC018926.2	0.1875	-2.415037	0.1627539	0	13	3	0	2	1
AC018926.3	1.5	0.5849625	0.8265197	0	1	1	0	1	2
AC074135.1	0.754717	-0.405992	0.259563	90	72	103	42	80	78
AC104836.2	#DIV/0!	#DIV/0!	0.6317974	0	0	0	1	1	0
AMDHD1	0.5	-1	0.7204949	1	0	5	1	0	2
AMDHD2	0.9972549	-0.003966	0.7094599	729	883	938	902	917	724
AC114728.1	2	1	0.8319548	0	1	0	0	2	0
HNRNPH1P1	0.5652174	-0.823122	0.5423243	8	8	7	9	0	4
HNRNPH1P3	0.3333333	-1.584963	0.7307796	0	0	3	0	1	0
AL135901.1	#DIV/0!	#DIV/0!	0.6411933	0	0	0	0	0	2
AL451139.1	0.7575758	-0.400538	0.6797152	16	6	11	7	6	12
NOXA1	0.7419355	-0.430634	0.6545986	9	9	13	2	8	13
RHPN1-AS1	1	0	0.9271047	6	3	6	8	5	2
TRIM71	0.8638377	-0.211168	0.2540691	5810	5484	5862	3324	5468	6028
TRIM72	2	1	0.7153585	1	1	0	0	1	3
TRIM73	#DIV/0!	#DIV/0!	0.8030358	0	0	0	0	1	0
TRIM74	2.3333333	1.2223924	0.6038896	0	1	2	0	7	0
ECE1	1.4735809	0.5593263	8.43E-08	2171	2381	2583	3218	3756	3540
ECE2	0.986317	-0.019877	0.7971869	485	635	634	615	583	532
RN7SL608P	1	0	0.9714396	4	0	0	0	3	1
ARL5B	1.226617	0.2946849	0.0824426	1676	1044	1578	1122	2075	2075

ARL5C	0.1578947	-2.662965	0.0854414	4	7	8	2	1	0
TRIM69	1.1896552	0.2505435	0.6821668	19	27	12	12	20	37
ARL5A	1.2942752	0.3721444	0.0060352	1131	850	1006	1043	1444	1379
TRIM65	0.8629442	-0.212661	0.347152	442	482	652	397	501	462
TRIM66	1.0454545	0.0641303	0.51513	211	206	287	208	272	256
TRIM67	1.1935484	0.2552571	0.6475799	6	7	18	10	11	16
TRIM60	1.3524904	0.4356184	0.0020619	430	381	494	462	683	620
TRIM62	0.8867521	-0.173397	0.5358752	283	287	366	263	301	266
TRIM63	2.25	1.169925	0.4822206	0	3	1	5	3	1
ARL4C	1.1451947	0.195593	0.0259743	2724	2809	3145	2889	3542	3507
ARL4D	0.8937198	-0.162105	0.6440687	357	555	537	475	437	383
PTP4A1P1	#DIV/0!	#DIV/0!	0.6099294	0	0	0	2	0	0
ARL4A	1.0879346	0.1215918	0.423984	145	150	194	151	217	164
PTP4A1P7	#DIV/0!	#DIV/0!	0.8030358	0	0	0	0	0	1
TRIM58	0.4710921	-1.085919	0.0001031	297	322	315	70	165	205
TRIM59	1.1430556	0.1928955	0.1104995	1090	748	1042	989	1253	1050
TRIM54	1	0	0.9249165	2	0	2	4	0	0
TRIM55	1.0520095	0.0731477	0.6093583	135	138	150	139	184	122
MIR3135A	1.6666667	0.7369656	0.7416804	0	0	3	0	0	5
TRIM56	1.023028	0.0328456	0.4690748	1844	1789	2186	1660	2093	2200
AL034347.1	0	#NUM!	0.824326	0	1	0	0	0	0
TRIM52	0.6616052	-0.595958	0.0229932	353	199	370	183	192	235
AC131391.1	#DIV/0!	#DIV/0!	0.5371397	0	0	0	0	0	3
MIR219A1	#DIV/0!	#DIV/0!	0.8030358	0	0	0	0	0	1
MIR219A2	0	#NUM!	0.5781912	0	0	3	0	0	0
MIR219A2	0	#NUM!	0.5781912	0	0	3	0	0	0
AL353764.1	0.75	-0.415037	0.801137	4	2	6	3	4	2
TRIM46	1.1428571	0.1926451	0.8788578	2	2	3	1	0	7
TRIM47	0	#NUM!	0.1195062	0	6	3	0	0	0
TRIM48	#DIV/0!	#DIV/0!	0.4737294	0	0	0	0	4	0
TRIM49	0.5454545	-0.874469	0.6773648	1	0	10	0	5	1
RILP	0.7018182	-0.510831	0.1146523	186	214	150	162	112	112
TRIM43	2.4814815	1.3112017	0.0564171	5	15	7	15	21	31
TRIM44	0.9103448	-0.135515	0.5070513	1211	1125	1289	889	1193	1218
TRIM45	0.828479	-0.271463	0.3937748	193	155	270	170	173	169
RN7SL605P	0	#NUM!	0.6604275	0	1	1	0	0	0
TRIM41	0.8599178	-0.217729	0.1860825	1596	1742	2016	1420	1660	1524
CABLES1	1.2914573	0.3689999	0.0576703	114	128	156	153	179	182
KIAA0232	0.9367463	-0.09427	0.7730036	1618	1176	1601	998	1419	1700
RNU7-10P	0	#NUM!	0.6840298	0	0	2	0	0	0
CABLES2	0.8338323	-0.262171	0.4690456	195	228	245	236	160	161
DBNDD1	1.2420682	0.3127443	0.011865	808	866	879	1077	1019	1075
DBNDD2	0.4193548	-1.253757	0.2976151	1	8	22	4	5	4
TRIM39	0.9229935	-0.115608	0.69623	286	343	293	247	334	270
TRIM35	0.726965	-0.460042	0.0089124	765	916	978	492	689	752
TRIM36	0.6389776	-0.646163	0.0693011	136	67	110	49	77	74
TRIM37	0.8980253	-0.155172	0.4637776	1226	939	1228	772	1184	1091
TRIM38	0.8786095	-0.186706	0.3424065	1169	995	1403	841	1142	1151
TRIM31	1.7333333	0.7935491	0.4278357	4	8	3	23	2	1
TRIM32	0.9280576	-0.107714	0.6718969	923	1052	944	827	977	905
TRIM33	1.0880199	0.1217049	0.3594728	2918	1803	2925	1861	3220	3238
TRIM34	0.6688742	-0.580193	0.2099109	51	51	49	31	25	45
RN7SL604P	#DIV/0!	#DIV/0!	0.5371397	0	0	0	0	0	3
ECI1	0.891568	-0.165583	0.5537254	1895	2442	2506	2271	2020	1810

RFESD	0.9253731	-0.111893	0.8604344	43	10	14	11	32	19
ECI2	0.3766816	-1.408582	0.0005278	65	68	90	33	28	23
MIR1302-3	0	#NUM!	0.4374838	0	4	0	0	0	0
AC010745.2	1.6206897	0.6966079	0.2913076	12	8	9	14	18	15
AC010745.3	0.8888889	-0.169925	0.9135627	8	1	0	3	5	0
AC010745.4	0.84375	-0.245112	0.7448335	20	25	19	21	16	17
RINL	1.138009	0.186512	0.2841378	121	148	173	173	185	145
RNU7-18P	7	2.8073549	0.2891467	1	0	0	0	4	3
TRIM28	0.8816644	-0.181698	0.4016289	13139	16437	17840	14529	14387	12889
TRIM29	0.4962406	-1.010888	2.49E-05	232	299	267	138	136	122
TRIM24	1.1802694	0.2391162	0.0937254	937	570	1017	783	1117	1079
TRIM25	#DIV/0!	#DIV/0!	6.96E-05	0	0	0	13	6	19
ARL16	0.8906667	-0.167042	0.641454	428	487	585	509	489	338
TRIM26	0.8369653	-0.25676	0.1314834	1380	1581	1995	1211	1568	1369
TRIM27	0.8541835	-0.227382	0.1262884	3426	3702	4023	2924	3399	3202
TRIM21	1.1315878	0.1783485	0.0729506	866	996	1117	1009	1218	1144
TRIM22	0.8617021	-0.214739	0.6900034	72	39	77	51	69	42
TRIM23	0.9715762	-0.041601	0.9666502	291	156	327	153	275	324
ARL10	1.4615385	0.5474878	0.6870963	3	8	2	2	15	2
ARL11	0.9079118	-0.139376	0.638453	467	523	552	482	487	431
ARL15	0.9454128	-0.080984	0.8271425	781	626	773	557	745	759
ECH1	0.8292126	-0.270186	0.2392509	729	890	1010	794	767	619
PSMD6-AS2	1.2666667	0.3410369	0.4208255	25	20	30	22	30	43
TRIM17	0.6666667	-0.584963	0.2336044	33	48	78	32	33	41
AC004825.2	1.2222222	0.2895066	0.8068822	6	2	1	5	1	5
TRIM13	1.1429681	0.1927852	0.1093098	393	361	533	481	531	459
TRIM14	1.2117647	0.2771096	0.4636129	15	31	39	33	35	35
RIN3	1.2092852	0.2741545	0.0097484	1181	1335	1555	1539	1847	1537
TRIM16	0.6697848	-0.57823	0.0091171	288	384	397	242	262	212
RIN1	0.7173913	-0.479168	0.3961792	30	27	35	20	21	25
RIN2	0.9232271	-0.115243	0.6706894	510	454	573	369	566	484
TRIM11	0.9207154	-0.119173	0.671136	1083	1223	1440	1098	1310	1041
SYNGR1	0.4285714	-1.222392	0.6336049	2	0	5	0	0	3
SYNGR2	0.8298012	-0.269162	0.1949326	1260	1684	1633	1340	1273	1185
SARAF	1.0323917	0.0459905	0.3843726	1623	1735	1952	1697	1966	1819
AC036214.1	3	1.5849625	0.512377	2	0	0	6	0	0
AC036214.2	1.4615385	0.5474878	0.5630191	0	4	9	10	6	3
AGPAT4-IT1	1.1481481	0.1993088	0.7158873	37	22	22	11	35	47
POLA1	1.0166367	0.0238042	0.7206142	800	600	824	517	942	802
POLA2	0.7923261	-0.335834	0.0393431	1789	2150	2316	1628	1760	1568
PFN1P11	0.1666667	-2.584963	0.196004	6	2	4	2	0	0
HERPUD2	1.0411413	0.0581659	0.4521226	878	978	1158	824	1071	1243
ECM1	1.3158981	0.3960478	0.0005101	844	927	1135	1234	1351	1239
ECM2	1.8958333	0.9228321	0.070681	11	9	28	35	26	30
RN7SL600P	1	0	0.9909781	4	3	1	0	0	8
MTMR9LP	1.3125	0.3923174	0.7611071	4	12	0	5	8	8
HERPUD1	1.4434772	0.5295483	2.66E-07	2467	2691	3113	3752	4317	3870
AL157831.2	0	#NUM!	0.824326	1	0	0	0	0	0
ATPAF2	0.7575962	-0.400499	0.0680544	417	485	579	403	392	327
ATPAF1	0.8842829	-0.17742	0.334817	1545	1671	1874	1406	1537	1558
AC009623.1	#DIV/0!	#DIV/0!	0.8030358	0	0	0	0	0	1
SEC14L1P1	#DIV/0!	#DIV/0!	0.5371397	0	0	0	0	0	3
MIR5697	3	1.5849625	0.4987241	2	0	0	3	0	3
MIR5695	0	#NUM!	0.4217812	0	0	5	0	0	0

AL354989.1	0.3125	-1.678072	0.1356908	12	10	10	1	9	0
CYP8B1	0.7442681	-0.426106	0.1175298	180	201	186	95	147	180
YIF1A	0.9522313	-0.070616	0.9657985	1285	1752	1736	1615	1644	1286
YIF1B	1.0058531	0.0084196	0.6063011	976	1125	1316	1064	1271	1102
RIT1	0.8252841	-0.277037	0.1656203	416	468	524	324	413	425
RIT2	0	#NUM!	0.824326	1	0	0	0	0	0
MIR5685	#DIV/0!	#DIV/0!	0.8030358	0	0	0	0	0	1
UBE2Q1-AS1	0.3333333	-1.584963	0.3175294	5	1	12	0	1	5
AC008056.1	#DIV/0!	#DIV/0!	0.5371397	0	0	0	0	0	3
AC008056.2	#DIV/0!	#DIV/0!	0.6411933	0	0	0	0	0	2
NUP188	0.8955589	-0.15914	0.3563576	4096	4820	4977	3452	4590	4400
AC069282.1	0.775	-0.367732	0.5492908	41	22	57	30	27	36
AC027104.1	#DIV/0!	#DIV/0!	0.2256364	0	0	0	3	3	0
AC007560.1	10	3.3219281	0.2154646	0	1	0	0	10	0
POLG2	1.0220538	0.0314711	0.5681173	513	393	545	454	502	527
BHLHB9	0.9905063	-0.013762	0.8462105	561	431	588	389	589	587
GLYAT	0.2045455	-2.289507	0.0156477	10	18	16	2	5	2
NUP160	0.8955125	-0.159215	0.4207943	3738	2941	3839	2269	3596	3554
AP005062.1	0.5	-1	0.812391	0	2	0	0	1	0
AL031705.1	0.8	-0.321928	0.8654554	2	4	4	1	0	7
ECT2	1.1023853	0.1406286	0.3302396	2230	1165	2055	1452	2337	2219
NUP155	0.8834865	-0.17872	0.3279675	6622	5662	6864	4084	6209	6624
NUP153	0.9451285	-0.081418	0.77252	6348	5183	6256	3902	6226	6683
AC016769.4	0	#NUM!	0.824326	0	1	0	0	0	0
AL050338.1	0	#NUM!	0.2324194	3	3	0	0	0	0
AL050338.2	2.4545455	1.2954559	0.0266718	5	9	19	25	31	25
AC016769.2	0.7878788	-0.343954	0.6905888	13	13	7	8	9	9
MKRN1	0.8695465	-0.201665	0.2257133	2053	2417	2498	1801	2185	2073
MKRN2	0.8513061	-0.23225	0.2408173	897	835	1254	683	944	915
MKRN3	0.9653795	-0.050832	0.9670746	233	203	315	186	271	268
SWSAP1	0.9506173	-0.073063	0.9779121	26	14	41	23	27	27
TNFAIP8	1.0564611	0.0792397	0.3048927	1411	1249	1573	1215	1670	1587
TNFAIP6	3.972973	1.990219	5.83E-05	7	13	17	40	65	42
TNFAIP2	2.4484233	1.291853	1.78E-23	556	625	690	1455	1683	1443
TNFAIP3	1.2503809	0.3223677	0.0166258	642	643	684	639	873	950
TNFAIP1	0.955327	-0.065933	0.9447742	1866	1852	2214	1739	2047	1881
NUP133	0.9656377	-0.050446	0.9967034	2928	2283	3374	2115	3287	2888
SHROOM2	0.6871616	-0.541279	0.0021207	718	953	915	480	643	654
SHROOM3	0.8628429	-0.21283	0.4403968	228	276	298	163	237	292
SHROOM1	0.875448	-0.191907	0.5263372	324	336	456	330	388	259
SYNGR3	1.2068966	0.271302	0.2668948	107	56	98	115	96	104
SYNGR4	2	1	0.3574126	1	5	4	8	9	3
SHROOM4	0.9799163	-0.02927	0.9027118	324	399	472	288	387	496
OXLD1	0.8945687	-0.160736	0.656722	348	414	490	415	410	295
CNN2P3	#DIV/0!	#DIV/0!	0.8030358	0	0	0	0	1	0
CNN2P9	1	0	0.9888245	1	1	1	1	2	0
SUMO1P3	3	1.5849625	0.3067401	1	1	2	3	8	1
SUMO1P4	#DIV/0!	#DIV/0!	0.8030358	0	0	0	0	1	0
AL009174.1	1.754717	0.8112384	0.1003718	13	10	30	40	28	25
POLD3	0.8602915	-0.217103	0.1869374	1929	1629	2068	1318	1805	1717
POLD4	1.2586207	0.3318436	0.543613	12	33	13	27	25	21
AC087742.2	3	1.5849625	0.4494171	0	1	1	2	3	1
LRP4-AS1	0.9375	-0.093109	0.9857599	5	1	10	6	9	0
POLD1	0.9250914	-0.112332	0.7034527	2359	2941	3177	2543	2678	2621

POLD2	0.9039753	-0.145645	0.5812847	2693	3153	3537	2985	2821	2676
SUMO1P1	0.5797101	-0.786596	0.3404699	26	9	34	16	5	19
AL138689.1	0.5	-1	0.6397162	3	1	2	0	3	0
AP006333.2	#DIV/0!	#DIV/0!	0.6554062	0	0	0	0	2	0
POLE2	1.1419822	0.1915401	0.1024106	593	465	738	594	779	678
POLE3	1.0202189	0.0288788	0.4505431	2749	2844	3359	2661	3416	3056
SARDH	1.5862069	0.665581	0.3234629	10	9	10	10	15	21
WIPF1	1.4266304	0.5126117	0.020981	119	110	139	124	214	187
WIPF2	0.8556005	-0.224991	0.2288955	2231	2555	2624	1431	2410	2499
KIAA0141	0.7968672	-0.327589	0.0498263	1280	1214	1528	1016	1247	942
FAM86FP	0.8214286	-0.283793	0.7340505	17	13	26	14	20	12
EVA1A	0.2777778	-1.847997	0.2904838	6	0	12	3	2	0
EVA1B	0.9165402	-0.12573	0.8762729	160	205	294	229	209	166
ZMIZ1	1.1315264	0.1782702	0.1174574	1070	1217	1218	1046	1452	1468
ZMIZ2	0.7858491	-0.347676	0.0507137	650	698	772	544	570	552
PXDC1	0.9821429	-0.025995	0.7559536	2167	2563	2886	2737	2511	2232
NUP214	0.8894798	-0.168966	0.3287282	2669	2950	3031	2258	2666	2770
NUP210	0.7484389	-0.418044	0.0051044	4221	5137	5215	2876	3927	4104
ARMC9	1.0907216	0.125283	0.2837985	302	303	365	351	388	319
RNU6-80P	0	#NUM!	0.1933766	0	5	2	0	0	0
ARMC7	0.8406489	-0.250425	0.3314364	317	347	384	308	334	239
ARMC8	1.0672659	0.0939196	0.2554643	1190	941	1318	1073	1343	1265
FAM86EP	0.8372093	-0.25634	0.4451181	121	129	137	112	107	105
ARMC1	1.0266623	0.0379617	0.5260611	1105	783	1150	903	1172	1044
ARMC2	0.8709677	-0.199309	0.5837017	116	114	111	99	103	95
ARMC5	0.9320388	-0.101538	0.8413848	213	232	173	221	199	156
ARMC6	0.8044963	-0.313842	0.1113699	966	1208	1340	988	961	878
ARMC3	2.4680851	1.3033921	0.0069182	10	16	21	35	36	45
ARMC4	0	#NUM!	0.6436235	2	0	0	0	0	0
LINC-ROR	0	#NUM!	0.824326	0	0	1	0	0	0
NUP205	0.9117527	-0.133285	0.5347345	2597	2102	2678	1620	2526	2580
AC079915.1	1	0	0.9803584	3	2	0	1	3	1
ARPP19	1.0341004	0.0483762	0.4920465	5402	4524	5675	4012	6005	6116
FAM86HP	0.6116505	-0.709221	0.1881507	32	30	41	21	19	23
AL049648.1	1	0	0.9922023	2	0	0	0	0	2
FDFT1	0.6821958	-0.551742	2.45E-05	4209	4863	5374	3049	3607	3199
FAM86GP	0.8893281	-0.169212	0.5393937	238	261	260	161	241	273
REC114	0.8888889	-0.169925	0.9686645	2	1	6	0	0	8
STT3A-AS1	0.2307692	-2.115477	0.249931	10	1	2	0	0	3
FAM86JP	0.8926174	-0.163886	0.7078617	99	85	114	83	97	86
AL159153.1	2	1	0.7760147	0	2	0	0	4	0
AL359880.1	0	#NUM!	0.1227606	10	0	0	0	0	0
DPP9-AS1	#DIV/0!	#DIV/0!	0.2905913	0	0	0	2	0	3
CDYL2	0.8046875	-0.313499	0.1715431	342	281	401	200	313	311
AL358074.1	0	#NUM!	0.6385096	0	2	0	0	0	0
FAM86LP	0.6634146	-0.592017	0.1585032	84	63	58	21	52	63
AL589674.1	1.75	0.8073549	0.679548	1	2	1	0	1	6
AL355336.1	0.2	-2.321928	0.408833	2	1	2	0	0	1
LMNB1-DT	0.7142857	-0.485427	0.793349	0	10	4	7	1	2
AC006483.2	0.5714286	-0.807355	0.7031161	0	4	3	0	4	0
Z82243.1	1.0862069	0.1192989	0.751656	14	18	26	16	26	21
SLC17A9	2.1666667	1.1154772	0.3231707	6	3	3	2	3	21
COX6CP1	0.25	-2	0.547139	0	4	0	0	1	0
SLC17A5	0.607667	-0.718647	1.03E-05	1089	1132	1118	508	754	767

SLC17A7	1.1481481	0.1993088	0.7319682	12	6	9	13	7	11
SLC17A8	#DIV/0!	#DIV/0!	0.6554062	0	0	0	0	2	0
SLC17A4	1.3225806	0.4033557	0.54531	5	6	20	16	22	3
FAM86KP	5	2.3219281	0.4840007	1	0	0	0	5	0
PBDC1	1.0691538	0.0964694	0.3265463	704	563	931	743	945	662
RNU6-82P	0	#NUM!	0.824326	0	0	1	0	0	0
PCYOX1L	0.7375635	-0.439161	0.0116982	636	559	775	454	514	485
DEFB109F	0	#NUM!	0.824326	0	0	1	0	0	0
DEFB109D	0.5	-1	0.8188512	2	0	0	0	1	0
IPO7	1.0069457	0.0099859	0.7435693	10381	6682	10292	6418	10496	10631
AC074134.1	0	#NUM!	0.6385096	0	2	0	0	0	0
IPO4	6	2.5849625	0.3276733	0	0	1	2	0	4
EDC3	0.8468518	-0.239819	0.159986	1104	1277	1256	876	1194	1010
IPO5	1.0082185	0.0118083	0.6296399	10898	8990	11748	8304	11974	11618
AC005162.2	0.6666667	-0.584963	0.7172569	2	7	0	0	6	0
EDC4	0.7506073	-0.41387	0.0082104	2505	2919	3221	1613	2399	2477
AC005162.3	0.5769231	-0.793549	0.5025742	10	6	10	9	5	1
IPO8	0.8842975	-0.177396	0.406487	2272	1857	2405	1309	2072	2397
AC005162.1	1	0	0.9904495	3	0	0	0	0	3
IPO9	0.856457	-0.223547	0.1070352	17117	16882	19114	12486	16613	16390
RNU6-88P	0.9565217	-0.06413	0.9876741	3	7	13	4	7	11
CCDC58P1	0	#NUM!	0.3482063	0	0	6	0	0	0
AL603756.1	7.5	2.9068906	0.0181225	3	0	1	16	9	5
SWAP70	1.1641437	0.2192692	0.0560502	3920	3156	3890	3249	4621	4896
CCDC58P3	1.6666667	0.7369656	0.7424298	1	0	2	0	4	1
IPMK	1.1538462	0.2064509	0.2289905	823	525	680	521	896	923
DYRK1A	1.0853971	0.118223	0.3601684	1713	1299	1672	1129	2012	1943
DYRK1B	0.8877551	-0.171766	0.7554616	74	100	120	99	91	71
AL033519.2	0.6111111	-0.710493	0.5234317	2	15	19	11	6	5
AL033519.3	1.2579324	0.3310544	0.0210048	287	285	405	400	447	382
RNU7-20P	2	1	0.807847	1	0	0	0	1	1
EDAR	1.1764706	0.2344653	0.852128	8	5	4	0	4	16
AC011479.2	0.3333333	-1.584963	0.7307796	0	0	3	0	1	0
IPPK	0.9122449	-0.132507	0.593081	557	650	753	491	687	610
AC011479.3	0.8333333	-0.263034	0.9548554	6	0	0	5	0	0
RNU6-87P	0	#NUM!	0.6436235	2	0	0	0	0	0
GLT8D1	0.9722381	-0.040618	0.866881	1253	949	1292	1124	1147	1126
GLT8D2	1.1595092	0.2135143	0.4903472	70	46	47	51	68	70
AC009133.1	1.1301775	0.1765494	0.6053724	43	76	50	41	71	79
AC009133.3	#DIV/0!	#DIV/0!	0.2546293	0	0	0	1	5	0
PXDNL	1.6666667	0.7369656	0.7274911	2	0	1	2	3	0
STK24-AS1	0.7142857	-0.485427	0.6603379	9	7	5	4	3	8
C18orf8	1.0749747	0.1043027	0.3373609	281	329	377	334	370	357
AL606490.2	0	#NUM!	0.824326	1	0	0	0	0	0
AC022748.2	0	#NUM!	0.5781912	0	0	3	0	0	0
KIAA0100	0.7636181	-0.389077	0.0162529	2976	3499	3475	1951	2619	3028
AC002429.2	0	#NUM!	0.4521054	1	0	3	0	0	0
TNPO1	1.0511169	0.0719231	0.5294023	9132	6018	8443	5481	9547	9771
TNPO2	0.8752183	-0.192285	0.2456387	1582	1622	1949	1277	1640	1593
TNPO3	0.9689832	-0.045456	0.9748995	4623	3995	5052	3544	4908	4794
ILKAP	0.9726321	-0.040034	0.8620378	1103	1197	1427	1160	1306	1159
EDF1	1.279384	0.3554493	0.0033383	7097	8604	9687	11862	11055	9564
WIPI1	1.0759369	0.1055934	0.2618622	581	656	791	663	841	678
WIPI2	1.044536	0.0628622	0.2939592	3607	4153	4859	4176	4785	4220

RAB6C-AS1	1.2116788	0.2770073	0.4025812	46	35	56	48	80	38
AL133255.1	2	1	0.7433853	2	0	0	0	3	1
DDC-AS1	0	#NUM!	0.6436235	2	0	0	0	0	0
AC134772.2	0.8333333	-0.263034	0.8267046	17	9	22	17	7	16
AC134772.1	0.25	-2	0.4410808	0	3	5	2	0	0
AC109826.1	1.3835616	0.4683869	0.3294084	40	12	21	34	35	32
AC073150.1	18	4.169925	0.0244546	1	0	0	15	1	2
AL606490.8	0	#NUM!	0.355562	5	0	0	0	0	0
AC020687.1	#DIV/0!	#DIV/0!	0.1506188	0	0	0	8	0	0
AL162730.1	#DIV/0!	#DIV/0!	0.8030358	0	0	0	1	0	0
HSBP1L1	0.8100233	-0.303965	0.2146495	267	234	357	190	248	257
AC091564.1	0.7142857	-0.485427	0.8324421	4	0	3	2	3	0
RN7SL614P	0	#NUM!	0.5387425	3	0	0	0	0	0
AC096759.2	#DIV/0!	#DIV/0!	0.8030358	0	0	0	0	0	1
STXBP3	1.0626841	0.0877127	0.4527069	920	496	961	669	979	878
STXBP4	0.9961089	-0.005625	0.8581179	177	135	202	120	206	186
STXBP1	1.1131204	0.1546096	0.0691971	2279	2512	2785	2477	3028	2928
STXBP2	1.0240659	0.0343085	0.6084004	430	585	564	678	517	422
CDRT15P1	1.5192308	0.603341	0.2109344	13	15	24	29	21	29
STXBP5	0.9500924	-0.07386	0.9580518	389	265	428	305	357	366
STXBP6	0.6519435	-0.617181	0.0204677	170	163	233	129	125	115
PRR13P5	0.9027778	-0.147557	0.9005426	43	49	52	64	32	34
PRR13P2	#DIV/0!	#DIV/0!	0.8030358	0	0	0	0	1	0
RNU6-703P	0	#NUM!	0.824326	0	0	1	0	0	0
B3GALNT1P1	0.5	-1	0.6899408	0	3	3	0	0	3
TMEM202-AS	0.7407407	-0.432959	0.6895384	2	17	8	5	4	11
EDN1	0.1666667	-2.584963	0.3802332	0	2	4	0	0	1
EDN2	1.0909091	0.1255309	0.7764369	5	10	18	14	16	6
EDN3	0	#NUM!	0.5200113	2	1	0	0	0	0
PPHLN1	1.0301757	0.0428904	0.4514376	1824	1484	1928	1537	2036	1821
CYP7B1	1.1014493	0.1394031	0.6769275	24	12	33	24	19	33
HSBP1P2	#DIV/0!	#DIV/0!	0.8030358	0	0	0	0	1	0
VWA8	0.816787	-0.291968	0.1321696	775	616	825	459	651	700
BDNF-AS	0.1111111	-3.169925	0.1599366	3	5	1	0	0	1
VWA1	1.1495712	0.2010958	0.0732412	4204	5409	5196	5985	5864	5175
VWA2	0.6	-0.736966	0.7418567	0	4	1	0	3	0
VWA7	0.8823529	-0.180572	0.9393184	9	0	8	2	1	12
RHGAP31-AS	0	#NUM!	0.5337954	0	3	0	0	0	0
RN7SL610P	0	#NUM!	0.5781912	0	0	3	0	0	0
VWCE	0.5212766	-0.939879	0.1266037	26	24	44	20	19	10
CYP7A1	0	#NUM!	0.824326	1	0	0	0	0	0
AL157832.1	2.0714286	1.0506261	0.2976157	0	8	6	8	13	8
AL157832.3	#DIV/0!	#DIV/0!	0.2545643	0	0	0	0	4	2
RNU6-705P	1.173913	0.2313255	0.7127446	9	4	10	13	10	4
AL607033.1	#DIV/0!	#DIV/0!	0.3096126	0	0	0	5	0	0
TRAM1L1	#DIV/0!	#DIV/0!	0.263214	0	0	0	0	5	1
SNX25P1	1.1666667	0.2223924	0.8708862	1	0	5	0	4	3
MPHOSPH9	1.1633987	0.2183456	0.2111513	599	301	630	431	685	664
TRIML2	0.7549407	-0.405565	0.1384823	168	135	203	108	142	132
AL451137.1	0	#NUM!	0.1796811	2	5	0	0	0	0
RNU6-704P	#DIV/0!	#DIV/0!	0.8030358	0	0	0	0	0	1
F2RL1	0.950335	-0.073492	0.8234797	809	860	868	608	889	914
TRIML1	0.904271	-0.145173	0.7393648	209	203	267	222	235	157
MPHOSPH8	1.0285253	0.0405773	0.549805	2014	1239	2321	1516	2218	1999

F2RL2	0	#NUM!	0.4217812	0	0	5	0	0	0
F2RL3	0	#NUM!	0.5387425	3	0	0	0	0	0
MPHOSPH6	1.1809101	0.2398991	0.0467131	569	497	736	694	793	641
AC097347.1	#DIV/0!	#DIV/0!	0.8030358	0	0	0	0	1	0
FOLH1B	#DIV/0!	#DIV/0!	0.8030358	0	0	0	1	0	0
AC233992.3	#DIV/0!	#DIV/0!	0.3668985	0	0	0	3	0	1
AC022113.1	0.5625	-0.830075	0.530269	7	3	6	0	6	3
AC022113.2	0	#NUM!	0.4460243	4	0	0	0	0	0
AC013733.2	#DIV/0!	#DIV/0!	0.8030358	0	0	0	0	0	1
AC116634.1	0	#NUM!	0.824326	0	0	1	0	0	0
AL035425.1	2	1	0.3435984	11	0	3	9	10	9
AC012557.2	#DIV/0!	#DIV/0!	0.6411933	0	0	0	0	0	2
RALBP1	13	3.7004397	0.0297406	2	0	0	2	22	2
AL035425.4	2	1	0.8186965	0	0	1	0	0	2
AC012557.1	0.8333333	-0.263034	0.8374918	10	9	5	11	5	4
AC129510.1	1.5333333	0.6166714	0.4912062	4	6	5	10	9	4
AL035425.3	0.925	-0.112475	0.99196	10	7	23	15	12	10
AC051618.1	0.3333333	-1.584963	0.7307796	0	0	3	0	1	0
VWDE	1.2390074	0.3091848	0.0413584	872	566	859	705	1069	1072
AC108112.1	0.950495	-0.073249	0.9873765	34	21	46	23	35	38
AC008055.1	0	#NUM!	0.824326	0	0	1	0	0	0
MGEA5	1.2122291	0.2776624	0.0211431	4775	3486	4937	4085	5927	5987
AL356414.1	0	#NUM!	0.5378194	0	2	1	0	0	0
Z99943.1	0.4254144	-1.233059	0.0060165	47	73	61	15	39	23
Z99943.2	0.8125	-0.29956	0.8098077	9	4	3	2	2	9
FAM86C1	0.9484346	-0.07638	0.863101	167	191	185	125	200	190
NDUFA9P1	#DIV/0!	#DIV/0!	0.8030358	0	0	0	0	1	0
FAM86B2	0.8734177	-0.195256	0.7678386	20	27	32	12	30	27
AL731556.1	0.6666667	-0.584963	0.8519305	0	2	4	3	1	0
IQCE	0.8948035	-0.160357	0.5873884	225	254	310	202	279	225
AL022393.1	1.4848485	0.5703157	0.3567034	4	4	25	21	14	14
FAM86B1	0.8181818	-0.289507	0.4669566	78	70	72	55	56	69
IQCC	1.0530504	0.0745745	0.603539	144	98	135	125	146	126
IQCD	1.2465753	0.3179701	0.4497915	12	28	33	30	34	27
IQCG	0.9384164	-0.0917	0.9000532	113	102	126	101	112	107
IQCH	0.8602151	-0.217231	0.8229927	33	14	46	27	21	32
IQCK	0.8446602	-0.243557	0.6885194	23	34	46	9	51	27
AL139132.1	0.4	-1.321928	0.6035926	4	0	1	0	2	0
AC000123.1	0.9375	-0.093109	0.9848317	23	21	36	33	19	23
XPOTP1	1.25	0.3219281	0.7339614	4	1	7	3	5	7
FAM86DP	0.7518892	-0.411408	0.0673009	249	259	286	158	211	228
AC000123.2	1.8	0.8479969	0.5479711	5	0	5	0	11	7
AC000123.3	1.2666667	0.3410369	0.5169394	46	34	25	19	28	86
ERICH5	#DIV/0!	#DIV/0!	0.5509701	0	0	0	0	3	0
ERICH6	0.3333333	-1.584963	0.6587914	2	0	1	1	0	0
ERICH1	1.060946	0.0853513	0.2527444	1026	990	1282	1012	1309	1178
ERICH2	0.0909091	-3.459432	0.1235461	2	2	7	0	0	1
ERICH4	#DIV/0!	#DIV/0!	0.8030358	0	0	0	0	1	0
S100PBP	1.0451389	0.0636947	0.4826679	905	939	1036	736	1151	1123
AC138207.5	0.625	-0.678072	0.5149716	5	11	8	4	8	3
AC138207.6	1.35	0.4329594	0.4780818	12	19	9	17	17	20
AC023825.2	#DIV/0!	#DIV/0!	0.8030358	0	0	0	0	0	1
AC138207.7	0.7	-0.514573	0.670249	4	8	18	5	3	13
AC138207.8	0.952381	-0.070389	0.9452466	9	1	11	11	5	4

AC138207.1	3.3333333	1.7369656	0.3392426	3	0	0	6	1	3
AC138207.2	3.3076923	1.725825	0.0378002	3	4	6	23	12	8
AP001094.2	3.6	1.8479969	0.1651534	1	0	4	10	1	7
AC138207.4	1.0990338	0.1362358	0.4322305	137	101	176	115	162	178
MTMR1	0.7588161	-0.398178	0.0065462	1916	2120	2316	1260	1853	1707
MTMR2	1.0348572	0.0494318	0.5213882	2084	1484	2141	1491	2291	2126
MTMR3	0.7692308	-0.378512	0.3538699	54	87	93	49	86	45
RHOT1P2	6	2.5849625	0.282881	1	0	0	4	1	1
RHOT1P3	1.1666667	0.2223924	0.8187319	0	1	5	3	1	3
MTMR8	0.2	-2.321928	0.2932187	0	4	6	1	0	1
MTMR9	0.8468635	-0.239799	0.2778161	354	334	396	233	342	343
RHOT1P1	2	1	0.6617645	0	2	1	0	4	2
MTMR4	0.7297851	-0.454456	0.0051793	1868	1986	2008	1025	1605	1648
MTMR6	1.0526782	0.0740644	0.554679	897	544	818	545	884	949
MTMR7	0.5	-1	0.1310581	21	21	48	5	26	14
AP000755.1	#DIV/0!	#DIV/0!	0.6554062	0	0	0	0	2	0
NAPEPLD	1.1504178	0.2021579	0.3823244	142	97	120	96	172	145
SASS6	1.0544662	0.0765129	0.6173634	572	238	567	316	570	566
AC003988.1	6	2.5849625	0.3098729	0	0	1	1	1	4
AL096677.1	0.6363636	-0.652077	0.6248221	9	2	11	6	2	6
MED28P3	0.1666667	-2.584963	0.4003623	1	0	5	0	0	1
HMGB3P10	0	#NUM!	0.6436235	2	0	0	0	0	0
LMF1-AS1	4	2	0.3599905	2	0	0	4	0	4
TRIOBP	0.8366888	-0.257237	0.1052562	3064	3505	4110	2842	3115	2978
EEA1	1.1697692	0.2262239	0.1518082	1584	900	1545	1092	1842	1779
RAB11B-AS1	0.6285714	-0.669851	0.4495277	15	12	8	8	5	9
AC090004.1	0.6666667	-0.584963	0.5846102	9	6	9	3	4	9
TRPC7-AS1	#DIV/0!	#DIV/0!	0.8030358	0	0	0	0	1	0
OPALIN	0.6666667	-0.584963	0.8015827	6	0	0	0	0	4
VWA5A	0.8519004	-0.231243	0.5215927	216	222	325	252	207	191
AC092641.1	1.75	0.8073549	0.6539736	1	2	1	3	4	0
P2RY12	0	#NUM!	0.6410557	1	1	0	0	0	0
P2RY13	#DIV/0!	#DIV/0!	0.3031768	0	0	0	2	3	0
KCNJ6-AS1	0	#NUM!	0.6436235	2	0	0	0	0	0
P2RY11	0.5784615	-0.789707	0.0158711	101	111	113	70	64	54
SLC16A1	0.8860843	-0.174484	0.2795543	13770	12107	15048	9640	13331	13292
P2RY14	0.6	-0.736966	0.7310277	1	2	2	0	2	1
VWA3B	1.2894737	0.3667823	0.541084	7	5	26	8	19	22
AL358075.1	#DIV/0!	#DIV/0!	0.6554062	0	0	0	0	2	0
AL358075.2	0.4615385	-1.115477	0.5170309	2	10	1	5	0	1
ERI3-IT1	0.8	-0.321928	0.8947424	0	2	3	0	4	0
RNU6-53P	1	0	0.9644635	3	0	0	3	0	0
AP001486.2	1.7826087	0.83399	0.2979771	4	3	16	8	25	8
KPNA6	0.9018894	-0.148978	0.4070052	3613	3743	4182	2710	3779	3917
SLC16A6	1.2123656	0.2778248	0.1958751	278	188	278	174	366	362
SLC16A7	0	#NUM!	0.5337954	0	3	0	0	0	0
KPNA4	0.9489529	-0.075592	0.8392928	2984	2320	3100	2126	3013	2836
SLC16A8	1.4791667	0.5647846	0.3932939	33	6	9	19	28	24
KPNA5	0.9972527	-0.003969	0.8243878	137	70	157	101	133	129
SLC16A9	8	3	0.2732425	0	1	0	0	0	8
KPNA2	0.9587375	-0.060792	0.9641819	15448	15382	18246	14249	17377	15425
PHF2P2	0.8333333	-0.263034	0.9214717	5	0	1	2	0	3
SLC16A2	3	1.5849625	0.6732949	0	0	1	0	0	3
KPNA3	1.0729505	0.1015836	0.3403782	2121	1385	2032	1585	2353	2004

SLC16A3	1.1132813	0.1548181	0.1043267	2682	3392	3654	3540	3896	3394
SLC16A4	1.6255708	0.7009464	3.99E-05	410	264	421	478	702	600
KPNA1	0.7617899	-0.392535	0.0201987	1272	1085	1375	697	1057	1089
SLC16A5	1	0	0.6948367	358	314	428	368	388	344
AL512652.1	1	0	0.9195944	9	2	8	8	3	8
AL512652.2	0.4130435	-1.275634	0.1327816	22	14	10	1	10	8
UNQ6494	1.875	0.9068906	0.4911377	4	2	2	5	0	10
KPNB1	0.9266416	-0.109917	0.6202157	11895	10660	13828	9429	11978	12307
SERBP1P1	1.1541667	0.2068516	0.1677258	207	187	326	280	295	256
BEAN1	0.8548009	-0.22634	0.3657849	236	324	294	194	291	245
SERBP1P4	2.75	1.4594316	0.4129302	2	0	2	0	5	6
TSACC	1.2153846	0.2814129	0.5011687	21	22	22	32	24	23
DDX39B-AS1	0	#NUM!	0.3042138	3	2	0	0	0	0
SERBP1P5	1.0114213	0.0163841	0.7176422	270	246	272	235	281	281
SERBP1P6	1.4615385	0.5474878	0.5906893	1	5	7	4	10	5
AC022497.1	1.1666667	0.2223924	0.878776	6	0	6	0	11	3
AC022507.1	#DIV/0!	#DIV/0!	0.3597364	0	0	0	0	0	5
AC093724.1	0.8072289	-0.30895	0.6915301	18	23	42	25	22	20
RNU6-50P	1.6666667	0.7369656	0.7418307	0	0	3	0	4	1
EEF2	0.9136404	-0.130302	0.5528745	58310	72678	74490	59944	65919	61870
AL161891.1	1	0	0.8727647	105	75	80	80	81	99
POMT2	0.9351485	-0.096733	0.7929967	1200	1371	1469	1226	1235	1317
POMT1	1.2223602	0.2896695	0.0591011	246	242	317	358	315	311
WISP3	1	0	0.9804499	4	1	0	0	1	4
AL132819.1	0	#NUM!	0.5075476	0	0	4	0	0	0
IQUB	1.2	0.2630344	0.7849961	3	1	6	3	5	4
AC009132.1	0	#NUM!	0.1796811	2	5	0	0	0	0
AC008543.5	0	#NUM!	0.6436235	2	0	0	0	0	0
AC092881.1	0.8888889	-0.169925	0.8966813	8	0	1	0	5	3
AC008543.1	0.7176471	-0.478654	0.4355635	30	20	35	20	26	15
AC079760.1	1	0	0.9443813	1	0	1	2	0	0
AC008543.3	0	#NUM!	0.4117982	0	3	1	0	0	0
AC079760.2	0.6875	-0.540568	0.6460509	3	9	4	1	6	4
NSMCE4A	1.0989655	0.1361461	0.1222046	870	903	1127	993	1132	1062
RNU6-57P	0.5	-1	0.81753	2	0	0	0	0	1
LPGAT1	0.5914787	-0.757602	0.0128992	460	248	489	136	209	363
AL356653.1	#DIV/0!	#DIV/0!	0.4121728	0	0	0	0	1	3
AC091959.1	0	#NUM!	0.824326	0	0	1	0	0	0
AC091959.2	0	#NUM!	0.824326	1	0	0	0	0	0
ARMT1	0.9592831	-0.059971	0.9153006	2162	1839	2360	1577	2415	2110
SCAF11	1.0936489	0.1291497	0.3149056	2226	1272	2375	1579	2553	2291
AL359644.1	0.9166667	-0.125531	0.9365348	7	10	7	7	4	11
COMMD8	0.9103064	-0.135576	0.6884607	688	422	685	498	680	456
COMMD9	1.0758841	0.1055227	0.2126768	1158	1297	1617	1448	1615	1318
AC015689.1	1.8095238	0.8556101	0.1853386	5	26	11	29	14	33
AL138781.1	0.8666667	-0.206451	0.8784672	2	19	9	11	5	10
AL138781.2	#DIV/0!	#DIV/0!	0.3721261	0	0	0	3	1	0
RN7SL625P	#DIV/0!	#DIV/0!	0.6411933	0	0	0	0	0	2
CRADD	0.9607843	-0.057715	0.9878097	184	187	241	180	223	185
AP001830.1	4	2	0.0788207	1	1	4	12	8	4
RNU6-1106P	0	#NUM!	0.824326	0	1	0	0	0	0
AL807761.4	#DIV/0!	#DIV/0!	0.8030358	0	0	0	1	0	0
YEATS2-AS1	0.6833333	-0.549339	0.5615268	4	26	30	7	5	29
AL807761.2	0	#NUM!	0.824326	0	0	1	0	0	0

AL807761.3	#DIV/0!	#DIV/0!	0.8030358	0	0	0	0	0	1
AC034154.1	1.5294118	0.6129769	0.5047543	6	9	2	18	3	5
COMMD2	0.9915584	-0.01223	0.7493893	1506	1423	1691	1281	1684	1616
COMMD3	1.1388889	0.187627	0.3688434	74	113	137	144	133	92
COMMD1	0.970297	-0.043502	0.8956654	242	335	433	312	376	292
COMMD6	1.0737463	0.1026532	0.2947042	481	555	659	642	631	547
COMMD7	0.9695509	-0.044611	0.9233552	1194	1280	1467	1165	1405	1251
AC005165.1	0.8387097	-0.253757	0.8111722	11	13	7	11	5	10
COMMD4	1.0327562	0.0464997	0.4673553	1159	1506	1609	1541	1571	1302
COMMD5	0.8722966	-0.197109	0.4214993	316	304	351	264	318	265
ARMS2	1.7027027	0.7678266	0.1828368	9	11	17	23	26	14
USP27X-AS1	0.7821229	-0.354533	0.3989054	58	66	55	23	72	45
SATB1	1.6044521	0.6820807	0.0021113	164	225	195	208	341	388
SATB2	0.6052632	-0.724366	0.0609559	74	71	83	33	40	65
HEATR1	0.8246929	-0.278071	0.1371028	2889	2291	2960	1483	2621	2609
MALSU1	0.9826754	-0.025213	0.7417032	1024	1093	1404	1248	1192	1020
MIR544B	0	#NUM!	0.5075476	0	0	4	0	0	0
AC026120.1	0.4444444	-1.169925	0.3993194	6	3	9	4	1	3
AC026120.2	0	#NUM!	0.824326	1	0	0	0	0	0
HEATR3	0.8179211	-0.289966	0.0815837	884	830	1076	680	861	741
HEATR4	0.8852459	-0.17585	0.8188656	30	11	20	4	18	32
HEATR6	0.838958	-0.25333	0.1942916	522	590	577	379	516	522
FRMD8P1	0	#NUM!	0.824326	0	1	0	0	0	0
AC011337.1	0.8089888	-0.305808	0.6993077	29	17	43	28	19	25
CMSS1	0.9440476	-0.083068	0.9373104	912	1061	1387	1048	1146	978
OR10AB1P	0	#NUM!	0.824326	0	1	0	0	0	0
AL627230.2	0.3888889	-1.36257	0.3198729	5	5	8	0	6	1
AL627230.4	1	0	0.9890559	0	0	1	0	0	1
ZNF123P	#DIV/0!	#DIV/0!	0.8030358	0	0	0	0	1	0
VIPR1-AS1	0.125	-3	0.226064	2	6	0	0	1	0
RN7SL620P	1	0	0.9890559	1	0	0	0	0	1
PCOLCE	1.0215232	0.0307219	0.620351	187	165	252	208	215	194
H1FX-AS1	0.6875	-0.540568	0.6213045	5	17	26	19	10	4
TANC2	0.9854627	-0.021127	0.9287581	2874	2694	2893	1904	2912	3522
TANC1	0.5948588	-0.749381	4.52E-05	991	761	1010	379	624	640
MIR548O	0.5	-1	0.7417349	1	0	3	0	2	0
MIR548P	#DIV/0!	#DIV/0!	0.6251462	0	0	0	1	0	1
C13orf42	0.5	-1	0.8638172	0	0	2	1	0	0
MIR548L	0	#NUM!	0.824326	1	0	0	0	0	0
HMGB3P31	#DIV/0!	#DIV/0!	0.3981469	0	0	0	4	0	0
AL162386.1	0	#NUM!	0.824326	0	1	0	0	0	0
AL009176.1	0.2	-2.321928	0.528477	0	0	5	0	1	0
AL162386.2	1.8	0.8479969	0.4423173	4	2	9	0	9	18
ZNF346-IT1	0.3368421	-1.569856	0.0086484	31	31	33	11	14	7
MIR548V	0	#NUM!	0.824326	0	0	1	0	0	0
MIR548S	0	#NUM!	0.5337954	0	3	0	0	0	0
HMGB3P24	0.8181818	-0.289507	0.8318868	2	8	1	1	1	7
HMGB3P22	0.6666667	-0.584963	0.8532957	0	0	6	0	4	0
AP003392.6	0.2692308	-1.893085	0.1277878	12	7	7	0	1	6
AC106052.1	1	0	0.9239532	0	0	2	2	0	0
HMGB3P20	1	0	0.9890559	0	0	1	0	0	1
AP003392.2	0.5952381	-0.748461	0.229907	31	20	33	9	14	27
AP003392.4	0.678481	-0.55962	0.0749082	122	166	107	80	88	100
AP003392.5	0.7368421	-0.440573	0.6592802	19	5	14	11	6	11

HMGB3P17	#DIV/0!	#DIV/0!	0.8030358	0	0	0	0	0	1
HMGB3P14	1.75	0.8073549	0.7435757	4	0	0	0	5	2
AP003392.1	0.44	-1.184425	0.0521589	18	31	51	11	14	19
AL512413.1	#DIV/0!	#DIV/0!	0.4737294	0	0	0	0	4	0
CLCN3P1	0	#NUM!	0.824326	0	1	0	0	0	0
AC006480.2	0.08	-3.643856	0.0200928	6	6	13	1	0	1
AC006480.1	1.2857143	0.3625701	0.8684657	2	3	2	0	9	0
HEXA-AS1	1.1081081	0.1480986	0.8062948	15	10	12	7	15	19
IRF3	1.0051066	0.0073486	0.5873304	1961	2076	2621	2522	2310	1860
IRF4	#DIV/0!	#DIV/0!	0.5371397	0	0	0	0	0	3
IRF1	0.6079439	-0.71799	0.0001229	606	681	853	462	454	385
IRF2	1.3206107	0.4012052	0.0072852	282	288	347	327	449	435
IRF7	4.7980769	2.2624563	1.92E-23	59	83	66	317	346	335
IRF8	0.5757576	-0.796467	0.1706028	25	32	42	14	14	29
IRF5	3	1.5849625	0.5169108	0	3	0	0	0	9
IRF6	0.879637	-0.18502	0.3294207	1068	1251	1428	923	1181	1192
IRF9	1.9852941	0.9893528	0.0318886	11	27	30	60	32	43
TPT1-AS1	0.9350649	-0.096862	0.9591113	23	23	31	25	25	22
AL139035.1	0.8039216	-0.314873	0.7770266	17	10	24	18	6	17
P2RX6P	#DIV/0!	#DIV/0!	0.4501902	0	0	0	0	0	4
IRGM	0.7466667	-0.421464	0.4837221	25	28	22	16	27	13
IRGQ	0.8313309	-0.266505	0.1371582	647	737	780	510	650	639
RBBP4P1	0.904	-0.145605	0.7791675	46	38	41	21	49	43
RBBP4P2	0.3333333	-1.584963	0.6622061	0	3	0	0	0	1
RBBP4P3	#DIV/0!	#DIV/0!	0.8030358	0	0	0	1	0	0
CREB3L3	0.6363636	-0.652077	0.6749782	2	8	1	3	4	0
CREB3L4	0.9490566	-0.075434	0.9552571	331	314	415	323	346	337
CREB3L1	1.4469274	0.5329925	0.0755377	65	63	51	74	94	91
AL121944.1	0.8575419	-0.221721	0.3924631	244	215	257	155	223	236
CREB3L2	0.7857047	-0.347941	0.0475784	2358	2599	2500	1410	2038	2411
PIP4K2A	1.2644156	0.3384707	0.0049083	1696	1893	2186	1819	2866	2617
PIP4K2B	0.8577034	-0.221449	0.1943455	1544	1608	1690	1082	1488	1583
PIP4K2C	0.7302825	-0.453473	0.0009648	2586	3031	3233	1840	2359	2264
SARNP	0	#NUM!	0.4324683	2	0	2	0	0	0
KIAA0368	1.058775	0.082396	0.2904245	3832	2897	4177	3174	4260	4113
TDRKH-AS1	2.2666667	1.1805722	0.235613	0	1	14	16	15	3
AC106782.1	#DIV/0!	#DIV/0!	0.8030358	0	0	0	1	0	0
KIAA0391	0.8721934	-0.19728	0.4977646	186	175	218	137	180	188
ZBTB40-IT1	0.7272727	-0.459432	0.8381185	4	0	7	3	1	4
TGFB1I1	0	#NUM!	0.4374838	0	4	0	0	0	0
AC106782.5	0.5454545	-0.874469	0.6495407	4	1	6	0	0	6
AC106782.2	0.9032258	-0.146841	0.8637081	31	22	40	25	41	18
AC147651.1	#DIV/0!	#DIV/0!	0.6411933	0	0	0	0	0	2
AC106782.6	0.8181818	-0.289507	0.8304832	5	5	1	2	2	5
RNU6-60P	#DIV/0!	#DIV/0!	0.6099294	0	0	0	2	0	0
AC025043.1	0	#NUM!	0.6436235	2	0	0	0	0	0
SSFA2	1.0433693	0.0612498	0.560977	1492	1058	1439	944	1603	1615
AC012414.5	0.5044248	-0.987289	0.0746372	34	33	46	14	13	30
AL049646.1	1.2941176	0.3719688	0.7447271	2	10	5	1	9	12
AC011825.4	0	#NUM!	0.824326	0	0	1	0	0	0
AC011825.2	2.1111111	1.0780025	0.3488381	1	5	3	4	7	8
AC020934.1	3.25	1.7004397	0.1573122	2	5	1	2	9	15
RLIM	1.001512	0.0021797	0.8125706	2676	1891	2708	1573	2795	2918
SARM1	0.3613445	-1.468553	0.0088962	48	32	39	12	9	22

IRS4	0.84	-0.251539	0.8324792	6	10	9	4	3	14
IRS2	0.9144816	-0.128974	0.5587322	851	794	834	627	814	826
METRNL	1.2174224	0.2838299	0.0393015	1134	1530	1526	1911	1743	1447
AL353763.1	0.375	-1.415037	0.3108237	9	6	1	0	4	2
AL353763.2	1.137931	0.1864131	0.4329409	90	40	102	97	92	75
SLC15A1	3	1.5849625	0.6136153	0	1	0	2	1	0
SLC15A2	2.4401114	1.286947	4.27E-10	113	110	136	247	278	351
AC008938.1	0	#NUM!	0.6840298	0	0	2	0	0	0
GLYR1	0.8013045	-0.319577	0.0188928	4383	4535	5340	3223	3997	4205
OR8G3P	1.1612903	0.2157287	0.5889178	16	22	24	28	22	22
PHF21A	1.0050505	0.007268	0.6662972	335	343	510	340	426	428
PHF21B	1.6363636	0.7104934	0.3425602	7	6	9	21	11	4
AL133351.1	0.5	-1	0.6450426	3	2	1	2	1	0
SLC15A3	1.5178876	0.6020649	0.0002636	347	420	407	653	597	532
KIAA0319	2.8571429	1.5145732	0.2180046	0	5	2	10	4	6
SLC15A4	1.0700676	0.097702	0.333229	462	559	606	524	672	545
CYBRD1	2.25	1.169925	0.2351855	13	0	3	19	8	9
BRWD1-AS1	0	#NUM!	0.2506258	0	4	2	0	0	0
BRWD1-AS2	0.5	-1	0.4917219	8	2	2	1	3	2
RLN2	0.8088235	-0.306103	0.5119883	44	51	41	32	39	39
RLN3	0	#NUM!	0.6840298	0	0	2	0	0	0
KC877373.1	0.7777778	-0.36257	0.826401	6	2	1	3	2	2
EFHB	0.6666667	-0.584963	0.1483769	60	50	73	33	47	42
RLN1	1.0217391	0.0310269	0.9242163	14	21	11	17	20	10
OR8G2P	0	#NUM!	0.5337954	0	3	0	0	0	0
AC145350.2	10	3.3219281	0.1629626	0	1	0	0	6	4
AC145350.3	#DIV/0!	#DIV/0!	0.6411933	0	0	0	0	0	2
AL592424.1	0.0909091	-3.459432	0.1228569	5	6	0	1	0	0
SMC5-AS1	0.9444444	-0.082462	0.9917434	23	17	14	24	8	19
AC131392.1	0.4647887	-1.105353	0.1059773	15	20	36	8	13	12
AC131392.2	#DIV/0!	#DIV/0!	0.8030358	0	0	0	0	0	1
SART1	0.8316644	-0.265927	0.1302085	2792	3179	3516	2713	2747	2430
CYP4F62P	1.1764706	0.2344653	0.8131321	10	1	6	2	6	12
SART3	0.9066851	-0.141327	0.455013	2605	2857	3229	2288	2765	2827
HMGN3-AS1	0.8225108	-0.281893	0.6097906	95	34	102	62	58	70
LCA5L	1.2222222	0.2895066	0.7885774	4	1	4	3	4	4
AC006001.3	0.8378378	-0.255257	0.3700177	316	269	451	291	329	248
AC006001.2	0.979798	-0.029444	0.9271773	61	43	94	45	87	62
SARS2	1.8709677	0.9037847	0.1652531	14	11	6	23	18	17
BX649601.1	1.3214286	0.4020984	0.5616438	1	11	16	12	13	12
OR8G7P	0	#NUM!	0.4460243	4	0	0	0	0	0
AC025283.2	1.3333333	0.4150375	0.8720582	0	1	2	0	4	0
KIAA0355	0.8029084	-0.316693	0.0437749	2232	2302	2549	1409	2130	2148
AC073548.1	0.8380952	-0.254814	0.7881556	26	16	63	30	38	20
IRX2	1.1432274	0.1931124	0.0308426	2405	2225	2701	2691	3019	2671
IRX6	0	#NUM!	0.824326	0	1	0	0	0	0
EFL1	1.0534874	0.075173	0.4003547	852	605	880	693	864	905
IRX3	1.2627551	0.3365749	0.1316367	91	147	154	133	201	161
IRX4	1.1958289	0.2580109	0.0147308	2016	2024	2481	2763	2569	2466
RNU6-1120P	0	#NUM!	0.824326	0	1	0	0	0	0
EDNRA	1.5	0.5849625	0.8637378	2	0	0	0	0	3
EDNRB	0.774957	-0.367812	0.0516427	829	602	893	494	640	667
AC015688.4	#DIV/0!	#DIV/0!	0.8030358	0	0	0	0	0	1
AC002524.1	1.4259259	0.511899	0.1990104	41	35	32	53	70	31

FLJ37453	0.9656489	-0.050429	0.8512132	87	50	125	119	66	68
AP001831.1	#DIV/0!	#DIV/0!	0.8030358	0	0	0	0	1	0
RNU6-1111P	0	#NUM!	0.824326	0	0	1	0	0	0
AC007225.2	#DIV/0!	#DIV/0!	0.3981469	0	0	0	4	0	0
CARMIL1	0.7741422	-0.369329	0.0365748	1487	1213	1701	828	1244	1335
CARMIL2	0.8460039	-0.241264	0.2709893	351	328	347	240	342	286
CARMIL3	0.3333333	-1.584963	0.669913	3	0	0	0	0	1
SS18L1	1.031477	0.0447116	0.5903131	679	418	555	550	577	577
AC007225.1	1.1428571	0.1926451	0.8194668	3	0	11	1	5	10
SS18L2	0.9872226	-0.018553	0.7886777	422	520	545	537	509	422
AL645504.1	1.2321429	0.3011695	0.5717593	25	2	29	19	21	29
AC055720.2	3	1.5849625	0.6896151	1	0	0	0	3	0
AC073641.1	3	1.5849625	0.1838165	3	0	4	14	5	2
MIR5581	0.5	-1	0.578571	2	5	7	6	1	0
AC147067.1	1.5	0.5849625	0.8582217	2	0	0	0	1	2
C9orf3	0.9430586	-0.084581	0.8711896	523	581	740	492	617	630
QTNF3-AMA(	1	0	0.9890559	0	1	0	0	0	1
AC121334.2	2	1	0.7275107	0	0	2	0	4	0
MIR5587	0.25	-2	0.1574434	5	12	3	3	0	2
MIR5582	0	#NUM!	0.6436235	2	0	0	0	0	0
ARNTL	1.0151057	0.02163	0.6761522	347	300	346	294	360	354
ZNF137P	1.0788644	0.1095135	0.635506	116	49	152	65	148	129
AC006971.1	0	#NUM!	0.6385096	0	2	0	0	0	0
ARNT2	#DIV/0!	#DIV/0!	0.1677595	0	0	0	3	0	4
ITM2BP1	0.6153846	-0.70044	0.6218945	6	6	1	0	8	0
GLYCAM1	#DIV/0!	#DIV/0!	0.8030358	0	0	0	0	0	1
TRIP10	0.9972519	-0.00397	0.6458183	3694	4347	4695	4218	4445	4038
TRIP11	1.3453177	0.4279469	0.0197303	876	455	1061	797	1300	1121
TRIP12	1.0392731	0.0555748	0.5565197	6553	5133	6418	4273	6834	7708
TRIP13	0.8764744	-0.190216	0.2538265	2372	2370	2973	2084	2474	2204
AL596244.1	0	#NUM!	0.3414317	0	2	3	0	0	0
MALRD1	0.3076923	-1.70044	0.2868013	9	3	1	0	3	1
CMTR1	1.0020669	0.0029789	0.6660577	2340	2601	2800	2163	2845	2749
CMTR2	0.9084531	-0.138516	0.5906837	1203	741	1049	658	1053	1008
AC106053.1	#DIV/0!	#DIV/0!	0.4737294	0	0	0	0	4	0
AC024558.1	0.1666667	-2.584963	0.3329202	2	4	0	1	0	0
DEFB130C	#DIV/0!	#DIV/0!	0.8030358	0	0	0	0	0	1
AL139130.1	#DIV/0!	#DIV/0!	0.3981469	0	0	0	4	0	0
RNU6-722P	0.8	-0.321928	0.7457602	11	18	16	18	8	10
ISCU	0.8606286	-0.216537	0.2400095	2941	3021	3997	2845	2987	2739
AL157834.2	2	1	0.8186965	0	0	1	0	0	2
DEFB131D	#DIV/0!	#DIV/0!	0.6411933	0	0	0	0	0	2
DEFB131E	0	#NUM!	0.5387425	3	0	0	0	0	0
DEFB131B	1.1428571	0.1926451	0.9165513	4	0	3	0	6	2
AC099494.3	0	#NUM!	0.2359908	1	0	6	0	0	0
AC091812.1	#DIV/0!	#DIV/0!	0.8030358	0	0	0	0	0	1
AC091812.2	4.6666667	2.2223924	0.1625522	0	0	3	9	1	4
AC099494.2	1.8461538	0.8845228	0.3967507	9	3	1	11	8	5
DDTP1	0.5	-1	0.8188512	2	0	0	0	1	0
AC091812.3	#DIV/0!	#DIV/0!	0.3981469	0	0	0	4	0	0
AC092296.2	0	#NUM!	0.6840298	0	0	2	0	0	0
AC138781.1	0	#NUM!	0.5172237	1	2	0	0	0	0
AC130324.1	0	#NUM!	0.6631647	1	0	1	0	0	0
AC130324.2	0	#NUM!	0.5387425	3	0	0	0	0	0

AP001092.1	0	#NUM!	0.6385096	0	2	0	0	0	0
AC016526.2	0.3333333	-1.584963	0.6628865	2	0	1	0	0	1
TESC-AS1	0	#NUM!	0.824326	1	0	0	0	0	0
PRTFDC1	0.6666667	-0.584963	0.7989349	2	0	7	2	2	2
AL360081.1	#DIV/0!	#DIV/0!	0.0960885	0	0	0	8	1	0
SASH3	0	#NUM!	0.5387425	3	0	0	0	0	0
U2AF1L5	0.8622754	-0.213779	0.7127449	37	72	58	57	49	38
AC069281.2	0.8	-0.321928	0.7749842	9	4	2	2	8	2
AC234582.1	0	#NUM!	0.2084189	0	3	4	0	0	0
U2AF1L4	1.1604938	0.2147388	0.6118868	29	33	19	39	34	21
SASH1	0.9512381	-0.072122	0.8092434	975	830	820	529	912	1056
CMTM7	1.1991525	0.2620152	0.3128209	59	98	79	104	81	98
CMTM8	0.9190283	-0.121819	0.7253195	466	482	534	475	489	398
CMTM3	1.0690035	0.0962666	0.2359663	5099	6379	6666	6519	6871	6006
CMTM4	0.6432466	-0.636556	4.40E-05	3707	3802	3863	1745	2689	2881
CMTM6	0.9576988	-0.062356	0.891816	1953	1854	2103	1544	2157	1959
GDAP1	1.0932203	0.1285842	0.4863494	108	89	157	95	164	128
GDAP2	1.0539368	0.0757883	0.366965	511	445	657	485	627	588
AL163952.1	0.6136364	-0.704544	0.374823	17	11	16	5	9	13
RNU6-720P	1	0	0.9829887	2	0	2	0	1	3
CMTM2	0.5	-1	0.81106	0	2	0	0	0	1
AL359643.3	1.4782609	0.5639009	0.5594999	16	3	4	6	21	7
AL355574.1	1.649635	0.7221469	0.0267514	49	47	41	83	74	69
AL359643.2	1.0454545	0.0641303	0.9292207	5	10	7	4	10	9
ISM1	0	#NUM!	0.6436235	2	0	0	0	0	0
ISM2	1.3945897	0.4798407	3.44E-06	5103	5935	6669	7841	8774	8079
AC012417.1	#DIV/0!	#DIV/0!	0.3981469	0	0	0	4	0	0
ISLR	2	1	0.691673	0	0	2	0	2	2
ARPIN	0.6295515	-0.667604	1.49E-05	1240	1164	1386	621	835	930
AC007952.6	1.1875	0.2479275	0.838319	9	3	4	5	14	0
AC007952.8	#DIV/0!	#DIV/0!	0.5070017	0	0	0	3	0	0
AC007952.9	0	#NUM!	0.824326	1	0	0	0	0	0
AC020931.1	0	#NUM!	0.6385096	0	2	0	0	0	0
AC007952.2	0.5405405	-0.887525	0.3836133	1	21	15	4	12	4
ISL1	0.6914894	-0.532221	0.3855314	29	32	33	30	17	18
AC007952.4	0.875	-0.192645	0.9988474	0	1	7	3	2	2
POM121L6P	#DIV/0!	#DIV/0!	0.6411933	0	0	0	0	0	2
ISL2	3.4666667	1.7935491	0.0036206	18	8	4	30	36	38
OBSCN-AS1	5	2.3219281	0.4600065	0	1	0	0	0	5
PLA2G3	1.2083333	0.2730185	0.7395746	5	8	11	2	11	16
MRPS36P1	0	#NUM!	0.4374838	0	4	0	0	0	0
PLA2G5	0.1666667	-2.584963	0.3767372	0	6	0	0	0	1
PLA2G6	1.1240768	0.1687406	0.2332167	186	208	283	231	283	247
PRELID3A	0.6538462	-0.612977	0.5969677	12	15	25	26	3	5
PRELID3B	1.0013365	0.0019269	0.6545446	3459	2612	3656	2802	3647	3291
AL353622.2	2	1	0.8324799	1	0	0	0	2	0
CYP4F2	0.35	-1.514573	0.1462607	19	9	12	5	0	9
AL353622.1	0.7978142	-0.325875	0.4798919	62	57	64	48	70	28
CYP4F3	0.2368421	-2.078003	0.0409178	15	6	17	3	4	2
AL031709.1	0.28125	-1.830075	0.2111785	7	6	19	9	0	0
FOXL2NB	1.4313725	0.5173992	0.3073411	15	19	17	22	21	30
AC113155.1	1	0	0.9699945	2	0	0	2	0	0
RMI2	0.9835218	-0.023971	0.7809817	1755	2066	2369	1915	2285	1888
RMI1	1.1495238	0.2010363	0.1395536	1189	732	1229	936	1390	1295

FOXC2-AS1	#DIV/0!	#DIV/0!	0.6411933	0	0	0	0	0	2
ISPD	0.6294643	-0.667804	0.0145568	159	136	153	78	115	89
SOCS5P3	#DIV/0!	#DIV/0!	0.8030358	0	0	0	0	1	0
SOCS5P4	0.8108108	-0.302563	0.7541848	18	7	12	9	7	14
EGFR	0.8820331	-0.181095	0.3640502	7135	6961	7054	4210	6474	7971
AL353768.1	0.75	-0.415037	0.8392802	1	3	0	0	1	2
AC068700.1	6	2.5849625	0.1579832	1	1	0	3	7	2
ST7-AS1	0.5	-1	0.8188512	2	0	0	0	1	0
ST7-AS2	#DIV/0!	#DIV/0!	0.5371397	0	0	0	0	0	3
POM121L4P	1.6666667	0.7369656	0.6697651	2	4	0	2	6	2
TAOK3	1.0726329	0.1011565	0.3278613	1100	831	1153	840	1271	1197
TAOK1	1.1129209	0.1543511	0.5806988	2206	1234	2024	1216	2218	2647
TAOK2	0.9161703	-0.126312	0.5698295	1947	2382	2387	1830	2122	2201
OR2A20P	0.7916667	-0.337035	0.8341079	2	3	19	3	12	4
NAB1	1.2918278	0.3694138	0.0086059	1390	1018	1471	1204	1936	1871
NAB2	0.8925209	-0.164042	0.4112086	1101	1337	1386	1033	1256	1124
AL133396.2	0.1111111	-3.169925	0.1633412	4	2	3	1	0	0
AL133406.2	0.6603774	-0.598637	0.4884206	24	12	17	20	8	7
MTPAP	1.0397066	0.0561765	0.3905036	1349	1087	1518	1170	1537	1404
IST1	0.9690576	-0.045346	0.9429903	6166	6949	7407	6049	7269	6569
NAAA	1.9473684	0.9615259	0.2620653	11	6	2	15	13	9
ANKRD62P1	0.6666667	-0.584963	0.8292596	1	2	0	0	0	2
PLA2R1	#DIV/0!	#DIV/0!	0.6554062	0	0	0	0	2	0
BRIX1	1.1176581	0.1604789	0.1428384	1861	1251	2217	1894	2240	1822
NAE1	0.9960582	-0.005698	0.7092818	2226	1598	2772	1814	2624	2132
NADK	1.120607	0.1642804	0.0779074	1826	2042	2392	2374	2464	2177
ARPC4	0.9617347	-0.056289	0.9293508	1263	1443	1606	1517	1427	1203
ARPC5	0.9214953	-0.117951	0.5435462	10352	10732	12328	9265	11390	10134
AC008937.3	0.8461538	-0.241008	0.8228285	6	11	9	0	6	16
ARPC2	1.0335178	0.0475632	0.3117579	15856	16911	20369	17249	20024	17644
ARPC3	1.0677799	0.0946142	0.2235258	3082	2926	4113	3851	3794	3162
NACA	1.118427	0.1614711	0.064105	15399	13501	19746	18312	19542	16553
AC008937.2	#DIV/0!	#DIV/0!	0.2887809	0	0	0	0	0	6
ISY1	1.0273224	0.038889	0.5831406	238	207	287	266	262	224
NFYC-AS1	0.8416667	-0.248679	0.6584663	32	43	45	16	31	54
RNU6-33P	#DIV/0!	#DIV/0!	0.8030358	0	0	0	1	0	0
CIITA	0.4880952	-1.034765	0.0867912	27	27	30	6	21	14
NKPD1	1.3333333	0.4150375	0.7871271	3	2	1	4	4	0
AC024901.1	3	1.5849625	0.2880674	0	0	5	3	9	3
RMRP	0.2857143	-1.807355	0.4401756	5	0	2	1	1	0
RMRP	0.2857143	-1.807355	0.4401756	5	0	2	1	1	0
CEACAMP6	#DIV/0!	#DIV/0!	0.8030358	0	0	0	0	1	0
RNU6-32P	2	1	0.6681183	0	4	0	0	4	4
STXBP5-AS1	6	2.5849625	0.1595788	0	0	2	3	7	2
CEACAMP9	6	2.5849625	0.1353486	2	1	0	0	10	8
AC008687.3	1	0	0.9617562	0	0	2	0	0	2
AC008687.4	1.0909091	0.1255309	0.8983866	5	0	6	2	8	2
AC008687.6	#DIV/0!	#DIV/0!	0.5230806	0	0	0	1	2	0
AC008687.2	0.75	-0.415037	0.8696703	3	0	5	2	1	3
NAF1	1.0821178	0.1138575	0.3391448	581	668	602	551	707	745
AC008687.8	#DIV/0!	#DIV/0!	0.6554062	0	0	0	0	2	0
BECN1	0.8355009	-0.259287	0.157144	835	961	1049	697	787	893
ROCK1	1.5320543	0.6154674	0.0004498	1561	893	1820	1547	2668	2333
ROCK2	1.1081016	0.1480901	0.3089005	1632	842	1661	1110	1724	1748

BECN2	0	#NUM!	0.824326	0	1	0	0	0	0
PPT2-EGFL8	0.6153846	-0.70044	0.5740235	8	8	10	6	0	10
AL136141.1	0.5	-1	0.6773186	0	4	2	2	1	0
MIR4771-2	0	#NUM!	0.824326	0	1	0	0	0	0
POLR2J3	0.7	-0.514573	0.2274747	41	80	89	45	55	47
POLR2J4	0.9853801	-0.021248	0.8089249	88	113	141	146	75	116
POLR2J4	0.9853801	-0.021248	0.8089249	88	113	141	146	75	116
POLR2J2	0.6071429	-0.719892	0.5051149	10	9	9	1	2	14
RNU6-30P	0.3	-1.736966	0.3488645	5	5	0	0	1	2
CYP4B1	1.4871795	0.5725788	0.3270938	6	15	18	20	24	14
NAGA	0.9077533	-0.139628	0.5049351	1052	1283	1199	907	1169	1132
NAGK	1.2350282	0.304544	0.0085609	2428	3026	3396	3787	3853	3290
NAGS	1.0581395	0.0815299	0.8038578	26	35	25	30	38	23
LDLRAD2	10	3.3219281	0.1378672	0	0	1	1	1	8
LDLRAD3	1.1983806	0.2610861	0.0736034	677	663	636	600	836	932
LDLRAD4	1.88	0.9107327	0.2153488	8	9	8	8	14	25
AC139768.1	0.8235294	-0.280108	0.8577907	14	2	1	8	2	4
CCDC102A	0.7582645	-0.399227	0.0707867	277	356	335	225	253	256
CCDC102B	0.9166667	-0.125531	0.9637442	3	7	2	7	4	0
AC007128.2	1.3728814	0.457207	0.4271787	29	11	19	12	38	31
AC093864.1	#DIV/0!	#DIV/0!	0.4737294	0	0	0	0	4	0
AC007128.1	0.8168421	-0.291871	0.4282696	174	120	181	48	165	175
AL136537.2	0.842723	-0.24687	0.5257386	159	82	185	71	157	131
AL136537.1	0	#NUM!	0.6840298	0	0	2	0	0	0
RNU6-1127P	0	#NUM!	0.824326	1	0	0	0	0	0
RNU6-37P	#DIV/0!	#DIV/0!	0.8030358	0	0	0	1	0	0
NAIP	0.7857143	-0.347923	0.7035211	9	13	20	8	10	15
AC104009.1	#DIV/0!	#DIV/0!	0.8030358	0	0	0	0	1	0
TBC1D9B	0.8620557	-0.214147	0.1464737	6145	6898	7690	5196	6438	6239
AC027348.1	#DIV/0!	#DIV/0!	0.8030358	0	0	0	1	0	0
AL158819.1	1	0	0.9644635	3	0	0	3	0	0
SEC14L2	1.05	0.0703893	0.8264438	9	12	19	12	10	20
SEC14L3	#DIV/0!	#DIV/0!	0.8030358	0	0	0	0	0	1
BRIP1	0.7207207	-0.472488	0.1031661	183	180	192	79	137	184
AC116631.1	2	1	0.8147319	1	0	0	0	0	2
SEC14L1	0.9260131	-0.110896	0.6228092	1222	1298	1305	891	1303	1348
SEC14L6	0.5	-1	0.643229	4	1	1	1	0	2
SEC14L4	#DIV/0!	#DIV/0!	0.3831122	0	0	0	0	5	0
SEC14L5	0.4791667	-1.061401	0.1911575	15	12	21	9	6	8
TBC1D8B	1.1591738	0.2130968	0.3796543	382	169	272	197	415	342
EGR1	7.2085106	2.8497012	3.24E-41	74	49	112	489	616	589
EGR2	1.4736842	0.5594274	0.5763145	9	8	2	11	15	2
RNU6-35P	0	#NUM!	0.824326	0	0	1	0	0	0
NANP	0.9250386	-0.112414	0.6839448	423	412	459	308	445	444
AC119428.1	0	#NUM!	0.6385096	0	2	0	0	0	0
AC119428.2	0	#NUM!	0.5337954	0	3	0	0	0	0
MIS18BP1	1.185295	0.2452462	0.1547729	1295	638	1440	956	1655	1387
CHAMP1	0.7649194	-0.38662	0.0061719	1419	1377	1611	1004	1255	1112
NANS	1.0099691	0.0143111	0.5893852	825	991	1093	940	1026	972
NAMA	0.7307692	-0.452512	0.6781713	6	10	10	4	3	12
AC026362.2	#DIV/0!	#DIV/0!	0.6554062	0	0	0	0	2	0
AC087257.1	0	#NUM!	0.6840298	0	0	2	0	0	0
AC008781.2	0.6	-0.736966	0.8149364	0	0	5	0	2	1
AC008781.3	1	0	0.9890559	0	1	0	0	1	0

AC008781.1	0.3333333	-1.584963	0.7307796	0	0	3	0	1	0
NAPA	0.8465104	-0.2404	0.1899315	1166	1172	1330	1048	1084	973
NAPB	1.0169779	0.0242884	0.6053068	592	523	652	501	678	618
NAPG	1.0436949	0.0617	0.4216978	869	744	996	723	982	1018
RNU6-1128P	0	#NUM!	0.6436235	2	0	0	0	0	0
AC004477.1	0.8095238	-0.304855	0.8039138	4	10	7	5	12	0
AC004477.3	2.2727273	1.1844246	0.3227361	2	4	5	1	20	4
ITCH	1.0583029	0.0817526	0.4078254	3211	2422	3406	2263	3557	3746
TBC1D3K	1.625	0.7004397	0.3181294	7	8	9	12	12	15
TBC1D3L	1.2280285	0.296344	0.1196297	115	115	191	181	171	165
SMUG1-AS1	0.0769231	-3.70044	0.018586	7	16	3	0	0	2
AC015883.1	2	1	0.8147319	1	0	0	0	0	2
NARF	0.9683058	-0.046465	0.881085	1558	1650	1998	1689	1806	1546
TBC1D3B	2.6666667	1.4150375	0.4959556	3	0	0	2	5	1
ZNF667-AS1	#DIV/0!	#DIV/0!	0.6099294	0	0	0	2	0	0
TBC1D3E	0.5	-1	0.421003	10	5	3	0	5	4
SNORD1B	#DIV/0!	#DIV/0!	0.8030358	0	0	0	0	1	0
TBC1D3F	1	0	0.9936882	3	16	13	4	21	7
TBC1D3C	#DIV/0!	#DIV/0!	0.3109758	0	0	0	0	6	0
TBC1D3D	1.1434263	0.1933634	0.4659063	110	47	94	91	124	72
TBC1D3G	2.7692308	1.4694853	0.1383904	5	2	6	5	26	5
TBC1D3H	#DIV/0!	#DIV/0!	0.6411933	0	0	0	0	0	2
NARS	0.9554328	-0.065774	0.9317802	2921	2191	3033	2204	2813	2765
SNORD19	0.3898305	-1.359081	0.0608184	26	15	18	6	9	8
SNORD19	0.3898305	-1.359081	0.0608184	26	15	18	6	9	8
SNORD19	0.3898305	-1.359081	0.0608184	26	15	18	6	9	8
AKR7A2	1.254717	0.327362	0.004323	1827	2101	2326	2739	2711	2397
SNORD17	0.9526814	-0.069934	0.9571199	85	86	146	46	106	150
AKR7A3	1.2246377	0.292355	0.3128266	40	40	58	59	53	57
SNORD18	0.8	-0.321928	0.8727609	2	1	2	0	3	1
SNORD18	0.8	-0.321928	0.8727609	2	1	2	0	3	1
TBC1D31	1.0177453	0.0253766	0.6230364	306	256	396	281	370	324
TBC1D32	1.2861035	0.3630068	0.1413055	137	103	127	99	175	198
TBC1D30	1.4090909	0.4947647	0.5767235	12	9	1	17	7	7
SNORD11	3.5	1.8073549	0.0682234	1	1	8	8	18	9
SNORD11	3.5	1.8073549	0.0682234	1	1	8	8	18	9
CDKAL1	0.938999	-0.090805	0.7688164	594	622	702	441	670	690
TBC1D2B	0.9246765	-0.112979	0.634955	1256	1513	1559	1047	1443	1512
NCKAP5-IT1	0	#NUM!	0.824326	0	1	0	0	0	0
TBC1D20	0.9709116	-0.042588	0.922951	2249	2292	2644	2026	2517	2433
TBC1D24	1.2727273	0.3479233	0.1639785	97	115	85	108	139	131
TBC1D25	0.8483271	-0.237307	0.257193	399	455	491	322	455	364
TBC1D23	1.0846809	0.1172706	0.4116381	971	518	861	636	1005	908
AL591686.1	0.4081633	-1.292782	0.1078772	13	14	22	5	10	5
TBC1D29	1.6	0.6780719	0.720554	5	0	0	5	3	0
EMID1	1.6666667	0.7369656	0.2752967	11	3	16	12	22	16
TBC1D26	0	#NUM!	0.5075476	0	0	4	0	0	0
TBC1D19	1.1244444	0.1692124	0.4865571	95	50	80	79	96	78
PKDCC	0.6298077	-0.667017	0.0198065	129	124	163	87	88	87
SNORD3J	#DIV/0!	#DIV/0!	0.3981469	0	0	0	4	0	0
SNORD3A	0.1764706	-2.5025	0.1204267	4	5	8	2	1	0
AL096870.2	0.75	-0.415037	0.8651122	0	3	1	1	2	0
TBC1D13	0.8852927	-0.175774	0.3785838	1811	2217	2275	1803	1911	1866
TBC1D14	0.8154848	-0.29427	0.1191605	831	938	995	524	857	873

TBC1D12	1.1813126	0.2403907	0.1457827	347	240	312	278	413	371
TBC1D17	1.1155116	0.1577055	0.1822554	483	640	695	712	660	656
TBC1D15	1.1816277	0.2407755	0.0710635	1072	691	1100	916	1290	1177
TBC1D16	0.6694915	-0.578862	0.1609348	57	99	80	30	49	79
UEVLD	0.9120952	-0.132744	0.6790215	573	376	564	423	506	451
NAT6	0.9017544	-0.149194	0.8141602	67	100	118	94	84	79
NASP	1.047619	0.0671142	0.222685	14478	12876	17754	14435	17031	15790
NAT1	1.1856148	0.2456354	0.1886823	136	106	189	193	155	163
NAT2	1.1969697	0.2593866	0.5559033	16	25	25	20	30	29
SNORD27	#DIV/0!	#DIV/0!	0.3981469	0	0	0	4	0	0
SNORD27	#DIV/0!	#DIV/0!	0.3981469	0	0	0	4	0	0
SNORD20	0.4444444	-1.169925	0.4846278	1	3	14	0	8	0
NAT9	0.8555748	-0.225034	0.4271309	414	613	704	521	518	442
ARHGEF34P	0.7972637	-0.326871	0.1991555	256	287	261	160	204	277
RND2	1.5989848	0.6771562	0.0129749	51	60	86	97	97	121
RND3	0.9463807	-0.079507	0.9616471	135	103	135	124	112	117
RND1	1.1923077	0.2537566	0.6742224	11	7	8	20	4	7
SNORD58	#DIV/0!	#DIV/0!	0.5371397	0	0	0	0	0	3
SNORD56	0.7777778	-0.36257	0.8236607	7	0	11	0	7	7
SNORD56	0.7777778	-0.36257	0.8236607	7	0	11	0	7	7
SNORD56	0.7777778	-0.36257	0.8236607	7	0	11	0	7	7
SNORD56	0.7777778	-0.36257	0.8236607	7	0	11	0	7	7
SNORD56	0.7777778	-0.36257	0.8236607	7	0	11	0	7	7
SNORD56	0.7777778	-0.36257	0.8236607	7	0	11	0	7	7
SNORD56	0.7777778	-0.36257	0.8236607	7	0	11	0	7	7
FBXL19-AS1	0.5465839	-0.871485	0.0064453	91	110	121	48	59	69
SNORD53	#DIV/0!	#DIV/0!	0.8030358	0	0	0	1	0	0
NAV1	0.733546	-0.447041	0.011204	11200	12073	12144	5733	9210	11037
NAV2	0.8242615	-0.278826	0.1052239	2159	2110	2400	1334	1952	2211
TAPBP	0.969842	-0.044178	0.8858855	1847	2031	2389	1941	2209	1928
NAV3	2.5	1.3219281	0.3036234	3	0	3	8	3	4
SNORD46	1.2173913	0.283793	0.7570903	8	8	7	2	12	14
SNORD46	1.2173913	0.283793	0.7570903	8	8	7	2	12	14
SNORD46	1.2173913	0.283793	0.7570903	8	8	7	2	12	14
NAXE	0.7451623	-0.424374	0.0377834	979	1166	1214	942	866	695
RNF8	0.9126357	-0.131889	0.6895761	626	634	766	654	635	560
RNF6	1.0110331	0.0158302	0.7874192	799	442	753	446	831	739
RSPH9	0.9846154	-0.022368	0.9726291	31	18	16	20	16	28
RNF7	0.985886	-0.020507	0.743648	1054	1269	1503	1267	1352	1153
RNF4	0.9466398	-0.079112	0.8338605	3575	3905	4439	3402	4136	3745
RNF5	2.9565217	1.5639009	0.0198627	3	10	10	26	26	16
RNF2	1.1141561	0.1559514	0.1056239	1743	1602	1946	1572	2308	2015
UNC5B-AS1	0	#NUM!	0.2883218	0	0	7	0	0	0
SNORD71	0	#NUM!	0.2290435	7	0	0	0	0	0
SNORD72	0.3	-1.736966	0.3957388	1	2	7	0	3	0
SNORD70	3	1.5849625	0.4907591	2	0	0	4	0	2
SNORD70	3	1.5849625	0.4907591	2	0	0	4	0	2
AP002812.2	#DIV/0!	#DIV/0!	0.0426047	0	0	0	3	4	4
RSPH3	0.6258206	-0.676179	0.0116859	151	154	152	76	110	100
AP002812.3	#DIV/0!	#DIV/0!	0.8030358	0	0	0	0	0	1
RSPH1	0.4	-1.321928	0.5163281	5	0	5	0	4	0
AP002812.1	9	3.169925	0.165035	1	0	0	7	2	0
AP003390.1	1.4074074	0.49304	0.5689402	3	16	8	5	19	14
AP002812.4	#DIV/0!	#DIV/0!	0.6317974	0	0	0	1	1	0

AP002812.5	0.5	-1	0.6904094	0	0	10	0	1	4
SNORD69	0.5	-1	0.5622709	4	0	14	0	2	7
SNORD67	1.8333333	0.8744691	0.4137925	5	0	7	4	9	9
SNORD67	1.8333333	0.8744691	0.4137925	5	0	7	4	9	9
NAXD	0.8542308	-0.227302	0.1705491	1580	1693	1927	1413	1568	1461
SNORD63	0.125	-3	0.2727783	8	0	0	0	0	1
SNORD63	0.125	-3	0.2727783	8	0	0	0	0	1
SNORD63	0.125	-3	0.2727783	8	0	0	0	0	1
RNH1	0.8406059	-0.250499	0.2042137	3229	3971	4419	3448	3218	3101
AC091078.1	0	#NUM!	0.6840298	0	0	2	0	0	0
ADARB1	0.9142857	-0.129283	0.66206	271	288	316	217	287	296
ADARB2	0.2	-2.321928	0.2528227	7	2	1	0	0	2
AC104581.3	#DIV/0!	#DIV/0!	0.5070017	0	0	0	3	0	0
AC104581.4	0.8684211	-0.203533	0.8521946	10	12	16	9	9	15
AL031708.1	1.8695652	0.9027028	0.3119288	12	8	3	21	1	21
SNORD99	0.7222222	-0.469485	0.6249125	17	5	14	5	19	2
SNORD93	1	0	0.907326	4	3	5	9	2	1
SNORD94	0.9767442	-0.033947	0.9637378	14	12	17	14	17	11
DDX17	0.8702071	-0.200569	0.2455706	16230	13887	17636	10235	15144	16176
DDX18	0.9862351	-0.019997	0.8181203	4750	3303	5169	3405	4999	4636
DDX11	0.908647	-0.138208	0.5224901	2014	1991	2344	1877	2063	1829
DDX10	0.9718449	-0.041202	0.9032991	1462	927	1660	1067	1469	1399
AC120498.4	0.7474048	-0.420038	0.2230766	82	108	99	45	83	88
AC120498.5	#DIV/0!	#DIV/0!	0.2164877	0	0	0	4	2	0
AC120498.7	0.125	-3	0.2282852	1	2	5	0	0	1
SNORD89	2	1	0.7736272	0	1	0	2	0	0
PDXDC1	0.8302479	-0.268386	0.0500725	5691	6002	6457	4177	5583	5309
AC083973.1	0	#NUM!	0.824326	1	0	0	0	0	0
NTHL1	0.9134045	-0.130674	0.8303972	265	253	325	312	229	229
EHD1	1.0974227	0.1341194	0.1647355	2033	2673	2705	2591	2954	2588
EHD2	0.7490909	-0.416787	0.2273448	88	105	82	46	77	83
EHD3	2.6444444	1.4029647	0.003089	13	15	17	36	41	42
EHD4	1.0277073	0.0394294	0.5182582	1367	1729	1632	1483	1703	1673
AC011921.1	0.5217391	-0.938599	0.0731695	33	35	47	13	19	28
ITPA	0.8846254	-0.176861	0.475103	1101	1225	1505	1183	1216	990
AC011921.3	0	#NUM!	0.4217812	0	0	5	0	0	0
SERPINA12	#DIV/0!	#DIV/0!	0.4501902	0	0	0	0	0	4
AC008050.1	0	#NUM!	0.0256679	8	7	0	0	0	0
RBPMS2	0.672209	-0.573018	0.0661264	109	152	160	94	117	72
JARID2	1.154575	0.2073619	0.0786065	2268	2263	2540	1984	2957	3223
MIR663AHG	#DIV/0!	#DIV/0!	0.8030358	0	0	0	1	0	0
AL391646.1	#DIV/0!	#DIV/0!	0.8030358	0	0	0	0	1	0
NBAS	0.9197975	-0.120612	0.5922928	1225	1214	1314	861	1236	1355
RNF219-AS1	0	#NUM!	0.4117982	0	3	1	0	0	0
RNMT	1.0010519	0.0015168	0.731489	1061	673	1118	769	1079	1007
FENDRR	#DIV/0!	#DIV/0!	0.5082165	0	0	0	1	0	2
NECTIN3-AS1	1	0	0.9259717	10	0	9	8	7	4
AC097103.2	0.8352941	-0.259644	0.6556025	23	38	24	20	32	19
AC097103.1	#DIV/0!	#DIV/0!	0.5509701	0	0	0	0	3	0
RNLS	2	1	0.7855869	0	1	0	1	1	0
RNMTL1P2	0	#NUM!	0.6436235	2	0	0	0	0	0
MALINC1	1.2178771	0.2843685	0.299558	44	52	83	70	90	58
SERF1A	2.9090909	1.5405684	0.0275876	6	6	21	8	60	28
SERF1B	1.0325203	0.0461702	0.7261741	28	39	56	64	30	33

HLCS-IT1	#DIV/0!	#DIV/0!	0.3981469	0	0	0	4	0	0
PAGE2B	1	0	0.9890559	0	0	1	0	1	0
OR52X1P	#DIV/0!	#DIV/0!	0.8030358	0	0	0	0	0	1
AL136380.1	0	#NUM!	0.824326	1	0	0	0	0	0
KCNH1-IT1	#DIV/0!	#DIV/0!	0.5509701	0	0	0	0	3	0
POLR3GL	0.8710938	-0.1991	0.6696442	74	91	91	84	80	59
RPL41P1	1.0321088	0.045595	0.4022826	6644	8352	9639	8595	9093	7738
RPL41P2	0.5	-1	0.5481763	5	3	2	2	3	0
AL049840.1	0.6763485	-0.564161	0.1478683	67	89	85	37	47	79
AL049840.2	0.5769231	-0.793549	0.2470521	14	27	37	19	15	11
AL049840.3	0.943662	-0.083658	0.9344934	24	24	23	11	14	42
NBDY	1.1975501	0.260086	0.0146272	1382	1470	1638	1745	2006	1626
AL049840.4	0.9393939	-0.090198	0.974096	218	185	323	236	234	212
NBEA	0.7671233	-0.38247	0.397771	106	50	63	37	45	86
AL049840.5	0.8292683	-0.270089	0.7981543	14	7	20	11	14	9
AL049840.6	1	0	0.9617562	0	0	2	0	0	2
RPL41P5	1.1159888	0.1583226	0.1450242	1255	1649	1743	1764	1896	1526
DIDO1	0.7677559	-0.38128	0.0070179	4577	4879	5356	2960	4137	4275
RNU6-1146P	#DIV/0!	#DIV/0!	0.8030358	0	0	0	0	0	1
RNU6-46P	0	#NUM!	0.824326	0	0	1	0	0	0
CEBPA-AS1	0.9456	-0.080698	0.9258527	195	165	265	162	245	184
MPRIP-AS1	0.25	-2	0.547139	0	4	0	0	1	0
GRTP1-AS1	0.4814815	-1.054448	0.3501774	8	3	16	2	7	4
AL590369.1	0.75	-0.415037	0.8804193	4	1	3	5	1	0
RNU6-1147P	#DIV/0!	#DIV/0!	0.8030358	0	0	0	0	1	0
FAM53B-AS1	0	#NUM!	0.4460243	4	0	0	0	0	0
AC112178.1	1.0588235	0.0824622	0.8339038	10	12	12	14	11	11
RNU6-45P	0	#NUM!	0.3473206	0	5	0	0	0	0
AC012020.1	0.4545455	-1.137504	0.6078262	1	0	10	2	3	0
EI24	0.8044256	-0.313969	0.0277568	5960	6523	7085	5092	5583	5066
AC026124.2	0.6666667	-0.584963	0.6186352	4	9	5	4	5	3
AC009275.1	1.0988593	0.1360067	0.4942361	86	72	105	78	112	99
CYP3A4	#DIV/0!	#DIV/0!	0.8030358	0	0	0	0	1	0
CYP3A5	0.8278689	-0.272526	0.6581588	39	33	50	15	25	61
BX322639.1	4.7142857	2.2370392	0.038121	2	0	5	8	7	18
CYP3A7	1.1	0.1375035	0.9408469	4	6	0	2	2	7
CEACAM20	0	#NUM!	0.5075476	0	0	4	0	0	0
AP000894.4	0.6153846	-0.70044	0.511606	6	10	10	7	6	3
AC007953.1	0	#NUM!	0.824326	0	0	1	0	0	0
AC007953.2	#DIV/0!	#DIV/0!	0.8030358	0	0	0	0	1	0
CEACAM19	0.5287356	-0.919382	0.1058879	29	33	25	11	17	18
AC027097.1	0.5652174	-0.823122	0.4545294	10	7	6	0	9	4
AC027097.2	0.4761905	-1.070389	0.3913692	2	9	10	4	3	3
AC074138.1	0.5365854	-0.89812	0.2884557	16	8	17	5	8	9
METTL1	0.7522559	-0.410704	0.0665746	325	446	448	302	293	322
METTL3	1.1000805	0.1376092	0.0941508	1595	1529	1842	1615	1936	1912
TXLNGY	0	#NUM!	0.6840298	0	0	2	0	0	0
AC004080.4	0	#NUM!	0.0921393	0	8	2	0	0	0
FLJ13224	0.1666667	-2.584963	0.4507335	0	0	6	0	0	1
CYP2U1	0.9635036	-0.053638	0.9522597	49	40	48	26	60	46
KATNAL1	0.8918919	-0.165059	0.9340804	10	8	19	11	7	15
KATNAL2	1.0357143	0.0506261	0.6252964	126	143	179	135	168	161
NBL1	#DIV/0!	#DIV/0!	0.6554062	0	0	0	0	2	0
AC093865.1	#DIV/0!	#DIV/0!	0.6099294	0	0	0	2	0	0

RNU6-1136P	0	#NUM!	0.824326	0	0	1	0	0	0
RALGDS	1.1917715	0.2531077	0.0232181	881	945	1115	1210	1163	1132
AC087498.1	0	#NUM!	0.6436235	2	0	0	0	0	0
CYP2S1	0.6168052	-0.697113	0.0037293	285	409	389	249	207	212
ROBO3	0.7	-0.514573	0.6852856	23	1	26	20	6	9
ROBO1	0.8492041	-0.235817	0.3066352	1242	917	1296	592	1110	1232
AL138686.1	1.5	0.5849625	0.6573129	2	1	3	3	1	5
AC104248.1	#DIV/0!	#DIV/0!	0.8030358	0	0	0	0	1	0
IGHV7-40	#DIV/0!	#DIV/0!	0.0407286	0	0	0	5	8	0
AL139274.2	1.3111111	0.39079	0.4834301	19	13	13	16	23	20
METTL4	0.9376855	-0.092824	0.8596593	195	204	275	171	247	214
METTL5	1.0397167	0.0561905	0.3638444	1012	884	1352	1047	1234	1096
METTL6	0.9176471	-0.123989	0.639433	531	576	593	480	542	538
METTL8	1.007535	0.0108299	0.7166661	689	503	666	504	697	671
METTL9	0.9562621	-0.064522	0.9186104	2038	2226	2435	1886	2322	2198
SCGB1B2P	#DIV/0!	#DIV/0!	0.8030358	0	0	0	0	1	0
AL831737.1	0	#NUM!	0.6840298	0	0	2	0	0	0
NBR2	0.922043	-0.117094	0.7682789	119	122	131	67	128	148
CYP2W1	0.4545455	-1.137504	0.5477945	2	9	0	4	0	1
NBR1	0.8924423	-0.164169	0.3505911	2235	2392	2690	1721	2406	2403
ZFHX2-AS1	1	0	0.9426297	2	0	4	2	2	2
AP005119.1	0.0909091	-3.459432	0.104559	4	6	1	0	0	1
AP005119.2	0.5774648	-0.792195	0.2615632	41	11	19	5	25	11
COL4A3BP	1.1095213	0.1499374	0.2693574	1415	1022	1407	961	1710	1594
RPS6P25	0.2647059	-1.917538	0.0576341	13	10	11	1	4	4
AC069185.1	0.7142857	-0.485427	0.8853618	0	0	7	0	1	4
AC067863.3	#DIV/0!	#DIV/0!	0.6411933	0	0	0	0	0	2
RNASEH1P1	1	0	0.9982489	29	31	14	19	38	17
RNASEH1P2	2	1	0.7782384	0	0	1	2	0	0
RPS6P16	#DIV/0!	#DIV/0!	0.6411933	0	0	0	0	0	2
AP001148.1	0	#NUM!	0.824326	0	0	1	0	0	0
AL356512.1	0.4545455	-1.137504	0.6064861	0	8	3	5	0	0
AC140063.1	#DIV/0!	#DIV/0!	0.8030358	0	0	0	0	1	0
CYP2J2	1.6122449	0.6890709	0.0604342	22	31	45	53	55	50
CAPRIN1	0.9524225	-0.070326	0.8297968	10710	9090	11118	7364	11161	10922
CAPRIN2	0.9181034	-0.123271	0.837375	279	130	287	187	210	242
AC016866.1	0.2	-2.321928	0.4777503	1	0	4	0	1	0
AC016866.3	#DIV/0!	#DIV/0!	0.6411933	0	0	0	0	0	2
AC092490.1	1.0333393	0.047314	0.4083118	1671	1891	2047	1747	2153	1896
POM121L9P	0.9090909	-0.137504	0.9281839	4	15	14	9	10	11
CYP2R1	2.5	1.3219281	0.5833197	2	0	0	3	0	2
AC092490.3	#DIV/0!	#DIV/0!	0.6411933	0	0	0	0	0	2
AC007619.1	0	#NUM!	0.5337954	0	3	0	0	0	0
AC007619.2	#DIV/0!	#DIV/0!	0.8030358	0	0	0	0	0	1
AL353133.1	0	#NUM!	0.5781912	0	0	3	0	0	0
VAC14	0.7824186	-0.353987	0.012963	1849	2048	2280	1446	1695	1692
RPL18AP7	0.6666667	-0.584963	0.830659	0	2	4	0	0	4
WASHC5-AS1	3.25	1.7004397	0.351892	3	1	0	0	6	7
VIM-AS1	#DIV/0!	#DIV/0!	0.4501902	0	0	0	0	0	4
RPL18AP2	#DIV/0!	#DIV/0!	0.3096126	0	0	0	5	0	0
RPL18AP3	1.0642061	0.0897775	0.3219207	2913	3568	3923	4160	3960	2952
AC079328.1	0	#NUM!	0.824326	0	0	1	0	0	0
AC100860.1	0.75	-0.415037	0.8399429	4	0	8	0	2	7
LPIN1	0.7885496	-0.342727	0.0850931	849	853	918	447	769	850

LPIN2	0.7973651	-0.326688	0.1000201	558	484	552	318	455	498
LPIN3	0.8624031	-0.213566	0.4201356	293	339	400	275	278	337
AP000897.2	0	#NUM!	0.6436235	2	0	0	0	0	0
CYP4F30P	0.1176471	-3.087463	0.0794101	2	8	7	0	2	0
NCOA7-AS1	2	1	0.8319548	0	1	0	0	2	0
EID1	0.9536061	-0.068535	0.949597	2428	2040	2839	2113	2615	2240
EID2	0.7130435	-0.487938	0.0236782	289	261	370	179	261	216
EID3	0.530303	-0.915111	0.1018332	31	36	65	32	19	19
NUP210L	2.3333333	1.2223924	0.5493276	0	3	0	3	2	2
HHLA1	#DIV/0!	#DIV/0!	0.6554062	0	0	0	0	2	0
AP005212.2	2	1	0.5765298	3	0	2	2	8	0
HHLA2	0.75	-0.415037	0.9354852	0	0	4	0	0	3
AP005212.3	#DIV/0!	#DIV/0!	0.2164227	0	0	0	3	0	3
HHLA3	0.8535354	-0.228477	0.6154014	56	57	85	40	70	59
TLK2P1	1.0058824	0.0084616	0.8553181	69	28	73	45	68	58
TLK2P2	0	#NUM!	0.1151465	2	5	1	0	0	0
KIAA0040	0.8461538	-0.241008	0.8890611	2	11	13	8	3	11
GHRHR	1.4444444	0.5305147	0.7517706	7	0	2	0	7	6
EIF1	1.0872356	0.1206647	0.1398759	6138	7096	7950	7779	8076	7177
FERMT1	0.9083681	-0.138651	0.5008821	1679	1543	1809	1141	1657	1772
EIF5	0.9353027	-0.096495	0.7198155	9963	7966	10743	7619	9807	9391
EIF6	0.9226123	-0.116204	0.6794683	2392	3100	3282	2610	2994	2491
FERMT2	0.953078	-0.069334	0.88208	1783	1342	1862	1241	1856	1656
FERMT3	1.1084337	0.1485225	0.6971388	25	26	32	18	35	39
NCAN	4	2	0.5325759	0	0	1	0	2	2
EDRF1	1.4174265	0.503274	0.0053331	356	233	398	345	576	478
IFNWP2	#DIV/0!	#DIV/0!	0.8030358	0	0	0	0	1	0
AC116049.2	0.25	-2	0.1656916	7	2	11	0	2	3
AC012513.2	#DIV/0!	#DIV/0!	0.247607	0	0	0	0	2	4
AC012513.3	0.6984127	-0.517848	0.522843	20	25	18	24	6	14
AC012513.1	#DIV/0!	#DIV/0!	0.8030358	0	0	0	0	1	0
ROM1	0.3333333	-1.584963	0.4394908	0	4	5	0	2	1
AC007950.2	0	#NUM!	0.5337954	0	3	0	0	0	0
DIEXF	0.8710602	-0.199156	0.2977241	971	916	1254	728	1022	986
AC090092.1	#DIV/0!	#DIV/0!	0.6099294	0	0	0	2	0	0
AC109927.1	0.2	-2.321928	0.4051918	2	1	2	1	0	0
AL591684.2	3	1.5849625	0.269809	0	0	5	7	1	7
AL591684.3	#DIV/0!	#DIV/0!	0.8030358	0	0	0	0	0	1
SERPINB12	3.5714286	1.8365013	0.1015169	0	2	5	11	5	9
SERPINB13	0	#NUM!	0.824326	0	1	0	0	0	0
PET117	2.25	1.169925	0.5095593	4	0	0	6	1	2
NCDN	0.9605809	-0.058021	0.9785922	389	555	502	446	492	451
CYP4F32P	0.5691057	-0.813231	0.129219	40	23	60	18	26	26
PET100	0.6666667	-0.584963	0.4928907	12	16	20	14	14	4
UGT2B24P	0	#NUM!	0.824326	1	0	0	0	0	0
NCF1	0.8	-0.321928	0.897811	2	6	2	7	1	0
27P1-BPTFP1-	0.75	-0.415037	0.878283	4	0	0	1	0	2
NCF2	1.0526316	0.0740006	0.8511107	3	4	12	7	8	5
NCF4	1.2	0.2630344	0.4284394	32	24	59	60	53	25
RORA	1.4339623	0.5200071	0.2297007	50	17	39	32	71	49
RORB	0	#NUM!	0.824326	1	0	0	0	0	0
TSEN2	0.8061927	-0.310803	0.2056959	293	239	340	215	286	202
Z82246.1	12	3.5849625	0.0882499	0	0	1	1	6	5
MTRF1	1.0485116	0.0683428	0.5632033	347	202	358	266	392	293

ROS1	1	0	0.9890559	0	1	0	0	0	1
AC144568.2	4.3333333	2.1154772	0.2635001	0	2	1	0	10	3
AC144568.1	0	#NUM!	0.2919745	1	0	5	0	0	0
ROR1	0.7656566	-0.385231	0.1410636	356	313	321	132	306	320
ROR2	1.0439669	0.0620759	0.368982	1566	1745	1761	1725	1848	1722
INO80-AS1	3.25	1.7004397	0.2570902	3	0	1	7	2	4
B3GALNT2P1	0	#NUM!	0.5387425	3	0	0	0	0	0
PORCN	0.2666667	-1.906891	0.1106914	8	5	17	1	1	6
HUS1B	1	0	0.8682732	0	4	23	10	10	7
ARRB1	0.9294271	-0.105586	0.6799969	1212	1381	1247	1097	1242	1230
ARRB2	1.0032086	0.0046216	0.6368682	772	965	1068	971	946	897
NCK2	0.943367	-0.084109	0.8155064	1885	2240	2320	1881	2164	2035
NCK1	0.9863269	-0.019862	0.8446619	545	452	612	410	565	612
AC108359.1	0	#NUM!	0.5337954	0	3	0	0	0	0
AC121338.2	0	#NUM!	0.5781912	0	0	3	0	0	0
BIRC5	0.947513	-0.077782	0.8837728	3013	3625	3917	3193	3534	3274
BIRC6	1.0124668	0.0178746	0.7295952	3320	2140	3203	1998	3122	3651
BIRC7	0.2970923	-1.751017	6.61E-13	232	275	284	63	101	71
BIRC2	1.0181237	0.0259128	0.6662607	1091	593	1130	756	1136	973
R3HCC1L	1.2847561	0.3614945	0.0189774	591	389	660	598	864	645
BIRC3	0.9464286	-0.079434	0.9337043	38	32	42	24	42	40
NCLN	0.8937548	-0.162049	0.4990968	2181	2725	2972	2425	2430	2186
RP9P	0	#NUM!	0.6410557	1	1	0	0	0	0
RNU6-1156P	#DIV/0!	#DIV/0!	0.8030358	0	0	0	0	0	1
AL121845.4	0.5	-1	0.8652929	0	0	2	0	1	0
RNU6-1157P	0	#NUM!	0.824326	0	0	1	0	0	0
Z92846.1	4	2	0.5525509	0	1	0	0	0	4
MTND4LP30	0	#NUM!	0.824326	1	0	0	0	0	0
ARRDC3-AS1	0.7948718	-0.331206	0.7588384	8	10	21	1	8	22
AC013244.1	0	#NUM!	0.6840298	0	0	2	0	0	0
AC013244.3	0	#NUM!	0.824326	1	0	0	0	0	0
AC022493.1	#DIV/0!	#DIV/0!	0.8030358	0	0	0	0	0	1
AC022493.2	0	#NUM!	0.824326	0	0	1	0	0	0
HNRNPDP1	0.5	-1	0.8188512	2	0	0	0	1	0
AC022398.1	#DIV/0!	#DIV/0!	0.6099294	0	0	0	2	0	0
CLEC9A	0.5686275	-0.814444	0.3078238	18	14	19	10	16	3
SPTLC1	1.0266387	0.0379286	0.6239403	3400	3196	3427	2413	3966	3911
SPTLC2	0.824106	-0.279098	0.1164694	1911	1672	1926	1096	1621	1823
RSRC1	1.0384413	0.0544196	0.5061176	671	463	765	540	775	657
SPTLC3	1.1497006	0.2012582	0.1796548	338	302	362	287	403	462
NCS1	#DIV/0!	#DIV/0!	0.0388003	0	0	0	6	7	0
AC087499.5	0	#NUM!	0.5337954	0	3	0	0	0	0
AC087499.6	1	0	0.978278	0	0	2	0	2	0
AC087499.3	0	#NUM!	0.5387425	3	0	0	0	0	0
RBBP8NL	1.101335	0.1392533	0.1954652	461	496	691	641	580	594
AC092155.1	1	0	0.9818745	10	4	4	4	5	9
NUFIP1P	0.7619048	-0.392317	0.7851042	5	3	13	5	9	2
NCR1	0.7108753	-0.492332	0.0983593	119	109	149	68	101	99
NCR3	0	#NUM!	0.824326	0	1	0	0	0	0
AC025287.2	0	#NUM!	0.6410557	1	1	0	0	0	0
AC025287.3	0.9032258	-0.146841	0.8656302	8	14	9	0	11	17
AC060766.1	0.5762712	-0.79518	0.2738345	29	10	20	5	15	14
AC060766.4	0.8571429	-0.222392	0.856982	4	10	14	4	8	12
AC060766.5	0.75	-0.415037	0.796666	3	2	7	0	5	4

CREG1	1.0993793	0.1366892	0.0737648	4707	4099	5372	4805	5597	5185
AC060766.6	#DIV/0!	#DIV/0!	0.5509701	0	0	0	0	3	0
CREG2	1.3876404	0.4726338	0.0959597	55	60	63	80	72	95
AC060766.7	0.7	-0.514573	0.7980552	2	3	5	4	3	0
ANKRD36BP2	0.6666667	-0.584963	0.8721476	1	0	5	3	1	0
RPA1	0.9411831	-0.087453	0.736415	7969	8571	9915	6809	9381	8709
RPA2	0.9957584	-0.006132	0.6382346	2096	2179	2562	2112	2381	2315
RSRC2	1.2825403	0.3590041	0.0030133	1850	1323	2228	2049	2491	2387
MTND4LP13	0	#NUM!	0.824326	0	0	1	0	0	0
RPA3	1.0386089	0.0546526	0.3827113	2115	2346	2584	2484	2653	2180
RPA4	0.8148148	-0.295456	0.8154575	15	0	12	1	10	11
RBBP8P1	0	#NUM!	0.284725	6	0	0	0	0	0
AC010595.1	0.5913978	-0.757799	0.0756268	51	56	79	24	55	31
AL008582.1	0.2222222	-2.169925	0.3568878	8	1	0	2	0	0
AC108693.1	0	#NUM!	0.824326	1	0	0	0	0	0
AC108693.2	0.5	-1	0.8254608	0	1	1	0	1	0
AC126768.2	0.4	-1.321928	0.5954536	2	3	0	0	0	2
H3PXD2A-AS	0.8333333	-0.263034	0.9321637	2	0	4	0	1	4
DDX60	6.7728395	2.7597608	5.08E-29	179	68	158	625	1044	1074
RPF2	1.1559934	0.2091332	0.0754388	1697	1161	2014	1782	2143	1707
RPF1	0.9954567	-0.00657	0.6719988	1368	1084	1730	1230	1567	1366
AC006538.1	0.6666667	-0.584963	0.6703384	7	4	1	0	3	5
AC013734.1	0	#NUM!	0.824326	0	1	0	0	0	0
PACSIN2	0.9952272	-0.006902	0.6828307	3185	3506	3785	3105	3654	3667
PACSIN3	1.0728244	0.101414	0.256264	2072	2432	2609	2695	2751	2185
AC007220.1	0	#NUM!	0.5337954	0	3	0	0	0	0
PACSIN1	1	0	0.9890559	0	0	1	0	1	0
IVNS1ABP	1.0334877	0.0475212	0.383983	7518	6462	8476	6329	8536	8343
RNU6-19P	0	#NUM!	0.6604275	0	1	1	0	0	0
RSRP1	1.2312793	0.300158	0.0317194	927	575	1062	942	1167	1048
SERGEF	0.765343	-0.385822	0.3423052	87	96	94	91	66	55
AP001972.4	5	2.3219281	0.121093	1	0	2	7	5	3
AP001972.5	0.8413284	-0.249259	0.5173482	113	92	66	69	105	54
DDX3Y	1.036603	0.0518635	0.6327279	2495	1492	2242	1437	2575	2445
DDX3X	0.9986594	-0.001935	0.8225835	10510	7912	9924	6546	10725	11037
DDX49	0.8814059	-0.182122	0.4478283	1460	1665	1968	1583	1612	1294
DDX46	1.1948787	0.2568641	0.0591731	3939	2358	4130	3230	4707	4522
DDX47	0.9166667	-0.125531	0.9603746	12	5	19	8	10	15
AC211476.3	#DIV/0!	#DIV/0!	0.8030358	0	0	0	0	0	1
AC211476.1	0	#NUM!	0.6436235	2	0	0	0	0	0
DDX42	0.8859383	-0.174722	0.2539572	7445	7034	8605	5746	7403	7302
AC211476.2	3.1666667	1.662965	0.1816145	2	0	4	12	5	2
DDX43	0.828201	-0.271947	0.3635022	237	141	239	135	212	164
ARHGEF10L	1.1202749	0.1638528	0.3345759	133	205	244	176	249	227
DDX41	0.9198473	-0.120534	0.6322707	4908	5981	6665	5161	5817	5169
RPGR	1.2580645	0.3312059	0.192613	162	67	174	125	209	173
AC007126.1	#DIV/0!	#DIV/0!	0.8030358	0	0	0	0	1	0
AP001005.4	0.9347826	-0.097297	0.935742	10	22	14	13	13	17
DDX59	0.9626338	-0.054941	0.9910818	1835	1615	2063	1541	2056	1710
DDX58	4.8058058	2.2647784	2.42E-41	396	274	329	1243	1953	1605
DDX55	0.8566315	-0.223253	0.2005274	1659	1394	1976	1301	1568	1439
DDX56	0.7995154	-0.322802	0.0328205	2418	2539	2884	2039	2302	1928
AC068594.2	0	#NUM!	0.824326	0	0	1	0	0	0
DDX53	2.5	1.3219281	0.1620932	4	0	12	5	13	22

DDX54	0.7705966	-0.375952	0.0178683	2761	3348	3747	2524	2587	2484
DDX51	0.7543554	-0.406684	0.0231595	806	972	1092	730	729	706
DDX52	0.925026	-0.112434	0.6631095	1700	1297	1818	1228	1679	1547
DDX50	1.2219101	0.2891382	0.0180476	1501	1035	1736	1505	1883	1832
AC068594.1	0.3333333	-1.584963	0.5086907	0	5	1	0	2	0
CREB3	1.0310118	0.0440608	0.4241777	1628	1983	2161	1933	2102	1916
CREB1	1.2203542	0.2872999	0.0165106	1568	1231	1662	1408	2105	1931
SAXO1	1	0	0.9890559	0	0	1	0	0	1
SAXO2	0.7727273	-0.371969	0.7153	12	10	22	15	13	6
CREB5	2.0055866	1.0040243	0.0003242	55	58	66	98	113	148
DDX28	0.8311938	-0.266743	0.2958236	455	560	543	491	437	367
DDX27	0.8864407	-0.173904	0.3283739	3339	3595	4276	3104	3728	3105
DDX24	0.9366937	-0.094351	0.7251508	6576	7049	8079	6167	7256	6907
DDX25	0	#NUM!	0.4374838	0	4	0	0	0	0
CYP4F29P	0.7222222	-0.469485	0.7189403	8	3	7	5	6	2
DDX23	0.8559717	-0.224365	0.1507048	4315	5084	5605	3800	4515	4528
DDX20	0.9009871	-0.150422	0.4368214	1094	1044	1205	807	1118	1087
DDX21	1.1053888	0.1445539	0.2978973	6479	4024	6254	4127	6805	7591
AC092645.1	0	#NUM!	0.824326	0	0	1	0	0	0
TARID	#DIV/0!	#DIV/0!	0.5230806	0	0	0	1	2	0
DDX31	1.0851984	0.1179588	0.1856833	1191	1349	1392	1358	1513	1396
MYO16-AS1	0	#NUM!	0.824326	0	1	0	0	0	0
RPIA	1.029563	0.0420321	0.3909283	1199	1170	1521	1233	1437	1335
ATP6V1B1-AS	0.5	-1	0.7332684	0	3	3	3	0	0
KATNBL1	0.9324631	-0.100881	0.8449679	621	428	713	514	619	510
RPL4	0.9502146	-0.073675	0.9333412	60426	69894	78416	66047	71135	61162
DOC2GP	1.0181818	0.0259952	0.764993	25	22	63	57	41	14
RPL5	1.0747543	0.1040069	0.1475813	33600	34652	42444	38930	44019	36022
RPL3	1.0512317	0.0720807	0.2988326	65224	81672	89071	84160	87959	75937
RPL8	0.9111216	-0.134285	0.6461466	29271	37926	36754	34463	33078	27171
RPL9	1.1094564	0.149853	0.1179473	846	785	1055	917	1135	928
RPL6	1.0735943	0.1024489	0.1613393	33269	30229	41483	37733	41260	33714
RPL7	1.0758012	0.1054115	0.1239064	18073	16818	22430	19626	22651	19389
LCE1B	0	#NUM!	0.824326	1	0	0	0	0	0
AP002957.1	0.4418605	-1.178337	0.1771898	7	19	17	2	8	9
AL358072.1	0.7647059	-0.387023	0.8384532	11	0	6	7	1	5
BISPR	2.6220472	1.3906937	4.65E-06	45	32	50	101	132	100
LCE2B	0.5714286	-0.807355	0.7345563	5	0	2	3	1	0
LCE2C	3	1.5849625	0.6944949	0	0	1	0	3	0
AC005410.2	#DIV/0!	#DIV/0!	0.5509701	0	0	0	0	3	0
LCE2A	0.6222222	-0.684498	0.1708137	49	29	57	29	31	24
AC138150.1	0.4	-1.321928	0.6712586	0	0	5	0	1	1
AC138150.2	#DIV/0!	#DIV/0!	0.1690953	0	0	0	4	3	0
AL450405.1	0.9387472	-0.091191	0.83795	2592	3310	3469	2975	3182	2640
RPN2	0.9675342	-0.047615	0.9093754	13410	14773	17003	13996	16040	13683
RPN1	0.821082	-0.284402	0.0556314	11206	13815	14349	9909	11818	10599
PTPMT1	2.25	1.169925	0.1447881	11	1	8	24	8	13
SLC19A3	1.0525368	0.0738706	0.3499626	2014	2245	2403	1865	2538	2609
AL157838.1	0.90625	-0.142019	0.8462987	38	20	38	9	29	49
SLC19A1	1.5146796	0.5990127	3.79E-06	1249	1325	1343	2068	2037	1828
SLC19A2	1.0758027	0.1054135	0.2632736	1054	917	1050	917	1132	1201
AC107375.1	0.9666667	-0.04891	0.9073746	21	18	51	30	29	28
TADA2B	0.904388	-0.144986	0.5238369	636	748	781	576	696	686
TADA2A	0.824015	-0.279257	0.1042262	922	783	960	614	788	794

AC120114.1	0.5890411	-0.76356	0.2513548	23	27	23	8	9	26
AC120114.2	0.7878788	-0.343954	0.5582295	35	32	65	25	29	50
AC105074.1	0	#NUM!	0.824326	1	0	0	0	0	0
AC120114.4	1.2058824	0.2700892	0.5866023	30	4	34	19	19	44
EP300-AS1	1.1025641	0.1408625	0.7608052	28	23	27	13	41	32
CLINT1	0.9635783	-0.053526	0.9464541	6913	5910	7522	4848	7370	7386
AP005117.1	#DIV/0!	#DIV/0!	0.6099294	0	0	0	2	0	0
AL355922.2	4	2	0.3379486	3	0	0	0	1	11
AL355922.3	0	#NUM!	0.0354907	6	1	6	0	0	0
RSPO4	0.8043478	-0.314109	0.6988175	13	13	20	4	16	17
NDC1	0.9421358	-0.085993	0.7377122	2829	2497	2952	1961	3006	2832
TAPT1-AS1	0.9571429	-0.063194	0.946635	21	23	26	36	17	14
NPAP1P7	2.5	1.3219281	0.2729691	7	0	7	0	19	16
NPAP1P4	0	#NUM!	0.355562	5	0	0	0	0	0
CD200R1	3.8	1.9259994	0.1604603	1	0	4	2	10	7
AC025871.2	0	#NUM!	0.4460243	4	0	0	0	0	0
RARA-AS1	2.0769231	1.0544478	0.2515864	4	5	4	17	5	5
LCE2D	0.45	-1.152003	0.3460856	5	11	4	0	7	2
RPS3	0.9615545	-0.056559	0.9525596	50346	62317	67202	59032	61681	52237
RPS2	1.0051215	0.0073699	0.5906392	70928	90966	98577	92999	90321	78485
RPRM	0.52	-0.943416	0.427954	7	3	15	4	7	2
AC005796.1	#DIV/0!	#DIV/0!	0.6554062	0	0	0	0	2	0
AL355578.1	3	1.5849625	0.6944949	0	0	1	0	3	0
AC012512.1	1.5	0.5849625	0.8175361	2	0	0	3	0	0
NDE1	0.8358341	-0.258712	0.162041	651	764	851	535	670	689
AC027682.6	#DIV/0!	#DIV/0!	0.5230806	0	0	0	1	2	0
AC027682.7	0.2413793	-2.050626	0.1210117	20	3	6	0	7	0
AC027682.1	0.1904762	-2.392317	0.0879369	5	7	9	0	3	1
AC027682.4	1	0	0.9656845	0	2	4	0	0	6
VEPH1	0.4090909	-1.289507	0.3531943	2	4	16	0	5	4
CLEC4M	0.5	-1	0.4819672	4	6	2	1	4	1
RNU6-1165P	0	#NUM!	0.6385096	0	2	0	0	0	0
RPTN	#DIV/0!	#DIV/0!	0.1307332	0	0	0	3	0	5
AC096996.1	#DIV/0!	#DIV/0!	0.6411933	0	0	0	0	0	2
RPS9	1.0681424	0.0951039	0.2837826	17785	23682	24762	25633	24846	20263
RPS7	1.0781827	0.1086017	0.1601046	16080	16762	20776	20160	20381	17269
RPS8	1.0952502	0.1312604	0.1414966	22084	26506	30360	30579	30116	25775
RPS5	0.8881103	-0.171189	0.4490633	22977	29211	30295	25573	26112	21569
RPS6	1.2247509	0.2924883	0.0067705	40523	43859	54603	59383	62218	48621
RPSA	0.9339088	-0.098646	0.7596119	48080	60570	62704	52492	57320	50217
CLEC4A	#DIV/0!	#DIV/0!	0.6554062	0	0	0	0	2	0
CLEC4D	#DIV/0!	#DIV/0!	0.8030358	0	0	0	0	0	1
AC093627.5	0.425	-1.234465	0.1531413	14	17	9	2	10	5
AC093627.4	0	#NUM!	0.5436524	2	0	1	0	0	0
CLEC4F	1.2368421	0.3066613	0.4718501	13	16	47	23	44	27
TMPO-AS1	0.7966102	-0.328054	0.3916606	68	72	96	51	79	58
AL355338.1	0.8876404	-0.171953	0.7281789	215	291	384	311	232	247
AC016134.1	#DIV/0!	#DIV/0!	0.5415473	0	0	0	0	2	1
HOTAIRM1	0.7163462	-0.481271	0.2593556	61	61	86	54	61	34
CLEC7A	0.8245614	-0.278301	0.7929087	18	15	24	16	4	27
SEPT14P4	1.25	0.3219281	0.8202793	7	4	1	1	4	10
MPDU1	0.9186972	-0.122339	0.7712277	1117	1388	1517	1394	1242	1059
RNU6-1164P	1	0	0.9890559	1	0	0	0	0	1
CLEC6A	0	#NUM!	0.824326	0	0	1	0	0	0

AC004967.1	0.921875	-0.117357	0.9321542	25	18	21	24	18	17
PTPN20	0.6363636	-0.652077	0.7595558	3	1	7	5	1	1
AC090739.1	1.4375	0.523562	0.6689864	7	5	4	0	3	20
PTPN21	1.120085	0.1636082	0.2236448	609	631	642	509	801	798
PTPN22	1.1111111	0.1520031	0.8570453	4	3	11	0	6	14
PTPN23	0.6873099	-0.540967	0.0006708	1038	1257	1322	678	876	932
CLEC1A	0.9946568	-0.007729	0.6478624	2719	2665	3225	2561	3039	2963
AC106786.1	1.8181818	0.8624965	0.3289105	5	12	5	4	13	23
AC106786.2	1	0	0.9988518	1	4	3	0	3	5
TAPT1	0.997114	-0.00417	0.8170094	246	168	279	175	271	245
CLEC2L	1	0	0.9544849	0	0	3	0	0	3
NDNF	0	#NUM!	0.824326	1	0	0	0	0	0
CLEC3A	1.1390728	0.18786	0.4997826	64	29	58	76	48	48
HIRIP3	0.9852071	-0.021501	0.7580622	441	543	706	556	585	524
PTPN18	0.9055539	-0.143128	0.543771	1851	2189	2694	1909	2224	1965
AHNAK2	0.5	-1	0.2674661	17	13	14	4	2	16
AC009032.1	0	#NUM!	0.5387425	3	0	0	0	0	0
PTPN11	0.9219268	-0.117276	0.6162361	5025	3749	4803	2869	4725	4923
PTPN12	1.1512225	0.2031667	0.0966887	1819	1252	1796	1469	2054	2080
PTPN13	1.7472119	0.8050546	0.0028785	87	51	131	126	191	153
PTPN14	0.912408	-0.132249	0.5670269	3400	3248	3273	1971	3085	3996
CLEC2A	0	#NUM!	0.5781912	0	0	3	0	0	0
CLEC2B	1.5	0.5849625	0.7734914	0	0	2	3	0	0
AL137127.1	1.8947368	0.9219975	0.3115892	3	13	3	8	11	17
CLEC2D	0.8396226	-0.252187	0.7048135	40	17	49	18	34	37
HNRNPCP3	#DIV/0!	#DIV/0!	0.8030358	0	0	0	0	1	0
HNRNPCP4	2.25	1.169925	0.4678927	1	2	1	6	0	3
HNRNPCP6	0.75	-0.415037	0.8609427	2	1	1	1	2	0
HNRNPCP7	1.4	0.4854268	0.6964494	13	2	0	6	8	7
RNASEH1-AS	0.9878665	-0.017612	0.8195357	311	316	362	307	362	308
AC040173.1	2	1	0.5540096	0	6	0	4	5	3
AC087749.1	0	#NUM!	0.2359908	1	0	6	0	0	0
AC087749.2	1	0	0.9890559	1	0	0	0	0	1
HNRNPCP1	1.0673077	0.0939761	0.7270635	30	29	45	36	52	23
HNRNPCP2	1.0296412	0.0421417	0.4321836	756	813	995	845	900	895
DTX2P1	2	1	0.7741804	1	0	0	2	0	0
KIFAP3	0.8859701	-0.17467	0.5351124	598	417	660	481	531	472
RNU6-26P	0	#NUM!	0.1312466	4	0	5	0	0	0
AL512656.1	#DIV/0!	#DIV/0!	0.1690953	0	0	0	4	3	0
CEP57L1	1.292113	0.3697322	0.0527094	421	202	404	431	492	404
CRYZL1	1.1294919	0.175674	0.1159675	540	483	591	569	677	577
AURKAIP1	1.0924966	0.1276288	0.2074611	2169	2430	2731	3049	2745	2214
OR8K4P	4	2	0.5760976	1	0	0	0	4	0
AC139769.3	1.0769231	0.1069152	0.9522717	2	7	4	0	8	6
AC139769.1	1.1014493	0.1394031	0.378555	237	167	217	188	259	237
AC139769.2	1.0769231	0.1069152	0.9256694	3	5	5	1	8	5
HM13-AS1	8.6666667	3.1154772	0.0300063	1	2	0	21	1	4
SMIM11A	2.3333333	1.2223924	0.5259522	0	1	2	3	4	0
CYP4X1	0	#NUM!	0.6385096	0	2	0	0	0	0
SMIM11B	0.6666667	-0.584963	0.8317553	2	1	0	1	1	0
AC119427.1	0.7941176	-0.332575	0.5463619	32	33	37	26	32	23
CMYA5	1.3685714	0.4526707	0.0315234	123	105	122	147	169	163
RNU6-24P	#DIV/0!	#DIV/0!	0.6554062	0	0	0	0	2	0
LRP2BP	2.0649351	1.0460964	0.0419689	34	10	33	21	78	60

HNRNPCL1	1	0	0.9647111	1	2	0	0	3	0
HNRNPCL2	0	#NUM!	0.6631647	1	0	1	0	0	0
AC080112.3	4	2	0.1831706	1	0	3	13	0	3
BICDL1	0.5046904	-0.986529	1.14E-05	314	396	356	187	162	189
BICDL2	1.3333333	0.4150375	0.0369412	132	125	154	199	175	174
PP2D1	0.52	-0.943416	0.3820375	9	5	11	3	4	6
AC080112.1	0.7124304	-0.489179	0.0828843	137	189	213	125	150	109
AL035427.1	4	2	0.4779902	0	1	0	1	1	2
CRYZP2	#DIV/0!	#DIV/0!	0.3096126	0	0	0	5	0	0
CRYZP1	0.6190476	-0.691878	0.5246229	12	4	5	2	7	4
TRIM43CP	16	4	0.0662185	0	0	1	0	14	2
PTCHD1	#DIV/0!	#DIV/0!	0.8030358	0	0	0	1	0	0
CILP2	0.4302326	-1.216811	0.0500338	23	33	30	16	11	10
PTCHD4	0.7837838	-0.351472	0.1490232	223	206	237	155	175	192
BX546450.2	1.2	0.2630344	0.8340277	1	1	3	2	0	4
GDF10	1	0	0.9756864	2	0	0	0	2	0
GDF11	0.6771181	-0.562521	0.0022317	429	475	595	311	382	322
BX546450.1	0	#NUM!	0.824326	1	0	0	0	0	0
DMRT1	1	0	0.894647	0	7	12	9	1	9
DMRT2	1.3505155	0.4335102	0.2493355	37	26	34	55	31	45
GDF15	1.0659513	0.0921416	0.2446536	3054	3616	4141	4097	3902	3525
AL390816.2	0	#NUM!	0.824326	0	0	1	0	0	0
GNAQP1	#DIV/0!	#DIV/0!	0.5371397	0	0	0	0	0	3
AL590617.2	0.9734513	-0.038819	0.9242441	59	79	88	85	86	49
FLJ37035	1.1666667	0.2223924	0.8629039	1	0	5	0	2	5
SERHL2	0.6570156	-0.606	0.0349291	164	114	171	80	102	113
TTC21B-AS1	1	0	0.9565098	2	0	1	2	0	1
NUP50-AS1	0.5990521	-0.739247	0.0005011	308	317	430	198	253	181
AC006539.2	0.5789474	-0.788496	0.5155887	5	5	9	3	5	3
AL356020.1	0.5	-1	0.8210345	0	1	1	1	0	0
AC010210.1	2.1428571	1.0995357	0.3852824	1	4	2	5	2	8
AC012511.1	0	#NUM!	0.6604275	0	1	1	0	0	0
PPP4R3A	1.0518213	0.0728896	0.355257	4756	3400	5159	3762	5189	5054
SMIM2-AS1	1.3333333	0.4150375	0.6759054	1	2	6	10	2	0
PPP4R3B	1.2023397	0.2658445	0.0556465	2573	1613	2738	2077	3078	3170
AC079174.2	0.6923077	-0.530515	0.7105144	4	4	5	2	2	5
MPEG1	1	0	0.9890559	1	0	0	0	0	1
PHOSPHO1	1.6	0.6780719	0.4230511	12	4	4	12	5	15
PHOSPHO2	1.2929293	0.3706434	0.2025583	63	64	71	99	53	104
AL157932.1	2	1	0.8324799	1	0	0	0	2	0
MYCNOS	1.3047776	0.3838039	0.0288878	190	173	244	203	297	292
PPP4R1L	0.9909707	-0.013086	0.8764478	147	138	158	117	134	188
RNU6-5P	0	#NUM!	0.824326	0	0	1	0	0	0
B4GALT2	0.8885794	-0.170427	0.681578	196	234	288	238	190	210
B4GALT3	1.0340393	0.0482911	0.4549266	1218	1378	1370	1362	1455	1284
B4GALT1	0.9397806	-0.089604	0.7057173	11033	12686	12648	9159	12448	12570
TMX2P1	1.0519481	0.0730635	0.6092166	95	90	123	93	108	123
CYP4V2	2.1818182	1.1255309	0.0260602	15	14	15	38	27	31
MYCNUT	1.2325581	0.3016557	0.2252116	113	94	94	101	109	161
AL080250.1	1.0714286	0.0995357	0.8093997	21	14	35	8	34	33
KLF10	0.8533649	-0.228765	0.1666535	1520	1781	1766	1236	1565	1523
KLF11	0.8198547	-0.28656	0.1101045	609	653	803	498	610	585
KLF12	0.7651123	-0.386257	0.1654334	193	172	214	88	173	182
KLF13	0.9468631	-0.078772	0.8017498	1379	1613	1487	1102	1435	1704

KLF18	2	1	0.807847	1	0	0	0	1	1
KLF14	0.5	-1	0.5327191	7	2	3	0	6	0
KLF15	1.2067371	0.2711114	0.0350934	456	481	577	630	652	545
KLF16	0.984787	-0.022116	0.7548608	622	604	746	718	632	592
AL603750.1	1.2937853	0.3715982	0.2280278	61	33	83	111	64	54
B4GALT6	0.7593361	-0.397189	0.2858135	77	71	93	40	67	76
B4GALT7	0.8254301	-0.276782	0.1574931	1340	1497	1929	1350	1408	1176
B4GALT4	0.9445863	-0.082245	0.8470341	1217	1232	1467	1144	1334	1221
B4GALT5	0.8409596	-0.249892	0.1330238	7269	8050	8524	4779	7489	7783
IWS1	1.2789807	0.3549945	0.0008144	2837	2241	3281	3298	3921	3472
RMND5B	0.84151	-0.248948	0.2370634	1851	2166	2526	1995	1841	1670
RMND5A	1.0430715	0.0608381	0.387102	3563	3354	3879	2942	4205	4114
LARS2-AS1	0	#NUM!	0.824326	1	0	0	0	0	0
AC025048.4	0	#NUM!	0.5337954	0	3	0	0	0	0
AC025048.6	1	0	0.9741133	2	1	5	0	4	4
TCAF1P1	0.6174242	-0.695666	0.1385609	81	64	119	17	53	93
AC025048.2	1.0357143	0.0506261	0.8681714	20	17	19	16	23	19
THSD4-AS1	0.25	-2	0.547139	0	4	0	0	1	0
RNU6-787P	0	#NUM!	0.6410557	1	1	0	0	0	0
AC244107.1	2	1	0.7782384	0	0	1	2	0	0
RNU6-786P	0	#NUM!	0.824326	0	0	1	0	0	0
KDELC1P1	#DIV/0!	#DIV/0!	0.6554062	0	0	0	0	2	0
PLBD1-AS1	0	#NUM!	0.4374838	0	4	0	0	0	0
AC092171.2	0.7448873	-0.424906	0.0052638	1113	1256	1445	871	1061	909
AC092171.3	0.8409091	-0.249978	0.6428489	88	71	105	94	52	76
AC092171.4	0.4833333	-1.04891	0.1412937	22	23	15	10	14	5
AC092171.5	0.92	-0.120294	0.9373052	32	15	53	21	35	36
NEBL	1.0231769	0.0330556	0.6708185	655	424	690	423	699	688
TMEM254-AS	1.4598269	0.5457974	0.0006525	261	225	323	402	404	375
AC092171.1	0.6585366	-0.602665	0.4822727	7	16	18	3	11	13
GLDCP1	0.9310345	-0.103093	0.957335	26	16	16	25	15	14
L1TD1	5	2.3219281	0.427622	1	0	0	0	1	4
KCNC1	1.8	0.8479969	0.6180325	1	0	4	0	4	5
KCNC3	1.0736698	0.1025504	0.3130728	493	373	600	459	541	574
KCNC4	0.550885	-0.860177	0.0026763	125	153	174	84	95	70
AL022313.2	#DIV/0!	#DIV/0!	0.1837925	0	0	0	3	1	2
AL022313.3	#DIV/0!	#DIV/0!	0.2887809	0	0	0	0	0	6
ZNF32-AS2	0.7560976	-0.403356	0.6694026	16	9	16	0	11	20
NEFL	0	#NUM!	0.5337954	0	3	0	0	0	0
NEFM	0.5	-1	0.8241474	0	1	1	0	0	1
NEFH	1.010101	0.0144996	0.8419205	63	76	59	76	68	56
ZNF32-AS1	2	1	0.6242561	0	0	3	2	4	0
KCND1	0.8541667	-0.22741	0.6835345	31	60	53	42	46	35
KCND3	1.1875	0.2479275	0.8341074	6	3	7	0	12	7
RPL3P3	0.75	-0.415037	0.9557496	0	1	3	3	0	0
RPL3P4	1.1661929	0.2218065	0.0856182	1628	1969	2384	2754	2382	1839
RPL3P7	0.8181818	-0.289507	0.8669795	1	7	3	4	2	3
EIF1B-AS1	3.5	1.8073549	0.1243941	1	3	4	13	0	15
RPL3P9	#DIV/0!	#DIV/0!	0.6251462	0	0	0	1	0	1
FAM3C2	0.839779	-0.251918	0.5472331	175	92	95	91	73	140
RPL3P1	#DIV/0!	#DIV/0!	0.8030358	0	0	0	0	0	1
AC092421.1	2	1	0.5486858	0	0	4	2	1	5
RPL3P2	1.1666667	0.2223924	0.4263341	55	114	83	78	110	106
ANKRD36P1	0.4	-1.321928	0.6282732	3	0	2	0	0	2

KCNA2	0.6666667	-0.584963	0.8871197	0	0	3	0	2	0
PTAFR	0.4833333	-1.04891	0.2430166	7	9	44	8	11	10
KCNA7	1	0	0.9697717	3	1	3	1	1	5
AC069528.2	1	0	0.9381352	3	0	2	4	1	0
AL137793.1	1.1548757	0.2077376	0.2129335	134	160	229	204	219	181
ARL5AP3	1	0	0.9890559	0	0	1	0	1	0
AL137793.2	1.1470588	0.1979394	0.6627906	7	7	20	18	7	14
ARL5AP2	0	#NUM!	0.5337954	0	3	0	0	0	0
KCNB1	3.5	1.8073549	0.3724872	0	0	2	2	2	3
AL161431.1	0.6363636	-0.652077	0.4305564	24	6	14	7	13	8
KCNB2	2.1818182	1.1255309	0.2732087	2	0	9	11	8	5
AL391832.3	0	#NUM!	0.6604275	0	1	1	0	0	0
KCNG1	0.3875969	-1.367371	4.89E-05	95	121	171	42	43	65
KCNG2	#DIV/0!	#DIV/0!	0.1884392	0	0	0	0	0	8
RASL11B	1.302045	0.3807793	0.0004818	2360	2767	2746	3154	3668	3429
PDLIM1P4	1.3333333	0.4150375	0.6802269	7	1	7	2	7	11
RASL11A	0.3225806	-1.632268	0.1259578	10	6	15	4	4	2
GOLIM4	2	1	0.6301184	1	0	2	0	2	4
PDLIM1P1	#DIV/0!	#DIV/0!	0.5338029	0	0	0	0	1	2
POTEM	0.5	-1	0.7956884	1	1	0	1	0	0
POFUT2	0.9287142	-0.106693	0.7591481	471	428	602	395	549	450
PTAR1	1.1851765	0.245102	0.4340703	1823	921	1533	1051	1878	2140
POTEH	2.25	1.169925	0.2660068	7	4	1	7	5	15
POFUT1	0.7240263	-0.465886	0.0008762	2641	3111	3209	1808	2360	2320
POTEI	0.5517241	-0.857981	0.3826617	13	11	5	1	5	10
POTEJ	0.25	-2	0.5155155	1	3	0	0	1	0
AP003497.2	0	#NUM!	0.824326	0	0	1	0	0	0
NEK1	1.2287582	0.297201	0.1402026	319	163	283	245	386	309
POTEE	0.6730769	-0.571157	0.3213996	33	32	39	8	23	39
TNFRSF8	2.1538462	1.1069152	0.2938608	7	2	4	3	18	7
AC021422.1	0.5	-1	0.7134057	2	0	2	0	1	1
NEK2	1.0137886	0.0197568	0.6176151	1124	799	1123	883	1111	1094
POTEF	0.9375	-0.093109	0.9026641	42	19	19	18	23	34
NEK3	1.0075949	0.0109158	0.7669571	149	108	138	141	128	129
POTEG	1.2	0.2630344	0.7087587	18	9	8	8	11	23
AC097460.2	0	#NUM!	0.6385096	0	2	0	0	0	0
AC097460.3	#DIV/0!	#DIV/0!	0.8030358	0	0	0	1	0	0
TNFRSF4	#DIV/0!	#DIV/0!	0.1752933	0	0	0	2	0	5
AC097460.1	0	#NUM!	0.1616603	4	1	2	0	0	0
KCNH1	1.027027	0.0384741	0.9847034	8	18	11	0	15	23
KCNH3	1.8333333	0.8744691	0.5147432	0	2	4	5	1	5
KCNH4	2	1	0.7782384	0	0	1	2	0	0
KCNH5	1.0746888	0.103919	0.6542896	207	120	155	99	188	231
KCNH6	#DIV/0!	#DIV/0!	0.6411933	0	0	0	0	0	2
KCNH7	0	#NUM!	0.5436524	2	0	1	0	0	0
KCNH8	1.0714286	0.0995357	0.8821375	12	10	6	8	11	11
AL359504.2	1.3012048	0.3798481	0.3389645	27	18	38	42	43	23
DMTF1	1.0765625	0.1064321	0.4162554	1508	790	1542	1011	1622	1501
MIR210HG	1.5954089	0.6739262	0.0003298	217	200	280	395	431	286
KCNE1	7	2.8073549	0.265924	1	0	0	2	0	5
RNU6-781P	8	3	0.2294733	0	1	0	8	0	0
KCNE2	1	0	0.9756864	2	0	0	0	2	0
KCNE3	0.7078477	-0.498489	0.0085219	371	426	490	255	335	321
KCNE4	2	1	0.1626512	22	2	9	20	28	18

KCNE5	0.625	-0.678072	0.6967279	0	7	1	0	3	2
NPAS1	1.9	0.9259994	0.3909708	3	5	2	13	2	4
NPAS2	1.8888889	0.9175378	0.5877913	0	9	0	1	8	8
NPAS3	2	1	0.6653989	0	3	0	2	0	4
AC138965.1	#DIV/0!	#DIV/0!	0.3096126	0	0	0	5	0	0
AC138965.2	0	#NUM!	0.6436235	2	0	0	0	0	0
AC138965.3	0.1666667	-2.584963	0.3802332	0	2	4	0	0	1
NEK8	0.9650171	-0.051374	0.9101826	330	370	472	365	331	435
NEK9	0.9084611	-0.138503	0.459702	6131	6498	7002	4536	6437	6861
NEK4	0.9343034	-0.098037	0.7321265	813	691	764	453	756	910
RASL10A	2.75	1.4594316	0.4271259	0	1	3	0	9	2
NEK5	0.8823529	-0.180572	0.7493459	51	38	47	38	44	38
RASL10B	0	#NUM!	0.824326	1	0	0	0	0	0
NEK6	0.9677342	-0.047317	0.9778956	5003	5701	5877	4650	5503	5893
NEK7	1.0843571	0.1168399	0.380855	1377	950	1336	887	1490	1595
DRAP1	0.823013	-0.281013	0.2462285	1138	1361	1716	1322	1174	973
KCNK5	1.0310442	0.0441062	0.5796491	258	384	421	363	403	330
KCNK6	1.1428571	0.1926451	0.9068007	0	6	1	2	1	5
KCNK7	0.8666667	-0.206451	0.9565698	3	7	20	12	5	9
KCNK9	0.25	-2	0.5148668	2	2	0	0	1	0
ONECUT2	1.1233333	0.1677861	0.5572825	91	95	114	51	149	137
ONECUT3	0	#NUM!	0.1851375	8	0	0	0	0	0
ONECUT1	0.9487179	-0.075949	0.920863	29	25	24	11	24	39
NENF	1.0572738	0.080349	0.4614441	204	271	398	364	313	246
AC004067.1	1.7619048	0.8171359	0.347057	13	0	8	13	11	13
TNFSF13B	0.8571429	-0.222392	0.9583633	6	1	7	8	4	0
AC009690.2	#DIV/0!	#DIV/0!	0.8030358	0	0	0	0	0	1
AC009690.1	0	#NUM!	0.5643268	1	0	2	0	0	0
NEO1	0.4642127	-1.107142	9.53E-05	153	157	179	54	74	99
CCDC183-AS1	0.2962963	-1.754888	0.1332233	4	13	10	4	3	1
AC129926.1	1.0588235	0.0824622	0.6669303	37	73	77	68	61	69
NEMF	1.1313466	0.178041	0.241082	711	371	730	547	772	731
DRAM1	0.8730603	-0.195847	0.242812	1410	1516	1714	1119	1493	1439
AC108215.1	0	#NUM!	0.6840298	0	0	2	0	0	0
DRAM2	1.025784	0.0367269	0.4907924	951	784	1135	868	1141	935
KCNJ2	0.7307692	-0.452512	0.6813669	6	6	14	4	9	6
AL031722.1	#DIV/0!	#DIV/0!	0.3096126	0	0	0	5	0	0
KCNJ4	0	#NUM!	0.4460243	4	0	0	0	0	0
KCNJ5	0	#NUM!	0.0046388	5	7	8	0	0	0
COL26A1	1.4090909	0.4947647	0.6146932	12	8	2	6	17	8
KCNJ6	#DIV/0!	#DIV/0!	0.2545643	0	0	0	0	4	2
KCNJ8	0.2857143	-1.807355	0.5385484	0	0	7	0	2	0
AC026367.1	0.8148148	-0.295456	0.921862	1	6	20	13	6	3
AC133041.1	0	#NUM!	0.2394948	0	0	8	0	0	0
AL078604.1	0	#NUM!	0.824326	0	0	1	0	0	0
AL078604.2	2	1	0.8319548	0	1	0	0	2	0
KCNK1	0.9587441	-0.060782	0.9951585	878	795	1066	797	948	881
AC021818.1	0	#NUM!	0.5781912	0	0	3	0	0	0
KCNK2	0.8	-0.321928	0.9391316	3	1	1	4	0	0
KCNK3	#DIV/0!	#DIV/0!	0.6411933	0	0	0	0	0	2
KCNK4	0	#NUM!	0.824326	0	1	0	0	0	0
AC100763.1	0	#NUM!	0.6436235	2	0	0	0	0	0
ATXN7L1	1.2300242	0.2986867	0.1101466	121	128	164	147	181	180
ATXN7L2	0.7916667	-0.337035	0.4102858	64	84	68	61	65	45

ATXN7L3	0.8894046	-0.169088	0.3893294	2181	2618	2742	2127	2265	2315
DRAIC	0	#NUM!	0.3267077	0	3	2	0	0	0
SOAT1	0.8931392	-0.163043	0.3863317	3445	2892	3414	2187	3151	3371
KCNQ2	1.3333333	0.4150375	0.8713205	0	6	0	0	3	5
KCNQ4	0.459854	-1.120752	0.024758	35	47	55	24	24	15
AC008038.1	0.9474565	-0.077868	0.9299986	2373	3232	3359	2919	3016	2558
AL136964.1	#DIV/0!	#DIV/0!	0.8030358	0	0	0	0	1	0
RRAD	0.9565217	-0.06413	0.9719272	15	4	4	7	6	9
AP000365.1	0.7777778	-0.36257	0.8642547	6	0	3	0	0	7
NEU2	1.1363636	0.1844246	0.7754564	2	11	9	10	4	11
PKHD1	1.2905405	0.3679755	0.1110821	95	81	120	97	132	153
NEU3	0.8045635	-0.313722	0.1311638	588	657	771	354	633	635
RRAS	1.0192	0.0274372	0.6130912	375	422	453	517	406	351
NEU1	0.9602761	-0.058479	0.9978469	2195	2669	2814	2397	2737	2239
KCNN1	0.7777778	-0.36257	0.873164	5	0	4	3	4	0
KCNN2	0.5	-1	0.8188512	2	0	0	0	1	0
KCNN3	1	0	0.9094265	0	0	3	3	0	0
AC074237.1	0.76	-0.395929	0.696657	16	5	4	0	8	11
AC087857.1	#DIV/0!	#DIV/0!	0.1903651	0	0	0	7	0	0
KCNN4	0.872279	-0.197138	0.4599027	1152	1187	1474	1262	1108	956
AC087222.1	0.7368421	-0.440573	0.7298749	3	9	7	6	5	3
NTNG1	0.6956522	-0.523562	0.5976765	13	4	29	5	11	16
NTNG2	2.25	1.169925	0.5867806	1	3	0	1	8	0
NET1	1.4794865	0.5650965	1.32E-07	8697	7511	8873	10176	13722	13209
OSBPL10-AS1	0.6666667	-0.584963	0.8234316	0	3	0	0	2	0
TFB1M	0.8793003	-0.185572	0.3745324	526	558	631	454	559	495
AC119677.1	0	#NUM!	0.824326	0	0	1	0	0	0
KCNT1	2	1	0.6959447	2	0	0	4	0	0
KCNT2	0	#NUM!	0.6840298	0	0	2	0	0	0
AC007688.1	3.5	1.8073549	0.214331	2	0	2	4	4	6
AGTRAP	1.0265734	0.0378368	0.5258911	736	1011	1113	1027	1054	855
TFB2M	0.8571429	-0.222392	0.2967678	566	528	775	456	620	526
AC007292.1	0.6666667	-0.584963	0.7758152	1	2	12	5	1	4
AC008132.1	0.8181818	-0.289507	0.8989759	9	1	1	7	1	1
AC007292.2	0.4545455	-1.137504	0.5823622	0	6	5	4	0	1
FAM32A	0.9858547	-0.020553	0.7864484	2328	2931	3083	2561	3054	2609
AC010834.2	0.25	-2	0.5415866	0	4	0	1	0	0
AC010834.3	0.7352941	-0.443607	0.4720014	23	23	22	10	25	15
AC092910.3	0.8352273	-0.259759	0.6379501	77	30	69	42	44	61
SSNA1	1.1757263	0.2335522	0.0443534	1553	1911	2147	2338	2348	1911
FAM35A	1.3506608	0.4336654	0.0107985	387	268	480	382	619	532
NEXN	1.1777778	0.2360674	0.5346805	36	22	32	39	45	22
AC008278.1	0	#NUM!	0.4005425	3	1	0	0	0	0
AC025539.1	0	#NUM!	0.824326	0	1	0	0	0	0
KRT87P	0.4074074	-1.295456	0.1646343	22	6	26	0	11	11
KLHL2	0.9396985	-0.08973	0.8577059	318	185	293	195	280	273
KLHL3	0.6563877	-0.60738	0.1202204	73	58	96	52	49	48
KCNRG	0	#NUM!	0.2773534	1	3	1	0	0	0
KLHL4	#DIV/0!	#DIV/0!	0.6554062	0	0	0	0	2	0
AC018766.1	0.7333333	-0.447459	0.7764421	6	3	6	6	4	1
RNU7-115P	0	#NUM!	0.824326	0	1	0	0	0	0
AC093155.1	0.5	-1	0.7932724	0	0	4	0	0	2
KLHL9	0.8997387	-0.152422	0.5325249	1199	806	1057	681	973	1101
TFAP4	0.8887946	-0.170078	0.4988366	643	743	970	666	747	681

KCNS1	0.625	-0.678072	0.6775901	2	14	0	8	1	1
KLHL5	1.0063898	0.0091892	0.7315714	646	582	650	497	677	716
KCNS2	0	#NUM!	0.1675055	0	3	5	0	0	0
KLHL6	0.8888889	-0.169925	0.8787695	3	6	0	0	4	4
KCNS3	0.9385516	-0.091492	0.8363881	395	452	520	392	461	430
KLHL7	1.0561639	0.0788337	0.3001695	942	848	1041	888	1106	996
KLHL8	0.9889426	-0.016041	0.8745437	517	432	498	368	516	547
ANP32AP1	0.6666667	-0.584963	0.8018249	1	5	0	3	0	1
CARD10	0.7809417	-0.356713	0.0426057	1307	1578	1575	1190	1217	1076
BRMS1	0.858475	-0.220152	0.2988605	1473	1830	2166	1580	1720	1395
CARD19	0.9967721	-0.004664	0.6982209	398	527	624	559	518	467
CARD17	0.3333333	-1.584963	0.2860378	12	0	15	0	1	8
CARD18	0.775	-0.367732	0.6375814	7	24	9	3	21	7
CARD16	0.8658147	-0.20787	0.629441	95	101	117	68	70	133
CARD14	0.69	-0.535332	0.1975788	43	78	79	31	58	49
CARD11	0.5279107	-0.921634	0.0015398	151	267	209	70	137	124
AL355773.1	#DIV/0!	#DIV/0!	0.8030358	0	0	0	0	1	0
AC109439.2	1.3333333	0.4150375	0.8928296	3	0	0	0	0	4
KCNV1	1.3650794	0.4489848	0.3753218	27	20	16	20	28	38
KCNV2	2.5555556	1.353637	0.1959143	2	1	6	10	3	10
AC016712.1	2.6666667	1.4150375	0.4066134	0	1	2	5	2	1
AL121772.3	1.1666667	0.2223924	0.765119	12	8	10	6	2	27
ELDR	0.4375	-1.192645	0.4037768	8	2	6	0	6	1
AL121772.1	5.5	2.4594316	0.2298313	2	0	0	6	5	0
AC132942.1	0.2307692	-2.115477	0.2089451	8	2	3	0	1	2
U62317.3	0.6721311	-0.573185	0.2641973	37	31	54	26	27	29
U62317.4	0.125	-3	0.1978075	3	3	2	1	0	0
RN7SL689P	2.6	1.3785116	0.3597925	0	2	3	6	0	7
SPATA31A3	2	1	0.6959447	2	0	0	4	0	0
AC027290.2	0.845679	-0.241818	0.6941545	51	38	73	22	34	81
ZMYND15	5	2.3219281	0.4094329	1	0	0	5	0	0
AL356361.2	0	#NUM!	0.6436235	2	0	0	0	0	0
ZMYND19	0.9938111	-0.008956	0.7095355	842	1072	1156	1061	1049	941
RN7SL688P	0.3333333	-1.584963	0.7290749	0	0	3	0	0	1
SPATA31C2	0.8888889	-0.169925	0.9460586	3	1	5	1	3	4
AL392172.1	0.9423077	-0.08573	0.9258531	57	71	80	61	89	46
MYBL2	0.8059346	-0.311265	0.0485021	9744	11981	12279	9058	9506	8841
SPATA31C1	0	#NUM!	0.824326	1	0	0	0	0	0
MYBL1	0.5930521	-0.753769	0.0199382	174	89	140	64	88	87
RN7SL687P	#DIV/0!	#DIV/0!	0.6411933	0	0	0	0	0	2
AC099669.1	0	#NUM!	0.824326	1	0	0	0	0	0
ELF1	1.0656886	0.091786	0.4556321	1613	1348	1606	1060	1915	1892
ELF2	1.1110039	0.1518638	0.1862084	1099	823	1186	885	1365	1203
ELF3	0.9580594	-0.061813	0.9907075	2995	3328	3715	3066	3310	3241
ELF4	0.783282	-0.352396	0.015323	3247	3795	4000	2422	3030	3197
ELF5	2.0632911	1.0449474	0.0334505	19	39	21	25	65	73
ZMYND10	2.5	1.3219281	0.1653673	5	1	6	9	5	16
ZMYND11	1.278449	0.3543946	0.0051544	1302	947	1310	1238	1663	1649
AC021078.1	0.8839442	-0.177973	0.5456136	949	505	838	393	752	881
SDCBPP3	#DIV/0!	#DIV/0!	0.362352	0	0	0	1	2	1
ZMYND12	1.25	0.3219281	0.8527774	0	0	4	0	3	2
RRN3	0.9976959	-0.003328	0.7850208	1407	1135	1364	1021	1517	1359
AL139220.2	5.2	2.3785116	0.0958986	0	2	3	0	12	14
RRM1	0.9028895	-0.147379	0.4075342	4958	4320	5396	3499	4975	4775

RRM2	1.0517253	0.072758	0.2171136	4477	4393	5359	4785	5245	4935
AC007938.3	0.25	-2	0.1387197	6	6	8	0	3	2
HS3ST5	#DIV/0!	#DIV/0!	0.3246733	0	0	0	0	3	2
HS3ST1	0	#NUM!	0.824326	1	0	0	0	0	0
AL117187.1	#DIV/0!	#DIV/0!	0.8030358	0	0	0	0	0	1
ZNF259P1	0.3333333	-1.584963	0.6321417	1	1	1	1	0	0
FRMPD3	0.9210526	-0.118644	0.9472041	13	10	15	12	11	12
FRMPD1	2.0441176	1.0314782	0.0003249	78	42	84	122	123	172
FRMPD2	1.0408511	0.0577636	0.5346039	385	393	397	345	438	440
RRP1	1.0057803	0.0083153	0.5987508	1276	1601	1794	1604	1610	1484
ELK1	0.8361063	-0.258242	0.1153419	1171	1381	1475	970	1256	1141
RRP8	0.7752855	-0.3672	0.0401919	712	877	863	607	656	638
ELK3	0.9363868	-0.094824	0.821495	379	326	474	281	412	411
RRP9	0.778567	-0.361107	0.0316664	1406	1619	1762	1266	1248	1213
ELK4	0.9740447	-0.03794	0.9394282	501	381	505	364	476	511
AC106882.1	#DIV/0!	#DIV/0!	0.6099294	0	0	0	2	0	0
AC026368.1	0.5	-1	0.8188512	2	0	0	0	1	0
DLX6-AS1	0.9039735	-0.145648	0.6356542	446	309	453	295	365	432
TARS2	0.9074074	-0.140178	0.5494939	2152	2461	2893	2199	2302	2310
PLEKHA8P1	1.1193694	0.1626862	0.4224389	193	76	175	177	170	150
AC004066.1	#DIV/0!	#DIV/0!	0.3831122	0	0	0	0	5	0
AC004066.2	#DIV/0!	#DIV/0!	0.6554062	0	0	0	0	2	0
RRS1	0.7605746	-0.394838	0.01615	1130	1186	1443	939	925	995
ST3GAL4	0.9451056	-0.081453	0.9324056	748	774	1083	788	958	716
ST3GAL5	1.5810147	0.6608508	0.0001363	199	203	209	285	356	325
ST3GAL6	1.4042715	0.4898219	0.0004966	432	391	488	606	685	550
AC099524.1	0.4444444	-1.169925	0.6403962	1	0	8	0	0	4
ST3GAL1	1.127116	0.172636	0.1246541	929	1155	1165	1001	1324	1337
ST3GAL2	0.7312883	-0.451488	0.0114267	676	908	861	500	662	626
ST3GAL3	0.5677966	-0.816554	0.0949112	36	41	41	18	24	25
ARL14EP	1.3508772	0.4338965	0.1524355	62	43	66	54	91	86
NFE2	1.0654206	0.091423	0.736678	33	43	31	53	37	24
ELL2	1.123592	0.1681182	0.1318392	1116	921	1159	934	1314	1343
ELL3	2.6666667	1.4150375	0.5218472	0	0	3	0	0	8
AL137802.2	0.7333333	-0.447459	0.7295129	9	3	3	4	5	2
RSPH10B	1	0	0.9876564	2	1	4	0	5	2
AC012501.1	0.75	-0.415037	0.7883885	7	1	8	4	4	4
AC012501.2	1.2232143	0.2906772	0.4460204	24	54	34	44	48	45
ELOA	1.0410348	0.0580183	0.3661507	2056	2279	2391	2035	2508	2459
ELOB	0.9842829	-0.022855	0.7507793	2081	2617	2937	2814	2605	2096
ELOC	0.9973988	-0.003758	0.6329678	1057	1066	1337	1199	1162	1090
AC011912.1	2.6875	1.4262648	0.068874	3	4	9	15	10	18
RNU6-795P	#DIV/0!	#DIV/0!	0.8030358	0	0	0	0	1	0
NFIX	0.7	-0.514573	0.7793576	0	10	0	4	1	2
AL022314.1	0	#NUM!	0.824326	1	0	0	0	0	0
METTL21A	1.0942065	0.1298851	0.221436	617	687	681	642	760	770
METTL21C	7	2.8073549	0.2474585	1	0	0	6	0	1
AL035588.1	0.6666667	-0.584963	0.9030659	0	0	3	0	0	2
RNU6-797P	0	#NUM!	0.824326	1	0	0	0	0	0
KPNA2P2	0	#NUM!	0.6840298	0	0	2	0	0	0
AL390755.1	#DIV/0!	#DIV/0!	0.8030358	0	0	0	0	0	1
AL356121.2	#DIV/0!	#DIV/0!	0.6099294	0	0	0	2	0	0
ELP1	1.0409455	0.0578945	0.3841493	2626	2235	2881	2166	2901	2992
RFLNB	1.6426819	0.7160532	3.73E-07	395	401	427	570	732	707

ELP2	0.9519213	-0.071086	0.9050651	1730	1601	1952	1542	1837	1650
ELP3	0.8042226	-0.314333	0.0477338	1113	1184	1350	824	1019	1090
ELP4	1.0377804	0.0535012	0.4825563	547	543	604	546	677	535
ELP5	0.9727228	-0.039899	0.9375838	554	788	711	649	717	631
ELP6	0.8191537	-0.287794	0.156035	619	747	879	614	623	602
RFLNA	1	0	0.9682051	5	19	5	12	7	10
RPL4P2	#DIV/0!	#DIV/0!	0.3196487	0	0	0	0	1	4
AC012157.1	0	#NUM!	0.2646708	3	0	3	0	0	0
RPL4P3	0	#NUM!	0.6385096	0	2	0	0	0	0
RPL4P4	0.9417249	-0.086622	0.9278072	247	314	297	311	266	231
RPL4P5	1.0714286	0.0995357	0.7465128	20	28	22	35	19	21
NFIA	1.5	0.5849625	0.1818986	14	38	32	35	40	51
RPL4P6	2.0967742	1.0681715	0.2420702	13	18	0	9	24	32
NFIB	0.9651163	-0.051225	0.9638694	23	31	32	31	22	30
NFIC	0.6402396	-0.643316	0.0002522	864	935	1039	649	573	595
RPL4P1	1	0	0.997461	0	2	0	1	1	0
FAM49A	1.6165691	0.6929352	2.99E-09	1087	963	1197	1456	1914	1879
FAM49B	1.1017005	0.139732	0.1017035	3196	2561	3417	3100	3697	3310
LCLAT1	1.1451173	0.1954954	0.1969429	617	539	677	453	833	813
IL13RA2	1	0	0.9644635	3	0	0	3	0	0
IL13RA1	0.7031802	-0.508034	0.0032846	906	782	859	446	707	638
AC096536.3	#DIV/0!	#DIV/0!	0.4501902	0	0	0	0	0	4
KCMF1	0.9070418	-0.140759	0.4772631	2039	2220	2529	1898	2258	2001
AC096536.1	0.1666667	-2.584963	0.3780413	0	6	0	0	1	0
COL27A1	1.1929055	0.2544798	0.0119845	1552	1409	2057	1726	2089	2171
MIRLET7G	0	#NUM!	0.0128466	3	9	4	0	0	0
AC090515.2	0.7037037	-0.50696	0.4876555	9	26	19	5	19	14
MIRLET7D	1.8153846	0.8602752	0.0584083	24	11	30	32	45	41
AC090515.4	0	#NUM!	0.3958203	2	2	0	0	0	0
TOM1L1	0.6071429	-0.719892	0.4848689	11	4	13	3	7	7
TOM1L2	1.0718563	0.1001115	0.3179343	454	404	478	450	519	463
OBSCN	1.1160018	0.1583394	0.2740104	638	790	753	561	916	957
AL033381.1	#DIV/0!	#DIV/0!	0.6411933	0	0	0	0	0	2
AC026462.1	0.6666667	-0.584963	0.6249357	7	4	13	6	6	4
AC073648.6	#DIV/0!	#DIV/0!	0.6411933	0	0	0	0	0	2
AP003498.1	1	0	0.9922023	2	0	0	0	0	2
AP001099.1	#DIV/0!	#DIV/0!	0.3768753	0	0	0	2	0	2
PGPEP1L	0.71875	-0.476438	0.7177568	7	13	12	15	8	0
SLC48A1	0.6564496	-0.607244	0.0026586	434	574	558	354	342	332
AC069133.1	0	#NUM!	0.824326	0	0	1	0	0	0
AL139128.1	0.3333333	-1.584963	0.669913	3	0	0	0	0	1
EVI5L	0.9149371	-0.128256	0.6134135	822	1041	1076	832	988	869
AL359399.1	1.2380952	0.3081223	0.7282907	6	13	2	12	8	6
OR52K3P	0.4166667	-1.263034	0.4404693	7	4	1	3	2	0
HDAC4	1.1162011	0.158597	0.3287776	245	337	313	250	349	400
AL161670.1	1.3	0.3785116	0.6898824	12	4	4	5	13	8
CEBPA	1.0491876	0.0692727	0.3755324	1904	2456	2410	2273	2624	2206
HDAC5	1.1115446	0.1525659	0.0783485	2558	2853	3303	3218	3357	3111
AL161670.2	0	#NUM!	0.6840298	0	0	2	0	0	0
CEBPB	1.3330565	0.4147379	0.0005285	3327	4002	4711	5681	5474	4895
HDAC2	1.1357237	0.1836119	0.0646963	4805	3567	5266	4616	5970	4903
HDAC3	0.8619247	-0.214366	0.2942096	1400	1596	1784	1431	1394	1295
CEBPD	1.4023858	0.4878833	0.0004499	956	1196	1285	1745	1669	1406
CEBPE	#DIV/0!	#DIV/0!	0.6411933	0	0	0	0	0	2

HDAC1	0.9703708	-0.043392	0.8913505	5617	6264	7188	5694	6708	6102
CEBPG	1.0808442	0.1121585	0.1527278	2060	1869	2231	2057	2444	2157
HDAC8	1.0315457	0.0448078	0.5499301	421	410	437	389	454	465
HDAC9	0.6111111	-0.710493	0.4062034	14	12	10	6	6	10
HDAC6	0.9198736	-0.120492	0.5815441	1624	1760	1995	1479	1794	1675
HDAC7	0.891358	-0.165923	0.582139	337	392	486	360	360	363
NFS1	0.8978755	-0.155413	0.5314364	794	919	970	815	885	709
HECTD1	1.0212386	0.03032	0.6905346	3978	2275	3823	2329	4024	3937
HECTD2	2.1092437	1.0767258	0.0074152	64	30	25	70	79	102
HECTD3	1.1233168	0.1677649	0.082914	1556	1952	2136	1996	2217	2127
HECTD4	0.6571827	-0.605634	0.0028232	1195	1174	1216	479	843	1034
XIRP1	0.9375	-0.093109	0.9888718	10	11	11	14	8	8
XIRP2	1.4545455	0.5405684	0.3486075	18	11	26	21	49	10
AL354694.1	1.75	0.8073549	0.6260417	1	0	3	4	3	0
MGME1	1.0420616	0.0594406	0.3718245	1142	951	1283	1014	1287	1217
AC040977.1	0.9811321	-0.027481	0.8731509	18	7	28	28	15	9
EVI2A	1	0	0.9293041	6	0	8	6	7	1
AP003352.1	0.4571429	-1.129283	0.240413	7	9	19	4	6	6
CEBPZ	1.1719822	0.2289507	0.1714792	1009	505	963	721	1130	1052
EVI2B	0.5	-1	0.7988788	1	1	0	0	0	1
AC104389.2	0	#NUM!	0.6840298	0	0	2	0	0	0
AC104389.3	#DIV/0!	#DIV/0!	0.6411933	0	0	0	0	0	2
C6orf89	0.8586681	-0.219827	0.1873675	1691	1824	2011	1329	1631	1785
NFU1	1.1985078	0.2612393	0.0085908	1455	1250	1718	1652	1846	1803
NIPAL4	0.6	-0.736966	0.8149364	0	0	5	0	2	1
NIPAL1	0.7789116	-0.360469	0.2702178	95	97	102	61	93	75
NIPAL3	1.015311	0.0219217	0.7422281	405	280	360	271	405	385
AC004890.3	0.5	-1	0.6808192	1	0	11	4	0	2
FAM41C	0.5714286	-0.807355	0.6651138	6	1	0	0	3	1
AC004890.2	0.8933333	-0.16273	0.816473	10	37	28	13	40	14
AL121929.2	2.2	1.1375035	0.5710143	2	2	1	0	11	0
AC035140.1	0	#NUM!	0.6840298	0	0	2	0	0	0
AC002984.1	1.5	0.5849625	0.7734914	0	0	2	3	0	0
TWSG1	0.7161085	-0.48175	0.0421488	209	193	225	135	162	152
OBSL1	0.626703	-0.674146	0.0591137	105	98	164	90	86	54
FXYD6P2	#DIV/0!	#DIV/0!	0.6411933	0	0	0	0	0	2
SPRR2G	0	#NUM!	0.6840298	0	0	2	0	0	0
CRHR2	0	#NUM!	0.4324683	2	0	2	0	0	0
FAM43A	3.05	1.6088092	0.031271	9	1	10	15	27	19
BTBD10P2	5	2.3219281	0.4840007	1	0	0	0	5	0
SPRR2A	0.25	-2	0.5622508	0	1	3	1	0	0
SPRR2D	0.53125	-0.912537	0.3737942	6	12	14	5	11	1
AC026951.1	#DIV/0!	#DIV/0!	0.5371397	0	0	0	0	0	3
C6orf99	0.5458015	-0.873552	0.0162505	65	82	115	37	59	47
FAM45A	1.1307902	0.1773313	0.160079	512	359	597	495	633	532
RSF1	1.0962718	0.1326056	0.3451035	803	441	875	573	855	895
C6orf47	0.6888702	-0.537696	0.0086941	362	369	455	282	268	267
C6orf48	1.0206534	0.029493	0.4913457	2711	3447	3913	3464	3699	3116
VWFP1	0.875	-0.192645	0.9257609	4	0	4	0	4	3
MTSS1	2.2857143	1.1926451	0.1004535	5	7	9	11	21	16
NFX1	1.0612602	0.0857784	0.247298	1700	1448	1994	1541	2031	1885
ANP32BP1	0	#NUM!	0.3451105	2	0	3	0	0	0
CALML3-AS1	2.1956522	1.1346495	0.0038196	33	29	30	42	68	92
FAM47C	0.25	-2	0.5318227	3	0	1	1	0	0

FAM47A	0	#NUM!	0.824326	0	1	0	0	0	0
C6orf62	0.9695056	-0.044679	0.9671337	4731	3919	5287	3490	5015	5007
EMC1	0.8484468	-0.237104	0.1104728	3452	3762	3924	2609	3447	3394
EMC2	1.0957049	0.1318594	0.2765858	774	536	832	707	976	664
EMC3	0.9245604	-0.113161	0.6726524	1057	1144	1325	1017	1165	1078
EMC4	0.9350649	-0.096862	0.7840476	1485	1695	1825	1552	1712	1416
FAM47E	1.15	0.2016339	0.8110318	11	5	4	6	6	11
EMC6	0.6111111	-0.710493	0.5001452	6	6	24	7	8	7
EMC7	0.9468023	-0.078865	0.9451721	1453	1605	2055	1596	1706	1539
EMC8	0.9323463	-0.101062	0.771405	1354	1621	1888	1435	1722	1377
EMC9	0.881	-0.182786	0.6082218	238	318	444	287	300	294
FAM46B	0.8513389	-0.232195	0.35087	318	367	398	314	306	302
NFYA	1.0430183	0.0607645	0.513543	945	912	979	726	1124	1108
FAM46C	1.2923077	0.3699496	0.2725131	57	31	42	57	56	55
NFYB	0.9344473	-0.097815	0.7632466	1168	813	1131	829	1102	977
NFYC	0.9540282	-0.067896	0.9584482	1870	2190	2531	1964	2234	2090
FAM46A	1.3451361	0.4277522	0.0021484	1920	1443	1890	1710	2658	2698
C6orf58	3	1.5849625	0.4150935	0	1	2	0	4	5
RSG1	0.7667845	-0.383107	0.3197829	67	110	106	74	64	79
C6orf52	0.9177215	-0.123872	0.839353	49	52	57	41	53	51
LRRN4CL	2.3333333	1.2223924	0.4959253	1	0	2	6	1	0
C8orf37-AS1	1	0	0.9890559	0	0	1	1	0	0
FAM46D	0.5714286	-0.807355	0.7939197	3	0	4	4	0	0
ROGDI	0.8041911	-0.31439	0.083199	2114	2516	2862	2099	2130	1796
PAIP2B	1.2218211	0.2890331	0.0116351	823	771	899	837	1153	1056
EME1	0.8790669	-0.185955	0.4196754	521	467	641	422	572	438
EME2	0.799458	-0.322906	0.4249289	115	107	147	121	110	64
MYADM	1.1869146	0.2472161	0.0216217	2025	2594	2641	2543	3130	2944
IGHVII-46-1	1	0	0.9395007	1	2	2	4	1	0
EMG1	1.021983	0.0313711	0.4898841	1242	1498	1718	1599	1558	1399
EMG1	1.021983	0.0313711	0.4898841	1242	1498	1718	1599	1558	1399
TASP1	1.0025094	0.0036158	0.7738569	234	282	281	243	299	257
AC004160.1	0.9230769	-0.115477	0.9360995	12	5	9	6	9	9
DIAPH1	0.8851793	-0.175958	0.2835412	10699	12522	13201	8770	11738	11732
DIAPH2	0.9764244	-0.03442	0.9398107	370	244	404	229	369	396
DIAPH3	0.800353	-0.321292	0.1235179	1599	1084	1850	839	1422	1367
PXMP2	1.057971	0.0813001	0.7989403	35	15	19	29	14	30
PXMP4	1.4444444	0.5305147	0.6693518	4	0	5	2	6	5
AL136234.1	0.7142857	-0.485427	0.8206448	2	1	4	0	1	4
COLGALT2	1.5714286	0.6520767	0.6480125	4	0	3	2	6	3
AC098936.1	0	#NUM!	0.6385096	0	2	0	0	0	0
NTMT1	0.9923274	-0.011112	0.743842	581	852	913	795	811	722
COLGALT1	0.9077368	-0.139654	0.4929011	6115	7145	7485	5976	6431	6424
MIR941-5	2	1	0.834705	0	0	1	0	2	0
OXSR1	0.981637	-0.026739	0.8971898	4328	3831	4584	3147	4563	4799
AC079768.1	0	#NUM!	0.824326	0	0	1	0	0	0
AL157791.1	0.5	-1	0.81753	2	0	0	0	0	1
AL157791.2	9	3.169925	0.165286	1	0	0	1	1	7
AC108704.1	0	#NUM!	0.4460243	4	0	0	0	0	0
RN7SL698P	0	#NUM!	0.6840298	0	0	2	0	0	0
AC108704.2	0.3333333	-1.584963	0.270342	6	4	5	1	1	3
NDUFV2-AS1	0.6	-0.736966	0.3784167	12	11	17	6	12	6
PDE11A	1.1333333	0.1805722	0.8356048	16	12	2	10	11	13
PDE11A	1.1333333	0.1805722	0.8356048	16	12	2	10	11	13

ATP13A1	0.95	-0.074001	0.8823562	1645	1879	2136	1600	1976	1801
PDE10A	0.4142012	-1.271596	0.007053	66	36	67	21	19	30
AC004556.1	1.3246136	0.4055715	0.0215026	204	300	337	382	411	321
ATP13A4	2.8	1.4854268	0.3801807	0	0	5	0	7	7
RN7SL697P	3	1.5849625	0.6732949	0	0	1	0	0	3
ATP13A5	1	0	0.9890559	1	0	0	0	1	0
ATP13A2	0.9410893	-0.087596	0.9148624	721	877	1101	822	831	887
ATP13A3	0.8767534	-0.189757	0.3941516	6937	4448	6295	3337	5932	6232
AL022315.1	1	0	0.963496	0	3	0	0	3	0
AL035587.1	0.6060606	-0.722466	0.1434181	32	60	40	24	36	20
IGLON5	0.25	-2	0.1593698	5	7	8	3	0	2
MIRLET7BHC	1	0	0.9388973	14	5	8	10	6	11
FIBCD1	1.5853659	0.6648158	0.0591004	38	77	49	101	98	61
NGDN	0.9803972	-0.028562	0.856967	1246	1248	1383	1114	1404	1283
RPL5P9	1	0	0.9440113	0	2	3	3	2	0
AC099667.1	0.125	-3	0.0899662	12	2	2	1	0	1
EML1	1.5396825	0.6226329	0.2826947	19	34	10	21	41	35
EML2	0.7945471	-0.331795	0.0529095	1661	1851	2173	1537	1628	1352
EML3	0.9370416	-0.093815	0.7816019	479	582	575	462	592	479
EML4	0.8504692	-0.233669	0.2178537	5432	3888	5493	3029	4694	4875
RPL5P1	0.2380952	-2.070389	0.1313655	3	6	12	2	2	1
EML5	16	4	0.0422877	1	0	0	1	3	12
EML6	1.1369427	0.1851595	0.2039301	195	177	256	213	249	252
RPL5P3	#DIV/0!	#DIV/0!	0.8030358	0	0	0	0	1	0
RPL5P4	1.0714286	0.0995357	0.8298772	10	18	14	14	19	12
RPL5P6	0	#NUM!	0.824326	1	0	0	0	0	0
GDF5OS	0.5	-1	0.8652929	0	0	2	0	1	0
RPL5P7	#DIV/0!	#DIV/0!	0.249172	0	0	0	0	1	5
RBCK1	1.0274778	0.0391072	0.4528348	1839	2041	2416	2362	2240	1867
NGEF	1.4545455	0.5405684	0.0351853	63	91	88	117	109	126
AAAS	0.8652083	-0.208881	0.2491966	1820	2120	2277	1755	1873	1751
RSU1	1.183542	0.2431109	0.0105651	1654	1726	2052	1853	2431	2145
NGFR	0.4	-1.321928	0.2790562	6	14	5	4	0	6
TAS2R15P	0.6111111	-0.710493	0.6479616	1	5	12	3	1	7
PARGP1	1.2601626	0.3336099	0.2510231	107	55	84	74	135	101
CU633967.1	0.9642857	-0.052467	0.9612194	11	3	14	9	9	9
AACS	0.9333787	-0.099466	0.7525861	396	533	542	357	574	442
AC091976.1	0.5454545	-0.874469	0.5860049	2	5	4	3	2	1
AC099521.2	0.625	-0.678072	0.6814718	2	4	2	0	3	2
AC099521.3	1.4	0.4854268	0.7148961	1	0	4	7	0	0
UGT1A12P	1	0	0.9890559	0	1	0	0	1	0
EMP1	0.1363636	-2.874469	0.0581509	2	8	12	0	2	1
EMP2	1.0571081	0.080123	0.3287708	930	1161	1201	1087	1176	1217
EMP3	0.8947368	-0.160465	0.9431866	5	18	34	17	16	18
TARDBPP2	2.5	1.3219281	0.3322254	2	1	3	1	6	8
FAHD2A	1.133596	0.1809066	0.0931148	828	886	1078	1112	1114	939
FAHD2B	3.71875	1.8948178	0.0012893	2	16	14	46	39	34
RNU4ATAC18	0.1428571	-2.807355	0.123395	6	3	5	0	2	0
DPY19L1P1	1.0101523	0.0145728	0.8517596	76	53	68	52	56	91
DPY19L1P2	2.2727273	1.1844246	0.4421143	1	10	0	0	12	13
MYCT1	1.1881188	0.2486791	0.3377444	55	77	70	80	86	74
AADAT	0.8948834	-0.160228	0.4330314	930	850	1093	755	911	905
AP001206.1	0	#NUM!	0.824326	1	0	0	0	0	0
RIMBP3	#DIV/0!	#DIV/0!	0.5371397	0	0	0	0	0	3

HDDC2	0.9174047	-0.12437	0.724158	1501	1422	1932	1558	1640	1256
FAM13B	0.9320875	-0.101463	0.7696369	862	528	804	511	755	779
UBE2D4	1.1428571	0.1926451	0.4843977	64	94	59	84	72	92
FAM13C	0.6666667	-0.584963	0.8308055	3	0	0	0	2	0
UBE2D2	1.0841768	0.1166	0.1353384	5234	6029	6521	6129	6529	6623
FAM13A	1.3024645	0.3812441	0.0106518	466	337	536	462	661	621
UBE2D3	1.1887818	0.2494839	0.0035441	8928	8577	10289	9500	12430	11111
AC004069.1	1.3214286	0.4020984	0.2341547	29	31	52	48	57	43
AC004069.2	#DIV/0!	#DIV/0!	0.5509701	0	0	0	0	3	0
UBE2D1	1.2154762	0.2815216	0.0256373	568	443	669	588	760	694
HDDC3	0.9745917	-0.03713	0.8994616	146	189	216	170	174	193
IL20RB-AS1	0	#NUM!	0.6840298	0	0	2	0	0	0
AL035681.1	1.25	0.3219281	0.7977202	3	4	5	0	6	9
UBE2E3	0.885563	-0.175333	0.3493753	1442	1661	1808	1355	1557	1437
UBE2E1	0.9777153	-0.032514	0.8809639	4981	4999	5681	4297	5767	5248
UBE2E2	0.9836216	-0.023825	0.7729525	1609	1772	2053	1616	1910	1819
ATP5J2P4	#DIV/0!	#DIV/0!	0.8030358	0	0	0	1	0	0
EMSY	0.8731003	-0.195781	0.3261691	920	780	932	574	839	885
AC012254.1	0	#NUM!	0.4217812	0	0	5	0	0	0
AC012254.3	0	#NUM!	0.2195149	0	2	5	0	0	0
FBLIM1	1.4666667	0.552541	0.1858074	20	18	37	33	45	32
NTPCR	0	#NUM!	0.824326	0	0	1	0	0	0
AC012254.4	0.4285714	-1.222392	0.6319825	1	0	6	0	3	0
ATP5J2LP	0	#NUM!	0.824326	1	0	0	0	0	0
AC012254.5	0.3333333	-1.584963	0.4977133	1	5	0	0	2	0
SOCS2	0.9157114	-0.127035	0.724437	402	465	616	422	467	469
SOCS3	1.0277296	0.0394608	0.666148	159	206	212	173	220	200
SOCS1	1.5222222	0.606179	0.0021014	166	143	231	304	288	230
SOCS6	1.0321508	0.0456537	0.5870671	332	211	359	272	335	324
SOCS7	0.8515284	-0.231873	0.2410303	793	956	999	571	853	916
SOCS4	0.8760575	-0.190902	0.4915275	989	503	872	488	783	800
SOCS5	1.0762977	0.1060772	0.4286686	588	415	596	397	618	706
COL28A1	0.5789474	-0.788496	0.3934194	12	6	20	6	8	8
BRPF1	0.7154564	-0.483064	0.0008362	1330	1467	1596	960	1081	1102
BRPF3	0.7271906	-0.459595	0.0027828	1684	1798	2053	1013	1470	1542
BCDIN3D	1.0140845	0.0201779	0.8304676	39	48	55	45	54	45
DCUN1D2-AS	11	3.4594316	0.1348306	0	0	1	0	3	8
AC117470.1	0.7666667	-0.383329	0.6906593	11	8	11	0	17	6
TFCP2	0.996625	-0.004877	0.7270362	1971	1789	2166	1579	2109	2218
AAK1	0.9887521	-0.016319	0.8996773	5783	5323	5875	3836	6007	6947
MYD88	2.2	1.1375035	0.1461614	9	7	4	25	11	8
ST6GALNAC1	0.3953488	-1.338802	0.1642588	10	14	19	0	6	11
ST6GALNAC2	0.6235409	-0.681444	0.0014025	290	320	418	157	249	235
ATRAID	1.0417625	0.0590264	0.3930539	1708	1942	2001	2018	2094	1775
NGRN	0.8291564	-0.270284	0.1441598	810	1049	974	687	903	759
EID2B	0.9489796	-0.075551	0.9651148	31	41	26	36	23	34
PTCRA	0.375	-1.415037	0.4771489	2	3	3	1	0	2
EMX1	0.6393443	-0.645335	0.2291013	25	45	52	20	41	17
EMX2	#DIV/0!	#DIV/0!	0.0724133	0	0	0	0	4	7
AC002350.2	0.4444444	-1.169925	0.5560882	0	0	18	0	5	3
AC032011.1	0.6666667	-0.584963	0.7526643	6	0	3	1	1	4
ST6GALNAC3	1.5294118	0.6129769	0.4883192	8	3	6	13	11	2
ST6GALNAC4	1.0742049	0.1032693	0.5916832	77	102	104	127	115	62
ST6GALNAC5	1.7142857	0.7776076	0.6290604	1	4	2	0	7	5

ST6GALNAC6	1.0539291	0.0757778	0.492582	177	220	252	216	228	240
SLC47A2	0.48	-1.058894	0.1818128	13	14	23	5	7	12
CECR2	1.0533333	0.0749621	0.4695826	490	586	649	450	652	715
CECR7	0.7272727	-0.459432	0.7402091	5	3	3	0	6	2
AC022154.1	#DIV/0!	#DIV/0!	0.0323226	0	0	0	3	3	6
AAMP	1.0274053	0.0390055	0.4248323	3498	4036	4325	4055	4259	3870
RNU4ATAC12	#DIV/0!	#DIV/0!	0.8030358	0	0	0	0	1	0
RTCA	0.8729228	-0.196074	0.2851854	1030	900	1139	803	998	878
RTCB	0.8479912	-0.237879	0.1384052	4271	5062	5153	3909	4360	4015
UBE2L6	2.2301804	1.1571604	3.40E-20	587	518	724	1246	1481	1352
UBE2L4	1.1666667	0.2223924	0.8664532	1	0	5	0	3	4
UBE2L5	#DIV/0!	#DIV/0!	0.8030358	0	0	0	1	0	0
UBE2L3	1.166375	0.2220317	0.0106438	6108	6216	7102	7241	8130	7287
BX323046.1	0.125	-3	0.2879131	1	0	7	0	1	0
AC007686.1	1	0	0.9013393	0	0	7	3	1	3
ENTPD1	1.361413	0.4451048	0.079894	140	74	154	217	162	122
AC007686.2	#DIV/0!	#DIV/0!	0.8030358	0	0	0	0	0	1
ENTPD2	1.0877193	0.1213063	0.4534499	116	140	143	143	153	138
AC007686.3	0.5970149	-0.744161	0.3690469	12	14	41	3	13	24
ENTPD3	#DIV/0!	#DIV/0!	0.8030358	0	0	0	1	0	0
ENTPD4	0.7380797	-0.438152	0.0014685	2478	2366	2811	1520	2034	2096
ENTPD5	0.7756449	-0.366532	0.0561959	559	499	609	321	469	503
ENTPD6	0.8258946	-0.27597	0.1193434	1448	1785	1965	1406	1505	1382
ENTPD7	1.1433333	0.1932461	0.2731689	329	278	293	242	354	433
ENTPD8	0.6666667	-0.584963	0.7199039	0	6	6	0	6	2
ASRGL1	0.7967914	-0.327726	0.4808391	49	68	70	57	51	41
RNU7-137P	#DIV/0!	#DIV/0!	0.3597364	0	0	0	0	0	5
AC011270.2	0	#NUM!	0.4217812	0	0	5	0	0	0
AC068448.1	#DIV/0!	#DIV/0!	0.8030358	0	0	0	1	0	0
AC135977.1	1.25	0.3219281	0.760038	0	5	7	3	7	5
RTF1	0.9267295	-0.10978	0.6578371	2608	2306	3152	2155	2854	2466
AARD	0.2083333	-2.263034	0.0859753	7	8	9	2	0	3
UBE2J2	1.0080957	0.0116326	0.5708855	1474	1831	2130	1800	1938	1741
UBE2J1	1	0	0.977656	8	4	1	6	3	4
AAR2	0.7831667	-0.352609	0.0231312	1769	2033	2198	1507	1573	1619
AP000221.1	1.7692308	0.8231222	0.5022197	3	4	6	0	2	21
AC022405.1	2	1	0.8319548	0	1	0	0	2	0
WAPL	1.4353503	0.5214029	0.0001833	1119	882	1139	1116	1683	1708
KLHL30-AS1	#DIV/0!	#DIV/0!	0.3927105	0	0	0	1	0	3
LINGO1-AS2	0	#NUM!	0.824326	1	0	0	0	0	0
AARS	0.9444353	-0.082476	0.8240175	10580	12531	13441	10655	12149	11717
AATK	1.2	0.2630344	0.7444629	2	8	5	10	4	4
WASL	1.0605203	0.0847722	0.3825579	1973	1639	2039	1458	2245	2290
AATF	0.8135056	-0.297776	0.0652029	3319	4052	4313	3109	3344	3052
MYDGF	0.922088	-0.117024	0.7667765	1337	1893	1904	1706	1614	1414
AL139124.1	2	1	0.639194	2	2	0	0	5	3
ENC1	0.8134136	-0.297939	0.0404887	3936	3421	4273	2522	3480	3458
AASS	1.2969477	0.3751204	0.0159428	1472	981	1413	1171	1869	1974
KBTBD11-OT1	4	2	0.5760976	1	0	0	0	4	0
KBTBD11-OT1	4	2	0.5760976	1	0	0	0	4	0
WARS	0.8612516	-0.215493	0.2206662	2052	2392	2475	1903	1998	2058
ENAH	1.2813697	0.3576868	0.0109054	4603	3590	4598	3771	6026	6593
CXorf40A	0.8587533	-0.219684	0.3278021	445	524	539	427	442	426
CRIP2	2.442404	1.2883019	6.95E-14	341	381	476	1111	1023	792

CXorf40B	0.8873543	-0.172418	0.4713671	678	839	800	695	737	624
CRIP3	1.5555556	0.6374299	0.5666104	1	0	8	6	1	7
CRIP1	0.893617	-0.162271	0.8321217	36	26	32	31	32	21
ARVCF	0.6378505	-0.64871	0.0567167	168	119	141	117	79	77
AC004893.2	0.7647059	-0.387023	0.5148503	49	32	55	4	54	46
AC004893.1	0	#NUM!	0.2218177	2	0	5	0	0	0
AL354943.1	#DIV/0!	#DIV/0!	0.6554062	0	0	0	0	2	0
NPDC1	0.8359375	-0.258533	0.7002583	42	34	52	46	29	32
ZNRD1ASP	0.8833693	-0.178911	0.6454779	171	115	177	110	125	174
YIPF1	0.9949664	-0.00728	0.7287469	735	807	842	780	839	753
YIPF2	1.077218	0.1073102	0.2670606	540	625	661	624	692	651
YIPF3	0.9565829	-0.064038	0.9852	1681	1887	2121	1899	1894	1649
YIPF4	1.0518732	0.0729608	0.3867268	1216	998	1256	969	1390	1291
YIPF5	0.9784115	-0.031487	0.8984079	2068	1919	2220	1672	2308	2093
CRIPT	1.252451	0.3247541	0.0074682	515	469	648	629	750	665
HUWE1	0.811082	-0.30208	0.1029547	16142	18370	17717	9530	14622	18210
KLK14	0.44	-1.184425	0.3597984	2	14	9	3	0	8
UBE2G1	0.9263908	-0.110307	0.5780655	4989	4790	5518	3897	5227	5047
UBE2G2	0.9818919	-0.026364	0.7677999	2197	2074	2632	2063	2468	2247
AC010680.1	#DIV/0!	#DIV/0!	0.8030358	0	0	0	0	1	0
AC010680.2	0.8333333	-0.263034	0.985353	1	0	5	5	0	0
YIPF6	0.9909409	-0.013129	0.7772889	1613	1458	1786	1287	1691	1835
RTKN	0.8001392	-0.321677	0.0547792	1674	1896	2179	1538	1514	1548
RTL9	9.375	3.2288187	1.22E-10	4	14	6	78	72	75
OGDHL	0.6311239	-0.664005	0.0481282	87	111	149	61	100	58
RTL6	0.713467	-0.487081	0.0240149	406	480	510	231	342	423
RTL5	#DIV/0!	#DIV/0!	0.2116454	0	0	0	5	1	0
UBE2V2	1.0430195	0.0607661	0.409473	1245	1005	1446	1025	1476	1354
UBE2V1	1.1715481	0.2284162	0.379495	79	73	87	83	130	67
AL513343.1	1.2	0.2630344	0.8113718	3	1	6	1	6	5
ADGRF5P1	#DIV/0!	#DIV/0!	0.8030358	0	0	0	0	1	0
RTN2	1.1931499	0.2547753	0.0556461	501	579	701	795	668	662
RTN3	1.176263	0.2342106	0.0068066	3074	3032	3811	3462	4333	3870
RTN1	#DIV/0!	#DIV/0!	0.4737294	0	0	0	0	4	0
RTN4	1.2564304	0.3293308	0.0002903	11303	10190	12836	12193	16004	14935
AL445465.1	0	#NUM!	0.824326	1	0	0	0	0	0
AC007780.1	2.5	1.3219281	0.4381599	1	2	1	1	7	2
AL445465.2	#DIV/0!	#DIV/0!	0.8030358	0	0	0	0	1	0
AL450326.1	0.8712871	-0.19878	0.7867089	19	36	46	26	35	27
AC113167.1	0	#NUM!	0.4005425	3	1	0	0	0	0
CRIM1	0.810766	-0.302642	0.0558198	6607	5727	6986	3890	5656	6118
PGM5P2	0.3846154	-1.378512	0.3529486	5	5	3	0	3	2
RTP4	#DIV/0!	#DIV/0!	0.1240349	0	0	0	7	1	0
AC005387.1	0.6428571	-0.63743	0.7004508	0	7	7	0	2	7
AC005387.2	0.45	-1.152003	0.402746	1	18	1	2	7	0
UBE2Q1	0.9623505	-0.055366	0.9500998	1964	2223	2586	2160	2410	1948
UBE2Q2	1.1652299	0.2206146	0.0726812	737	540	811	713	947	773
AL356515.1	0	#NUM!	0.3235237	4	0	1	0	0	0
RFPL2	#DIV/0!	#DIV/0!	0.4785195	0	0	0	1	1	1
RFPL3	1	0	0.9890559	1	0	0	0	1	0
CIP2A	1.2181192	0.2846553	0.112126	795	371	865	647	971	856
AC010200.1	0.5	-1	0.8188512	2	0	0	0	1	0
UBE2R2	1.1187236	0.1618536	0.0693122	2486	2892	3146	3051	3347	3138
CXorf49B	1	0	0.9347171	2	0	2	3	0	1

TGFBRAP1	0.7691971	-0.378575	0.0089979	2115	2358	2390	1442	1946	1891
AC090907.1	3.5	1.8073549	0.2301267	2	0	2	4	8	2
AC090907.2	1.3	0.3785116	0.5878791	10	9	11	14	21	4
UBE2NL	1	0	0.9890559	0	0	1	1	0	0
AC016710.1	0.8115942	-0.30117	0.7201075	19	19	31	21	12	23
AL021331.1	0.8333333	-0.263034	0.9165975	0	2	4	0	3	2
LNCSRLR	1.2	0.2630344	0.8594808	8	0	2	3	8	1
AC008764.2	1.031746	0.0450879	0.765721	13	17	33	38	15	12
DPY19L2P3	1.1382114	0.1867685	0.3539244	151	181	160	136	193	231
AC008764.6	0.8461538	-0.241008	0.6538772	53	49	80	52	47	55
DPY19L2P1	0.375	-1.415037	0.1382065	11	14	15	2	2	11
DPY19L2P2	2.5	1.3219281	0.6279416	2	0	0	0	2	3
AC008764.9	0.6521739	-0.616671	0.6331647	4	8	11	0	15	0
ABAT	1.4719101	0.5576896	0.1881809	23	42	24	49	37	45
AC008764.8	0.4201681	-1.250962	0.0384975	30	21	68	19	17	14
ENO1	1.0496196	0.0698666	0.3007589	78663	97633	102787	96982	103727	92222
ENO2	1.0385604	0.0545851	0.5708779	207	254	317	243	330	235
ENO3	0.9278351	-0.10806	0.8575647	250	283	340	294	277	239
ENO4	0	#NUM!	0.6840298	0	0	2	0	0	0
RTTN	0.9037559	-0.145995	0.6130584	331	251	270	172	277	321
DEPDC1-AS1	6	2.5849625	0.3030898	0	0	1	2	3	1
AC139795.1	1.16	0.2141248	0.7453802	11	5	9	8	9	12
AC139795.2	0.6751269	-0.566769	0.2314361	74	37	86	38	34	61
AC139795.3	0.9230769	-0.115477	0.9450386	2	3	21	11	7	6
ANKK1	0	#NUM!	0.824326	1	0	0	0	0	0
ALG10B	1.0585339	0.0820675	0.5464691	705	508	615	438	786	711
AC009019.1	1.3333333	0.4150375	0.837068	0	0	3	0	2	2
GPR137B	0.7796742	-0.359057	0.0939899	358	429	502	316	322	367
GPR137C	1.1052632	0.1443899	0.6000089	77	46	48	91	57	41
AC020978.4	0.5833333	-0.777608	0.6096349	4	5	3	4	2	1
AC020978.5	1.2333333	0.3025628	0.6704624	18	3	9	10	13	14
RPL6P2	#DIV/0!	#DIV/0!	0.5371397	0	0	0	0	0	3
AC073254.1	0.7241379	-0.465664	0.5622434	8	20	30	11	22	9
AL596275.2	0	#NUM!	0.3473206	0	5	0	0	0	0
AC020978.2	0	#NUM!	0.355562	5	0	0	0	0	0
AC020978.3	2	1	0.7433853	2	0	0	0	3	1
AC020978.8	1.2	0.2630344	0.739343	4	4	7	6	6	6
RPS29P14	4	2	0.5810589	0	0	1	0	4	0
RPS29P16	1.5	0.5849625	0.8192369	0	0	2	0	2	1
RPS29P17	1	0	0.9659345	0	2	5	1	5	1
RPS29P19	0	#NUM!	0.6840298	0	0	2	0	0	0
ENSA	0.9476805	-0.077527	0.8936294	6005	7083	7822	6443	7135	6238
AC009113.1	0.6751152	-0.566794	0.0478003	161	112	161	85	111	97
AL353625.1	1.3846154	0.4694853	0.3950593	12	13	14	22	13	19
UBE2V1P15	0	#NUM!	0.824326	1	0	0	0	0	0
UBE2V1P13	0	#NUM!	0.824326	0	0	1	0	0	0
AC053503.2	1.1818182	0.2410081	0.7461551	3	7	12	4	3	19
AC099522.2	0.5	-1	0.1322635	29	26	29	19	6	17
AAED1	1.0825826	0.1144771	0.2341456	881	846	937	863	1125	896
TIMMDC1	0.9448743	-0.081806	0.915884	2108	2425	2705	2382	2322	2135
MYBPH	0.7142857	-0.485427	0.7816207	2	5	0	2	2	1
AC009509.1	0.5	-1	0.4730509	9	5	2	5	1	2
ABI2	1.094043	0.1296695	0.1314824	1106	1125	1395	1127	1470	1370
AC009509.4	1	0	0.9480217	0	0	2	1	1	0

AC124242.1	0.5454545	-0.874469	0.4677461	2	13	7	0	5	7
ABI1	1.0587666	0.0823846	0.3151598	2792	2411	3067	2252	3229	3275
AC124242.2	0	#NUM!	0.4460243	4	0	0	0	0	0
AC007448.2	0	#NUM!	0.824326	1	0	0	0	0	0
AC007448.3	0.5416667	-0.884523	0.4261927	2	15	7	4	5	4
AC005730.3	4.6666667	2.2223924	0.18728	0	2	1	1	8	5
FAM24B	0	#NUM!	0.6385096	0	2	0	0	0	0
AC006369.2	0.3333333	-1.584963	0.5469293	3	0	3	0	0	2
AC005730.2	4	2	0.5532124	1	0	0	0	0	4
FAM24A	#DIV/0!	#DIV/0!	0.6411933	0	0	0	0	0	2
AC012409.5	1	0	0.9890559	1	0	0	0	0	1
AC012409.2	0	#NUM!	0.824326	1	0	0	0	0	0
NHP2	0.8981916	-0.154905	0.539766	2721	3422	3866	3092	3109	2789
AL078605.1	0	#NUM!	0.6436235	2	0	0	0	0	0
FAM27B	2	1	0.6905599	0	2	0	3	0	1
IQCA1	0.7619048	-0.392317	0.7129756	11	4	6	2	7	7
ENY2	0.9892067	-0.015656	0.7064999	579	528	746	651	600	582
FAM27C	0	#NUM!	0.824326	0	0	1	0	0	0
TBXA2R	2.15	1.1043367	0.1589489	9	6	5	13	21	9
ABL1	0.845613	-0.241931	0.1440586	1903	2337	2302	1485	2098	1949
ABL2	0.7051662	-0.503965	0.0097098	1188	830	1021	506	789	848
AC097461.1	0.4615385	-1.115477	0.1610719	18	14	20	8	4	12
AC092814.1	0	#NUM!	0.6631647	1	0	1	0	0	0
AP002761.3	0.5333333	-0.906891	0.4203422	5	10	15	0	14	2
TVP23A	1.1470588	0.1979394	0.6924521	7	10	17	13	13	13
AP002761.4	0.362069	-1.465664	0.079388	22	7	29	9	7	5
TVP23B	1.1046951	0.1436482	0.2368193	713	522	618	609	732	706
TVP23C	1.0676692	0.0944647	0.5691516	95	68	103	96	94	94
AP002761.2	#DIV/0!	#DIV/0!	0.6099294	0	0	0	2	0	0
AC002351.1	1.7333333	0.7935491	0.2528754	11	12	7	12	25	15
KRT8P3	0.9666308	-0.048963	0.9387387	458	710	690	598	587	611
AL162412.1	#DIV/0!	#DIV/0!	0.5371397	0	0	0	0	0	3
PTBP1	0.83424	-0.261466	0.0702572	19245	22957	25076	16848	20045	19233
AC069277.1	0.3670886	-1.4458	0.02985	33	20	26	9	15	5
DNMT3L	2.0628352	1.0446286	8.73E-11	399	395	511	1007	917	768
AC069277.2	0	#NUM!	0.0926584	2	3	4	0	0	0
AP003110.1	#DIV/0!	#DIV/0!	0.1181139	0	0	0	0	2	7
KRT8P8	#DIV/0!	#DIV/0!	0.8030358	0	0	0	0	0	1
KRT8P5	0.3333333	-1.584963	0.6378737	1	1	1	0	1	0
DNMT3B	0.9346159	-0.097555	0.667257	15594	17389	18528	12813	17352	17978
IQCB1	1.1215862	0.1655405	0.172721	981	604	1088	930	1098	970
DNMT3A	0.7978583	-0.325796	0.0252244	1810	1997	2263	1467	1689	1687
KCNAB1	0.8514286	-0.232043	0.6588186	77	29	69	22	65	62
KCNAB2	0.8362147	-0.258055	0.3403566	290	413	396	324	341	254
KCNAB3	0.9583333	-0.061401	0.9721841	7	17	24	16	19	11
PTBP2	0.7638732	-0.388595	0.0745558	700	417	649	372	514	463
PTBP3	1.1976713	0.260232	0.0464048	11148	7875	10865	8637	13285	13874
AL031963.2	0	#NUM!	0.5781912	0	0	3	0	0	0
TXNDC12-AS1	2	1	0.8186965	0	0	1	0	0	2
AC026464.5	0.3333333	-1.584963	0.6404063	2	1	0	0	0	1
TRHDE-AS1	0.5952381	-0.748461	0.2891471	27	19	38	21	7	22
AC022395.1	0	#NUM!	0.0124379	4	9	3	0	0	0
AL031963.3	1.0833333	0.1154772	0.7731212	11	12	13	19	8	12
CX3CR1	0.9166667	-0.125531	0.9192682	3	8	1	4	7	0

AC093503.1	0	#NUM!	0.824326	0	0	1	0	0	0
WBP1	0.6666667	-0.584963	0.1836085	44	62	110	43	52	49
WBP2	0.9653245	-0.050914	0.9222185	2306	2647	2920	2604	2714	2282
AC093503.3	0	#NUM!	0.824326	1	0	0	0	0	0
WBP4	0.9824561	-0.025535	0.8494095	382	287	471	310	445	365
AC096632.1	#DIV/0!	#DIV/0!	0.2256364	0	0	0	3	3	0
AC096632.2	0	#NUM!	0.0907616	1	9	0	0	0	0
RBBP4	0.974446	-0.037346	0.8889775	5008	4864	5742	4354	5596	5265
RBBP5	0.8465658	-0.240306	0.2071334	1045	984	1145	659	942	1086
ANKAR	0.9487179	-0.075949	0.9667923	19	14	45	25	36	13
RBBP8	1.2663348	0.3406589	0.0565801	662	350	595	515	784	736
RBBP9	0.8985507	-0.154328	0.4825632	618	604	572	476	592	544
RBBP6	1.0814012	0.1129018	0.3228759	2228	1323	2444	1694	2560	2229
OSMR-AS1	#DIV/0!	#DIV/0!	0.1511264	0	0	0	1	2	4
RBBP7	0.9364296	-0.094758	0.7370933	7777	7304	9443	6913	8377	7675
FDXACB1	0.7785714	-0.361099	0.51864	49	38	53	48	35	26
SLC46A3	0.5289256	-0.918863	0.1085939	34	54	33	13	12	39
AC019117.1	1.3333333	0.4150375	0.8999008	0	3	0	0	0	4
SLC46A1	0.842654	-0.246988	0.2933599	302	394	359	274	311	304
AL354702.1	0.3333333	-1.584963	0.5332054	3	0	3	0	2	0
SLC46A2	1	0	0.9582877	5	4	0	0	5	4
ABRA	0	#NUM!	0.3626892	1	0	4	0	0	0
GOLGA2	0.9969463	-0.004412	0.6460867	4210	4337	5207	3879	5014	4819
GOLGA3	0.7815248	-0.355637	0.0153349	2681	3063	3241	1962	2437	2623
GOLGA4	1.2642078	0.3382336	0.057361	2155	994	2165	1802	2829	2087
GOLGA5	1.1407116	0.1899341	0.1262931	2169	1328	2096	1782	2371	2227
AC127526.4	0.375	-1.415037	0.5332297	2	0	6	0	1	2
GOLGA1	1.0095714	0.0137429	0.7272155	797	785	821	614	913	899
GOLGA7	1.2540046	0.3265426	0.0014285	1586	1500	1721	1727	2244	2057
AC005000.1	0.9506347	-0.073037	0.9516106	213	225	271	218	257	199
AC005000.2	#DIV/0!	#DIV/0!	0.6554062	0	0	0	0	2	0
ABT1	0.8358233	-0.25873	0.1264929	1796	2122	2173	1654	1770	1667
MCCC2	0.6111111	-0.710493	0.1967951	30	42	36	27	25	14
IQCF6	0.2	-2.321928	0.2359419	3	5	2	0	2	0
IQCF3	2	1	0.8186965	0	0	1	0	0	2
RRP7BP	0.7451182	-0.424459	0.0698704	287	340	346	225	213	287
AC024060.1	1.0227273	0.0324215	0.7893119	27	12	49	34	34	22
MCCC1	0.9651056	-0.051241	0.9453236	1009	934	1324	868	1119	1166
MTUS1	1.245766	0.3170332	0.0568989	1014	543	982	801	1169	1193
MTUS2	2.2777778	1.187627	0.1767424	1	9	8	6	16	19
AC114485.1	0.3829787	-1.384664	0.0990221	18	13	16	8	7	3
MTURN	0.7912525	-0.33779	0.1870414	306	263	437	258	253	285
AC013726.1	#DIV/0!	#DIV/0!	0.5070017	0	0	0	3	0	0
AL358178.1	0	#NUM!	0.6436235	2	0	0	0	0	0
FP236241.2	#DIV/0!	#DIV/0!	0.8030358	0	0	0	0	0	1
TSHZ1	1.3652174	0.4491307	0.2236485	37	25	53	33	55	69
TSHZ2	1.6571429	0.728698	0.3921932	16	8	11	3	6	49
FP236241.1	0.9615385	-0.056584	0.9536029	6	7	13	11	8	6
GMCL1	1.1205534	0.1642114	0.2141581	1082	896	1058	795	1289	1318
RPL5P11	0	#NUM!	0.824326	0	0	1	0	0	0
RPL5P17	1.9166667	0.9385995	0.3317207	6	3	3	11	6	6
STK11IP	0.9499249	-0.074115	0.9959188	642	587	768	698	625	574
RNU7-123P	0.2	-2.321928	0.4376609	4	0	1	0	1	0
AC010931.2	0	#NUM!	0.5378194	0	2	1	0	0	0

GOLGB1	1.2083425	0.2730294	0.0930953	1762	942	1851	1343	2276	1885
RPL5P18	#DIV/0!	#DIV/0!	0.5415473	0	0	0	0	2	1
AC008035.1	1	0	0.9784571	3	2	0	0	1	4
CX3CL1	1.797619	0.8460873	0.0018435	68	98	86	172	167	114
AL591721.1	#DIV/0!	#DIV/0!	0.8030358	0	0	0	0	1	0
AL132657.1	#DIV/0!	#DIV/0!	0.2106225	0	0	0	4	0	2
AC005480.1	7	2.8073549	0.3462448	1	0	0	0	7	0
AC005480.2	0	#NUM!	0.5781912	0	0	3	0	0	0
ATP1B1P1	0.5555556	-0.847997	0.7037056	2	0	7	0	5	0
FAM20B	0.7798805	-0.358675	0.0225651	1639	1521	1860	992	1433	1490
FAM20C	0	#NUM!	0.824326	0	1	0	0	0	0
FAM20A	0.8518519	-0.231326	0.857824	3	10	14	5	9	9
RPL34-AS1	0.6666667	-0.584963	0.6911847	10	0	5	0	6	4
MYCBP	1.2587327	0.331972	0.0078588	809	713	940	1047	1158	894
AC133435.1	2.3636364	1.2410081	0.2641675	9	0	2	14	4	8
AL139125.2	1.5	0.5849625	0.8582217	2	0	0	0	1	2
AC015871.3	0.9183673	-0.122857	0.9116609	87	44	114	68	69	88
AC015871.4	#DIV/0!	#DIV/0!	0.8030358	0	0	0	0	0	1
AC104971.2	0.5	-1	0.5145057	4	3	11	3	0	6
AC013476.1	1	0	0.9716937	0	3	0	3	0	0
AC098934.1	0.7513778	-0.41239	0.015879	952	1099	1215	817	798	839
AC098934.2	0.9760479	-0.034976	0.9300924	129	81	124	106	130	90
AC110760.2	0.4	-1.321928	0.62387	0	5	0	0	0	2
AC098934.3	1.7058824	0.7705182	0.4329817	5	3	9	1	10	18
AC098934.4	#DIV/0!	#DIV/0!	0.4242335	0	0	0	0	3	1
GPR143P	#DIV/0!	#DIV/0!	0.6411933	0	0	0	0	0	2
FMO11P	#DIV/0!	#DIV/0!	0.3096126	0	0	0	5	0	0
ILVBL	1.0264407	0.0376503	0.4745426	1634	2043	2223	2123	2103	1830
RPL7L1	0.8883422	-0.170813	0.2743123	4991	5220	5641	3937	5145	5000
EOGT	0.9983633	-0.002363	0.8254673	178	187	246	152	256	202
AC091100.1	#DIV/0!	#DIV/0!	0.8030358	0	0	0	0	0	1
AC018529.2	1	0	0.9905468	2	0	1	0	0	3
NID1	1.1183334	0.1613504	0.1602443	6940	7353	7476	5877	8975	9493
NID2	1.4444444	0.5305147	0.42154	10	6	11	13	13	13
HHATL-AS1	0	#NUM!	0.824326	1	0	0	0	0	0
PTCH1	1.0558091	0.0783489	0.535654	889	1003	957	668	1133	1207
PTCH2	0.3	-1.736966	0.1189348	4	12	14	1	6	2
ANKRD44-IT1	#DIV/0!	#DIV/0!	0.3981469	0	0	0	4	0	0
RPL5P23	0.6785714	-0.559427	0.6531139	3	8	17	6	3	10
RPL5P22	1.6666667	0.7369656	0.714925	0	2	1	3	2	0
WTAPP1	1.6666667	0.7369656	0.4859611	3	10	2	6	13	6
RPL5P29	0	#NUM!	0.4217812	0	0	5	0	0	0
RPL5P34	1.2424942	0.3132391	0.1123578	138	125	170	198	198	142
AL645940.1	#DIV/0!	#DIV/0!	0.2533058	0	0	0	0	7	0
RPL7P9	1.1370654	0.1853152	0.0669008	2401	2014	3034	2972	2960	2538
RPL5P30	0.2857143	-1.807355	0.4776477	2	0	5	1	0	1
RPL7P1	1.2278755	0.2961643	0.0088761	1235	1034	1426	1518	1643	1376
RPL7P4	3	1.5849625	0.5682898	2	0	0	0	0	6
GCSAML	#DIV/0!	#DIV/0!	0.8030358	0	0	0	1	0	0
AC215522.2	0.8888889	-0.169925	0.9309118	3	1	5	0	5	3
AC007541.1	1.1071429	0.1468414	0.7360627	23	16	17	21	25	16
ACAN	0.3009709	-1.732304	1.70E-06	93	116	100	35	30	28
PTCD3	1.1124927	0.1537959	0.1491489	3205	2169	3231	2677	3646	3250
PTCD1	0.665272	-0.587984	0.1924651	60	88	91	75	48	36

PTCD2	0.9603384	-0.058385	0.953502	727	493	671	479	637	700
NIFK	1.1489413	0.2003052	0.0516506	1403	1105	1601	1456	1774	1491
TSPEAR	1.5568182	0.6386005	0.1426177	21	16	51	27	56	54
HMGB1P31	0.9583333	-0.061401	0.9919689	3	6	15	4	14	5
ACCS	#DIV/0!	#DIV/0!	0.1161291	0	0	0	1	4	3
F11R	0.9428614	-0.084882	0.8640857	3844	4539	4918	4227	4542	3772
AL035541.1	0.125	-3	0.2709892	0	2	6	0	1	0
AP000851.2	0	#NUM!	0.5781912	0	0	3	0	0	0
AC109631.1	#DIV/0!	#DIV/0!	0.6099294	0	0	0	2	0	0
HMGB1P24	0.6666667	-0.584963	0.7573575	4	2	0	1	1	2
AC073111.5	1.0148148	0.0212165	0.7992599	32	49	54	56	41	40
AC115223.1	1.1199295	0.1634079	0.1559062	692	711	865	913	931	696
AC073111.1	0	#NUM!	0.2219629	0	7	0	0	0	0
HMGB1P21	0	#NUM!	0.101298	1	3	5	0	0	0
HMGB1P20	#DIV/0!	#DIV/0!	0.6099294	0	0	0	2	0	0
ACE2	0.5	-1	0.4018088	7	7	8	6	2	3
HMGB1P14	0.1538462	-2.70044	0.21349	4	0	9	0	0	2
GAPDHP15	7	2.8073549	0.2469806	1	0	0	5	0	2
THAP7-AS1	0.7874564	-0.344728	0.3290075	74	114	99	68	85	73
RNU6-1	#DIV/0!	#DIV/0!	0.8030358	0	0	0	1	0	0
AC008763.1	#DIV/0!	#DIV/0!	0.8030358	0	0	0	0	1	0
HMGB1P10	1.0077071	0.0110764	0.7954493	190	169	160	168	179	176
RNU6-8	0.2941176	-1.765535	0.2575929	8	1	8	0	1	4
F13B	#DIV/0!	#DIV/0!	0.5070017	0	0	0	3	0	0
AC110620.1	0	#NUM!	0.4460243	4	0	0	0	0	0
AL133245.1	0	#NUM!	0.5387425	3	0	0	0	0	0
ACHE	1.5015823	0.5864835	5.01E-05	353	418	493	632	664	602
SNRPCP2	0.5	-1	0.6951127	2	1	1	1	1	0
CATSPER2P1	0.7421875	-0.430144	0.4058425	36	33	59	17	42	36
SNRPCP3	#DIV/0!	#DIV/0!	0.247607	0	0	0	0	2	4
PCMTD2	0.9365612	-0.094555	0.7970925	1904	1486	2269	1362	1887	2051
AC015878.1	0	#NUM!	0.5387425	3	0	0	0	0	0
GAPDHP42	0	#NUM!	0.6840298	0	0	2	0	0	0
GAPDHP44	#DIV/0!	#DIV/0!	0.8030358	0	0	0	0	1	0
NINL	1.0578035	0.0810716	0.408422	289	348	401	339	378	381
GAPDHP40	0.6666667	-0.584963	0.8147581	0	5	1	3	0	1
PCMTD1	1.0418327	0.0591236	0.5673651	558	301	647	411	634	524
EPS15L1	0.9335949	-0.099131	0.7424014	3124	3530	3797	3172	3454	3131
SETSIP	1.440367	0.5264364	0.1946326	37	43	29	34	54	69
AL683813.1	1.1290323	0.1750867	0.6728519	3	3	25	25	6	4
POC1B-AS1	0.59375	-0.752072	0.5473128	3	8	21	1	2	16
AC004063.1	#DIV/0!	#DIV/0!	0.5070017	0	0	0	3	0	0
AC021074.1	#DIV/0!	#DIV/0!	0.2497949	0	0	0	0	3	3
GAPDHP51	0	#NUM!	0.824326	1	0	0	0	0	0
GAPDHP52	0.6666667	-0.584963	0.858005	0	0	6	0	1	3
NIP7	0.8455909	-0.241968	0.1182202	1743	1729	1994	1233	1798	1591
FABP7P1	0	#NUM!	0.2219629	0	7	0	0	0	0
AC006460.2	1.3272727	0.4084648	0.4299166	14	19	22	14	23	36
HMGB1P49	0	#NUM!	0.824326	1	0	0	0	0	0
AC006460.1	0	#NUM!	0.824326	0	1	0	0	0	0
GAPDHP24	0	#NUM!	0.824326	0	1	0	0	0	0
HMGB1P45	0	#NUM!	0.6436235	2	0	0	0	0	0
GAPDHP21	2.3	1.2016339	0.1064754	6	9	5	20	11	15
GAPDHP22	2	1	0.8186965	0	0	1	0	0	2

HMGB1P41	0.4615385	-1.115477	0.6463385	0	0	13	2	0	4
AC134511.1	1.1666667	0.2223924	0.9444547	2	4	0	1	6	0
GAPDHP39	0	#NUM!	0.824326	0	0	1	0	0	0
AC009498.1	0	#NUM!	0.1905915	0	1	7	0	0	0
AC010203.1	0.4081633	-1.292782	0.1160486	18	17	14	1	9	10
AC010203.2	2.6666667	1.4150375	0.541762	0	3	0	0	2	6
GAPDHP35	0	#NUM!	0.6631647	1	0	1	0	0	0
TTC30B	0.9775785	-0.032716	0.8676625	152	99	195	135	154	147
GAPDHP37	3.5	1.8073549	0.3598493	0	0	2	4	1	2
GAPDHP38	0.2857143	-1.807355	0.4447102	1	1	5	1	1	0
TTC30A	1.2006079	0.2637651	0.1079509	239	195	224	236	278	276
GAPDHP32	4	2	0.223423	0	1	3	0	9	7
GAPDHP33	0	#NUM!	0.6840298	0	0	2	0	0	0
CRLF3	0.8791146	-0.185877	0.3701374	568	561	633	444	624	481
CRLF1	1.018018	0.0257631	0.836516	36	28	47	33	48	32
PLEKHA3P1	2.3333333	1.2223924	0.6116547	0	0	3	0	7	0
AC244093.4	1.5	0.5849625	0.8573244	1	1	0	0	2	1
AC244093.5	0.3333333	-1.584963	0.6833782	1	0	2	0	0	1
AC108063.1	#DIV/0!	#DIV/0!	0.6482162	0	0	0	0	1	1
IGHEP2	0.8333333	-0.263034	0.9012954	4	0	2	1	4	0
AC018761.3	0	#NUM!	0.824326	0	0	1	0	0	0
ACLY	0.8659	-0.207728	0.1917301	9092	10430	11261	7019	9697	9939
AL354793.1	0.6666667	-0.584963	0.7096344	8	3	1	5	2	1
VPS25P1	3	1.5849625	0.4765113	0	2	0	3	1	2
AC018521.7	0.3076923	-1.70044	0.1246941	10	12	4	2	4	2
AC018521.5	2.1025641	1.0721498	0.0064642	19	25	34	62	54	48
AC018521.6	0.9444444	-0.082462	0.9998312	6	9	3	11	2	4
AC018521.3	1.6666667	0.7369656	0.7332361	1	2	0	3	2	0
HSP90B1	1.1196376	0.1630319	0.1273975	55546	35132	60328	50868	64044	54160
GAPDHP69	#DIV/0!	#DIV/0!	0.8030358	0	0	0	1	0	0
GAPDHP64	0	#NUM!	0.5781912	0	0	3	0	0	0
GAPDHP65	0.7804878	-0.357552	0.6940395	17	13	11	15	12	5
RAB9BP1	0	#NUM!	0.6385096	0	2	0	0	0	0
AC012506.2	1.5714286	0.6520767	0.7052697	1	5	1	0	2	9
GAPDHP60	0	#NUM!	0.6840298	0	0	2	0	0	0
AC012506.3	0.6363636	-0.652077	0.7178403	4	0	7	1	4	2
GAPDHP61	1	0	0.9265675	0	0	3	2	1	0
AC012506.4	0.7272727	-0.459432	0.7780562	4	1	6	0	6	2
GAPDHP62	#DIV/0!	#DIV/0!	0.6411933	0	0	0	0	0	2
GAPDHP63	0.7692308	-0.378512	0.8163778	4	3	6	4	5	1
GAPDHP70	0	#NUM!	0.3482063	0	0	6	0	0	0
AC244093.1	2	1	0.6540525	0	0	2	4	0	0
AC244093.2	#DIV/0!	#DIV/0!	0.8030358	0	0	0	0	1	0
DMXL1	1.077783	0.1080667	0.493055	919	416	812	542	882	890
AC244093.3	1	0	0.9756864	2	0	0	0	2	0
AL049776.1	1.5555556	0.6374299	0.2063063	22	13	19	34	16	34
DMXL2	1.2681505	0.342726	0.3587837	824	347	716	490	877	1026
ACO1	0.7805794	-0.357383	0.012105	1694	1826	2072	1223	1582	1560
ACO2	0.7572626	-0.401134	0.0039463	2680	2948	3081	2055	2283	2257
NIT1	0.9688716	-0.045623	0.9529984	816	879	875	845	929	716
GAPDHP72	0.6666667	-0.584963	0.8319544	0	2	1	0	2	0
NIT2	0.9209402	-0.118821	0.7078554	891	841	1076	866	904	816
GAPDHP74	0	#NUM!	0.824326	1	0	0	0	0	0
MCFD2	0.9642658	-0.052497	0.950676	5383	4939	5825	4005	5721	5844

AL732292.2	0	#NUM!	0.6436235	2	0	0	0	0	0
AC068205.2	0.8571429	-0.222392	0.68412	37	39	29	24	28	38
ACP6	1.0365769	0.0518272	0.4704986	421	471	557	466	556	480
ACP4	1.1666667	0.2223924	0.9216137	1	5	0	1	2	4
ACP5	0.7648553	-0.386741	0.0187091	1187	1274	1477	1013	1076	923
ACP2	0.8766816	-0.189875	0.3791966	526	652	606	469	600	495
ACP1	1.0485684	0.068421	0.2331897	5214	5314	6726	5732	6570	5790
AC005332.10	0	#NUM!	0.6436235	2	0	0	0	0	0
EIF5A	0.8990023	-0.153603	0.5106534	11681	14740	15976	12966	13456	11693
EIF5B	1.6713843	0.7410435	0.0127664	5170	2615	6417	8068	9454	6215
ACPP	0.6021651	-0.731769	0.0052213	272	226	241	87	172	186
MGRN1	0.8273417	-0.273445	0.0871597	1845	1985	2234	1586	1658	1773
AC010099.4	1.9411765	0.9569313	0.4012072	14	0	3	6	22	5
EIF4H	0.8919765	-0.164922	0.3369438	19125	23472	24842	17469	21901	20784
EIF4E	1.2084978	0.2732148	0.0198671	1471	1208	1628	1336	2037	1832
EIF4B	1.0584836	0.0819989	0.1701538	18446	18248	22314	18158	23027	21274
NTRK1	0.7777778	-0.36257	0.8366309	1	6	2	0	0	7
NTRK2	1.75	0.8073549	0.6614672	2	2	0	5	2	0
AC005777.1	#DIV/0!	#DIV/0!	0.8030358	0	0	0	0	1	0
NTRK3	0	#NUM!	0.1151465	2	5	1	0	0	0
TTC39C	1.453202	0.5392352	0.0005605	242	252	318	326	449	405
AC090518.1	0	#NUM!	0.824326	1	0	0	0	0	0
TTC39A	2.8828125	1.527477	9.86E-06	36	57	35	80	163	126
TTC39B	0.9950495	-0.00716	0.8819275	119	140	145	98	122	182
AL033384.1	2	1	0.8319548	0	1	0	0	2	0
AL033384.2	1.0952381	0.1312445	0.8885949	11	3	7	1	6	16
EIF3M	1.0057945	0.0083356	0.5744364	7121	5660	8446	6340	8227	6783
EIF3K	0.9390685	-0.090698	0.891185	7252	8583	9866	8660	8521	6954
EIF3L	0.9280069	-0.107793	0.608036	5588	6273	6738	4715	6313	6232
EIF3I	1.000162	0.0002337	0.6157299	5290	6306	6924	6183	6531	5809
EIF3J	0.9797377	-0.029533	0.8257702	4340	3492	4901	3530	4700	4245
EIF3G	0.9522838	-0.070536	0.9921907	3055	3884	4336	3876	3689	3172
EIF3H	1.0219494	0.0313237	0.3958081	6820	6013	8261	6809	7847	6901
EIF3E	1.213013	0.2785951	0.0214587	8683	5596	9574	8491	11038	9405
EIF3F	1.0190319	0.0271992	0.4872656	7342	8290	9221	8909	8974	7443
EIF3C	0.7870075	-0.345551	0.0290573	885	938	994	647	798	772
EIF3D	0.8156513	-0.293976	0.0513337	11391	13744	14670	10379	11709	10379
EIF3A	1.2434115	0.3143038	0.0139386	10246	6731	11292	9172	13310	12668
AC096533.1	0	#NUM!	0.4117982	0	3	1	0	0	0
EIF3B	0.8747255	-0.193098	0.2269283	8335	9503	10396	7685	8939	8073
PRKCQ-AS1	0.8202532	-0.285859	0.3458608	124	138	133	94	112	118
ACTB	0.898836	-0.15387	0.478718	201802	238929	256365	213542	221611	191422
NRADDP	1.25	0.3219281	0.847739	2	2	0	5	0	0
MIR7854	#DIV/0!	#DIV/0!	0.5371397	0	0	0	0	0	3
EPC1	1.1292683	0.1753883	0.2558545	274	229	317	226	324	376
EIF2D	0.9141058	-0.129567	0.6192848	1147	1360	1463	1179	1388	1062
EPC2	1.2812354	0.3575355	0.0411547	773	524	840	575	1083	1080
RNU4-77P	0	#NUM!	0.6436235	2	0	0	0	0	0
MIR7851	#DIV/0!	#DIV/0!	0.6099294	0	0	0	2	0	0
EIF2A	1.1786293	0.2371101	0.0831815	3391	1911	3336	2894	3978	3309
CIRBP	0.7969914	-0.327364	0.0704178	1942	2171	2734	1908	1864	1685
CSNK1D	0.7740501	-0.369501	0.0182976	2923	3501	3919	2654	2742	2610
CSNK1E	0.9541864	-0.067657	0.9814891	1551	1649	1864	1647	1649	1536
MIR7849	0.5	-1	0.8188512	2	0	0	0	1	0

CSNK2B	0.7482014	-0.418501	0.5814443	36	31	72	57	27	20
RNU4-78P	14	3.8073549	0.0886937	0	1	0	0	12	2
EIF1B	1.0118139	0.016944	0.5544663	1197	1318	1548	1379	1502	1230
AL023284.4	1.1515152	0.2035334	0.755011	8	11	14	5	16	17
AL049870.3	1	0	0.9073448	9	5	13	12	11	4
AL049870.2	1	0	0.9424071	6	0	3	6	3	0
XRRA1	0.7509778	-0.413158	0.0940201	225	259	283	123	242	211
AC027544.1	#DIV/0!	#DIV/0!	0.2758481	0	0	0	4	0	1
AC138969.1	1.125	0.169925	0.7295283	8	12	12	17	8	11
AC027544.2	3	1.5849625	0.6552898	1	0	0	0	2	1
AC027544.3	0.1428571	-2.807355	0.3120456	3	0	4	0	1	0
AC138969.2	0.6513761	-0.618437	0.2938155	29	52	28	7	21	43
RAB27A	1.0685764	0.09569	0.4480985	380	385	387	313	438	480
RAB27B	7.5	2.9068906	0.1204487	0	1	1	0	5	10
IL1F10	1.2777778	0.353637	0.6792761	7	3	8	8	13	2
AC004803.1	1.0273973	0.0389941	0.8926531	21	15	37	6	31	38
AL391244.1	0.9598901	-0.059059	0.9872714	559	627	634	579	618	550
AL391244.2	1.2857143	0.3625701	0.6545413	2	2	10	9	7	2
AL391244.3	1.01	0.0143553	0.7193032	116	118	166	138	140	126
ACY3	#DIV/0!	#DIV/0!	0.4121728	0	0	0	0	1	3
RN7SL648P	0.3333333	-1.584963	0.6628865	2	0	1	0	0	1
ACY1	3	1.5849625	0.6529844	0	0	1	0	1	2
RAB22A	0.8332683	-0.263147	0.1479938	830	756	975	546	786	802
AC005537.1	2	1	0.3858048	2	1	5	4	6	6
EPG5	0.9345709	-0.097624	0.7174865	1156	980	1150	652	1168	1251
AL590399.3	0	#NUM!	0.824326	1	0	0	0	0	0
AC093788.1	1.5609756	0.642448	0.2876404	22	4	15	29	19	16
PBRM1	0.8585481	-0.220029	0.3187342	1853	1304	1926	947	1657	1760
AL773545.3	1	0	0.9890559	0	0	1	0	0	1
AC103808.6	#DIV/0!	#DIV/0!	0.6099294	0	0	0	2	0	0
AC103808.4	#DIV/0!	#DIV/0!	0.5371397	0	0	0	0	0	3
AC103808.3	3	1.5849625	0.6552898	1	0	0	0	2	1
FAM177A1	1.022164	0.0316266	0.4556629	1309	1289	1598	1268	1588	1433
AC007204.1	#DIV/0!	#DIV/0!	0.4909737	0	0	0	2	0	1
AC113410.1	1.3333333	0.4150375	0.6352093	7	2	9	4	12	8
AC113410.3	1.2352941	0.3048546	0.7370181	1	4	12	1	6	14
ATP5BP1	0	#NUM!	0.824326	0	0	1	0	0	0
AL031727.1	0.78	-0.358454	0.612549	18	18	14	10	19	10
SCHLAP1	0	#NUM!	0.6840298	0	0	2	0	0	0
AL035448.1	1.5	0.5849625	0.8695629	0	2	0	0	0	3
SLX4IP	0.9144603	-0.129008	0.6912908	351	260	371	255	327	316
OR13I1P	#DIV/0!	#DIV/0!	0.8030358	0	0	0	0	1	0
AC093642.2	0	#NUM!	0.5337954	0	3	0	0	0	0
ANKS6	0.5307018	-0.914027	0.0003619	296	399	445	251	187	167
HIGD1A	1.1128443	0.1542517	0.1175719	1358	1207	1538	1525	1710	1331
HIGD1B	0.2	-2.321928	0.2697697	6	0	4	0	1	1
NCKIPSD	0.9066587	-0.141368	0.5890516	999	1093	1272	1024	1008	1018
AP006248.2	0	#NUM!	0.6436235	2	0	0	0	0	0
SPRY4-AS1	4	2	0.1604902	5	0	0	10	6	4
AC074085.2	0	#NUM!	0.6385096	0	2	0	0	0	0
AP006248.3	2	1	0.7348385	3	0	0	0	6	0
AP006248.4	#DIV/0!	#DIV/0!	0.8030358	0	0	0	1	0	0
AC234781.1	0	#NUM!	0.5436524	2	0	1	0	0	0
AC234781.2	0	#NUM!	0.824326	0	0	1	0	0	0

ADA2	1.3636364	0.447459	0.4270663	8	38	20	22	24	44
HIGD2B	0	#NUM!	0.3958203	2	2	0	0	0	0
HIGD2A	0.9176471	-0.123989	0.8713024	454	568	678	663	513	384
CUEDC2	1.0531915	0.0747678	0.3866437	460	580	652	606	605	571
NGLY1	0.894213	-0.161309	0.4537035	943	744	1026	687	929	810
ADAR	1.1065519	0.1460711	0.2047518	8924	9759	9782	7631	11159	12708
ADAL	0.8529718	-0.22943	0.3600676	357	255	347	242	319	257
AC010442.2	3.5	1.8073549	0.0795758	3	1	4	10	5	13
EPN1	1.0004671	0.0006737	0.6162809	3066	3604	4035	3922	3523	3265
EPN2	0.8337688	-0.262281	0.1181669	952	994	1116	700	907	946
AC073257.2	#DIV/0!	#DIV/0!	0.5371397	0	0	0	0	0	3
EPN3	0.7572973	-0.401068	0.0320546	538	582	730	453	524	424
OR2B11	1.5	0.5849625	0.8898407	0	2	0	0	3	0
CUEDC1	0.8147882	-0.295503	0.1493485	420	429	544	330	460	345
AGPAT5	0.9630689	-0.054289	0.9664723	1351	1124	1370	1035	1376	1292
ANKS3	0.9715385	-0.041657	0.8967993	466	402	432	480	385	398
ADD3	1.3740809	0.4584669	0.0043102	1691	1046	1615	1424	2222	2334
AGPAT1	1.083533	0.1157431	0.2226281	798	1004	1119	1044	1127	994
AGPAT2	1.0632653	0.0885016	0.4340902	258	336	386	386	374	282
ADD1	0.9474019	-0.077952	0.8565696	4204	4678	5263	4110	4737	4554
AGPAT3	1.073857	0.1028019	0.38827	226	276	351	284	274	358
ADD2	0.5	-1	0.7932724	0	0	4	0	0	2
AGPAT4	0.8140244	-0.296856	0.1427069	426	389	497	275	429	364
AL357093.2	1	0	0.9362872	2	0	4	2	0	4
WDCP	1.0562471	0.0789474	0.4001231	742	654	773	614	784	893
C10orf10	0.8044094	-0.313998	0.1509853	805	1116	1254	900	846	808
SSRP1	0.8540202	-0.227658	0.16393	5707	6589	7330	5427	6022	5312
AC093159.1	2.3333333	1.2223924	0.5561901	3	0	0	2	3	2
EPOP	1.1783987	0.2368278	0.0682834	484	670	707	707	784	702
EPOR	1.0079787	0.0114652	0.8060399	101	132	143	99	130	150
RHGEF26-AS	0.3333333	-1.584963	0.6638759	0	3	0	0	1	0
EPRS	1.2184443	0.2850403	0.0513557	6869	3772	6535	5485	8054	7389
EPS8	0.7912621	-0.337772	0.0745214	974	733	971	526	774	819
RNU6-751P	0	#NUM!	0.6385096	0	2	0	0	0	0
ADGB	#DIV/0!	#DIV/0!	0.6099294	0	0	0	2	0	0
AC008522.1	4.5	2.169925	0.3174541	0	1	1	0	7	2
AC090519.1	4	2	0.1986871	2	0	2	8	8	0
MSL3P1	0.9	-0.152003	0.8863257	14	4	12	4	16	7
BX679664.3	1.1421435	0.1917439	0.0341272	2075	1889	2642	2427	2710	2408
OOEP-AS1	#DIV/0!	#DIV/0!	0.6411933	0	0	0	0	0	2
C10orf25	0.6666667	-0.584963	0.7108966	2	5	2	0	2	4
ANAPC13	1.1156615	0.1578993	0.1010395	1419	1110	1500	1405	1541	1549
ANAPC15	0.9093484	-0.137095	0.8121336	90	130	133	119	103	99
ANAPC16	1.0856206	0.1185201	0.2261555	710	836	895	783	1011	856
ADH5	1.2720724	0.3471808	0.0002309	8256	9126	10243	11129	12343	11669
ANAPC10	1.2585034	0.3317091	0.0750839	200	165	223	269	280	191
ADH6	4.2	2.0703893	0.1065284	3	0	2	11	2	8
ANAPC11	0.9609659	-0.057443	0.9360417	1704	2113	2229	2194	2027	1589
ADH4	1	0	0.978278	0	0	2	0	2	0
C10orf55	1	0	0.9756864	2	0	0	0	2	0
ADI1	0.7550129	-0.405427	0.0111271	1784	2101	2299	1540	1716	1413
MAGEA8-AS1	1.85	0.8875253	0.2942763	9	3	8	5	10	22
TOLLIP	0.9764619	-0.034364	0.8329038	741	947	1031	955	925	775
C10orf82	1.8484848	0.8863432	0.1531766	10	6	17	13	25	23

GMEB1	1.1122965	0.1535414	0.144536	589	541	651	659	675	647
GMEB2	0.7749652	-0.367797	0.0295067	1244	1522	1540	1090	1110	1137
AC123905.1	#DIV/0!	#DIV/0!	0.8030358	0	0	0	0	1	0
C10orf76	1.1203117	0.1639002	0.1413934	680	652	721	641	797	862
GLYCTK-AS1	0.0833333	-3.584963	0.0876073	3	4	5	0	0	1
ADIG	0	#NUM!	0.0768277	1	1	9	0	0	0
C10orf71	#DIV/0!	#DIV/0!	0.8030358	0	0	0	0	0	1
AL109976.1	0.5490196	-0.86507	0.3002029	15	16	20	15	5	8
AC115618.2	0.726087	-0.461786	0.2144256	71	68	91	39	62	66
AC115618.3	0.0810811	-3.624491	0.0058084	9	7	21	0	1	2
AC115618.1	0.7534247	-0.408465	0.5645238	15	28	30	22	18	15
ATP2C2-AS1	1.037037	0.0524674	0.9438086	8	10	9	4	16	8
TP53TG3	0.963964	-0.052949	0.9310075	35	23	53	38	35	34
TP53TG1	0.7333333	-0.447459	0.7521025	5	5	5	4	7	0
TP53TG5	0.7123288	-0.489385	0.508173	24	20	29	14	7	31
C10orf90	4	2	0.5246085	1	0	0	0	1	3
C10orf91	1	0	0.8468994	11	16	42	24	26	19
C10orf95	3	1.5849625	0.4940935	1	0	1	0	1	5
AC034213.1	0.3478261	-1.523562	0.2167088	4	7	12	1	2	5
ADM5	1.5818182	0.6615838	0.1675307	14	12	29	38	21	28
ADM2	1.6820513	0.7502217	5.90E-06	281	398	491	647	708	613
C10orf88	1.2106703	0.275806	0.1028719	236	253	242	254	357	274
AC078785.2	0	#NUM!	0.824326	0	1	0	0	0	0
RPL10L	0.2	-2.321928	0.2864431	7	0	3	0	2	0
RPL10A	0.9982455	-0.002533	0.6181412	27193	31616	35803	31539	34021	28886
VDAC3P1	0.5	-1	0.8652929	0	0	2	0	1	0
AL357833.1	0.64	-0.643856	0.6195055	4	5	16	0	15	1
AC106886.5	0.7692308	-0.378512	0.8873448	1	5	7	7	0	3
AC005776.1	1	0	0.9544849	0	0	3	0	0	3
GSTA10P	1	0	0.9890559	0	1	0	0	1	0
AC005776.2	0.8095238	-0.304855	0.9136845	7	4	10	14	2	1
MINDY4B	#DIV/0!	#DIV/0!	0.6099294	0	0	0	2	0	0
TTC21A	0.6846847	-0.546488	0.3340329	22	41	48	27	23	26
AC106886.1	#DIV/0!	#DIV/0!	0.3981469	0	0	0	4	0	0
AC106886.4	1.1666667	0.2223924	0.6704859	7	10	13	18	7	10
AAGAB	0.9391701	-0.090542	0.8135676	1467	1404	1732	1367	1490	1466
ADNP	0.9108202	-0.134762	0.5278383	4742	3553	4690	2913	4457	4457
BRSK1	1.0740741	0.1030935	0.8454574	6	13	8	11	7	11
BRSK2	3	1.5849625	0.6732949	0	0	1	0	0	3
AC119674.2	0	#NUM!	0.084153	3	7	0	0	0	0
TTC21B	1.05685	0.0797706	0.5524056	433	209	431	292	431	411
AC099520.1	1.0526316	0.0740006	0.8836156	15	12	11	9	15	16
AC108062.1	0.4210526	-1.247928	0.3990265	6	8	5	7	1	0
MAFG-AS1	1.3	0.3785116	0.6086798	3	7	10	10	10	6
SAP30L-AS1	0.9767442	-0.033947	0.9557365	31	23	32	23	20	41
AC103798.2	#DIV/0!	#DIV/0!	0.8030358	0	0	0	1	0	0
WDR1	0.8095661	-0.304779	0.0432419	8660	10392	11347	7924	8539	8147
WDR3	0.9288071	-0.106549	0.6734638	1976	1644	2139	1259	2018	2072
WDR4	0.9101487	-0.135826	0.5539665	914	1108	1072	850	969	997
AL928654.3	0.85	-0.234465	0.743752	21	19	20	15	23	13
AL928654.2	1.1428571	0.1926451	0.7290268	11	6	18	7	9	24
WDR5	0.9737708	-0.038346	0.8618526	3463	3817	4272	3552	4068	3629
WDR6	0.7736357	-0.370274	0.0113803	2733	3336	3588	2282	2717	2472
AC068050.1	6	2.5849625	0.2040017	0	0	2	0	7	5

AL928654.5	0.7184751	-0.47699	0.1246745	102	127	112	63	104	78
WDR7	0.8823529	-0.180572	0.6940263	97	79	113	53	75	127
HPN-AS1	#DIV/0!	#DIV/0!	0.3877895	0	0	0	2	2	0
TTC23L	0.9714286	-0.04182	0.9776401	48	15	42	26	42	34
ADSL	0.8390501	-0.253171	0.5428425	137	105	137	118	77	123
RPL13A	1.0724315	0.1008856	0.2370167	30647	38805	41646	42133	42023	34989
TFDP1	0.954394	-0.067343	0.8997493	5521	5707	6774	4935	6344	5902
ZMIZ1-AS1	1.3333333	0.4150375	0.8408051	0	2	1	2	2	0
TFDP2	1.1043041	0.1431375	0.0601808	3625	3788	4715	4002	4819	4572
AC068446.1	0.5333333	-0.906891	0.3804364	15	3	12	5	7	4
AC068446.2	0	#NUM!	0.080407	0	5	6	0	0	0
AP001931.1	0.5428571	-0.881356	0.3576589	8	18	9	0	13	6
RNU4-82P	0.6666667	-0.584963	0.8537226	2	0	1	0	0	2
ARPC4-TTLL3	0	#NUM!	0.824326	0	1	0	0	0	0
AL022310.1	0	#NUM!	0.5387425	3	0	0	0	0	0
ADSS	0.9772883	-0.033144	0.8391675	1859	1700	2297	1618	2149	1956
MORF4L1P1	0.820029	-0.286253	0.0611084	1532	1541	1750	1044	1509	1402
MORF4L1P3	0	#NUM!	0.824326	1	0	0	0	0	0
MORF4L1P4	0	#NUM!	0.284725	6	0	0	0	0	0
RNU4-83P	#DIV/0!	#DIV/0!	0.8030358	0	0	0	0	1	0
AC136469.2	0	#NUM!	0.5387425	3	0	0	0	0	0
AC004801.1	0	#NUM!	0.824326	0	1	0	0	0	0
AC004801.2	0.0952381	-3.392317	0.0349893	6	5	10	0	1	1
AC004801.6	0.7916667	-0.337035	0.7451322	18	6	0	4	14	1
AL445072.1	1	0	0.978278	0	0	2	0	2	0
CISD1	0.8214838	-0.283696	0.1949679	418	351	525	325	395	343
CISD2	1.1154768	0.1576605	0.1956693	807	543	737	755	836	737
CISD3	0.7427386	-0.429074	0.0701377	350	432	423	326	332	237
ZNF204P	1.2147541	0.2806643	0.2024183	242	110	258	186	311	244
RPL18A	0.9949582	-0.007292	0.6627317	15101	19238	21593	19704	19696	16250
RNU4-89P	0.4285714	-1.222392	0.5685015	0	6	1	0	3	0
CEP83-AS1	0.9176471	-0.123989	0.8986672	28	31	26	30	22	26
SEPHS1	1.0651001	0.090989	0.1884456	3209	3617	4111	3608	4113	3928
SEPHS2	0.8766644	-0.189903	0.2622056	3333	3881	4502	2954	3981	3336
THCAT158	#DIV/0!	#DIV/0!	0.8030358	0	0	0	0	0	1
AC005046.1	1.1666667	0.2223924	0.5436586	40	28	28	37	31	44
MAPK8IP2	0.7238184	-0.4663	0.0462004	273	388	418	266	261	254
MAPK8IP3	0.9640074	-0.052884	0.966773	830	837	1028	790	971	837
MAPK8IP1	0.6	-0.736966	0.4667095	21	2	12	1	10	10
AC116447.1	0.25	-2	0.5197231	3	1	0	0	1	0
AL139123.1	4	2	0.5017	0	1	0	4	0	0
AC068051.1	0	#NUM!	0.5337954	0	3	0	0	0	0
ZFAND2A	1.0014749	0.0021263	0.7963186	215	244	219	229	239	211
ZFAND2B	1.0465116	0.0655883	0.4720274	252	232	333	305	294	256
NKAP	1.1215569	0.1655028	0.1816764	547	408	715	651	690	532
AC130686.1	5	2.3219281	0.38196	1	0	0	4	0	1
NCK1-AS1	0.6548043	-0.610864	0.1075628	81	88	112	69	45	70
AL445228.2	0.6923077	-0.530515	0.6350758	11	8	7	10	4	4
AP000266.1	0.25	-2	0.5449396	0	2	2	0	0	1
AC015971.1	1.9090909	0.9328858	0.3891597	7	1	3	5	7	9
NAP1L1P1	0	#NUM!	0.824326	0	0	1	0	0	0
RNU6-1313P	#DIV/0!	#DIV/0!	0.6554062	0	0	0	0	2	0
CNEP1R1	1.1173036	0.1600213	0.1860189	660	556	668	544	800	761
NAP1L1P3	#DIV/0!	#DIV/0!	0.6411933	0	0	0	0	0	2

MIM10L2B-AS	0	#NUM!	0.6840298	0	0	2	0	0	0
AC018523.1	0	#NUM!	0.3320541	3	0	2	0	0	0
NKD1	0.744868	-0.424943	0.228806	121	105	115	84	63	107
NKD2	1.3008502	0.3794549	0.0099409	462	507	560	747	618	624
KANSL1L	0.8986842	-0.154114	0.7010752	317	156	287	209	251	223
MYEF2	#DIV/0!	#DIV/0!	0.247607	0	0	0	0	2	4
AC055753.1	0	#NUM!	0.6410557	1	1	0	0	0	0
FBXW4P1	1.0923077	0.1273793	0.6894125	14	24	27	36	12	23
RAB11A	0.9875382	-0.018092	0.7199403	7695	7385	8833	7058	8711	7846
RAB11B	1.0221495	0.0316062	0.5168132	1047	1354	1572	1441	1438	1182
AC004552.1	1.7083333	0.7725895	0.3124279	7	13	4	19	9	13
AC023866.2	0	#NUM!	0.6840298	0	0	2	0	0	0
AC006128.1	1.7222222	0.7842713	0.3660704	1	2	15	11	15	5
RPS27AP16	1.1572577	0.2107102	0.0251536	3564	3853	4646	4581	5143	4236
RPS27AP15	#DIV/0!	#DIV/0!	0.6099294	0	0	0	2	0	0
RPS27AP12	1	0	0.9878959	1	2	0	1	2	0
RPS27AP13	0	#NUM!	0.824326	0	0	1	0	0	0
RPS27AP11	1.5	0.5849625	0.6797448	3	1	2	2	2	5
AC019257.2	#DIV/0!	#DIV/0!	0.5070017	0	0	0	3	0	0
RPS27AP18	1	0	0.9890559	0	1	0	0	0	1
AC019257.1	#DIV/0!	#DIV/0!	0.8030358	0	0	0	0	1	0
AC087071.1	7	2.8073549	0.3235909	0	1	0	0	0	7
AC008429.4	0	#NUM!	0.5622741	0	1	2	0	0	0
AC087071.2	0.7462687	-0.422233	0.5205583	32	19	16	3	28	19
AC008429.1	0.6349206	-0.655352	0.380062	27	11	25	15	9	16
AC008429.2	1.1428571	0.1926451	0.8427275	1	0	6	2	4	2
AC093012.1	1.4	0.4854268	0.7105728	3	0	7	0	3	11
HRASLS2	2.7142857	1.4405726	0.2489576	1	1	5	2	11	6
AC131274.4	0	#NUM!	0.6385096	0	2	0	0	0	0
AC131274.3	0	#NUM!	0.1787666	0	8	0	0	0	0
HRASLS5	#DIV/0!	#DIV/0!	0.8030358	0	0	0	0	0	1
SCUBE2	1.1052632	0.1443899	0.9146032	5	8	6	0	12	9
SCUBE3	0.62	-0.68966	0.3464102	16	25	9	5	12	14
SCUBE1	0	#NUM!	0.1651379	1	3	3	0	0	0
AL162419.1	1	0	0.9544849	0	0	3	0	0	3
NKG7	0	#NUM!	0.0391092	7	2	3	0	0	0
CRLS1	0.8816234	-0.181765	0.3323867	816	885	960	676	852	818
AC008669.1	1.3333333	0.4150375	0.3345846	22	18	38	21	36	47
RNU6-748P	0	#NUM!	0.4217812	0	0	5	0	0	0
RN7SL653P	0	#NUM!	0.824326	0	1	0	0	0	0
SOWAHB	1.3574297	0.4408775	0.0270596	150	179	169	195	277	204
SOWAHC	0.9590112	-0.06038	0.8899312	1509	1572	1652	1163	1673	1703
SOWAHD	0.6415094	-0.640458	0.1503278	60	77	128	75	48	47
MCF2L	1.1275168	0.1731489	0.5227138	43	36	70	44	71	53
AL122023.1	0.9642857	-0.052467	0.9961517	10	8	10	9	11	7
RNU6-743P	#DIV/0!	#DIV/0!	0.8030358	0	0	0	0	1	0
MYEOV	1.2818533	0.3582311	0.218113	102	84	73	82	152	98
AC093158.1	0	#NUM!	0.6840298	0	0	2	0	0	0
SOWAHA	3	1.5849625	0.6253252	0	0	1	3	0	0
MIR2117HG	1	0	0.9838634	1	0	1	0	0	2
ABRAXAS1	1.4074074	0.49304	0.0492331	115	77	105	102	149	167
ABRAXAS2	1.3045761	0.3835812	0.0086904	459	327	547	511	657	571
RN7SL650P	0	#NUM!	0.6385096	0	2	0	0	0	0
AC007685.1	0	#NUM!	0.6840298	0	0	2	0	0	0

WEE1	1.047091	0.0663869	0.3006888	7074	6782	8059	6210	8452	8285
WEE2	#DIV/0!	#DIV/0!	0.8030358	0	0	0	0	0	1
PGAP1	0.9893617	-0.01543	0.9255534	723	491	666	380	673	807
PGAP2	0.8406072	-0.250496	0.2137844	682	892	1061	632	845	738
PGAP3	0.845987	-0.241293	0.4092324	140	171	150	103	144	143
EQTN	1.2857143	0.3625701	0.8030855	2	0	5	0	4	5
SNRPEP2	1.8888889	0.9175378	0.3910119	2	3	4	7	4	6
AC092819.1	#DIV/0!	#DIV/0!	0.6099294	0	0	0	2	0	0
SNRPEP4	1.1080051	0.1479645	0.3165014	246	248	293	305	347	220
AC243756.2	1.25	0.3219281	0.7970153	0	0	4	4	1	0
SNRPEP5	#DIV/0!	#DIV/0!	0.8030358	0	0	0	1	0	0
SNRPEP6	#DIV/0!	#DIV/0!	0.8030358	0	0	0	0	0	1
AC092423.1	#DIV/0!	#DIV/0!	0.288876	0	0	0	3	2	0
HDHD5	0.8553361	-0.225437	0.2216617	1262	1547	1698	1221	1336	1298
HDHD2	1.16	0.2141248	0.7541669	11	4	10	8	14	7
FBXO36-IT1	0.6666667	-0.584963	0.87348	1	0	2	0	0	2
HDHD3	0.9788283	-0.030872	0.7961959	962	1160	1326	1148	1125	1102
CNBD1	0	#NUM!	0.5075476	0	0	4	0	0	0
CNBD2	1.0235294	0.0335526	0.7850973	17	31	37	51	22	14
RNU6-762P	1.3333333	0.4150375	0.6263177	7	1	7	9	6	5
NYAP1	1.0425532	0.060121	0.7945461	20	32	42	26	40	32
FABP5P2	0.9473684	-0.078003	0.993227	3	7	9	7	7	4
FABP5P3	#DIV/0!	#DIV/0!	0.0411996	0	0	0	4	4	3
NCAPG	1.2875563	0.3646356	0.0166039	3075	1707	3206	2700	3955	3630
NYAP2	0.3333333	-1.584963	0.6622061	0	3	0	0	0	1
NCAPH	0.8744593	-0.193537	0.2000104	3174	3237	3761	2497	3319	3079
FABP5P7	1.2641243	0.3381383	0.0628508	239	243	226	329	313	253
AL122020.1	1.5454545	0.6280312	0.5691358	2	6	3	6	8	3
MYH7B	1.1142857	0.1561192	0.6980115	6	4	25	21	13	5
TTC4P1	0	#NUM!	0.2891624	2	1	2	0	0	0
LRRC36	1.4166667	0.5025003	0.6505498	5	0	7	2	4	11
LRRC34	#DIV/0!	#DIV/0!	0.8030358	0	0	0	0	1	0
EEF1GP1	3	1.5849625	0.530131	0	2	0	1	0	5
LRRC39	1.3809524	0.4656636	0.5479089	9	0	12	17	4	8
PTH1R	12.333333	3.6244909	0.0031422	2	0	1	20	9	8
LRRC32	1.1282051	0.1740294	0.7381366	12	17	10	24	14	6
METTL11B	0.7058824	-0.5025	0.7953215	0	4	13	5	6	1
LRRC31	0.5	-1	0.8652929	0	0	2	0	0	1
NKRF	1.0059253	0.0085231	0.6661531	709	595	890	579	823	805
LRRC47	0.9923433	-0.011089	0.6842716	1689	1849	2078	1836	1931	1806
LRRC45	1.1898667	0.2507999	0.0957609	559	647	669	914	708	609
LRRC46	0.8970588	-0.156726	0.883275	15	27	26	25	25	11
PGAM1	1.2413006	0.3118525	0.0020203	10159	12164	13168	14264	15641	14150
LRRC40	1.2329545	0.3021196	0.0884944	820	479	813	585	1115	904
PTH2R	1.0628141	0.0878892	0.5373402	122	124	152	126	162	135
LRRC43	#DIV/0!	#DIV/0!	0.5230806	0	0	0	1	2	0
PTGR1	9.6666667	3.2730185	0.0146011	0	2	1	9	16	4
PTGR2	0.8567208	-0.223103	0.4118303	253	186	238	169	219	192
LRRC41	0.8417962	-0.248457	0.1707014	865	987	1043	739	813	885
LRRC42	0.9660675	-0.049804	0.9211477	1416	1724	1811	1651	1644	1488
VAMP8	0.9954817	-0.006533	0.6660563	3035	3787	4244	3868	3900	3248
AC114730.1	#DIV/0!	#DIV/0!	0.6554062	0	0	0	0	2	0
AC114730.3	0.9285714	-0.106915	0.9516426	2	5	7	1	7	5
VAMP7	1.0770516	0.1070874	0.2535204	1923	1390	2073	1577	2113	2111

PGAM4	0.8589744	-0.219313	0.8117098	19	24	35	25	16	26
VAMP1	0.7906977	-0.338802	0.3412109	108	76	117	49	105	84
PGAM5	0.8087054	-0.306314	0.086	1453	1876	2024	1422	1503	1404
VAMP4	0.8978583	-0.15544	0.6574644	240	147	220	145	251	149
VAMP5	0.527027	-0.924051	0.0494643	56	43	49	28	20	30
VAMP2	0.771345	-0.374552	0.0373423	508	532	670	399	487	433
VAMP3	1.2142428	0.280057	0.0026644	2574	2526	3227	2967	3855	3289
LRRC14	0.8470948	-0.239405	0.2203887	490	558	587	405	512	468
LRRC15	3	1.5849625	0.0228537	5	5	9	17	22	18
LRRC19	4	2	0.2514327	3	0	0	7	1	4
LRRC17	#DIV/0!	#DIV/0!	0.5082165	0	0	0	1	0	2
PTGS2	1.0206186	0.0294438	0.8873115	32	22	43	13	37	49
PTGS1	0	#NUM!	0.5436524	2	0	1	0	0	0
AC022150.4	0.9015152	-0.149576	0.7183648	205	109	214	98	184	194
ANO10	0.9944183	-0.008075	0.6877099	1783	1817	2133	1800	2161	1740
AC022150.2	1.2447552	0.3158621	0.2893444	52	36	55	56	62	60
AC022150.3	0.4333333	-1.206451	0.2431061	13	5	12	1	8	4
NKTR	1.0422156	0.0596538	0.7798056	1459	657	1603	798	1499	1579
LRRC25	#DIV/0!	#DIV/0!	0.8030358	0	0	0	0	1	0
MRPL35P2	2	1	0.4785477	2	2	2	2	7	3
LRRC23	1.1406926	0.1899101	0.3046177	146	164	152	204	170	153
LRRC29	0.893617	-0.162271	0.8905364	5	27	15	18	15	9
LRRC27	1.4814815	0.5670406	0.1008868	36	30	42	50	52	58
AC105129.3	#DIV/0!	#DIV/0!	0.8030358	0	0	0	0	0	1
LRRC28	0.7891566	-0.341616	0.1356029	383	337	442	325	314	278
LRRC20	1.5393939	0.6223625	0.0774747	79	45	41	70	108	76
BEND3P3	0.5	-1	0.6927702	1	0	5	0	2	1
AL591475.1	1.5	0.5849625	0.6356708	3	1	4	2	2	8
BEND3P1	1.1102362	0.1508667	0.607899	75	96	83	50	99	133
EEF1GP5	0.25	-2	0.5688068	1	0	3	0	0	1
AC003080.1	#DIV/0!	#DIV/0!	0.2327526	0	0	0	0	0	7
LRRC73	#DIV/0!	#DIV/0!	0.8030358	0	0	0	0	1	0
CRAMP1	0.952241	-0.070601	0.969799	446	389	526	415	456	425
LRRC70	0	#NUM!	0.824326	1	0	0	0	0	0
LRRC71	1.6	0.6780719	0.4286677	2	2	11	17	2	5
AL022311.1	1.1140351	0.1557947	0.6818545	32	41	41	26	66	35
LCNL1	1.0222222	0.0317089	0.8225787	7	7	31	15	16	15
SH3GLB1	1.1617965	0.2163574	0.0226993	1863	1667	2014	1847	2315	2279
MORF4L2P1	0	#NUM!	0.824326	1	0	0	0	0	0
SH3GLB2	1.3572385	0.4406742	0.0005574	1445	1679	2105	2556	2424	2117
ANAPC7	0.9453953	-0.08101	0.9249981	1299	983	1399	1143	1160	1177
N4BP2L2-IT2	0.96	-0.058894	0.9175849	39	17	44	45	22	29
AL512363.1	0.6612903	-0.596644	0.3622946	19	21	22	11	18	12
MYHAS	0.5	-1	0.81753	2	0	0	0	0	1
AC090360.1	1.6666667	0.7369656	0.8187196	3	0	0	0	5	0
ANAPC4	1.125	0.169925	0.1734695	954	638	1184	886	1266	971
ANAPC5	0.9152915	-0.127697	0.5130217	3372	3449	4087	2999	3744	3241
AC021072.1	#DIV/0!	#DIV/0!	0.6554062	0	0	0	0	2	0
ANAPC1	0.868326	-0.203691	0.3173022	2715	2411	2628	1479	2386	2868
ANAPC2	1.1112143	0.152137	0.1435333	1445	1904	2037	2035	2135	1815
LRRC58	0.9543839	-0.067358	0.8805059	2120	1137	1807	1095	1944	1794
LRRC59	0.7719209	-0.373475	0.0092985	4646	5612	5469	3694	4405	4041
LRRC56	1.2987013	0.3770696	0.3869408	12	28	37	27	43	30
LRRC57	0.8835413	-0.178631	0.4048219	549	564	570	461	537	489

PLA2G4E-AS1	0.7142857	-0.485427	0.8384498	4	0	3	1	0	4
AC022306.3	0.7777778	-0.36257	0.9158723	2	0	7	2	0	5
AL132996.1	2.75	1.4594316	0.3721798	0	0	4	8	0	3
AL354707.3	0.8829787	-0.179549	0.8401625	15	38	41	30	33	20
AL354707.1	1.077193	0.1072767	0.5392719	74	93	118	120	112	75
AL354707.2	1.25	0.3219281	0.877819	8	0	0	3	7	0
UBE2V1P1	0.2222222	-2.169925	0.3614591	0	9	0	0	0	2
VDAC2P2	0.5	-1	0.8652929	0	0	2	0	1	0
UBE2V1P2	0.7492625	-0.416457	0.1919806	96	110	133	70	108	76
VDAC2P3	#DIV/0!	#DIV/0!	0.6099294	0	0	0	2	0	0
CRNDE	0.7792208	-0.359896	0.4424705	36	58	60	33	41	46
AC022306.1	2	1	0.2756319	7	3	5	5	13	12
UBE2V1P9	0	#NUM!	0.6385096	0	2	0	0	0	0
AC022306.2	1.6071429	0.6844982	0.3174693	8	9	11	11	22	12
LRRC4B	0	#NUM!	0.5643268	1	0	2	0	0	0
UBE2V1P4	#DIV/0!	#DIV/0!	0.6411933	0	0	0	0	0	2
LRRC69	1.0871795	0.1205901	0.5470414	48	62	85	75	61	76
LRRC61	0.9005011	-0.1512	0.6976863	423	515	459	512	394	352
AC022296.3	1	0	0.9890559	0	0	1	0	1	0
LRRC66	3.2	1.6780719	0.291054	0	5	0	9	6	1
LRRC63	2.1234568	1.0864148	0.0070933	26	18	37	39	61	72
AC010205.1	#DIV/0!	#DIV/0!	0.5070017	0	0	0	3	0	0
ANO7L1	1.2272727	0.2954559	0.6897349	7	5	10	5	9	13
ERC1	0.8262003	-0.275437	0.1928541	1454	1653	1496	818	1298	1687
ERC2	0.7157895	-0.482393	0.2300254	69	52	69	39	46	51
AC044802.1	1.1666667	0.2223924	0.8265817	3	1	2	6	0	1
AC096775.1	0	#NUM!	0.824326	0	1	0	0	0	0
AL356124.1	0.8333333	-0.263034	0.9312158	3	0	3	2	3	0
AL356124.2	0	#NUM!	0.5387425	3	0	0	0	0	0
VEZF1	0.8834655	-0.178754	0.3847683	1218	908	1152	746	1074	1076
AC009506.1	0.6818182	-0.552541	0.5318366	9	11	24	9	11	10
ERAS	4	2	0.5054618	1	0	0	1	0	3
AC008761.3	8	3	0.2574152	0	0	1	0	7	1
USH1C	0.7961165	-0.328949	0.4712902	63	52	91	57	59	48
BEND3	0.7160256	-0.481917	0.0017784	921	1023	1176	649	772	813
LRRC8C	2.2384106	1.1624747	0.0009965	69	28	54	70	145	123
LRRC8D	0.9420485	-0.086127	0.8380095	2352	1726	2600	1818	2439	2034
LRRC8A	0.9104101	-0.135412	0.5008917	1938	2226	2176	1667	2002	2103
BEND2	#DIV/0!	#DIV/0!	0.8030358	0	0	0	0	1	0
LRRC8B	1.2162431	0.2824317	0.0266282	703	558	746	651	899	891
BEND7	1.3211538	0.4017985	0.0535851	176	143	201	158	235	294
FCGR1A	0.8684211	-0.203533	0.8381477	14	7	17	4	13	16
FCGR1B	2	1	0.486394	1	1	3	3	5	2
BEND5	2	1	0.8324799	1	0	0	0	2	0
LRRC8E	0.6363636	-0.652077	0.685264	1	6	4	2	1	4
BEND6	1.3577982	0.441269	0.2190914	40	21	48	42	55	51
ERAP2	0.8103448	-0.303392	0.5996827	87	21	66	44	60	37
MBOAT7	0.9078697	-0.139443	0.5198358	3130	3878	4009	3167	3475	3360
ERAP1	1.0233516	0.033302	0.6677981	1051	919	942	747	1067	1166
MBOAT1	0.8966725	-0.157347	0.6206401	172	185	214	107	202	203
MBOAT2	1.3751651	0.4596049	0.0010441	830	681	760	820	1159	1144
RPL23A	1.0347313	0.0492562	0.5334478	431	535	560	553	589	437
MBOAT4	5.5	2.4594316	0.2069313	0	2	0	2	3	6
USH2A	1.6666667	0.7369656	0.5809085	3	5	1	15	0	0

FCGR2A	#DIV/0!	#DIV/0!	0.3109758	0	0	0	0	6	0
USH1G	1.7777778	0.830075	0.4977558	4	4	1	4	6	6
FCGR2B	0	#NUM!	0.5387425	3	0	0	0	0	0
RNU4-52P	0.1578947	-2.662965	0.0816782	3	9	7	1	2	0
ERFE	1.0697674	0.0972972	0.8190232	10	15	18	10	12	24
AL583808.1	5	2.3219281	0.1599625	0	0	3	3	1	11
AC079035.1	0.3333333	-1.584963	0.7290749	0	0	3	0	0	1
ZNF790-AS1	1.0522876	0.073529	0.683698	39	42	72	48	57	56
RNU4-53P	#DIV/0!	#DIV/0!	0.6411933	0	0	0	0	0	2
EREG	#DIV/0!	#DIV/0!	0.8030358	0	0	0	0	1	0
DAZAP2P1	0.6	-0.736966	0.7517529	0	3	2	0	1	2
AC021321.1	0.8181818	-0.289507	0.761674	7	17	20	12	15	9
PGBD1	1.0989247	0.1360926	0.3862295	131	120	214	174	198	139
PGBD2	0.8921283	-0.164677	0.6167272	247	188	251	196	205	211
PGBD4	0.9647059	-0.051839	0.9749875	105	104	131	98	115	115
PGBD5	0.3380282	-1.564785	0.0647866	22	8	41	10	12	2
TSPAN8	0	#NUM!	0.824326	1	0	0	0	0	0
ERI1	0.9474768	-0.077837	0.8483037	996	805	1112	722	1084	954
TSPAN9	0.7466025	-0.421588	0.0029215	2088	2435	2541	1562	1865	1847
ERI2	0.9082745	-0.1388	0.6408144	637	375	547	265	572	579
TSPAN6	1.0055786	0.0080259	0.5953447	2171	2155	2665	2022	2736	2272
ERI3	0.9204162	-0.119642	0.7128229	1068	1241	1247	1168	1142	963
TSPAN7	4.247619	2.0866544	1.50E-09	44	26	35	188	162	96
TSPAN4	1.0407873	0.0576752	0.4183008	1279	1464	1474	1570	1389	1430
TSPAN5	1.1918699	0.2532268	0.110321	177	199	239	240	262	231
TSPAN2	0.5714286	-0.807355	0.7939197	3	0	4	4	0	0
TSPAN3	0.8919649	-0.164941	0.3580488	1384	1478	1581	1125	1551	1287
TSPAN1	0.8931298	-0.163058	0.7537818	34	61	36	33	46	38
ERAL1	1.0118966	0.0170619	0.5464656	1250	1473	1648	1460	1518	1445
RPL27A	1.049665	0.0699289	0.4492694	19292	24597	22073	26058	25459	17721
LYNX1	0.3333333	-1.584963	0.4845224	3	0	6	2	1	0
AJAP1	2.4	1.2630344	0.4083846	3	0	2	4	7	1
PDZRN3	4.8	2.2630344	0.1482138	1	4	0	0	15	9
TMPRSS9	0.5652174	-0.823122	0.4019972	16	2	28	4	15	7
TMPRSS7	0.9473684	-0.078003	0.9934426	2	7	10	7	10	1
TMPRSS5	0.0769231	-3.70044	0.0770769	8	1	4	1	0	0
TMPRSS6	0.2051282	-2.285402	0.0014507	31	18	29	4	7	5
TMPRSS3	1.0809249	0.1122662	0.6812794	44	70	59	82	79	26
TMPRSS4	1.1666667	0.2223924	0.761292	3	0	9	7	1	6
TMPRSS2	0.9028698	-0.14741	0.7525058	124	136	193	133	152	124
RXRB	0.9467425	-0.078956	0.9028437	577	635	722	581	619	631
RXRA	1.1537507	0.2063315	0.0227495	8836	10515	11257	10840	12590	11884
AC234783.1	1	0	0.963496	0	3	0	0	3	0
RFTN2	0.9176471	-0.123989	0.8659106	33	18	34	10	34	34
TSNAX	1.0365154	0.0517415	0.5078109	701	550	666	572	723	692
RFTN1	0.8571429	-0.222392	0.7538337	25	17	35	11	41	14
AC012498.2	#DIV/0!	#DIV/0!	0.6099294	0	0	0	2	0	0
C3orf67-AS1	0	#NUM!	0.6436235	2	0	0	0	0	0
NLE1	0.9260992	-0.110761	0.7275202	1231	1526	1519	1341	1360	1259
PLGLB1	0.4285714	-1.222392	0.1685936	20	6	16	6	6	6
PLGLB2	0	#NUM!	0.1909345	0	6	1	0	0	0
RNU6-759P	0.5	-1	0.6951675	3	0	3	0	0	3
AP003500.1	0.8	-0.321928	0.8886991	5	0	0	0	0	4
DIMT1	0.9433266	-0.084171	0.8523606	1794	1556	2067	1539	1740	1831

SSU72	1.0859591	0.1189697	0.1335285	4108	4449	5217	4967	5406	4585
ERN1	0.9983888	-0.002326	0.7450233	557	583	722	482	660	717
PLA2G15	1.2443712	0.3154169	0.0134374	540	596	685	759	833	674
PLA2G16	3	1.5849625	0.0404275	8	6	3	24	13	14
AC092329.2	2	1	0.8324799	1	0	0	0	2	0
PLA2G10	2.2222222	1.1520031	0.3746581	7	2	0	12	7	1
AC092329.4	1.0237154	0.0338147	0.8828492	79	110	64	60	95	104
CKMT2-AS1	1.0295139	0.0419633	0.6244304	203	119	254	189	215	189
KHSRPP1	0	#NUM!	0.6410557	1	1	0	0	0	0
Z68871.1	0.9137931	-0.130061	0.8287496	33	16	9	1	23	29
MCHR1	0.2142857	-2.222392	0.1734526	6	3	5	0	2	1
DEAF1	1.0209003	0.029842	0.5920301	562	666	638	670	646	589
NPHP1	1.5416667	0.6244909	0.0618112	44	34	42	62	65	58
CAMTA1	1.0879752	0.1216457	0.2303097	624	763	875	876	808	777
NPHP3	0.8811189	-0.182591	0.6720607	165	100	164	127	160	91
CAMTA2	0.8398876	-0.251732	0.1886996	537	586	657	390	561	544
NPHP4	0.8333333	-0.263034	0.3709975	223	248	309	215	184	251
BEAN1-AS1	1	0	0.9533169	11	0	5	5	0	11
AP000759.1	0.9042553	-0.145198	0.9303428	14	26	54	23	26	36
AFDN	0.9366331	-0.094444	0.716645	7082	6092	7373	4622	6848	7775
RNU6-1327P	#DIV/0!	#DIV/0!	0.8030358	0	0	0	0	1	0
AC016954.1	#DIV/0!	#DIV/0!	0.8030358	0	0	0	0	0	1
AC093157.1	0.516129	-0.954196	0.0502253	47	41	36	18	27	19
AC093157.2	0.2222222	-2.169925	0.0791354	12	9	6	2	0	4
AC087468.1	0	#NUM!	0.824326	0	1	0	0	0	0
OR6L2P	0	#NUM!	0.6840298	0	0	2	0	0	0
AC109779.1	#DIV/0!	#DIV/0!	0.6411933	0	0	0	0	0	2
AFF3	1.5151515	0.5994621	0.4706277	11	18	4	14	30	6
AFF4	1.0112704	0.0161688	0.7921515	4958	3402	4683	2645	4785	5760
AFF1	1.367268	0.4512961	0.0121883	767	758	803	652	1143	1388
AFF2	0.9111111	-0.134301	0.8946372	11	12	22	4	21	16
AC083880.1	1.75	0.8073549	0.389582	9	0	7	17	7	4
RNF212B	1.2424242	0.3131579	0.5672565	7	10	16	18	8	15
AL031729.1	1.3387097	0.4208431	0.4228417	14	32	16	24	34	25
AL139120.1	#DIV/0!	#DIV/0!	0.8030358	0	0	0	0	1	0
RNU6-755P	#DIV/0!	#DIV/0!	0.6554062	0	0	0	0	2	0
AL035446.1	1.0076046	0.0109296	0.6887844	225	254	310	265	289	241
TRBV20-1	0.65	-0.621488	0.6262974	5	14	21	13	0	13
PLA2G4F	1	0	0.8624362	142	142	139	126	176	121
NCBP1	1.0725578	0.1010553	0.2151685	4081	3555	4699	3464	4997	4769
PLA2G4D	0	#NUM!	0.004004	5	16	1	0	0	0
NCBP2	0.9280192	-0.107773	0.6440157	3121	2631	3431	2435	3242	2845
PLA2G4E	0.8235294	-0.280108	0.867601	5	2	10	1	3	10
PLA2G4B	2.75	1.4594316	0.4564358	1	3	0	2	9	0
PLA2G4C	0.5	-1	0.7117835	4	0	4	4	0	0
PLA2G4A	0.5982906	-0.741082	0.0050637	230	168	187	85	147	118
SSTR2	1.1304348	0.1768778	0.7817242	11	4	8	9	8	9
SSTR5	#DIV/0!	#DIV/0!	0.8030358	0	0	0	0	0	1
RN7SL663P	0.2	-2.321928	0.4184479	1	4	0	0	1	0
NCBP3	0.902391	-0.148175	0.4889115	2151	1838	2619	1494	2239	2230
KF455155.1	0	#NUM!	0.5387425	3	0	0	0	0	0
CRMP1	0	#NUM!	0.6436235	2	0	0	0	0	0
AC130689.1	0.9340659	-0.098404	0.9165553	61	58	63	53	43	74
PTHLH	0.3888889	-1.36257	0.2942915	10	3	5	1	2	4

AC087072.2	0	#NUM!	0.6840298	0	0	2	0	0	0
PLA2G2F	2.5980392	1.3774232	5.16E-05	29	23	50	90	90	85
AC099329.2	0.7284072	-0.457183	0.0030395	965	905	1175	700	773	745
NPHS1	1.3333333	0.4150375	0.7470981	1	2	3	3	3	2
S100Z	1.3333333	0.4150375	0.6724431	6	8	7	3	20	5
S100P	0.9921783	-0.011329	0.7156061	715	840	1002	870	927	740
AC099329.1	0.3529412	-1.5025	0.2903912	13	3	1	0	4	2
RXFP4	0	#NUM!	0.5781912	0	0	3	0	0	0
BACH1	1.1616207	0.216139	0.172955	862	594	790	592	954	1063
RXFP1	0	#NUM!	0.5781912	0	0	3	0	0	0
BACH2	0.86875	-0.202987	0.7082999	61	41	58	13	48	78
AC010300.1	2.1428571	1.0995357	0.4223101	4	0	3	14	0	1
DIP2A	0.9563431	-0.0644	0.9065077	1221	1269	1404	998	1292	1434
RN7SL684P	0	#NUM!	0.5075476	0	0	4	0	0	0
DIP2B	0.9122388	-0.132517	0.5733769	2439	2016	2245	1287	2193	2632
DIP2C	0.9994599	-0.000779	0.6726735	1126	1211	1366	1071	1300	1330
AP001347.1	0.5	-1	0.2721622	7	22	7	4	5	9
DYNC1LI1	0.8438874	-0.244878	0.1129193	1406	1470	1672	1091	1400	1347
AL133342.1	1	0	0.9946879	6	0	0	2	3	1
DYNC1LI2	0.8981892	-0.154909	0.4803928	4686	3810	4813	2617	4331	5006
RN7SL683P	#DIV/0!	#DIV/0!	0.6099294	0	0	0	2	0	0
RN7SKP5	#DIV/0!	#DIV/0!	0.8030358	0	0	0	0	1	0
RNU6-774P	#DIV/0!	#DIV/0!	0.5371397	0	0	0	0	0	3
AC073352.1	0.2647059	-1.917538	0.0990268	8	11	15	6	0	3
SYCP2L	1.019802	0.028289	0.7375006	120	72	111	110	100	99
SNRPFP1	2	1	0.8319548	0	1	0	0	2	0
SNRPFP2	0	#NUM!	0.2117164	3	0	4	0	0	0
ERP29P1	0.1666667	-2.584963	0.1803314	2	3	7	1	1	0
AC092818.1	#DIV/0!	#DIV/0!	0.8030358	0	0	0	1	0	0
SNRPFP4	2	1	0.5241071	0	0	4	5	1	2
AC073508.3	0.5744681	-0.799701	0.1892168	34	28	32	26	13	15
VWA8-AS1	0.1111111	-3.169925	0.1628093	3	3	3	0	0	1
AC003965.1	0.7391304	-0.436099	0.70984	4	10	9	7	6	4
AC003965.2	0.3333333	-1.584963	0.5460964	1	0	5	0	1	1
KDM5C-IT1	0	#NUM!	0.4217812	0	0	5	0	0	0
AP001107.1	0.1851852	-2.432959	0.0458515	5	11	11	1	2	2
AP001107.6	0.1764706	-2.5025	0.1385639	4	4	9	0	0	3
AP001107.7	#DIV/0!	#DIV/0!	0.4501902	0	0	0	0	0	4
AP001107.4	1.2631579	0.337035	0.7228314	10	2	7	3	13	8
C17orf58	1.0318323	0.0452085	0.564476	444	399	445	403	515	411
C17orf53	0.928535	-0.106972	0.7523681	597	656	706	597	705	517
C17orf51	0.3134328	-1.673772	0.0003619	41	99	61	20	21	22
AC073592.5	#DIV/0!	#DIV/0!	0.8030358	0	0	0	0	0	1
AC073592.3	#DIV/0!	#DIV/0!	0.8030358	0	0	0	0	0	1
AC073592.7	0	#NUM!	0.824326	1	0	0	0	0	0
TTC3P1	0.8047619	-0.313366	0.5626511	68	34	108	39	57	73
AC073592.1	9	3.169925	0.1776957	1	0	0	4	5	0
RPL19P16	0.5	-1	0.655154	1	3	2	0	0	3
BACE1	0.744186	-0.426265	0.3128295	78	56	81	56	43	61
BACE2	0.7860732	-0.347264	0.0425195	853	982	951	693	779	718
AP001107.8	#DIV/0!	#DIV/0!	0.6554062	0	0	0	0	2	0
AP001107.9	1.2321429	0.3011695	0.5613604	16	20	20	19	36	14
C17orf49	0.2666667	-1.906891	0.2279256	2	5	8	0	2	2
RN7SL4P	1	0	0.9805667	1	5	1	0	2	5

PHLPP2	0.6966292	-0.521537	0.0636194	203	149	271	120	138	176
PHLPP1	1.0025654	0.0036964	0.7608021	625	639	685	505	670	779
TTC41P	0.761194	-0.393664	0.5446683	22	21	24	5	25	21
RN7SL5P	#DIV/0!	#DIV/0!	0.8030358	0	0	0	0	1	0
C17orf75	0.8605201	-0.216719	0.3324227	519	528	645	484	479	493
OR2AG1	4	2	0.5810589	0	0	1	0	4	0
C17orf78	1.5	0.5849625	0.741652	7	0	1	0	4	8
OR2AG2	0.6315789	-0.662965	0.4331565	18	11	9	2	9	13
AC062031.1	#DIV/0!	#DIV/0!	0.8030358	0	0	0	0	0	1
RYBP	1.2842194	0.3608917	0.0052021	2174	1625	2126	1938	2829	2842
C17orf67	1.0889831	0.1229815	0.5997765	55	105	76	85	82	90
C17orf62	0.804328	-0.314144	0.1083565	1338	1395	1657	1289	1211	1031
C17orf97	0.6428571	-0.63743	0.4847824	7	11	24	7	9	11
AL359397.1	#DIV/0!	#DIV/0!	0.5070017	0	0	0	3	0	0
ZBED3-AS1	0.75	-0.415037	0.8694662	0	2	2	0	3	0
RN7SL8P	0.5714286	-0.807355	0.7520899	0	3	4	3	1	0
C17orf82	2.1666667	1.1154772	0.453708	0	2	4	13	0	0
AC008760.1	1.3076923	0.3870231	0.5644995	7	13	19	6	29	16
AC008760.2	0.6923077	-0.530515	0.7266618	0	7	6	1	6	2
C17orf80	1.0185629	0.026535	0.7104584	641	422	607	407	649	645
OR2A9P	0.5890411	-0.76356	0.228748	32	19	22	13	12	18
RASAL1	0.384273	-1.379796	5.36E-06	227	232	215	117	67	75
RASAL2	1.0887223	0.122636	0.3590433	1187	889	1125	799	1231	1455
RASAL3	0.3333333	-1.584963	0.7307796	0	0	3	0	1	0
Z97180.1	0.9051095	-0.143836	0.8286742	41	53	43	44	36	44
AL354696.1	1.3333333	0.4150375	0.8401502	1	0	2	0	1	3
WFS1	0.8908251	-0.166786	0.3941464	2243	2641	2691	2187	2419	2142
SCCPDH	0.5	-1	0.7988788	1	1	0	0	0	1
ESAM	1.3710021	0.4552308	0.0165813	128	156	185	216	222	205
AL354696.2	0.6610169	-0.597241	0.4416411	13	14	32	4	18	17
RNPEPL1	1.1220743	0.1661683	0.1470743	629	735	815	840	901	704
Y_RNA	0.7574124	-0.400849	0.0914033	364	291	458	191	311	341
Y_RNA	0.7574124	-0.400849	0.0914033	364	291	458	191	311	341
Y_RNA	0.7574124	-0.400849	0.0914033	364	291	458	191	311	341
Y_RNA	0.7574124	-0.400849	0.0914033	364	291	458	191	311	341
Y_RNA	0.7574124	-0.400849	0.0914033	364	291	458	191	311	341
Y_RNA	0.7574124	-0.400849	0.0914033	364	291	458	191	311	341
Y_RNA	0.7574124	-0.400849	0.0914033	364	291	458	191	311	341
Y_RNA	0.7574124	-0.400849	0.0914033	364	291	458	191	311	341
Y_RNA	0.7574124	-0.400849	0.0914033	364	291	458	191	311	341
Y_RNA	0.7574124	-0.400849	0.0914033	364	291	458	191	311	341
Y_RNA	0.7574124	-0.400849	0.0914033	364	291	458	191	311	341
Y_RNA	0.7574124	-0.400849	0.0914033	364	291	458	191	311	341
Y_RNA	0.7574124	-0.400849	0.0914033	364	291	458	191	311	341
Y_RNA	0.7574124	-0.400849	0.0914033	364	291	458	191	311	341
Y_RNA	0.7574124	-0.400849	0.0914033	364	291	458	191	311	341
Y_RNA	0.7574124	-0.400849	0.0914033	364	291	458	191	311	341
Y_RNA	0.7574124	-0.400849	0.0914033	364	291	458	191	311	341
Y_RNA	0.7574124	-0.400849	0.0914033	364	291	458	191	311	341
Y_RNA	0.7574124	-0.400849	0.0914033	364	291	458	191	311	341
Y_RNA	0.7574124	-0.400849	0.0914033	364	291	458	191	311	341
Y_RNA	0.7574124	-0.400849	0.0914033	364	291	458	191	311	341
Y_RNA	0.7574124	-0.400849	0.0914033	364	291	458	191	311	341
Y_RNA	0.7574124	-0.400849	0.0914033	364	291	458	191	311	341

Y_RNA	0.7574124	-0.400849	0.0914033	364	291	458	191	311	341
Y_RNA	0.7574124	-0.400849	0.0914033	364	291	458	191	311	341
Y_RNA	0.7574124	-0.400849	0.0914033	364	291	458	191	311	341
Y_RNA	0.7574124	-0.400849	0.0914033	364	291	458	191	311	341
Y_RNA	0.7574124	-0.400849	0.0914033	364	291	458	191	311	341
Y_RNA	0.7574124	-0.400849	0.0914033	364	291	458	191	311	341
Y_RNA	0.7574124	-0.400849	0.0914033	364	291	458	191	311	341
Y_RNA	0.7574124	-0.400849	0.0914033	364	291	458	191	311	341
Y_RNA	0.7574124	-0.400849	0.0914033	364	291	458	191	311	341
Y_RNA	0.7574124	-0.400849	0.0914033	364	291	458	191	311	341
Y_RNA	0.7574124	-0.400849	0.0914033	364	291	458	191	311	341
Y_RNA	0.7574124	-0.400849	0.0914033	364	291	458	191	311	341
Y_RNA	0.7574124	-0.400849	0.0914033	364	291	458	191	311	341
Y_RNA	0.7574124	-0.400849	0.0914033	364	291	458	191	311	341
Y_RNA	0.7574124	-0.400849	0.0914033	364	291	458	191	311	341
Y_RNA	0.7574124	-0.400849	0.0914033	364	291	458	191	311	341
Y_RNA	0.7574124	-0.400849	0.0914033	364	291	458	191	311	341
Y_RNA	0.7574124	-0.400849	0.0914033	364	291	458	191	311	341
Y_RNA	0.7574124	-0.400849	0.0914033	364	291	458	191	311	341
Y_RNA	0.7574124	-0.400849	0.0914033	364	291	458	191	311	341
Y_RNA	0.7574124	-0.400849	0.0914033	364	291	458	191	311	341
Y_RNA	0.7574124	-0.400849	0.0914033	364	291	458	191	311	341
Y_RNA	0.7574124	-0.400849	0.0914033	364	291	458	191	311	341
Y_RNA	0.7574124	-0.400849	0.0914033	364	291	458	191	311	341
Y_RNA	0.7574124	-0.400849	0.0914033	364	291	458	191	311	341
Y_RNA	0.7574124	-0.400849	0.0914033	364	291	458	191	311	341
Y_RNA	0.7574124	-0.400849	0.0914033	364	291	458	191	311	341
Y_RNA	0.7574124	-0.400849	0.0914033	364	291	458	191	311	341
Y_RNA	0.7574124	-0.400849	0.0914033	364	291	458	191	311	341
Y_RNA	0.7574124	-0.400849	0.0914033	364	291	458	191	311	341
Y_RNA	0.7574124	-0.400849	0.0914033	364	291	458	191	311	341
Y_RNA	0.7574124	-0.400849	0.0914033	364	291	458	191	311	341
Y_RNA	0.7574124	-0.400849	0.0914033	364	291	458	191	311	341
Y_RNA	0.7574124	-0.400849	0.0914033	364	291	458	191	311	341
Y_RNA	0.7574124	-0.400849	0.0914033	364	291	458	191	311	341
Y_RNA	0.7574124	-0.400849	0.0914033	364	291	458	191	311	341
Y_RNA	0.7574124	-0.400849	0.0914033	364	291	458	191	311	341
Y_RNA	0.7574124	-0.400849	0.0914033	364	291	458	191	311	341
Y_RNA	0.7574124	-0.400849	0.0914033	364	291	458	191	311	341
Y_RNA	0.7574124	-0.400849	0.0914033	364	291	458	191	311	341
Y_RNA	0.7574124	-0.400849	0.0914033	364	291	458	191	311	341
Y_RNA	0.7574124	-0.400849	0.0914033	364	291	458	191	311	341
Y_RNA	0.7574124	-0.400849	0.0914033	364	291	458	191	311	341
Y_RNA	0.7574124	-0.400849	0.0914033	364	291	458	191	311	341
Y_RNA	0.7574124	-0.400849	0.0914033	364	291	458	191	311	341
Y_RNA	0.7574124	-0.400849	0.0914033	364	291	458	191	311	341
Y_RNA	0.7574124	-0.400849	0.0914033	364	291	458	191	311	341
Y_RNA	0.7574124	-0.400849	0.0914033	364	291	458	191	311	341
Y_RNA	0.7574124	-0.400849	0.0914033	364	291	458	191	311	341
Y_RNA	0.7574124	-0.400849	0.0914033	364	291	458	191	311	341
Y_RNA	0.7574124	-0.400849	0.0914033	364	291	458	191	311	341
Y_RNA	0.7574124	-0.400849	0.0914033	364	291	458	191	311	341
Y_RNA	0.7574124	-0.400849	0.0914033	364	291	458	191	311	341
Y_RNA	0.7574124	-0.400849	0.0914033	364	291	458	191	311	341

Y_RNA	0.7574124	-0.400849	0.0914033	364	291	458	191	311	341
Y_RNA	0.7574124	-0.400849	0.0914033	364	291	458	191	311	341
Y_RNA	0.7574124	-0.400849	0.0914033	364	291	458	191	311	341
Y_RNA	0.7574124	-0.400849	0.0914033	364	291	458	191	311	341
Y_RNA	0.7574124	-0.400849	0.0914033	364	291	458	191	311	341
Y_RNA	0.7574124	-0.400849	0.0914033	364	291	458	191	311	341
Y_RNA	0.7574124	-0.400849	0.0914033	364	291	458	191	311	341
Y_RNA	0.7574124	-0.400849	0.0914033	364	291	458	191	311	341
Y_RNA	0.7574124	-0.400849	0.0914033	364	291	458	191	311	341
Y_RNA	0.7574124	-0.400849	0.0914033	364	291	458	191	311	341
Y_RNA	0.7574124	-0.400849	0.0914033	364	291	458	191	311	341
Y_RNA	0.7574124	-0.400849	0.0914033	364	291	458	191	311	341
Y_RNA	0.7574124	-0.400849	0.0914033	364	291	458	191	311	341
Y_RNA	0.7574124	-0.400849	0.0914033	364	291	458	191	311	341
Y_RNA	0.7574124	-0.400849	0.0914033	364	291	458	191	311	341
Y_RNA	0.7574124	-0.400849	0.0914033	364	291	458	191	311	341
Y_RNA	0.7574124	-0.400849	0.0914033	364	291	458	191	311	341
Y_RNA	0.7574124	-0.400849	0.0914033	364	291	458	191	311	341
Y_RNA	0.7574124	-0.400849	0.0914033	364	291	458	191	311	341
Y_RNA	0.7574124	-0.400849	0.0914033	364	291	458	191	311	341
Y_RNA	0.7574124	-0.400849	0.0914033	364	291	458	191	311	341
Y_RNA	0.7574124	-0.400849	0.0914033	364	291	458	191	311	341
Y_RNA	0.7574124	-0.400849	0.0914033	364	291	458	191	311	341
Y_RNA	0.7574124	-0.400849	0.0914033	364	291	458	191	311	341
Y_RNA	0.7574124	-0.400849	0.0914033	364	291	458	191	311	341
Y_RNA	0.7574124	-0.400849	0.0914033	364	291	458	191	311	341
Y_RNA	0.7574124	-0.400849	0.0914033	364	291	458	191	311	341
Y_RNA	0.7574124	-0.400849	0.0914033	364	291	458	191	311	341
Y_RNA	0.7574124	-0.400849	0.0914033	364	291	458	191	311	341
Y_RNA	0.7574124	-0.400849	0.0914033	364	291	458	191	311	341
Y_RNA	0.7574124	-0.400849	0.0914033	364	291	458	191	311	341
Y_RNA	0.7574124	-0.400849	0.0914033	364	291	458	191	311	341
Y_RNA	0.7574124	-0.400849	0.0914033	364	291	458	191	311	341
Y_RNA	0.7574124	-0.400849	0.0914033	364	291	458	191	311	341
Y_RNA	0.7574124	-0.400849	0.0914033	364	291	458	191	311	341
Y_RNA	0.7574124	-0.400849	0.0914033	364	291	458	191	311	341
Y_RNA	0.7574124	-0.400849	0.0914033	364	291	458	191	311	341
Y_RNA	0.7574124	-0.400849	0.0914033	364	291	458	191	311	341
Y_RNA	0.7574124	-0.400849	0.0914033	364	291	458	191	311	341
Y_RNA	0.7574124	-0.400849	0.0914033	364	291	458	191	311	341
Y_RNA	0.7574124	-0.400849	0.0914033	364	291	458	191	311	341
Y_RNA	0.7574124	-0.400849	0.0914033	364	291	458	191	311	341
Y_RNA	0.7574124	-0.400849	0.0914033	364	291	458	191	311	341
Y_RNA	0.7574124	-0.400849	0.0914033	364	291	458	191	311	341
Y_RNA	0.7574124	-0.400849	0.0914033	364	291	458	191	311	341
Y_RNA	0.7574124	-0.400849	0.0914033	364	291	458	191	311	341
Y_RNA	0.7574124	-0.400849	0.0914033	364	291	458	191	311	341
Y_RNA	0.7574124	-0.400849	0.0914033	364	291	458	191	311	341
Y_RNA	0.7574124	-0.400849	0.0914033	364	291	458	191	311	341
Y_RNA	0.7574124	-0.400849	0.0914033	364	291	458	191	311	341
Y_RNA	0.7574124	-0.400849	0.0914033	364	291	458	191	311	341
Y_RNA	0.7574124	-0.400849	0.0914033	364	291	458	191	311	341
Y_RNA	0.7574124	-0.400849	0.0914033	364	291	458	191	311	341
Y_RNA	0.7574124	-0.400849	0.0914033	364	291	458	191	311	341

Y_RNA	0.7574124	-0.400849	0.0914033	364	291	458	191	311	341
Y_RNA	0.7574124	-0.400849	0.0914033	364	291	458	191	311	341
Y_RNA	0.7574124	-0.400849	0.0914033	364	291	458	191	311	341
Y_RNA	0.7574124	-0.400849	0.0914033	364	291	458	191	311	341
Y_RNA	0.7574124	-0.400849	0.0914033	364	291	458	191	311	341
Y_RNA	0.7574124	-0.400849	0.0914033	364	291	458	191	311	341
Y_RNA	0.7574124	-0.400849	0.0914033	364	291	458	191	311	341
Y_RNA	0.7574124	-0.400849	0.0914033	364	291	458	191	311	341
Y_RNA	0.7574124	-0.400849	0.0914033	364	291	458	191	311	341
Y_RNA	0.7574124	-0.400849	0.0914033	364	291	458	191	311	341
Y_RNA	0.7574124	-0.400849	0.0914033	364	291	458	191	311	341
Y_RNA	0.7574124	-0.400849	0.0914033	364	291	458	191	311	341
Y_RNA	0.7574124	-0.400849	0.0914033	364	291	458	191	311	341
Y_RNA	0.7574124	-0.400849	0.0914033	364	291	458	191	311	341
Y_RNA	0.7574124	-0.400849	0.0914033	364	291	458	191	311	341
Y_RNA	0.7574124	-0.400849	0.0914033	364	291	458	191	311	341
Y_RNA	0.7574124	-0.400849	0.0914033	364	291	458	191	311	341
Y_RNA	0.7574124	-0.400849	0.0914033	364	291	458	191	311	341
Y_RNA	0.7574124	-0.400849	0.0914033	364	291	458	191	311	341
Y_RNA	0.7574124	-0.400849	0.0914033	364	291	458	191	311	341
Y_RNA	0.7574124	-0.400849	0.0914033	364	291	458	191	311	341
Y_RNA	0.7574124	-0.400849	0.0914033	364	291	458	191	311	341
Y_RNA	0.7574124	-0.400849	0.0914033	364	291	458	191	311	341
Y_RNA	0.7574124	-0.400849	0.0914033	364	291	458	191	311	341
Y_RNA	0.7574124	-0.400849	0.0914033	364	291	458	191	311	341
Y_RNA	0.7574124	-0.400849	0.0914033	364	291	458	191	311	341
Y_RNA	0.7574124	-0.400849	0.0914033	364	291	458	191	311	341
Y_RNA	0.7574124	-0.400849	0.0914033	364	291	458	191	311	341
Y_RNA	0.7574124	-0.400849	0.0914033	364	291	458	191	311	341
Y_RNA	0.7574124	-0.400849	0.0914033	364	291	458	191	311	341
Y_RNA	0.7574124	-0.400849	0.0914033	364	291	458	191	311	341
Y_RNA	0.7574124	-0.400849	0.0914033	364	291	458	191	311	341
Y_RNA	0.7574124	-0.400849	0.0914033	364	291	458	191	311	341
Y_RNA	0.7574124	-0.400849	0.0914033	364	291	458	191	311	341
Y_RNA	0.7574124	-0.400849	0.0914033	364	291	458	191	311	341
Y_RNA	0.7574124	-0.400849	0.0914033	364	291	458	191	311	341
Y_RNA	0.7574124	-0.400849	0.0914033	364	291	458	191	311	341
Y_RNA	0.7574124	-0.400849	0.0914033	364	291	458	191	311	341
Y_RNA	0.7574124	-0.400849	0.0914033	364	291	458	191	311	341
Y_RNA	0.7574124	-0.400849	0.0914033	364	291	458	191	311	341
Y_RNA	0.7574124	-0.400849	0.0914033	364	291	458	191	311	341
Y_RNA	0.7574124	-0.400849	0.0914033	364	291	458	191	311	341
Y_RNA	0.7574124	-0.400849	0.0914033	364	291	458	191	311	341
Y_RNA	0.7574124	-0.400849	0.0914033	364	291	458	191	311	341
Y_RNA	0.7574124	-0.400849	0.0914033	364	291	458	191	311	341
Y_RNA	0.7574124	-0.400849	0.0914033	364	291	458	191	311	341
Y_RNA	0.7574124	-0.400849	0.0914033	364	291	458	191	311	341
Y_RNA	0.7574124	-0.400849	0.0914033	364	291	458	191	311	341
Y_RNA	0.7574124	-0.400849	0.0914033	364	291	458	191	311	341
Y_RNA	0.7574124	-0.400849	0.0914033	364	291	458	191	311	341
Y_RNA	0.7574124	-0.400849	0.0914033	364	291	458	191	311	341
Y_RNA	0.7574124	-0.400849	0.0914033	364	291	458	191	311	341
Y_RNA	0.7574124	-0.400849	0.0914033	364	291	458	191	311	341
Y_RNA	0.7574124	-0.400849	0.0914033	364	291	458	191	311	341

Y_RNA	0.7574124	-0.400849	0.0914033	364	291	458	191	311	341
Y_RNA	0.7574124	-0.400849	0.0914033	364	291	458	191	311	341
Y_RNA	0.7574124	-0.400849	0.0914033	364	291	458	191	311	341
Y_RNA	0.7574124	-0.400849	0.0914033	364	291	458	191	311	341
Y_RNA	0.7574124	-0.400849	0.0914033	364	291	458	191	311	341
Y_RNA	0.7574124	-0.400849	0.0914033	364	291	458	191	311	341
Y_RNA	0.7574124	-0.400849	0.0914033	364	291	458	191	311	341
Y_RNA	0.7574124	-0.400849	0.0914033	364	291	458	191	311	341
Y_RNA	0.7574124	-0.400849	0.0914033	364	291	458	191	311	341
Y_RNA	0.7574124	-0.400849	0.0914033	364	291	458	191	311	341
Y_RNA	0.7574124	-0.400849	0.0914033	364	291	458	191	311	341
Y_RNA	0.7574124	-0.400849	0.0914033	364	291	458	191	311	341
Y_RNA	0.7574124	-0.400849	0.0914033	364	291	458	191	311	341
Y_RNA	0.7574124	-0.400849	0.0914033	364	291	458	191	311	341
Y_RNA	0.7574124	-0.400849	0.0914033	364	291	458	191	311	341
Y_RNA	0.7574124	-0.400849	0.0914033	364	291	458	191	311	341
Y_RNA	0.7574124	-0.400849	0.0914033	364	291	458	191	311	341
Y_RNA	0.7574124	-0.400849	0.0914033	364	291	458	191	311	341
Y_RNA	0.7574124	-0.400849	0.0914033	364	291	458	191	311	341
Y_RNA	0.7574124	-0.400849	0.0914033	364	291	458	191	311	341
Y_RNA	0.7574124	-0.400849	0.0914033	364	291	458	191	311	341
Y_RNA	0.7574124	-0.400849	0.0914033	364	291	458	191	311	341
Y_RNA	0.7574124	-0.400849	0.0914033	364	291	458	191	311	341
Y_RNA	0.7574124	-0.400849	0.0914033	364	291	458	191	311	341
Y_RNA	0.7574124	-0.400849	0.0914033	364	291	458	191	311	341
Y_RNA	0.7574124	-0.400849	0.0914033	364	291	458	191	311	341
Y_RNA	0.7574124	-0.400849	0.0914033	364	291	458	191	311	341
Y_RNA	0.7574124	-0.400849	0.0914033	364	291	458	191	311	341
Y_RNA	0.7574124	-0.400849	0.0914033	364	291	458	191	311	341
Y_RNA	0.7574124	-0.400849	0.0914033	364	291	458	191	311	341
Y_RNA	0.7574124	-0.400849	0.0914033	364	291	458	191	311	341
Y_RNA	0.7574124	-0.400849	0.0914033	364	291	458	191	311	341
Y_RNA	0.7574124	-0.400849	0.0914033	364	291	458	191	311	341
Y_RNA	0.7574124	-0.400849	0.0914033	364	291	458	191	311	341
Y_RNA	0.7574124	-0.400849	0.0914033	364	291	458	191	311	341
Y_RNA	0.7574124	-0.400849	0.0914033	364	291	458	191	311	341
Y_RNA	0.7574124	-0.400849	0.0914033	364	291	458	191	311	341
Y_RNA	0.7574124	-0.400849	0.0914033	364	291	458	191	311	341
Y_RNA	0.7574124	-0.400849	0.0914033	364	291	458	191	311	341
Y_RNA	0.7574124	-0.400849	0.0914033	364	291	458	191	311	341
Y_RNA	0.7574124	-0.400849	0.0914033	364	291	458	191	311	341
Y_RNA	0.7574124	-0.400849	0.0914033	364	291	458	191	311	341
Y_RNA	0.7574124	-0.400849	0.0914033	364	291	458	191	311	341
Y_RNA	0.7574124	-0.400849	0.0914033	364	291	458	191	311	341
Y_RNA	0.7574124	-0.400849	0.0914033	364	291	458	191	311	341
Y_RNA	0.7574124	-0.400849	0.0914033	364	291	458	191	311	341
Y_RNA	0.7574124	-0.400849	0.0914033	364	291	458	191	311	341
Y_RNA	0.7574124	-0.400849	0.0914033	364	291	458	191	311	341
Y_RNA	0.7574124	-0.400849	0.0914033	364	291	458	191	311	341
Y_RNA	0.7574124	-0.400849	0.0914033	364	291	458	191	311	341
Y_RNA	0.7574124	-0.400849	0.0914033	364	291	458	191	311	341
Y_RNA	0.7574124	-0.400849	0.0914033	364	291	458	191	311	341
Y_RNA	0.7574124	-0.400849	0.0914033	364	291	458	191	311	341
Y_RNA	0.7574124	-0.400849	0.0914033	364	291	458	191	311	341

Y_RNA	0.7574124	-0.400849	0.0914033	364	291	458	191	311	341
Y_RNA	0.7574124	-0.400849	0.0914033	364	291	458	191	311	341
Y_RNA	0.7574124	-0.400849	0.0914033	364	291	458	191	311	341
Y_RNA	0.7574124	-0.400849	0.0914033	364	291	458	191	311	341
Y_RNA	0.7574124	-0.400849	0.0914033	364	291	458	191	311	341
Y_RNA	0.7574124	-0.400849	0.0914033	364	291	458	191	311	341
Y_RNA	0.7574124	-0.400849	0.0914033	364	291	458	191	311	341
Y_RNA	0.7574124	-0.400849	0.0914033	364	291	458	191	311	341
Y_RNA	0.7574124	-0.400849	0.0914033	364	291	458	191	311	341
Y_RNA	0.7574124	-0.400849	0.0914033	364	291	458	191	311	341
Y_RNA	0.7574124	-0.400849	0.0914033	364	291	458	191	311	341
Y_RNA	0.7574124	-0.400849	0.0914033	364	291	458	191	311	341
Y_RNA	0.7574124	-0.400849	0.0914033	364	291	458	191	311	341
Y_RNA	0.7574124	-0.400849	0.0914033	364	291	458	191	311	341
Y_RNA	0.7574124	-0.400849	0.0914033	364	291	458	191	311	341
Y_RNA	0.7574124	-0.400849	0.0914033	364	291	458	191	311	341
Y_RNA	0.7574124	-0.400849	0.0914033	364	291	458	191	311	341
Y_RNA	0.7574124	-0.400849	0.0914033	364	291	458	191	311	341
Y_RNA	0.7574124	-0.400849	0.0914033	364	291	458	191	311	341
Y_RNA	0.7574124	-0.400849	0.0914033	364	291	458	191	311	341
Y_RNA	0.7574124	-0.400849	0.0914033	364	291	458	191	311	341
Y_RNA	0.7574124	-0.400849	0.0914033	364	291	458	191	311	341
Y_RNA	0.7574124	-0.400849	0.0914033	364	291	458	191	311	341
Y_RNA	0.7574124	-0.400849	0.0914033	364	291	458	191	311	341
Y_RNA	0.7574124	-0.400849	0.0914033	364	291	458	191	311	341
Y_RNA	0.7574124	-0.400849	0.0914033	364	291	458	191	311	341
Y_RNA	0.7574124	-0.400849	0.0914033	364	291	458	191	311	341
Y_RNA	0.7574124	-0.400849	0.0914033	364	291	458	191	311	341
Y_RNA	0.7574124	-0.400849	0.0914033	364	291	458	191	311	341
Y_RNA	0.7574124	-0.400849	0.0914033	364	291	458	191	311	341
Y_RNA	0.7574124	-0.400849	0.0914033	364	291	458	191	311	341
Y_RNA	0.7574124	-0.400849	0.0914033	364	291	458	191	311	341
Y_RNA	0.7574124	-0.400849	0.0914033	364	291	458	191	311	341
Y_RNA	0.7574124	-0.400849	0.0914033	364	291	458	191	311	341
Y_RNA	0.7574124	-0.400849	0.0914033	364	291	458	191	311	341
Y_RNA	0.7574124	-0.400849	0.0914033	364	291	458	191	311	341
Y_RNA	0.7574124	-0.400849	0.0914033	364	291	458	191	311	341
Y_RNA	0.7574124	-0.400849	0.0914033	364	291	458	191	311	341
Y_RNA	0.7574124	-0.400849	0.0914033	364	291	458	191	311	341
Y_RNA	0.7574124	-0.400849	0.0914033	364	291	458	191	311	341
Y_RNA	0.7574124	-0.400849	0.0914033	364	291	458	191	311	341
Y_RNA	0.7574124	-0.400849	0.0914033	364	291	458	191	311	341
Y_RNA	0.7574124	-0.400849	0.0914033	364	291	458	191	311	341
Y_RNA	0.7574124	-0.400849	0.0914033	364	291	458	191	311	341
Y_RNA	0.7574124	-0.400849	0.0914033	364	291	458	191	311	341
Y_RNA	0.7574124	-0.400849	0.0914033	364	291	458	191	311	341
Y_RNA	0.7574124	-0.400849	0.0914033	364	291	458	191	311	341
Y_RNA	0.7574124	-0.400849	0.0914033	364	291	458	191	311	341
Y_RNA	0.7574124	-0.400849	0.0914033	364	291	458	191	311	341
Y_RNA	0.7574124	-0.400849	0.0914033	364	291	458	191	311	341
Y_RNA	0.7574124	-0.400849	0.0914033	364	291	458	191	311	341
Y_RNA	0.7574124	-0.400849	0.0914033	364	291	458	191	311	341
Y_RNA	0.7574124	-0.400849	0.0914033	364	291	458	191	311	341
Y_RNA	0.7574124	-0.400849	0.0914033	364	291	458	191	311	341

Y_RNA	0.7574124	-0.400849	0.0914033	364	291	458	191	311	341
Y_RNA	0.7574124	-0.400849	0.0914033	364	291	458	191	311	341
Y_RNA	0.7574124	-0.400849	0.0914033	364	291	458	191	311	341
Y_RNA	0.7574124	-0.400849	0.0914033	364	291	458	191	311	341
Y_RNA	0.7574124	-0.400849	0.0914033	364	291	458	191	311	341
Y_RNA	0.7574124	-0.400849	0.0914033	364	291	458	191	311	341
Y_RNA	0.7574124	-0.400849	0.0914033	364	291	458	191	311	341
Y_RNA	0.7574124	-0.400849	0.0914033	364	291	458	191	311	341
Y_RNA	0.7574124	-0.400849	0.0914033	364	291	458	191	311	341
Y_RNA	0.7574124	-0.400849	0.0914033	364	291	458	191	311	341
Y_RNA	0.7574124	-0.400849	0.0914033	364	291	458	191	311	341
Y_RNA	0.7574124	-0.400849	0.0914033	364	291	458	191	311	341
Y_RNA	0.7574124	-0.400849	0.0914033	364	291	458	191	311	341
Y_RNA	0.7574124	-0.400849	0.0914033	364	291	458	191	311	341
Y_RNA	0.7574124	-0.400849	0.0914033	364	291	458	191	311	341
Y_RNA	0.7574124	-0.400849	0.0914033	364	291	458	191	311	341
Y_RNA	0.7574124	-0.400849	0.0914033	364	291	458	191	311	341
Y_RNA	0.7574124	-0.400849	0.0914033	364	291	458	191	311	341
Y_RNA	0.7574124	-0.400849	0.0914033	364	291	458	191	311	341
Y_RNA	0.7574124	-0.400849	0.0914033	364	291	458	191	311	341
Y_RNA	0.7574124	-0.400849	0.0914033	364	291	458	191	311	341
Y_RNA	0.7574124	-0.400849	0.0914033	364	291	458	191	311	341
Y_RNA	0.7574124	-0.400849	0.0914033	364	291	458	191	311	341
Y_RNA	0.7574124	-0.400849	0.0914033	364	291	458	191	311	341
Y_RNA	0.7574124	-0.400849	0.0914033	364	291	458	191	311	341
Y_RNA	0.7574124	-0.400849	0.0914033	364	291	458	191	311	341
Y_RNA	0.7574124	-0.400849	0.0914033	364	291	458	191	311	341
Y_RNA	0.7574124	-0.400849	0.0914033	364	291	458	191	311	341
Y_RNA	0.7574124	-0.400849	0.0914033	364	291	458	191	311	341
Y_RNA	0.7574124	-0.400849	0.0914033	364	291	458	191	311	341
Y_RNA	0.7574124	-0.400849	0.0914033	364	291	458	191	311	341
Y_RNA	0.7574124	-0.400849	0.0914033	364	291	458	191	311	341
Y_RNA	0.7574124	-0.400849	0.0914033	364	291	458	191	311	341
Y_RNA	0.7574124	-0.400849	0.0914033	364	291	458	191	311	341
Y_RNA	0.7574124	-0.400849	0.0914033	364	291	458	191	311	341
Y_RNA	0.7574124	-0.400849	0.0914033	364	291	458	191	311	341
Y_RNA	0.7574124	-0.400849	0.0914033	364	291	458	191	311	341
Y_RNA	0.7574124	-0.400849	0.0914033	364	291	458	191	311	341
Y_RNA	0.7574124	-0.400849	0.0914033	364	291	458	191	311	341
Y_RNA	0.7574124	-0.400849	0.0914033	364	291	458	191	311	341
Y_RNA	0.7574124	-0.400849	0.0914033	364	291	458	191	311	341
Y_RNA	0.7574124	-0.400849	0.0914033	364	291	458	191	311	341
Y_RNA	0.7574124	-0.400849	0.0914033	364	291	458	191	311	341
Y_RNA	0.7574124	-0.400849	0.0914033	364	291	458	191	311	341
Y_RNA	0.7574124	-0.400849	0.0914033	364	291	458	191	311	341
Y_RNA	0.7574124	-0.400849	0.0914033	364	291	458	191	311	341
Y_RNA	0.7574124	-0.400849	0.0914033	364	291	458	191	311	341
Y_RNA	0.7574124	-0.400849	0.0914033	364	291	458	191	311	341
Y_RNA	0.7574124	-0.400849	0.0914033	364	291	458	191	311	341
Y_RNA	0.7574124	-0.400849	0.0914033	364	291	458	191	311	341
Y_RNA	0.7574124	-0.400849	0.0914033	364	291	458	191	311	341
Y_RNA	0.7574124	-0.400849	0.0914033	364	291	458	191	311	341
Y_RNA	0.7574124	-0.400849	0.0914033	364	291	458	191	311	341
Y_RNA	0.7574124	-0.400849	0.0914033	364	291	458	191	311	341

Y_RNA	0.7574124	-0.400849	0.0914033	364	291	458	191	311	341
Y_RNA	0.7574124	-0.400849	0.0914033	364	291	458	191	311	341
Y_RNA	0.7574124	-0.400849	0.0914033	364	291	458	191	311	341
Y_RNA	0.7574124	-0.400849	0.0914033	364	291	458	191	311	341
Y_RNA	0.7574124	-0.400849	0.0914033	364	291	458	191	311	341
Y_RNA	0.7574124	-0.400849	0.0914033	364	291	458	191	311	341
Y_RNA	0.7574124	-0.400849	0.0914033	364	291	458	191	311	341
Y_RNA	0.7574124	-0.400849	0.0914033	364	291	458	191	311	341
Y_RNA	0.7574124	-0.400849	0.0914033	364	291	458	191	311	341
Y_RNA	0.7574124	-0.400849	0.0914033	364	291	458	191	311	341
Y_RNA	0.7574124	-0.400849	0.0914033	364	291	458	191	311	341
Y_RNA	0.7574124	-0.400849	0.0914033	364	291	458	191	311	341
Y_RNA	0.7574124	-0.400849	0.0914033	364	291	458	191	311	341
Y_RNA	0.7574124	-0.400849	0.0914033	364	291	458	191	311	341
Y_RNA	0.7574124	-0.400849	0.0914033	364	291	458	191	311	341
Y_RNA	0.7574124	-0.400849	0.0914033	364	291	458	191	311	341
Y_RNA	0.7574124	-0.400849	0.0914033	364	291	458	191	311	341
Y_RNA	0.7574124	-0.400849	0.0914033	364	291	458	191	311	341
Y_RNA	0.7574124	-0.400849	0.0914033	364	291	458	191	311	341
Y_RNA	0.7574124	-0.400849	0.0914033	364	291	458	191	311	341
Y_RNA	0.7574124	-0.400849	0.0914033	364	291	458	191	311	341
Y_RNA	0.7574124	-0.400849	0.0914033	364	291	458	191	311	341
Y_RNA	0.7574124	-0.400849	0.0914033	364	291	458	191	311	341
Y_RNA	0.7574124	-0.400849	0.0914033	364	291	458	191	311	341
Y_RNA	0.7574124	-0.400849	0.0914033	364	291	458	191	311	341
Y_RNA	0.7574124	-0.400849	0.0914033	364	291	458	191	311	341
Y_RNA	0.7574124	-0.400849	0.0914033	364	291	458	191	311	341
Y_RNA	0.7574124	-0.400849	0.0914033	364	291	458	191	311	341
Y_RNA	0.7574124	-0.400849	0.0914033	364	291	458	191	311	341
Y_RNA	0.7574124	-0.400849	0.0914033	364	291	458	191	311	341
Y_RNA	0.7574124	-0.400849	0.0914033	364	291	458	191	311	341
Y_RNA	0.7574124	-0.400849	0.0914033	364	291	458	191	311	341
Y_RNA	0.7574124	-0.400849	0.0914033	364	291	458	191	311	341
Y_RNA	0.7574124	-0.400849	0.0914033	364	291	458	191	311	341
Y_RNA	0.7574124	-0.400849	0.0914033	364	291	458	191	311	341
Y_RNA	0.7574124	-0.400849	0.0914033	364	291	458	191	311	341
Y_RNA	0.7574124	-0.400849	0.0914033	364	291	458	191	311	341
Y_RNA	0.7574124	-0.400849	0.0914033	364	291	458	191	311	341
Y_RNA	0.7574124	-0.400849	0.0914033	364	291	458	191	311	341
Y_RNA	0.7574124	-0.400849	0.0914033	364	291	458	191	311	341
Y_RNA	0.7574124	-0.400849	0.0914033	364	291	458	191	311	341
Y_RNA	0.7574124	-0.400849	0.0914033	364	291	458	191	311	341
Y_RNA	0.7574124	-0.400849	0.0914033	364	291	458	191	311	341
Y_RNA	0.7574124	-0.400849	0.0914033	364	291	458	191	311	341
Y_RNA	0.7574124	-0.400849	0.0914033	364	291	458	191	311	341
Y_RNA	0.7574124	-0.400849	0.0914033	364	291	458	191	311	341
Y_RNA	0.7574124	-0.400849	0.0914033	364	291	458	191	311	341
Y_RNA	0.7574124	-0.400849	0.0914033	364	291	458	191	311	341
Y_RNA	0.7574124	-0.400849	0.0914033	364	291	458	191	311	341
Y_RNA	0.7574124	-0.400849	0.0914033	364	291	458	191	311	341
Y_RNA	0.7574124	-0.400849	0.0914033	364	291	458	191	311	341
Y_RNA	0.7574124	-0.400849	0.0914033	364	291	458	191	311	341
Y_RNA	0.7574124	-0.400849	0.0914033	364	291	458	191	311	341
Y_RNA	0.7574124	-0.400849	0.0914033	364	291	458	191	311	341

Y_RNA	0.7574124	-0.400849	0.0914033	364	291	458	191	311	341
Y_RNA	0.7574124	-0.400849	0.0914033	364	291	458	191	311	341
Y_RNA	0.7574124	-0.400849	0.0914033	364	291	458	191	311	341
Y_RNA	0.7574124	-0.400849	0.0914033	364	291	458	191	311	341
Y_RNA	0.7574124	-0.400849	0.0914033	364	291	458	191	311	341
Y_RNA	0.7574124	-0.400849	0.0914033	364	291	458	191	311	341
Y_RNA	0.7574124	-0.400849	0.0914033	364	291	458	191	311	341
Y_RNA	0.7574124	-0.400849	0.0914033	364	291	458	191	311	341
Y_RNA	0.7574124	-0.400849	0.0914033	364	291	458	191	311	341
Y_RNA	0.7574124	-0.400849	0.0914033	364	291	458	191	311	341
Y_RNA	0.7574124	-0.400849	0.0914033	364	291	458	191	311	341
Y_RNA	0.7574124	-0.400849	0.0914033	364	291	458	191	311	341
Y_RNA	0.7574124	-0.400849	0.0914033	364	291	458	191	311	341
Y_RNA	0.7574124	-0.400849	0.0914033	364	291	458	191	311	341
Y_RNA	0.7574124	-0.400849	0.0914033	364	291	458	191	311	341
Y_RNA	0.7574124	-0.400849	0.0914033	364	291	458	191	311	341
Y_RNA	0.7574124	-0.400849	0.0914033	364	291	458	191	311	341
Y_RNA	0.7574124	-0.400849	0.0914033	364	291	458	191	311	341
Y_RNA	0.7574124	-0.400849	0.0914033	364	291	458	191	311	341
Y_RNA	0.7574124	-0.400849	0.0914033	364	291	458	191	311	341
Y_RNA	0.7574124	-0.400849	0.0914033	364	291	458	191	311	341
Y_RNA	0.7574124	-0.400849	0.0914033	364	291	458	191	311	341
Y_RNA	0.7574124	-0.400849	0.0914033	364	291	458	191	311	341
Y_RNA	0.7574124	-0.400849	0.0914033	364	291	458	191	311	341
Y_RNA	0.7574124	-0.400849	0.0914033	364	291	458	191	311	341
Y_RNA	0.7574124	-0.400849	0.0914033	364	291	458	191	311	341
Y_RNA	0.7574124	-0.400849	0.0914033	364	291	458	191	311	341
Y_RNA	0.7574124	-0.400849	0.0914033	364	291	458	191	311	341
Y_RNA	0.7574124	-0.400849	0.0914033	364	291	458	191	311	341
Y_RNA	0.7574124	-0.400849	0.0914033	364	291	458	191	311	341
Y_RNA	0.7574124	-0.400849	0.0914033	364	291	458	191	311	341
Y_RNA	0.7574124	-0.400849	0.0914033	364	291	458	191	311	341
Y_RNA	0.7574124	-0.400849	0.0914033	364	291	458	191	311	341
Y_RNA	0.7574124	-0.400849	0.0914033	364	291	458	191	311	341
Y_RNA	0.7574124	-0.400849	0.0914033	364	291	458	191	311	341
Y_RNA	0.7574124	-0.400849	0.0914033	364	291	458	191	311	341
Y_RNA	0.7574124	-0.400849	0.0914033	364	291	458	191	311	341
Y_RNA	0.7574124	-0.400849	0.0914033	364	291	458	191	311	341
Y_RNA	0.7574124	-0.400849	0.0914033	364	291	458	191	311	341
Y_RNA	0.7574124	-0.400849	0.0914033	364	291	458	191	311	341
Y_RNA	0.7574124	-0.400849	0.0914033	364	291	458	191	311	341
Y_RNA	0.7574124	-0.400849	0.0914033	364	291	458	191	311	341
Y_RNA	0.7574124	-0.400849	0.0914033	364	291	458	191	311	341
Y_RNA	0.7574124	-0.400849	0.0914033	364	291	458	191	311	341
Y_RNA	0.7574124	-0.400849	0.0914033	364	291	458	191	311	341
Y_RNA	0.7574124	-0.400849	0.0914033	364	291	458	191	311	341
Y_RNA	0.7574124	-0.400849	0.0914033	364	291	458	191	311	341
Y_RNA	0.7574124	-0.400849	0.0914033	364	291	458	191	311	341
Y_RNA	0.7574124	-0.400849	0.0914033	364	291	458	191	311	341
Y_RNA	0.7574124	-0.400849	0.0914033	364	291	458	191	311	341
Y_RNA	0.7574124	-0.400849	0.0914033	364	291	458	191	311	341
Y_RNA	0.7574124	-0.400849	0.0914033	364	291	458	191	311	341
Y_RNA	0.7574124	-0.400849	0.0914033	364	291	458	191	311	341
Y_RNA	0.7574124	-0.400849	0.0914033	364	291	458	191	311	341

Y_RNA	0.7574124	-0.400849	0.0914033	364	291	458	191	311	341
Y_RNA	0.7574124	-0.400849	0.0914033	364	291	458	191	311	341
Y_RNA	0.7574124	-0.400849	0.0914033	364	291	458	191	311	341
Y_RNA	0.7574124	-0.400849	0.0914033	364	291	458	191	311	341
Y_RNA	0.7574124	-0.400849	0.0914033	364	291	458	191	311	341
Y_RNA	0.7574124	-0.400849	0.0914033	364	291	458	191	311	341
Y_RNA	0.7574124	-0.400849	0.0914033	364	291	458	191	311	341
Y_RNA	0.7574124	-0.400849	0.0914033	364	291	458	191	311	341
Y_RNA	0.7574124	-0.400849	0.0914033	364	291	458	191	311	341
Y_RNA	0.7574124	-0.400849	0.0914033	364	291	458	191	311	341
Y_RNA	0.7574124	-0.400849	0.0914033	364	291	458	191	311	341
Y_RNA	0.7574124	-0.400849	0.0914033	364	291	458	191	311	341
Y_RNA	0.7574124	-0.400849	0.0914033	364	291	458	191	311	341
Y_RNA	0.7574124	-0.400849	0.0914033	364	291	458	191	311	341
Y_RNA	0.7574124	-0.400849	0.0914033	364	291	458	191	311	341
Y_RNA	0.7574124	-0.400849	0.0914033	364	291	458	191	311	341
Y_RNA	0.7574124	-0.400849	0.0914033	364	291	458	191	311	341
Y_RNA	0.7574124	-0.400849	0.0914033	364	291	458	191	311	341
Y_RNA	0.7574124	-0.400849	0.0914033	364	291	458	191	311	341
Y_RNA	0.7574124	-0.400849	0.0914033	364	291	458	191	311	341
Y_RNA	0.7574124	-0.400849	0.0914033	364	291	458	191	311	341
Y_RNA	0.7574124	-0.400849	0.0914033	364	291	458	191	311	341
Y_RNA	0.7574124	-0.400849	0.0914033	364	291	458	191	311	341
Y_RNA	0.7574124	-0.400849	0.0914033	364	291	458	191	311	341
Y_RNA	0.7574124	-0.400849	0.0914033	364	291	458	191	311	341
Y_RNA	0.7574124	-0.400849	0.0914033	364	291	458	191	311	341
Y_RNA	0.7574124	-0.400849	0.0914033	364	291	458	191	311	341
Y_RNA	0.7574124	-0.400849	0.0914033	364	291	458	191	311	341
Y_RNA	0.7574124	-0.400849	0.0914033	364	291	458	191	311	341
Y_RNA	0.7574124	-0.400849	0.0914033	364	291	458	191	311	341
Y_RNA	0.7574124	-0.400849	0.0914033	364	291	458	191	311	341
Y_RNA	0.7574124	-0.400849	0.0914033	364	291	458	191	311	341
Y_RNA	0.7574124	-0.400849	0.0914033	364	291	458	191	311	341
Y_RNA	0.7574124	-0.400849	0.0914033	364	291	458	191	311	341
Y_RNA	0.7574124	-0.400849	0.0914033	364	291	458	191	311	341
Y_RNA	0.7574124	-0.400849	0.0914033	364	291	458	191	311	341
Y_RNA	0.7574124	-0.400849	0.0914033	364	291	458	191	311	341
Y_RNA	0.7574124	-0.400849	0.0914033	364	291	458	191	311	341
Y_RNA	0.7574124	-0.400849	0.0914033	364	291	458	191	311	341
Y_RNA	0.7574124	-0.400849	0.0914033	364	291	458	191	311	341
Y_RNA	0.7574124	-0.400849	0.0914033	364	291	458	191	311	341
Y_RNA	0.7574124	-0.400849	0.0914033	364	291	458	191	311	341
Y_RNA	0.7574124	-0.400849	0.0914033	364	291	458	191	311	341
Y_RNA	0.7574124	-0.400849	0.0914033	364	291	458	191	311	341
Y_RNA	0.7574124	-0.400849	0.0914033	364	291	458	191	311	341
Y_RNA	0.7574124	-0.400849	0.0914033	364	291	458	191	311	341
Y_RNA	0.7574124	-0.400849	0.0914033	364	291	458	191	311	341
Y_RNA	0.7574124	-0.400849	0.0914033	364	291	458	191	311	341
Y_RNA	0.7574124	-0.400849	0.0914033	364	291	458	191	311	341
Y_RNA	0.7574124	-0.400849	0.0914033	364	291	458	191	311	341
Y_RNA	0.7574124	-0.400849	0.0914033	364	291	458	191	311	341
Y_RNA	0.7574124	-0.400849	0.0914033	364	291	458	191	311	341
Y_RNA	0.7574124	-0.400849	0.0914033	364	291	458	191	311	341
Y_RNA	0.7574124	-0.400849	0.0914033	364	291	458	191	311	341

Y_RNA	0.7574124	-0.400849	0.0914033	364	291	458	191	311	341
Y_RNA	0.7574124	-0.400849	0.0914033	364	291	458	191	311	341
Y_RNA	0.7574124	-0.400849	0.0914033	364	291	458	191	311	341
Y_RNA	0.7574124	-0.400849	0.0914033	364	291	458	191	311	341
Y_RNA	0.7574124	-0.400849	0.0914033	364	291	458	191	311	341
Y_RNA	0.7574124	-0.400849	0.0914033	364	291	458	191	311	341
Y_RNA	0.7574124	-0.400849	0.0914033	364	291	458	191	311	341
Y_RNA	0.7574124	-0.400849	0.0914033	364	291	458	191	311	341
Y_RNA	0.7574124	-0.400849	0.0914033	364	291	458	191	311	341
Y_RNA	0.7574124	-0.400849	0.0914033	364	291	458	191	311	341
Y_RNA	0.7574124	-0.400849	0.0914033	364	291	458	191	311	341
Y_RNA	0.7574124	-0.400849	0.0914033	364	291	458	191	311	341
Y_RNA	0.7574124	-0.400849	0.0914033	364	291	458	191	311	341
Y_RNA	0.7574124	-0.400849	0.0914033	364	291	458	191	311	341
Y_RNA	0.7574124	-0.400849	0.0914033	364	291	458	191	311	341
Y_RNA	0.7574124	-0.400849	0.0914033	364	291	458	191	311	341
Y_RNA	0.7574124	-0.400849	0.0914033	364	291	458	191	311	341
Y_RNA	0.7574124	-0.400849	0.0914033	364	291	458	191	311	341
Y_RNA	0.7574124	-0.400849	0.0914033	364	291	458	191	311	341
Y_RNA	0.7574124	-0.400849	0.0914033	364	291	458	191	311	341
Y_RNA	0.7574124	-0.400849	0.0914033	364	291	458	191	311	341
Y_RNA	0.7574124	-0.400849	0.0914033	364	291	458	191	311	341
Y_RNA	0.7574124	-0.400849	0.0914033	364	291	458	191	311	341
Y_RNA	0.7574124	-0.400849	0.0914033	364	291	458	191	311	341
Y_RNA	0.7574124	-0.400849	0.0914033	364	291	458	191	311	341
Y_RNA	0.7574124	-0.400849	0.0914033	364	291	458	191	311	341
Y_RNA	0.7574124	-0.400849	0.0914033	364	291	458	191	311	341
Y_RNA	0.7574124	-0.400849	0.0914033	364	291	458	191	311	341
Y_RNA	0.7574124	-0.400849	0.0914033	364	291	458	191	311	341
Y_RNA	0.7574124	-0.400849	0.0914033	364	291	458	191	311	341
Y_RNA	0.7574124	-0.400849	0.0914033	364	291	458	191	311	341
Y_RNA	0.7574124	-0.400849	0.0914033	364	291	458	191	311	341
Y_RNA	0.7574124	-0.400849	0.0914033	364	291	458	191	311	341
Y_RNA	0.7574124	-0.400849	0.0914033	364	291	458	191	311	341
Y_RNA	0.7574124	-0.400849	0.0914033	364	291	458	191	311	341
Y_RNA	0.7574124	-0.400849	0.0914033	364	291	458	191	311	341
Y_RNA	0.7574124	-0.400849	0.0914033	364	291	458	191	311	341
Y_RNA	0.7574124	-0.400849	0.0914033	364	291	458	191	311	341
Y_RNA	0.7574124	-0.400849	0.0914033	364	291	458	191	311	341
Y_RNA	0.7574124	-0.400849	0.0914033	364	291	458	191	311	341
Y_RNA	0.7574124	-0.400849	0.0914033	364	291	458	191	311	341
Y_RNA	0.7574124	-0.400849	0.0914033	364	291	458	191	311	341
Y_RNA	0.7574124	-0.400849	0.0914033	364	291	458	191	311	341
Y_RNA	0.7574124	-0.400849	0.0914033	364	291	458	191	311	341
Y_RNA	0.7574124	-0.400849	0.0914033	364	291	458	191	311	341
Y_RNA	0.7574124	-0.400849	0.0914033	364	291	458	191	311	341
Y_RNA	0.7574124	-0.400849	0.0914033	364	291	458	191	311	341
Y_RNA	0.7574124	-0.400849	0.0914033	364	291	458	191	311	341
Y_RNA	0.7574124	-0.400849	0.0914033	364	291	458	191	311	341
Y_RNA	0.7574124	-0.400849	0.0914033	364	291	458	191	311	341
Y_RNA	0.7574124	-0.400849	0.0914033	364	291	458	191	311	341
Y_RNA	0.7574124	-0.400849	0.0914033	364	291	458	191	311	341
Y_RNA	0.7574124	-0.400849	0.0914033	364	291	458	191	311	341
Y_RNA	0.7574124	-0.400849	0.0914033	364	291	458	191	311	341

Y_RNA	0.7574124	-0.400849	0.0914033	364	291	458	191	311	341
Y_RNA	0.7574124	-0.400849	0.0914033	364	291	458	191	311	341
Y_RNA	0.7574124	-0.400849	0.0914033	364	291	458	191	311	341
Y_RNA	0.7574124	-0.400849	0.0914033	364	291	458	191	311	341
Y_RNA	0.7574124	-0.400849	0.0914033	364	291	458	191	311	341
Y_RNA	0.7574124	-0.400849	0.0914033	364	291	458	191	311	341
Y_RNA	0.7574124	-0.400849	0.0914033	364	291	458	191	311	341
Y_RNA	0.7574124	-0.400849	0.0914033	364	291	458	191	311	341
Y_RNA	0.7574124	-0.400849	0.0914033	364	291	458	191	311	341
Y_RNA	0.7574124	-0.400849	0.0914033	364	291	458	191	311	341
Y_RNA	0.7574124	-0.400849	0.0914033	364	291	458	191	311	341
Y_RNA	0.7574124	-0.400849	0.0914033	364	291	458	191	311	341
Y_RNA	0.7574124	-0.400849	0.0914033	364	291	458	191	311	341
Y_RNA	0.7574124	-0.400849	0.0914033	364	291	458	191	311	341
Y_RNA	0.7574124	-0.400849	0.0914033	364	291	458	191	311	341
Y_RNA	0.7574124	-0.400849	0.0914033	364	291	458	191	311	341
Y_RNA	0.7574124	-0.400849	0.0914033	364	291	458	191	311	341
Y_RNA	0.7574124	-0.400849	0.0914033	364	291	458	191	311	341
Y_RNA	0.7574124	-0.400849	0.0914033	364	291	458	191	311	341
Y_RNA	0.7574124	-0.400849	0.0914033	364	291	458	191	311	341
Y_RNA	0.7574124	-0.400849	0.0914033	364	291	458	191	311	341
Y_RNA	0.7574124	-0.400849	0.0914033	364	291	458	191	311	341
Y_RNA	0.7574124	-0.400849	0.0914033	364	291	458	191	311	341
Y_RNA	0.7574124	-0.400849	0.0914033	364	291	458	191	311	341
Y_RNA	0.7574124	-0.400849	0.0914033	364	291	458	191	311	341
Y_RNA	0.7574124	-0.400849	0.0914033	364	291	458	191	311	341
Y_RNA	0.7574124	-0.400849	0.0914033	364	291	458	191	311	341
Y_RNA	0.7574124	-0.400849	0.0914033	364	291	458	191	311	341
Y_RNA	0.7574124	-0.400849	0.0914033	364	291	458	191	311	341
Y_RNA	0.7574124	-0.400849	0.0914033	364	291	458	191	311	341
Y_RNA	0.7574124	-0.400849	0.0914033	364	291	458	191	311	341
Y_RNA	0.7574124	-0.400849	0.0914033	364	291	458	191	311	341
Y_RNA	0.7574124	-0.400849	0.0914033	364	291	458	191	311	341
Y_RNA	0.7574124	-0.400849	0.0914033	364	291	458	191	311	341
Y_RNA	0.7574124	-0.400849	0.0914033	364	291	458	191	311	341
Y_RNA	0.7574124	-0.400849	0.0914033	364	291	458	191	311	341
Y_RNA	0.7574124	-0.400849	0.0914033	364	291	458	191	311	341
Y_RNA	0.7574124	-0.400849	0.0914033	364	291	458	191	311	341
Y_RNA	0.7574124	-0.400849	0.0914033	364	291	458	191	311	341
Y_RNA	0.7574124	-0.400849	0.0914033	364	291	458	191	311	341
Y_RNA	0.7574124	-0.400849	0.0914033	364	291	458	191	311	341
Y_RNA	0.7574124	-0.400849	0.0914033	364	291	458	191	311	341
Y_RNA	0.7574124	-0.400849	0.0914033	364	291	458	191	311	341
Y_RNA	0.7574124	-0.400849	0.0914033	364	291	458	191	311	341
Y_RNA	0.7574124	-0.400849	0.0914033	364	291	458	191	311	341
Y_RNA	0.7574124	-0.400849	0.0914033	364	291	458	191	311	341
Y_RNA	0.7574124	-0.400849	0.0914033	364	291	458	191	311	341
Y_RNA	0.7574124	-0.400849	0.0914033	364	291	458	191	311	341
Y_RNA	0.7574124	-0.400849	0.0914033	364	291	458	191	311	341
Y_RNA	0.7574124	-0.400849	0.0914033	364	291	458	191	311	341
Y_RNA	0.7574124	-0.400849	0.0914033	364	291	458	191	311	341
Y_RNA	0.7574124	-0.400849	0.0914033	364	291	458	191	311	341
Y_RNA	0.7574124	-0.400849	0.0914033	364	291	458	191	311	341
Y_RNA	0.7574124	-0.400849	0.0914033	364	291	458	191	311	341

Y_RNA	0.7574124	-0.400849	0.0914033	364	291	458	191	311	341
Y_RNA	0.7574124	-0.400849	0.0914033	364	291	458	191	311	341
Y_RNA	0.7574124	-0.400849	0.0914033	364	291	458	191	311	341
Y_RNA	0.7574124	-0.400849	0.0914033	364	291	458	191	311	341
Y_RNA	0.7574124	-0.400849	0.0914033	364	291	458	191	311	341
Y_RNA	0.7574124	-0.400849	0.0914033	364	291	458	191	311	341
Y_RNA	0.7574124	-0.400849	0.0914033	364	291	458	191	311	341
Y_RNA	0.7574124	-0.400849	0.0914033	364	291	458	191	311	341
Y_RNA	0.7574124	-0.400849	0.0914033	364	291	458	191	311	341
Y_RNA	0.7574124	-0.400849	0.0914033	364	291	458	191	311	341
Y_RNA	0.7574124	-0.400849	0.0914033	364	291	458	191	311	341
Y_RNA	0.7574124	-0.400849	0.0914033	364	291	458	191	311	341
Y_RNA	0.7574124	-0.400849	0.0914033	364	291	458	191	311	341
Y_RNA	0.7574124	-0.400849	0.0914033	364	291	458	191	311	341
Y_RNA	0.7574124	-0.400849	0.0914033	364	291	458	191	311	341
Y_RNA	0.7574124	-0.400849	0.0914033	364	291	458	191	311	341
Y_RNA	0.7574124	-0.400849	0.0914033	364	291	458	191	311	341
Y_RNA	0.7574124	-0.400849	0.0914033	364	291	458	191	311	341
Y_RNA	0.7574124	-0.400849	0.0914033	364	291	458	191	311	341
Y_RNA	0.7574124	-0.400849	0.0914033	364	291	458	191	311	341
Y_RNA	0.7574124	-0.400849	0.0914033	364	291	458	191	311	341
Y_RNA	0.7574124	-0.400849	0.0914033	364	291	458	191	311	341
Y_RNA	0.7574124	-0.400849	0.0914033	364	291	458	191	311	341
Y_RNA	0.7574124	-0.400849	0.0914033	364	291	458	191	311	341
Y_RNA	0.7574124	-0.400849	0.0914033	364	291	458	191	311	341
Y_RNA	0.7574124	-0.400849	0.0914033	364	291	458	191	311	341
Y_RNA	0.7574124	-0.400849	0.0914033	364	291	458	191	311	341
Y_RNA	0.7574124	-0.400849	0.0914033	364	291	458	191	311	341
Y_RNA	0.7574124	-0.400849	0.0914033	364	291	458	191	311	341
Y_RNA	0.7574124	-0.400849	0.0914033	364	291	458	191	311	341
Y_RNA	0.7574124	-0.400849	0.0914033	364	291	458	191	311	341
Y_RNA	0.7574124	-0.400849	0.0914033	364	291	458	191	311	341
Y_RNA	0.7574124	-0.400849	0.0914033	364	291	458	191	311	341
Y_RNA	0.7574124	-0.400849	0.0914033	364	291	458	191	311	341
Y_RNA	0.7574124	-0.400849	0.0914033	364	291	458	191	311	341
Y_RNA	0.7574124	-0.400849	0.0914033	364	291	458	191	311	341
Y_RNA	0.7574124	-0.400849	0.0914033	364	291	458	191	311	341
Y_RNA	0.7574124	-0.400849	0.0914033	364	291	458	191	311	341
Y_RNA	0.7574124	-0.400849	0.0914033	364	291	458	191	311	341
Y_RNA	0.7574124	-0.400849	0.0914033	364	291	458	191	311	341
Y_RNA	0.7574124	-0.400849	0.0914033	364	291	458	191	311	341
Y_RNA	0.7574124	-0.400849	0.0914033	364	291	458	191	311	341
Y_RNA	0.7574124	-0.400849	0.0914033	364	291	458	191	311	341
Y_RNA	0.7574124	-0.400849	0.0914033	364	291	458	191	311	341
Y_RNA	0.7574124	-0.400849	0.0914033	364	291	458	191	311	341
Y_RNA	0.7574124	-0.400849	0.0914033	364	291	458	191	311	341
Y_RNA	0.7574124	-0.400849	0.0914033	364	291	458	191	311	341
Y_RNA	0.7574124	-0.400849	0.0914033	364	291	458	191	311	341
Y_RNA	0.7574124	-0.400849	0.0914033	364	291	458	191	311	341
Y_RNA	0.7574124	-0.400849	0.0914033	364	291	458	191	311	341
Y_RNA	0.7574124	-0.400849	0.0914033	364	291	458	191	311	341
Y_RNA	0.7574124	-0.400849	0.0914033	364	291	458	191	311	341
Y_RNA	0.7574124	-0.400849	0.0914033	364	291	458	191	311	341
Y_RNA	0.7574124	-0.400849	0.0914033	364	291	458	191	311	341

Y_RNA	0.7574124	-0.400849	0.0914033	364	291	458	191	311	341
Y_RNA	0.7574124	-0.400849	0.0914033	364	291	458	191	311	341
Y_RNA	0.7574124	-0.400849	0.0914033	364	291	458	191	311	341
Y_RNA	0.7574124	-0.400849	0.0914033	364	291	458	191	311	341
Y_RNA	0.7574124	-0.400849	0.0914033	364	291	458	191	311	341
Y_RNA	0.7574124	-0.400849	0.0914033	364	291	458	191	311	341
Y_RNA	0.7574124	-0.400849	0.0914033	364	291	458	191	311	341
Y_RNA	0.7574124	-0.400849	0.0914033	364	291	458	191	311	341
Y_RNA	0.7574124	-0.400849	0.0914033	364	291	458	191	311	341
Y_RNA	0.7574124	-0.400849	0.0914033	364	291	458	191	311	341
Y_RNA	0.7574124	-0.400849	0.0914033	364	291	458	191	311	341
Y_RNA	0.7574124	-0.400849	0.0914033	364	291	458	191	311	341
Y_RNA	0.7574124	-0.400849	0.0914033	364	291	458	191	311	341
Y_RNA	0.7574124	-0.400849	0.0914033	364	291	458	191	311	341
Y_RNA	0.7574124	-0.400849	0.0914033	364	291	458	191	311	341
Y_RNA	0.7574124	-0.400849	0.0914033	364	291	458	191	311	341
Y_RNA	0.7574124	-0.400849	0.0914033	364	291	458	191	311	341
Y_RNA	0.7574124	-0.400849	0.0914033	364	291	458	191	311	341
Y_RNA	0.7574124	-0.400849	0.0914033	364	291	458	191	311	341
Y_RNA	0.7574124	-0.400849	0.0914033	364	291	458	191	311	341
Y_RNA	0.7574124	-0.400849	0.0914033	364	291	458	191	311	341
Y_RNA	0.7574124	-0.400849	0.0914033	364	291	458	191	311	341
Y_RNA	0.7574124	-0.400849	0.0914033	364	291	458	191	311	341
Y_RNA	0.7574124	-0.400849	0.0914033	364	291	458	191	311	341
Y_RNA	0.7574124	-0.400849	0.0914033	364	291	458	191	311	341
Y_RNA	0.7574124	-0.400849	0.0914033	364	291	458	191	311	341
Y_RNA	0.7574124	-0.400849	0.0914033	364	291	458	191	311	341
Y_RNA	0.7574124	-0.400849	0.0914033	364	291	458	191	311	341
Y_RNA	0.7574124	-0.400849	0.0914033	364	291	458	191	311	341
Y_RNA	0.7574124	-0.400849	0.0914033	364	291	458	191	311	341
Y_RNA	0.7574124	-0.400849	0.0914033	364	291	458	191	311	341
Y_RNA	0.7574124	-0.400849	0.0914033	364	291	458	191	311	341
Y_RNA	0.7574124	-0.400849	0.0914033	364	291	458	191	311	341
Y_RNA	0.7574124	-0.400849	0.0914033	364	291	458	191	311	341
Y_RNA	0.7574124	-0.400849	0.0914033	364	291	458	191	311	341
Y_RNA	0.7574124	-0.400849	0.0914033	364	291	458	191	311	341
Y_RNA	0.7574124	-0.400849	0.0914033	364	291	458	191	311	341
Y_RNA	0.7574124	-0.400849	0.0914033	364	291	458	191	311	341
Y_RNA	0.7574124	-0.400849	0.0914033	364	291	458	191	311	341
Y_RNA	0.7574124	-0.400849	0.0914033	364	291	458	191	311	341
Y_RNA	0.7574124	-0.400849	0.0914033	364	291	458	191	311	341
Y_RNA	0.7574124	-0.400849	0.0914033	364	291	458	191	311	341
Y_RNA	0.7574124	-0.400849	0.0914033	364	291	458	191	311	341
Y_RNA	0.7574124	-0.400849	0.0914033	364	291	458	191	311	341
Y_RNA	0.7574124	-0.400849	0.0914033	364	291	458	191	311	341
Y_RNA	0.7574124	-0.400849	0.0914033	364	291	458	191	311	341
Y_RNA	0.7574124	-0.400849	0.0914033	364	291	458	191	311	341
Y_RNA	0.7574124	-0.400849	0.0914033	364	291	458	191	311	341
Y_RNA	0.7574124	-0.400849	0.0914033	364	291	458	191	311	341
Y_RNA	0.7574124	-0.400849	0.0914033	364	291	458	191	311	341
Y_RNA	0.7574124	-0.400849	0.0914033	364	291	458	191	311	341
Y_RNA	0.7574124	-0.400849	0.0914033	364	291	458	191	311	341
Y_RNA	0.7574124	-0.400849	0.0914033	364	291	458	191	311	341
Y_RNA	0.7574124	-0.400849	0.0914033	364	291	458	191	311	341

Y_RNA	0.7574124	-0.400849	0.0914033	364	291	458	191	311	341
Y_RNA	0.7574124	-0.400849	0.0914033	364	291	458	191	311	341
Y_RNA	0.7574124	-0.400849	0.0914033	364	291	458	191	311	341
Y_RNA	0.7574124	-0.400849	0.0914033	364	291	458	191	311	341
Y_RNA	0.7574124	-0.400849	0.0914033	364	291	458	191	311	341
Y_RNA	0.7574124	-0.400849	0.0914033	364	291	458	191	311	341
Y_RNA	0.7574124	-0.400849	0.0914033	364	291	458	191	311	341
Y_RNA	0.7574124	-0.400849	0.0914033	364	291	458	191	311	341
Y_RNA	0.7574124	-0.400849	0.0914033	364	291	458	191	311	341
Y_RNA	0.7574124	-0.400849	0.0914033	364	291	458	191	311	341
Y_RNA	0.7574124	-0.400849	0.0914033	364	291	458	191	311	341
Y_RNA	0.7574124	-0.400849	0.0914033	364	291	458	191	311	341
Y_RNA	0.7574124	-0.400849	0.0914033	364	291	458	191	311	341
Y_RNA	0.7574124	-0.400849	0.0914033	364	291	458	191	311	341
Y_RNA	0.7574124	-0.400849	0.0914033	364	291	458	191	311	341
Y_RNA	0.7574124	-0.400849	0.0914033	364	291	458	191	311	341
Y_RNA	0.7574124	-0.400849	0.0914033	364	291	458	191	311	341
Y_RNA	0.7574124	-0.400849	0.0914033	364	291	458	191	311	341
Y_RNA	0.7574124	-0.400849	0.0914033	364	291	458	191	311	341
Y_RNA	0.7574124	-0.400849	0.0914033	364	291	458	191	311	341
Y_RNA	0.7574124	-0.400849	0.0914033	364	291	458	191	311	341
Y_RNA	0.7574124	-0.400849	0.0914033	364	291	458	191	311	341
Y_RNA	0.7574124	-0.400849	0.0914033	364	291	458	191	311	341
Y_RNA	0.7574124	-0.400849	0.0914033	364	291	458	191	311	341
Y_RNA	0.7574124	-0.400849	0.0914033	364	291	458	191	311	341
Y_RNA	0.7574124	-0.400849	0.0914033	364	291	458	191	311	341
Y_RNA	0.7574124	-0.400849	0.0914033	364	291	458	191	311	341
Y_RNA	0.7574124	-0.400849	0.0914033	364	291	458	191	311	341
Y_RNA	0.7574124	-0.400849	0.0914033	364	291	458	191	311	341
Y_RNA	0.7574124	-0.400849	0.0914033	364	291	458	191	311	341
Y_RNA	0.7574124	-0.400849	0.0914033	364	291	458	191	311	341
Y_RNA	0.7574124	-0.400849	0.0914033	364	291	458	191	311	341
Y_RNA	0.7574124	-0.400849	0.0914033	364	291	458	191	311	341
VDAC1P4	0	#NUM!	0.5781912	0	0	3	0	0	0
VDAC1P6	#DIV/0!	#DIV/0!	0.8030358	0	0	0	0	1	0
VDAC1P1	0.7525773	-0.410088	0.4770187	23	40	34	21	20	32
VDAC1P2	0.2	-2.321928	0.0893836	7	6	7	1	1	2
UBE2V2P4	#DIV/0!	#DIV/0!	0.3981469	0	0	0	4	0	0
VDAC1P8	0.4511628	-1.14828	0.0053407	65	58	92	19	47	31
UBE2V2P3	0.5	-1	0.7565533	0	1	3	1	0	1
AL137060.1	1.3888889	0.4739312	0.5407662	3	3	12	8	9	8
RTN4RL2	0.7239264	-0.466085	0.385453	35	67	61	50	25	43
TBX18-AS1	0.25	-2	0.5449396	0	2	2	0	0	1
AC090517.2	0.8205128	-0.285402	0.7895949	9	8	22	8	16	8
AC009495.3	#DIV/0!	#DIV/0!	0.5509701	0	0	0	0	3	0
AC009495.1	#DIV/0!	#DIV/0!	0.8030358	0	0	0	0	1	0
AC009495.2	0	#NUM!	0.6385096	0	2	0	0	0	0
RTN4RL1	0.4	-1.321928	0.2575733	3	11	11	4	2	4
RPL37A	1.1560803	0.2092416	0.05622	11838	14193	16146	17594	17500	13666
UNC93B4	0.6814159	-0.553392	0.4169351	30	21	62	35	20	22
UNC93B5	0.25	-2	0.5415866	0	4	0	1	0	0
AL118516.1	0.6934985	-0.528035	0.1981942	94	96	133	104	62	58
UNC93B2	3.25	1.7004397	0.252818	1	0	3	7	5	1
UNC93B3	#DIV/0!	#DIV/0!	0.8030358	0	0	0	0	0	1

IL12A-AS1	0.6	-0.736966	0.7640702	0	0	10	0	4	2
UNC93B1	1.056057	0.0786877	0.3097062	1232	1434	1544	1536	1494	1416
RPL36A	0.7826087	-0.353637	0.2585302	162	117	227	109	146	141
UNC93B8	#DIV/0!	#DIV/0!	0.8030358	0	0	0	0	1	0
UNC93B6	#DIV/0!	#DIV/0!	0.0202685	0	0	0	3	2	9
UNC93B7	1.2	0.2630344	0.8775803	2	3	0	4	2	0
AC005632.2	1.5	0.5849625	0.8175361	2	0	0	3	0	0
AC073842.2	0.5	-1	0.2926288	14	5	17	5	9	4
RPL35A	1.0616145	0.08626	0.2600256	9759	11811	13227	12968	12974	10999
CROCC	1.0906149	0.1251418	0.3240586	285	274	368	406	279	326
ESF1	1.1544586	0.2072164	0.2351083	485	217	554	443	561	446
SOGA3	0.8823529	-0.180572	0.8567278	9	6	2	0	5	10
AC025244.1	0	#NUM!	0.6436235	2	0	0	0	0	0
SOGA1	0.5899768	-0.76127	1.71E-06	1557	1830	1781	776	1083	1190
ACTG1P3	2.6666667	1.4150375	0.3614586	2	0	4	0	2	14
ACTG1P1	2.2222222	1.1520031	0.3806456	1	6	2	3	15	2
OR2A42	#DIV/0!	#DIV/0!	0.3597364	0	0	0	0	0	5
PTGDS	4	2	0.0635658	2	6	3	23	21	0
LMAN1L	0	#NUM!	0.2919745	1	0	5	0	0	0
AC080023.1	0.3333333	-1.584963	0.0765059	18	7	23	3	10	3
AC080023.2	1.3428571	0.4253058	0.5286299	15	11	9	9	19	19
RNU6-1333P	1	0	0.9984637	0	3	1	0	0	4
AC005288.1	1.1460177	0.1966293	0.2026643	322	283	299	274	364	398
LLGL1	0.8112601	-0.301764	0.0715729	1048	1285	1397	918	1085	1023
LLGL2	0.8992152	-0.153262	0.5876559	661	797	963	732	743	702
PTGES	1.1736505	0.2310029	0.022434	6171	7754	8102	8370	9179	8303
LMAN2L	1.006862	0.0098659	0.6097712	1074	1292	1423	1169	1378	1268
MGST3	1.1320161	0.1788945	0.0543479	5970	7007	7369	7479	8352	7201
MGST1	0.8571429	-0.222392	0.438221	187	160	192	122	163	177
MGST2	0.785059	-0.349127	0.204563	253	243	267	227	174	198
NMD3	1.0484848	0.068306	0.4906215	1958	1151	1841	1361	1988	1841
LCN15	0.875	-0.192645	0.9154906	0	4	4	0	5	2
LCN12	0.7868852	-0.345775	0.6876811	15	14	32	14	28	6
NTSR1	0	#NUM!	0.2605273	0	3	3	0	0	0
NTSR2	#DIV/0!	#DIV/0!	0.8030358	0	0	0	0	1	0
RPL39L	0.9335472	-0.099205	0.9065827	592	705	885	760	737	540
FMO9P	3.8	1.9259994	0.1347971	2	0	3	12	2	5
SERPINH1P1	0.8888889	-0.169925	0.9893291	1	0	8	2	0	6
FMO8P	#DIV/0!	#DIV/0!	0.8030358	0	0	0	0	1	0
RYR1	1.1061947	0.1456053	0.3335922	199	150	216	182	234	209
RYR2	0.4807692	-1.056584	0.1789721	26	15	11	11	9	5
RYR3	0.7553191	-0.404842	0.4309493	64	32	92	47	49	46
ZMYM1	1.2005495	0.2636948	0.1188261	554	309	593	435	678	635
ZMYM2	1.2523918	0.324686	0.0700712	1737	834	1819	1304	2193	2001
ZMYM3	0.8194067	-0.287348	0.0779381	2255	2877	2958	1790	2425	2414
ZMYM4	1.1745789	0.2321436	0.117921	1222	762	1281	919	1391	1525
ZMYM5	1.1185185	0.1615891	0.3943145	154	111	140	122	172	159
ZMYM6	1.4208	0.5067035	0.0037802	220	163	242	285	317	286
FMO7P	#DIV/0!	#DIV/0!	0.8030358	0	0	0	1	0	0
AC234782.2	0.8333333	-0.263034	0.9270602	1	2	3	1	0	4
AC234782.3	#DIV/0!	#DIV/0!	0.5509701	0	0	0	0	3	0
AC234782.4	#DIV/0!	#DIV/0!	0.5338029	0	0	0	0	1	2
AC234782.5	0	#NUM!	0.6840298	0	0	2	0	0	0
AC234782.1	0	#NUM!	0.3042138	3	2	0	0	0	0

NME2	0.7448276	-0.425022	0.3478265	46	45	54	22	42	44
NME3	1.6145251	0.6911099	0.066467	25	84	70	140	78	71
NME4	1.1	0.1375035	0.7748155	5	12	13	13	6	14
NME5	0.2307692	-2.115477	0.3504898	13	0	0	3	0	0
NME1	0.8374317	-0.255957	0.4809475	371	544	549	542	352	332
AC007349.1	#DIV/0!	#DIV/0!	0.6411933	0	0	0	0	0	2
RNU6-1337P	#DIV/0!	#DIV/0!	0.6554062	0	0	0	0	2	0
NME6	0.8190552	-0.287967	0.1059765	749	877	872	636	743	667
NME7	1.2491349	0.3209293	0.0734867	170	158	250	184	278	260
NME9	0.6060606	-0.722466	0.4617753	10	12	11	1	4	15
FIBIN	0.826087	-0.275634	0.9031786	3	6	14	10	9	0
DNASE1L2	1.119403	0.1627295	0.6587399	22	21	24	26	21	28
DNASE1L3	1.6382979	0.7121977	0.0245683	47	80	61	122	111	75
DNASE1L1	0.739726	-0.434937	0.2601823	82	113	97	89	64	63
FP236383.1	0	#NUM!	0.5643268	1	0	2	0	0	0
FP236383.2	1.5	0.5849625	0.7337883	2	0	2	2	0	4
LCMT2	0.7268482	-0.460274	0.0157003	403	427	455	257	376	301
LY6G6C	3.0775623	1.6217881	1.51E-10	123	87	151	461	406	244
LCMT1	1.0672844	0.0939447	0.3182009	738	852	892	912	962	775
ERBIN	1.1916747	0.2529905	0.1377799	1663	910	1559	1093	1907	1924
AC244102.1	0.6666667	-0.584963	0.9395362	0	0	3	2	0	0
LY6G5B	0.8231293	-0.280809	0.5382401	50	51	46	32	41	48
LY6G5C	2	1	0.6063488	3	2	0	2	8	0
ESPN	0.7609254	-0.394173	0.1105459	228	247	303	178	180	234
PPM1AP1	1	0	0.978278	0	0	2	0	2	0
AL096816.1	0.8	-0.321928	0.9352949	0	0	5	0	4	0
MYH15	0	#NUM!	0.0867763	5	3	1	0	0	0
MYH16	1.1818182	0.2410081	0.7701825	1	5	5	10	0	3
AC079766.1	0	#NUM!	0.2359908	1	0	6	0	0	0
KCTD1	0.6598322	-0.599829	0.0043659	327	343	403	179	309	220
KCTD2	0.8912	-0.166179	0.4386361	1024	1346	1380	1024	1210	1108
KCTD3	0.8409091	-0.249978	0.2071624	1852	1282	1926	1050	1606	1599
KCTD4	0	#NUM!	0.3626892	1	0	4	0	0	0
KCTD5	0.9182019	-0.123117	0.6280991	1518	1858	2052	1560	1762	1662
KCTD6	1.1606498	0.2149328	0.1914642	169	149	236	206	250	187
KCTD7	0.7079772	-0.498225	0.0594209	202	284	216	155	152	190
KCTD9	1.0067193	0.0096614	0.7103011	1198	904	1321	844	1331	1271
SHC1P1	#DIV/0!	#DIV/0!	0.5070017	0	0	0	3	0	0
SHC1P2	0	#NUM!	0.824326	1	0	0	0	0	0
AL513190.1	0.8461538	-0.241008	0.9417042	7	6	0	11	0	0
CSF2RB	0.9965086	-0.005046	0.740247	1072	1062	1303	894	1299	1232
AGER	0.8667279	-0.206349	0.4349059	600	724	852	666	716	504
CSF2RA	0.9437613	-0.083506	0.7833155	3168	3525	3798	2921	3593	3387
ESS2	0.9458333	-0.080342	0.8925743	691	668	801	667	740	636
MYH13	0.4736842	-1.078003	0.3757657	5	13	1	2	3	4
MYH14	1.6802817	0.7487031	5.02E-05	356	605	459	716	864	806
MYH11	0.7173913	-0.479168	0.6064473	5	21	20	1	10	22
MYH10	0.9811903	-0.027395	0.8822504	17202	15076	19610	12496	18576	19840
PSAPL1	0.4411765	-1.180572	0.2069694	15	7	12	4	5	6
RN7SL677P	#DIV/0!	#DIV/0!	0.8030358	0	0	0	1	0	0
AC069272.1	0	#NUM!	0.824326	1	0	0	0	0	0
ESR1	0.7692308	-0.378512	0.6274025	20	13	6	5	11	14
ESR2	0.8604651	-0.216811	0.6351776	62	47	63	27	61	60
AC004554.2	#DIV/0!	#DIV/0!	0.2887809	0	0	0	0	0	6

AL023775.1	2.7857143	1.4780473	0.1028852	6	6	2	7	17	15
SNRPGP2	0.9	-0.152003	0.9422623	3	4	3	3	2	4
PKMP1	0.6666667	-0.584963	0.81955	0	3	0	0	1	1
SNRPGP3	2	1	0.8147319	1	0	0	0	0	2
RNU6-767P	0.0869565	-3.523562	0.0347423	2	10	11	0	0	2
SNRPGP4	0.2727273	-1.874469	0.3351082	9	2	0	0	3	0
SNRPGP5	#DIV/0!	#DIV/0!	0.8030358	0	0	0	0	1	0
PKMP4	#DIV/0!	#DIV/0!	0.6099294	0	0	0	2	0	0
PKMP2	#DIV/0!	#DIV/0!	0.8030358	0	0	0	0	0	1
AC007683.1	6	2.5849625	0.3096061	0	0	1	5	0	1
PKMP3	#DIV/0!	#DIV/0!	0.3981469	0	0	0	4	0	0
PTGIS	0.8888889	-0.169925	0.9645994	8	3	16	5	0	19
RASA4B	0	#NUM!	0.824326	0	0	1	0	0	0
LMNTD1	2	1	0.6959447	2	0	0	4	0	0
LMNTD2	0.974359	-0.037475	0.8789253	32	15	31	47	15	14
AC022400.4	0.7777778	-0.36257	0.7989139	5	3	1	0	7	0
AC022400.5	0.8333333	-0.263034	0.7652889	30	13	35	26	19	20
AC022400.7	1.1025641	0.1408625	0.4509543	169	90	170	169	186	118
AC022400.1	1.2765957	0.3523017	0.4960638	13	17	17	17	17	26
AC022400.2	0	#NUM!	0.1851375	8	0	0	0	0	0
AC107081.1	1	0	0.9890559	0	0	1	0	1	0
AC107081.2	0.8	-0.321928	0.8232286	4	4	12	4	10	2
AL671511.1	3	1.5849625	0.5108244	2	0	0	3	3	0
AL590648.3	0	#NUM!	0.3100057	4	1	0	0	0	0
NCALD	0.978481	-0.031384	0.9286366	234	279	277	217	265	291
ERBB3	0.9556306	-0.065475	0.872937	2642	2872	3073	2153	2960	3093
ERBB4	1.3691275	0.4532568	0.1690492	56	33	60	47	72	85
MLANA	1.1	0.1375035	0.882978	1	11	8	2	8	12
ERBB2	0.8890423	-0.169676	0.392827	1602	2059	2134	1557	1832	1763
NCAM1	1.2249135	0.2926799	0.2467173	77	124	88	121	112	121
ROMO1	1.045738	0.0645215	0.449161	996	1383	1469	1535	1416	1073
NCAM2	3.1428571	1.6520767	0.1552932	4	1	2	6	4	12
FMNL3	1.2241379	0.2917661	0.5219103	15	22	21	22	24	25
FMNL1	1.1021992	0.140385	0.267604	236	222	315	298	273	281
FMNL2	1.2334205	0.3026647	0.0262386	6695	4692	6979	5335	8565	8753
NASEH2B-AS	1.5	0.5849625	0.7905556	0	3	1	0	2	4
AL138974.1	1.3392857	0.4214638	0.3572886	11	13	32	33	18	24
RNU7-194P	#DIV/0!	#DIV/0!	0.5509701	0	0	0	0	3	0
AC022784.3	1.3965517	0.481869	0.3126152	16	17	25	19	27	35
AC022784.4	3	1.5849625	0.6944949	0	0	1	0	3	0
AC022784.1	0.979351	-0.030102	0.8012502	827	885	1000	966	888	802
AC022784.7	1.25	0.3219281	0.8134029	0	0	4	2	0	3
AC024941.2	#DIV/0!	#DIV/0!	0.8030358	0	0	0	0	0	1
AC022784.8	0.2	-2.321928	0.1679176	2	10	3	0	3	0
ALOX15P2	3.5	1.8073549	0.4081888	0	0	2	7	0	0
AC022784.6	0.5	-1	0.560615	5	2	5	3	0	3
AC067817.2	1.6666667	0.7369656	0.7011525	2	0	1	5	0	0
RNU7-193P	#DIV/0!	#DIV/0!	0.4737294	0	0	0	0	4	0
AC135050.4	#DIV/0!	#DIV/0!	0.8030358	0	0	0	1	0	0
AC135050.5	0.5185185	-0.947533	0.3976768	8	8	11	0	4	10
AC135050.6	0.7670455	-0.382616	0.2128605	151	138	239	133	149	123
AC135050.7	2.6666667	1.4150375	0.2961731	1	4	1	3	7	6
MPPE1	0.6095764	-0.714121	0.0076001	182	178	183	77	151	103
LYPD2	2	1	0.1952529	8	14	3	13	18	19

RN7SL258P	#DIV/0!	#DIV/0!	0.8030358	0	0	0	0	0	1
LYPD3	1.5265797	0.6103029	5.03E-06	572	670	752	1024	1068	952
LYPD4	0	#NUM!	0.6840298	0	0	2	0	0	0
LYPD5	2.9921875	1.5812006	3.25E-06	48	52	28	144	131	108
LYPD6	0.6896552	-0.536053	0.4856525	19	24	15	8	5	27
DEDD2	1.2443247	0.315363	0.0284902	723	961	915	1127	1207	900
LYPD8	0.0588235	-4.087463	0.0425801	2	3	12	0	0	1
AGMO	0.744898	-0.424885	0.4375431	36	37	25	9	45	19
JAG2	1.4365482	0.5226064	0.0602711	49	61	87	98	77	108
JAG1	1.0918919	0.12683	0.4225269	247	231	262	188	330	290
AC007395.1	#DIV/0!	#DIV/0!	0.6554062	0	0	0	0	2	0
AGO3	1.0371486	0.0526226	0.5889681	339	280	377	261	422	350
AGO4	0.9029304	-0.147313	0.5735003	577	429	632	373	568	538
AGO1	1.1235079	0.1680102	0.1573809	2155	2043	2085	1750	2652	2657
AGO2	0.8286489	-0.271167	0.1907041	1464	1278	1314	705	1234	1422
NMT1	0.8867567	-0.17339	0.2917303	4708	5246	5888	4245	4985	4818
NMT2	0.9699042	-0.044086	0.9680327	237	201	293	175	276	258
AC018628.1	1.28125	0.357552	0.4659742	22	20	22	22	42	18
AC099567.1	1.5	0.5849625	0.8194094	0	0	2	0	0	3
JAK2	1.2787879	0.354777	0.2633245	62	44	59	46	76	89
JAK3	1.3223684	0.4031242	0.1930645	54	45	53	51	75	75
AC137055.1	0	#NUM!	0.2394948	0	0	8	0	0	0
JAK1	1.0502618	0.070749	0.4950723	1919	1683	2128	1325	2269	2424
AC114776.1	#DIV/0!	#DIV/0!	0.6099294	0	0	0	2	0	0
RN7SL255P	#DIV/0!	#DIV/0!	0.8030358	0	0	0	0	0	1
SDHAF2	0.9357143	-0.09586	0.9077913	88	97	95	92	77	93
SDHAF3	1.0887255	0.1226402	0.2058488	675	550	815	665	829	727
LINC00969	0.7278846	-0.458218	0.0379919	343	277	420	239	248	270
AGPS	1.028375	0.0403664	0.5375503	3230	2578	3355	2361	3575	3487
SDHAF1	0.8503095	-0.23394	0.4561715	436	634	707	591	506	414
LINC00963	1.1098039	0.1503048	0.2687451	264	378	378	341	398	393
AL157902.2	2.3333333	1.2223924	0.5643857	2	0	1	0	3	4
SDHAF4	0.9884393	-0.016776	0.8892112	54	59	60	65	56	50
LINC00960	1.44	0.5260688	0.4929546	2	11	12	11	18	7
TP53I13	0.8933915	-0.162636	0.5418268	466	523	615	479	510	444
SPESP1	1.1428571	0.1926451	0.8264057	1	3	3	8	0	0
AC135050.1	0.8333333	-0.263034	0.9069654	4	16	28	24	5	11
AC135050.3	1.6666667	0.7369656	0.8256482	0	3	0	0	5	0
LGALS9DP	0	#NUM!	0.6436235	2	0	0	0	0	0
AGR3	4	2	0.5810589	0	0	1	0	4	0
LINC00997	1.4162162	0.5020415	0.0808005	54	53	78	95	64	103
LINC00992	1.0504587	0.0710195	0.539649	120	129	187	147	157	154
TP53I11	0.8648649	-0.209453	0.8730345	10	9	18	12	13	7
JAM2	0.6071429	-0.719892	0.5918538	1	13	14	8	9	0
JAM3	1.2019583	0.2653868	0.0040435	3642	3719	4384	3924	5309	4884
DCBLD1	0.9549663	-0.066478	0.9307452	802	679	895	591	764	914
DCBLD2	0.8149321	-0.295248	0.1452443	845	625	740	447	651	703
AC009948.2	0.8235294	-0.280108	0.9166041	6	3	8	10	2	2
AC009948.3	1.5	0.5849625	0.6399871	2	1	3	5	2	2
AC009948.1	1.2629247	0.3367686	0.1209862	257	144	276	185	362	308
AGRN	1.1414128	0.1908207	0.0528541	13290	16559	16441	15382	18338	19116
AC009948.4	1	0	0.9667675	4	0	0	0	4	0
AC009948.5	0.7142857	-0.485427	0.8222107	7	0	0	0	0	5
THAP9-AS1	1.23603	0.3057138	0.0332345	870	531	997	987	1034	943

)KFZP434A06	#DIV/0!	#DIV/0!	0.8030358	0	0	0	0	0	1
AC108161.1	#DIV/0!	#DIV/0!	0.8030358	0	0	0	0	1	0
RNU6-343P	0.75	-0.415037	0.8950223	0	1	3	0	1	2
AC105460.2	0.3333333	-1.584963	0.5506802	6	0	0	0	0	2
AL096711.1	0	#NUM!	0.6436235	2	0	0	0	0	0
AL662907.1	0.8	-0.321928	0.8566238	2	8	10	9	4	3
AL096711.2	0	#NUM!	0.1212581	7	0	2	0	0	0
AL662907.2	0.3684211	-1.440573	0.1531832	10	7	21	4	2	8
JAML	0	#NUM!	0.4460243	4	0	0	0	0	0
AP001150.1	#DIV/0!	#DIV/0!	0.3597364	0	0	0	0	0	5
AC069234.1	2	1	0.814195	0	1	0	0	0	2
KHDC1	0.7758913	-0.366073	0.2836229	169	208	212	198	136	123
KHDC4	0.8225029	-0.281907	0.1332957	1361	1160	1834	1084	1214	1284
AC069234.4	0.5	-1	0.81753	2	0	0	0	0	1
AC069234.5	0.1	-3.321928	0.1760855	0	4	6	1	0	0
AC009802.1	#DIV/0!	#DIV/0!	0.5230806	0	0	0	1	2	0
AC004263.2	0.8333333	-0.263034	0.9225986	2	1	9	0	1	9
SH3BP5L	0.9208243	-0.119002	0.6689656	978	1331	1379	1001	1175	1220
RNU6-344P	#DIV/0!	#DIV/0!	0.8030358	0	0	0	0	1	0
AC024597.1	#DIV/0!	#DIV/0!	0.8030358	0	0	0	1	0	0
ETFA	0.8837301	-0.178322	0.3137578	3028	3320	3775	2833	3312	2801
ZBTB20-AS5	0	#NUM!	0.6385096	0	2	0	0	0	0
ETFB	0.8118132	-0.30078	0.3350765	197	251	280	231	179	181
ADIRF-AS1	1.2106703	0.275806	0.078082	202	214	315	308	303	274
AL161725.1	0.5	-1	0.5434752	5	1	6	0	2	4
AL161725.2	3	1.5849625	0.6552898	1	0	0	0	2	1
LINCR-0001	0.0666667	-3.906891	0.0542397	3	3	9	0	0	1
ETF1	0.9573567	-0.062871	0.9321047	13165	11023	14552	10422	13848	12818
AGXT	1	0	0.9094265	0	0	3	3	0	0
AC007405.1	0.6578947	-0.604071	0.3288365	19	26	31	8	23	19
AC007405.3	0.9279279	-0.107915	0.9141752	21	55	35	35	25	43
AC010197.1	#DIV/0!	#DIV/0!	0.8030358	0	0	0	0	0	1
AL590542.1	0.7727273	-0.371969	0.5566021	28	19	19	6	29	16
NNAT	0.5935252	-0.752619	0.0262789	105	75	98	47	59	59
ACVR2B-AS1	1.1594203	0.2134036	0.6486403	29	22	18	24	36	20
10-Sep	1.4550499	0.5410687	6.05E-05	1571	1169	1765	1656	2517	2382
11-Sep	1.4352289	0.5212808	0.0003028	8008	5896	7919	7232	11899	12190
12-Sep	0.4615385	-1.115477	0.3004026	12	8	6	6	4	2
14-Sep	1.0216963	0.0309664	0.6366131	326	324	364	291	394	351
AC024451.2	0.8571429	-0.222392	0.8556726	17	3	15	7	8	15
AC024451.3	#DIV/0!	#DIV/0!	0.5509701	0	0	0	0	3	0
AC024451.4	0.5	-1	0.5298554	1	7	6	0	7	0
AC010207.1	1.0984456	0.1354634	0.5534436	58	53	82	53	71	88
AL355480.1	0	#NUM!	0.5781912	0	0	3	0	0	0
AL355480.4	#DIV/0!	#DIV/0!	0.5070017	0	0	0	3	0	0
ANPEP	1.3470483	0.4298016	0.035799	162	186	211	290	281	182
C6orf106	0.846443	-0.240515	0.1156695	3281	3787	3840	2517	3455	3261
AC046168.1	2.0987654	1.0695409	0.0105735	22	36	23	68	44	58
AC046168.2	2.5	1.3219281	0.633036	2	0	0	0	3	2
C6orf120	0.9187742	-0.122218	0.599675	1092	989	1280	847	1131	1110
AP000561.1	4	2	0.1152108	3	1	1	9	8	3
AL117335.1	2	1	0.807847	1	0	0	0	1	1
ROPN1	0.7142857	-0.485427	0.862404	3	0	4	3	2	0
AC006077.2	3.1428571	1.6520767	0.0791374	6	1	7	5	29	10

DECR2	0.9811558	-0.027446	0.8443679	238	263	295	275	269	237
DECR1	0.9036735	-0.146127	0.6323822	366	396	463	371	370	366
NDUFAF8	0.9051144	-0.143828	0.6859294	820	1045	1107	1013	983	694
NDUFAF6	0.7871012	-0.345379	0.0790934	422	461	559	338	449	348
NDUFAF7	1.0766962	0.1066112	0.2715912	537	524	634	547	708	570
AC005831.1	1	0	0.9314762	6	2	5	7	4	2
NDUFAF4	0.8379501	-0.255064	0.191647	728	634	804	583	686	546
NDUFAF5	0.7697715	-0.377498	0.2052226	188	165	216	162	125	151
NDUFAF2	0.9951235	-0.007052	0.657489	942	939	1195	1067	1054	940
NDUFAF3	0.7753456	-0.367089	0.2234702	187	335	346	244	217	212
NDUFAF1	0.9654219	-0.050769	0.8930845	401	427	618	452	505	439
SPAG16	#DIV/0!	#DIV/0!	0.32239	0	0	0	1	4	0
SPAG17	1.2625	0.3362834	0.4338015	38	19	23	33	37	31
MTRNR2L5	#DIV/0!	#DIV/0!	0.8030358	0	0	0	0	1	0
SPATC1L	2.3333333	1.2223924	0.519096	6	0	0	0	7	7
MTRNR2L8	#DIV/0!	#DIV/0!	0.5371397	0	0	0	0	0	3
MTRNR2L3	2	1	0.7782384	0	0	1	2	0	0
MTRNR2L4	0.5714286	-0.807355	0.7244452	4	0	3	1	0	3
PPP3CA	1.1744344	0.2319662	0.0385928	2785	2205	3099	2443	3443	3614
PPP3CB	1.0655914	0.0916543	0.3064904	957	805	1028	814	1153	1006
PPP3CC	1.025878	0.0368592	0.7323939	148	196	197	146	237	172
C6orf163	1.1818182	0.2410081	0.5793855	37	5	35	32	21	38
AC084864.1	0.4	-1.321928	0.563105	0	1	9	0	4	0
CTTNBP2NL	1.0601244	0.0842336	0.5033813	567	407	473	394	554	586
ARHGEF9	1.5844156	0.6639508	0.0110705	104	55	149	138	188	162
AL391684.1	1.0194175	0.027745	0.8400296	36	15	52	29	52	24
AL596325.1	0	#NUM!	0.4217812	0	0	5	0	0	0
AL596325.2	1.5454545	0.6280312	0.6263778	3	0	8	0	7	10
AC097263.1	1	0	0.9726246	7	0	1	4	2	2
ARHGEF3	0.829469	-0.26974	0.2017244	473	373	491	286	419	404
ARHGEF4	0.8724203	-0.196905	0.5354063	162	181	190	155	160	150
ARHGEF1	1.0256964	0.0366038	0.4944092	1153	1472	1539	1479	1438	1354
ARHGEF2	0.8475959	-0.238552	0.1329746	2361	2834	3020	1975	2518	2470
ARHGEF7	0.9095343	-0.1368	0.4842131	1986	2112	2258	1577	2027	2177
ARHGEF5	0.8470588	-0.239466	0.2264582	476	473	581	384	482	430
UQCRHP1	0	#NUM!	0.3414317	0	2	3	0	0	0
ARHGEF6	1.5789474	0.6589631	0.4397922	11	1	7	13	11	6
UQCRHP2	#DIV/0!	#DIV/0!	0.6411933	0	0	0	0	0	2
AHCY	0.8764394	-0.190274	0.3582973	6347	8089	8751	6908	7053	6361
COL6A4P1	1.3461538	0.4288433	0.6472914	7	11	8	1	12	22
COL6A4P2	#DIV/0!	#DIV/0!	0.8030358	0	0	0	0	1	0
AC012354.2	0.5	-1	0.6946839	0	4	0	0	1	1
C6orf141	1.0409483	0.0578984	0.6190127	170	109	185	141	181	161
SPRYD3	0.8960739	-0.15831	0.5313654	498	583	651	502	546	504
SPRYD4	0.778626	-0.360998	0.0668636	403	511	527	337	407	378
SPRYD7	0.9495733	-0.074649	0.9258527	725	834	1019	744	894	810
AP005120.1	0	#NUM!	0.824326	1	0	0	0	0	0
AC131235.3	0.8205128	-0.285402	0.7575747	7	25	7	12	6	14
RP1L1	2	1	0.5610304	1	2	1	1	3	4
AC131235.4	1.4545455	0.5405684	0.6504747	5	5	1	4	4	8
AC131235.1	0.8695652	-0.201634	0.8740828	7	9	7	7	5	8
AC131235.2	0	#NUM!	0.824326	0	1	0	0	0	0
NDUFAB1	0.9438623	-0.083352	0.9084549	2372	2580	3064	2617	2716	2233
C6orf136	0.9206049	-0.119346	0.835312	320	303	435	358	352	264

C6orf132	0.7644394	-0.387526	0.0211308	805	833	1011	666	701	658
CELSR1	0.7808429	-0.356896	0.0229717	5381	6195	6078	3541	4827	5417
CELSR2	0.6998543	-0.514873	0.0018798	1233	1491	1394	753	1025	1104
ETS1	0.6470588	-0.628031	0.6473855	1	11	5	5	2	4
CELSR3	0.6626263	-0.593733	0.043078	116	185	194	85	113	130
ETS2	1.1787968	0.2373151	0.0168648	1444	1547	1763	1572	2019	2013
LINC00939	0.8	-0.321928	0.6520439	33	14	63	16	41	31
POTEKP	1.3423423	0.4247527	0.2625513	33	32	46	30	47	72
GNPTAB	0.9799453	-0.029227	0.9745651	1742	1061	1585	905	1694	1701
LINC00937	0.6944444	-0.526069	0.5080892	28	13	31	15	6	29
AC005487.1	0	#NUM!	0.6436235	2	0	0	0	0	0
AC073115.1	0	#NUM!	0.2506258	0	4	2	0	0	0
AC112722.1	1.0588235	0.0824622	0.8678605	4	5	8	8	8	2
AC073115.2	7	2.8073549	0.1514753	1	0	1	0	1	13
AL034418.1	0.25	-2	0.5682537	0	1	3	0	1	0
SLFN12	0.4612676	-1.116324	0.0002119	225	176	167	49	98	115
SLFN13	4	2	0.5580302	0	0	1	0	0	4
SLFN11	0.5	-1	0.81753	2	0	0	0	0	1
GNG12-AS1	1.3738318	0.4582054	0.2488492	53	19	35	45	50	52
LINC00926	1	0	0.853756	3	1	19	10	3	10
LINC00920	0.8624535	-0.213481	0.5976426	98	77	94	76	91	65
AC005091.1	0	#NUM!	0.6840298	0	0	2	0	0	0
LINC00921	0.75	-0.415037	0.5592648	14	24	14	5	14	20
OR52B5P	3	1.5849625	0.6199808	0	1	0	3	0	0
NDUFB10	0.9732454	-0.039125	0.8372904	2253	2846	3236	2893	2921	2298
PLGRKT	1.8340136	0.8750043	1.32E-07	226	228	281	458	487	403
AP003119.2	1.2666667	0.3410369	0.6897405	7	3	5	12	6	1
NDUFB11	1.0471019	0.0664018	0.406574	2163	2847	3185	3357	2904	2320
AP003119.3	0.7479675	-0.418953	0.5203999	52	13	58	28	30	34
PGDP1	#DIV/0!	#DIV/0!	0.5070017	0	0	0	3	0	0
SLC41A2	1.0103567	0.0148648	0.7514786	311	271	287	249	328	301
SLC41A3	0.8420586	-0.248008	0.1683502	1283	1642	1583	1187	1385	1224
SLC41A1	1.0331061	0.0469885	0.5484563	250	348	429	325	376	360
LINC00957	2.5	1.3219281	0.5242175	0	0	2	3	1	1
AC240274.1	0.9856115	-0.020909	0.9093756	133	128	156	102	147	162
LINC00958	0.7754335	-0.366925	0.0175193	1082	1123	1255	736	956	991
LINC00951	#DIV/0!	#DIV/0!	0.1690953	0	0	0	4	3	0
AC109635.6	#DIV/0!	#DIV/0!	0.8030358	0	0	0	1	0	0
AC109635.2	0.9230769	-0.115477	0.8588601	19	19	1	8	16	12
AC109635.3	0.9166667	-0.125531	0.9153369	3	5	4	1	10	0
AC109635.4	0.9396985	-0.08973	0.8963562	335	262	398	291	332	312
AHI1	0.964497	-0.052151	0.9797872	186	123	198	130	164	195
AC098583.1	#DIV/0!	#DIV/0!	0.8030358	0	0	0	0	1	0
LINC00941	1.0729167	0.101538	0.7005483	23	24	49	26	36	41
LINC00942	0.7391304	-0.436099	0.6927845	13	8	2	8	5	4
LINC00943	1	0	0.9890559	0	0	1	0	1	0
LINC00944	0.4444444	-1.169925	0.505818	4	5	0	0	3	1
RNU4-36P	0	#NUM!	0.6840298	0	0	2	0	0	0
OR52B3P	#DIV/0!	#DIV/0!	0.3831122	0	0	0	0	5	0
AC002116.2	1.1276596	0.1733316	0.7756917	22	12	13	10	26	17
NF670-ZNF69	0.8636364	-0.211504	0.8710868	20	20	26	27	6	24
AP000811.1	0.25	-2	0.140573	9	4	7	0	3	2
ETV1	1.0070922	0.0101958	0.8543945	165	103	155	96	182	148
ETV2	0.5348837	-0.902703	0.3125256	5	17	21	8	9	6

ETV3	0.7464321	-0.421917	0.0152362	925	925	1163	544	829	876
ETV4	1.17603	0.2339248	0.2891515	85	78	104	93	126	95
ETV5	0.7270408	-0.459892	0.1586365	128	100	164	98	88	99
ETV6	0.963807	-0.053184	0.9888667	516	401	575	371	560	507
ETV7	1	0	0.8903973	33	40	38	39	39	33
PTGES3L	0.8181818	-0.289507	0.7912035	6	12	4	5	3	10
OR52B1P	#DIV/0!	#DIV/0!	0.8030358	0	0	0	0	1	0
AL353572.1	1.1818182	0.2410081	0.7390628	2	0	9	9	1	3
AL353572.2	5	2.3219281	0.3675823	1	0	0	2	1	2
LINC00919	0	#NUM!	0.824326	0	0	1	0	0	0
ZBED1P1	2.48	1.3103401	0.0418736	6	6	13	18	19	25
BNIP1	0.8719632	-0.197661	0.4022317	419	511	593	412	503	413
BNIP2	1.0972193	0.1338519	0.1910175	1750	1346	1687	1473	2050	1725
PTK2B	0.9020761	-0.148679	0.4611018	1802	1881	2097	1661	1772	1781
LINC00910	0.9810127	-0.027656	0.9498894	47	51	60	37	48	70
RNU4-39P	1	0	0.9752725	3	2	2	0	7	0
NPIPB10P	0.6666667	-0.584963	0.608304	9	12	6	11	6	1
AC127521.1	0.8035714	-0.315502	0.5819942	35	39	38	36	32	22
NIPSNAP3B	0.9405941	-0.088356	0.9887588	49	8	44	32	38	25
NIPSNAP3A	0.8402695	-0.251076	0.1372424	824	787	912	610	787	723
BNIP3	1.8917599	0.919729	2.45E-20	5619	5612	6997	10739	12605	11139
AL450226.2	0	#NUM!	0.5378194	0	2	1	0	0	0
SDHAP1	0.9443059	-0.082674	0.8398182	343	402	458	284	437	415
LINC00909	0.6959459	-0.522953	0.1462734	79	94	123	71	83	52
SDHAP3	0.7843137	-0.350497	0.6203908	21	18	12	12	17	11
LINC00905	0	#NUM!	0.4374838	0	4	0	0	0	0
LINC00907	#DIV/0!	#DIV/0!	0.5371397	0	0	0	0	0	3
TSPO2	1	0	0.9890559	0	0	1	1	0	0
ERCC3	0.9349884	-0.09698	0.7261078	1930	2129	2386	1826	2184	2016
ERCC4	1.013267	0.0190144	0.8605975	260	158	185	133	236	242
ERCC1	1.150694	0.2025042	0.1222495	482	483	548	656	576	509
CTBP1-AS2	0.8470806	-0.239429	0.3135957	368	291	420	266	319	329
ERCC2	0.8695761	-0.201616	0.3060421	1180	1376	1431	1143	1265	1059
ERCC8	0.7864322	-0.346606	0.1553592	243	238	315	186	199	241
ERCC5	0.8503937	-0.233797	0.6623382	51	29	47	22	41	45
ERCC6	1.0431965	0.061011	0.6668713	542	336	511	290	590	569
AC009312.1	#DIV/0!	#DIV/0!	0.5371397	0	0	0	0	0	3
AC103792.1	#DIV/0!	#DIV/0!	0.5371397	0	0	0	0	0	3
AC018868.1	0.5	-1	0.8652929	0	0	2	0	1	0
AC018868.2	1.6666667	0.7369656	0.7264935	1	0	2	0	1	4
BNIPL	1.0212766	0.0303736	0.9519197	17	13	17	3	18	27
BX322559.1	#DIV/0!	#DIV/0!	0.5070017	0	0	0	3	0	0
AC078789.1	1	0	0.9938611	0	3	2	0	1	4
MRGPRF	0.2105263	-2.247928	0.1271361	7	7	5	3	1	0
AC007406.2	#DIV/0!	#DIV/0!	0.6099294	0	0	0	2	0	0
AC007406.3	0	#NUM!	0.6840298	0	0	2	0	0	0
MRGPRD	#DIV/0!	#DIV/0!	0.8030358	0	0	0	0	0	1
MRGPRE	0	#NUM!	0.1599361	0	4	4	0	0	0
AC007406.5	0.7103825	-0.493332	0.2647522	79	38	66	36	58	36
AC004898.1	0	#NUM!	0.6436235	2	0	0	0	0	0
CIRBP-AS1	2.1666667	1.1154772	0.2245537	5	8	5	4	7	28
LINC00898	0	#NUM!	0.824326	1	0	0	0	0	0
LUC7L	1.0754325	0.104917	0.2259052	883	885	1122	1069	1103	936
AC004908.1	0.745098	-0.424498	0.5817801	17	15	19	2	16	20

LINC00899	0.3214286	-1.63743	0.2045146	1	18	9	1	8	0
AC004908.2	0.2244898	-2.155278	0.0253795	12	20	17	3	8	0
AC004908.3	0.6666667	-0.584963	0.6929886	9	3	0	0	2	6
AL357054.1	#DIV/0!	#DIV/0!	0.8030358	0	0	0	1	0	0
LINC00893	0.3870968	-1.369234	0.204681	8	9	14	0	5	7
AL357054.2	#DIV/0!	#DIV/0!	0.8030358	0	0	0	0	1	0
LINC00894	1.2131148	0.278716	0.5640838	12	10	39	12	19	43
AL357054.4	0	#NUM!	0.824326	0	1	0	0	0	0
LINC00896	0	#NUM!	0.4460243	4	0	0	0	0	0
LINC00891	0.6	-0.736966	0.7494396	4	0	1	0	3	0
LINC00886	0.5301205	-0.915608	0.1402399	37	26	20	15	10	19
LINC00887	0.5	-1	0.5712872	3	4	1	0	1	3
LINC00888	0.9312081	-0.102825	0.9005151	166	186	244	196	185	174
LINC00882	1.1923077	0.2537566	0.7813799	17	8	1	13	12	6
LINC00884	0.5877193	-0.766801	0.0859526	61	43	124	34	55	45
LINC00885	0.5751634	-0.797956	0.0179045	116	86	104	63	57	56
SNX5P1	0	#NUM!	0.824326	0	1	0	0	0	0
AC024940.5	1.3333333	0.4150375	0.8550826	0	1	2	0	3	1
AC024940.3	5	2.3219281	0.4275856	1	0	0	0	2	3
AC024940.1	2.46875	1.3037807	0.0272538	14	6	12	23	33	23
AC024940.2	0.8461538	-0.241008	0.9181455	3	3	7	5	4	2
EIF3EP1	0.8	-0.321928	0.9079403	0	3	2	1	0	3
AC010542.5	0	#NUM!	0.824326	1	0	0	0	0	0
AC009061.2	0.875	-0.192645	0.836645	8	6	2	0	10	4
LINC00857	0.9671233	-0.048228	0.9808635	129	130	106	132	108	113
LINC00858	1.2736842	0.3490076	0.3680254	36	21	38	47	21	53
RN7SL268P	0.8571429	-0.222392	0.9309018	4	0	3	1	1	4
LINC00853	4.5	2.169925	0.273189	2	0	0	5	1	3
LINC00854	0	#NUM!	0.824326	0	0	1	0	0	0
ADAMTS7P3	2.1666667	1.1154772	0.5022704	1	5	0	1	6	6
ADAMTS7P4	0	#NUM!	0.824326	1	0	0	0	0	0
LINC00852	0.6984127	-0.517848	0.4482884	20	17	26	14	16	14
AHSG	#DIV/0!	#DIV/0!	0.8030358	0	0	0	0	0	1
AHRR	1.1115108	0.152522	0.2120218	368	356	388	360	444	432
LINC00843	0.6	-0.736966	0.5266537	11	2	12	4	4	7
AL118558.3	0.6186441	-0.692818	0.0934195	48	83	105	51	55	40
AL118558.4	0.3684211	-1.440573	0.2765079	6	10	3	1	6	0
PPP1R2	0.8046842	-0.313505	0.0691384	764	708	919	593	660	671
PPP1R7	1.0864027	0.1195589	0.1481497	2186	2342	2694	2552	2905	2389
PPP1R8	0.9700722	-0.043836	0.9267104	1453	1494	1898	1276	1707	1717
AL118558.1	0.4	-1.321928	0.3405731	7	5	3	1	1	4
LINC00877	8	3	0.025524	1	1	1	6	12	6
AC099568.2	0.6680328	-0.582009	0.1074468	83	74	87	48	49	66
WHRN	0.7867178	-0.346082	0.1482069	229	288	266	150	238	228
AL354796.1	0	#NUM!	0.5337954	0	3	0	0	0	0
AC080162.1	0.75	-0.415037	0.7279205	10	2	4	1	9	2
LINC00869	1.2336245	0.3029033	0.1413337	141	160	157	190	230	145
RN7SL265P	#DIV/0!	#DIV/0!	0.5371397	0	0	0	0	0	3
CPSF4L	0	#NUM!	0.1178492	2	0	8	0	0	0
LINC00867	#DIV/0!	#DIV/0!	0.6554062	0	0	0	0	2	0
LINC00862	0.6666667	-0.584963	0.7716577	1	2	3	0	3	1
LINC00863	0.7161572	-0.481652	0.2168886	52	88	89	38	72	54
PPP1CB	1.3574836	0.4409348	0.0001397	6763	5023	7289	7163	10048	8683
PPP1CC	1.0902419	0.1246482	0.1132105	7323	6569	7772	6732	8914	7973

AC078883.2	0	#NUM!	0.6631647	1	0	1	0	0	0
AC078883.3	0	#NUM!	0.824326	0	0	1	0	0	0
AC078883.4	0	#NUM!	0.824326	0	1	0	0	0	0
HMCES	0.8303421	-0.268222	0.1233508	2001	2455	2676	1949	2046	1927
AC078883.1	1.2125984	0.2781019	0.3785162	44	42	41	60	47	47
SSUH2	0.4444444	-1.169925	0.6008741	0	2	7	0	4	0
MORF4L1	0.9444309	-0.082483	0.7859442	10571	10017	12308	9060	11467	10541
RN7SL263P	0.9166667	-0.125531	0.9111211	1	8	3	0	5	6
MORF4L2	1.1625234	0.2172598	0.0065976	20317	19783	23940	21679	27542	25227
RNU6-354P	0	#NUM!	0.6436235	2	0	0	0	0	0
C20orf96	1.2977625	0.3760264	0.0619794	135	174	272	243	311	200
AL359845.1	1.3333333	0.4150375	0.7299668	0	0	6	8	0	0
THEM7P	0.3333333	-1.584963	0.7290749	0	0	3	0	0	1
AC139451.1	0.1428571	-2.807355	0.1472798	0	10	4	1	1	0
TPM3P8	0.25	-2	0.5537648	4	0	0	0	0	1
TPM3P9	1.3470831	0.4298388	1.85E-05	2212	1949	2627	2733	3224	3187
TPM3P6	2	1	0.0622164	11	9	25	20	31	39
Z93022.1	0	#NUM!	0.1795979	4	3	0	0	0	0
NOB1	0.7356522	-0.442904	0.0078334	1444	1758	1973	1289	1272	1246
AC012603.1	1.2	0.2630344	0.6563016	12	2	16	13	13	10
ATP5HP4	#DIV/0!	#DIV/0!	0.0610076	0	0	0	4	0	7
ATP5HP2	0.6428571	-0.63743	0.6337893	1	10	3	2	3	4
RPL35AP4	0	#NUM!	0.5387425	3	0	0	0	0	0
PLA2G12A	1.1374786	0.1858394	0.1021258	589	460	704	609	687	698
PLA2G12B	1	0	0.9890559	0	0	1	0	1	0
NOA1	1.1058533	0.1451601	0.0874958	1382	1392	1685	1587	1718	1626
LCORL	1.1196809	0.1630876	0.2484578	404	257	467	355	500	408
AC007250.1	#DIV/0!	#DIV/0!	0.5070017	0	0	0	3	0	0
AL354890.1	0.3333333	-1.584963	0.6394405	1	2	0	0	1	0
FAM136BP	1	0	0.9890559	0	0	1	0	1	0
NOD1	0.6928623	-0.529359	0.0038729	407	448	532	284	338	339
NOCT	1.1651197	0.2204782	0.0601582	1239	1426	1429	1257	1716	1797
RNU6-353P	0	#NUM!	0.6631647	1	0	1	0	0	0
AL096712.1	0	#NUM!	0.0230981	7	0	10	0	0	0
OTOGL	1.8715596	0.904241	0.0146589	47	23	39	52	71	81
AC027307.3	0.173913	-2.523562	0.0666133	4	7	12	2	1	1
AC027307.1	0.8333333	-0.263034	0.9452533	0	6	0	5	0	0
AC027307.2	0.5690516	-0.813369	0.0007249	184	195	222	99	134	109
KRTAP20-4	0	#NUM!	0.5172237	1	2	0	0	0	0
AC097512.1	#DIV/0!	#DIV/0!	0.2106225	0	0	0	4	0	2
HMCN2	2.2051282	1.1408625	0.046203	5	18	16	35	26	25
HMCN1	1	0	0.9709111	5	8	10	4	13	6
ARRDC1	1.0585912	0.0821455	0.4106329	439	523	557	633	491	484
ARRDC2	1.6398104	0.713529	0.0126287	59	69	83	73	139	134
ARRDC3	1.2368153	0.3066301	0.0338307	2536	1798	2549	1948	3406	3159
CRNKL1	1.1668167	0.2225779	0.0977934	1238	722	1373	1174	1448	1267
WFDC21P	0	#NUM!	0.824326	1	0	0	0	0	0
AC124283.4	0.9655172	-0.050626	0.9681632	16	6	7	4	7	17
AC124283.5	3	1.5849625	0.6191077	0	0	1	2	1	0
AC124283.2	1.137931	0.1864131	0.754949	10	13	6	19	3	11
AC124283.3	0.6	-0.736966	0.5351424	15	3	2	3	8	1
RUBCNL	0.7714286	-0.374396	0.6723025	8	18	9	9	8	10
AL157414.1	4.4545455	2.1552782	0.0107608	2	4	5	11	23	15
AC068491.4	0.4545455	-1.137504	0.3519704	3	6	13	2	4	4

AC068491.2	1	0	0.9890559	0	0	1	0	1	0
AC068491.3	0.5714286	-0.807355	0.7464368	0	2	5	2	2	0
SRP14-AS1	1.0854701	0.11832	0.4467605	174	206	205	169	253	213
AC097658.1	0	#NUM!	0.824326	0	1	0	0	0	0
LRRCC1	1.1349096	0.1825774	0.2975422	276	130	313	255	288	273
AC016813.1	#DIV/0!	#DIV/0!	0.8030358	0	0	0	1	0	0
STRADA	0.8726115	-0.196589	0.8415615	62	19	76	52	40	45
STRADB	0.8906728	-0.167033	0.4895367	480	393	435	355	441	369
AC005736.1	0	#NUM!	0.3608108	0	1	4	0	0	0
ZDHHC12	1.1271186	0.1726394	0.141542	929	1118	1139	1311	1244	1036
AC005736.2	6	2.5849625	0.3146264	1	0	0	4	2	0
ZDHHC13	1.1232877	0.1677274	0.1148245	707	575	762	676	825	795
ZDHHC11	0.6964286	-0.521953	0.1558857	77	72	75	51	54	51
ZDHHC16	1.1591356	0.2130493	0.0534495	710	861	974	938	1063	949
AL591767.3	0.3333333	-1.584963	0.3654016	6	1	5	2	2	0
ZDHHC17	1.1703831	0.2269808	0.1937387	699	363	687	481	789	777
ZDHHC14	0.9298867	-0.104873	0.766454	428	450	534	393	450	470
AC244090.2	1	0	0.9815391	3	0	1	0	4	0
AC246787.1	4.3333333	2.1154772	0.0683989	0	1	5	13	8	5
AIDA	1.0108161	0.0155206	0.6734341	1751	1503	1831	1295	1996	1849
FYTTD1	0.9367642	-0.094242	0.7635802	1051	798	1124	693	1078	1014
U40455.1	0	#NUM!	0.824326	1	0	0	0	0	0
ZDHHC20	1.0257573	0.0366894	0.6426645	3861	2740	3765	2417	4062	4154
GDPD1	0.825	-0.277534	0.5418873	61	55	44	36	39	57
NOL7	0.9628429	-0.054628	0.8769574	2573	2543	3523	2889	2918	2511
ZDHHC23	0.6820135	-0.552128	0.0102616	575	425	629	264	377	470
GDPD2	1.6666667	0.7369656	8.94E-05	195	194	238	392	363	290
NOL8	1.2019027	0.2653202	0.0861212	1863	968	1899	1572	2286	1827
ZDHHC24	0.8540318	-0.227638	0.375012	438	605	656	502	476	473
GDPD3	0.7564612	-0.402662	0.0816445	256	347	403	237	284	240
NOL9	0.875	-0.192645	0.2754216	1259	1115	1426	989	1216	1120
PHKA1P1	0.7393939	-0.435585	0.3247084	57	48	60	19	58	45
ZDHHC21	1.0375427	0.0531707	0.6022023	525	388	552	351	598	571
AC025580.1	0	#NUM!	0.824326	0	0	1	0	0	0
GDPD4	0.8187135	-0.288569	0.4991386	63	60	48	41	61	38
GDPD5	1.0218579	0.0311946	0.5932096	335	344	419	373	426	323
NOL3	0.8537604	-0.228097	0.3729626	455	381	600	402	462	362
AIF1	0.5	-1	0.6232156	2	2	2	1	1	1
NOL6	0.7919648	-0.336492	0.0334407	2301	2935	2729	1807	2281	2220
PPP2CA	1.0125697	0.0180212	0.4915747	5692	5346	6544	5645	6609	5549
PPP2CB	0.890693	-0.167	0.3214549	1906	1913	2155	1564	1997	1760
ZDHHC18	1.0079845	0.0114735	0.646014	1159	1493	1481	1374	1536	1256
ZDHHC19	0.2631579	-1.925999	0.1689085	5	7	7	1	4	0
AC068647.1	0	#NUM!	0.824326	0	0	1	0	0	0
AP000560.1	1.6666667	0.7369656	0.648725	2	2	2	0	3	7
OTOP1	0	#NUM!	0.5436524	2	0	1	0	0	0
AIG1	0.8544474	-0.226936	0.2920891	559	628	668	535	566	484
AC008629.1	0.8571429	-0.222392	0.9336219	5	1	1	4	2	0
AL355385.1	0	#NUM!	0.824326	0	0	1	0	0	0
NDUFA13	0.6907216	-0.533824	0.4094521	44	19	34	26	32	9
NDUFA11	1.0150754	0.0215869	0.7834044	60	64	75	74	71	57
NDUFA12	0.9942792	-0.008277	0.6734612	1643	1557	2044	1651	1997	1566
NDUFA10	0.9643811	-0.052325	0.9393091	3289	3575	4366	3355	4038	3437
AC243654.2	0	#NUM!	0.6631647	1	0	1	0	0	0

AC243654.3	5	2.3219281	0.2616004	1	0	1	0	6	4
AC243654.1	#DIV/0!	#DIV/0!	0.5371397	0	0	0	0	0	3
CNDP2	0.9262419	-0.110539	0.6744426	6162	7462	8177	6389	7159	6645
ARHGDIA	0.930905	-0.103294	0.7725673	4711	5766	6572	5251	5490	5130
ARHGDIB	0.8663881	-0.206915	0.2602521	2480	3095	3279	2445	2843	2383
RNU4-49P	0	#NUM!	0.5781912	0	0	3	0	0	0
NOM1	0.6834171	-0.549162	0.0003168	1129	1180	1472	675	1028	881
CDV3P1	0	#NUM!	0.5075476	0	0	4	0	0	0
JCAD	0.607941	-0.717997	2.32E-05	1290	1501	1415	608	966	983
NOP2	0.7590375	-0.397757	0.0029296	2568	2839	3196	1961	2354	2215
NOP9	0.7416948	-0.431103	0.0148123	1222	1510	1422	755	1107	1219
LINC00839	2.25	1.169925	0.5083526	1	3	0	8	0	1
LINC00836	3.8640577	1.9501166	1.67E-43	318	288	365	1001	1404	1347
RNU4-47P	0	#NUM!	0.5337954	0	3	0	0	0	0
AC112721.2	0.8	-0.321928	0.9014499	0	3	2	0	0	4
AC112721.1	6	2.5849625	0.3547041	0	0	1	0	3	3
NONO	0.9324115	-0.100961	0.6663155	26949	30743	33862	25315	30909	29142
LINC00824	0	#NUM!	0.6840298	0	0	2	0	0	0
BRWD1	1.1462008	0.1968598	0.1633173	1093	653	1202	851	1287	1241
BRWD3	1.1412043	0.1905571	0.2520853	1465	955	1383	888	1633	1819
SLC40A1	1.7402043	0.7992567	2.25E-11	2341	1885	2333	3158	4120	4136
AC092953.1	0	#NUM!	0.6840298	0	0	2	0	0	0
AC092953.2	2.2727273	1.1844246	0.3807302	0	10	1	12	0	13
NOS1	#DIV/0!	#DIV/0!	0.3096126	0	0	0	5	0	0
MAPK1IP1L	0.8862179	-0.174267	0.250906	5027	4963	5610	4077	5078	4670
STIP1P3	#DIV/0!	#DIV/0!	0.8030358	0	0	0	0	0	1
GPR1-AS	1.0128109	0.0183648	0.6887558	473	419	435	405	455	484
AKT1S1	0.9873713	-0.018335	0.7360861	2102	2488	2695	2518	2508	2167
AL449283.1	2.5	1.3219281	0.6749786	2	0	0	0	5	0
NOTO	0.3	-1.736966	0.420312	10	0	0	1	0	2
NOS2	5.8333333	2.5443205	7.53E-05	6	3	9	20	50	35
NOS3	1.144208	0.1943494	0.2797137	132	104	187	142	191	151
KANSL1	0.8519826	-0.231104	0.1971725	1464	1589	1764	972	1596	1536
SLFNL1	0.6323529	-0.661198	0.3351674	16	18	34	11	15	17
KANSL2	0.8459231	-0.241402	0.1636339	1116	1169	1382	996	1115	991
KANSL3	0.9300835	-0.104568	0.6594607	1607	1654	1888	1373	1726	1690
SNX6P1	0.375	-1.415037	0.5141715	3	0	5	0	2	1
AC104046.1	1	0	0.9890559	0	0	1	0	0	1
FAM91A3P	1.3333333	0.4150375	0.8377415	5	1	0	1	5	2
SAMD14	0.9166667	-0.125531	0.8974988	8	4	0	2	7	2
SAMD15	0.9716981	-0.04142	0.9303833	39	13	54	26	32	45
MPRIP	0.8429955	-0.246403	0.0902443	7346	8434	9111	5740	7803	7440
SAMD12	0.7487437	-0.417456	0.2245564	134	94	170	60	108	130
SAMD13	0.6976744	-0.519374	0.5104552	21	10	12	6	10	14
SAMD10	1.7647059	0.8194278	0.3306634	8	5	4	14	9	7
SAMD11	0.6842105	-0.547488	0.7173432	4	7	8	6	0	7
EIF3FP1	#DIV/0!	#DIV/0!	0.8030358	0	0	0	1	0	0
EIF3FP3	0.9419544	-0.086271	0.9351992	352	460	549	445	464	373
FLJ31356	1.009009	0.0129391	0.867544	31	43	37	38	30	44
ROCK1P1	1	0	0.9890559	0	1	0	1	0	0
MCM3AP-AS1	1.2103659	0.2754432	0.2655281	136	67	125	109	169	119
AL160394.1	0	#NUM!	0.6436235	2	0	0	0	0	0
PARPBP	0.8123791	-0.299775	0.1969602	350	317	367	190	307	343
AIRE	#DIV/0!	#DIV/0!	0.1500119	0	0	0	0	3	5

KLRK1	0	#NUM!	0.4217812	0	0	5	0	0	0
GPIHBP1	0	#NUM!	0.5436524	2	0	1	0	0	0
SDHCP2	2	1	0.8319548	0	1	0	0	2	0
NOX4	#DIV/0!	#DIV/0!	0.3831122	0	0	0	0	5	0
NOX5	0.5	-1	0.8652929	0	0	2	0	0	1
NOX1	0.6666667	-0.584963	0.5874614	14	7	12	13	5	4
ATF7IP2	1.2031163	0.2667761	0.0573114	672	446	679	591	783	788
DIS3L	0.8061744	-0.310836	0.0482126	982	975	1185	707	921	905
AC072039.1	0.25	-2	0.5455026	0	4	0	0	0	1
C20orf196	1.2903226	0.3677318	0.2653927	31	41	52	48	66	46
C20orf197	0.5	-1	0.6694314	6	0	0	0	3	0
C20orf194	0.9472494	-0.078184	0.9026761	412	409	506	333	393	531
AC125611.1	#DIV/0!	#DIV/0!	0.8030358	0	0	0	1	0	0
AC125611.2	1.3333333	0.4150375	0.820886	9	0	0	2	7	3
AC005104.1	1.1363636	0.1844246	0.7452152	7	16	21	7	23	20
AC125611.3	0	#NUM!	0.824326	0	0	1	0	0	0
AC005104.2	1	0	0.9699945	2	0	0	2	0	0
AC125611.4	1.5	0.5849625	0.8307205	1	0	1	0	2	1
ENO1-IT1	1.2272727	0.2954559	0.7185336	6	11	5	7	7	13
RN7SL279P	#DIV/0!	#DIV/0!	0.8030358	0	0	0	0	0	1
PROCA1	1	0	0.9034813	17	13	27	16	24	17
ZNF602P	2.25	1.169925	0.5119861	0	0	4	2	0	7
MIR600HG	1.001297	0.00187	0.8183968	247	214	310	181	336	255
SAMD4A	0.848554	-0.236922	0.183209	1322	1154	1466	868	1188	1289
LYRM9	1.1037037	0.1423529	0.6277966	53	39	43	58	60	31
SAMD4B	0.9744243	-0.037378	0.9209797	3382	3612	3993	2977	3973	3756
AL162311.1	0	#NUM!	0.824326	0	0	1	0	0	0
AL162311.2	0	#NUM!	0.824326	1	0	0	0	0	0
AL162311.3	0.9285714	-0.106915	0.8632852	12	23	7	7	21	11
LYRM1	0.9246488	-0.113023	0.7743566	281	237	265	243	261	220
LYRM2	1.049345	0.0694891	0.4379284	784	603	903	644	986	773
LYRM4	0.8793046	-0.185565	0.369508	828	1049	1114	809	959	862
LYRM7	0.9683033	-0.046469	0.9999885	1220	800	1198	758	1162	1196
MPLKIP	0.9852025	-0.021508	0.7582687	1138	1289	1425	1423	1297	1075
HS6ST1P1	3.4	1.7655347	0.0487865	11	1	3	9	16	26
MYLK2	1.0731707	0.1018796	0.7756849	11	13	17	20	14	10
AC024595.1	#DIV/0!	#DIV/0!	0.6554062	0	0	0	0	2	0
DEFB1	0	#NUM!	0.6436235	2	0	0	0	0	0
MYLK3	0.8310992	-0.266907	0.219951	470	472	550	274	476	490
MYLK4	0.1538462	-2.70044	0.1568738	4	7	2	2	0	0
MYLIP	0.7982512	-0.325085	0.0535047	1490	1579	1620	957	1303	1483
AC138625.1	2.2857143	1.1926451	0.16332	4	4	6	11	7	14
AC138625.2	0	#NUM!	0.824326	0	0	1	0	0	0
AL161629.1	#DIV/0!	#DIV/0!	0.3831122	0	0	0	0	5	0
AC025580.2	0	#NUM!	0.1599361	0	4	4	0	0	0
NPAT	0.9709855	-0.042478	0.9511485	712	434	853	453	805	683
CLTCL1	0.9518272	-0.071228	0.8995449	184	217	201	157	198	218
EVI5	0.9935162	-0.009385	0.8655872	740	492	773	429	780	783
AL096855.1	#DIV/0!	#DIV/0!	0.1221024	0	0	0	0	1	8
DEFA3	#DIV/0!	#DIV/0!	0.8030358	0	0	0	0	1	0
AL117332.1	6.25	2.6438562	0.0053541	0	5	3	24	9	17
SMARCA5-AS	#DIV/0!	#DIV/0!	0.5509701	0	0	0	0	3	0
CELF1	0.8660867	-0.207417	0.1812686	4656	4738	5205	3312	4640	4692
CELF2	1.0053749	0.0077336	0.7602222	1252	1093	1376	853	1415	1473

SCHIP1	1.1724138	0.2294818	0.7109057	25	3	30	5	25	38
CELF3	#DIV/0!	#DIV/0!	0.6411933	0	0	0	0	0	2
CELF4	0.25	-2	0.2030659	12	1	3	0	3	1
CELF5	0.9662921	-0.049469	0.9373547	64	43	71	64	57	51
MCM10	1.2241696	0.2918034	0.0269574	1700	1360	1847	1422	2279	2306
AC096677.1	0.9149798	-0.128188	0.8433868	96	56	95	68	71	87
NPC1	1.0193148	0.0275997	0.5858869	1342	1430	1577	1154	1597	1682
NPC2	1.1011827	0.1390538	0.1762939	2235	3009	3127	3273	3153	2792
AC078788.2	0	#NUM!	0.6436235	2	0	0	0	0	0
STAG3L5P	0.982906	-0.024875	0.9052032	39	27	51	34	32	49
AL031768.1	0	#NUM!	0.6631647	1	0	1	0	0	0
AL357055.3	1.1304348	0.1768778	0.7816239	1	11	11	9	6	11
PANDAR	0	#NUM!	0.824326	0	0	1	0	0	0
AC021224.1	#DIV/0!	#DIV/0!	0.5070017	0	0	0	3	0	0
AC116050.1	2.4615385	1.2995603	0.032503	20	4	15	53	23	20
AC063943.3	0.625	-0.678072	0.7935792	2	1	5	4	0	1
ZDHHC5	0.8407748	-0.250209	0.0982754	5225	6187	6192	4123	5347	5331
ZDHHC6	1.3045654	0.3835692	0.0005396	1081	930	1187	1224	1564	1384
ZDHHC7	0.7824186	-0.353987	0.0078579	3973	4452	4839	3057	3849	3472
ZDHHC8	2.2909091	1.1959202	0.0005533	43	37	30	85	93	74
ZDHHC1	2.5	1.3219281	0.0810511	4	7	9	11	27	12
ZDHHC2	1.2192029	0.2859382	0.0327186	325	318	461	404	495	447
ZDHHC3	0.9463104	-0.079615	0.8062668	2314	2808	2738	2193	2704	2541
ZDHHC4	0.9325795	-0.100701	0.7249164	1369	1641	1677	1357	1686	1328
GOLGA6L10	1.8333333	0.8744691	0.4247146	9	0	3	9	5	8
AC131571.1	#DIV/0!	#DIV/0!	0.8030358	0	0	0	0	0	1
SFRP4	0	#NUM!	0.2084189	0	3	4	0	0	0
SFRP1	0.8676761	-0.204771	0.1994448	5093	5314	5773	3527	5385	5127
SFRP2	0	#NUM!	0.5781912	0	0	3	0	0	0
AC132219.2	1.3636364	0.447459	0.637392	3	0	8	7	3	5
AL031133.1	3.5	1.8073549	0.3647213	0	0	2	3	2	2
EVPL	0.7045455	-0.505235	0.5075896	16	14	14	4	13	14
OLA1P1	1.0582011	0.0816138	0.6801952	68	61	60	69	56	75
RBM12	0.8716826	-0.198125	0.1932783	4617	4639	5138	3356	4647	4544
RIMKLA	1.239782	0.3100865	0.1750032	86	142	139	129	187	139
RIMKLB	0.800417	-0.321176	0.0863225	2612	1825	2758	1455	2035	2269
RBM10	0.9273549	-0.108807	0.7276968	3027	3649	3951	3266	3304	3285
AC132219.1	#DIV/0!	#DIV/0!	0.8030358	0	0	0	0	1	0
RBM11	1.1925676	0.254071	0.245502	73	100	123	121	132	100
C20orf24	0.8252788	-0.277046	0.2201042	413	444	488	390	381	339
C20orf27	1.306969	0.386225	0.016336	496	602	710	930	823	610
CELA1	1.0789474	0.1096245	0.7592118	13	8	17	23	12	6
MYLPF	1.1801242	0.2389387	0.2097707	204	251	189	268	268	224
GOLGA6L22	13	3.7004397	0.0581684	0	1	0	4	3	6
AL122126.1	1.1029412	0.1413558	0.6962	18	24	26	30	30	15
CFAP44-AS1	0	#NUM!	0.4521054	1	0	3	0	0	0
LHFPL3-AS2	1.6222222	0.6979715	0.103321	33	19	38	55	24	67
AP000959.1	#DIV/0!	#DIV/0!	0.8030358	0	0	0	0	0	1
AC078881.1	1.2266667	0.2947433	0.0854103	157	166	277	224	266	246
DIRC2	0.9553607	-0.065883	0.875536	1601	1613	1804	1316	1837	1641
DIRC3	2.3333333	1.2223924	0.2750784	4	4	1	8	8	5
AC136428.2	2	1	0.8319548	0	1	0	0	2	0
C20orf85	0	#NUM!	0.824326	0	1	0	0	0	0
AC027644.3	#DIV/0!	#DIV/0!	0.3981469	0	0	0	4	0	0

NPM1	1.0628705	0.0879658	0.1916388	65953	53642	80992	65886	78154	69158
CAVIN1	0.8787879	-0.186413	0.8156972	15	36	48	22	34	31
CAVIN2	0.525	-0.929611	0.3172419	7	10	23	7	7	7
CAVIN3	#DIV/0!	#DIV/0!	0.8030358	0	0	0	0	0	1
AP005264.5	0.1666667	-2.584963	0.0560012	13	7	4	0	1	3
CAVIN4	1.7183099	0.7809902	0.0160538	37	40	65	70	108	66
AC027644.1	0.4444444	-1.169925	0.2909804	17	4	6	4	7	1
NPM3	1.2064661	0.2707873	0.0283286	917	1000	1207	1384	1296	1089
AC027644.2	2.5	1.3219281	0.6056824	1	0	1	0	0	5
PPP1CA	0.8535397	-0.22847	0.2407479	4885	5891	6669	5174	5183	4533
ZDHHC9	0.8206636	-0.285137	0.0829406	1428	1670	1664	1237	1385	1286
AC005833.1	2.3333333	1.2223924	0.5167805	0	0	3	2	4	1
ENGASE	1.0306818	0.043599	0.4506819	524	522	714	601	602	611
PCDHB18P	2	1	0.4665023	5	3	0	6	8	2
SAMD9L	3.1333333	1.6476983	0.0478397	9	1	5	14	15	18
IFI44L	4.2903226	2.1010861	2.15E-08	20	17	25	70	116	80
NPPB	0.9425947	-0.08529	0.9061564	253	299	319	280	281	260
NPPC	0	#NUM!	0.6410557	1	1	0	0	0	0
NPPA	4.5	2.169925	0.3094412	2	0	0	4	0	5
PCDHB17P	0.68	-0.556393	0.1275846	81	80	64	43	59	51
ADPRHL1	0.8658854	-0.207752	0.4443496	218	266	284	210	241	214
ADPRHL2	1.0112117	0.0160851	0.5908572	660	765	894	865	796	684
LOXL1-AS1	0	#NUM!	0.1286255	0	4	5	0	0	0
THEMIS	0	#NUM!	0.3042138	3	2	0	0	0	0
RPGRIP1L	1.1889908	0.2497376	0.1827007	198	123	224	183	262	203
NPNT	0.9339623	-0.098564	0.7788322	266	357	331	247	328	316
AP002373.2	1	0	0.9890559	1	0	0	0	0	1
RNU4-14P	0	#NUM!	0.824326	0	1	0	0	0	0
C20orf144	0.3214286	-1.63743	0.1749667	10	3	15	0	5	4
C20orf141	1	0	0.9890154	4	0	0	0	0	4
RBM8A	0.9259701	-0.110962	0.759205	934	1029	1387	965	1098	1039
NPR1	0.6627907	-0.593375	0.3618567	14	38	34	21	15	21
RBM8B	1	0	0.9838634	1	0	1	0	0	2
NPR2	1.739726	0.7988601	0.0628575	21	26	26	32	40	55
NPR3	2.7142857	1.4405726	0.3102862	3	4	0	16	3	0
RNPS1P1	0.3793103	-1.398549	0.1862333	6	16	7	1	8	2
VARS2	0.9823633	-0.025671	0.7693564	483	546	672	592	529	550
PCDHB19P	4	2	0.5810589	0	0	1	0	4	0
HMGA1	1.0422404	0.059688	0.3411486	43689	55338	59447	54441	58755	51972
HMGA2	8	3	0.1811746	1	0	0	3	3	2
EVX2	1.625	0.7004397	0.6051501	0	4	4	4	8	1
AL078459.1	2.4285714	1.2801079	0.4120082	7	0	0	12	0	5
PNLIPRP1	1.1	0.1375035	0.8580574	1	4	5	4	2	5
RBM27	1.2291473	0.2976578	0.0356327	1227	732	1266	1150	1523	1291
PNLIPRP2	2	1	0.6733244	0	1	1	1	0	3
RBM28	0.9820039	-0.026199	0.8164208	2764	2413	3047	2316	3032	2728
CD164L2	1	0	0.9993879	1	4	1	1	1	4
RBM25	1.1072273	0.1469514	0.2204072	4469	2536	4839	3603	4987	4524
RBM26	1.0969124	0.1334483	0.2323114	2021	1307	2275	1635	2396	2115
HMGB2	1.1721248	0.2291262	0.0056836	9607	9477	11940	11406	13142	11816
HMGB3	1.1333157	0.1805498	0.0335189	7913	8451	10137	9877	10695	9462
HMGB1	0.9504089	-0.07338	0.8690063	12796	11965	15105	11203	14292	12394
CD200R1L	#DIV/0!	#DIV/0!	0.5230806	0	0	0	1	2	0
NPTN	0.9432303	-0.084318	0.7657572	2449	2359	2696	2058	2666	2354

UQCRFS1	0.886935	-0.1731	0.4114216	4011	4653	5098	4175	4379	3652
RBM34	0.4666667	-1.099536	0.3369797	3	10	32	11	9	1
RBM33	0.8563209	-0.223777	0.2237201	2013	1916	2154	1263	1824	2122
RBM17	1.2126841	0.2782038	0.004156	4136	4138	5507	5516	6033	5163
RBM14	0.9196577	-0.120831	0.603979	2758	2946	3295	2658	2851	2767
RBM15	1.0428082	0.0604739	0.5481455	796	706	834	565	960	911
RBM18	1.1623844	0.2170872	0.0443205	961	843	1115	945	1336	1112
RBM19	0.8498967	-0.234641	0.1411645	1374	1313	1670	1059	1402	1242
RBM20	0.4444444	-1.169925	0.4092119	2	5	11	0	6	2
RBM23	0.9233096	-0.115114	0.6567805	1915	2142	2554	1902	2218	1984
RBM22	0.9275849	-0.108449	0.609485	4162	4360	4873	3703	4561	4161
RBM47	0.9822204	-0.025881	0.8738393	9454	8636	10482	7127	10272	10665
RBM48	0.8319007	-0.265517	0.2505928	304	314	429	236	317	318
AL121872.1	#DIV/0!	#DIV/0!	0.3200852	0	0	0	0	2	3
RBM4B	0.7761798	-0.365537	0.03468	646	708	871	527	647	553
HMGCR	0.957731	-0.062308	0.8767748	4246	3343	4240	2702	4385	4242
HMGCL	1.0352152	0.0499307	0.4952274	484	604	701	585	716	551
RAB40A	0.7816092	-0.355481	0.470632	76	31	67	42	49	45
RAB40B	1.4504425	0.5364931	0.0003109	376	371	383	535	594	510
RAB40C	1.0157288	0.0225152	0.5581427	749	978	1134	933	1029	944
NPVF	0	#NUM!	0.6436235	2	0	0	0	0	0
AL353726.2	#DIV/0!	#DIV/0!	0.6099294	0	0	0	2	0	0
RBM38	0.9891169	-0.015787	0.703915	1458	1883	2264	1948	1925	1671
RBM39	1.0871824	0.120594	0.1836955	8646	6085	9712	7485	9896	9193
GOLGA6L5P	2.5	1.3219281	0.5767355	1	1	0	1	3	1
RBM41	0.8882175	-0.171015	0.5931464	451	295	578	282	460	434
AC098487.1	1.7777778	0.830075	0.3586429	4	10	4	12	16	4
RBM42	0.8478247	-0.238162	0.1988637	4163	5347	5821	4335	4534	4129
RBM45	0.8857868	-0.174969	0.5526104	381	369	432	377	342	328
RBM46	0	#NUM!	0.5781912	0	0	3	0	0	0
RBM43	0.8951049	-0.159871	0.6136875	207	167	198	147	179	186
RBM44	1.1142857	0.1561192	0.8119021	19	6	10	7	10	22
AC011037.1	0	#NUM!	0.824326	0	1	0	0	0	0
RPL7AP66	1.3621262	0.4458604	0.0847585	62	99	140	175	122	113
SDHDP6	1.3707865	0.4550039	0.2444736	26	30	33	31	46	45
MIS18A-AS1	0.8695652	-0.201634	0.9700998	5	6	12	14	6	0
RPL7AP60	0.2307692	-2.115477	0.2456433	0	11	2	0	1	2
RPL7AP64	0.5714286	-0.807355	0.6305105	4	1	9	4	3	1
EEF1DP3	0	#NUM!	0.824326	1	0	0	0	0	0
EEF1DP4	0	#NUM!	0.824326	0	0	1	0	0	0
EEF1DP1	3.5	1.8073549	0.3837746	1	0	1	3	0	4
HIPK1-AS1	1.5	0.5849625	0.7271132	3	0	3	2	7	0
AP000229.1	#DIV/0!	#DIV/0!	0.3981469	0	0	0	4	0	0
AL118556.1	1	0	0.9890559	1	0	0	0	1	0
RPL7AP50	6.1666667	2.6244909	0.0169849	0	4	2	16	16	5
RAB3IP	0.9815085	-0.026927	0.8493188	724	531	800	564	766	687
PCSK6-AS1	0.3333333	-1.584963	0.418486	3	2	4	0	3	0
RPL7AP34	1	0	0.8683658	8	0	14	14	5	3
RPL7AP30	1.8888889	0.9175378	0.1064506	9	10	17	22	18	28
RPL7AP31	#DIV/0!	#DIV/0!	0.8030358	0	0	0	0	0	1
MSTO2P	0.8020478	-0.31824	0.4783826	97	64	132	89	74	72
TSPY2	0	#NUM!	0.6840298	0	0	2	0	0	0
RPL7AP26	0	#NUM!	0.824326	0	1	0	0	0	0
COL21A1	1.3754029	0.4598543	0.0006403	2825	2085	3156	2717	4176	4201

JDP2	1.0138366	0.0198252	0.5223853	2056	2469	2919	2732	2507	2308
ASAH1	0.7787206	-0.360822	0.02599	1561	1341	1694	1112	1406	1061
ASAH2	1.8220339	0.8655498	0.0146033	40	23	55	83	76	56
RPL7AP11	1.4285714	0.5145732	0.3395026	8	20	21	25	30	15
AC018475.1	3.3333333	1.7369656	0.2955703	1	1	1	3	1	6
RPL7AP10	1.8333333	0.8744691	0.1339293	15	9	12	22	17	27
AC115220.1	0.5	-1	0.5799728	3	3	2	2	1	1
AL138733.1	1	0	0.9890559	1	0	0	0	1	0
RFWD2	1.023086	0.0329274	0.4863332	1452	1239	1554	1376	1620	1347
RFWD3	0.657532	-0.604867	3.51E-06	3763	4045	4307	2176	2879	2911
AC138866.1	3	1.5849625	0.503002	0	0	2	0	3	3
B3GAT3P1	0.0833333	-3.584963	0.1205173	0	6	6	0	1	0
AC138866.2	0.9166667	-0.125531	0.9598387	2	7	3	5	2	4
BEST2	0	#NUM!	0.1119855	7	2	0	0	0	0
BEST3	1	0	0.9741715	0	1	3	0	2	2
BEST1	0.3647059	-1.455195	0.0247563	32	23	30	14	11	6
AC138470.1	0.8571429	-0.222392	0.9709946	1	0	6	2	4	0
BEST4	0.5	-1	0.6715928	3	0	7	4	1	0
AC004264.1	0.4347826	-1.201634	0.3902265	8	0	15	2	6	2
AC004264.2	0	#NUM!	0.2883218	0	0	7	0	0	0
DISP1	0.7851986	-0.348871	0.2140468	157	186	211	143	136	156
AC233724.10	3	1.5849625	0.3350985	0	5	1	0	11	7
DISP2	0.7837838	-0.351472	0.6056703	19	19	36	11	30	17
AC233724.11	#DIV/0!	#DIV/0!	0.5070017	0	0	0	3	0	0
DISP3	0	#NUM!	0.824326	0	0	1	0	0	0
AC233724.13	0.5875	-0.767339	0.2098513	29	29	22	13	11	23
AC233724.15	1	0	0.978278	0	0	2	0	2	0
AC233724.16	1.6	0.6780719	0.7302879	3	0	2	0	0	8
AL592114.1	0	#NUM!	0.284725	6	0	0	0	0	0
AL592114.3	3.1875	1.6724253	0.0339392	4	8	4	11	20	20
CEMIP	0.7307692	-0.452512	0.3724035	62	27	67	27	38	49
EIF4E2P2	0	#NUM!	0.824326	0	0	1	0	0	0
CT45A5	0.2068966	-2.273018	0.0703028	4	8	17	0	4	2
CYP4F22	0	#NUM!	0.824326	0	1	0	0	0	0
CT45A1	0.3483755	-1.521285	3.00E-07	180	147	227	77	54	62
WEE2-AS1	0.2666667	-1.906891	0.0749339	7	12	11	2	4	2
CYP4F11	0	#NUM!	0.6436235	2	0	0	0	0	0
AC027309.2	1.1525424	0.2048198	0.4243146	61	56	60	79	60	65
AC092368.1	#DIV/0!	#DIV/0!	0.8030358	0	0	0	0	0	1
AL354794.2	2.6666667	1.4150375	0.3461185	3	2	1	1	13	2
AC092368.3	0.9632353	-0.05404	0.9958111	39	56	41	52	46	33
AC093206.1	0	#NUM!	0.5781912	0	0	3	0	0	0
ASB16-AS1	0.6656151	-0.58724	0.0601946	101	101	115	65	80	66
AL031767.1	0	#NUM!	0.6840298	0	0	2	0	0	0
CNGA1	0.25	-2	0.5358646	3	0	1	0	0	1
CNGA2	0	#NUM!	0.6436235	2	0	0	0	0	0
CNGA4	1.1363636	0.1844246	0.8123706	6	7	9	4	13	8
BET1L	0.8597194	-0.218062	0.2879411	858	1016	1120	862	905	807
CNGB3	#DIV/0!	#DIV/0!	0.0394092	0	0	0	2	1	9
CNGB1	0.8599641	-0.217652	0.4711221	178	168	211	152	188	139
AC005009.2	1.6666667	0.7369656	0.7451461	0	3	0	5	0	0
AC005009.1	0	#NUM!	0.4460243	4	0	0	0	0	0
RNU6-377P	1	0	0.9890559	1	0	0	0	0	1
RPH3AL	1.2924528	0.3701116	0.2959023	30	33	43	35	46	56

KCNJ10	0.9	-0.152003	0.9875654	2	0	8	3	1	5
KCNIP1	0.25	-2	0.6138887	0	0	4	1	0	0
KCNJ11	0.9767442	-0.033947	0.9907829	20	14	9	10	8	24
KCNIP2	1.826087	0.8687555	0.2803554	4	0	19	15	10	17
KCNJ12	0.125	-3	0.2709892	0	2	6	0	1	0
KCNIP3	0.8	-0.321928	0.9041629	3	1	1	2	0	2
KCNJ13	#DIV/0!	#DIV/0!	0.0126641	0	0	0	2	4	10
KCNIP4	0.5670103	-0.818553	0.1759319	53	21	23	15	14	26
KCNJ14	0.8270677	-0.273923	0.6281956	52	30	51	39	41	30
THAP12P7	0.7272727	-0.459432	0.6515541	14	6	24	13	14	5
KCNJ15	2.4736842	1.3066613	0.0917347	8	7	4	11	20	16
AL139811.1	4	2	0.478626	1	0	0	1	1	2
DNAJB1P1	0	#NUM!	0.824326	1	0	0	0	0	0
FAM35BP	1.7272727	0.7884959	0.4411222	3	2	6	4	7	8
RN7SL280P	0	#NUM!	0.824326	0	0	1	0	0	0
FAAP100	0.8182686	-0.289354	0.1419757	845	1145	1152	845	873	853
AC037487.3	0.25	-2	0.2845955	6	0	6	1	1	1
AC246785.1	0	#NUM!	0.0216084	4	5	5	0	0	0
AC037487.1	0.5	-1	0.6874864	6	0	0	0	0	3
AC246785.2	#DIV/0!	#DIV/0!	0.8030358	0	0	0	0	1	0
AC037487.2	#DIV/0!	#DIV/0!	0.6099294	0	0	0	2	0	0
AC005832.1	1.5	0.5849625	0.8582217	2	0	0	0	1	2
AC005832.3	3	1.5849625	0.6684577	1	0	0	0	0	3
AP005899.1	0.547619	-0.868755	0.3946682	4	14	24	1	6	16
AC005832.4	#DIV/0!	#DIV/0!	0.8030358	0	0	0	0	0	1
SSX4B	#DIV/0!	#DIV/0!	0.1199037	0	0	0	9	0	0
RPL15P18	1.125	0.169925	0.9383399	4	3	1	0	4	5
RPL15P14	0	#NUM!	0.0739098	8	1	1	0	0	0
RPL15P20	0.3333333	-1.584963	0.3865501	3	5	1	1	2	0
FAM35DP	1.1555556	0.2085866	0.7139225	16	18	11	15	22	15
AP000812.1	#DIV/0!	#DIV/0!	0.5371397	0	0	0	0	0	3
AP000812.2	1.3076923	0.3870231	0.702444	2	0	11	3	11	3
RABGAP1L	1.3088033	0.3882483	0.0179232	565	323	566	548	669	686
RAB39A	0.8858696	-0.174834	0.7014693	75	50	59	25	64	74
RAB39B	#DIV/0!	#DIV/0!	0.6554062	0	0	0	0	2	0
PARP16	0.4137168	-1.273285	1.94E-05	153	161	138	59	78	50
PARP14	3.2857143	1.716207	0.1660366	3	0	4	4	15	4
PARP12	1.5848496	0.6643459	1.22E-08	827	884	982	1304	1486	1478
PARP10	3.130742	1.6465046	6.04E-10	73	130	80	316	256	314
PARP11	0.9679487	-0.046997	0.9617425	205	153	266	138	208	258
NCEH1	0.7560427	-0.40346	0.0565005	542	635	602	287	514	544
AL389915.1	0	#NUM!	0.824326	0	0	1	0	0	0
AC145207.9	1.7272727	0.7884959	0.3071437	8	2	12	10	18	10
AL391839.1	0	#NUM!	0.824326	1	0	0	0	0	0
AL391839.2	2.5	1.3219281	0.4184705	0	2	2	2	5	3
AC145207.5	0.8275862	-0.273018	0.5585216	38	63	44	33	46	41
AC090796.1	#DIV/0!	#DIV/0!	0.6554062	0	0	0	0	2	0
AC145207.8	0.5365854	-0.89812	0.3573489	6	16	19	11	9	2
MYL6B	1.1698915	0.2263747	0.1173235	571	771	777	988	767	724
RNU4-21P	2	1	0.807847	1	0	0	0	1	1
WBP1LP2	0.6315789	-0.662965	0.6012691	7	7	5	0	12	0
RAB33A	0.3333333	-1.584963	0.6715904	3	0	0	0	1	0
RAB33B	1.1500647	0.201715	0.1803654	276	237	260	268	298	323
AC016957.2	0.2857143	-1.807355	0.4265331	5	0	2	0	1	1

RNU4-22P	0	#NUM!	0.824326	1	0	0	0	0	0
IGHVII-40-1	0	#NUM!	0.3473206	0	5	0	0	0	0
AC063944.2	#DIV/0!	#DIV/0!	0.8030358	0	0	0	0	0	1
AC063944.1	0.75	-0.415037	0.9064715	0	2	2	2	1	0
DISC1	0.7374517	-0.439379	0.2177215	107	72	80	51	69	71
AAMDC	1.1527778	0.2051144	0.4630616	30	45	69	64	62	40
NQO1	0.4748393	-1.074489	1.35E-15	5816	7155	7259	3083	3448	3075
NQO2	0.8962592	-0.158012	0.4755646	796	799	998	726	870	728
SSX2B	#DIV/0!	#DIV/0!	0.6554062	0	0	0	0	2	0
ERG28	0.9748892	-0.03669	0.859737	1597	1884	1935	1777	1784	1719
AC006566.1	#DIV/0!	#DIV/0!	0.2164877	0	0	0	4	2	0
HDLBP	1.0596255	0.0835544	0.1771855	22691	24352	28143	23397	28987	27285
CHCHD4	0.8353331	-0.259577	0.1600212	709	865	843	604	763	652
CHCHD5	1.2486631	0.3203843	0.1162584	120	98	156	175	138	154
CHCHD2	0.8892439	-0.169349	0.5241392	3881	4908	5296	4599	4383	3543
CHCHD3	0.8780348	-0.18765	0.2882244	1545	1642	2044	1333	1632	1628
CHCHD6	0.6565962	-0.606922	0.0160583	285	379	329	233	259	160
CHCHD7	0.8947194	-0.160493	0.5167	1005	886	1120	929	919	846
AC124045.1	1	0	0.9592305	0	0	6	0	5	1
CHCHD1	1.0992853	0.1365659	0.1744902	1135	1348	1435	1468	1569	1270
CD300A	1	0	0.9890559	1	0	0	1	0	0
RPL39P38	0	#NUM!	0.824326	1	0	0	0	0	0
EPS15P1	0	#NUM!	0.6840298	0	0	2	0	0	0
CD300C	#DIV/0!	#DIV/0!	0.6554062	0	0	0	0	2	0
AKNA	0.8655526	-0.208307	0.3040375	760	911	962	676	750	853
KLRB1	0	#NUM!	0.284725	6	0	0	0	0	0
CTTNBP2	1.5472637	0.6297191	0.0389915	62	51	88	60	128	123
NCF1B	3	1.5849625	0.6944949	0	0	1	0	3	0
FO393401.1	0	#NUM!	0.5643268	1	0	2	0	0	0
AC011131.1	0	#NUM!	0.6436235	2	0	0	0	0	0
AL034417.2	1.625	0.7004397	0.6691325	1	7	0	2	7	4
AL034417.3	#DIV/0!	#DIV/0!	0.8030358	0	0	0	0	1	0
KLRC3	0	#NUM!	0.824326	0	0	1	0	0	0
AL034417.4	1.2068966	0.271302	0.6082596	19	11	28	10	37	23
DSCR10	0.1666667	-2.584963	0.3723105	3	0	3	0	1	0
VTI1BP4	#DIV/0!	#DIV/0!	0.6554062	0	0	0	0	2	0
AC138230.1	1.875	0.9068906	0.5022787	4	4	0	6	6	3
RFXAP	1.0161812	0.0231577	0.6829906	272	268	387	231	367	344
COL20A1	4	2	0.5067587	0	0	1	4	0	0
RNU4-24P	#DIV/0!	#DIV/0!	0.6554062	0	0	0	0	2	0
KRTAP22-2	#DIV/0!	#DIV/0!	0.6554062	0	0	0	0	2	0
MRPL30P1	#DIV/0!	#DIV/0!	0.8030358	0	0	0	0	0	1
AC040970.1	0.8723404	-0.197037	0.8671433	25	3	19	11	12	18
CD163L1	0.5	-1	0.0032588	116	126	148	85	61	49
AC005342.1	#DIV/0!	#DIV/0!	0.8030358	0	0	0	1	0	0
RPL39P36	1.0833333	0.1154772	0.9037718	7	2	3	4	6	3
KLRG2	0.930745	-0.103542	0.8010905	533	604	769	557	649	568
STRCP1	1.0909091	0.1255309	0.7870842	7	4	11	12	2	10
KLRG1	0.8225806	-0.281771	0.6117961	47	60	79	62	40	51
RASGRF2-AS1	1.2631579	0.337035	0.7004444	10	6	3	8	5	11
TBKBP1	0.7967667	-0.327771	0.3269983	130	151	152	134	115	96
LINC00540	0.8340249	-0.261838	0.346498	219	224	280	190	181	232
FLJ31104	0.32	-1.643856	0.1974385	10	5	10	6	1	1
LINC00539	0.1111111	-3.169925	0.0284123	6	5	16	1	0	2

AKT2	0.9039411	-0.145699	0.4575922	3537	4223	4597	3432	4016	3722
;GO:0007165|	#VALUE!	#VALUE!	nucleus;GO:	4|hsa04915|h	hway|Long	e/threonine kinase 2			
AKT3	1.0587092	0.0823063	0.3350118	883	720	969	748	1003	972
LINC00534	1.1111111	0.1520031	0.8990832	1	6	2	3	0	7
AKT1	1.0070931	0.010197	0.5873728	2440	2789	2948	2523	2945	2767
)030334|regula	#DIV/0!	#DIV/0!							
y postsynaptic	#DIV/0!	#DIV/0!							
;GO:0100002|	#DIV/0!	#DIV/0!							
0507|negative	#DIV/0!	#DIV/0!							
:0007399|nerv	#DIV/0!	#DIV/0!							
ene expression;	#VALUE!	#VALUE!	rotein-conta	4|hsa04915|h	hway|Long	e/threonine kinase 1			
AL118511.1	1	0	0.9139145	4	6	10	8	5	7
LINC00535	0	#NUM!	0.6385096	0	2	0	0	0	0
AP001542.3	1.3225806	0.4033557	0.5019843	5	8	18	21	4	16
LINC00533	0.5652174	-0.823122	0.4600121	7	10	6	0	5	8
TSTD3	0.7380952	-0.438121	0.4390472	30	36	18	17	22	23
TSTD1	0.5555556	-0.847997	0.6040769	5	2	2	0	4	1
TSTD2	0.8634795	-0.211766	0.376483	653	700	676	311	731	710
RN7SL211P	0	#NUM!	0.6385096	0	2	0	0	0	0
MAFTRR	0.2142857	-2.222392	0.2271179	4	4	6	3	0	0
LINC00525	#DIV/0!	#DIV/0!	0.6317974	0	0	0	1	1	0
LINC00526	0	#NUM!	0.2219629	0	7	0	0	0	0
RNU6-302P	#DIV/0!	#DIV/0!	0.5509701	0	0	0	0	3	0
SKAP2	0.8947179	-0.160495	0.4127172	1948	1564	2035	1323	1792	1848
EXD1	0.6	-0.736966	0.7950369	2	0	3	1	0	2
EXD2	0.9936638	-0.00917	0.7203599	821	860	1002	783	945	938
TSTA3	1.0078829	0.011328	0.6346667	749	912	1003	894	1005	786
EXD3	1.0446429	0.0630098	0.5863172	103	142	203	151	178	139
LINC00562	0.6923077	-0.530515	0.7985069	0	2	11	0	3	6
CRABP2	0.5625	-0.830075	0.3833555	8	10	14	6	8	4
LINC00551	3	1.5849625	0.6238578	1	0	0	1	0	2
CDIPT-AS1	1.25	0.3219281	0.710247	2	4	6	10	0	5
AF287957.1	#DIV/0!	#DIV/0!	0.2327526	0	0	0	0	0	7
AC096577.1	0.6666667	-0.584963	0.6553461	2	8	5	0	5	5
FAM155A-IT1	#DIV/0!	#DIV/0!	0.4501902	0	0	0	0	0	4
LINC00543	0.68	-0.556393	0.5516915	16	15	19	14	2	18
ARHGAP9	0.7142857	-0.485427	0.783166	3	2	2	0	5	0
SFTA2	#DIV/0!	#DIV/0!	0.0750283	0	0	0	9	1	0
ARHGAP8	0.9022727	-0.148365	0.6928869	145	125	170	78	150	169
ARHGAP1	0.8498803	-0.234669	0.1627335	2562	3195	3429	2409	2849	2549
ARHGAP5	1.1788039	0.2373237	0.1799807	1994	1078	1844	1220	2193	2382
ARHGAP6	2.7272727	1.447459	0.1541916	3	4	4	3	9	18
ARHGAP4	0.9344262	-0.097847	0.8887133	121	117	128	123	131	88
NR2F1-AS1	1.1071429	0.1468414	0.7059027	26	35	23	27	25	41
WNT9A	0.6012187	-0.734038	8.49E-05	464	484	529	241	375	272
HIST1H3PS1	0	#NUM!	0.6436235	2	0	0	0	0	0
AC022540.1	4.3333333	2.1154772	0.2806081	2	1	0	0	11	2
AC098484.1	0.5636364	-0.827163	0.2832432	17	16	22	3	9	19
AC098484.2	1.3571429	0.4405726	0.6962426	6	4	4	1	5	13
PPARG	1.1884328	0.2490604	0.0111188	1593	1792	1975	1977	2231	2162
f blood vessel e	#VALUE!	#VALUE!	231|intracel	0|hsa04714|h	ition|Therm	ator activated receptor gamma			
AC011374.1	0.6666667	-0.584963	0.8871197	0	0	3	0	2	0
PPARA	0.7998091	-0.322272	0.1024621	1454	1388	1349	719	1238	1395
PPARD	0.8920594	-0.164788	0.3720653	1847	1904	2382	1671	1984	1816

WNT8B	3.2	1.6780719	0.2095542	3	0	2	8	4	4
SOS1-IT1	1.5138889	0.5982593	0.185047	30	12	30	35	27	47
CENPA	1.1776589	0.2359218	0.0425365	490	549	644	613	723	646
CENPB	0.8843552	-0.177302	0.4156033	1279	1638	1666	1339	1467	1247
CENPC	1.3063636	0.3855565	0.0618621	477	202	421	411	560	466
NRAS	1.0469899	0.0662475	0.4769405	3415	2702	3268	2380	3702	3744
EXOSC10	1.1615599	0.2160635	0.0188619	1915	1813	2375	2020	2694	2375
AL359258.3	1.3333333	0.4150375	0.7844136	1	5	3	0	5	7
AL591135.1	0.8759124	-0.191141	0.678105	57	45	35	26	52	42
AL359258.1	0.5	-1	0.7936757	1	0	3	2	0	0
LINC00518	1.0294118	0.0418202	0.879176	10	13	11	14	7	14
NRAP	0.2	-2.321928	0.5227064	0	0	5	1	0	0
LINC00513	2	1	0.7861378	1	0	0	1	1	0
LINC00514	2.8	1.4854268	0.2730273	1	0	4	6	3	5
RNU6-301P	#DIV/0!	#DIV/0!	0.4501902	0	0	0	0	0	4
NRAV	0.7956989	-0.329705	0.1771863	222	215	214	153	184	181
LINC00511	1.1538462	0.2064509	0.8129633	5	5	3	8	5	2
CENPT	1.0566775	0.0795352	0.2879827	952	957	1161	1054	1188	1002
CENPU	1.2626661	0.3364732	0.0169883	1880	1097	1839	1789	2233	2059
CENPV	0.2857143	-1.807355	0.4313099	1	2	4	0	2	0
CENPW	0.9578408	-0.062142	0.9629826	1518	1654	1904	1730	1683	1449
CENPX	0.9855872	-0.020945	0.7863634	866	1151	1244	1083	1185	946
COL22A1	#DIV/0!	#DIV/0!	0.6411933	0	0	0	0	0	2
WNT7B	0.8043017	-0.314191	0.0613953	944	1145	1212	794	928	933
WNT7A	1.0896861	0.1239126	0.2747259	418	535	608	631	555	515
CENPE	1.5207799	0.6048114	0.0028575	784	363	802	762	1271	931
CENPF	1.3874698	0.4724564	0.0065174	3005	1511	3353	2844	4467	3607
CENPH	0.9800203	-0.029116	0.7906615	951	831	1171	916	1066	912
CENPI	1.1386536	0.1873289	0.1541417	797	574	872	630	970	954
LINC00506	1.2571429	0.3301486	0.6716921	15	5	15	3	26	15
CENPJ	1.2201507	0.2870594	0.061013	947	540	1034	840	1220	1016
RBM12B-AS1	0.875	-0.192645	0.8617737	20	3	9	0	5	23
CENPK	1.0222772	0.0317865	0.6401584	1419	840	1377	949	1381	1387
CENPL	0.803516	-0.315601	0.0863066	643	578	713	403	561	590
LINC00501	0	#NUM!	0.6436235	2	0	0	0	0	0
ADGRL1	0.8813441	-0.182223	0.3732802	818	1029	1010	745	872	901
CENPM	0.8125503	-0.299471	0.3374088	322	484	437	421	296	293
CENPN	0.8553114	-0.225478	0.2020646	1003	970	1303	784	1034	984
CENPO	0.9204372	-0.119609	0.6006855	1174	1327	1433	1020	1280	1321
CENPP	0.8596838	-0.218122	0.306328	674	565	785	432	632	676
CENPQ	1.2095808	0.2745072	0.1044054	443	279	447	367	603	444
ADGRL2	1.1425585	0.1922681	0.1526014	1742	1267	1775	1271	2021	2174
ADGRL3	1.7713178	0.8248231	0.000875	161	197	158	180	341	393
CENPS	0.6666667	-0.584963	0.6229799	5	9	4	5	4	3
DNAH10OS	0.1	-3.321928	0.1511164	5	5	0	0	1	0
AP001052.1	0.1818182	-2.459432	0.2381643	1	6	4	2	0	0
AC116345.2	0	#NUM!	0.5781912	0	0	3	0	0	0
AC116345.3	#DIV/0!	#DIV/0!	0.5371397	0	0	0	0	0	3
BOD1L1	1.1220657	0.1661572	0.2939526	1105	608	1056	707	1176	1224
NREP	1.3628895	0.4466686	2.51E-05	1107	1090	1333	1393	1766	1652
NRF1	0.9289664	-0.106302	0.70443	695	804	852	642	776	766
AL162411.1	1.0761905	0.1059334	0.7325209	31	33	41	24	46	43
HNRNPDL	1.197703	0.2602702	0.0060241	6822	5638	8263	7829	8948	8043
G3BP1	0.9493345	-0.075012	0.803066	16788	15081	17869	12227	17353	17638

G3BP2	1.1204718	0.1641063	0.176113	3807	3145	3731	2812	4599	4559
AC009704.2	1	0	0.982714	2	2	1	0	3	2
AL161729.1	1.4	0.4854268	0.8045569	1	4	0	2	0	5
AL161729.2	0	#NUM!	0.824326	0	0	1	0	0	0
AL161729.3	2	1	0.6995813	0	0	2	0	3	1
AL161729.4	3.3043478	1.7243656	0.0184143	15	4	4	15	22	39
AL391097.2	0	#NUM!	0.824326	1	0	0	0	0	0
NRDC	1.0296117	0.0421003	0.4816653	3534	2823	3943	2744	3984	3877
AC007391.1	0.2352941	-2.087463	0.2765062	3	0	14	0	4	0
AC020633.1	1	0	0.9890559	0	0	1	0	1	0
SPRY4-IT1	0.25	-2	0.5537648	4	0	0	0	0	1
TBXAS1	#DIV/0!	#DIV/0!	0.8030358	0	0	0	0	1	0
AP001208.2	#DIV/0!	#DIV/0!	0.8030358	0	0	0	0	0	1
AF117829.1	1.3448276	0.4274212	0.0508202	90	84	145	142	147	140
LINC00582	#DIV/0!	#DIV/0!	0.3597364	0	0	0	0	0	5
SNAP25	#DIV/0!	#DIV/0!	0.8030358	0	0	0	1	0	0
SNAP23	0.8831635	-0.179248	0.2945593	2776	2638	3222	1944	2881	2802
NRGN	1.0147059	0.0210616	0.7807283	77	60	67	91	53	63
ALAD	1.0644058	0.0900482	0.1760287	4962	5422	6276	5305	6514	5914
AL157896.1	0.8888889	-0.169925	0.9560858	3	0	6	0	3	5
EXO1	0.9442031	-0.082831	0.818243	1481	1206	1686	1051	1571	1507
PPP6R1	1.0329454	0.046764	0.4144209	2965	3765	3924	3510	3777	3718
PPP6R2	0.9704342	-0.043298	0.8359574	1936	1996	2562	2149	2223	1930
PPP6R3	0.9215597	-0.117851	0.6277851	3988	2884	4079	2424	3887	3781
LINC00578	1.3333333	0.4150375	0.8465825	4	2	0	0	2	6
AC020779.2	0	#NUM!	0.6840298	0	0	2	0	0	0
EXO5	1.0652742	0.0912248	0.5162929	131	105	147	136	131	141
SNAP29	0.9080625	-0.139136	0.4835952	1561	1524	1777	1354	1626	1435
LINC00570	#DIV/0!	#DIV/0!	0.6554062	0	0	0	0	2	0
LINC00571	0.8333333	-0.263034	0.8811965	2	2	2	0	3	2
ADGRV1	0.8509197	-0.232905	0.2911767	330	353	350	251	339	289
NRG1	0.1046512	-3.25634	9.93E-05	33	18	35	0	3	6
MUC5AC	0	#NUM!	0.5781912	0	0	3	0	0	0
IMPACT	1.1345154	0.1820762	0.1244161	1105	978	1188	913	1388	1410
MYO3B	5	2.3219281	0.2273401	3	0	0	0	8	7
NRG4	0.877193	-0.189034	0.9229678	19	4	34	19	27	4
IMPAD1	0.8629044	-0.212727	0.2148845	2231	2046	2368	1427	2121	2186
MYO3A	#DIV/0!	#DIV/0!	0.8030358	0	0	0	1	0	0
LINC00567	1	0	0.9890559	1	0	0	0	1	0
SPACA3	#DIV/0!	#DIV/0!	0.5070017	0	0	0	3	0	0
SPACA4	#DIV/0!	#DIV/0!	0.6411933	0	0	0	0	0	2
LINC00563	#DIV/0!	#DIV/0!	0.1241408	0	0	0	6	2	0
SPACA6	0.7936508	-0.333424	0.6046553	18	55	53	39	32	29
LINC00565	2	1	0.6995813	0	0	2	0	3	1
SPACA9	0.856102	-0.224145	0.5765668	119	219	211	178	158	134
SNAP47	0.9018977	-0.148964	0.5427676	885	1089	1135	921	1035	848
AL590640.2	#DIV/0!	#DIV/0!	0.8030358	0	0	0	0	0	1
NRIR	0	#NUM!	0.6840298	0	0	2	0	0	0
BAHD1	0.8399703	-0.25159	0.1108041	2381	2852	2853	2050	2471	2271
HNRNPA1	1.0810409	0.1124212	0.0998592	37212	35205	45400	40400	46443	40522
LINC00598	1.3333333	0.4150375	0.699844	6	3	3	4	7	5
HNRNPA0	0.9078847	-0.139419	0.4685356	10893	13030	14062	10516	12581	11389
LINC00592	0.7142857	-0.485427	0.6800956	7	4	17	6	8	6
HNRNPA3	1.0432002	0.0610161	0.2702102	12953	11596	15011	12206	15639	13424

MYO10	1.0363557	0.0515193	0.5317187	4544	4475	4734	3440	5207	5606
AL662799.1	4.3333333	2.1154772	0.2341187	0	0	3	6	7	0
EXOG	0.8279221	-0.272433	0.2339128	316	278	330	218	260	287
HNRNPAB	0.8229664	-0.281095	0.0677194	26676	32435	35227	25002	27724	24911
MYO19	0.7820338	-0.354697	0.0248428	1448	1713	1982	1067	1507	1448
MYO16	0.5555556	-0.847997	0.5151961	3	8	7	0	9	1
MYO1D	0.7780347	-0.362094	0.0615033	581	564	585	322	503	521
MYO1E	1.2993492	0.3777893	0.0009972	1390	1521	1699	1593	2164	2233
MYO1B	1.0216456	0.0308948	0.6052726	2995	2153	2983	2094	3194	3019
MYO1C	0.9347074	-0.097413	0.734312	3581	4552	4870	3549	4332	4273
AC093151.2	1.8	0.8479969	0.5894813	0	1	4	2	6	1
MYO1A	2.5	1.3219281	0.6749786	2	0	0	0	5	0
AC093151.3	0.7142857	-0.485427	0.9037557	0	3	4	5	0	0
LINC00589	#DIV/0!	#DIV/0!	0.8030358	0	0	0	0	1	0
MYO1H	0.6875	-0.540568	0.4633785	14	18	16	5	13	15
MYO1F	0.7772512	-0.363547	0.3718885	64	56	91	42	63	59
TBATA	0	#NUM!	0.6840298	0	0	2	0	0	0
MYO1G	#DIV/0!	#DIV/0!	0.356754	0	0	0	1	1	2
NPFFR2	1.3317445	0.4133173	0.0069021	371	306	372	449	535	413
CCDC89	0.704698	-0.504923	0.3220059	32	54	63	37	46	22
AL844908.1	1.037037	0.0524674	0.964824	9	11	7	2	13	13
AL844908.2	0.3	-1.736966	0.3689593	0	9	1	0	1	2
CCDC91	1.3468927	0.4296349	0.0260607	333	179	373	353	482	357
ADGRA1	4.3636364	2.1255309	0.0106612	2	3	6	13	18	17
OPHN1	0.9685792	-0.046058	0.9711699	243	253	236	188	259	262
ADGRA2	0.775974	-0.36592	0.3090738	80	97	131	77	80	82
CCDC96	0.6923077	-0.530515	0.718943	2	4	7	1	7	1
CCDC97	1.0270383	0.0384899	0.4967676	1326	1728	1754	1600	1770	1568
CCDC92	1.0441176	0.0622843	0.7805429	43	46	47	36	49	57
CCDC93	0.9147659	-0.128525	0.6221044	1695	1319	1984	1092	1690	1790
CCDC94	0.877787	-0.188057	0.6114452	321	396	494	414	334	315
ALG8	0.9588176	-0.060672	0.9862164	1168	1266	1524	1127	1372	1296
ALG9	0.8042453	-0.314293	0.317812	157	114	153	90	110	141
ALG6	1.0640394	0.0895516	0.3866741	749	510	771	593	838	729
CCDC78	3.1666667	1.662965	0.3161409	0	6	0	1	13	5
KCNK10	9.5	3.2479275	0.0417122	1	1	0	5	9	5
KCNK12	0.7368421	-0.440573	0.5486655	24	18	34	4	16	36
ALG5	1.0500879	0.0705101	0.3495566	695	610	971	750	887	753
KCNK13	0	#NUM!	0.6436235	2	0	0	0	0	0
ALG2	0.9319253	-0.101714	0.6953266	1845	2026	2284	1733	2118	1885
MYO7B	1	0	0.9862713	2	7	2	4	4	3
ALG3	0.8893874	-0.169116	0.4385311	1737	1879	2179	1764	1780	1610
ADGRA3	0.9886855	-0.016416	0.7784859	1620	1338	1903	1265	1731	1810
ALG1	0.9552586	-0.066037	0.9908531	511	592	618	586	571	487
MYO7A	0.8864011	-0.173968	0.3360835	2991	3674	3652	2655	3173	3317
CCDC80	0.6866288	-0.542398	0.001544	647	630	660	353	488	489
ADGRB2	0.4948276	-1.015002	0.0007708	188	135	257	90	76	121
ADGRB3	0	#NUM!	0.1909345	0	6	1	0	0	0
ADGRB1	0	#NUM!	0.824326	0	1	0	0	0	0
CCDC86	0.7879425	-0.343838	0.0595814	815	991	1047	746	744	758
CCDC87	1.6	0.6780719	0.7167735	2	3	0	1	3	4
CCDC81	1.0247934	0.0353331	0.7866123	46	30	45	49	36	39
CCDC82	1.1568123	0.2101548	0.2302867	637	306	613	449	685	666
CCDC84	0.7752874	-0.367197	0.148641	580	372	788	427	483	439

AC019118.2	0.6	-0.736966	0.7728769	2	0	3	0	1	2
AC093297.2	1.4367246	0.5227835	0.0262587	125	84	194	168	247	164
SLC45A4	1.0607375	0.0850677	0.3847369	428	468	487	427	528	512
ADCYAP1R1	#DIV/0!	#DIV/0!	0.6411933	0	0	0	0	0	2
SLC45A2	0.5384615	-0.893085	0.5597234	4	2	7	0	5	2
AC019118.1	5	2.3219281	0.4650112	0	0	1	0	0	5
NRN1	1.5208333	0.6048621	0.3213141	25	8	15	14	37	22
SLC45A3	0.9677419	-0.047306	0.9952987	6	13	12	9	17	4
AP000619.1	0.8064516	-0.31034	0.7491254	7	10	14	4	12	9
SLC45A1	1.5	0.5849625	0.8331449	0	4	0	0	0	6
AP000619.3	0.5555556	-0.847997	0.690814	4	0	5	3	1	1
CEND1	0	#NUM!	0.4521054	1	0	3	0	0	0
RTBDN	1.2173913	0.283793	0.7773239	0	13	10	4	17	7
MYO5A	0.8573046	-0.22212	0.3344515	696	453	617	374	583	557
EXT1	1.0204693	0.0292328	0.5483249	606	675	722	651	686	707
EXT2	0.7738716	-0.369834	0.0184341	1964	2432	2317	1507	1801	1887
MYO5B	0.8363574	-0.257808	0.174934	918	910	1093	572	916	955
MYO5C	0.7698289	-0.37739	0.0203146	763	814	995	603	728	649
CSRNP1	0.7766943	-0.364581	0.0638558	567	698	786	537	569	487
NRP1	2.6190476	1.3890423	0.0001937	19	29	36	55	84	81
CSRNP2	0.9467224	-0.078987	0.8115941	1256	1191	1382	1004	1339	1282
NRP2	0.5665399	-0.81975	0.0003337	331	381	340	154	186	256
CSRNP3	1.4090909	0.4947647	0.2131632	56	38	38	36	91	59
ZBED6CL	0.8511236	-0.232559	0.4022029	221	233	258	208	216	182
PYROXD1	0.858804	-0.219599	0.6027077	208	104	290	128	212	177
OTUD7A	0.2962963	-1.754888	0.2287111	16	0	11	3	0	5
OTUD7B	0.8240934	-0.27912	0.1210256	796	778	825	502	728	747
AC084036.1	0.7083333	-0.4975	0.6038373	9	10	5	2	8	7
OTUD6B	0.9453901	-0.081018	0.8552308	524	356	530	341	545	447
MUC12	#DIV/0!	#DIV/0!	0.8030358	0	0	0	0	1	0
MUC15	1.9512103	0.9643693	6.38E-09	1015	632	997	1259	2038	1862
AC073359.1	0	#NUM!	0.6436235	2	0	0	0	0	0
SFTPB	0	#NUM!	0.824326	0	1	0	0	0	0
SREBF1	1.434136	0.5201818	3.80E-05	840	987	997	1331	1399	1320
SFTPD	2.1639344	1.1136568	0.0346674	5	32	24	40	49	43
AP003355.1	0	#NUM!	0.5781912	0	0	3	0	0	0
SREBF2	0.8439203	-0.244821	0.1261833	7339	9475	9429	6455	7927	7765
AC007390.2	1	0	0.9841497	12	8	10	2	8	20
PDIK1L	0.7739362	-0.369714	0.329358	165	67	144	84	116	91
CAB39	1.1828813	0.2423053	0.0373329	2841	2427	3027	2412	3729	3671
C1orf35	0.94061	-0.088331	0.9019649	1038	1284	1416	1265	1321	930
CL2L2-PABPN	1.25	0.3219281	0.8692373	2	0	2	0	2	3
FAM133DP	1.0990991	0.1363215	0.6526679	49	25	37	42	43	37
C1orf21	1.6275269	0.7026814	4.19E-09	692	713	920	1186	1344	1254
C1orf27	1.1578454	0.2114427	0.1865997	864	425	846	679	953	840
MYO9B	0.8432347	-0.245994	0.101573	6426	7389	7714	5094	6360	6700
MYO9A	0.9479686	-0.077089	0.9060029	542	299	562	265	457	608
AC125618.1	0.3333333	-1.584963	0.6638759	0	3	0	0	1	0
RN7SL209P	0	#NUM!	0.6840298	0	0	2	0	0	0
AC068152.1	0.8008915	-0.320321	0.224719	183	239	251	176	185	178
FAM133CP	2	1	0.8118689	0	0	1	0	1	1
C1orf50	1	0	0.9987363	3	5	4	2	9	1
OTUD4	1.1997799	0.2627697	0.104375	2067	1506	1878	1378	2548	2614
OTUD5	1.0269482	0.0383634	0.4721107	2430	3024	3118	2800	3242	2761

C1orf52	1.1090047	0.1492655	0.1211795	812	881	1050	1017	1056	969
CRTC2	0.8736443	-0.194882	0.4366326	601	519	724	552	573	486
C1orf53	0.71875	-0.476438	0.3667465	43	42	43	38	36	18
CRTC3	0.825842	-0.276062	0.0625454	4570	5308	5918	3529	4786	4730
C1orf54	1.2	0.2630344	0.8729088	0	3	2	1	2	3
CRTC1	0.8525565	-0.230133	0.4122286	219	291	331	231	276	210
C1orf56	1.4342105	0.5202568	0.2371652	28	27	21	51	30	28
CAAP1	1.0738368	0.1027748	0.2826533	1009	828	1129	831	1171	1183
RNU7-154P	#DIV/0!	#DIV/0!	0.2088383	0	0	0	5	0	1
COX6A1	0.893578	-0.162334	0.7151026	165	171	209	185	184	118
RN7SL208P	1	0	0.9890559	0	0	1	0	1	0
NRTN	2.4347826	1.283793	0.0182182	4	17	25	39	43	30
DFFBP1	4	2	0.1510326	0	2	2	7	4	5
FAM103A2P	1	0	0.9861959	1	3	1	0	2	3
C1orf43	1.026383	0.0375692	0.4352783	2036	2353	2661	2282	2672	2282
COX6B2	7	2.8073549	0.0919615	1	0	1	9	2	3
MUC3A	1.1542416	0.2069453	0.2641202	137	105	147	165	144	140
COX6B1	0.9379719	-0.092383	0.8798427	3879	4880	5412	4742	4623	3927
AL117339.4	0.6363636	-0.652077	0.7022326	1	3	7	0	4	3
AL117339.5	0.7272727	-0.459432	0.8246741	3	0	8	0	3	5
C1orf74	0.8616601	-0.214809	0.4714746	172	156	178	136	160	140
UBIAD1	0.8427621	-0.246803	0.2213287	710	802	892	583	642	801
DIABLO	0.6304348	-0.665581	0.1466415	64	42	78	40	52	24
PKD1P5	1.0031646	0.0045583	0.7816251	93	85	138	92	108	117
PKD1P6	0.8953488	-0.159478	0.8154656	47	39	86	33	62	59
PKD1P1	0.1666667	-2.584963	0.3862605	6	0	0	0	1	0
MTND3P10	0	#NUM!	0.824326	0	1	0	0	0	0
ADGRG3	1.2453704	0.3165749	0.2362614	57	66	93	125	79	65
ADGRG4	#DIV/0!	#DIV/0!	0.6099294	0	0	0	2	0	0
ADGRG1	0.857974	-0.220994	0.1641852	8145	9846	10025	7385	8396	8256
AANAT	1.1724138	0.2294818	0.67892	5	12	12	19	4	11
ADGRG2	0.7910448	-0.338169	0.5852282	26	28	13	15	21	17
ADGRG7	1.25	0.3219281	0.8191178	3	6	3	0	5	10
ADGRG5	2.3333333	1.2223924	0.4918498	1	1	1	6	1	0
ADGRG6	0.9580838	-0.061776	0.8998545	841	573	757	467	762	851
AC004895.1	0.9166667	-0.125531	0.9955078	11	9	16	19	10	4
C1orf61	0.5092066	-0.973677	1.51E-11	1403	1249	1747	692	747	801
CRTAP	0.7139522	-0.486101	0.0004262	3128	3592	3988	2449	2665	2531
MUC16	1.0555556	0.0780025	0.9200991	6	7	5	3	3	13
CRTAM	0	#NUM!	0.4217812	0	0	5	0	0	0
MUC19	0.7835821	-0.351844	0.5484831	52	27	55	42	32	31
AC022447.2	#DIV/0!	#DIV/0!	0.8030358	0	0	0	1	0	0
RN7SL205P	1.4	0.4854268	0.8318749	5	0	0	1	5	1
AC104534.1	1.1428571	0.1926451	0.8281558	4	8	2	9	4	3
ALPP	0.5389222	-0.891851	0.0627622	35	63	69	24	47	19
RNU7-151P	#DIV/0!	#DIV/0!	0.6099294	0	0	0	2	0	0
ALPL	1.3394231	0.4216117	0.0115971	261	312	467	508	476	409
ALPI	0.4	-1.321928	0.4565636	1	5	4	0	1	3
DEGS1	0.6660404	-0.586318	0.0009028	602	760	767	452	482	484
PYROXD2	1.2260274	0.2939912	0.3220876	47	38	61	47	63	69
DEGS2	2.5	1.3219281	0.4269978	1	5	0	1	11	3
DNAL4	0.8677196	-0.204699	0.4842452	262	337	414	288	338	253
MUC20	0.125	-3	0.2209097	3	1	4	0	1	0
DNAL1	1.0030864	0.0044459	0.856284	220	212	216	153	250	247

CYP4A22	#DIV/0!	#DIV/0!	0.6554062	0	0	0	0	2	0
ADGRE1	0.8372093	-0.25634	0.738548	20	12	11	9	17	10
AC106748.1	0	#NUM!	0.5622741	0	1	2	0	0	0
ADGRE2	1.0740741	0.1030935	0.4542733	147	125	241	154	202	195
ADGRE5	0.9908537	-0.013256	0.7783372	1147	1194	1267	1044	1322	1209
AC096921.2	0.84	-0.251539	0.8002148	9	8	8	4	9	8
MYOCD	2.025	1.0179219	0.1337369	18	4	18	8	40	33
FIGLA	0	#NUM!	0.824326	0	1	0	0	0	0
ADGRF2	0.5294118	-0.917538	0.5322328	4	9	4	6	0	3
ADGRF3	0.2121212	-2.237039	0.0350206	10	16	7	2	3	2
ADGRF1	0.8823529	-0.180572	0.8764151	5	9	3	5	7	3
ALS2	0.778777	-0.360718	0.0548769	748	691	785	428	602	702
ADGRF4	1.1441441	0.1942688	0.2972944	165	125	154	144	165	199
MYOD1	1.5953488	0.6738719	0.0136348	63	75	77	128	92	123
ADGRF5	0.9930151	-0.010112	0.8337887	299	253	307	231	306	316
OTUB1	0.9926168	-0.010691	0.7062134	1902	2435	2706	2397	2549	2045
DNAH3	0.6407767	-0.642106	0.1046096	58	80	68	30	64	38
OTUB2	1.0437828	0.0618216	0.5560021	171	160	240	176	236	184
DNAH1	0.5803571	-0.784987	0.1681744	25	45	42	26	14	25
DNAH2	0.9166667	-0.125531	0.9896002	3	1	8	2	3	6
DNAH7	1.3636364	0.447459	0.6796244	3	4	4	3	6	6
DNAH8	0.9375	-0.093109	0.9882796	7	2	7	6	9	0
DNAH5	1.0787879	0.1094112	0.6898272	50	52	63	36	79	63
DNAH6	2.2222222	1.1520031	0.3425112	2	5	2	11	0	9
ASAP3	1.1356895	0.1835685	0.0823221	2052	2526	2637	2194	3060	2940
ASB13	1.0370968	0.0525505	0.6270492	188	218	214	199	252	192
ASB14	1.8333333	0.8744691	0.3867078	3	1	8	4	10	8
PKD1L1	1.0588235	0.0824622	0.8739256	16	3	15	7	19	10
ASAP1	0.9058796	-0.142609	0.5086457	2977	2537	3092	1692	3002	3102
DNAH9	0	#NUM!	0.824326	0	1	0	0	0	0
PKD1L2	1.0550071	0.0772526	0.3424067	600	720	807	722	775	747
ASAP2	0.6490066	-0.623695	0.1963721	61	28	62	28	39	31
PKD1L3	3.5	1.8073549	0.4305471	0	0	2	0	1	6
ASB18	0.3333333	-1.584963	0.2423852	2	16	3	1	6	0
ASB15	#DIV/0!	#DIV/0!	0.8030358	0	0	0	0	0	1
ASB16	0.6075949	-0.718818	0.2889243	24	20	35	4	17	27
AC012358.1	0	#NUM!	0.355562	5	0	0	0	0	0
AC012358.2	0.9677419	-0.047306	0.9964357	23	15	24	13	20	27
DNAI1	0.7559055	-0.403722	0.5115836	58	23	46	30	55	11
AC012358.3	0.5555556	-0.847997	0.6119827	2	5	2	2	1	2
EYA1	1.3	0.3785116	0.7890924	3	4	3	0	4	9
EYA2	1.1428571	0.1926451	0.8651881	0	3	4	2	3	3
EYA3	1.0561519	0.0788174	0.361097	857	656	909	722	901	935
EYA4	1.3714286	0.4556795	0.4806917	6	16	13	11	14	23
AL137067.1	#DIV/0!	#DIV/0!	0.6099294	0	0	0	2	0	0
MUC5B	1.25	0.3219281	0.6549096	12	7	5	19	2	9
AL137067.2	1	0	0.9617562	0	0	2	0	0	2
ADGRD1	0.6	-0.736966	0.727294	2	3	0	0	3	0
OTUD1	0.9734104	-0.03888	0.9085923	250	240	375	216	321	305
OTUD3	0.7071362	-0.49994	0.0223375	352	316	411	178	274	311
AC007298.2	#DIV/0!	#DIV/0!	0.8030358	0	0	0	0	0	1
FGFRL1	0.9589041	-0.060542	0.9384081	147	135	156	106	171	143
RNU6-315P	1	0	0.9644635	3	0	0	3	0	0
CEP95	0.9746228	-0.037084	0.8400122	998	652	1266	845	1070	927

CEP97	0.9730022	-0.039485	0.9476792	347	227	352	243	351	307
LINC00412	0.4285714	-1.222392	0.5649263	3	4	0	2	1	0
COL23A1	1.1290323	0.1750867	0.7980806	10	18	3	19	8	8
LINC00402	4.5	2.169925	0.2535513	0	0	2	4	1	4
LINC00440	0	#NUM!	0.824326	0	0	1	0	0	0
LINC00441	1	0	0.9748325	8	0	1	0	5	4
AC100812.1	2.25	1.169925	0.5328396	0	0	4	0	3	6
AC103706.1	0.9375	-0.093109	0.9686879	18	10	20	12	13	20
CEP70	1.2084063	0.2731056	0.1460917	476	211	455	408	524	448
AC148477.7	0	#NUM!	0.5337954	0	3	0	0	0	0
ALX4	0.25	-2	0.6181385	0	0	4	0	0	1
CEP72	0.8602804	-0.217121	0.2740416	635	749	756	541	626	674
MCMBP	1.0701128	0.0977629	0.2072901	3378	3453	3809	3130	4209	4047
ALX1	0.9979592	-0.002947	0.8739252	128	194	168	153	212	124
CEP76	0.9146341	-0.128733	0.6848771	205	210	241	156	240	204
CEP78	1.1443644	0.1945466	0.0783591	2164	1561	2246	1867	2623	2343
DACT3-AS1	0.2857143	-1.807355	0.4577715	1	3	3	2	0	0
SNAPIN	0.8248743	-0.277754	0.1813285	598	775	814	579	699	526
RN7SL221P	5	2.3219281	0.4886016	0	0	1	0	5	0
CEP83	1.2533013	0.3257333	0.1066244	332	158	343	270	400	374
CEP85	1.2140343	0.2798092	0.0028134	3942	4178	4948	4409	5788	5668
LINC00421	1.5	0.5849625	0.7734914	0	0	2	3	0	0
CEP89	0.8250743	-0.277404	0.1341553	614	672	732	525	617	523
LINC00423	#DIV/0!	#DIV/0!	0.5070017	0	0	0	3	0	0
CEP57	1.0601607	0.084283	0.4190019	1994	1134	1975	1472	2064	1874
ATP5LP6	#DIV/0!	#DIV/0!	0.6099294	0	0	0	2	0	0
CEP55	1.3097508	0.3892924	0.008227	1190	717	1344	1208	1617	1433
CEP68	1.1106326	0.1513816	0.0744355	2295	2468	2667	2481	2971	2800
NSA2	1.0814332	0.1129446	0.1638148	4150	3363	5074	4269	5156	4187
CEP63	1.1911038	0.2522991	0.1357664	490	252	472	494	521	431
AC026422.1	0	#NUM!	0.4460243	4	0	0	0	0	0
ASCL5	1	0	0.9890559	1	0	0	0	1	0
SLCO4A1-AS1	1.8571429	0.8930848	0.5049284	1	5	1	5	4	4
AC024592.2	#DIV/0!	#DIV/0!	0.8030358	0	0	0	0	1	0
ASCL1	1	0	0.9716937	0	3	0	3	0	0
ASCL2	1.4125	0.4982509	0.0619203	81	61	98	146	99	94
NSD3	0.9482009	-0.076735	0.8616554	1839	1277	1942	1106	1761	1929
NSD1	0.7734351	-0.370648	0.056389	3621	2861	3343	1702	2580	3317
NSD2	0.9375329	-0.093059	0.705552	5836	6261	6873	4586	6382	6817
CEP41	0.9383378	-0.091821	0.8039435	654	582	629	564	613	573
RNU6-312P	3.5	1.8073549	0.4605386	0	2	0	2	5	0
CEP44	1.2868852	0.3638834	0.0420797	396	248	454	336	555	522
AC005972.1	4.5	2.169925	0.2785165	2	0	0	3	2	4
CEP19	1.0652174	0.0911479	0.6658794	62	54	68	52	63	81
AC005972.4	0	#NUM!	0.5781912	0	0	3	0	0	0
AC039056.2	2.875	1.523562	0.2735274	3	4	1	0	15	8
HMGN4	0.8915475	-0.165616	0.4018871	1174	1274	1397	1074	1293	1061
HMGN5	1.0909091	0.1255309	0.3530852	399	226	475	387	459	354
OR2L1P	0	#NUM!	0.824326	1	0	0	0	0	0
AC092718.7	1.2777778	0.353637	0.6755009	9	4	5	7	8	8
AC092718.8	1.25	0.3219281	0.6541214	9	1	10	12	3	10
HMGN2	0.9427807	-0.085006	0.823923	12150	13493	15829	12215	14334	12550
HMGN3	1.338934	0.4210848	0.0001912	1092	953	1407	1443	1663	1516
AC093390.1	1.5	0.5849625	0.7734914	0	0	2	3	0	0

HMGN1	1.0818608	0.1135149	0.0962964	9707	9123	11673	10274	11995	10731
AC092718.1	0	#NUM!	0.1151465	2	5	1	0	0	0
AC092718.2	0.2222222	-2.169925	0.2810776	4	2	3	1	1	0
LINC00494	#DIV/0!	#DIV/0!	0.5338029	0	0	0	0	1	2
AC092718.5	0.5833333	-0.777608	0.5858581	6	3	3	0	5	2
AC092718.6	1.2	0.2630344	0.8154105	3	6	6	1	11	6
AC092718.3	1.4727273	0.5584903	0.3146347	28	7	20	19	36	26
LINC00491	1.1096491	0.1501036	0.4596791	66	62	100	77	84	92
AC092718.4	1.0037175	0.0053532	0.7466807	157	122	259	179	234	127
LINC00492	#DIV/0!	#DIV/0!	0.6411933	0	0	0	0	0	2
VASH2	0.9795918	-0.029747	0.9701395	93	68	84	39	121	80
FRMD4A	0.8094463	-0.304993	0.0972327	604	574	664	388	541	562
FRMD4B	0.7441341	-0.426366	0.1136442	317	190	388	173	270	223
VASH1	0.2941176	-1.765535	0.1139395	3	22	9	2	1	7
OR2L13	0	#NUM!	0.824326	0	0	1	0	0	0
TSSK6	0.4233333	-1.240134	0.0006825	75	93	132	48	39	40
AP001207.1	0	#NUM!	0.4217812	0	0	5	0	0	0
TSSK3	0.1428571	-2.807355	0.2729746	3	4	0	0	0	1
TSSK4	0.8108108	-0.302563	0.4068932	81	78	100	60	77	73
AP001207.3	0	#NUM!	0.6631647	1	0	1	0	0	0
LINC00486	#DIV/0!	#DIV/0!	0.6482162	0	0	0	0	1	1
CCDC27	0.5	-1	0.7689504	0	0	4	0	1	1
AL136968.1	0	#NUM!	0.1983952	5	0	2	0	0	0
CCDC24	0.8912281	-0.166133	0.7579635	89	83	113	92	76	86
CCDC25	1.0433828	0.0612685	0.4171652	630	498	693	574	685	641
KRBOX4	0.9650735	-0.051289	0.9374505	420	253	415	338	350	362
KRBOX1	0	#NUM!	0.6840298	0	0	2	0	0	0
CCDC30	1.245283	0.3164737	0.5514191	27	5	21	22	27	17
CCDC32	0.7712418	-0.374745	0.2143979	143	130	186	73	143	138
CCDC33	1.2821782	0.3585968	0.2004344	63	78	61	97	81	81
AL591866.1	1	0	0.9983056	2	1	1	0	1	3
TRAPPC2	1.1125	0.1538053	0.3389906	185	142	233	168	257	198
CCDC17	0.8461538	-0.241008	0.7145242	44	26	21	30	21	26
TRAPPC3	0.9856412	-0.020865	0.7652395	1402	1772	1910	1684	1783	1544
CCDC18	1.3360825	0.4180091	0.0728983	124	114	247	140	252	256
TRAPPC1	1.0332635	0.0472082	0.5002179	1029	1406	1383	1375	1411	1159
CCDC12	1.0499024	0.0702553	0.3609224	936	1223	1428	1272	1330	1164
CCDC13	0.7966102	-0.328054	0.7910078	8	7	44	13	26	8
AMBN	0	#NUM!	0.824326	1	0	0	0	0	0
CCDC14	1.1078337	0.1477413	0.303266	1721	811	1604	1268	1727	1587
TRAPPC4	0.8342995	-0.261363	0.1882884	569	677	824	532	624	571
CCDC15	0	#NUM!	0.824326	0	0	1	0	0	0
TRAPPC5	0.78	-0.358454	0.7145668	11	21	18	20	6	13
TRAPPC8	1.0299529	0.0425784	0.6288337	875	542	920	562	927	918
NSG1	0.3943662	-1.342392	0.0006575	73	73	67	26	32	26
TRAPPC9	0.7322835	-0.449526	0.0488229	232	271	259	171	206	181
ERICD	1.7586207	0.8144443	0.2791352	6	0	23	15	17	19
CNIH1	0.9955958	-0.006368	0.6711313	2779	2332	3063	2384	2997	2757
NSG2	0.5555556	-0.847997	0.7326545	0	1	8	3	1	1
CCDC22	0.842079	-0.247973	0.3724146	628	674	699	684	548	453
CNIH2	0.9164345	-0.125896	0.8076658	216	243	259	253	200	205
CNIH3	2.25	1.169925	0.0885037	5	17	10	28	35	9
CNIH4	0.9809342	-0.027772	0.7976745	581	708	809	723	712	623
LINC00460	#DIV/0!	#DIV/0!	0.8030358	0	0	0	0	0	1

LINC00463	#DIV/0!	#DIV/0!	0.8030358	0	0	0	0	1	0
CCDC47	1.0096922	0.0139155	0.65475	4155	2993	3995	3057	4250	3944
AMD1	1.1110674	0.1519464	0.1528592	4644	3377	4692	3661	5328	5136
AC010306.1	0.7555556	-0.40439	0.6136965	27	7	11	8	19	7
IDH3B	0.98042	-0.028528	0.7755268	2524	3137	3481	3138	3058	2767
LINC00458	5.4246575	2.4395321	1.15E-13	20	21	32	107	166	123
CCDC54	0.6666667	-0.584963	0.8537226	2	0	1	0	0	2
LINC00459	#DIV/0!	#DIV/0!	0.6411933	0	0	0	0	0	2
LINC00454	#DIV/0!	#DIV/0!	0.6554062	0	0	0	0	2	0
CCDC50	0.8164957	-0.292483	0.1019816	852	703	979	600	725	744
CCDC51	0.8548114	-0.226322	0.2132014	1352	1602	1764	1272	1528	1233
IDH3A	0.7826797	-0.353506	0.1309214	457	300	467	240	345	373
LINC00456	0.95	-0.074001	0.9503434	14	12	14	7	16	15
CCDC39	0.9144737	-0.128986	0.8577577	57	35	60	31	41	67
LINC00452	0	#NUM!	0.6385096	0	2	0	0	0	0
CCDC34	1.26917	0.3438853	0.0119277	860	594	1076	1064	1137	1010
CCDC36	7	2.8073549	0.2507339	1	0	0	4	0	3
AL137796.1	4	2	0.5532124	1	0	0	0	0	4
CCDC42	1	0	0.8667136	3	0	17	8	3	9
CCDC43	0.9746622	-0.037026	0.8830227	838	636	894	716	880	712
LINC00449	1	0	0.9656845	0	2	4	0	0	6
LINC00442	#DIV/0!	#DIV/0!	0.8030358	0	0	0	0	1	0
LINC00443	0	#NUM!	0.824326	0	1	0	0	0	0
LINC00444	#DIV/0!	#DIV/0!	0.6554062	0	0	0	0	2	0
CCDC40	1.8548387	0.8912937	0.0523904	14	17	31	53	30	32
LINC00482	0.8637771	-0.211269	0.6282971	121	72	130	88	91	100
LINC00484	0.6097561	-0.713696	0.4270531	15	6	20	2	13	10
LINC00484	0.6097561	-0.713696	0.4270531	15	6	20	2	13	10
TSSC4	0.8307488	-0.267516	0.2707994	553	770	934	631	702	542
TSSC2	1.0928151	0.1280494	0.1276685	1261	1165	1485	1395	1464	1415
CCDC69	0.8266108	-0.27472	0.2249796	536	667	706	558	565	455
AC044849.2	#DIV/0!	#DIV/0!	0.4501902	0	0	0	0	0	4
NSL1	1.1289976	0.1750424	0.0637588	1190	1082	1449	1179	1570	1452
LINC00479	1.6261682	0.7014765	0.0433686	33	27	47	59	54	61
AP000223.1	0	#NUM!	0.3473206	0	5	0	0	0	0
ASCC2	0.8979592	-0.155278	0.4677801	1884	2129	2455	1897	2091	1820
ASCC3	1.0261123	0.0371886	0.6793002	3622	1929	3372	2069	3610	3477
CCDC77	1.2496413	0.3215141	0.0130344	677	549	865	742	1044	827
LINC00475	0.751938	-0.411314	0.4842094	34	34	61	40	30	27
CCDC71	0.9414209	-0.087088	0.9062204	660	838	909	795	765	706
LINC00476	0.8035191	-0.315596	0.3686784	102	114	125	99	85	90
LINC00477	#DIV/0!	#DIV/0!	0.8030358	0	0	0	0	1	0
CCDC73	0.2222222	-2.169925	0.2444362	4	0	14	0	4	0
LINC00471	1.5494505	0.6317567	0.0980738	32	29	30	51	49	41
AL645608.7	#DIV/0!	#DIV/0!	0.6482162	0	0	0	0	1	1
LINC00472	1.125	0.169925	0.9330512	4	3	1	2	7	0
AL645608.4	0	#NUM!	0.824326	0	0	1	0	0	0
LINC00474	0	#NUM!	0.824326	0	1	0	0	0	0
ASCC1	1.1944571	0.256355	0.0224959	648	719	834	783	900	946
CCDC57	0.7978227	-0.32586	0.0834893	666	592	671	437	506	596
IDH3G	0.916586	-0.125658	0.7233728	1452	1779	1936	1728	1582	1426
AL645608.8	0.8837209	-0.178337	0.8337932	13	15	15	9	17	12
AMFR	0.6403081	-0.643162	2.96E-05	1180	1339	1376	809	825	860
CCDC58	1.0475295	0.0669909	0.315175	1108	1107	1509	1240	1455	1206

AL645608.9	0.7142857	-0.485427	0.7745063	3	2	2	0	4	1
CCDC59	1.1675147	0.2234407	0.0953822	937	597	1021	977	1137	869
LINC00470	1.0514403	0.072367	0.5356894	162	112	212	193	176	142
AL645608.2	0.7714286	-0.374396	0.6765492	20	7	8	9	10	8
AC034111.1	1	0	0.9633892	3	1	0	0	4	0
AL645608.1	0	#NUM!	0.3473206	0	5	0	0	0	0
FRAS1	0.9897698	-0.014835	0.9764363	264	268	250	146	290	338
CCDC65	0.765625	-0.38529	0.3504749	82	55	119	40	81	75
CCDC66	0.9884454	-0.016767	0.8494253	336	246	370	251	375	315
CCDC60	0.3787879	-1.400538	0.1050981	33	5	28	12	10	3
CCDC61	1.0656814	0.0917763	0.4019183	361	419	438	479	456	363
LINC00466	0.9	-0.152003	0.9317337	7	2	1	3	1	5
CCDC62	1.3125	0.3923174	0.6529823	3	9	4	12	2	7
LINC00467	1.1423324	0.1919825	0.1263566	350	329	410	421	403	420
FRAT1	1.5	0.5849625	0.1304441	25	50	33	50	58	54
FRAT2	1.2419499	0.312607	0.0253365	635	846	755	903	1016	858
C20orf204	1.075	0.1043367	0.8604979	12	16	12	7	15	21
AC092811.1	#DIV/0!	#DIV/0!	0.5371397	0	0	0	0	0	3
C20orf202	0	#NUM!	0.0408242	5	2	5	0	0	0
AC092811.2	0	#NUM!	0.6436235	2	0	0	0	0	0
C20orf203	#DIV/0!	#DIV/0!	0.1397629	0	0	0	3	5	0
NSMF	1.0318644	0.0452534	0.5013856	398	471	606	485	574	463
ABHD17AP1	1.037037	0.0524674	0.8905849	11	8	8	10	6	12
ABHD17AP6	2	1	0.221138	4	6	8	17	15	4
ABHD17AP3	0	#NUM!	0.824326	0	1	0	0	0	0
AC096920.1	#DIV/0!	#DIV/0!	0.8030358	0	0	0	1	0	0
ABHD17AP4	0.6666667	-0.584963	0.8537226	2	0	1	0	0	2
C2CD4A	0.5	-1	0.81753	2	0	0	0	0	1
C2CD4C	0.5	-1	0.4542509	4	6	6	0	6	2
HNRNPLL	0.9329852	-0.100074	0.7255107	982	1129	1336	849	1216	1151
ABCC6P1	0.6666667	-0.584963	0.6539812	8	3	4	3	6	1
ABCC6P2	0.2222222	-2.169925	0.1434002	3	6	9	0	2	2
SNAP91	0.8688525	-0.202817	0.8249322	24	11	26	14	16	23
PM20D1	#DIV/0!	#DIV/0!	0.8030358	0	0	0	0	0	1
PM20D2	1	0	0.9286496	10	3	12	6	6	13
CABYR	#DIV/0!	#DIV/0!	0.0676226	0	0	0	1	2	7
C2CD2L	0.7905983	-0.338983	0.2914504	165	148	155	140	106	124
SLC44A5	0.5154639	-0.956057	0.0836456	32	28	37	11	24	15
SLC44A3	1.5035461	0.5883691	0.005145	136	104	183	208	247	181
SLC44A4	#DIV/0!	#DIV/0!	0.4737294	0	0	0	0	4	0
SLC44A1	1.0373867	0.0529538	0.5011655	1851	1530	1915	1361	2043	2090
SLC44A2	0.9357036	-0.095876	0.7239713	9894	11725	12582	9786	11725	10491
AC090260.1	1.3333333	0.4150375	0.8657302	1	1	1	0	3	1
PGGHG	1.4709141	0.556713	0.0013914	349	255	479	589	526	478
USMG5	1.3181295	0.3984921	0.0012633	1299	1389	1653	2032	2034	1656
AC018865.2	0	#NUM!	0.5387425	3	0	0	0	0	0
AC018865.1	#DIV/0!	#DIV/0!	0.5509701	0	0	0	0	3	0
C2CD4D	1.1666667	0.2223924	0.8082286	0	6	6	5	1	8
AL122125.1	0.9111111	-0.134301	0.9337651	13	19	13	19	7	15
HNRNPH1	0.9458388	-0.080334	0.8617102	17369	13536	19777	13770	17436	16731
HNRNPH2	0.9844439	-0.022619	0.809181	2718	2151	2845	2164	2840	2590
HNRNPH3	1.2679785	0.3425302	0.001864	5506	4117	5979	5555	7527	6701
SAA4	0	#NUM!	0.4005425	3	1	0	0	0	0
AL355483.3	0.1176471	-3.087463	0.1047398	4	1	12	0	0	2

AL355483.1	2.5	1.3219281	0.6803959	0	2	0	0	5	0
AL355483.2	0	#NUM!	0.824326	0	0	1	0	0	0
NT5C	1.1155235	0.1577209	0.3154904	172	169	213	250	192	176
AMN1	0.6623377	-0.594361	0.3247744	22	29	26	8	28	15
NT5E	0	#NUM!	0.5781912	0	0	3	0	0	0
AC084035.1	1.5	0.5849625	0.8175361	2	0	0	3	0	0
NT5M	1.0666667	0.0931094	0.7473882	37	42	26	53	33	26
AF111169.1	1.6444444	0.7176003	0.1691634	10	11	24	23	27	24
AF111169.4	#DIV/0!	#DIV/0!	0.3668985	0	0	0	3	0	1
ARNTL2-AS1	0.125	-3	0.2190945	1	3	4	0	1	0
SAA1	1.1127049	0.1540711	0.3782283	133	181	174	198	199	146
SAA2	0.4117647	-1.280108	0.181609	10	8	16	4	6	4
CWC25	0.8692748	-0.202116	0.3600344	649	618	829	589	682	551
DISC1FP1	0.6666667	-0.584963	0.8614507	2	0	4	3	0	1
AC112236.1	#DIV/0!	#DIV/0!	0.4121728	0	0	0	0	1	3
CWC27	1.2637631	0.337726	0.0291904	1058	625	1187	1129	1463	1035
AC112236.2	1.2	0.2630344	0.917847	3	2	0	0	1	5
AMOT	0.6306664	-0.665051	0.0003452	3302	3499	3523	1349	2365	2797
RBMX2	0.9736842	-0.038474	0.8558986	817	897	1098	878	1009	851
CWC22	1.4600794	0.5460468	0.0036219	890	421	956	1009	1301	1000
SACS	0.8016529	-0.31895	0.21501	392	308	510	336	361	273
RPL29P14	6	2.5849625	0.1627703	0	0	2	2	7	3
RPL29P12	1.2380952	0.3081223	0.6656599	4	9	8	11	4	11
AC009119.1	#DIV/0!	#DIV/0!	0.8030358	0	0	0	0	1	0
RPL29P11	0.9697322	-0.044342	0.9235699	274	269	316	290	311	232
AC009119.2	1.75	0.8073549	0.5184579	5	0	3	5	3	6
AC009119.3	0.6153846	-0.70044	0.7036385	0	5	8	0	1	7
WNT5A	0.2962963	-1.754888	0.0002646	47	66	49	11	22	15
RPL9P5	#DIV/0!	#DIV/0!	0.1199037	0	0	0	9	0	0
AC117422.1	1.125	0.169925	0.8432873	0	4	4	7	2	0
RPL9P7	0.9704797	-0.04323	0.9723684	85	102	84	95	90	78
RN7SL219P	0.2	-2.321928	0.2905519	3	0	7	0	1	1
RPL9P9	1.0843541	0.1168359	0.151833	13608	15501	17753	17241	18378	15196
AC078980.1	0.6666667	-0.584963	0.6771929	6	2	13	0	4	10
RPL9P2	#DIV/0!	#DIV/0!	0.249172	0	0	0	0	1	5
RPL9P3	8	3	0.1808589	0	1	0	3	3	2
IQSEC3P1	0	#NUM!	0.4374838	0	4	0	0	0	0
AC013439.1	0	#NUM!	0.5075476	0	0	4	0	0	0
AC121158.1	3	1.5849625	0.446028	0	0	2	2	2	2
RPL29P24	0.1666667	-2.584963	0.3471114	5	1	0	0	1	0
CABP7	0.25	-2	0.262849	8	2	2	0	3	0
CABP4	3	1.5849625	0.6896151	1	0	0	0	3	0
CABP1	0	#NUM!	0.1188281	1	1	7	0	0	0
RPL30P11	0	#NUM!	0.5436524	2	0	1	0	0	0
KLHDC2	0.8964613	-0.157687	0.4990615	717	650	922	565	737	750
KLHDC3	0.9554338	-0.065772	0.9902766	3070	3744	4136	3512	3750	3200
KLHDC4	0.8310259	-0.267035	0.147002	1763	1908	2324	1720	1747	1515
WNT3A	2.4444444	1.2895066	0.2852101	3	6	0	8	8	6
RPL29P30	#DIV/0!	#DIV/0!	0.6099294	0	0	0	2	0	0
KLHDC1	0	#NUM!	0.824326	0	0	1	0	0	0
CWC15	0.9571737	-0.063147	0.9449147	2269	2100	3033	2366	2602	2117
XAGE5	#DIV/0!	#DIV/0!	0.6554062	0	0	0	0	2	0
XAGE3	1.0888295	0.122778	0.2030066	874	1074	1418	1259	1279	1127
SAE1	0.9589108	-0.060532	0.9742651	7532	9119	10047	8524	8795	8282

XAGE2	1.1280628	0.1738474	0.0806797	6964	8475	9783	9910	10197	8345
KLHDC9	1.3178295	0.3981637	0.2415763	40	31	58	51	78	41
WNT2B	1.0757576	0.105353	0.7724227	22	11	33	12	22	37
AL121871.1	0.0714286	-3.807355	0.0820936	1	2	11	0	0	1
AL450469.1	2.75	1.4594316	0.3252086	2	1	1	6	3	2
AL450469.2	4.5	2.169925	0.3059574	2	0	0	2	6	1
SNAPC5	0.8585622	-0.220005	0.329593	547	498	666	482	553	434
P3H2-AS1	#DIV/0!	#DIV/0!	0.1952829	0	0	0	0	1	6
SNAPC1	1.419214	0.5050921	0.0298435	112	114	232	270	209	171
SNAPC2	0.8777555	-0.188109	0.5477361	156	164	179	131	151	156
RNU6-307P	0	#NUM!	0.6436235	2	0	0	0	0	0
SNAPC3	1.234083	0.3034394	0.0088833	1084	918	1155	1051	1420	1425
SNAPC4	1.0630631	0.0882272	0.3267534	728	793	810	721	910	847
SAFB	0.974427	-0.037374	0.8291533	5614	6141	7093	5899	6507	5960
WNT16	1	0	0.9714163	3	3	0	0	2	4
ERHP1	0	#NUM!	0.6604275	0	1	1	0	0	0
IGDCC4	0.5207547	-0.941324	0.0129348	90	61	114	28	55	55
DDX50P1	0.8235294	-0.280108	0.7486541	8	15	11	6	11	11
DDX50P2	1	0	0.9395902	0	0	2	1	0	1
IGDCC3	0.4613583	-1.11604	1.20E-06	281	314	259	113	152	129
RBMS1	1.0612678	0.0857888	0.3261767	4803	3441	4944	3701	5262	5033
AC079866.1	#DIV/0!	#DIV/0!	0.5509701	0	0	0	0	3	0
AC079866.2	#DIV/0!	#DIV/0!	0.2758481	0	0	0	4	0	1
RBMS2	0.9163004	-0.126107	0.5225456	1802	1676	1982	1380	1870	1753
RBMS3	0.9578313	-0.062156	0.973896	70	37	59	45	64	50
AL135744.1	#DIV/0!	#DIV/0!	0.8030358	0	0	0	0	1	0
ATXN2-AS	0.7272727	-0.459432	0.7429896	4	4	3	0	4	4
WNT11	0.9595633	-0.05955	0.9988909	694	865	914	765	844	764
PCED1A	1.0756972	0.105272	0.285564	454	458	594	578	555	487
PCED1B	0.9187146	-0.122311	0.8782471	137	161	231	181	164	141
SAPCD2P3	7	2.8073549	0.29254	0	0	1	0	4	3
MIR7111	1.8333333	0.8744691	0.6094944	0	6	0	9	0	2
VAT1L	1	0	0.9872082	3	0	4	0	5	2
AC023034.2	0	#NUM!	0.4324683	2	0	2	0	0	0
AC023034.1	1	0	0.9127856	18	5	21	11	14	19
AC090510.2	0.047619	-4.392317	0.0195466	1	10	10	1	0	0
RNU6-308P	0	#NUM!	0.6436235	2	0	0	0	0	0
AC007881.3	0	#NUM!	0.5781912	0	0	3	0	0	0
RN7SL236P	0.2307692	-2.115477	0.2100125	7	2	4	0	1	2
PPP4R1	1.0614334	0.0860139	0.2658034	2075	1998	2373	1830	2549	2463
PPP4R2	1.0981739	0.1351066	0.2318547	2104	1358	2069	1624	2258	2192
PPP4R4	0.36	-1.473931	0.1942927	7	7	11	3	2	4
AC074044.1	1	0	0.9265837	0	1	2	3	0	0
MRPL18	0.9063628	-0.141839	0.5244837	1594	1647	2024	1525	1770	1477
MRPL19	1.0041352	0.0059536	0.6486948	1451	1180	1480	1212	1410	1506
MRPL16	0.95665	-0.063937	0.9475244	1211	1359	1836	1351	1406	1458
IL20RB	0.9424572	-0.085501	0.9162931	187	195	261	190	243	173
MRPL17	0.9749172	-0.036648	0.8127999	1650	2226	2463	2244	2010	1926
MRPL14	0.9289289	-0.10636	0.7925433	1366	1755	1874	1621	1617	1402
MRPL15	0.979596	-0.029741	0.7905913	1376	1588	1888	1523	1700	1530
MRPL12	0.8928571	-0.163499	0.8987974	25	64	79	65	37	48
MRPL13	0.894913	-0.160181	0.4565099	948	939	1101	870	937	867
MRPL10	0.9494606	-0.07482	0.8726037	2011	2381	2375	2036	2273	2116
MRPL11	0.9650333	-0.051349	0.9014017	2526	2848	3177	3018	2874	2360

MRPL20	1.0690365	0.0963111	0.2407671	1506	1747	2092	2008	1969	1737
KCNMB2	0.2857143	-1.807355	0.494116	1	0	6	0	2	0
KCNMB3	0.887218	-0.172639	0.706313	140	51	75	59	86	91
KCNMB4	1.0501319	0.0705706	0.6430628	85	151	143	121	147	130
AMZ2	0.9281967	-0.107498	0.6832753	1990	2092	2589	1855	2195	2142
AMZ1	0.5333333	-0.906891	0.2530553	17	12	16	3	12	9
CADM4	0.5244216	-0.931201	0.0067621	110	104	175	62	51	91
CADM1	0.475962	-1.071082	2.32E-16	3524	2943	3538	1347	1707	1708
COL24A1	2	1	0.5596494	4	1	0	2	1	7
CADM2	1.1111111	0.1520031	0.863924	3	14	1	13	0	7
MRPL27	0.8918179	-0.165179	0.5128568	1668	2016	2158	1863	1800	1547
MRPL28	0.9160225	-0.126545	0.7373603	2577	3234	3632	3201	3002	2447
MRPL23	1.1268095	0.1722436	0.2163412	459	632	636	782	606	558
MRPL24	0.865713	-0.208039	0.3640987	1783	2174	2544	1982	2045	1601
MRPL21	0.9277196	-0.108239	0.8333104	1110	1378	1621	1384	1422	1006
MRPL22	0.9640686	-0.052792	0.9381379	1624	1762	2208	1672	2020	1701
MRPL30	0.9617458	-0.056272	0.9970182	1193	1282	1420	1177	1406	1163
AL354809.1	1.3333333	0.4150375	0.8633745	1	2	0	1	0	3
EZH1	0.9159159	-0.126713	0.6336932	841	806	1017	764	827	849
EZH2	1.0664837	0.0928619	0.2146122	2166	1836	2556	1987	2632	2375
GMPSP1	1.025974	0.0369942	0.8752454	24	24	29	16	26	37
AL354893.1	0.2285714	-2.129283	0.0386048	11	10	14	1	4	3
AL354893.2	2	1	0.8118689	0	0	1	0	1	1
MRPL38	0.8584071	-0.220266	0.6978284	32	45	36	31	46	20
MRPL39	1.0490434	0.0690743	0.3011728	1182	1063	1466	1217	1440	1236
MRPL36	1.0138368	0.0198255	0.5915721	997	1211	1261	1140	1307	1070
MRPL37	0.9372714	-0.093461	0.8069253	3139	3729	4068	3395	3657	3198
MRPL34	0.8834535	-0.178774	0.4590006	1532	1769	1830	1648	1536	1349
MRPL35	0.9434637	-0.083961	0.841131	1427	1260	1505	1259	1372	1324
MRPL32	0.9812663	-0.027283	0.7323149	1284	1084	1849	1353	1489	1296
MRPL33	1.1087038	0.148874	0.1482434	707	816	1154	996	1033	939
MRPL41	0.9812669	-0.027283	0.7613537	1695	2154	2450	2360	2056	1765
MRPL42	1.024939	0.0355381	0.5269701	1354	1010	1325	1102	1350	1329
MRPL40	1.0667879	0.0932734	0.2247171	1200	1186	1462	1366	1456	1283
TRGV5P	1.1428571	0.1926451	0.9712048	0	7	0	0	6	2
AC005006.1	0	#NUM!	0.1381813	4	4	0	0	0	0
RNU6-323P	#DIV/0!	#DIV/0!	0.5371397	0	0	0	0	0	3
AL390026.1	1	0	0.9890559	1	0	0	0	0	1
EIPR1	1.1237432	0.1683124	0.1030648	1104	1358	1417	1446	1448	1465
MRPL49	0.8249822	-0.277565	0.0879708	1611	1915	2102	1435	1736	1472
MRPL47	0.996659	-0.004828	0.6527669	1324	1080	1487	1296	1363	1219
MRPL48	0.9069644	-0.140882	0.6661942	619	542	720	603	611	492
MRPL45	1.0095677	0.0137376	0.5823214	1709	1907	2028	1871	2069	1758
MRPL46	0.6541787	-0.612243	0.042487	117	111	119	62	95	70
MRPL43	1.437663	0.5237256	0.0007672	548	592	777	1067	927	762
MRPL44	0.9378414	-0.092584	0.7375847	1641	1725	1943	1466	1935	1578
MRPL52	0.9775007	-0.03283	0.7837228	2036	2464	2878	2701	2457	2054
MRPL53	1.3386581	0.4207876	0.0708771	107	107	99	150	146	123
MRPL50	1.1968431	0.259234	0.0402943	1165	801	1265	1207	1493	1167
MRPL51	0.9602766	-0.058478	0.9516778	2135	2371	2870	2366	2591	2126
AC121493.1	#DIV/0!	#DIV/0!	0.1819446	0	0	0	4	1	1
KCNMA1	0.3333333	-1.584963	0.3928148	1	2	9	0	1	3
RNU6-322P	2.4	1.2630344	0.3673554	2	0	3	5	2	5
RPLP1P6	0.8695652	-0.201634	0.8648427	7	4	12	1	16	3

MRPL58	0.8131229	-0.298455	0.1785411	706	802	900	723	684	551
MRPL57	1.0059756	0.0085954	0.6088342	1212	1407	1732	1519	1638	1220
MRPL54	0.9464494	-0.079403	0.9566533	242	254	363	328	243	242
MRPL55	1.0257848	0.036728	0.5412515	487	590	707	703	631	496
NTF4	1.1073826	0.1471537	0.5852252	48	53	48	64	60	41
CEPT1	0.9552105	-0.066109	0.8744078	1103	1025	1221	755	1189	1255
USP9X	0.9722268	-0.040635	0.9751929	2275	1460	2314	1343	2172	2366
USP9Y	0.945122	-0.081428	0.8718527	261	152	243	123	232	265
AC006070.1	2	1	0.834705	0	0	1	0	2	0
GASAL1	0.7555556	-0.40439	0.4941048	29	25	36	19	34	15
RNU7-171P	0	#NUM!	0.4217812	0	0	5	0	0	0
ADH5P3	5.5	2.4594316	0.1625285	0	1	1	5	3	3
ADH5P4	1.2534562	0.3259116	0.2341062	73	71	73	75	120	77
AL670729.1	1.6551724	0.7269815	0.2605305	12	8	9	21	13	14
SAT2	0.8379447	-0.255073	0.637726	60	89	104	88	52	72
SAT1	1.1857835	0.2458407	0.0263698	990	988	1117	1247	1297	1126
EIF4ENIF1	0.863671	-0.211446	0.4021382	382	440	491	391	372	371
OR52H2P	0	#NUM!	0.824326	0	1	0	0	0	0
AC034114.1	4	2	0.4849955	1	0	0	3	1	0
SARS	1.1133569	0.1549161	0.0724462	8735	10752	11980	10877	12521	11636
TPT1P14	#DIV/0!	#DIV/0!	0.5371397	0	0	0	0	0	3
TPT1P13	0	#NUM!	0.4324683	2	0	2	0	0	0
SAV1	1.0549065	0.0771152	0.3452996	1425	1314	1541	1190	1705	1620
AC090164.2	8	3	0.2298001	1	0	0	8	0	0
AC024257.1	0.2	-2.321928	0.4760937	1	0	4	0	0	1
AC010401.2	0	#NUM!	0.5172237	1	2	0	0	0	0
AC024257.2	#DIV/0!	#DIV/0!	0.8030358	0	0	0	0	1	0
AC024257.3	#DIV/0!	#DIV/0!	0.6554062	0	0	0	0	2	0
MYPOP	0.9639279	-0.053003	0.9091144	103	171	225	161	142	178
RBPMS	1.0233278	0.0332684	0.4239066	2666	2842	3537	3098	3326	2832
TFPI2	0.7636364	-0.389042	0.5635583	26	16	13	6	24	12
BUD13P1	0	#NUM!	0.4324683	2	0	2	0	0	0
PTPRD	1.25	0.3219281	0.8590344	1	0	3	0	3	2
PTPRE	2.0915698	1.0645861	1.90E-10	230	222	236	415	485	539
PTPRB	0.8333333	-0.263034	0.6866321	48	19	35	30	30	25
CFLAR-AS1	1.5	0.5849625	0.8536377	0	1	1	0	3	0
PTPRA	0.9397993	-0.089575	0.7811045	1529	1617	1937	1384	1711	1682
VPS9D1-AS1	0.8344595	-0.261086	0.5114508	101	69	126	71	89	87
NFE2L3P1	#DIV/0!	#DIV/0!	0.5070017	0	0	0	3	0	0
SLC25A5-AS1	0.9166667	-0.125531	0.782138	80	63	61	49	83	55
AC104771.1	#DIV/0!	#DIV/0!	0.4501902	0	0	0	0	0	4
AC139792.1	0	#NUM!	0.158009	5	0	3	0	0	0
AC139792.2	0	#NUM!	0.6436235	2	0	0	0	0	0
DIS3L2	0.9312562	-0.10275	0.7513833	619	695	708	602	647	634
TUBB8P1	0.4	-1.321928	0.2972746	6	4	15	0	3	7
TUBB8P2	2	1	0.7054974	0	1	1	0	3	1
TUBB8P7	1.4	0.4854268	0.8045569	1	4	0	2	0	5
TUBB8P8	0.6	-0.736966	0.7511269	3	0	2	0	2	1
AC010547.1	4.25	2.0874628	0.2003323	1	0	3	0	11	6
AC010547.3	0	#NUM!	0.3482063	0	0	6	0	0	0
AC010547.5	0	#NUM!	0.1551432	2	3	2	0	0	0
AC226118.1	0	#NUM!	0.824326	0	0	1	0	0	0
RBPJL	0.2	-2.321928	0.2964117	0	10	0	1	1	0
AC116347.1	0.9166667	-0.125531	0.9837502	1	6	5	6	4	1

NTN4	3.5	1.8073549	0.3938106	1	1	0	2	4	1
NTN5	1.125	0.169925	0.8849196	7	1	0	6	1	2
NTN1	0.7092176	-0.4957	0.0059355	942	1217	998	616	790	833
NTN3	2	1	0.834705	0	0	1	0	2	0
AC109486.1	0	#NUM!	0.824326	1	0	0	0	0	0
MAPT-IT1	#DIV/0!	#DIV/0!	0.8030358	0	0	0	0	0	1
PTPN1	0.9658506	-0.050128	0.9752583	3258	3781	4030	2845	3936	3910
AL391095.3	0	#NUM!	0.1038898	0	4	6	0	0	0
PTPN9	0.7078907	-0.498401	0.0145495	384	435	499	324	318	291
PTPN6	0.9449397	-0.081706	0.9867105	531	617	759	680	600	522
PTPN7	0.3478261	-1.523562	0.2063252	2	15	6	2	4	2
PTPN4	1.0468114	0.0660015	0.5399458	547	336	591	382	594	567
AL391095.1	0.75	-0.415037	0.7790464	2	5	5	1	8	0
PTPN2	1.0849185	0.1175866	0.3223539	538	329	605	478	586	533
PTPN3	1.0758825	0.1055206	0.2619382	1003	900	1128	853	1195	1213
AC116348.1	#DIV/0!	#DIV/0!	0.249172	0	0	0	0	1	5
PPP5D1	1.2666667	0.3410369	0.7417667	8	0	7	4	11	4
KDELR1	0.9366715	-0.094385	0.8458751	4099	4855	5605	4698	4939	4000
THUMPD2	1.2024666	0.2659968	0.0938185	692	377	877	820	809	711
THUMPD3	0.9318714	-0.101797	0.7378022	1624	1269	1804	1233	1576	1568
SPATA20	0.7291005	-0.45581	0.0334099	272	324	349	214	258	217
THUMPD1	1.0611345	0.0856076	0.4072462	1989	1285	1944	1410	2080	2047
SPATA25	1.1428571	0.1926451	0.8139513	1	9	4	9	3	4
SPATA22	0.6700434	-0.577674	0.0250784	250	192	249	168	143	152
SPATA24	1.1727447	0.229889	0.1968266	137	175	209	174	248	189
ANK2	1.9565217	0.9682911	0.0082785	33	22	37	62	60	58
ANK3	1.3368757	0.4188653	0.0172996	720	457	705	562	994	960
ANK1	1.7380952	0.7975071	0.1829326	12	21	9	19	24	30
SPATA16	0	#NUM!	0.824326	1	0	0	0	0	0
SPATA17	1.7037037	0.7686745	0.3555898	17	1	9	8	22	16
SPATA12	3	1.5849625	0.4102074	3	0	0	4	4	1
SPATA13	1.0534351	0.0751015	0.5388675	325	270	322	239	349	378
SPATA13	1.0534351	0.0751015	0.5388675	325	270	322	239	349	378
KDELR2	0.9530183	-0.069424	0.9140367	8021	8530	9672	7804	8987	8200
KDELR3	7.5	2.9068906	0.0841616	0	0	2	5	5	5
WFDC10B	0	#NUM!	0.3052077	1	4	0	0	0	0
SPATA18	2.8333333	1.5025003	0.2464717	4	0	2	10	2	5
KIAA1211L	1.1356547	0.1835242	0.0668624	814	912	1031	936	1138	1057
WNK4	1.54	0.6229304	0.1510135	31	26	43	23	51	80
MIR3142HG	0	#NUM!	0.6840298	0	0	2	0	0	0
AC138473.1	0	#NUM!	0.5387425	3	0	0	0	0	0
ANKH	0.5134938	-0.961581	9.50E-07	435	454	482	180	238	286
AC010307.2	#DIV/0!	#DIV/0!	0.8030358	0	0	0	0	0	1
NRBF2P5	0.5294118	-0.917538	0.4755503	5	6	6	0	4	5
NRBF2P2	0	#NUM!	0.824326	0	0	1	0	0	0
NRBF2P3	2	1	0.8319548	0	1	0	0	2	0
AL450322.1	0	#NUM!	0.824326	1	0	0	0	0	0
IFITM10	1	0	0.993954	3	6	2	2	5	4
ANLN	1.0174461	0.0249524	0.6980313	3807	2071	3637	2297	3719	3665
WNK1	0.8663055	-0.207052	0.2922178	4820	4171	4854	2656	4313	5025
3|protein phospl	#VALUE!	#VALUE!	osol;GO:000	NA	NA	eficient protein kinase 1			
WNK2	1.2433781	0.3142651	0.0072738	759	894	952	940	1214	1085
WNK3	1.0097087	0.0139392	0.8176709	83	29	94	56	68	84
YES1P1	2.4285714	1.2801079	0.3167445	1	0	6	8	8	1

PTPRT	0	#NUM!	0.824326	0	0	1	0	0	0
PTPRU	0.9012784	-0.149955	0.4021173	4769	5667	5913	4287	5336	5112
PTPRR	#DIV/0!	#DIV/0!	0.5371397	0	0	0	0	0	3
PTPRS	0.9807322	-0.028069	0.8556481	167	160	192	179	151	179
USPL1	1.0357942	0.0507374	0.6063095	709	358	721	481	713	658
PTPRQ	1.25	0.3219281	0.8259377	1	0	7	0	9	1
PTPRN	1	0	0.9083075	3	0	13	3	4	9
PTPRO	0.8977273	-0.155651	0.8253881	29	30	29	26	33	20
PTPRM	1.23932	0.3095487	0.0146527	744	676	874	724	1033	1086
PTPRJ	0.7983952	-0.324825	0.1197478	622	663	709	375	534	683
PTPRK	0.7677139	-0.381359	0.0519205	1074	813	1105	531	833	933
PTPRH	0.75	-0.415037	0.8622918	1	5	6	6	0	3
AL355482.1	3.25	1.7004397	0.2786745	3	0	1	4	7	2
AL355482.2	0.3333333	-1.584963	0.7290749	0	0	3	0	0	1
PTPRF	0.9390109	-0.090786	0.7153461	17622	21474	21341	14760	20439	21552
PTPRG	0.92978	-0.105039	0.6933478	845	702	817	467	846	885
BABAM1	1.1244696	0.1692446	0.233022	226	216	265	287	266	242
BABAM2	0.8295419	-0.269613	0.1625403	991	1040	1178	932	909	821
YBX1P6	0.5	-1	0.8241474	0	1	1	0	0	1
ANO8	0.964209	-0.052582	0.9754845	420	435	542	391	502	454
ANO9	0.9659401	-0.049994	0.8727447	202	190	342	238	252	219
YBX1P1	1.2394366	0.3096845	0.073408	193	167	208	226	269	209
ANO6	0.9140313	-0.129685	0.560826	4406	3469	4141	2487	4024	4472
ANO7	3.2413793	1.6966079	0.0053299	8	14	7	34	41	19
ANO4	2.25	1.169925	0.5156363	3	1	0	6	3	0
YBX1P4	3	1.5849625	0.6136153	0	1	0	2	1	0
ANO5	0.7066667	-0.500898	0.4833988	36	9	30	15	16	22
ANO2	0.9642857	-0.052467	0.9595308	136	147	221	125	169	192
YBX1P2	0.7636364	-0.389042	0.5982596	14	22	19	15	15	12
ANO3	1.5714286	0.6520767	0.6932584	4	0	3	2	9	0
ANO1	1.36	0.4436067	0.5509222	10	3	12	6	12	16
AL513303.2	0	#NUM!	0.4217812	0	0	5	0	0	0
AL513303.1	1.3129771	0.3928418	0.2430154	27	46	58	53	72	47
SLC43A2	1.0870809	0.1204594	0.1908474	2893	3528	3363	3164	3905	3567
SLC43A1	1.0416667	0.0588937	0.8564228	12	35	25	20	27	28
CAMKK1	0.7622378	-0.391687	0.4177862	46	41	56	28	32	49
CAMKK2	0.6720721	-0.573312	3.54E-05	1231	1204	1450	800	932	879
AC099560.1	#DIV/0!	#DIV/0!	0.1700664	0	0	0	0	9	0
AC099560.2	0.9732938	-0.039053	0.8544738	92	90	155	122	105	101
AD001527.1	6	2.5849625	0.3096061	0	0	1	5	0	1
AC145098.1	0.6666667	-0.584963	0.8574108	0	2	1	1	0	1
AC145098.2	1	0	0.8854874	6	13	30	11	12	26
ATP8A2P2	#DIV/0!	#DIV/0!	0.8030358	0	0	0	0	0	1
IFITM4P	0.1666667	-2.584963	0.3847728	2	0	4	0	1	0
IGBP1P1	1.125	0.169925	0.9254316	1	7	0	3	3	3
AC104532.1	#DIV/0!	#DIV/0!	0.5509701	0	0	0	0	3	0
AC104532.2	0.1818182	-2.459432	0.2271914	7	4	0	0	2	0
BX322650.1	#DIV/0!	#DIV/0!	0.2116658	0	0	0	1	4	1
IGBP1P5	0	#NUM!	0.824326	0	1	0	0	0	0
IGBP1P4	10.5	3.3923174	0.0275528	1	0	1	11	2	8
GIMAP2	0.1428571	-2.807355	0.0766097	2	14	5	0	0	3
GIMAP4	1.2692308	0.3439544	0.6167708	7	10	9	13	14	6
GIMAP6	0	#NUM!	0.1996036	0	0	9	0	0	0
SBF1	0.9223668	-0.116587	0.5666566	3989	4461	4766	3510	4359	4321

AL160408.3	#DIV/0!	#DIV/0!	0.0453056	0	0	0	8	4	0
SBF2	0.8223056	-0.282253	0.2058426	1063	826	1043	486	892	1033
CAMKMT	0.9845095	-0.022523	0.8250481	365	387	410	386	383	375
SBDS	1.0436659	0.0616599	0.3060934	1839	1662	2293	1829	2275	1943
UQCRBP1	5	2.3219281	0.3814269	0	1	0	4	0	1
UQCRBP2	#DIV/0!	#DIV/0!	0.8030358	0	0	0	0	1	0
WNT6	0.9334177	-0.099405	0.8982634	2168	2708	3024	2867	2486	2021
AC135586.1	0	#NUM!	0.5643268	1	0	2	0	0	0
WNT1	5.5	2.4594316	0.2387574	0	2	0	5	6	0
WNT2	2	1	0.6684796	0	0	2	1	0	3
WNT3	0.528169	-0.920928	0.0004323	239	218	253	71	162	142
WNT4	1.0529801	0.0744782	0.7638927	36	59	56	43	73	43
SPATA6L	1.7416667	0.8004685	0.0165693	34	30	56	58	75	76
AC009118.3	1.8235294	0.8667335	0.3077002	7	6	4	9	11	11
LINC00701	0.90625	-0.142019	0.9100363	14	6	12	7	10	12
LINC00702	0	#NUM!	0.6385096	0	2	0	0	0	0
AL391834.1	1.0588235	0.0824622	0.9511527	4	7	6	0	9	9
AL391834.2	1.6428571	0.716207	0.3048961	9	1	18	17	14	15
RNU6-319P	0	#NUM!	0.824326	0	0	1	0	0	0
TEX13B	0.7708333	-0.375509	0.641906	21	13	14	15	12	10
PPDPF	1.2766689	0.3523844	0.0130766	2619	2987	3412	4628	3865	3020
TEX13C	1.3571429	0.4405726	0.5450566	6	13	9	12	19	7
AL355388.2	0.4954128	-1.013297	0.0738036	39	27	43	7	20	27
AL450468.1	0.5	-1	0.8188512	2	0	0	0	1	0
SPATA41	1.5	0.5849625	0.6489463	10	9	1	1	22	7
SPATA42	#DIV/0!	#DIV/0!	0.8030358	0	0	0	0	0	1
KDELC1	0.9917313	-0.011979	0.7986488	548	664	723	564	766	589
KDELC2	0.6840429	-0.547841	0.0002021	2457	2004	2445	1233	1805	1686
SPATA45	0	#NUM!	0.5781912	0	0	3	0	0	0
SPATA46	0	#NUM!	0.0791808	0	12	0	0	0	0
AC090809.1	0.9411765	-0.087463	0.9621938	5	6	6	3	5	8
RN7SL225P	#DIV/0!	#DIV/0!	0.8030358	0	0	0	1	0	0
AC022445.1	0.3571429	-1.485427	0.4520161	5	0	9	0	0	5
SPATA2L	1.0147643	0.0211447	0.5827466	521	580	660	663	563	561
SBK2	0	#NUM!	0.3958203	2	2	0	0	0	0
SBK3	0.5	-1	0.7090565	2	0	4	0	0	3
SBK1	0.8041237	-0.314511	0.6059584	40	18	39	15	29	34
SPATA32	1.8571429	0.8930848	0.4949328	0	4	3	5	4	4
SPATA33	0.9961929	-0.005503	0.7075535	248	201	339	323	251	211
ROPN1B	0.9279279	-0.107915	0.9292074	11	56	44	37	53	13
RPL9P29	2.1818182	1.1255309	0.2689706	5	3	3	15	7	2
RPLP0P6	0.9696902	-0.044404	0.897445	2594	3197	3183	3134	3032	2536
RPLP0P2	0.2857143	-1.807355	0.3918082	5	1	1	0	1	1
AC004656.1	1.2686567	0.3433017	0.0857007	129	96	177	166	183	161
RPL9P21	#DIV/0!	#DIV/0!	0.8030358	0	0	0	1	0	0
NUB1	0.9293989	-0.10563	0.6440419	1421	1499	1655	1192	1650	1410
LINC00659	1.5	0.5849625	0.3730105	9	6	13	17	17	8
LINC00656	1	0	0.9239746	1	0	2	3	0	0
RPLP0P1	0	#NUM!	0.3320541	3	0	2	0	0	0
ROPN1L	1.8235294	0.8667335	0.2836985	4	6	7	11	8	12
MPV17	0.9676755	-0.047405	0.8646278	1401	1461	1995	1526	1718	1456
LINC00652	0.8484848	-0.237039	0.8992299	10	4	19	13	11	4
LINC00654	1.75	0.8073549	0.6882666	2	0	2	0	5	2
AC015977.1	#DIV/0!	#DIV/0!	0.3109758	0	0	0	0	6	0

NARF-IT1	0.5625	-0.830075	0.4337723	12	4	16	4	3	11
RPL9P31	0	#NUM!	0.824326	0	1	0	0	0	0
LINC00648	1.1246944	0.169533	0.3854093	163	96	150	151	182	127
LINC00649	0.9386617	-0.091323	0.8111091	189	164	185	118	207	180
LINC00645	0.8	-0.321928	0.7982249	4	9	7	3	2	11
LINC00640	1.3333333	0.4150375	0.7779595	1	4	1	3	1	4
LINC00641	0.977588	-0.032701	0.9422009	340	229	368	212	360	344
PHACTR2P1	1	0	0.9756864	2	0	0	0	2	0
ASF1A	1.1885475	0.2491995	0.0372065	1294	900	1386	1342	1574	1339
LINC00642	0.1666667	-2.584963	0.1980682	3	1	8	1	0	1
ASF1B	0.8819802	-0.181182	0.362905	1390	1738	1922	1384	1605	1465
LINC00643	2.0166667	1.0119726	0.0479977	23	12	25	18	55	48
LINC00680	0.8759124	-0.191141	0.6050306	189	152	207	170	146	164
LINC00681	0	#NUM!	0.824326	1	0	0	0	0	0
LINC00683	#DIV/0!	#DIV/0!	0.8030358	0	0	0	0	0	1
AC074043.1	0	#NUM!	0.4460243	4	0	0	0	0	0
PPP3R1	1.106755	0.1463358	0.1034447	1230	1104	1441	1245	1591	1342
RN7SL246P	0	#NUM!	0.6840298	0	0	2	0	0	0
AC138907.10	#DIV/0!	#DIV/0!	0.6554062	0	0	0	0	2	0
LINC00671	0	#NUM!	0.824326	1	0	0	0	0	0
LINC00672	0.5384615	-0.893085	0.5294211	3	7	3	3	3	1
AL354798.1	0.3333333	-1.584963	0.5612825	5	0	1	2	0	0
TYSND1	0.9647836	-0.051723	0.9379848	395	461	507	443	432	440
LINC00667	0	#NUM!	0.6840298	0	0	2	0	0	0
LINC00668	1.9318996	0.9500202	3.26E-05	88	78	113	181	197	161
LINC00662	1.2571429	0.3301486	0.1941461	141	79	95	117	132	147
LINC00663	0.8925234	-0.164038	0.7398278	65	64	85	52	93	46
LINC00664	0.8931298	-0.163058	0.9057754	39	35	57	54	23	40
LINC00665	0.9493525	-0.074984	0.9270411	988	1184	1303	1058	1102	1139
MYOM1	0.6666667	-0.584963	0.6596502	4	6	2	1	3	4
PRSS27	1.1136364	0.1552782	0.5588914	75	81	64	83	104	58
PRSS22	1.0283749	0.0403663	0.5655854	289	403	471	464	399	333
PRSS23	0.8607509	-0.216332	0.3573947	405	522	538	411	436	414
MYOM2	0.9552239	-0.066089	0.9415676	42	40	52	64	37	27
PRSS21	0.9151194	-0.127968	0.8077505	122	124	131	124	105	116
AL136084.1	0	#NUM!	0.824326	1	0	0	0	0	0
LINC00615	1	0	0.9890559	1	0	0	1	0	0
LINC00618	1.25	0.3219281	0.9199625	0	3	1	0	5	0
NUF2	1.205068	0.2691146	0.1138794	807	401	923	726	1049	793
ANXA6	0.7822354	-0.354325	0.0205696	6072	7658	7582	5339	5809	5523
ANXA7	1.1473354	0.1982872	0.0282974	2129	2180	2390	2280	2812	2594
LINC00612	0.5555556	-0.847997	0.7458677	0	0	9	2	2	1
ANXA8	0.7888906	-0.342103	0.0334708	1802	2056	2461	1630	1796	1559
LINC00613	#DIV/0!	#DIV/0!	0.8030358	0	0	0	0	1	0
ANXA9	1.3094141	0.3889214	0.004616	453	517	549	601	782	606
XXYLT1	0.7924528	-0.335603	0.0859808	444	545	548	377	448	393
NUDC	1.0267054	0.0380223	0.426886	4059	4798	5597	5234	5073	4533
ANXA1	2.1496206	1.1040821	3.91E-16	2308	1644	2638	4388	5470	4308
ANXA2	0.9956568	-0.00628	0.6427173	28453	32250	35540	30954	34571	30300
ANXA3	1.6885246	0.7557632	0.0006495	168	113	146	254	268	199
ANXA4	0.8650794	-0.209096	0.7332387	45	41	40	21	21	67
ANXA5	1.1914313	0.2526957	0.0041958	18423	19957	23146	23089	26880	23335
PRSS16	0.8387742	-0.253646	0.2588513	435	475	591	362	400	497
PRSS12	2.3505155	1.2329772	2.81E-12	196	159	227	357	519	492

AC008622.2	0	#NUM!	0.6436235	2	0	0	0	0	0
LINC00601	0	#NUM!	0.5337954	0	3	0	0	0	0
AC100814.1	0.5833333	-0.777608	0.7123008	0	7	5	6	1	0
PRSS48	1	0	0.9978496	2	0	2	0	4	0
PRSS46	#DIV/0!	#DIV/0!	0.6411933	0	0	0	0	0	2
PRSS44	1.1627907	0.2175914	0.678976	8	11	24	8	19	23
SBSN	20.333333	4.3457748	0.0001071	1	2	0	27	24	10
PRSS45	#DIV/0!	#DIV/0!	0.8030358	0	0	0	0	1	0
PRSS51	1.3684211	0.4525122	0.6599397	10	8	1	6	9	11
RNU7-160P	0	#NUM!	0.5387425	3	0	0	0	0	0
PRSS50	1	0	0.9765764	5	0	4	0	0	9
PRSS50	1	0	0.9765764	5	0	4	0	0	9
LINC00637	#DIV/0!	#DIV/0!	0.6411933	0	0	0	0	0	2
LINC00638	0.7419355	-0.430634	0.6729905	10	6	15	5	15	3
AC005005.4	0.3	-1.736966	0.3727855	4	2	4	0	0	3
LINC00639	2	1	0.8072994	0	1	0	0	1	1
AC005005.3	7	2.8073549	0.2346976	0	1	0	2	1	4
LINC00634	0.2777778	-1.847997	0.1860602	10	2	6	1	3	1
SC5D	0.7047024	-0.504914	0.0023015	1099	820	1122	602	813	728
LINC00635	1.5	0.5849625	0.7286698	0	0	4	1	3	2
LINC00636	0.125	-3	0.2730992	2	0	6	0	1	0
LINC00630	1.0912863	0.1260297	0.5743106	91	75	75	74	86	103
AOC3	0.7692308	-0.378512	0.6633486	13	10	16	8	11	11
RPL9P18	0	#NUM!	0.6436235	2	0	0	0	0	0
AOC1	0	#NUM!	0.3963337	1	3	0	0	0	0
AOC2	0.6	-0.736966	0.1754619	31	26	48	14	27	22
PRSS38	0	#NUM!	0.4005425	3	1	0	0	0	0
PRSS35	0	#NUM!	0.6436235	2	0	0	0	0	0
PRSS36	0.9642857	-0.052467	0.9462086	26	24	34	33	23	25
PRSS41	0.3157895	-1.662965	0.2272736	5	3	11	2	3	1
LINC00629	5.5	2.4594316	0.0662998	3	0	1	6	5	11
AC010642.1	1.3333333	0.4150375	0.90049	3	0	0	0	3	1
AP001781.2	#DIV/0!	#DIV/0!	0.263214	0	0	0	0	5	1
LINC00622	0.1052632	-3.247928	0.0011979	15	10	32	1	2	3
AC010642.2	0.8016529	-0.31895	0.5590343	49	27	45	27	30	40
LINC00623	1.0578158	0.0810885	0.5482265	130	171	166	171	169	154
LINC00624	1.5433333	0.6260497	0.0204882	105	56	139	109	174	180
LINC00620	0	#NUM!	0.3912131	1	1	2	0	0	0
AC110079.1	0.8021033	-0.31814	0.1495575	297	362	387	230	290	319
AC110079.2	0.2777778	-1.847997	0.189962	2	9	7	2	1	2
ABHD12	1.042607	0.0601955	0.3179727	2692	3165	3625	3144	3545	3197
ABHD13	0.9336531	-0.099042	0.8041598	450	317	484	281	420	467
ABHD10	0.9907149	-0.013458	0.7381456	1015	979	1237	904	1121	1176
ABHD11	0.8215436	-0.283591	0.1454905	1967	2479	2693	2058	2075	1732
AC007546.1	1.25	0.3219281	0.8886369	1	2	1	0	3	2
AC092070.2	0.8971429	-0.15659	0.6243571	641	325	609	302	571	540
CU634019.6	#DIV/0!	#DIV/0!	0.8030358	0	0	0	1	0	0
LHCGR	0	#NUM!	0.5337954	0	3	0	0	0	0
BLZF2P	1	0	0.9890559	1	0	0	0	0	1
TEN1-CDK3	0.2542373	-1.975752	0.0265291	21	9	29	9	3	3
AL354892.2	0.880597	-0.183446	0.8652958	23	22	22	29	15	15
HVCN1	1.5833333	0.662965	0.2759807	5	8	23	33	15	9
AL354892.3	0.3921569	-1.350497	0.1006043	26	8	17	3	8	9
AC234644.1	0.1666667	-2.584963	0.4507335	0	0	6	0	0	1

AC040975.1	0.3333333	-1.584963	0.6715904	3	0	0	0	1	0
ZNF658B	0.95	-0.074001	0.9359917	13	16	11	6	12	20
ABHD18	1.0851927	0.1179512	0.4102554	339	312	335	262	374	434
AP003354.1	0	#NUM!	0.824326	1	0	0	0	0	0
ABHD15	0.8193833	-0.28739	0.2082936	262	316	330	224	270	250
VAC14-AS1	0.9791667	-0.030374	0.9865019	25	10	13	12	10	25
OR13C1P	0	#NUM!	0.5387425	3	0	0	0	0	0
LINC00691	#DIV/0!	#DIV/0!	0.5509701	0	0	0	0	3	0
COL25A1	1	0	0.9922023	2	0	0	0	0	2
LLPH-AS1	1.4946809	0.5798375	0.0580176	59	47	82	116	106	59
NUMB	0.9913688	-0.012506	0.7745783	3071	2689	3277	2393	3289	3277
LINC00685	0.65625	-0.607683	0.4136956	19	34	11	21	10	11
LINC00687	#DIV/0!	#DIV/0!	0.8030358	0	0	0	1	0	0
AL355877.2	0.9130435	-0.131245	0.9363558	12	11	23	12	17	13
AL355877.1	#DIV/0!	#DIV/0!	0.2116454	0	0	0	5	1	0
FLJ21408	0.6	-0.736966	0.4761438	18	2	40	16	10	10
RSBN1L	1.0995079	0.136858	0.252574	664	479	686	533	783	695
IMPDH1	0.9620127	-0.055872	0.9310594	797	1013	1191	969	1008	910
IMPDH2	0.9591408	-0.060185	0.9798784	7059	8622	9087	8123	8275	7358
MYORG	0.4592377	-1.122687	6.50E-12	1167	1354	1257	447	710	578
PCED1B-AS1	0.2142857	-2.222392	0.2305817	1	5	8	0	0	3
AC012213.3	0	#NUM!	0.5387425	3	0	0	0	0	0
VSIG2	0.1142857	-3.129283	0.0085495	12	13	10	0	2	2
MYOZ3	0.8333333	-0.263034	0.8655088	5	1	0	0	5	0
MYOZ1	1.3121019	0.3918798	0.0767351	95	109	110	144	133	135
MYOZ2	2	1	0.8147319	1	0	0	0	0	2
MUCL1	1	0	0.9890559	0	1	0	0	1	0
AL137798.1	0	#NUM!	0.824326	1	0	0	0	0	0
SP110	2.002649	1.0019096	1.30E-15	691	639	935	1407	1640	1489
PTOV1	0.9387454	-0.091194	0.9597235	344	438	573	572	346	354
AL137798.2	1	0	0.9890559	1	0	0	0	1	0
TBCEL	0.8368087	-0.25703	0.222873	749	577	880	470	664	712
TLE1P1	4	2	0.478626	1	0	0	1	1	2
TEX264	0.6303725	-0.665724	0.0006758	468	626	651	372	394	334
SP140	1.5238095	0.6076826	0.4963929	15	4	2	12	9	11
TEX261	0.9333449	-0.099518	0.75495	1660	1943	2158	1698	1877	1802
AC084033.1	0	#NUM!	0.355562	5	0	0	0	0	0
AC084033.3	0.6410256	-0.641546	0.4519432	9	21	9	10	8	7
AF111167.1	0	#NUM!	0.1967154	6	0	1	0	0	0
SCAP	0.8420032	-0.248102	0.1468651	2300	2940	3007	2165	2422	2357
AF111167.2	0.516129	-0.954196	0.0724791	40	28	56	18	17	29
SCAI	1.1453815	0.1958282	0.2222412	464	286	495	347	500	579
ATP5F1P3	#DIV/0!	#DIV/0!	0.8030358	0	0	0	0	0	1
NUS1	1.0813712	0.1128618	0.2279341	5166	4321	5420	4088	5990	6042
AC007785.3	3	1.5849625	0.3442375	2	3	1	0	0	18
AC007785.1	2	1	0.790186	0	0	1	1	1	0
AL117336.2	0.6868687	-0.541894	0.3844022	43	15	41	14	20	34
AC007785.2	#DIV/0!	#DIV/0!	0.8030358	0	0	0	0	0	1
AL117336.3	0.870229	-0.200533	0.8198523	49	14	68	33	49	32
HTR7P1	0.3095238	-1.691878	0.0662897	21	7	14	2	5	6
AL049829.3	0	#NUM!	0.824326	0	0	1	0	0	0
METTL8P1	0	#NUM!	0.824326	0	1	0	0	0	0
AC099811.6	0	#NUM!	0.3482063	0	0	6	0	0	0
AC099811.4	0.5	-1	0.7561909	0	4	0	2	0	0

AC099811.5	0.6666667	-0.584963	0.8154189	2	1	0	0	2	0
PPCDC	0.9722222	-0.040642	0.895916	154	143	207	175	193	122
AC099811.3	2	1	0.8186965	0	0	1	0	0	2
AC099811.1	2	1	0.8072994	0	1	0	0	1	1
LRRIQ3	1.4516129	0.5376568	0.5550225	7	2	22	0	24	21
LRRIQ1	0.8604651	-0.216811	0.9339152	18	6	19	25	8	4
AC004990.1	1.6666667	0.7369656	0.7364736	0	1	2	0	3	2
AL591623.1	0	#NUM!	0.2919745	1	0	5	0	0	0
AL591623.2	#DIV/0!	#DIV/0!	0.2116454	0	0	0	5	1	0
SP100	1.8754038	0.9072012	3.92E-11	712	582	873	1206	1622	1236
RASIP1	0.9932886	-0.009715	0.9177834	44	52	53	41	56	51
SCEL	3.6666667	1.8744691	0.3697529	3	0	0	0	5	6
U82695.1	10	3.3219281	0.0466046	0	2	0	5	2	13
SCD5	#DIV/0!	#DIV/0!	0.1171991	0	0	0	0	3	6
AC105265.3	#DIV/0!	#DIV/0!	0.3196487	0	0	0	0	1	4
AC105265.2	0	#NUM!	0.824326	1	0	0	0	0	0
AL513302.2	1.1578947	0.2115041	0.6013355	16	18	23	28	21	17
AL355001.1	2	1	0.834705	0	0	1	0	2	0
AL355001.2	0.6923077	-0.530515	0.7333221	3	14	9	15	1	2
AC068057.1	1.6071429	0.6844982	0.2872118	8	5	15	23	10	12
CADPS2	0.543131	-0.880628	0.0138056	96	95	122	27	75	68
CEACAMP10	1.7307692	0.7914134	0.2620502	12	6	8	17	19	9
PRSS57	1.5	0.5849625	0.8552295	1	1	0	0	0	3
PRSS55	1	0	0.9890559	0	0	1	0	0	1
PRSS56	0	#NUM!	0.3958203	2	2	0	0	0	0
PRSS53	0.8285714	-0.271302	0.6713895	29	17	24	10	33	15
AC104041.1	0.9043478	-0.14505	0.8083974	36	40	39	24	35	45
SCG5	10	3.3219281	0.1616451	0	1	0	0	2	8
SCG3	3	1.5849625	0.6122545	0	0	1	2	0	1
SCG2	0.3636364	-1.459432	0.4591081	2	6	3	4	0	0
USP53	1.4156005	0.5014142	0.0086389	987	469	967	848	1355	1227
USP54	1.0931099	0.1284384	0.3668018	540	519	552	410	602	749
COX7A2	1.0183326	0.0262088	0.5399715	2542	3230	3392	3251	3374	2707
COX7A1	0.7222222	-0.469485	0.6886734	6	12	0	2	3	8
TPRG1LP1	0.3333333	-1.584963	0.7249542	0	0	3	1	0	0
MGAT4EP	0.7592593	-0.397335	0.4614967	28	45	35	27	36	19
SCIN	1.5675676	0.6485276	0.2744369	11	5	21	15	21	22
USP50	5	2.3219281	0.4606115	1	0	0	0	0	5
USP51	0.9672762	-0.048	0.9939014	398	251	390	260	392	353
CEACAMP11	0.2	-2.321928	0.4444947	3	0	2	0	0	1
WSCD1	1.3090909	0.3885653	0.252531	35	34	41	57	40	47
COX7B2	1.2580645	0.3312059	0.4944808	12	31	19	40	14	24
AC068880.4	0	#NUM!	0.5075476	0	0	4	0	0	0
USP35	0.9249564	-0.112543	0.7519529	308	470	368	320	332	408
USP36	0.7275238	-0.458934	0.000909	2055	2159	2304	1327	1683	1732
USP37	0.9624542	-0.05521	0.9412694	401	295	396	138	400	513
BX322557.1	1.3192182	0.3996833	0.1556079	100	103	104	73	159	173
USP38	1.0512669	0.0721291	0.4757349	585	420	692	440	683	661
USP31	0.6656151	-0.58724	0.0018642	409	415	444	236	291	317
USP32	0.8949847	-0.160065	0.4692446	1497	1193	1557	910	1352	1539
USP33	1.0602172	0.0843598	0.4576754	1546	941	1565	1032	1680	1584
USP34	1.0263653	0.0375444	0.8709689	1513	910	1294	666	1491	1658
USP39	0.8739545	-0.19437	0.2449187	2945	3498	3839	2691	3251	3044
GMPPB	0.8622201	-0.213872	0.2997521	671	662	721	588	599	584

AC103703.1	1.3333333	0.4150375	0.841436	3	0	0	4	0	0
AL132857.3	0.6470588	-0.628031	0.593203	4	9	4	3	5	3
GMPPA	1.0284611	0.0404872	0.4183142	1991	2323	2678	2394	2551	2246
AOX1	#DIV/0!	#DIV/0!	0.8030358	0	0	0	0	1	0
USP30	0.8857356	-0.175052	0.4536554	446	477	416	343	405	438
USP46	1.1426554	0.1923903	0.0993317	1747	1368	1841	1421	2080	2162
USP47	1.1671495	0.2229894	0.1247865	1802	942	1743	1491	1983	1763
USP48	1.0441627	0.0623466	0.3960047	2535	1763	2880	2081	2737	2677
CAMK1D	0.7837838	-0.351472	0.6977859	7	17	13	4	7	18
USP49	0.9471291	-0.078367	0.8787871	605	468	686	389	584	693
USP42	1.0207218	0.0295897	0.5734549	1382	1289	1624	1119	1516	1749
USP43	0.7956698	-0.329758	0.2067594	207	211	321	172	229	187
USP44	0.4444444	-1.169925	0.5047341	6	2	1	0	1	3
USP45	1.0586124	0.0821745	0.5187387	314	218	304	227	328	330
RN7SL239P	2.75	1.4594316	0.4308343	3	0	1	0	5	6
USP40	1.0261476	0.0372382	0.5229691	1142	1109	1191	1035	1364	1133
AC005244.1	1.0625	0.0874628	0.9300105	14	0	2	7	8	2
CAMK1G	1.3333333	0.4150375	0.8178289	0	2	4	0	7	1
MCPH1	0.9254019	-0.111848	0.7541563	587	379	589	392	517	530
CAMK2B	0.5714286	-0.807355	0.6012918	0	9	5	3	4	1
USP13	0.9	-0.152003	0.5973317	224	208	228	134	237	223
USP14	0.9981411	-0.002684	0.695156	3066	2734	3345	2478	3370	3280
CAMK2D	1.0202406	0.0289094	0.5822195	1702	1546	1989	1329	2105	1909
USP15	1.218543	0.2851572	0.0662982	1514	850	1562	1214	1935	1635
USP16	1.262668	0.3364754	0.0444218	745	398	791	703	980	759
USP10	0.9566172	-0.063986	0.8899386	7560	7443	8670	6139	8323	8184
AC002115.1	#DIV/0!	#DIV/0!	0.8030358	0	0	0	1	0	0
GMPR2	0.9124634	-0.132161	0.5263585	2563	2811	3171	2444	2840	2513
TEX21P	0.9405941	-0.088356	0.9967316	32	30	39	36	16	43
USP11	1.1059778	0.1453224	0.1011185	1713	1918	2040	1851	2237	2184
CAMK2A	0.75	-0.415037	0.9557496	0	1	3	3	0	0
USP12	1.0194175	0.027745	0.7235928	699	488	667	429	751	710
USP18	1.8205805	0.8643985	2.75E-08	414	318	405	698	684	688
USP19	0.7814097	-0.355849	0.0114002	2199	2419	2816	1768	2154	1887
CAMK2G	1.0215594	0.030773	0.4620667	1067	1027	1292	1085	1156	1218
USP24	1.0639907	0.0894855	0.46029	2714	1657	2505	1707	2678	2931
USP25	1.278481	0.3544307	0.0289041	891	476	1003	865	1092	1073
USP26	2	1	0.5860257	1	3	0	2	3	3
USP20	1.1196538	0.1630527	0.1585468	573	705	686	720	768	711
USP21	0.8900077	-0.16811	0.5699265	385	447	459	413	410	326
USP22	0.7848882	-0.349441	0.0151501	7107	8214	8197	4995	6614	6850
CADPS	0.3333333	-1.584963	0.4917895	2	0	7	0	3	0
EIF3KP1	3	1.5849625	0.6896151	1	0	0	0	3	0
USP28	0.8763075	-0.190491	0.3000136	1329	1119	1376	927	1184	1240
NEURL4	0.8902522	-0.167714	0.394646	962	888	1084	659	1001	952
NEURL2	1.6666667	0.7369656	0.6576667	0	2	4	0	6	4
NEURL3	1.0098684	0.0141673	0.8015312	90	100	114	113	130	64
NEURL1	3.7434211	1.9043573	3.08E-10	60	35	57	223	216	130
SCLY	1.3395062	0.4217012	0.172906	42	63	57	66	62	89
SCOC	1.2080114	0.2726341	0.0416597	1343	918	1234	1126	1624	1472
AL354993.2	1.0727273	0.1012833	0.7918239	19	21	15	22	15	22
AL355488.1	0.9587302	-0.060803	0.9787437	121	64	130	91	107	104
SCP2	1.0955653	0.1316754	0.1167736	2141	1813	2450	2012	2638	2366
PRKACG	2	1	0.6503036	3	0	0	6	0	0

EPSTI1	1.0909091	0.1255309	0.8952655	0	13	9	2	7	15
AC011365.1	0	#NUM!	0.824326	0	1	0	0	0	0
AL354993.1	0.7777778	-0.36257	0.8473379	3	1	5	1	3	3
PRKACA	0.8607595	-0.216318	0.2638213	1188	1425	1574	1184	1254	1166
PRKACB	1.0355594	0.0504103	0.654395	909	609	788	542	952	894
ZNF17	1.0027027	0.0038939	0.8003414	144	94	132	122	105	144
ZNF18	0.7989691	-0.323788	0.2839177	206	146	230	157	181	127
PRKAB2	0.8609494	-0.216	0.2599104	1940	1570	2157	1158	1849	1872
ZNF19	0.7730263	-0.371411	0.3164139	122	66	116	48	99	88
RC3H1	0.9809561	-0.02774	0.9030099	939	627	1007	601	963	960
ZNF10	0.9682081	-0.046611	0.9812884	125	94	127	85	161	89
AL139412.1	0.2857143	-1.807355	0.2673983	4	2	8	0	2	2
BHLHA15	#DIV/0!	#DIV/0!	0.0627253	0	0	0	12	0	0
ZNF12	0.9052298	-0.143644	0.5960168	1272	730	1153	720	1118	1018
PRKAB1	0.6956897	-0.523484	0.0016241	1036	1156	1288	833	849	739
ZNF14	1.0131004	0.0187772	0.8149654	201	84	173	120	193	151
RC3H2	1.1231251	0.1675187	0.184624	1613	1188	1666	1192	1877	1948
ZNF16	0.5888717	-0.763975	0.0063256	200	234	213	146	144	91
SIGIRR	1.3442819	0.4268358	0.0010057	1261	1335	1575	2060	1935	1612
SCO1	0.8101501	-0.303739	0.0337222	2089	2301	2605	1640	2035	1992
SCO2	1.0120482	0.017278	0.8499906	36	58	72	40	70	58
SCO2	1.0120482	0.017278	0.8499906	36	58	72	40	70	58
COX7A2P1	1	0	0.9052994	0	0	4	3	0	1
MIR4713HG	0.8333333	-0.263034	0.8942539	1	8	21	8	7	10
ZNF28	1.0631007	0.0882783	0.4881973	963	489	941	636	958	950
UNC119B	0.8878011	-0.171692	0.3530092	1532	1761	1814	1216	1616	1702
AASDH	1.1911765	0.2523872	0.1659832	292	157	299	253	347	291
NPRL2	1.1153497	0.1574961	0.188806	341	341	419	424	391	413
NPRL3	0.9552659	-0.066026	0.9797592	852	959	1028	1010	878	824
ZNF20	1.3333333	0.4150375	0.9154174	3	0	0	0	4	0
ZNF22	3.5	1.8073549	0.2586389	1	3	0	3	8	3
ZNF23	0.7699115	-0.377235	0.5095009	33	25	55	21	39	27
ZNF24	0.859739	-0.218029	0.3314398	742	600	804	399	712	734
ZNF25	0.9818182	-0.026472	0.9648948	28	8	19	16	20	18
ZNF26	0.7897503	-0.340531	0.20152	315	185	261	172	216	213
AL355102.1	0	#NUM!	0.824326	0	0	1	0	0	0
LINC01405	2.2105263	1.1443899	0.0372535	9	16	13	33	25	26
ZNF32	1.2704545	0.3453448	0.1140569	164	119	157	229	141	189
ATP1A1-AS1	0.7058824	-0.5025	0.5652204	27	14	10	17	4	15
ZNF34	1.1037037	0.1423529	0.676628	26	59	50	32	61	56
ZNF35	3.0454545	1.6066576	0.0205744	9	5	8	11	29	27
PPFIBP1	0.927683	-0.108296	0.6598194	2382	2065	2467	1384	2414	2616
PPFIBP2	1.0536694	0.0754223	0.3172946	1583	1613	1723	1475	1826	1882
AC067750.1	1.6333333	0.7078192	0.3425432	9	5	16	5	11	33
ZNF30	0	#NUM!	0.2290435	7	0	0	0	0	0
SFXN3	2.5154639	1.3308245	1.53E-12	124	157	204	404	415	401
SFXN4	1.0237412	0.033851	0.4384951	1140	1192	1501	1257	1405	1262
LGALS17A	3	1.5849625	0.6074549	1	0	0	2	0	1
SFXN1	1.0407971	0.0576888	0.349373	6698	6691	7838	5909	8152	8032
SFXN2	1.2253788	0.2932278	0.0512189	306	371	379	418	498	378
AC074286.1	0	#NUM!	0.6631647	1	0	1	0	0	0
SFXN5	0.5972763	-0.74353	0.0125759	152	179	183	116	79	112
PRKAG1	0.9171049	-0.124841	0.6006877	1210	1370	1413	1169	1300	1193
PRKAG2	0.9309309	-0.103254	0.7427682	319	322	358	238	351	341

ZNF43	0.9624924	-0.055153	0.9863976	544	416	693	401	611	579
ZNF44	1.0580645	0.0814276	0.5302209	366	212	352	280	407	297
FRG2B	1.6034483	0.6811778	0.2908287	24	20	14	7	41	45
ZNF45	0.7472426	-0.420351	0.0337887	346	357	385	228	291	294
AC087163.3	3	1.5849625	0.1909295	4	0	3	12	7	2
FRG2C	#DIV/0!	#DIV/0!	0.8030358	0	0	0	0	1	0
ZNF48	0.7790974	-0.360124	0.112163	277	260	305	205	255	196
AC087163.2	0	#NUM!	0.2084189	0	3	4	0	0	0
ZNF41	0.9671593	-0.048175	0.9930833	261	121	227	145	210	234
ENOX1-AS1	#DIV/0!	#DIV/0!	0.8030358	0	0	0	1	0	0
APC2	0.4727891	-1.080731	1.43E-06	238	319	325	113	161	143
USP3-AS1	0.6	-0.736966	0.6351825	3	7	0	0	3	3
ZNF57	1.0748503	0.1041357	0.4361602	236	157	275	202	271	245
PTRH2	0.9224673	-0.11643	0.7434817	924	854	1124	915	985	777
RAPGEF1	1.0318922	0.0452922	0.4706827	9081	11138	11607	8796	11776	12269
PTRH1	0.0769231	-3.70044	0.0681439	5	4	4	0	1	0
RAPGEF2	1.1974045	0.2599106	0.1002415	2925	2026	3063	1999	3628	3969
RAPGEF5	3	1.5849625	0.6944949	0	0	1	0	3	0
CRYAB	0.8479821	-0.237894	0.2674911	641	737	852	645	671	575
RAPGEF6	1.233677	0.3029647	0.1734523	230	91	261	228	266	224
RAPGEF3	0.576087	-0.795642	0.070233	59	50	75	43	36	27
AP003066.1	3	1.5849625	0.3642724	0	2	1	3	2	4
RAPGEF4	1.245614	0.3168571	0.4024752	28	43	43	30	44	68
AC087893.1	0.3333333	-1.584963	0.5452456	1	2	3	2	0	0
AL049830.1	#DIV/0!	#DIV/0!	0.8030358	0	0	0	0	0	1
ZNF66	0.8359788	-0.258462	0.613846	57	51	81	30	45	83
ZNF69	0.7678571	-0.38109	0.3197888	71	62	91	47	65	60
AC022098.1	0.8834951	-0.178706	0.8169232	28	31	44	31	32	28
AL049830.3	#DIV/0!	#DIV/0!	0.3981469	0	0	0	4	0	0
AL049830.4	0	#NUM!	0.824326	1	0	0	0	0	0
AC022098.4	2.3333333	1.2223924	0.5806697	0	3	0	7	0	0
MCRS1	0.8771331	-0.189132	0.3309233	1814	2030	2309	1793	1923	1681
SCTR	0.9473684	-0.078003	0.9177921	4	12	3	0	6	12
ZNF76	0.7329925	-0.44813	0.0130142	777	762	1004	648	632	584
AL662795.2	0.9176471	-0.123989	0.907077	78	21	71	46	52	58
ZNF77	1.0990338	0.1362358	0.4996605	169	122	123	136	183	136
ZNF79	1.0078628	0.0112992	0.6974303	467	454	478	418	498	494
AP000424.1	0.6	-0.736966	0.7418567	0	4	1	0	3	0
ZNF70	0.7608696	-0.394279	0.1896714	120	113	135	68	108	104
ZNF71	1.0531674	0.0747348	0.5302222	271	333	280	300	320	311
ZNF74	0.9009346	-0.150506	0.5796746	453	561	591	472	537	437
LINC01473	5.1428571	2.3625701	0.0200913	2	1	4	7	17	12
LINC01474	0.6666667	-0.584963	0.8861276	0	2	1	2	0	0
LINC01471	1.3	0.3785116	0.8074942	4	6	0	6	7	0
IL1RAP	1.107462	0.1472572	0.2667232	1676	1261	1539	1163	1798	1996
APEH	0.8833743	-0.178903	0.3226334	5824	6815	7288	5665	6456	5482
AL358234.1	0.1111111	-3.169925	0.1635091	5	2	2	0	1	0
MAP3K8	1.3495868	0.4325177	0.0001415	725	753	942	1009	1164	1093
MAP3K9	0.9818927	-0.026363	0.9705104	941	1042	944	682	983	1209
LINC01465	0.2222222	-2.169925	0.0150181	9	19	26	1	6	5
MAP3K6	1.1864952	0.2467062	0.1468941	157	206	259	271	251	216
MAP3K7	1.0893296	0.1234405	0.2749932	1958	1244	2093	1489	2156	2123
LINC01467	1	0	0.9890559	0	0	1	0	1	0
MAP3K4	1.0590953	0.0828323	0.3421036	2177	1792	2309	1702	2420	2527

MAP3K5	0.9425466	-0.085364	0.8444756	215	200	229	164	242	201
Z69666.1	0.8	-0.321928	0.8886991	5	0	0	0	0	4
NCLP1	0	#NUM!	0.276989	0	6	0	0	0	0
SLC25A15P3	0	#NUM!	0.6840298	0	0	2	0	0	0
LINC01456	1.5384615	0.6214884	0.5433888	4	2	7	7	12	1
SLC25A15P5	1.2857143	0.3625701	0.5790878	14	4	17	7	17	21
LINC01494	2.75	1.4594316	0.3998016	4	0	0	4	2	5
LINC01497	0	#NUM!	0.4521054	1	0	3	0	0	0
AL135838.1	#DIV/0!	#DIV/0!	0.8030358	0	0	0	0	1	0
AC090695.1	1.5	0.5849625	0.7634851	1	0	3	0	0	6
AL672207.1	1.5333333	0.6166714	0.5341631	2	8	5	4	6	13
LINC01484	1.3333333	0.4150375	0.9154174	3	0	0	0	4	0
LINC01485	0.5666667	-0.819428	0.4171078	7	11	12	1	5	11
API5	1.0139712	0.0200166	0.6184739	3123	2475	3349	2320	3513	3239
LINC01480	0.25	-2	0.6181385	0	0	4	0	0	1
LINC01481	1.5384615	0.6214884	0.3601657	8	8	10	20	13	7
LINC01481	1.5384615	0.6214884	0.3601657	8	8	10	20	13	7
LINC01482	0.5	-1	0.7063518	0	4	0	0	2	0
LINC01431	1.6666667	0.7369656	0.2715308	7	4	19	25	19	6
APIP	0.8290269	-0.270509	0.1773359	569	523	727	480	523	505
AC010536.1	1	0	0.998786	25	26	9	14	19	27
AC010536.2	0.2727273	-1.874469	0.1408891	8	10	4	0	2	4
AC010536.3	1.1111111	0.1520031	0.9813303	1	8	0	0	7	3
LINC01426	1.375	0.4594316	0.2094853	27	59	58	41	75	82
LINC01424	0.8837209	-0.178337	0.8419722	17	12	14	8	11	19
CERS3	0.5696203	-0.811928	0.2376304	9	35	35	10	15	20
UNC45A	0.8493083	-0.23564	0.1602829	1807	2055	2210	1659	1828	1670
CERS4	1.2028986	0.266515	0.5845596	11	36	22	33	34	16
UNC45B	1.122449	0.1666499	0.5952012	36	23	39	34	33	43
CERS5	0.9444037	-0.082524	0.8585976	1715	1756	1979	1689	1801	1657
MIR548AN	0	#NUM!	0.2919745	1	0	5	0	0	0
MIR548AT	0	#NUM!	0.824326	0	0	1	0	0	0
MIR548AC	0.8	-0.321928	0.9296989	0	5	0	4	0	0
LINC01410	0.6666667	-0.584963	0.8829054	3	0	0	2	0	0
LINC01411	0.5	-1	0.7932724	0	0	4	0	0	2
CERS1	0.2	-2.321928	0.5267773	0	0	5	0	0	1
CERS2	0.756912	-0.401803	0.0130858	1331	1680	1691	971	1269	1319
LINC01450	0.25	-2	0.5537648	4	0	0	0	0	1
AL121580.1	3	1.5849625	0.6684577	1	0	0	0	0	3
LINC01451	1.5049505	0.589716	0.0008909	252	267	289	464	395	357
PRKAA1	0.9748823	-0.0367	0.9243254	1115	798	1272	751	1151	1203
ative regulatior	#VALUE!	#VALUE!	7|nuclear spe	0|hsa04710|hum	|Thermog	tivated catalytic subunit alpha 1			
PRKAA2	1.3014528	0.380123	0.0485982	299	227	300	239	382	454
KRTAP29-1	2.0588235	1.0418202	0.2403446	7	5	5	5	19	11
AC092123.1	0.33	-1.599462	0.0071452	38	36	26	10	16	7
AC092123.2	0.5092402	-0.973582	0.0068655	137	157	193	34	81	133
MLLT1	1.0804236	0.1115971	0.1612336	3331	4060	4695	4202	4589	4267
MLLT3	1.3217778	0.4024796	0.0183071	410	258	457	381	559	547
MLLT6	0.8756477	-0.191578	0.736667	43	77	73	61	52	56
AC131182.1	0	#NUM!	0.6840298	0	0	2	0	0	0
LINC01447	1.0833333	0.1154772	0.9190046	7	0	5	2	9	2
LINC01448	1.2222222	0.2895066	0.7824671	6	1	2	8	0	3
MTUS2-AS1	#DIV/0!	#DIV/0!	0.4737294	0	0	0	0	4	0
LINC01446	0.625	-0.678072	0.8870731	0	0	8	5	0	0

uc_338	1.3333333	0.4150375	0.7233078	2	2	5	5	7	0
uc_338	1.3333333	0.4150375	0.7233078	2	2	5	5	7	0
uc_338	1.3333333	0.4150375	0.7233078	2	2	5	5	7	0
uc_338	1.3333333	0.4150375	0.7233078	2	2	5	5	7	0
uc_338	1.3333333	0.4150375	0.7233078	2	2	5	5	7	0
uc_338	1.3333333	0.4150375	0.7233078	2	2	5	5	7	0
uc_338	1.3333333	0.4150375	0.7233078	2	2	5	5	7	0
uc_338	1.3333333	0.4150375	0.7233078	2	2	5	5	7	0
uc_338	1.3333333	0.4150375	0.7233078	2	2	5	5	7	0
uc_338	1.3333333	0.4150375	0.7233078	2	2	5	5	7	0
uc_338	1.3333333	0.4150375	0.7233078	2	2	5	5	7	0
uc_338	1.3333333	0.4150375	0.7233078	2	2	5	5	7	0
uc_338	1.3333333	0.4150375	0.7233078	2	2	5	5	7	0
uc_338	1.3333333	0.4150375	0.7233078	2	2	5	5	7	0
uc_338	1.3333333	0.4150375	0.7233078	2	2	5	5	7	0
uc_338	1.3333333	0.4150375	0.7233078	2	2	5	5	7	0
uc_338	1.3333333	0.4150375	0.7233078	2	2	5	5	7	0
uc_338	1.3333333	0.4150375	0.7233078	2	2	5	5	7	0
uc_338	1.3333333	0.4150375	0.7233078	2	2	5	5	7	0
uc_338	1.3333333	0.4150375	0.7233078	2	2	5	5	7	0
uc_338	1.3333333	0.4150375	0.7233078	2	2	5	5	7	0
uc_338	1.3333333	0.4150375	0.7233078	2	2	5	5	7	0
uc_338	1.3333333	0.4150375	0.7233078	2	2	5	5	7	0
uc_338	1.3333333	0.4150375	0.7233078	2	2	5	5	7	0
uc_338	1.3333333	0.4150375	0.7233078	2	2	5	5	7	0
uc_338	1.3333333	0.4150375	0.7233078	2	2	5	5	7	0
uc_338	1.3333333	0.4150375	0.7233078	2	2	5	5	7	0
uc_338	1.3333333	0.4150375	0.7233078	2	2	5	5	7	0
uc_338	1.3333333	0.4150375	0.7233078	2	2	5	5	7	0
uc_338	1.3333333	0.4150375	0.7233078	2	2	5	5	7	0
uc_338	1.3333333	0.4150375	0.7233078	2	2	5	5	7	0
uc_338	1.3333333	0.4150375	0.7233078	2	2	5	5	7	0
uc_338	1.3333333	0.4150375	0.7233078	2	2	5	5	7	0
uc_338	1.3333333	0.4150375	0.7233078	2	2	5	5	7	0
uc_338	1.3333333	0.4150375	0.7233078	2	2	5	5	7	0
uc_338	1.3333333	0.4150375	0.7233078	2	2	5	5	7	0
SORL1	0.6779964	-0.56065	0.0022198	1007	1213	1134	568	762	944
CDKN2AIPNI	1.0091417	0.0131288	0.6132741	1057	1411	1470	1323	1393	1258
APLF	0.6722689	-0.57289	0.2359158	41	37	41	17	32	31
LINC01436	#DIV/0!	#DIV/0!	0.6411933	0	0	0	0	0	2
LINC01438	0	#NUM!	0.6436235	2	0	0	0	0	0
AC018467.1	#DIV/0!	#DIV/0!	0.8030358	0	0	0	0	0	1
LINC01433	0	#NUM!	0.3963337	1	3	0	0	0	0
AC023271.1	1.75	0.8073549	0.6614627	3	0	1	1	2	4
CERKL	0.5714286	-0.807355	0.6847946	1	2	4	0	3	1
AARS2	0.83192	-0.265483	0.1290827	993	1094	1215	744	946	1057
SDC4	0.8087126	-0.306301	0.0326602	12493	15278	15844	10685	12781	11806
SDC2	#DIV/0!	#DIV/0!	0.2677757	0	0	0	1	1	3
SDC3	1.1827957	0.2422009	0.0891135	318	461	523	469	521	550
CWF19L2	1.1329011	0.180022	0.2582965	459	231	544	418	542	438
APOO	0.6475645	-0.626904	0.0629279	102	115	132	88	63	75
APOM	1.4130435	0.4988059	0.3487889	12	12	22	16	29	20
APOF	0.1666667	-2.584963	0.3417667	1	5	0	0	1	0
APOD	0.6976744	-0.519374	0.561753	11	20	12	16	8	6

APOE	1.1109924	0.151849	0.1459051	13314	17244	18121	20084	18173	15825
APOB	#DIV/0!	#DIV/0!	0.6251462	0	0	0	1	0	1
AC017002.5	#DIV/0!	#DIV/0!	0.5509701	0	0	0	0	3	0
AC017002.2	0	#NUM!	0.824326	1	0	0	0	0	0
AC017002.1	0	#NUM!	0.2919745	1	0	5	0	0	0
CWF19L1	1.0610113	0.08544	0.3161868	780	745	868	721	975	843
SDC1	2.1112349	1.0780871	3.08E-17	1723	2207	2354	4378	4665	4224
Z97634.1	0.7850467	-0.34915	0.6058267	25	39	43	30	14	40
EIF4A1P4	1.3333333	0.4150375	0.787987	0	0	3	4	0	0
LINC01395	3.8571429	1.9475326	0.0701703	3	1	3	14	9	4
MRPS17	1.1331445	0.1803318	0.3923615	84	141	128	116	149	135
EIF4A1P5	2	1	0.7766092	0	1	0	1	0	1
EIF4A1P2	1.1363636	0.1844246	0.792699	8	10	4	12	4	9
MRPS15	0.9835069	-0.023993	0.7850855	2306	2792	2966	2659	2877	2395
LINC01398	1.75	0.8073549	0.6701206	3	0	1	1	4	2
MRPS16	1.0741197	0.1031548	0.2228457	2790	3413	3538	3443	3803	3217
LINC01391	10.5	3.3923174	0.0639138	2	0	0	0	8	13
MRPS14	0.8856672	-0.175163	0.4533943	862	1040	1133	911	939	838
LINC01393	0.75	-0.415037	0.7893691	4	3	1	0	2	4
MRPS11	0.8571987	-0.222298	0.3242559	687	889	980	742	753	696
MRPS12	0.9724813	-0.040258	0.8331289	1180	1452	1656	1603	1437	1130
MRPS10	0.8950901	-0.159895	0.3656826	1693	1603	1918	1385	1707	1575
AC006273.1	1.6	0.6780719	0.432017	9	2	9	6	18	8
EIF4A1P7	1.7142857	0.7776076	0.5788255	3	3	1	2	4	6
SDF4	1.0889652	0.1229578	0.1850813	3978	4826	5179	5531	5080	4616
SDF2	1.046525	0.0656068	0.3602584	1033	1147	1302	1253	1296	1095
LINC01389	0.9	-0.152003	0.9682572	1	5	4	5	4	0
MRPS28	0.8863636	-0.174029	0.7566769	25	43	20	13	26	39
LINC01385	0	#NUM!	0.824326	0	0	1	0	0	0
MRPS26	0.9410411	-0.08767	0.9477519	921	1245	1311	1275	1103	894
LINC01387	#DIV/0!	#DIV/0!	0.3721261	0	0	0	3	1	0
MRPS27	0.9076566	-0.139782	0.4326309	3789	4214	4483	3315	4218	3800
MRPS24	0.6071429	-0.719892	0.4822522	9	8	11	6	9	2
MRPS25	0.8288348	-0.270844	0.2366033	969	812	1257	902	846	770
MRPS22	0.9549427	-0.066514	0.9252921	1145	1152	1365	1017	1253	1227
MRPS23	0.9307595	-0.10352	0.7685099	1372	1496	1898	1411	1602	1423
MRPS21	0.9931182	-0.009963	0.6718056	2559	2617	3252	2841	3113	2416
RNU2-70P	0.2	-2.321928	0.528477	0	0	5	0	1	0
APRT	1.0445533	0.0628861	0.4343334	2352	3139	3285	3717	2950	2500
AL157888.1	4.5	2.169925	0.0265844	2	1	5	17	13	6
SDE2	0.7840142	-0.351048	0.0581095	925	898	992	524	784	899
AC118658.1	0	#NUM!	0.3473206	0	5	0	0	0	0
AC118658.2	0.5	-1	0.81106	0	2	0	0	0	1
SELPLG	0.6458333	-0.630766	0.2790598	35	27	34	13	15	34
MRPS35	0.7918138	-0.336767	0.0109055	3135	2866	3625	2231	2786	2605
MRPS36	1.0766883	0.1066006	0.225287	766	816	1039	942	1023	857
MRPS33	1.0993421	0.1366404	0.1586434	815	974	1251	1044	1241	1057
MRPS34	1.1258434	0.1710062	0.1243133	1765	2176	2432	2627	2539	2009
MRPS31	1.0452073	0.0637892	0.3835226	959	794	1189	938	1208	929
PRICKLE2	1.65625	0.7279205	0.2945365	17	5	10	10	21	22
PRICKLE3	0.8035226	-0.315589	0.1209365	875	1107	1311	914	868	864
MRPS30	0.960249	-0.05852	0.991779	2221	2154	2694	1997	2474	2317
PRICKLE1	0.25	-2	0.5148668	2	2	0	0	1	0
AC009414.2	0.8441558	-0.244419	0.7614369	15	25	37	19	36	10

PRICKLE4	10	3.3219281	0.1204481	1	0	0	3	1	6
MAP3K2	0.8517325	-0.231528	0.3268293	915	588	979	516	780	818
MAP3K3	0.7358834	-0.442451	0.0365016	300	390	408	249	281	278
AL450124.1	0.4892473	-1.031364	0.0198011	74	48	64	17	30	44
MAP3K1	0.8336629	-0.262464	0.1974281	1650	1241	1660	826	1456	1512
SORT1	0.9728987	-0.039639	0.9666343	9754	9825	10604	7868	10580	10917
APTX	1.0656004	0.0916665	0.2669498	1136	1466	1712	1474	1619	1504
TRBV20OR9-2	0.5657143	-0.821854	0.1071656	63	73	39	50	28	21
APTR	1.1284109	0.1742925	0.3005468	169	223	231	293	212	198
LINC01399	0.5970149	-0.744161	0.3438199	32	17	18	0	18	22
AC231981.1	0.5925926	-0.754888	0.4882811	6	7	14	6	7	3
NUS1P4	#DIV/0!	#DIV/0!	0.8030358	0	0	0	1	0	0
AL596330.1	#DIV/0!	#DIV/0!	0.8030358	0	0	0	0	0	1
NUS1P1	1.0769231	0.1069152	0.8894063	20	19	13	4	20	32
NCMAP	0.8565574	-0.223378	0.5583437	78	82	84	56	76	77
ZNF81	1.0220441	0.0314574	0.752209	182	127	190	116	183	211
ZNF83	0.982801	-0.025029	0.8367505	416	254	551	336	500	364
ZNF84	1.1364522	0.1845371	0.2682905	369	201	456	300	459	407
ZNF85	0.9878197	-0.01768	0.8050407	252	230	339	238	306	267
AC007497.1	1.4	0.4854268	0.721748	2	0	3	4	2	1
DNAJB12	1.0620282	0.0868221	0.2913642	937	1013	1097	1108	1097	1031
DNAJB13	0	#NUM!	0.3414317	0	2	3	0	0	0
ZNF98	1.238806	0.3089502	0.4831231	28	16	23	30	33	20
AL359313.1	0.5652174	-0.823122	0.2128438	28	21	20	4	22	13
DNAJB14	1.3624535	0.446207	0.0147369	848	474	830	667	1165	1100
SDHC	0.8642394	-0.210497	0.1953901	4038	4693	4535	3453	3981	4031
ZNF99	1.4	0.4854268	0.3788679	9	9	32	14	35	21
SDHD	0.7900578	-0.33997	0.030211	2071	1941	2385	1696	1788	1570
SDHA	0.9126027	-0.131941	0.5732525	4974	5956	6725	5198	5859	5055
SDHB	1.0740741	0.1030935	0.177187	2163	2297	2641	2514	2714	2399
ZNF90	3.5	1.8073549	0.3862188	0	1	1	1	4	2
ZNF91	1.3785005	0.4630997	0.018517	405	233	469	351	636	539
ZNF92	1.0440647	0.0622112	0.5655644	440	222	450	343	446	372
ZNF93	1.0864078	0.1195657	0.3878591	399	245	386	301	429	389
DNAJB11	0.8638196	-0.211198	0.201331	1627	1726	2103	1361	1821	1531
RPL15P2	3.3333333	1.7369656	0.3426277	0	3	0	5	2	3
RPL15P3	0.8632688	-0.212118	0.2863393	1132	1440	1509	1146	1241	1136
SDK1	0.8874408	-0.172277	0.4880263	464	596	628	419	493	586
SDK2	0.9018692	-0.14901	0.5060953	851	942	989	610	884	1015
STEAP3-AS1	0.2	-2.321928	0.2137167	5	0	10	0	1	2
LDHAP4	0.6231884	-0.68226	0.1576445	60	42	36	29	24	33
LDHAP5	#DIV/0!	#DIV/0!	0.6554062	0	0	0	0	2	0
MYSM1	0.9588294	-0.060654	0.9760449	761	408	847	445	777	711
EEF2K	0.8496766	-0.235014	0.1596676	1841	1906	1974	1263	1736	1862
APOA1-AS	2.3333333	1.2223924	0.6187626	3	0	0	0	5	2
AL358473.1	1.1428571	0.1926451	0.8851599	5	6	3	1	8	7
AL358473.2	0.3333333	-1.584963	0.6833782	1	0	2	0	0	1
BX005040.4	0	#NUM!	0.824326	1	0	0	0	0	0
EEF1D	0.6876268	-0.540302	0.0025628	558	648	766	449	469	438
IL1RL2	0	#NUM!	0.4217812	0	0	5	0	0	0
NWD1	0.3676471	-1.443607	0.0627478	24	34	10	1	11	13
NWD2	1.1666667	0.2223924	0.8648688	7	3	2	2	7	5
AC073551.1	0	#NUM!	0.824326	0	0	1	0	0	0
ZNF793-AS1	#DIV/0!	#DIV/0!	0.8030358	0	0	0	0	1	0

WARS2	1.1899907	0.2509503	0.0541878	353	310	416	380	457	447
AC079336.1	0	#NUM!	0.1265638	5	0	4	0	0	0
AC079336.2	1.5	0.5849625	0.8695629	0	2	0	0	0	3
AC079336.3	0	#NUM!	0.4374838	0	4	0	0	0	0
AC079336.6	3	1.5849625	0.6238578	1	0	0	1	0	2
AC093838.1	1.4285714	0.5145732	0.6329868	6	0	8	2	9	9
AC079336.7	#DIV/0!	#DIV/0!	0.6554062	0	0	0	0	2	0
MSANTD3	0.9506745	-0.072977	0.9285636	736	711	925	684	828	743
CWH43	#DIV/0!	#DIV/0!	0.3109758	0	0	0	0	6	0
MSANTD4	0.9151163	-0.127973	0.7333145	281	213	366	177	315	295
SLCO6A1	0.3529412	-1.5025	0.3244891	10	0	7	1	2	3
AL450998.1	1.047619	0.0671142	0.8835026	4	13	4	14	3	5
MSANTD1	1.3636364	0.447459	0.6539419	4	2	5	11	4	0
MSANTD2	0.9409283	-0.087843	0.9253239	238	154	319	180	285	204
AL450998.2	0.4444444	-1.169925	0.2184162	12	7	26	8	3	9
AFAP1	0.9593922	-0.059807	0.8962163	2239	2534	2664	1785	2629	2721
DAAM1	1.3058118	0.384947	0.0197691	854	528	786	685	1078	1068
DAAM2	2	1	0.5470734	3	2	0	2	3	5
FREM1	2.4480519	1.2916342	6.93E-06	48	52	54	104	119	154
FREM2	1.1235955	0.1681228	0.487313	271	209	232	133	322	345
PVALEF	0	#NUM!	0.824326	1	0	0	0	0	0
SDSL	0.2061856	-2.277985	0.0023081	21	25	51	7	12	1
AL031663.3	#DIV/0!	#DIV/0!	0.8030358	0	0	0	0	0	1
HDAC2-AS2	0.72	-0.473931	0.6395427	8	16	1	6	6	6
AC005839.1	1.0128205	0.0183785	0.8701532	25	18	35	21	33	25
ASH2L	0.9930728	-0.010029	0.6982037	1694	1838	2098	1651	1942	1998
ASGR1	0.9069767	-0.140863	0.9948478	11	37	38	48	13	17
ASGR2	0.3157895	-1.662965	0.2394421	3	11	5	3	0	3
AC004854.2	1.6666667	0.7369656	0.7792639	2	1	0	0	0	5
AC092851.1	2.3333333	1.2223924	0.4190145	2	3	1	5	9	0
RPL14P1	1.000565	0.0008149	0.665785	527	573	670	635	600	536
RPL14P3	1.5625	0.6438562	0.2991373	6	12	14	20	18	12
ASAP1-IT2	2.3333333	1.2223924	0.5271605	2	0	1	4	3	0
ASH1L	0.9324003	-0.100979	0.7525213	1509	908	1370	696	1338	1497
FSCN1P1	4	2	0.5760976	1	0	0	0	4	0
AATBC	0.6792453	-0.557995	0.4704773	25	15	13	5	7	24
AC087164.2	0	#NUM!	0.824326	0	1	0	0	0	0
ERMP1	1.0114897	0.0164816	0.6628245	3298	3156	3555	2540	3577	4007
AL359697.1	2.1666667	1.1154772	0.3868019	2	1	3	4	5	4
AC244250.3	#DIV/0!	#DIV/0!	0.2327526	0	0	0	0	0	7
HOXB-AS3	0	#NUM!	0.5337954	0	3	0	0	0	0
AL357500.1	0	#NUM!	0.824326	1	0	0	0	0	0
LINC01352	#DIV/0!	#DIV/0!	0.15395	0	0	0	0	1	7
LINC01353	#DIV/0!	#DIV/0!	0.5509701	0	0	0	0	3	0
AC145285.7	0.6352941	-0.654503	0.3340029	15	27	43	9	15	30
LINC01348	0	#NUM!	0.824326	0	0	1	0	0	0
AC145285.1	#DIV/0!	#DIV/0!	0.6554062	0	0	0	0	2	0
AC145285.2	0.6829268	-0.550197	0.552093	15	8	18	12	7	9
AC145285.4	0.7222222	-0.469485	0.7657034	14	0	4	6	7	0
AC145285.5	#DIV/0!	#DIV/0!	0.2164227	0	0	0	3	0	3
AC145285.6	0.5	-1	0.8188512	2	0	0	0	1	0
LINC01347	0.6	-0.736966	0.6614123	4	1	10	3	6	0
LINC01340	#DIV/0!	#DIV/0!	0.3109758	0	0	0	0	6	0
MAP2K3	1.0684863	0.0955684	0.2365361	1749	2022	2201	2149	2261	1971

LINC01341	0.8888889	-0.169925	0.9295731	2	15	10	12	7	5
MAP2K4	0.9924966	-0.010866	0.7878382	1046	783	1103	770	1082	1058
AC108752.1	1.25	0.3219281	0.572358	13	23	16	17	34	14
MAP2K1	0.9308574	-0.103368	0.6583237	1553	1786	1723	1335	1703	1674
MAP2K2	1.1381593	0.1867025	0.1301216	1354	1597	1732	2071	1830	1429
RALGAPA1	0.899723	-0.152447	0.6079099	754	430	621	323	586	715
RALGAPA2	0.8835363	-0.178639	0.3783611	1978	1718	1971	1129	1847	2031
AC130352.1	0.6666667	-0.584963	0.856923	1	0	2	0	2	0
LINC01337	2	1	0.5879726	1	0	2	4	2	0
LINC01339	0.4	-1.321928	0.6095056	0	5	0	0	2	0
PPEF1	2.75	1.4594316	0.3759326	3	0	1	9	0	2
LINC01335	#DIV/0!	#DIV/0!	0.5070017	0	0	0	3	0	0
LINC01376	1.1948052	0.2567754	0.502845	21	24	32	31	37	24
AC008575.2	0.6	-0.736966	0.8149364	0	0	5	0	2	1
LINC01372	0.5	-1	0.7069023	1	0	5	0	3	0
MAP2K7	0.7985915	-0.32447	0.0672388	815	1040	985	685	808	775
MAP2K5	0.8343146	-0.261337	0.3131065	392	352	445	366	345	281
MAP2K6	1.156051	0.209205	0.1991891	213	180	235	188	266	272
AP002478.1	0.8	-0.321928	0.7106669	19	16	5	14	13	5
LINC01360	#DIV/0!	#DIV/0!	0.6554062	0	0	0	0	2	0
AC055713.1	0.9635036	-0.053638	0.9961843	126	54	94	84	101	79
LINC01359	0	#NUM!	0.4374838	0	4	0	0	0	0
LINC01355	0.7424242	-0.429684	0.2951011	136	55	139	76	78	91
LINC01356	1.596402	0.674824	5.38E-08	2541	2561	2958	4607	4406	3854
LINC01358	0	#NUM!	0.5781912	0	0	3	0	0	0
GAL3ST1	1.3846154	0.4694853	0.17053	38	79	52	100	58	76
ASH1L-AS1	1.0582011	0.0816138	0.6420704	58	48	83	61	72	67
LINC01305	0.4193548	-1.253757	0.2295833	15	10	6	0	5	8
GAL3ST2	0	#NUM!	0.5378194	0	2	1	0	0	0
LINC01300	6.75	2.7548875	0.0276535	2	1	1	4	12	11
LINC01301	0.40625	-1.29956	0.2149338	8	22	2	2	3	8
GAL3ST4	0.4210526	-1.247928	0.1691243	8	12	18	4	7	5
AC007842.1	0.5	-1	0.8285813	0	0	4	2	0	0
LEPROT	1.1210981	0.1649125	0.1114637	2501	1929	2746	2118	3138	2789
LINC01331	#DIV/0!	#DIV/0!	0.5415473	0	0	0	0	2	1
WASF4P	0	#NUM!	0.4460243	4	0	0	0	0	0
ABLIM1	0.377907	-1.403897	3.41E-06	244	190	254	50	77	133
ABLIM2	0.859545	-0.218355	0.3600126	293	335	383	263	308	298
ABLIM3	0.952381	-0.070389	0.9061303	65	75	70	46	86	68
LINC01327	0	#NUM!	0.6840298	0	0	2	0	0	0
Z95331.1	0.6029412	-0.729911	0.2521311	21	21	26	12	15	14
LINC01322	0.64	-0.643856	0.6620555	2	9	14	11	2	3
LINC01315	2.025	1.0179219	0.067539	12	12	16	42	16	23
LINC01311	0.5882353	-0.765535	0.0566007	62	75	84	40	62	28
LINC01312	0	#NUM!	0.5387425	3	0	0	0	0	0
AQP8	1	0	0.9890559	1	0	0	0	1	0
AQP6	0	#NUM!	0.6840298	0	0	2	0	0	0
AQP7	#DIV/0!	#DIV/0!	0.055816	0	0	0	5	1	4
AQP4	0	#NUM!	0.5337954	0	3	0	0	0	0
AQP2	0	#NUM!	0.824326	0	0	1	0	0	0
AQP3	4.7063492	2.2346084	3.18E-14	50	24	52	145	221	227
AQP1	0.547619	-0.868755	0.2353532	19	11	54	12	21	13
AC244157.2	1	0	0.9760479	0	2	0	2	0	0
PCMTD1P3	0.4	-1.321928	0.3573473	5	4	11	5	3	0

AL591438.2	0.3333333	-1.584963	0.553241	0	5	1	2	0	0
AC051649.2	1	0	0.9920196	0	3	0	2	1	0
Z97633.1	1	0	0.9265084	4	7	12	5	1	17
AC009319.1	#DIV/0!	#DIV/0!	0.8030358	0	0	0	0	0	1
CAGE1	2.6666667	1.4150375	0.1708701	4	5	3	2	13	17
AC025682.1	2.2857143	1.1926451	0.3909966	2	0	5	3	12	1
RTEL1	1.0666667	0.0931094	0.8365113	3	3	9	7	3	6
AC012442.1	1.6666667	0.7369656	0.7096551	0	0	3	1	0	4
AC012442.2	0.25	-2	0.5455026	0	4	0	0	0	1
STRIP1	0.7705408	-0.376057	0.0153078	1033	1137	1177	754	910	915
STRIP2	0.8681785	-0.203936	0.3034635	1847	1434	2143	1239	1680	1790
AL672291.1	3	1.5849625	0.3928756	2	2	0	0	6	6
OCEL1	1.0225806	0.0322146	0.5940164	417	497	636	500	652	433
VN1R96P	0	#NUM!	0.5337954	0	3	0	0	0	0
AC009163.5	1	0	0.9890559	1	0	0	0	1	0
AC009163.3	0.1666667	-2.584963	0.0440036	12	2	16	2	1	2
AC009163.7	0.9487179	-0.075949	0.9158255	32	20	26	9	31	34
LINC01277	#DIV/0!	#DIV/0!	0.8030358	0	0	0	0	1	0
LINC01270	0.6958525	-0.523146	0.1762662	58	71	88	39	68	44
LINC01271	1.8333333	0.8744691	0.3098955	6	0	12	13	11	9
LINC01273	0.9310345	-0.103093	0.9247802	36	27	24	30	21	30
LINC01267	1.6666667	0.7369656	0.7526548	2	0	1	0	3	2
ERMAP	0.3882353	-1.364997	0.0869967	37	16	32	13	1	19
LINC01269	0.2	-2.321928	0.2626522	2	3	5	0	0	2
AC104435.2	0	#NUM!	0.1272889	0	1	9	0	0	0
LINC01262	#DIV/0!	#DIV/0!	0.5509701	0	0	0	0	3	0
SCAND2P	0.4910714	-1.025995	0.0924424	41	19	52	23	12	20
LINC01257	0	#NUM!	0.6385096	0	2	0	0	0	0
SDCCAG8	1.1042553	0.1430738	0.2501212	325	248	367	313	361	364
LINC01297	1.0385852	0.0546196	0.5938541	213	116	293	194	194	258
LINC01297	1.0385852	0.0546196	0.5938541	213	116	293	194	194	258
LINC01298	0	#NUM!	0.6385096	0	2	0	0	0	0
SDCCAG3	1.1487452	0.2000589	0.050441	1658	1966	2393	2332	2459	2121
LINC01293	1.7272727	0.7884959	0.503852	5	1	5	2	13	4
LINC01290	#DIV/0!	#DIV/0!	0.6411933	0	0	0	0	0	2
LINC01291	1.8695652	0.9027028	0.3083978	7	15	1	28	7	8
AL355581.1	0.6666667	-0.584963	0.7771994	3	3	0	0	0	4
NOP14-AS1	0.9909366	-0.013135	0.7964356	182	221	259	213	223	220
AP001157.1	0.8231047	-0.280852	0.4657611	120	68	89	71	88	69
LINC01285	0.0333333	-4.906891	0.002788	12	8	10	0	0	1
TM4SF18	#DIV/0!	#DIV/0!	0.8030358	0	0	0	1	0	0
TM4SF19	#DIV/0!	#DIV/0!	0.5371397	0	0	0	0	0	3
AC097066.1	#DIV/0!	#DIV/0!	0.8030358	0	0	0	1	0	0
LINC01284	0	#NUM!	0.824326	0	0	1	0	0	0
LINC01278	0.9459854	-0.08011	0.8807403	905	800	1035	812	988	792
ABHD11-AS1	1.1428571	0.1926451	0.9437677	0	5	2	0	7	1
MYRFL	0.1818182	-2.459432	0.3184074	0	0	11	0	1	1
AC068580.1	0.5	-1	0.3947153	9	16	3	2	0	12
AC068580.3	0.5	-1	0.5789995	5	3	0	0	2	2
AC068580.4	0.5789474	-0.788496	0.522597	5	6	8	2	2	7
PCNAP3	#DIV/0!	#DIV/0!	0.8030358	0	0	0	1	0	0
PCNAP4	0.5789474	-0.788496	0.5144693	4	7	8	3	3	5
HTR5BP	2.75	1.4594316	0.4176522	1	0	3	0	8	3
AC025588.1	0.5104167	-0.970253	0.121667	41	14	41	18	16	15

AJ239328.1	#DIV/0!	#DIV/0!	0.8030358	0	0	0	0	1	0
C15orf61	0.5102881	-0.970616	0.0073199	70	74	99	37	43	44
C15orf65	0.6666667	-0.584963	0.3636058	20	27	16	10	21	11
C15orf62	0.3636364	-1.459432	0.0426404	19	16	31	7	7	10
LONRF3	0.1538462	-2.70044	0.1528547	2	5	6	0	2	0
LONRF1	1.1563016	0.2095177	0.102437	670	500	647	582	740	779
LONRF2	0	#NUM!	0.6604275	0	1	1	0	0	0
AC092757.2	2.8	1.4854268	0.2795918	0	2	3	6	2	6
AC092757.3	2	1	0.713121	0	2	0	2	2	0
TUBB3P1	#DIV/0!	#DIV/0!	0.6411933	0	0	0	0	0	2
SELE	0	#NUM!	0.824326	0	0	1	0	0	0
SELP	0	#NUM!	0.2515625	5	0	1	0	0	0
SEM1	1.0875026	0.1210189	0.1777841	1390	1459	1928	1765	1926	1504
SELL	0.7662338	-0.384143	0.5468662	16	30	31	15	21	23
BAMBI	0.9774515	-0.032903	0.7930526	1129	1194	1491	1251	1309	1168
CRYL1	0.8367953	-0.257053	0.3873152	308	364	339	326	233	287
C15orf39	0.8303426	-0.268221	0.1657075	1019	1306	1353	1019	1029	1006
AC002553.4	0.4444444	-1.169925	0.412085	5	1	12	1	4	3
SMG1P1	0.6242424	-0.679822	0.0065875	475	349	496	170	265	389
SMG1P2	0.6639004	-0.590961	0.0616734	117	176	189	112	86	122
SMG1P3	0.75	-0.415037	0.1969565	134	155	159	79	95	162
SMG1P4	0.5	-1	0.692805	1	2	1	1	1	0
SMG1P5	0.5658915	-0.821403	0.1340209	49	26	54	32	22	19
SMG1P6	0.5384615	-0.893085	0.2837815	17	10	12	5	8	8
SMG1P7	1.0659341	0.0921182	0.5951742	103	55	115	94	110	87
OPLAH	1.3282443	0.4095205	0.1389917	83	108	71	113	135	100
AC002553.1	0.4285714	-1.222392	0.5701525	4	1	2	0	0	3
C15orf40	0.9159292	-0.126692	0.6895507	356	321	453	295	402	338
C15orf41	0.8477331	-0.238318	0.3377659	451	286	432	269	391	331
AC025442.1	0	#NUM!	0.824326	1	0	0	0	0	0
C15orf48	2.6666667	1.4150375	0.4723932	2	0	1	3	5	0
MYRIP	0.8804348	-0.183712	0.7100501	49	60	75	28	62	72
FLJ16779	0.4393939	-1.186413	0.0295769	38	31	63	25	20	13
TDGF1P6	0.5	-1	0.8652929	0	0	2	0	1	0
TDGF1P7	4	2	0.5760976	1	0	0	0	4	0
TDGF1P4	#DIV/0!	#DIV/0!	0.3597364	0	0	0	0	0	5
TDGF1P5	#DIV/0!	#DIV/0!	0.4909737	0	0	0	2	0	1
TDGF1P2	1	0	0.9890559	0	1	0	0	1	0
C15orf52	0.6734694	-0.570316	0.4387895	16	18	15	4	21	8
AC005838.2	0.6333333	-0.658963	0.5154555	5	9	16	4	8	7
C15orf56	0.8541667	-0.22741	0.7954049	17	12	19	11	14	16
NXF1	0.8821488	-0.180906	0.2870368	1796	2005	2249	1563	1939	1835
NXF3	1	0	0.971885	1	1	0	0	2	0
NXF4	0.25	-2	0.3607059	5	3	0	0	2	0
CRYGS	0.90625	-0.142019	0.8992466	23	1	8	8	19	2
CRYGN	1.1818182	0.2410081	0.8827232	3	4	4	0	11	2
AC005682.1	3.5454545	1.8259706	0.0420209	2	2	7	13	18	8
AC092611.3	1.2834646	0.3600435	0.1681287	96	62	96	139	106	81
AC092611.1	0.8823529	-0.180572	0.9199586	4	9	4	8	3	4
CSNK1A1L	0	#NUM!	0.824326	1	0	0	0	0	0
AC092611.2	0.890411	-0.167457	0.72479	45	58	43	19	49	62
ADAMDEC1	#DIV/0!	#DIV/0!	0.8030358	0	0	0	0	1	0
ZFYVE9	0.8916031	-0.165527	0.5599719	469	371	470	191	491	486
ARAF	0.9535706	-0.068588	0.9329442	1277	1519	1727	1301	1584	1428

C22orf24	0.5	-1	0.4376618	3	10	3	2	2	4
C4BPA	0	#NUM!	0.1395282	5	3	0	0	0	0
C22orf23	0.6509434	-0.619396	0.0549519	93	92	133	62	72	73
C4BPB	1.325	0.4059924	0.5169195	15	8	17	18	3	32
ZFYVE1	0.987638	-0.017946	0.7833472	681	762	822	689	743	805
LINC01627	#DIV/0!	#DIV/0!	0.8030358	0	0	0	0	1	0
EDDM13	0.5142857	-0.959358	0.3003011	7	12	16	4	10	4
LINC01629	0.5555556	-0.847997	0.5324168	7	5	6	0	10	0
LINC01623	0.3773585	-1.405992	0.0850393	22	13	18	10	4	6
LINC01624	0.5	-1	0.5732264	3	3	4	1	0	4
LINC01625	0.1666667	-2.584963	0.3630032	4	0	2	0	1	0
ENPP7P10	0.6666667	-0.584963	0.8484366	0	2	1	0	0	2
ENPP7P12	0	#NUM!	0.1038898	0	4	6	0	0	0
AC021851.1	1.225	0.2927817	0.5558119	13	6	21	23	15	11
AC013270.1	2.5	1.3219281	0.609118	2	0	0	2	3	0
AL354751.3	#DIV/0!	#DIV/0!	0.8030358	0	0	0	1	0	0
C22orf34	0.2631579	-1.925999	0.1753772	8	8	3	3	0	2
C22orf39	0.7755102	-0.366782	0.0911098	423	462	585	403	386	351
C22orf42	0	#NUM!	0.6840298	0	0	2	0	0	0
LINC01618	5	2.3219281	0.3864151	0	1	0	3	0	2
AL354751.1	0.5	-1	0.81753	2	0	0	0	0	1
LINC01619	1.4230769	0.5090136	0.4951495	12	2	12	8	10	19
LINC01612	1.645	0.7180876	0.0089461	78	58	64	107	115	107
LINC01615	3.5	1.8073549	0.4618526	0	0	2	0	0	7
LINC01611	0.3333333	-1.584963	0.637755	1	2	0	0	0	1
C22orf46	1.0503164	0.0708239	0.3477454	1082	1037	1200	971	1319	1196
VN2R19P	0	#NUM!	0.4374838	0	4	0	0	0	0
SDR39U1	1.0643275	0.0899421	0.6477698	33	62	76	64	50	68
EDDM3A	0	#NUM!	0.1286255	0	4	5	0	0	0
SLCO5A1	0.6666667	-0.584963	0.8829054	3	0	0	2	0	0
MEM132D-AS	0.1052632	-3.247928	0.0670579	3	4	12	0	0	2
LINC01645	1.1149425	0.1569693	0.6780566	27	19	41	20	50	27
MCTS1	0.9780153	-0.032071	0.8246386	2057	2084	2409	2061	2356	1989
AC004951.3	0.6666667	-0.584963	0.7817788	7	0	2	3	0	3
AL662797.1	0.3181818	-1.652077	0.2071445	6	8	8	0	1	6
AC004951.1	1.5	0.5849625	0.7522999	3	0	1	2	0	4
AC004951.2	0.7142857	-0.485427	0.6615064	9	13	6	12	4	4
SETX	1.2272592	0.2954399	0.0849376	1381	740	1232	970	1600	1545
AC022106.1	#DIV/0!	#DIV/0!	0.5509701	0	0	0	0	3	0
AC068389.1	#DIV/0!	#DIV/0!	0.5371397	0	0	0	0	0	3
AC068389.2	#DIV/0!	#DIV/0!	0.8030358	0	0	0	0	0	1
LINC01634	1	0	0.9890559	0	0	1	0	1	0
LINC01637	0	#NUM!	0.4374838	0	4	0	0	0	0
LINC01630	0.1428571	-2.807355	0.3234806	2	0	5	0	1	0
ARF3	0.8817583	-0.181545	0.2921821	4773	5954	6289	4370	5481	5153
ARF4	1.0505477	0.0711416	0.2386099	8119	8410	9308	8133	9916	9094
ARF1	0.8701705	-0.20063	0.2481664	11559	13839	14901	11405	12273	11389
IGHV1-45	0.5	-1	0.5880344	2	3	3	0	1	3
IGHV1-46	1.4615385	0.5474878	0.6592416	1	5	7	0	11	8
AREG	0.2857143	-1.807355	0.4142849	2	5	0	1	1	0
AC007495.1	0.4516129	-1.146841	0.3247586	3	19	9	0	2	12
ARG2	1.2329749	0.3021434	0.1712895	89	72	118	102	127	115
AL031666.3	0.2666667	-1.906891	0.262944	11	0	4	0	3	1
ARG1	2.3333333	1.2223924	0.4613047	4	0	2	0	9	5

AC011461.1	0.6666667	-0.584963	0.8466706	0	0	6	0	3	1
AL031666.1	0.7083333	-0.4975	0.6333346	12	4	8	3	7	7
AL031666.2	0.25	-2	0.2225846	4	5	7	0	0	4
ARF5	0.9133453	-0.130768	0.6327125	2672	3079	3181	2874	2891	2393
ARF6	0.9464067	-0.079468	0.8387221	4496	4823	5403	4329	4996	4608
STAU1	0.9096786	-0.136571	0.4525846	6591	6997	8223	5489	7130	7222
STAU2	1.1039501	0.142675	0.221175	501	395	547	442	617	534
SEZ6	0.7142857	-0.485427	0.8898223	1	0	6	2	0	3
IGHV1-24	#DIV/0!	#DIV/0!	0.3831122	0	0	0	0	5	0
LINC01605	0.2	-2.321928	0.453612	0	5	0	0	1	0
LINC01606	#DIV/0!	#DIV/0!	0.8030358	0	0	0	0	0	1
LINC01607	0	#NUM!	0.5172237	1	2	0	0	0	0
STAT4	0.3157895	-1.662965	0.2801303	11	0	8	0	3	3
LINC01602	1.1111111	0.1520031	0.9201868	4	1	4	0	7	3
RPL13P2	1	0	0.9804516	1	1	0	0	1	1
STAT6	0.7256703	-0.462614	0.0016171	1886	2342	2523	1434	1830	1635
RPL13P4	0	#NUM!	0.6385096	0	2	0	0	0	0
RPL13P5	1.4583333	0.5443205	0.4400767	8	4	12	12	6	17
LINC01600	2.8	1.4854268	0.1383464	3	2	5	13	2	13
STAT1	1.9673148	0.9762279	1.07E-15	6159	5263	6629	8971	13395	13146
STAT2	1.3159772	0.3961345	6.27E-05	2163	2235	2612	2615	3397	3213
STAT3	0.8127652	-0.299089	0.0839259	1778	2054	2059	1159	1754	1875
AL139806.1	#DIV/0!	#DIV/0!	0.8030358	0	0	0	0	1	0
C22orf15	3	1.5849625	0.3404784	0	4	1	5	10	0
AC009902.2	0	#NUM!	0.5075476	0	0	4	0	0	0
AC009902.3	0	#NUM!	0.824326	0	0	1	0	0	0
UBAP1	1.2596829	0.3330606	0.0016407	1438	1682	1863	1959	2207	2111
UBAP2	0.9848454	-0.022031	0.8244414	2978	3244	3742	2589	3533	3691
ERO1A	0.9556774	-0.065404	0.8700099	3729	3339	4055	2660	3978	3992
ERO1B	0.8478478	-0.238123	0.3703623	337	263	399	267	272	308
HEPHL1	0.6556777	-0.608941	0.0778285	96	81	96	37	78	64
AC010632.1	2.3333333	1.2223924	0.5744178	1	0	2	0	0	7
AC010632.2	4	2	0.5532124	1	0	0	0	0	4
KHNYN	0.7886688	-0.342508	0.05554	1254	1361	1374	736	1160	1250
ADCYAP1	#DIV/0!	#DIV/0!	0.6411933	0	0	0	0	0	2
NPTXR	1	0	0.9763403	4	0	0	3	1	0
LINC01697	0.078125	-3.678072	0.0002957	14	28	22	1	0	4
LINC01698	0	#NUM!	0.4324683	2	0	2	0	0	0
ARL9	#DIV/0!	#DIV/0!	0.2796611	0	0	0	4	1	0
Vax2os1_3	0.3666667	-1.447459	0.2136558	5	7	18	0	5	6
ARL6	1.1872792	0.2476592	0.3194524	83	60	140	75	143	118
ARL3	1.3298969	0.4113144	0.008507	327	437	400	504	548	496
ARL1	0.946849	-0.078794	0.8109028	2428	2107	2558	1897	2470	2349
ARL2	0.9572799	-0.062987	0.9759071	332	373	442	390	401	307
RAP1GAP	1.0245499	0.0349903	0.6256444	160	182	269	181	220	225
BCL2L12	1.1019317	0.1400348	0.16686	1567	1952	2279	2346	2126	1917
BCL2L13	0.8683949	-0.203577	0.1915317	2493	2682	2887	2080	2485	2436
SBNO1	1.084845	0.117489	0.3459846	4083	2405	4096	2735	4437	4310
SBNO2	0.9321442	-0.101375	0.8167389	766	872	941	832	766	806
BCL2L11	1.0379134	0.0536861	0.5412522	1102	1293	1324	941	1416	1503
CPAMD8	1.0342857	0.0486348	0.8735764	47	89	39	52	71	58
NPTX1	#DIV/0!	#DIV/0!	0.4501902	0	0	0	0	0	4
NPTX2	1	0	0.9922023	2	0	0	0	0	2
BCL2L14	0	#NUM!	0.5045503	1	1	1	0	0	0

BCL2L15	0	#NUM!	0.6840298	0	0	2	0	0	0
TCP1P3	0	#NUM!	0.6385096	0	2	0	0	0	0
LINC01671	0.9444444	-0.082462	0.9847646	80	47	125	52	76	110
NXT1	0.9035964	-0.14625	0.6692945	553	680	769	661	631	517
COX4I1	0.9635699	-0.053539	0.9053561	15600	18675	21009	18926	18458	15886
COX4I2	2	1	0.8147319	1	0	0	0	0	2
NXT2	1.1382879	0.1868655	0.2121072	778	573	775	540	936	944
MHENCR	1.46875	0.5545889	0.043633	77	101	110	186	147	90
AL139316.1	0.5	-1	0.7063518	0	4	0	0	2	0
ARNT	0.8135537	-0.29769	0.0553945	1778	2117	2155	1385	1769	1768
LINC01656	0	#NUM!	0.3320541	3	0	2	0	0	0
LINC01659	0.2	-2.321928	0.2650324	5	1	4	0	0	2
FAM167A-AS1	#DIV/0!	#DIV/0!	0.8030358	0	0	0	0	1	0
AL513548.3	#DIV/0!	#DIV/0!	0.6411933	0	0	0	0	0	2
LINC01694	1.2439479	0.314926	0.0631669	163	153	221	209	224	235
LINC01695	0	#NUM!	0.5337954	0	3	0	0	0	0
AC004461.1	1	0	0.9861498	0	2	0	0	0	2
PRIMPOL	1.0230061	0.0328148	0.6553902	218	169	265	180	241	246
CNNM1	1	0	0.9890559	0	0	1	0	1	0
PCBD1	0.9532258	-0.06911	0.9545604	170	202	248	241	199	151
PCBD2	0.8895582	-0.168839	0.6306847	144	195	159	150	166	127
CNNM2	1.3717224	0.4559885	0.0002275	633	623	689	792	916	960
CNNM3	0.9964286	-0.005162	0.7371307	718	733	789	677	826	729
LINC01686	1.0945946	0.1303966	0.7284249	52	42	54	27	91	44
CNNM4	0.9531286	-0.069257	0.8574628	2679	3007	3104	2393	3068	2917
LINC01687	0.8024691	-0.317482	0.6160608	26	31	24	5	21	39
LINC01681	1	0	0.9890559	1	0	0	1	0	0
AC000403.1	0.125	-3	0.1518016	1	0	15	0	1	1
METAP1D	0.8956743	-0.158954	0.6184091	276	223	287	229	235	240
AL008721.1	2.3333333	1.2223924	0.429157	5	1	0	12	2	0
AL008721.2	1.012987	0.0186157	0.8422265	22	8	47	20	34	24
AP000916.1	#DIV/0!	#DIV/0!	0.6411933	0	0	0	0	0	2
LINC01678	3	1.5849625	0.6944949	0	0	1	0	3	0
LINC01679	5	2.3219281	0.4094329	1	0	0	5	0	0
OTX2-AS1	0.7368421	-0.440573	0.7705207	3	3	13	1	13	0
AC005050.1	4	2	0.4842289	0	0	1	3	0	1
LINC01675	#DIV/0!	#DIV/0!	0.6099294	0	0	0	2	0	0
AC109583.2	0.75	-0.415037	0.9331362	1	0	7	4	0	2
AC109583.3	0.75	-0.415037	0.8881408	2	0	2	1	1	1
AC073063.1	12	3.5849625	0.071703	0	1	0	4	4	4
TAS2R20	1	0	0.8723009	1	1	11	9	4	0
TONSL	0.9731801	-0.039221	0.8823119	804	1019	1048	944	971	879
DNHD1	1.047619	0.0671142	0.6130311	73	54	146	91	91	104
ARR3	1.4333333	0.5193742	0.4834774	5	15	10	7	16	20
NUBP1	0.8889758	-0.169784	0.4970354	367	438	474	339	457	341
NUBP2	1.0255327	0.0363735	0.5120854	1387	1870	1952	1927	1833	1582
AC010538.1	0.5714286	-0.807355	0.6685491	1	6	0	0	1	3
MYOCOS	#DIV/0!	#DIV/0!	0.0920859	0	0	0	0	3	7
BCAP29	0.992163	-0.011351	0.7447011	662	523	729	565	646	688
ARSA	1.1562705	0.209479	0.1169994	403	514	606	625	634	502
TAS2R10	0	#NUM!	0.3482063	0	0	6	0	0	0
TAS2R13	0.5	-1	0.8276497	1	0	1	0	0	1
TAS2R14	1.8372093	0.877516	0.1108198	14	7	22	34	29	16
TAS2R16	#DIV/0!	#DIV/0!	0.3981469	0	0	0	4	0	0

TAS2R19	1	0	0.9481037	0	3	1	4	0	0
AP004289.2	#DIV/0!	#DIV/0!	0.8030358	0	0	0	0	1	0
AP004289.1	0	#NUM!	0.824326	0	0	1	0	0	0
TAS2R42	1	0	0.9890559	0	0	1	0	0	1
ART5	1.1341223	0.1815762	0.3316192	114	174	219	254	179	142
ART3	0.5	-1	0.6978881	3	1	0	1	1	0
ART4	0.9466667	-0.079072	0.9140803	84	74	67	66	71	76
ART1	#DIV/0!	#DIV/0!	0.8030358	0	0	0	0	0	1
RAB11FIP1	0.899677	-0.152521	0.4408062	5197	5750	5769	3475	5475	6089
PCBP3	0.6666667	-0.584963	0.7269783	8	0	1	0	5	1
PCBP4	0.5761589	-0.795461	0.0865218	41	45	65	16	38	33
PCBP1	1.0425752	0.0601514	0.3875583	3057	4199	4347	3848	4291	3958
VN1R83P	0.0714286	-3.807355	0.0962272	4	0	10	0	1	0
ARSK	1.1321267	0.1790354	0.1384343	733	629	848	630	1014	858
PCBP2	0.9674774	-0.0477	0.8402154	3183	3377	4294	3795	3565	3141
ARSH	1.24	0.3103401	0.807522	8	15	2	1	14	16
ARSI	0.2440191	-2.034934	3.37E-05	47	88	74	12	11	28
ARSF	3.6363636	1.8624965	0.0332909	5	2	4	13	10	17
ARSG	1	0	0.8106004	149	123	186	121	182	155
ARSD	0.8181818	-0.289507	0.8295065	8	5	9	7	5	6
RAB11FIP2	1.2102009	0.2752466	0.0937462	478	308	508	377	597	592
ARSE	0.2222222	-2.169925	0.1568242	2	9	7	2	0	2
RAB11FIP3	0.8306031	-0.267769	0.1561993	747	856	1083	696	801	734
ARSB	0.834507	-0.261004	0.1939611	702	715	855	451	677	768
RAB11FIP4	0.8230056	-0.281026	0.0513954	7891	9429	9743	6310	8063	7900
RAB11FIP5	1.1012028	0.1390802	0.1295802	1332	1622	1868	1552	1877	1881
AC002064.3	0	#NUM!	0.6840298	0	0	2	0	0	0
WWTR1	1.1409353	0.190217	0.1287006	1040	1042	1040	896	1346	1320
TAS2R2P	0.9354839	-0.096215	0.9609651	14	1	16	2	13	14
TAS2R31	1.5	0.5849625	0.7357188	2	0	2	3	3	0
VN1R84P	0.375	-1.415037	0.3979287	13	0	3	2	0	4
AC009318.3	1.5	0.5849625	0.7069368	10	1	1	0	10	8
ZBED5-AS1	0.8157895	-0.293731	0.430346	97	88	119	84	77	87
AC009318.4	0	#NUM!	0.241242	5	1	0	0	0	0
AC009318.1	0.5294118	-0.917538	0.5799477	0	4	13	0	7	2
AC009318.2	#DIV/0!	#DIV/0!	0.1415245	0	0	0	3	3	1
CETN2	1.1187584	0.1618986	0.1118737	950	922	1092	1105	1229	982
CETN3	1.1889587	0.2496986	0.0259291	711	612	905	783	983	883
AC092120.2	0.6666667	-0.584963	0.841388	0	3	3	3	0	1
AC092120.1	3	1.5849625	0.6142763	1	0	0	2	1	0
ARV1	0.8222087	-0.282423	0.1874035	548	433	667	424	471	460
STBD1	0.8181818	-0.289507	0.8596443	2	4	5	2	4	3
NCOA1	0.9937592	-0.009032	0.861728	1998	1450	2000	1186	2030	2198
NCOA2	0.8719807	-0.197632	0.4510942	796	547	727	334	685	786
NCOA5	0.826441	-0.275016	0.0697109	1480	1440	1747	1189	1374	1294
NCOA6	0.89	-0.168123	0.3832199	2978	2905	3417	2036	2906	3335
NCOA3	0.8839833	-0.177909	0.4032094	10133	7829	10577	5383	9503	10342
NCOA4	1.0506676	0.0713063	0.3941625	10626	9374	10631	8109	11957	12117
VSNL1	0.2758621	-1.857981	0.0917297	16	5	8	2	4	2
BANK1	0.8648649	-0.209453	0.8294389	17	5	15	3	13	16
AC136624.2	0	#NUM!	0.824326	0	0	1	0	0	0
AC136624.3	#DIV/0!	#DIV/0!	0.8030358	0	0	0	0	0	1
NCOA7	1.0224383	0.0320138	0.7143118	532	338	467	329	508	530
RRM1-AS1	#DIV/0!	#DIV/0!	0.4737294	0	0	0	0	4	0

CRELD1	0.9477823	-0.077372	0.9552532	931	1036	1212	1059	1046	908
CRELD2	0.9606368	-0.057937	0.9383233	1608	1964	2144	1975	1867	1649
ITFG1-AS1	0	#NUM!	0.1909345	0	6	1	0	0	0
SFI1	1.1133005	0.154843	0.3729896	134	91	181	171	158	123
VN1R87P	1.5	0.5849625	0.8253999	2	0	0	2	1	0
SAMM50	0.9813961	-0.027093	0.7945504	1771	2100	2203	2077	2055	1829
AL671710.1	0	#NUM!	0.5075476	0	0	4	0	0	0
AC009558.2	0	#NUM!	0.824326	0	0	1	0	0	0
BCAP31	0.9975735	-0.003505	0.6259745	9211	11326	12844	11384	11588	10328
NUBPL	1.0399386	0.0564983	0.5607407	739	511	703	511	792	728
HNRNPA3P11	3	1.5849625	0.6122545	0	0	1	2	0	1
HNRNPA3P12	1.5	0.5849625	0.4510166	4	9	7	12	9	9
AC126389.1	1	0	0.9922023	2	0	0	0	0	2
HNRNPA3P13	#DIV/0!	#DIV/0!	0.8030358	0	0	0	0	0	1
AC079921.2	2	1	0.7094398	0	1	1	0	0	4
AC079921.1	0.6666667	-0.584963	0.6515483	4	3	20	2	6	10
HNRNPA3P10	1.3098592	0.3894117	0.1427127	80	66	67	93	91	95
AC107926.1	0.2	-2.321928	0.4307274	0	4	1	0	1	0
KLHL36	0.7371446	-0.43998	0.0053772	1970	2265	2377	1202	1816	1856
KLHL38	#DIV/0!	#DIV/0!	0.8030358	0	0	0	1	0	0
KLHL32	0.40625	-1.29956	0.2070855	13	4	15	4	4	5
RNU2-51P	2.5	1.3219281	0.4387542	0	0	4	4	0	6
KLHL33	1	0	0.9890559	1	0	0	0	1	0
AL008627.1	1.1538462	0.2064509	0.7932078	3	5	5	7	4	4
KLHL35	2	1	0.8186965	0	0	1	0	0	2
SEPT4-AS1	#DIV/0!	#DIV/0!	0.2796611	0	0	0	4	1	0
KLHL30	0.7641654	-0.388043	0.1895248	173	243	237	192	163	144
KLHL31	0.7241379	-0.465664	0.6641611	18	2	9	7	9	5
RNU2-52P	0	#NUM!	0.5337954	0	3	0	0	0	0
BANF1	0.8965045	-0.157617	0.526386	3418	3899	4355	3739	3628	3097
BANF2	0	#NUM!	0.4374838	0	4	0	0	0	0
AC092756.1	4.5	2.169925	0.290646	1	0	1	9	0	0
KLHL40	2.3333333	1.2223924	0.4968663	2	0	1	4	2	1
KLHL42	0.8361916	-0.258095	0.1106436	1083	1026	1273	824	1080	924
UBBP3	0	#NUM!	0.824326	0	1	0	0	0	0
UBBP4	1.2619048	0.335603	0.3642501	24	25	35	37	35	34
AL161621.1	0.9090909	-0.137504	0.9529033	3	4	4	4	5	1
UBBP1	1.2148148	0.2807364	0.4099962	58	42	35	60	57	47
UBBP2	1.2444444	0.3155018	0.5520376	20	10	15	17	19	20
AC026271.4	0.5789474	-0.788496	0.0742433	51	46	74	20	36	43
KLHL18	0.7657143	-0.385122	0.0365169	654	666	780	416	546	646
KLHL14	#DIV/0!	#DIV/0!	0.2758481	0	0	0	4	0	1
KLHL15	0.7852349	-0.348804	0.1924734	491	279	422	195	343	398
AC007255.1	0.5740741	-0.800691	0.325871	11	14	29	8	7	16
KLHL17	1.1779141	0.2362343	0.3729764	37	47	79	71	52	69
KLHL10	0.4	-1.321928	0.5106547	0	5	5	0	4	0
KLHL11	0.6689655	-0.579996	0.1270657	106	96	88	30	67	97
KLHL12	0.8799634	-0.184485	0.2220644	4757	4870	5660	3787	4896	4769
KLHL13	0.7737789	-0.370007	0.2402745	98	150	141	88	120	93
AC244154.1	0.6993737	-0.515865	0.06092	139	153	187	110	111	114
BANCR	#DIV/0!	#DIV/0!	0.3721261	0	0	0	3	1	0
KLHL29	1.2245008	0.2921937	0.0254683	523	646	684	630	790	849
KLHL25	0.9222413	-0.116784	0.6913663	489	583	677	455	585	573
KLHL26	0.8301887	-0.268489	0.2335162	511	672	513	462	483	463

KLHL28	1.127907	0.1736481	0.2764718	536	335	505	367	563	622
KLHL21	1.039071	0.0552942	0.4124579	1442	1560	1605	1430	1809	1548
KLHL22	0.9034853	-0.146427	0.6735611	206	263	277	215	219	240
KLHL23	1.4000796	0.4855089	0.0011539	881	641	990	854	1316	1347
KLHL24	1.0935082	0.1289641	0.2686161	1113	782	1078	838	1225	1188
KLHL20	0.9782474	-0.031729	0.950692	579	469	561	388	553	633
AP003063.1	0	#NUM!	0.824326	1	0	0	0	0	0
LGALS3BP	1.9874505	0.9909189	7.15E-15	2159	2570	2841	5203	5253	4589
YWHAE	1.0326823	0.0463964	0.3067685	27372	29106	34091	28523	33629	31377
MCUR1	0.9266653	-0.10988	0.6785392	1462	1410	1887	1250	1600	1560
YWHAB	1.0022418	0.0032306	0.5466487	12662	12361	15124	11962	14511	13764
AC112777.1	0.5643564	-0.825321	0.1173529	36	35	30	13	21	23
CES5AP1	0	#NUM!	0.824326	1	0	0	0	0	0
SFR1	1.3410088	0.4233187	0.0052473	302	263	347	397	457	369
VN1R81P	1.3247863	0.4057597	0.3055651	55	21	41	35	59	61
YWHAQ	1.0145464	0.0208348	0.4427731	14949	14723	17625	14216	17282	16487
AC009652.1	#DIV/0!	#DIV/0!	0.8030358	0	0	0	1	0	0
YWHAG	0.8387124	-0.253752	0.0824042	7464	7848	8484	5240	7322	7396
YWHAH	1.12098	0.1647605	0.0433475	12647	14023	15494	14961	16832	15472
AC009652.2	0.25	-2	0.3609761	1	4	3	0	0	2
TBC1D8	1.0309084	0.0439162	0.4574181	800	829	1024	772	1044	919
TBC1D9	1.2144588	0.2803136	0.0460919	856	739	881	707	1162	1138
YWHAZ	0.9796323	-0.029688	0.834986	25464	22982	28195	21093	27238	26749
TBC1D1	1.1663738	0.2220302	0.0115514	2200	2065	2563	2377	2853	2734
SFPQ	0.957613	-0.062485	0.929668	17306	17089	20150	15087	18835	18311
TBC1D2	1.2083022	0.2729813	0.0088206	813	825	1036	980	1162	1089
TBC1D3	0	#NUM!	0.824326	1	0	0	0	0	0
TBC1D4	0.9583914	-0.061313	0.8772795	1511	1255	1536	896	1591	1636
TBC1D5	0.8293064	-0.270023	0.1491515	2796	2557	2966	1511	2553	2835
TBC1D7	0.9040698	-0.145494	0.5900905	382	498	496	374	500	370
AC092610.1	#DIV/0!	#DIV/0!	0.8030358	0	0	0	0	0	1
AC026271.1	1.1005803	0.1382644	0.3846281	183	143	191	176	228	165
AL162595.1	0.6629213	-0.59309	0.3243673	32	28	29	8	17	34
AL162595.2	0	#NUM!	0.284725	6	0	0	0	0	0
AC104184.1	#DIV/0!	#DIV/0!	0.5230806	0	0	0	1	2	0
SMYD3-IT1	0	#NUM!	0.824326	1	0	0	0	0	0
AC026427.1	#DIV/0!	#DIV/0!	0.2327526	0	0	0	0	0	7
VN1R80P	#DIV/0!	#DIV/0!	0.4980481	0	0	0	2	1	0
ASB8	0.8098237	-0.30432	0.1249381	469	507	612	416	465	405
ASB9	0.5829596	-0.778532	0.0407377	73	57	93	39	51	40
ASB6	0.9385513	-0.091492	0.8927865	751	1029	1133	946	932	856
ASB7	1.0130597	0.0187192	0.7374992	382	294	396	261	410	415
ASB4	2	1	0.8324799	1	0	0	0	2	0
QRICH1	0.8781796	-0.187412	0.2360078	3425	3800	4333	2971	3736	3443
ASB5	#DIV/0!	#DIV/0!	0.288876	0	0	0	3	2	0
QRICH2	1.4125874	0.4983401	0.1292782	53	42	48	82	48	72
ASB2	1.037037	0.0524674	0.8454891	5	9	13	15	9	4
ASB3	2.3928571	1.2587343	0.0662338	5	15	8	13	29	25
ASB1	0.7878788	-0.343954	0.0236178	1072	1166	1359	838	1030	966
MYT1L	2.6363636	1.3985494	0.1596771	2	6	3	6	12	11
RPL12P1	1.6666667	0.7369656	0.7011525	2	0	1	5	0	0
LINC01503	1.106383	0.1458509	0.7174713	11	14	22	17	19	16
LINC01504	0.9839572	-0.023333	0.9106224	57	42	88	40	68	76
LINC01505	1.5	0.5849625	0.8244883	1	3	0	0	5	1

LINC01505	1.5	0.5849625	0.8244883	1	3	0	0	5	1
RPL12P4	1.2287335	0.297172	0.1200612	132	194	203	199	257	194
AC018814.1	#DIV/0!	#DIV/0!	0.3096126	0	0	0	5	0	0
RPL12P6	1.4285714	0.5145732	0.7180875	0	4	3	2	4	4
RPL12P7	0	#NUM!	0.5643268	1	0	2	0	0	0
LINC01501	0	#NUM!	0.3320541	3	0	2	0	0	0
RPL12P8	1	0	0.9890559	0	1	0	0	0	1
WSB1	1.0525407	0.073876	0.3073136	7441	5577	8413	6235	8288	8034
WSB2	0.8900263	-0.16808	0.393145	991	1121	1307	891	1093	1059
AL139807.1	0.6	-0.736966	0.7543606	5	0	0	0	3	0
UBA52	0.9998039	-0.000283	0.6139938	13971	17227	19792	17818	17998	15164
FTLP15	0	#NUM!	0.2325162	2	4	0	0	0	0
FTLP14	1.0152339	0.0218122	0.6738103	241	347	331	342	303	288
LDHBP1	0.25	-2	0.5310185	0	3	1	0	1	0
LDHBP2	0.6875	-0.540568	0.6674579	5	8	3	4	2	5
FTLP12	0.6666667	-0.584963	0.6057982	11	6	7	8	6	2
NEDD4L	0.9917893	-0.011894	0.7553748	957	924	1042	873	1076	950
FTLP17	1.1538462	0.2064509	0.8595119	3	10	0	8	2	5
LINC01529	0.0625	-4	0.0575638	0	11	5	0	1	0
LINC01524	#DIV/0!	#DIV/0!	0.3981469	0	0	0	4	0	0
AL139161.2	0	#NUM!	0.824326	0	0	1	0	0	0
LINC01526	2.5	1.3219281	0.5116907	3	1	0	0	7	3
AL139161.1	1.5	0.5849625	0.8637378	2	0	0	0	0	3
LINC01521	0.8604651	-0.216811	0.7039107	48	22	16	11	29	34
FA2H	0.7464789	-0.421827	0.5144849	18	27	26	15	27	11
LINC01518	1.0480769	0.0677446	0.8056124	43	31	30	37	45	27
LINC01513	1.3333333	0.4150375	0.857432	2	1	0	2	2	0
LINC01515	1.3571429	0.4405726	0.166675	46	46	48	51	65	74
LINC01511	10.95	3.452859	6.36E-12	9	5	6	82	74	63
MIR5689HG	0.5333333	-0.906891	0.5597184	0	6	9	1	4	3
FN3KRP	0.8709339	-0.199365	0.3555031	1095	1231	1486	1131	1128	1061
SLCO4A1	1.4958814	0.5809958	7.47E-05	335	389	490	585	675	556
AC022107.1	0.9105882	-0.135129	0.7226202	136	137	152	111	137	139
NPSR1	0.4166667	-1.263034	0.583233	1	0	11	0	0	5
PLPPR1	7	2.8073549	0.3240543	1	0	0	0	0	7
PLPPR2	0.8202247	-0.285909	0.4953196	57	111	99	72	71	76
PLPPR3	1.03125	0.0443941	0.9204102	3	18	11	8	10	15
ANKRD55	0.6388889	-0.646363	0.4913811	9	9	18	6	6	11
PLPPR4	0.6666667	-0.584963	0.7555304	5	1	0	0	2	2
ASIP	0.4117647	-1.280108	0.3707614	9	2	6	1	6	0
ANKRD53	2	1	0.5372863	3	0	3	0	4	8
ANKRD54	0.9872312	-0.01854	0.7488254	420	474	594	495	492	482
ANKRD52	0.6195396	-0.690732	0.0001091	1762	1803	1952	767	1191	1460
ANKRD50	1.3711414	0.4553774	0.0145088	504	342	547	404	746	760
ANKRD66	2	1	0.7065596	1	0	1	0	0	4
AL031665.2	0.6666667	-0.584963	0.8287635	3	3	0	4	0	0
ERP44	1.2312249	0.3000943	0.002294	3185	2665	3604	3711	4159	3770
AC244394.1	0.8	-0.321928	0.8746154	5	0	0	0	1	3
ZNFX1	0.9684548	-0.046243	0.990391	1798	2028	2102	1518	2058	2165
HMGA1P6	#DIV/0!	#DIV/0!	0.8030358	0	0	0	0	0	1
HMGA1P8	0.9090909	-0.137504	0.949155	2	15	5	12	7	1
SLCO4C1	1.2088608	0.2736481	0.3325888	53	41	64	57	56	78
HMGA1P2	0.4864865	-1.039528	0.2416437	8	15	14	6	7	5
ANKRD65	0.884058	-0.177787	0.8314967	19	58	61	44	42	36

HMGA1P3	1.4285714	0.5145732	0.7742274	5	0	2	0	6	4
ANKRD62	0	#NUM!	0.6385096	0	2	0	0	0	0
HMGA1P4	2.3333333	1.2223924	0.5627837	2	0	1	0	2	5
ANKRD61	0.5323194	-0.909636	0.059572	95	68	100	10	50	80
HMGA1P1	1	0	0.9890559	0	0	1	0	0	1
ANKRD39	1.045584	0.064309	0.6144171	121	88	142	110	117	140
ANKRD37	1.5878378	0.6670636	0.046292	57	34	57	98	83	54
LINC01595	#DIV/0!	#DIV/0!	0.5371397	0	0	0	0	0	3
ANKRD35	2	1	0.3236536	4	3	4	5	9	8
ANKRD36	1.0982143	0.1351596	0.5613586	125	68	143	73	162	134
ANKRD33	0.7419355	-0.430634	0.5044572	21	81	22	39	42	11
LINC01592	1	0	0.953663	1	5	0	0	5	1
DACH2	0.2384615	-2.068172	0.0002421	52	38	40	13	7	11
DACH1	0.6870748	-0.541461	0.2215751	51	49	47	29	32	40
GRB14	0.9842105	-0.022961	0.8913331	72	43	75	59	53	75
GRAP2	1.5	0.5849625	0.8158917	4	0	0	0	2	4
ANKRD31	1.4416667	0.5277376	0.1666672	44	17	59	44	74	55
GRB10	1.0602137	0.0843551	0.2586848	1410	1645	1811	1562	1803	1794
RBMX2P5	0	#NUM!	0.824326	0	1	0	0	0	0
LINC01586	#DIV/0!	#DIV/0!	0.8030358	0	0	0	0	1	0
LINC01588	0.8635851	-0.21159	0.4162166	281	272	334	208	250	308
LINC01589	0.6666667	-0.584963	0.8318384	2	1	0	0	0	2
LINC01582	0.7692308	-0.378512	0.7774606	4	6	3	3	3	4
ANKRD49	0.8224719	-0.281962	0.2676408	500	307	528	321	368	409
LINC01585	#DIV/0!	#DIV/0!	0.1108064	0	0	0	3	6	0
ANKRD46	0.950782	-0.072814	0.8331982	764	667	743	522	779	766
ANKRD44	1.5803571	0.6602506	0.0768171	32	40	40	37	68	72
ANKRD45	1.25	0.3219281	0.8175938	3	3	2	1	2	7
MAMDC2-AS1	0.6875	-0.540568	0.8325862	0	0	16	3	8	0
BSCL2	0.7846154	-0.349942	0.6219588	17	23	25	11	13	27
ANKRD42	0.9723502	-0.040452	0.9603711	146	127	161	108	130	184
ERP29	1.0360927	0.0511531	0.4135287	4962	6322	6836	6636	6631	5507
LINC01579	2.5	1.3219281	0.5834769	1	0	1	0	2	3
ANKRD40	0.7044951	-0.505339	0.0024927	1597	1553	1811	796	1365	1334
ERP27	1.3075435	0.386859	0.0481595	201	136	180	192	234	250
GRAPL	0.4	-1.321928	0.6095056	0	5	0	0	2	0
MED4-AS1	1	0	0.987763	2	0	0	1	0	1
LINC01578	1.1102582	0.1508952	0.1942712	469	453	511	459	623	509
ANKRD17	1.0475339	0.0669969	0.5471023	3595	3124	3905	2329	3970	4830
PARP4P2	#DIV/0!	#DIV/0!	0.8030358	0	0	0	1	0	0
ANKRD16	0.8364419	-0.257663	0.4962754	154	253	290	223	168	192
ANKRD11	0.8287444	-0.271001	0.0854334	6287	6408	7462	4218	5920	6567
SUZ12P1	1.1101449	0.150748	0.2769078	226	179	285	277	253	236
ANKRD12	1.0104012	0.0149282	0.7785931	240	135	298	146	250	284
ASMT	0.45	-1.152003	0.3754126	3	10	7	5	1	3
PARP4P3	4	2	0.5101708	0	0	1	1	0	3
NDUFB1P2	3.25	1.7004397	0.303734	3	1	0	8	0	5
AC008083.1	0	#NUM!	0.5172237	1	2	0	0	0	0
AC008083.2	0	#NUM!	0.824326	0	0	1	0	0	0
ANKRD10	1.0714079	0.0995079	0.2603075	1176	847	1436	1140	1316	1250
NDUFB1P1	0	#NUM!	0.1905915	0	1	7	0	0	0
ANKRD28	0.9366463	-0.094424	0.7585744	1537	1111	1598	963	1539	1475
ANKRD26	1.3545586	0.4378228	0.0291816	246	152	293	239	402	295
ANKRD27	0.8967036	-0.157297	0.3693809	2169	1960	2454	1663	2193	2047

ANKRD24	0.8522495	-0.230652	0.3142786	572	653	731	585	542	540
ANKRD22	#DIV/0!	#DIV/0!	0.8030358	0	0	0	1	0	0
ANKRD23	2.1666667	1.1154772	0.3089423	2	0	10	3	5	18
ASNS	2	1	0.2380839	5	1	10	11	9	12
CNOT4	0.9663706	-0.049352	0.9671345	527	471	578	372	608	543
CNOT6	0.9701786	-0.043678	0.9734829	4545	3632	4532	2861	4632	4837
CNOT7	0.9829103	-0.024868	0.805309	2011	1693	2206	1687	2172	1950
CNOT1	0.8862231	-0.174258	0.4177081	9004	7674	8872	4633	8233	9777
CNOT2	0.9864446	-0.01969	0.8171667	1780	1484	1900	1389	1945	1760
CNOT3	0.9629883	-0.05441	0.9901184	1710	1982	2144	1740	2057	1823
SLC25A14P1	0	#NUM!	0.824326	1	0	0	0	0	0
CNOT8	0.8762684	-0.190555	0.2641534	2233	1885	2485	1669	2074	2043
LINC01597	0	#NUM!	0.824326	0	0	1	0	0	0
CNOT9	1.0102359	0.0146922	0.5274569	2868	2832	3288	2682	3227	3171
LINC01598	0	#NUM!	0.6436235	2	0	0	0	0	0
LINC01598	0	#NUM!	0.6436235	2	0	0	0	0	0
LINC01551	#DIV/0!	#DIV/0!	0.6099294	0	0	0	2	0	0
SGCE	0.9904014	-0.013915	0.7806043	319	356	471	328	433	374
SGCD	1.1428571	0.1926451	0.9253008	1	6	0	2	3	3
SGCA	#DIV/0!	#DIV/0!	0.1147049	0	0	0	1	2	5
SGCB	1.0919913	0.1269614	0.195867	595	541	712	582	766	670
LINC01547	1.1473684	0.1983287	0.3711145	66	97	122	97	136	94
GRASP	1.2567568	0.3297054	0.4984597	24	26	24	15	39	39
LINC01543	#DIV/0!	#DIV/0!	0.2164877	0	0	0	4	2	0
SGCG	0.6153846	-0.70044	0.7626457	2	0	11	5	1	2
LINC01545	0.4615385	-1.115477	0.5132451	0	8	5	2	4	0
TBC1D3P1	#DIV/0!	#DIV/0!	0.0093851	0	0	0	8	3	5
ELOA-AS1	1.2708333	0.3457748	0.5038395	19	15	14	20	20	21
TBC1D3P2	12	3.5849625	0.0805424	1	0	0	3	7	2
ASPN	0	#NUM!	0.824326	0	1	0	0	0	0
ASPM	1.1298701	0.176157	0.2477711	1256	624	1354	931	1424	1299
ASPH	1.0510012	0.0717643	0.4327619	2099	1472	2272	1521	2355	2265
TBC1D3P5	0	#NUM!	0.5200113	2	1	0	0	0	0
TBC1D3P7	0	#NUM!	0.6436235	2	0	0	0	0	0
ASPG	1.2	0.2630344	0.8821863	2	0	3	0	4	2
LINC01535	0.974026	-0.037968	0.9155357	49	47	58	61	47	42
LINC01537	0.5	-1	0.6899408	0	3	3	0	0	3
ASPA	0	#NUM!	0.3749909	2	1	1	0	0	0
DDR1-AS1	0	#NUM!	0.6436235	2	0	0	0	0	0
LINC01534	1.0789474	0.1096245	0.7876393	25	31	20	21	22	39
MIR155HG	0	#NUM!	0.6631647	1	0	1	0	0	0
NLGN3	0.4230769	-1.241008	0.0014374	90	76	146	53	32	47
LINC01572	0.9454545	-0.08092	0.9723378	66	47	107	71	66	71
NLGN1	2.15625	1.1085245	0.1021818	18	9	5	24	13	32
AC100810.1	0.9048843	-0.144195	0.7802255	87	127	175	101	152	99
NLGN2	1.0812604	0.112714	0.392385	166	158	279	191	236	225
AC100810.3	0.5833333	-0.777608	0.7052162	9	2	1	7	0	0
LINC01569	0.8632479	-0.212153	0.6843198	48	31	38	21	54	26
WASF1	0.7304965	-0.453051	0.0175138	446	392	572	292	386	352
WASF2	0.927477	-0.108617	0.6235824	4481	5140	5781	3717	5385	5183
WASF3	0.6143187	-0.702941	0.0122882	126	147	160	64	107	95
LINC01560	0.744186	-0.426265	0.274555	94	61	60	44	57	59
AC002066.1	0.9375	-0.093109	0.9849234	2	5	9	5	2	8
LINC01562	#DIV/0!	#DIV/0!	0.5509701	0	0	0	0	3	0

LINC01563	0	#NUM!	0.5781912	0	0	3	0	0	0
SGCZ	#DIV/0!	#DIV/0!	0.8030358	0	0	0	0	1	0
ASS1	1.2744637	0.3498903	0.0033771	902	1160	1341	1353	1526	1458
LINC01558	1.5	0.5849625	0.8307205	1	0	1	0	2	1
RHEBL1	0.752809	-0.409644	0.4759259	34	29	26	14	19	34
LINC01554	1.1428571	0.1926451	0.9222379	0	5	2	0	0	8
LINC01556	0.8125	-0.29956	0.8370669	4	3	9	1	6	6
FAAH	0.7460938	-0.422571	0.1626894	181	137	194	115	175	92
5S_rRNA	0.3333333	-1.584963	0.60684	0	0	6	0	2	0
5S_rRNA	0.3333333	-1.584963	0.60684	0	0	6	0	2	0
5S_rRNA	0.3333333	-1.584963	0.60684	0	0	6	0	2	0
5S_rRNA	0.3333333	-1.584963	0.60684	0	0	6	0	2	0
5S_rRNA	0.3333333	-1.584963	0.60684	0	0	6	0	2	0
5S_rRNA	0.3333333	-1.584963	0.60684	0	0	6	0	2	0
5S_rRNA	0.3333333	-1.584963	0.60684	0	0	6	0	2	0
5S_rRNA	0.3333333	-1.584963	0.60684	0	0	6	0	2	0
5S_rRNA	0.3333333	-1.584963	0.60684	0	0	6	0	2	0
5S_rRNA	0.3333333	-1.584963	0.60684	0	0	6	0	2	0
5S_rRNA	0.3333333	-1.584963	0.60684	0	0	6	0	2	0
5S_rRNA	0.3333333	-1.584963	0.60684	0	0	6	0	2	0
5S_rRNA	0.3333333	-1.584963	0.60684	0	0	6	0	2	0
5S_rRNA	0.3333333	-1.584963	0.60684	0	0	6	0	2	0
5S_rRNA	0.3333333	-1.584963	0.60684	0	0	6	0	2	0
5S_rRNA	0.3333333	-1.584963	0.60684	0	0	6	0	2	0
5S_rRNA	0.3333333	-1.584963	0.60684	0	0	6	0	2	0
SCARNA1	0.5	-1	0.7231878	2	0	2	0	2	0
PKD2L2	0.7254902	-0.462972	0.7048149	15	1	35	13	13	11
SCARNA3	0	#NUM!	0.5387425	3	0	0	0	0	0
AL445991.1	0.6	-0.736966	0.7458929	4	0	1	0	1	2
NADSYN1	0.8156561	-0.293967	0.0605357	1535	1584	1812	1291	1467	1264
ITGB2-AS1	0	#NUM!	0.3958203	2	2	0	0	0	0
ASTL	0.3333333	-1.584963	0.5110014	1	5	0	0	0	2
AL355974.2	1.5	0.5849625	0.8307205	1	0	1	0	2	1
AL355974.3	0	#NUM!	0.824326	1	0	0	0	0	0
NUAK1	0.8003565	-0.321285	0.0831815	770	998	1037	562	868	815
NUAK2	0.8495896	-0.235162	0.1616823	1224	1334	1584	1085	1247	1187
FADD	0.6968934	-0.52099	0.0147742	303	388	500	235	319	276
ZRSR1	0	#NUM!	0.4324683	2	0	2	0	0	0
ZRSR2	1.0336323	0.047723	0.6205989	140	123	183	158	174	129
AC004221.1	1	0	0.9831576	4	0	0	0	1	3
TNRC6C-AS1	0.6333333	-0.658963	0.4609744	11	13	6	3	11	5
UBAC1	0.9872408	-0.018526	0.7257457	1616	1879	2148	1945	1951	1675
UBAC2	1.0077655	0.01116	0.5475094	2627	3031	3485	3123	3182	2909
RHEBP1	0.2	-2.321928	0.5267773	0	0	5	0	0	1
SCARNA8	#DIV/0!	#DIV/0!	0.1307332	0	0	0	3	0	5
RHEBP2	1	0	0.7280764	479	474	571	420	557	547
STAG1	1.0100191	0.0143826	0.7790236	838	497	761	489	826	802
STAG2	1.208589	0.2733237	0.3457106	3321	1559	3270	2283	3990	3577
SCARNA4	0	#NUM!	0.355562	5	0	0	0	0	0
SCARNA4	0	#NUM!	0.355562	5	0	0	0	0	0
SCARNA4	0	#NUM!	0.355562	5	0	0	0	0	0
STAG3	1.1079646	0.1479118	0.3152298	141	173	251	198	221	207
SCARNA5	0	#NUM!	0.824326	1	0	0	0	0	0

SCARNA6	0.4	-1.321928	0.5729881	4	1	0	0	1	1
SCARNA6	0.4	-1.321928	0.5729881	4	1	0	0	1	1
SCARNA7	1	0	0.9890559	0	0	1	0	1	0
FASTKD1	1.1934545	0.2551436	0.0777352	1030	679	1041	808	1311	1163
FASTKD2	1.0538956	0.0757319	0.4248481	1303	846	1432	1009	1506	1259
TPI1P1	0.9546926	-0.066892	0.9418598	168	223	227	174	229	187
FAF1	1.0193293	0.0276202	0.4657444	2089	2158	2582	2090	2632	2239
FASTKD3	0.912844	-0.13156	0.6317648	371	316	403	254	393	348
TPI1P2	0.4888889	-1.032421	0.0355561	52	35	48	20	18	28
FAF2	0.8273203	-0.273482	0.0455316	6110	6102	7107	4335	5863	5785
TPI1P3	0	#NUM!	0.824326	0	0	1	0	0	0
STAB2	1.28	0.3561438	0.3238968	27	30	43	47	27	54
TPI1P4	0	#NUM!	0.6840298	0	0	2	0	0	0
IGLV7-46	0	#NUM!	0.6436235	2	0	0	0	0	0
STAB1	0.5638298	-0.826668	0.2351545	24	22	48	20	7	26
AC115621.1	4	2	0.5754166	0	1	0	0	4	0
MAP4K1	1.1071429	0.1468414	0.7752431	8	1	19	11	16	4
AP001888.1	1.5	0.5849625	0.7502258	0	6	0	6	3	0
MAP4K2	1.0605802	0.0848537	0.3025262	724	775	845	750	878	858
AC079922.2	0.9411765	-0.087463	0.9734547	7	6	4	6	3	7
FASTKD5	0.8669424	-0.205992	0.2497556	929	887	1085	763	928	824
SGK3	0.9790941	-0.030481	0.9037566	321	178	362	220	355	268
)004672|proteir	#VALUE!	#VALUE!	coid-regulatıway|PI3K-Al·egulated kinase family member 3						
SGK1	2.0666041	1.0472621	1.72E-11	374	318	374	599	897	707
SGK2	0	#NUM!	0.6840298	0	0	2	0	0	0
LYZL1	#DIV/0!	#DIV/0!	0.6411933	0	0	0	0	0	2
LYZL4	0.4444444	-1.169925	0.5468717	6	1	2	0	0	4
'RAPPC12-AS	1.0277778	0.0395284	0.8419455	19	45	44	27	35	49
AC008969.1	0.8338926	-0.262066	0.3412434	187	168	241	133	198	166
MAP4K5	1.1775362	0.2357715	0.1212334	1316	729	1267	993	1470	1437
MPZL3	0.8318777	-0.265557	0.2491189	291	280	345	196	277	289
MAP4K3	0.9827781	-0.025062	0.891617	1356	774	1412	824	1346	1311
MPZL1	0.8957452	-0.15884	0.3032867	16750	17680	19956	13974	17827	16915
THRB-AS1	1.4166667	0.5025003	0.5698744	1	2	9	10	3	4
MAP4K4	1.08428	0.1167374	0.1515566	8793	8903	10033	8074	10928	11064
MPZL2	1.75	0.8073549	0.3840257	6	4	6	7	17	4
AP006545.1	1.3	0.3785116	0.757292	1	7	2	6	0	7
AP006545.2	0.4666667	-1.099536	0.2180635	12	8	25	2	12	7
STAC3	1.0412371	0.0582986	0.7706777	14	34	49	41	42	18
C2-AS1	#DIV/0!	#DIV/0!	0.8030358	0	0	0	0	0	1
STAC2	6	2.5849625	0.3090696	0	1	0	5	1	0
TOMM7	1.0627005	0.0877351	0.3190215	1864	2407	2587	2659	2548	2081
TOMM5	1.2619048	0.335603	0.3417356	48	29	49	34	62	63
RBFADN	0.7142857	-0.485427	0.6220407	11	5	19	2	15	8
AC007256.1	1.75	0.8073549	0.650862	2	0	2	1	4	2
ARID4A	1.0776456	0.1078828	0.4233533	636	335	626	454	651	616
ARID4B	1.2086787	0.2734308	0.1260577	828	432	791	576	984	919
PCAT1	0.6363636	-0.652077	0.7339958	4	2	5	4	0	3
PTTG1	1.1088203	0.1490256	0.0968312	3971	4545	5259	5105	5588	4581
PCAT6	0.9562363	-0.064561	0.9246729	250	291	373	342	269	263
PCAT7	0.89	-0.168123	0.8261692	13	52	35	29	31	29
UNC5CL	3.75	1.9068906	0.0020167	5	8	11	45	23	22
ARID5A	0.9023546	-0.148234	0.6212478	384	506	554	420	505	378
RNU2-62P	0	#NUM!	0.1796811	2	5	0	0	0	0

ARID5B	1.1451078	0.1954834	0.2162599	422	354	430	315	503	563
SGO1	1.1112205	0.1521451	0.31459	380	226	410	290	431	408
SGO2	1.689418	0.7565263	0.0003348	740	321	829	887	1325	981
DNAJC30	1.0784314	0.1089344	0.5370424	81	73	101	87	86	102
RNU2-63P	0	#NUM!	0.824326	1	0	0	0	0	0
STAM2	1.1439791	0.1940606	0.14122	964	716	994	748	1236	1075
FAIM	1.0598349	0.0838396	0.3637156	491	394	569	467	585	489
AL606519.1	0.6	-0.736966	0.733745	4	1	0	0	3	0
AL008628.1	#DIV/0!	#DIV/0!	0.3721261	0	0	0	3	1	0
DNAJC22	0.8418972	-0.248284	0.3970405	254	187	318	161	220	258
DNAJC24	1.0973684	0.134048	0.3231433	246	195	319	223	310	301
DNAJC25	0.8885163	-0.17053	0.5838439	434	279	480	321	384	355
DNAJC27	0.7201166	-0.473698	0.1620556	101	95	147	52	91	104
DNAJC28	1.6352941	0.7095501	0.0925164	25	19	41	32	67	40
AL591926.2	#DIV/0!	#DIV/0!	0.8030358	0	0	0	0	1	0
AC000058.1	#DIV/0!	#DIV/0!	0.5070017	0	0	0	3	0	0
ASIC4	0	#NUM!	0.174358	1	2	4	0	0	0
ASIC5	0.5	-1	0.8254608	0	1	1	0	1	0
HSPA8P15	0.4146341	-1.270089	0.1654797	8	22	11	3	3	11
DNAJC21	1.0166752	0.0238589	0.5101146	2700	2032	3064	2400	2938	2588
HSPA8P18	0	#NUM!	0.824326	1	0	0	0	0	0
HSPA8P11	1	0	0.9890559	0	1	0	0	0	1
STAP2	1.1629464	0.2177846	0.2490694	141	156	151	167	204	150
HSPA8P14	2.1111111	1.0780025	0.4423878	7	2	0	2	3	14
ASIC3	1.0697674	0.0972972	0.6922621	28	13	45	34	22	36
ASIC1	1.1669086	0.2226915	0.1684563	174	242	273	276	247	281
DNAJC19	0.9725948	-0.040089	0.8785831	1025	1070	1335	1045	1289	1002
AC123595.1	0.3157895	-1.662965	0.322527	0	4	15	0	5	1
DNAJC11	0.9268156	-0.109646	0.624515	2112	2363	2685	1815	2501	2320
DNAJC12	1.7777778	0.830075	0.3271404	8	5	5	20	9	3
DNAJC13	1.0778267	0.1081253	0.4586275	2281	1201	1966	1334	2325	2213
DNAJC14	0.7946237	-0.331656	0.0862569	541	666	653	376	540	562
DNAJC15	2.1666667	1.1154772	0.1339716	12	8	4	23	20	9
DNAJC16	0.9508027	-0.072782	0.8438875	668	596	667	450	667	719
DNAJC17	0.9160935	-0.126433	0.7847273	206	245	276	240	230	196
DNAJC18	0.8956885	-0.158931	0.6031232	235	228	256	209	232	203
ERVK3-1	0.6374134	-0.649699	0.0983021	163	110	160	42	72	162
AL365330.1	1.2	0.2630344	0.5861808	6	15	24	18	16	20
DNAJC10	1.2626542	0.3364595	0.0234531	2166	1290	1977	1780	2670	2410
MCTP1	0.4264706	-1.229482	0.0736637	19	17	32	7	11	11
SGSH	0.5854722	-0.772328	1.12E-05	568	723	774	384	443	382
ENOSF1	0.9180566	-0.123345	0.6540763	440	434	505	377	461	428
HIBCH	1.2271361	0.2952953	0.0140448	826	633	870	914	997	947
AC007016.1	0	#NUM!	0.824326	0	1	0	0	0	0
ARID1A	0.9168499	-0.125242	0.564046	4976	5834	6015	3815	5402	6209
ARID1B	0.9192799	-0.121424	0.568778	2225	2221	2442	1553	2300	2479
VN1R70P	0	#NUM!	0.824326	1	0	0	0	0	0
EHHADH	0	#NUM!	0.6631647	1	0	1	0	0	0
MCTP2	1.0837989	0.1160971	0.4066664	252	185	279	202	303	271
FAN1	0.8422847	-0.24762	0.2919724	433	349	391	226	362	400
AL662890.2	0.7166667	-0.480626	0.3351482	40	48	32	19	26	41
AF131216.1	2.1111111	1.0780025	0.3881795	3	4	2	3	1	15
AF131216.3	1.2777778	0.353637	0.7154169	4	6	8	8	15	0
AF131216.4	1.2	0.2630344	0.8377619	0	0	5	1	3	2

ATE1	1.2086479	0.273394	0.075536	522	409	526	416	672	673
SGTB	0.863671	-0.211446	0.3769964	462	377	474	248	434	452
WTAP	0.9786861	-0.031082	0.8192101	2339	2257	2817	2127	2689	2439
SGTA	1.0534208	0.0750819	0.3083015	2497	2919	3120	3151	3062	2779
AC092755.2	0	#NUM!	0.5387425	3	0	0	0	0	0
ARID3A	0.9644749	-0.052184	0.9754724	17986	19804	21858	17385	20371	19773
ARID3B	1.0259648	0.0369813	0.5194845	2080	2488	2480	2022	2661	2548
ARID3C	0	#NUM!	0.5781912	0	0	3	0	0	0
AC092755.1	1	0	0.9873925	7	0	1	0	2	6
ATF1	0.9811486	-0.027456	0.9040227	833	663	785	632	864	742
ATF2	1.4277429	0.5137362	0.0039028	734	531	804	618	1131	1205
PARP1P2	0	#NUM!	0.6436235	2	0	0	0	0	0
MT-CO1	0.8898762	-0.168323	0.2842433	505690	519024	545991	369184	515783	512766
PARP1P1	0	#NUM!	0.3626892	1	0	4	0	0	0
ENDOU	2.1583012	1.1098962	4.22E-06	84	63	112	175	220	164
ENDOV	0.9834938	-0.024012	0.7821725	186	237	304	283	208	224
RNU2-24P	#DIV/0!	#DIV/0!	0.8030358	0	0	0	0	0	1
AC007493.2	0	#NUM!	0.824326	0	0	1	0	0	0
ATG3	0.9496326	-0.074559	0.9441741	1993	1941	2598	1981	2302	1920
ENDOG	0.7673611	-0.382022	0.2328617	147	213	216	175	141	126
ATF7	0.9578834	-0.062078	0.9767711	334	267	325	274	291	322
FAR1	1.0029657	0.0042723	0.782992	2224	1115	2056	1355	2122	1934
MT-CO2	0.8888925	-0.169919	0.2898605	155022	162495	181297	139761	161882	141749
FAR2	1.1162791	0.1586977	0.3217585	188	129	199	174	200	202
MT-CO3	0.9552981	-0.065977	0.8981322	166824	171181	189959	152313	186117	165933
ATF5	0.8030303	-0.316474	0.4029506	125	191	212	174	120	130
ATF6	1.093518	0.1289769	0.191091	1634	1578	1771	1410	2124	1915
ATF3	1.3308962	0.412398	0.0003439	1298	1592	1897	2023	2286	2062
ATF4	1.1977741	0.2603558	0.0098347	9559	11078	12608	13436	14233	12151
EPB41L5	0.8544372	-0.226954	0.3148743	676	471	701	393	593	593
AC021755.2	1	0	0.9699949	4	0	4	1	3	4
AC021755.3	3	1.5849625	0.4741617	1	1	0	4	2	0
EPB41L1	0.5499203	-0.862706	2.68E-08	985	1075	1075	463	678	583
EPB41L2	0.8858803	-0.174816	0.355522	2795	2714	3096	1833	2737	3053
EPB41L3	1.2780275	0.3539189	0.0012262	2555	2049	2737	2579	3416	3387
PIP5K1A	0.9144825	-0.128973	0.5059155	4118	4308	5080	3295	4489	4567
RPL11P3	0.3846154	-1.378512	0.3690708	3	4	6	2	2	1
PIP5K1B	1.018565	0.026538	0.6038424	678	575	740	550	784	696
PIP5K1C	1.0756039	0.1051469	0.2198845	1884	2379	2443	2263	2456	2494
ATG7	0.8798099	-0.184736	0.4010331	1218	1519	1681	1291	1419	1177
ATG5	1.0268522	0.0382285	0.5212837	1126	754	1211	983	1196	995
OR7A1P	#DIV/0!	#DIV/0!	0.6317974	0	0	0	1	1	0
CRIP1P2	#DIV/0!	#DIV/0!	0.8030358	0	0	0	0	0	1
CRIP1P4	#DIV/0!	#DIV/0!	0.4737294	0	0	0	0	4	0
AC009806.1	0.3650794	-1.453718	0.0599838	23	10	30	3	12	8
RNU2-25P	0	#NUM!	0.824326	1	0	0	0	0	0
FASN	0.8593267	-0.218721	0.1860592	16586	20897	19820	14113	17021	18108
FAT1	0.4536642	-1.140303	1.05E-07	878	827	874	231	410	529
FAT2	0.7161049	-0.481757	0.0132442	6322	7080	6623	3013	4896	6431
FAT3	0.5454545	-0.874469	0.6321237	3	1	7	0	1	5
FAT4	1.4390244	0.525091	0.3696981	17	11	13	15	26	18
BHLHE41	1.6866029	0.7541203	0.000991	114	162	142	168	285	252
OR7A8P	0.8	-0.321928	0.8886991	5	0	0	0	0	4
PINK1-AS	1.2772277	0.3530158	0.3429701	30	28	43	36	30	63

ATIC	1.1876522	0.2481125	0.0078483	6926	8018	8866	8976	10128	9174
ENOPH1	1.2479191	0.3195245	0.0008782	2809	2506	3095	3033	3760	3702
MIR646HG	0	#NUM!	0.824326	1	0	0	0	0	0
AC018463.1	1.3181818	0.3985494	0.6942604	5	10	7	2	20	7
ATL3	1.5862069	0.665581	0.3098984	9	8	12	11	18	17
ATL1	0.9540816	-0.067815	0.9972228	64	48	84	53	68	66
FAXC	0.5918367	-0.756729	0.1381829	49	30	68	34	30	23
ATL2	0.9748157	-0.036799	0.9459981	1818	1305	1761	1234	1726	1801
AC092127.1	1.6666667	0.7369656	0.5395924	3	7	2	3	13	4
CNPY4	0.829664	-0.269401	0.2813083	268	290	305	247	246	223
AC092127.2	0.6938776	-0.527247	0.4880511	28	9	12	6	20	8
RPL10AP6	0.9299065	-0.104842	0.8676344	76	68	70	65	72	62
RPL10AP1	0	#NUM!	0.6840298	0	0	2	0	0	0
FPGT-TNNI3K	2	1	0.834705	0	0	1	0	2	0
WTIP	0.8365217	-0.257525	0.4672643	169	222	184	194	173	114
AL136172.1	0.9333333	-0.099536	0.9805868	9	5	16	7	12	9
ATN1	0.8869368	-0.173097	0.3216879	2456	2773	3138	2264	2666	2491
AL138725.1	0.1666667	-2.584963	0.3862605	6	0	0	0	1	0
AL031668.1	#DIV/0!	#DIV/0!	0.5509701	0	0	0	0	3	0
CNPY2	0.9154229	-0.12749	0.87648	68	53	80	67	61	56
CNPY3	0.9474244	-0.077917	0.9119011	2462	2995	3007	2792	2744	2483
SHC4	0.4166667	-1.263034	0.3269783	9	1	14	1	3	6
LINC01011	1.2159091	0.2820354	0.3784608	71	59	46	69	89	56
LINC01012	0.9419355	-0.0863	0.918279	52	50	53	39	46	61
SHC3	0	#NUM!	0.1909345	0	6	1	0	0	0
SHC1	0.834945	-0.260247	0.1270594	2091	2731	2727	1936	2232	2135
AC023818.2	0.78125	-0.356144	0.6960089	24	7	33	7	11	32
EIF4EBP1	1.1223548	0.1665288	0.1909274	619	985	995	977	1017	923
LINC01007	7.75	2.9541963	0.0147954	0	2	2	16	11	4
EIF4EBP2	1.2168733	0.2831789	0.0066878	7466	8515	9029	8143	11048	11243
EIF4EBP3	0.2142857	-2.222392	0.2154633	6	5	3	3	0	0
LINC01003	0.8204134	-0.285577	0.2439116	249	242	283	206	237	192
LINC01004	1.0789474	0.1096245	0.7119123	17	15	44	29	39	14
LINC01006	0	#NUM!	0.5781912	0	0	3	0	0	0
LINC01001	0.2906977	-1.782409	0.0367531	27	6	53	14	7	4
LINC01002	0.6872587	-0.541075	0.1200106	97	78	84	45	62	71
MFSD6L	1.1186441	0.1617511	0.5028911	68	92	76	82	105	77
TSPY26P	0.8571429	-0.222392	0.8989382	7	4	17	8	10	6
MT-CYB	0.8249795	-0.27757	0.0328713	90491	95367	108399	66597	90858	85301
SHBG	2.5	1.3219281	0.5686736	0	0	2	2	3	0
OR7A10	0	#NUM!	0.824326	0	0	1	0	0	0
AKAP8L	0.9429461	-0.084753	0.8474649	1111	1356	1389	1175	1261	1200
AL451050.1	0	#NUM!	0.276989	0	6	0	0	0	0
AL451050.2	0.6666667	-0.584963	0.6006525	4	17	6	9	8	1
AC105020.1	0.4166667	-1.263034	0.2853243	13	4	7	5	4	1
AC105020.2	0	#NUM!	0.5781912	0	0	3	0	0	0
RNU6-387P	1.2	0.2630344	0.8847211	3	2	0	2	1	3
AC105020.5	0	#NUM!	0.824326	1	0	0	0	0	0
AC105020.6	0.3333333	-1.584963	0.327671	3	2	16	5	0	2
AL158824.1	1.0666667	0.0931094	0.9134047	11	14	5	6	11	15
ATRX	1.1409865	0.1902817	0.3079398	969	463	859	561	1025	1028
ATRN	0.7924285	-0.335647	0.0374359	2329	2640	2797	1527	2247	2380
WAC-AS1	1.2227357	0.2901126	0.0175234	692	583	889	813	1022	811
AC079331.1	0	#NUM!	0.6385096	0	2	0	0	0	0

AC079331.2	1	0	0.9617562	0	0	2	0	0	2
AC122133.1	0	#NUM!	0.824326	0	1	0	0	0	0
AC073316.1	1.6666667	0.7369656	0.6964946	2	0	1	4	1	0
PCDH1	0.6857639	-0.544216	0.0690155	149	239	188	115	110	170
PCDH9	1.03125	0.0443941	0.9845192	29	26	9	9	33	24
PCDH7	1.7142857	0.7776076	0.0610868	33	26	25	34	57	53
PCDH8	0.4166667	-1.263034	0.2819327	5	10	9	0	4	6
AL645949.1	0.6666667	-0.584963	0.8318384	2	1	0	0	0	2
RNU6-388P	0	#NUM!	0.4217812	0	0	5	0	0	0
AC005096.1	1	0	0.9890559	0	1	0	0	0	1
NUDT1	0.9890431	-0.015895	0.754835	366	471	532	524	447	383
NUDT2	1.2151989	0.2811924	0.061654	378	517	513	555	668	488
NUDT5	1.2016275	0.2649897	0.0059947	2032	2093	2511	2598	2843	2533
RPS3AP23	0	#NUM!	0.824326	0	0	1	0	0	0
NUDT6	0.9856459	-0.020859	0.8459465	58	56	95	77	74	55
RPS3AP24	1.2	0.2630344	0.7758274	3	2	5	6	5	1
NUDT3	0.9875776	-0.018034	0.7680516	191	182	271	231	194	211
IL27RA	0.9512195	-0.07215	0.9097148	15	15	11	2	19	18
NUDT4	1.1765835	0.2346037	0.0342931	2756	2299	2760	2395	3429	3371
RPS3AP25	0.25	-2	0.5310185	0	3	1	0	1	0
RPS3AP26	1.1747159	0.2323119	0.1128776	215	220	269	261	298	268
MFSD2B	0.475	-1.074001	0.235974	5	20	15	7	7	5
MFSD2A	2.8444444	1.5081469	0.002777	16	12	17	63	38	27
MFSD11	0.8994614	-0.152867	0.505626	553	491	627	416	592	495
MFSD12	1.1556092	0.2086536	0.0872913	746	760	981	1093	849	932
MFSD10	1.1941928	0.2560358	0.0305014	1278	1365	1662	1880	1792	1469
RPS3AP14	2	1	0.8147319	1	0	0	0	0	2
RPS3AP15	7	2.8073549	0.3274177	0	0	1	0	0	7
FBF1	0.8842105	-0.177538	0.7418657	55	55	80	51	70	47
RPS3AP40	0	#NUM!	0.824326	1	0	0	0	0	0
RPS3AP43	0	#NUM!	0.355562	5	0	0	0	0	0
RPS3AP49	0.5	-1	0.6946839	0	4	0	0	1	1
RPS3AP47	1.24	0.3103401	0.3827507	32	35	33	44	43	37
GREB1	0.4837398	-1.047697	0.0002987	134	174	184	53	109	76
BSDC1	1.0246415	0.0351192	0.4288874	1471	1601	1879	1603	1713	1757
RPS3AP34	0.2	-2.321928	0.2831282	1	9	0	0	0	2
RPS3AP35	0	#NUM!	0.824326	0	0	1	0	0	0
AC090709.1	1.8	0.8479969	0.716368	5	0	0	0	0	9
RPS3AP38	0.6363636	-0.652077	0.5622125	8	10	4	7	5	2
AL121972.2	0	#NUM!	0.824326	0	0	1	0	0	0
RPS3AP39	0	#NUM!	0.824326	1	0	0	0	0	0
RPS3AP37	13	3.7004397	0.0959863	1	0	0	0	2	11
UBAC2-AS1	0.9490446	-0.075452	0.9665296	43	64	50	46	27	76
AC017100.1	1.4782609	0.5639009	0.4614421	10	2	11	9	7	18
DHRS12	0.6128134	-0.70648	0.03094	119	108	132	87	66	67
WFDC8	0	#NUM!	0.6840298	0	0	2	0	0	0
DHRS13	0.9475138	-0.077781	0.9401556	204	266	254	247	243	196
WFDC6	0	#NUM!	0.2515625	5	0	1	0	0	0
DHRS11	1.1141361	0.1559255	0.2353303	318	267	370	363	401	300
TBX2-AS1	0.8135593	-0.297681	0.6264015	30	67	21	36	19	41
WFDC1	#DIV/0!	#DIV/0!	0.4737294	0	0	0	0	4	0
WFDC2	0.7083333	-0.4975	0.6559816	8	5	11	5	6	6
WFDC3	2	1	0.506583	2	3	1	2	8	2
AKAP12	0.8722741	-0.197146	0.3140813	4728	3944	5131	2724	4453	4863

AKAP13	0.9641084	-0.052733	0.9008891	1813	1634	1791	1090	1888	2072
AKAP10	1.0425182	0.0600726	0.4005566	1462	1200	1595	1197	1624	1617
AKAP11	1.0393551	0.0556887	0.6863675	827	429	853	408	881	903
AKAP14	#DIV/0!	#DIV/0!	0.0572987	0	0	0	0	4	8
AC243562.1	0.1666667	-2.584963	0.3359778	2	4	0	0	0	1
AC243562.2	1.3333333	0.4150375	0.9009891	0	3	0	0	2	2
PRORSD1P	1.0982659	0.1352274	0.5738598	60	46	67	52	58	80
RPS24P17	1.2	0.2630344	0.8797727	3	6	1	2	9	1
RPS24P12	5	2.3219281	0.4102632	1	0	0	2	3	0
ZADH2	0.805298	-0.312405	0.2135522	298	219	238	149	234	225
RNU1-91P	#DIV/0!	#DIV/0!	0.8030358	0	0	0	0	1	0
AC115522.1	2.6666667	1.4150375	0.4399576	1	0	2	1	1	6
MFSD4A	1.2941176	0.3719688	0.7007035	8	5	4	4	10	8
SHOX	3	1.5849625	0.6191077	0	0	1	2	1	0
MFSD4B	0.954141	-0.067726	0.9108153	470	434	557	343	509	542
PNPLA7	0.7228916	-0.468149	0.5475079	15	28	40	30	17	13
PNPLA8	1.3231478	0.4039742	0.0202264	914	465	1010	850	1206	1105
BUB1B-PAK6	0.5	-1	0.8002255	1	1	0	0	1	0
NUDT9	1.1356084	0.1834655	0.1060298	750	600	877	853	928	748
NUDT7	0.8214286	-0.283793	0.4753451	78	93	137	83	93	77
MIS18A	1.0477506	0.0672953	0.3477534	723	788	1023	804	881	970
NUDT8	1.0736544	0.1025297	0.4878258	104	84	165	147	123	109
OTULIN	0.8428884	-0.246586	0.1287351	1533	1414	1623	999	1466	1387
Z82217.1	0.75	-0.415037	0.8604653	0	1	11	0	9	0
PNPLA3	1.0357143	0.0506261	0.8431082	7	7	14	15	0	14
PNPLA4	0.9663785	-0.04934	0.9999977	315	398	328	338	352	316
PNPLA6	0.8224456	-0.282008	0.0904187	1410	1654	1712	1262	1370	1296
AC068481.1	0.375	-1.415037	0.4832401	3	5	0	0	3	0
PNPLA1	2.5	1.3219281	0.2233355	7	1	2	10	11	4
PNPLA2	1.0648228	0.0906134	0.3045909	642	769	903	869	820	775
UBE3C	0.8797562	-0.184824	0.2729827	3256	3171	3744	2375	3159	3414
UBE3D	1.0963636	0.1327264	0.3401728	175	148	227	211	200	192
UBE3A	0.9973545	-0.003822	0.7677104	1790	1107	2017	1209	1902	1790
AC012447.1	0	#NUM!	0.6436235	2	0	0	0	0	0
SBF2-AS1	1.1595745	0.2135955	0.5463971	31	12	51	29	36	44
UBE3B	0.7743561	-0.368931	0.02075	1117	1151	1304	709	1115	942
UBE2Z	0.9095265	-0.136812	0.4562082	4227	4859	5106	3637	4679	4592
AC104088.1	1.0714286	0.0995357	0.8011351	16	15	11	22	7	16
UBE4A	0.7253411	-0.463268	0.0274683	1745	1562	1823	721	1332	1668
UBE4B	1.0366915	0.0519867	0.4536706	1975	2085	2372	1703	2503	2462
POU3F1	0.0769231	-3.70044	0.06742	4	7	2	0	1	0
POU3F2	0.762931	-0.390375	0.2062424	121	161	182	123	111	120
POU3F3	0.9981168	-0.00272	0.8917314	164	166	201	109	228	193
SHQ1	0.8316374	-0.265974	0.1514787	719	711	732	446	718	634
MAP7D3	1.3406187	0.422899	0.006083	1109	641	1224	1121	1496	1370
MAP7D1	1.0158684	0.0227136	0.5217956	2945	3631	3822	3476	3700	3387
SHPK	1.0778098	0.1081026	0.5491487	100	106	141	160	145	69
EHBP1L1	0.6714868	-0.574569	0.0003365	859	844	994	598	597	616
MAP7D2	0	#NUM!	0.0490776	5	3	3	0	0	0
FBN2	0.8434155	-0.245685	0.2273147	3980	3960	3830	1987	3690	4250
FBN3	0.7337461	-0.446647	0.1897709	105	90	128	84	76	77
AC026523.2	#DIV/0!	#DIV/0!	0.5070017	0	0	0	3	0	0
AC060809.1	0.4	-1.321928	0.6029531	4	1	0	0	0	2
AL512326.1	0	#NUM!	0.824326	1	0	0	0	0	0

DHRS7B	0.9360223	-0.095385	0.8015965	598	764	795	637	769	613
MFAP3L	0.4848485	-1.044394	0.2724234	8	12	13	6	7	3
TFAP2A	1.116127	0.1585012	0.0903324	1021	889	1302	1067	1292	1226
UBE2F	1.1987315	0.2615086	0.1384276	134	131	208	173	211	183
AC018953.1	0.8571429	-0.222392	0.9383084	4	3	0	5	1	0
TFAP2B	0.5	-1	0.2993446	6	13	13	2	6	8
TFAP2C	1.0220219	0.0314261	0.4425467	9863	10614	11582	9475	11980	11310
UBE2H	1.1629074	0.2177363	0.0166514	5036	5058	6044	5102	6818	6847
UBE2I	0.912033	-0.132842	0.618401	1974	2201	2623	2095	2196	1909
TFAP2E	1.0151515	0.0216951	0.8647969	31	10	25	26	23	18
UBE2B	1.0856458	0.1185535	0.143367	1573	1484	1952	1748	2005	1685
UBE2C	1.0625717	0.0875602	0.2433828	3840	4173	5060	4986	4739	4166
AC017006.2	#DIV/0!	#DIV/0!	0.8030358	0	0	0	0	0	1
AC017006.1	0.7272727	-0.459432	0.7500867	2	5	4	0	5	3
UBE2A	0.924758	-0.112852	0.6174759	2213	2251	2766	1960	2474	2252
GVQW2	1.1734694	0.2307802	0.4915414	32	28	38	42	26	47
AP001885.1	#DIV/0!	#DIV/0!	0.6554062	0	0	0	0	2	0
UBE2W	1.1430536	0.1928931	0.2550032	252	148	327	227	318	286
AL078644.1	0.9333333	-0.099536	0.9665038	12	11	22	22	8	12
UBE2S	1.0592472	0.0830393	0.3335194	4534	5959	6031	6532	5847	5124
UBE2T	0.9784529	-0.031426	0.760282	6624	6761	8799	6771	7854	7081
BHLHE40	1.1920983	0.2535032	0.0060229	2410	2521	2966	2742	3558	3114
UBE2U	7	2.8073549	0.1137958	1	1	0	2	8	4
UBE2N	0.9849791	-0.021835	0.761789	3727	3701	4289	3453	4234	3854
UBE2O	0.7586162	-0.398558	0.0031415	2311	2469	2822	1682	2023	2062
UBE2K	1.064295	0.0898981	0.273797	3998	3095	4432	3263	4566	4437
FBN1	0.4146341	-1.270089	0.2108667	9	21	11	9	8	0
UBE2M	0.9336158	-0.099099	0.8584155	1581	1855	2228	1875	1826	1587
AGGF1P2	1.0238095	0.0339473	0.8006275	4	4	34	17	14	12
AGGF1P3	0	#NUM!	0.824326	0	0	1	0	0	0
AGGF1P1	1.3846154	0.4694853	0.6404657	7	0	6	6	6	6
RASGEF1A	0.4849138	-1.0442	0.0004901	128	151	185	55	69	101
AGGF1P7	0	#NUM!	0.824326	0	0	1	0	0	0
RASGEF1B	0.94081	-0.088025	0.8701372	109	108	104	75	94	133
RASGEF1C	1.8571429	0.8930848	0.2270354	3	8	10	17	10	12
DACT2	1.4130435	0.4988059	0.3342363	14	9	23	27	24	14
DACT3	0.7692308	-0.378512	0.6781726	12	14	13	11	6	13
AC044840.1	#DIV/0!	#DIV/0!	0.3831122	0	0	0	0	5	0
ARFGEF3	0.8287093	-0.271062	0.3483851	337	196	296	151	246	290
DACT1	0.2	-2.321928	0.0738746	8	7	15	0	6	0
AC097063.1	0.4	-1.321928	0.6282732	3	0	2	0	0	2
OR2S1P	0	#NUM!	0.6385096	0	2	0	0	0	0
RPS3AP53	#DIV/0!	#DIV/0!	0.6411933	0	0	0	0	0	2
RPS3AP54	0.7123288	-0.489385	0.4262975	17	33	23	9	27	16
GREM2	1.1098765	0.1503992	0.27079	240	268	302	247	346	306
RNU2-31P	#DIV/0!	#DIV/0!	0.6411933	0	0	0	0	0	2
ZDHHC20-IT1	1	0	0.9690435	0	6	2	0	7	1
FBP2	1.8	0.8479969	0.5244975	0	2	8	0	6	12
RAP1GDS1	1.2361371	0.3058387	0.0150549	503	498	604	534	751	699
FBRS	1.2846029	0.3613224	0.0003929	1719	1796	2012	2259	2454	2387
ARFGEF1	1.0126601	0.0181501	0.7372119	2771	1337	2527	1629	2496	2594
ARFGEF2	0.9248864	-0.112652	0.6669736	2288	1720	2156	1163	2111	2427
SLC50A1	0.9979036	-0.003028	0.6644483	1103	1253	1460	1273	1404	1131
MZF1-AS1	0.7272727	-0.459432	0.458786	24	22	31	15	18	23

AL662791.4	1.0731707	0.1018796	0.8128044	35	32	15	27	27	34
AL441943.3	#DIV/0!	#DIV/0!	0.4737294	0	0	0	0	4	0
AC087752.3	1.0761905	0.1059334	0.7426205	34	39	32	32	47	34
AC087752.4	1	0	0.9362872	2	0	4	2	0	4
AC087752.2	6	2.5849625	0.3180916	0	0	1	4	2	0
PPIEL	0.4666667	-1.099536	0.1765919	17	16	12	9	6	6
AC105415.1	0	#NUM!	0.6385096	0	2	0	0	0	0
AC108516.2	0	#NUM!	0.824326	1	0	0	0	0	0
HMOX1	0.5303826	-0.914895	1.25E-08	1546	1781	2005	1012	948	868
AC007494.2	1	0	0.9672374	2	1	0	0	3	0
AC007494.3	0	#NUM!	0.824326	1	0	0	0	0	0
HMOX2	1.0298108	0.0423792	0.4876655	1700	2169	2102	2044	2204	1901
RNU2-38P	0.5151515	-0.956931	0.3526994	7	10	16	5	2	10
BARD1	1.1418893	0.1914229	0.1395097	919	763	975	721	1160	1153
IRF2BP1	0.9564482	-0.064241	0.9792969	693	843	829	784	785	693
IRF2BP2	0.8553891	-0.225347	0.1534605	1755	1653	2131	1402	1772	1564
RNU2-37P	#DIV/0!	#DIV/0!	0.5070017	0	0	0	3	0	0
HSPA8P4	1.8571429	0.8930848	0.5502528	6	1	0	5	6	2
AC015922.4	#DIV/0!	#DIV/0!	0.5509701	0	0	0	0	3	0
AC016907.2	1.6666667	0.7369656	0.7964	3	0	0	0	4	1
HSPA8P5	1.6	0.6780719	0.6479001	2	2	1	6	0	2
HSPA8P8	0	#NUM!	0.824326	1	0	0	0	0	0
HSPA8P9	#DIV/0!	#DIV/0!	0.8030358	0	0	0	0	1	0
AC015922.1	1	0	0.9890559	0	0	1	0	1	0
AL139805.1	0	#NUM!	0.824326	1	0	0	0	0	0
MZT1P2	0.3333333	-1.584963	0.669913	3	0	0	0	0	1
NCOR2	0.9460945	-0.079944	0.8060188	6740	7774	8415	6044	7642	8007
NCOR1	0.8193302	-0.287483	0.1386903	2359	1956	2493	1280	1922	2376
HSPA8P1	0.3333333	-1.584963	0.6538103	0	0	6	2	0	0
HSPA8P3	0.8	-0.321928	0.914487	1	1	3	0	0	4
AL627171.1	0.5769231	-0.793549	0.5445885	5	13	8	10	0	5
AL627171.2	0.6891892	-0.537028	0.4348278	30	14	30	10	33	8
AL512324.3	0.5280899	-0.921145	0.1414893	21	39	29	4	21	22
AL512324.1	0.8181818	-0.289507	0.7675476	19	7	29	9	12	24
SIAE	0.8714716	-0.198474	0.2450219	1204	1197	1248	924	1187	1069
AC011462.4	5.6666667	2.5025003	0.103532	0	1	2	3	2	12
AL138820.1	1.4210526	0.50696	0.5414886	5	8	6	7	10	10
AC011462.3	0	#NUM!	0.6385096	0	2	0	0	0	0
SYNJ2BP	1.0902742	0.124691	0.2473605	1400	1016	1450	1091	1538	1586
NUCB1	0.9746923	-0.036981	0.8462994	3199	4155	4263	3927	3857	3539
ELL2P1	0	#NUM!	0.0663324	7	4	0	0	0	0
NUCB2	1.351057	0.4340886	0.0066881	1141	646	1335	1313	1628	1277
ELL2P2	#DIV/0!	#DIV/0!	0.4737294	0	0	0	0	4	0
AC114684.1	#DIV/0!	#DIV/0!	0.6411933	0	0	0	0	0	2
AUP1	0.9436502	-0.083676	0.9323982	3187	3551	4034	3735	3514	2916
AC027601.3	0.8076923	-0.308122	0.7381918	12	7	7	2	16	3
AC027601.5	5	2.3219281	0.4322862	1	0	0	0	3	2
AC027601.1	0.4528302	-1.142958	0.2255289	3	18	32	8	5	11
BOLA2B	0.5263158	-0.925999	0.4556422	5	4	10	2	5	3
CNOT10-AS1	0	#NUM!	0.824326	0	1	0	0	0	0
AC055811.3	#DIV/0!	#DIV/0!	0.4737294	0	0	0	0	4	0
AC055811.1	0.4615385	-1.115477	0.3149352	14	6	6	0	5	7
AL031667.2	0	#NUM!	0.154788	7	0	1	0	0	0
AL031667.3	1	0	0.959473	3	5	2	0	10	0

AC009065.8	0.7388724	-0.436603	0.2287159	87	136	114	90	67	92
AC055811.4	0.721393	-0.471143	0.1264312	138	153	111	73	96	121
DUSP5P1	1.1428571	0.1926451	0.941609	4	1	2	0	8	0
AC009065.2	#DIV/0!	#DIV/0!	0.6482162	0	0	0	0	1	1
AC009065.3	#DIV/0!	#DIV/0!	0.6317974	0	0	0	1	1	0
AC009065.4	1.137931	0.1864131	0.7983493	10	12	7	10	17	6
AC009065.5	0	#NUM!	0.5075476	0	0	4	0	0	0
FCAR	0.0769231	-3.70044	0.1122126	4	0	9	0	1	0
PIP5KL1	1.740458	0.799467	0.0134209	33	44	54	86	73	69
RPL10P3	1.3333333	0.4150375	0.8221461	0	2	1	3	1	0
RPL10P4	#DIV/0!	#DIV/0!	0.8030358	0	0	0	0	1	0
AC122134.1	#DIV/0!	#DIV/0!	0.8030358	0	0	0	0	0	1
RPL10P6	0.2	-2.321928	0.4085823	1	2	2	0	1	0
RPL10P9	1.0663825	0.092725	0.416536	413	633	596	610	625	516
FOXD4L6	0.6	-0.736966	0.7454667	0	5	0	0	3	0
FOXD4L3	1.5	0.5849625	0.8194094	0	0	2	0	0	3
FOXD4L4	1.5	0.5849625	0.8347492	2	0	0	1	0	2
AL023581.2	0	#NUM!	0.824326	0	1	0	0	0	0
FOXD4L1	0.75	-0.415037	0.8480138	1	2	9	3	6	0
AL138726.1	0	#NUM!	0.5387425	3	0	0	0	0	0
SELENBP1	0.9693431	-0.044921	0.8625513	207	188	290	245	221	198
AL158825.2	1.125	0.169925	0.8869298	4	9	3	2	6	10
PPIL1	0.9612371	-0.057036	0.9689221	2922	3132	3646	3005	3343	2976
PPIL2	0.9862155	-0.020025	0.7499783	1680	1827	2079	1677	1926	1906
PPIL3	1.1176036	0.1604085	0.2123172	494	356	502	482	585	444
PPIL4	1.1646222	0.219862	0.0666248	1483	1008	1585	1338	1830	1579
PPIL6	1.6428571	0.716207	0.1692791	8	25	23	41	18	33
FCF1	0.9185205	-0.122616	0.5220013	2461	2446	2690	1927	2565	2486
SIL1	1.0088959	0.0127773	0.5865986	1107	1282	1433	1358	1339	1159
SCML2P1	#DIV/0!	#DIV/0!	0.6554062	0	0	0	0	2	0
SCML2P2	1.5	0.5849625	0.8084072	0	3	1	0	5	1
SIK3	0.7711204	-0.374972	0.0069519	2543	2800	3002	1761	2394	2280
SIK1	0.7569444	-0.401741	0.424983	37	50	57	41	33	35
SIK2	0.7272189	-0.459538	0.0113117	1072	1081	1227	570	869	1019
AC073314.1	0	#NUM!	0.6385096	0	2	0	0	0	0
GAPDHP2	#DIV/0!	#DIV/0!	0.6554062	0	0	0	0	2	0
AC020928.1	0.8421053	-0.247928	0.8158016	20	13	24	23	16	9
AC020928.2	0.5294118	-0.917538	0.2989501	13	15	23	0	19	8
GAPDHP1	1.05	0.0703893	0.6940873	37	58	65	60	52	56
TP53BP2	0.936276	-0.094994	0.7645779	1010	952	1255	758	1098	1156
TP53BP1	0.9327509	-0.100436	0.725658	2055	1701	2311	1270	2095	2294
AC006030.1	1	0	0.9617562	0	0	2	0	0	2
PYCR1	1.0609319	0.0853321	0.4664434	195	294	348	279	273	336
PYCR2	0.8910675	-0.166393	0.5161677	396	462	519	390	461	376
PYCR3	0.8777698	-0.188085	0.4874392	403	531	465	433	428	367
SNORA74C-1	#DIV/0!	#DIV/0!	0.5509701	0	0	0	0	3	0
SIM2	2.3181818	1.2129937	0.1162553	10	8	4	10	14	27
AP001646.2	#DIV/0!	#DIV/0!	0.6251462	0	0	0	1	0	1
TOP2A	1.2439611	0.3149414	0.052731	10743	5270	11020	8572	13326	11730
TOP2B	1.175681	0.2334967	0.107214	4002	2156	3974	3118	4669	4125
ETF1P2	0.25	-2	0.6181385	0	0	4	0	0	1
ZNRF2P2	0.6226415	-0.683526	0.3588158	14	16	23	4	18	11
CITED1	1	0	0.9647348	2	1	0	3	0	0
CITED2	1.1024463	0.1407084	0.0844445	6090	6975	7619	7163	8261	7379

ETF1P1	0	#NUM!	0.824326	0	0	1	0	0	0
ZNRF2P1	0.6965517	-0.521698	0.2627774	44	50	51	24	29	48
CITED4	1.2880859	0.3652288	0.0172184	278	346	400	463	452	404
KIZ-AS1	0.3333333	-1.584963	0.5432136	0	2	4	0	2	0
TOP3B	1	0	0.9900502	6	11	13	0	12	18
TOP3A	0.7624038	-0.391373	0.0082414	1853	2050	2204	1330	1580	1746
AL390119.1	#DIV/0!	#DIV/0!	0.8030358	0	0	0	1	0	0
AC012050.1	#DIV/0!	#DIV/0!	0.2497949	0	0	0	0	3	3
CU639417.5	0.5	-1	0.8652929	0	0	2	0	1	0
CU639417.1	1.1153846	0.1575413	0.3010685	159	160	227	237	191	181
FCN3	0.5	-1	0.7031599	1	1	2	0	2	0
TATDN3	1.080417	0.1115882	0.284246	455	374	514	481	527	443
TATDN1	1.1640043	0.2190963	0.1619286	361	230	348	312	421	360
TATDN2	1.0326609	0.0463666	0.5341891	271	335	435	328	399	348
AC244153.1	1.1123096	0.1535585	0.2096022	425	546	605	633	609	511
NDUFB8P2	0.125	-3	0.232088	1	7	0	0	1	0
AC007014.2	4.3333333	2.1154772	0.2242936	0	0	3	12	1	0
AC003043.1	0.2	-2.321928	0.4521254	0	5	0	0	0	1
RPS19BP1	0.9250061	-0.112465	0.8492069	1032	1495	1540	1466	1201	1095
AC015923.1	7	2.8073549	0.3498528	0	0	1	0	7	0
TIMM23B	0.9554455	-0.065754	0.9755981	204	156	246	148	198	233
RNU2-42P	0	#NUM!	0.824326	1	0	0	0	0	0
AC023078.3	0.5	-1	0.7553122	0	0	6	1	0	2
POU2F1	1.0043551	0.0062695	0.8132876	2227	1674	2069	1321	2224	2451
POU2F2	0.5980392	-0.741688	0.201195	42	28	32	6	37	18
AC023078.5	0	#NUM!	0.4460243	4	0	0	0	0	0
POU2F3	2.3913043	1.2577978	0.0145336	20	7	19	31	45	34
AC023078.7	3	1.5849625	0.5085179	0	0	2	0	4	2
MAP6D1	1	0	0.9480819	7	7	22	4	24	8
AVEN	0.9012915	-0.149934	0.5803575	316	390	378	301	352	324
SIX1	0.7323944	-0.449307	0.5251701	24	15	32	19	19	14
IRF2BPL	0.959221	-0.060065	0.9429682	2266	2714	2671	2223	2587	2529
SIX4	0.8552835	-0.225525	0.2427798	1125	962	1140	641	1065	1054
SIX5	0.9467593	-0.07893	0.947987	115	153	164	130	130	149
AC006270.1	0	#NUM!	0.824326	0	0	1	0	0	0
AC006270.2	#DIV/0!	#DIV/0!	0.1199037	0	0	0	9	0	0
OR2T33	0.5	-1	0.81753	2	0	0	0	0	1
QPCTL	1.0726681	0.1012038	0.3513172	281	283	358	353	309	327
AC145676.1	#DIV/0!	#DIV/0!	0.0616513	0	0	0	2	5	3
AVIL	0.4489796	-1.155278	0.1840397	22	8	19	8	3	11
AGAP12P	0.7575758	-0.400538	0.7132217	5	12	16	9	6	10
AL137013.1	0.9565217	-0.06413	0.9723002	5	7	11	1	10	11
NDUFB4P5	0.4	-1.321928	0.629514	2	0	3	1	1	0
NDUFB4P6	0	#NUM!	0.284725	6	0	0	0	0	0
AC002310.6	0.6666667	-0.584963	0.2856642	31	34	31	13	20	31
NDUFB4P7	0	#NUM!	0.824326	1	0	0	0	0	0
NDUFB4P8	#DIV/0!	#DIV/0!	0.5070017	0	0	0	3	0	0
AC002310.2	1.2857143	0.3625701	0.7800417	0	2	5	1	3	5
AC002310.3	2	1	0.7861378	1	0	0	1	1	0
AC002310.1	1.0588235	0.0824622	0.6989569	39	35	62	48	63	33
AGAP13P	0.7567568	-0.402098	0.6578597	20	5	12	1	13	14
AL138963.1	1.25	0.3219281	0.8454485	2	1	1	2	2	1
AGAP10P	1.5	0.5849625	0.7337883	2	0	2	2	0	4
ACOXL-AS1	2	1	0.8324799	1	0	0	0	2	0

ALS2CR12	1.6666667	0.7369656	0.7088539	0	2	1	2	2	1
AVL9	0.8442831	-0.244201	0.1313418	2065	1858	1998	1366	1782	1851
CTD-2201I18.1	#DIV/0!	#DIV/0!	0.6411933	0	0	0	0	0	2
JPH1	0.4186047	-1.25634	0.0305737	29	41	59	24	9	21
JPH2	0.7871094	-0.345364	0.2176205	144	181	187	92	175	136
JPH3	4	2	0.5760976	1	0	0	0	4	0
AL137159.1	0.8947368	-0.160465	0.9073192	7	6	6	5	6	6
AC106706.1	0.6788991	-0.558731	0.2833715	25	43	41	19	28	27
MRPL23-AS1	0.5098039	-0.971986	0.2578144	8	17	26	12	6	8
AL359706.1	2	1	0.7760147	0	2	0	0	4	0
AC135782.2	#DIV/0!	#DIV/0!	0.8030358	0	0	0	0	1	0
AC135782.3	#DIV/0!	#DIV/0!	0.5415473	0	0	0	0	2	1
AC024405.2	0.8131868	-0.298341	0.6097051	24	33	34	21	31	22
AC074386.1	1.4	0.4854268	0.6648085	4	3	3	4	6	4
AC093835.1	1	0	0.9617562	0	0	2	0	0	2
AC002550.2	0.8571429	-0.222392	0.9442311	2	0	5	0	2	4
AC015921.1	1.0588235	0.0824622	0.7572513	11	15	25	26	15	13
AGAP14P	0.6285714	-0.669851	0.4688424	6	13	16	3	8	11
7SK	5	2.3219281	0.0517363	5	1	0	18	7	5
7SK	5	2.3219281	0.0517363	5	1	0	18	7	5
7SK	5	2.3219281	0.0517363	5	1	0	18	7	5
7SK	5	2.3219281	0.0517363	5	1	0	18	7	5
7SK	5	2.3219281	0.0517363	5	1	0	18	7	5
7SK	5	2.3219281	0.0517363	5	1	0	18	7	5
7SK	5	2.3219281	0.0517363	5	1	0	18	7	5
RABAC1	1.0514145	0.0723315	0.3823734	1217	1499	1738	1769	1642	1272
HSPA9P1	2	1	0.6073167	4	0	0	3	4	1
AC109588.1	6.3333333	2.662965	0.0630421	2	0	1	10	3	6
LINC01232	0.6415479	-0.640371	0.0134915	166	160	165	93	114	108
SH3BP5-AS1	0.6631331	-0.59263	0.0074684	275	262	312	148	189	226
LINC01225	0	#NUM!	0.4217812	0	0	5	0	0	0
LINC01226	1.3448276	0.4274212	0.353435	21	16	21	34	18	26
LINC01220	0.9428571	-0.084889	0.9941141	18	25	27	26	16	24
AGTPBP1	1.3326967	0.4143485	0.022883	1082	550	986	866	1444	1179
OLFM4	3.12	1.641546	0.0063181	7	7	11	26	24	28
TON1-GTF2A1	6	2.5849625	0.1415859	1	1	0	8	2	2
FRMD1	2.75	1.4594316	0.3877511	3	0	1	7	4	0
FRMD3	0.5757576	-0.796467	0.3736534	11	11	11	4	11	4
OLFM1	0	#NUM!	0.824326	1	0	0	0	0	0
FRMD5	1.8518519	0.8889687	0.2183108	14	2	11	18	21	11
OLFM2	0.4418605	-1.178337	0.1925577	7	18	18	5	12	2
FRMD6	0.8577667	-0.221343	0.3338651	599	434	570	351	480	544
TXLNA	0.8593383	-0.218702	0.1518104	3332	3725	4005	2708	3383	3415
FRMD7	1.6428571	0.716207	0.4435521	8	2	4	10	7	6
MAPKAPK3	0.7734017	-0.37071	0.0270021	1053	1322	1332	907	1023	937
TXLNB	0.1818182	-2.459432	0.2680273	5	0	6	0	0	2
FRMD8	0.9007032	-0.150876	0.4941622	1020	1203	1332	996	1109	1097
MAPKAPK2	1.0076135	0.0109424	0.6326004	1055	1345	1409	1163	1396	1279
PFN1P1	1.0462185	0.0651842	0.7066569	53	105	80	99	58	92
PFN1P2	0.6666667	-0.584963	0.4234976	25	15	14	12	9	15
MAPKAPK5	0.8554673	-0.225215	0.2263756	770	872	1001	696	834	731
AL590705.5	0	#NUM!	0.5387425	3	0	0	0	0	0
PFN1P6	0.25	-2	0.4273495	0	2	6	0	0	2
AL590705.3	0.8873239	-0.172467	0.7880449	27	22	22	7	24	32

LINC01214	0.3	-1.736966	0.4218826	2	8	0	3	0	0
PFN1P3	0.2352941	-2.087463	0.1835543	8	6	3	0	0	4
LINC01215	0.5	-1	0.5773968	3	3	2	0	1	3
PFN1P4	#DIV/0!	#DIV/0!	0.4737294	0	0	0	0	4	0
LINC01254	#DIV/0!	#DIV/0!	0.8030358	0	0	0	1	0	0
LINC01250	2	1	0.834705	0	0	1	0	2	0
LINC01251	0	#NUM!	0.824326	1	0	0	0	0	0
UBFD1	0.8495396	-0.235247	0.107744	3037	3254	3592	2315	3176	2905
MAPRE1P1	3	1.5849625	0.6684577	1	0	0	0	0	3
AC092125.1	0	#NUM!	0.1787666	0	8	0	0	0	0
LINC01249	0.3846154	-1.378512	0.467975	4	0	9	2	3	0
AC104825.2	1.2335766	0.3028474	0.3216048	43	42	52	56	67	46
AC103987.1	0	#NUM!	0.824326	1	0	0	0	0	0
LINC01238	0.9349593	-0.097024	0.8689418	133	96	140	98	136	111
LINC01238	0.9349593	-0.097024	0.8689418	133	96	140	98	136	111
LINC01239	1.3125	0.3923174	0.7056201	10	4	2	5	8	8
LINC01234	0.5530973	-0.854395	0.0476587	90	49	87	19	65	41
LINC01237	1.3636364	0.447459	0.7668674	4	2	5	0	14	1
CALR3	0.7142857	-0.485427	0.8250553	3	1	3	0	0	5
EEF1A1P10	0	#NUM!	0.0490776	5	3	3	0	0	0
EEF1A1P11	1.2857143	0.3625701	0.5759537	6	5	17	11	18	7
EEF1A1P12	0.9	-0.152003	0.9546919	1	4	5	3	5	1
EEF1A1P13	1.1293103	0.175442	0.576951	29	48	39	47	45	39
EEF1A1P16	0.7333333	-0.447459	0.6622091	8	9	13	6	5	11
DNM1L	1.1872534	0.2476279	0.0739197	2583	1534	2473	2056	3025	2743
EEF1A1P19	1.2941176	0.3719688	0.6671251	26	1	7	9	19	16
AC007491.1	0	#NUM!	0.824326	1	0	0	0	0	0
FIRRE	1.4060606	0.4916588	0.0222503	246	122	292	249	350	329
AC011465.1	1.1666667	0.2223924	0.8824158	0	6	0	7	0	0
QTRT1	0.9553703	-0.065868	0.9557789	608	660	771	758	663	527
PPM1A	0.8376398	-0.255598	0.0906767	1681	1543	1962	1246	1610	1488
QTRT2	0.7678364	-0.381129	0.036254	1432	1318	1553	776	1145	1383
PPM1B	1.303778	0.3826982	0.0003138	4163	3704	4494	4285	5955	5876
PPM1L	1.0059701	0.0085875	0.8343512	133	81	121	90	128	119
PPM1M	1.6652542	0.7357425	0.0062371	55	77	104	150	136	107
PPM1N	1.2773826	0.3531908	0.0357121	208	236	259	298	343	257
PPM1H	0.6545203	-0.61149	0.0001588	675	686	807	379	519	521
AFG1L	0.8909774	-0.166539	0.6833819	161	150	221	154	148	172
AC254633.1	0.557377	-0.843274	0.211733	15	28	18	8	14	12
PPM1J	0.4411765	-1.180572	0.0940648	46	13	43	24	8	13
PPM1K	1.5243243	0.6081699	0.003996	130	93	147	180	202	182
BARX1	#DIV/0!	#DIV/0!	0.1930205	0	0	0	0	3	4
PPM1D	0.9571729	-0.063149	0.8962645	1217	1258	1331	1015	1335	1293
BARX2	1.9428571	0.9581798	0.0266686	15	18	37	39	41	56
PPM1E	0.7058824	-0.5025	0.6952557	6	5	6	3	2	7
PPM1F	0.7548924	-0.405657	0.0052592	1757	2073	2302	1404	1687	1538
PPM1G	1.0740811	0.1031029	0.1823136	7657	9049	9792	9428	10040	8993
AP000662.1	1	0	0.8647775	103	75	83	82	87	92
ELMSAN1	0.9874458	-0.018227	0.9187403	1295	1558	1528	994	1594	1738
LINC01205	1.0869565	0.1202942	0.7724206	26	22	21	20	34	21
LINC01206	0	#NUM!	0.6436235	2	0	0	0	0	0
LINC01202	0.8571429	-0.222392	0.8667588	4	3	0	0	4	2
LINC01204	0	#NUM!	0.824326	0	1	0	0	0	0
C4BPAP2	0	#NUM!	0.824326	1	0	0	0	0	0

MCOLN3	1.1417323	0.1912244	0.3520264	123	73	185	113	152	170
C4BPAP1	0.1	-3.321928	0.171089	6	0	4	0	0	1
AL357153.4	#DIV/0!	#DIV/0!	0.8030358	0	0	0	1	0	0
MCOLN1	0.8949631	-0.1601	0.5368773	449	607	572	466	547	444
TPD52L1	0.8216118	-0.283471	0.078372	1998	2116	2512	1800	1862	1782
MCOLN2	1.3592233	0.4427825	0.324142	38	42	23	28	50	62
TPD52L2	0.9353273	-0.096457	0.7555214	5581	6573	7004	5778	6383	5758
TTTY14	0.9555556	-0.065588	0.9469929	18	2	25	16	14	13
TTTY15	0.9764706	-0.034352	0.9639645	341	272	322	220	279	414
TTTY10	0	#NUM!	0.824326	0	0	1	0	0	0
TMPRSS11CP	0.3	-1.736966	0.425336	0	3	7	0	3	0
ASLP1	1.6	0.6780719	0.6621765	1	0	4	1	4	3
JPT1	1.0500766	0.0704946	0.3751289	2283	2945	3259	3216	3228	2468
STRADBP1	0	#NUM!	0.824326	1	0	0	0	0	0
LINC01194	1.1044177	0.1432859	0.4816318	68	87	94	87	85	103
JPT2	0.9792114	-0.030308	0.8433993	9605	11595	12328	9792	12085	10954
JMJD1C	1.3712807	0.4555239	0.2693596	621	282	643	369	894	857
AJ009632.2	0	#NUM!	0.0997075	0	0	13	0	0	0
RASSF1	0.836853	-0.256954	0.2441667	748	837	830	716	754	551
AP000911.3	1.8888889	0.9175378	0.3646091	5	4	9	3	28	3
RASSF2	0.8323721	-0.264699	0.0838709	3985	4460	4733	2841	4048	4080
RASSF3	0.9448497	-0.081843	0.7638241	2263	2167	2424	1770	2433	2273
AP000911.1	#DIV/0!	#DIV/0!	0.0557135	0	0	0	7	2	1
RASSF4	0.9216758	-0.117669	0.741321	206	191	152	161	189	156
RASSF5	0.25	-2	0.5138831	1	3	0	0	0	1
RASSF6	1.1182519	0.1612452	0.2339761	417	263	487	381	478	446
CALN1	1	0	0.9890559	0	0	1	0	1	0
LINC01186	0.25	-2	0.6181385	0	0	4	0	0	1
LINC01184	1.009901	0.0142139	0.9045662	23	37	41	19	37	46
LINC01185	#DIV/0!	#DIV/0!	0.4501902	0	0	0	0	0	4
CD2BP2	0.9105406	-0.135205	0.5418675	2172	2700	2841	2190	2528	2305
AC010240.3	0.5	-1	0.8638172	0	0	2	1	0	0
LINC01179	0	#NUM!	0.5387425	3	0	0	0	0	0
AC010240.1	2	1	0.636246	3	1	0	0	4	4
AC010240.2	0	#NUM!	0.824326	0	1	0	0	0	0
PLA1A	0	#NUM!	0.824326	0	0	1	0	0	0
ZMPSTE24	1.0515645	0.0725374	0.3494905	2887	2550	3096	2331	3333	3309
EEF1A1P30	2	1	0.6003078	2	0	2	0	4	4
EEF1A1P31	#DIV/0!	#DIV/0!	0.8030358	0	0	0	0	0	1
AC006329.1	0.8553055	-0.225488	0.3088973	471	467	617	430	464	436
EEF1A1P39	0	#NUM!	0.4521054	1	0	3	0	0	0
RASSF7	1.121021	0.1648133	0.1226753	1908	2322	2587	2849	2447	2346
AC092364.1	#DIV/0!	#DIV/0!	0.5070017	0	0	0	3	0	0
RASSF8	1.0870511	0.1204198	0.4101388	1885	986	1724	1148	1951	1896
AC092364.2	0.6206897	-0.688056	0.5439414	7	5	17	0	11	7
RASSF9	0.4821429	-1.052467	0.1832017	22	9	25	11	10	6
CALM3	1.0362416	0.0513604	0.3728138	10248	12803	14199	12872	13751	11977
KIAA1614-AS	1.2352941	0.3048546	0.7332348	6	1	10	2	8	11
CALM1	1.1583135	0.2120258	0.0085412	19335	18313	23686	21329	26541	23174
EEF1A1P29	#DIV/0!	#DIV/0!	0.8030358	0	0	0	1	0	0
CALM2	1.1378342	0.1862903	0.034566	6884	5886	7878	7009	9068	7417
INTS11	0.9912795	-0.012636	0.6721086	2258	2281	2800	2444	2606	2225
LINC01153	0	#NUM!	0.824326	1	0	0	0	0	0
INTS12	1.0918197	0.1267346	0.3840957	203	129	267	197	221	236

LINC01154	#DIV/0!	#DIV/0!	0.8030358	0	0	0	0	1	0
INTS13	1.1684588	0.2246068	0.1062523	986	592	933	848	1127	959
INTS14	0.8739009	-0.194458	0.2488019	1469	1484	1710	1219	1555	1301
LINC01150	7	2.8073549	0.3240543	1	0	0	0	0	7
INTS10	0.8784267	-0.187006	0.4144704	799	750	968	753	778	680
LINC01152	0	#NUM!	0.4374838	0	4	0	0	0	0
SECTM1	1.1376147	0.186012	0.5609316	16	34	59	35	35	54
AC139713.2	#DIV/0!	#DIV/0!	0.6554062	0	0	0	0	2	0
LINC01143	0	#NUM!	0.1377176	2	1	5	0	0	0
LINC01144	1.3	0.3785116	0.5010194	19	7	14	19	13	20
AC026765.1	0	#NUM!	0.824326	0	0	1	0	0	0
LINC01140	1.7647059	0.8194278	0.3711261	3	7	7	4	16	10
AC026765.2	0.7352941	-0.443607	0.6415764	9	10	15	8	8	9
LINC01139	0.3134328	-1.673772	0.0236747	17	18	32	3	9	9
AC092614.1	0.55	-0.862496	0.5139457	2	8	10	5	5	1
LINC01135	0	#NUM!	0.5781912	0	0	3	0	0	0
LINC01136	0.3157895	-1.662965	0.2598926	6	4	9	0	6	0
LINC01137	1.2571429	0.3301486	0.5898441	15	12	8	19	14	11
LINC01138	0.9927273	-0.010531	0.8219047	92	69	114	91	80	102
POPDC2	1	0	0.9890559	1	0	0	1	0	0
LINC01176	0.9632353	-0.05404	0.9384072	69	33	34	29	48	54
POPDC3	1.293578	0.371367	0.039938	179	173	193	241	258	206
DERL3	0	#NUM!	0.154788	7	0	1	0	0	0
DERL1	0.8014184	-0.319372	0.0436161	1078	1034	1131	772	902	925
DERL2	0.8993552	-0.153037	0.5381392	1103	1140	1324	1094	1201	913
TXLNG	1.0291333	0.0414298	0.6757518	489	373	511	309	502	602
AC103746.1	3.2727273	1.7104934	0.0137186	9	9	4	25	33	14
LINC01168	1	0	0.9889794	2	0	2	0	2	2
LINC01164	0.4285714	-1.222392	0.4455076	1	11	2	3	0	3
LINC01162	1	0	0.916143	12	9	21	8	9	25
LINC01163	17	4.0874628	0.0275909	0	0	1	4	9	4
AC117430.1	0	#NUM!	0.3451105	2	0	3	0	0	0
WWC1	0.7783361	-0.361535	0.0052263	6144	6850	7704	4703	6023	5384
WWC2	1.0535406	0.0752459	0.584034	2248	1566	1976	1254	2173	2673
PPME1	1.1646154	0.2198536	0.0306255	1648	2001	2201	2134	2505	2174
KHSRP	0.8653241	-0.208687	0.1804745	10814	12622	13757	9938	11612	10634
RNU1-70P	0	#NUM!	0.6436235	2	0	0	0	0	0
WWC3	0.7166152	-0.48073	0.0012441	1714	1987	2125	1134	1470	1571
AL159166.1	1.7848101	0.8357706	0.0398594	27	19	33	35	54	52
ERVK9-11	0.5714286	-0.807355	0.684514	5	0	2	0	3	1
AC024884.2	2	1	0.6991259	0	3	0	0	3	3
CALCB	0.6666667	-0.584963	0.8466488	3	0	0	0	0	2
RNU1-73P	0	#NUM!	0.1915466	1	0	7	0	0	0
AC024884.1	0	#NUM!	0.3626892	1	0	4	0	0	0
MIR4435-2HG	0.8689394	-0.202673	0.417842	368	430	522	364	444	339
CALD1	1.0600295	0.0841044	0.3546001	5243	3704	5296	3908	5810	5380
AK3P5	#DIV/0!	#DIV/0!	0.8030358	0	0	0	0	0	1
AK3P3	1.4705882	0.5563933	0.5193194	7	0	10	16	6	3
RNU1-72P	0.6666667	-0.584963	0.5530177	5	15	7	1	7	10
FDPS	0.7628246	-0.390577	0.0405918	789	1115	1254	721	818	870
TIMM10B	0.7368799	-0.440499	0.0200733	430	458	503	326	341	358
AC073651.3	1	0	0.9890559	0	0	1	1	0	0
PRKCSH	0.949825	-0.074266	0.9311782	7875	9828	10299	8985	9258	8354
CALB2	3.6238532	1.8575245	4.60E-09	21	36	52	120	134	141

CALB1	#DIV/0!	#DIV/0!	0.2887809	0	0	0	0	0	6
AC008966.1	0.8782201	-0.187345	0.6598595	108	124	195	90	125	160
MLST8	0.9636041	-0.053488	0.9126397	1267	1528	1711	1546	1478	1318
TIMM17A	0.9646146	-0.051975	0.9470288	12245	12351	14714	11973	14091	11855
AC008966.2	1	0	0.9760479	0	2	0	2	0	0
AC090061.1	2.8333333	1.5025003	0.2795884	4	1	1	15	0	2
TIMM17B	0.948444	-0.076365	0.9613905	1174	1496	1636	1467	1490	1127
POU1F1	0	#NUM!	0.2359908	1	0	6	0	0	0
AC106800.2	4	2	0.5580302	0	0	1	0	0	4
AZGP1P1	0	#NUM!	0.3482063	0	0	6	0	0	0
AZGP1P2	#DIV/0!	#DIV/0!	0.8030358	0	0	0	0	0	1
AC002456.1	0.7777778	-0.36257	0.7716883	10	2	6	3	5	6
BTF3L4P1	1	0	0.9696357	0	2	0	0	2	0
BTF3L4P2	1.1769231	0.23502	0.1655115	141	160	219	173	216	223
SAMHD1	1.6748208	0.7440067	1.16E-08	778	740	853	1052	1413	1506
AL512328.1	0	#NUM!	0.5337954	0	3	0	0	0	0
AC013275.1	2.5	1.3219281	0.609118	2	0	0	2	3	0
AC009554.1	#DIV/0!	#DIV/0!	0.8030358	0	0	0	0	1	0
FO681492.1	0.5	-1	0.1462734	32	13	21	8	11	14
AC005837.1	2.6666667	1.4150375	0.0316815	6	11	7	26	20	18
AC005837.3	#DIV/0!	#DIV/0!	0.8030358	0	0	0	1	0	0
AC002551.1	#DIV/0!	#DIV/0!	0.6554062	0	0	0	0	2	0
SOX15	1.676009	0.7450299	1.73E-06	276	266	350	511	496	488
AC116351.1	0.9865169	-0.019584	0.857048	378	440	517	332	433	552
FDXR	0.7585373	-0.398708	0.0275528	1780	2302	2536	1757	1678	1585
AC116351.2	0.7142857	-0.485427	0.5667753	16	8	32	11	15	14
UNC13B	1.0455293	0.0642334	0.4883516	1891	2095	2098	1555	2326	2480
UNC13C	1.5	0.5849625	0.35887	34	3	15	22	22	34
UNC13A	1.2256809	0.2935835	0.1972834	67	82	108	113	102	100
ZNF705G	2.3448276	1.2294818	0.0524033	14	4	11	21	25	22
ZNF705E	#DIV/0!	#DIV/0!	0.3246733	0	0	0	0	3	2
SOX13	0.9579713	-0.061946	0.956239	8700	10200	10794	8755	10024	9667
SOX14	0	#NUM!	0.824326	0	1	0	0	0	0
ZNF705A	#DIV/0!	#DIV/0!	0.6554062	0	0	0	0	2	0
SOX11	#DIV/0!	#DIV/0!	0.8030358	0	0	0	0	1	0
SOX12	1.2445367	0.3156088	0.0038085	1303	1502	1771	1888	1979	1828
UNC13D	0.8553719	-0.225376	0.2585088	996	1065	1327	972	981	945
RNU2-11P	1.1428571	0.1926451	0.8864005	3	4	0	6	2	0
FDX1	0.856702	-0.223135	0.2681495	1475	1669	2041	1515	1560	1367
AC009949.1	0.5	-1	0.81106	0	2	0	0	0	1
SKA3	1.0212342	0.0303138	0.5549363	1654	1149	1718	1358	1787	1472
HIF1A	1.3070998	0.3863693	0.0083906	7103	4413	7048	5903	9600	8762
SKA1	0.7713664	-0.374512	0.0376703	605	517	715	433	518	466
SKA2	0.98659	-0.019477	0.7870395	2094	1841	2329	1752	2378	2050
AC100858.2	0	#NUM!	0.6385096	0	2	0	0	0	0
AC106707.2	0	#NUM!	0.824326	1	0	0	0	0	0
H3F3B	1.0993551	0.1366575	0.0843503	11907	13299	14802	14166	15772	14045
H3F3C	1.6956522	0.7618403	0.1672778	9	11	26	31	34	13
H3F3A	1.1239878	0.1686264	0.0664484	2173	2547	2813	2668	3107	2692
MAD2L1BP	0.8858268	-0.174903	0.4029767	707	896	937	648	859	743
AC091045.1	0.885906	-0.174774	0.7482529	49	45	55	40	52	40
SOX30	1.0344828	0.0489096	0.9575904	13	9	7	5	17	8
KCNK15-AS1	0.1428571	-2.807355	0.1323658	10	2	2	0	0	2
HOXC-AS1	0.5	-1	0.7913401	0	1	3	2	0	0

HOXC-AS3	1.0833333	0.1154772	0.8307458	11	1	12	7	8	11
ZNF702P	0.9539474	-0.068018	0.9041614	500	378	490	359	515	431
UNC119	0.7804444	-0.357632	0.0940241	312	354	459	254	337	287
HIF3A	0.5	-1	0.4059724	4	10	6	0	6	4
FAM90A2P	0.8181818	-0.289507	0.9003841	1	2	8	0	2	7
AC025198.1	1.8461538	0.8845228	0.4363699	7	3	3	1	10	13
BATF2	6.16	2.6229304	1.66E-06	3	9	13	67	43	44
BATF3	1.3333333	0.4150375	0.730684	6	9	0	7	8	5
CNTD1	0.7846154	-0.349942	0.6807256	14	16	35	11	12	28
CNTD2	0.92	-0.120294	0.8869044	7	10	8	2	17	4
AL078645.1	#DIV/0!	#DIV/0!	0.4737294	0	0	0	0	4	0
AL078645.2	#DIV/0!	#DIV/0!	0.8030358	0	0	0	0	0	1
MIR503HG	1.2786177	0.354585	0.0525054	281	183	462	369	428	387
ASNA1	0.9890999	-0.015812	0.727968	1675	2094	2286	2023	2115	1851
AL603865.2	0	#NUM!	0.824326	1	0	0	0	0	0
AC055813.1	0	#NUM!	0.4278221	0	2	2	0	0	0
WWOX	0.8283499	-0.271688	0.1424895	568	570	668	417	526	553
WWP1	0.949846	-0.074234	0.9302709	835	537	901	607	819	733
WWP2	0.7716717	-0.373941	0.0099957	1977	2244	2712	1624	1950	1776
AACSP1	1.3501326	0.4331011	0.03487	126	97	154	147	180	182
RNU2-17P	3.8	1.9259994	0.1738403	0	4	1	16	1	2
COX6A1P2	1.2467532	0.318176	0.5767224	18	35	24	16	59	21
AL121877.1	0.3333333	-1.584963	0.6622061	0	3	0	0	0	1
TOR2A	1.1470302	0.1979034	0.1451529	316	321	390	437	384	357
AC109587.1	0.5217391	-0.938599	0.431428	16	2	5	0	8	4
CLSTN2	0.8285714	-0.271302	0.6660331	32	22	16	17	22	19
CLSTN3	0.7315585	-0.450955	0.014999	587	608	608	444	459	416
CLSTN1	0.7731248	-0.371227	0.0114994	2117	2543	2886	1736	2144	1954
RTKN2	1.962039	0.9723538	1.17E-06	328	191	403	441	699	669
AC132807.2	0.7786885	-0.360882	0.4702986	49	45	28	27	28	40
AC090948.2	1	0	0.9735258	12	1	4	4	3	10
AC090948.3	0.7241379	-0.465664	0.6672473	14	3	12	3	5	13
AC090948.1	0.1538462	-2.70044	0.1782671	7	1	5	2	0	0
TOR3A	2.252588	1.1715835	5.00E-11	148	155	180	351	374	363
INTS6L	0.8307692	-0.26748	0.5413772	137	65	188	78	130	116
AL023583.1	0.75	-0.415037	0.8178877	5	0	3	0	5	1
LINC01132	1.8333333	0.8744691	0.5581566	4	0	2	3	6	2
DNMT1	0.9209292	-0.118838	0.5322834	6986	7573	8041	5721	7547	7545
MINDY3	1.0274885	0.0391223	0.5626745	856	672	873	652	887	928
LINC01134	0.1666667	-2.584963	0.3136982	2	3	1	0	1	0
MINDY4	1.7333333	0.7935491	0.4029426	7	3	5	4	9	13
RTL10	0.8413258	-0.249263	0.1992593	816	857	1163	758	838	790
FECH	0.6014464	-0.733492	2.35E-06	785	734	970	445	513	539
MINDY1	0.8015873	-0.319068	0.3762047	105	118	155	111	91	101
MINDY2	0.9654842	-0.050675	0.9418951	1239	742	1148	615	1196	1210
AL138724.1	1.3870968	0.4720684	0.4703348	4	8	19	10	20	13
LINC01128	1.1374527	0.1858066	0.2000929	617	446	523	480	716	608
LINC01124	0.3974359	-1.331206	0.0480119	21	27	30	15	9	7
SKIL	1.1176471	0.1604647	0.2838891	778	450	727	536	839	810
LINC01126	1.9166667	0.9385995	0.3446374	2	1	9	5	6	12
LINC01127	1.15	0.2016339	0.5218271	24	30	46	45	42	28
AL158827.1	#DIV/0!	#DIV/0!	0.8030358	0	0	0	0	0	1
LINC01121	0	#NUM!	0.6385096	0	2	0	0	0	0
LINC01122	1.6666667	0.7369656	0.4589832	8	6	1	13	3	9

LINC01123	0	#NUM!	0.6385096	0	2	0	0	0	0
TOR1A	0.9541922	-0.067648	0.9390382	1409	1587	1894	1399	1774	1493
AC010731.2	#DIV/0!	#DIV/0!	0.3196487	0	0	0	0	1	4
TOR1B	1.0093822	0.0134725	0.5535413	1292	1398	1680	1333	1627	1451
AL158827.2	0.875	-0.192645	0.9836517	1	0	7	0	0	7
LINC01118	1.25	0.3219281	0.8835214	0	1	3	0	5	0
LINC01119	0.375	-1.415037	0.4608932	3	2	3	0	1	2
LINC01115	0.4	-1.321928	0.5529968	1	0	9	1	3	0
LINC01115	0.4	-1.321928	0.5529968	1	0	9	1	3	0
LINC01116	#DIV/0!	#DIV/0!	0.8030358	0	0	0	0	1	0
DAGLA	0.4719101	-1.083416	0.0026073	87	98	82	43	49	34
CSAG4	0.6760563	-0.564785	0.4348466	24	17	30	22	17	9
SNX18P3	1.4090909	0.4947647	0.6793468	6	15	1	2	15	14
CSAG2	2.2804878	1.1893425	0.4076634	5	72	5	113	7	67
CSAG3	0.9309036	-0.103296	0.8168974	406	344	567	325	545	356
CSAG1	1.1449016	0.1952236	0.1267814	518	475	684	708	690	522
LIMS1-AS1	0.6363636	-0.652077	0.6672391	3	4	4	2	2	3
AL117379.1	1.0793651	0.1101829	0.7430713	23	12	28	19	17	32
ASMTL	0.8151469	-0.294868	0.0806479	1476	1809	1957	1352	1444	1477
TESMIN	0.904878	-0.144205	0.6248668	247	268	305	209	263	270
DAGLB	1.0430069	0.0607487	0.3855093	780	878	1109	964	1066	856
SLC31A1P1	#DIV/0!	#DIV/0!	0.6099294	0	0	0	2	0	0
LINC01076	0.8	-0.321928	0.8579174	0	5	0	0	4	0
LINC01075	0	#NUM!	0.4521054	1	0	3	0	0	0
DNM1P51	0	#NUM!	0.2394948	0	0	8	0	0	0
AC113368.1	0	#NUM!	0.5075476	0	0	4	0	0	0
DNM1P46	3.4	1.7655347	0.2524354	3	2	0	1	3	13
DNM1P47	0.7204301	-0.47307	0.4586437	18	36	39	18	15	34
C6orf229	0	#NUM!	0.824326	0	1	0	0	0	0
C6orf226	0.6421569	-0.639002	0.1018722	57	62	85	39	46	46
C6orf222	0	#NUM!	0.3482063	0	0	6	0	0	0
SKP2	1.1320948	0.1789947	0.033042	8339	7766	9301	8008	10537	10217
C6orf223	0	#NUM!	0.6631647	1	0	1	0	0	0
SKP1	1.0480431	0.067698	0.2695405	2983	2533	3580	3044	3431	3058
ST3GAL6-AS1	0.984375	-0.02272	0.9231753	12	31	21	32	18	13
LINC01068	0.6	-0.736966	0.7951999	3	0	2	2	0	1
LINC01063	0.6842105	-0.547488	0.6792174	6	8	5	2	0	11
LINC01060	0	#NUM!	0.5387425	3	0	0	0	0	0
BAK1P1	0.3823529	-1.387023	0.1442926	8	14	12	3	7	3
LINC01058	0	#NUM!	0.824326	0	1	0	0	0	0
LINC01098	#DIV/0!	#DIV/0!	0.8030358	0	0	0	0	0	1
LINC01099	0.7586207	-0.398549	0.6799626	14	5	10	5	11	6
LINC01094	1.6666667	0.7369656	0.5377754	2	5	2	6	1	8
LINC01095	#DIV/0!	#DIV/0!	0.3200852	0	0	0	0	2	3
LINC01090	1.0238095	0.0339473	0.8658825	38	25	63	20	71	38
LINC01091	0.6067416	-0.720846	0.2351712	18	35	36	7	24	23
LINC01093	0	#NUM!	0.3267077	0	3	2	0	0	0
C6orf203	1.0412225	0.0582783	0.4364766	457	399	551	455	540	470
C6orf201	0.5	-1	0.8285813	0	0	4	2	0	0
LINC01087	0.7643979	-0.387604	0.39622	81	36	74	28	59	59
LINC01088	0.75	-0.415037	0.7626528	5	9	10	9	9	0
LINC01089	0.9727626	-0.03984	0.8774148	164	161	189	198	143	159
SENP3-EIF4A1	0.5454545	-0.874469	0.581558	5	6	0	1	5	0
LINC01082	#DIV/0!	#DIV/0!	0.8030358	0	0	0	0	0	1

SCARNA18B	0	#NUM!	0.5387425	3	0	0	0	0	0
AC139712.1	0.1818182	-2.459432	0.2202397	2	9	0	1	1	0
RTL8A	0.7837394	-0.351554	0.0692047	992	1393	1268	954	956	953
RTL8B	#DIV/0!	#DIV/0!	0.4737294	0	0	0	0	4	0
RTL8C	0.9469175	-0.078689	0.9594257	1435	1601	1749	1697	1531	1303
LINC01033	1	0	0.9890559	0	1	0	1	0	0
FEN1	0.8406582	-0.250409	0.1397013	4036	4740	5263	3929	4159	3714
LINC01031	0.2	-2.321928	0.2099776	0	10	5	0	3	0
AL358232.1	#DIV/0!	#DIV/0!	0.8030358	0	0	0	0	0	1
CAMKV	#DIV/0!	#DIV/0!	0.8030358	0	0	0	0	1	0
NKIRAS1	1.0075075	0.0107906	0.7264592	213	213	240	241	245	185
PCF11	1.0691569	0.0964736	0.3731346	1733	1052	1900	1288	1923	1798
NKIRAS2	0.9085154	-0.138417	0.5589925	1793	2105	2420	1811	1918	2011
LINC01029	2.8	1.4854268	0.2728185	1	1	3	12	1	1
AC103591.3	2	1	0.5301489	5	1	1	0	7	7
LINC01023	0.605042	-0.724893	0.2516251	25	26	68	31	24	17
CAMLG	0.9137826	-0.130077	0.5445562	1068	1003	1223	868	1154	988
AC069224.1	1.5	0.5849625	0.4471393	10	5	7	9	13	11
LINC01018	#DIV/0!	#DIV/0!	0.6482162	0	0	0	0	1	1
DAP3P2	0	#NUM!	0.824326	0	0	1	0	0	0
OPRD1	0.5	-1	0.571763	8	0	2	0	4	1
TOR4A	0.6851064	-0.5456	0.1600946	52	93	90	44	55	62
LINC01055	#DIV/0!	#DIV/0!	0.6099294	0	0	0	2	0	0
EIF4A2P2	1	0	0.9658162	3	0	2	2	2	1
AC073410.1	2.6666667	1.4150375	0.5105046	0	3	0	1	5	2
AC073410.2	#DIV/0!	#DIV/0!	0.6411933	0	0	0	0	0	2
TBL1Y	1.3277778	0.4090137	0.2098793	37	83	60	68	80	91
TBL1X	0.7926379	-0.335266	0.0169033	3111	3386	3799	2210	2978	2973
AC103591.1	4	2	0.5810589	0	0	1	0	4	0
AC019294.1	0	#NUM!	0.824326	0	0	1	0	0	0
AC019294.2	0.4285714	-1.222392	0.6785079	0	0	7	1	0	2
AC019294.3	#DIV/0!	#DIV/0!	0.4737294	0	0	0	0	4	0
SMIM15-AS1	#DIV/0!	#DIV/0!	0.4501902	0	0	0	0	0	4
RSL1D1	1.1279656	0.173723	0.0594818	8636	6611	9369	8861	10121	8784
DNM1P35	2	1	0.4227025	1	0	6	9	4	1
DNM1P33	#DIV/0!	#DIV/0!	0.6554062	0	0	0	0	2	0
CAMK4	1	0	0.9871653	9	2	5	3	9	4
B9D1	0.9836957	-0.023716	0.8243691	325	368	411	393	400	293
B9D2	1.0470588	0.0663425	0.7844128	28	25	32	27	26	36
CAMK1	1.1355932	0.1834461	0.6928582	15	21	23	13	24	30
LINC01036	0.8939394	-0.161751	0.7844637	28	28	10	11	21	27
LINC01037	1	0	0.9295557	8	13	25	6	17	23
EIF2S2P3	0.5	-1	0.8289598	1	0	1	0	1	0
EIF2S2P4	1.125525	0.1705981	0.1179069	702	566	875	759	908	745
STEAP3	0.5338346	-0.905535	3.40E-07	478	582	669	263	357	303
EIF2S2P2	#DIV/0!	#DIV/0!	0.5371397	0	0	0	0	0	3
STEAP4	0.4333333	-1.206451	0.2730508	6	11	13	6	1	6
AC106791.2	0	#NUM!	0.824326	1	0	0	0	0	0
AC106791.1	0.8648649	-0.209453	0.7834834	16	33	25	17	14	33
BASP1	0.9427933	-0.084987	0.8383129	15519	17983	20163	16300	18221	16074
AK4P3	0.755102	-0.405256	0.6194049	13	17	19	11	22	4
AK4P1	2.6	1.3785116	0.355518	9	1	0	0	7	19
RAP2C-AS1	0.6666667	-0.584963	0.4750609	15	10	14	6	11	9
NPY1R	1.1764706	0.2344653	0.8815119	15	0	2	2	13	5

STEAP1	1.0251736	0.0358682	0.626505	363	336	453	289	448	444
STEAP2	5	2.3219281	0.3198252	1	0	1	0	10	0
AC010387.1	#DIV/0!	#DIV/0!	0.8030358	0	0	0	0	0	1
AC090312.2	0	#NUM!	0.824326	0	1	0	0	0	0
SEZ6L2	0.8670886	-0.205749	0.4802301	262	288	398	254	339	229
AC109347.2	0.4285714	-1.222392	0.5918744	1	1	5	0	3	0
AC109347.1	0.7746479	-0.368387	0.5839968	27	18	26	19	23	13
RAP1GAP2	0.7860629	-0.347283	0.0306177	1059	1279	1350	807	1069	1023
AC106801.1	0	#NUM!	0.1069957	4	5	0	0	0	0
AC005586.1	0.5	-1	0.5529676	5	2	3	0	1	4
AC005586.2	1	0	0.9555239	1	3	2	3	0	3
DNMBP	0.750242	-0.414572	0.0525122	315	372	346	239	260	276
CRISP2	0.3333333	-1.584963	0.7290749	0	0	3	0	0	1
NPY6R	0.3333333	-1.584963	0.5378981	3	3	0	2	0	0
CRISP3	0.25	-2	0.3566294	2	2	4	0	2	0
PNPT1P1	2	1	0.8147319	1	0	0	0	0	2
AC092999.1	0	#NUM!	0.5643268	1	0	2	0	0	0
MADCAM1	0.84	-0.251539	0.9280371	4	5	16	12	6	3
DESI1	0.7476013	-0.419659	0.0111811	1037	1285	1430	865	942	998
DESI2	0.929821	-0.104975	0.6525677	1690	1506	1663	1024	1747	1747
MIR2909	0	#NUM!	0.824326	1	0	0	0	0	0
AC091046.2	0	#NUM!	0.6436235	2	0	0	0	0	0
AL132800.1	1.0833333	0.1154772	0.9436427	9	1	2	1	6	6
ITGB1BP1	0.9354839	-0.096215	0.8035702	719	673	933	625	777	773
ITGB1BP2	0.8813559	-0.182203	0.8533688	26	10	23	18	22	12
AC009313.1	0	#NUM!	0.3482063	0	0	6	0	0	0
OPRL1	1.0952381	0.1312445	0.7809579	11	21	10	22	10	14
RNU2-22P	0.4137931	-1.273018	0.2387253	11	7	11	0	8	4
DUXAP10	1.0054705	0.0078707	0.8336053	376	198	340	217	396	306
CYP1B1-AS1	1.2857143	0.3625701	0.566681	3	1	24	14	6	16
AL358472.1	1	0	0.9890559	0	1	0	1	0	0
AL358472.4	0.3584906	-1.479993	0.0587282	25	15	13	3	8	8
AL358472.5	0.5	-1	0.7417242	0	0	6	0	3	0
OPRK1	2.556338	1.3540786	5.73E-06	46	53	43	97	141	125
AL358472.2	1.0227273	0.0324215	0.8908219	26	38	24	28	35	27
AL358472.3	1.75	0.8073549	0.4778713	1	2	5	7	5	2
SHCBP1	0.9747813	-0.036849	0.9462405	3042	2565	3196	2254	3323	3004
GNPDA1	0.7479798	-0.418929	0.0019	4033	4734	5093	3134	3712	3521
GNPDA2	0.9803279	-0.028664	0.928095	107	58	140	59	114	126
PWWP2B	1.2398844	0.3102056	0.006115	1489	1613	1742	2075	2070	1861
SLA2	1.2307692	0.2995603	0.7565501	3	6	4	10	6	0
TIPARP-AS1	1.3	0.3785116	0.7284985	2	2	6	3	8	2
DPYSL4	1.2	0.2630344	0.8729088	0	3	2	1	2	3
DPYSL5	0.993617	-0.009238	0.8210365	694	821	835	616	891	828
DPYSL2	0.9263158	-0.110424	0.7143182	467	458	500	331	440	549
DPYSL3	0.5833333	-0.777608	0.5746816	8	3	1	1	4	2
PWWP2A	0.9633193	-0.053914	0.9896841	1177	889	1260	863	1217	1124
VSTM5	14	3.8073549	0.0699509	0	0	1	6	8	0
FNBP4	0.8475978	-0.238548	0.1725531	2097	1707	2482	1395	2010	1923
FNBP1	1.2652455	0.3394174	0.0032381	1153	1227	1326	1253	1729	1707
MTHFD2P7	0.75	-0.415037	0.9156204	0	0	4	0	2	1
FEZ1	0.8454198	-0.24226	0.4832111	136	202	186	164	151	128
FEZ2	1.0010042	0.001448	0.6656078	2276	2043	2652	1883	2699	2396
PLBD1	0.8982939	-0.154741	0.370372	2758	3000	3327	2418	2959	2784

TXNIP	1.490566	0.5758603	0.233524	14	15	24	25	21	33
PLBD2	0.7278769	-0.458234	0.0013891	1544	1792	1930	1083	1424	1326
SLBP	0.9236179	-0.114632	0.6326606	2779	3018	3446	2695	3107	2735
AL137847.1	0	#NUM!	0.5172237	1	2	0	0	0	0
AP000766.1	1.7058824	0.7705182	0.3158997	12	0	22	9	25	24
LMCD1-AS1	0.8157895	-0.293731	0.7735173	11	8	19	9	14	8
AC010149.1	1.3333333	0.4150375	0.8484702	0	3	0	4	0	0
BTBD19	1.2121212	0.277534	0.6486375	6	14	13	13	17	10
CREBBP	0.7517506	-0.411674	0.0190477	2352	2482	2735	1311	1996	2383
BTBD18	0.125	-3	0.2240213	4	4	0	0	1	0
BTBD16	1	0	0.9890559	0	0	1	0	0	1
BTBD11	0.2121212	-2.237039	0.004483	28	21	17	2	4	8
Z95624.1	#DIV/0!	#DIV/0!	0.5371397	0	0	0	0	0	3
BTBD10	0.9609923	-0.057403	0.970684	1811	1689	2063	1583	2123	1640
AC008438.1	1	0	0.9483166	19	19	25	15	35	13
AC012306.2	0.8055556	-0.311944	0.368885	93	86	109	54	87	91
AC133681.1	0.9642857	-0.052467	0.9949671	14	8	6	11	4	12
AC116158.1	0.25	-2	0.348119	4	0	8	0	0	3
JRKL	0.918259	-0.123027	0.6419356	680	520	684	389	686	655
AC005786.4	#DIV/0!	#DIV/0!	0.8030358	0	0	0	0	1	0
AL021920.3	#DIV/0!	#DIV/0!	0.1593104	0	0	0	6	0	1
AL021920.2	0.6666667	-0.584963	0.8234316	0	3	0	0	2	0
NPM1P46	0	#NUM!	0.5337954	0	3	0	0	0	0
AC061975.2	0	#NUM!	0.824326	1	0	0	0	0	0
AC068587.6	0.72	-0.473931	0.4645017	30	18	52	10	45	17
AC061975.3	0.4375	-1.192645	0.3912743	2	8	6	0	5	2
AC061975.1	0.88	-0.184425	0.7427366	21	38	16	13	33	20
AC061975.6	1.3333333	0.4150375	0.6409804	2	2	8	6	5	5
AC061975.7	1.2717149	0.3467753	0.1265405	137	134	178	119	196	256
AC068587.4	0	#NUM!	0.3626892	1	0	4	0	0	0
AC061975.5	1.0408163	0.0577155	0.9037964	15	12	22	4	25	22
AC061975.8	0.2631579	-1.925999	0.2084943	1	9	9	3	2	0
NPM1P39	1.0297619	0.0423108	0.7257878	38	54	76	74	59	40
AC020922.4	1.5	0.5849625	0.769649	0	0	4	0	5	1
NPM1P38	0	#NUM!	0.6385096	0	2	0	0	0	0
NPM1P32	1	0	0.9960348	4	3	2	1	4	4
OR2X1P	0	#NUM!	0.824326	0	1	0	0	0	0
SLF1	1.1749814	0.2326379	0.1642621	532	275	536	428	604	546
SLF2	1.1620284	0.2166454	0.1801375	626	383	608	443	725	711
NPM1P26	0.375	-1.415037	0.4785081	3	1	4	0	1	2
NPM1P27	1.0926142	0.1277841	0.1375007	4736	3849	5970	5044	6044	4815
NPM1P24	0.6363636	-0.652077	0.7036421	5	2	4	4	1	2
NPM1P25	2.0909091	1.0641303	0.2991228	7	1	3	11	5	7
MYBBP1A	0.8125786	-0.299421	0.0747929	2352	3043	3350	2233	2463	2410
AC104823.1	1	0	0.9890559	0	0	1	0	1	0
THRB-IT1	0.1666667	-2.584963	0.3987778	0	1	5	0	0	1
NPM1P21	2.5	1.3219281	0.3031603	3	1	2	4	4	7
CIAPIN1	0.8652423	-0.208824	0.2633802	3056	3767	3989	3099	3283	2973
OR7E4P	5	2.3219281	0.4461666	0	0	1	0	4	1
MAP3K7CL	3.75	1.9068906	0.2575301	0	1	3	0	2	13
AC003973.1	0	#NUM!	0.6385096	0	2	0	0	0	0
AC003973.2	0	#NUM!	0.6436235	2	0	0	0	0	0
KIR3DX1	0	#NUM!	0.824326	0	0	1	0	0	0
AC003973.3	2	1	0.7025867	1	0	1	0	3	1

AC078942.1	0	#NUM!	0.6385096	0	2	0	0	0	0
TSPOAP1-AS1	0.8	-0.321928	0.9024935	2	0	3	0	2	2
AC012150.1	0	#NUM!	0.5387425	3	0	0	0	0	0
AC012150.2	#DIV/0!	#DIV/0!	0.3109758	0	0	0	0	6	0
AJUBA	0.9113733	-0.133886	0.5015776	2845	3427	3646	2616	3287	3136
AC005306.1	#DIV/0!	#DIV/0!	0.6099294	0	0	0	2	0	0
GRIA2	0	#NUM!	0.6840298	0	0	2	0	0	0
OR7E2P	0	#NUM!	0.824326	0	0	1	0	0	0
CREBL2	0.8294162	-0.269832	0.1516693	827	815	996	512	894	782
AC104333.4	0	#NUM!	0.6436235	2	0	0	0	0	0
AC104333.2	0	#NUM!	0.4278221	0	2	2	0	0	0
AC104333.3	#DIV/0!	#DIV/0!	0.5082165	0	0	0	1	0	2
TNFRSF17	0.5	-1	0.8652929	0	0	2	0	1	0
TNFRSF14	3.25	1.7004397	0.3481466	0	0	4	0	9	4
GRIA3	0	#NUM!	0.5387425	3	0	0	0	0	0
GRIA4	2	1	0.3422637	5	1	6	3	5	16
AC096720.2	3.375	1.7548875	0.1074581	4	2	2	4	10	13
AL133241.1	0	#NUM!	0.824326	0	1	0	0	0	0
AC096720.1	0.1666667	-2.584963	0.3235376	1	3	2	0	1	0
TXNP4	#DIV/0!	#DIV/0!	0.288876	0	0	0	3	2	0
BX324167.2	#DIV/0!	#DIV/0!	0.6554062	0	0	0	0	2	0
TXNP5	3	1.5849625	0.6253252	0	0	1	3	0	0
TXNP6	1.75	0.8073549	0.4902236	3	1	4	5	6	3
AC009121.1	1.060241	0.0843922	0.7476123	26	29	28	36	23	29
OR7E1P	0.4	-1.321928	0.6304764	3	2	0	2	0	0
AC009121.2	1.0652174	0.0911479	0.9138207	4	22	20	2	30	17
OR7E8P	#DIV/0!	#DIV/0!	0.8030358	0	0	0	0	1	0
AC016026.1	0.5	-1	0.4693701	4	3	9	1	3	4
GRID2	0.9964664	-0.005107	0.8833132	95	62	126	53	105	124
GRID1	#DIV/0!	#DIV/0!	0.8030358	0	0	0	1	0	0
OR7E7P	0.5	-1	0.6003191	1	4	3	0	4	0
TNFRSF19	0.7916667	-0.337035	0.759537	8	6	10	5	10	4
AP003168.1	0	#NUM!	0.824326	0	0	1	0	0	0
ARFGAP3	0.9052946	-0.143541	0.5650702	455	395	491	319	449	446
TNFRSF1B	3.119296	1.6412204	2.27E-32	460	451	623	1552	1808	1425
AP003168.2	0	#NUM!	0.824326	1	0	0	0	0	0
ARFGAP1	0.8420867	-0.247959	0.1747205	3208	3829	4330	3266	3323	2983
ARFGAP2	0.9263188	-0.110419	0.6889236	1992	2431	2648	2065	2339	2146
TNFRSF1A	0.8837847	-0.178233	0.4336603	1599	1985	2250	1750	1796	1610
CCNL2P1	1.5	0.5849625	0.8695629	0	2	0	0	0	3
TNFRSF25	1.1751825	0.2328848	0.2974544	70	59	145	111	114	97
AC091182.2	#DIV/0!	#DIV/0!	0.4501902	0	0	0	0	0	4
TNFRSF21	1.1692847	0.2256262	0.016164	2904	3057	3461	3075	3924	4018
CANT1	0.7603297	-0.395303	0.0066707	2537	3063	3379	2042	2590	2195
CARNMT1	1.1159592	0.1582843	0.1958072	1446	1125	1646	1100	1801	1805
AC083843.1	1.6666667	0.7369656	0.8035901	0	3	0	0	4	1
AC083843.2	0.8377153	-0.255468	0.4867176	501	170	432	273	344	307
AC083843.3	1.6	0.6780719	0.3332711	5	8	17	15	27	6
OR7E5P	0	#NUM!	0.6631647	1	0	1	0	0	0
RN7SL738P	2.125	1.0874628	0.4192272	1	7	0	10	4	3
AC005546.1	0.5	-1	0.4057767	5	10	5	0	4	6
TXNL1	0.9244089	-0.113397	0.6664148	963	901	1139	839	1029	908
AL133387.2	3	1.5849625	0.3641941	1	0	2	2	1	6
AC025449.1	2.2222222	1.1520031	0.3031615	4	0	5	12	2	6

AL121932.1	0	#NUM!	0.824326	1	0	0	0	0	0
THSD7B	1	0	0.997461	0	2	0	1	1	0
POLH-AS1	0.8948413	-0.160296	0.6341533	176	148	180	137	177	137
THSD7A	0.3924051	-1.349584	0.0727927	15	24	40	2	22	7
ITFG2-AS1	0.3333333	-1.584963	0.6646052	2	0	1	0	1	0
ITFG2-AS1	0.3333333	-1.584963	0.6646052	2	0	1	0	1	0
RN7SL736P	#DIV/0!	#DIV/0!	0.6099294	0	0	0	2	0	0
SLPI	0.8567901	-0.222986	0.5505452	154	102	149	120	121	106
PNLDC1	0	#NUM!	0.824326	0	0	1	0	0	0
CHCHD2P2	0.0833333	-3.584963	0.1137596	0	10	2	0	1	0
CHCHD2P4	0	#NUM!	0.5387425	3	0	0	0	0	0
ACTR3B	0.874818	-0.192945	0.5935231	209	199	279	216	184	201
TTBK2	0.8349705	-0.260203	0.4353751	209	119	181	101	140	184
ACTR3C	0.2978723	-1.747234	0.0910984	13	6	28	8	1	5
AC016027.5	0.2857143	-1.807355	0.5378347	1	0	6	2	0	0
AF213884.3	0.2727273	-1.874469	0.288548	1	9	1	0	2	1
AC016027.2	0	#NUM!	0.5781912	0	0	3	0	0	0
AC016027.1	0.9302326	-0.104337	0.9837839	11	7	25	12	23	5
NBEAL1	0.9627119	-0.054824	0.9747023	565	201	414	231	375	530
NBEAL2	0.8868763	-0.173195	0.357305	899	960	1067	740	934	921
NAP1L4P2	0	#NUM!	0.2605273	0	3	3	0	0	0
NAP1L4P3	2	1	0.7300836	1	0	1	0	4	0
NAP1L4P1	0.6551724	-0.610053	0.4746606	18	10	30	17	8	13
AL008727.1	1.6666667	0.7369656	0.7000735	0	5	1	0	4	6
CHCHD2P8	#DIV/0!	#DIV/0!	0.8030358	0	0	0	0	0	1
CHCHD2P9	1.25	0.3219281	0.8505473	0	0	4	0	0	5
HIBADH	0.9393207	-0.09031	0.7590717	2653	2797	3235	2423	2890	2845
AC027117.1	1.0555556	0.0780025	0.9081444	5	8	5	7	9	3
GRIK5	6	2.5849625	0.3544141	0	0	1	0	1	5
GRIK4	9	3.169925	0.0483894	0	0	2	7	4	7
AC027117.2	0	#NUM!	0.6436235	2	0	0	0	0	0
GRIK1	4.5	2.169925	0.1907863	0	3	1	0	9	9
GRIK2	0.2631579	-1.925999	0.3444214	4	0	15	5	0	0
ACTR1A	1.0882105	0.1219576	0.1707213	2350	2882	3055	3062	2980	2976
ACTR1B	1.163944	0.2190216	0.0388673	1502	1751	1889	1990	2178	1817
RNU6-824P	#DIV/0!	#DIV/0!	0.8030358	0	0	0	0	0	1
AC009996.1	0.5	-1	0.6077657	1	2	7	0	5	0
CNTRL	1.3192376	0.3997045	0.0167547	644	366	669	595	868	752
AC096721.1	0	#NUM!	0.3963337	1	3	0	0	0	0
SLTM	1.2806229	0.3568457	0.0195881	2329	1265	2635	2257	3049	2671
SLU7	1.0382214	0.0541141	0.4420346	1956	1380	1949	1598	2054	1835
AL133481.1	#DIV/0!	#DIV/0!	0.8030358	0	0	0	0	1	0
CELSR3-AS1	0.65	-0.621488	0.7654175	11	2	7	13	0	0
FITM1	0.6923077	-0.530515	0.823293	4	0	9	6	2	1
FITM2	0.6956032	-0.523664	0.0243576	262	305	320	170	190	257
AC007359.1	#DIV/0!	#DIV/0!	0.5371397	0	0	0	0	0	3
SLX4	0.7510149	-0.413087	0.0325897	674	701	842	402	569	694
ORMDL2	0.8945827	-0.160713	0.6065606	194	237	252	190	230	191
RNU6-826P	0.5	-1	0.8652929	0	0	2	0	1	0
ORMDL3	1.0282087	0.0401332	0.5180108	971	1283	1291	1115	1350	1180
AC112187.3	1.75	0.8073549	0.670142	1	3	0	7	0	0
AC099518.1	0.844086	-0.244538	0.6613522	50	60	76	64	54	39
AC099518.2	2	1	0.8319548	0	1	0	0	2	0
AC099518.4	0	#NUM!	0.4278221	0	2	2	0	0	0

AC099518.6	0.6666667	-0.584963	0.8372258	2	0	1	0	2	0
ORMDL1	1.0467179	0.0658727	0.4208219	1276	878	1228	1059	1190	1291
GRIN1	0.952381	-0.070389	0.9868061	18	27	39	20	34	26
AL136133.1	0.875	-0.192645	0.790362	18	32	38	17	26	34
ACTR10	0.9831461	-0.024522	0.7779857	1389	1297	1586	1299	1510	1391
GRINA	0.8998162	-0.152298	0.544083	1933	2106	2489	2052	2065	1757
OR2W6P	0.65	-0.621488	0.6150638	5	5	10	2	3	8
NPM1P19	0	#NUM!	0.824326	1	0	0	0	0	0
AL121839.2	2	1	0.1230533	15	1	21	32	18	24
NPM1P18	0	#NUM!	0.6385096	0	2	0	0	0	0
GRIP1	0.9962567	-0.005411	0.8158396	665	513	692	448	674	741
NPM1P14	#DIV/0!	#DIV/0!	0.8030358	0	0	0	1	0	0
NPM1P10	0	#NUM!	0.824326	0	0	1	0	0	0
FBXW12	1.05	0.0703893	0.8674985	9	5	6	11	4	6
PCID2	0.8892163	-0.169394	0.361144	1023	1143	1228	856	1143	1019
FBXW10	0	#NUM!	0.0962815	2	0	9	0	0	0
FBXW11	0.8756843	-0.191517	0.3033047	1902	1839	1922	1208	1782	1969
TTC7B	0.9590999	-0.060247	0.9559946	1893	1983	2212	1738	2085	2016
TTC7A	0.919084	-0.121731	0.6201379	1158	1331	1441	1134	1302	1176
GRIP2	0.3333333	-1.584963	0.5728962	0	6	0	2	0	0
AC105411.1	1.1621622	0.2168114	0.6140747	21	25	28	19	40	27
CNTLN	1.2857143	0.3625701	0.8043701	4	2	1	2	3	4
CNTN5	0.4545455	-1.137504	0.3510939	10	4	8	0	8	2
AL354710.2	0	#NUM!	0.824326	0	1	0	0	0	0
PCIF1	0.8686834	-0.203098	0.3570667	880	928	1101	872	817	838
AC017104.5	0.7619048	-0.392317	0.5012335	34	25	46	9	36	35
CNTN1	0.0666667	-3.906891	0.0530821	5	2	8	0	0	1
RSPH14	1	0	0.9667675	4	0	0	0	4	0
AC017104.2	0	#NUM!	0.5075476	0	0	4	0	0	0
CNTN2	0	#NUM!	0.5337954	0	3	0	0	0	0
AC017104.3	0.3333333	-1.584963	0.6851201	1	0	2	0	1	0
AC017104.4	1.1931818	0.2548139	0.5417395	37	13	38	52	42	11
CNTN4	0.5268817	-0.924449	0.0100724	93	93	93	30	49	68
TTC25	1.4794521	0.5650629	0.1784533	25	15	33	41	29	38
TTC26	0.9151671	-0.127893	0.7977605	144	106	139	116	112	128
TTC23	0.9409357	-0.087832	0.8301517	531	561	618	491	550	568
RNU1-51P	0.8571429	-0.222392	0.8877252	0	5	2	0	6	0
TTC22	1.0886076	0.122484	0.7636754	25	34	20	26	34	26
AC130456.1	0	#NUM!	0.6840298	0	0	2	0	0	0
AC130456.2	0	#NUM!	0.0653515	2	6	2	0	0	0
AC130456.3	0	#NUM!	0.824326	0	0	1	0	0	0
AC130456.4	1	0	0.9890559	1	0	0	1	0	0
TTC29	#DIV/0!	#DIV/0!	0.242062	0	0	0	6	0	0
TTC27	1.0369565	0.0523554	0.5084762	783	691	826	621	929	835
TTC28	0.9777448	-0.03247	0.9765748	239	203	232	148	219	292
AC130456.7	0	#NUM!	0.5781912	0	0	3	0	0	0
GUSBP9	0.5625	-0.830075	0.6887098	0	0	16	2	4	3
GUSBP3	0.6941176	-0.526748	0.3895932	23	27	35	20	16	23
TTC36	1.6666667	0.7369656	0.8009609	0	3	0	0	0	5
TTC37	1.2167862	0.2830757	0.3357798	2880	1272	2818	2085	3352	3044
GUSBP1	1.0809917	0.1123555	0.2745993	355	368	487	428	420	460
TTC34	2	1	0.4941251	0	4	2	9	0	3
GUSBP2	0.4901961	-1.028569	0.2069656	15	14	22	7	4	14
TTC33	1.0396197	0.0560558	0.6333826	237	174	220	173	245	238

MIR205HG	1.12	0.1634987	0.7865072	4	11	10	9	8	11
AC010148.1	0.6995885	-0.515422	0.2085322	90	54	99	52	46	72
TTC31	0.7745491	-0.368571	0.0392913	629	644	723	509	553	484
TTC38	0.7834395	-0.352106	0.0410829	727	858	927	612	678	678
AC092656.1	0.4	-1.321928	0.4660774	3	6	6	6	0	0
SLC3A1	0.3846154	-1.378512	0.3893891	3	6	4	3	2	0
SLC3A2	1.0935541	0.1290245	0.1576676	13487	16949	17408	17928	18402	15990
CAND2	0.3529412	-1.5025	0.2715647	4	11	2	0	3	3
CAND1	1.0811185	0.1125247	0.3558366	7010	4294	6756	4614	7490	7421
TTC14	0.854083	-0.227552	0.4602902	617	281	596	300	495	481
TTC12	0.8513324	-0.232206	0.4553977	208	197	308	183	192	232
TTC13	0.8938193	-0.161945	0.516294	426	368	468	297	398	433
AP000526.1	1.1298701	0.176157	0.6479132	15	30	32	22	30	35
NBEAP1	0.5588235	-0.839535	0.452147	6	6	22	0	13	6
TTC19	0.8443131	-0.24415	0.1652095	1224	987	1341	846	1074	1079
TTC16	0	#NUM!	0.6385096	0	2	0	0	0	0
TTC17	0.9085917	-0.138296	0.5942177	1021	853	1222	715	970	1128
OPN1SW	1.1330472	0.180208	0.4405429	91	50	92	81	101	82
AC023813.3	#DIV/0!	#DIV/0!	0.6317974	0	0	0	1	1	0
AC023813.4	0	#NUM!	0.5337954	0	3	0	0	0	0
SMC5	1.3661972	0.4501657	0.0057228	1463	812	1559	1349	2010	1879
SLC4A2	0.8626266	-0.213192	0.1919431	3066	3618	4075	2761	3262	3258
SMC6	1.1658125	0.2213358	0.1583301	1277	660	1380	1052	1613	1202
SLC4A3	0.7555556	-0.40439	0.2334389	80	131	104	68	85	85
SMC3	1.5647554	0.6459372	0.0001473	2088	1108	2263	2335	3452	2755
SLC4A4	#DIV/0!	#DIV/0!	0.6554062	0	0	0	0	2	0
SMC4	1.0587683	0.0823869	0.5066694	2891	1513	3049	1785	3188	2918
SLC4A5	2.1666667	1.1154772	0.4006909	1	2	3	4	1	8
SMC2	1.3198883	0.4004159	0.0288181	3001	1366	2798	2317	3696	3444
CDH12P1	0	#NUM!	0.5337954	0	3	0	0	0	0
CDH12P2	0	#NUM!	0.6436235	2	0	0	0	0	0
RNY1P16	0.2857143	-1.807355	0.4902857	2	0	5	0	0	2
SBDSP1	0.8117647	-0.300866	0.0766833	891	772	972	597	758	784
AC022007.1	1.0188679	0.026967	0.8336036	7	22	24	29	14	11
RPS20P10	#DIV/0!	#DIV/0!	0.2760964	0	0	0	1	3	1
AL929472.4	#DIV/0!	#DIV/0!	0.8030358	0	0	0	0	0	1
RPS20P14	0.9740933	-0.037868	0.8519659	49	52	92	88	60	40
NUGGC	0	#NUM!	0.3414317	0	2	3	0	0	0
AC008677.2	0	#NUM!	0.824326	0	1	0	0	0	0
PLAC8	0.9509595	-0.072544	0.9460668	139	148	182	134	178	134
FAM171A1	3	1.5849625	0.2229175	3	5	0	7	1	16
PLAC9	2	1	0.7491496	0	2	0	0	3	1
RPS20P22	0.8504673	-0.233672	0.6476803	41	37	29	23	39	29
FAM171A2	0.9536785	-0.068425	0.9391475	117	138	112	120	128	102
NCSTN	0.8753946	-0.191995	0.2461346	3259	3901	4244	2828	3789	3366
PLAC1	1.8042553	0.8514035	0.0001119	130	183	157	255	359	234
PLAC4	2.5866667	1.3710942	0.0004681	28	15	32	56	64	74
RPL10P16	0.9827229	-0.025143	0.7970103	381	494	572	523	501	398
RPL10P19	3	1.5849625	0.5468748	1	0	1	0	6	0
POU6F2	1.2701422	0.34499	0.0767519	146	111	165	150	196	190
RNY1P13	0.8333333	-0.263034	0.8983226	2	1	3	0	3	2
RPL10P12	1	0	0.9940832	0	2	0	1	0	1
RPL10P13	0.4285714	-1.222392	0.6569726	0	3	4	3	0	0
RPL10P14	#DIV/0!	#DIV/0!	0.6411933	0	0	0	0	0	2

RNY1P12	0	#NUM!	0.6436235	2	0	0	0	0	0
RPL10P15	0	#NUM!	0.824326	0	1	0	0	0	0
POU6F1	1.472973	0.558731	0.1982518	18	23	33	31	50	28
RPS20P33	0.6734694	-0.570316	0.5415945	5	23	21	16	12	5
RPS20P35	4	2	0.5355961	0	1	0	0	3	1
RPS20P31	0	#NUM!	0.824326	0	0	1	0	0	0
SMG1	1.0104065	0.0149358	0.8882657	3602	2271	3352	1768	3458	4095
AL049875.1	1.8571429	0.8930848	0.4931644	0	3	4	5	1	7
SMG9	0.8215509	-0.283578	0.1282288	931	1005	1275	868	972	798
SMG7	1.0395257	0.0559254	0.436773	5048	5334	5810	4281	6240	6311
RNU1-59P	4	2	0.5760976	1	0	0	0	4	0
SMG8	0.7925072	-0.335504	0.0477753	728	814	887	520	704	701
SMG5	0.9197847	-0.120632	0.5401879	4525	5016	5506	4050	5015	4775
SMG6	0.7472296	-0.420377	0.0211336	586	647	662	351	538	527
CYCSP4	0.3333333	-1.584963	0.3730333	5	7	0	0	4	0
AC021752.1	0	#NUM!	0.5622741	0	1	2	0	0	0
AC084337.1	1.0454545	0.0641303	0.9440432	10	7	5	2	1	20
CYCSP6	1.421875	0.5077946	0.3844596	30	15	19	10	39	42
AC090844.2	0.6	-0.736966	0.622122	3	8	4	4	0	5
AC090114.3	0.6875	-0.540568	0.6329797	16	2	14	0	8	14
AC090114.1	6	2.5849625	0.3098729	0	0	1	1	1	4
AC090114.2	0.7916667	-0.337035	0.5965152	50	30	64	48	47	19
MYZAP	0.6106195	-0.711655	0.189487	32	32	49	16	38	15
AC005785.1	0.9393939	-0.090198	0.991928	14	2	17	10	10	11
AC005785.2	1	0	0.9120852	3	3	11	6	7	4
AC113361.1	#DIV/0!	#DIV/0!	0.2003758	0	0	0	0	5	2
FGD1	0.8071006	-0.30918	0.2438962	242	293	310	248	217	217
FGD2	0.5	-1	0.6695854	1	1	4	0	3	0
FGD3	1.211236	0.2764799	0.0659898	312	277	301	325	407	346
FGD4	1.0742227	0.1032931	0.5084819	348	254	395	218	401	452
FGD5	0.6666667	-0.584963	0.5420196	9	8	19	8	10	6
FGD6	1.1059229	0.1452508	0.3543334	1273	723	1195	786	1223	1520
ASPDH	0.5	-1	0.8638172	0	0	2	1	0	0
PCGF1	1.152458	0.2047142	0.0933326	463	555	589	607	667	578
AL512422.2	0	#NUM!	0.824326	0	1	0	0	0	0
PCGF6	1.1868381	0.2471232	0.0681732	447	382	493	410	617	542
RSPH4A	3	1.5849625	0.3701849	0	5	0	1	10	4
PCGF5	1.53149	0.614936	0.0003279	523	307	472	588	752	654
PCGF2	1.0274964	0.0391333	0.4579077	854	893	1017	915	1006	919
PCGF3	1.0498052	0.0701217	0.387881	1094	1164	1336	1037	1227	1509
AP001596.1	0.5438596	-0.878694	0.2578938	19	9	29	10	10	11
RPL34P33	0.7777778	-0.36257	0.90471	3	1	5	5	2	0
FGF1	0.125	-3	0.2302046	2	1	5	0	1	0
FGF2	1.3333333	0.4150375	0.8475284	3	0	0	3	1	0
FGF5	0	#NUM!	0.6436235	2	0	0	0	0	0
FGF7	0	#NUM!	0.0191126	2	6	7	0	0	0
FGF8	4.5	2.169925	0.2776395	1	1	0	8	0	1
FGF9	0	#NUM!	0.824326	1	0	0	0	0	0
AC008771.1	0.8923077	-0.164387	0.8741848	21	27	17	29	17	12
MIR4477B	0.7142857	-0.485427	0.80068	1	4	2	2	1	2
MIR4477A	#DIV/0!	#DIV/0!	0.8030358	0	0	0	0	1	0
PYGO1	2.1428571	1.0995357	0.1080535	13	4	11	12	28	20
PYGO2	0.9309524	-0.103221	0.7549387	1688	1925	2267	1765	2011	1698
RPL34P31	#DIV/0!	#DIV/0!	0.8030358	0	0	0	1	0	0

UTAT33	0.2881356	-1.79518	0.0342438	36	8	15	2	10	5
RTN4R	0.8146341	-0.295776	0.498916	71	56	78	52	76	39
DDX11L5	0.4615385	-1.115477	0.4555167	3	6	4	3	1	2
DDX11L8	1	0	0.9890559	1	0	0	0	1	0
DDX11L9	0.0769231	-3.70044	0.0694409	7	5	1	1	0	0
FGGY	1.1098726	0.1503941	0.3251486	173	228	227	223	260	214
SMN2	0.923284	-0.115154	0.6571922	719	670	840	571	749	738
SMN1	1.1220107	0.1660865	0.2166156	649	680	720	632	1010	657
SLC4A10	0.6666667	-0.584963	0.8407741	1	1	1	0	0	2
SLC4A11	0.7943925	-0.332076	0.6191227	12	42	53	19	44	22
AC010973.2	0	#NUM!	0.2043502	2	3	1	0	0	0
AC010973.1	0	#NUM!	0.0401975	8	0	6	0	0	0
DDX11L1	1.5	0.5849625	0.8253999	2	0	0	2	1	0
DDX11L2	0.8390805	-0.253119	0.4997136	99	119	130	105	91	96
AL136528.2	0	#NUM!	0.6840298	0	0	2	0	0	0
AL512662.2	3	1.5849625	0.6552898	1	0	0	0	2	1
AL136528.1	21	4.3923174	0.0112108	1	0	0	8	9	4
UFSP2	1.2477771	0.3193603	0.0129998	504	483	700	667	806	632
DDX3P1	1.5	0.5849625	0.8637378	2	0	0	0	0	3
UFSP1	0.5890411	-0.76356	0.230092	24	30	19	10	25	8
HMGN1P2	2.3333333	1.2223924	0.6152708	0	3	0	0	3	4
AP003419.2	#DIV/0!	#DIV/0!	0.6099294	0	0	0	2	0	0
HMGN1P4	0.75	-0.415037	0.9600825	0	0	4	2	0	1
AC144652.1	1.0546218	0.0767258	0.6927205	85	81	72	82	97	72
HMGN1P8	0	#NUM!	0.5436524	2	0	1	0	0	0
SVILP1	0.2352941	-2.087463	0.1625022	3	9	5	1	3	0
CTDSPL	0.8687185	-0.203039	0.177214	3074	3156	3680	2490	3282	2837
RNU6-1231P	0	#NUM!	0.824326	0	1	0	0	0	0
CTDSP1	1.0565834	0.0794067	0.3521294	1497	2019	2051	1992	2129	1761
CTDSP2	0.8932502	-0.162864	0.3517195	5211	6182	6415	4198	5897	5812
AC009120.1	0.2631579	-1.925999	0.1997536	1	14	4	2	0	3
AC009120.2	1.1505376	0.2023082	0.5850109	35	26	32	26	40	41
AC009120.3	0.2777778	-1.847997	0.0923274	15	7	14	0	2	8
AC009120.5	1	0	0.9766497	5	1	0	1	4	1
RPL34P18	0.1818182	-2.459432	0.2629081	3	0	8	1	1	0
RPL34P19	0	#NUM!	0.5075476	0	0	4	0	0	0
SMPX	0	#NUM!	0.6631647	1	0	1	0	0	0
PLAG1	1.2129145	0.2784778	0.2000709	224	132	217	150	278	267
FGL2	#DIV/0!	#DIV/0!	0.8030358	0	0	0	0	1	0
AC134915.1	#DIV/0!	#DIV/0!	0.8030358	0	0	0	0	0	1
AC011466.4	0	#NUM!	0.1312466	4	0	5	0	0	0
NLGN4Y	#DIV/0!	#DIV/0!	0.6554062	0	0	0	0	2	0
NLGN4X	0.9009009	-0.15056	0.7171998	80	80	62	29	73	98
AC011466.1	#DIV/0!	#DIV/0!	0.1930205	0	0	0	0	3	4
SMOX	1.2708333	0.3457748	0.0292868	302	420	430	527	485	452
AC011466.2	#DIV/0!	#DIV/0!	0.6482162	0	0	0	0	1	1
AC011466.3	0.5263158	-0.925999	0.4521021	7	5	7	0	7	3
AC010733.1	0.6923077	-0.530515	0.7389948	8	0	5	0	4	5
AC023469.1	#DIV/0!	#DIV/0!	0.8030358	0	0	0	0	0	1
RN7SL749P	0.5	-1	0.81106	0	2	0	0	0	1
AL512329.1	0.2962963	-1.754888	0.1431521	4	10	13	0	5	3
AL136372.1	#DIV/0!	#DIV/0!	0.5070017	0	0	0	3	0	0
RNU6-1223P	0	#NUM!	0.824326	0	0	1	0	0	0
RN7SL748P	#DIV/0!	#DIV/0!	0.4737294	0	0	0	0	4	0

AL731569.1	1.3285714	0.4098758	0.357859	26	21	23	30	40	23
SLC51A	1.106383	0.1458509	0.7520621	21	8	18	20	24	8
AC006960.3	#DIV/0!	#DIV/0!	0.4737294	0	0	0	0	4	0
AC006960.1	0	#NUM!	0.4374838	0	4	0	0	0	0
DDX10P1	0.5	-1	0.701864	1	3	0	0	0	2
DDX10P2	2	1	0.8186965	0	0	1	0	0	2
GINM1	0.9516889	-0.071438	0.907527	796	756	994	690	973	760
AC044787.1	1.6666667	0.7369656	0.5792018	5	4	0	5	3	7
RPAP1	0.9334983	-0.099281	0.7099992	987	1147	1099	890	1074	1054
RPAP2	1.0800244	0.111064	0.3005927	584	398	655	521	628	619
RPAP3	1.2029148	0.2665345	0.0953631	1052	526	1098	926	1227	1066
SMU1	1.1619308	0.2165242	0.0260158	2633	2686	3092	2641	3819	3313
SMTN	0.8806647	-0.183335	0.4093392	980	1121	1209	1001	998	916
AC091181.1	0.2307692	-2.115477	0.3139531	9	0	4	3	0	0
DLX2-AS1	0.8571429	-0.222392	0.9892847	3	0	11	8	0	4
RPAIN	0.9435435	-0.083839	0.8562066	523	507	635	465	576	530
RN7SL745P	1.5	0.5849625	0.8233276	0	2	0	3	0	0
PDCD1LG2	1.6666667	0.7369656	0.576721	3	3	3	1	1	13
ACTRT3	#DIV/0!	#DIV/0!	0.8030358	0	0	0	1	0	0
GINS1	0.803523	-0.315589	0.03246	2218	2473	2689	1579	2230	2121
GINS2	0.9330206	-0.100019	0.7822125	1151	1351	1335	1234	1283	1063
GINS3	0.7844732	-0.350204	0.0427175	830	911	964	687	789	646
GINS4	0.8072351	-0.308939	0.0895455	576	627	732	475	533	554
GRHL3	1.1435339	0.1934991	0.0659968	645	676	790	757	882	775
GRHL1	1.1157688	0.1580381	0.1238641	9485	7678	10271	7671	11282	11657
GRHL2	0.8271735	-0.273738	0.1037434	992	877	972	665	890	795
AL021578.1	1.0625	0.0874628	0.8922559	9	18	5	11	5	18
ARGFXP1	0	#NUM!	0.3608108	0	1	4	0	0	0
ZWILCH	1.0275572	0.0392187	0.6410902	821	522	798	555	911	734
ARGFXP2	0.875	-0.192645	0.9749866	3	3	2	6	0	1
DEUP1	0	#NUM!	0.824326	0	1	0	0	0	0
AZI2	1.0279823	0.0398155	0.4829204	697	534	806	674	731	689
RPL23AP42	3.5	1.8073549	0.3650613	0	1	1	3	3	1
RPL23AP43	0.5714286	-0.807355	0.6880501	0	4	3	0	3	1
RPL23AP24	0.9473684	-0.078003	0.9564171	5	4	10	0	13	5
RPL23AP25	6	2.5849625	0.3849176	0	1	0	0	0	6
RPL23AP29	0	#NUM!	0.6436235	2	0	0	0	0	0
DNAJC19P5	2.4642857	1.3011695	0.0792506	9	15	4	11	37	21
DNAJC19P6	0	#NUM!	0.824326	0	1	0	0	0	0
RPL23AP30	1	0	0.9922023	2	0	0	0	0	2
ADCY10P1	1.0854701	0.11832	0.6853962	44	26	47	39	59	29
RPL23AP15	0	#NUM!	0.824326	1	0	0	0	0	0
AC128685.1	#DIV/0!	#DIV/0!	0.5070017	0	0	0	3	0	0
RPL23AP18	0	#NUM!	0.6631647	1	0	1	0	0	0
AC135983.2	0.99	-0.0145	0.891258	128	94	178	89	184	123
RNU6-834P	4	2	0.5412414	0	0	1	0	3	1
TCTEX1D2	0.6875	-0.540568	0.5363429	11	11	10	5	11	6
TCTEX1D4	1.5	0.5849625	0.705036	1	4	1	2	3	4
AL391001.1	0.6585366	-0.602665	0.5449188	20	6	15	15	6	6
AC107871.2	1	0	0.9984637	0	3	1	0	0	4
AC135983.3	#DIV/0!	#DIV/0!	0.5509701	0	0	0	0	3	0
RPL23AP22	0	#NUM!	0.6436235	2	0	0	0	0	0
AC135983.4	#DIV/0!	#DIV/0!	0.8030358	0	0	0	1	0	0
OGFRP1	0.7076923	-0.498806	0.4408043	23	21	21	12	14	20

AC135983.5	#DIV/0!	#DIV/0!	0.8030358	0	0	0	1	0	0
RPL23AP20	0.4	-1.321928	0.584623	3	1	1	1	0	1
GRHPR	1.0914798	0.1262855	0.1653882	2639	2955	3326	3371	3547	2818
AC013470.3	5	2.3219281	0.2453784	0	2	0	2	4	4
AC013470.1	#DIV/0!	#DIV/0!	0.8030358	0	0	0	0	0	1
AC013470.2	1.5	0.5849625	0.8141514	4	0	0	0	1	5
RPL23AP12	0.6	-0.736966	0.7824522	0	3	2	2	1	0
RPL23AP10	#DIV/0!	#DIV/0!	0.1958503	0	0	0	0	4	3
RABEPK	1.0404135	0.0571571	0.3916757	939	1071	1182	1117	1161	1043
OASL	5.5	2.4594316	1.21E-05	6	9	9	60	29	43
LRRTM3	0	#NUM!	0.5781912	0	0	3	0	0	0
LRRTM4	0.2941176	-1.765535	0.0035388	26	35	41	6	9	15
LRRTM2	0.7142857	-0.485427	0.8779389	1	0	6	0	0	5
AL136038.3	0.7096774	-0.494765	0.6617853	8	6	17	0	8	14
AL136038.4	1.1702128	0.2267709	0.5612756	32	21	41	20	45	45
AC107871.1	0.3724138	-1.425022	0.0063447	60	35	50	7	28	19
AL136038.5	1.2820513	0.358454	0.5000277	7	10	22	19	19	12
PLAUR	1.0225225	0.0321326	0.7132083	260	316	312	271	397	240
AP000525.1	1.1410256	0.1903312	0.6176021	28	21	29	26	38	25
SUPV3L1	1.0895326	0.1237093	0.1643967	918	965	1155	961	1264	1085
RABEP1	1.017107	0.0244714	0.5768641	1681	1113	1824	1334	1679	1684
OAS1	10.284211	3.3623591	1.49E-65	59	94	132	880	1122	929
RABEP2	1.0681564	0.0951229	0.4469737	323	286	286	352	287	317
AL031717.1	0.1666667	-2.584963	0.1292489	1	9	8	0	0	3
OAS2	16.571429	4.0506261	3.52E-75	28	49	42	578	761	633
OAS3	4.1400943	2.0496636	3.76E-37	631	809	680	2185	3190	3402
RAB30-AS1	0.7365854	-0.441075	0.276168	64	64	77	45	70	36
AL137845.2	0	#NUM!	0.824326	0	1	0	0	0	0
PKIA-AS1	3	1.5849625	0.4767741	1	0	2	0	9	0
DNAJC19P9	2.4	1.2630344	0.3898417	2	2	1	8	0	4
OGFRL1	1.008089	0.011623	0.6199785	666	562	750	598	717	679
AC116407.1	0.6666667	-0.584963	0.8418928	1	3	2	4	0	0
AC116407.2	2.2580645	1.1750867	0.061103	9	12	10	17	35	18
SNCG	1.5	0.5849625	0.0781989	57	48	47	85	91	52
SNCB	#DIV/0!	#DIV/0!	0.1224888	0	0	0	7	0	1
LURAP1	1.34	0.422233	0.4220088	14	18	18	17	30	20
AC116407.3	0.9	-0.152003	0.9499418	12	11	17	18	9	9
AC116407.4	0.5625	-0.830075	0.5591556	8	1	7	3	5	1
SNCA	1.1007752	0.1385199	0.5657567	57	107	94	92	122	70
AP006587.5	0	#NUM!	0.6436235	2	0	0	0	0	0
SLC4A7	1.213555	0.2792395	0.1563661	622	375	567	387	681	830
SLC4A8	0.490566	-1.027481	0.1194339	25	25	56	6	13	33
SLC4A9	1.4	0.4854268	0.6816479	7	2	1	6	2	6
SND1	0.8713737	-0.198637	0.2183575	15511	18704	19662	14221	16436	16290
HINT1P1	0	#NUM!	0.4117982	0	3	1	0	0	0
AL135786.1	0	#NUM!	0.5337954	0	3	0	0	0	0
SLC5A6	0.9062805	-0.14197	0.4448821	10143	12281	12414	9485	11714	10374
SLC5A7	0	#NUM!	0.0300083	2	1	12	0	0	0
SLC5A9	0	#NUM!	0.6840298	0	0	2	0	0	0
TBPL2	0.6666667	-0.584963	0.8082319	4	0	2	0	0	4
TBPL1	0.9818822	-0.026378	0.8173962	617	608	762	581	716	654
SLC5A2	0	#NUM!	0.3626892	1	0	4	0	0	0
OAZ1	0.9389143	-0.090935	0.8949294	4039	5278	5531	5152	4920	3869
SLC5A3	0.9141221	-0.129541	0.6527112	626	398	548	283	583	571

OAZ2	0.8230088	-0.28102	0.1123326	1060	1126	1317	956	952	975
SLC5A4	1	0	0.9774058	0	2	0	0	1	1
OAZ3	0.9272727	-0.108934	0.9517168	16	11	28	13	25	13
SLC5A5	0.991274	-0.012644	0.7999898	519	591	609	507	628	569
AC083841.1	0.9130435	-0.131245	0.9418288	6	9	8	6	3	12
RNU6-850P	0.75	-0.415037	0.7851667	3	2	11	1	9	2
TUBB4BP2	1.5	0.5849625	0.8265197	0	1	1	0	1	2
AC008040.2	0.5	-1	0.8188512	2	0	0	0	1	0
AC008040.1	0	#NUM!	0.824326	0	1	0	0	0	0
AC019254.1	0.3	-1.736966	0.5535888	0	0	10	3	0	0
AC019254.2	#DIV/0!	#DIV/0!	0.5070017	0	0	0	3	0	0
AZU1	3	1.5849625	0.451274	0	0	2	5	0	1
SNF8	0.8663564	-0.206967	0.4661629	441	483	580	484	460	359
RPL23AP92	0.3333333	-1.584963	0.6792283	1	0	2	1	0	0
RPL23AP90	#DIV/0!	#DIV/0!	0.5230806	0	0	0	1	2	0
RPL23AP95	8	3	0.0703239	1	0	1	7	1	8
AC106794.1	0	#NUM!	0.2245369	2	1	3	0	0	0
RPL23AP93	0	#NUM!	0.5337954	0	3	0	0	0	0
RPL23AP97	#DIV/0!	#DIV/0!	0.6554062	0	0	0	0	2	0
AC027702.1	0.6909091	-0.533432	0.5547592	13	10	32	16	14	8
AC008280.3	2	1	0.4667928	0	0	6	5	4	3
AC008280.1	1	0	0.9890559	0	0	1	0	0	1
AL731661.1	0.625	-0.678072	0.7574099	3	2	3	4	1	0
AC244197.3	4.5	2.169925	0.2899185	0	2	0	2	3	4
RPL23AP81	0	#NUM!	0.6385096	0	2	0	0	0	0
RPL23AP85	#DIV/0!	#DIV/0!	0.242062	0	0	0	6	0	0
RPL23AP82	1.0013333	0.0019223	0.6808696	452	434	614	441	577	484
RPL23AP83	0.5	-1	0.81106	0	2	0	0	0	1
FGFBP3	0.8	-0.321928	0.5582599	27	37	41	24	33	27
RPL23AP88	#DIV/0!	#DIV/0!	0.3877895	0	0	0	2	2	0
RPL23AP86	1.5	0.5849625	0.8194094	0	0	2	0	0	3
RPL23AP87	0.73	-0.454032	0.4802384	28	35	37	31	33	9
ELOVL1	0.9585217	-0.061117	0.9985113	2979	3484	3711	3248	3452	3052
FGFBP1	1	0	0.8981116	0	0	4	4	0	0
RPL23AP69	#DIV/0!	#DIV/0!	0.8030358	0	0	0	1	0	0
ELOVL4	#DIV/0!	#DIV/0!	0.6554062	0	0	0	0	2	0
ELOVL5	0.856087	-0.224171	0.1632317	3072	2780	3348	2003	2958	2915
ELOVL2	0.8800461	-0.184349	0.3608556	575	570	589	411	592	523
ELOVL3	0.9264706	-0.110183	0.8654049	49	56	31	46	41	39
ELOVL6	1.3321043	0.4137071	0.0008429	2649	1897	2777	2630	3575	3550
ELOVL7	0.6747881	-0.567493	0.0075577	337	266	341	169	236	232
POU5F1	0.8333333	-0.263034	0.9497566	0	0	6	0	4	1
POU5F2	0.5	-1	0.6294942	1	4	3	3	1	0
AC006529.1	0	#NUM!	0.6840298	0	0	2	0	0	0
TFAP2A-AS1	1.0714286	0.0995357	0.8108304	6	10	12	15	10	5
AC099850.1	0.8	-0.321928	0.7285087	11	10	9	6	8	10
WSPAR	0	#NUM!	0.824326	1	0	0	0	0	0
AC099850.3	1.0862573	0.1193659	0.3693654	219	230	235	246	251	246
RPL23AP66	0	#NUM!	0.6840298	0	0	2	0	0	0
RPL23AP67	0	#NUM!	0.5200113	2	1	0	0	0	0
RPL23AP64	0.7777778	-0.36257	0.5954341	24	22	17	2	22	25
RPL23AP65	0.625	-0.678072	0.7328828	2	4	2	3	0	2
LINC00266-1	1	0	0.9838634	1	0	1	0	0	2
AC118553.1	0.1428571	-2.807355	0.2753887	2	5	0	0	1	0

AC118553.2	1.25	0.3219281	0.8441079	0	0	4	0	1	4
AL035530.2	0	#NUM!	0.284725	6	0	0	0	0	0
RPL23AP53	1.3658537	0.4498029	0.5844104	19	5	17	1	24	31
CSE1L	1.0731284	0.1018227	0.2915035	10708	7102	11016	8193	11818	10923
STK19	0.9871032	-0.018727	0.7656529	330	287	391	356	310	329
RNU1-27P	0	#NUM!	0.6840298	0	0	2	0	0	0
AC005392.2	0	#NUM!	0.6840298	0	0	2	0	0	0
STK10	0.9093491	-0.137094	0.4691172	3953	4479	4949	3606	4315	4247
AC245036.4	#DIV/0!	#DIV/0!	0.8030358	0	0	0	0	1	0
STK11	0.9054239	-0.143335	0.5911773	1452	1674	1981	1578	1563	1483
STK16	1.0420831	0.0594703	0.4592819	581	652	668	690	704	587
AL390066.1	1.3333333	0.4150375	0.8526162	2	4	0	0	3	5
AC003005.2	0	#NUM!	0.824326	0	0	1	0	0	0
STK39	0.4990826	-1.00265	2.18E-06	368	303	419	135	192	217
FHIT	1.18429	0.2440224	0.2420035	107	106	118	134	150	108
STK31	0.7425	-0.429537	0.2006613	160	84	156	90	111	96
STK33	0.4824561	-1.05153	0.0574818	46	25	43	22	15	18
STK35	0.8222812	-0.282296	0.0984879	1499	1736	1666	1053	1426	1551
STK36	0.9365079	-0.094637	0.8063108	403	360	434	334	397	390
STK38	1.027929	0.0397407	0.4132108	1909	1690	2094	1747	2111	1994
AC018665.1	0.6585366	-0.602665	0.3820697	33	20	29	27	18	9
AC008770.1	1.2275862	0.2958243	0.2117247	82	85	123	92	159	105
AC006133.1	0	#NUM!	0.824326	0	0	1	0	0	0
STK24	1.0115906	0.0166255	0.61011	2915	3327	3421	2699	3592	3484
STK25	1.1875908	0.2480379	0.0199246	2534	3121	3290	3570	3635	3418
STK26	1.0789452	0.1096217	0.2963758	4402	3318	4681	3203	5139	5038
FHL1	1.9770642	0.9833597	9.32E-05	59	76	83	125	160	146
FHL2	1.0444742	0.0627769	0.2888833	4138	4678	5372	4817	5233	4769
FHL3	1.2250454	0.2928352	0.052897	320	329	453	501	479	370
MIR3150BHG	0	#NUM!	0.2756435	2	2	1	0	0	0
CAPZB	1.0926266	0.1278004	0.1387692	4684	5739	6462	6234	6393	5822
AC008676.1	2.7142857	1.4405726	0.3355491	4	0	3	0	3	16
AC008676.2	0	#NUM!	0.4521054	1	0	3	0	0	0
MIR6835	0.5	-1	0.3193477	11	4	19	3	9	5
SNPH	0.75	-0.415037	0.84505	0	4	0	0	3	0
AL049872.1	6	2.5849625	0.3283186	0	0	1	3	3	0
AL133243.1	0	#NUM!	0.6604275	0	1	1	0	0	0
AL133243.2	0.7666667	-0.383329	0.5785583	29	15	16	13	24	9
SNRK	0.9025471	-0.147926	0.5872422	671	468	667	354	610	666
MIR6812	#DIV/0!	#DIV/0!	0.8030358	0	0	0	0	0	1
AL133243.3	0.5526316	-0.85561	0.3374128	21	9	8	9	6	6
AL133243.4	0.3333333	-1.584963	0.6622061	0	3	0	0	0	1
AL162742.1	3.625	1.857981	0.1126082	7	1	0	16	5	8
OGFOD3	0.8229273	-0.281163	0.174593	801	1151	979	788	847	777
RPS15AP1	0.5384615	-0.893085	0.5367212	7	2	4	0	5	2
RPS15AP6	0	#NUM!	0.824326	1	0	0	0	0	0
MIR6767	0	#NUM!	0.1560297	3	2	2	0	0	0
OGFOD1	0.8731301	-0.195731	0.2572206	1730	1522	2029	1252	1715	1644
MIR6768	0.75	-0.415037	0.8207133	4	4	0	2	0	4
OGFOD2	1.1190476	0.1622714	0.7058117	14	7	21	15	11	21
AL118508.1	2	1	0.7855869	0	1	0	1	1	0
AL118508.2	#DIV/0!	#DIV/0!	0.055816	0	0	0	5	1	4
FNDC9	1.8	0.8479969	0.6502907	2	0	3	0	0	9
AL109936.8	1.7	0.7655347	0.5418909	1	5	4	1	3	13

FNDC8	#DIV/0!	#DIV/0!	0.6099294	0	0	0	2	0	0
FNDC5	2.125	1.0874628	0.2369241	3	11	2	16	7	11
AC091180.2	0.3333333	-1.584963	0.669913	3	0	0	0	0	1
FNDC4	0.25	-2	0.3946239	6	0	2	0	0	2
FNDC1	5	2.3219281	0.4886016	0	0	1	0	5	0
PCBP1-AS1	1.0233196	0.0332568	0.6024663	231	202	296	233	269	244
TAGLN2	0.8758157	-0.191301	0.3116767	8390	9988	11046	8623	8949	8198
MIR6753	0.6	-0.736966	0.7612956	0	4	1	0	0	3
TAGLN3	#DIV/0!	#DIV/0!	0.8030358	0	0	0	1	0	0
AC091180.4	#DIV/0!	#DIV/0!	0.3096126	0	0	0	5	0	0
BVES-AS1	2.3333333	1.2223924	0.6086527	3	0	0	0	1	6
CAPSL	#DIV/0!	#DIV/0!	0.6482162	0	0	0	0	1	1
MIR4422HG	0.5	-1	0.812391	0	2	0	0	1	0
SNX3	1.0538454	0.0756633	0.2127601	6409	6834	8003	6665	8277	7448
MRE11P1	0	#NUM!	0.824326	0	1	0	0	0	0
SNX4	0.9985853	-0.002042	0.6874791	1729	1319	1900	1381	1809	1751
SNX1	0.8991487	-0.153368	0.4263798	2379	2444	2812	2201	2372	2292
SNX2	1.2990208	0.3774245	0.0024379	9443	6166	9921	9857	12598	10709
MIR6746	2	1	0.8186965	0	0	1	0	0	2
SNX9	1.0172108	0.0246187	0.5061165	1712	1695	2229	1574	2158	2001
SNX7	#DIV/0!	#DIV/0!	0.1261023	0	0	0	0	7	2
MIR6740	0.375	-1.415037	0.3306155	3	5	8	3	1	2
SNX8	0.9317254	-0.102023	0.7936685	2162	2842	2832	2528	2455	2318
SNX5	1.0215956	0.0308243	0.4343324	9504	7625	10747	8513	10539	9426
SNX6	1.0215065	0.0306984	0.545607	6651	4851	7097	5020	7287	6692
AC073655.2	0.5555556	-0.847997	0.7418998	0	0	9	1	1	3
RPE65	0	#NUM!	0.824326	0	1	0	0	0	0
MIR6739	1.025	0.0356239	0.939247	13	12	15	2	3	36
SNW1	0.9966155	-0.004891	0.6134439	2721	2356	3196	2569	2886	2790
USP24P1	0	#NUM!	0.824326	1	0	0	0	0	0
CAB39P1	0.9444444	-0.082462	0.9534169	13	10	13	8	15	11
AC008770.4	1.2857143	0.3625701	0.7634297	11	1	2	3	7	8
RN7SL756P	#DIV/0!	#DIV/0!	0.6411933	0	0	0	0	0	2
ARHGAP23P1	0.5	-1	0.0564849	35	35	38	16	24	14
AL929236.1	1.5714286	0.6520767	0.6499399	2	3	2	1	5	5
CSDE1	0.9685438	-0.046111	0.9500223	17810	13097	18813	13306	17745	17105
RNU6-847P	#DIV/0!	#DIV/0!	0.8030358	0	0	0	1	0	0
ACTN1-AS1	0.6428571	-0.63743	0.5207249	7	15	6	4	3	11
MIR6797	0	#NUM!	0.0653515	2	6	2	0	0	0
STK40	1.1263774	0.1716903	0.1138865	854	1018	1032	998	1244	1029
CAPS2	0.6666667	-0.584963	0.7518705	2	4	9	7	2	1
AL365181.2	0.5384615	-0.893085	0.1455483	23	22	33	9	19	14
AL365181.3	0.7709677	-0.375258	0.2955654	70	112	128	62	87	90
SERTAD3	0.9969318	-0.004433	0.6897754	1150	1462	1625	1428	1561	1235
RNU6-1237P	#DIV/0!	#DIV/0!	0.5371397	0	0	0	0	0	3
SERTAD4	0.820712	-0.285052	0.1661006	484	456	605	313	468	487
SERTAD1	0.7863248	-0.346803	0.136389	407	446	551	405	381	318
SERTAD2	1.0173688	0.0248428	0.6407678	1816	1624	1972	1329	2117	2060
MIR6784	4	2	0.5580302	0	0	1	0	0	4
RNU6-1238P	0.3333333	-1.584963	0.6659102	3	0	0	1	0	0
CSDC2	1	0	0.9699945	2	0	0	2	0	0
MIR6779	0	#NUM!	0.5075476	0	0	4	0	0	0
MIR6775	1.2857143	0.3625701	0.8450348	1	2	4	0	9	0
MIR6772	0	#NUM!	0.2732884	0	2	4	0	0	0

AC092164.1	1.9705882	0.9786263	0.1025493	15	6	13	20	26	21
GLVIVOR22-	0.6666667	-0.584963	0.856923	1	0	2	0	2	0
CYP46A1	0.5	-1	0.620371	1	5	0	0	2	1
CAPN8	3.5	1.8073549	0.5234342	2	0	0	0	7	0
CAPN9	0.2	-2.321928	0.1754012	4	11	0	0	1	2
CAPN6	1.2102181	0.2752671	0.0036773	11962	12936	14209	13107	17006	17215
CAPN7	0.9781111	-0.03193	0.9520769	913	642	912	576	974	863
DUOXA1	0.75	-0.415037	0.7403529	0	13	7	2	6	7
CAPN5	0.8078995	-0.307752	0.2551004	185	185	187	98	178	174
DUOXA2	#DIV/0!	#DIV/0!	0.8030358	0	0	0	1	0	0
CAPN2	0.6502719	-0.620885	4.61E-07	4195	3694	4798	2352	3062	2836
CAPN3	0	#NUM!	0.5781912	0	0	3	0	0	0
CAPN1	0.8617846	-0.214601	0.2506512	8081	9959	10213	8284	8475	7589
COQ10BP2	0.5	-1	0.8276497	1	0	1	0	0	1
DHRSX-IT1	1.125	0.169925	0.9588248	3	5	0	1	7	1
BBIP1P1	#DIV/0!	#DIV/0!	0.2116658	0	0	0	1	4	1
AL157700.1	1.2857143	0.3625701	0.7376556	0	0	7	4	1	4
AC078846.1	0.8993056	-0.153117	0.7154662	82	101	105	53	90	116
NDUFB9P1	0	#NUM!	0.6410557	1	1	0	0	0	0
RN7SL751P	0.8	-0.321928	0.8485651	10	3	7	10	5	1
AC093388.1	0.6	-0.736966	0.6710488	7	3	0	1	0	5
RN7SL750P	0.5	-1	0.7417349	1	0	3	0	2	0
AL139022.2	0.4	-1.321928	0.7347189	0	0	5	2	0	0
AL139022.1	0.8571429	-0.222392	0.9463495	9	1	4	8	0	4
SOBP	0.6208531	-0.687676	0.1399387	60	64	87	19	36	76
DNPH1	0.9689629	-0.045487	0.9278294	737	978	927	884	865	811
ZNF252P	1	0	0.9016831	254	185	235	137	288	249
AL360093.1	0.8	-0.321928	0.8419358	5	1	9	0	12	0
AP004245.1	0.7609756	-0.394078	0.3021504	67	64	74	43	62	51
H2BFWT	2	1	0.5908788	0	1	2	2	3	1
AC091826.2	#DIV/0!	#DIV/0!	0.2887809	0	0	0	0	0	6
AL049539.1	1.3333333	0.4150375	0.6530957	0	2	10	7	6	3
DNPEP	1.0920916	0.1270938	0.1656432	1699	1977	2090	2147	2200	1950
SOD2	0.8509665	-0.232826	0.1421272	1546	1457	1808	1119	1509	1466
SOD3	0.9369025	-0.094029	0.8711002	296	363	387	330	317	333
SOD1	1.1116799	0.1527415	0.0853631	3153	3410	3985	3944	4239	3543
EEPD1	2.7154472	1.4411898	1.10E-05	24	44	55	123	101	110
AL591479.1	0.8	-0.321928	0.8851909	0	5	0	2	2	0
AL008729.1	0.3823529	-1.387023	0.1581439	16	5	13	4	5	4
RNU1-31P	0.25	-2	0.6199202	0	0	4	0	1	0
PLCE1	1.8765957	0.9081179	0.0007426	217	109	144	182	353	347
JUNB	1.5204678	0.6045153	0.0007339	264	241	350	510	395	395
AC080129.2	0.6666667	-0.584963	0.7518732	4	1	4	2	1	3
AC080129.1	0.5	-1	0.7214899	0	4	0	0	0	2
NHLRC3	1.0380711	0.0539052	0.4114173	1481	1184	1669	1242	1687	1570
NHLRC4	1	0	0.9480217	0	0	2	1	1	0
AL049779.1	#DIV/0!	#DIV/0!	0.8030358	0	0	0	0	1	0
AL049779.3	0.4444444	-1.169925	0.545103	0	9	0	0	2	2
AL049779.4	0	#NUM!	0.6436235	2	0	0	0	0	0
AL031719.1	#DIV/0!	#DIV/0!	0.8030358	0	0	0	0	1	0
AC106795.2	1.516129	0.6003925	0.4490574	19	1	11	13	25	9
AC106795.3	0	#NUM!	0.824326	0	0	1	0	0	0
NHLRC1	0	#NUM!	0.012028	5	7	4	0	0	0
AC106795.1	1.0381074	0.0539557	0.3986313	1172	1262	1476	1407	1456	1196

NHLRC2	1.2025272	0.2660695	0.117085	1068	792	989	714	1295	1417
FICD	0.7472376	-0.420361	0.0799339	217	275	232	144	205	192
CRACR2A	0.3536585	-1.499571	0.0001183	79	93	74	23	40	24
CRACR2B	0.7937337	-0.333273	0.2783638	124	124	135	100	109	95
PPP4C	0.9594779	-0.059678	0.9511298	4082	4920	5632	4825	4978	4238
PLCG2	0.6826635	-0.550754	0.0021722	470	555	642	352	396	390
AL445363.1	0	#NUM!	0.5781912	0	0	3	0	0	0
AL445363.2	#DIV/0!	#DIV/0!	0.4501902	0	0	0	0	0	4
AL445363.3	1.5	0.5849625	0.8233996	1	0	1	0	1	2
PLCG1	0.8586371	-0.21988	0.1455289	3096	3185	3566	2615	2946	2894
NSUN5P1	0.8933951	-0.16263	0.6103647	534	484	708	544	519	479
JUND	1.1694756	0.2258617	0.0643195	2621	3263	3669	4241	3842	3089
NSUN5P2	0.7608696	-0.394279	0.7749055	10	8	28	19	4	12
MTHFD1P1	0.25	-2	0.5682537	0	1	3	0	1	0
FIBP	1.0027907	0.0040205	0.6101042	901	1042	1282	1182	1070	982
SLC6A6	0.5570833	-0.844035	3.33E-07	1459	1628	1713	638	955	1081
PPP5C	0.9354796	-0.096222	0.7472454	2178	2688	2651	2226	2550	2256
SLC6A7	0	#NUM!	0.824326	0	0	1	0	0	0
SLC6A8	1.006199	0.0089157	0.6152177	920	1032	1113	1008	1073	1003
SLC6A9	2.1825243	1.1259977	1.37E-09	143	154	218	296	431	397
GIPC1	0.9712721	-0.042053	0.8589381	2235	2905	3075	2894	2735	2350
GIPC2	0.25	-2	0.5645669	1	0	3	1	0	0
PLCH1	0.8188406	-0.288345	0.563788	61	30	47	22	45	46
AC090498.1	1.0442804	0.0625092	0.7092053	81	108	82	105	100	78
PLCH2	#DIV/0!	#DIV/0!	0.6251462	0	0	0	1	0	1
HRCT1	3	1.5849625	0.2316697	0	1	5	8	9	1
OR2W2P	#DIV/0!	#DIV/0!	0.6554062	0	0	0	0	2	0
SLC6A2	1	0	0.9760479	0	2	0	2	0	0
SLC6A3	5.9354839	2.5693656	7.83E-07	14	2	15	55	76	53
SLC6A4	2.25	1.169925	0.1685664	5	7	4	9	16	11
IMPA1	0.9877217	-0.017824	0.8022784	244	190	299	220	247	257
IMPA2	1.1455008	0.1959785	0.0423057	1707	1955	2228	2143	2497	2107
OPN1LW	3.3333333	1.7369656	0.3077596	2	0	1	3	2	5
AC027607.1	0.6666667	-0.584963	0.7586995	2	3	4	1	0	5
PLCB3	0.9134432	-0.130613	0.5850315	1655	1937	2173	1672	1819	1775
AKR7A2P1	0.6888889	-0.537657	0.6007634	8	12	25	8	3	20
PLCB4	0	#NUM!	0.5781912	0	0	3	0	0	0
OPN1MW	1.3076923	0.3870231	0.747794	0	9	4	3	3	11
AC092809.2	0	#NUM!	0.824326	1	0	0	0	0	0
AC092809.3	0	#NUM!	0.6604275	0	1	1	0	0	0
PLCB1	0.9725275	-0.040189	0.9542173	279	192	257	209	265	234
7420|brain dev	#VALUE!	#VALUE!	005634|nucl	0|hsa04911|h	ocannabino	olipase C beta 1			
PLCB2	0.9067797	-0.141176	0.6140831	359	358	463	313	413	344
FAM71F1	#DIV/0!	#DIV/0!	0.8030358	0	0	0	1	0	0
OCA2	0.8666667	-0.206451	0.956916	2	0	13	11	1	1
AC092809.4	0.8571429	-0.222392	0.9234346	1	4	2	3	2	1
FIGN	1.1982006	0.2608694	0.0586765	1341	1055	1383	1051	1746	1731
FAM71F2	0	#NUM!	0.2515625	5	0	1	0	0	0
CARD8	1.1887584	0.2494555	0.0661902	380	352	460	369	522	526
CARD9	0.6418919	-0.639598	0.1560706	40	58	50	27	30	38
CARD6	1.7333333	0.7935491	0.4242344	8	6	1	12	9	5
TTC9B	0.25	-2	0.5498031	4	0	0	1	0	0
TTC9C	1.1384157	0.1870274	0.0924768	688	800	860	911	908	854
POU4F1	1.0685358	0.0956353	0.5483427	101	90	130	98	133	112

FAM71E1	0.2631579	-1.925999	0.0548615	15	16	7	3	1	6
POU4F2	0	#NUM!	0.6385096	0	2	0	0	0	0
POU4F3	0.1818182	-2.459432	0.2844268	2	0	9	0	2	0
FIG4	0.9819444	-0.026287	0.8488219	494	396	550	399	505	510
PLCD3	0.8866638	-0.173541	0.4128947	1877	2522	2642	1975	2214	2054
PLCD4	0.7715134	-0.374237	0.2475344	102	121	114	54	119	87
AC092653.1	2.1666667	1.1154772	0.4237748	2	0	4	12	0	1
PLCD1	1.2574257	0.3304732	0.1487652	114	92	97	125	137	119
AC092653.2	0.8571429	-0.222392	0.9206739	0	3	4	0	3	3
CSF3R	1.1288272	0.1748246	0.0335605	3192	3225	3806	3650	4097	3793
SLC35E1P1	0.8095238	-0.304855	0.7679613	8	7	6	1	7	9
AL049873.2	#DIV/0!	#DIV/0!	0.2327526	0	0	0	0	0	7
YEATS4	1.0565435	0.0793522	0.2994677	702	660	831	734	815	768
AL049873.1	4.5	2.169925	0.1017854	1	2	1	8	4	6
HOXA-AS3	0.1111111	-3.169925	0.196938	8	1	0	0	0	1
AC093582.1	0.5217391	-0.938599	0.2432516	18	12	16	3	8	13
AC098679.2	1.4285714	0.5145732	0.5270986	4	5	12	4	7	19
ENKUR	0.4603175	-1.119299	0.109498	22	14	27	8	11	10
CR383656.13	0	#NUM!	0.824326	1	0	0	0	0	0
STIM1	0.8483572	-0.237256	0.1975154	738	774	862	639	731	644
STIM2	0.864374	-0.210272	0.2547905	871	762	955	677	812	748
CSF1R	0.6498698	-0.621777	4.40E-05	2822	3618	3542	2006	2121	2360
STIP1	0.9479267	-0.077153	0.8638457	14649	16970	18445	14898	17025	15534
AC110769.2	0.7083333	-0.4975	0.6169977	14	5	5	1	11	5
AL683807.1	1.2222222	0.2895066	0.5401735	21	34	17	27	31	30
AL683807.2	2	1	0.8186965	0	0	1	0	0	2
IMPG1	1.6666667	0.7369656	0.6797397	4	0	2	0	6	4
IMPG2	0.5	-1	0.4976704	10	1	3	3	3	1
AC011468.3	#DIV/0!	#DIV/0!	0.3981469	0	0	0	4	0	0
AC011468.5	0.1666667	-2.584963	0.3263023	3	1	2	0	1	0
DAMTSL4-AS	0.5	-1	0.4531617	3	10	13	7	0	6
YEATS2	0.88482	-0.176544	0.3362045	2155	1932	2303	1395	2101	2158
MIR199A1	0	#NUM!	0.6436235	2	0	0	0	0	0
AC073415.1	1.7222222	0.7842713	0.4114404	6	1	11	2	9	20
AC004812.2	0.8205128	-0.285402	0.4908984	106	59	108	71	89	64
PPP6C	1.1885174	0.249163	0.0064366	2926	2854	3312	3105	4070	3631
SOST	0	#NUM!	0.2290435	7	0	0	0	0	0
SORD	1.0211063	0.030133	0.563457	420	443	511	432	495	476
AC009264.1	1.0882353	0.1219905	0.8230429	18	29	21	13	41	20
AC092903.2	3	1.5849625	0.3772308	2	0	1	6	0	3
AC022639.1	2.3636364	1.2410081	0.2311311	3	5	3	4	13	9
SOS1	1.0657929	0.0919272	0.5363861	1465	915	1435	793	1577	1696
SP3P	#DIV/0!	#DIV/0!	0.3597364	0	0	0	0	0	5
SOS2	1.1482558	0.1994441	0.2811742	590	253	533	407	609	564
AC005391.1	0.95	-0.074001	0.9743981	5	3	12	5	5	9
SKOR1	1.2	0.2630344	0.8611489	0	1	4	0	2	4
PLCL1	2	1	0.6005889	0	0	3	1	2	3
PLCL2	0.7918216	-0.336753	0.1157984	513	359	473	275	422	368
AL390067.1	0.1666667	-2.584963	0.1644024	9	2	1	0	1	1
RNF138P1	#DIV/0!	#DIV/0!	0.4098001	0	0	0	1	3	0
AC022483.1	#DIV/0!	#DIV/0!	0.5415473	0	0	0	0	2	1
AC110373.1	0.5806452	-0.784271	0.4395481	6	9	16	6	5	7
TTLL10	#DIV/0!	#DIV/0!	0.2378905	0	0	0	2	4	0
CHCHD3P3	5	2.3219281	0.3910149	0	0	1	3	0	2

DDX60L	1.952381	0.9652346	1.59E-08	526	318	542	721	1038	947
SOX2	0.7368421	-0.440573	0.6897625	14	5	0	1	9	4
CA3-AS1	1.5555556	0.6374299	0.622526	3	5	1	3	5	6
SOX8	5.75	2.523562	0.0500107	0	1	3	14	5	4
SOX6	5.75	2.523562	0.0925818	1	2	1	0	15	8
SOX7	2.4545455	1.2954559	0.2078571	6	0	5	13	9	5
SOX4	0.9806779	-0.028149	0.8883644	4842	5046	5483	4023	5602	5449
SOX5	0.5555556	-0.847997	0.6318132	7	0	2	1	3	1
JMJD4	0.8150685	-0.295007	0.6367915	37	51	58	54	30	35
JMJD7	0.4285714	-1.222392	0.4016606	3	2	16	3	1	5
JMJD8	0.9476082	-0.077637	0.8774032	257	306	315	231	298	303
JMJD6	1.0568439	0.0797623	0.3033595	1014	1196	1326	1226	1335	1176
TTLL11	0.8055556	-0.311944	0.6712356	32	25	15	28	18	12
TTLL12	0.9509161	-0.07261	0.9030906	2040	2585	2689	2154	2449	2352
AL162741.1	0.6875	-0.540568	0.7002094	3	6	7	3	8	0
FIS1	0.9297297	-0.105117	0.8415085	1529	1884	2137	1902	1814	1444
ZWINT	1.2180405	0.2845621	0.0028991	2917	3283	3578	3563	4310	4037
OCLM	1.4705882	0.5563933	0.5411198	7	2	8	5	14	6
OCLN	1.3682635	0.4523461	0.0041346	485	376	475	452	689	687
MIR6856	0	#NUM!	0.4374838	0	4	0	0	0	0
CREBRF	1.2924962	0.37016	0.0757572	573	261	472	405	614	669
CC2D1A	0.8550028	-0.225999	0.1729222	1085	1156	1297	888	1140	997
CC2D1B	0.8641488	-0.210648	0.2560806	1035	1185	1328	965	1131	970
BX004987.1	0.6756757	-0.565597	0.2117407	44	49	55	30	45	25
CCHCR1	1.1269841	0.1724672	0.1072067	608	706	765	748	811	784
CC2D2A	0.9332443	-0.099673	0.8223427	266	189	294	174	266	259
CC2D2B	0.8076923	-0.308122	0.7934314	4	12	10	8	11	2
TOMM70	0.9605883	-0.05801	0.9446135	5357	4423	5723	3958	5442	5492
AC103982.1	0.7142857	-0.485427	0.8283335	3	0	18	1	0	14
ATP6V1E1P1	1	0	0.9760479	0	2	0	2	0	0
AC104237.3	0.5	-1	0.2752642	13	13	6	3	9	4
TOMM40	0.8719671	-0.197654	0.3295649	4350	5138	5844	4582	4793	3994
AC104237.1	0	#NUM!	0.824326	0	1	0	0	0	0
AC104237.2	1.2857143	0.3625701	0.7672688	0	0	7	2	5	2
KMT2E-AS1	1.0217391	0.0310269	0.8504736	28	36	28	36	30	28
MIR6895	#DIV/0!	#DIV/0!	0.8030358	0	0	0	0	1	0
PIK3IP1-AS1	#DIV/0!	#DIV/0!	0.136155	0	0	0	3	2	2
AC093001.1	2.4285714	1.2801079	0.3356217	3	3	1	14	3	0
TOMM20	1.033511	0.0475538	0.3654221	7289	6134	7585	6433	7796	7483
TOMM22	0.879069	-0.185952	0.3212416	3178	3930	4320	3167	3713	3166
RNU6-856P	0.3333333	-1.584963	0.669913	3	0	0	0	0	1
AC087612.1	0	#NUM!	0.6840298	0	0	2	0	0	0
TOMM34	0.803638	-0.315382	0.0536789	3174	4067	4029	2902	3301	2854
OCRL	0.9080487	-0.139158	0.4843179	1409	1319	1546	1010	1432	1439
AC007848.1	0	#NUM!	0.4217812	0	0	5	0	0	0
FIZ1	0.7184725	-0.476995	0.0263465	349	399	378	271	255	283
AC022973.3	0.5	-1	0.81753	2	0	0	0	0	1
AC022973.4	2.875	1.523562	0.2085088	3	0	5	2	10	11
AC067852.2	0	#NUM!	0.824326	0	0	1	0	0	0
AC067852.6	0.5925926	-0.754888	0.4833188	9	12	6	3	1	12
AC067852.7	3.3333333	1.7369656	0.3167099	0	1	2	9	0	1
ENKD1	1.1194763	0.1628239	0.2973701	185	166	260	276	230	178
RN7SL762P	0	#NUM!	0.824326	1	0	0	0	0	0
CREBZF	0.7226901	-0.468551	0.0018786	2804	2317	3191	1555	2185	2267

TUBB8P11	4	2	0.5580302	0	0	1	0	0	4
TUBB8P12	1.1904762	0.2515388	0.5408364	13	17	33	21	23	31
AC091825.1	0.6666667	-0.584963	0.5463315	7	10	16	7	7	8
AL359510.2	1.6764706	0.7454272	0.2092197	13	6	15	22	16	19
SPEG	0.9685265	-0.046137	0.9164388	250	193	256	234	207	236
Z98752.4	5	2.3219281	0.4275856	1	0	0	0	2	3
AC103409.1	0.4736842	-1.078003	0.397167	1	14	4	1	3	5
CIAPIN1P	3	1.5849625	0.6238578	1	0	0	1	0	2
AL731567.1	0.9756098	-0.035624	0.9971629	18	11	12	12	17	11
AL139023.1	0.6986301	-0.517399	0.4073393	20	25	28	16	20	15
AL031719.2	3.1818182	1.6698514	0.0893024	0	6	5	15	6	14
SPG7	0.8686001	-0.203236	0.2884478	1755	2070	2347	1764	1863	1734
AC012640.3	1	0	0.9890559	1	0	0	1	0	0
AC012640.4	2	1	0.6367038	0	3	1	0	6	2
AC012640.5	0.7647059	-0.387023	0.7692164	9	2	6	0	4	9
AC012640.1	0.5714286	-0.807355	0.6960926	5	2	0	0	0	4
AC012640.2	0.7401747	-0.434062	0.1146136	137	165	156	86	136	117
RNU6-854P	#DIV/0!	#DIV/0!	0.8030358	0	0	0	0	0	1
SPEN	0.9689579	-0.045494	0.9614025	4123	3663	4391	2564	4227	5008
AL159169.2	1	0	0.9906088	6	0	2	0	4	4
AL159169.3	0.125	-3	0.2737675	8	0	0	0	1	0
AC010245.2	1.0555556	0.0780025	0.8506322	5	1	12	6	7	6
AC007598.3	0	#NUM!	0.824326	1	0	0	0	0	0
RNU6-853P	0	#NUM!	0.4460243	4	0	0	0	0	0
AC127070.2	2.3	1.2016339	0.1064967	8	3	9	20	17	9
AC136475.1	2	1	0.0807538	9	13	14	20	21	31
AC127070.3	1.1688312	0.2250666	0.6224071	20	15	42	13	49	28
AC127070.5	0.6551724	-0.610053	0.5084908	10	11	8	5	10	4
AC136475.5	0.375	-1.415037	0.3432266	2	8	6	3	3	0
AC136475.2	1.3	0.3785116	0.6213922	10	17	3	16	10	13
AC136475.3	1.3486239	0.431488	0.2061093	33	31	45	43	50	54
AC136475.8	3.5	1.8073549	0.3894289	0	0	2	1	1	5
AC136475.9	0	#NUM!	0.3482063	0	0	6	0	0	0
AC136475.6	#DIV/0!	#DIV/0!	0.6554062	0	0	0	0	2	0
AC136475.7	3.25	1.7004397	0.2715373	3	0	1	3	4	6
RPL13AP25	1.1051345	0.1442219	0.361135	118	125	166	155	148	149
ANAPC1P1	0.2	-2.321928	0.1942717	1	7	7	0	0	3
SPIB	0	#NUM!	0.6840298	0	0	2	0	0	0
AL356277.3	1.0769231	0.1069152	0.8426429	5	3	5	9	2	3
MLYCD	0.9065934	-0.141472	0.6015745	548	606	666	537	541	572
AL360091.3	0	#NUM!	0.6385096	0	2	0	0	0	0
AL360091.1	4	2	0.3067363	0	0	2	3	3	2
ZNF271P	1.1691094	0.2254099	0.1299018	323	232	332	301	352	384
RPL13AP20	0.40625	-1.29956	0.2249317	8	5	19	1	6	6
PRDX1P1	#DIV/0!	#DIV/0!	0.8030358	0	0	0	0	1	0
NLRX1	0.5833333	-0.777608	0.4815572	7	5	12	3	5	6
KCNMB2-AS1	1.4576271	0.5436217	0.031103	67	77	151	160	142	128
BAZ2A	0.8782405	-0.187312	0.3019691	6673	7292	7405	4416	6884	7468
BAZ2B	1.3454936	0.4281355	0.0978685	209	81	176	156	238	233
AFMID	1.308209	0.387593	0.005134	407	393	540	609	609	535
HUNK-AS1	#DIV/0!	#DIV/0!	0.8030358	0	0	0	0	0	1
DDX12P	0.8658038	-0.207888	0.3835453	444	423	601	336	439	496
OSBPL11	1.0971395	0.1337469	0.2444448	1729	1467	1838	1302	2200	2021
OSBPL10	1.1006494	0.1383549	0.1483997	1189	1340	1475	1201	1624	1582

MT1XP1	2.6666667	1.4150375	0.4227044	0	2	1	5	2	1
BAZ1A	1.071708	0.0999118	0.4102426	2396	1245	2495	1702	2640	2234
BAZ1B	0.9219783	-0.117195	0.5933613	4906	4001	5307	3271	5040	4794
AL078590.2	#DIV/0!	#DIV/0!	0.6099294	0	0	0	2	0	0
AL078590.3	3.4	1.7655347	0.1627594	1	2	2	8	5	4
INCENP	0.8534248	-0.228664	0.1834373	2676	3246	3261	2016	2825	2996
AC093249.2	0.6363636	-0.652077	0.6638438	2	7	2	2	1	4
AC093249.6	0.8666667	-0.206451	0.8493256	16	16	13	19	10	10
MIRLET7DHC	0	#NUM!	0.6436235	2	0	0	0	0	0
AC093249.4	0	#NUM!	0.4374838	0	4	0	0	0	0
ZSWIM1	0.7495327	-0.415937	0.0196125	473	529	603	350	439	414
ODC1	1.2760415	0.3516753	0.0001798	9637	10623	11641	12630	14653	13424
ABCB11	#DIV/0!	#DIV/0!	0.8030358	0	0	0	1	0	0
SPOP	1.1617598	0.2163119	0.0453824	1051	827	1145	1088	1304	1120
SPP1	2	1	0.5861243	3	1	0	4	0	4
ZSWIM8	1.0861865	0.1192719	0.2532798	494	588	612	567	617	656
ZSWIM9	1.0425532	0.060121	0.4954917	225	277	344	303	280	299
ZSWIM6	0.7531286	-0.409032	0.1341665	363	197	319	153	242	267
ZSWIM7	0.9868735	-0.019063	0.7810687	247	240	351	271	299	257
ZSWIM4	1.045929	0.0647849	0.4749106	487	479	471	494	518	491
ZSWIM5	1.2682927	0.3428877	0.6037141	22	11	8	12	14	26
ZSWIM2	0	#NUM!	0.6436235	2	0	0	0	0	0
ZSWIM3	0.7961373	-0.328911	0.2714194	142	130	194	115	132	124
ODAM	0.8209877	-0.284568	0.5124866	66	49	47	36	55	42
AC233279.1	0	#NUM!	0.2874822	1	2	2	0	0	0
ABCB10	0.9194749	-0.121118	0.594413	1757	1485	1862	1160	1729	1804
SLCO3A1	0.9923077	-0.011141	0.9674191	46	40	44	24	52	53
CASC8	1.5	0.5849625	0.6818942	0	7	1	5	3	4
CASC9	1.1529851	0.2053738	0.3312357	89	62	117	97	109	103
CASC6	0	#NUM!	0.4374838	0	4	0	0	0	0
CASC4	1.1133644	0.1549258	0.2000888	1112	819	1077	855	1231	1263
AL365184.1	#DIV/0!	#DIV/0!	0.8030358	0	0	0	1	0	0
MPV17L2	0.7533025	-0.408699	0.0175339	876	1026	1126	762	805	714
CASC2	1.6025641	0.6803821	0.1087742	29	12	37	42	42	41
CASC3	0.7689608	-0.379018	0.02447	2950	3672	3636	1934	2825	3129
RNU6-1262P	0.375	-1.415037	0.5141715	3	0	5	0	2	1
SIGLEC22P	#DIV/0!	#DIV/0!	0.8030358	0	0	0	1	0	0
CASC1	1.75	0.8073549	0.2692536	11	6	7	12	18	12
IGKV1OR-3	#DIV/0!	#DIV/0!	0.8030358	0	0	0	0	0	1
ODCP	0.9864865	-0.019629	0.9990266	16	27	31	3	26	44
AL138921.1	1.6666667	0.7369656	0.6823487	3	0	3	0	0	10
AL138921.2	2	1	0.4625762	3	0	3	4	4	4
CASD1	0.8524173	-0.230368	0.4137162	311	200	275	140	269	261
MIR1283-2	0.375	-1.415037	0.4542561	2	3	3	0	2	1
SPATA9	0.5833333	-0.777608	0.6889811	4	0	8	3	1	3
SPATA1	0.875	-0.192645	0.9021844	11	1	4	5	5	4
SPATA2	0.9274262	-0.108696	0.7879267	680	799	891	744	696	758
SPATA3	2	1	0.4814995	3	1	4	0	11	5
SPATA4	3	1.5849625	0.6122545	0	0	1	2	0	1
SPATA5	1.0997732	0.1372061	0.2860299	432	352	539	353	567	535
AL135936.1	#DIV/0!	#DIV/0!	0.3597364	0	0	0	0	0	5
SPATA6	0.9411765	-0.087463	0.968731	72	30	68	45	40	75
SPATA7	1.0917722	0.1266718	0.3835282	230	152	250	196	254	240
SPATA8	0.3333333	-1.584963	0.223973	3	9	9	0	5	2

AC011815.2	3	1.5849625	0.4758579	2	0	0	3	2	1
AC011815.3	0.2631579	-1.925999	0.1687572	9	3	7	0	2	3
AC011815.1	0.7605634	-0.39486	0.5487438	26	16	29	9	21	24
AC103740.1	1.0208333	0.0297473	0.7980234	60	28	56	52	50	45
AC103740.2	1.3060109	0.385167	0.1661881	47	65	71	91	72	76
AL132656.2	0.3846154	-1.378512	0.2445282	17	4	5	6	2	2
ODF1	0	#NUM!	0.6385096	0	2	0	0	0	0
AL132656.3	0	#NUM!	0.824326	0	0	1	0	0	0
ODF2	0.9545687	-0.067079	0.9832231	1823	1796	2258	1823	2010	1777
RABL2A	1.0166163	0.0237753	0.6977988	198	235	229	219	235	219
RABL2B	0.9247713	-0.112832	0.6568089	925	932	1094	803	959	967
TNK2-AS1	0.8285714	-0.271302	0.7255883	15	14	6	5	13	11
AL132656.4	0.9677419	-0.047306	0.9802947	13	11	7	10	13	7
AL139260.1	1	0	0.9479153	5	19	10	12	8	14
AC006277.1	0.55	-0.862496	0.1951951	19	20	41	13	11	20
AC008434.1	0.5714286	-0.807355	0.5927475	1	7	6	0	2	6
OSBPL1A	1.0056497	0.0081279	0.6916988	449	468	499	432	505	487
AC005052.1	0.25	-2	0.1062725	2	9	17	1	4	2
AC090607.2	0.9821429	-0.025995	0.9392684	16	10	30	11	22	22
SPRN	1.1674419	0.2233507	0.3416954	51	64	100	84	91	76
NUMA1	0.9251223	-0.112284	0.6605725	2249	2770	2954	2255	2588	2533
ZNRD1	1.0894309	0.1235747	0.5405427	57	91	98	88	99	81
SPTB	1.047619	0.0671142	0.8575973	14	16	12	16	14	14
ADORA2A	0.1111111	-3.169925	0.288313	0	0	9	0	0	1
ADORA2B	1.3287671	0.4100883	0.3311301	28	17	28	28	31	38
TMEM5-AS1	#DIV/0!	#DIV/0!	0.3096126	0	0	0	5	0	0
AL160290.2	#DIV/0!	#DIV/0!	0.3768753	0	0	0	2	0	2
ZNRF1	1.0823303	0.1141408	0.2550595	1761	2143	2327	2581	2283	1880
ZNRF2	0.8781619	-0.187441	0.3195046	775	759	838	550	810	723
ZNRF3	0.936528	-0.094606	0.7783798	712	769	898	655	796	777
FEM1C	0.8238401	-0.279564	0.1660631	1005	757	889	535	774	875
TM4SF19-AS1	0.2857143	-1.807355	0.4441615	3	0	4	0	1	1
AC131280.1	0	#NUM!	0.824326	0	0	1	0	0	0
FEM1A	1.2463768	0.3177403	0.5583368	34	17	18	14	30	42
FEM1B	0.9118818	-0.133081	0.5600764	1204	998	1316	826	1170	1212
AC009263.1	0	#NUM!	0.824326	0	1	0	0	0	0
POU2AF1	0	#NUM!	0.6385096	0	2	0	0	0	0
RNU6-1266P	0.5	-1	0.4729795	6	3	5	1	2	4
AP001350.2	0.6363636	-0.652077	0.6687504	6	1	15	6	2	6
SPZ1	0	#NUM!	0.824326	0	0	1	0	0	0
SPA17	1.0675676	0.0943274	0.6677375	54	37	57	55	34	69
TBC1D22A	0.9345558	-0.097647	0.772929	923	1160	1080	971	1039	946
TBC1D22B	0.9858282	-0.020592	0.8780261	1001	1202	1184	882	1202	1255
AP001350.1	0.7777778	-0.36257	0.9192743	2	2	5	6	1	0
AL627223.1	0	#NUM!	0.5337954	0	3	0	0	0	0
AC009262.1	0.75	-0.415037	0.9323259	1	1	2	3	0	0
UNC93A	1.2142857	0.2801079	0.8164388	0	10	4	3	6	8
CDC37L1-AS1	2.8461538	1.5090136	0.0841331	5	2	6	8	12	17
AC010976.1	1.1532847	0.2057487	0.4999533	47	21	69	44	65	49
FLJ36000	0.5252918	-0.928809	6.06E-05	274	398	356	136	186	218
AL359317.1	0.1666667	-2.584963	0.0986011	3	12	3	1	0	2
AL359317.2	0.4	-1.321928	0.4931207	3	7	0	3	0	1
NUMBL	0.7250674	-0.463813	0.0754491	231	192	319	184	189	165
RNU6-1257P	0	#NUM!	0.6385096	0	2	0	0	0	0

AL354714.2	1.2121212	0.277534	0.4687751	44	24	31	33	39	48
AC022387.1	1.2857143	0.3625701	0.7662105	0	3	4	3	2	4
AC004224.1	#DIV/0!	#DIV/0!	0.1690953	0	0	0	4	3	0
AC022001.3	#DIV/0!	#DIV/0!	0.6554062	0	0	0	0	2	0
AC022001.1	3	1.5849625	0.6206259	1	0	0	3	0	0
AC022001.2	0	#NUM!	0.6840298	0	0	2	0	0	0
FJX1	1.0468619	0.0660712	0.4853555	329	419	447	412	475	364
GNAI3	1.0607126	0.0850339	0.246176	3679	3079	4047	3167	4277	4017
GNAI1	1.1286432	0.1745895	0.0664916	2690	2345	2925	2412	3310	3262
GNAI2	1.0256754	0.0365743	0.4969092	1691	2135	2133	2060	2134	1918
AL133383.1	#DIV/0!	#DIV/0!	0.8030358	0	0	0	1	0	0
AL138828.1	0	#NUM!	0.6385096	0	2	0	0	0	0
YARS2	0.9957931	-0.006082	0.7482424	1236	1417	1388	1273	1511	1240
AC025881.1	0.6666667	-0.584963	0.7828402	6	0	0	1	3	0
AC106900.1	0	#NUM!	0.824326	1	0	0	0	0	0
AC084824.3	1.2857143	0.3625701	0.8014503	1	2	4	0	1	8
AC084824.4	1.1818182	0.2410081	0.9080869	11	0	0	1	6	6
AC106900.2	1.0666667	0.0931094	0.8119841	7	2	21	8	14	10
AC084824.5	1.4285714	0.5145732	0.7321111	10	1	3	2	18	0
CARS2	0.9377654	-0.092701	0.8585199	1756	1986	2380	1945	1966	1830
AC084824.1	0.85	-0.234465	0.8397464	11	3	6	4	9	4
VCPIP1	0.9364706	-0.094694	0.7891695	718	567	840	490	770	730
IRAK1	0.9441782	-0.082869	0.8316927	2800	3559	3655	2931	3400	3124
haride;GO:000	#VALUE!	#VALUE!	GO:000582	05140|hsa05	asis|Chaga	eptor associated kinase 1			
IRAK2	1.1307692	0.1773045	0.5645857	32	45	53	36	42	69
AL360007.1	0	#NUM!	0.824326	0	1	0	0	0	0
GNAO1	0.4501336	-1.151575	1.90E-11	641	813	792	285	388	338
AL731566.1	0.8965517	-0.157541	0.8184427	27	17	14	8	17	27
AL731566.2	1	0	0.9756864	2	0	0	0	2	0
CARMN	1.5	0.5849625	0.7514841	1	3	0	3	1	2
IRAK4	0.8924205	-0.164204	0.5386697	432	338	457	341	387	367
GNB1L	1.1440064	0.1940952	0.1425575	361	448	434	455	512	455
AC027455.1	#DIV/0!	#DIV/0!	0.6411933	0	0	0	0	0	2
CARM1	1.0088446	0.012704	0.6100474	2978	3845	3918	3300	3982	3554
AC019155.3	0.3333333	-1.584963	0.6715904	3	0	0	0	1	0
HNRNPU	0.9867462	-0.019249	0.7003294	34178	33063	41558	31429	38806	37122
HNRNPR	1.1481471	0.1993074	0.0185951	11210	10162	12736	10942	14274	13945
ATP5A1P2	#DIV/0!	#DIV/0!	0.2106225	0	0	0	4	0	2
AC105219.4	0.5	-1	0.1094709	28	20	34	6	21	14
AC105219.1	0.5833333	-0.777608	0.5805694	7	4	1	0	4	3
AC105219.2	0	#NUM!	0.824326	1	0	0	0	0	0
MIR22HG	0.8139098	-0.297059	0.2158098	353	287	424	287	317	262
AC005291.1	0.3333333	-1.584963	0.4271998	0	6	3	0	1	2
AC005291.2	1.3333333	0.4150375	0.8841586	0	6	0	0	6	2
AC019155.1	3	1.5849625	0.6253252	0	0	1	3	0	0
AC084083.1	0	#NUM!	0.5075476	0	0	4	0	0	0
GAS1RR	1.4468085	0.532874	0.4149507	29	6	12	13	28	27
ASTN2	0.6283582	-0.670341	0.0079759	198	241	231	144	126	151
AC069079.1	0	#NUM!	0.5337954	0	3	0	0	0	0
MID1IP1-AS1	1.0697674	0.0972972	0.9113399	9	18	16	3	31	12
NCKAP1L	1.5789474	0.6589631	0.5120557	12	1	6	3	16	11
PLEK2	2.2489796	1.1692706	9.92E-09	126	171	193	395	399	308
NHEJ1	0.5	-1	0.6865066	2	2	0	0	2	0
AC008764.10	0.2	-2.321928	0.5267773	0	0	5	0	0	1

PAFAH2	1.0453304	0.063959	0.4164761	521	613	697	558	706	650
HNRNPL	0.8693199	-0.202041	0.1886056	6732	7467	8666	6061	7085	6731
HNRNPM	0.9156927	-0.127065	0.5685078	12419	13245	15661	12283	13590	11968
SQLE	0.9509804	-0.072512	0.8193753	1831	1781	1998	1377	2031	1927
HNRNPK	0.9932938	-0.009708	0.6805376	24966	25939	29617	22889	29008	28085
HNRNPF	0.9799374	-0.029238	0.8680106	18174	20781	21456	16886	21517	20796
HNRNPD	1.1359001	0.183836	0.0265017	10886	12065	14216	13248	15326	13644
LYPLAL1-AS1	0	#NUM!	0.4374838	0	4	0	0	0	0
HNRNPC	0.9852173	-0.021486	0.7623138	31578	36468	39580	32104	38644	35287
DANCR	1.2132582	0.2788866	0.0075391	1543	1680	1921	2068	2230	1943
DAND5	#DIV/0!	#DIV/0!	0.6317974	0	0	0	1	1	0
ABCC10	0.8830645	-0.179409	0.4431741	354	450	436	290	425	380
ABCC11	1.36	0.4436067	0.5184423	6	7	12	13	15	6
ABCC12	0.25	-2	0.5682537	0	1	3	0	1	0
ABCC13	0	#NUM!	0.4460243	4	0	0	0	0	0
WASIR1	0	#NUM!	0.824326	1	0	0	0	0	0
ZNF280D	0.9329234	-0.100169	0.8393914	562	460	767	443	582	644
CATIP	0.7948718	-0.331206	0.7459246	11	26	2	18	7	6
ZNF280A	2.0222222	1.0159415	8.76E-09	199	192	194	369	395	419
ZNF280B	1.2296919	0.2982969	0.1434074	126	102	129	141	137	161
ZNF280C	0.7834008	-0.352177	0.1708443	387	242	359	173	287	314
DNAJC9-AS1	1.0625	0.0874628	0.8040573	16	26	22	21	27	20
DNAJC9-AS1	1.0625	0.0874628	0.8040573	16	26	22	21	27	20
C9orf41-AS1	0.9565217	-0.06413	0.9602782	11	3	9	2	13	7
AC073167.1	#DIV/0!	#DIV/0!	0.6411933	0	0	0	0	0	2
AL162258.2	#DIV/0!	#DIV/0!	0.5371397	0	0	0	0	0	3
FO704657.1	0.5	-1	0.6623132	0	2	6	0	4	0
AL356276.2	0	#NUM!	0.6436235	2	0	0	0	0	0
AC007450.2	#DIV/0!	#DIV/0!	0.8030358	0	0	0	0	0	1
GLYCTK	0.8540146	-0.227667	0.7680738	43	49	45	61	38	18
SQOR	1.5	0.5849625	0.7229181	2	0	2	2	2	2
WASIR2	0.6538462	-0.612977	0.5622046	7	8	11	1	14	2
VKORC1L1	1.0014042	0.0020245	0.7335669	1931	1753	2013	1470	2104	2131
AC036222.1	0	#NUM!	0.824326	0	1	0	0	0	0
AL160291.1	1.1	0.1375035	0.9347031	5	3	2	0	0	11
CHKB-AS1	0.5149701	-0.95744	0.0294409	46	68	53	28	26	32
TM7SF3	0.6757169	-0.565509	8.61E-06	7056	8023	8530	4500	5710	5743
TM7SF2	1.1669394	0.2227297	0.0994968	329	426	467	475	508	443
SLCO2B1	0.15	-2.736966	0.0002928	55	15	30	1	7	7
AL132655.1	0	#NUM!	0.284725	6	0	0	0	0	0
AL132655.2	0.2	-2.321928	0.4444947	3	0	2	0	0	1
RNU1-16P	1.5	0.5849625	0.742095	2	0	4	0	8	1
THNSL1	1.2104499	0.2755434	0.0892067	241	189	259	229	298	307
THNSL2	1.0400501	0.056653	0.4596214	469	516	613	463	609	590
AC005197.1	#DIV/0!	#DIV/0!	0.3831122	0	0	0	0	5	0
SLCO2A1	0.1304348	-2.938599	0.0559912	5	5	13	0	3	0
MAGEL2	1.5121951	0.5966443	0.3196897	5	19	17	18	25	19
RNU6-1272P	0	#NUM!	0.824326	1	0	0	0	0	0
RNU1-18P	0	#NUM!	0.824326	1	0	0	0	0	0
ID2-AS1	2	1	0.2723332	2	7	7	6	6	20
EDNRB-AS1	0	#NUM!	0.5075476	0	0	4	0	0	0
AC091185.1	0.8333333	-0.263034	0.858766	2	4	0	0	5	0
AL805961.1	3	1.5849625	0.6206259	1	0	0	3	0	0
AL805961.2	#DIV/0!	#DIV/0!	0.2887809	0	0	0	0	0	6

RN7SL704P	#DIV/0!	#DIV/0!	0.3597364	0	0	0	0	0	5
IDH2-DT	2	1	0.7703774	2	0	0	0	4	0
FAAH2	0.8677536	-0.204643	0.4972824	203	151	198	111	179	189
ECEL1P2	1.4193548	0.5052353	0.4675867	4	17	10	27	4	13
ECEL1P1	1.3333333	0.4150375	0.8999008	0	3	0	0	0	4
MIR34AHG	0.6848204	-0.546202	0.0078224	600	424	702	392	409	381
FRRS1	0.9982407	-0.00254	0.90051	1013	845	984	541	1067	1229
RPEL1	5.5	2.4594316	0.2357695	1	0	1	0	9	2
NLRC5	0.4186047	-1.25634	0.1362667	10	16	17	6	6	6
DENND3	1.0176991	0.0253111	0.6774144	210	210	258	193	250	247
NLRC3	1.75	0.8073549	0.3940553	7	7	2	7	10	11
NLRC4	0.75	-0.415037	0.7306955	5	7	12	1	4	13
AC095055.1	2	1	0.4278089	2	8	0	7	4	9
ASTE1	0.9598338	-0.059143	0.9726356	215	222	285	213	218	262
FKTN	1.1450749	0.195442	0.2838666	836	390	642	534	775	830
MLXIP	0.6112572	-0.710148	6.08E-05	602	619	680	380	446	336
FKRP	0.8480707	-0.237743	0.2991497	388	352	504	324	377	354
RN7SL702P	0	#NUM!	0.824326	0	0	1	0	0	0
NCKAP5L	0.9523337	-0.070461	0.9287175	508	787	719	600	655	663
AC114488.1	0.7762238	-0.365455	0.4618174	48	35	60	17	57	37
AC114488.2	0.84375	-0.245112	0.8812945	3	6	23	5	11	11
AC114488.3	#DIV/0!	#DIV/0!	0.8030358	0	0	0	0	1	0
AP002336.1	0	#NUM!	0.824326	0	0	1	0	0	0
AP002336.2	6	2.5849625	0.3146264	1	0	0	4	2	0
DOLPP1	0.9371206	-0.093693	0.8270058	1344	1486	1782	1411	1504	1407
RNU6-1278P	#DIV/0!	#DIV/0!	0.6411933	0	0	0	0	0	2
AC055855.2	1.08	0.1110313	0.8444469	6	3	16	2	9	16
IFI27L1	1.0475524	0.0670225	0.5024847	234	218	263	273	245	231
AC093732.1	1	0	0.9571562	1	0	6	1	6	0
IFI27L2	2.2900763	1.1953957	0.0003202	41	35	55	132	92	76
AC073896.3	0.9047619	-0.14439	0.9325622	12	13	38	15	29	13
AC073896.4	0.6086957	-0.716207	0.5538332	7	4	12	6	4	4
MAGEA9	0	#NUM!	0.824326	1	0	0	0	0	0
MAGEA8	0.91583	-0.126848	0.5801635	1779	2030	2167	1741	2015	1717
MAGEA1	0.3157895	-1.662965	0.0960441	10	14	14	1	2	9
PCLAF	1.2262314	0.2942312	0.0441823	571	394	781	748	685	708
TBC1D10C	0	#NUM!	0.4521054	1	0	3	0	0	0
MAGEA2	0.6742424	-0.568661	0.2338402	46	36	50	16	40	33
AC026470.3	2.375	1.2479275	0.3076316	2	5	1	5	3	11
AC073896.2	1.4444444	0.5305147	0.6339841	5	1	3	7	4	2
MAGEA5	0	#NUM!	0.0416498	1	8	3	0	0	0
AC114341.1	#DIV/0!	#DIV/0!	0.6099294	0	0	0	2	0	0
MAGEA6	1.2190594	0.2857684	0.0823776	264	263	281	343	374	268
MAGEA3	0.5839025	-0.776201	9.45E-10	2845	3196	3476	1680	1956	1921
TBC1D10A	0.955036	-0.066373	0.9719315	309	360	443	366	341	355
MAGEA4	2.9037037	1.5378942	9.90E-12	68	90	112	206	309	269
TBC1D10B	0.9238464	-0.114275	0.7210546	2185	2446	2867	2353	2359	2215
MAGEB2	0.9481455	-0.07682	0.8423854	824	960	993	769	1007	857
MAGEB1	14.5	3.857981	0.000141	1	2	1	17	24	17
MIR2278	#DIV/0!	#DIV/0!	0.5070017	0	0	0	3	0	0
MAGEB6	0.5	-1	0.5276494	9	3	2	5	2	0
CASZ1	2.2	1.1375035	0.4927508	4	0	1	2	7	2
DCUN1D5	0.9076997	-0.139713	0.5313572	1868	2031	2309	1843	1957	1835
MAGED1	1.0581542	0.0815499	0.3747804	470	516	596	612	569	493

DCUN1D3	0.8711898	-0.198941	0.3952125	326	317	374	237	362	287
AC128689.1	1.0705128	0.0983021	0.6917631	52	57	47	51	53	63
DCUN1D4	1.1832733	0.2427833	0.0650961	928	615	956	810	1084	1063
DCUN1D1	1.0795589	0.1104419	0.4175084	983	668	888	632	1098	1011
DCUN1D2	0.7607099	-0.394582	0.0661908	544	405	685	401	443	399
MAGEC3	0	#NUM!	0.5075476	0	0	4	0	0	0
MAGEC1	0.2	-2.321928	0.2956655	7	0	3	0	0	2
MAGEC2	0.6060606	-0.722466	0.5662831	4	4	25	5	3	12
ZRANB3	1.015942	0.0228181	0.6627382	459	415	506	400	578	424
ZRANB1	1.0633166	0.0885712	0.5008367	339	321	335	259	388	411
ZRANB2	1.0830522	0.1151027	0.3039878	2946	1777	2983	2235	3293	2818
MAGEE1	0.7491448	-0.416683	0.0951875	210	312	355	198	224	235
REX1BD	1.1073211	0.1470737	0.2371917	697	763	944	1085	909	668
AP004242.1	#DIV/0!	#DIV/0!	0.5371397	0	0	0	0	0	3
MAGED4	0.6666667	-0.584963	0.7503702	1	1	13	2	4	4
MAGED2	0.8576668	-0.221511	0.2005682	3232	3813	4133	3128	3440	3019
MAGEF1	0.9338787	-0.098693	0.8066727	1674	1996	2198	1869	1977	1634
SRA1	0.9593148	-0.059924	0.9719854	1408	1809	1920	1697	1725	1506
SGF29	0.8280543	-0.272203	0.3749562	231	362	291	280	250	202
SLC22A13	0.6666667	-0.584963	0.8350603	2	0	1	0	1	1
SLC22A14	0	#NUM!	0.4324683	2	0	2	0	0	0
SLC22A15	0.7404181	-0.433588	0.1098104	195	154	225	128	178	119
SLC22A17	1.3006536	0.3792368	0.2372255	47	57	49	56	85	58
SLC22A18	0.7291399	-0.455732	0.1034957	203	266	310	215	206	147
AL365434.1	#DIV/0!	#DIV/0!	0.6554062	0	0	0	0	2	0
SLC22A11	0.6924469	-0.530225	0.0006041	1790	2048	2239	1436	1437	1335
PPRC1	0.9451386	-0.081402	0.7992629	2048	2485	2430	1857	2283	2441
SLC22A23	0.6612903	-0.596644	0.0001595	903	963	1172	537	707	765
MAGEH1	0.9196941	-0.120774	0.6971703	780	834	1001	790	799	816
AL022068.1	0	#NUM!	0.3482063	0	0	6	0	0	0
SLC22A31	1.4259598	0.5119333	0.0031473	300	365	429	593	555	412
RTRAF	1.092213	0.1272542	0.1029356	5232	5114	6875	6170	6859	5780
UBL4A	0.8566413	-0.223237	0.2142216	2972	3555	3727	2930	3068	2786
AC004223.4	0.75	-0.415037	0.848523	2	2	4	0	0	6
AL023875.1	#DIV/0!	#DIV/0!	0.3096126	0	0	0	5	0	0
AL662844.3	0	#NUM!	0.824326	0	0	1	0	0	0
AL662844.4	1.6666667	0.7369656	0.3934746	8	3	7	9	16	5
AC114737.3	0	#NUM!	0.824326	0	0	1	0	0	0
KDM1A	1.0944018	0.1301426	0.0985684	7871	8751	9458	8248	10409	9885
KDM1B	0.8345477	-0.260934	0.1299974	1123	1051	1120	728	982	1039
AC004129.2	3	1.5849625	0.6253252	0	0	1	3	0	0
AC009511.2	#DIV/0!	#DIV/0!	0.8030358	0	0	0	0	0	1
GAS2L3	0.8781646	-0.187437	0.4581649	470	352	442	277	380	453
KDM2A	0.8479981	-0.237867	0.1287271	2496	2797	3174	2045	2500	2635
KDM2B	0.8221979	-0.282442	0.1290038	790	932	1017	641	742	869
AC024293.1	0.9280899	-0.107664	0.7894405	2177	2561	2827	2517	2468	2036
AC006372.4	0.75	-0.415037	0.9016026	1	1	6	4	2	0
ARL4AP2	0.1666667	-2.584963	0.2277644	5	0	7	0	2	0
ARL4AP4	0.25	-2	0.5132079	2	2	0	0	0	1
ARL4AP5	#DIV/0!	#DIV/0!	0.2497949	0	0	0	0	3	3
AL139021.1	6.5	2.7004397	0.1100817	1	0	1	7	2	4
AL139021.2	0.5454545	-0.874469	0.6417274	2	3	6	0	0	6
CSNK1A1P1	#DIV/0!	#DIV/0!	0.5371397	0	0	0	0	0	3
CSNK1A1P3	#DIV/0!	#DIV/0!	0.3877895	0	0	0	2	2	0

NLRP6	1.3541667	0.4374053	0.3979888	13	12	23	34	7	24
NLRP7	1.3442902	0.4268446	9.35E-05	2178	2613	2845	3095	3861	3309
NLRP4	1.1578947	0.2115041	0.638556	22	26	28	18	45	25
NLRP2	0.88495	-0.176332	0.2761489	48446	54857	60591	45043	51352	48643
NLRP3	0.8133333	-0.298081	0.5099997	64	43	43	26	46	50
NLRP1	0.7019231	-0.510615	0.3496269	30	29	45	19	24	30
GAS2L1	0.9754902	-0.035801	0.8525192	390	500	538	501	465	427
ST3GAL5-AS1	4	2	0.1900734	2	2	0	2	5	9
AC108463.1	0.25	-2	0.2600005	5	1	10	0	0	4
AC108463.2	0	#NUM!	0.1188281	1	1	7	0	0	0
AC005540.1	7	2.8073549	0.2798517	0	0	1	7	0	0
AC108463.3	3	1.5849625	0.6074549	1	0	0	2	0	1
AC022148.1	1.0833333	0.1154772	0.8215887	5	3	16	6	14	6
CASS4	2.6666667	1.4150375	0.4231314	1	0	2	7	0	1
BMPR1B	0.8	-0.321928	0.9391316	3	1	1	4	0	0
BMPR1A	1.1287129	0.1746785	0.4309117	122	69	112	90	122	130
KDM5A	0.9915547	-0.012236	0.8135978	1726	1529	1955	1257	1873	2036
MUS81	0.9610338	-0.057341	0.9399225	737	837	941	863	809	745
KDM5B	1.133836	0.181212	0.0631123	23770	23179	26529	21406	30241	31665
KDM5C	0.799177	-0.323413	0.0416351	3728	4746	4891	2813	3893	3975
KDM5D	1.0152439	0.0218264	0.7052249	667	648	653	515	758	725
HS2ST1	1.0177515	0.0253853	0.6657391	646	475	738	452	690	750
CASP9	1.3663484	0.4503254	0.0102594	238	293	307	381	456	308
FLCN	0.8214058	-0.283833	0.1279542	900	981	1249	852	889	830
CASP7	1.2868421	0.363835	0.0033638	617	573	710	674	959	812
CASP8	1.0971957	0.1338208	0.1348037	968	965	1205	967	1277	1199
CASP5	0.6666667	-0.584963	0.7320616	9	0	9	7	3	2
CASP6	1.1588974	0.2127529	0.0490725	707	784	976	929	1038	892
CASP3	1.138736	0.1874333	0.088726	1596	1212	1733	1371	1887	1913
CASP4	1.0960393	0.1322995	0.1518736	925	882	1046	1003	1151	973
CASP1	1.1348315	0.1824781	0.5083077	118	65	84	76	138	89
CASP2	0.7784211	-0.361377	0.0208419	1144	1214	1442	772	1126	1060
KDM6A	0.9237332	-0.114452	0.6441602	1324	1095	1449	907	1325	1341
KDM6B	0.9955025	-0.006503	0.7198739	1804	2236	2408	1832	2321	2266
SRGN	0	#NUM!	0.6410557	1	1	0	0	0	0
SPATS2	0.8761345	-0.190776	0.3413361	643	600	630	469	584	588
AC016999.1	#DIV/0!	#DIV/0!	0.5371397	0	0	0	0	0	3
CASQ2	0.5757576	-0.796467	0.2898354	9	34	23	7	8	23
RSU1P2	7	2.8073549	0.3235909	0	1	0	0	0	7
CASQ1	1.061828	0.08655	0.5529241	119	72	181	116	136	143
KDM7A	1.0336022	0.047681	0.7369462	308	180	256	165	295	309
KDM3A	1.2121701	0.2775921	0.0265815	1736	1337	1890	1509	2180	2327
KDM3B	0.7411752	-0.432113	0.0058813	5740	6353	6661	3447	4842	5611
AC104964.2	0	#NUM!	0.824326	0	0	1	0	0	0
AC104964.3	0.6363636	-0.652077	0.6434572	3	6	2	0	5	2
AL022318.1	#DIV/0!	#DIV/0!	0.8030358	0	0	0	1	0	0
TBRG4	0.8113545	-0.301596	0.0818499	3165	3714	3760	2997	2892	2743
GNA13	1.0563051	0.0790266	0.4855483	2831	1908	2525	1807	2888	2978
TBRG1	0.7692308	-0.378512	0.0307589	607	569	761	442	517	531
AC010487.1	1.1318681	0.1787059	0.6532613	36	25	30	25	49	29
GNA14	1	0	0.9890559	0	0	1	0	1	0
AC010487.2	1.3076923	0.3870231	0.6744527	7	2	4	10	5	2
GNA15	0.6666667	-0.584963	0.8863447	2	0	4	4	0	0
GNA11	0.946961	-0.078623	0.914403	1075	1137	1276	1139	1199	965

GNA12	1.2339828	0.3033223	0.0061791	1594	2011	2092	2099	2583	2348
ZNF286B	1.1338583	0.1812403	0.5177705	25	35	67	45	53	46
RAP1AP	0.5714286	-0.807355	0.6645587	3	4	0	0	1	3
ZNF286A	1.2801724	0.3563381	0.0410059	250	165	281	263	300	328
KDM4A	0.8643154	-0.21037	0.2864584	714	787	909	535	750	798
KDM4B	0.7619911	-0.392154	0.006372	2109	2372	2691	1655	1852	1958
KDM4C	1.0699366	0.0975254	0.2514102	1348	1266	1647	1193	1726	1640
KDM4D	0.7773109	-0.363436	0.3159241	77	85	76	56	65	64
KDM4E	0.2857143	-1.807355	0.4670648	4	0	3	0	0	2
KDM4F	2	1	0.7741804	1	0	0	2	0	0
AL109933.2	0	#NUM!	0.6840298	0	0	2	0	0	0
ZNF285B	#DIV/0!	#DIV/0!	0.6554062	0	0	0	0	2	0
DDX18P1	7	2.8073549	0.1501281	0	0	2	0	6	8
DDX18P3	0.25	-2	0.3622943	6	0	2	0	1	1
AL450263.1	0.826087	-0.275634	0.8138702	14	2	7	4	6	9
VPREB3	#DIV/0!	#DIV/0!	0.8030358	0	0	0	0	0	1
HCCAT5	#DIV/0!	#DIV/0!	0.8030358	0	0	0	0	0	1
AP003068.2	1.4823529	0.567889	0.1702538	23	36	26	52	35	39
RBFOX2	0.8381336	-0.254748	0.0934857	3487	3331	3598	2270	3260	3200
AP003068.4	1.375	0.4594316	0.7185799	0	2	6	1	4	6
RBFOX3	0.3636364	-1.459432	0.2307835	11	4	7	1	6	1
USP2-AS1	1.35	0.4329594	0.5326425	15	12	13	5	18	31
DDX39A	0.9155725	-0.127254	0.6614442	4414	5250	6101	4918	4805	4711
DDX39B	0.8966452	-0.157391	0.5210979	1970	1477	2455	1487	1894	1911
ABCB1	2.4444444	1.2895066	0.2444336	5	1	3	6	11	5
ABCB6	0.6388889	-0.646363	0.4728306	8	13	15	6	6	11
ABCB7	0.9075044	-0.140024	0.5485987	722	705	865	472	855	753
ABCB4	1.8823529	0.9125372	0.2824607	17	16	1	9	23	32
FLII	0.7604468	-0.395081	0.0052297	3483	4045	4557	2912	3199	3079
PRDX3P3	0	#NUM!	0.6840298	0	0	2	0	0	0
ABCB8	0.7701863	-0.376721	0.037842	642	737	875	568	623	545
OR4D12P	0.4444444	-1.169925	0.5608952	5	1	3	3	0	1
ABCB9	0.806841	-0.309644	0.2603878	151	154	192	112	152	137
IQGAP1	1.0510253	0.0717974	0.4775107	25537	17271	25511	16560	27432	27813
TAPBPL	0.6153846	-0.70044	0.2773683	43	20	54	15	12	45
IQGAP2	5.6666667	2.5025003	1.62E-09	15	14	13	75	96	67
IQGAP3	0.6594047	-0.600764	5.61E-05	1005	1074	1180	614	844	691
PCNX3	0.8112778	-0.301732	0.0633195	2150	2641	2675	1947	2135	1975
PCNX4	0.8976401	-0.155791	0.5255599	1254	833	1303	747	1195	1101
PCNX1	0.8662226	-0.20719	0.2727417	1259	1086	1258	742	1217	1162
PCNX2	0.702403	-0.509629	0.065545	200	131	210	101	136	143
JHDM1D-AS1	0.8712446	-0.19885	0.75706	71	48	114	65	61	77
MTCYBP3	0	#NUM!	0.5337954	0	3	0	0	0	0
AC007603.1	1	0	0.9621306	2	0	4	0	0	6
ABCA1	0.6798246	-0.556766	0.1455149	100	58	70	40	64	51
ABCA2	0.57222	-0.805358	7.45E-06	710	841	976	515	484	447
AC005544.1	0	#NUM!	0.824326	0	1	0	0	0	0
SRMS	0	#NUM!	0.824326	1	0	0	0	0	0
ABCA5	0.8371134	-0.256505	0.4588451	180	114	191	83	142	181
ABCA6	0.5	-1	0.8079011	0	2	0	1	0	0
ABCA3	0.4	-1.321928	0.3227941	3	11	6	4	4	0
ABCA4	1.3180516	0.3984068	0.0041768	417	467	512	566	583	691
ABCA9	0.2	-2.321928	0.3816683	0	0	10	0	2	0
ABCA7	0.9797482	-0.029517	0.8050591	482	629	716	623	583	584

ABCA8	2	1	0.7782384	0	0	1	2	0	0
AC131532.1	0.9779412	-0.03218	0.983669	40	40	56	28	66	39
AC084082.1	0.5714286	-0.807355	0.7098946	1	3	3	2	0	2
SRPX	7.7	2.9448584	0.0003142	0	4	6	20	39	18
LMBR1	0.8826008	-0.180167	0.3506887	1144	1100	1078	733	1081	1118
AL356275.1	0	#NUM!	0.6436235	2	0	0	0	0	0
OFD1	1.0911493	0.1258485	0.3703175	601	284	629	498	601	553
SS18	0.9477415	-0.077435	0.8372414	2765	2382	3177	2123	2951	2815
SRP9	1.0241351	0.034406	0.4798536	5317	4059	5250	4473	5491	5015
PRDX3P1	0.6129032	-0.706269	0.4548693	19	5	7	5	8	6
PRDX3P2	1.3333333	0.4150375	0.797185	2	1	3	0	4	4
AL731563.2	0.5	-1	0.7214899	0	4	0	0	0	2
MIR1285-1	0.2222222	-2.169925	0.4271876	0	0	9	0	2	0
AL731563.3	1.72	0.7824086	0.2626824	10	5	10	13	18	12
AL731563.1	#DIV/0!	#DIV/0!	0.4242335	0	0	0	0	3	1
SRRT	1.0325328	0.0461877	0.3560607	5029	5424	6453	5933	6068	5455
AC073264.3	0.5	-1	0.7057127	3	1	0	1	0	1
FLNA	0.8956406	-0.159008	0.4020438	14623	18735	17131	12273	15660	17287
FLNB	0.8282345	-0.271889	0.1285856	11877	14487	13318	7621	11936	13309
FLNC	1.5376884	0.6207632	0.0140465	124	152	122	129	234	249
FABP9	1	0	0.9722049	5	0	0	0	3	2
RNU6-1281P	0	#NUM!	0.824326	1	0	0	0	0	0
FABP1	0	#NUM!	0.1851375	8	0	0	0	0	0
FABP2	0	#NUM!	0.824326	0	0	1	0	0	0
FABP3	0.323601	-1.627712	1.24E-07	124	127	160	47	43	43
FABP5	1.1370968	0.185355	0.5230792	37	40	47	51	33	57
FABP6	0.8181818	-0.289507	0.8213544	5	21	7	17	4	6
FABP7	1.4296905	0.5157029	2.62E-05	1604	1699	1931	2585	2720	2178
SRRD	0.8191409	-0.287816	0.1614436	398	412	517	276	426	385
AL365436.1	1.6666667	0.7369656	0.6164139	4	0	2	3	1	6
AL365436.2	0.6666667	-0.584963	0.6493124	3	12	0	0	8	2
AC114489.1	0	#NUM!	0.6631647	1	0	1	0	0	0
AL121832.2	0.9151786	-0.127875	0.8076921	74	66	84	57	78	70
AL121832.3	0.7256098	-0.462734	0.3490433	41	47	76	43	34	42
AL121832.1	1.6666667	0.7369656	0.6728597	0	1	2	3	1	1
RCC1L	0.9303271	-0.10419	0.8003873	1506	1977	2172	1785	1809	1667
MIR4520-1	0	#NUM!	0.6840298	0	0	2	0	0	0
SLCO1B1	1.0833333	0.1154772	0.8214441	3	2	7	9	0	4
HMGN2P4	0	#NUM!	0.130371	3	1	4	0	0	0
HMGN2P5	1.03125	0.0443941	0.4995574	419	519	598	533	547	504
AC068831.6	0	#NUM!	0.824326	0	0	1	0	0	0
AC068831.7	0.4285714	-1.222392	0.5816372	0	6	1	2	1	0
HMGN2P3	0.5789474	-0.788496	0.1982181	24	22	30	14	16	14
AC068831.2	0.2352941	-2.087463	0.18663	7	3	7	0	4	0
HMGN2P6	0	#NUM!	0.824326	0	1	0	0	0	0
RNU6-1285P	#DIV/0!	#DIV/0!	0.8030358	0	0	0	1	0	0
SLCO1A2	1.053528	0.0752286	0.5689106	144	100	167	128	159	146
MYCBPAP	1.0222222	0.0317089	0.8595015	14	15	16	21	12	13
AC106798.1	8	3	0.1801949	0	0	1	4	1	3
CNN2P10	0	#NUM!	0.4185478	3	0	1	0	0	0
FLT1	0.8194444	-0.287282	0.6434354	19	30	23	7	25	27
FLT3	#DIV/0!	#DIV/0!	0.6554062	0	0	0	0	2	0
FLT4	1.3338048	0.4155476	0.0061555	411	404	599	691	666	529
AC095056.1	0	#NUM!	0.824326	0	1	0	0	0	0

AL136370.1	1.6222222	0.6979715	0.2064653	11	15	19	19	36	18
AL358334.3	0.2307692	-2.115477	0.1979974	5	6	2	0	1	2
AL358334.1	#DIV/0!	#DIV/0!	0.3334379	0	0	0	0	4	1
AL358334.2	#DIV/0!	#DIV/0!	0.6554062	0	0	0	0	2	0
ATP11A-AS1	#DIV/0!	#DIV/0!	0.8030358	0	0	0	0	0	1
AC100757.4	0.25	-2	0.4120095	0	1	7	0	1	1
AC100757.1	2.375	1.2479275	0.2921533	0	4	4	6	7	6
RNU6-807P	1.8333333	0.8744691	0.5797331	4	2	0	8	3	0
TIMM8AP1	0.5789474	-0.788496	0.6219976	1	2	16	3	3	5
AC005304.3	0	#NUM!	0.6410557	1	1	0	0	0	0
PCOLCE2	#DIV/0!	#DIV/0!	0.0915464	0	0	0	1	1	7
AC005304.1	0.2307692	-2.115477	0.2176706	8	1	4	0	2	1
BICRA-AS1	#DIV/0!	#DIV/0!	0.6554062	0	0	0	0	2	0
AP001505.2	1.1521739	0.2043585	0.548333	32	29	31	49	31	26
AC107241.1	1	0	0.9890559	0	0	1	0	1	0
AL589666.2	0	#NUM!	0.4217812	0	0	5	0	0	0
HIP1R	0.9349532	-0.097034	0.7957506	1151	1307	1493	1199	1283	1212
AC008432.1	0	#NUM!	0.824326	1	0	0	0	0	0
AL136526.1	0.8333333	-0.263034	0.9118737	4	1	7	4	4	2
AC128688.1	0.9047619	-0.14439	0.9404503	9	2	10	5	9	5
AC128688.2	1.1111111	0.1520031	0.7851336	16	2	18	7	12	21
SHQ1P1	#DIV/0!	#DIV/0!	0.8030358	0	0	0	1	0	0
GRK6P1	1.3333333	0.4150375	0.8341076	1	2	0	4	0	0
CDYLP1	#DIV/0!	#DIV/0!	0.0167931	0	0	0	4	0	13
RN7SL711P	#DIV/0!	#DIV/0!	0.5371397	0	0	0	0	0	3
DANT2	0.7272727	-0.459432	0.6149101	16	6	11	2	10	12
AC026471.4	0.7826087	-0.353637	0.751525	8	5	10	3	7	8
ST8SIA4	1.3575051	0.4409576	0.0007158	2201	1579	2136	2121	2949	2961
AC026471.2	0.2142857	-2.222392	0.1707708	5	6	3	1	2	0
ST8SIA6	0	#NUM!	0.0273098	3	12	0	0	0	0
A2M	1.6666667	0.7369656	0.6418558	5	0	1	3	5	2
ST8SIA1	0.7913669	-0.337581	0.4940016	50	50	39	41	43	26
HSPA12A	0.5735294	-0.802061	0.0060298	110	131	167	70	91	73
HSPA12B	4	2	0.1437624	1	0	4	2	4	14
AL136131.2	#DIV/0!	#DIV/0!	0.6554062	0	0	0	0	2	0
ARMC10	1.0529086	0.0743802	0.2923342	1207	1054	1349	1142	1396	1263
ARMC12	#DIV/0!	#DIV/0!	0.6411933	0	0	0	0	0	2
DIRAS3	2.5	1.3219281	0.107546	7	2	7	13	8	19
AC004466.1	0	#NUM!	0.6840298	0	0	2	0	0	0
AC004466.2	0	#NUM!	0.4521054	1	0	3	0	0	0
AC004466.3	0	#NUM!	0.2394948	0	0	8	0	0	0
DIRAS1	3	1.5849625	0.660331	0	0	1	0	2	1
AL022069.1	0.7391304	-0.436099	0.6676743	10	9	4	5	5	7
DIRAS2	0.4814815	-1.054448	0.3065803	12	9	6	1	4	8
LMCD1	0.518732	-0.946939	0.0023952	95	110	142	50	64	66
AC026471.1	0.6865672	-0.542527	0.2902323	38	32	64	30	34	28
AL161669.3	0	#NUM!	0.824326	0	0	1	0	0	0
AC010978.1	15	3.9068906	0.044306	0	1	0	13	1	1
AL512274.1	0.7407407	-0.432959	0.7406955	7	3	17	7	8	5
GLCCI1	0.9960552	-0.005702	0.8889954	352	320	342	241	429	340
MKRN9P	1.6111111	0.688056	0.5177327	8	5	5	1	19	9
AC002558.3	#DIV/0!	#DIV/0!	0.6482162	0	0	0	0	1	1
MAST4-AS1	1.3333333	0.4150375	0.4543893	19	6	20	15	23	22
SPC24	0.9856864	-0.020799	0.8142174	388	539	610	455	545	515

SPC25	1.0034037	0.0049021	0.7696393	976	912	1050	716	1179	1053
GEMIN2	0.9521762	-0.070699	0.952896	575	595	691	586	634	552
ADAMTS9-AS	0.8478261	-0.23816	0.7740816	20	10	16	10	16	13
ADAMTS9-AS	0.5978261	-0.742202	0.1812317	31	28	33	10	26	19
AC010342.1	2	1	0.5963347	3	1	0	1	2	5
AL050327.1	0	#NUM!	0.6436235	2	0	0	0	0	0
KIRREL3	1.1315789	0.1783372	0.7358266	8	14	16	10	14	19
KIRREL1	1.1545455	0.207325	0.5592466	36	39	35	31	41	55
KIRREL2	0.9875	-0.018147	0.8455807	13	23	44	35	16	28
GEMIN4	0.7863558	-0.346746	0.0156542	1983	2130	2234	1539	1761	1691
GEMIN5	0.8116672	-0.30104	0.0542389	2147	2167	2440	1437	1922	2123
MKRN5P	0	#NUM!	0.824326	1	0	0	0	0	0
GEMIN6	1.0954231	0.1314883	0.174214	715	732	869	855	906	776
AC092658.1	1	0	0.9890154	4	0	0	0	0	4
GEMIN7	1.2692308	0.3439544	0.1128472	91	130	143	173	155	134
GEMIN8	1.0211132	0.0301429	0.6470047	154	159	208	179	183	170
SLC1A1	2.0291734	1.0208922	2.68E-07	239	183	195	315	447	490
SLC1A2	8.2142857	3.0381351	4.98E-05	12	0	2	38	38	39
SLC1A3	0.9040878	-0.145465	0.5558304	2174	1569	2544	1493	2104	2087
SLC1A4	1.5029104	0.587759	0.0004334	244	225	390	413	467	411
SLC1A5	1.2389267	0.3090908	0.0120596	1571	1926	1944	2366	2371	2004
SLC1A6	2.4868035	1.3142925	6.47E-08	128	102	111	351	265	232
SLC1A7	1.3846154	0.4694853	0.6877128	1	8	4	2	7	9
AL356274.1	2.75	1.4594316	0.4066182	2	0	2	3	8	0
AL356274.2	0.7272727	-0.459432	0.7086192	6	4	12	4	6	6
AC097359.2	0.6141975	-0.703225	0.0058764	196	250	202	114	167	117
MRPS21P6	#DIV/0!	#DIV/0!	0.4737294	0	0	0	0	4	0
SSH2	0.8334143	-0.262894	0.1980238	680	720	659	400	632	684
SSH3	0.8430394	-0.246328	0.2033325	526	579	698	446	547	527
SSH1	0.9744156	-0.037391	0.9597016	2333	2484	2883	1800	2702	3001
NUTF2P7	3	1.5849625	0.4532547	1	1	0	3	2	1
FMC1	0.6923077	-0.530515	0.7006206	4	5	4	3	3	3
MRPS21P9	0	#NUM!	0.6436235	2	0	0	0	0	0
AC022730.3	2	1	0.6554224	1	0	1	1	1	2
AC022730.4	1.528	0.6116445	0.0025422	111	116	148	174	193	206
MKRN7P	4	2	0.0712737	6	1	1	5	15	12
AC125807.1	0.5	-1	0.8638172	0	0	2	1	0	0
AC125807.2	0.7146958	-0.484599	0.0078814	585	580	577	355	417	473
HSFY1P1	#DIV/0!	#DIV/0!	0.1700664	0	0	0	0	9	0
NUP62	0.964112	-0.052727	0.924791	4016	4813	5354	4558	4729	4387
VCPKMT	0.948168	-0.076785	0.9572134	394	297	428	369	431	261
CELA2B	0	#NUM!	0.6604275	0	1	1	0	0	0
TMIGD3	0	#NUM!	0.5781912	0	0	3	0	0	0
TMIGD1	#DIV/0!	#DIV/0!	0.8030358	0	0	0	0	1	0
AC136628.3	0.3333333	-1.584963	0.4252607	4	2	3	0	0	3
CDC20P1	1.5185185	0.6026645	0.2926736	9	29	16	20	37	25
TMIGD2	1.3504274	0.433416	0.2186962	31	48	38	52	49	57
CLK3P2	0.6428571	-0.63743	0.7262451	0	6	8	5	2	2
NUP50	0.9646733	-0.051888	0.9700597	6876	5231	7057	4707	6867	6913
NUP54	1.2211991	0.2882985	0.0186834	1678	1137	1855	1656	2139	1908
AL049637.1	1	0	0.971258	1	0	2	0	0	3
AL049637.2	0	#NUM!	0.824326	0	1	0	0	0	0
NUP58	0.9461194	-0.079906	0.7663112	3513	3119	3650	2241	3818	3669
MKRN4P	0.7643759	-0.387646	0.1152922	200	258	255	151	184	210

NUP43	0.7904828	-0.339194	0.0162093	2766	2768	3124	1880	2413	2551
AL022163.1	#DIV/0!	#DIV/0!	0.5509701	0	0	0	0	3	0
ZBTB46-AS1	0.8172043	-0.291231	0.2851863	164	195	199	117	159	180
NUP35	1.102649	0.1409736	0.150412	774	656	986	795	1007	862
NUP37	0.9656726	-0.050394	0.984482	1945	2012	2481	1717	2410	2090
SPAAR	0.1666667	-2.584963	0.0443229	8	11	11	0	5	0
PRDX2P4	0	#NUM!	0.5387425	3	0	0	0	0	0
STMN3	1.0425532	0.060121	0.8497632	20	7	20	15	22	12
STMN4	0	#NUM!	0.824326	0	0	1	0	0	0
PCMT1	0.936801	-0.094186	0.7549423	2151	2453	2817	2084	2527	2341
GNAT3	0.5	-1	0.7177262	0	3	1	0	0	2
STMN1	1.3313722	0.4129139	4.61E-05	6643	7179	8274	9557	10763	9098
TSPYL2	0.687983	-0.539555	0.0002115	1596	1599	1981	1177	1265	1119
TSPYL1	0.9020432	-0.148732	0.4598456	1632	1738	1867	1512	1665	1547
DAPK1	0.9080256	-0.139195	0.5157579	1795	1758	2079	1173	1930	2011
DAPK2	0	#NUM!	0.824326	0	0	1	0	0	0
DAPK3	1.0678491	0.0947078	0.2543124	989	1156	1407	1308	1303	1182
AP000919.4	1.0909091	0.1255309	0.8852976	4	2	5	2	4	6
AP000919.1	1.1666667	0.2223924	0.7183769	13	10	13	12	23	7
AP000523.1	0.725	-0.463947	0.5753413	17	9	14	5	10	14
AL078601.2	1.375	0.4594316	0.6770739	4	0	4	9	1	1
TSPYL4	0.8174924	-0.290723	0.1218535	590	673	715	454	549	614
ENPEP	1.3046296	0.3836403	0.0233036	379	323	378	327	551	531
ABO	2	1	0.100722	18	5	13	24	30	18
ABR	1.0573222	0.0804151	0.2180103	4400	4808	5289	4625	5483	5220
STMP1	1.0721241	0.1004718	0.2826304	915	918	940	949	1103	921
SSR1	0.9646517	-0.05192	0.9999071	5351	4247	5820	3997	5498	5378
ACD	0.8872666	-0.172561	0.5456906	791	1062	1092	955	893	765
ST14	0.6125812	-0.707027	2.17E-06	1691	2198	2422	1172	1388	1306
ST13	0.9821322	-0.026011	0.7396464	16958	15365	19838	15570	18261	17398
THAP12	0.7921562	-0.336143	0.0660251	2062	1419	2052	1108	1630	1645
ST18	#DIV/0!	#DIV/0!	0.8030358	0	0	0	0	1	0
THAP11	1.0923345	0.1274147	0.257936	436	604	682	649	643	589
ACR	0	#NUM!	0.824326	1	0	0	0	0	0
OGDH	0.7429635	-0.428637	0.0039281	2474	3060	3064	1881	2206	2301
SSPN	0.5	-1	0.8289598	1	0	1	0	1	0
PRDX2P3	0	#NUM!	0.6436235	2	0	0	0	0	0
SSPO	1.3333333	0.4150375	0.8656803	3	0	0	2	2	0
ST20	0.725	-0.463947	0.534316	23	14	43	17	14	27
ADA	0.8226002	-0.281737	0.4531481	215	302	306	301	190	186
OGFR	1.1326452	0.179696	0.0807765	3545	4612	4825	4951	5219	4534
OGG1	0.8924731	-0.164119	0.4868765	397	414	398	322	398	359
ADK	1.2638102	0.3377798	0.0019879	1683	1875	2090	2261	2712	2165
ADM	1.3984018	0.483779	0.0003762	1076	1203	1225	1745	1751	1404
FMN1	0.6275479	-0.672203	0.0008704	567	452	600	231	379	406
FMN2	2.75	1.4594316	0.3703122	1	2	1	1	6	4
ADO	1.0996823	0.1370868	0.2553334	863	739	916	663	1092	1014
AP003559.1	0.7	-0.514573	0.6522527	6	6	8	3	5	6
AC012645.2	5	2.3219281	0.385839	0	0	1	4	0	1
AC012645.4	0.3571429	-1.485427	0.4024588	0	8	6	2	0	3
AC012645.1	1.2727273	0.3479233	0.2315621	45	58	62	63	84	63
GARNL3	0.6862745	-0.543142	0.3770396	31	23	48	11	20	39
SSR4	0.9199687	-0.120343	0.7360266	3691	4462	4642	4361	4020	3390
SSR2	0.933001	-0.100049	0.7751899	4904	5798	6149	5344	5436	4942

SSR3	0.9782939	-0.03166	0.8049818	5194	4745	5863	4736	5557	5166
AEN	0.6551876	-0.61002	1.26E-06	4057	4589	5002	2725	3206	3011
BAAT	1.3333333	0.4150375	0.769583	0	1	5	1	5	2
AL513497.1	0.55	-0.862496	0.2812486	11	18	31	2	13	18
AES	1.1625301	0.2172681	0.0642504	2350	2931	3019	3546	3282	2821
SCARNA13	1	0	0.9784571	3	2	0	0	1	4
SCARNA14	0	#NUM!	0.6385096	0	2	0	0	0	0
SCARNA11	0	#NUM!	0.824326	0	1	0	0	0	0
SCARNA11	0	#NUM!	0.824326	0	1	0	0	0	0
SCARNA11	0	#NUM!	0.824326	0	1	0	0	0	0
SCARNA11	0	#NUM!	0.824326	0	1	0	0	0	0
SCARNA17	0	#NUM!	0.3473206	0	5	0	0	0	0
SCARNA17	0	#NUM!	0.3473206	0	5	0	0	0	0
SCARNA17	0	#NUM!	0.3473206	0	5	0	0	0	0
SCARNA17	0	#NUM!	0.3473206	0	5	0	0	0	0
SCARNA17	0	#NUM!	0.3473206	0	5	0	0	0	0
SCARNA18	0	#NUM!	0.824326	1	0	0	0	0	0
SCARNA18	0	#NUM!	0.824326	1	0	0	0	0	0
SCARNA18	0	#NUM!	0.824326	1	0	0	0	0	0
SCARNA15	0.3142857	-1.669851	0.133557	11	2	22	2	5	4
SCARNA15	0.3142857	-1.669851	0.133557	11	2	22	2	5	4
SCARNA15	0.3142857	-1.669851	0.133557	11	2	22	2	5	4
SCARNA15	0.3142857	-1.669851	0.133557	11	2	22	2	5	4
SCARNA15	0.3142857	-1.669851	0.133557	11	2	22	2	5	4
LMAN1	1.0070469	0.0101308	0.7622712	2390	1554	2158	1481	2314	2350
LMAN2	0.949653	-0.074528	0.9416655	10734	13491	14685	12510	13198	11243
AC025884.1	0.4105263	-1.284453	0.0431853	24	20	51	12	18	9
AC025884.2	0.7647059	-0.387023	0.7084263	2	21	11	10	11	5
AC104472.1	#DIV/0!	#DIV/0!	0.6099294	0	0	0	2	0	0
AGA	1.1924847	0.2539707	0.0487673	372	436	496	509	522	524
SCARNA24	0.7368421	-0.440573	0.8288404	9	1	9	9	0	5
SCARNA24	0.7368421	-0.440573	0.8288404	9	1	9	9	0	5
SCARNA20	1	0	0.978278	0	0	2	0	2	0
SCARNA20	1	0	0.978278	0	0	2	0	2	0
SCARNA20	1	0	0.978278	0	0	2	0	2	0
SCARNA20	1	0	0.978278	0	0	2	0	2	0
SCARNA20	1	0	0.978278	0	0	2	0	2	0
SCARNA20	1	0	0.978278	0	0	2	0	2	0
SCARNA20	1	0	0.978278	0	0	2	0	2	0
AGK	0.7891566	-0.341616	0.0881307	592	456	612	322	529	459
SCARNA21	0.25	-2	0.5141485	3	1	0	1	0	0
SCARNA21	0.25	-2	0.5141485	3	1	0	1	0	0
SCARNA21	0.25	-2	0.5141485	3	1	0	1	0	0
AC104472.2	0.1	-3.321928	0.1280173	3	5	2	0	1	0
AGL	1.133438	0.1807055	0.210664	1504	898	1420	1057	1539	1736
FMO1	2.2857143	1.1926451	0.4185366	1	0	6	0	7	9
FMO2	0.1666667	-2.584963	0.3674512	3	0	3	1	0	0
FMO4	0.6237942	-0.680858	0.0370462	102	85	124	47	83	64
FMO5	0.8529412	-0.229482	0.6444239	64	47	93	44	63	67
AGT	0.9243986	-0.113413	0.9583207	82	87	122	121	84	64
HINFP	0.9101928	-0.135756	0.659865	483	564	768	476	601	575
AC003002.2	0	#NUM!	0.355562	5	0	0	0	0	0
XAB2	0.9869387	-0.018968	0.7333541	1980	2248	2586	2318	2436	1971
FMOD	#DIV/0!	#DIV/0!	0.6554062	0	0	0	0	2	0

AC093484.1	#DIV/0!	#DIV/0!	0.8030358	0	0	0	0	0	1
FMR1	1.1263069	0.1715999	0.140674	2531	1823	2724	1983	3086	2903
AC093484.5	1.5434783	0.6261852	0.1329954	33	17	42	32	66	44
AHR	1.0714286	0.0995357	0.5655152	128	89	133	100	115	160
WASH2P	0.7413074	-0.431856	0.1049004	217	217	285	187	209	137
AC011816.2	#DIV/0!	#DIV/0!	0.2758481	0	0	0	4	0	1
AC073263.1	0.8571429	-0.222392	0.919487	5	0	2	2	4	0
SSX3	2.5	1.3219281	0.6419999	2	0	0	0	4	1
AL451007.3	0	#NUM!	0.824326	0	1	0	0	0	0
AL451007.1	0	#NUM!	0.0749753	0	10	1	0	0	0
PPTC7	1.0558003	0.078337	0.4663009	726	578	739	520	806	831
AIP	0.8950884	-0.159898	0.6458263	680	911	954	879	774	625
ARMCX3	1.0099146	0.0142333	0.5812433	1179	1102	1350	1095	1397	1175
ST7L	1.0723781	0.1008137	0.4766536	208	254	215	254	250	222
ARMCX4	0	#NUM!	0.2325162	2	4	0	0	0	0
GPATCH11	1.0744111	0.1035462	0.3950119	680	424	764	501	772	734
ARMCX2	0.8656716	-0.208108	0.8508383	37	5	25	19	12	27
ARMCX5	0.9119071	-0.133041	0.6159605	360	316	357	214	407	321
ARMCX6	1.3285714	0.4098758	0.333039	26	14	30	42	27	24
AC106799.2	1.5	0.5849625	0.7575715	2	2	0	4	2	0
AC106799.3	4	2	0.5580302	0	0	1	0	0	4
RPS10P18	3	1.5849625	0.6199808	0	1	0	3	0	0
AC106799.1	#DIV/0!	#DIV/0!	0.263214	0	0	0	0	5	1
NOTCH2	0.7823344	-0.354143	0.0665245	3408	3800	3570	1846	2897	3689
NOTCH3	0.9867813	-0.019198	0.8799897	2363	2753	2676	1986	2728	2975
RPS10P14	0	#NUM!	0.5337954	0	3	0	0	0	0
NOTCH1	0.9901573	-0.01427	0.8823775	2654	3238	2947	2219	3151	3382
NOTCH4	0.375	-1.415037	0.4970888	1	2	5	1	2	0
AK1	1.6440678	0.7172698	0.1514125	12	29	18	31	30	36
AK2	1.033224	0.0471531	0.341483	5692	6165	7045	6014	7202	6314
AK3	0.9496434	-0.074542	0.8717894	1658	1271	1698	1266	1659	1469
AK4	0.2727273	-1.874469	0.3216921	4	7	0	2	1	0
AK5	0	#NUM!	0.6840298	0	0	2	0	0	0
GPATCH2L	0.927118	-0.109175	0.7093459	1058	694	951	622	913	971
WASH4P	2.1428571	1.0995357	0.4641166	2	5	0	10	5	0
AK6	1.2897527	0.3670944	0.0141156	420	359	636	635	650	540
AK7	1.3406897	0.4229753	0.0154242	258	226	241	263	388	321
AK8	0.5333333	-0.906891	0.6124847	5	0	10	4	1	3
AK9	1.3583333	0.4418376	0.2285631	48	41	31	47	55	61
BAG6	0.9228228	-0.115874	0.6301097	5771	6645	7564	5801	6655	5982
DSCR3	1.0382311	0.0541277	0.3249058	1729	1738	2209	1771	2182	1940
BAG4	1.012792	0.0183379	0.6594891	640	421	737	504	724	593
DSCR4	1.4576271	0.5436217	0.1166686	43	35	40	63	64	45
BAG5	0.894314	-0.161147	0.4186664	2668	2225	3074	1781	2655	2689
AC095057.2	0	#NUM!	0.1851375	8	0	0	0	0	0
BAG2	1.3828073	0.4676001	0.000651	461	402	626	636	775	648
AC095057.3	1.125	0.169925	0.8005411	6	4	6	8	4	6
BAG3	1.13046	0.1769099	0.0544388	2521	2963	3147	3044	3508	3205
BAG1	1.0599224	0.0839586	0.2817111	1947	2356	2656	2499	2665	2212
DYNLT3P1	#DIV/0!	#DIV/0!	0.8030358	0	0	0	0	0	1
DSCR8	1.1770833	0.2352165	0.2171386	127	229	220	232	229	217
DSCR9	1	0	0.9729238	2	5	0	0	1	6
FN3K	1.3103448	0.3899465	0.0776307	149	144	171	269	188	151
WASH3P	0.781293	-0.356064	0.1017438	407	480	567	389	419	328

AC100756.2	0	#NUM!	0.824326	1	0	0	0	0	0
RCAN1	0.9675243	-0.04763	0.9805553	754	744	873	634	836	824
AC100756.3	1	0	0.9890559	0	0	1	0	1	0
RCAN2	0.2857143	-1.807355	0.4301611	1	3	3	0	0	2
RCAN3	1.0225131	0.0321193	0.5820652	604	614	692	527	701	725
SDAD1P1	1.6783217	0.7470193	0.0157687	43	34	66	72	87	81
XAF1	8.5714286	3.0995357	0.0006221	4	1	2	11	25	24
SDAD1P2	#DIV/0!	#DIV/0!	0.8030358	0	0	0	1	0	0
ALK	#DIV/0!	#DIV/0!	0.6099294	0	0	0	2	0	0
AC104232.1	0	#NUM!	0.824326	0	1	0	0	0	0
WASH6P	0.7846154	-0.349942	0.2047436	176	202	272	170	168	172
AMH	1	0	0.8152354	69	64	73	93	60	53
AMN	3.4	1.7655347	0.2009215	0	2	3	3	3	11
AMT	0.0740741	-3.754888	0.0152325	12	8	7	2	0	0
WASH5P	1.2	0.2630344	0.7953732	2	1	7	1	3	8
MIR2116	0.25	-2	0.4634206	0	0	12	3	0	0
ANG	1.15	0.2016339	0.7601202	10	5	5	14	4	5
LUZP1	0.7739362	-0.369714	0.0307608	2464	2472	2584	1349	2122	2349
LUZP2	#DIV/0!	#DIV/0!	0.8030358	0	0	0	0	0	1
AC022240.1	0.6666667	-0.584963	0.8392586	0	3	0	0	0	2
ENOX2	0.9049479	-0.144093	0.6429268	241	227	300	189	254	252
MGAT5B	0.9274194	-0.108706	0.8157985	83	89	76	52	74	104
BAK1	1.0178571	0.0255351	0.5625665	494	627	727	625	648	608
FNIP1	0.9103532	-0.135502	0.5928636	1601	1039	1465	850	1426	1461
FNIP2	0.967509	-0.047653	0.9453106	1473	1072	1333	822	1349	1581
SPAG9	1.041353	0.0584592	0.4870838	3561	2606	3796	2499	3911	3965
SPAG7	1.2413187	0.3118735	0.0310228	614	821	840	1014	1026	784
SPAG8	0.25	-2	0.1937394	7	2	11	0	5	0
SPAG5	0.9247997	-0.112787	0.6611829	1941	1825	2351	1742	1978	1937
SPAG6	5.3333333	2.4150375	0.1380718	3	0	0	4	4	8
SPAG4	1.3212121	0.4018621	0.0382773	223	223	214	329	314	229
WASH7P	0.6946108	-0.525723	0.2214327	66	50	51	38	36	42
ENOX1	1.8695652	0.9027028	0.031013	20	19	30	43	37	49
MGAT4C	#DIV/0!	#DIV/0!	0.6482162	0	0	0	0	1	1
MGAT4D	0	#NUM!	0.824326	0	0	1	0	0	0
APC	1.2539683	0.3265008	0.0667474	556	302	591	447	695	675
MGAT4A	1.7443469	0.802687	9.70E-08	3494	2394	3222	3838	6015	6038
MGAT4B	0.8384636	-0.25418	0.1597385	5166	6099	6960	5257	5360	4664
SPAG1	0.7990033	-0.323727	0.2548017	178	187	237	168	177	136
APP	0.9770517	-0.033493	0.8919761	14508	16213	17605	13030	16996	17191
ABCC5-AS1	1	0	0.9890559	0	0	1	0	1	0
TP53TG3D	0.9183673	-0.122857	0.9183588	24	7	18	14	27	4
SPARC	1.134694	0.1823033	0.0334685	7415	8538	9475	9015	10345	9493
MIR203A	0	#NUM!	0.080407	0	5	6	0	0	0
B2M	1.1692237	0.225551	0.0086329	22424	25231	28540	27518	32047	29524
AQR	0.948937	-0.075616	0.8203863	1883	1476	1909	1195	1932	1872
DSTNP1	0.7037037	-0.50696	0.5949773	12	8	7	4	6	9
DSTNP2	1.3142857	0.3942789	0.367007	26	18	26	29	21	42
MIF4GD	1.0297158	0.0422462	0.5807653	203	238	333	295	294	208
INPP4A	1.0112471	0.0161356	0.6467848	998	934	1091	812	1067	1178
INPP4B	1.2838346	0.3604593	0.0967262	223	110	199	189	260	234
ARC	#DIV/0!	#DIV/0!	0.3877895	0	0	0	2	2	0
AP001351.1	0.6938776	-0.527247	0.5795027	22	4	23	4	7	23
AL034550.1	0	#NUM!	0.5337954	0	3	0	0	0	0

AL034550.2	0	#NUM!	0.6840298	0	0	2	0	0	0
AC092167.1	1	0	0.9253413	12	10	19	9	14	18
ARX	0.6153846	-0.70044	0.6078882	6	7	0	2	4	2
STC1	1.1541632	0.2068473	0.1813784	398	346	469	314	527	559
AL669942.1	2.25	1.169925	0.5207703	0	1	3	2	7	0
INPP5B	1.1015145	0.1394885	0.1493951	758	782	903	769	941	981
INPP5A	1.0462963	0.0652915	0.5219424	194	207	247	210	218	250
SPAST	1.1050452	0.1441054	0.3293393	1019	658	979	651	1149	1135
INPP5F	1.3000773	0.3785974	0.0193412	483	334	476	418	651	612
BICD1P1	#DIV/0!	#DIV/0!	0.5509701	0	0	0	0	3	0
INPP5D	0.5359952	-0.899708	5.37E-07	471	584	598	246	333	307
STC2	1.4010065	0.4864636	8.08E-07	6075	6615	7579	8983	10249	9165
INPP5E	0.9515789	-0.071605	0.9429368	604	564	732	561	658	589
INPP5J	0.6741573	-0.568843	0.3718798	26	25	38	21	12	27
INPP5K	0.8994389	-0.152903	0.5507233	649	768	900	677	747	660
ASL	0.9273743	-0.108776	0.8090598	356	431	466	407	406	349
BANP	0.8912978	-0.166021	0.468405	915	1081	1325	931	1041	988
AC024937.1	0.875	-0.192645	0.9023994	5	3	0	0	0	7
AC024937.2	4.5	2.169925	0.1898257	0	4	0	5	12	1
STAM	1.1150366	0.1570911	0.1795364	744	662	915	638	1037	913
SPART	0.9438429	-0.083381	0.8476308	2800	2023	3119	2051	2827	2618
STAR	1	0	0.9890559	0	1	0	0	1	0
STAM-AS1	1.1496063	0.2011399	0.5534313	58	15	54	43	51	52
SPATS2L	1.2271622	0.295326	0.0017883	1786	1727	2245	2179	2536	2351
ATM	0.8586475	-0.219862	0.4484058	676	367	761	351	634	564
g in cell cycle a	#VALUE!	#VALUE!	5634|nucleu	5|hsa04210|h	ıce|Human	ıne/threonine kinase			
AL109837.2	0	#NUM!	0.5781912	0	0	3	0	0	0
BAP1	0.8455647	-0.242013	0.1307153	2595	3042	3111	2319	2642	2436
ATR	1.0783172	0.1087816	0.4111734	606	344	595	434	642	590
HINT1	0.932185	-0.101312	0.8367472	3091	3717	4222	3701	3656	2925
HINT2	0.9044503	-0.144887	0.7251032	171	284	309	220	242	229
AUH	0.9528921	-0.069615	0.9745579	532	427	718	523	592	483
HINT3	0.9549458	-0.066509	0.9078767	1067	796	1089	761	1091	967
AL450992.1	0.5	-1	0.4735277	15	0	5	0	6	4
AL450992.2	#DIV/0!	#DIV/0!	0.4737294	0	0	0	0	4	0
AC119044.1	0.8	-0.321928	0.7396786	9	9	7	0	11	9
SPAM1	3.5	1.8073549	0.3963792	0	0	2	4	3	0
AC128687.2	0.3333333	-1.584963	0.669913	3	0	0	0	0	1
DZANK1	1.2366412	0.306427	0.4023059	32	51	48	35	46	81
AC006130.2	0	#NUM!	0.5781912	0	0	3	0	0	0
AC006130.1	#DIV/0!	#DIV/0!	0.6411933	0	0	0	0	0	2
BATF	1.152381	0.2046177	0.6280942	7	53	45	51	49	21
AXL	0.8854962	-0.175442	0.787575	39	34	58	32	45	39
MIR2052	#DIV/0!	#DIV/0!	0.6099294	0	0	0	2	0	0
WASHC4	1.1927409	0.2542807	0.1454129	1353	669	1174	916	1474	1422
WASHC5	0.8561426	-0.224077	0.3262334	519	427	625	324	574	447
DSCC1	1.064257	0.0898466	0.4220846	475	322	448	385	454	486
AL445222.1	4.375	2.129283	0.0285939	4	1	3	14	12	9
AL356273.1	#DIV/0!	#DIV/0!	0.1903651	0	0	0	7	0	0
AL356273.2	5.5	2.4594316	0.2248201	2	0	0	1	7	3
AL356273.3	0.9642857	-0.052467	0.9591143	11	5	12	10	4	13
WASHC1	1.058309	0.081761	0.5640311	82	117	144	123	111	129
WASHC3	1.0947119	0.1305513	0.240173	409	387	471	424	443	520
[APKAPK5-AS	0.9281122	-0.107629	0.8465059	578	491	642	583	495	510

ABCG8	0	#NUM!	0.824326	0	0	1	0	0	0
AC097358.1	0	#NUM!	0.5075476	0	0	4	0	0	0
ABCG5	0.25	-2	0.5682537	0	1	3	0	1	0
AC097358.2	0.2222222	-2.169925	0.1612397	4	6	8	3	0	1
DSCAS	0.1666667	-2.584963	0.2356003	5	0	7	0	0	2
AC080125.1	1.2857143	0.3625701	0.8548197	3	1	3	0	9	0
HDGFL1	0	#NUM!	0.0279239	5	4	4	0	0	0
ABCG4	0	#NUM!	0.4185478	3	0	1	0	0	0
HDGFL2	0.9897783	-0.014823	0.7175099	1844	2165	2350	2267	2161	1866
ABCG1	12.5	3.6438562	1.20E-05	0	0	8	26	40	34
HDGFL3	0.5242031	-0.931802	6.38E-05	282	218	347	110	165	169
ABCG2	1.3102638	0.3898573	8.15E-05	2532	2346	2780	2911	3553	3570
PLCXD1	1.1072946	0.147039	0.1182132	1579	1872	1964	1967	2115	1914
STK4	0.8964801	-0.157657	0.4420846	2741	2238	2720	1589	2584	2729
PLCXD2	0.566474	-0.819918	0.0166002	114	93	139	35	76	85
STK3	1.0353682	0.0501439	0.5993762	731	549	784	513	927	697
RNU6-810P	1.5	0.5849625	0.8637378	2	0	0	0	0	3
STIL	0.9953903	-0.006666	0.7748537	1170	804	1280	819	1198	1222
SLC2A9	0.3796296	-1.397335	0.0138432	49	30	29	11	20	10
ABCD4	0.9633838	-0.053817	0.9533821	1009	987	1172	987	1061	1004
ABCD2	0	#NUM!	0.4185478	3	0	1	0	0	0
ABCD3	1.0794483	0.1102941	0.2690294	1297	913	1415	1089	1529	1295
SLC2A1	1.4513162	0.5373619	3.42E-07	10657	12784	13635	16793	18895	18121
SLC2A3	0.9438769	-0.083329	0.7426962	41963	45615	48195	34321	46486	47346
SLC2A4	1.1012658	0.1391627	0.7248563	20	36	23	33	36	18
CHTF18	1.0036517	0.0052588	0.576648	1557	1607	2039	1852	1785	1585
SLC2A5	0.4166667	-1.263034	0.1757352	7	18	11	6	5	4
SLC2A6	0.4205607	-1.249614	0.0323908	29	37	41	20	17	8
SLC2A7	5	2.3219281	0.2415309	1	1	0	6	4	0
SLC2A8	1.0337079	0.0478285	0.484882	497	550	644	663	558	527
HEBP1	0.5888889	-0.763933	0.0002415	441	507	492	313	282	253
HEBP2	0.9225198	-0.116348	0.6715422	2709	2819	3171	2718	2773	2534
ABCD1	1.1308947	0.1774646	0.0746881	2007	2362	2438	2565	2728	2405
ABCC3	0.612069	-0.708234	0.2265133	22	44	50	30	27	14
ABCC4	0.7121375	-0.489772	0.0311314	317	264	350	165	226	272
ABCC1	0.7932385	-0.334173	0.017295	2089	2302	2501	1566	1995	1906
ABCC2	0.4398977	-1.18476	0.0002911	132	129	130	62	42	68
ABCC8	0	#NUM!	0.6385096	0	2	0	0	0	0
ABCC5	0.9174563	-0.124289	0.5530145	1525	1466	1758	1220	1567	1570
ABCC6	1.0560472	0.0786743	0.5969615	112	99	128	99	124	135
AC109449.1	1.2222222	0.2895066	0.8860251	0	9	0	2	4	5
ABCC9	1.5	0.5849625	0.7734914	0	0	2	3	0	0
TGFBR1	0.9554639	-0.065727	0.8849805	878	661	886	500	866	951
TGFBR2	1.3861712	0.4711055	0.00128	694	699	733	729	1045	1173
TGFBR3	0.8702113	-0.200562	0.3049562	3445	2931	3517	1889	3259	3461
DDX19A	0.902545	-0.147929	0.5796048	438	532	641	415	589	450
DDX19B	0.8737271	-0.194745	0.6470061	148	155	188	166	119	144
AC007114.1	1.5428571	0.6256045	0.2917107	7	12	16	29	13	12
AC007114.2	0	#NUM!	0.824326	0	1	0	0	0	0
AC099513.1	1.6	0.6780719	0.6395003	2	1	2	6	2	0
AC010343.1	1.1682243	0.2243173	0.3519164	48	71	95	102	71	77
AC010343.2	0	#NUM!	0.5387425	3	0	0	0	0	0
AC010343.3	0.6486486	-0.624491	0.3847641	15	26	33	19	9	20
STOM	0.9726402	-0.040022	0.9380318	472	454	536	397	508	517

ABCF2	0.7735738	-0.370389	0.0118553	1982	2359	2478	1524	1869	1882
ABCF3	0.828881	-0.270763	0.1003609	1540	1735	2078	1408	1551	1478
ABCF1	0.8720996	-0.197435	0.222824	6181	6978	8045	5775	6651	6066
MUTYH	0.9155944	-0.127219	0.7304061	558	653	732	641	628	510
STN1	1.1009174	0.1387063	0.3287202	271	227	265	231	320	289
BAD	1.1666667	0.2223924	0.4335703	32	64	72	59	73	64
AL592166.1	1.75	0.8073549	0.6450266	2	0	2	1	3	3
AC013472.3	0.4285714	-1.222392	0.5529749	0	5	2	1	1	1
AC013472.1	1.4	0.4854268	0.6419932	5	1	4	7	1	6
AC013472.2	#DIV/0!	#DIV/0!	0.3096126	0	0	0	5	0	0
CLK2P1	0.5	-1	0.8276497	1	0	1	0	0	1
BAX	0.8591796	-0.218968	0.336499	3167	3869	4397	3595	3404	2824
ABCE1	1.1541885	0.2068789	0.1101864	2373	1435	2483	1954	2822	2485
AC121758.1	1.5	0.5849625	0.8637378	2	0	0	0	0	3
BBX	1.0583544	0.0818228	0.5306387	3197	1721	2982	1836	3046	3479
WDR83OS	0.8992806	-0.153157	0.8205958	84	85	109	102	99	49
AL022326.1	6	2.5849625	0.1513379	0	0	2	3	5	4
BCR	0.9669795	-0.048443	0.9969099	3336	4163	4009	3314	3995	3819
VOPP1	0.9405963	-0.088352	0.849018	1323	1473	1564	1384	1352	1365
AL035456.1	0.7837838	-0.351472	0.7186396	16	6	15	10	11	8
TARSL2	1.0031596	0.0045511	0.7832045	232	154	247	176	251	208
FOXP1-IT1	0	#NUM!	0.824326	0	1	0	0	0	0
FAM187B2P	1	0	0.9890559	0	0	1	0	0	1
GTF2IRD2B	0.7777778	-0.36257	0.2914412	115	79	121	75	91	79
SRP72P2	0	#NUM!	0.6840298	0	0	2	0	0	0
SRP72P1	#DIV/0!	#DIV/0!	0.5371397	0	0	0	0	0	3
AC128714.1	0	#NUM!	0.6436235	2	0	0	0	0	0
SPAG5-AS1	0.2954545	-1.758992	0.1210135	13	2	29	6	1	6
AL604028.2	0.3157895	-1.662965	0.3441206	9	0	10	0	0	6
AL604028.1	1.1428571	0.1926451	0.7942136	3	8	10	6	13	5
STRC	0.952381	-0.070389	0.9366691	9	10	2	5	7	8
STRN	1.1238463	0.1684448	0.2593414	3565	2307	3446	2231	3964	4277
AC010478.1	0.1333333	-2.906891	0.1448103	0	9	6	0	0	2
BBC3	0.9414414	-0.087057	0.8748312	340	371	399	350	372	323
AC124254.1	1.6666667	0.7369656	0.7894956	0	2	1	0	5	0
STUM	0.8888889	-0.169925	0.9193835	5	0	4	0	7	1
FLJ20021	1.3903509	0.475449	0.0227069	132	169	155	225	187	222
AP000962.1	#DIV/0!	#DIV/0!	0.8030358	0	0	0	0	1	0
BGN	1.5154877	0.5997822	1.49E-06	625	652	886	965	1240	1073
AC006357.1	#DIV/0!	#DIV/0!	0.3109758	0	0	0	0	6	0
Z83843.1	0.770428	-0.376268	0.3554081	194	74	246	92	144	160
PAXBP1-AS1	0.9516129	-0.071553	0.9697156	29	18	15	25	24	10
STX6	1.0754315	0.1049156	0.2417831	1562	1504	1627	1377	1830	1840
STX5	0.837774	-0.255367	0.1301519	1296	1603	1755	1106	1443	1350
STX4	1.1036649	0.1423022	0.1972821	522	646	742	737	733	638
STX3	0.7592791	-0.397298	0.0022317	3183	3217	3865	2206	2784	2804
BID	1.2753623	0.3509072	0.4242703	20	13	36	20	36	32
STX2	1.0685112	0.095602	0.328939	527	458	533	494	553	575
SPDEF	0.7	-0.514573	0.8501554	2	4	4	7	0	0
BIK	1.8823529	0.9125372	0.2852634	5	6	6	6	10	16
AF165147.1	#DIV/0!	#DIV/0!	0.1903651	0	0	0	7	0	0
AL158152.2	0.5454545	-0.874469	0.4401357	5	11	6	4	2	6
AL158152.1	1.0731707	0.1018796	0.8018688	12	18	11	24	12	8
FADS3	1.0240336	0.034263	0.4596949	2632	2771	3418	3154	3319	2560

FADS2	0.6806861	-0.554939	9.84E-05	1942	2243	2520	1433	1703	1428
FNTA	0.9930417	-0.010074	0.7859978	1186	658	1174	844	1093	1060
FADS6	#DIV/0!	#DIV/0!	0.8030358	0	0	0	0	0	1
FNTB	0.8901099	-0.167945	0.6531145	165	180	201	172	156	158
FADS1	0.6673077	-0.583576	3.45E-06	5401	5679	6080	3158	4165	4128
AC018992.1	1.5	0.5849625	0.8552295	1	1	0	0	0	3
STX8	0.815453	-0.294326	0.1594261	509	588	702	497	514	456
STX7	0.9773667	-0.033028	0.9119142	1790	1392	1899	1304	1937	1725
AC096644.4	0.5	-1	0.8289598	1	0	1	0	1	0
FO393419.3	0	#NUM!	0.5781912	0	0	3	0	0	0
AC007040.2	0.7142857	-0.485427	0.713086	5	4	5	2	7	1
AC007040.1	0.3333333	-1.584963	0.6851201	1	0	2	0	1	0
FO393419.1	0	#NUM!	0.6436235	2	0	0	0	0	0
BLM	1.049491	0.0696897	0.5405101	1474	692	1370	978	1374	1359
IREB2	0.9486661	-0.076028	0.8381633	1859	1309	1780	1090	1714	1890
AC090527.3	0.5	-1	0.5305538	5	1	16	1	0	10
FAM111A-DT	0.712766	-0.4885	0.4829963	32	14	48	26	23	18
BMF	0.8322996	-0.264825	0.0971384	1303	1431	1458	997	1245	1247
STYX	1.0867067	0.1199626	0.2202493	1321	1137	1371	1097	1583	1481
RGMB-AS1	1.5185185	0.6026645	0.4062383	8	10	9	16	20	5
AL354718.2	#DIV/0!	#DIV/0!	0.4501902	0	0	0	0	0	4
AL354718.1	0.6875	-0.540568	0.6578592	4	9	3	0	4	7
AC018752.1	4.5	2.169925	0.2822373	1	1	0	6	3	0
LRRC37BP1	2	1	0.522404	0	1	4	3	0	7
PPWD1	1.0974589	0.1341669	0.2338426	1230	759	1356	1063	1340	1268
BOC	0.75	-0.415037	0.8564162	0	4	0	1	2	0
SPDL1	1.2436765	0.3146112	0.0232236	2508	1503	2789	2366	3277	2814
BOK	3.9411765	1.9786263	0.0064987	2	6	9	17	32	18
SLC36A1	0.8720009	-0.197599	0.2193038	3024	2976	3336	2143	2964	3034
SLC36A3	0	#NUM!	0.1670145	3	1	3	0	0	0
SLC36A2	7	2.8073549	0.285525	1	0	0	0	2	5
AL589642.1	#DIV/0!	#DIV/0!	0.4501902	0	0	0	0	0	4
RABGGTB	1.1361714	0.1841805	0.1072247	1319	930	1298	1155	1531	1344
RABGGTA	0.8886319	-0.170342	0.5317364	785	1036	1205	919	950	820
AC110285.6	0.8947368	-0.160465	0.9615732	8	12	18	17	5	12
AC110285.7	5	2.3219281	0.1739275	3	0	0	2	5	8
C1D	0.9427839	-0.085001	0.8095893	773	697	872	599	810	799
AC110285.2	0.52657	-0.925303	0.0268432	60	72	75	46	31	32
AC110285.1	0.625	-0.678072	0.7317369	2	3	3	0	0	5
AC110285.3	4	2	0.5760976	1	0	0	0	4	0
CYP51A1P1	0.3333333	-1.584963	0.6822731	0	1	2	0	1	0
C1S	0.431677	-1.211976	0.0001842	114	105	103	43	49	47
C1R	1.031746	0.0450879	0.8648301	29	17	17	26	24	15
CYP51A1P3	#DIV/0!	#DIV/0!	0.6554062	0	0	0	0	2	0
CYP51A1P2	0.25	-2	0.3729542	3	1	4	0	0	2
SEPT7-AS1	1.2521008	0.3243508	0.4197201	47	26	46	29	78	42
SLC36A4	1.1253012	0.1703112	0.2022812	427	327	491	366	564	471
TSPOAP1	0.6530612	-0.61471	0.4377012	7	30	12	8	9	15
AL365295.1	0.7204969	-0.472936	0.2688902	47	60	54	23	51	42
AC016355.1	0.7956989	-0.329705	0.6040577	31	25	37	26	34	14
CDRT15	0.75	-0.415037	0.7302678	14	3	15	10	5	9
LGALSL	1.4400294	0.5260983	0.0001706	377	458	524	611	720	626
AC013714.1	0	#NUM!	0.824326	0	0	1	0	0	0
C4B	0.7291667	-0.455679	0.644921	16	14	18	21	6	8

LGALS4	0	#NUM!	0.075725	1	4	5	0	0	0
C4A	1.1578947	0.2115041	0.6808182	7	11	20	11	9	24
LGALS3	1.5342466	0.6175304	0.1375395	23	17	33	45	33	34
LGALS2	1.4615385	0.5474878	0.4701332	6	5	15	4	14	20
HMGN2P46	1.2857143	0.3625701	0.8332482	2	3	2	0	5	4
LGALS1	0.8896817	-0.168639	0.6064806	901	1375	1368	1216	1170	856
MCIDAS	0.9056047	-0.143047	0.8056728	102	80	157	63	99	145
FOXD3-AS1	0.7107143	-0.492658	0.2291016	87	85	108	81	80	38
AC093909.1	#DIV/0!	#DIV/0!	0.5338029	0	0	0	0	1	2
BSG	0.9496464	-0.074538	0.9581407	15583	18861	20706	18299	18174	15900
HMGN2P41	1	0	0.9959663	28	28	18	13	33	28
AC002480.4	3.25	1.7004397	0.2738166	0	0	4	3	3	7
HMGN2P40	#DIV/0!	#DIV/0!	0.6554062	0	0	0	0	2	0
AC002480.3	0.962963	-0.054448	0.9552927	6	7	14	9	6	11
AC002480.2	0.8161765	-0.293047	0.500319	87	88	97	93	75	54
LGALS9	0.6941176	-0.526748	0.0664587	168	142	200	119	144	91
BSN	0.4878049	-1.035624	0.0571508	62	37	24	12	20	28
LGALS8	0.9759834	-0.035071	0.9120855	839	677	899	645	898	814
INHBA-AS1	0	#NUM!	0.5378194	0	2	1	0	0	0
AC067942.2	0	#NUM!	0.6410557	1	1	0	0	0	0
AC002480.1	#DIV/0!	#DIV/0!	0.6411933	0	0	0	0	0	2
AC067942.1	0	#NUM!	0.2904576	0	1	5	0	0	0
AC093909.6	1.4255319	0.5115003	0.3790262	24	9	14	22	31	14
AC108718.1	1.4347826	0.5208322	0.3665664	14	18	14	17	33	16
BTD	0.8596939	-0.218105	0.4380395	280	278	226	224	209	241
CFDP1	1.1559774	0.2091132	0.0445169	1238	1082	1578	1507	1630	1369
SUB1	1.0436777	0.0616763	0.2923094	5498	4664	6620	5366	6619	5530
BTK	0.2972973	-1.750022	0.1473506	21	2	14	6	0	5
AL355297.3	5	2.3219281	0.4650112	0	0	1	0	0	5
AL355297.2	0	#NUM!	0.5075476	0	0	4	0	0	0
AL355297.4	0.7826087	-0.353637	0.811796	4	2	17	2	10	6
NKAIN2	1.7707006	0.8243203	0.0173343	60	52	45	54	106	118
NKAIN1	0.4285714	-1.222392	0.388035	9	3	2	2	3	1
NKAIN4	0.3761261	-1.410712	1.31E-06	127	132	185	52	62	53
RACGAP1	1.0329682	0.0467959	0.3752171	5762	5902	6687	5297	7033	6626
BBS2	0.8482247	-0.237482	0.201056	819	788	956	706	765	703
BBS1	0.3684211	-1.440573	0.15898	8	12	18	0	5	9
XBP1	0.9821256	-0.026021	0.7785135	4588	5194	5771	4837	5540	4898
HSD17B1P1	0.6	-0.736966	0.0277198	90	83	122	51	58	68
SPDYC	1.1835132	0.2430759	0.0943879	324	281	414	428	443	335
SUCO	1.1848107	0.2446565	0.1081149	1774	1043	1831	1300	2159	2048
SPDYA	0.6666667	-0.584963	0.4550052	23	9	13	6	11	13
AC011411.1	0.3333333	-1.584963	0.5011613	1	2	3	0	2	0
C8B	#DIV/0!	#DIV/0!	0.3831122	0	0	0	0	5	0
C8G	0.8040541	-0.314636	0.6014003	53	48	47	59	30	30
SUFU	1.1326189	0.1796625	0.1500097	419	535	539	540	618	533
BBS9	1.0136116	0.019505	0.6803135	361	290	451	281	411	425
AC027313.1	#DIV/0!	#DIV/0!	0.6411933	0	0	0	0	0	2
BBS7	0.9445235	-0.082341	0.8597843	243	204	256	178	269	217
BBS5	0	#NUM!	0.824326	1	0	0	0	0	0
BBS4	0.9036861	-0.146106	0.6485444	284	221	336	207	283	270
PCYT1A	0.9017463	-0.149206	0.4135935	1786	1739	2144	1457	1879	1776
EIPR1-IT1	0.625	-0.678072	0.7217028	0	5	3	1	1	3
AL031736.2	3.25	1.7004397	0.1247429	1	5	2	10	12	4

AL031736.1	1.0526316	0.0740006	0.7881264	36	16	43	27	50	23
TAX1BP1	1.1842193	0.2439363	0.069319	3444	1976	3591	3068	4135	3468
TAX1BP3	0.7142857	-0.485427	0.6559477	6	7	15	6	8	6
AC010967.2	0	#NUM!	0.824326	0	0	1	0	0	0
AC114495.2	0.7142857	-0.485427	0.6225004	8	9	11	4	6	10
AC022634.1	#DIV/0!	#DIV/0!	0.8030358	0	0	0	0	1	0
RBM22P2	0	#NUM!	0.4374838	0	4	0	0	0	0
ENPP2	0.4411765	-1.180572	0.2737034	3	15	16	6	8	1
ENPP1	0.1976744	-2.338802	0.0011569	34	23	29	1	9	7
ENPP4	0.7333333	-0.447459	0.620305	13	8	9	3	9	10
ENPP3	#DIV/0!	#DIV/0!	0.4121728	0	0	0	0	1	3
ENPP6	1	0	0.9792606	0	1	2	0	1	2
ENPP5	0.909416	-0.136988	0.6764971	264	236	339	176	287	300
AC010331.1	0.4333333	-1.206451	0.2381211	7	10	13	5	3	5
AL158151.1	1.8823529	0.9125372	0.0946135	22	20	9	36	36	24
AL122013.1	0	#NUM!	0.824326	1	0	0	0	0	0
AL158151.4	0.6428571	-0.63743	0.7179955	1	6	7	6	1	2
DAPP1	1.4057217	0.491311	0.0027404	273	213	283	333	355	393
BSPRY	1.1144307	0.1563069	0.1901489	487	635	652	556	721	700
RBM22P5	5	2.3219281	0.2253355	1	0	1	9	1	0
DIAPH2-AS1	2	1	0.8147319	1	0	0	0	0	2
CA1	#DIV/0!	#DIV/0!	0.6554062	0	0	0	0	2	0
CA3	2	1	0.7741804	1	0	0	2	0	0
CA2	1.1863986	0.2465887	0.0103969	1380	1279	1767	1573	1907	1771
AC003956.1	0.8333333	-0.263034	0.8621488	4	2	0	0	5	0
CA9	0	#NUM!	0.0962574	1	4	4	0	0	0
SUN3	1.3666008	0.4505919	0.0002311	667	578	779	890	942	934
SUN2	0.8424444	-0.247347	0.1381232	1539	1726	1857	1386	1501	1428
SUN1	0.9489533	-0.075591	0.8258364	1878	2022	2310	1592	2238	2063
ATP6AP1	0.8764351	-0.190281	0.2850353	7854	8919	10055	7742	8257	7514
ATP6AP2	0.9192807	-0.121423	0.5770032	1753	1446	1806	1261	1839	1501
CAD	0.9005434	-0.151132	0.411557	4262	5067	5210	3721	4582	4790
CH17-340M24.	1.0454545	0.0641303	0.5809783	130	130	158	177	135	125
CAT	0.9572298	-0.063063	0.9318285	1935	1872	2272	1672	2193	1954
SHANK3	0.8172232	-0.291198	0.3140656	195	147	227	130	198	137
SHANK2	0.7268777	-0.460215	0.0116187	1056	1008	1158	522	884	936
SHANK1	1.6	0.6780719	0.7455052	4	0	1	0	6	2
CBL	0.7891118	-0.341698	0.0644888	3483	3588	3546	1853	2970	3555
AL139353.2	0.8490566	-0.236067	0.8367294	25	4	24	14	12	19
AL139353.1	8	3	0.1761296	1	0	0	4	2	2
AL139353.3	#DIV/0!	#DIV/0!	0.1506188	0	0	0	8	0	0
SV2C	0.4285714	-1.222392	0.5828138	6	1	0	0	0	3
SV2B	0.2142857	-2.222392	0.3215508	0	0	14	0	1	2
CBS	2.5172786	1.3318649	7.93E-14	259	366	301	794	802	735
SV2A	1.4	0.4854268	0.0370578	82	99	109	148	125	133
TBX20	0.8453608	-0.242361	0.5670224	70	71	53	19	74	71
AL359851.1	0.4444444	-1.169925	0.5333557	3	2	4	0	4	0
AC110774.1	#DIV/0!	#DIV/0!	0.2758481	0	0	0	4	0	1
SUOX	0.8802784	-0.183968	0.4011452	621	769	765	577	634	686
TBX19	0.5986395	-0.740241	0.1195317	57	30	60	26	34	28
AC009093.6	0	#NUM!	0.5781912	0	0	3	0	0	0
TBX18	0.7037794	-0.506805	0.0057532	665	566	727	334	516	528
TBX15	0.3333333	-1.584963	0.669913	3	0	0	0	0	1
CCS	1.0695476	0.0970007	0.3592813	349	490	642	577	527	480

CD4	1.1111111	0.1520031	0.8472563	4	3	2	10	0	0
AL136368.1	2	1	0.7016593	0	2	0	2	0	2
CD6	1.5	0.5849625	0.562676	5	0	9	4	10	7
CD5	0.3333333	-1.584963	0.524883	0	2	4	0	1	1
TBX10	0.5584416	-0.840522	0.2502932	28	27	22	21	4	18
CD7	0.5882353	-0.765535	0.6453312	7	0	10	5	2	3
CD9	1.058303	0.0817527	0.3388415	546	642	733	691	697	645
CDA	0	#NUM!	0.6436235	2	0	0	0	0	0
AC004449.1	1.3	0.3785116	0.6881668	2	2	6	6	1	6
AC004053.1	0.2	-2.321928	0.4521254	0	5	0	0	0	1
AC008753.2	0.3584906	-1.479993	0.1195034	3	16	34	7	7	5
AC008753.1	1.203125	0.2667865	0.5403172	20	23	21	26	29	22
AC069120.1	8	3	0.2317398	1	0	0	1	0	7
AC008753.3	#DIV/0!	#DIV/0!	0.2808669	0	0	0	3	0	2
CLEC11A	0.8787062	-0.186547	0.7374037	233	235	274	311	203	138
CEL	0.4517766	-1.146318	0.0116017	41	87	69	18	29	42
NUP93	0.8311632	-0.266796	0.0607439	4072	4543	5132	3475	4086	3865
BCAN	1.1136364	0.1552782	0.785397	13	20	11	12	12	25
BCAM	0.8928145	-0.163568	0.4436586	5726	7043	7355	6062	6407	5498
HECW2	1.008316	0.0119479	0.7963126	332	281	349	217	359	394
HECW1	0.8367347	-0.257158	0.8228878	13	6	30	10	18	13
NUP98	0.8403958	-0.250859	0.1223581	5890	6113	6593	3907	5544	6177
AC069360.1	1.25	0.3219281	0.78062	3	0	5	2	3	5
CFD	1.0526055	0.0739648	0.4542514	518	746	751	771	768	582
AC027801.5	1.6666667	0.7369656	0.745689	1	1	1	0	0	5
AC027801.4	1.2989691	0.3773671	0.3475169	39	25	33	35	29	62
AC027801.1	1.8571429	0.8930848	0.3998633	8	4	2	3	8	15
CFP	0.5531915	-0.854149	0.2641692	15	20	12	9	8	9
AC027801.3	0.25	-2	0.1897014	7	8	1	1	3	0
AC027801.2	0.6111111	-0.710493	0.6080314	3	4	11	0	5	6
NUP85	0.9107098	-0.134937	0.5291371	2390	2756	2884	2318	2515	2480
NUP88	1.0862302	0.1193299	0.1322677	2128	1882	2635	2356	2566	2296
CGA	2.0058659	1.0042251	2.41E-16	4419	5022	6243	10545	11599	9316
CGN	1.0903226	0.124755	0.2139789	970	1279	1316	1128	1383	1376
TMEM225B	0	#NUM!	0.5172237	1	2	0	0	0	0
EM110-MUST	7.3333333	2.8744691	0.041195	1	2	0	8	4	10
STON1	0.9254587	-0.111759	0.750418	310	226	336	174	328	305
CHM	1.0643121	0.0899212	0.5776353	758	472	667	400	783	836
STON2	0.6800643	-0.556257	0.047813	225	144	253	136	130	157
CIC	0.9472779	-0.07814	0.8229166	1011	1264	1215	882	1149	1275
EDARADD	1.4057508	0.4913409	0.0405468	86	106	121	103	164	173
CIT	0.8270402	-0.273971	0.120285	1541	1623	1762	978	1477	1619
SLC9B1P4	0	#NUM!	0.5075476	0	0	4	0	0	0
PCOTH	0	#NUM!	0.6840298	0	0	2	0	0	0
PLIN4	0.8697674	-0.201298	0.5255117	187	189	269	138	191	232
PLIN3	1.0319415	0.0453612	0.4572185	1523	1847	1827	1782	1863	1718
AL133477.1	0.7	-0.514573	0.7845879	4	2	4	4	1	2
PLIN2	1.2887966	0.3660246	5.63E-05	15037	16166	18621	19780	23402	21031
PLIN1	1.1666667	0.2223924	0.8673089	7	5	0	8	6	0
RCCD1	0.8295082	-0.269672	0.1466338	1106	1312	1547	1082	1188	1019
PLIN5	2.4878049	1.3148733	0.0124754	10	16	15	32	45	25
MIR4307HG	1	0	0.9329691	3	0	2	4	0	1
CKB	1.5166297	0.6008689	0.004273	140	150	161	260	238	186
BCHE	0.45	-1.152003	0.2533577	10	4	26	5	5	8

HIPK4	1.8181818	0.8624965	0.4157095	1	2	8	3	6	11
CKM	0.6277372	-0.671767	0.1484063	40	52	45	28	35	23
AL590632.1	0	#NUM!	0.824326	0	1	0	0	0	0
HIPK1	0.7669032	-0.382884	0.040704	1813	1737	1804	896	1497	1713
HIPK3	1.0088195	0.0126681	0.8105158	1087	757	1104	582	1149	1243
HIPK2	0.6661392	-0.586104	0.0178657	195	213	224	103	148	170
FOSB	2.1940928	1.1336246	6.85E-05	57	110	70	173	182	165
CLC	7.3333333	2.8744691	0.0462554	2	0	1	6	13	3
TMEM229B	0.7068966	-0.500429	0.4848925	20	17	21	10	10	21
CLU	0.687692	-0.540166	0.0019221	1398	1659	1826	1209	1093	1056
OIP5	1.0440751	0.0622255	0.45821	399	482	503	455	503	487
AC117386.2	1.2857143	0.3625701	0.7208002	0	8	6	7	8	3
MTNR1B	0.2675159	-1.902303	9.27E-05	48	51	58	14	12	16
CNP	1.1106706	0.151431	0.0959829	1726	2056	2272	2064	2484	2176
EDRF1-AS1	2.75	1.4594316	0.4260061	0	2	2	0	1	10
TCEANC	0.8125	-0.29956	0.6201611	53	17	42	23	46	22
BCL9	0.9142924	-0.129272	0.5703232	1257	1522	1468	872	1453	1558
UBOX5	0.8139535	-0.296982	0.2574216	180	232	233	164	192	169
BCL6	1.4739336	0.5596716	0.0011101	203	191	239	255	368	310
CPD	0.686754	-0.542135	0.0062566	1333	930	1172	533	862	964
BCL3	0.7674419	-0.381871	0.3695097	77	102	79	84	71	43
BCL2	0.6781915	-0.560235	0.0553277	129	124	123	70	105	80
CPE	1.25	0.3219281	0.8810394	2	1	1	0	0	5
Z83844.2	1.8571429	0.8930848	0.4846359	2	1	4	2	5	6
CPM	1.8729216	0.9052905	1.51E-13	838	712	976	1341	1799	1591
CPO	0.75	-0.415037	0.9161536	0	0	4	0	3	0
CPQ	0.3310811	-1.594744	0.0012108	58	38	52	17	19	13
CPZ	0.8682505	-0.203817	0.5486732	160	122	181	73	182	147
OIT3	5	2.3219281	0.3869708	1	0	0	3	0	2
DPRXP4	1.0689655	0.0962153	0.7838358	6	24	28	19	23	20
CR2	0.5652174	-0.823122	0.5186872	2	8	13	0	5	8
DPRXP2	0	#NUM!	0.824326	0	0	1	0	0	0
DPRXP1	0	#NUM!	0.824326	0	1	0	0	0	0
HMGN2P19	0	#NUM!	0.824326	0	1	0	0	0	0
HMGN2P17	#DIV/0!	#DIV/0!	0.8030358	0	0	0	1	0	0
OR6C66P	0	#NUM!	0.5075476	0	0	4	0	0	0
SAPCD2	1.2570978	0.3300969	0.0029779	889	1041	1240	1193	1420	1372
HMGN2P11	1.3333333	0.4150375	0.8695235	1	0	2	0	4	0
SAPCD1	1	0	0.8831434	6	1	17	8	6	10
CRH	#DIV/0!	#DIV/0!	0.8030358	0	0	0	0	1	0
XCL2	0.6296296	-0.667425	0.4826013	10	10	7	2	11	4
XCL1	1.893617	0.9211446	0.1255744	12	13	22	14	56	19
CRK	1.0010397	0.0014993	0.7303453	2972	2516	3168	2175	3241	3249
SELENOKP1	2.5	1.3219281	0.6099065	0	0	2	0	0	5
C1RL-AS1	1	0	0.9890559	1	0	0	1	0	0
SELENOKP3	#DIV/0!	#DIV/0!	0.8030358	0	0	0	1	0	0
HMGN2P28	0	#NUM!	0.4217812	0	0	5	0	0	0
STOX2	0.5619048	-0.831602	0.108424	37	33	35	16	20	23
SVBP	1	0	0.805896	86	69	105	91	93	76
CSK	0.9284797	-0.107058	0.8314853	628	742	965	731	754	683
BCO2	1.1875	0.2479275	0.799197	7	5	4	4	1	14
BCO1	0.6754967	-0.565979	0.2208906	46	40	65	21	38	43
STOX1	1.1969697	0.2593866	0.3256038	72	64	62	82	71	84
SLC35A2	0.7328496	-0.448411	0.0207895	804	1096	1132	776	748	698

SLC35A1	0.9625	-0.055142	0.8883642	148	137	195	196	128	138
SLC35A4	0.9244868	-0.113275	0.6385827	4284	5217	5503	4304	4989	4578
SLC35A3	1.0356883	0.0505898	0.5978352	544	342	487	383	512	527
AC003070.2	0	#NUM!	0.0859015	3	5	1	0	0	0
AC003070.1	0	#NUM!	0.6436235	2	0	0	0	0	0
CPPED1	0.8494289	-0.235435	0.2702822	590	614	722	559	575	502
KBTBD11	2.7857143	1.4780473	0.0285226	5	17	6	18	26	34
ASXL1	0.8786216	-0.186686	0.2701346	3123	3490	3776	2373	3342	3413
AC007041.1	2.5	1.3219281	0.3964265	3	0	3	0	5	10
ASXL3	1.4230769	0.5090136	0.3183952	33	30	15	24	33	54
CTH	1.9800532	0.9855392	3.43E-08	241	282	229	452	541	496
ASXL2	0.943719	-0.083571	0.7852281	1593	1345	1504	846	1458	1888
HMGN2P31	#DIV/0!	#DIV/0!	0.6411933	0	0	0	0	0	2
BCOR	1.0439727	0.062084	0.5342872	846	878	914	681	907	1166
DSG2-AS1	0.3428571	-1.544321	0.12107	8	9	18	2	7	3
SLC35B3	0.8706349	-0.19986	0.4548015	432	346	482	366	351	380
AC009831.1	0.5227273	-0.93587	0.3249427	20	3	21	9	7	7
SLC35B2	0.7972598	-0.326878	0.0447601	1521	1784	2096	1386	1529	1391
AC018754.1	0.5714286	-0.807355	0.7044573	0	3	4	0	2	2
SLC35B4	0.9041778	-0.145322	0.4798603	892	895	822	616	901	842
SLC35B1	0.8512132	-0.232408	0.1735612	1482	1690	1815	1354	1526	1365
ARFIP2	0.8262585	-0.275335	0.1041015	1527	1757	2040	1420	1606	1373
ARFIP1	1.2391747	0.3093796	0.0131236	1327	1013	1586	1280	1737	1848
SLC35A5	1.0803279	0.1114692	0.3708685	683	492	655	512	647	818
AC068282.1	1.0223881	0.0319429	0.7662929	48	20	66	50	45	42
TANGO6	0.7184874	-0.476965	0.026025	304	423	463	227	327	301
AL355306.2	#DIV/0!	#DIV/0!	0.6099294	0	0	0	2	0	0
SLC35C2	0.9908999	-0.013189	0.7302671	1377	1727	1841	1655	1756	1489
TANGO2	0.8157543	-0.293793	0.2984346	213	243	293	209	178	224
SLC35C1	0.9228753	-0.115792	0.6875472	738	984	949	777	926	762
SLC35D1	0.8132628	-0.298206	0.1356741	858	802	798	461	719	819
SLC35D3	0.3333333	-1.584963	0.7290749	0	0	3	0	0	1
SLC35D2	0.75	-0.415037	0.7666073	3	4	9	0	7	5
RNA5-8SN1	0	#NUM!	0.4117982	0	3	1	0	0	0
RNA5-8SN5	#DIV/0!	#DIV/0!	0.8030358	0	0	0	0	1	0
RNA5-8SN4	0	#NUM!	0.5378194	0	2	1	0	0	0
XCR1	0.6666667	-0.584963	0.4904657	13	12	17	10	7	11
SLC35E2	0.4672897	-1.097611	0.0498028	48	37	22	18	17	15
SLC35E1	0.829932	-0.268935	0.0886202	1373	1593	1738	1151	1428	1325
SLC35E4	0.5906433	-0.759641	0.0813882	45	65	61	27	49	25
SLC35E3	0.8726186	-0.196577	0.3291917	757	863	1057	698	828	810
AC131025.1	0	#NUM!	0.6436235	2	0	0	0	0	0
AC022144.1	2.3	1.2016339	0.2654982	2	6	2	12	2	9
SLC35F6	0.7714423	-0.37437	0.0128745	1260	1424	1490	978	1153	1089
SLC35F3	0.030303	-5.044394	0.0018946	15	14	4	0	0	1
CAB39L	1.576087	0.6563471	0.0001222	266	210	260	315	444	401
SLC35F2	0.8366347	-0.25733	0.1011423	1616	1463	1818	1265	1426	1406
SLC35F5	1.1184501	0.1615009	0.2284925	1756	1476	1749	1239	2173	2159
SLC35F4	0	#NUM!	0.824326	0	1	0	0	0	0
SLC35F1	#DIV/0!	#DIV/0!	0.3768753	0	0	0	2	0	2
TTC39C-AS1	0.3333333	-1.584963	0.5619338	1	0	5	1	1	0
AC146949.1	0.3333333	-1.584963	0.5161379	2	1	3	0	0	2
SLC35G6	#DIV/0!	#DIV/0!	0.6411933	0	0	0	0	0	2
SLC35G2	2.3	1.2016339	0.2363041	2	2	6	12	2	9

SLC35G1	0.8855989	-0.175275	0.4429818	503	425	558	362	476	478
MPRIPP1	0.8333333	-0.263034	0.9148909	6	0	0	2	0	3
AC068042.1	#DIV/0!	#DIV/0!	0.1160693	0	0	0	0	11	0
RGPD6	0.5833333	-0.777608	0.5825148	3	6	3	0	5	2
RGPD5	0.6470588	-0.628031	0.2809293	37	18	47	14	32	20
RGPD8	0.8146341	-0.295776	0.5268163	81	45	79	52	49	66
RGPD2	1.2119403	0.2773186	0.2348191	126	70	139	106	168	132
RGPD1	1.2330097	0.3021842	0.2083814	112	65	132	116	162	103
RGPD4	1	0	0.9940505	1	1	1	0	2	1
BEGAIN	0.6956522	-0.523562	0.7353758	6	5	12	10	6	0
RGPD3	0.9583333	-0.061401	0.9825936	27	5	16	19	14	13
AC090371.1	2	1	0.7276346	1	1	0	0	3	1
TCEAL8	0.8359926	-0.258438	0.0554162	4617	4821	5604	3693	4607	4275
TCEAL7	0.7206463	-0.472637	0.0017624	904	851	1030	603	747	657
TCEAL5	#DIV/0!	#DIV/0!	0.8030358	0	0	0	0	1	0
TCEAL4	1.2230508	0.2904844	0.0054622	4670	4071	6009	6162	6331	5547
TCEAL3	0.8	-0.321928	0.7581165	7	8	5	0	8	8
AC090371.2	#DIV/0!	#DIV/0!	0.5070017	0	0	0	3	0	0
TCEAL1	1.0930065	0.128302	0.2182193	411	384	592	476	536	504
AC080038.2	1.5	0.5849625	0.7067061	0	0	4	6	0	0
TCEAL9	1.1131855	0.1546941	0.1249715	6900	4832	7979	6794	8482	6666
SVIL	0.8235816	-0.280017	0.1197526	1121	1139	1124	688	1009	1090
AC079385.1	0.5	-1	0.4224694	10	12	0	0	6	5
SVIP	0.9986816	-0.001903	0.7055203	507	408	602	440	547	528
SPCS3	1.1363636	0.1844246	0.0950875	3669	2734	3871	2985	4430	4260
SPCS2	0.8623422	-0.213668	0.1606745	2112	2125	2577	1680	2176	2020
SPCS1	0.9843112	-0.022814	0.7384569	1767	1999	2353	2104	2108	1811
MT-RNR2	1.2017858	0.2651798	0.0069771	95339	75806	108498	92539	126587	116945
MT-RNR1	0.9917937	-0.011888	0.6317786	12263	11718	15501	12296	14419	12443
NUPL2	1.0115108	0.0165117	0.6371649	964	757	1059	775	1117	920
AC112191.2	0.2857143	-1.807355	0.4744769	0	2	5	0	2	0
APOOP1	0	#NUM!	0.6840298	0	0	2	0	0	0
CLEC18C	0	#NUM!	0.5172237	1	2	0	0	0	0
CLEC18B	0	#NUM!	0.824326	0	0	1	0	0	0
CLEC18A	1.4	0.4854268	0.6236702	12	0	8	8	17	3
FPGT	1.1535039	0.2060229	0.2380553	329	265	305	236	418	383
FPGS	1.0832655	0.1153869	0.2441825	1050	1285	1352	1399	1437	1158
AL136221.1	1.4782609	0.5639009	0.5053197	7	12	4	7	12	15
NUPR1	0.6211348	-0.687022	8.70E-05	1822	2207	2633	1502	1447	1189
AC008514.2	1.4583333	0.5443205	0.4745739	3	8	13	10	19	6
AC008514.1	0	#NUM!	0.0290801	6	0	10	0	0	0
CLEC16A	0.820752	-0.284982	0.0678906	1050	1094	1287	806	1044	966
CLEC17A	#DIV/0!	#DIV/0!	0.6554062	0	0	0	0	2	0
DAP	1.0747386	0.1039858	0.1782448	2940	3487	3996	3673	3872	3657
DAO	0.9117647	-0.133267	0.9449138	7	13	14	12	9	10
SKIV2L	0.9972426	-0.003984	0.6725664	950	1060	1254	986	1200	1069
AC009102.2	0.6363636	-0.652077	0.7677402	2	0	9	0	1	6
AC009102.1	0.2222222	-2.169925	0.0784664	16	10	1	2	2	2
DBI	1.0598951	0.0839215	0.2640868	3178	3490	4201	4110	4030	3380
DBH	0	#NUM!	0.2762615	2	0	4	0	0	0
AL139354.1	#DIV/0!	#DIV/0!	0.8030358	0	0	0	0	0	1
DBP	1.2424623	0.3132021	0.0770855	213	284	299	391	316	282
DBT	1.2602453	0.3337046	0.0476211	1364	797	1182	990	1691	1532
STPG1	1.0889894	0.12299	0.3477817	215	203	245	216	266	240

STPG3	0.7727273	-0.371969	0.7386181	4	9	9	4	8	5
STPG2	1.4166667	0.5025003	0.6071665	4	3	5	5	3	9
SVOP	1.5	0.5849625	0.1608018	18	36	30	42	48	36
DCC	0	#NUM!	0.0841869	6	4	0	0	0	0
STPG4	0.5789474	-0.788496	0.4195875	15	5	18	10	6	6
STPG4	0.5789474	-0.788496	0.4195875	15	5	18	10	6	6
TAF9BP1	0	#NUM!	0.4117982	0	3	1	0	0	0
DCK	0.9161345	-0.126369	0.625904	836	642	871	622	793	737
DCN	0.6666667	-0.584963	0.8749464	0	0	6	0	0	4
ZAP70	2.2777778	1.187627	0.2197906	3	2	13	1	15	25
DCT	0	#NUM!	0.824326	0	0	1	0	0	0
DCX	0.5	-1	0.6443193	3	3	0	1	0	2
DNASE2	1.0192388	0.0274921	0.5167904	723	722	946	894	815	728
DNASE1	0.8181818	-0.289507	0.1377791	539	570	717	462	496	536
DDC	0.4310345	-1.214125	0.0293675	37	49	30	13	28	9
CLEC12A	0	#NUM!	0.6436235	2	0	0	0	0	0
DDO	#DIV/0!	#DIV/0!	0.0103756	0	0	0	8	6	2
DDN	0.4	-1.321928	0.653698	0	1	4	1	0	1
MTRF1LP1	0	#NUM!	0.3608108	0	1	4	0	0	0
DDT	0.7826087	-0.353637	0.7209556	27	14	51	36	14	22
AC005277.2	#DIV/0!	#DIV/0!	0.8030358	0	0	0	0	1	0
AL117209.1	0.9655172	-0.050626	0.962774	13	8	8	4	11	13
DEK	1.1401821	0.1892643	0.1714752	7029	3813	6728	5235	7980	6818
AC010332.1	0.3333333	-1.584963	0.6622061	0	3	0	0	0	1
DES	1.0201342	0.028759	0.8401803	37	65	47	56	57	39
AP000867.2	0	#NUM!	0.1234296	0	5	4	0	0	0
LIF-AS1	#DIV/0!	#DIV/0!	0.2546293	0	0	0	1	5	0
RPL31P49	3	1.5849625	0.4887137	0	0	2	2	4	0
AL356133.1	#DIV/0!	#DIV/0!	0.5509701	0	0	0	0	3	0
KLF7-IT1	0	#NUM!	0.6385096	0	2	0	0	0	0
AC006355.2	#DIV/0!	#DIV/0!	0.4737294	0	0	0	0	4	0
PHC1P1	0.9072	-0.140507	0.6349531	206	231	188	156	211	200
DHH	0	#NUM!	0.824326	1	0	0	0	0	0
AL845472.1	#DIV/0!	#DIV/0!	0.1219606	0	0	0	0	6	3
FPR1	1	0	0.9890559	1	0	0	0	1	0
AC092338.2	0.5	-1	0.8652929	0	0	2	0	1	0
AC092338.1	0.6666667	-0.584963	0.7801659	4	0	2	0	2	2
RNU6-414P	2.5	1.3219281	0.6099065	0	0	2	0	0	5
AC008752.1	2	1	0.651797	0	1	1	3	1	0
AC008752.3	1.3333333	0.4150375	0.7811765	1	5	3	0	3	9
AC008752.2	#DIV/0!	#DIV/0!	0.6099294	0	0	0	2	0	0
RPL31P52	0.4285714	-1.222392	0.6089514	4	0	3	0	0	3
RPL31P61	1	0	0.9621224	1	0	1	1	0	1
DCAF13P3	1.1666667	0.2223924	0.9023039	4	2	0	2	0	5
RPLP1	1.0019377	0.0027928	0.6206743	15813	20258	22761	22440	20147	16359
RPLP0	0.9234943	-0.114825	0.6933325	49081	61292	63235	54685	56905	48736
DCAF13P1	5	2.3219281	0.385839	0	0	1	4	0	1
RPLP2	0.9784736	-0.031395	0.7602664	12192	15512	18286	17508	15009	12483
AC006115.1	0.6875	-0.540568	0.7732284	2	1	13	4	3	4
AC053481.2	0	#NUM!	0.6385096	0	2	0	0	0	0
BDH2	0.7598784	-0.396159	0.242428	103	88	138	59	113	78
BDH1	0.8913043	-0.16601	0.9073296	15	9	22	14	14	13
CDC42EP5	0.7954545	-0.330149	0.6141245	24	30	34	27	29	14
CDC42EP4	1.1330828	0.1802533	0.0678579	2247	2700	2770	2893	2952	2899

CDC42EP3	1.280917	0.3571769	0.0003195	1602	1496	1875	1981	2223	2166
CDC42EP2	0.7865854	-0.346325	0.4803135	35	61	68	40	40	49
CDC42EP1	1.3949561	0.4802197	0.0001379	1220	1465	1756	2151	2154	1890
DLD	0.8898652	-0.168341	0.4371804	3668	2476	3871	2464	3425	3023
CCNDBP1	0.9192582	-0.121458	0.6028215	1070	1147	1288	983	1195	1044
DMD	0.8309305	-0.2672	0.1594921	783	677	829	490	668	744
CSMD3	#DIV/0!	#DIV/0!	0.5509701	0	0	0	0	3	0
CSMD2	0	#NUM!	0.6410557	1	1	0	0	0	0
CSMD1	4.2222222	2.0780025	1.37E-06	20	16	18	44	78	106
RPL31P12	#DIV/0!	#DIV/0!	0.8030358	0	0	0	0	1	0
AC009487.2	0.8789474	-0.186151	0.657759	166	74	140	72	120	142
TTLL7	0.8502994	-0.233957	0.6747458	68	31	68	9	75	58
TTLL6	0.5	-1	0.7186765	0	2	2	0	2	0
TTLL5	0.896978	-0.156855	0.5136382	948	804	1160	669	911	1032
TTLL4	0.9831969	-0.024448	0.834366	3579	3936	4090	3300	4146	3964
TTLL3	0.8862344	-0.17424	0.6026276	262	227	390	225	282	272
TTLL2	1.4814815	0.5670406	0.3918602	6	8	13	22	12	6
TTLL1	1.2531646	0.3255759	0.4124616	22	29	28	42	22	35
TTLL9	0.3333333	-1.584963	0.446284	7	2	0	0	0	3
AL137782.1	0	#NUM!	0.2919745	1	0	5	0	0	0
AC233723.2	0.8688525	-0.202817	0.6957355	62	49	72	51	52	56
AC233723.1	2.5	1.3219281	0.328039	0	0	6	6	5	4
DPT	0	#NUM!	0.824326	0	0	1	0	0	0
AC048380.2	5	2.3219281	0.4840007	1	0	0	0	5	0
DR1	1.0172009	0.0246047	0.5412513	1664	1632	1820	1470	1861	1873
BDNF	1.5	0.5849625	0.7889207	0	0	2	1	0	2
AC114498.1	0	#NUM!	0.255947	4	0	2	0	0	0
DSE	1.0297619	0.0423108	0.5588131	1559	1267	1542	1138	1727	1633
SPG11	0.8974359	-0.156119	0.5181255	1541	1063	1569	920	1341	1484
DSP	0.967173	-0.048154	0.9390207	25505	21013	25892	15041	25736	29256
AL355304.1	0	#NUM!	0.824326	0	1	0	0	0	0
DST	1.1846206	0.2444251	0.1019096	1074	682	1196	828	1367	1302
CALM2P3	0.6666667	-0.584963	0.850543	1	1	1	1	0	1
CALM2P2	1.893617	0.9211446	0.1462479	26	8	13	12	45	32
AC124016.1	1.5	0.5849625	0.7419304	2	0	2	1	3	2
AC092828.1	1.1666667	0.2223924	0.8878263	2	0	4	0	5	2
BDP1	1.0942749	0.1299753	0.421374	1144	618	1155	658	1223	1311
DTL	0.7569832	-0.401667	0.0056793	2016	1972	2098	1266	1629	1712
AC124016.2	0.3333333	-1.584963	0.4625982	0	9	0	1	0	2
IFNA20P	0	#NUM!	0.824326	0	0	1	0	0	0
HSD17B14	0.9285714	-0.106915	0.9257607	38	51	51	47	35	48
HSD17B13	2.5	1.3219281	0.5527374	0	0	2	3	2	0
HSD17B12	0.7637854	-0.388761	0.0719179	456	413	473	224	386	415
HSD17B11	1.1611665	0.2155749	0.0439439	808	853	945	862	1147	1017
HSD17B10	0.9420655	-0.086101	0.8712757	3641	4426	4637	4150	4229	3589
SLITRK2	1	0	0.9890559	1	0	0	0	0	1
SLITRK1	#DIV/0!	#DIV/0!	0.0006151	0	0	0	10	5	12
SLITRK3	1.6666667	0.7369656	0.6931533	0	0	3	3	2	0
SLITRK6	0.7	-0.514573	0.7808235	1	3	6	2	2	3
SLITRK5	0.625	-0.678072	0.15664	37	41	50	25	32	23
AC094103.1	0	#NUM!	0.824326	1	0	0	0	0	0
LINGO1	0.2702703	-1.887525	0.0158138	16	19	39	10	3	7
DUT	0.9894407	-0.015315	0.716098	1734	2013	2314	2096	2162	1739
AC104135.1	1.04	0.0565835	0.9540545	1	14	10	3	17	6

MDGA2	1.0537634	0.075551	0.8037983	41	22	30	25	22	51
MDGA1	0.5	-1	0.5740288	9	0	1	2	2	1
SPG21	0.8787206	-0.186524	0.2746112	3000	3304	3607	2774	3072	2863
RETREG3	0.8684211	-0.203533	0.2679581	1019	1149	1404	885	1133	1084
AC027315.1	0	#NUM!	0.6436235	2	0	0	0	0	0
SLC34A3	1.5	0.5849625	0.8224159	0	4	0	0	1	5
SLC34A2	2.1434978	1.0999669	6.30E-06	75	68	80	132	177	169
SLC34A1	0.25	-2	0.6199202	0	0	4	0	1	0
OR5E1P	0.0909091	-3.459432	0.1023991	5	4	2	0	0	1
DXO	1.2340967	0.3034554	0.1391227	136	97	160	177	175	133
AL441992.1	1.2012987	0.2645949	0.1769469	127	165	170	162	230	163
EPHA10	0.5	-1	0.457858	4	6	4	2	3	2
SWI5	1.2686567	0.3433017	0.0141222	462	520	559	682	705	568
DYM	0.9391481	-0.090575	0.772817	969	901	1088	805	1012	961
AL359853.2	1.8	0.8479969	0.5932613	1	3	1	4	0	5
AL445238.1	0	#NUM!	0.1983952	5	0	2	0	0	0
HDHD5-AS1	0	#NUM!	0.6604275	0	1	1	0	0	0
AC244452.4	0.9166667	-0.125531	0.9950064	8	2	14	9	5	8
AC244452.1	#DIV/0!	#DIV/0!	0.6482162	0	0	0	0	1	1
RETREG2	0.9923789	-0.011037	0.735291	1952	2680	2716	2374	2587	2331
RETREG1	1.1297787	0.1760402	0.2892967	354	270	370	258	493	372
PCSK1	1	0	0.9804516	1	1	0	0	1	1
SLC7A14	#DIV/0!	#DIV/0!	0.6554062	0	0	0	0	2	0
PCSK9	0.6666667	-0.584963	0.8465573	2	0	7	4	0	2
PCSK7	0.7561587	-0.403239	0.0310935	740	807	848	479	585	747
SLC7A11	1.5649123	0.6460818	0.0046898	192	162	216	181	304	407
PCSK6	0.6749915	-0.567059	0.0001749	1672	1965	2249	1293	1310	1370
SLC7A10	#DIV/0!	#DIV/0!	0.2887809	0	0	0	0	0	6
PCSK5	1.0741777	0.1032327	0.291609	1001	847	1010	846	1216	1008
PCSK4	0.9772727	-0.033167	0.9812347	21	32	35	20	37	29
VDAC1P13	3.3333333	1.7369656	0.4171158	0	0	3	0	0	10
GNGT1	1.5	0.5849625	0.8409011	0	4	0	0	5	1
VDAC1P11	0	#NUM!	0.4460243	4	0	0	0	0	0
AC093166.4	#DIV/0!	#DIV/0!	0.4501902	0	0	0	0	0	4
AC116655.1	0	#NUM!	0.6840298	0	0	2	0	0	0
L3HYPDH	0.7810384	-0.356535	0.2244567	136	141	166	106	109	131
G2E3	1.2560373	0.3288793	0.0509697	1416	857	1371	1019	1898	1660
AC018755.3	0.826087	-0.275634	0.6965731	9	31	6	4	17	17
AC091114.1	4.3333333	2.1154772	0.2257846	1	1	1	0	5	8
AC018755.1	#DIV/0!	#DIV/0!	0.6411933	0	0	0	0	0	2
IGHMBP2	0.8892521	-0.169336	0.4826673	740	779	928	741	771	664
AC018755.4	0	#NUM!	0.4460243	4	0	0	0	0	0
AC087477.6	0	#NUM!	0.5378194	0	2	1	0	0	0
AC087477.2	0.5185185	-0.947533	0.4063863	7	3	17	3	7	4
AF274856.1	1.1428571	0.1926451	0.9079379	3	1	3	1	7	0
AL359999.1	1	0	0.9890559	1	0	0	0	1	0
LBX2-AS1	0.5955056	-0.747813	0.0387108	76	160	120	78	69	65
RN7SL317P	7	2.8073549	0.2588739	1	0	0	1	5	1
AC008659.1	#DIV/0!	#DIV/0!	0.8030358	0	0	0	0	1	0
ATP11AUN	#DIV/0!	#DIV/0!	0.1506188	0	0	0	8	0	0
AC003958.1	0.9285714	-0.106915	0.9415842	8	1	5	0	5	8
RN7SL316P	1	0	0.9890559	0	0	1	0	1	0
IGHVIII-76-1	1	0	0.9890559	1	0	0	1	0	0
PLCG1-AS1	5	2.3219281	0.4275856	1	0	0	0	2	3

RNU6-407P	0.6666667	-0.584963	0.8537226	2	0	1	0	0	2
ZNF337-AS1	0.9268293	-0.109624	0.9059149	22	7	12	6	14	18
AC010969.2	1.0461812	0.0651327	0.5439348	185	127	251	194	219	176
AC010969.1	0.5	-1	0.7019715	2	2	0	0	0	2
EBP	0.9306856	-0.103634	0.824467	1814	2305	2503	2205	2066	1892
CASP1P1	0	#NUM!	0.6385096	0	2	0	0	0	0
CASP1P2	0.28125	-1.830075	0.0926197	13	7	12	5	3	1
AC134407.3	0.2222222	-2.169925	0.1624547	7	9	2	0	0	4
ECD	1.2502256	0.3221884	0.003618	1152	924	1249	1252	1495	1410
AC103881.1	0	#NUM!	0.6436235	2	0	0	0	0	0
FERP1	#DIV/0!	#DIV/0!	0.6251462	0	0	0	1	0	1
AL357060.2	0.6779661	-0.560715	0.4610761	19	10	30	8	22	10
AL357060.1	#DIV/0!	#DIV/0!	0.8030358	0	0	0	0	1	0
STT3A	0.7917263	-0.336926	0.0133987	9164	10464	10419	7066	8806	7917
AC134407.2	4	2	0.5051795	0	0	1	2	2	0
AC134407.1	0.6666667	-0.584963	0.856923	1	0	2	0	2	0
DIMT1P1	0	#NUM!	0.6840298	0	0	2	0	0	0
STT3B	1.0209641	0.0299321	0.5625588	8106	6599	8239	6049	8798	8578
EDA	0.8019802	-0.318361	0.4113786	97	91	115	76	62	105
AL022328.4	1.4050633	0.4906351	0.233121	31	19	29	32	38	41
EED	0.9816216	-0.026761	0.7850373	1576	1289	1760	1413	1640	1487
AL022328.3	0.2424242	-2.044394	0.0641124	9	11	13	0	6	2
AL022328.2	1.3409091	0.4232114	0.4406985	18	10	16	16	24	19
AL022328.1	0.8648649	-0.209453	0.8029946	9	14	14	2	17	13
DARS2	0.7133161	-0.487387	0.0002793	2532	2654	2917	1581	2254	1945
AC010333.2	#DIV/0!	#DIV/0!	0.1700664	0	0	0	0	9	0
AC010333.1	0	#NUM!	0.4460243	4	0	0	0	0	0
ACVR1B	0.6630571	-0.592795	0.0005527	718	834	849	407	579	606
ACVR1C	0.7692308	-0.378512	0.5936831	20	17	15	4	16	20
EFS	0.9300112	-0.10468	0.7131027	559	614	613	503	623	535
AC243960.3	0.6	-0.736966	0.7793612	5	0	0	2	1	0
AC243960.2	1.5789474	0.6589631	0.1822765	9	20	28	28	23	39
APOPT1	1	0	0.7173831	149	149	248	194	189	163
MDH1B	1.1587302	0.2125446	0.6267364	18	22	23	21	32	20
EGF	0.5185185	-0.947533	0.4527083	3	5	19	4	2	8
SWT1	1.262	0.3357119	0.0852176	180	126	194	162	245	224
ACVR2B	1.0544574	0.0765009	0.4116095	1751	1523	1684	1339	1909	1980
ACVR2A	1.1177249	0.1605651	0.2769237	257	221	278	223	288	334
INAFM2	0.8329975	-0.263616	0.1244837	1199	1377	1394	1060	1151	1096
INAFM1	1.1358696	0.1837972	0.3296042	148	194	210	298	168	161
RNU6-405P	0	#NUM!	0.6385096	0	2	0	0	0	0
AC022872.1	2.6	1.3785116	0.1179768	2	9	4	20	13	6
EHF	3.5	1.8073549	0.2169893	2	1	1	3	7	4
RPL30	0.9887794	-0.016279	0.7461798	872	1340	1442	1335	1216	1062
RPL32	1.0227239	0.0324167	0.4676407	15465	17991	20320	19603	19577	15818
TMEM200B	0	#NUM!	0.5781912	0	0	3	0	0	0
RPL31	1.1147059	0.1566632	0.0731708	17309	19219	24053	22855	24336	20339
TMEM200A	0.9722222	-0.040642	0.991632	11	10	15	3	12	20
ZNF561-AS1	0.790795	-0.338624	0.423156	66	70	103	68	65	56
RPL34	1.2612013	0.3347985	0.0023026	4671	5452	6393	7100	7492	6238
TMEM200C	0.8251748	-0.277228	0.3977108	123	151	155	122	137	95
ASB9P1	1.3333333	0.4150375	0.8269684	2	4	0	1	2	5
GPR173	0	#NUM!	0.2919745	1	0	5	0	0	0
GPR176	#DIV/0!	#DIV/0!	0.4121728	0	0	0	0	1	3

MKI67P1	0	#NUM!	0.824326	1	0	0	0	0	0
RN7SL333P	0.0714286	-3.807355	0.0190654	9	2	17	0	2	0
GPR171	1	0	0.9621224	1	0	1	1	0	1
RPL36	1.0685986	0.0957201	0.2917755	4635	5715	6662	7056	6188	4935
RPL35	1.1386	0.187261	0.0808376	23626	31273	34430	35821	36224	29665
RPL38	1.0183824	0.0262793	0.4929678	6055	6813	8076	7908	7339	6082
RPL37	1.0071623	0.0102962	0.5750784	16730	20185	23401	21751	21912	17085
ANKS1A	0.7993658	-0.323072	0.058159	1388	1710	1632	978	1369	1434
RPL39	0.9507813	-0.072815	0.9415375	1102	1149	1589	1075	1443	1133
ANKS1B	0.1666667	-2.584963	0.0933369	3	12	3	0	1	2
RPL21	1.1446565	0.1949147	0.0548381	817	815	988	964	1092	943
RPL23	0.9882567	-0.017042	0.6804231	27253	30322	36266	30807	33286	28646
RPL22	1.097231	0.1338673	0.1111018	12279	14391	16125	15598	16884	14474
AARSD1	1.0285714	0.040642	0.8642495	8	9	18	10	11	15
AC010335.1	0.4545455	-1.137504	0.4961569	7	1	3	1	4	0
GPR162	0.2692308	-1.893085	0.1035453	8	7	11	3	2	2
AC008897.1	0.8235294	-0.280108	0.8789691	5	0	12	0	9	5
AC008897.3	0.75	-0.415037	0.891637	5	0	3	5	1	0
AC008897.2	0	#NUM!	0.3473206	0	5	0	0	0	0
GPR161	0.9545455	-0.067114	0.9972687	4	10	8	9	10	2
GPR160	1.1932692	0.2549196	0.0613874	359	313	368	396	428	417
RPL24	1.0417619	0.0590256	0.3358445	17817	19620	22929	22189	22320	18378
RPL27	1.0156562	0.0224121	0.5162224	19078	23189	24927	24316	23471	20459
RPL26	1.1468927	0.1977304	0.1879253	266	255	364	380	374	261
RPL29	0.9812268	-0.027341	0.779686	20480	26034	27581	25219	25286	22199
RPL28	1.0537796	0.0755731	0.3098981	21347	26380	30165	29736	28618	23727
TAGLN2P1	4.6666667	2.2223924	0.1609581	2	0	1	4	7	3
KATNBL1P3	#DIV/0!	#DIV/0!	0.5070017	0	0	0	3	0	0
KATNBL1P6	0.6206897	-0.688056	0.527044	15	4	10	5	2	11
RPL41	1.0149829	0.0214554	0.5432362	4662	5519	5904	5503	6066	4757
SLC16A12	1.7826087	0.83399	0.3341839	13	6	4	5	14	22
PRRC2C	0.9284341	-0.107129	0.6710566	10529	8668	10454	5908	9824	11797
SLC16A10	1.0402576	0.0569409	0.491519	408	382	452	373	491	428
PRRC2B	0.7931017	-0.334422	0.0728803	11537	13808	13099	6651	10879	12960
RPL3L	1.2692308	0.3439544	0.5820603	5	8	13	13	7	13
SLC16A14	1.5	0.5849625	0.79451	0	3	1	0	3	3
PRRC2A	0.8233453	-0.28043	0.0678367	9229	11427	10982	7198	9305	9546
SLC16A13	#DIV/0!	#DIV/0!	0.1181139	0	0	0	0	2	7
PHBP13	#DIV/0!	#DIV/0!	0.3831122	0	0	0	0	5	0
AC006450.2	#DIV/0!	#DIV/0!	0.249172	0	0	0	0	1	5
AC006450.1	3	1.5849625	0.6889342	0	1	0	0	3	0
GPR180	1.0063442	0.0091238	0.8099735	461	363	437	301	525	443
PHBP17	3	1.5849625	0.6206259	1	0	0	3	0	0
GPR183	0.5	-1	0.6934823	2	2	0	1	1	0
GPR182	0.8095238	-0.304855	0.7509821	9	11	1	0	9	8
PHBP19	1	0	0.9890559	0	0	1	0	0	1
RNU6-425P	0	#NUM!	0.5387425	3	0	0	0	0	0
AC099535.1	#DIV/0!	#DIV/0!	0.6317974	0	0	0	1	1	0
PHBP12	1.5	0.5849625	0.8898407	0	2	0	0	3	0
SPEF2	4.75	2.2479275	0.1164829	2	2	0	3	7	9
ELL	0.9956722	-0.006257	0.7274581	985	1209	1272	1050	1284	1117
SPEF1	0.6285714	-0.669851	0.4331971	9	14	12	4	10	8
ELN	0.2972973	-1.750022	0.1059171	9	15	13	6	0	5
AP001062.2	0.4285714	-1.222392	0.6304391	0	2	5	0	0	3

AP001062.1	2	1	0.1915181	7	7	8	23	17	4
AC006450.3	3.5	1.8073549	0.4287787	0	0	2	0	3	4
CHIAP3	#DIV/0!	#DIV/0!	0.5070017	0	0	0	3	0	0
PCOLCE-AS1	1.4285714	0.5145732	0.7180083	2	5	0	7	1	2
GPR132	0.0909091	-3.459432	0.1265306	1	3	7	0	0	1
KRT8P15	3	1.5849625	0.4815018	2	0	0	2	1	3
KRT8P14	#DIV/0!	#DIV/0!	0.2088383	0	0	0	5	0	1
ZAR1L	1.2352941	0.3048546	0.6985888	4	2	11	5	10	6
GPR137	0.9759632	-0.035101	0.8092541	833	922	1074	1024	905	832
EMB	3	1.5849625	0.3995947	0	0	3	3	0	6
KRT8P13	1.0909091	0.1255309	0.8732745	3	3	5	3	3	6
EMD	0.9445983	-0.082227	0.9296932	1998	2592	2991	2461	2643	2057
KRT8P12	0.9439252	-0.083256	0.9324059	146	186	203	173	186	146
BX649632.1	1.0833333	0.1154772	0.8606448	4	2	6	4	2	7
KRT8P11	16	4	0.0361804	0	0	1	2	5	9
GPR135	0.6330935	-0.659509	0.202823	46	31	62	17	26	45
KRT8P10	1.5454545	0.6280312	0.2462089	14	14	16	19	29	20
EN2	0	#NUM!	0.824326	1	0	0	0	0	0
AFTPH	1.0558155	0.0783578	0.381806	1069	756	1167	866	1219	1074
KRT8P26	0	#NUM!	0.5781912	0	0	3	0	0	0
KRT8P29	1	0	0.9756864	2	0	0	0	2	0
KRT8P20	#DIV/0!	#DIV/0!	0.8030358	0	0	0	1	0	0
KRT8P24	0	#NUM!	0.824326	1	0	0	0	0	0
KRT8P22	0	#NUM!	0.5172237	1	2	0	0	0	0
ENG	1.617191	0.6934901	1.97E-10	3204	3542	4411	5817	6499	5727
PCYOX1	0.9182004	-0.123119	0.5603327	3247	3058	3475	2131	3311	3538
RPL10	0.979404	-0.030024	0.774867	17306	20909	23156	22136	20601	17370
RPL12	1.1336864	0.1810217	0.061655	22850	26507	30883	31596	32726	26645
RPL11	1.2230619	0.2904974	0.0080503	20639	23399	27552	31427	30259	25873
KRT8P39	0.8717949	-0.197939	0.8221104	12	12	15	3	13	18
GPR151	#DIV/0!	#DIV/0!	0.6099294	0	0	0	2	0	0
KRT8P37	0.6666667	-0.584963	0.7348581	0	7	2	1	1	4
AC097724.1	#DIV/0!	#DIV/0!	0.6411933	0	0	0	0	0	2
GPR153	1.1292135	0.1753183	0.3841027	132	112	112	128	152	122
KRT8P36	1.4	0.4854268	0.8254658	1	2	2	0	7	0
GPR150	#DIV/0!	#DIV/0!	0.6099294	0	0	0	2	0	0
KRT8P31	#DIV/0!	#DIV/0!	0.8030358	0	0	0	0	1	0
RPL14	0.9853044	-0.021359	0.7155986	17377	18160	21555	18805	20330	17118
KRT8P30	1.75	0.8073549	0.4346207	1	7	4	13	6	2
RPL13	1.0182342	0.0260695	0.5166623	63841	80023	87898	89351	79697	66940
RPL15	0.9143671	-0.129155	0.5773911	29064	34773	37596	30368	33702	28677
GPR156	0.2884615	-1.793549	0.0554606	13	11	28	1	12	2
KRT8P35	0	#NUM!	0.6840298	0	0	2	0	0	0
RPL18	0.9869213	-0.018993	0.7238374	21368	26773	29084	27899	26127	22189
GPR155	0.8679245	-0.204358	0.5783716	110	87	121	69	104	103
RNU6-422P	#DIV/0!	#DIV/0!	0.5509701	0	0	0	0	3	0
RPL17	1.0533333	0.0749621	0.6692862	50	39	61	67	44	47
GPR158	1.3508772	0.4338965	0.0567759	170	102	241	160	277	256
KRT8P33	1.1680672	0.2241233	0.5162899	28	23	68	26	52	61
GPR157	1.2539573	0.3264883	0.0165172	405	480	568	501	718	603
KRT8P32	7.5	2.9068906	0.0765617	0	1	1	6	5	4
RPL19	1.0229245	0.0326996	0.4461284	26470	31383	35846	33659	33388	28800
F10	0.5238095	-0.932886	0.4283288	8	7	6	0	4	7
F12	1.1756757	0.2334901	0.5725731	23	31	20	42	24	21

AC006116.2	0	#NUM!	0.824326	0	0	1	0	0	0
AC006116.5	#DIV/0!	#DIV/0!	0.6099294	0	0	0	2	0	0
CERNA1	0.4375	-1.192645	0.3992171	2	8	6	0	6	1
NMNAT2	1.3992322	0.4846354	0.0141073	146	196	179	204	283	242
AC006116.8	0	#NUM!	0.824326	0	0	1	0	0	0
GPR141	0.75	-0.415037	0.8471659	1	3	0	1	1	1
KRT8P49	0.4	-1.321928	0.6927589	0	1	4	2	0	0
GPR143	0.739726	-0.434937	0.5947516	11	29	33	26	14	14
KRT8P48	3	1.5849625	0.6206259	1	0	0	3	0	0
AC073517.1	0.3333333	-1.584963	0.21298	9	3	9	2	4	1
NMNAT1	0.7543103	-0.40677	0.1067176	178	255	263	155	196	174
CERNA3	1	0	0.9890559	1	0	0	0	1	0
KRT8P42	0.5	-1	0.7290146	6	0	0	3	0	0
KRT8P41	0	#NUM!	0.6631647	1	0	1	0	0	0
KRT8P46	0.53125	-0.912537	0.3360527	12	9	11	3	4	10
RNU6-421P	#DIV/0!	#DIV/0!	0.6554062	0	0	0	0	2	0
KRT8P45	0.7076923	-0.498806	0.5941944	12	18	35	16	4	26
GPR146	1.6363636	0.7104934	0.0532632	37	40	33	45	63	72
EPO	2.6756757	1.4199033	0.0121639	17	5	15	23	37	39
EPX	0	#NUM!	0.6604275	0	1	1	0	0	0
AC106897.1	0.3333333	-1.584963	0.6715904	3	0	0	0	1	0
AC105942.1	1.0888889	0.1228567	0.7462928	29	21	40	18	53	27
AC233724.6	2	1	0.4995671	0	1	4	6	0	4
LRP1-AS	1.5	0.5849625	0.6856069	2	2	4	0	7	5
AC092431.2	0.6666667	-0.584963	0.8419932	1	1	1	1	1	0
AC092431.1	1	0	0.9890559	0	0	1	0	1	0
F2R	1.1164057	0.1588614	0.1916593	479	473	517	435	613	592
ERF	0.755799	-0.403926	0.0049543	1436	1513	1707	1091	1219	1209
GAPDHS	#DIV/0!	#DIV/0!	0.8030358	0	0	0	1	0	0
ERH	1.1258642	0.1710328	0.060252	3153	3424	3982	3902	4399	3587
ERG	0	#NUM!	0.824326	0	0	1	0	0	0
AC068675.1	0	#NUM!	0.5387425	3	0	0	0	0	0
ABHD4	0.7293651	-0.455287	0.0057119	757	866	897	580	663	595
ABHD3	0.8497706	-0.234855	0.3911745	309	255	308	245	303	193
GPR119	#DIV/0!	#DIV/0!	0.8030358	0	0	0	1	0	0
ABHD2	0.6724704	-0.572457	0.0014111	1356	1283	1324	616	948	1101
ABHD1	0.9230769	-0.115477	0.9552817	4	2	7	9	0	3
ABHD8	0.6436007	-0.635762	0.0006984	485	565	583	331	415	305
ABHD6	1.4153471	0.5011559	0.0001447	719	880	864	1140	1235	1111
ABHD5	1.0564065	0.0791651	0.2841661	1394	1246	1473	1287	1597	1461
STRA8	5	2.3219281	0.4650112	0	0	1	0	0	5
ESD	1.1724695	0.2295504	0.0178439	1794	1564	2214	1985	2461	2087
STRA6	1.030697	0.0436203	0.4351204	829	858	1082	964	1025	865
LAMC1-AS1	1.0967742	0.1332665	0.7789877	22	20	20	12	26	30
GPR108	0.9052378	-0.143631	0.5746633	837	1054	1011	873	880	874
TDRD12	1.0612245	0.0857299	0.8159672	22	6	21	15	21	16
AC024933.1	#DIV/0!	#DIV/0!	0.4737294	0	0	0	0	4	0
GPR107	0.9429938	-0.08468	0.7452376	4051	4398	4725	3322	4567	4534
SPERT	0.71875	-0.476438	0.4580012	17	28	19	7	20	19
TDRD15	0	#NUM!	0.824326	0	0	1	0	0	0
CALCOCO1	0.9390505	-0.090725	0.7582572	984	1128	1153	860	1079	1127
CALCOCO2	1.2202696	0.2871999	0.0051808	1971	1697	2193	1967	2650	2535
ADRA1D	#DIV/0!	#DIV/0!	0.4980481	0	0	0	2	1	0
RPL7A	1.1323016	0.1792582	0.0670333	47818	57541	60376	65012	65652	56998

PHBP21	#DIV/0!	#DIV/0!	0.8030358	0	0	0	0	0	1
PHBP20	0	#NUM!	0.824326	0	1	0	0	0	0
AL731571.1	1.6964286	0.7625007	0.2047063	20	5	31	11	35	49
PDCD6IP	0.8999224	-0.152128	0.4359457	4850	3853	5466	3516	4666	4569
AC083899.1	1.4417476	0.5278186	0.084191	94	48	64	102	114	81
ADRA2B	0.9155447	-0.127298	0.7368308	258	256	303	248	244	256
COASY	1.1397199	0.1886793	0.0518052	1699	1822	2262	2274	2252	2065
EVC	0.5	-1	0.8143938	2	0	0	1	0	0
AC108073.2	#DIV/0!	#DIV/0!	0.5509701	0	0	0	0	3	0
AC108073.1	#DIV/0!	#DIV/0!	0.242062	0	0	0	6	0	0
EVL	1.5212947	0.6052997	2.28E-07	1301	1261	1547	2090	2255	1906
AC104134.1	0.7607362	-0.394532	0.5150216	42	30	91	45	40	39
SFT2D2	1.0109125	0.0156581	0.5935951	1755	1554	2006	1440	2001	1932
SFT2D1	0.9662605	-0.049516	0.9273881	648	707	779	717	730	615
MAP3K20-AS1	1.5454545	0.6280312	0.54657	2	2	7	4	9	4
PUF60	0.8113421	-0.301618	0.0773127	2471	3110	3271	2387	2488	2307
AC026477.1	#DIV/0!	#DIV/0!	0.8030358	0	0	0	0	0	1
AC006210.2	1	0	0.9890559	1	0	0	0	0	1
AC073611.1	0.84	-0.251539	0.8899364	6	6	13	9	3	9
BET1	0.9832144	-0.024422	0.7896495	776	668	939	690	848	805
GEMIN8P4	1.1741071	0.2315641	0.3418054	61	62	101	126	64	73
POT1-AS1	0.6556777	-0.608941	0.0927259	114	67	92	41	65	73
GEMIN8P2	#DIV/0!	#DIV/0!	0.6411933	0	0	0	0	0	2
AC009975.1	0	#NUM!	0.6840298	0	0	2	0	0	0
AL139352.1	0	#NUM!	0.824326	0	0	1	0	0	0
EYS	2.1	1.0703893	0.3126137	5	2	3	6	9	6
AF274855.1	#DIV/0!	#DIV/0!	0.0016993	0	0	0	20	1	4
AKAP9	0.9774229	-0.032945	0.9223031	660	365	791	402	769	604
ANKRA2	0.8261706	-0.275488	0.1716772	526	463	570	312	502	474
EZR	0.8685569	-0.203308	0.1959624	22899	26194	29344	21373	24599	22155
AC046134.1	0	#NUM!	0.824326	1	0	0	0	0	0
BEX4	0.927942	-0.107893	0.6491451	4791	5101	6109	4501	5512	4835
BEX3	1.0086642	0.012446	0.5436487	8171	10049	11673	10193	10734	9225
BEX2	0.8892681	-0.16931	0.469666	1485	1795	1994	1607	1676	1407
BEX1	0.8333333	-0.263034	0.9593655	0	2	4	3	2	0
GNG10	0.3333333	-1.584963	0.5728962	0	6	0	2	0	0
NHLH1	0.7692308	-0.378512	0.8098673	2	3	8	1	8	1
BTN2A3P	0.2372881	-2.075288	0.0135004	19	21	19	7	6	1
AC125238.2	#DIV/0!	#DIV/0!	0.8030358	0	0	0	0	1	0
AC125238.1	0	#NUM!	0.824326	0	0	1	0	0	0
YPEL5P2	0	#NUM!	0.824326	0	0	1	0	0	0
SLC33A1	0.7615441	-0.393001	0.0120016	1367	1191	1535	838	1102	1177
AC005529.1	#DIV/0!	#DIV/0!	0.6411933	0	0	0	0	0	2
AC003072.1	1.2222222	0.2895066	0.7907889	2	2	5	2	5	4
GNG12	1.1779668	0.2362988	0.0716187	4500	3703	4378	3431	5669	5720
GNG11	0.3333333	-1.584963	0.6851201	1	0	2	0	1	0
GNG13	0.4347826	-1.201634	0.3147737	6	14	3	6	2	2
RNVU1-15	1.2	0.2630344	0.9226728	5	0	0	0	1	5
SRGAP2-AS1	0	#NUM!	0.3451105	2	0	3	0	0	0
RNVU1-18	1	0	0.9890559	0	0	1	0	1	0
MEIS3P1	#DIV/0!	#DIV/0!	0.3981469	0	0	0	4	0	0
BEX5	1.1104816	0.1511855	0.3906299	96	110	147	142	142	108
ACVRL1	1	0	0.9890559	1	0	0	1	0	0
AC245041.1	1.2017901	0.2651849	0.1483468	487	294	448	336	530	611

OLA1	1.0891395	0.1231887	0.0930664	4745	4317	5836	5098	5873	5255
SNRK-AS1	0.25	-2	0.3403528	5	0	7	0	0	3
snoZ196	4	2	0.5362768	1	0	0	0	3	1
ANKRD9	0.9706594	-0.042963	0.8895923	950	1125	1231	1050	1100	1059
GLIPR1	0.7808219	-0.356935	0.6001463	37	15	21	20	18	19
AC116903.2	0	#NUM!	0.6436235	2	0	0	0	0	0
GLIPR2	0.4528302	-1.142958	0.0059731	80	101	137	65	27	52
BNIP3P9	0.7058824	-0.5025	0.617561	9	14	28	19	8	9
ANKRD1	0.9259259	-0.111031	0.8423552	34	39	35	20	52	28
CPB2-AS1	2.4	1.2630344	0.3930029	1	2	2	5	0	7
TNFAIP8L1	1.0080906	0.0116253	0.7823477	151	224	243	159	228	236
BNIP3P5	2	1	0.8324799	1	0	0	0	2	0
TNFAIP8L3	#DIV/0!	#DIV/0!	0.4098001	0	0	0	1	3	0
ANKRD2	1.025	0.0356239	0.7426008	44	65	91	68	77	60
BNIP3P4	#DIV/0!	#DIV/0!	0.1209607	0	0	0	6	0	2
TNFAIP8L2	0.125	-3	0.2856934	0	1	7	0	0	1
ANKRD7	#DIV/0!	#DIV/0!	0.8030358	0	0	0	1	0	0
BNIP3P1	1.3846154	0.4694853	0.6878423	4	6	3	2	10	6
ANKRD6	1.0729685	0.1016077	0.2129051	1398	1177	1646	1272	1657	1600
STRBP	1.1285599	0.174483	0.0744268	2125	1697	2393	1926	2552	2536
AC025253.1	0	#NUM!	0.824326	1	0	0	0	0	0
MDFIC	1.1072226	0.1469453	0.2751635	478	438	427	409	572	506
FRG2	1.5714286	0.6520767	0.2900151	12	11	12	13	24	18
AC244453.4	#DIV/0!	#DIV/0!	0.6482162	0	0	0	0	1	1
FRG1	1.2184225	0.2850145	0.021658	1129	926	1571	1390	1731	1297
AC244453.2	0.9189189	-0.121991	0.9390484	13	26	35	19	18	31
STRAP	0.923547	-0.114743	0.5786343	4719	5001	5662	4276	5133	4797
AC244453.1	0.3636364	-1.459432	0.4135973	4	4	3	0	0	4
AC090525.2	#DIV/0!	#DIV/0!	0.3246733	0	0	0	0	3	2
AKAP8	0.8046064	-0.313645	0.0488537	1491	1856	1950	1197	1605	1460
AKAP7	1.1122807	0.1535209	0.4460577	102	59	124	93	107	117
AKAP6	1.1428571	0.1926451	0.4743734	113	48	105	68	105	131
AKAP5	0.9917526	-0.011948	0.8620287	198	107	180	135	166	180
AKAP3	1.0952381	0.1312445	0.9104454	6	11	4	4	14	5
AKAP2	0.125	-3	0.0347369	7	4	21	0	0	4
AKAP1	0.8852268	-0.175881	0.3268921	2110	2131	2616	1807	2051	2212
AC093850.1	#DIV/0!	#DIV/0!	0.6411933	0	0	0	0	0	2
OLAH	1.8358209	0.8764253	0.0072739	41	47	46	66	107	73
FAH	1.0472201	0.0665647	0.3315173	761	822	1043	883	962	905
FAP	1.4	0.4854268	0.2245316	30	19	36	36	50	33
FAS	0.2535211	-1.979822	0.008355	30	12	29	4	6	8
IKBKGP1	0.5	-1	0.4423995	4	5	9	1	7	1
PPY2P	0.1666667	-2.584963	0.3355598	4	2	0	1	0	0
FAU	0.9619952	-0.055898	0.8913777	1724	2140	2451	2309	1973	1793
RN7SL329P	0	#NUM!	0.824326	1	0	0	0	0	0
FBL	1.0222281	0.0317171	0.4720242	7340	9158	10135	9460	9575	8190
AC012146.1	0.5088757	-0.974615	0.0307854	74	41	54	30	32	24
AC012146.3	#DIV/0!	#DIV/0!	0.5070017	0	0	0	3	0	0
AC012146.2	#DIV/0!	#DIV/0!	0.6411933	0	0	0	0	0	2
AC009090.2	#DIV/0!	#DIV/0!	0.8030358	0	0	0	0	0	1
AC009090.5	0	#NUM!	0.5643268	1	0	2	0	0	0
AC009090.4	0.3589744	-1.478047	0.193802	5	19	15	0	1	13
AC009090.1	0.3333333	-1.584963	0.3710845	2	3	10	0	0	5
ANGEL2	0.8599641	-0.217652	0.3054701	582	489	600	340	596	501

ANGEL1	0.8660204	-0.207527	0.2426784	1198	1289	1536	947	1238	1299
TBL1XR1	1.1707458	0.2274279	0.0704151	2612	1682	2611	2158	2966	2960
AC226101.1	1.1538462	0.2064509	0.8395584	11	0	2	7	3	5
AC003957.1	1.5	0.5849625	0.8102929	0	6	0	0	6	3
TGFB2	#DIV/0!	#DIV/0!	0.304772	0	0	0	1	0	4
TGFB1	1.4678348	0.5536896	9.53E-06	1172	1331	1492	2044	2003	1817
TGFB3	1.0958904	0.1321035	0.6953349	28	15	30	24	26	30
BFAR	1	0	0.6629472	1962	2222	2377	1928	2373	2260
NFKBIA	0.8602151	-0.217231	0.2597769	1795	1986	2264	1785	1801	1614
FER	1.1937683	0.2555228	0.1644023	1219	643	1219	767	1476	1435
FES	1.0906516	0.1251903	0.3557742	208	238	260	217	291	262
FEV	0	#NUM!	0.5337954	0	3	0	0	0	0
AC245041.2	0.9593426	-0.059882	0.99452	433	266	457	314	397	398
TGFBI	0.8181818	-0.289507	0.7435107	5	17	11	5	16	6
GRSF1	1.0868146	0.1201059	0.0832192	5808	5623	6953	6080	7054	6846
NFKBIE	1.0260546	0.0371075	0.6786402	195	317	294	276	322	229
NFKBID	0.7258883	-0.46218	0.2507338	72	68	57	47	43	53
NFKBIB	0.7825688	-0.35371	0.0830632	1173	1484	1703	1245	1106	1061
RPS19P3	1.4285714	0.5145732	0.6280278	5	2	7	1	4	15
DHTKD1	0.9481709	-0.076781	0.7787372	4538	4546	4885	3358	4914	4973
NFKBIZ	0.6548913	-0.610673	0.0150271	273	200	263	105	190	187
FGB	0	#NUM!	0.824326	0	0	1	0	0	0
FGG	#DIV/0!	#DIV/0!	0.8030358	0	0	0	0	0	1
RPS19P1	1	0	0.9890559	0	1	0	1	0	0
RNU6-415P	0.7407407	-0.432959	0.7106167	9	7	11	10	6	4
RGS17	1.375	0.4594316	0.4115944	17	2	29	26	13	27
RGS19	1.1463146	0.1970031	0.2072561	267	332	310	370	384	288
RGS14	0.9681129	-0.046753	0.9058022	504	698	711	675	635	542
RGS16	1.3268689	0.4080259	0.0001623	1902	2115	2377	2808	2965	2711
STRN4	0.8712276	-0.198878	0.3214362	2104	2447	2772	2170	2231	1979
STRN3	1.2818846	0.3582664	0.028965	904	591	967	713	1255	1188
RNU6-418P	2	1	0.7812375	0	0	1	1	0	1
ACBD3-AS1	0.2307692	-2.115477	0.2503212	8	1	4	0	0	3
RGS20	0.6911765	-0.532874	0.4071458	16	25	27	10	22	15
AL022329.1	4	2	0.3985669	2	0	0	0	2	6
CCDC150P1	0.9545455	-0.067114	0.90337	516	275	463	265	497	435
RGS10	1.3377049	0.4197599	0.0035363	432	532	561	695	738	607
RGS12	1.2802404	0.3564148	0.0107475	417	453	461	486	582	636
PHBP3	#DIV/0!	#DIV/0!	0.8030358	0	0	0	0	1	0
PHBP5	3	1.5849625	0.5682898	2	0	0	0	0	6
EVX1-AS	0	#NUM!	0.824326	1	0	0	0	0	0
FRS2	0.8815022	-0.181964	0.4344685	1695	1105	1487	840	1403	1536
LDOC1	0.8992095	-0.153271	0.5803199	839	1055	1142	949	908	873
FRS3	0.9496982	-0.074459	0.9556904	150	131	216	183	157	132
AP001599.1	#DIV/0!	#DIV/0!	0.3246733	0	0	0	0	3	2
PHBP9	1.0470588	0.0663425	0.7530011	26	15	44	27	28	34
PHBP8	2	1	0.8319548	0	1	0	0	2	0
FLG	0	#NUM!	0.4521054	1	0	3	0	0	0
AC062021.1	0	#NUM!	0.3728105	1	2	1	0	0	0
AL033397.2	0.7058824	-0.5025	0.7103714	4	5	8	2	2	8
AL033397.1	0	#NUM!	0.824326	0	0	1	0	0	0
RPL21P16	1.0660802	0.092316	0.2396922	1511	1540	1837	1753	1904	1554
AC008906.2	0	#NUM!	0.824326	1	0	0	0	0	0
AC008906.1	0.4444444	-1.169925	0.4066658	10	8	0	0	2	6

RPL21P11	1	0	0.9988754	1	1	0	1	1	0
RPL21P10	0.8235294	-0.280108	0.816929	6	4	7	0	9	5
PLCB2-AS1	#DIV/0!	#DIV/0!	0.4737294	0	0	0	0	4	0
FN1	1.1758507	0.2337048	0.0784379	8682	9633	9487	7647	11614	13430
TPBGL	0	#NUM!	0.6385096	0	2	0	0	0	0
RPL21P28	1.1746032	0.2321734	0.6087137	27	20	16	33	25	16
AF196972.1	#DIV/0!	#DIV/0!	0.6317974	0	0	0	1	1	0
AC068672.3	#DIV/0!	#DIV/0!	0.5371397	0	0	0	0	0	3
OLR1	1.7409639	0.7998863	0.0108185	56	52	58	61	107	121
SPHK2	1.0008258	0.0011908	0.681983	351	388	472	417	417	378
SPHK1	0.7717391	-0.373815	0.5017592	40	75	69	67	47	28
HNRNPABP1	1.2	0.2630344	0.9229862	2	3	0	0	2	4
PDCD5P1	0.9302326	-0.104337	0.9103626	16	14	13	9	16	15
FOS	3.3266389	1.7340653	2.67E-41	758	815	1066	2861	2827	3091
AC009245.1	1.1970954	0.2595382	0.1479502	147	168	167	183	205	189
AC083906.6	0	#NUM!	0.087794	5	2	2	0	0	0
AP000864.1	0	#NUM!	0.1172014	0	7	2	0	0	0
AC083906.1	0	#NUM!	0.824326	0	0	1	0	0	0
AC105118.1	0.6944444	-0.526069	0.5508729	18	6	12	4	16	5
FRZB	0.7	-0.514573	0.7245739	3	7	0	0	3	4
TRAPPC10	0.8590415	-0.2192	0.2652053	803	854	826	503	814	816
NUDT19P5	0	#NUM!	0.3320541	3	0	2	0	0	0
AC010336.2	4	2	0.5067587	0	0	1	4	0	0
AC010336.1	0.9	-0.152003	0.9139587	2	5	3	0	3	6
NUDT19P4	0	#NUM!	0.824326	1	0	0	0	0	0
AL356473.1	1.6666667	0.7369656	0.6816433	1	0	2	3	0	2
FRK	0.7606178	-0.394756	0.0575791	1044	704	842	471	709	790
FRYL	1.1375661	0.1859504	0.2737661	1697	879	1582	1025	1803	1902
RAI1-AS1	#DIV/0!	#DIV/0!	0.4737294	0	0	0	0	4	0
PLOD3	0.8015533	-0.31913	0.0310345	2156	2527	2785	1823	2145	2018
AC018904.1	1.2	0.2630344	0.6316434	19	4	27	10	25	25
PLOD2	1.1115846	0.1526177	0.208942	5346	3388	5112	3885	5819	5687
PLOD1	0.953746	-0.068323	0.9375232	1588	2045	2053	1738	1882	1803
FRY	1	0	0.9785155	28	18	11	18	26	13
RPL21P75	1.1111111	0.1520031	0.563031	37	39	50	59	45	36
SYBU	0.4887082	-1.032955	3.02E-07	348	345	414	186	181	174
BNIP3L	1.2139903	0.2797569	0.0075593	1907	1508	2146	1947	2478	2326
CNTNAP3P2	0.8398058	-0.251872	0.6331323	31	78	97	40	73	60
FST	1.1258147	0.1709694	0.0632385	1122	1121	1286	1231	1405	1337
RPL21P93	1	0	0.9398037	0	0	3	1	1	1
AL022322.2	0.4375	-1.192645	0.4466745	3	7	6	0	0	7
AL022322.1	1.2727273	0.3479233	0.5470076	9	9	26	10	31	15
CLUHP3	0.7993989	-0.323012	0.0997694	432	407	492	277	394	393
FTL	0.6874003	-0.540778	0.0006524	95645	117651	130124	83637	81442	70988
RPL21P89	0.6666667	-0.584963	0.8300111	2	0	4	2	1	1
FTO	0.8357997	-0.258771	0.0915095	1484	1589	1860	1155	1528	1440
FTX	1.0261708	0.0372709	0.6575886	278	132	316	224	275	246
FUK	0.9594293	-0.059752	0.9751866	605	746	892	604	860	688
FUS	0.9461704	-0.079828	0.8808199	11965	13531	15448	12548	13678	12514
LBX1-AS1	0	#NUM!	0.6385096	0	2	0	0	0	0
AL592293.2	3.5	1.8073549	0.3789921	1	0	1	1	3	3
AL592293.1	0.3	-1.736966	0.4902101	0	2	8	3	0	0
FUZ	0.699422	-0.515765	0.2377007	49	53	71	41	37	43
NDC80	1.1963651	0.2586577	0.0659463	858	539	1134	903	1118	1007

AL359263.1	0	#NUM!	0.6436235	2	0	0	0	0	0
RPL21P40	2	1	0.8147319	1	0	0	0	0	2
RLBP1	0	#NUM!	0.824326	0	0	1	0	0	0
PKN2-AS1	1	0	0.9445541	3	3	3	5	2	2
PSMC1P9	0	#NUM!	0.824326	1	0	0	0	0	0
RPL21P39	#DIV/0!	#DIV/0!	0.8030358	0	0	0	0	1	0
AC013468.1	0.4285714	-1.222392	0.5961525	2	0	5	0	2	1
AC008750.1	0	#NUM!	0.5781912	0	0	3	0	0	0
AC072046.1	0	#NUM!	0.824326	1	0	0	0	0	0
COMMD10	0.8369478	-0.25679	0.2144086	404	371	470	286	384	372
AC097375.3	0	#NUM!	0.3482063	0	0	6	0	0	0
PSMC1P1	1.0467013	0.0658497	0.4164156	421	415	513	454	519	439
PSMC1P2	0	#NUM!	0.4217812	0	0	5	0	0	0
PSMC1P5	#DIV/0!	#DIV/0!	0.6411933	0	0	0	0	0	2
PSMC1P4	#DIV/0!	#DIV/0!	0.4098001	0	0	0	1	3	0
PSMC1P7	0	#NUM!	0.824326	0	1	0	0	0	0
SYF2	1.1468971	0.1977359	0.0917524	1342	928	1549	1472	1599	1309
FXN	1.1659872	0.2215519	0.0578141	516	530	671	677	709	616
AL589740.1	0.68	-0.556393	0.5141244	16	27	32	28	5	18
AC068338.2	0.6388889	-0.646363	0.5657513	4	11	21	12	6	5
RCHY1	0.9569472	-0.063489	0.9039893	365	321	336	262	354	362
FSD2	0.6666667	-0.584963	0.81955	0	3	0	0	1	1
FYN	2.0536044	1.0381583	3.41E-08	195	134	212	350	414	347
FSD1	1.485782	0.5712224	0.0007425	273	298	273	389	482	383
MIF-AS1	0.4038462	-1.308122	0.0106538	53	30	73	11	32	20
AC008750.2	0	#NUM!	0.824326	0	0	1	0	0	0
AC008750.5	1.9090909	0.9328858	0.3892882	3	1	7	3	3	15
AC008750.7	0.6666667	-0.584963	0.8308055	3	0	0	0	2	0
CD46P1	0	#NUM!	0.6385096	0	2	0	0	0	0
AC006509.2	1	0	0.9799273	3	0	1	1	0	3
AL158156.1	#DIV/0!	#DIV/0!	0.4501902	0	0	0	0	0	4
RPL21P65	2	1	0.7741804	1	0	0	2	0	0
FSBP	0.8	-0.321928	0.9355678	0	1	4	0	0	4
STUB1	1.0344288	0.0488343	0.4953367	544	641	732	788	633	562
SLC9A3-AS1	1.8481013	0.8860438	0.0057084	64	37	57	114	105	73
AC112206.4	#DIV/0!	#DIV/0!	0.1269961	0	0	0	1	6	1
AC112206.3	#DIV/0!	#DIV/0!	0.075415	0	0	0	7	1	1
AC112206.2	#DIV/0!	#DIV/0!	0.3109758	0	0	0	0	6	0
AC138956.1	1.9473684	0.9615259	0.2502701	6	1	12	7	14	16
AC005034.5	2.4285714	1.2801079	0.3777974	4	1	2	0	5	12
AC005034.4	2.25	1.169925	0.5287158	3	1	0	3	0	6
AC138956.2	0.5970149	-0.744161	0.3195732	26	13	28	5	9	26
AC005034.3	0.8392371	-0.25285	0.4496179	144	101	122	91	127	90
AC005034.2	0.0769231	-3.70044	0.0701371	3	5	5	0	0	1
ZNF324B	0.6640316	-0.590676	0.1067942	70	85	98	60	60	48
OMA1	1.0949153	0.1308192	0.3598953	318	260	307	272	404	293
AL359513.1	1.4186047	0.5044726	0.3437999	9	11	23	23	24	14
SMIM10L2B	0.7045455	-0.505235	0.2065422	59	80	81	57	57	41
SMIM10L2A	0.7941176	-0.332575	0.7390573	6	12	16	6	18	3
BMS1P2	1.5454545	0.6280312	0.4492342	7	6	9	4	8	22
BMS1P1	0.6727273	-0.571906	0.1203615	136	89	160	54	73	132
BMS1P4	0.6694215	-0.579013	0.3054208	53	20	48	10	40	31
BMS1P4	0.6694215	-0.579013	0.3054208	53	20	48	10	40	31
DSG1-AS1	0	#NUM!	0.824326	0	0	1	0	0	0

BMS1P9	0.5	-1	0.5624554	4	2	8	4	0	3
BMS1P8	0.9541547	-0.067705	0.9594189	133	91	125	89	102	142
BMS1P7	#DIV/0!	#DIV/0!	0.8030358	0	0	0	0	1	0
SAGE3P	0	#NUM!	0.824326	0	1	0	0	0	0
SYNM	0.7526882	-0.409876	0.2491982	89	114	76	48	72	90
RNA5SP305	0.5	-1	0.7575219	1	0	3	0	0	2
RNA5SP303	#DIV/0!	#DIV/0!	0.6411933	0	0	0	0	0	2
ZNF321P	0.657754	-0.60438	0.2121895	66	40	81	44	27	52
RNA5SP306	0	#NUM!	0.824326	1	0	0	0	0	0
AC005274.1	1	0	0.9890559	1	0	0	0	0	1
GAA	0.9110266	-0.134435	0.5729166	4255	5145	5065	4449	4497	4232
SYN3	1.2666667	0.3410369	0.742266	3	8	4	3	4	12
SYN2	1.7777778	0.830075	0.1727612	16	11	9	26	13	25
SYN1	#DIV/0!	#DIV/0!	0.6554062	0	0	0	0	2	0
GAK	0.9254981	-0.111698	0.6802576	2677	3145	3614	2736	3060	2937
GAN	1.1035124	0.1421028	0.2171297	2031	1457	1893	1528	2246	2164
FSHR	#DIV/0!	#DIV/0!	0.1771678	0	0	0	3	4	0
RNA5SP323	0.7647059	-0.387023	0.7587314	6	5	6	0	4	9
GBA	0.8298788	-0.269028	0.0809502	1552	1704	1858	1269	1480	1495
AC004540.2	3.5	1.8073549	0.4030153	0	0	2	1	5	1
AC004540.1	0.375	-1.415037	0.4697467	4	2	2	0	3	0
GCA	2.6144068	1.3864836	1.12E-07	76	80	80	130	249	238
RNA5SP312	0	#NUM!	0.6631647	1	0	1	0	0	0
RNA5SP311	0.4444444	-1.169925	0.6038305	4	0	5	3	1	0
RNA5SP310	#DIV/0!	#DIV/0!	0.6099294	0	0	0	2	0	0
RNA5SP315	0	#NUM!	0.824326	0	0	1	0	0	0
GCG	#DIV/0!	#DIV/0!	0.3096126	0	0	0	5	0	0
GCK	#DIV/0!	#DIV/0!	0.8030358	0	0	0	0	1	0
ıeostasis;GO:0(	#DIV/0!	#DIV/0!							
0005536|gluco:	#VALUE!	#VALUE!	ucokinase [E	arbon metab	glucokinase				
RNA5SP317	0.5	-1	0.686714	2	0	6	0	0	4
CFHR1	1	0	0.9890559	1	0	0	0	1	0
CFHR4	0	#NUM!	0.199693	0	4	3	0	0	0
CFHR3	1.6595745	0.7308134	0.1747619	17	14	16	17	26	35
SYS1	0.9694357	-0.044783	0.9630264	755	937	860	810	892	772
GDA	0.7818182	-0.355095	0.4097386	113	55	162	54	118	86
RPP30	1.0686695	0.0958158	0.3077717	614	511	739	660	752	580
RNA5SP342	#DIV/0!	#DIV/0!	0.4737294	0	0	0	0	4	0
AL161449.1	#DIV/0!	#DIV/0!	0.4737294	0	0	0	0	4	0
AC079140.5	1.0465116	0.0655883	0.8338092	9	11	23	11	19	15
AC079140.2	1.4047619	0.4903256	0.393428	12	18	12	17	21	21
AC079140.1	1	0	0.9586204	0	4	0	0	4	0
AC079140.4	0.1428571	-2.807355	0.2885999	0	6	1	0	1	0
AC079140.3	1.3820225	0.4667811	0.2342132	32	26	31	38	51	34
BORCS8	0.7631579	-0.389947	0.2301999	123	136	197	124	128	96
BORCS7	0.953869	-0.068137	0.9534623	255	202	215	221	215	205
BORCS6	0.7808696	-0.356847	0.2548525	123	249	203	101	168	180
RPP38	1.272093	0.3472042	0.034583	249	265	346	382	420	292
BORCS5	0.998088	-0.002761	0.8055826	176	166	181	171	185	166
RNA5SP335	0	#NUM!	0.3473206	0	5	0	0	0	0
GEM	0.4285714	-1.222392	0.3976488	7	2	12	2	0	7
RPP21	1.032967	0.0467942	0.8626346	37	26	28	27	43	24
RPP25	0.8112058	-0.30186	0.2752396	280	232	309	245	238	183
AC007671.2	0	#NUM!	0.824326	0	0	1	0	0	0

AC020629.1	2	1	0.5631389	0	1	3	2	0	6
RN7SL775P	#DIV/0!	#DIV/0!	0.4737294	0	0	0	0	4	0
RNA5SP364	#DIV/0!	#DIV/0!	0.3109758	0	0	0	0	6	0
AC138466.1	0.5	-1	0.737303	1	3	0	2	0	0
AC079776.1	2	1	0.66599	1	0	1	2	2	0
AC138466.5	0.1428571	-2.807355	0.161533	10	0	4	0	2	0
RPS18P9	0.8536585	-0.228269	0.8459792	28	13	41	31	17	22
SYCE3	2.8333333	1.5025003	0.2346516	1	2	3	9	7	1
SYCE2	0.4528302	-1.142958	0.1203922	19	17	17	6	8	10
SYCE1	1	0	0.9890559	0	1	0	0	0	1
RR5-ARHGAI	1.3243243	0.4052565	0.5754444	7	6	24	2	16	31
GFY	1.4347826	0.5208322	0.4582547	8	6	9	20	7	6
AC132008.2	0.8044693	-0.313891	0.122524	492	387	553	346	421	385
AC132008.1	0.5	-1	0.8652929	0	0	2	0	1	0
SYT5	1.4482759	0.5343364	0.4124167	2	8	19	20	12	10
RNA5SP351	0	#NUM!	0.824326	0	0	1	0	0	0
SYT3	0.375	-1.415037	0.5071527	2	1	5	0	3	0
SYT2	1.1728395	0.2300056	0.3958685	39	31	92	63	59	68
PRKX-AS1	0	#NUM!	0.5387425	3	0	0	0	0	0
SYT1	1.0394432	0.0558109	0.6434042	306	200	356	201	385	310
RPP40	0.9175134	-0.124199	0.6539755	511	531	631	469	586	480
GGH	1.0909352	0.1255654	0.151206	1166	1025	1295	1107	1421	1275
SYT9	4	2	0.5525509	0	1	0	0	0	4
SYT8	0.9082969	-0.138764	0.9466416	59	70	100	101	45	62
AC093895.1	1.0535714	0.0752881	0.8019193	16	18	22	25	24	10
MANBAL	0.9084564	-0.138511	0.611417	1620	2202	2339	1860	2008	1729
SYT7	0.6170142	-0.696624	3.46E-05	907	1087	1027	476	750	638
GGN	1.25	0.3219281	0.726442	2	2	8	5	0	10
GH2	0.5491329	-0.864773	0.0417455	65	56	52	30	28	37
AC092042.1	#DIV/0!	#DIV/0!	0.8030358	0	0	0	0	0	1
AC092042.3	0	#NUM!	0.824326	0	1	0	0	0	0
RNA5SP383	0.7021277	-0.510195	0.5559978	15	14	18	0	26	7
GHR	1.0966209	0.1330649	0.3426593	684	467	743	469	773	835
AL512624.2	2	1	0.4315397	1	1	4	6	4	2
AL512624.1	0.4897959	-1.029747	0.1779368	16	22	11	7	7	10
AL020996.1	1.8	0.8479969	0.6996933	0	3	2	0	9	0
TGIF1	0.8974474	-0.156101	0.4562217	1871	2269	2520	1892	2132	1953
RNA5SP372	0	#NUM!	0.6436235	2	0	0	0	0	0
TGIF2	1.1459898	0.1965942	0.0882511	931	1159	1239	1214	1482	1119
GIF	0	#NUM!	0.6385096	0	2	0	0	0	0
RNA5SP379	0.5	-1	0.7019715	2	2	0	0	0	2
SPIDR	0.8371747	-0.256399	0.1280144	875	815	1000	671	808	773
RNU6ATAC18	#DIV/0!	#DIV/0!	0.8030358	0	0	0	1	0	0
GIP	0.9230769	-0.115477	0.8964942	14	24	27	9	23	28
Z98884.2	0.8571429	-0.222392	0.7209793	30	18	22	7	31	22
SAMMSON	4.3	2.1043367	0.0455255	2	0	8	4	28	11
AL080284.1	#DIV/0!	#DIV/0!	0.3981469	0	0	0	4	0	0
CASC11	1.2	0.2630344	0.9062807	4	1	0	1	3	2
CASC10	1.0192837	0.0275557	0.6247041	279	370	440	339	423	348
AL645998.1	#DIV/0!	#DIV/0!	0.6554062	0	0	0	0	2	0
GK5	0.7914013	-0.337519	0.0851936	666	500	718	402	529	560
AL008718.3	1.5	0.5849625	0.6497351	6	0	2	6	1	5
AP005329.1	1	0	0.9922023	2	0	0	0	0	2
AC104623.1	0	#NUM!	0.824326	0	1	0	0	0	0

CASC19	4.5	2.169925	0.3117124	0	2	0	1	1	7
CASC18	3.5	1.8073549	0.4428425	0	2	0	2	0	5
RN7SL771P	8	3	0.2077429	0	0	1	4	0	4
AL157911.1	1	0	0.9890559	0	1	0	0	1	0
AP005329.2	0.8571429	-0.222392	0.8444044	9	2	3	1	9	2
RPP14	0.8620253	-0.214198	0.3222057	518	459	603	409	458	495
CASC15	0.7362205	-0.44179	0.2166475	74	91	89	62	65	60
AC007431.2	#DIV/0!	#DIV/0!	0.8030358	0	0	0	0	1	0
ATF7IP	1.1101336	0.1507333	0.2805674	1156	835	1078	792	1210	1405
RNA5SP395	#DIV/0!	#DIV/0!	0.5371397	0	0	0	0	0	3
CASC21	#DIV/0!	#DIV/0!	0.8030358	0	0	0	0	0	1
CASC20	1.5625	0.6438562	0.4642658	3	2	11	7	13	5
FAHD1	0.8374291	-0.255961	0.2200108	626	682	808	605	582	585
AC114491.1	1.1891892	0.2499783	0.668568	10	18	9	19	13	12
MIR4500HG	0.6222222	-0.684498	0.5014202	16	2	27	5	8	15
AC008417.1	#DIV/0!	#DIV/0!	0.1081142	0	0	0	2	0	7
AC087239.1	1.5	0.5849625	0.8246284	0	2	0	2	0	1
GLA	0.9510127	-0.072463	0.9029117	652	748	723	671	780	568
ANKLE2	0.8914454	-0.165782	0.338655	2605	2549	3321	2140	2816	2599
ANKLE1	2.2162162	1.1480986	0.048649	12	6	19	17	36	29
G2E3-AS1	1.1355932	0.1834461	0.5459399	63	69	45	58	66	77
GLS	1.2359503	0.3056208	0.0149077	4394	3194	4334	3718	5555	5462
CSPP1	0.8992629	-0.153185	0.6432098	788	413	834	470	724	636
AC008655.2	0.375	-1.415037	0.4829106	3	3	2	0	0	3
AP1S2	0.9032922	-0.146735	0.7441092	172	106	208	104	159	176
AP1S1	1.0289356	0.0411528	0.4280386	7928	9606	10079	9585	10023	8804
AP1S3	0.8435233	-0.2455	0.359506	355	323	287	155	296	363
SPANXB1	1.7096774	0.7737241	0.2378284	14	9	8	25	9	19
AC117382.2	1	0	0.9745815	0	4	0	0	1	3
AC117382.1	0.7058824	-0.5025	0.7531762	4	4	9	7	3	2
RNU6-883P	0.4444444	-1.169925	0.6008741	0	2	7	0	4	0
GNE	1.3023256	0.3810902	0.0009382	2589	2431	2978	2632	3839	3945
OR11H13P	0	#NUM!	0.824326	0	0	1	0	0	0
GNS	0.7536435	-0.408046	0.0072448	7598	8185	8507	4549	6537	7220
AC021218.1	#DIV/0!	#DIV/0!	0.6411933	0	0	0	0	0	2
GRTP1	0.9345912	-0.097593	0.7967745	721	735	929	691	790	748
GP6	0.75	-0.415037	0.7472225	7	4	9	5	4	6
GP5	0	#NUM!	0.824326	0	0	1	0	0	0
H19	0.583427	-0.777376	0.4163634	2319	415	826	1092	637	348
AL031009.1	1.2058824	0.2700892	0.5984044	19	24	25	11	28	43
GPI	0.9665662	-0.04906	0.8983663	6399	7333	8162	7203	7338	6621
RNU6-880P	0.5	-1	0.8652929	0	0	2	0	0	1
GPT	2.3333333	1.2223924	0.3653456	2	2	2	2	3	9
COX11P1	#DIV/0!	#DIV/0!	0.5371397	0	0	0	0	0	3
AL161937.2	0.5	-1	0.5455312	3	3	8	1	0	6
SUPT4H1P2	0	#NUM!	0.824326	0	1	0	0	0	0
SUPT4H1P1	#DIV/0!	#DIV/0!	0.6411933	0	0	0	0	0	2
AL161937.1	#DIV/0!	#DIV/0!	0.8030358	0	0	0	0	0	1
NUTF2	0.9416811	-0.08669	0.9049695	6850	7620	9193	7777	7756	6750
EGLN3-AS1	0.4285714	-1.222392	0.4772578	13	1	0	1	0	5
GRN	0.8693107	-0.202056	0.3166284	7660	9439	10233	8227	8211	7322
GRP	0	#NUM!	0.6604275	0	1	1	0	0	0
SGMS1	1.0128949	0.0184845	0.6925739	497	457	597	380	562	629
SGMS2	0.6842105	-0.547488	0.8657644	0	0	19	9	2	2

AC120049.1	0.5	-1	0.2421881	20	8	16	8	5	9
APOLD1	0.7401575	-0.434096	0.4647755	40	21	66	29	37	28
SCAF8	0.8184722	-0.288995	0.1200557	1145	1275	1337	768	1041	1266
RNU6-882P	0	#NUM!	0.1824345	5	2	0	0	0	0
GSN	0.8756915	-0.191505	0.3854351	912	1111	1050	918	948	825
GSS	0.7820035	-0.354753	0.0215404	2294	2718	3034	2039	2236	2017
GSR	0.4522756	-1.144726	2.74E-19	2700	2980	3131	1124	1493	1368
SCAF1	0.9648846	-0.051572	0.892689	2864	3789	4140	3728	3448	3238
SCAF4	0.9236505	-0.114581	0.5965871	1925	1971	2273	1584	2038	2076
AC009244.1	1.2340426	0.3033921	0.0611255	189	194	228	241	274	239
ANKRD13C	1.2033149	0.2670143	0.1912717	367	215	323	236	466	387
RPL21P110	0.6666667	-0.584963	0.8319544	0	2	1	0	2	0
ANKRD13B	0.8627451	-0.212994	0.868024	19	6	26	14	12	18
ANKRD13D	1.1110545	0.1519296	0.2059011	559	654	750	839	623	719
SYAP1	1.0757024	0.105279	0.1857941	1274	1164	1406	1279	1422	1434
SLC39A9	0.8843692	-0.177279	0.3088138	3395	3068	3690	2255	3349	3375
SLC39A6	0.7192374	-0.47546	0.0187226	661	464	606	304	474	467
SLC39A5	#DIV/0!	#DIV/0!	0.5338029	0	0	0	0	1	2
AC010087.1	0.3333333	-1.584963	0.7290749	0	0	3	0	0	1
SLC39A8	1.6102293	0.6872661	2.03E-08	1771	1402	1930	2192	3116	2909
RPL21P119	1.6	0.6780719	0.6729315	0	3	2	3	4	1
SLC39A7	0.8119794	-0.300485	0.0451533	5153	5890	6604	4650	5108	4571
SLC39A2	#DIV/0!	#DIV/0!	0.0411444	0	0	0	5	4	2
SLC39A1	0.8869984	-0.172997	0.3611697	7080	8644	9373	7127	8171	6963
ANKRD13A	0.761121	-0.393802	0.0059211	1436	1406	1654	962	1330	1130
SLC39A4	0.8792271	-0.185692	0.5998125	256	299	273	286	213	229
SLC39A3	0.9783491	-0.031579	0.8779902	189	293	257	301	247	175
RPL21P121	0	#NUM!	0.5172237	1	2	0	0	0	0
RPL21P120	#DIV/0!	#DIV/0!	0.8030358	0	0	0	1	0	0
AC024681.2	0	#NUM!	0.6840298	0	0	2	0	0	0
AC109309.1	0	#NUM!	0.6840298	0	0	2	0	0	0
AP1M2	0.7171129	-0.479728	0.0048003	1251	1432	1612	1069	1102	909
AP1M1	0.8447127	-0.243467	0.19853	1372	1728	1704	1351	1414	1293
AC097374.2	0	#NUM!	0.0878436	3	3	3	0	0	0
AC097374.1	0.1578947	-2.662965	0.0070629	34	4	19	1	3	5
AL022323.4	11	3.4594316	0.1040418	0	1	0	2	7	2
AC022140.1	0.4210526	-1.247928	0.3457525	11	6	2	5	1	2
CSPG5	0.9767442	-0.033947	0.9402462	10	16	17	18	12	12
CSPG4	0.3636364	-1.459432	0.0303998	26	21	30	4	9	15
FTCD	0.1666667	-2.584963	0.2163983	5	0	7	1	1	0
AP1G2	1.013308	0.0190727	0.5742525	477	468	633	606	495	498
AP1G1	0.9113122	-0.133983	0.5059718	4320	4193	4747	2887	4424	4773
AL732314.4	0	#NUM!	0.824326	0	1	0	0	0	0
AL732314.6	0.3076923	-1.70044	0.3625549	0	9	4	3	1	0
AC008895.1	2.8333333	1.5025003	0.2680856	4	1	1	2	6	9
'BC1D22A-AS	0.25	-2	0.518449	1	1	2	0	0	1
NUTM1	0.7219512	-0.470027	0.2406592	49	76	80	41	53	54
FRG1-DT	0.6883117	-0.538866	0.37737	21	26	30	10	19	24
PAICSP1	0.5	-1	0.4323216	12	5	5	3	0	8
AC026333.4	5.6666667	2.5025003	0.0870728	1	0	2	5	5	7
PAICSP4	0.5	-1	0.6714478	0	2	4	0	2	1
PAICSP7	1.8333333	0.8744691	0.6407676	2	4	0	0	2	9
BX293535.1	6	2.5849625	0.3502123	0	1	0	0	3	3
AL079303.2	0.1538462	-2.70044	0.2085685	2	1	10	2	0	0

AC005523.1	#DIV/0!	#DIV/0!	0.0548352	0	0	0	6	1	3
TRAF3IP3	1.5454545	0.6280312	0.4217473	8	9	5	10	12	12
TRAF3IP2	0.8102386	-0.303581	0.0847989	766	921	911	641	738	726
TRAF3IP1	0.9543974	-0.067338	0.907265	303	273	345	223	362	294
KAT5	0.9208592	-0.118947	0.7169255	506	542	721	482	574	573
FTH1	0.6836511	-0.548668	0.0005713	15765	19836	22727	13912	13781	12183
KAT8	0.8540459	-0.227615	0.2419026	1196	1361	1410	1155	1194	1039
KAT7	0.7776406	-0.362825	0.0118369	1719	1889	2063	1208	1681	1521
TKTL1	2.0153846	1.0110552	0.0275308	16	31	18	43	46	42
ESCO1	1.8222222	0.8656989	4.19E-05	515	228	472	595	883	736
AP1B1	0.7795872	-0.359218	0.0182537	3390	4003	4090	2888	2985	3079
AP1AR	1.0544252	0.0764568	0.5443862	752	460	754	463	805	805
ESCO2	0.8776758	-0.18824	0.6202379	259	125	270	149	222	203
ATP6V0E1P2	#DIV/0!	#DIV/0!	0.8030358	0	0	0	0	0	1
ATP6V0E1P3	0	#NUM!	0.1397042	2	6	0	0	0	0
AC100797.4	3.6666667	1.8744691	0.156482	0	0	6	10	1	11
AC100797.1	2	1	0.834705	0	0	1	0	2	0
KARS	0.9194451	-0.121165	0.5825883	9233	9819	11871	8916	10252	9264
LUCAT1	0.4074074	-1.295456	0.2872701	13	10	4	1	0	10
TWIST2	0.2857143	-1.807355	0.2619536	6	5	3	0	4	0
TWIST1	0.9818731	-0.026391	0.8644817	110	102	119	125	90	110
RNA5SP402	0	#NUM!	0.6436235	2	0	0	0	0	0
AL020995.1	1.5	0.5849625	0.8175361	2	0	0	3	0	0
FUT8-AS1	0.9871795	-0.018616	0.9236851	19	67	70	44	66	44
USP32P2	0.8518519	-0.231326	0.7667451	25	8	21	5	24	17
USP32P1	1.0217391	0.0310269	0.8744664	51	11	30	27	25	42
USP32P3	0.9708029	-0.04275	0.9370735	59	17	61	40	45	48
TYMSOS	0.5916667	-0.757143	0.1480009	38	28	54	21	32	18
HAL	2.2828283	1.1908223	0.0020147	37	32	30	45	80	101
ZFAS1	1.2045455	0.2684888	0.061563	303	242	335	331	381	348
FAM160A1-DT	0.0882353	-3.5025	0.0060717	7	16	11	1	1	1
GABPA	1.1935964	0.2553151	0.0956178	1025	634	1027	769	1266	1171
AC007182.1	0.6176471	-0.695145	0.2795252	17	26	25	13	16	13
AC007182.2	3.5	1.8073549	0.1905772	0	5	1	7	1	13
AP000529.1	0	#NUM!	0.2290435	7	0	0	0	0	0
AC135721.2	0.7272727	-0.459432	0.7312447	3	6	2	0	7	1
RPL21P132	0.3333333	-1.584963	0.6638759	0	3	0	0	1	0
RPL21P134	0.2857143	-1.807355	0.5104392	7	0	0	2	0	0
RNA5SP443	#DIV/0!	#DIV/0!	0.6099294	0	0	0	2	0	0
RNA5SP449	#DIV/0!	#DIV/0!	0.5371397	0	0	0	0	0	3
PSMC3IP	0.9009009	-0.15056	0.7503612	160	154	241	166	162	172
AL356961.1	0	#NUM!	0.824326	1	0	0	0	0	0
AC024587.2	#DIV/0!	#DIV/0!	0.6099294	0	0	0	2	0	0
UBTD2	1.036351	0.0515128	0.4380178	1828	1877	2072	1593	2226	2168
UBTD1	1.2081218	0.2727659	0.1845516	114	143	137	183	151	142
GABRE	0.6222363	-0.684465	3.16E-05	2705	1911	3254	1414	1700	1783
RPL21P136	0	#NUM!	0.6385096	0	2	0	0	0	0
GABRD	2.8	1.4854268	0.1488334	4	4	2	17	2	9
HDX	0.3	-1.736966	0.3290115	4	3	3	0	1	2
GABRQ	0.7051745	-0.503948	0.0463057	281	262	288	166	162	258
GABRP	0.3513514	-1.509014	0.0349858	22	15	37	9	6	11
AC093510.2	#DIV/0!	#DIV/0!	0.8030358	0	0	0	0	1	0
AC138811.1	#DIV/0!	#DIV/0!	0.8030358	0	0	0	0	0	1
FAM189A2	2.3946488	1.2598141	2.48E-08	93	84	122	260	211	245

FAM189A1	0.7884615	-0.342888	0.5931884	31	23	50	28	27	27
RNA5SP435	#DIV/0!	#DIV/0!	0.6411933	0	0	0	0	0	2
RNA5SP434	0.5	-1	0.6089767	7	1	0	0	4	0
AC093510.1	1	0	0.9736889	5	1	6	1	6	5
RNA5SP465	0	#NUM!	0.6410557	1	1	0	0	0	0
HFE	0.8108108	-0.302563	0.7807087	3	16	18	3	7	20
RNA5SP464	0.1818182	-2.459432	0.2497203	7	0	4	0	2	0
RNA5SP463	0	#NUM!	0.824326	0	1	0	0	0	0
RPS17P8	#DIV/0!	#DIV/0!	0.6554062	0	0	0	0	2	0
AP000769.1	0.8901099	-0.167945	0.8208996	41	21	29	27	26	28
AL513211.1	0.6842105	-0.547488	0.6440993	12	2	5	3	8	2
VASH1-AS1	0.5365854	-0.89812	0.3173473	23	9	9	10	4	8
TRAPPC12	0.9529954	-0.069459	0.9392569	1236	1466	1638	1286	1461	1389
TRAPPC11	1.2178928	0.2843871	0.052994	1206	774	1228	956	1490	1461
TRAPPC13	0.9780702	-0.03199	0.8945719	510	365	493	422	523	393
RNA5SP451	#DIV/0!	#DIV/0!	0.4909737	0	0	0	2	0	1
RNA5SP450	0	#NUM!	0.6385096	0	2	0	0	0	0
SZT2	0.8277489	-0.272735	0.2909155	624	687	808	294	673	787
TRAPPC2L	0.9989836	-0.001467	0.6208079	2032	2193	2662	2474	2391	2015
HGD	0	#NUM!	0.2883218	0	0	7	0	0	0
RNA5SP452	0	#NUM!	0.6436235	2	0	0	0	0	0
KAZN	0.7159624	-0.482044	0.0589634	253	259	340	209	250	151
TRAPPC2B	1.0297398	0.0422798	0.6879489	96	68	105	104	86	87
AC145138.1	1.375	0.4594316	0.5984865	6	3	7	6	3	13
HGS	0.7933844	-0.333908	0.0209552	1908	1928	2301	1549	1724	1596
RPS17P2	1	0	0.9890559	0	0	1	0	1	0
AL138762.1	0.2727273	-1.874469	0.3182572	7	1	3	2	0	1
A2M-AS1	0.8421053	-0.247928	0.8399835	4	16	18	11	8	13
RNA5SP490	1	0	0.9890559	0	0	1	1	0	0
RNA5SP473	#DIV/0!	#DIV/0!	0.6099294	0	0	0	2	0	0
RNA5SP474	0.25	-2	0.5498031	4	0	0	1	0	0
SEPSECS	1.0645856	0.0902919	0.5403643	377	223	329	255	416	318
AC092437.1	0.25	-2	0.5688068	1	0	3	0	0	1
HK2	0.9826899	-0.025192	0.9029575	6585	7332	7400	5431	7612	7905
HK1	1.3897246	0.4747991	2.20E-05	3350	4015	4002	4963	5405	5429
HK3	0	#NUM!	0.6436235	2	0	0	0	0	0
AL008719.1	#DIV/0!	#DIV/0!	0.8030358	0	0	0	1	0	0
AC027796.5	0	#NUM!	0.1540435	0	5	3	0	0	0
AP005328.2	0.2	-2.321928	0.2359419	3	5	2	0	2	0
AP005328.1	0.3478261	-1.523562	0.2293773	13	5	5	4	4	0
RNU6-876P	#DIV/0!	#DIV/0!	0.3831122	0	0	0	0	5	0
AC027796.4	1.1666667	0.2223924	0.8032976	6	6	6	3	8	10
AC027796.2	0.7073171	-0.499571	0.6363224	18	4	19	14	10	5
AC027796.1	0.1428571	-2.807355	0.2729746	3	4	0	0	0	1
RNA5SP494	2	1	0.6959447	2	0	0	4	0	0
RNA5SP498	0.6	-0.736966	0.7543606	5	0	0	0	3	0
AXIN1	1.2143922	0.2802344	0.0102876	1408	1614	1675	1882	1986	1836
AL359752.1	2	1	0.2913036	5	8	2	21	5	4
AXIN2	1.1727494	0.2298948	0.0229202	1021	1045	1222	1090	1424	1342
TRAPPC6A	1.0394069	0.0557606	0.4616121	660	921	982	949	901	814
TRAPPC6B	1.1180521	0.1609875	0.2354479	755	498	780	564	899	810
RNU6-875P	0.5	-1	0.8638172	0	0	2	1	0	0
HLF	0	#NUM!	0.824326	1	0	0	0	0	0
AP001357.2	0	#NUM!	0.824326	0	0	1	0	0	0

AC091849.2	0.5	-1	0.6312231	4	1	1	0	3	0
HLX	1.1272727	0.1728366	0.7824293	24	18	13	10	30	22
LEPROTL1	0.9241497	-0.113802	0.6523835	956	876	1108	770	976	971
PDIA3P2	0.5333333	-0.906891	0.6046125	2	3	10	4	0	4
PDIA3P1	1.0302698	0.0430222	0.3903787	1408	1411	1740	1573	1591	1533
TP73-AS1	0.9118881	-0.133071	0.686148	394	425	611	370	477	457
TP73-AS1	0.9118881	-0.133071	0.686148	394	425	611	370	477	457
AL122010.1	0.6091954	-0.715023	0.2319557	22	36	29	14	13	26
MAPKBP1	1.1654412	0.2208762	0.052251	743	812	893	788	996	1069
EPB41L4A-AS	1.1129303	0.1543633	0.1346568	675	806	901	876	896	879
EPB41L4A-AS	0.4	-1.321928	0.2997525	13	11	1	0	1	9
NCBP2-AS1	0.6428571	-0.63743	0.5156927	12	8	8	7	6	5
HPD	1.0743802	0.1035046	0.7106912	52	32	37	44	48	38
AL591222.1	0	#NUM!	0.824326	0	0	1	0	0	0
NCBP2-AS2	0.7793048	-0.35974	0.1264955	696	804	974	737	692	499
AC092436.2	4	2	0.5754166	0	1	0	0	4	0
HPN	0.7857143	-0.347923	0.6581332	10	19	27	10	23	11
FIGNL2	0.788472	-0.342869	0.0703891	975	1423	1280	772	1045	1083
AP000720.2	0	#NUM!	0.824326	0	0	1	0	0	0
HPR	0.8333333	-0.263034	0.9166484	6	0	0	3	2	0
AC004448.4	#DIV/0!	#DIV/0!	0.6099294	0	0	0	2	0	0
HPX	1.25	0.3219281	0.8621254	1	3	0	3	0	2
GCNT1P3	#DIV/0!	#DIV/0!	0.3831122	0	0	0	0	5	0
FIGNL1	1.0809434	0.112291	0.3775668	1370	925	1436	908	1560	1565
SCDP1	6	2.5849625	0.1438781	1	1	0	4	3	5
EXOC6B	1.0870638	0.1204366	0.3205841	3027	2498	2986	2098	3478	3676
HRC	1	0	0.9838634	1	0	1	0	0	2
HRG	0	#NUM!	0.4374838	0	4	0	0	0	0
HRK	0.954955	-0.066495	0.9602908	37	51	23	47	41	18
AC099676.1	0.7382716	-0.437776	0.1867903	157	89	159	64	121	114
LRRC37A4P	0.5384615	-0.893085	0.6726281	7	0	6	6	0	1
AC006017.1	0.5882353	-0.765535	0.6047123	3	7	7	4	0	6
RNU6-892P	#DIV/0!	#DIV/0!	0.5371397	0	0	0	0	0	3
RNU6-891P	#DIV/0!	#DIV/0!	0.6554062	0	0	0	0	2	0
AL022324.3	2.5	1.3219281	0.6338788	0	0	2	0	5	0
AL022324.2	0.2244898	-2.155278	0.0348583	10	13	26	8	1	2
MANEAL	#DIV/0!	#DIV/0!	0.8030358	0	0	0	0	1	0
AL022324.1	0.75	-0.415037	0.8724887	4	0	0	0	0	3
AC239600.1	0.9230769	-0.115477	0.9432635	5	3	5	0	3	9
ERVFRD-1	2.2126133	1.1457514	1.08E-24	2366	2089	2934	5207	5897	5245
AC104131.1	1.1090909	0.1493776	0.6335494	27	41	42	33	47	42
HTT	0.8885062	-0.170546	0.3696745	3727	3951	3937	2358	3866	4096
AP2S1	0.9935975	-0.009267	0.6730972	1497	1959	2323	2134	1923	1685
RPS16P5	0	#NUM!	0.824326	0	0	1	0	0	0
HSD17B7P2	3.8181818	1.9328858	0.002651	5	7	10	20	21	43
SH2D7	0.7142857	-0.485427	0.5961053	9	12	7	1	11	8
AC012485.1	0.3333333	-1.584963	0.3710082	4	2	6	0	4	0
AC012485.3	1	0	0.9755406	4	2	0	4	1	1
SH2D6	0.2222222	-2.169925	0.1446917	3	4	11	1	2	1
SH2D5	1.1481481	0.1993088	0.7798135	9	6	12	6	19	6
AP2M1	0.879886	-0.184611	0.3111322	9792	11943	13007	9847	10821	9901
SLC38A1	1.0982686	0.1352309	0.2577408	6347	4400	6349	4438	6967	7371
SNRNP200	0.72284	-0.468252	0.0007346	17402	20246	21253	11242	15289	16045
SLC38A7	0.8147089	-0.295643	0.0882412	1285	1269	1294	824	1074	1237

SLC38A6	0.8590604	-0.219169	0.4614272	314	234	346	253	314	201
SLC38A9	1.0377153	0.0534107	0.4767923	3202	2765	3207	2425	3594	3501
SLC38A3	4.2558824	2.0894583	2.75E-29	98	121	121	439	541	467
SLC38A2	1.1403897	0.1895269	0.1551106	6262	4338	6132	4390	7231	7460
SLC38A5	0	#NUM!	0.2218177	2	0	5	0	0	0
SLC38A4	1.8054363	0.8523475	3.42E-05	272	177	250	296	446	520
GNL3L	0.6490471	-0.623605	0.008552	795	1112	874	332	662	811
AC027559.1	0.8571429	-0.222392	0.8465005	21	16	33	28	17	15
UBLCP1	1.0974707	0.1341824	0.2361327	1225	840	1177	988	1392	1178
AC084149.1	1.6666667	0.7369656	0.6309225	0	4	2	10	0	0
LARP1P1	1	0	0.9890559	1	0	0	0	0	1
AC006111.2	0.4590164	-1.123382	0.1390961	18	18	25	4	6	18
AC006111.3	#DIV/0!	#DIV/0!	0.8030358	0	0	0	0	0	1
AC096642.1	1.8235294	0.8667335	0.3606139	4	4	9	3	21	7
L3MBTL4	0.8333333	-0.263034	0.8724127	6	0	0	0	4	1
AC016598.2	0	#NUM!	0.5387425	3	0	0	0	0	0
SH2B3	0.8118196	-0.300769	0.2489695	197	204	242	141	173	208
SH2B2	1.1982138	0.2608853	0.0562898	1026	1075	1370	1618	1483	1058
SH2B1	0.8419576	-0.248181	0.1646513	986	1053	1169	861	1021	819
AC008894.2	0.4347826	-1.201634	0.2780889	9	11	3	1	4	5
AL359265.3	1.3333333	0.4150375	0.6817898	11	0	4	9	7	4
AC008894.3	0.3333333	-1.584963	0.0562436	19	18	23	10	9	1
AL359265.1	2.6666667	1.4150375	0.4632014	2	0	1	8	0	0
AC016597.1	0	#NUM!	0.5643268	1	0	2	0	0	0
AHCYL2	0.857868	-0.221172	0.319457	504	491	581	338	458	556
AHCYL1	0.9052087	-0.143678	0.3986567	8961	9239	10041	6809	9362	9393
AC011511.5	#DIV/0!	#DIV/0!	0.8030358	0	0	0	1	0	0
AL592301.1	0	#NUM!	0.5436524	2	0	1	0	0	0
AC011511.2	#DIV/0!	#DIV/0!	0.1903651	0	0	0	7	0	0
TSSK5P	0.5833333	-0.777608	0.2851765	15	22	23	12	7	16
HNF1B	0.375	-1.415037	0.4981814	5	3	0	2	0	1
PPP1R3G	0.592233	-0.755763	0.2667562	9	37	57	13	21	27
PPP1R3F	0.5084746	-0.975752	0.0491043	40	35	43	16	24	20
STX1B	0	#NUM!	0.2084189	0	3	4	0	0	0
PPP1R3C	0	#NUM!	0.4374838	0	4	0	0	0	0
PPP1R3B	0.8851207	-0.176054	0.3744719	1065	1100	1108	715	1041	1141
PPP1R3E	0.7870091	-0.345548	0.2859424	209	142	311	147	170	204
PPP1R3D	0.8115061	-0.301326	0.0971061	642	666	830	469	613	653
PPP1R42	2	1	0.8324799	1	0	0	0	2	0
VSIG10L2	#DIV/0!	#DIV/0!	0.8030358	0	0	0	1	0	0
STX1A	0.7974277	-0.326574	0.3335456	121	86	104	74	90	84
L3MBTL1	0.9368421	-0.094122	0.9979784	169	86	220	166	163	116
PPP1R37	0.9691828	-0.045159	0.8804006	829	927	1132	911	990	898
PPP1R36	1.1481481	0.1993088	0.8328288	12	2	13	0	15	16
L3MBTL3	0.8297716	-0.269214	0.2344812	517	387	453	285	388	453
L3MBTL2	0.8544124	-0.226996	0.2966322	680	850	929	709	739	653
PPP1R32	0.8275862	-0.273018	0.8227678	13	20	25	27	8	13
PPP1R35	0.9861432	-0.020131	0.8149553	165	96	172	167	127	133
PPP1R3A	0	#NUM!	0.824326	1	0	0	0	0	0
PPP1R26	0	#NUM!	0.824326	0	0	1	0	0	0
PPP1R27	0.7258065	-0.462343	0.611472	32	12	18	27	13	5
PPP1R21	1.1289676	0.1750041	0.0848059	1045	826	1122	1012	1218	1149
ICK	1.002521	0.0036325	0.7982336	1813	1216	1731	1090	1747	1935
PPP1R1A	0.25	-2	0.6199202	0	0	4	0	1	0

ID2	1.2412226	0.3117619	0.0080415	737	754	930	988	1117	900
PPP1R1C	1.6141304	0.6907572	0.0126937	56	64	64	85	108	104
ID1	1.1059028	0.1452246	0.3243639	180	191	205	199	233	205
PPP1R1B	3	1.5849625	0.512377	2	0	0	6	0	0
ID4	0.7391304	-0.436099	0.6787996	7	7	9	2	7	8
ID3	0.722377	-0.469176	0.0347793	316	344	417	276	271	231
ANKRD18B	#DIV/0!	#DIV/0!	0.1252473	0	0	0	0	0	10
ANKRD18A	1.7535211	0.8102548	0.0214703	54	24	64	73	111	65
AHCYP2	0	#NUM!	0.824326	1	0	0	0	0	0
AP004833.2	#DIV/0!	#DIV/0!	0.6411933	0	0	0	0	0	2
PPP1R11	0.8751009	-0.192479	0.26823	1807	2004	2386	1656	1931	1836
AHCYP7	0	#NUM!	0.824326	0	0	1	0	0	0
PPP1R10	0.7031357	-0.508125	0.0002395	2650	3085	3067	1861	2202	2126
HNF4G	0.8636364	-0.211504	0.7496904	28	21	39	11	34	31
KIF14	1.1321435	0.1790569	0.2673758	2092	1071	2104	1331	2327	2305
AP2A1	0.8544268	-0.226971	0.1819487	5396	6756	7467	5278	6002	5483
KIF12	1.5576923	0.6394103	0.0938816	29	34	41	40	77	45
IDE	1.1263244	0.1716224	0.1857828	851	600	1003	678	1039	1047
KIF11	1.4903475	0.5756487	0.0003429	1788	1011	1863	1856	2763	2329
AP2A2	0.9795218	-0.029851	0.8050873	2458	2908	3375	2587	3082	2893
KIF17	1.0833333	0.1154772	0.7142188	18	5	25	31	10	11
PPP1R18	1.1027308	0.1410807	0.2907998	212	262	295	250	305	293
KIF15	1.3693972	0.453541	0.03279	486	216	592	439	703	630
SPANXN1	0	#NUM!	0.824326	1	0	0	0	0	0
SPANXN5	9.5	3.2479275	0.0486671	2	0	0	5	9	5
HNF4A	4	2	0.205709	1	0	3	6	10	0
AC080037.1	0.6428571	-0.63743	0.6448588	2	12	0	3	5	1
IDS	0.7139785	-0.486047	0.0269131	522	446	427	229	355	412
KIF1C	0.8797519	-0.184831	0.3056163	3598	4358	4460	3449	3752	3722
KIF1B	0.9985478	-0.002097	0.8306893	1147	1070	1226	772	1180	1486
KIF1A	2.1428571	1.0995357	0.3926547	3	1	3	13	2	0
NDEL1	1.1595689	0.2135885	0.0308824	1110	1212	1482	1298	1517	1596
AL034379.1	1.375	0.4594316	0.5079427	8	5	11	21	2	10
ZNRF3-IT1	0.6666667	-0.584963	0.8871197	0	0	3	0	2	0
KIF25	0.7959184	-0.329308	0.7000531	25	5	19	9	21	9
KIF24	1.0014451	0.0020833	0.7983466	434	460	490	343	470	573
AC018511.1	0	#NUM!	0.6385096	0	2	0	0	0	0
KIF23	1.0371773	0.0526626	0.4556464	2552	1671	2905	2067	2795	2531
KIF22	0.9200395	-0.120232	0.6617196	3163	3646	4334	3350	3591	3311
MMEL1	1.5	0.5849625	0.5134898	19	3	2	13	9	14
KIF27	1.2784314	0.3543747	0.2164911	116	40	99	111	110	105
OOEP	0.6299213	-0.666757	0.1816629	35	37	55	24	24	32
KIF2A	1.087352	0.120819	0.3432155	2304	1380	2143	1544	2359	2433
KIF2C	0.9995678	-0.000624	0.6055334	2804	3027	3425	2957	3207	3088
STX12	0.9900415	-0.014439	0.7222433	1184	1042	1389	1050	1269	1260
AC074194.2	0	#NUM!	0.5622741	0	1	2	0	0	0
LANCL1-AS1	1	0	0.937228	10	5	4	11	4	4
STX17	1.1197411	0.1631652	0.1702855	849	705	918	692	1011	1065
STX16	0.9375663	-0.093007	0.7931701	1798	1657	2199	1629	1929	1743
STX19	1	0	0.9680135	6	0	0	0	4	2
STX18	0.9416244	-0.086776	0.8616028	1106	1063	1377	1069	1203	1067
AC102953.1	#DIV/0!	#DIV/0!	0.8030358	0	0	0	0	1	0
XIAP	1.0569501	0.0799072	0.4744659	1382	983	1340	930	1354	1632
AC102953.2	0.5867769	-0.769116	0.1231698	34	51	36	13	30	28

FUOM	1.7701493	0.823871	0.0003068	105	111	119	228	184	181
AC011897.1	#DIV/0!	#DIV/0!	0.6411933	0	0	0	0	0	2
STX11	1.6466165	0.7195046	0.0286918	44	46	43	64	66	89
AC091212.1	#DIV/0!	#DIV/0!	0.2546293	0	0	0	1	5	0
STX10	1.1006711	0.1383835	0.21575	363	360	469	455	443	414
CCNJP2	#DIV/0!	#DIV/0!	0.3981469	0	0	0	4	0	0
AP2B1	0.8616778	-0.21478	0.1382374	6116	6415	7126	4657	6198	6083
AC016691.1	#DIV/0!	#DIV/0!	0.8030358	0	0	0	0	0	1
FOXD2-AS1	1.0495726	0.069802	0.506052	179	147	259	194	195	225
ERCC6L	1.0625301	0.0875037	0.3946945	752	497	830	591	823	795
IHH	4	2	0.386915	0	2	0	8	0	0
AL110292.1	1.0444444	0.0627358	0.8106312	11	18	16	26	6	15
LRRC37A9P	1.0784314	0.1089344	0.9122928	22	20	9	3	16	36
KIF5C	1.1693811	0.2257452	0.3275674	114	80	113	87	148	124
KIF5B	1.2396022	0.3098772	0.047463	3434	1938	3476	2629	4090	4249
KIF5A	1.46875	0.5545889	0.4060128	12	6	14	8	16	23
G6PD	0.7401685	-0.434074	0.0045795	2660	3230	3366	2242	2484	2125
AP000866.2	1.2711864	0.3461756	0.4246621	13	16	30	30	25	20
AP000866.1	0.7555556	-0.40439	0.6905898	15	7	23	0	12	22
AL845331.3	#DIV/0!	#DIV/0!	0.6554062	0	0	0	0	2	0
AP000866.6	0.8095238	-0.304855	0.6643716	26	15	43	13	35	20
AP000866.5	1.3846154	0.4694853	0.5638203	5	12	9	6	21	9
AP000866.4	0.8333333	-0.263034	0.8981925	4	0	2	0	2	3
RNU6ATAC35	0.25	-2	0.6199202	0	0	4	0	1	0
FUT2	0.52	-0.943416	0.1870986	11	22	42	6	19	14
FUT1	1.0344828	0.0489096	0.9040522	3	17	9	11	8	11
LRRC37A6P	1.5	0.5849625	0.7308627	1	3	2	0	4	5
FUT4	0.5636523	-0.827123	0.0001246	335	383	421	142	272	228
FUT3	0.5526316	-0.85561	0.3256978	11	17	10	1	10	10
FUT6	0	#NUM!	0.6631647	1	0	1	0	0	0
FUT8	1.1850519	0.2449502	0.0372559	2613	2051	2561	2198	3236	3128
KIF3B	0.9137856	-0.130072	0.5149657	1560	1402	1608	1132	1543	1501
FUT9	0.90625	-0.142019	0.9591812	9	6	17	10	9	10
KIF3A	0.942996	-0.084676	0.8923094	875	449	939	517	843	774
KIF3C	0.9244876	-0.113274	0.7578268	313	294	320	281	289	287
PHF10	0.8860732	-0.174502	0.392232	1513	1211	1814	1116	1519	1386
PHF12	0.9081386	-0.139016	0.562223	1548	1513	1903	1375	1442	1691
PHF11	1.1916058	0.2529071	0.0732184	403	301	392	424	447	435
AGBL5-AS1	2	1	0.5487978	1	2	1	4	0	4
RYKP1	1.5	0.5849625	0.8552295	1	1	0	0	0	3
LRRC37A5P	1.2619048	0.335603	0.5289027	9	16	17	28	17	8
IL4	0	#NUM!	0.6840298	0	0	2	0	0	0
IL3	0	#NUM!	0.4460243	4	0	0	0	0	0
IL6	1.0277778	0.0395284	0.8590196	24	18	30	18	32	24
IL5	#DIV/0!	#DIV/0!	0.2116454	0	0	0	5	1	0
IL7	0.125	-3	0.2471647	5	0	3	0	0	1
KIF4B	0.2727273	-1.874469	0.1383995	7	5	10	2	3	1
AL136366.1	0	#NUM!	0.824326	1	0	0	0	0	0
KIF4A	0.978063	-0.032001	0.8398084	2751	2687	3132	2475	3003	2904
AL445430.1	0	#NUM!	0.824326	1	0	0	0	0	0
PHF14	1.1300871	0.1764339	0.1382205	2048	1250	2329	1927	2387	2045
PHF13	1.0709647	0.0989109	0.2054781	1875	2151	2287	2041	2423	2297
PHF19	1.2083333	0.2730185	0.5998681	20	33	19	17	40	30
ILK	0.4545455	-1.137504	0.1767846	16	14	14	4	4	12

PPP1R9A	1.0973574	0.1340335	0.4524284	277	151	291	177	302	310
PPP1R9B	1.0211335	0.0301715	0.4902638	2145	2531	2611	2367	2552	2522
CNPPD1	0.9867978	-0.019174	0.7343858	967	1213	1380	1266	1174	1073
AC136944.3	#DIV/0!	#DIV/0!	0.4785195	0	0	0	1	1	1
AL591368.1	0.5384615	-0.893085	0.6053028	2	1	10	2	2	3
RPL6P12	#DIV/0!	#DIV/0!	0.6411933	0	0	0	0	0	2
BX088645.1	0.6428571	-0.63743	0.5850554	17	2	9	9	4	5
AC012005.1	0	#NUM!	0.824326	0	0	1	0	0	0
PDLIM1	0.8913768	-0.165893	0.4737951	5918	7122	7950	6514	6664	5532
AL136126.1	0.5	-1	0.812391	0	2	0	0	1	0
PDLIM3	1.9481481	0.9621034	0.0050039	54	27	54	94	66	103
PDLIM2	2.0625	1.0443941	0.0018411	35	26	51	68	85	78
CIR1P2	0	#NUM!	0.6840298	0	0	2	0	0	0
TAS1R2	0	#NUM!	0.6840298	0	0	2	0	0	0
TAS1R1	2.6666667	1.4150375	0.5149152	2	1	0	0	5	3
AC084879.1	#DIV/0!	#DIV/0!	0.8030358	0	0	0	0	1	0
PDLIM5	1.3716349	0.4558966	0.0012223	496	486	541	537	814	738
PDLIM4	6	2.5849625	0.3098729	0	0	1	1	1	4
TAS1R3	0.6	-0.736966	0.6264515	5	1	9	0	7	2
PDLIM7	0.9565046	-0.064156	0.9332407	1552	1598	1931	1902	1636	1322
RPL6P27	1.0783249	0.108792	0.1997248	1664	1426	2068	1918	1989	1655
U2SURP	1.0029103	0.0041926	0.7603514	4426	2284	4629	2763	4494	4115
PMFBP1	0.78	-0.358454	0.618924	14	18	18	7	20	12
ACAP2-IT1	1.2666667	0.3410369	0.7124138	5	1	9	3	10	6
IPP	0.8063241	-0.310568	0.2116551	382	289	341	185	281	350
CHKB-CPT1B	0.4285714	-1.222392	0.4241031	8	2	4	0	1	5
NUDT16P1	#DIV/0!	#DIV/0!	0.8030358	0	0	0	0	1	0
AC233728.1	1.9090909	0.9328858	0.4525227	2	4	5	0	10	11
EARS2	0.740866	-0.432715	0.0026795	1479	1425	1530	934	1251	1100
PRIMA1	0.5925926	-0.754888	0.0387718	73	118	133	53	52	87
BIN3	0.7918623	-0.336679	0.2248885	182	198	259	174	163	169
BIN2	1.4254545	0.511422	0.0336473	93	92	90	132	144	116
BIN1	0.6626506	-0.59368	9.22E-05	1191	1316	1477	873	935	832
MAPKAP1	1.0716277	0.0998038	0.1685484	3340	3725	4411	3775	4585	3938
AL023881.1	0	#NUM!	0.824326	1	0	0	0	0	0
AL079305.1	0.1111111	-3.169925	0.1700945	3	2	4	0	0	1
ISX	0.8476395	-0.238477	0.4154873	142	161	163	114	156	125
AC009148.1	0.7446809	-0.425306	0.6174743	12	16	19	14	9	12
ITK	0.4	-1.321928	0.6181888	4	0	1	0	0	2
SPIN4	0.8214177	-0.283812	0.1479446	1212	986	1089	618	1002	1080
SPIN3	0.9142674	-0.129312	0.6785918	541	396	626	399	536	494
RPS15P9	0.4444444	-1.169925	0.5783168	2	6	1	4	0	0
AP002770.2	3	1.5849625	0.4907591	2	0	0	4	0	2
PIP4P2	0.7901786	-0.339749	0.3626441	78	74	72	44	62	71
CSRP2	1.0681075	0.0950568	0.2420173	2126	2312	2595	2610	2684	2218
PIP4P1	0.7794321	-0.359505	0.0179605	1687	1949	2140	1431	1561	1510
CSRP1	0.9589694	-0.060443	0.9925261	8297	9812	10845	8690	9829	9247
SPIN1	1.0974495	0.1341545	0.2430259	5144	4517	5434	3814	6179	6573
AP3S2	0.886058	-0.174527	0.4437274	452	511	450	377	460	415
AP3S1	0.9405183	-0.088472	0.7599018	2095	2154	2543	1850	2466	2072
AL354719.2	0.5	-1	0.7359052	1	2	1	2	0	0
NUDT16L1	0.9022127	-0.14846	0.6644569	381	517	503	461	426	377
RPS15P5	0	#NUM!	0.824326	1	0	0	0	0	0
FALEC	0.6451613	-0.632268	0.5448897	11	5	15	8	8	4

IVD	0.8	-0.321928	0.6786346	11	19	20	7	14	19
IVL	0.0882353	-3.5025	0.0094496	20	4	10	1	0	2
AC099677.1	0.84	-0.251539	0.7483607	25	10	15	5	13	24
AC099677.4	4	2	0.5246085	1	0	0	0	1	3
AP002530.1	0	#NUM!	0.5781912	0	0	3	0	0	0
IYD	0	#NUM!	0.084153	3	7	0	0	0	0
SPART-AS1	1.9285714	0.9475326	0.2913772	7	3	4	14	6	7
TPD52	1.0160133	0.0229192	0.5770781	1312	961	1349	1119	1393	1168
ANKRD36C	0.8837209	-0.178337	0.7313925	76	32	64	30	70	52
BIVM	0.5826087	-0.779401	0.1224033	37	41	37	15	34	18
XIST	2.25	1.169925	0.592433	2	2	0	0	0	9
TTPAL	0.8245756	-0.278276	0.1536645	754	768	952	494	727	819
ANKRD36B	1.1508621	0.2027149	0.4011605	79	73	80	71	88	108
VTCN1	0.8897216	-0.168574	0.4389955	630	578	660	515	628	519
ANKRD34A	1.3461538	0.4288433	0.4808079	32	3	17	22	26	22
SLC37A2	0.6666667	-0.584963	0.8537226	2	0	1	0	0	2
SLC37A1	1.1328725	0.1799855	0.1374509	377	431	494	461	557	457
AL589743.4	2.5	1.3219281	0.414668	0	2	2	2	2	6
AL589743.5	1.2045455	0.2684888	0.6340489	16	13	15	17	27	9
AC009242.1	0.1578947	-2.662965	0.0894471	8	7	4	0	0	3
AL589743.2	1	0	0.9890559	0	0	1	0	0	1
AL589743.1	#DIV/0!	#DIV/0!	0.1161291	0	0	0	1	4	3
AP003025.2	0	#NUM!	0.4185478	3	0	1	0	0	0
ANKRD33B	0.8659907	-0.207576	0.2763491	2765	3157	3152	1750	2968	3140
SYCP2	2.0674157	1.0478285	0.021048	33	8	48	51	82	51
SYCP1	0.1666667	-2.584963	0.3630032	4	0	2	0	1	0
DNALI1	1.7058824	0.7705182	0.1907486	12	27	12	18	25	44
OPA1	0.9932338	-0.009795	0.7639744	1631	937	1718	1185	1509	1563
OPA3	0.8766311	-0.189958	0.2850447	1506	1692	1860	1373	1547	1514
SLC37A4	0.68	-0.556393	0.0006124	707	730	788	458	582	473
SLC37A3	0.9253459	-0.111935	0.6043568	1215	1281	1335	954	1377	1214
AC233968.1	1.6153846	0.6918777	0.5487067	5	5	3	1	7	13
PLPBP	0.8531309	-0.229161	0.1997251	867	769	999	669	828	751
AL032819.2	0	#NUM!	0.4217812	0	0	5	0	0	0
AP3M2	0.8047493	-0.313389	0.2112376	272	223	263	205	215	190
AP3M1	1.1479468	0.1990558	0.0782523	2547	1884	2558	2106	3074	2843
AC097376.1	1	0	0.9252623	1	2	2	5	0	0
ANKRD30B	#DIV/0!	#DIV/0!	0.8030358	0	0	0	0	0	1
WDR82P2	0	#NUM!	0.824326	0	1	0	0	0	0
ANKRD30A	0.6666667	-0.584963	0.8600781	4	0	2	4	0	0
WDR82P1	0	#NUM!	0.824326	1	0	0	0	0	0
CDC42BPG	0.805007	-0.312927	0.0294768	3143	3621	4021	2658	3192	2832
AC097376.2	1.1848739	0.2447336	0.3243892	81	44	113	112	99	71
CDC42BPB	0.7167546	-0.480449	0.0061636	3684	4041	4021	1879	3029	3511
CFLAR	0.7036082	-0.507156	0.0082977	480	455	617	295	365	432
CDC42BPA	0.9351899	-0.096669	0.7995501	800	440	735	427	713	707
TPCN2	0.9213883	-0.118119	0.624336	1026	1131	1214	954	1113	1039
TPCN1	0.7288765	-0.456254	0.0067622	665	624	865	460	597	513
SYCP3	0.2162162	-2.209453	0.0397466	17	4	16	3	2	3
AC027808.2	0	#NUM!	0.5075476	0	0	4	0	0	0
BOLA1	1.7017544	0.7670228	0.1200211	13	9	35	30	38	29
PLPP7	0.6358025	-0.653349	0.1610885	43	50	69	17	55	31
PLPP6	0.8826847	-0.18003	0.3986922	535	607	631	470	550	545
PLPP5	0.7338195	-0.446503	0.029504	334	326	437	218	326	261

PLPP4	0	#NUM!	0.824326	0	1	0	0	0	0
BOLA3	1.1958352	0.2580186	0.0331536	630	748	879	923	953	823
GRWD1	0.7428365	-0.428883	0.0094345	1483	1882	2184	1334	1451	1337
BOLA2	0	#NUM!	0.2904576	0	1	5	0	0	0
PLPP3	1.3098039	0.3893509	0.0010912	543	556	686	721	826	791
PLPP2	0	#NUM!	0.5781912	0	0	3	0	0	0
PLPP1	1.0461049	0.0650276	0.628828	154	256	219	191	242	225
AC016596.1	1.1	0.1375035	0.5624963	58	77	65	72	63	85
AC021945.1	0.8333333	-0.263034	0.7802018	19	7	16	7	9	19
PTDSS2	0.9425982	-0.085285	0.9499927	1250	1589	1795	1617	1452	1299
PTDSS1	0.8503759	-0.233827	0.1851981	1479	1925	1916	1216	1738	1570
SGPL1	0.9986441	-0.001958	0.7925362	902	1013	1035	746	1050	1150
PPP4R1-AS1	0.6666667	-0.584963	0.8693987	2	1	0	2	0	0
IMPDH1P8	1.2666667	0.3410369	0.5571454	6	17	22	11	27	19
SESTD1	1.3620292	0.4457576	0.0005625	1396	1096	1411	1376	1950	1990
IMPDH1P4	1	0	0.9890559	0	1	0	1	0	0
IMPDH1P6	1	0	0.9602092	4	0	0	4	0	0
IMPDH1P5	1.1848341	0.2446851	0.3345433	58	75	78	68	90	92
DAZAP2	0.7371734	-0.439924	0.004868	951	1029	1197	709	905	728
DAZAP1	1.0098083	0.0140815	0.5218081	6001	6511	7471	6802	7172	6205
HILPDA	1.1301352	0.1764953	0.0937205	1108	1278	1387	1472	1500	1292
AC078795.1	0.5151515	-0.956931	0.2908284	11	12	10	4	5	8
AP3B1	1.0285894	0.0406673	0.5166102	3395	2396	3723	2540	3617	3629
AP3B2	0.2769231	-1.852443	0.0153639	19	22	24	8	3	7
CEACAM1	1.0063291	0.0091022	0.7826762	101	99	116	110	110	98
CEACAM7	#DIV/0!	#DIV/0!	0.8030358	0	0	0	0	1	0
AC093752.2	0.0833333	-3.584963	0.0964122	6	1	5	0	0	1
ANKMY2	1.0195962	0.0279979	0.6425419	673	389	622	513	575	629
AC110048.2	1.25	0.3219281	0.8637128	7	0	1	1	7	2
AC110048.1	0	#NUM!	0.284725	6	0	0	0	0	0
AC007336.1	3	1.5849625	0.0591501	3	4	6	14	16	9
JHY	1.75	0.8073549	0.6846877	1	2	1	0	4	3
AC093752.1	0.8427518	-0.24682	0.4710547	161	119	127	50	154	139
ANKMY1	0.9213075	-0.118245	0.7538486	237	271	318	235	276	250
IGHV5-78	0	#NUM!	0.824326	1	0	0	0	0	0
AP3D1	0.9260593	-0.110824	0.6690861	5582	6545	7037	5704	6164	5879
AL772337.4	1.4444444	0.5305147	0.5064549	4	5	9	8	5	13
AL772337.3	0.7857143	-0.347923	0.8439719	3	1	10	1	6	4
AL772337.2	0.5	-1	0.6341511	6	2	0	3	0	1
AL772337.1	0.2727273	-1.874469	0.2966649	6	4	1	2	1	0
RAB3GAP2	1.0883523	0.1221457	0.3111102	1419	901	1449	1022	1523	1557
AL162274.2	1.56	0.641546	0.3774778	3	9	13	9	15	15
ZNF354A	1.1512821	0.2032413	0.1755903	1216	656	1248	930	1433	1229
AL162274.1	3.6296296	1.8598223	0.0055871	9	2	16	13	43	42
TMEM212-AS	#DIV/0!	#DIV/0!	0.8030358	0	0	0	1	0	0
RAB3GAP1	1.1070053	0.1466621	0.1693972	2022	1535	2153	1633	2272	2416
ZNF354C	0.4444444	-1.169925	0.5379923	5	2	2	0	0	4
AL162274.3	0	#NUM!	0.2325162	2	4	0	0	0	0
ZNF354B	1.1353887	0.1831863	0.2826831	610	275	607	457	664	573
SYDE2	0.9589989	-0.060399	0.8885014	649	576	653	408	686	707
SYDE1	1.047235	0.0665853	0.4131481	512	589	635	592	658	568
AC114744.1	0	#NUM!	0.824326	1	0	0	0	0	0
AF186192.2	#DIV/0!	#DIV/0!	0.6099294	0	0	0	2	0	0
BTF3P10	0	#NUM!	0.6385096	0	2	0	0	0	0

BTF3P13	4	2	0.5532124	1	0	0	0	0	4
BTF3P12	0.5	-1	0.8652929	0	0	2	0	1	0
AP001453.3	0.5	-1	0.5391045	1	5	6	0	2	4
AP001453.2	0.1666667	-2.584963	0.3847728	2	0	4	0	1	0
AP001453.1	0.7142857	-0.485427	0.7992973	1	6	0	0	0	5
AC091691.1	0	#NUM!	0.4374838	0	4	0	0	0	0
AL713851.1	0	#NUM!	0.5387425	3	0	0	0	0	0
CASKIN2	0.8093977	-0.305079	0.1242302	420	511	580	352	442	429
CASKIN1	1.1133333	0.1548856	0.4668897	56	106	138	96	102	136
AL512625.1	0.5294118	-0.917538	0.5631021	2	6	9	5	0	4
AL512625.3	2	1	0.8324799	1	0	0	0	2	0
OPN3	0.7359307	-0.442358	0.2571638	70	63	98	55	66	49
RNU6ATAC24	0	#NUM!	0.824326	1	0	0	0	0	0
OPN5	0	#NUM!	0.824326	0	1	0	0	0	0
AC016700.3	#DIV/0!	#DIV/0!	0.8030358	0	0	0	0	1	0
AC016700.2	0.0909091	-3.459432	0.1135086	1	6	4	0	0	1
JMY	1.3138253	0.3937734	0.0124594	872	596	890	727	1186	1185
C10orf111	1.5	0.5849625	0.5114168	1	3	10	9	8	4
AC034229.4	0.6666667	-0.584963	0.8600781	4	0	2	4	0	0
AC079779.3	1	0	0.9861498	0	2	0	0	0	2
AC079779.2	1.3076923	0.3870231	0.6569502	0	3	10	9	1	7
SLC7A11-AS1	3.5	1.8073549	0.4127083	1	1	0	2	0	5
BDKRB2	0.7	-0.514573	0.1801488	73	64	63	41	57	42
BDKRB1	#DIV/0!	#DIV/0!	0.6411933	0	0	0	0	0	2
TAS2R1	12	3.5849625	0.0937656	1	0	0	11	0	1
C10orf128	1	0	0.9956048	0	1	1	0	2	0
TAS2R3	#DIV/0!	#DIV/0!	0.6411933	0	0	0	0	0	2
TAS2R5	0.6666667	-0.584963	0.8498166	1	0	5	0	0	4
TAS2R4	1.3333333	0.4150375	0.7554714	1	0	5	1	2	5
AL035458.1	0.5	-1	0.8276497	1	0	1	0	0	1
C10orf143	3.5555556	1.830075	0.082563	1	0	8	10	14	8
JPX	5	2.3219281	0.0835876	1	0	3	5	5	10
S1PR3	1	0	0.9710643	0	0	4	0	4	0
S1PR2	1.1130435	0.1545099	0.1466417	605	757	823	754	839	839
S1PR5	0.5869565	-0.768674	0.3483569	15	21	10	7	4	16
S1PR4	#DIV/0!	#DIV/0!	0.8030358	0	0	0	0	0	1
KDF1	0.9648594	-0.051609	0.9591343	280	385	331	282	385	294
SGPP2	1.2162162	0.2823997	0.680642	10	12	15	4	17	24
SGPP1	1.2224532	0.2897793	0.0202646	3315	2949	3356	2860	4306	4594
AC074099.1	2.8888889	1.5305147	0.128611	4	2	3	9	9	8
JRK	0.6590179	-0.60161	0.001075	689	769	721	340	570	526
AP001893.1	1	0	0.9103134	10	5	21	6	8	22
AP001893.3	#DIV/0!	#DIV/0!	0.6411933	0	0	0	0	0	2
AL512378.1	0	#NUM!	0.4374838	0	4	0	0	0	0
AC004490.1	5	2.3219281	0.4840007	1	0	0	0	5	0
ANKFY1	0.824772	-0.277933	0.1288635	2177	2090	2313	1254	1924	2249
HEMK1	0.9398173	-0.089548	0.8536375	598	584	679	585	613	551
CXADR	0.7886978	-0.342455	0.2039609	338	198	278	160	225	257
AC093890.1	0	#NUM!	0.6840298	0	0	2	0	0	0
EWSR1	0.8473536	-0.238964	0.0973527	9741	10537	12484	8643	9840	9278
OPTC	0.5	-1	0.7145709	4	0	0	0	2	0
JTB	0.8936392	-0.162236	0.4800342	1931	2285	2497	2046	2174	1779
OPTN	1.4944444	0.5796093	7.17E-07	1415	1253	1832	2222	2340	2163
RPS14P8	0.952381	-0.070389	0.959613	5	11	5	5	6	9

HLA-DRB5	#DIV/0!	#DIV/0!	0.8030358	0	0	0	1	0	0
JUN	0.9716446	-0.041499	0.9466921	162	171	196	151	179	184
JUP	1.139747	0.1887136	0.0407318	14878	17435	18672	19179	20664	18267
EIF2AK1	1.0128864	0.0184724	0.4629743	9320	9025	11221	8695	10774	10478
EIF2AK3	0.9922449	-0.011232	0.8634628	878	771	801	644	939	848
EIF2AK2	2.0856448	1.0604935	1.95E-13	2973	2142	3105	4190	6346	6608
nse to virus;GC	#VALUE!	#VALUE!	me;GO:0005	05164|hsa05	ction|Kapo	initiation factor 2 alpha kinase 2			
EIF2AK4	0.9242249	-0.113684	0.6143405	3474	3029	3883	2262	3760	3577
HLA-DRB1	1.6	0.6780719	0.3239391	13	3	14	22	5	21
KDM8	0.862069	-0.214125	0.6506719	115	69	106	95	85	70
TFCP2L1	0.9113645	-0.1339	0.5231035	2514	2712	2931	1802	2664	2968
NHSL2	0.9444444	-0.082462	0.9138153	11	17	8	6	14	14
NHSL1	1.0626667	0.0876891	0.3078903	716	671	863	650	876	865
NIFKP4	#DIV/0!	#DIV/0!	0.8030358	0	0	0	1	0	0
MUC20P1	0	#NUM!	0.824326	0	0	1	0	0	0
RNU6-479P	#DIV/0!	#DIV/0!	0.5371397	0	0	0	0	0	3
PABPC1P4	1	0	0.9890559	0	1	0	1	0	0
PABPC1P3	1.2	0.2630344	0.6855675	5	1	14	9	7	8
ABRACL	0.8920252	-0.164844	0.471268	1081	1151	1417	1086	1156	1013
PABPC1P2	0	#NUM!	0.3749909	2	1	1	0	0	0
PABPC1P1	0.1666667	-2.584963	0.3723105	3	0	3	0	1	0
ATAD1	1.3732644	0.4576094	0.0004732	1311	1000	1362	1299	1914	1831
ATAD5	1.0669291	0.0934644	0.520002	488	269	513	304	534	517
ATAD2	1.1105586	0.1512856	0.2907475	1372	669	1378	1020	1347	1430
AC091493.1	0.5625	-0.830075	0.6037242	5	2	9	2	0	7
AC083798.2	0.6707317	-0.576192	0.3229931	36	23	23	16	24	15
AC083798.1	0.625	-0.678072	0.6252079	8	2	6	4	2	4
AC034223.2	0.6	-0.736966	0.7640999	0	5	0	0	0	3
EFEMP2	1.0967742	0.1332665	0.8486061	11	10	10	4	6	24
EFEMP1	1.2307692	0.2995603	0.5033997	9	32	37	30	43	23
KMT5B	0.99549	-0.006521	0.6929358	1661	1378	1839	1402	1798	1656
KMT5A	0.9418042	-0.086501	0.8227281	3124	3155	3842	2977	3312	3243
RNU6-476P	0.7222222	-0.469485	0.7993661	8	2	8	8	0	5
TYRO3P	0.3333333	-1.584963	0.5170759	5	1	0	0	0	2
AC034223.1	#DIV/0!	#DIV/0!	0.6554062	0	0	0	0	2	0
UGT2B10	#DIV/0!	#DIV/0!	0.8030358	0	0	0	1	0	0
PCYT2	0.7404063	-0.433611	0.0208899	1154	1342	1491	1064	1056	832
UGT2B11	0	#NUM!	0.824326	0	1	0	0	0	0
KMT5C	0.975312	-0.036064	0.8288013	1079	1201	1406	1278	1292	1025
UGT2B15	0	#NUM!	0.824326	0	1	0	0	0	0
UGT2B17	0	#NUM!	0.6385096	0	2	0	0	0	0
RN7SL30P	0	#NUM!	0.5781912	0	0	3	0	0	0
AC004156.1	0.8181818	-0.289507	0.878123	6	0	5	2	7	0
KMT2E	1.0493781	0.0695345	0.4934506	923	611	1119	661	1088	1035
KMT2D	0.7177482	-0.47845	0.0155649	3555	4184	3772	1768	2785	3709
KMT2A	0.7558237	-0.403878	0.0678644	2129	1805	2033	858	1550	2102
KMT2C	0.9115789	-0.13356	0.5989046	2885	2087	2628	1370	2516	3042
KMT2B	0.8376585	-0.255566	0.1481927	1442	1822	1861	1131	1535	1627
AP4S1	0.9548023	-0.066726	0.9499028	227	223	258	205	250	221
KDSR	0.8786591	-0.186625	0.377111	624	644	850	543	683	635
IQCA1-AS1	0.375	-1.415037	0.5072213	1	7	0	0	0	3
BFSP2	0.75	-0.415037	0.9223671	0	0	4	0	1	2
BFSP1	0.9615385	-0.056584	0.9155445	0	5	21	9	7	9
AC016708.1	1	0	0.9993647	3	1	3	0	5	2

AC048338.2	3.5	1.8073549	0.4796955	0	2	0	1	6	0
AC007382.1	1.2066116	0.2709613	0.5232201	53	28	40	22	63	61
AP4M1	0.847302	-0.239052	0.3639489	276	275	320	255	238	245
OR5P4P	0	#NUM!	0.6436235	2	0	0	0	0	0
NKX3-2	1.1276596	0.1733316	0.5570369	60	112	63	90	59	116
NKX3-1	1	0	0.744482	138	148	193	174	132	173
RN7SL381P	0.8442623	-0.244237	0.6828676	38	28	56	13	43	47
AP003716.1	1.7741935	0.8271634	0.3056603	8	16	7	5	33	17
UGT2B28	0	#NUM!	0.824326	0	1	0	0	0	0
KC6	1.1851852	0.2451125	0.6265008	20	11	23	15	33	16
STARD7-AS1	0.9948718	-0.007417	0.8480059	61	65	69	77	57	60
SMIM10L1	0.8122833	-0.299945	0.1263058	642	566	811	511	633	496
STYK1	0.810219	-0.303616	0.2550505	176	182	190	128	184	132
MAP1LC3B2	1.4125	0.4982509	0.2646857	12	32	36	60	29	24
AL031779.1	0	#NUM!	0.4217812	0	0	5	0	0	0
KCP	1.3461538	0.4288433	0.5581641	5	15	6	18	9	8
AC006064.3	1.0333333	0.0473057	0.8090639	7	5	18	17	5	9
AC006064.2	0.88	-0.184425	0.8906224	4	8	13	4	11	7
PLEKHM1P1	0.8055944	-0.311874	0.2132366	207	226	282	162	196	218
KEL	0.5454545	-0.874469	0.5888782	4	3	4	2	4	0
AC068633.1	0	#NUM!	0.824326	0	0	1	0	0	0
RN7SL23P	0.4285714	-1.222392	0.5545532	4	3	0	0	3	0
AC074290.1	1	0	0.9890559	1	0	0	1	0	0
FAM9B	0	#NUM!	0.3049182	1	1	3	0	0	0
AL132989.1	1.1125	0.1538053	0.6515704	28	10	42	25	23	41
BRI3BP	0.8753914	-0.192	0.3877208	504	554	539	451	510	437
AC092821.1	6	2.5849625	0.3391613	0	0	1	6	0	0
AC092821.3	1.2040816	0.2679332	0.6576864	4	25	20	16	30	13
AP4E1	1.0323543	0.0459382	0.6217587	1753	1037	1568	1092	1699	1708
KHK	1.2569316	0.3299062	0.0546019	154	169	218	227	227	226
AL606468.1	0	#NUM!	0.2883218	0	0	7	0	0	0
PHGDH	1.4155255	0.5013377	3.14E-05	6779	8203	8824	11847	11526	10325
AC074050.4	#DIV/0!	#DIV/0!	0.8030358	0	0	0	0	0	1
KIN	1.2811804	0.3574736	0.0188861	642	395	759	768	782	751
ANKFN1	1.61	0.6870607	0.0135558	66	69	65	121	108	93
KIT	0.6666667	-0.584963	0.741579	5	1	3	2	1	3
AFAP1-AS1	0.3333333	-1.584963	0.1816132	19	4	4	4	1	4
KIZ	1.0751445	0.1045306	0.477998	283	157	252	219	271	254
SOX2-OT	0	#NUM!	0.1632507	4	0	4	0	0	0
AC105749.1	0.1428571	-2.807355	0.3884527	0	0	7	0	1	0
AC019109.1	1	0	0.9699945	2	0	0	2	0	0
SGSM1	0.8571429	-0.222392	0.9814849	0	2	5	6	0	0
SGSM3	0.625	-0.678072	0.6597241	8	3	5	0	0	10
SGSM2	1.0427807	0.0604359	0.412368	624	692	741	673	725	747
AC011753.4	1.4545455	0.5405684	0.7538573	6	5	0	0	1	15
AC011753.2	0	#NUM!	0.824326	0	0	1	0	0	0
AC011753.1	0	#NUM!	0.824326	0	0	1	0	0	0
KLB	0.4814815	-1.054448	0.3053579	9	10	8	2	3	8
AC093797.1	6	2.5849625	0.4126257	0	0	1	0	6	0
AC004980.2	0.6	-0.736966	0.7677886	4	0	1	1	0	2
AC004980.1	0.7486339	-0.417668	0.3580892	70	43	70	50	50	37
ZNF252P-AS1	0.7272727	-0.459432	0.716484	2	11	9	4	2	10
FAM3A	1.036783	0.052114	0.4062897	917	1025	1266	1206	1054	1066
FAM3C	0.8927232	-0.163715	0.5863602	1229	938	1832	820	1623	1127

MMGT1	0.9523487	-0.070438	0.8881017	1467	1290	1671	1143	1540	1534
TMCC1-AS1	1.2258065	0.2937312	0.5040504	14	17	31	22	34	20
ZNF322P1	0.6666667	-0.584963	0.6931752	7	1	4	2	4	2
KMO	0.6060606	-0.722466	0.467256	15	4	14	4	5	11
CCNB1IP1	0.8981648	-0.154948	0.3897413	3207	3227	3592	2855	3238	2912
FO393411.1	1.2571429	0.3301486	0.4252329	14	21	35	27	34	27
TSG101	0.9466557	-0.079088	0.8981532	1767	1897	2391	1800	2227	1705
INAVA	0.9036077	-0.146232	0.4306979	8166	10151	10150	7028	9328	9367
TCAF2	1.0583658	0.0818383	0.7022599	88	85	84	66	118	88
TCAF1	0.8728967	-0.196117	0.3059938	1331	1050	1482	927	1234	1211
AP4B1	0.7662162	-0.384177	0.1017415	232	224	284	167	190	210
AL513550.1	0.89	-0.168123	0.7591693	96	42	62	53	53	72
LMNB2	0.9450282	-0.081571	0.8376315	9635	11852	12585	9970	11420	10809
LMNB1	0.9365552	-0.094564	0.6928645	13893	15095	16658	12282	15221	15247
ANOS2P	0.5757576	-0.796467	0.2292271	32	15	19	9	14	15
MRPL20P1	0.5	-1	0.8638172	0	0	2	1	0	0
SFSWAP	0.9354992	-0.096192	0.8054392	1834	1932	2544	1840	2127	1936
LINC00266-4P	0.25	-2	0.547139	0	4	0	0	1	0
FAM241B	0.9893293	-0.015477	0.8293817	174	249	233	236	214	199
FAM241A	1.5196536	0.6037425	3.46E-05	531	444	526	614	916	751
AC022532.1	0.625	-0.678072	0.7001031	6	1	1	0	1	4
COL7A1	0.851626	-0.231708	0.4194643	263	295	426	277	303	258
AL356966.1	0.5925926	-0.754888	0.4274338	9	11	7	3	7	6
RNU6ATAC40	0	#NUM!	0.5387425	3	0	0	0	0	0
AC093901.1	1	0	0.9927105	3	6	6	0	5	10
XAGE1B	0	#NUM!	0.824326	0	0	1	0	0	0
KNSTRN	1.0320867	0.0455642	0.3707583	1445	1450	1811	1443	1737	1677
RPS13P2	1.6290323	0.7040152	0.1297981	22	20	20	44	33	24
AC091492.1	0.0625	-4	0.0713639	4	0	12	0	0	1
AC083799.1	1.0687831	0.0959691	0.4732023	178	128	261	167	207	232
SNORD3B-2	0	#NUM!	0.5387425	3	0	0	0	0	0
SNORD3B-1	1	0	0.9759299	5	0	2	2	2	3
AC233280.19	1.1142857	0.1561192	0.7878941	14	3	18	5	12	22
AC007383.2	1	0	0.9315358	27	17	21	22	29	14
AC007383.1	#DIV/0!	#DIV/0!	0.8030358	0	0	0	1	0	0
XKRX	3.75	1.9068906	0.2831043	0	3	1	0	11	4
OSER1-AS1	1.2142857	0.2801079	0.2212804	73	60	119	101	98	107
XKR8	0.9747102	-0.036955	0.9119344	278	317	354	285	346	294
XKR7	0.737037	-0.440191	0.2104299	73	102	95	45	75	79
XKR9	0.3492063	-1.517848	0.0371231	24	14	25	6	7	9
AL138962.1	0	#NUM!	0.355562	5	0	0	0	0	0
XKR4	#DIV/0!	#DIV/0!	0.1923616	0	0	0	0	2	5
XKR3	0.875	-0.192645	0.8906274	3	3	2	0	4	3
XKR6	0	#NUM!	0.6436235	2	0	0	0	0	0
FANK1	1.0100334	0.0144031	0.720678	88	80	131	128	94	80
PHF20	0.8888046	-0.170062	0.3449841	3501	3265	3783	2302	3465	3609
PHF23	0.9497147	-0.074434	0.9434591	1584	1877	2147	1772	1908	1646
AP001412.1	1	0	0.9987044	1	3	0	2	2	0
HELLS	1.285869	0.3627436	0.0257509	1454	773	1467	1283	1913	1554
MIR8055	#DIV/0!	#DIV/0!	0.6554062	0	0	0	0	2	0
USP30-AS1	#DIV/0!	#DIV/0!	0.4501902	0	0	0	0	0	4
DBH-AS1	#DIV/0!	#DIV/0!	0.3196487	0	0	0	0	1	4
KIFC2	1.1474359	0.1984136	0.1643532	265	309	362	393	315	366
KIFC1	0.9845822	-0.022416	0.7615772	3725	4378	4869	3895	4543	4334

AC010435.1	#DIV/0!	#DIV/0!	0.8030358	0	0	0	0	1	0
KIFC3	0.7336042	-0.446926	0.0398332	503	617	603	465	382	417
HLA-DQB1	0	#NUM!	0.0635539	4	4	2	0	0	0
AC073857.1	1.0775862	0.1078033	0.6249438	28	20	68	64	38	23
AP5Z1	0.8257202	-0.276275	0.1781714	1446	1662	1752	1452	1442	1119
AC083809.1	0	#NUM!	0.5337954	0	3	0	0	0	0
AL079307.1	1	0	0.9958612	5	3	3	1	6	4
AC100803.1	0	#NUM!	0.4460243	4	0	0	0	0	0
SLC8A1-AS1	1.1428571	0.1926451	0.9363018	1	4	2	0	7	1
PHF5A	0.7704419	-0.376242	0.0183053	1589	1963	2037	1363	1588	1355
AC100803.2	0.8571429	-0.222392	0.9859551	0	0	7	0	0	6
AL034370.1	0	#NUM!	0.1789363	2	0	6	0	0	0
MROH2A	0	#NUM!	0.284725	6	0	0	0	0	0
N6AMT1	0.8429542	-0.246474	0.2596944	394	333	451	263	379	351
CBWD4P	1.1	0.1375035	0.6650301	52	20	38	38	41	42
SLC26A11	0.8148335	-0.295423	0.1499115	602	587	793	543	524	548
AP5S1	0.8248276	-0.277836	0.297274	190	244	291	184	218	196
AC091982.2	0.5555556	-0.847997	0.5165405	13	4	1	1	9	0
AC091982.4	0.7131474	-0.487728	0.2478754	57	74	120	58	51	70
MROH3P	0.9166667	-0.125531	0.9897172	46	25	73	56	30	46
AC069222.1	1.1714286	0.228269	0.7661811	20	10	5	15	21	5
AC126696.3	0	#NUM!	0.6840298	0	0	2	0	0	0
AC126696.2	1.25	0.3219281	0.8377392	0	2	2	2	0	3
MIR8089	0.5714286	-0.807355	0.8505274	0	0	7	4	0	0
FAM149B1	1.2074789	0.271998	0.044252	590	450	618	547	802	653
ORC5	0.9640288	-0.052852	0.9289571	832	678	992	750	861	801
ORC4	1.1804227	0.2393036	0.0411364	1348	1025	1601	1241	1786	1664
RN7SL395P	#DIV/0!	#DIV/0!	0.8030358	0	0	0	0	1	0
ORC6	0.9637384	-0.053287	0.9029333	2257	2177	2874	2308	2474	2261
ORC1	0.890625	-0.16711	0.4032243	1476	1408	1852	1310	1441	1467
ORC3	1.0623044	0.0871972	0.3675005	1286	847	1382	1142	1491	1101
ORC2	1.0384822	0.0544765	0.4575859	1209	1119	1388	983	1481	1395
AC132938.1	5	2.3219281	0.4088931	0	1	0	5	0	0
FANCI	0.8885424	-0.170487	0.3837773	3071	2329	3258	1956	2927	2810
OSBPL9	1.2246319	0.2923481	0.0178401	4197	2739	4269	3829	5167	4726
OSBPL8	1.1144219	0.1562956	0.3386165	1309	723	1324	784	1458	1498
MIR8076	#DIV/0!	#DIV/0!	0.8030358	0	0	0	0	1	0
OSBPL7	0.8636364	-0.211504	0.7940398	15	13	16	5	15	18
MIR8075	1	0	0.9930503	0	3	1	0	1	3
OSBPL6	1.6602452	0.7313963	3.69E-05	197	169	205	265	356	327
FANCM	1.311674	0.3914092	0.0294449	326	200	382	325	485	381
OSBPL5	0.4499253	-1.152243	2.00E-06	190	218	261	92	98	111
FANCL	1.2622835	0.336036	0.0143674	1073	684	1072	1034	1321	1216
AC132938.3	1.1111111	0.1520031	0.8175676	12	16	8	9	8	23
OSBPL3	0.8659325	-0.207673	0.2605876	2107	1580	2034	1280	1863	1811
AC132938.2	0	#NUM!	0.284725	6	0	0	0	0	0
OSBPL2	1.0465373	0.0656237	0.3037232	1297	1457	1780	1511	1648	1586
FANCA	0.8204681	-0.285481	0.107776	1271	1129	1616	1052	1103	1140
MIR8070	1	0	0.9922023	2	0	0	0	0	2
FANCC	0.9604558	-0.058209	0.9744267	868	1035	1081	860	1012	994
AC132938.6	0.3333333	-1.584963	0.1419481	11	9	13	0	9	2
FANCB	1.5822785	0.6620035	0.0196904	94	43	100	103	138	134
FANCE	1.0537404	0.0755195	0.2942357	1585	1913	2103	1925	2051	1926
FANCG	1.0600869	0.0841825	0.2622118	1198	1390	1556	1404	1512	1477

FANCF	0.8385502	-0.254031	0.2898499	341	422	451	347	359	312
ɔCJ-SCHIP1-A	2.5	1.3219281	0.5870135	0	0	2	0	2	3
BCL2L2	0.8572676	-0.222183	0.2311735	712	689	890	563	749	652
LAT	0.125	-3	0.211243	6	1	1	0	1	0
BCL2L1	0.9599897	-0.058909	0.9944699	1183	1324	1392	1247	1287	1209
RNU6-484P	#DIV/0!	#DIV/0!	0.4501902	0	0	0	0	0	4
AP001318.4	0	#NUM!	0.6436235	2	0	0	0	0	0
AP001318.3	0.9692308	-0.045088	0.9439379	17	34	14	16	31	16
AP001318.2	0	#NUM!	0.6840298	0	0	2	0	0	0
LBH	0.1666667	-2.584963	0.1229257	3	5	10	0	0	3
PHACTR3	0.5	-1	0.7087804	0	2	2	0	1	1
PHACTR2	0.8750281	-0.192599	0.3873556	1727	1109	1605	926	1482	1478
PHACTR1	0.584	-0.77596	0.1118741	47	31	47	17	30	26
LBP	1.8	0.8479969	0.3460883	1	7	7	11	9	7
LMLN2	1.9166667	0.9385995	0.4635952	0	1	11	5	18	0
LBR	0.8692337	-0.202184	0.2607039	3662	3173	3657	2198	3335	3587
PHACTR4	0.8800847	-0.184286	0.3604091	731	775	854	548	729	800
RNU6-483P	1	0	0.9699945	2	0	0	2	0	0
TMPOP2	1.0408163	0.0577155	0.7560899	8	5	36	28	16	7
PLRG1	1.2346421	0.3040929	0.0039464	1789	1479	2055	1954	2518	2100
DNAJC3	1.1746883	0.232278	0.0616867	2541	1796	2561	2057	3191	2855
DNAJC2	1.104697	0.1436507	0.339576	1159	475	1155	987	1148	946
DNAJC1	1.2698595	0.3446689	0.0011322	1324	1344	1675	1811	1968	1736
DNAJC7	0.9537794	-0.068273	0.9968777	2538	2130	3164	2402	2766	2302
DNAJC6	1.0305922	0.0434736	0.5966279	1256	1080	1227	894	1307	1471
LCK	#DIV/0!	#DIV/0!	0.8030358	0	0	0	0	1	0
DNAJC5	0.8451654	-0.242694	0.1144441	4536	5429	5600	3987	4653	4515
DNAJC4	1.0277228	0.0394512	0.7469774	159	191	155	154	210	155
DNAJC9	1.0298154	0.0423858	0.4616405	682	625	806	723	679	774
DNAJC8	1.1295932	0.1758033	0.0287234	5437	5572	6762	6423	7036	6615
LCT	0	#NUM!	0.1172014	0	7	2	0	0	0
DNAJB2	1.1518528	0.2039564	0.0494529	1135	1273	1451	1517	1531	1397
DNAJB1	1.034789	0.0493366	0.3814739	2703	3269	3600	3028	3601	3276
DNAJB6	1.0117563	0.0168618	0.5219217	3001	3192	3589	2925	3598	3374
DNAJB5	1.3993326	0.4847389	0.0022039	254	278	367	337	444	477
DNAJB4	1.16793	0.2239538	0.0941248	624	489	602	529	720	754
DNAJB3	#DIV/0!	#DIV/0!	0.8030358	0	0	0	1	0	0
DNAJB9	1.0427807	0.0604359	0.4914444	1213	1111	1229	926	1415	1364
DNAJB7	1.5	0.5849625	0.5545254	6	1	9	3	15	6
RPRD2	0.7490494	-0.416867	0.0118534	1327	1420	1461	761	1195	1196
AC106028.2	1.5	0.5849625	0.8233276	0	2	0	3	0	0
DNAJA1	1.3474128	0.4301919	1.13E-05	7533	6497	9219	9796	11119	10411
AC106028.3	0.4693878	-1.091148	0.2558393	15	21	13	17	2	4
DNAJA4	1	0	0.9812448	1	5	1	4	3	0
DNAJA3	0.8683232	-0.203696	0.2254408	2909	3497	3816	2703	3172	3001
LEP	1.9012346	0.9269365	0.016205	27	24	30	54	49	51
DNAJA2	0.9093497	-0.137093	0.4373644	5569	5868	6831	4848	6003	5761
AC022188.1	#DIV/0!	#DIV/0!	0.8030358	0	0	0	0	1	0
RPS15	0.998512	-0.002148	0.6321015	13934	17293	19847	18285	18056	14657
RPS14	0.926188	-0.110623	0.7259764	43555	54083	57960	49083	52006	43024
RPS17	1.0546048	0.0767025	0.3342288	2771	3401	3937	4076	3505	3080
RPS16	0.9874245	-0.018258	0.7269347	23166	29212	32072	30871	29370	23147
RPS19	0.9728086	-0.039772	0.8315951	24774	31336	33992	32336	30168	25148
YBX1P10	1.1899225	0.2508676	0.2692533	67	69	122	86	115	106

RPS18	0.9502589	-0.073607	0.9611719	34436	42949	47743	40789	42069	36046
AP003071.1	0.4285714	-1.222392	0.5924565	0	5	2	0	0	3
AP003071.5	0.3125	-1.678072	0.2712818	5	3	8	2	3	0
RPS11	1.0389983	0.0551932	0.3806873	22317	27432	30511	29222	30038	24130
RPS10	0.6376812	-0.649093	0.1902954	51	54	33	21	22	45
RPS13	1.0008941	0.0012894	0.6131947	11038	12633	14355	13107	13867	11086
RPS12	1.0922779	0.1273399	0.151294	11408	13462	15909	15792	15670	13080
P3H2	0.5610807	-0.83372	2.34E-09	1513	1692	1903	907	1054	905
P3H1	1.097561	0.1343011	0.2579005	411	588	600	564	619	572
P3H4	0.6583578	-0.603056	0.0094287	208	205	269	140	162	147
P3H3	0.7272727	-0.459432	0.7842039	9	1	1	0	0	8
MTND4P35	#DIV/0!	#DIV/0!	0.8030358	0	0	0	0	1	0
RN7SKP50	#DIV/0!	#DIV/0!	0.8030358	0	0	0	0	1	0
ASNSP1	4.3333333	2.1154772	0.2371565	1	0	2	0	6	7
ASNSP3	0.5	-1	0.8188512	2	0	0	0	1	0
AP003467.1	1	0	0.9890559	1	0	0	0	0	1
AC069368.1	3	1.5849625	0.4756362	0	0	2	6	0	0
CSTF3	1.0074405	0.0106946	0.6045888	1104	877	1379	960	1265	1160
CSTF2	0.7711392	-0.374937	0.0159141	1708	1974	2243	1446	1512	1611
LHB	2	1	0.6050759	0	0	3	1	3	2
RN7SKP23	0	#NUM!	0.1138391	3	4	1	0	0	0
CBARP	0.8906883	-0.167008	0.729899	68	90	89	76	81	63
FXR1	1.1452748	0.1956938	0.1237374	4681	2741	4514	3886	5345	4439
HELZ2	2.5946029	1.3755137	3.46E-26	974	1145	1142	2562	2774	3125
AC090425.2	0.5714286	-0.807355	0.551291	6	8	0	1	3	4
AC090425.1	2	1	0.6959901	2	0	0	2	0	2
SCFD1	1.2312231	0.3000922	0.0221472	1675	1068	1664	1663	2051	1712
AP5M1	1.0108382	0.015552	0.6591064	990	712	1066	738	1054	1006
FXR2	0.8308655	-0.267313	0.1086918	1374	1673	1748	1226	1411	1347
SCFD2	0.900978	-0.150436	0.4727312	731	834	889	630	845	736
RN7SKP16	0.1666667	-2.584963	0.0611766	6	11	7	3	1	0
CSTF1	1.0727069	0.101256	0.1521077	2314	2118	2720	2376	2756	2540
RNU6-481P	1.8	0.8479969	0.5772678	3	0	2	3	3	3
ATF4P2	0	#NUM!	0.5781912	0	0	3	0	0	0
LIF	0.4903772	-1.028036	1.87E-07	351	441	507	197	242	198
AP003717.1	1	0	0.9890559	1	0	0	0	0	1
ATF4P3	2.7142857	1.4405726	0.2583116	3	4	0	9	6	4
RN7SKP30	0	#NUM!	0.5387425	3	0	0	0	0	0
RPS26	0.9789529	-0.030689	0.8025208	2203	2582	2817	2562	2606	2274
RPS25	0.89845	-0.15449	0.5023117	13373	15262	18721	14358	15120	13069
RPS28	1.0067469	0.009701	0.6092934	3487	4249	4566	4511	4418	3456
RPS27	0.965127	-0.051209	0.8934509	7157	8053	9623	8139	8527	7301
RN7SKP26	#DIV/0!	#DIV/0!	0.0573275	0	0	0	0	5	7
RPS29	1.0695661	0.0970257	0.2504064	2552	3244	3792	3615	3566	3074
RPS20	1.0425094	0.0600603	0.3228002	12151	13596	16479	15182	15880	12959
RPS21	1.0728527	0.101452	0.2598528	3350	3952	4585	4760	4476	3517
RPS24	1.421252	0.5071624	3.31E-05	17910	16028	23619	28522	30272	23009
RPS23	1.0407407	0.0576107	0.3735428	771	872	1057	914	962	934
HOTAIR	0	#NUM!	0.0494329	8	0	5	0	0	0
AHCTF1	1.1804433	0.2393288	0.3919325	1935	864	2028	1430	2239	2029
UBXN1	0.9867148	-0.019295	0.7520208	2064	2678	3011	2664	2831	2155
ORM2	0	#NUM!	0.824326	1	0	0	0	0	0
HSD3BP1	2.5	1.3219281	0.655915	0	2	0	0	0	5
HSD3BP3	2	1	0.834705	0	0	1	0	2	0

HSD3BP2	0	#NUM!	0.824326	0	0	1	0	0	0
HSD3BP5	1.0183486	0.0262315	0.8464207	96	121	110	73	142	118
HSD3BP4	0.5	-1	0.7689504	0	0	4	0	1	1
AL031289.1	0.8823529	-0.180572	0.9099032	11	0	6	3	6	6
UBXN8	0.8769716	-0.189398	0.5626266	341	231	379	282	309	243
UBXN7	0.985647	-0.020857	0.9207897	1666	1196	1597	976	1621	1798
UBXN4	1.0181359	0.0259302	0.6479666	4139	2598	3960	2773	4187	3931
FEZF1	0.1428571	-2.807355	0.3232985	7	0	0	0	0	1
AC084876.2	0.4285714	-1.222392	0.2180821	8	13	14	6	8	1
UBXN6	0.8894892	-0.168951	0.5108695	569	707	760	608	601	602
AC084876.1	0	#NUM!	0.6840298	0	0	2	0	0	0
AC012486.1	#DIV/0!	#DIV/0!	0.5509701	0	0	0	0	3	0
RPRML	0.1666667	-2.584963	0.3386442	4	2	0	0	0	1
PGGT1BP1	#DIV/0!	#DIV/0!	0.6411933	0	0	0	0	0	2
AL589745.1	#DIV/0!	#DIV/0!	0.8030358	0	0	0	0	0	1
ATAT1	0.8470874	-0.239417	0.3633422	260	233	331	222	258	218
AL445183.3	0	#NUM!	0.6436235	2	0	0	0	0	0
BLID	0	#NUM!	0.6436235	2	0	0	0	0	0
SELENOTP1	0.9166667	-0.125531	0.8564595	8	18	10	1	21	11
AC025259.3	0	#NUM!	0.6410557	1	1	0	0	0	0
TMEM9B-AS1	0.7024793	-0.509472	0.3224769	30	43	48	28	36	21
AP5B1	0.8768018	-0.189677	0.3222109	1504	1794	1905	1237	1550	1775
LOX	0.8085106	-0.306661	0.7449853	18	9	20	13	21	4
AC098847.2	0.5	-1	0.8188512	2	0	0	0	1	0
AC100793.1	0	#NUM!	0.0963198	0	11	0	0	0	0
CERCAM	1.172379	0.2294391	0.0502316	2300	2913	2723	3196	3265	2843
AC100793.3	0	#NUM!	0.824326	1	0	0	0	0	0
AC100793.2	0	#NUM!	0.824326	1	0	0	0	0	0
AL353680.1	0	#NUM!	0.824326	1	0	0	0	0	0
LPA	#DIV/0!	#DIV/0!	0.6411933	0	0	0	0	0	2
UBE2Q2P6	0	#NUM!	0.4374838	0	4	0	0	0	0
ZNF213-AS1	0.9700704	-0.043839	0.970846	162	196	210	160	213	178
UBE2Q2P9	#DIV/0!	#DIV/0!	0.3981469	0	0	0	4	0	0
LPO	3	1.5849625	0.6122545	0	0	1	2	0	1
LPP	0.9083871	-0.138621	0.6703654	615	350	585	323	506	579
BLMH	0.9297853	-0.10503	0.7663105	615	763	858	654	771	654
AC018616.1	3.5	1.8073549	0.3879206	0	0	2	5	2	0
RN7SKP80	0.3548387	-1.494765	0.2408398	7	2	22	0	9	2
AC002094.5	0.3333333	-1.584963	0.6792283	1	0	2	1	0	0
ASNSD1	1.0863585	0.1195003	0.1847205	1552	1169	1714	1449	1789	1580
RN7SKP87	0	#NUM!	0.0986963	6	0	4	0	0	0
ST8SIA6-AS1	1.2106271	0.2757545	0.0157541	679	589	821	730	900	899
AL445673.1	0	#NUM!	0.824326	0	1	0	0	0	0
RPS4X	0.9648372	-0.051643	0.9192855	34327	38850	44305	37762	40801	34788
BLNK	1.0748299	0.1041084	0.4573017	118	136	187	171	166	137
TOPBP1	1.0336086	0.0476899	0.6120031	2827	1438	2638	1765	2725	2645
AC073367.1	1.3	0.3785116	0.686924	8	0	12	3	15	8
RN7SKP98	0	#NUM!	0.355562	5	0	0	0	0	0
LSR	1.057501	0.0806591	0.2974041	2154	2456	2729	2800	2633	2328
RPS3A	1.1795794	0.2382725	0.0059995	6892	7053	8307	8070	9818	8360
RN7SKP95	#DIV/0!	#DIV/0!	0.8030358	0	0	0	0	1	0
LSS	0.9782153	-0.031776	0.7965335	1349	1565	1860	1524	1644	1502
RN7SKP97	8	3	0.2035548	0	0	1	5	0	3
FCHSD1	0.8616751	-0.214784	0.335505	449	514	613	431	498	429

AC083902.2	0.4642857	-1.106915	0.1871189	18	7	31	8	6	12
FCHSD2	0.9412557	-0.087341	0.7556548	1205	1182	1324	950	1282	1261
COL6A2	1	0	0.7635358	77	69	106	118	65	69
COL6A1	1.2098093	0.2747796	0.167165	105	103	159	168	131	145
COL6A3	1.423913	0.509861	0.17095	24	25	43	39	45	47
TERF2IP	0.9832655	-0.024347	0.7848884	664	721	826	718	738	718
COL6A6	0.5	-1	0.81753	2	0	0	0	0	1
COL6A5	1.4444444	0.5305147	0.6774124	5	0	4	2	2	9
LTB	0	#NUM!	0.14911	1	1	6	0	0	0
UBE2Q2P2	0.8	-0.321928	0.7244313	14	7	14	6	13	9
LTF	0.25	-2	0.1550616	7	3	10	0	2	3
LTK	#DIV/0!	#DIV/0!	0.1752933	0	0	0	2	0	5
MTND4P26	1.25	0.3219281	0.9009729	0	2	2	0	5	0
RN7SKP68	#DIV/0!	#DIV/0!	0.6554062	0	0	0	0	2	0
MTND4P25	#DIV/0!	#DIV/0!	0.8030358	0	0	0	0	1	0
MTND4P24	0	#NUM!	0.824326	0	1	0	0	0	0
MTND4P23	0	#NUM!	0.0834281	5	5	0	0	0	0
RN7SL15P	0	#NUM!	0.6385096	0	2	0	0	0	0
RN7SKP58	#DIV/0!	#DIV/0!	0.3096126	0	0	0	5	0	0
MTND4P19	#DIV/0!	#DIV/0!	0.6099294	0	0	0	2	0	0
RN7SKP70	0	#NUM!	0.824326	1	0	0	0	0	0
LUM	1.7694301	0.8232847	0.0001888	132	85	169	193	247	243
MTND4P15	0	#NUM!	0.5643268	1	0	2	0	0	0
MTND4P14	1.75	0.8073549	0.7310758	4	0	0	0	2	5
MTND4P12	1.015873	0.0227201	0.9039304	23	19	21	15	14	35
RN7SKP72	2	1	0.7812375	0	0	1	1	0	1
RN7SKP74	0	#NUM!	0.0678753	8	3	0	0	0	0
PMS2P10	0.78125	-0.356144	0.7979503	6	7	19	11	5	9
OSTCP1	#DIV/0!	#DIV/0!	0.1500119	0	0	0	0	3	5
PITHD1	0.9110125	-0.134457	0.5913884	1252	1670	1809	1321	1597	1392
RN7SKP69	0	#NUM!	0.4324683	2	0	2	0	0	0
OSTCP6	1.5	0.5849625	0.8121926	0	0	2	0	1	2
RNA5SP187	0.8888889	-0.169925	0.9172069	3	3	3	0	2	6
AL021877.1	0	#NUM!	0.824326	0	1	0	0	0	0
CHORDC2P	0.5	-1	0.567089	2	4	2	0	3	1
RNA5SP188	0	#NUM!	0.824326	0	0	1	0	0	0
AC010530.1	0.8461538	-0.241008	0.8733416	6	5	15	4	5	13
RNA5SP194	0	#NUM!	0.6840298	0	0	2	0	0	0
GABARAPL3	0.2	-2.321928	0.4169927	1	4	0	0	0	1
GABARAPL3	0.2	-2.321928	0.4169927	1	4	0	0	0	1
GABARAPL2	0.9867811	-0.019198	0.7571449	1313	1423	1576	1358	1528	1369
GABARAPL1	0.9307026	-0.103608	0.7538412	953	983	1181	928	1003	970
RNA5SP179	0	#NUM!	0.824326	0	1	0	0	0	0
AKIP1	0.8728265	-0.196233	0.3501974	842	1032	1059	831	954	775
AC112484.1	1.5	0.5849625	0.7947728	3	0	1	0	0	6
HACD1	0.765625	-0.38529	0.5433958	32	22	74	23	38	37
HS3ST3A1	1.3757576	0.4602263	0.1797944	55	70	40	81	58	88
LXN	1.3924051	0.477579	0.0786873	80	64	93	75	145	110
AC112484.5	0	#NUM!	0.6436235	2	0	0	0	0	0
AC112484.4	0.9285714	-0.106915	0.9841326	4	3	7	5	8	0
AC112484.2	0.4642857	-1.106915	0.2183306	9	9	38	2	14	10
HACD3	0.9184403	-0.122742	0.5089195	7749	8143	8446	6035	8081	8237
BLACAT1	0.7272727	-0.459432	0.7067686	5	5	12	2	13	1
HACD2	1.0405134	0.0572956	0.521194	5291	4355	5312	3627	6063	5874

LY9	1.0666667	0.0931094	0.8306172	4	1	10	7	3	6
HACD4	0.3529412	-1.5025	0.1848315	20	3	11	7	1	4
FAM221B	0.25	-2	0.3100579	0	7	5	0	3	0
FAM221A	1.5683646	0.649261	0.0061729	152	83	138	172	235	178
VTI1A	1.0895124	0.1236826	0.2189344	481	451	565	478	580	573
AC005237.2	#DIV/0!	#DIV/0!	0.8030358	0	0	0	0	0	1
VTI1B	0.943662	-0.083658	0.83284	761	529	769	505	771	667
LYN	1.551538	0.6336991	3.19E-09	2317	2396	2699	3156	4306	4038
RNA5SP197	0	#NUM!	0.2290435	7	0	0	0	0	0
FYB2	1.8125	0.857981	0.3896743	10	5	1	9	13	7
FYB1	1.8270659	0.8695287	1.35E-07	3407	2044	3032	3720	5970	5809
HACE1	1.187037	0.2473649	0.1623879	572	381	667	400	708	815
INCA1	0.7719298	-0.373458	0.4080417	59	47	65	19	58	55
BBIP1	1.5657895	0.6468902	0.0039322	89	82	133	156	162	158
LYZ	0	#NUM!	0.824326	0	1	0	0	0	0
FAM220A	1.0881658	0.1218984	0.1430282	1142	1219	1450	1280	1520	1347
MTX1P1	1.3709677	0.4551946	0.1035794	75	69	104	119	69	152
STMN1P1	4	2	0.3472863	0	0	2	2	0	6
PQBP1	0.928662	-0.106774	0.8025847	859	1074	1221	1007	1011	911
AC004492.1	2.8571429	1.5145732	0.2157599	5	0	2	11	3	6
HLA-DPA1	0.5	-1	0.738891	2	0	2	0	0	2
FAM222B	1.0196007	0.0280043	0.599637	750	978	1027	774	1012	1023
FAM222A	0.8071066	-0.309169	0.4396986	55	83	59	41	66	52
SH3RF3-AS1	#DIV/0!	#DIV/0!	0.6411933	0	0	0	0	0	2
AC010280.2	0	#NUM!	0.6840298	0	0	2	0	0	0
Z84478.1	0	#NUM!	0.824326	0	0	1	0	0	0
AL163153.1	0	#NUM!	0.824326	0	1	0	0	0	0
STYXL1	0.8988016	-0.153925	0.5877267	670	759	824	721	728	576
ANKIB1	1.064197	0.0897652	0.5217128	1654	1037	1655	931	1799	1895
FAM225B	0.2580645	-1.954196	0.0166659	21	10	31	3	5	8
FAM225A	0.6746988	-0.567685	0.1995975	57	50	59	39	30	43
STXBP5L	0.1538462	-2.70044	0.1444127	5	5	3	0	0	2
FAM224B	#DIV/0!	#DIV/0!	0.8030358	0	0	0	0	1	0
AC078905.1	1.027027	0.0384741	0.8820452	13	8	16	11	15	12
HLA-DPB1	0.5	-1	0.6352778	2	5	3	5	0	0
CASC4P1	1.875	0.9068906	0.469011	0	2	6	3	2	10
HCG2040054	0.5172414	-0.95109	0.3593165	7	12	10	0	8	7
HLA-DPB2	0	#NUM!	0.6840298	0	0	2	0	0	0
ANKRD20A18	0	#NUM!	0.1795979	4	3	0	0	0	0
AP000577.1	0.2352941	-2.087463	0.06784	21	9	4	0	2	6
FAM228B	0.4875	-1.036526	0.1080462	31	21	28	8	24	7
ATCAY	0.8636364	-0.211504	0.9874547	5	5	12	18	0	1
AL356235.1	0.3846154	-1.378512	0.1267067	16	8	15	3	8	4
OSBP	0.7888769	-0.342128	0.0173023	2837	3058	3365	1919	2718	2668
ANKRD20A19	0.8157895	-0.293731	0.8143446	12	6	20	13	10	8
FAM227B	0.7606838	-0.394631	0.4694843	46	33	38	29	21	39
FAM227A	0.7878788	-0.343954	0.7557793	5	8	20	2	12	12
MAG	7	2.8073549	0.0028581	8	0	1	19	29	15
MAF	0.84375	-0.245112	0.8516962	12	4	16	6	6	15
MAK	1.375	0.4594316	0.3550693	37	15	20	23	42	34
RBM14-RBM4	0.9166667	-0.125531	0.9395626	10	9	5	11	11	0
FSCN1	1.1762012	0.2341348	0.0215406	22039	27467	28876	30926	32078	29189
FSCN2	0.25	-2	0.0016579	37	46	37	1	13	16
AC116552.1	0.3548387	-1.494765	0.185548	6	6	19	5	2	4

AC015961.2	0.4285714	-1.222392	0.5696884	2	1	4	1	2	0
MAX	1.0834675	0.1156558	0.1883107	1260	1491	1598	1492	1720	1500
MAZ	1.0115979	0.016636	0.680769	217	263	296	249	273	263
KRCC1	0.7993779	-0.32305	0.184814	202	208	233	141	189	184
CNTNAP3B	0.8636364	-0.211504	0.7376509	32	28	28	25	31	20
HACL1	0.9018303	-0.149072	0.5130498	972	971	1062	898	967	845
MBP	1.0680556	0.0949867	0.5764188	228	240	252	158	306	305
FAM229B	1.2755906	0.3511653	0.3065378	53	35	39	67	54	41
FAM229A	1.3032787	0.3821456	0.2416221	27	34	61	56	56	47
ANKRD20A17	0	#NUM!	0.3473206	0	5	0	0	0	0
MCC	0.7428984	-0.428763	0.0175954	658	658	761	372	579	592
AL359955.1	0.35	-1.514573	0.2223947	9	7	4	3	3	1
AL356475.1	4	2	0.5810589	0	0	1	0	4	0
MCU	1.1900312	0.2509993	0.0544359	652	775	820	677	1027	970
ADD3-AS1	0.6	-0.736966	0.666466	6	0	9	2	1	6
FSD1L	0.9888579	-0.016165	0.8711634	122	77	160	91	151	113
MDK	0.8712017	-0.198921	0.4437518	5385	6723	7605	6520	5814	4840
ME1	1.4	0.4854268	0.6931618	6	0	4	3	7	4
ME3	0.4285714	-1.222392	0.2923792	6	8	7	3	4	2
ME2	0.9026746	-0.147722	0.5083862	870	795	1027	741	856	833
RNU6-495P	0	#NUM!	0.824326	1	0	0	0	0	0
AL355490.1	0.5833333	-0.777608	0.5928187	4	5	3	3	2	2
COX6B1P5	4.6666667	2.2223924	0.1785549	0	0	3	2	3	9
AC010186.1	#DIV/0!	#DIV/0!	0.0782277	0	0	0	0	1	10
AC010186.3	0.8363636	-0.257798	0.6360771	51	26	33	12	37	43
AC010186.2	0.75	-0.415037	0.7141571	14	3	15	8	6	10
AC090912.2	#DIV/0!	#DIV/0!	0.5509701	0	0	0	0	3	0
AC090912.1	3	1.5849625	0.344293	4	0	0	5	3	4
AC090912.3	1.2105263	0.2756344	0.7316702	10	5	4	11	8	4
MET	1.0424383	0.059962	0.5672649	888	694	1010	583	1061	1058
AC091887.1	2.25	1.169925	0.4498554	1	1	2	3	4	2
AL359715.5	0.2777778	-1.847997	0.1749433	7	5	6	2	1	2
AL359715.4	1.4090909	0.4947647	0.4880556	7	6	9	15	10	6
AL359715.3	0.5454545	-0.874469	0.6434578	3	2	6	4	2	0
AL359715.2	0.9736842	-0.038474	0.8534635	5	5	28	21	7	9
MFF	1.0532625	0.0748651	0.2690264	1776	1807	2256	1996	2279	1875
AC004408.2	#DIV/0!	#DIV/0!	0.3668985	0	0	0	3	0	1
MGA	1.0041993	0.0060456	0.836304	2068	1301	1870	1083	1969	2209
RPS12P2	#DIV/0!	#DIV/0!	0.8030358	0	0	0	0	1	0
P4HB	0.8754712	-0.191868	0.2779162	16548	20066	21477	16458	18006	16393
AC118758.3	#DIV/0!	#DIV/0!	0.8030358	0	0	0	1	0	0
AC090568.2	#DIV/0!	#DIV/0!	0.4737294	0	0	0	0	4	0
SPON2	1.0546139	0.076715	0.5012397	151	167	213	190	196	174
RN7SL49P	2	1	0.6435174	0	0	3	1	5	0
SPON1	0.8461538	-0.241008	0.8462873	13	2	11	5	9	8
AL353093.1	0	#NUM!	0.5075476	0	0	4	0	0	0
AC241952.1	0.807947	-0.307667	0.2949132	268	183	304	133	209	268
AC118758.1	0	#NUM!	0.6385096	0	2	0	0	0	0
AL391825.1	3.1111111	1.6374299	0.1236386	2	1	6	4	15	9
ANKRD20A11	1.4761905	0.5618789	0.4966275	3	9	9	6	15	10
NSMCE3	0.7094409	-0.495246	0.0038283	666	777	739	476	562	510
NSMCE2	0.8106583	-0.302834	0.1255061	536	523	536	380	531	382
NSMCE1	1.0021453	0.0030918	0.6623265	1124	1282	1323	1272	1331	1134
MIF	0.8	-0.321928	0.7931703	10	10	25	15	6	15

GNPAT	0.9510015	-0.07248	0.9504517	1070	885	1290	933	1105	1048
MIP	0.75	-0.415037	0.8602864	3	1	0	0	0	3
RNU6-494P	0	#NUM!	0.6631647	1	0	1	0	0	0
AC025594.3	0.2	-2.321928	0.5267773	0	0	5	0	0	1
AC025594.2	0.8387097	-0.253757	0.8206316	19	6	6	9	3	14
RPS26P15	1.3333333	0.4150375	0.8418953	3	0	0	3	0	1
AC019205.2	0.7394958	-0.435386	0.4705016	42	17	60	22	35	31
COL9A1	0	#NUM!	0.6385096	0	2	0	0	0	0
RPS26P11	2.5	1.3219281	0.3950344	1	2	1	3	2	5
COL9A3	1.1245701	0.1693736	0.0718004	11001	12506	14584	15238	14472	13126
COL9A2	0.816641	-0.292226	0.3847166	188	188	273	204	192	134
RCOR3	1.0102848	0.0147621	0.6325447	883	687	958	713	959	882
RCOR2	0.9165808	-0.125666	0.6749408	278	352	341	253	319	318
RCOR1	1.0570809	0.0800858	0.3231606	5006	4557	5661	4061	5859	6173
FKBP11	1.1851852	0.2451125	0.1331941	203	158	287	261	238	269
AC093799.1	0	#NUM!	0.5378194	0	2	1	0	0	0
FKBP10	0.9354839	-0.096215	0.9107814	14	25	23	11	26	21
DOC2A	1.2024014	0.2659186	0.1096001	180	171	232	212	218	271
FKBP15	0.9802862	-0.028725	0.8470552	2173	2033	2642	1780	2498	2435
FKBP14	0.8664459	-0.206818	0.4131636	695	461	656	355	555	660
MOGAT2	#DIV/0!	#DIV/0!	0.6099294	0	0	0	2	0	0
MOGAT1	2	1	0.8147319	1	0	0	0	0	2
DOC2B	#DIV/0!	#DIV/0!	0.8030358	0	0	0	0	0	1
BMI1	1.1384124	0.1870233	0.1574445	926	547	1034	840	1082	932
RN7SL45P	0	#NUM!	0.6604275	0	1	1	0	0	0
AC138028.2	1.3333333	0.4150375	0.5644669	4	3	17	6	13	13
MLX	1.0561599	0.0788282	0.307744	1094	1316	1543	1230	1586	1359
AC138028.4	0.9533679	-0.068895	0.9880657	46	50	97	36	77	71
AC138028.6	1.4166667	0.5025003	0.3277896	17	11	20	23	28	17
PDZK1P1	0.875	-0.192645	0.8750046	16	2	6	6	7	8
ANKDD1B	1.05	0.0703893	0.9535537	17	1	2	4	6	11
TMC3-AS1	1.0163934	0.023459	0.8735173	22	19	20	24	19	19
ANKDD1A	1.284507	0.3612148	0.1114461	130	78	147	119	170	167
MME	1.7672679	0.8215208	2.32E-06	869	573	831	908	1553	1556
MMD	1.0096536	0.0138604	0.7295617	555	585	621	450	693	635
OSMR	4.5	2.169925	0.4909927	8	0	0	0	20	16
MN1	1.7	0.7655347	0.2342701	8	7	15	12	22	17
ATP5G1P5	0.5	-1	0.680026	3	1	0	0	1	1
ATP5G1P4	0.8333333	-0.263034	0.793796	7	11	6	3	5	12
RABGAP1	1.0615485	0.0861703	0.3779943	1559	1318	1721	1174	1795	1912
ATP5G1P1	1	0	0.962226	1	5	1	0	6	1
AL358876.2	#DIV/0!	#DIV/0!	0.8030358	0	0	0	0	1	0
AC133919.3	#DIV/0!	#DIV/0!	0.1958503	0	0	0	0	4	3
AC133919.2	2.2	1.1375035	0.1472824	8	9	3	15	16	13
AC133919.1	0	#NUM!	0.6410557	1	1	0	0	0	0
MNT	0.9198056	-0.120599	0.7024634	504	516	626	465	494	555
AL358876.1	#DIV/0!	#DIV/0!	0.8030358	0	0	0	1	0	0
AP005131.7	0	#NUM!	0.824326	0	0	1	0	0	0
AP005131.6	0.8333333	-0.263034	0.9931924	0	0	6	2	0	3
AC093809.1	#DIV/0!	#DIV/0!	0.8030358	0	0	0	0	0	1
MOK	1.1384181	0.1870305	0.3747099	93	110	151	90	152	161
AC097372.3	2	1	0.814195	0	1	0	0	0	2
AC104763.1	3.6666667	1.8744691	0.1214726	2	3	1	13	7	2
FKBP1A	1.1391452	0.1879517	0.0448825	5173	6208	6823	6695	7552	6490

FKBP1C	0.7552448	-0.404984	0.3671438	43	53	47	24	40	44
FKBP1B	0.5	-1	0.6949177	2	4	0	3	0	0
PLVAP	0	#NUM!	0.824326	0	0	1	0	0	0
MPG	0.9307692	-0.103505	0.887046	355	464	481	474	391	345
RNA5SP101	#DIV/0!	#DIV/0!	0.3981469	0	0	0	4	0	0
MPI	1.0021853	0.0031493	0.6663386	653	774	861	706	827	760
FAM21FP	0.4883721	-1.033947	0.2228658	14	22	7	4	14	3
MPL	1.8333333	0.8744691	0.5648671	5	1	0	5	3	3
RNA5SP106	0	#NUM!	0.824326	1	0	0	0	0	0
WBP1L	1.2275661	0.2958007	0.0079449	1963	2289	2256	2239	3031	2719
RNA5SP109	0	#NUM!	0.2756435	2	2	1	0	0	0
RNA5SP108	1.3333333	0.4150375	0.8093949	0	0	3	2	2	0
AC068870.2	0.8484848	-0.237039	0.7887496	3	25	5	8	12	8
AC068870.1	#DIV/0!	#DIV/0!	0.8030358	0	0	0	0	0	1
MPZ	0.7766497	-0.364664	0.4235789	55	55	87	55	52	46
OSR2	1.0106157	0.0152345	0.7691542	136	172	163	158	178	140
OSR1	0	#NUM!	0.6385096	0	2	0	0	0	0
WBP11	0.8730636	-0.195841	0.2125455	4531	5025	5743	3804	4733	4820
MR1	0.5076792	-0.978011	8.19E-07	413	378	381	187	214	194
FAM21EP	0.4473684	-1.160465	0.2532315	4	10	24	6	7	4
AC074052.1	2	1	0.8324799	1	0	0	0	2	0
RNA5SP124	0	#NUM!	0.5781912	0	0	3	0	0	0
RNA5SP123	0	#NUM!	0.824326	0	0	1	0	0	0
RNA5SP122	0	#NUM!	0.2762615	2	0	4	0	0	0
UTP23	0.8851423	-0.176019	0.5436959	304	263	382	202	295	343
RNA5SP129	0	#NUM!	0.824326	1	0	0	0	0	0
MRO	1.0869565	0.1202942	0.7420186	23	23	23	24	30	21
AC005726.3	0	#NUM!	0.6385096	0	2	0	0	0	0
AC005726.2	0.952381	-0.070389	0.9889383	59	51	79	60	57	63
AC005726.4	1.0952381	0.1312445	0.8042573	3	6	12	7	6	10
TAC4	2.125	1.0874628	0.3595984	2	0	6	5	7	5
TAC3	1.5526316	0.6347155	0.3590888	19	14	5	29	22	8
UTP20	0.8201982	-0.285956	0.1498029	1566	1142	1530	788	1318	1370
OST4	1.1133896	0.1549585	0.179591	959	1059	1298	1403	1317	972
ZFHX3	0.9723226	-0.040493	0.9495088	3040	2822	2990	1777	3102	3728
ZFHX2	0.6666667	-0.584963	0.6840171	3	3	9	2	5	3
RNA5SP114	0	#NUM!	0.355562	5	0	0	0	0	0
LIX1L-AS1	#DIV/0!	#DIV/0!	0.0127102	0	0	0	7	5	3
MSN	0.7383178	-0.437686	0.4369398	38	23	46	12	34	33
MT4	5.0833333	2.3457748	0.0140143	7	5	0	11	39	11
BMP7	0.71875	-0.476438	0.6112485	4	17	11	5	14	4
ZFHX4	1.2647059	0.3388019	0.594227	11	11	12	9	11	23
ANKHD1	0.5491329	-0.864773	0.0483325	55	53	65	33	41	21
BMP6	3	1.5849625	0.1912889	1	4	2	3	8	10
BMP4	0.9985141	-0.002145	0.7586939	219	205	249	217	217	238
n of pathway-re	#VALUE!	#VALUE!	culum lume	04550|hsa04	uripotency	ohogenetic protein 4			
BMP3	#DIV/0!	#DIV/0!	0.8030358	0	0	0	1	0	0
BMP2	0.6842105	-0.547488	0.6255687	10	7	2	0	6	7
BMP1	0.707529	-0.499139	0.0140495	311	363	362	221	262	250
OSTC	1.233717	0.3030115	0.0015532	2252	2072	2800	2732	3214	2843
TAB3	1.1122853	0.1535269	0.2675398	831	603	837	584	935	1007
AC005476.1	#DIV/0!	#DIV/0!	0.4501902	0	0	0	0	0	4
TAB2	1.0204188	0.0291613	0.613888	2148	1541	2090	1516	2212	2169
TPRKBP2	0	#NUM!	0.5387425	3	0	0	0	0	0

OSTN	#DIV/0!	#DIV/0!	0.8030358	0	0	0	1	0	0
TAB1	0.867884	-0.204426	0.3753661	996	1301	1427	1101	1185	946
LSM14B	0.9655598	-0.050562	0.9048024	1800	2112	2563	2000	2221	2031
AC005476.2	1.7368421	0.7964666	0.1087976	8	21	28	37	37	25
LSM14A	0.8978027	-0.15553	0.3718483	6219	5384	6920	4551	6020	6059
AC023024.2	0.5396825	-0.889817	0.238717	10	37	16	2	18	14
AC023024.1	0.6031746	-0.729352	0.3406956	19	17	27	1	24	13
RNA5SP144	0	#NUM!	0.5781912	0	0	3	0	0	0
MTR	0.9460784	-0.079968	0.8147887	2040	1497	1971	1235	1887	2089
AC004982.2	0.6451613	-0.632268	0.2486076	28	31	65	22	36	22
AC004982.1	0.3636364	-1.459432	0.4599346	4	0	7	1	3	0
RPS26P28	1	0	0.9890559	0	0	1	0	1	0
LRRFIP2	0.9192644	-0.121448	0.7003507	1950	1410	2350	1536	1909	1804
LRRFIP1	1.4294946	0.5155052	0.0001579	6065	4295	6043	5804	8721	8923
HS3ST3B1	0.9554994	-0.065673	0.9394466	1594	1524	2028	1323	1777	1817
UTP15	0.9281929	-0.107503	0.6745096	1337	1057	1380	974	1348	1181
RNA5SP130	2	1	0.8147319	1	0	0	0	0	2
UTP11	1.1742424	0.2317303	0.0439845	1239	924	1533	1348	1642	1350
RNA5SP137	0	#NUM!	0.5337954	0	3	0	0	0	0
MUT	0.9787007	-0.03106	0.9233753	1025	748	1044	710	1106	941
UTP18	0.8270122	-0.274019	0.0535575	2589	2601	3221	2087	2456	2413
SPOPL	1.2215715	0.2887383	0.0627056	763	491	795	604	968	931
MVD	0.9597586	-0.059257	0.9497878	1030	1430	1516	1384	1347	1085
RPS26P31	2.0625	1.0443941	0.2088578	4	7	5	12	11	10
RNA5SP163	0	#NUM!	0.824326	0	0	1	0	0	0
MVK	0.8277276	-0.272772	0.2498945	352	366	501	316	404	289
MVP	0.7283905	-0.457216	0.0271197	1445	1784	2139	1443	1455	1012
TAF9	1.2591424	0.3324414	0.0063521	1917	1365	2105	2259	2423	2101
RPS26P49	0	#NUM!	0.0496355	12	1	0	0	0	0
RPS26P47	1.2857143	0.3625701	0.7857044	4	0	3	2	1	6
BMS1	1.2266127	0.2946798	0.012994	3678	2540	3905	3409	4619	4389
RPS26P45	#DIV/0!	#DIV/0!	0.8030358	0	0	0	1	0	0
AC012103.1	0.4285714	-1.222392	0.4100547	2	7	5	0	2	4
RPS26P41	#DIV/0!	#DIV/0!	0.2256364	0	0	0	3	3	0
RNA5SP172	0	#NUM!	0.5387425	3	0	0	0	0	0
AL449403.1	0	#NUM!	0.824326	0	0	1	0	0	0
AL353583.1	0.25	-2	0.5216253	0	0	8	2	0	0
RNA5SP157	#DIV/0!	#DIV/0!	0.3981469	0	0	0	4	0	0
MX2	5.1666667	2.3692338	0.0088133	0	8	4	27	28	7
MX1	1.9230769	0.9434165	0.0197448	27	26	25	53	57	40
AC005330.1	0.5	-1	0.519574	4	7	1	0	6	0
ESRRAP2	3	1.5849625	0.6896151	1	0	0	0	3	0
SCIMP	0.1176471	-3.087463	0.0795319	5	9	3	2	0	0
AC073464.1	0.1666667	-2.584963	0.3571349	5	0	1	0	0	1
BMT2	1.3850467	0.4699347	0.0173641	201	150	184	192	294	255
AC073464.2	0	#NUM!	0.0010104	4	6	19	0	0	0
TAF8	0.8788834	-0.186256	0.3758867	639	790	792	599	693	660
TAF7	0.9446095	-0.08221	0.8313004	5299	4562	6297	4408	5735	5120
TAF6	0.7761748	-0.365547	0.0113699	1473	1576	1803	1037	1413	1316
RPS26P52	0	#NUM!	0.5075476	0	0	4	0	0	0
TAF5	1.399361	0.4847682	0.0004912	555	423	587	630	755	805
TAF4	0.9176548	-0.123977	0.5549419	1449	1426	1679	1197	1499	1483
RNA5SP160	0.2857143	-1.807355	0.5012145	0	7	0	2	0	0
TAF3	1.1002246	0.1377981	0.1451265	1144	1125	1293	1066	1483	1370

TAF2	0.9570957	-0.063265	0.9232079	722	425	671	415	677	648
TAF1	0.7542747	-0.406838	0.0198535	1799	1551	1855	921	1413	1592
AL590434.1	0.5287356	-0.919382	0.1413881	39	26	22	21	15	10
AL357559.1	0	#NUM!	0.6385096	0	2	0	0	0	0
TPGS1	1.1531532	0.2055841	0.527598	29	35	47	31	45	52
TPGS2	1.0245137	0.0349392	0.4492637	2613	2990	3290	2739	3403	2969
MYC	0.7111819	-0.491709	0.0006406	1984	2435	2485	1434	1799	1677
MYB	0.5555556	-0.847997	0.650508	2	1	6	1	2	2
AC096543.2	2.5	1.3219281	0.6211837	1	1	0	0	4	1
SUCLA2-AS1	1.2222222	0.2895066	0.8448925	0	4	5	0	6	5
RNA5SP296	0	#NUM!	0.5378194	0	2	1	0	0	0
RNA5SP298	1.5	0.5849625	0.8186252	2	0	0	2	0	1
AL451064.2	0.6666667	-0.584963	0.7650708	1	3	2	0	4	0
AL451064.1	0.6666667	-0.584963	0.7518072	5	1	0	0	3	1
AC000089.1	0.7142857	-0.485427	0.7052079	7	3	4	2	5	3
AC010531.3	2.3333333	1.2223924	0.4495716	5	1	0	7	7	0
AL021878.2	1.3095238	0.3890423	0.480241	16	11	15	17	12	26
AC010531.6	0.5217391	-0.938599	0.2883315	3	25	18	6	11	7
AC010531.5	7	2.8073549	0.2891305	1	0	0	0	1	6
AP002387.1	1.109375	0.1497471	0.6729398	38	47	43	26	57	59
AL138720.1	0.7176471	-0.478654	0.5352572	13	51	21	32	10	19
NKX2-5	#DIV/0!	#DIV/0!	0.4737294	0	0	0	0	4	0
AC024581.1	#DIV/0!	#DIV/0!	0.0578304	0	0	0	0	6	6
NKX2-3	1.5555556	0.6374299	0.6265959	0	7	2	8	5	1
NKX2-2	1.25	0.3219281	0.7627113	2	3	3	6	3	1
FZD1	0.7478802	-0.419121	0.0331528	547	645	577	334	467	522
DNAH12	0.5	-1	0.3424085	10	6	18	0	9	8
WNT10B	1.106383	0.1458509	0.5203145	64	55	69	89	49	70
DNAH11	0.875	-0.192645	0.9987155	3	0	5	5	2	0
FZD3	0.8684583	-0.203472	0.4823396	276	186	245	139	228	247
WNT10A	0.5827338	-0.779091	0.164103	48	34	57	40	27	14
DNAH10	1.1818182	0.2410081	0.7862962	4	4	3	8	3	2
FZD2	0.5070922	-0.97968	0.0060163	68	104	110	48	52	43
DNAH17	0.6395349	-0.644905	0.1469382	37	65	70	23	50	37
FZD5	1.2502417	0.322207	0.0041808	4102	3987	4323	4084	5456	5978
SLC12A9-AS1	1.3589744	0.4425182	0.5331475	16	11	12	9	34	10
FZD4	0.6793893	-0.55769	0.0010428	629	570	766	382	457	496
FZD7	0.7472222	-0.420391	0.0233957	466	413	561	321	392	363
DNAH14	1.0583942	0.081877	0.37659	636	534	748	638	817	575
FZD6	0.9338782	-0.098694	0.7677341	503	371	472	340	461	456
FZD9	0.4	-1.321928	0.5112402	1	1	8	0	1	3
FZD8	0.533557	-0.906286	0.0122639	128	95	75	43	72	44
CIDECP	0.7671541	-0.382412	0.1970713	242	295	352	258	272	152
AL160262.1	#DIV/0!	#DIV/0!	0.8030358	0	0	0	0	0	1
ODAPH	1.8636364	0.8981204	0.2529457	7	11	4	11	15	15
AC018462.1	2	1	0.5641138	2	0	2	1	1	6
AP002001.1	0	#NUM!	0.6385096	0	2	0	0	0	0
FAM234B	0.7953443	-0.330349	0.0759548	603	759	700	449	603	588
FAM234A	0.8732535	-0.195528	0.3979996	610	636	758	599	612	539
TAL2	#DIV/0!	#DIV/0!	0.2116454	0	0	0	5	1	0
FAM238C	0.8235294	-0.280108	0.7309336	10	20	4	6	9	13
TANK	1.3007316	0.3793233	0.0153798	1027	606	964	891	1231	1256
FAM238B	1.3243243	0.4052565	0.4856718	13	8	16	12	18	19
FAM238A	1.5	0.5849625	0.8840043	2	0	0	0	3	0

NKAPP1	1.0942623	0.1299586	0.5108163	62	79	103	103	98	66
LINC-PINT	0.5965909	-0.745186	0.124385	71	43	62	37	20	48
AC010677.1	#DIV/0!	#DIV/0!	0.6099294	0	0	0	2	0	0
TCAIM	1.0967912	0.1332889	0.2091733	637	497	767	581	778	726
FAM237B	0.5	-1	0.8143938	2	0	0	1	0	0
FAM237A	#DIV/0!	#DIV/0!	0.8030358	0	0	0	0	1	0
GUCA2A	2.25	1.169925	0.5283277	4	0	0	3	1	5
AC009108.3	1.25	0.3219281	0.8471932	0	2	2	1	1	3
AC009108.1	1.3333333	0.4150375	0.7922935	1	0	2	4	0	0
LAMTOR2	0.8900245	-0.168083	0.5512382	691	856	899	790	765	622
AC018618.2	0	#NUM!	0.824326	0	1	0	0	0	0
LAMTOR1	0.9514293	-0.071832	0.9988578	2321	2743	3192	2780	2633	2442
EEF1AKMT3	0.8092643	-0.305317	0.3353745	136	103	128	89	114	94
LAMTOR4	1.0665328	0.0929284	0.3366395	942	1215	1330	1358	1343	1018
EEF1AKMT2	1.5026455	0.5875047	0.0363283	73	52	64	96	99	89
LAMTOR3	1.2686763	0.343324	0.0020985	1335	1140	1340	1424	1814	1602
EEF1AKMT1	1.2093784	0.2742657	0.0561546	266	306	345	340	418	351
LAMTOR5	1.2054795	0.2696071	0.0436276	433	484	616	623	699	526
GUCA1B	1.1875	0.2479275	0.6613831	9	4	19	12	18	8
RN7SL40P	#DIV/0!	#DIV/0!	0.2164877	0	0	0	4	2	0
MACROD1	0.8144578	-0.296088	0.3369124	122	128	165	93	143	102
AL157762.1	#DIV/0!	#DIV/0!	0.5338029	0	0	0	0	1	2
MACROD2	0.6228571	-0.683027	0.1176019	68	58	49	35	28	46
NBN	1.2852889	0.3620926	0.0550695	638	357	684	505	965	688
LZTS2	1.1570089	0.2104	0.0381967	1201	1380	1578	1594	1659	1559
LZTS1	0.3484848	-1.520832	0.0418219	15	22	29	2	12	9
LZTS3	0.8375394	-0.255771	0.2463844	377	432	459	342	352	368
FAM239A	0.6928021	-0.529485	0.022046	253	257	268	146	175	218
TAP2	1.1587429	0.2125606	0.0856267	402	360	479	441	546	451
TAP1	0.913691	-0.130222	0.5721007	1543	1767	1927	1496	1628	1661
AC027020.2	0.1875	-2.415037	0.156234	6	1	9	1	2	0
FAM239C	0.2857143	-1.807355	0.4479409	0	6	1	0	0	2
FAM239B	1.3207547	0.4013626	0.3971966	15	12	26	30	12	28
AC019197.1	1.5714286	0.6520767	0.4649517	4	5	5	10	4	8
NCL	0.9788267	-0.030875	0.7703578	49051	42172	58116	44640	53298	48239
NKAPD1	0.8635747	-0.211607	0.238906	1436	1259	1725	1000	1416	1401
TARS	1.1468762	0.1977096	0.047949	7831	5671	8074	7319	9195	8231
KRBA2	0.6666667	-0.584963	0.467381	20	11	11	3	10	15
KRBA1	1.1524752	0.2047358	0.2352819	154	114	237	182	190	210
AL513218.1	0.7	-0.514573	0.8254818	1	2	7	4	1	2
NDN	0	#NUM!	0.824326	0	1	0	0	0	0
AC013565.3	0	#NUM!	0.6385096	0	2	0	0	0	0
NEB	1.1462945	0.1969778	0.3203709	415	222	402	245	471	475
NF1	0.911231	-0.134111	0.6042485	3864	2778	3553	1774	3354	4162
NF2	0.7613412	-0.393385	0.021258	590	671	767	455	558	531
LZTR1	0.8629948	-0.212576	0.2419306	866	960	1079	767	921	819
NES	0.4222222	-1.243926	0.0151001	34	57	44	23	17	17
BNC2	1.1208633	0.1646104	0.430083	266	163	266	152	270	357
BNC1	0	#NUM!	0.824326	0	1	0	0	0	0
AL117344.1	3	1.5849625	0.4480945	3	0	0	4	5	0
PGPEP1	0.9513081	-0.072015	0.9139827	824	905	1023	815	961	842
NAV2-AS5	#DIV/0!	#DIV/0!	0.263214	0	0	0	0	5	1
NAV2-AS4	2.4615385	1.2995603	0.0532823	21	2	16	20	55	21
NAV2-AS2	0.9473684	-0.078003	0.8950983	71	84	73	54	79	83

VSTM2L	0	#NUM!	0.5387425	3	0	0	0	0	0
NGF	1.3333333	0.4150375	0.8441278	0	0	3	0	3	1
AC068631.1	4.3333333	2.1154772	0.1783709	0	2	1	7	3	3
RPS11P6	1.8	0.8479969	0.3468118	6	1	8	18	2	7
AC114980.1	1	0	0.9732672	3	0	8	0	9	2
RPS11P5	0.4210526	-1.247928	0.1213215	13	23	21	1	9	14
AC024341.1	2	1	0.7760147	0	2	0	0	4	0
AC069366.2	0.1666667	-2.584963	0.3558995	0	4	2	0	0	1
AC069366.1	2.25	1.169925	0.5149592	2	0	2	1	7	1
AC007381.1	#DIV/0!	#DIV/0!	0.8030358	0	0	0	0	1	0
AC110921.1	0	#NUM!	0.824326	0	0	1	0	0	0
FZR1	0.9650138	-0.051379	0.9245227	1029	1247	1354	1203	1210	1090
NHS	1.2530447	0.3254378	0.0600836	488	441	549	384	718	750
PAXIP1-AS1	1.0846774	0.1172661	0.4583542	186	145	165	161	198	179
PAXIP1-AS2	0.7698413	-0.377367	0.4727832	44	30	52	27	47	23
UGGT2	1.1892052	0.2499977	0.0755624	627	403	656	560	735	710
EEF1B2P3	0.9002347	-0.151627	0.6112391	453	610	641	520	548	466
UGGT1	0.9388427	-0.091045	0.7061095	7155	6514	7277	4755	7202	7708
EEF1B2P7	#DIV/0!	#DIV/0!	0.6411933	0	0	0	0	0	2
EEF1B2P6	1.25	0.3219281	0.7938256	2	2	4	2	6	2
MCX5-GPRA!	0.9809524	-0.027745	0.9721208	33	44	28	21	45	37
EGFLAM-AS4	0.6666667	-0.584963	0.8308055	3	0	0	0	2	0
AC007537.1	0.1666667	-2.584963	0.3816882	0	2	4	0	1	0
AP003715.1	#DIV/0!	#DIV/0!	0.1593104	0	0	0	6	0	1
NIN	1.0363636	0.0515303	0.5750572	1276	779	1410	845	1399	1347
BRI3P1	11	3.4594316	0.1162001	0	1	0	4	0	7
AF230666.1	0.5714286	-0.807355	0.6907295	0	7	0	1	3	0
RBM26-AS1	0.7155963	-0.482782	0.2411841	77	58	83	60	54	42
HABP4	1.2616679	0.3353323	0.0138946	387	445	475	517	619	513
HABP2	0	#NUM!	0.824326	0	0	1	0	0	0
C21orf91-OT1	2	1	0.834705	0	0	1	0	2	0
AC083900.1	1.7	0.7655347	0.5544423	1	5	4	2	13	2
COL8A2	#DIV/0!	#DIV/0!	0.8030358	0	0	0	0	0	1
COL8A1	1.1666667	0.2223924	0.8553674	1	0	5	2	5	0
NLK	1.0143652	0.0205771	0.6314077	710	693	755	620	734	835
NLN	0.9225248	-0.11634	0.6165144	5483	4210	5912	3847	5412	5137
OTOA	0.0909091	-3.459432	0.1102191	1	8	2	1	0	0
OTOG	3.509434	1.8112384	2.67E-05	15	20	18	50	84	52
OTOF	1.3333333	0.4150375	0.7708108	3	2	1	3	2	3
AC092474.2	0.0526316	-4.247928	0.0235339	5	4	10	0	1	0
NMB	0.1111111	-3.169925	0.1728848	5	1	3	0	1	0
AC008124.1	0.8398329	-0.251826	0.3604222	197	262	259	193	238	172
NMI	1.5268817	0.6105883	7.88E-05	605	402	667	819	964	773
AC093414.1	0	#NUM!	0.5781912	0	0	3	0	0	0
AL513217.1	0	#NUM!	0.4117982	0	3	1	0	0	0
RPTOR	0.8529048	-0.229543	0.1799496	1620	1992	2051	1328	1733	1769
AC131953.1	12	3.5849625	0.1105645	1	0	0	0	4	8
NNT	0.8891954	-0.169428	0.4235787	706	667	802	477	721	736
NOG	0	#NUM!	0.3168171	0	4	1	0	0	0
KREMEN1	0.9120297	-0.132847	0.6211481	565	643	679	546	575	600
AC091980.2	1.3333333	0.4150375	0.8441278	0	0	3	0	3	1
KREMEN2	1.0204678	0.0292307	0.7117133	212	261	211	253	233	212
AC023513.1	0	#NUM!	0.6631647	1	0	1	0	0	0
OTOR	2	1	0.50521	1	2	2	2	1	7

RN7SL34P	#DIV/0!	#DIV/0!	0.8030358	0	0	0	0	0	1
AL445435.1	0.6666667	-0.584963	0.8318384	2	1	0	0	0	2
AC019206.1	1	0	0.9836317	3	0	0	0	1	2
AP003465.2	#DIV/0!	#DIV/0!	0.6482162	0	0	0	0	1	1
AC004158.1	0.75	-0.415037	0.9580436	1	0	3	3	0	0
AL589987.1	2.8	1.4854268	0.3251556	0	1	4	1	2	11
RNA5SP200	2	1	0.7703774	2	0	0	0	4	0
TAF9P3	0.3333333	-1.584963	0.6833782	1	0	2	0	0	1
CXCR4	2.4166667	1.2730185	0.1579877	3	3	6	12	6	11
RNA5SP204	#DIV/0!	#DIV/0!	0.8030358	0	0	0	0	0	1
CXCR6	1.4358974	0.5219527	0.0360159	104	108	100	185	136	127
NPL	1	0	0.9890559	0	1	0	0	0	1
RNA5SP203	0.5	-1	0.701864	1	3	0	0	0	2
AC092724.1	1	0	0.9707229	3	0	0	0	3	0
CXCR1	#DIV/0!	#DIV/0!	0.8030358	0	0	0	0	0	1
NPW	1.1034483	0.142019	0.7533827	11	3	15	14	5	13
CXCR2	0.6332518	-0.659149	0.0042973	305	253	260	128	212	178
RN7SL32P	#DIV/0!	#DIV/0!	0.8030358	0	0	0	0	0	1
CTAGE10P	#DIV/0!	#DIV/0!	0.5371397	0	0	0	0	0	3
CEACAM22P	1	0	0.9802778	26	22	15	10	40	13
RNA5SP220	0	#NUM!	0.5643268	1	0	2	0	0	0
AC018712.1	2.1666667	1.1154772	0.4399896	4	2	0	5	3	5
NRK	1.3913043	0.476438	0.1853735	41	22	52	44	67	49
NRM	0.969876	-0.044128	0.8815637	484	555	654	565	571	506
NRL	1.4	0.4854268	0.244706	19	20	36	34	44	27
AC083806.3	3	1.5849625	0.5008807	1	0	1	1	5	0
AC083806.2	1.75	0.8073549	0.5848712	3	3	2	0	7	7
DLG5-AS1	1.0930233	0.1283241	0.7307718	14	7	22	19	16	12
NSF	0.8673409	-0.205329	0.1994902	1608	1536	1899	1255	1617	1502
SPNS3	1.0909091	0.1255309	0.8251054	6	1	15	3	4	17
SPNS2	0.7385892	-0.437156	0.3797332	81	53	107	81	58	39
RNA5SP217	2	1	0.6836413	0	0	3	0	6	0
WRNIP1	0.8225441	-0.281835	0.0671279	2019	2048	2560	1738	1972	1741
RNA5SP219	1	0	0.9890559	0	0	1	0	0	1
AL672032.1	0	#NUM!	0.824326	0	0	1	0	0	0
SPNS1	0.9777778	-0.032421	0.9600607	19	3	23	6	11	27
AC144450.1	0.4303797	-1.216318	0.0108256	58	38	62	13	23	32
OR51B8P	#DIV/0!	#DIV/0!	0.4155775	0	0	0	0	2	2
RNA5SP246	0.5714286	-0.807355	0.7209581	3	0	4	0	4	0
RNA5SP245	#DIV/0!	#DIV/0!	0.8030358	0	0	0	0	1	0
NTS	0.2222222	-2.169925	0.4039635	0	0	9	0	1	1
CTAGE16P	3	1.5849625	0.4433029	1	0	1	5	0	1
OTX2	1.1755725	0.2333635	0.491182	62	33	36	57	52	45
OTX1	0.9602996	-0.058443	0.9712613	337	453	545	381	433	468
OSGEPL1-AS1	1.6363636	0.7104934	0.5078823	4	5	2	6	5	7
TBCD	0.7659197	-0.384735	0.0101021	3312	4060	4453	2882	3261	2914
TBCC	0.9072566	-0.140417	0.604854	803	1024	998	872	890	801
TBCB	0.8776916	-0.188214	0.4510719	1276	1659	1709	1474	1387	1215
TBCA	1.0196833	0.0281211	0.4454575	2569	2821	3450	2958	3234	2822
TBCK	1.2649573	0.3390886	0.0165695	440	331	516	484	542	602
TBCE	1.0941291	0.1297829	0.1409002	1336	1267	1519	1314	1749	1447
DOCK6	0.9178785	-0.123625	0.6955824	340	392	437	333	381	359
DOCK5	0.9156404	-0.127147	0.7182668	562	396	666	384	512	591
DOCK4	1.4620563	0.5479989	0.0036325	624	412	598	510	893	986

DOCK3	0.6588785	-0.601916	0.1292586	65	67	82	24	60	57
RNA5SP268	1.2	0.2630344	0.8618909	1	0	4	0	0	6
DOCK9	1.0133634	0.0191516	0.6737089	2135	1702	2374	1472	2341	2481
NVL	0.9833156	-0.024274	0.784439	953	759	1105	844	1021	905
DOCK8	0.822401	-0.282086	0.1945635	557	504	555	279	535	515
DOCK7	1.1621984	0.2168564	0.1914519	1210	677	1097	790	1368	1310
FOXP4-AS1	1.3333333	0.4150375	0.6570772	5	6	4	6	8	6
LMOD1	0.9722222	-0.040642	0.9192875	30	57	57	56	36	48
CXCL3	0.7391304	-0.436099	0.7061017	1	18	4	0	4	13
CXCL2	5.4	2.4329594	0.0493003	0	0	5	15	6	6
CXCL5	0	#NUM!	0.824326	0	0	1	0	0	0
LMOD3	#DIV/0!	#DIV/0!	0.5509701	0	0	0	0	3	0
LMOD2	0.5789474	-0.788496	0.5383643	5	11	3	7	2	2
RUBCN	0.9093915	-0.137027	0.601028	423	532	557	383	491	501
MRGPRX2	7.5	2.9068906	0.0055248	3	0	3	6	21	18
MRGPRX1	2.259542	1.1760304	1.28E-06	74	97	91	185	240	167
AC092964.1	0	#NUM!	0.6840298	0	0	2	0	0	0
AC114490.1	0.4285714	-1.222392	0.5474404	5	1	1	0	3	0
DOCK2	#DIV/0!	#DIV/0!	0.0435352	0	0	0	3	2	6
RNA5SP260	0	#NUM!	0.824326	0	0	1	0	0	0
DOCK1	1.1748842	0.2325186	0.0421782	1255	1048	1368	1114	1630	1569
NXN	0.9549237	-0.066543	0.9664074	803	1070	1011	957	965	832
AC093904.4	1.027972	0.039801	0.8057289	60	38	45	50	48	49
AC093904.3	1.4	0.4854268	0.7391465	0	2	3	3	3	1
AC093904.2	0.2391304	-2.06413	0.0327671	10	20	16	0	4	7
OR51B3P	0	#NUM!	0.4521054	1	0	3	0	0	0
AC138123.2	0	#NUM!	0.6436235	2	0	0	0	0	0
NYX	0.6923077	-0.530515	0.7192847	2	6	5	3	2	4
TBK1	1.0937149	0.1292368	0.3379451	1413	670	1481	1089	1432	1377
LINC01869	0.7931034	-0.334419	0.8039697	10	12	7	16	5	2
LINC01865	0.3333333	-1.584963	0.6805352	0	1	2	0	0	1
AC024580.2	#DIV/0!	#DIV/0!	0.4501902	0	0	0	0	0	4
LINC01864	0	#NUM!	0.5075476	0	0	4	0	0	0
AC024580.1	0.36	-1.473931	0.2215851	13	11	1	0	6	3
LINC01863	#DIV/0!	#DIV/0!	0.8030358	0	0	0	0	1	0
PHKA1	0.9132639	-0.130896	0.5319096	1143	1311	1293	934	1238	1250
PHKA2	0.9679267	-0.04703	0.9779055	760	854	1005	655	903	977
LINC01854	0.75	-0.415037	0.6301541	10	13	17	9	13	8
LINC01856	0.3214286	-1.63743	0.1518434	11	7	10	0	7	2
LINC01850	0	#NUM!	0.6436235	2	0	0	0	0	0
LINC01852	1.0465116	0.0655883	0.8261388	18	7	18	16	6	23
DOCK9-AS2	0.8358209	-0.258734	0.8041419	21	14	32	24	12	20
DOCK9-AS1	2	1	0.7703774	2	0	0	0	4	0
RNU6-438P	2.7777778	1.4739312	0.1689827	5	3	1	10	9	6
C14orf93	1.1679909	0.2240291	0.1245608	286	282	313	369	349	311
AC098591.2	1	0	0.9890559	0	1	0	1	0	0
LINC01889	2.64	1.4005379	0.0022967	18	13	19	41	38	53
LINC01881	1.3389831	0.4211377	0.3638748	51	33	34	28	92	38
NR2F1	1.4779412	0.5635888	0.0734267	43	39	54	77	65	59
NR2F2	0.9529062	-0.069594	0.8730735	1515	1448	1751	1223	1687	1582
NR2F6	1.0543887	0.0764068	0.3463262	3085	4088	4539	4548	4276	3525
AC091987.1	1	0	0.8546861	24	21	44	32	35	22
TBL3	0.8814856	-0.181991	0.3796629	2018	2519	2652	2110	2191	2036
TBL2	0.867911	-0.204381	0.2592523	1253	1421	1505	1155	1277	1195

LINC01876	1.3350785	0.4169246	0.1914545	81	30	80	68	84	103
LINC01873	1.1111111	0.1520031	0.9087974	2	5	2	2	4	4
LINC01875	1.3392857	0.4214638	0.3864247	10	24	22	35	21	19
LINC01874	4	2	0.2729956	1	0	2	0	6	6
PTPN2P1	2	1	0.6565374	0	1	1	4	0	0
PTPN2P2	#DIV/0!	#DIV/0!	0.4501902	0	0	0	0	0	4
TWF1P1	0.8742857	-0.193823	0.6809059	83	42	50	39	57	57
LINC01829	0	#NUM!	0.5781912	0	0	3	0	0	0
LINC01822	0	#NUM!	0.824326	0	0	1	0	0	0
LINC01821	#DIV/0!	#DIV/0!	0.6554062	0	0	0	0	2	0
LINC01820	#DIV/0!	#DIV/0!	0.4909737	0	0	0	2	0	1
LINC01819	0.5025381	-0.992695	0.0153214	50	64	83	28	42	29
AL627402.1	0.2	-2.321928	0.4234685	4	1	0	0	1	0
OAF	1.902439	0.9278502	0.0270921	26	22	34	78	42	36
LINC01816	1.4	0.4854268	0.7703931	3	0	2	3	4	0
AC009107.2	0.5	-1	0.3339593	13	5	18	0	11	7
ERGIC3	1.0126134	0.0180834	0.5462914	5367	6680	7139	6762	6808	5858
ERGIC2	1.1217639	0.165769	0.1769425	1266	772	1477	1154	1513	1276
ERGIC1	0.8857532	-0.175023	0.3794465	2260	2958	2861	2255	2492	2409
RNU6-431P	0.5714286	-0.807355	0.6077366	5	5	4	0	0	8
OAT	0.9434261	-0.084019	0.8235878	1359	1339	1721	1189	1546	1434
AC092336.1	0	#NUM!	0.824326	1	0	0	0	0	0
PIGFP3	0	#NUM!	0.5337954	0	3	0	0	0	0
LINC01844	0	#NUM!	0.6631647	1	0	1	0	0	0
AC099336.2	1.1046512	0.1435909	0.6636265	31	25	30	33	36	26
LINC01843	2.8	1.4854268	0.2733985	2	0	3	6	3	5
LINC01846	0	#NUM!	0.824326	1	0	0	0	0	0
IL12RB1	0	#NUM!	0.6385096	0	2	0	0	0	0
AC099336.1	1.1666667	0.2223924	0.8430573	3	1	2	4	0	3
IL12RB2	1	0	0.953845	0	2	10	0	7	5
LINC01841	#DIV/0!	#DIV/0!	0.4980481	0	0	0	2	1	0
CRYM-AS1	0.4880952	-1.034765	0.1452432	23	32	29	9	4	28
AC009097.2	0.375	-1.415037	0.4555656	3	2	3	0	2	1
COL3A1	0.4829932	-1.049925	0.0225923	59	47	41	20	24	27
LINC01836	0.8714286	-0.198546	0.5570301	132	123	165	106	143	117
LINC01835	0	#NUM!	0.6410557	1	1	0	0	0	0
RNU6-433P	0	#NUM!	0.824326	0	0	1	0	0	0
TAF1A-AS1	0.960177	-0.058628	0.9764306	163	130	159	161	151	122
AC079148.1	3	1.5849625	0.2405964	1	0	4	5	4	6
TC2N	1.3995657	0.4849792	0.0040152	691	435	716	619	968	991
LDHAL6FP	2	1	0.6385527	0	0	2	2	1	1
INO80C	0.8598131	-0.217905	0.5092974	146	212	177	161	162	137
INO80B	0.6	-0.736966	0.6719258	1	3	6	1	2	3
INO80E	0.9693593	-0.044897	0.837574	756	691	1066	827	876	733
INO80D	1.1613924	0.2158555	0.2570487	452	317	495	296	482	690
TXNDC9	1.1589279	0.2127908	0.0493514	853	716	1080	981	1125	964
AC080078.2	0.5384615	-0.893085	0.630902	3	0	10	0	3	4
RN7SL75P	0	#NUM!	0.0976961	9	0	1	0	0	0
AC118755.2	0	#NUM!	0.1286255	0	4	5	0	0	0
AC118755.1	0.25	-2	0.5498031	4	0	0	1	0	0
AP001065.2	1.6875	0.7548875	0.4406872	11	0	5	15	8	4
AP001065.1	1.122449	0.1666499	0.5894718	26	36	36	45	32	33
TXNDC2	3	1.5849625	0.6429407	0	0	1	1	2	0
TXNDC5	1.0486486	0.0685314	0.7134035	52	75	58	79	47	68

AF274858.3	0.3	-1.736966	0.3267196	6	2	2	0	2	1
BOD1	1.0589763	0.0826702	0.2250206	2401	2572	3115	2765	3120	2680
RPS10P7	0.7688679	-0.379192	0.1330451	200	183	253	135	202	152
RPS10P5	1.1818182	0.2410081	0.8413732	3	4	4	1	6	6
OGN	5	2.3219281	0.1123666	4	0	0	12	5	3
CNTROB	0.9566464	-0.063942	0.9402893	759	886	1146	919	896	855
EPHA1-AS1	0.4347826	-1.201634	0.1486385	27	10	9	6	8	6
RPS10P3	1.0986842	0.1357768	0.6096816	30	66	56	61	47	59
OGT	0.9276568	-0.108337	0.7333326	5525	3663	5962	3373	5029	5652
AL137784.2	1	0	0.9837501	1	0	2	0	2	1
AL137784.1	1	0	0.9951317	0	3	1	1	3	0
AL137784.3	1	0	0.9890559	0	0	1	0	1	0
C14orf28	0.9572426	-0.063044	0.9805095	351	326	469	354	425	318
LSM3P3	0	#NUM!	0.824326	1	0	0	0	0	0
AL355987.5	0.6818182	-0.552541	0.7262012	0	5	17	0	9	6
CTSL3P	2.6923077	1.4288433	0.1152794	6	2	5	7	17	11
AL355987.4	1.4751773	0.5608884	0.1057178	61	39	41	88	64	56
SMIM12	0.9498861	-0.074174	0.9368987	879	1033	1161	968	1064	887
SMIM14	0.997254	-0.003967	0.8196777	705	712	768	532	810	837
SMIM13	0.8622168	-0.213877	0.2371439	1162	932	1172	805	1031	980
SMIM15	0.8451133	-0.242783	0.2190448	1140	808	1009	668	1016	815
SMIM18	5	2.3219281	0.3910149	0	0	1	3	0	2
SMIM17	0.68	-0.556393	0.6573567	3	8	14	6	3	8
SMIM19	0.9085072	-0.13843	0.8335318	170	167	286	201	179	186
C14orf37	0.3181818	-1.652077	0.19558	8	11	3	3	4	0
C14orf39	3.5	1.8073549	0.3750585	0	0	2	2	1	4
MGC16275	0.1666667	-2.584963	0.3515341	0	5	1	0	1	0
SMIM20	0.9174362	-0.12432	0.7262927	513	579	749	554	616	519
SMIM22	0.2068966	-2.273018	0.06084	16	5	8	0	2	4
SMIM25	0.2666667	-1.906891	0.3143795	2	0	13	0	2	2
SMIM24	1.1052632	0.1443899	0.7769732	3	3	13	8	5	8
SMIM27	1.2058824	0.2700892	0.5697506	18	15	35	14	16	52
SMIM26	1.0995671	0.1369356	0.5168973	78	68	85	91	98	65
SMIM29	1.3739669	0.4583473	0.0235678	131	168	185	249	229	187
SMIM28	0.5	-1	0.81753	2	0	0	0	0	1
PHKG1	0.7428571	-0.428843	0.6541808	16	5	14	8	12	6
PHKG2	1.0799794	0.1110037	0.2888667	637	595	706	786	710	597
AL353194.1	0.7681159	-0.380604	0.6379493	29	19	21	22	6	25
SMIM30	3.3636364	1.7500217	0.0767555	3	0	8	9	20	8
SMIM32	0	#NUM!	0.824326	0	0	1	0	0	0
TBX1	0.7307692	-0.452512	0.702173	2	10	14	4	6	9
AL122018.1	3	1.5849625	0.4868176	1	1	0	6	0	0
TUBA3FP	0.6666667	-0.584963	0.850543	1	1	1	1	0	1
TBX6	0.2962963	-1.754888	0.180876	4	5	18	3	5	0
TBX4	4	2	0.409782	1	1	0	0	0	8
TBX3	1.9016333	0.927239	2.22E-13	891	826	977	1350	1890	1883
TBX2	0.7802747	-0.357946	0.1378026	237	244	320	206	225	194
SUCLA2	1.0112057	0.0160766	0.6996821	804	581	846	596	946	714
HERC5	2.2497078	1.1697377	8.98E-19	904	723	940	1556	2267	1952
HERC4	1.2625321	0.3363201	0.0274141	1158	780	1174	922	1563	1444
C1GALT1C1	1.0956921	0.1318424	0.1345898	896	838	1098	910	1136	1057
HERC3	1.107567	0.147394	0.2694942	978	947	1022	740	1231	1293
HERC2	0.6474973	-0.627054	0.0008938	1723	2060	1851	808	1289	1551
HERC1	0.7734279	-0.370661	0.0860018	1463	1180	1444	681	1031	1449

FARSA-AS1	#DIV/0!	#DIV/0!	0.6554062	0	0	0	0	2	0
HERC6	1.7877462	0.8381419	2.12E-05	155	154	148	242	274	301
AC005230.1	1	0	0.9890559	1	0	0	0	1	0
AL122017.1	1	0	0.9733892	0	2	3	0	0	5
AC023908.3	0.5396825	-0.889817	0.2593399	23	11	29	17	11	6
OMG	0.4285714	-1.222392	0.4428664	0	5	16	4	1	4
AC007773.1	0.9354839	-0.096215	0.9455635	6	13	12	7	13	9
EIF4A1P10	0.9196697	-0.120812	0.8146693	398	451	483	484	389	352
AC062029.1	0.9955947	-0.00637	0.9104512	82	72	73	63	88	75
AL357552.2	#DIV/0!	#DIV/0!	0.3877895	0	0	0	2	2	0
AC063976.3	1	0	0.9756864	2	0	0	0	2	0
AC063976.2	1.1764706	0.2344653	0.8356354	5	5	7	0	10	10
AC063976.1	0.65	-0.621488	0.2217881	27	51	42	25	27	26
AC023512.1	#DIV/0!	#DIV/0!	0.3096126	0	0	0	5	0	0
AC137630.3	0	#NUM!	0.6840298	0	0	2	0	0	0
AC137630.2	1.625	0.7004397	0.6128938	1	5	2	4	0	9
GNRH2	0.1428571	-2.807355	0.3019873	4	0	3	0	0	1
GNRH1	1.2	0.2630344	0.5981455	10	9	21	15	18	15
PAK1IP1	0.9448293	-0.081874	0.9012118	1431	981	1630	1116	1452	1251
BOLL	1.4594595	0.5454341	0.4155292	13	10	14	16	32	6
NUDCD3	0.8159494	-0.293448	0.050109	2357	2688	3181	1975	2362	2375
NUDCD2	1.0514066	0.0723207	0.2954609	1643	1387	1911	1588	1969	1638
NUDCD1	1.05309	0.0746287	0.4541404	901	576	934	669	973	897
GNRHR	0.6923077	-0.530515	0.7128096	4	3	6	1	3	5
KIF7	0.7454545	-0.423808	0.4975024	28	22	60	16	30	36
KIF6	1.2268041	0.2949049	0.2095297	99	99	93	98	121	138
3LC25A25-AS	0.7046784	-0.504963	0.1117408	135	95	112	58	91	92
DEPDC7	1.4102564	0.4959575	0.4588739	15	4	20	9	32	14
KIF9	1.0432692	0.0611115	0.6879974	79	52	77	77	66	74
DEPDC1	1.3696809	0.4538398	0.0050359	1344	775	1265	1177	1815	1643
DEPDC5	0.992281	-0.011179	0.8473297	776	877	938	680	1099	792
EEF1E1	1.1896104	0.2504892	0.1925826	133	97	155	140	170	148
DEPDC4	0.8786408	-0.186655	0.7132584	63	60	83	59	66	56
ARNTL2	1.093709	0.129229	0.2293808	516	417	593	486	640	543
RN7SL67P	1	0	0.9688499	5	3	2	0	8	2
CYP17A1	0.7222222	-0.469485	0.6816672	3	9	6	2	8	3
OS9	0.8939519	-0.161731	0.3743563	4954	5820	6190	4752	5376	5037
AC104521.1	2	1	0.6867323	2	2	0	0	8	0
ISCA1P6	#DIV/0!	#DIV/0!	0.8030358	0	0	0	0	0	1
PGGT1B	1.1236264	0.1681624	0.1591194	339	312	441	359	440	428
ISCA1P4	0	#NUM!	0.824326	0	0	1	0	0	0
AL513314.2	1.0769231	0.1069152	0.906861	1	2	10	0	11	3
ISCA1P1	1.3188406	0.3992702	0.3470886	19	23	27	34	34	23
PICART1	3.5	1.8073549	0.4069639	2	0	0	2	1	4
AC007533.1	#DIV/0!	#DIV/0!	0.8030358	0	0	0	0	1	0
RN7SL68P	0	#NUM!	0.5387425	3	0	0	0	0	0
BOP1	0.8633794	-0.211933	0.3278608	2156	2724	2725	2343	2214	2009
SMAD1-AS2	#DIV/0!	#DIV/0!	0.8030358	0	0	0	0	1	0
TCEA3	1.2648649	0.3389833	0.2386968	47	72	66	79	91	64
TCEA2	1.2885045	0.3656975	0.0160389	475	619	698	883	740	686
TCAP	0.875	-0.192645	0.9027333	3	10	3	7	3	4
TCEA1	0.9810407	-0.027615	0.958118	1857	1426	1675	1160	1812	1892
FP700111.2	2.5	1.3219281	0.6338788	0	0	2	0	5	0
MANEA-AS1	0.4761905	-1.070389	0.2529019	19	4	19	7	5	8

AL451165.2	0.5609756	-0.83399	0.3069561	19	10	12	6	8	9
OTP	#DIV/0!	#DIV/0!	0.8030358	0	0	0	0	0	1
NR3C1	0.8424191	-0.24739	0.2713197	351	349	474	264	361	364
NR3C2	1.5639098	0.6451573	0.0135115	75	85	106	94	143	179
EEF1B2	0.9793309	-0.030132	0.7772751	11933	13562	15097	14193	13720	11840
BORA	0.9468792	-0.078748	0.9369291	558	362	586	445	517	464
EEF1A1	0.9763903	-0.03447	0.8388545	172165	182747	209433	165202	202917	182902
EEF1A2	1.3969336	0.4822634	0.0098451	160	213	214	287	277	256
ODF3B	0.3195876	-1.645717	0.0104626	42	21	34	3	14	14
TCF7	0.9760274	-0.035006	0.9982314	78	116	98	72	120	93
LINC01807	1.32	0.4005379	0.58906	6	14	5	17	7	9
ODF2L	1.4926151	0.5778422	0.0086389	470	194	487	453	692	573
LINC01804	#DIV/0!	#DIV/0!	0.6411933	0	0	0	0	0	2
LINC01806	1.1648649	0.2201626	0.287334	137	97	136	111	151	169
LINC01800	0.3636364	-1.459432	0.2918029	6	4	12	4	0	4
LINC01801	#DIV/0!	#DIV/0!	0.6554062	0	0	0	0	2	0
AC011611.2	0.1428571	-2.807355	0.3837182	0	0	7	1	0	0
AC104667.2	0.7477064	-0.419456	0.2939352	62	68	88	55	63	45
AMOTL2	0.9126697	-0.131835	0.4812783	5079	5692	6096	4114	5613	5667
TCF4	0.6666667	-0.584963	0.8484366	0	2	1	0	0	2
AMOTL1	0.8722088	-0.197255	0.325819	3556	3901	3694	2123	3483	4120
TCF3	1.1387221	0.1874157	0.0633795	1332	1586	1746	1720	1938	1653
GALK2	0.7770954	-0.363836	0.0299376	824	915	1029	572	769	810
GALK1	0.7723283	-0.372714	0.0693878	539	730	668	505	520	471
TCHH	0.5565093	-0.845522	0.0002369	218	203	278	106	149	134
AL354919.2	0	#NUM!	0.6840298	0	0	2	0	0	0
TCHP	0.7855228	-0.348275	0.1071381	385	311	423	253	293	333
GDAP1L1	1	0	0.9890559	0	1	0	1	0	0
UST-AS1	#DIV/0!	#DIV/0!	0.8030358	0	0	0	0	1	0
AC008759.2	#DIV/0!	#DIV/0!	0.6411933	0	0	0	0	0	2
RN7SL336P	#DIV/0!	#DIV/0!	0.8030358	0	0	0	0	1	0
CHI3L2	2.25	1.169925	0.5246446	1	2	1	0	4	5
SLC4A1APP1	2.12	1.0840643	0.0998976	8	7	10	16	20	17
SGK494	0.8839286	-0.177998	0.8300577	42	21	49	24	24	51
NECAP1P1	0	#NUM!	0.824326	0	0	1	0	0	0
AC073320.1	2	1	0.8319548	0	1	0	0	2	0
KCTD21-AS1	0.7124183	-0.489204	0.2884327	43	55	55	38	31	40
GALNS	0.8393997	-0.25257	0.1513922	935	1108	1089	785	1002	842
TCIM	5.3333333	2.4150375	0.0020208	5	4	3	23	29	12
LINC01748	1.2608696	0.334419	0.7530958	17	6	0	9	7	13
RASSF8-AS1	0.8924051	-0.164229	0.6874121	140	141	193	134	133	156
LINC01740	0.5	-1	0.81106	0	2	0	0	0	1
ATG10	0.9005337	-0.151148	0.4804499	661	659	741	549	668	639
ATG14	1.0222762	0.0317851	0.5267708	796	676	997	694	951	879
ATG13	0.9329643	-0.100106	0.7057776	1912	2286	2306	1858	2235	1975
ATG12	1.0094107	0.0135133	0.6107238	1578	1162	1723	1284	1688	1533
GABRG2	1.3173653	0.3976554	0.164469	45	42	80	64	75	81
GABRG1	0.5	-1	0.7190215	1	1	2	0	0	2
LINC01738	0	#NUM!	0.824326	0	0	1	0	0	0
LINC01737	#DIV/0!	#DIV/0!	0.8030358	0	0	0	1	0	0
RNU6-447P	1	0	0.9239532	0	0	2	2	0	0
LINC01730	#DIV/0!	#DIV/0!	0.8030358	0	0	0	0	0	1
LINC01732	#DIV/0!	#DIV/0!	0.8030358	0	0	0	0	1	0
TCL6	1.828125	0.8703647	0.0559799	19	13	32	40	49	28

LINC01731	0	#NUM!	0.824326	0	0	1	0	0	0
LINC01770	0.9912281	-0.012711	0.9439517	53	26	35	34	44	35
LINC01772	0.7727273	-0.371969	0.7593929	5	8	9	0	4	13
LINC01771	0	#NUM!	0.5643268	1	0	2	0	0	0
PRKRIP1	0.9260143	-0.110894	0.7693699	824	969	1140	920	983	813
AC022432.1	#DIV/0!	#DIV/0!	0.5371397	0	0	0	0	0	3
LINC01767	#DIV/0!	#DIV/0!	0.6411933	0	0	0	0	0	2
AC007285.2	1.6206897	0.6966079	0.3214033	9	1	19	16	19	12
LINC01766	#DIV/0!	#DIV/0!	0.6554062	0	0	0	0	2	0
AC007285.1	0.125	-3	0.2028407	5	2	1	0	0	1
LINC01762	1.3846154	0.4694853	0.6399403	5	4	4	7	9	2
AC016065.1	0.875	-0.192645	0.6925245	116	87	101	109	85	72
NR1D2	0.8875817	-0.172048	0.5330293	588	361	581	344	501	513
AC015726.1	0.8636364	-0.211504	0.9493652	5	3	14	9	5	5
NR1D1	0.7537879	-0.407769	0.1796666	155	204	169	140	162	96
AC114546.1	0	#NUM!	0.4460243	4	0	0	0	0	0
GNPTG	1.0437441	0.0617681	0.4097619	612	678	836	805	768	646
TCN2	0.3165468	-1.659509	0.0009818	45	47	47	16	17	11
LINC01759	0.4387755	-1.188445	0.0527064	33	21	44	5	21	17
PAH	1	0	0.9898221	2	3	3	2	6	0
ATG2A	1.1067524	0.1463325	0.141974	1252	1644	1769	1517	1872	1774
LINC01756	2	1	0.8147319	1	0	0	0	0	2
AC006946.1	0.1428571	-2.807355	0.2984508	0	4	3	0	1	0
FARSA	0.9321988	-0.10129	0.8174236	2097	2477	2771	2418	2447	1982
PAM	0.9849441	-0.021886	0.8198195	3348	3153	3794	2723	3806	3611
LINC01754	1	0	0.9645583	0	7	1	0	5	3
ATG2B	1.1735016	0.2308198	0.1166992	1357	849	1281	991	1584	1517
FARSB	1.119487	0.1628377	0.0392667	4925	4398	5959	5097	6215	5796
GABRB3	1.52	0.6040713	0.4873775	13	3	9	3	19	16
GABRB2	0.1111111	-3.169925	0.1628093	3	3	3	0	0	1
GABRB1	3.2857143	1.716207	0.1309021	3	2	2	6	4	13
FARS2	0.8088601	-0.306038	0.1305802	616	682	711	559	595	471
AC092335.1	0.8	-0.321928	0.879748	0	5	0	0	0	4
LINC01707	#DIV/0!	#DIV/0!	0.8030358	0	0	0	0	0	1
AL354761.1	0	#NUM!	0.824326	1	0	0	0	0	0
PBK	0.8302642	-0.268358	0.2343752	450	340	459	331	379	327
LINC01703	0.8888889	-0.169925	0.8167434	23	28	12	19	15	22
SGMS1-AS1	0.9147287	-0.128584	0.8289246	47	39	43	28	41	49
U52111.1	0.3055556	-1.710493	0.0970819	16	9	11	0	4	7
HADHB	0.9288148	-0.106537	0.6934403	2340	2572	3011	2300	2633	2426
HADHA	1.0275651	0.0392298	0.4117579	5941	6834	7468	6156	7710	6935
COL2A1	0.8823529	-0.180572	0.7126977	76	60	68	51	50	79
TCP1	0.9566525	-0.063933	0.9535745	11589	10935	13718	10380	12589	11702
AC044810.2	1.2903226	0.3677318	0.494968	33	11	18	17	32	31
NR1I3	1.3220339	0.4027592	0.4153555	8	27	24	27	27	24
NR1I2	0.625	-0.678072	0.6963846	2	6	0	0	1	4
AL451164.1	0	#NUM!	0.824326	0	1	0	0	0	0
PDC	0	#NUM!	0.5781912	0	0	3	0	0	0
RN7SL351P	#DIV/0!	#DIV/0!	0.3597364	0	0	0	0	0	5
PDF	0.8710462	-0.199179	0.5483584	217	263	342	248	233	235
LINC01726	0	#NUM!	0.5337954	0	3	0	0	0	0
LINC01722	0	#NUM!	0.5172237	1	2	0	0	0	0
LINC01725	#DIV/0!	#DIV/0!	0.6317974	0	0	0	1	1	0
FARP2	1.0323707	0.0459611	0.5295778	1000	988	1163	821	1128	1304

FARP1	0.9226638	-0.116123	0.6157122	1246	1159	1319	915	1205	1316
GABRA4	1.2352941	0.3048546	0.7927656	6	10	1	4	5	12
NR1H2	0.864124	-0.21069	0.2195823	1497	1725	1812	1342	1531	1477
GABRA3	0.63331	-0.659016	0.0008788	415	479	535	214	339	352
NR1H4	#DIV/0!	#DIV/0!	0.5509701	0	0	0	0	3	0
NR1H3	1.2388451	0.3089959	0.0135088	824	1086	1138	1260	1255	1261
LINC01719	0.7365854	-0.441075	0.2678838	74	62	69	26	71	54
LINC01716	0.9082569	-0.138828	0.9552684	28	21	60	38	34	27
LINC01715	1.5	0.5849625	0.8307205	1	0	1	0	2	1
LINC01714	1.525	0.6088092	0.316994	15	16	9	25	24	12
LINC01713	#DIV/0!	#DIV/0!	0.5509701	0	0	0	0	3	0
RN7SL65P	#DIV/0!	#DIV/0!	0.8030358	0	0	0	0	0	1
GPAT2P2	#DIV/0!	#DIV/0!	0.2327526	0	0	0	0	0	7
PGD	0.7426118	-0.42932	0.0020914	5191	6234	6509	4153	4878	4287
MIR100HG	0.8971963	-0.156504	0.7615239	55	28	24	17	42	37
PGC	0.7941176	-0.332575	0.5667527	28	35	39	27	24	30
PGF	1.6953721	0.761602	6.92E-09	7805	9238	10745	17155	16206	13750
PGP	0.8770875	-0.189207	0.5190725	1680	2059	2249	2046	1860	1346
TCTA	0.7200393	-0.473852	0.0077222	636	673	727	496	489	481
KRBOX1-AS1	#DIV/0!	#DIV/0!	0.8030358	0	0	0	0	0	1
PHB	0.8993738	-0.153007	0.4797466	4512	5604	5854	4752	5079	4532
Z99916.1	0	#NUM!	0.5387425	3	0	0	0	0	0
ZNF767P	0.6808847	-0.554518	0.0383151	203	179	251	149	123	159
PI3	0.6290323	-0.668794	0.3006853	22	23	17	10	17	12
RUVBL1-AS1	0.3333333	-1.584963	0.6622061	0	3	0	0	0	1
PIR	0.7016	-0.511279	0.0148542	400	359	491	267	367	243
RSL24D1	1.0979631	0.1348296	0.1919609	2650	1795	2772	2227	3063	2634
THEMIS3P	0	#NUM!	0.824326	0	1	0	0	0	0
TCIRG1	0.9632314	-0.054046	0.9005226	1628	1866	2163	1930	1765	1754
PP14571	1.3386752	0.420806	0.0055585	320	252	364	408	450	395
LINC01789	0	#NUM!	0.824326	0	1	0	0	0	0
NPIPA3	1.75	0.8073549	0.7520285	4	0	0	0	6	1
AC005865.2	3.5	1.8073549	0.39438	0	0	2	3	0	4
LINC01788	0	#NUM!	0.824326	0	0	1	0	0	0
NPIPA2	1.5714286	0.6520767	0.6552667	3	0	4	1	7	3
NPIPA1	0.8902439	-0.167727	0.7629712	87	88	153	96	105	91
HMGCS2	0.7640449	-0.388271	0.47764	28	36	25	18	30	20
NPIPA7	0.4545455	-1.137504	0.3880109	7	3	12	6	2	2
TOP3BP1	0	#NUM!	0.6385096	0	2	0	0	0	0
NPIPA5	1.6666667	0.7369656	0.2888849	5	8	11	14	10	16
LINC01786	1.2	0.2630344	0.8950908	5	0	0	2	0	4
LINC01781	0	#NUM!	0.6840298	0	0	2	0	0	0
LINC01783	1.3333333	0.4150375	0.6593139	2	1	9	4	7	5
HMGCS1	0.8459129	-0.241419	0.1571078	4440	3807	4843	2679	4103	4291
AC023906.5	1.2	0.2630344	0.8171321	0	0	5	2	1	3
AC023906.4	0.5	-1	0.7343547	0	2	2	0	0	2
BPGM	1.1606914	0.2149845	0.0658823	474	492	596	568	682	563
AC023906.1	#DIV/0!	#DIV/0!	0.8030358	0	0	0	0	0	1
PKM	0.9550671	-0.066326	0.9645497	32695	39361	42359	35744	38857	34673
AL109741.2	#DIV/0!	#DIV/0!	0.8030358	0	0	0	0	0	1
NOS1AP	0.7362637	-0.441705	0.2936221	53	66	63	31	42	61
SUPT16HP1	0.8888889	-0.169925	0.9336703	6	0	3	2	4	2
LINC01779	0.3333333	-1.584963	0.6628865	2	0	1	0	0	1
LINC01775	0.8076923	-0.308122	0.8365785	4	7	15	7	4	10

NPIPB9	0.5769231	-0.793549	0.2744659	22	13	17	6	12	12
BPHL	0.8991098	-0.153431	0.5999019	272	367	372	263	307	339
NPIPA8	0.6071429	-0.719892	0.5754821	10	8	10	13	1	3
AC004057.1	0.9857446	-0.020714	0.7247566	4272	5267	6034	5281	5383	4687
AC023510.2	#DIV/0!	#DIV/0!	0.4155775	0	0	0	0	2	2
PML	1.4619377	0.5478818	7.99E-05	611	846	855	1036	1173	1171
GYG2P1	1.1391304	0.187933	0.4041364	76	69	85	102	91	69
NPIPB4	0.8157895	-0.293731	0.3983793	125	111	144	109	92	109
NPIPB3	1.0934066	0.12883	0.424981	103	100	161	161	110	127
NPIPB2	1.3333333	0.4150375	0.3120789	14	23	38	33	35	32
AL445187.1	0.6	-0.736966	0.6498842	6	0	9	0	6	3
LINC01796	1	0	0.9890559	0	1	0	0	0	1
NPIPB8	0.5	-1	0.54835	7	2	3	3	0	3
NPIPB6	0.6818182	-0.552541	0.7062295	3	3	16	5	4	6
NPIPB5	0.8373959	-0.256018	0.227909	3361	2238	4124	2419	2966	2757
ATP5G2P4	1.4	0.4854268	0.7321373	1	2	2	5	2	0
AC062028.1	2.1363636	1.0951572	0.1939216	13	6	3	7	22	18
PNN	1.0482956	0.0680456	0.4427936	5744	3213	6738	4591	6367	5495
PNP	0.913496	-0.13053	0.5813295	4141	4849	5518	4300	4804	4149
AC062028.2	#DIV/0!	#DIV/0!	0.1338503	0	0	0	4	1	2
GYG1P3	#DIV/0!	#DIV/0!	0.8030358	0	0	0	0	0	1
GYG1P1	2	1	0.834705	0	0	1	0	2	0
POR	0.9124189	-0.132232	0.5841223	1908	2089	2477	1923	2039	1945
FP565260.1	0.7155477	-0.48288	0.0548885	169	189	208	123	157	125
OXCT2P1	2.2	1.1375035	0.6933506	10	0	0	15	0	7
FP565260.3	0.8948413	-0.160296	0.5953462	278	384	346	303	316	283
FP565260.4	0.6666667	-0.584963	0.8097894	1	2	0	0	1	1
SUCLG2	0.7302834	-0.453472	0.00722	892	851	1186	553	780	806
FP565260.7	0.9937888	-0.008989	0.8035215	35	47	79	70	45	45
SUCLG1	0.9724039	-0.040372	0.84877	4123	4539	5253	4443	4843	4245
FP565260.6	0.9753086	-0.036069	0.9302646	19	24	38	25	27	27
PPL	1.1460211	0.1966336	0.0782278	1060	1287	1536	1168	1681	1601
AC068775.1	1.0650685	0.0909462	0.6789887	100	89	103	69	146	96
PPY	0.3421053	-1.547488	0.032116	28	22	26	12	3	11
AC244505.1	0.1666667	-2.584963	0.3558995	0	4	2	0	0	1
LIN28AP2	#DIV/0!	#DIV/0!	0.8030358	0	0	0	0	0	1
LIN28AP1	#DIV/0!	#DIV/0!	0.3109758	0	0	0	0	6	0
SPPL3	0.9310418	-0.103082	0.7033412	1388	1666	1659	1356	1682	1350
CU633906.2	0.9456522	-0.080618	0.9221093	25	38	29	24	38	25
CU633906.1	0.8	-0.321928	0.7797717	9	5	6	0	5	11
GABRR2	0.375	-1.415037	0.4880211	6	0	2	0	2	1
GABRR1	0.8157895	-0.293731	0.741392	20	13	5	13	9	9
XPO1	1.1575535	0.2110789	0.0900448	11598	8142	11570	8800	13577	13866
XPO4	0.9583529	-0.061371	0.9271587	1689	991	1570	905	1449	1719
PRY	1	0	0.9998014	3	0	0	1	1	1
XPO6	0.8426419	-0.247008	0.0870259	3299	3550	4037	2558	3340	3275
PRX	0.9873708	-0.018336	0.8699228	250	339	282	281	260	319
XPO5	0.836	-0.258425	0.0727626	3652	3989	4109	2755	3615	3453
PSD	1.0285714	0.040642	0.7969295	12	2	21	26	6	4
CU633906.3	0	#NUM!	0.6385096	0	2	0	0	0	0
AC244517.12	5	2.3219281	0.4094329	1	0	0	5	0	0
AC244517.11	#DIV/0!	#DIV/0!	0.5509701	0	0	0	0	3	0
NHP2P1	1.75	0.8073549	0.6263615	1	1	2	2	4	1
RN7SL55P	0	#NUM!	0.824326	0	1	0	0	0	0

PTN	1.0334204	0.0474273	0.3524743	2357	2197	2717	2379	2730	2405
AC140481.3	#DIV/0!	#DIV/0!	0.6251462	0	0	0	1	0	1
PTS	0.8016194	-0.319011	0.1001924	660	664	899	597	611	574
AL589843.2	#DIV/0!	#DIV/0!	0.8030358	0	0	0	1	0	0
ZMYM4-AS1	0	#NUM!	0.824326	0	0	1	0	0	0
SLC31A2	1.6363636	0.7104934	0.3350347	3	8	11	13	13	10
SLC31A1	1.1211721	0.1650077	0.0636315	3950	3925	4240	3803	5049	4731
XPO7	0.8594606	-0.218497	0.1544921	3233	3109	3520	2291	3046	3139
XPOT	1.0013142	0.0018948	0.728217	5074	4146	5237	3657	5269	5550
AC127537.3	0.3333333	-1.584963	0.4798099	1	4	1	0	2	0
RIPOR3	0.4444444	-1.169925	0.3577359	8	5	5	0	5	3
RIPOR2	1.2166667	0.282934	0.1204542	171	192	177	188	238	231
RIPOR1	0.6373821	-0.64977	0.0003943	478	530	688	353	384	344
PVR	1.0454545	0.0641303	0.8921476	6	10	6	9	2	12
C1RL	0.734375	-0.445411	0.4072476	39	33	56	35	36	23
AC114296.1	1	0	0.9991771	1	3	2	0	2	4
AC011120.1	#DIV/0!	#DIV/0!	0.6099294	0	0	0	2	0	0
XPR1	1.0165532	0.0236858	0.7151687	1972	1646	1819	1300	2058	2169
EIF3J-AS1	0.8521257	-0.230862	0.3271242	331	327	424	272	373	277
PXK	0.9125506	-0.132024	0.6550793	380	359	496	290	416	421
PXN	0.8572405	-0.222228	0.1661224	5897	7175	7410	4876	6219	6463
RNU11-3P	#DIV/0!	#DIV/0!	0.6411933	0	0	0	0	0	2
NDUFA5P11	1.7931034	0.8424587	0.2495476	9	7	13	6	30	16
NDUFA5P10	0	#NUM!	0.4374838	0	4	0	0	0	0
BPTF	1.0659756	0.0921745	0.4161857	2293	1476	2309	1540	2358	2581
AL627309.1	0.5	-1	0.81106	0	2	0	0	0	1
SREK1IP1	1.1122129	0.153433	0.2305665	722	456	738	580	787	764
RNU11-4P	#DIV/0!	#DIV/0!	0.8030358	0	0	0	1	0	0
PYY	0.875	-0.192645	0.8774918	10	6	8	7	8	6
AL627309.7	1.0428571	0.0605415	0.7913521	22	12	36	26	33	14
AL627309.8	1.0674157	0.0941222	0.7401569	30	20	39	32	44	19
AL627309.2	0.6285714	-0.669851	0.5181938	11	4	20	1	10	11
AL627309.5	2	1	0.6417445	3	0	1	0	0	8
PZP	1.5	0.5849625	0.8695629	0	2	0	0	0	3
NR2E1	0	#NUM!	0.0759911	9	0	2	0	0	0
NR2E3	1.75	0.8073549	0.6354168	0	2	2	2	3	2
AC036176.1	1.2	0.2630344	0.8701743	0	0	5	0	5	1
AC087280.2	0.3333333	-1.584963	0.5638631	1	0	5	0	2	0
AC087280.1	#DIV/0!	#DIV/0!	0.8030358	0	0	0	0	0	1
NPIPP1	0.65	-0.621488	0.5806324	6	9	5	5	5	3
AC015727.1	0	#NUM!	0.824326	1	0	0	0	0	0
AC139099.1	0.6956522	-0.523562	0.6481772	9	4	10	0	13	3
ATF6B	0.9459172	-0.080214	0.8751999	2789	2936	3668	2753	3255	2877
AC139099.4	2	1	0.7741804	1	0	0	2	0	0
NR2C2	0.8542865	-0.227208	0.349147	1222	1025	1404	635	1060	1424
RTEL1P1	3.4	1.7655347	0.2667105	2	0	3	0	12	5
NR2C1	1.090937	0.1255678	0.2666188	1158	794	1303	921	1415	1215
MCRIP2	1.1502783	0.2019829	0.2652257	149	199	191	240	210	170
MCRIP1	1.0547599	0.0769146	0.3646805	1059	1180	1322	1404	1310	1042
AC008758.4	#DIV/0!	#DIV/0!	0.4501902	0	0	0	0	0	4
AC008758.6	0	#NUM!	0.824326	1	0	0	0	0	0
AL034374.2	0	#NUM!	0.5781912	0	0	3	0	0	0
OR1F12	0.75	-0.415037	0.9223671	0	0	4	0	1	2
RABGEF1	1.1676984	0.2236677	0.0492767	594	495	688	622	744	709

RABGEF1	1.1676984	0.2236677	0.0492767	594	495	688	622	744	709
TDO2	2.625	1.3923174	0.0841941	5	6	5	10	19	13
AC078899.2	0	#NUM!	0.824326	0	1	0	0	0	0
RUFY1	0.8561039	-0.224142	0.1735538	1180	1214	1456	958	1255	1083
AC078899.1	1.1052632	0.1443899	0.8443513	5	19	14	5	22	15
ARSDP1	0.5384615	-0.893085	0.3975205	6	11	9	2	3	9
RUFY3	0.9424597	-0.085497	0.8499308	1091	829	1243	779	1046	1156
RUFY2	1.2131148	0.278716	0.1361161	444	289	365	306	538	488
AC002091.1	1.2676056	0.342106	0.3915712	18	21	32	31	28	31
AC002091.2	0.7	-0.514573	0.3899037	32	18	40	15	24	24
TTYH3	0.7548599	-0.405719	0.0084093	2488	3241	3119	2000	2397	2282
AC026770.1	#DIV/0!	#DIV/0!	0.3981469	0	0	0	4	0	0
TTYH2	5	2.3219281	0.2255041	0	1	1	1	5	4
FO680682.1	#DIV/0!	#DIV/0!	0.8030358	0	0	0	0	1	0
TTYH1	1.483871	0.5693656	0.0243734	83	78	87	118	149	101
AGAP2	1.4655172	0.5514099	0.0636345	39	67	68	86	88	81
CLCNKB	1.36	0.4436067	0.2991209	12	24	39	38	23	41
AGAP1	0.9311433	-0.102925	0.6721723	2693	2712	3149	2039	2821	3105
CLCNKA	0.625	-0.678072	0.7204629	2	4	2	0	0	5
AGAP4	1.1160714	0.1584294	0.2659741	265	190	329	290	256	329
AGAP3	0.9572265	-0.063068	0.9465139	576	765	880	779	730	617
HESX1	0.9152542	-0.127756	0.9079061	22	15	22	17	19	18
PUSL1	0.8811245	-0.182582	0.6170021	332	432	481	432	366	299
HAGLR	#DIV/0!	#DIV/0!	0.0325685	0	0	0	7	4	1
AC009095.1	1.4074074	0.49304	0.4816819	9	5	13	8	18	12
ABTB2	1.9718388	0.9795416	2.64E-14	548	608	655	1032	1347	1192
ABTB1	0.9120674	-0.132788	0.7787156	271	364	434	354	332	289
TDP2	0.9178957	-0.123598	0.6140242	1510	1296	1737	1254	1633	1283
TDP1	0.9554009	-0.065822	0.9030877	1223	1255	1401	1035	1311	1360
WFIKKN1	1.9166667	0.9385995	0.1691661	5	8	11	21	12	13
ODF3L2	0	#NUM!	0.2452252	0	5	1	0	0	0
TDRP	1.2171205	0.283472	0.001811	2574	2498	3117	2894	3582	3491
ODF3L1	5.5	2.4594316	0.0691624	0	2	2	3	10	9
RCSD1	4.4	2.1375035	0.1057813	0	0	5	10	9	3
FASTK	0.8683657	-0.203625	0.418466	1063	1218	1525	1198	1073	1034
AC011363.1	0.5	-1	0.8241474	0	1	1	0	0	1
AP001793.2	0	#NUM!	0.6604275	0	1	1	0	0	0
LEFTY1	0	#NUM!	0.824326	0	0	1	0	0	0
AC006213.6	1.4117647	0.4974997	0.5394522	6	5	6	13	7	4
AC006213.5	1.3333333	0.4150375	0.859548	2	0	1	0	1	3
AC006213.4	0.2777778	-1.847997	0.1798736	10	3	5	2	2	1
AC006213.3	0.8055556	-0.311944	0.7038449	22	10	4	2	10	17
AC006213.2	4.5	2.169925	0.1100348	1	1	2	3	6	9
AC006213.1	0.95	-0.074001	0.9524939	8	4	8	13	4	2
AC005379.1	1.1666667	0.2223924	0.9253452	1	4	1	0	4	3
MZT2B	1.3898305	0.474909	0.1012179	41	65	71	88	82	76
MZT2A	1.0320405	0.0454995	0.5154434	357	361	468	448	429	347
AC008610.1	0.6486486	-0.624491	0.3432058	27	19	28	9	31	8
BTAF1	1.2424508	0.3131887	0.1159479	937	452	896	605	1050	1184
CLPTM1	0.8248299	-0.277831	0.1129272	2728	3492	3776	2712	2816	2717
AC096540.1	3.6666667	1.8744691	0.2936565	3	0	0	4	3	4
OR1F2P	#DIV/0!	#DIV/0!	0.0960372	0	0	0	10	0	0
AC125232.2	0.7972973	-0.32681	0.5328322	45	63	40	44	26	48
AC125232.1	1.1278195	0.1735363	0.400723	75	85	106	104	114	82

PLXDC2	1.7928135	0.8422254	1.09E-09	422	416	470	644	870	831
PLXDC1	1	0	0.9009018	28	14	34	20	31	25
SPSB3	0.5333333	-0.906891	0.3953589	14	13	3	3	13	0
SPSB2	0.8630573	-0.212472	0.5402439	198	202	228	207	197	138
RN7SL362P	0	#NUM!	0.5387425	3	0	0	0	0	0
SPSB1	0.918784	-0.122202	0.6728152	621	777	806	650	737	638
SPSB4	0.0625	-4	0.0401575	4	11	1	1	0	0
AGBL5-IT1	0.2439024	-2.035624	0.0623899	28	2	11	3	6	1
SCMH1	0.9825871	-0.025343	0.938893	147	103	152	88	142	165
TRABD2B	#DIV/0!	#DIV/0!	0.6099294	0	0	0	2	0	0
TRABD2A	1.0224241	0.0319937	0.5368326	2475	1963	2608	1915	2703	2586
CYP19A1	2.2159091	1.1478987	5.39E-20	2765	2134	2845	4559	6570	6031
AL354760.1	1	0	0.9890559	0	0	1	0	1	0
AC023905.1	0.2	-2.321928	0.4051918	2	1	2	1	0	0
QKI	0.9544448	-0.067266	0.8528414	6393	4965	6247	4048	6299	6456
FASLG	3.6666667	1.8744691	0.048161	3	2	7	3	24	17
AC093162.2	0.6	-0.736966	0.3244395	35	14	16	19	13	7
AC093162.1	1.5	0.5849625	0.7228062	5	1	0	4	4	1
RDH10-AS1	0.25	-2	0.4139298	0	3	5	0	0	2
CSTF3-AS1	0	#NUM!	0.6631647	1	0	1	0	0	0
AC006453.2	0.9243856	-0.113433	0.8822301	145	178	206	190	161	138
GANAB	0.8026168	-0.317217	0.0230466	16238	19377	19415	12726	15903	15539
ATF1P1	0	#NUM!	0.824326	0	0	1	0	0	0
FARP1-AS1	0	#NUM!	0.6436235	2	0	0	0	0	0
AC092574.2	0.4	-1.321928	0.6016774	5	0	0	0	1	1
AC092574.1	#DIV/0!	#DIV/0!	0.4501902	0	0	0	0	0	4
AC078909.2	1.3333333	0.4150375	0.9154174	3	0	0	0	4	0
AL031733.1	#DIV/0!	#DIV/0!	0.6554062	0	0	0	0	2	0
AC120042.2	4	2	0.5272053	0	0	1	1	3	0
AC120042.1	#DIV/0!	#DIV/0!	0.5371397	0	0	0	0	0	3
Z84723.1	0	#NUM!	0.6385096	0	2	0	0	0	0
AL137786.1	10	3.3219281	0.1336134	0	0	1	7	0	3
KEAP1	0.7381939	-0.437928	0.0106318	1447	1589	1792	1269	1175	1120
MYCBP2-AS1	#DIV/0!	#DIV/0!	0.2164227	0	0	0	3	0	3
TET2-AS1	0	#NUM!	0.6436235	2	0	0	0	0	0
SCN1B	0.725	-0.463947	0.6054163	11	19	10	11	4	14
MYCBP2-AS2	0	#NUM!	0.824326	1	0	0	0	0	0
DIAPH3-AS2	#DIV/0!	#DIV/0!	0.6411933	0	0	0	0	0	2
AC008267.4	3	1.5849625	0.6677907	0	1	0	0	0	3
AC008267.3	2	1	0.4268786	4	0	3	8	2	4
AC008267.2	1	0	0.9890559	0	1	0	0	0	1
AC008267.1	#DIV/0!	#DIV/0!	0.4737294	0	0	0	0	4	0
AC008267.7	1.5	0.5849625	0.8192369	0	0	2	0	2	1
AC005722.2	0	#NUM!	0.2106783	2	2	2	0	0	0
SYMPK	0.8775325	-0.188476	0.301626	3112	3693	4202	3065	3373	3221
SCN3B	1.9285714	0.9475326	0.3010439	4	7	3	14	6	7
SCN2A	1	0	0.9176589	6	5	19	5	12	13
AC019097.1	1.5	0.5849625	0.3621526	19	6	13	12	26	19
SNORA15B-1	0	#NUM!	0.6840298	0	0	2	0	0	0
TECR	1.0874453	0.1209429	0.163242	3161	3913	4133	3973	4363	3851
HDAC1P2	0	#NUM!	0.255947	4	0	2	0	0	0
HDAC1P1	1.5	0.5849625	0.7988854	4	0	0	1	0	5
N4BP2L1	2	1	0.814195	0	1	0	0	0	2
N4BP2L2	1.1504959	0.2022558	0.1405656	1809	984	1845	1503	1960	1873

SCML2	0.806891	-0.309554	0.1584809	444	403	401	243	356	408
SCML1	0.625	-0.678072	0.126989	60	42	58	29	46	25
SCML4	1.5833333	0.662965	0.5472917	0	7	5	6	3	10
AC003075.1	1	0	0.9890559	0	1	0	0	1	0
AC073072.1	0.375	-1.415037	0.326411	6	4	6	2	4	0
SUPT3H	1.073501	0.1023235	0.3047106	515	489	547	577	552	536
AC105399.1	#DIV/0!	#DIV/0!	0.6411933	0	0	0	0	0	2
NR5A1	0.4166667	-1.263034	0.4843642	8	0	4	2	3	0
NR5A2	0.8823529	-0.180572	0.7435166	40	39	40	30	46	29
AC012441.2	0.75	-0.415037	0.8644309	0	3	1	1	1	1
ZNF780B	0.6086957	-0.716207	0.5217438	13	5	5	5	8	1
SNORA15B-2	#DIV/0!	#DIV/0!	0.1633359	0	0	0	5	2	0
ZNF780A	0.967943	-0.047006	0.9934627	440	250	433	255	458	374
TERF1P5	1.1666667	0.2223924	0.8824989	2	0	4	0	4	3
TERF1P4	0.8	-0.321928	0.9218198	4	0	1	3	1	0
AC083801.2	0	#NUM!	0.5622741	0	1	2	0	0	0
AL359711.2	0.9583333	-0.061401	0.9571032	14	5	5	4	7	12
TEFM	0.8715328	-0.198373	0.463272	215	219	251	137	236	224
YTHDF2P1	0.75	-0.415037	0.8824289	3	1	0	2	0	1
DCLRE1B	0.8656061	-0.208217	0.3051719	527	646	650	426	606	546
DCLRE1A	1.3061497	0.3853203	0.0210453	554	388	554	456	727	771
DCLRE1C	1.097622	0.1343813	0.3598358	298	187	314	236	333	308
AC026436.1	1.2608696	0.334419	0.6389764	9	5	9	8	7	14
FRRS1L	0	#NUM!	0.824326	0	0	1	0	0	0
AC068189.1	0	#NUM!	0.824326	0	1	0	0	0	0
SLC30A7	0.9807692	-0.028014	0.8979652	1122	820	1178	766	1159	1135
SLC30A6	0.9966061	-0.004905	0.8167167	1482	1166	1477	998	1620	1493
SLC30A9	1.1135669	0.1551882	0.2271441	1631	1031	1591	1191	1858	1687
SLC30A8	#DIV/0!	#DIV/0!	0.2378905	0	0	0	2	4	0
SLC30A3	2.2173913	1.1488634	0.116735	3	13	7	20	15	16
SLC30A2	1.2307692	0.2995603	0.4031531	30	39	35	38	49	41
ATG9B	0.5631579	-0.828389	0.0070663	124	105	151	48	94	72
SLC30A5	0.9446194	-0.082195	0.8642944	1625	1729	2045	1615	1794	1691
ATG9A	1.0536055	0.0753348	0.3442373	1510	1887	1845	1756	1981	1786
SLC30A4	0.8222222	-0.2824	0.7334795	7	18	20	4	22	11
LMTK3	1.1607565	0.2150654	0.0956449	352	419	498	499	468	506
LMTK2	0.8049946	-0.312949	0.0846234	1437	1532	1636	827	1439	1441
SLC30A1	1.8488698	0.8866436	5.66E-11	1753	1295	1597	2323	3208	3057
ATAD3B	1.073637	0.1025064	0.2239988	1872	2286	2537	2461	2505	2222
ATAD3A	1.0235129	0.0335292	0.4954924	1394	1745	1837	1770	1671	1652
SUPT7L	0.9219597	-0.117224	0.7196182	1537	1257	1819	1392	1503	1358
ATAD3C	1.2307692	0.2995603	0.6429502	18	7	14	8	23	17
AL357314.1	0	#NUM!	0.5781912	0	0	3	0	0	0
SCN8A	0.5671902	-0.818095	0.000159	348	305	493	203	204	243
SUPT6H	0.8403081	-0.25101	0.0957338	5769	6270	6528	4115	5652	5835
SPTA1	0.6	-0.736966	0.7458929	4	0	1	0	1	2
INHBB	#DIV/0!	#DIV/0!	0.6554062	0	0	0	0	2	0
INHBA	0.7368421	-0.440573	0.5293264	17	18	22	12	18	12
TPTEP1	1.2163212	0.2825243	0.0070033	1460	1460	1712	1520	2074	2040
SUPT5H	0.9025383	-0.14794	0.4825463	4963	6148	6578	5111	5575	5279
INHBC	2.6666667	1.4150375	0.4410159	1	0	2	3	0	5
INHBE	0.5555556	-0.847997	0.6791511	3	0	6	0	2	3
COL5A1	0.5610178	-0.833882	2.96E-09	2845	3499	3481	1722	1873	1917
COL5A3	0	#NUM!	0.6436235	2	0	0	0	0	0

COL5A2	0.9275136	-0.10856	0.728275	407	389	487	283	440	467
GTF2IRD2P1	1.7142857	0.7776076	0.6270419	4	0	3	0	5	7
SCN5A	1.2285714	0.2969817	0.7217732	12	20	3	15	21	7
AL121761.2	1.9	0.9259994	0.422731	6	0	4	16	2	1
AL121761.1	0.8167939	-0.291956	0.5605845	38	55	38	27	60	20
ATG4C	1.0901909	0.1245808	0.3192791	569	343	607	485	639	532
ATG4B	1.0616246	0.0862738	0.2175084	1741	1813	2158	1972	2138	1954
ATG4A	0.9237229	-0.114468	0.6839767	456	431	542	348	519	453
SCN4A	4.5	2.169925	0.27039	1	0	1	4	0	5
AC004884.2	#DIV/0!	#DIV/0!	0.0949296	0	0	0	8	0	1
SCN4B	3	1.5849625	0.6373113	0	1	0	1	2	0
ATG4D	0.9840256	-0.023232	0.8553462	213	195	218	211	202	203
AL121917.2	3.1666667	1.662965	0.2207776	4	0	2	2	9	8
AL121917.1	1.490566	0.5758603	0.2888951	14	8	31	14	25	40
BCL2A1	0	#NUM!	0.824326	1	0	0	0	0	0
ATAD2B	1.0124922	0.0179108	0.8082698	676	366	559	348	561	712
AC011999.1	0	#NUM!	0.5337954	0	3	0	0	0	0
PDAP1	0.9603165	-0.058418	0.9868247	4956	6021	6335	5486	5863	5276
AL451069.3	0.5	-1	0.5675482	1	5	6	2	0	4
AL451069.2	#DIV/0!	#DIV/0!	0.6411933	0	0	0	0	0	2
AC090666.1	#DIV/0!	#DIV/0!	0.8030358	0	0	0	0	0	1
TEN1	0	#NUM!	0.6436235	2	0	0	0	0	0
AL445928.1	0	#NUM!	0.824326	0	0	1	0	0	0
CLUAP1	0.8381818	-0.254665	0.3766034	185	155	210	145	168	148
RAN	0.9305097	-0.103907	0.6955438	18415	20408	22941	18433	20668	18371
LINC01990	1.8809524	0.9114633	0.1264853	17	14	11	19	42	18
RB1	0.888773	-0.170113	0.486587	1134	719	1024	556	1031	970
LINC01992	#DIV/0!	#DIV/0!	0.5070017	0	0	0	3	0	0
GABPB2	0.9295039	-0.105467	0.848444	247	199	320	166	219	327
LINC01991	0	#NUM!	0.6840298	0	0	2	0	0	0
AC026771.1	0	#NUM!	0.255947	4	0	2	0	0	0
RAX	2.1666667	1.1154772	0.1699696	8	4	6	24	10	5
GABPAP	1.2027027	0.2662801	0.6184574	21	30	23	15	50	24
GABPB1	1.0463062	0.0653051	0.3357476	1237	1055	1444	1168	1485	1256
RPS4XP8	#DIV/0!	#DIV/0!	0.6251462	0	0	0	1	0	1
RPS4XP7	0	#NUM!	0.824326	0	1	0	0	0	0
SPCS2P4	1.2679856	0.3425384	0.2674322	228	148	180	103	218	384
TEPP	1.4186047	0.5044726	0.1336406	25	42	62	55	65	63
SPCS2P2	#DIV/0!	#DIV/0!	0.5070017	0	0	0	3	0	0
LINC01988	1.75	0.8073549	0.6233491	1	6	1	0	8	6
LINC01983	0.8355705	-0.259167	0.4737791	94	93	111	80	94	75
AC009094.1	#DIV/0!	#DIV/0!	0.5509701	0	0	0	0	3	0
LINC01981	0	#NUM!	0.824326	1	0	0	0	0	0
LINC01980	1.8139535	0.8591375	0.1008138	11	10	22	29	22	27
RPS4XP3	1.8571429	0.8930848	0.3783366	11	1	2	14	6	6
RPS4XP6	1	0	0.9520787	3	0	1	3	0	1
RPS4XP5	0.375	-1.415037	0.583512	1	0	7	2	0	1
TEP1	0.8104004	-0.303293	0.1122221	764	634	775	464	602	695
RN7SL81P	0.7857143	-0.347923	0.7370175	11	5	12	0	16	6
RPS4XP1	0	#NUM!	0.2891624	2	1	2	0	0	0
CLPSL1	0	#NUM!	0.824326	1	0	0	0	0	0
CLPSL2	2	1	0.6050759	0	0	3	1	3	2
LINC01977	0.2	-2.321928	0.5267773	0	0	5	0	0	1
DLEU7-AS1	3.75	1.9068906	0.2282795	3	0	1	8	0	7

GALR3	1.75	0.8073549	0.634083	3	0	1	6	0	1
TERT	1.4331066	0.5191459	0.0120202	108	153	180	219	220	193
GALR2	0.9	-0.152003	0.9860596	2	13	5	15	1	2
BRAP	0.9256653	-0.111437	0.6879641	1058	933	1278	822	1078	1126
AC114402.2	1.625	0.7004397	0.6585852	5	0	3	0	7	6
RDX	1.1002741	0.137863	0.3186502	4446	2815	4048	2801	4943	4699
UPF3B	1.3379339	0.4200069	0.0067714	824	522	1045	1074	1178	947
BRAF	1.1060606	0.1454304	0.2309392	617	491	608	500	732	666
UPF3A	1.0115889	0.0166231	0.5582142	1098	842	1339	1061	1214	1042
IRS3P	#DIV/0!	#DIV/0!	0.3981469	0	0	0	4	0	0
AC114402.1	0.5	-1	0.5434889	7	2	5	5	2	0
RN7SL378P	0.3333333	-1.584963	0.6715904	3	0	0	0	1	0
GALNTL5	3	1.5849625	0.4633206	1	1	0	5	0	1
REL	0.8320158	-0.265317	0.7283866	175	146	185	30	142	249
AL445189.2	5.5	2.4594316	0.1614422	0	1	1	6	2	3
REN	0	#NUM!	0.3026413	2	3	0	0	0	0
LINC01999	0	#NUM!	0.824326	0	1	0	0	0	0
LINC01993	2	1	0.3972017	1	8	2	3	8	11
LINC01996	0	#NUM!	0.6385096	0	2	0	0	0	0
AC022182.3	1.125	0.169925	0.9181075	8	0	0	4	4	1
RET	0.125	-3	0.2868191	0	1	7	0	1	0
AC132192.2	0.5833333	-0.777608	0.1867601	32	21	67	24	24	22
TCF25	0.9082512	-0.138837	0.5698177	2266	2698	3047	2400	2468	2408
TCF24	1.1730769	0.2302976	0.6503129	25	8	19	21	28	12
TCF23	0	#NUM!	0.6840298	0	0	2	0	0	0
AL356968.2	1.6666667	0.7369656	0.4866821	6	1	8	2	17	6
TCF21	1	0	0.9977367	5	0	3	0	4	4
TCF20	0.8362248	-0.258037	0.1217893	2524	2423	2777	1573	2377	2509
NPTN-IT1	1.0212766	0.0303736	0.9808338	37	29	28	4	46	46
DSTYK	0.784689	-0.349807	0.1608145	237	309	290	143	251	262
AL590282.1	0.2413793	-2.050626	0.087055	10	5	14	0	5	2
LINC01949	1.6222222	0.6979715	0.2276794	17	7	21	13	25	35
RFK	1.3184611	0.398855	7.20E-05	5279	4921	5915	5736	7978	7533
LINC01943	1.6	0.6780719	0.5573393	0	0	10	14	2	0
AP001160.1	0	#NUM!	0.2232483	1	2	3	0	0	0
AP001160.4	1.75	0.8073549	0.6351387	1	0	3	2	0	5
AP001160.3	0.5833333	-0.777608	0.6292866	5	2	5	0	7	0
XBP1P1	0.3846154	-1.378512	0.4635135	6	0	7	0	5	0
LINC01941	#DIV/0!	#DIV/0!	0.8030358	0	0	0	0	0	1
BRD3	1.0975855	0.1343333	0.2135575	1454	1718	1798	1394	1936	2125
TCF19	0.8542413	-0.227284	0.2622894	490	561	623	436	510	484
BRD2	0.887754	-0.171768	0.2697153	8288	9039	10282	7217	8944	8349
BRD1	0.9018201	-0.149088	0.4610141	1707	1938	2069	1558	1734	1861
TCF15	#DIV/0!	#DIV/0!	0.3196487	0	0	0	0	1	4
TCF12	0.8506542	-0.233355	0.2827131	1471	1156	1424	705	1299	1442
TET2	1.2730853	0.3483291	0.0850332	877	526	882	555	1053	1301
TET1	1.5795091	0.6594762	5.84E-05	1313	1040	1395	1285	2161	2474
AC073957.1	0	#NUM!	0.3451105	2	0	3	0	0	0
AC073957.3	0.7525773	-0.410088	0.3213594	74	52	68	42	45	59
TET3	1.1287567	0.1747346	0.2217614	4141	4129	4374	3044	5125	6103
RGN	#DIV/0!	#DIV/0!	0.1593104	0	0	0	6	0	1
AC132192.1	0.5	-1	0.81753	2	0	0	0	0	1
DNAJC8P1	2.125	1.0874628	0.3306872	2	3	3	8	5	4
LINC01933	0	#NUM!	0.824326	1	0	0	0	0	0

RGR	0	#NUM!	0.5075476	0	0	4	0	0	0
LINC01970	1	0	0.9000607	8	4	24	8	17	11
MIR2052HG	0.6428571	-0.63743	0.7685085	2	2	10	6	0	3
RHD	1.5142857	0.5986374	0.3021433	9	12	14	22	14	17
LINC01969	3	1.5849625	0.6889342	0	1	0	0	3	0
LINC01968	0.75	-0.415037	0.7335519	13	1	6	0	14	1
BRD9	0.8221163	-0.282586	0.1389044	1465	1579	2201	1440	1504	1368
LINC01967	0.8181818	-0.289507	0.8526376	2	4	5	1	4	4
BRD8	0.9510314	-0.072435	0.9091275	3358	2856	4160	2722	3708	3436
BRD7	1.0174773	0.0249967	0.4686926	3598	3099	4117	3177	4016	3810
LINC01960	0.8378995	-0.255151	0.2104104	424	427	463	325	380	396
LINC01963	0.6736842	-0.569856	0.3447256	36	22	37	6	38	20
BRD4	0.9982574	-0.002516	0.6512361	4247	4786	5313	4150	5160	5011
LINC01962	0	#NUM!	0.5387425	3	0	0	0	0	0
BRF2	0.6935818	-0.527862	0.05681	136	150	197	95	140	100
BRF1	0.8266667	-0.274622	0.1496991	828	899	1048	764	738	792
MOCS1P1	0	#NUM!	0.6840298	0	0	2	0	0	0
AGBL5	1.0703166	0.0980376	0.1927716	2207	2557	2816	2480	2855	2778
AGBL4	1.9	0.9259994	0.4094471	6	0	4	7	6	6
AGBL1	#DIV/0!	#DIV/0!	0.092722	0	0	0	7	0	2
AGBL3	0.9863014	-0.0199	0.9332321	11	28	34	19	27	26
AGBL2	#DIV/0!	#DIV/0!	0.8030358	0	0	0	0	1	0
RNU6-469P	0	#NUM!	0.824326	0	0	1	0	0	0
LINC01952	1.25	0.3219281	0.7611895	7	0	5	4	6	5
BRDT	4.8571429	2.2801079	0.0226476	3	1	3	13	8	13
PUS10	0.9587156	-0.060825	0.9984989	223	167	264	174	223	230
PURPL	0.9082892	-0.138776	0.7147945	725	316	660	351	562	632
TEX2	1.0338266	0.0479943	0.49834	879	954	1005	794	1056	1084
CTDNEP1	1.1701299	0.2266687	0.1159935	374	583	583	567	657	578
AC027514.2	0.5	-1	0.8652929	0	0	2	0	0	1
AP000238.1	#DIV/0!	#DIV/0!	0.6554062	0	0	0	0	2	0
TEX9	1.6697248	0.7396103	0.062532	44	42	23	55	68	59
BRI3	1.1964925	0.2588114	0.0816124	2136	2913	3048	4103	3096	2489
KLC1	0.9527027	-0.069902	0.962814	684	652	884	659	815	641
RLF	1.2861858	0.363099	0.0718005	886	424	811	597	1140	991
RAD51AP1	1.0207809	0.0296732	0.6016132	1147	746	1283	869	1280	1093
KLC4	0.9292035	-0.105933	0.7585352	409	392	442	368	427	360
KLC3	0.6554622	-0.609416	0.1828831	56	102	80	61	71	24
KLC2	1.1087912	0.1489877	0.1581991	540	615	665	638	720	660
RAD51AP2	0	#NUM!	0.5436524	2	0	1	0	0	0
BTBD1	0.9391386	-0.09059	0.7807284	1137	853	1214	754	1180	1075
BTBD3	0.9051467	-0.143776	0.5632393	812	649	890	537	751	840
BTBD2	0.8781329	-0.187489	0.4748163	1236	1522	1591	1429	1256	1134
NKX6-1	1.3090909	0.3885653	0.1307686	53	74	93	105	95	88
RNU6-466P	#DIV/0!	#DIV/0!	0.6411933	0	0	0	0	0	2
AC116609.2	#DIV/0!	#DIV/0!	0.5371397	0	0	0	0	0	3
AL596218.1	#DIV/0!	#DIV/0!	0.6411933	0	0	0	0	0	2
AC116609.1	0.4285714	-1.222392	0.6908084	0	1	6	3	0	0
BTBD7	1.0433996	0.0612918	0.5744444	1363	1129	1379	876	1510	1653
BTBD6	1.0290557	0.0413211	0.4838921	712	841	925	880	887	783
BTBD9	0.7583677	-0.399031	0.0263517	613	768	800	452	589	613
BTBD8	1	0	0.9988411	1	0	1	0	2	0
BRK1	1.0535596	0.0752719	0.254942	3497	3927	4656	4244	4509	3974
AL096701.1	12	3.5849625	0.0953384	0	1	0	1	9	2

AKIRIN1P2	0	#NUM!	0.6385096	0	2	0	0	0	0
AL096701.3	0.5	-1	0.8652929	0	0	2	0	0	1
AC016949.1	1.5625	0.6438562	0.3286837	9	8	15	8	19	23
EFHD2	1.2737604	0.3490939	0.0029779	4544	5876	6299	7202	7674	6420
EFHD1	0.943899	-0.083296	0.9872642	180	225	308	260	224	189
C4orf36	1.0063694	0.00916	0.8122419	32	34	91	36	45	77
C4orf33	1.3963356	0.4816457	0.0012199	742	502	830	837	1168	891
PIGCP1	0.691358	-0.532495	0.4030404	23	32	26	3	29	24
AC068533.3	0.2857143	-1.807355	0.507281	1	0	6	0	0	2
AC092573.2	0.15	-2.736966	0.0886131	2	8	10	0	3	0
POM121L2	0.311828	-1.681178	0.0092013	38	21	34	5	8	16
DALRD3	1.0681998	0.0951816	0.369494	377	423	461	386	526	435
RP2	1.1508838	0.2027422	0.1353379	2336	1854	2146	1695	2825	2772
FAM27E3	1.3333333	0.4150375	0.7923768	0	1	5	0	5	3
EFHC1	0.995255	-0.006862	0.7486414	279	210	354	255	303	281
C4orf48	1.4279476	0.513943	0.0100323	219	220	248	406	323	252
C4orf47	1.7368421	0.7964666	0.0959983	19	20	18	41	34	24
RP9	0.8170732	-0.291463	0.2948249	237	201	300	209	208	186
C4orf46	1.1929964	0.2545897	0.029125	830	736	947	816	1034	1148
EFHC2	0.5	-1	0.8652929	0	0	2	0	1	0
C4orf45	0	#NUM!	0.4117982	0	3	1	0	0	0
GLRX3P2	0.875	-0.192645	0.9038066	4	4	0	3	3	1
AL359220.1	1.0074074	0.0106472	0.8601685	55	33	47	42	37	57
DRICH1	0.25	-2	0.5512175	2	0	2	0	1	0
AL137785.1	0.4255319	-1.232661	0.133221	10	16	21	6	9	5
RPE	1.0331889	0.047104	0.4685877	2892	2374	2809	2288	3068	2987
AMBRA1	0.7699573	-0.37715	0.0160799	996	1136	1150	732	937	858
AL162171.3	0.6511628	-0.61891	0.4660464	11	10	22	5	11	12
AL162171.2	0.7	-0.514573	0.7522332	10	0	0	0	6	1
AL162171.1	0.6923077	-0.530515	0.5908541	9	11	32	13	20	3
C4orf19	0.7836478	-0.351723	0.1234853	240	276	279	178	247	198
C4orf17	0	#NUM!	0.824326	1	0	0	0	0	0
AC067945.1	1	0	0.9890559	0	0	1	0	1	0
HRAT92	0.5087719	-0.974909	0.2189442	16	14	27	12	5	12
SALRNA3	0	#NUM!	0.824326	0	0	1	0	0	0
KLF4	1.8500682	0.8875784	9.05E-13	680	731	790	1262	1505	1305
KLF3	1.1279045	0.1736449	0.0968576	1626	1355	1710	1400	1928	1963
OXR1	1.1361702	0.184179	0.2548122	467	247	461	371	532	432
KLF2	0.7572816	-0.401098	0.3081817	53	78	75	40	67	49
KLF1	0.3333333	-1.584963	0.6659102	3	0	0	1	0	0
KLF8	0.8282525	-0.271857	0.1413372	776	735	818	476	716	737
KLF7	0.75	-0.415037	0.8797867	1	0	7	0	2	4
KLF6	0.9401741	-0.089	0.7543055	2584	2484	3089	2024	2802	2843
KLF5	0.8214369	-0.283778	0.0677137	2812	2755	3007	1762	2666	2615
AC138951.2	0	#NUM!	0.1018701	5	0	5	0	0	0
AC138951.1	0.2727273	-1.874469	0.2979924	6	4	1	0	3	0
FO393418.1	1.1333333	0.1805722	0.8552875	9	4	2	5	2	10
KLF9	1.1077086	0.1475783	0.2612907	328	282	337	289	374	386
RUNDC1	0.9941672	-0.00844	0.8778556	484	538	521	368	572	594
AC067945.3	#DIV/0!	#DIV/0!	0.2545643	0	0	0	0	4	2
OXTR	0.6133333	-0.705257	0.3181455	32	11	32	18	18	10
DIXDC1	0.820698	-0.285077	0.2494007	273	266	292	147	261	274
BBA01000935	0.1428571	-2.807355	0.2874178	0	5	2	1	0	0
RS1	0	#NUM!	0.6840298	0	0	2	0	0	0

AC008026.1	1.4	0.4854268	0.7202346	0	0	5	7	0	0
AC008026.3	0	#NUM!	0.2646708	3	0	3	0	0	0
UTS2R	0	#NUM!	0.4374838	0	4	0	0	0	0
BROX	1.0297095	0.0422374	0.5432502	1667	1165	1712	1238	1849	1592
OXSM	0.9271318	-0.109154	0.7437459	402	419	469	378	442	376
YTHDF1P1	0	#NUM!	0.3482063	0	0	6	0	0	0
UTS2B	0.25	-2	0.6138887	0	0	4	1	0	0
GBP1P1	1.3760684	0.4605522	0.1974041	24	35	58	39	61	61
AL157371.2	1.0952381	0.1312445	0.8694455	6	7	8	3	11	9
XRN1	1.1079967	0.1479536	0.4598088	514	278	421	266	511	567
XRN2	0.9365122	-0.09463	0.7188832	3700	3229	4144	2866	3764	3740
TFAM	1.1243721	0.1691195	0.1491575	2304	1773	2492	1781	2902	2703
HRASLS	1.1811024	0.240134	0.2893412	127	144	110	131	181	138
ANP32A	1.0288368	0.0410142	0.3543729	9088	9519	11320	9955	11109	9726
KLK1	#DIV/0!	#DIV/0!	0.5338029	0	0	0	0	1	2
KLK3	0	#NUM!	0.824326	0	1	0	0	0	0
KLK2	0.6363636	-0.652077	0.3243602	42	13	33	19	27	10
KLK8	1.3448276	0.4274212	0.390278	25	17	16	31	15	32
KLK7	0.9272727	-0.108934	0.9051341	19	18	18	14	23	14
KLK6	0.3043478	-1.716207	0.016756	29	17	23	3	10	8
BCL11A	0.4	-1.321928	0.6604335	1	0	4	0	0	2
PYHIN1	0.91875	-0.122256	0.818795	58	44	58	32	72	43
ANKEF1	1.0637652	0.0891797	0.4612225	332	314	342	272	405	374
ANP32E	1.1589497	0.212818	0.0579345	4307	3320	4446	3560	5258	5174
SPRTN	0.9608108	-0.057676	0.9896155	461	406	613	382	538	502
ANP32D	1.5	0.5849625	0.7729581	0	0	2	2	0	1
ANP32C	0.7352941	-0.443607	0.6906627	6	10	18	11	10	4
ANP32B	1.1787465	0.2372534	0.0127369	9521	10515	12815	13016	13774	11933
AC010285.3	0	#NUM!	0.6840298	0	0	2	0	0	0
KLLN	1.0493827	0.0695409	0.7638947	70	46	46	52	60	58
BCL2L12P1	0.75	-0.415037	0.8942358	2	0	2	0	0	3
BRS3	#DIV/0!	#DIV/0!	0.8030358	0	0	0	0	1	0
AL359710.1	1	0	0.9254253	4	5	6	7	1	7
RNU6ATAC3I	0.1111111	-3.169925	0.2432984	0	1	8	0	0	1
FFAR4	5.6666667	2.5025003	0.0929721	0	1	2	10	1	6
FFAR2	0	#NUM!	0.824326	0	1	0	0	0	0
AL358779.1	0	#NUM!	0.6436235	2	0	0	0	0	0
ISCA2P1	0.3333333	-1.584963	0.6659102	3	0	0	1	0	0
TFE3	0.6787521	-0.559043	2.83E-05	1773	1892	2265	1215	1494	1316
TFEB	0.9739833	-0.038031	0.8492663	1175	1312	1472	1349	1373	1134
TFEC	1.25	0.3219281	0.8708511	9	3	0	0	8	7
TFF3	#DIV/0!	#DIV/0!	0.6554062	0	0	0	0	2	0
TFF2	8	3	0.2294733	0	1	0	8	0	0
TFF1	4.037037	2.0132968	0.0007169	6	15	6	42	39	28
AL353588.1	0.4666667	-1.099536	0.4071693	6	5	4	2	2	3
PRNCR1	0	#NUM!	0.824326	0	1	0	0	0	0
SPRR3	0.0416667	-4.584963	0.0170752	0	19	5	0	1	0
PDCD6IPP2	3	1.5849625	0.2479932	0	1	4	11	3	1
HRAT17	1.5	0.5849625	0.8294286	1	0	1	0	0	3
RYK	0.9304279	-0.104034	0.6988449	1201	1142	1466	942	1289	1313
CTSLP8	1.3793103	0.4639471	0.4742933	7	8	14	15	18	7
CTSLP6	0.8636364	-0.211504	0.7853506	15	21	8	10	10	18
PUS7L	1.1155015	0.1576925	0.3281422	666	354	625	442	732	661
CTSLP1	0.8023256	-0.31774	0.6253435	36	20	30	25	29	15

CTSLP4	2.0806452	1.0570309	0.0216888	11	16	35	40	37	52
CTSLP2	1.2647059	0.3388019	0.3887466	32	21	49	28	64	37
CTSLP2	1.2647059	0.3388019	0.3887466	32	21	49	28	64	37
GALNT12	0.5	-1	0.7021691	0	3	1	0	2	0
GALNT11	0.9801249	-0.028962	0.8884636	556	580	625	485	583	658
GALNT13	1	0	0.9949321	0	1	1	0	1	1
GALNT16	0	#NUM!	0.1178492	2	0	8	0	0	0
GALNT15	0	#NUM!	0.6840298	0	0	2	0	0	0
GALNT18	0.2222222	-2.169925	0.0045403	28	23	21	1	10	5
GALNT17	0.3392857	-1.559427	0.046893	25	19	12	7	4	8
GALNT10	0.4987147	-1.003714	7.61E-12	1237	1436	1606	602	735	797
LINC01907	0.7884615	-0.342888	0.6187268	15	21	16	7	22	12
LINC01909	1.75	0.8073549	0.6195838	2	0	2	6	1	0
LINC01908	#DIV/0!	#DIV/0!	0.6554062	0	0	0	0	2	0
LINC01901	0.9333333	-0.099536	0.9471356	16	5	9	6	6	16
LINC01900	#DIV/0!	#DIV/0!	0.8030358	0	0	0	0	1	0
COL4A2	0.8576722	-0.221502	0.1633948	22217	27366	27331	18886	23001	24080
COL4A1	0.8987203	-0.154056	0.4128477	14518	17251	16444	10798	15506	17026
COL4A4	0	#NUM!	0.5075476	0	0	4	0	0	0
COL4A3	2.6875	1.4262648	0.1027566	1	9	6	28	7	8
RNU6ATAC8I	0	#NUM!	0.824326	0	0	1	0	0	0
COL4A6	1.1944444	0.2563398	0.6626495	7	15	14	13	19	11
AC046130.2	0.7777778	-0.36257	0.7414128	8	9	1	1	3	10
COL4A5	0.8974476	-0.1561	0.4618077	686	673	835	553	765	651
SUCNR1	2	1	0.8324799	1	0	0	0	2	0
C5AR2	#DIV/0!	#DIV/0!	0.4501902	0	0	0	0	0	4
C5AR1	2.25	1.169925	0.504704	0	2	2	4	5	0
AC078991.1	0	#NUM!	0.824326	0	1	0	0	0	0
LINC01925	0	#NUM!	0.5387425	3	0	0	0	0	0
AP002383.4	0	#NUM!	0.6385096	0	2	0	0	0	0
HIST1H4PS1	0.4	-1.321928	0.6657942	5	0	0	2	0	0
AC123912.1	0	#NUM!	0.5781912	0	0	3	0	0	0
LINC01918	#DIV/0!	#DIV/0!	0.5371397	0	0	0	0	0	3
NR4A2	0.8700265	-0.200869	0.6025246	117	96	164	87	126	115
NR4A1	2.6002304	1.3786395	2.07E-20	271	292	305	713	802	742
NR4A3	1.8617021	0.8966223	0.0304772	23	33	38	31	80	64
LINC01914	2.5	1.3219281	0.5527374	0	0	2	3	2	0
LINC01911	#DIV/0!	#DIV/0!	0.6411933	0	0	0	0	0	2
CKAP2	1.1210201	0.1648121	0.2321016	2584	1396	2647	1902	2949	2578
CKAP5	1.0133749	0.019168	0.6571129	5432	4107	5863	3744	5671	6193
CKAP4	0.8823308	-0.180608	0.3443123	1515	1846	1959	1465	1705	1524
AGAP9	0.9100775	-0.135939	0.8109161	213	148	284	199	229	159
AGAP6	0.9532539	-0.069068	0.9958839	352	227	512	260	403	377
AGAP5	0.9545455	-0.067114	0.9879789	5	5	12	5	8	8
MST1P2	2.047619	1.0339473	0.2366566	5	12	4	19	2	22
SAG	#DIV/0!	#DIV/0!	0.5509701	0	0	0	0	3	0
GTF2IRD1P1	#DIV/0!	#DIV/0!	0.242062	0	0	0	6	0	0
SLFN5	2.0909091	1.0641303	0.1621356	10	1	11	17	11	18
PPP1R14BP3	1.0592105	0.0829894	0.5327275	118	153	185	147	182	154
TFPI	1.3774105	0.4619585	0.0475579	142	78	143	175	180	145
AL133338.2	1	0	0.9890559	0	0	1	1	0	0
PPP1R14BP2	2.5	1.3219281	0.5884129	0	1	1	1	4	0
AL133338.1	1.0935673	0.1290419	0.555471	36	47	88	58	60	69
PPP1R14BP5	0	#NUM!	0.5781912	0	0	3	0	0	0

TFPT	0.8344262	-0.261144	0.4262668	165	192	253	176	149	184
TCFL5	1.0460455	0.0649456	0.3822535	551	551	744	554	692	685
HAGHL	0.8867925	-0.173332	0.8640767	23	17	13	22	14	11
SPRY4	1.0966387	0.1330882	0.2869635	281	333	338	320	371	353
SCLT1	1.2934051	0.3711742	0.0134308	511	372	603	491	719	712
AC097523.1	1.2	0.2630344	0.8111799	6	9	5	3	17	4
TFP1	0.5882353	-0.765535	0.6117651	3	7	7	5	0	5
SCD	1.1206516	0.1643378	0.0936023	30491	34277	35239	29419	40871	41783
AP000821.1	#DIV/0!	#DIV/0!	0.5371397	0	0	0	0	0	3
SPRY3	1.64	0.7136958	0.1512223	17	12	21	23	28	31
SPRY2	1.0925	0.1276333	0.4283914	209	332	259	266	291	317
SPRY1	1.6777042	0.7464884	0.0015008	138	185	130	190	275	295
PGLYRP4	4	2	0.5810589	0	0	1	0	4	0
TFRC	0.7725683	-0.372266	0.0367196	12748	9974	12449	6034	10066	11072
SCT	1.6666667	0.7369656	0.7070812	2	4	0	0	1	9
SCX	0.925	-0.112475	0.9703182	13	10	17	16	16	5
AL590096.1	0.8636364	-0.211504	0.8891433	4	6	12	5	10	4
MIR3936	0.5263158	-0.925999	0.5227607	9	0	10	3	3	4
AP000437.1	1.6923077	0.7589919	0.4873563	5	2	6	2	15	5
PGLYRP1	0.5454545	-0.874469	0.6185461	7	0	4	0	2	4
TFR2	1.75	0.8073549	0.6260417	1	0	3	4	3	0
RRS1-AS1	10	3.3219281	0.1342958	0	1	0	2	7	1
SF1	0.8541868	-0.227377	0.1215509	8455	9625	10964	7463	9020	8326
AC011498.6	0.5	-1	0.7297487	4	0	0	0	0	2
AC011498.7	0.7849462	-0.349334	0.5527528	30	34	29	28	24	21
SET	1.1460595	0.196682	0.0117542	36325	34068	41979	37644	47376	43765
AC011498.1	#DIV/0!	#DIV/0!	0.5371397	0	0	0	0	0	3
AC011498.4	1.75	0.8073549	0.6821345	0	0	4	0	5	2
AC011498.3	#DIV/0!	#DIV/0!	0.6554062	0	0	0	0	2	0
MIR3909	0	#NUM!	0.0204758	7	9	0	0	0	0
ADAMTS12	0	#NUM!	0.2773534	1	3	1	0	0	0
ADAMTS10	0	#NUM!	0.824326	0	0	1	0	0	0
ADAMTS16	0.46875	-1.093109	0.2562376	9	9	14	2	6	7
ADAMTS15	0.8666667	-0.206451	0.8939527	2	7	6	4	6	3
ADAMTS14	0	#NUM!	0.0279248	3	7	3	0	0	0
ADAMTS13	1.8135593	0.8588239	0.0705968	24	17	18	30	31	46
ADAMTS18	0.9705882	-0.043069	0.9548132	13	14	7	7	16	10
ADAMTS17	0.8128079	-0.299014	0.4934957	57	68	78	54	71	40
SFN	0.7379923	-0.438322	0.0240569	2380	3052	3354	2403	2278	1803
KRTAP16-1	1.2	0.2630344	0.8107806	0	0	5	3	2	1
CCND3P1	3	1.5849625	0.4876533	0	0	2	1	0	5
AP000281.1	#DIV/0!	#DIV/0!	0.6099294	0	0	0	2	0	0
AL031281.2	1.4	0.4854268	0.8012032	5	0	0	3	0	4
SHB	0.9536424	-0.06848	0.954656	48	58	45	49	51	44
AL031281.1	1	0	0.9920044	1	0	2	0	3	0
SHD	1.7570093	0.8131219	0.020253	34	28	45	73	61	54
SHF	1.741573	0.800391	0.036453	22	31	36	44	51	60
AC137932.3	1.0943396	0.1300605	0.617859	31	30	45	53	22	41
SHE	0	#NUM!	0.6631647	1	0	1	0	0	0
AC137932.2	1.4285714	0.5145732	0.8211718	0	5	2	0	10	0
MMP25	0.4	-1.321928	0.5848116	2	2	1	0	0	2
SHH	0.6060606	-0.722466	0.2865745	31	13	22	12	18	10
AC137932.1	0.5555556	-0.847997	0.3188232	14	28	39	0	12	33
MMP24	0.25	-2	0.0032874	23	23	34	5	7	8

MMP28	0	#NUM!	0.4374838	0	4	0	0	0	0
AC093214.1	0.25	-2	0.4007591	0	8	0	0	2	0
AL583810.1	0.7727273	-0.371969	0.7734509	7	4	11	3	14	0
MMP12	0	#NUM!	0.824326	1	0	0	0	0	0
MMP11	0.4466667	-1.16273	0.0145821	38	49	63	13	31	23
MMP14	0.9858121	-0.020615	0.7825549	34534	42077	42998	35527	41470	40915
AL583810.2	1.5	0.5849625	0.7207794	2	0	2	2	1	3
MMP16	#DIV/0!	#DIV/0!	0.8030358	0	0	0	0	1	0
MMP15	2.4372215	1.2852374	7.51E-23	1104	1292	1419	3189	3254	2855
MMP17	0.8181818	-0.289507	0.8694653	6	0	5	0	4	5
MMP19	0.9056326	-0.143002	0.5115147	1432	1605	1721	1393	1546	1370
HHIP-AS1	1.2307692	0.2995603	0.6948807	2	1	10	11	0	5
AC103834.1	0	#NUM!	0.6840298	0	0	2	0	0	0
NBPF25P	1.0178571	0.0255351	0.7946215	49	10	53	50	32	32
UBE2D3P1	2.9545455	1.5629362	0.0171051	9	6	7	25	20	20
AC096586.2	1.5555556	0.6374299	0.3627421	10	11	6	19	13	10
RICTOR	1.0699413	0.0975316	0.5001354	746	387	740	454	744	806
AL391427.1	4.5	2.169925	0.3575376	1	1	0	0	0	9
AL137002.1	0	#NUM!	0.824326	0	0	1	0	0	0
BBOX1	2.3802817	1.2511323	3.43E-05	36	53	53	96	138	104
ZNF736P9Y	1.7777778	0.830075	0.6847216	5	4	0	0	16	0
AL137002.2	1.0769231	0.1069152	0.8896168	5	15	19	0	16	26
MIR3939	1	0	0.9656876	3	0	1	2	0	2
SKI	1.1330749	0.1802433	0.0765468	1073	1309	1488	1270	1536	1579
MIR3944	0	#NUM!	0.824326	1	0	0	0	0	0
MIR3942	2	1	0.7049414	0	0	2	0	0	4
AC004988.1	#DIV/0!	#DIV/0!	0.5371397	0	0	0	0	0	3
PDCD7	0.9537496	-0.068318	0.915938	972	948	1107	855	1020	1012
PDCD6	0.8929674	-0.163321	0.4482246	1652	1986	2192	1672	1982	1552
PDCD5	1.0588067	0.0824392	0.2997527	1027	966	1493	1346	1238	1107
SLA	1.5072464	0.5919153	0.2509302	7	22	40	20	48	36
PLS3-AS1	1.0076923	0.0110552	0.8651126	37	33	60	26	41	64
SLK	1.3441358	0.4266789	0.0264286	1041	491	1060	834	1422	1228
SLN	2.8380952	1.504923	4.05E-05	26	33	46	108	132	58
NFATC2IP	0.9457241	-0.080509	0.8783739	2376	2496	3290	2287	2843	2589
AC139256.1	2.8	1.4854268	0.3087749	2	1	2	1	8	5
IGHVIII-38-1	0	#NUM!	0.4374838	0	4	0	0	0	0
NDUFA6-AS1	0.7125	-0.489038	0.5249731	22	13	45	16	13	28
SMO	2.0689655	1.0489096	0.0922473	12	7	10	20	20	20
SMS	1.0539163	0.0757603	0.22867	3151	3273	3777	3341	3911	3499
SNN	0.7704312	-0.376262	0.0240632	711	818	906	548	718	610
NDOR1	0.9268987	-0.109516	0.7165672	944	1094	1122	962	1006	961
ZKSCAN7	#DIV/0!	#DIV/0!	0.4737294	0	0	0	0	4	0
ZKSCAN8	0.8465415	-0.240347	0.3244024	767	570	846	400	646	802
ZKSCAN3	0.7959184	-0.329308	0.171715	250	247	336	182	223	258
ZKSCAN2	0.9041096	-0.14543	0.5909771	354	351	390	264	333	393
ZKSCAN5	0.8783722	-0.187096	0.4033509	785	569	833	490	760	671
ZKSCAN4	0.8864143	-0.173947	0.4885641	293	300	305	228	320	248
DLG1-AS1	0.3809524	-1.392317	0.3469816	2	8	11	6	2	0
ZKSCAN1	0.8794003	-0.185408	0.3569731	1101	887	1080	662	1013	1023
AC108860.2	0.9789474	-0.030697	0.9110907	24	28	43	34	34	25
LRCH2	3.25	1.7004397	0.2482269	2	1	1	7	5	1
LRCH1	0.9439181	-0.083266	0.8124578	1380	1127	1398	994	1272	1420
SON	0.9790685	-0.030518	0.9633769	5392	4817	5700	3501	5723	6352

SP2	0.8864511	-0.173887	0.3819214	883	800	915	706	802	795
SP1	0.8660674	-0.207449	0.237334	5111	4798	5442	3223	4753	5319
SP4	1.0968208	0.1333278	0.4481929	227	229	236	182	230	347
SP3	1.1796531	0.2383627	0.0803748	2905	2011	2810	2164	3583	3367
SP6	1.5555687	0.6374421	4.25E-09	4886	5704	6272	8328	9578	8324
SP5	0.7073171	-0.499571	0.6429019	8	22	11	19	3	7
SP8	1.3629032	0.4466831	0.1989305	43	44	37	46	59	64
SP7	0	#NUM!	0.6436235	2	0	0	0	0	0
LRCH4	1.023845	0.0339973	0.6987365	194	243	234	215	279	193
LRCH3	0.8807882	-0.183133	0.3630959	671	617	742	462	676	650
SP9	0	#NUM!	0.6385096	0	2	0	0	0	0
AKAP8P1	0	#NUM!	0.824326	0	1	0	0	0	0
SPN	1.0416667	0.0588937	0.8050186	19	24	29	28	30	17
SPR	0.7440115	-0.426603	0.0279261	913	1240	1312	904	883	791
PRADC1	1.1772152	0.2353781	0.067106	438	504	559	608	629	530
SPDYE2	0.7536232	-0.408085	0.5821121	18	28	23	3	12	37
SPDYE1	0.8	-0.321928	0.7648137	9	10	21	12	11	9
SPX	#DIV/0!	#DIV/0!	0.6411933	0	0	0	0	0	2
AC010273.1	0.7142857	-0.485427	0.7750572	6	1	0	0	3	2
FAUP1	0.5555556	-0.847997	0.5580232	3	6	9	5	5	0
ADAMTS20	1.2307692	0.2995603	0.8043594	2	8	3	3	9	4
AL121723.1	5	2.3219281	0.4886016	0	0	1	0	5	0
GAPDH	0.8246655	-0.278119	0.0678624	81066	94090	100825	74636	81677	71279
SRC	1.0617874	0.086495	0.2480674	2311	2809	3037	2754	3091	2816
SRF	0.8976646	-0.155752	0.4820119	1733	2055	2378	1767	1863	1905
SRI	0.9836368	-0.023802	0.8056454	1127	1337	1325	1267	1262	1198
SRM	0.9213974	-0.118105	0.7116845	2480	2948	3045	2772	2628	2407
SRR	0.7236842	-0.466568	0.0550601	192	218	274	157	171	167
KRIT1	1.027959	0.0397827	0.5841172	773	481	892	572	789	845
BOLA3P4	#DIV/0!	#DIV/0!	0.8030358	0	0	0	0	0	1
BOLA3P3	0	#NUM!	0.5075476	0	0	4	0	0	0
SSB	1.2453339	0.3165326	0.015543	4865	3140	5711	5222	6730	5129
BOLA3P2	0.3333333	-1.584963	0.5779416	1	0	5	0	0	2
SPDYE4	0.5757576	-0.796467	0.4197183	16	10	7	0	16	3
SPDYE3	0.8028169	-0.316857	0.4643517	71	48	94	38	72	61
SPDYE6	0.8511905	-0.232446	0.7067411	55	40	73	48	35	60
SPDYE5	0.5205479	-0.941897	0.1461341	25	24	24	9	9	20
MRVI1-AS1	0.75	-0.415037	0.8790046	2	0	2	0	1	2
NECAP2	1.1120348	0.1532019	0.1086329	1664	1809	1820	1831	2167	1888
NECAP1	0.8933162	-0.162757	0.44029	713	753	868	553	874	658
ST5	0.7698259	-0.377396	0.1732583	159	190	168	87	156	155
IGF2BP2-AS1	0.6666667	-0.584963	0.8018743	4	0	2	2	1	1
ST7	0.9859846	-0.020363	0.7753508	418	464	545	479	481	447
PDCD4	1.6264032	0.701685	7.48E-07	790	573	864	965	1388	1269
PDCD2	0.8135939	-0.297619	0.0784352	1611	1921	2353	1501	1759	1528
AC092375.1	#DIV/0!	#DIV/0!	0.8030358	0	0	0	0	1	0
STS	1.0525642	0.0739083	0.4222149	6573	5368	6817	4688	7501	7555
QRSL1P2	0	#NUM!	0.6840298	0	0	2	0	0	0
TGFA	0	#NUM!	0.2084189	0	3	4	0	0	0
TGDS	0.9456647	-0.080599	0.8602307	616	466	648	454	625	557
AP000770.1	0.2857143	-1.807355	0.4731236	0	3	4	0	0	2
BST2	3.5517896	1.8285461	1.38E-45	1050	1219	1419	4435	4664	4000
BST1	1	0	0.9988411	1	0	1	0	2	0
AL035252.3	0.6617375	-0.595669	0.0307007	194	181	166	121	142	95

AL035252.2	#DIV/0!	#DIV/0!	0.3831122	0	0	0	0	5	0
AC006020.1	1.6666667	0.7369656	0.7660827	0	0	3	0	5	0
AC239803.3	6.3333333	2.662965	0.0542466	1	1	1	9	5	5
SYK	#DIV/0!	#DIV/0!	0.6554062	0	0	0	0	2	0
GAB1	1.3387819	0.420921	0.0078372	1566	1173	1727	1285	2335	2359
GAB3	0	#NUM!	0.2349421	4	2	0	0	0	0
GAB2	1.0011211	0.0016165	0.7405108	3847	4232	4409	3199	4521	4782
SYP	2.2758621	1.1864131	0.0694231	11	9	9	13	33	20
CNKSR2	#DIV/0!	#DIV/0!	0.5371397	0	0	0	0	0	3
CNKSR1	0.9412335	-0.087375	0.888495	986	928	1183	993	1013	909
CNKSR3	0.5521978	-0.856743	0.0005023	205	286	237	121	134	147
MICU3	0.8240343	-0.279224	0.6087069	90	40	103	54	48	90
TLCD2	1.1452991	0.1957245	0.5399522	28	23	66	32	59	43
MICU2	1.1228916	0.1672186	0.1754982	1453	899	1383	1225	1620	1349
TLCD1	0.7900447	-0.339994	0.1229059	452	582	533	432	441	365
MICU1	1.1282807	0.174126	0.0560042	1164	1169	1401	1231	1554	1428
AC104819.3	#DIV/0!	#DIV/0!	0.8030358	0	0	0	0	1	0
AC104819.1	0.375	-1.415037	0.5241252	2	1	5	0	0	3
SH3BGRL	1.0731089	0.1017964	0.3179718	1626	1157	1553	1240	1768	1645
ZFP14	1.0680473	0.0949756	0.6308095	115	72	151	71	148	142
AP001025.3	1	0	0.9904495	3	0	0	0	0	3
AC005920.3	1	0	0.9890559	1	0	0	0	0	1
AC005920.1	0	#NUM!	0.6436235	2	0	0	0	0	0
GAD1	0.0895522	-3.481127	0.0001512	30	18	19	1	3	2
GAD2	0.875	-0.192645	0.8720007	2	6	0	0	4	3
AL513412.1	2	1	0.7782384	0	0	1	2	0	0
PDCL3	1.1511691	0.2030998	0.0752243	582	507	750	644	811	662
PDCL2	#DIV/0!	#DIV/0!	0.4909737	0	0	0	2	0	1
CLYBL-AS2	0	#NUM!	0.824326	0	0	1	0	0	0
CLYBL-AS1	0	#NUM!	0.6436235	2	0	0	0	0	0
ZFP28	0.6898148	-0.535719	0.0728227	149	118	165	103	91	104
OR1C1	1.5	0.5849625	0.8175361	2	0	0	3	0	0
DENND5B	0.9326425	-0.100604	0.8962531	117	112	157	108	112	140
AC015563.1	0.3333333	-1.584963	0.7307796	0	0	3	0	1	0
TGM1	0.9718129	-0.041249	0.9126673	514	482	565	487	551	479
DENND5A	0.9458695	-0.080287	0.7569176	3173	3548	3532	2595	3674	3429
TGM7	0	#NUM!	0.355562	5	0	0	0	0	0
TGM5	3	1.5849625	0.4520918	2	1	0	0	6	3
TGM4	0.6666667	-0.584963	0.7635984	0	6	0	0	2	2
TGM3	#DIV/0!	#DIV/0!	0.5509701	0	0	0	0	3	0
TGM2	0.8017751	-0.31873	0.179491	291	356	367	274	257	282
DENND6B	1.2	0.2630344	0.6252315	5	15	15	31	5	6
BBOF1	1.1497326	0.2012984	0.4045232	62	55	70	72	69	74
DENND6A	0.8042516	-0.314281	0.1391196	359	369	401	216	366	326
DENND4B	0.7337542	-0.446631	0.0047515	837	801	1055	578	687	711
DENND4A	0.9970082	-0.004323	0.872644	533	304	500	279	502	552
DENND4C	1.1907577	0.2518798	0.4728046	1500	899	1323	765	1731	1936
AC015813.6	0.6698586	-0.578071	0.002356	754	685	754	324	589	556
TAT	0.6666667	-0.584963	0.8308055	3	0	0	0	2	0
TAZ	1.0419374	0.0592686	0.4895864	452	597	644	658	625	481
AC015813.3	0	#NUM!	0.3482063	0	0	6	0	0	0
AC015813.5	0.5546875	-0.850253	0.1199658	26	54	48	8	39	24
AL596223.1	0.3333333	-1.584963	0.6587914	2	0	1	1	0	0
AC015813.4	#DIV/0!	#DIV/0!	0.8030358	0	0	0	0	1	0

KHDC1P1	#DIV/0!	#DIV/0!	0.3981469	0	0	0	4	0	0
SFTA1P	0.875	-0.192645	0.8385283	9	5	2	1	10	3
AC015813.1	0.5384615	-0.893085	0.0245681	175	59	195	49	109	73
TBP	0.883973	-0.177926	0.3298533	1052	1083	1278	830	1100	1087
OR1F1	1.05	0.0703893	0.8234577	12	13	15	17	7	18
AC136759.1	0.4285714	-1.222392	0.6110829	3	4	0	3	0	0
MRPS30-DT	1.6875	0.7548875	0.3327971	17	6	9	6	33	15
GALT	1.3898459	0.4749249	0.0023891	331	327	445	537	571	425
OR1E2	1	0	0.9985363	2	2	1	0	0	5
OR1E1	1.1153846	0.1575413	0.783348	5	6	15	5	8	16
P4HA2-AS1	0.75	-0.415037	0.8915585	1	0	3	0	3	0
TROVE2	1.0436985	0.0617051	0.5747898	625	384	570	413	615	620
IGLV3-7	0	#NUM!	0.824326	0	1	0	0	0	0
VN1R107P	0.4545455	-1.137504	0.630476	0	1	10	0	0	5
TDG	0.942161	-0.085954	0.7747721	1659	1312	1749	1022	1757	1668
TGS1	0.8454631	-0.242186	0.2608977	794	547	775	461	649	679
MVB12B	0.0980392	-3.350497	0.0015118	10	19	22	3	1	1
MVB12A	1.0528937	0.0743598	0.3440548	1215	1350	1651	1697	1439	1303
SLIT1	1.3333333	0.4150375	0.8390427	0	0	3	0	0	4
TDH	0.656	-0.608232	0.3154764	52	23	50	36	16	30
AL355355.2	0.6666667	-0.584963	0.8292596	1	2	0	0	0	2
SLIT3	2.6666667	1.4150375	0.5124394	0	0	3	0	7	1
SLIT2	0.7589744	-0.397877	0.1814242	125	133	132	94	115	87
RAB5CP2	0	#NUM!	0.824326	1	0	0	0	0	0
RAB5CP1	#DIV/0!	#DIV/0!	0.8030358	0	0	0	1	0	0
AC005670.1	0.1666667	-2.584963	0.2405537	8	0	4	2	0	0
GALE	0.8625781	-0.213273	0.3963376	930	1212	1380	1099	1022	917
TEC	1.1958763	0.2580682	0.4848926	45	17	35	42	37	37
VN1R108P	1	0	0.9679397	2	4	6	3	8	1
TEF	0.8502387	-0.23406	0.3162873	467	547	662	479	523	423
AC103739.2	1.5454545	0.6280312	0.5904845	5	0	6	2	4	11
AC103739.1	0.6545455	-0.611435	0.5019484	25	3	27	4	11	21
GALM	0.8419983	-0.248111	0.1612053	1593	2055	2257	1494	1740	1738
SLIRP	1.1057916	0.1450796	0.1188637	1662	1747	2168	2153	2181	1833
TEK	1.2395833	0.3098553	0.4154768	27	36	33	31	38	50
TES	0.9001176	-0.151815	0.3671175	3260	3146	3796	2541	3455	3187
AL139400.1	0	#NUM!	0.4460243	4	0	0	0	0	0
AC079193.2	#DIV/0!	#DIV/0!	0.5509701	0	0	0	0	3	0
FAR2P2	1.2727273	0.3479233	0.6290726	1	3	18	7	4	17
FAR2P1	1.0353288	0.0500889	0.5540995	367	208	444	333	341	381
FAR2P4	0.8373206	-0.256148	0.6148363	162	58	198	105	114	131
TFG	0.9427719	-0.085019	0.8044623	4567	5088	5757	4409	5126	4995
GAMT	0.935851	-0.095649	0.8462256	553	678	702	635	668	506
DNAAF3	0.6206897	-0.688056	0.4962196	2	18	9	4	8	6
DNAAF2	1.0765588	0.1064271	0.2494467	842	793	899	765	1020	943
DNAAF1	0.952381	-0.070389	0.9609083	7	4	10	1	13	6
DNAAF5	0.9287692	-0.106608	0.659033	1182	1339	1452	1046	1384	1260
DNAAF4	0.3	-1.736966	0.4032843	0	6	4	2	0	1
GANC	0.8276299	-0.272942	0.2779956	237	250	302	152	242	259
AC018695.4	0.6046512	-0.725825	0.2842513	22	19	45	5	20	27
AC018695.3	0.8333333	-0.263034	0.9061312	3	0	3	0	3	2
AC018695.2	1	0	0.971258	1	0	2	0	0	3
AC022336.3	0	#NUM!	0.1074699	3	6	0	0	0	0
AC022336.1	4.5	2.169925	0.3775978	0	0	2	0	9	0

AC018695.6	1.5	0.5849625	0.8186252	2	0	0	2	0	1
AL158834.2	0.7	-0.514573	0.7750962	3	3	4	1	0	6
AC087521.2	0.8571429	-0.222392	0.8526126	4	8	2	0	2	10
AC087521.3	0	#NUM!	0.824326	1	0	0	0	0	0
AC005180.2	0	#NUM!	0.3414317	0	2	3	0	0	0
AC005180.1	1	0	0.9502571	0	0	5	0	2	3
GAR1	1.1133905	0.1549597	0.1086956	801	810	1070	918	1133	934
FAM120AOS	0.9385774	-0.091452	0.7373515	2068	2242	2593	1807	2405	2267
BTF3	1.0501289	0.0705664	0.2428203	22663	22442	27847	25388	27912	23309
AC002540.1	0	#NUM!	0.5387425	3	0	0	0	0	0
AC084880.4	0.3333333	-1.584963	0.2069671	8	5	8	2	1	4
AC084880.3	1	0	0.9783687	2	5	1	4	2	2
AC084880.1	1.8942308	0.9216121	0.0094875	26	40	38	69	65	63
AC005336.2	0	#NUM!	0.824326	0	0	1	0	0	0
OR4C14P	#DIV/0!	#DIV/0!	0.8030358	0	0	0	0	0	1
GAPT	0	#NUM!	0.824326	1	0	0	0	0	0
BTG4	0.5	-1	0.6161591	0	4	4	0	3	1
DENND1C	0.9001314	-0.151792	0.6517632	190	258	313	188	265	232
KIF3AP1	0.28	-1.836501	0.1088434	12	7	6	2	3	2
BTG3	1.0085878	0.0123367	0.557007	1013	858	1273	1045	1128	998
DENND1B	0.8008048	-0.320477	0.4193751	251	86	160	78	126	194
BTG2	0.7640803	-0.388204	0.0023026	8377	9474	10451	6366	7900	7359
BTG1	1.2640515	0.3380553	0.0011822	1631	1679	1814	2019	2378	2080
DENND1A	1.2859529	0.3628378	0.0013371	1681	1873	1835	2167	2348	2415
AC060780.2	#DIV/0!	#DIV/0!	0.1884392	0	0	0	0	0	8
AC060780.1	0.9594814	-0.059673	0.9420797	209	127	281	167	188	237
AC060780.3	1.1666667	0.2223924	0.8527693	3	0	3	2	1	4
ADAMTSL1	0.5	-1	0.7417349	1	0	3	0	2	0
AL132838.2	0	#NUM!	0.1144598	4	3	1	0	0	0
ADAMTSL5	1.4145078	0.5003001	0.0861722	76	38	79	99	83	91
ADAMTSL4	0.9073293	-0.140302	0.4666532	4771	5357	6231	4507	5269	5067
ADAMTSL3	0.6	-0.736966	0.620968	2	6	2	1	4	1
AL132838.1	0.5	-1	0.8289598	1	0	1	0	1	0
ADAMTSL2	#DIV/0!	#DIV/0!	0.3927105	0	0	0	1	0	3
GAST	3.0842105	1.6249012	1.51E-05	27	21	47	136	79	78
TK2	0.7196653	-0.474602	0.2814462	83	72	84	64	81	27
TK1	0.9974612	-0.003667	0.6538197	2865	3563	3813	3704	3388	3123
DENND2D	2.4675325	1.3030691	1.88E-05	58	39	57	84	144	152
DENND2C	0.8901099	-0.167945	0.7583852	40	27	24	15	37	29
DENND2A	0.703466	-0.507447	0.0105683	461	511	586	387	356	353
AL162713.1	2	1	0.834705	0	0	1	0	2	0
COPZ1	0.9637818	-0.053222	0.9944268	3874	4749	4630	4103	4559	4111
GAS2	2	1	0.4108695	4	6	1	2	12	8
GAS1	0.9235154	-0.114792	0.6946291	596	736	773	582	666	696
GARS	1.1498859	0.2014907	0.0143897	6285	5897	7533	6748	8527	7395
SINHCAF	0.963724	-0.053308	0.9082301	5652	5655	6115	3988	6298	6504
GAS6	1.0963855	0.1327552	0.2520614	616	736	889	969	824	664
LNX1-AS1	0	#NUM!	0.824326	0	1	0	0	0	0
TKT	0.779567	-0.359255	0.0169826	19752	23330	25973	18010	18772	17051
GART	0.9236703	-0.11455	0.5872403	5144	5617	6480	4373	5720	5832
GAS5	1.1232247	0.1676466	0.0505647	3744	3824	4613	4515	4929	4238
GAS8	1.1616052	0.2161198	0.1500487	275	314	333	303	338	430
GAS7	0.5074074	-0.978784	0.0076092	72	100	98	26	58	53
LNX1-AS2	0.8571429	-0.222392	0.8790244	7	2	5	1	3	8

AC004987.3	0.5	-1	0.7173905	2	0	4	2	0	1
AC004987.2	0.8993289	-0.153079	0.6619128	122	178	147	118	155	129
AC004987.1	#DIV/0!	#DIV/0!	0.6554062	0	0	0	0	2	0
AC007485.2	0.8237288	-0.279759	0.4158221	111	86	98	69	90	84
AC116366.2	0	#NUM!	0.6840298	0	0	2	0	0	0
AC116366.1	1	0	0.8695108	9	7	32	11	5	32
AL160162.1	1.1504425	0.2021889	0.3846975	47	78	101	89	82	89
CEP295NL	0.9361702	-0.095157	0.9621504	19	32	43	25	29	34
AC004987.4	0	#NUM!	0.6840298	0	0	2	0	0	0
AC133473.1	0.3333333	-1.584963	0.6646052	2	0	1	0	1	0
AL031280.1	#DIV/0!	#DIV/0!	0.1541435	0	0	0	0	5	3
AP003174.3	2	1	0.6300114	1	2	0	6	0	0
AC079203.1	2	1	0.6905599	0	2	0	3	0	1
AP003174.1	0	#NUM!	0.4005425	3	1	0	0	0	0
AC087761.1	1	0	0.9602092	4	0	0	4	0	0
IL10RB	0.7236328	-0.46667	0.0394482	275	403	346	221	261	259
IL10RA	0.4736842	-1.078003	0.3705986	6	12	1	1	5	3
GATC	0.7504899	-0.414095	0.0244059	480	534	517	343	419	387
GATB	1.0671581	0.0937739	0.3537684	674	913	855	789	934	883
GATM	0.9054054	-0.143364	0.933677	25	13	36	26	25	16
GATS	0.8932292	-0.162898	0.4998864	387	398	367	257	372	400
COQ8B	1.1226158	0.1668643	0.2385215	273	402	426	438	438	360
COQ8A	1	0	0.651561	1582	1925	2044	1848	1954	1749
AL021391.1	0	#NUM!	0.4005425	3	1	0	0	0	0
TNC	1.6	0.6780719	0.3786669	2	8	10	15	8	9
TNF	0.64	-0.643856	0.5315285	17	4	4	4	7	5
TNN	4.5	2.169925	0.2844998	2	0	0	4	4	1
TNR	2	1	0.753159	0	2	0	0	0	4
TCF3P1	10	3.3219281	0.1737028	0	0	1	0	8	2
AC091390.1	1.8461538	0.8845228	0.3538062	4	5	4	9	3	12
AC091390.3	1.1	0.1375035	0.9189523	8	2	0	4	2	5
AL031186.1	0.754386	-0.406625	0.5762551	12	31	14	15	20	8
AC091390.4	0.5	-1	0.6347853	4	2	0	1	2	0
TOX	0.652968	-0.614916	0.0989608	62	77	80	45	50	48
INSIG2	1.297379	0.3756	0.0026371	683	577	724	713	967	894
INSIG1	1.1351953	0.1829405	0.0513966	1541	1627	1773	1611	2015	1983
AP000926.4	0	#NUM!	0.824326	0	1	0	0	0	0
TPO	0.6808511	-0.554589	0.2174744	41	51	49	28	39	29
ESPNL	0.6	-0.736966	0.7699772	0	5	0	2	1	0
ESPNP	#DIV/0!	#DIV/0!	0.0721209	0	0	0	5	2	2
TPR	1.2651126	0.3392657	0.0268676	3310	1836	3605	2922	4496	3653
AL136162.1	1.3070175	0.3862785	0.2844431	27	43	44	40	64	45
BTLA	0	#NUM!	0.824326	0	0	1	0	0	0
AC005086.1	2	1	0.6540525	0	0	2	4	0	0
SYNJ2	0.8469663	-0.239623	0.1981277	1426	1262	1383	812	1284	1352
COPRS	0.9539749	-0.067977	0.9755533	683	772	935	824	797	659
COPS4	1.2916341	0.3691974	0.0006115	1302	1121	1414	1483	1850	1623
COPS3	0.9733067	-0.039034	0.8453499	2652	2591	3261	2586	3048	2643
COPS6	0.8244824	-0.278439	0.1065714	2701	3151	3469	2613	2701	2371
COPS5	0.9019608	-0.148863	0.476948	1664	1421	1964	1370	1696	1488
SYNJ1	1.1781464	0.2365188	0.1391131	1036	820	917	727	1196	1344
COPS2	1.2257651	0.2936825	0.0601676	2149	1123	2185	1764	2605	2320
AL603832.1	0.625	-0.678072	0.4756374	9	9	14	3	6	11
COPS8	1.0757185	0.1053005	0.1512106	1805	1837	2169	1924	2233	2094

COPS9	1.0876133	0.1211657	0.3186088	407	428	489	579	468	393
SYNE4	0.6923077	-0.530515	0.4910455	12	17	36	15	18	12
SYNE3	0.6025337	-0.730886	5.83E-05	1551	1751	1750	672	1092	1280
SYNE2	1.144727	0.1950036	0.1662404	5927	3814	6303	4102	7083	7181
SYNE1	0.8141026	-0.296718	0.1913689	533	349	522	347	415	381
WDFY3-AS2	1.877551	0.9088521	0.0970049	8	11	30	28	45	19
TRO	3.1111111	1.6374299	0.124061	1	1	7	16	10	2
THBD	0.84375	-0.245112	0.7619956	17	20	27	16	15	23
AC005086.4	0	#NUM!	0.824326	1	0	0	0	0	0
AC105250.1	0.3333333	-1.584963	0.5317512	0	3	3	1	0	1
BOLA2P2	0.1176471	-3.087463	0.0295541	4	5	25	0	4	0
SECISBP2L	0.9609484	-0.057469	0.9281949	1420	890	1275	758	1206	1481
AC010524.1	0.4285714	-1.222392	0.5821056	5	0	2	0	3	0
TSN	1.027057	0.0385163	0.4151946	2363	2200	2644	2146	2731	2525
SUCLG2P2	2.8571429	1.5145732	0.2079316	2	0	5	8	2	10
ESPL1	0.9385848	-0.091441	0.7548048	1284	1571	1639	1158	1518	1542
TST	0.9303944	-0.104086	0.9520938	235	274	353	330	232	240
AL356432.2	#DIV/0!	#DIV/0!	0.6411933	0	0	0	0	0	2
AL513318.2	1.3133803	0.3932847	0.097452	105	83	96	128	138	107
AL513318.1	1	0	0.9890559	0	1	0	0	1	0
TTK	1.1551418	0.20807	0.1594802	1803	942	1767	1375	1988	1849
TTL	0.9667129	-0.048841	0.9783385	735	645	783	528	784	779
TTN	1.2004802	0.2636116	0.2165016	319	197	317	201	345	454
45214|sarcome	#DIV/0!	#DIV/0!							
binding;GO:0(	#VALUE!	#VALUE!	7|titin [EC:2.	ιy (HCM)|Dil.	titin				
TTR	1.2	0.2630344	0.8658107	5	0	0	6	0	0
ALOXE3	0.26	-1.943416	0.0219621	18	21	11	5	5	3
TUB	0.6420526	-0.639237	0.0008345	513	516	569	236	407	383
AL353689.1	#DIV/0!	#DIV/0!	0.6099294	0	0	0	2	0	0
AC092135.1	1	0	0.9832201	0	3	0	0	0	3
AC078852.2	0.0833333	-3.584963	0.1306295	4	0	8	0	0	1
AC078852.1	0.6	-0.736966	0.7612956	0	4	1	0	0	3
AC092135.3	2.5	1.3219281	0.4033988	2	1	1	7	0	3
AC092135.2	0	#NUM!	0.6436235	2	0	0	0	0	0
TCERG1	0.9808792	-0.027853	0.8738595	6071	4504	6736	4259	6524	6197
AL157996.1	0	#NUM!	0.824326	0	1	0	0	0	0
C12orf57	1.77	0.8237494	0.0326871	29	37	34	78	41	58
C12orf56	1.0030303	0.0043652	0.7966902	95	112	123	112	115	104
AL133523.1	2.2	1.1375035	0.4550179	2	0	3	5	0	6
PITPNB	0.953053	-0.069372	0.9112641	3426	3008	3982	2856	3623	3448
PITPNA	0.9575617	-0.062563	0.9885774	2278	2429	3069	2225	2741	2480
GBA3	6	2.5849625	0.3501193	0	1	0	0	1	5
RPS4XP22	0	#NUM!	0.6604275	0	1	1	0	0	0
GBA2	1.0589677	0.0826586	0.2327017	1932	2143	2454	2058	2524	2332
RPS4XP21	#DIV/0!	#DIV/0!	0.1513955	0	0	0	0	4	4
SYNPR	#DIV/0!	#DIV/0!	0.1700664	0	0	0	0	9	0
AC093525.10	1.1	0.1375035	0.9495281	7	1	2	0	7	4
C12orf60	1.0431894	0.0610011	0.6559854	104	73	124	98	127	89
BTRC	1.0930615	0.1283746	0.2246611	1021	901	1119	843	1212	1269
C12orf66	0.7576628	-0.400372	0.0610427	369	325	350	232	324	235
SYNPO	0.9769981	-0.033572	0.9183252	5404	6749	6715	5115	6607	6712
C12orf65	1.1055351	0.1447448	0.1814474	424	413	518	446	562	490
RPS4XP13	#DIV/0!	#DIV/0!	0.8030358	0	0	0	0	1	0
C12orf45	0.9508145	-0.072764	0.9794974	964	971	1257	1027	1087	921

RPS4XP18	0	#NUM!	0.824326	0	1	0	0	0	0
RPS4XP17	0.875	-0.192645	0.9564786	2	3	3	4	1	2
C12orf49	1.0695641	0.0970229	0.2406886	2235	2626	2801	2278	3101	2816
RPS4XP16	1.3333333	0.4150375	0.5885181	5	5	8	9	5	10
AC092134.1	1.125	0.169925	0.919697	6	2	0	3	4	2
KNL1	1.0069194	0.0099482	0.8018061	1300	696	1328	685	1313	1349
RPS4XP11	0.6666667	-0.584963	0.7317891	3	3	3	2	1	3
RPS4XP10	1.5	0.5849625	0.8395779	0	0	2	0	3	0
TATDN2P2	0.6156463	-0.699826	0.035218	102	95	97	39	78	64
TATDN2P3	0.75	-0.415037	0.6518819	14	6	20	2	20	8
IGKV2D-23	0	#NUM!	0.824326	0	0	1	0	0	0
UHRF1BP1	0.7962276	-0.328747	0.105979	1113	1100	1021	552	944	1079
DIP2A-IT1	0.1904762	-2.392317	0.0837766	10	8	3	0	3	1
TXK	3	1.5849625	0.6206259	1	0	0	3	0	0
ST20-AS1	0.6549708	-0.610498	0.1351394	53	63	55	30	46	36
TXN	1.1393429	0.188202	0.0413996	5347	5448	7132	6794	7577	6054
C12orf80	0	#NUM!	0.5200113	2	1	0	0	0	0
AC006511.2	1.2307692	0.2995603	0.7081784	4	1	8	7	2	7
AC006511.1	0.2727273	-1.874469	0.2828172	4	2	5	1	1	1
AC006511.4	0	#NUM!	0.0529648	3	3	5	0	0	0
AC006511.3	1.28	0.3561438	0.6333438	6	7	12	6	18	8
AC239802.2	#DIV/0!	#DIV/0!	0.3831122	0	0	0	0	5	0
AC073073.2	0.8297872	-0.269187	0.820007	17	4	26	12	14	13
AC073073.1	0.7333333	-0.447459	0.765124	4	3	8	3	3	5
SYNRG	0.8576653	-0.221513	0.2142913	1211	1011	1333	822	1114	1113
C12orf73	0.962	-0.055891	0.990784	142	168	190	136	182	163
C12orf71	0.3333333	-1.584963	0.6715904	3	0	0	0	1	0
AC090229.1	0.3333333	-1.584963	0.6363795	2	1	0	1	0	0
C12orf76	0.8760331	-0.190943	0.5508932	250	311	286	255	317	170
C12orf75	0.9935576	-0.009324	0.759732	880	901	1013	787	1055	934
AC139495.3	0.2	-2.321928	0.2552706	3	2	5	0	2	0
GBE1	0.7961165	-0.328949	0.0696947	764	610	789	481	605	636
RPL27AP	0	#NUM!	0.824326	0	0	1	0	0	0
AC136612.1	1.5357143	0.6189098	0.3698106	15	4	9	20	13	10
AP001024.1	2.5	1.3219281	0.5484377	1	0	1	3	2	0
SEC61G	1.0077964	0.0112042	0.5822751	1209	1454	1698	1519	1552	1324
SEC61B	1.1162925	0.1587151	0.1107569	2423	2996	3223	3299	3451	2897
AC020763.5	0	#NUM!	0.5781912	0	0	3	0	0	0
AC011841.1	1.5	0.5849625	0.8713737	0	2	0	0	2	1
AC020763.4	0.1111111	-3.169925	0.1744863	4	1	4	1	0	0
AC020763.3	0.5641026	-0.825971	0.3782576	25	9	5	8	3	11
AC020763.1	0.3333333	-1.584963	0.7249542	0	0	3	1	0	0
SCNM1	0.9565012	-0.064161	0.9652073	1232	1336	1662	1394	1432	1220
AC004112.1	0.8571429	-0.222392	0.9319721	0	3	4	0	1	5
AC015468.3	0	#NUM!	0.6385096	0	2	0	0	0	0
AC015468.1	0	#NUM!	0.824326	0	0	1	0	0	0
AL359075.2	0	#NUM!	0.824326	0	0	1	0	0	0
C12orf10	1.0823529	0.114171	0.3154932	510	620	655	693	716	523
AC012085.1	1.1880395	0.2485828	0.0323693	493	516	613	566	705	656
C12orf40	0.8547009	-0.226509	0.676025	50	26	41	22	46	32
C12orf43	0.9183585	-0.122871	0.7008062	632	799	884	670	690	766
C12orf42	0.3333333	-1.584963	0.1424582	10	11	12	1	1	9
C12orf29	0.8810618	-0.182685	0.4407532	659	570	730	567	559	600
AL157756.1	1.6666667	0.7369656	0.7354542	3	0	0	2	1	2

GBF1	1.0694548	0.0968755	0.3171437	2807	3206	3360	2517	3576	3931
NR6A1	1.0091724	0.0131727	0.6459234	10677	11461	12313	9022	12456	13289
AC008958.1	0	#NUM!	0.824326	0	0	1	0	0	0
COPG2	1.048198	0.0679113	0.3811688	719	692	892	651	884	879
COPG1	0.7872416	-0.345122	0.0107852	4950	5620	6219	4061	4756	4400
FRA10AC1	1.3910256	0.476149	0.0062423	354	234	504	463	576	480
AC139495.1	0.5555556	-0.847997	0.6215928	4	4	1	3	1	1
AC011591.2	#DIV/0!	#DIV/0!	0.8030358	0	0	0	0	0	1
HIST1H2AE	#DIV/0!	#DIV/0!	0.0406338	0	0	0	4	3	4
HIST1H2AG	0.6588735	-0.601927	0.006029	325	274	342	153	267	200
HIST1H2AC	1.0895954	0.1237925	0.1973267	690	620	766	730	825	707
HIST1H2AM	1	0	0.9890559	0	1	0	0	1	0
HIST1H2AL	0.5909091	-0.758992	0.5763618	13	2	7	8	1	4
HIST1H2AI	0.4727273	-1.08092	0.1689567	8	26	21	10	9	7
HIST1H2AH	0.5	-1	0.7561909	0	4	0	2	0	0
IL11RA	1.2396694	0.3099555	0.1514876	126	106	131	185	137	128
HIST1H2AJ	#DIV/0!	#DIV/0!	0.6099294	0	0	0	2	0	0
MBNL1-AS1	0.5	-1	0.6750635	2	0	4	0	2	1
HIST1H2BF	0.5283019	-0.920566	0.0855264	40	33	33	10	29	17
HIST1H2BE	1.1190476	0.1622714	0.8192061	20	11	11	5	10	32
HIST1H2BG	1.6	0.6780719	0.5746138	2	6	2	3	4	9
HIST1H2BB	#DIV/0!	#DIV/0!	0.8030358	0	0	0	0	1	0
HIST1H2BD	1.0417616	0.0590251	0.4295478	390	369	558	438	493	441
HIST1H2BC	0.9644219	-0.052264	0.9859083	291	223	273	234	250	275
ZCCHC8	1.1859986	0.2461023	0.0312352	1588	1154	1629	1466	1909	1809
COPB2	0.9029465	-0.147288	0.37667	5806	5418	6560	4494	5878	5686
ZCCHC7	1.2645914	0.3386714	0.0149467	746	491	819	775	973	852
ZCCHC6	1.40321	0.4887309	0.0010881	1177	772	1104	1102	1614	1568
COPB1	1.0079947	0.0114881	0.660908	3472	2204	3455	2588	3475	3141
ALOX15	0.6187215	-0.692638	0.011337	130	142	166	82	102	87
ZCCHC9	1.098923	0.1360903	0.1790881	1766	1322	1926	1574	2213	1723
ALOX12	0.8742515	-0.19388	0.7367541	44	51	72	46	50	50
IBA57-AS1	1	0	0.9991439	5	8	0	5	3	5
YWHAZP2	0.7246377	-0.464668	0.5181384	28	19	22	20	9	21
YWHAZP4	2.6666667	1.4150375	0.1411272	6	2	4	6	17	9
YWHAZP3	1.2	0.2630344	0.6538121	12	23	20	8	26	32
THPO	0.5254237	-0.928447	0.1792344	22	20	17	11	10	10
UBB	0.970795	-0.042761	0.8920217	3084	4421	4445	4012	4156	3433
UBC	0.9077421	-0.139646	0.4808078	12942	15873	17219	12515	14694	14578
YWHAZP5	1.3333333	0.4150375	0.8780815	0	3	0	1	2	1
FAM90A12P	0.3333333	-1.584963	0.7249542	0	0	3	1	0	0
FAM90A11P	0	#NUM!	0.824326	0	0	1	0	0	0
AC233280.1	2.1666667	1.1154772	0.5028013	0	3	3	0	9	4
AC233280.2	0.6666667	-0.584963	0.6627745	5	3	16	6	8	2
ZCCHC4	0.9985455	-0.0021	0.7590962	533	327	515	418	508	447
ZCCHC3	0.9520611	-0.070874	0.87588	2154	2766	2673	2188	2672	2369
ZCCHC2	1.0160428	0.0229611	0.6893607	325	260	350	251	317	382
UCN	1.4895833	0.5749088	0.1405947	20	36	40	53	55	35
THRB	1.0930537	0.1283643	0.3558989	490	416	620	378	555	735
THRA	0.7839465	-0.351173	0.0665574	428	485	582	346	413	413
AC091057.1	5	2.3219281	0.2048243	1	0	1	5	4	1
AC091057.3	1	0	0.9680569	36	33	26	19	35	41
AC091057.2	0	#NUM!	0.824326	1	0	0	0	0	0
AC068473.3	#DIV/0!	#DIV/0!	0.8030358	0	0	0	1	0	0

AC011747.1	#DIV/0!	#DIV/0!	0.2905913	0	0	0	2	0	3
AC068473.5	1.1282051	0.1740294	0.5413443	31	53	72	44	75	57
AC068473.1	0	#NUM!	0.5781912	0	0	3	0	0	0
AL163051.2	1.4823529	0.567889	0.1968207	39	19	27	28	48	50
AL163051.1	1.0502283	0.070703	0.5351162	121	129	188	164	154	142
AL031770.1	0.2	-2.321928	0.4169927	1	4	0	0	0	1
HIST1H2BN	0.826	-0.275786	0.4192143	139	131	230	129	147	137
HIST1H2BM	#DIV/0!	#DIV/0!	0.3981469	0	0	0	4	0	0
HIST1H2BO	0.7698413	-0.377367	0.5153045	44	21	61	20	51	26
HIST1H2BJ	0.9289559	-0.106318	0.8509471	289	291	349	314	291	258
CCRL1P1	1.6666667	0.7369656	0.8187196	3	0	0	0	5	0
HIST1H2BL	1.8	0.8479969	0.444997	4	0	6	9	1	8
HIST1H2BK	1.1690044	0.2252804	0.0446437	2105	2526	2641	2947	3071	2483
AL354766.2	2.15625	1.1085245	0.0635437	12	10	10	22	18	29
BUB3	1.1495261	0.2010393	0.0136758	4931	4749	5936	5270	6661	6020
AC087667.1	1	0	0.9621224	1	0	1	1	0	1
GBP2	1.1268966	0.1723551	0.056658	1345	1358	1647	1422	1858	1622
BUB1	1.087587	0.1211308	0.2025666	4286	3186	4459	3444	4827	4705
GBP1	1.5834921	0.6631096	1.49E-07	1095	870	1185	1348	1900	1740
GBP4	1.5354331	0.6186456	0.1009434	50	39	38	40	65	90
GBP3	0.9052369	-0.143633	0.6885591	338	175	289	185	267	274
FSIP1	0.625	-0.678072	0.7716751	5	0	3	3	0	2
FSIP2	1.1517857	0.2038723	0.5400022	40	31	41	32	42	55
AC005829.1	0.25	-2	0.3215019	9	0	3	0	0	3
CYYR1-AS1	0.9090909	-0.137504	0.9173355	4	4	3	2	7	1
OR2M4	#DIV/0!	#DIV/0!	0.8030358	0	0	0	0	0	1
AC018946.1	#DIV/0!	#DIV/0!	0.5070017	0	0	0	3	0	0
AL159174.1	0.5	-1	0.8652929	0	0	2	0	0	1
AC007878.1	0.3	-1.736966	0.2533151	10	0	10	1	1	4
GBP5	2.1052632	1.0740006	0.1830102	10	4	5	14	18	8
GBP7	2.6	1.3785116	0.1641699	1	3	6	13	4	9
OR2L2	0.5	-1	0.7417349	1	0	3	0	2	0
NR2F2-AS1	0.6576577	-0.604591	0.3492612	51	18	42	4	29	40
NOS2P3	0	#NUM!	0.6631647	1	0	1	0	0	0
NOS2P2	#DIV/0!	#DIV/0!	0.8030358	0	0	0	0	0	1
AP000679.1	#DIV/0!	#DIV/0!	0.5415473	0	0	0	0	2	1
AL391425.1	0.8333333	-0.263034	0.9000037	6	0	0	0	0	5
AC022239.1	0	#NUM!	0.824326	0	0	1	0	0	0
PTRHD1	0.9788918	-0.030779	0.86767	79	143	157	138	118	115
COL1A1	1.3	0.3785116	0.0009796	840	852	998	1151	1241	1105
COL1A2	0.0965909	-3.371969	1.60E-09	55	57	64	4	7	6
AC021744.1	0	#NUM!	0.6436235	2	0	0	0	0	0
AP000533.1	0	#NUM!	0.824326	0	1	0	0	0	0
HIST2H2AA3	2.5	1.3219281	0.3832601	1	2	1	6	1	3
HIST2H2AA4	1	0	0.9988754	1	1	0	1	1	0
AP000533.2	0.7142857	-0.485427	0.8972593	0	0	7	2	3	0
HMGB3P9	#DIV/0!	#DIV/0!	0.8030358	0	0	0	0	1	0
TUBA1C	0.9981999	-0.002599	0.6350403	8730	10491	11333	9860	10864	9775
AL590094.1	0.8503401	-0.233888	0.7246328	27	48	72	41	43	41
TUBA1B	0.8305396	-0.267879	0.1136165	3722	4732	5148	3640	4034	3623
TUBA1A	1.0505952	0.071207	0.4927895	291	365	352	385	359	315
HMGB3P6	5	2.3219281	0.4410369	0	1	0	0	4	1
AC087762.2	1.5	0.5849625	0.7734914	0	0	2	3	0	0
HMGB3P8	#DIV/0!	#DIV/0!	0.8030358	0	0	0	0	0	1

HMGB3P4	2.2307692	1.1575413	0.3410491	7	4	2	0	11	18
AL513165.1	1.062963	0.0880913	0.4734399	156	173	211	175	200	199
RBMY1F	0.6650485	-0.588468	0.1728557	67	63	76	31	79	27
RBMY1E	0.2	-2.321928	0.4485616	0	5	0	1	0	0
RBMY1J	0.1111111	-3.169925	0.1580758	4	4	1	1	0	0
RN7SL809P	0.8	-0.321928	0.824795	4	7	4	6	4	2
HEXDC	1.1839888	0.2436554	0.1395154	167	216	329	297	278	268
RILPL2	1.1327731	0.1798589	0.3005517	181	208	206	271	229	174
RILPL1	0.9379172	-0.092467	0.7897767	445	482	571	381	574	450
GS1-279B7.1	#DIV/0!	#DIV/0!	0.2497949	0	0	0	0	3	3
RBMY1D	0.996124	-0.005603	0.8371455	72	46	140	54	93	110
RBMY1B	1.4782609	0.5639009	0.5237488	1	16	6	14	14	6
SUMO2P17	1.0641026	0.0896372	0.7550231	15	38	25	45	18	20
SUMO2P19	0.2142857	-2.222392	0.2139884	1	11	2	0	0	3
AL137244.1	2.3333333	1.2223924	0.365385	0	0	6	7	2	5
MIEF2	0.7297116	-0.454602	0.034289	405	522	564	386	370	332
MIEF1	0.7655546	-0.385423	0.0080183	3807	4056	4127	2386	3364	3429
SUMO2P16	#DIV/0!	#DIV/0!	0.5509701	0	0	0	0	3	0
UNG	0.7640577	-0.388247	0.0128053	2697	3524	3489	2298	2699	2422
TUBA3E	0.9090909	-0.137504	0.9347909	7	5	10	5	9	6
TUBA3D	0.5769231	-0.793549	0.3315018	18	10	24	11	6	13
UNK	0.9455504	-0.080774	0.9215712	550	498	660	499	510	606
GBX2	0.9916107	-0.012154	0.756601	162	178	256	219	198	174
AC012339.1	0.8026316	-0.31719	0.626378	17	27	32	6	33	22
AC012339.2	#DIV/0!	#DIV/0!	0.8030358	0	0	0	1	0	0
SUMO2P20	3	1.5849625	0.6896151	1	0	0	0	3	0
AL031283.1	1.9047619	0.9296107	0.2472587	2	9	10	17	18	5
AL031283.3	0	#NUM!	0.824326	0	0	1	0	0	0
RN7SL806P	#DIV/0!	#DIV/0!	0.5371397	0	0	0	0	0	3
AC008467.1	1.8571429	0.8930848	0.4858906	1	1	5	2	6	5
SPDYE20P	7.5	2.9068906	0.134963	0	0	2	0	10	5
OR2T8	0.2777778	-1.847997	0.2324466	7	4	7	0	0	5
OR2T7	#DIV/0!	#DIV/0!	0.8030358	0	0	0	0	1	0
TUBA4A	0.6486486	-0.624491	0.5658467	6	12	19	11	12	1
RBMXL3	0	#NUM!	0.5075476	0	0	4	0	0	0
RBMXL2	#DIV/0!	#DIV/0!	0.5338029	0	0	0	0	1	2
RBMXL1	1.1388245	0.1875454	0.0662255	2618	2078	2637	2316	2959	3076
AL121721.1	#DIV/0!	#DIV/0!	0.5509701	0	0	0	0	3	0
SNAP23P1	1.5714286	0.6520767	0.6778372	0	6	1	5	0	6
SPDYE21P	0.5384615	-0.893085	0.5180553	6	3	4	1	4	2
OVCH1-AS1	2.3333333	1.2223924	0.4092779	1	4	1	5	0	9
RBMXP4	#DIV/0!	#DIV/0!	0.1171991	0	0	0	0	3	6
RBMXP2	0.6538462	-0.612977	0.5970437	9	15	2	11	3	3
RBMXP1	0.4285714	-1.222392	0.5685015	0	6	1	0	3	0
AC109597.2	3	1.5849625	0.6206259	1	0	0	3	0	0
TIA1	0.9785016	-0.031354	0.9028134	1762	1027	1816	1132	1724	1650
AC109597.1	0	#NUM!	0.5337954	0	3	0	0	0	0
UST	0.5454545	-0.874469	0.2193763	14	42	21	12	26	4
ALG1L10P	0.7878788	-0.343954	0.7929485	12	4	17	12	11	3
Z98749.2	1.1179245	0.1608228	0.5907166	96	37	79	49	87	101
Z98749.1	#DIV/0!	#DIV/0!	0.6411933	0	0	0	0	0	2
AL158835.2	1	0	0.9000728	6	4	8	12	5	1
AL158835.1	0	#NUM!	0.6436235	2	0	0	0	0	0
RN7SL802P	0	#NUM!	0.5337954	0	3	0	0	0	0

AL163540.1	3	1.5849625	0.25057	0	2	3	4	3	8
UTY	0.9856631	-0.020834	0.8692401	393	330	393	303	376	421
MIR378J	1.25	0.3219281	0.8621254	1	3	0	3	0	2
AL021392.1	0.8511905	-0.232446	0.7259987	52	37	79	55	55	33
MIR378H	0	#NUM!	0.1223437	5	1	2	0	0	0
MIR378A	0	#NUM!	0.824326	0	1	0	0	0	0
AC079684.1	0.8793103	-0.185556	0.937765	22	5	31	20	7	24
MIEN1	0.841133	-0.249594	0.3798826	231	273	308	246	236	201
OR2W3	0.3043478	-1.716207	0.0242624	16	32	21	7	12	2
TIFA	1.0879433	0.1216033	0.3114189	493	454	463	427	559	548
AC111170.3	1.2	0.2630344	0.8574702	3	2	0	6	0	0
TIE1	7	2.8073549	0.3235909	0	1	0	0	0	7
AKR7L	1.2673267	0.3417885	0.334305	33	25	43	45	51	32
AC010525.1	#DIV/0!	#DIV/0!	0.6411933	0	0	0	0	0	2
AC004594.1	0	#NUM!	0.6840298	0	0	2	0	0	0
OR2V2	0	#NUM!	0.6436235	2	0	0	0	0	0
GCC2	1.3197317	0.4002447	0.023202	1373	743	1462	1089	1882	1751
GCC1	0.7901488	-0.339804	0.0371732	1168	1398	1332	840	1164	1076
AC023162.1	#DIV/0!	#DIV/0!	0.2887809	0	0	0	0	0	6
UXT	1.0197595	0.0282289	0.6064313	323	397	444	486	374	327
TCL1B	2.4166667	1.2730185	0.1577287	3	3	6	10	8	11
TCL1A	0	#NUM!	0.1753461	2	1	4	0	0	0
ALG1L15P	1.3333333	0.4150375	0.8885862	3	0	0	0	1	3
AC010275.1	1.453125	0.5391588	0.2567575	27	15	22	49	24	20
ADPGK-AS1	0	#NUM!	0.824326	0	0	1	0	0	0
GCAT	0.8375116	-0.255819	0.3019743	312	334	431	304	301	297
AC008957.2	3	1.5849625	0.6122545	0	0	1	2	0	1
AC020916.1	0.8988604	-0.153831	0.6701846	217	203	282	205	248	178
AC008957.1	0.3333333	-1.584963	0.2396304	3	3	21	0	4	5
AC008957.3	0.6538462	-0.612977	0.1956751	68	38	50	15	53	34
AC020916.2	1	0	0.9988411	1	0	1	0	2	0
GCDH	0.9529164	-0.069578	0.9927803	457	402	564	459	462	435
ZBED9	0.9641694	-0.052641	0.8999536	109	55	143	107	98	91
ZBED8	0.9197397	-0.120702	0.7971364	133	136	192	117	192	115
MIER3	1.057603	0.0807981	0.4460812	1219	774	1236	859	1329	1227
MIER2	0.8624242	-0.21353	0.3767476	466	512	672	467	473	483
MIER1	1.0099751	0.0143197	0.7740617	854	472	679	526	750	749
ZBED2	0.75	-0.415037	0.7653924	4	5	3	3	4	2
ZBED1	0.7718991	-0.373516	0.0152226	3756	4639	4722	3266	3406	3453
ZBED6	1.25	0.3219281	0.8564646	7	4	1	0	10	5
ZBED5	0.9563046	-0.064458	0.9950109	874	549	980	677	851	770
ZBED4	0.9158961	-0.126744	0.5444325	2166	2119	2528	1597	2259	2384
ZBED3	1.1428571	0.1926451	0.8767319	1	0	6	0	5	3
AC025946.1	1.8	0.8479969	0.5238146	1	8	1	11	7	0
MTCO1P12	0.9110553	-0.13439	0.5546997	633	622	735	499	708	606
MTCO1P11	1.0769231	0.1069152	0.8902687	4	2	7	1	3	10
AC073326.1	3.25	1.7004397	0.3192385	1	0	3	0	7	6
MTCO1P15	0.6666667	-0.584963	0.8466488	3	0	0	0	0	2
AL731537.2	1	0	0.9890559	1	0	0	0	1	0
AL731537.1	0	#NUM!	0.824326	0	0	1	0	0	0
MTCO1P28	0.5116279	-0.966833	0.2464106	15	11	17	7	11	4
MTCO1P22	2	1	0.807847	1	0	0	0	1	1
ALG1L11P	0.6818182	-0.552541	0.6487826	9	3	10	5	4	6
MTCO1P25	0	#NUM!	0.5387425	3	0	0	0	0	0

MTCO1P30	0	#NUM!	0.0493849	5	5	1	0	0	0
STARD3NL	1.0049934	0.0071861	0.6574128	1130	1323	1352	1215	1427	1182
RSL24D1P1	0	#NUM!	0.6604275	0	1	1	0	0	0
ALG1L12P	0.5833333	-0.777608	0.4073589	21	4	11	5	6	10
MTCO1P31	0.4	-1.321928	0.6326277	5	0	0	0	0	2
OR3A3	0.8863636	-0.174029	0.8941539	14	4	26	6	22	11
OR3A2	1.0888889	0.1228567	0.6622597	25	30	35	40	23	35
OR3A1	1	0	0.941895	13	14	0	0	11	16
MTCO1P40	0.25	-2	0.6181385	0	0	4	0	0	1
TUFMP1	1	0	0.9870974	0	1	1	0	0	2
ALG1L13P	0.4468085	-1.162271	0.0564976	32	35	27	4	24	14
RSL24D1P6	0.0909091	-3.459432	0.1457242	0	5	6	0	0	1
MTCO1P44	#DIV/0!	#DIV/0!	0.5070017	0	0	0	3	0	0
GCH1	1.0676466	0.0944341	0.2301227	1921	1898	2168	1755	2396	2241
ALG1L14P	1.5	0.5849625	0.7362006	1	0	3	1	4	1
AP001267.3	#DIV/0!	#DIV/0!	0.5371397	0	0	0	0	0	3
AP001267.1	1.8	0.8479969	0.4408997	5	0	5	7	4	7
AL035250.2	0	#NUM!	0.824326	1	0	0	0	0	0
PHRF1	0.8490961	-0.236	0.1493726	1966	2361	2366	1695	2004	1984
TPRA1	1.0607811	0.0851269	0.3106722	964	1186	1486	1388	1321	1148
RPP25L	1.0639481	0.0894278	0.3450338	579	768	811	774	814	708
AP001994.1	0.6666667	-0.584963	0.7698164	6	0	0	0	3	1
ERICH6B	0.7058824	-0.5025	0.797986	4	0	13	5	4	3
VCL	0.8930848	-0.163131	0.3624758	5649	6230	6631	4216	5918	6397
GCM2	2.8	1.4854268	0.3567756	0	1	4	0	4	10
GCM1	1.4804128	0.5659995	6.33E-09	3262	3020	3699	4337	5329	5110
NDRG4	0.9055441	-0.143143	0.8134035	140	138	209	151	119	171
VCP	1.0922228	0.1272672	0.114529	12080	14350	15089	14183	16077	15088
NDRG3	0.8459449	-0.241364	0.1360523	1500	1775	1879	1251	1628	1481
VCX	0.1666667	-2.584963	0.1838312	7	5	0	1	1	0
NDRG2	0.5	-1	0.268248	16	10	24	0	13	12
NDRG1	0.7882251	-0.34332	0.017273	2317	2728	3006	1823	2379	2144
MCPH1-AS1	1.4714286	0.5572175	0.2033124	23	24	23	35	26	42
AL355353.1	0.5	-1	0.4450311	7	2	11	4	3	3
GCN1	0.7569323	-0.401764	0.004499	9001	10420	10764	5963	8465	8420
GCKR	0	#NUM!	0.824326	0	0	1	0	0	0
VDR	1.826087	0.8687555	0.2420988	4	12	7	19	9	14
AC244021.2	0	#NUM!	0.824326	1	0	0	0	0	0
AC244021.1	0.375	-1.415037	0.4043936	7	0	9	2	4	0
RPL26P6	0.5	-1	0.5621104	4	3	1	0	3	1
GCLC	0.772685	-0.372048	0.0216328	1109	934	1186	667	912	916
AC007388.1	0.7284946	-0.45701	0.1723021	103	128	141	103	82	86
PQLC1	0.8739316	-0.194408	0.522944	251	376	309	288	273	257
PQLC3	0.9087719	-0.13801	0.5784558	614	483	613	446	576	532
PQLC2	0.8760393	-0.190932	0.416758	789	902	955	819	785	714
GCLM	0.569902	-0.811214	4.11E-08	1550	1251	1691	725	885	950
RPL26P3	0	#NUM!	0.824326	1	0	0	0	0	0
AP000439.1	0.8	-0.321928	0.9017373	3	2	0	3	1	0
AP000439.3	0.7747748	-0.368151	0.4713775	60	84	78	83	55	34
GATAD1	0.9355124	-0.096171	0.7127924	1124	1045	1227	842	1178	1157
Z98259.3	0.5	-1	0.5762866	3	5	0	0	2	2
GCNA	0.6722689	-0.57289	0.2731181	35	29	55	21	36	23
AC114316.2	0.9444444	-0.082462	0.9532047	6	11	1	7	3	7
VGF	0.3795256	-1.397731	2.25E-07	226	261	314	126	102	76

MMRN2	1.2244898	0.2921808	0.5670584	6	23	20	24	19	17
WHAMMP3	1.1666667	0.2223924	0.7397513	19	9	14	5	17	27
WHAMMP2	0.5526316	-0.85561	0.3834634	12	7	19	4	3	14
TUBAL3	#DIV/0!	#DIV/0!	0.242062	0	0	0	6	0	0
AP003170.5	0	#NUM!	0.824326	1	0	0	0	0	0
AP003170.4	0	#NUM!	0.0377969	0	9	5	0	0	0
AP003170.3	9	3.169925	0.1688707	0	1	0	4	0	5
AP003170.1	0.05	-4.321928	0.0171338	6	6	8	0	0	1
CDC144NL-AS	1.707649	0.7720115	4.39E-10	725	614	792	1123	1270	1246
VHL	0.8553114	-0.225478	0.1777053	1836	1881	2289	1333	1868	1936
OGFR-AS1	0.7560976	-0.403356	0.698525	1	20	20	7	20	4
OR5BK1P	0	#NUM!	0.4521054	1	0	3	0	0	0
VIM	1.5	0.5849625	0.8582217	2	0	0	0	1	2
VIP	2.0909091	1.0641303	0.1322768	8	5	9	13	16	17
RPL26L1	1.0191083	0.0273073	0.5031384	1682	1829	2141	2023	2066	1671
VIT	#DIV/0!	#DIV/0!	0.8030358	0	0	0	0	1	0
GCSH	1.1838565	0.2434942	0.1922799	153	121	172	159	214	155
BVES	1.1388889	0.187627	0.4947359	48	43	53	49	59	56
KRTAP17-1	1.2962963	0.3743955	0.5871971	8	4	15	6	13	16
OR1J1	#DIV/0!	#DIV/0!	0.4737294	0	0	0	0	4	0
LIN28B-AS1	1.2727273	0.3479233	0.6280255	1	6	15	8	10	10
AL031772.1	#DIV/0!	#DIV/0!	0.4501902	0	0	0	0	0	4
ADAM20P1	1.2222222	0.2895066	0.6824229	1	5	12	9	7	6
AC005578.1	0.75	-0.415037	0.9014748	0	3	5	4	0	2
AC005828.1	3	1.5849625	0.6732949	0	0	1	0	0	3
SLAIN2	1.0545105	0.0765734	0.3036525	1515	1346	1817	1328	1819	1786
SLAIN1	0.8333333	-0.263034	0.4203165	105	122	151	82	132	101
AC007879.3	0.1428571	-2.807355	0.2875112	0	6	1	0	0	1
AC005828.6	7	2.8073549	0.2588739	1	0	0	1	5	1
GSDMD	0.5583424	-0.840778	0.0020835	229	343	345	203	136	173
GSDMC	4	2	0.5246085	1	0	0	0	1	3
GSDMB	1.1263941	0.1717116	0.2958546	158	116	264	208	212	186
AC011509.2	0.6521739	-0.616671	0.563716	10	8	5	5	3	7
AC011509.1	0	#NUM!	0.824326	0	0	1	0	0	0
ASAH2B	1.8046358	0.8517077	0.0019435	116	73	113	106	222	217
CHRFAM7A	0	#NUM!	0.0962815	2	0	9	0	0	0
GSDME	1.0125392	0.0179777	0.6368086	499	515	581	460	585	570
AL137003.2	0.65	-0.621488	0.677847	3	5	12	6	1	6
AL137003.1	0.0416667	-4.584963	0.0104201	6	4	14	0	1	0
CACNA1C-IT3	0.6	-0.736966	0.7886603	1	1	3	2	1	0
AC116618.1	0.58	-0.785875	0.0347589	109	62	129	62	58	54
HOXD13	1.125	0.169925	0.9030197	3	5	0	5	3	1
HOXD12	1.2307692	0.2995603	0.8562069	3	10	0	2	7	7
HOXD10	0.25	-2	0.3738853	5	2	1	2	0	0
OR1M1	2	1	0.814195	0	1	0	0	0	2
OR1L8	0	#NUM!	0.4460243	4	0	0	0	0	0
AC093117.1	0	#NUM!	0.5337954	0	3	0	0	0	0
AC093801.1	0	#NUM!	0.824326	1	0	0	0	0	0
AC027612.3	1.040724	0.0575875	0.6870465	71	61	89	70	68	92
AL138955.1	6.3333333	2.662965	0.07042	0	0	3	12	4	3
AC027612.1	#DIV/0!	#DIV/0!	0.6482162	0	0	0	0	1	1
ZFP36	1.0909091	0.1255309	0.3239798	200	235	280	245	274	261
ZFP30	0.8295455	-0.269607	0.3843121	244	137	235	152	197	162
ZFP37	1.2430556	0.3138908	0.2508939	106	46	136	84	137	137

AC004593.1	2.25	1.169925	0.536979	4	0	0	7	2	0
SYPL1	1.0881007	0.1218121	0.1628423	2597	2530	2739	2359	3128	3072
ZFP41	1.0816327	0.1132106	0.4985951	89	91	114	108	100	110
ZFP42	0.9337223	-0.098935	0.6711807	3954	4574	4840	3379	4569	4534
AC010620.1	#DIV/0!	#DIV/0!	0.8030358	0	0	0	0	0	1
AL445524.1	1.2122905	0.2777355	0.3383274	58	32	89	86	84	47
PHPT1	1.0701317	0.0977883	0.2962927	2719	3550	3755	3839	3924	2964
AC022968.1	1.6	0.6780719	0.4985759	3	10	2	10	9	5
AP000532.2	0.3809524	-1.392317	0.2719908	9	6	6	5	1	2
AP000532.1	0	#NUM!	0.6840298	0	0	2	0	0	0
AL031282.2	1.1568627	0.2102177	0.653106	12	14	25	12	20	27
AL031282.1	0.8241758	-0.278976	0.365916	134	177	144	117	119	139
VTN	0.2857143	-1.807355	0.1749764	8	4	9	0	2	4
HMBOX1	0.9633508	-0.053867	0.9717794	266	227	271	183	243	310
SPDYE10P	0	#NUM!	0.6840298	0	0	2	0	0	0
AL513164.1	2	1	0.7300836	1	0	1	0	4	0
ESRRA	0.8461812	-0.240962	0.2571527	1540	1848	2242	1670	1552	1542
ZG16B	0.6923077	-0.530515	0.7387281	0	7	6	0	4	5
ESRRB	10	3.3219281	0.1638217	0	0	1	0	2	8
ESRRG	1.6535433	0.7255608	0.0042747	97	69	88	110	150	160
OR1P1	0	#NUM!	0.824326	0	0	1	0	0	0
AC091544.8	0.5	-1	0.81106	0	2	0	0	0	1
ZFP69	0.8319559	-0.265421	0.4685971	97	110	156	96	119	87
ZFP62	1.0204484	0.0292032	0.578261	1460	977	1622	1115	1538	1489
ZFP64	0.8796888	-0.184935	0.2930103	1131	1190	1278	903	1227	1036
AC091544.2	0	#NUM!	0.824326	1	0	0	0	0	0
AC091544.1	0	#NUM!	0.824326	0	1	0	0	0	0
AC078850.1	2	1	0.3631551	3	6	2	13	8	1
AC091544.4	0.2	-2.321928	0.436139	4	0	1	0	0	1
AC091544.3	1.2857143	0.3625701	0.7566915	0	1	6	3	0	6
AL021393.1	5	2.3219281	0.4322862	1	0	0	0	3	2
AL139294.1	#DIV/0!	#DIV/0!	0.5509701	0	0	0	0	3	0
ZFP82	1.0866873	0.1199369	0.3904323	228	164	254	210	276	216
PROSER2-AS1	0.9090353	-0.137592	0.4857773	2006	2131	2404	1500	2214	2232
SPDYE12P	1.2631579	0.337035	0.6218869	3	17	18	9	26	13
VWF	0.7	-0.514573	0.5226856	13	15	12	9	10	9
ZFP91	0.8798526	-0.184666	0.3217448	1361	1226	1483	875	1401	1305
ZFP90	0.9772462	-0.033206	0.9039837	575	469	670	420	575	680
AC009054.2	0.6428571	-0.63743	0.6733662	2	3	9	1	4	4
ESRP2	0.9760472	-0.034977	0.8699475	2734	2818	3257	2465	3083	3050
ESRP1	1.0194284	0.0277605	0.4874412	4659	4300	5247	3964	5331	5187
SPDYE13P	0	#NUM!	0.6840298	0	0	2	0	0	0
AC018697.1	0	#NUM!	0.199693	0	4	3	0	0	0
SNRPA1	0.865984	-0.207588	0.2075018	3500	3264	4369	3019	3520	3102
AP001360.2	2.0555556	1.0395284	0.1945322	9	3	6	17	12	8
AP001360.1	0	#NUM!	0.6436235	2	0	0	0	0	0
AC245100.4	0.8451613	-0.242701	0.6079882	49	51	55	24	46	61
AC245100.3	0.2857143	-1.807355	0.3628983	0	3	11	0	4	0
RN7SL812P	0.2083333	-2.263034	0.1021365	6	8	10	4	0	1
AC245100.2	0.3478261	-1.523562	0.3676662	5	0	18	0	8	0
AC245100.1	1.5	0.5849625	0.6424642	1	0	5	7	0	2
AP005233.2	3.3333333	1.7369656	0.3760575	0	3	0	8	2	0
AC245100.8	0.8685446	-0.203328	0.6540504	58	74	81	55	63	67
AC245100.7	1.5	0.5849625	0.8642665	0	2	0	0	1	2

AL136164.4	6.2	2.6322682	0.0204487	3	0	2	11	9	11
RNU6-907P	0	#NUM!	0.6840298	0	0	2	0	0	0
AL136164.3	#DIV/0!	#DIV/0!	0.8030358	0	0	0	0	0	1
AL136164.1	0	#NUM!	0.6840298	0	0	2	0	0	0
PRRT1B	0.9714286	-0.04182	0.9751301	12	14	9	8	14	12
SPDYE14P	6	2.5849625	0.3145	1	0	0	3	0	3
SNRPB2	1.037643	0.0533101	0.3235943	2172	2011	2724	2269	2629	2269
AC024145.1	0	#NUM!	0.824326	0	0	1	0	0	0
RCVRN	0	#NUM!	0.6436235	2	0	0	0	0	0
ERVMER34-1	2.3125	1.2094534	0.1460689	5	7	4	10	14	13
TXNRD3	0.6716418	-0.574236	0.2008976	66	55	80	59	38	38
TXNRD2	0.8782344	-0.187322	0.4843581	394	421	499	396	429	329
AC092279.2	0.75	-0.415037	0.706167	13	2	25	5	14	11
TXNRD1	0.5844989	-0.774728	1.49E-08	24633	23465	26116	10760	16022	16596
AC092279.1	0.7181467	-0.477649	0.1755504	73	89	97	60	66	60
AC021016.2	1.75	0.8073549	0.2149623	12	7	13	37	10	9
AC021016.1	1.3333333	0.4150375	0.8719217	2	0	1	0	3	1
CBLN3	0.7846154	-0.349942	0.6363685	18	18	29	17	17	17
CBLN2	0.1153846	-3.115477	0.0284323	7	5	14	0	2	1
CBLN1	0.7272727	-0.459432	0.602533	16	9	19	12	9	11
AC104170.2	#DIV/0!	#DIV/0!	0.3831122	0	0	0	0	5	0
AC104170.1	0.5555556	-0.847997	0.6078284	2	4	3	0	3	2
RN7SL810P	0.9166667	-0.125531	0.9639154	4	5	3	6	4	1
GDE1	0.9566197	-0.063983	0.9016085	1649	1787	1889	1464	1863	1767
DHFRP1	0.6033654	-0.728896	0.0135828	494	257	497	311	200	242
CU633904.1	0.2	-2.321928	0.4541539	0	2	3	0	0	1
DHFRP6	0	#NUM!	0.2919745	1	0	5	0	0	0
AC008560.1	1.0909091	0.1255309	0.5520308	88	58	85	107	88	57
ZFPL1	1.1696429	0.2260681	0.473122	22	39	51	60	38	33
AGFG2	0.9028476	-0.147446	0.6535511	322	383	489	340	362	376
AGFG1	1.1476289	0.1986562	0.0346227	5242	4507	5729	4785	6622	6356
AC062017.1	2	1	0.6461654	0	1	1	3	0	1
PDE12	0.7818305	-0.355072	0.0242019	1439	1447	1528	877	1310	1264
CBLL1	0.7932181	-0.334211	0.0447316	1057	878	1073	669	841	876
ZFPM1	0.8586762	-0.219814	0.6352396	164	180	215	215	137	128
NECAB2	0.4761905	-1.070389	0.4479756	9	0	12	2	3	5
NECAB1	2.5555556	1.353637	0.255671	5	1	3	5	16	2
NECAB3	0.887384	-0.17237	0.4833931	1173	1279	1535	1244	1252	1042
PDE1C	2.8	1.4854268	0.1415743	3	3	4	5	15	8
PDE1B	0.2580645	-1.954196	0.0854337	11	5	15	3	1	4
PDE1A	0.5501859	-0.862009	0.017463	73	107	89	53	53	42
AC023161.2	1.2	0.2630344	0.9016998	3	2	0	1	0	5
AC012181.3	3.1428571	1.6520767	0.152003	3	3	1	8	9	5
ODF2-AS1	5	2.3219281	0.4650112	0	0	1	0	0	5
CD81-AS1	1.9	0.9259994	0.4117462	4	1	5	17	0	2
PDE2A	0.6349206	-0.655352	0.2686036	40	43	43	38	33	9
AC012181.2	2.7272727	1.447459	0.0430541	9	7	6	24	27	9
GDF3	0	#NUM!	0.1795979	4	3	0	0	0	0
GDF6	0.4850394	-1.043826	1.52E-07	337	429	504	170	227	219
HSPBAP1	1.0499349	0.0702998	0.3711543	798	689	816	738	878	802
GDF5	0.5	-1	0.6011019	3	4	1	0	0	4
GDF7	1.8181818	0.8624965	0.4312185	1	6	4	5	10	5
GDF9	0.4556962	-1.133856	0.0721213	34	27	18	8	11	17
AC020917.1	0.5714286	-0.807355	0.6287547	0	8	6	0	8	0

AC020917.4	0.7129187	-0.488191	0.255791	67	45	97	36	53	60
CORO6	1.3605313	0.4441702	0.0227435	175	124	228	218	284	215
PDE3A	0	#NUM!	0.824326	1	0	0	0	0	0
CORO7	1.2605459	0.3340487	0.1478964	130	103	170	231	164	113
OR2A1	#DIV/0!	#DIV/0!	0.6554062	0	0	0	0	2	0
GDI1	1.0217317	0.0310164	0.4423565	2467	2744	3532	2715	3370	2848
SH3KBP1	1.0123689	0.0177351	0.5374188	2091	1908	2388	1836	2408	2222
GDI2	1.1754248	0.2331822	0.0062487	13342	12113	15212	13533	17479	16789
ZBTB8OS	1.1416122	0.1910727	0.073505	560	561	715	674	708	714
AC005921.3	0.2	-2.321928	0.4324956	4	0	1	1	0	0
ATP6V0B	0.9034142	-0.146541	0.6126585	1820	2375	2483	2154	2181	1698
BBS10	1.6216216	0.6974372	0.2247167	14	10	13	19	21	20
BBS12	1.7798742	0.8317753	0.0041401	52	46	61	81	112	90
AP001266.2	0	#NUM!	0.3473206	0	5	0	0	0	0
WAC	1.2333333	0.3025628	0.0059983	4653	3636	5211	4402	6302	5946
KPTN	0.910045	-0.13599	0.643067	383	414	537	355	479	380
ATP6V0C	0.5	-1	0.1784638	19	11	28	8	11	10
WAS	0.6216216	-0.685891	0.3058105	14	37	23	16	11	19
TLDC2	2.75	1.4594316	0.3707269	0	0	4	2	5	4
TLDC1	0.8082132	-0.307192	0.0415612	1358	1498	1722	1056	1344	1300
CORIN	1.3333333	0.4150375	0.6789527	3	4	5	4	7	5
TJP1	0.8462217	-0.240892	0.1950826	3884	3312	4145	2183	3514	3900
AC107294.2	0.5833333	-0.777608	0.6246483	2	3	7	1	2	4
TPPP3	4.0909091	2.0324215	2.15E-07	19	14	22	79	74	72
AC093458.2	0	#NUM!	0.4324683	2	0	2	0	0	0
AC107294.3	0.5	-1	0.812391	0	2	0	0	1	0
AC012671.1	0	#NUM!	0.4217812	0	0	5	0	0	0
TJP3	0.9168796	-0.125196	0.6527371	1657	2044	2170	1808	1861	1714
TJP2	0.9868748	-0.019061	0.775953	12455	12416	14519	10649	14159	14065
AC104076.1	0	#NUM!	0.6840298	0	0	2	0	0	0
PDE8B	1.3636364	0.447459	0.1447146	89	47	62	71	103	96
PDE8A	0.8171472	-0.291332	0.2455863	492	295	531	285	411	381
AL022721.1	0.75	-0.415037	0.8416855	0	4	4	0	1	5
AC007389.5	0	#NUM!	0.4460243	4	0	0	0	0	0
BX284668.6	#DIV/0!	#DIV/0!	0.6411933	0	0	0	0	0	2
AC007389.2	#DIV/0!	#DIV/0!	0.6411933	0	0	0	0	0	2
BX284668.1	0	#NUM!	0.5387425	3	0	0	0	0	0
AC090071.1	2	1	0.6540525	0	0	2	4	0	0
LMX1B	0.4	-1.321928	0.6311157	1	0	4	0	1	1
UGDH-AS1	1.1315789	0.1783372	0.8147785	13	17	8	6	16	21
BX284668.5	1	0	0.9406094	0	0	5	1	3	1
BX284668.4	0.3333333	-1.584963	0.4292802	5	7	0	4	0	0
PDE9A	0.9714286	-0.04182	0.9345348	9	8	18	15	14	5
BX284668.2	2.8666667	1.5193742	0.0588981	3	7	5	15	16	12
ATP6V1A	1.074308	0.1034077	0.3040867	4924	4167	4999	3669	5748	5720
SEL1L2	#DIV/0!	#DIV/0!	0.3831122	0	0	0	0	5	0
SEL1L3	0.6445087	-0.633728	0.0021269	323	334	381	162	271	236
AL049629.1	#DIV/0!	#DIV/0!	0.3109758	0	0	0	0	6	0
PIGHP1	2.8	1.4854268	0.3379768	1	1	3	0	7	7
AC093698.1	3.6666667	1.8744691	0.2978098	0	3	0	6	1	4
BCAP31P1	#DIV/0!	#DIV/0!	0.8030358	0	0	0	0	1	0
ATP6V1H	0.9366712	-0.094385	0.858379	1123	1130	1442	1186	1231	1044
SUDS3	0.880659	-0.183345	0.2505927	2579	2413	2717	1872	2532	2385
OR2B6	0.5258621	-0.927244	0.082741	42	25	49	21	19	21

ATP6V1D	1.2518879	0.3241054	0.001899	1928	1693	2338	2197	2905	2358
ATP6V1F	1.1295982	0.1758097	0.172187	503	595	669	750	704	542
OR2B2	0	#NUM!	0.4217812	0	0	5	0	0	0
OR2A7	0.6842105	-0.547488	0.5901918	16	8	14	7	19	0
PDE3B	0	#NUM!	0.6604275	0	1	1	0	0	0
AC011603.2	5	2.3219281	0.1315798	1	1	1	3	9	3
AC011603.1	0	#NUM!	0.824326	0	1	0	0	0	0
AC063926.1	0	#NUM!	0.824326	1	0	0	0	0	0
UPK1B	5	2.3219281	0.111831	0	4	0	9	6	5
UPK1A	0	#NUM!	0.0069665	5	4	10	0	0	0
AP003171.2	0	#NUM!	0.5387425	3	0	0	0	0	0
AP003171.1	0.1686747	-2.567685	0.0051322	46	5	32	0	5	9
PDE4B	1.3333333	0.4150375	0.6165126	12	3	6	7	9	12
PDE4A	0.8864119	-0.173951	0.5113307	260	320	362	219	341	275
PDE4D	1.2156908	0.2817763	0.0508457	1576	1131	1665	1227	1973	2115
PDE4C	0.7297297	-0.454566	0.3855814	55	41	52	49	31	28
UPK3B	0.1428571	-2.807355	0.1138469	3	10	1	0	1	1
UPK3A	#DIV/0!	#DIV/0!	0.5509701	0	0	0	0	3	0
RCN1P1	#DIV/0!	#DIV/0!	0.6554062	0	0	0	0	2	0
RCN1P2	0.9626168	-0.054966	0.9679285	311	312	340	256	336	335
PDE5A	1.1519435	0.2040699	0.358215	109	66	108	93	123	110
AL163195.2	0.0967742	-3.369234	0.0183101	6	5	20	2	0	1
IER5L	1.4905134	0.5758093	0.0003655	453	587	752	1003	901	767
WIZ	0.8829787	-0.179549	0.3888605	1233	1376	1527	1224	1254	1174
DBF4B	0.9398885	-0.089438	0.8184154	742	839	931	732	834	795
AC239800.3	#DIV/0!	#DIV/0!	0.8030358	0	0	0	1	0	0
AC239800.2	2	1	0.834705	0	0	1	0	2	0
AC239800.1	8	3	0.2032008	1	0	0	7	1	0
PDE6D	0.8569189	-0.222769	0.2670518	740	882	929	705	815	666
PDE6C	0.6666667	-0.584963	0.7635303	3	1	2	0	2	2
PDE6B	3.2	1.6780719	0.3146875	5	0	0	8	0	8
PDE6A	1	0	0.9139773	0	3	4	6	1	0
AC146507.2	2.5	1.3219281	0.6268263	2	0	0	0	1	4
PDE6H	#DIV/0!	#DIV/0!	0.3981469	0	0	0	4	0	0
OR2F1	1.5	0.5849625	0.7839097	1	2	1	0	0	6
PDE6G	0	#NUM!	0.4496863	0	1	3	0	0	0
ZFC3H1	1.2104851	0.2755854	0.1171164	958	482	1116	744	1241	1109
AL049869.2	3	1.5849625	0.6552898	1	0	0	0	2	1
AL049869.1	1.5	0.5849625	0.8653223	2	0	0	0	2	1
AL049869.3	2.8888889	1.5305147	0.262153	6	0	3	0	12	14
PDE7B	0.16	-2.643856	0.0490817	16	6	3	0	2	2
PDE7A	1.0614319	0.0860118	0.3108377	714	642	809	645	839	814
NDST2	0.2222222	-2.169925	0.2635764	4	4	1	0	1	1
NDST1	0.6395744	-0.644816	6.63E-06	2595	3013	3226	1474	2056	2120
NDST4	#DIV/0!	#DIV/0!	0.8030358	0	0	0	1	0	0
NDST3	1	0	0.9681214	7	1	2	0	9	1
AC007240.1	0.9125	-0.132104	0.9982676	16	23	41	39	18	16
AC005083.1	1.1785714	0.2370392	0.7223279	2	11	15	8	3	22
WLS	1.198185	0.2608506	0.0062252	4747	5257	5643	5282	6893	6573
MCEMP1	0.2857143	-1.807355	0.1511953	5	7	9	1	3	2
AC005479.3	2.4	1.2630344	0.208152	3	1	6	10	9	5
AC005479.2	1.35	0.4329594	0.6264307	7	8	5	9	15	3
GATA6	1.0166006	0.023753	0.565138	1148	1384	1504	1233	1576	1294
PDGFB	0.7077922	-0.498602	0.011831	364	314	400	232	277	254

GATA5	#DIV/0!	#DIV/0!	0.4737294	0	0	0	0	4	0
PDGFA	0.964558	-0.05206	0.9333864	1271	1699	1883	1522	1668	1491
GATA4	2.5283019	1.3381687	0.0049807	17	17	19	29	65	40
GATA3	1.4523028	0.5383423	5.13E-08	9843	11202	12479	14812	17518	16357
AC025458.1	1	0	0.9890559	1	0	0	0	0	1
GATA2	1.1825276	0.2418739	0.0114403	10537	12727	13640	13826	15509	14305
GATA1	0	#NUM!	0.6436235	2	0	0	0	0	0
AC133134.1	#DIV/0!	#DIV/0!	0.6099294	0	0	0	2	0	0
PDGFD	0.5279503	-0.921526	0.0449277	61	37	63	23	25	37
PDGFC	1.0115108	0.0165117	0.7205754	504	408	478	372	485	549
OVAAL	0.8571429	-0.222392	0.9470621	1	2	4	2	1	3
AL451063.1	#DIV/0!	#DIV/0!	0.6411933	0	0	0	0	0	2
HNRNPA1L2	1.1295117	0.1756992	0.1147305	706	696	953	916	989	755
LIN54	1.1980882	0.2607341	0.0884757	652	535	696	503	877	876
HNRNPA1P1	0	#NUM!	0.0422365	2	4	6	0	0	0
AC007386.1	0.8461538	-0.241008	0.9231225	2	2	9	0	1	10
AC016885.3	#DIV/0!	#DIV/0!	0.6554062	0	0	0	0	2	0
AC016885.2	0	#NUM!	0.824326	0	0	1	0	0	0
HOXC13	0.8467593	-0.239976	0.2705209	676	695	789	637	572	620
HOXC12	1.25	0.3219281	0.7925376	0	3	5	3	6	1
HOXC11	0	#NUM!	0.6385096	0	2	0	0	0	0
HOXC10	1.203125	0.2667865	0.5204223	8	19	37	30	16	31
INTS6L-AS1	2.6666667	1.4150375	0.4693541	0	3	0	4	1	3
LIN52	1.0634038	0.0886895	0.4581379	281	275	343	244	323	389
GATD1	0.8634067	-0.211888	0.2161528	1670	1682	2190	1482	1705	1598
PHF20L1	1.0855763	0.1184611	0.2755904	507	359	583	478	555	540
AC007000.2	1	0	0.9890559	1	0	0	0	0	1
AC007000.1	1.3333333	0.4150375	0.4444963	19	4	22	22	20	18
TPSD1	0	#NUM!	0.1146274	3	3	2	0	0	0
LIN37	2.0697674	1.0494687	0.0521053	7	18	18	25	33	31
WRB	1.1246819	0.1695171	0.1194205	626	657	682	663	788	759
AC011450.1	0.5535714	-0.853159	0.1268594	46	34	32	18	12	32
WRN	0.9468864	-0.078737	0.8707891	429	236	427	180	418	436
FAM201A	1.1538462	0.2064509	0.8637176	4	9	0	7	3	5
FAM200B	1.1836066	0.2431896	0.1066235	325	224	366	315	410	358
RPL24P8	0.9418605	-0.086415	0.9452447	29	22	35	19	31	31
FAM200A	1.1670429	0.2228576	0.1695397	335	224	327	268	376	390
ACTN4P1	1	0	0.9890559	0	1	0	1	0	0
WT1	0	#NUM!	0.029339	2	8	3	0	0	0
SLMAP	0.9453287	-0.081112	0.8613501	1127	661	1102	656	1033	1043
HNRNPA1P5	#DIV/0!	#DIV/0!	0.3981469	0	0	0	4	0	0
HNRNPA1P4	1.2941176	0.3719688	0.6312395	4	5	8	8	6	8
RPL24P2	2	1	0.7861378	1	0	0	1	1	0
ADAM21P1	#DIV/0!	#DIV/0!	0.8030358	0	0	0	0	1	0
HNRNPA1P7	0.25	-2	0.546683	0	2	2	0	1	0
AC074011.1	1.8461538	0.8845228	0.3751771	3	3	7	5	15	4
AL031775.2	0.7142857	-0.485427	0.7209143	11	3	0	2	2	6
EBAG9	1.1051051	0.1441836	0.2194941	569	492	604	489	734	617
HNRNPA1P6	#DIV/0!	#DIV/0!	0.4909737	0	0	0	2	0	1
RPL24P4	1.0003691	0.0005325	0.5879481	3263	3183	4390	3675	3999	3166
AL031775.1	0.2727273	-1.874469	0.3042433	6	2	3	0	3	0
LIMS2	1.3272727	0.4084648	0.3744696	16	16	23	30	17	26
RPL24P7	3	1.5849625	0.4588249	1	1	0	2	1	3
HNRNPA1P8	0.0952381	-3.392317	0.0679928	3	1	17	0	0	2

LIMS1	0.9747418	-0.036908	0.978761	2969	2218	2850	1914	2963	2957
LINC02321	0.7096774	-0.494765	0.6193495	9	11	11	8	4	10
FAM204A	1.1467204	0.1975136	0.0994943	575	490	673	566	803	624
AC019185.1	1.3333333	0.4150375	0.8501391	3	0	0	2	1	1
AC019185.2	0.4444444	-1.169925	0.58029	7	0	2	3	1	0
LINC02317	1.3333333	0.4150375	0.8390427	0	0	3	0	0	4
LINC02318	#DIV/0!	#DIV/0!	0.8030358	0	0	0	0	0	1
LINC02313	0.5	-1	0.8652929	0	0	2	0	0	1
LINC02315	1.0413223	0.0584167	0.7406196	20	39	62	45	49	32
SPAG11B	0	#NUM!	0.824326	1	0	0	0	0	0
SPAG11A	#DIV/0!	#DIV/0!	0.5509701	0	0	0	0	3	0
NCCRP1	1.0510806	0.0718732	0.5307462	123	166	220	202	176	157
TPST2	1.0691114	0.0964122	0.2596809	646	690	791	730	786	758
TPST1	1.057971	0.0813001	0.5765747	236	300	223	241	280	282
LINC02308	0.3548387	-1.494765	0.0717265	11	15	36	5	14	3
LINC02301	1.4583333	0.5443205	0.2870763	18	10	20	21	23	26
FLJ30679	0.9777778	-0.032421	0.9643185	15	17	13	18	12	14
LINC02341	1	0	0.9931217	5	1	1	1	0	6
LINC02343	#DIV/0!	#DIV/0!	0.6099294	0	0	0	2	0	0
AL353150.1	0.5	-1	0.592685	3	0	9	2	2	2
LINC02340	0.5714286	-0.807355	0.7106522	2	3	2	3	0	1
FAM206A	1.2652317	0.3394016	0.0309462	1017	596	1128	886	1363	1219
LIN7A	1	0	0.9916015	1	0	1	0	1	1
LIN7C	1.0205691	0.0293738	0.6171492	1126	659	1132	832	1097	1048
AC093690.1	0.1666667	-2.584963	0.1215033	5	2	11	2	0	1
LIN7B	1.1467391	0.1975372	0.4127281	57	54	73	83	69	59
TKFC	0.8032708	-0.316042	0.1742993	597	742	740	624	591	455
LINC02338	#DIV/0!	#DIV/0!	0.8030358	0	0	0	0	0	1
LINC02334	0.3333333	-1.584963	0.6638759	0	3	0	0	1	0
LINC02331	0.8518519	-0.231326	0.7870152	6	14	7	1	14	8
AC009053.2	1.3333333	0.4150375	0.7393898	3	0	3	5	2	1
AC009053.1	1.1151515	0.1572397	0.5028398	40	49	76	60	62	62
CTAG2	0.5209302	-0.940838	0.0421674	53	64	98	53	32	27
DDX11-AS1	0.9496403	-0.074547	0.9662049	33	41	65	34	74	24
AC009053.3	0	#NUM!	0.824326	1	0	0	0	0	0
FAM205A	#DIV/0!	#DIV/0!	0.4501902	0	0	0	0	0	4
LINC02328	0.3571429	-1.485427	0.0645078	21	22	13	2	5	13
LINC02327	0.8027523	-0.316973	0.4164233	77	62	79	36	70	69
LINC02324	#DIV/0!	#DIV/0!	0.4121728	0	0	0	0	1	3
LINC02323	0.8823529	-0.180572	0.7291117	48	51	71	39	63	48
LRGUK	#DIV/0!	#DIV/0!	0.5070017	0	0	0	3	0	0
AC003666.1	#DIV/0!	#DIV/0!	0.5509701	0	0	0	0	3	0
LEAP2	1.0588235	0.0824622	0.7767594	24	19	25	24	16	32
FAM208B	0.9866775	-0.01935	0.8894411	3286	1968	3378	1835	3132	3550
FAM208A	0.990735	-0.013429	0.8514882	1194	785	1259	745	1256	1207
TSPAN12	1.1875	0.2479275	0.0293735	681	652	811	691	942	913
TSPAN11	1.1111111	0.1520031	0.9264989	2	4	3	1	8	1
TSPAN10	0.801105	-0.319937	0.6091238	52	52	77	66	27	52
SLC25A24P1	0.525	-0.929611	0.4670552	12	1	27	9	1	11
AC104564.4	2	1	0.6785894	1	1	1	0	6	0
AC104564.3	0.5555556	-0.847997	0.495893	9	4	5	0	8	2
AC137936.2	5	2.3219281	0.2715077	2	0	0	5	5	0
AC104564.2	#DIV/0!	#DIV/0!	0.6554062	0	0	0	0	2	0
AC137936.1	0.4	-1.321928	0.6326277	5	0	0	0	0	2

AC114550.1	1.2777778	0.353637	0.7520534	2	12	4	2	6	15
AC114550.3	0.4	-1.321928	0.62387	0	5	0	0	0	2
AC104564.6	0.1111111	-3.169925	0.1700945	3	2	4	0	0	1
SLC25A24P2	0.25	-2	0.1381057	6	9	5	0	4	1
AL109809.1	#DIV/0!	#DIV/0!	0.8030358	0	0	0	0	1	0
APAF1	0.8940663	-0.161546	0.5203689	1046	778	923	469	925	1062
FAM207A	1.1574631	0.2109662	0.1100095	912	1341	1405	1566	1442	1226
TTC39A-AS1	0	#NUM!	0.6436235	2	0	0	0	0	0
HAND1	1.3579677	0.4414491	0.0306174	141	150	142	206	204	178
TPSP2	0.5195531	-0.944657	0.0276027	62	64	53	34	36	23
AC093700.1	0	#NUM!	0.6631647	1	0	1	0	0	0
TSPAN33	0.8604651	-0.216811	0.9151788	12	9	22	21	11	5
TSPAN32	0.5	-1	0.6523927	2	4	0	0	0	3
TSPAN31	0.8354244	-0.259419	0.2324198	386	433	536	356	396	380
AC105393.2	#DIV/0!	#DIV/0!	0.5371397	0	0	0	0	0	3
'MPRSS11BN	0	#NUM!	0.6840298	0	0	2	0	0	0
AC115285.1	0	#NUM!	0.824326	0	0	1	0	0	0
MID1IP1	1.1800819	0.2388869	0.0297589	1501	1787	1843	2024	2163	1868
KIAA1549L	0.6538462	-0.612977	0.4293226	13	15	24	12	11	11
SYT16	1.4537037	0.5397332	0.181892	38	35	35	28	64	65
SYT15	0.8860759	-0.174498	0.8674916	15	34	30	31	21	18
FAM209B	2	1	0.8118689	0	0	1	0	1	1
SYT14	#DIV/0!	#DIV/0!	0.1541435	0	0	0	0	5	3
SUGCT	1.1261261	0.1713684	0.3574292	95	83	155	134	101	140
SYT17	0.730608	-0.452831	0.0402074	284	311	359	238	244	215
TSPAN16	2	1	0.7415925	0	3	0	0	6	0
TSPAN15	1.0571429	0.0801703	0.3799097	590	674	696	716	742	614
TSPAN14	1.3089784	0.3884413	0.0004203	1762	2089	2130	2409	2741	2679
TSPAN13	0.4424227	-1.176503	5.19E-16	1265	1134	1448	535	645	522
SYT12	0.7946429	-0.331621	0.5290392	32	42	38	29	31	29
SYT11	#DIV/0!	#DIV/0!	0.5371397	0	0	0	0	0	3
TSPAN19	0	#NUM!	0.824326	1	0	0	0	0	0
TSPAN18	1.8627451	0.8974303	0.066941	13	17	21	26	39	30
TSPAN17	0.7627308	-0.390754	0.0152684	1050	1246	1278	860	981	885
PIK3AP1	2.2962963	1.1993088	0.0086722	21	36	24	29	75	82
SMARCAL1	0.9724991	-0.040231	0.8757923	869	940	1100	886	992	951
LINC02289	1.2571429	0.3301486	0.4168107	23	20	27	35	21	32
LINC02288	1.6	0.6780719	0.6412184	2	0	3	3	1	4
LINC02281	0	#NUM!	0.824326	0	0	1	0	0	0
LINC02280	1.4545455	0.5405684	0.6480627	2	4	5	2	11	3
LINC02274	0.5	-1	0.7778439	0	0	4	0	2	0
LINC02273	1.2045455	0.2684888	0.5520459	9	10	25	26	12	15
BCORL1	1.1900973	0.2510795	0.0454898	650	746	866	705	940	1047
LINC02268	0.5333333	-0.906891	0.5321554	7	2	6	4	3	1
LINC02298	0.6888889	-0.537657	0.5639333	7	25	13	16	5	10
LINC02297	1	0	0.9890559	0	0	1	1	0	0
LINC02293	#DIV/0!	#DIV/0!	0.0412138	0	0	0	7	1	3
CTB-178M22.2	2.5	1.3219281	0.3177743	1	3	2	3	3	9
LINC02290	0	#NUM!	0.0155637	0	7	12	0	0	0
OSGIN1	0.3843931	-1.379346	1.09E-09	405	486	493	205	145	182
OSGIN2	1.039897	0.0564407	0.537127	574	440	540	443	627	546
EIF1AX-AS1	0.4	-1.321928	0.6282732	3	0	2	0	0	2
MRAP2	#DIV/0!	#DIV/0!	0.4501902	0	0	0	0	0	4
SUGP2	0.8076211	-0.30825	0.0275541	2366	2273	2919	1839	2174	2091

SUGP1	0.9575236	-0.06262	0.9528669	1059	1281	1262	1110	1248	1091
LINC02243	#DIV/0!	#DIV/0!	0.4501902	0	0	0	0	0	4
LINC02241	1.3333333	0.4150375	0.8938282	0	3	0	1	3	0
B3GNTL1	0.8239757	-0.279326	0.2770299	214	198	247	171	197	175
SVIL-AS1	2.3932584	1.2589762	6.23E-06	50	61	67	100	158	168
LINC02231	0	#NUM!	0.824326	0	0	1	0	0	0
LINC02234	0	#NUM!	0.3626892	1	0	4	0	0	0
ATMIN	0.9564541	-0.064232	0.9033038	4827	4564	5398	3996	5164	4985
RNF157-AS1	0.375	-1.415037	0.380399	2	12	10	9	0	0
SNX18P14	0	#NUM!	0.6436235	2	0	0	0	0	0
SNX18P13	#DIV/0!	#DIV/0!	0.4501902	0	0	0	0	0	4
SNX18P12	1.2307692	0.2995603	0.6941432	2	3	8	10	1	5
LINC02228	0.7777778	-0.36257	0.6939263	8	11	8	4	11	6
AC064799.1	0.9399142	-0.089399	0.9350286	151	118	197	133	152	153
LINC02225	1.5	0.5849625	0.7254754	2	1	1	4	2	0
LINC02265	0	#NUM!	0.824326	0	0	1	0	0	0
AC079834.2	0.1875	-2.415037	0.0509999	6	8	18	1	5	0
LINC02267	1.064257	0.0898466	0.6473512	80	75	94	92	118	55
AC079834.1	#DIV/0!	#DIV/0!	0.6099294	0	0	0	2	0	0
LINC02266	1.3292683	0.4106323	0.3054094	28	19	35	33	46	30
AC005822.1	0.5	-1	0.7231878	2	0	2	0	2	0
SNX18P1Y	5	2.3219281	0.4840007	1	0	0	0	5	0
PDZD11	0.8788014	-0.186391	0.3517278	1352	1515	1605	1302	1443	1185
XDH	0.8327759	-0.264	0.4768282	120	96	83	76	73	100
LINC02254	0.3181818	-1.652077	0.1932791	4	9	9	0	3	4
LINC02253	1.164751	0.2200215	0.326933	55	87	119	105	104	95
LINC02256	0	#NUM!	0.2219629	0	7	0	0	0	0
LINC02255	2	1	0.3966953	1	4	5	2	13	5
AL158201.1	5.5	2.4594316	0.2082614	1	1	0	4	0	7
KITLG	0.5478723	-0.868088	0.0345526	62	48	78	32	38	33
HNRNPA2B1	1.0727288	0.1012853	0.1404807	39846	33936	47628	39299	48613	42328
AGGF1	1.1410346	0.1903425	0.1227595	1588	1003	1720	1421	1933	1565
LINC02247	#DIV/0!	#DIV/0!	0.6099294	0	0	0	2	0	0
LINC02246	2.5	1.3219281	0.0664442	9	9	6	9	25	26
LINC02249	0	#NUM!	0.6840298	0	0	2	0	0	0
AC018690.1	1.2678571	0.3423922	0.3460037	37	21	54	32	57	53
SEMA5A-AS1	1.4	0.4854268	0.7829253	0	1	4	0	5	2
GEN1	0.9721448	-0.040757	0.9228954	1079	584	1209	714	1106	972
TPTE2	#DIV/0!	#DIV/0!	0.6554062	0	0	0	0	2	0
AL132780.1	1.2285714	0.2969817	0.593739	8	4	23	11	11	21
AL132780.2	0.6241611	-0.68001	0.1443196	49	34	66	23	36	34
AL132780.5	0.4516129	-1.146841	0.1434808	33	14	15	3	6	19
AL132780.4	0.7179487	-0.478047	0.6077365	14	7	18	10	11	7
AL137779.2	3.2105263	1.6828098	0.0185419	4	7	8	32	11	18
CLRN1-AS1	1.09375	0.129283	0.8264949	10	6	16	7	22	6
CLRN1-AS1	1.09375	0.129283	0.8264949	10	6	16	7	22	6
AC002347.2	4	2	0.5760976	1	0	0	0	4	0
CACNA1B	0.0434783	-4.523562	0.0116736	16	3	4	0	1	0
CACNA1A	0.6	-0.736966	0.6101891	3	3	9	2	3	4
CACNA1D	0.9344978	-0.097737	0.9166061	58	84	87	70	67	77
CACNA1C	0.3728814	-1.423211	0.0625821	27	10	22	6	7	9
CACNA1F	1.8333333	0.8744691	0.3611393	4	4	4	11	7	4
HOXB13	0.7941176	-0.332575	0.4331894	54	90	94	37	62	90
CACNA1E	3.3333333	1.7369656	0.4355308	3	0	0	0	8	2

CACNA1H	1	0	0.9163502	12	37	25	25	16	33
CACNA1G	2.4444444	1.2895066	0.2524989	0	4	5	6	5	11
potential;GO:0	#VALUE!	#VALUE!	mbrane;GO:	3|hsa04925|h	synthesis	ated channel subunit alpha1 G			
CACNA1I	1.3868421	0.4718035	0.0039887	214	240	306	343	397	314
AC009303.2	#DIV/0!	#DIV/0!	0.8030358	0	0	0	0	0	1
AC009303.1	0	#NUM!	0.3626892	1	0	4	0	0	0
CACNA1S	0.8717949	-0.197939	0.8418204	14	14	11	11	7	16
AC009303.4	1.1666667	0.2223924	0.771787	4	10	10	4	11	13
AC005332.7	0.724026	-0.465887	0.2402208	94	65	149	75	84	64
PDHA1	0.9717053	-0.041409	0.8520732	3852	4295	4965	4140	4457	4144
AC015911.1	#DIV/0!	#DIV/0!	0.5371397	0	0	0	0	0	3
AC005332.1	2.5	1.3219281	0.2583229	0	2	6	5	8	7
AC005332.3	0.68	-0.556393	0.5051149	16	11	23	2	26	6
AC015911.4	4.5	2.169925	0.2775212	2	0	0	4	1	4
AC005332.5	1.6666667	0.7369656	0.7265171	0	0	3	0	1	4
AC015911.3	#DIV/0!	#DIV/0!	0.4737294	0	0	0	0	4	0
MANSC4	0.1666667	-2.584963	0.3678402	0	3	3	0	1	0
RNY1P4	0	#NUM!	0.824326	0	0	1	0	0	0
MANSC1	0.7573314	-0.401003	0.0386205	421	389	554	303	376	354
RNY1P9	0	#NUM!	0.3451105	2	0	3	0	0	0
PHTF2	0.9637757	-0.053231	0.9457946	1082	795	994	731	1075	961
ZSCAN12P1	0.8348348	-0.260437	0.5151775	92	84	157	77	113	88
KCNAB1-AS1	0	#NUM!	0.824326	1	0	0	0	0	0
AP002340.1	0.2	-2.321928	0.528477	0	0	5	0	1	0
AC073912.2	1	0	0.9781918	1	0	3	0	3	1
AC073912.1	0.8333333	-0.263034	0.9650222	0	0	6	0	1	4
PHTF1	0.7450199	-0.424649	0.1418979	155	146	201	120	111	143
GET4	1.2093023	0.274175	0.1569692	106	129	152	142	153	173
SLC12A5-AS1	0.6470588	-0.628031	0.6568928	10	3	4	7	2	2
NAMPTP1	1.2247899	0.2925343	0.1751488	164	139	173	124	220	239
AC138035.1	0.5605787	-0.835011	0.0019117	184	165	204	68	112	130
AC138035.3	0.5555556	-0.847997	0.4073992	6	11	10	1	6	8
AC138035.2	#DIV/0!	#DIV/0!	0.3096126	0	0	0	5	0	0
AP001992.1	0.6666667	-0.584963	0.8484366	0	2	1	0	0	2
SETBP1	0.7651297	-0.386224	0.1303972	260	211	223	117	209	205
AC068134.3	0.7142857	-0.485427	0.7662411	2	5	0	1	3	1
AC068134.2	0	#NUM!	0.6385096	0	2	0	0	0	0
KCNMB3P1	0.75	-0.415037	0.8256736	5	0	3	0	6	0
AC096582.1	0	#NUM!	0.824326	0	1	0	0	0	0
AC096582.3	#DIV/0!	#DIV/0!	0.8030358	0	0	0	0	1	0
ZNF804B	#DIV/0!	#DIV/0!	0.6099294	0	0	0	2	0	0
ZNF804A	#DIV/0!	#DIV/0!	0.6554062	0	0	0	0	2	0
NRSN2-AS1	1.0515873	0.0725686	0.6669847	67	88	97	76	105	84
AC145422.1	0.6363636	-0.652077	0.4933056	13	9	11	9	9	3
AC013264.1	0	#NUM!	0.824326	1	0	0	0	0	0
KRAS	1.0180477	0.0258051	0.6160464	2774	2088	2729	1991	2906	2831
SUGT1	1.2143435	0.2801766	0.0103258	2473	2021	2924	2922	3421	2665
AC078950.1	0	#NUM!	0.6604275	0	1	1	0	0	0
GTPBP6	0.9861573	-0.02011	0.7383193	993	1200	1419	1231	1253	1078
GTPBP8	1.0317848	0.0451422	0.6500205	259	256	303	203	336	305
SCRIB	0.8663983	-0.206898	0.2591935	2290	2608	2804	2225	2363	2085
GTPBP2	0.9100557	-0.135973	0.4837616	1738	1881	2129	1511	1897	1823
GTPBP1	0.9246853	-0.112966	0.6675699	1370	1540	1777	1307	1490	1537
GTPBP4	1.0036319	0.0052302	0.6440059	3676	3217	4396	2991	4435	3904

GTPBP3	0.8995911	-0.152659	0.5043304	1252	1435	1715	1276	1428	1256
RPL23P2	1.15	0.2016339	0.7704114	12	0	8	12	5	6
AC009542.2	0.3333333	-1.584963	0.2473844	5	13	3	0	7	0
RPL23P8	0.4166667	-1.263034	0.2642981	5	11	8	4	2	4
CTBP2	1.2275832	0.2958208	0.0045014	1422	1461	1656	1630	2161	1781
CTBP1	0.9468119	-0.07885	0.9456644	914	1023	1184	1016	954	985
XPA	0.9524362	-0.070306	0.9212394	289	261	312	247	330	244
XPC	0.7286739	-0.456655	0.0018506	1218	1109	1436	818	950	974
AC073911.3	#DIV/0!	#DIV/0!	0.6317974	0	0	0	1	1	0
AC073911.2	0	#NUM!	0.824326	1	0	0	0	0	0
AC073911.1	3	1.5849625	0.6253252	0	0	1	3	0	0
PCDHA1	0.4986979	-1.003762	1.46E-05	230	266	272	102	129	152
ZNF812P	#DIV/0!	#DIV/0!	0.6411933	0	0	0	0	0	2
PCDHA5	0.6269663	-0.67354	0.0063112	316	281	293	121	189	248
AC016394.1	0.7073171	-0.499571	0.5443095	21	10	10	9	8	12
AC093311.1	0	#NUM!	0.824326	0	1	0	0	0	0
GSG1L	0	#NUM!	0.824326	0	1	0	0	0	0
PCDHA4	0.8064516	-0.31034	0.1871256	288	341	332	262	277	236
PCDHA3	0.8617284	-0.214695	0.3445039	406	345	464	290	423	334
AC005082.1	2	1	0.7049414	0	0	2	0	0	4
PCDHA2	0.7477876	-0.4193	0.2990658	86	63	77	50	45	74
PCDHA9	1.2641509	0.3381687	0.5624755	16	18	19	8	27	32
PCDHA8	0.7	-0.514573	0.4243631	33	18	19	12	14	23
PCDHA7	0.7308642	-0.452325	0.0619851	213	255	342	156	245	191
PCDHA6	0.7616761	-0.39275	0.020382	769	751	771	465	649	631
AC027124.1	#DIV/0!	#DIV/0!	0.4501902	0	0	0	0	0	4
TPRG1	1.0923077	0.1273793	0.6338615	46	34	50	43	58	41
SETD1B	0.7597974	-0.396313	0.0157314	1356	1567	1619	920	1178	1353
AC126118.1	0.875	-0.192645	0.9088498	6	7	3	8	5	1
SETD1A	0.8794654	-0.185301	0.3304174	1255	1287	1573	1118	1211	1290
AC016644.1	0.7142857	-0.485427	0.8757718	0	0	7	0	5	0
AC084782.2	#DIV/0!	#DIV/0!	0.8030358	0	0	0	0	0	1
AC084782.3	0	#NUM!	0.824326	1	0	0	0	0	0
PCDHB2	0.741476	-0.431528	0.0115776	676	810	831	460	646	612
AC007637.1	0.7671233	-0.38247	0.5620149	28	27	18	21	13	22
PCDHB1	0.7857143	-0.347923	0.7955819	3	5	6	1	5	5
PCDHB6	0.5301587	-0.915504	0.0046739	93	119	103	48	58	61
AC093607.1	0	#NUM!	0.824326	0	1	0	0	0	0
PCDHB5	0.9677419	-0.047306	0.9266299	8	18	5	4	16	10
MMS19	1.1303351	0.1767506	0.0493459	2107	2348	2527	2438	2834	2620
PCDHB4	1.020202	0.0288549	0.8111841	34	20	45	34	37	30
PCDHB3	0.6275862	-0.672114	0.1386212	57	40	48	30	32	29
PCDHB9	0.6610169	-0.597241	0.1340429	85	65	86	24	62	70
PCDHB8	0.5530493	-0.85452	6.37E-06	359	380	458	180	243	239
PCDHB7	0.5013405	-0.996137	0.0011638	97	128	148	57	65	65
AL353151.1	#DIV/0!	#DIV/0!	0.0928943	0	0	0	0	5	5
KRI1	0.9126606	-0.13185	0.604194	888	877	960	821	837	829
IFIT1P1	2.75	1.4594316	0.2020362	1	0	7	7	6	9
BRD7P4	0	#NUM!	0.5337954	0	3	0	0	0	0
BRD7P2	1.1275168	0.1731489	0.5388868	49	38	62	40	69	59
BRD7P5	0.5	-1	0.8002255	1	1	0	0	1	0
FAM210B	0.8691317	-0.202353	0.1933657	2328	2385	2508	1847	2327	2102
FAM210A	0.8859951	-0.174629	0.4428706	719	573	743	446	645	712
AC007387.1	1	0	0.9860002	3	9	2	2	5	7

FMR1-IT1	0.7692308	-0.378512	0.6711472	18	6	15	7	11	12
AC023794.1	1.25	0.3219281	0.8119057	2	0	2	5	0	0
AC112504.1	1	0	0.9890559	1	0	0	0	1	0
PSPC1P1	1.3478261	0.4306344	0.5664419	13	2	8	15	10	6
FAM214B	1.1409496	0.190235	0.2777353	171	263	240	214	299	256
AC023794.5	0.1818182	-2.459432	0.2006774	6	1	4	0	1	1
FAM214A	1.5026643	0.5875227	0.0017998	423	247	456	410	644	638
AC023794.4	1.0909091	0.1255309	0.9514149	7	3	1	0	0	12
AC023794.3	#DIV/0!	#DIV/0!	0.4737294	0	0	0	0	4	0
FAM213B	1.3916084	0.4767533	0.1343482	41	45	57	88	55	56
FAM213A	1.1941448	0.2559778	0.0237428	588	607	752	757	783	785
AL669970.3	3.5	1.8073549	0.5234342	2	0	0	0	7	0
AL669970.2	0	#NUM!	0.824326	1	0	0	0	0	0
AC092139.3	0	#NUM!	0.1312466	4	0	5	0	0	0
FAM212B	0.3125	-1.678072	0.28211	5	4	7	0	5	0
AC092139.2	1	0	0.9890559	1	0	0	0	0	1
FAM212A	#DIV/0!	#DIV/0!	0.5509701	0	0	0	0	3	0
AC004889.1	0.8947368	-0.160465	0.813273	11	18	9	1	24	9
SCRG1	1.3333333	0.4150375	0.8390427	0	0	3	0	0	4
LINC02201	2.037037	1.0264722	0.1694945	11	13	3	14	23	18
AC139749.1	0.5555556	-0.847997	0.6799543	0	4	5	2	3	0
LINC02200	#DIV/0!	#DIV/0!	0.5070017	0	0	0	3	0	0
TXNL4B	0.886031	-0.174571	0.4211678	652	737	866	616	702	680
PLXND1	1.1035857	0.1421986	0.1002243	3989	4716	5100	4390	5380	5465
AC004253.1	1.2	0.2630344	0.6651369	14	6	10	14	12	10
FAM216A	1.8849558	0.9145307	0.0008309	60	93	73	154	137	135
AC012184.3	0.6742857	-0.568568	0.213594	38	53	84	35	46	37
PHYHD1	0.8218391	-0.283072	0.2742598	230	205	261	188	198	186
PLXNC1	0.875	-0.192645	0.863452	25	4	19	12	14	16
TXNL4A	1.0259186	0.0369163	0.4952315	917	984	1147	1168	1057	902
TLE4	1.0826162	0.1145219	0.2865537	1211	1359	1497	1088	1554	1761
TLE3	1.0184793	0.0264166	0.4848926	5462	6284	6653	5597	6634	6508
TLE2	1.2795031	0.3555836	0.0657609	159	152	172	233	208	177
TLE1	1.1938823	0.2556606	0.0044755	3282	3505	3936	3887	4626	4289
HARBI1	0.7823741	-0.354069	0.1832289	164	191	201	100	173	162
SPICE1	0.9873204	-0.01841	0.8949543	418	280	485	248	478	442
FAM215B	2.5	1.3219281	0.5466929	0	1	1	1	1	3
TLE7	0.5	-1	0.7021691	0	3	1	0	2	0
TLE6	1.1986607	0.2614234	0.0683535	300	271	325	334	407	333
LINC02223	0.625	-0.678072	0.3421615	11	31	14	9	13	13
FAM218A	0.9411765	-0.087463	0.9715084	16	5	30	15	15	18
MRPS17P1	0.5	-1	0.6763245	3	0	3	0	3	0
ARF4P2	1	0	0.8999148	1	7	5	12	0	1
AC007731.2	0.4375	-1.192645	0.0765958	21	24	19	8	11	9
SCRT2	0	#NUM!	0.6385096	0	2	0	0	0	0
ALOX12P2	0.4583333	-1.125531	0.3302651	3	8	13	2	5	4
AC019186.1	1.8333333	0.8744691	0.548878	2	0	4	2	7	2
ALOX12P1	0.5	-1	0.8652929	0	0	2	0	0	1
PLXNA2	0.7608364	-0.394342	0.0162932	3741	4875	4488	2537	3637	3796
LINC02217	#DIV/0!	#DIV/0!	0.058607	0	0	0	7	4	0
PLXNA1	0.8245468	-0.278327	0.0708227	2925	3424	3249	2186	2910	2818
LINC02219	1.4	0.4854268	0.832591	5	0	0	2	5	0
PLXNA4	0	#NUM!	0.824326	1	0	0	0	0	0
PLXNA3	1.0687603	0.0959384	0.2293432	940	917	1168	1010	1053	1170

LINC02210	0.9409142	-0.087865	0.9459937	267	284	346	317	289	238
LINC02212	1.025	0.0356239	0.8828731	12	7	21	7	13	21
SEC16A	0.9358928	-0.095585	0.7041784	3255	3844	4023	2676	3756	3977
FAM217A	0.2222222	-2.169925	0.3042824	6	3	0	1	0	1
SCRT1	3	1.5849625	0.3230278	0	0	4	2	3	7
FAM217B	0.9534706	-0.06874	0.8640747	945	763	914	583	892	1025
GFAP	0.6666667	-0.584963	0.8115277	2	0	4	0	4	0
LRFN3	0.6636771	-0.591447	0.1822209	35	97	91	39	65	44
LRFN2	0	#NUM!	0.3320541	3	0	2	0	0	0
LRFN5	0	#NUM!	0.824326	1	0	0	0	0	0
LRFN4	1.2307692	0.2995603	0.6451302	10	4	12	9	8	15
LRFN1	1.0359771	0.0509921	0.5328313	359	414	450	503	418	346
PLXNB3	0.4285714	-1.222392	0.5798715	3	1	3	0	0	3
PLXNB2	0.7847568	-0.349682	0.0169665	12710	16190	16235	10402	12460	12558
PLXNB1	0.8521333	-0.230849	0.1853743	1538	1675	1920	1202	1477	1695
LINC02208	2	1	0.5374161	0	2	2	3	2	3
AC114947.1	0.3333333	-1.584963	0.6715904	3	0	0	0	1	0
AC114947.2	0.3928571	-1.347923	0.2222948	19	3	6	2	7	2
LINC02204	0	#NUM!	0.5172237	1	2	0	0	0	0
SRRM1P3	1	0	0.9890559	1	0	0	0	0	1
CRTC3-AS1	0.3555556	-1.491853	0.0885197	18	12	15	7	7	2
PIK3C2G	1.8604651	0.8956633	0.0085948	50	27	52	62	101	77
PIK3C2A	1.155096	0.2080128	0.2308042	1700	805	1557	1082	1859	1751
PIK3C2B	0.8105409	-0.303043	0.0524916	4047	4753	4918	2873	4011	4235
AP001020.3	0.5	-1	0.6906809	3	1	0	0	2	0
SCRN1	0.8125	-0.29956	0.6783379	29	22	13	17	10	25
AL390835.1	3	1.5849625	0.4545911	0	0	2	4	0	2
AP001020.2	#DIV/0!	#DIV/0!	0.4909737	0	0	0	2	0	1
SCRN3	1.1279887	0.1737527	0.190495	507	378	537	417	637	550
SCRN2	1.2151601	0.2811464	0.0431647	581	718	825	988	821	772
TLK2	1.0435559	0.0615078	0.3741836	920	803	1078	825	1049	1049
TLK1	1.1782451	0.2366397	0.0793759	3090	1898	3001	2418	3604	3391
AL663058.1	0	#NUM!	0.5337954	0	3	0	0	0	0
FAM219B	0.8948787	-0.160236	0.6535178	117	126	128	79	117	136
FAM219A	1.1981707	0.2608335	0.018841	671	723	902	870	918	963
AC025219.1	1.2272727	0.2954559	0.7112921	6	6	10	3	7	17
AL451062.1	0	#NUM!	0.2605273	0	3	3	0	0	0
ALMS1-IT1	1.0493274	0.0694648	0.644475	71	59	93	83	85	66
POTEH-AS1	2.6666667	1.4150375	0.5056781	3	0	0	4	4	0
AL031284.1	0.5555556	-0.847997	0.6010067	5	3	1	0	2	3
AC104563.1	0.941704	-0.086654	0.892779	126	164	156	138	162	120
P2RY6	0.8802817	-0.183963	0.549782	191	247	272	198	217	210
P2RY4	0	#NUM!	0.5781912	0	0	3	0	0	0
P2RY2	1.1363636	0.1844246	0.6557403	18	21	27	32	5	38
P2RY1	0.3568905	-1.486447	0.0003593	92	78	113	19	25	57
AP000432.1	1	0	0.9720437	4	2	0	4	0	2
SEC11B	0.3333333	-1.584963	0.4735788	2	3	1	1	1	0
SEC11A	0.9995601	-0.000635	0.6124229	4636	5320	5956	5088	5718	5099
SEC11C	0.944186	-0.082857	0.9304931	270	280	310	283	250	279
KRR1	1.1220703	0.1661631	0.2572771	787	439	822	579	865	854
TPRKB	1.1530516	0.2054571	0.0802862	1088	810	1297	1321	1233	1130
GFER	0.8676399	-0.204832	0.4225592	595	726	734	649	627	507
P2RX7	0.8571429	-0.222392	0.8481339	12	3	6	4	7	7
P2RX6	0.5391705	-0.891187	0.0187483	70	77	70	26	50	41

P2RX5	#DIV/0!	#DIV/0!	0.0175471	0	0	0	11	5	0
P2RX4	0.858172	-0.220661	0.3094515	636	802	783	616	739	551
P2RX3	4	2	0.5246085	1	0	0	0	1	3
P2RX2	0.2682927	-1.89812	0.0428909	18	14	9	4	2	5
P2RX1	0	#NUM!	0.824326	1	0	0	0	0	0
TLL2	1.2380952	0.3081223	0.6758907	0	8	13	12	9	5
TLL1	#DIV/0!	#DIV/0!	0.3334379	0	0	0	0	4	1
LINC02166	1	0	0.9757152	1	9	5	3	3	9
LINC02168	0	#NUM!	0.824326	1	0	0	0	0	0
OLA2-SMG1F	1.5	0.5849625	0.6570334	0	0	6	6	0	3
GFI1	0.2307692	-2.115477	0.05189	30	1	21	7	2	3
LINC02163	0.9390244	-0.090765	0.8906744	89	74	83	72	93	66
AC005821.1	4	2	0.1663717	0	0	4	10	3	3
LINC02160	1.25	0.3219281	0.9327823	2	2	0	0	5	0
TPRXL	0.8422392	-0.247698	0.4891104	94	128	171	81	126	124
AC115284.2	2	1	0.8324799	1	0	0	0	2	0
LINC02159	0.8367347	-0.257158	0.7441302	11	22	16	12	15	14
LINC02158	#DIV/0!	#DIV/0!	0.1241408	0	0	0	6	2	0
IL15RA	1.2432432	0.3141086	0.188312	60	92	107	114	102	106
LINC02157	0	#NUM!	0.5781912	0	0	3	0	0	0
KRT2	#DIV/0!	#DIV/0!	0.3096126	0	0	0	5	0	0
KRT8	1.0118245	0.016959	0.5390009	111977	139513	150135	142436	140777	123161
FLT3LG	2.1666667	1.1154772	0.501115	6	0	0	9	0	4
KRT7	0.6888889	-0.537657	0.000996	3896	4679	5330	3396	3327	2856
KRT5	0	#NUM!	0.1996036	0	0	9	0	0	0
KRT9	0.9677419	-0.047306	0.9626865	20	30	12	14	11	35
AC092868.3	0	#NUM!	0.824326	1	0	0	0	0	0
AC145423.2	0.75	-0.415037	0.8968527	3	0	5	4	2	0
AC145423.3	0.9130435	-0.131245	0.8515213	49	27	39	28	35	42
TLN2	1.0539789	0.0758459	0.4291372	465	635	697	559	741	594
TLN1	1.0889194	0.1228972	0.2583197	10303	13619	12493	10447	13857	15349
LINC02188	1	0	0.9890559	0	1	0	0	1	0
LINC02185	0.125	-3	0.2625623	3	0	5	0	1	0
LINC02182	0	#NUM!	0.1075214	5	4	0	0	0	0
LINC02181	0	#NUM!	0.6385096	0	2	0	0	0	0
HTATSF1	1.1192857	0.1625784	0.0864185	4383	3208	5009	4490	5063	4550
PPATP1	1.1428571	0.1926451	0.94004	3	4	0	1	5	2
SETDB1	0.7578826	-0.399954	0.0111882	1015	1144	1298	695	980	945
LINC02172	0	#NUM!	0.824326	1	0	0	0	0	0
LINC02175	0.7333333	-0.447459	0.5401846	16	17	12	6	14	13
SETDB2	1.0718133	0.1000536	0.5075683	224	129	204	173	203	221
LINC02170	0.75	-0.415037	0.6384145	36	17	43	43	16	13
FTH1P11	1.3529412	0.4360991	0.5759315	2	4	11	9	8	6
GFM1	0.8585627	-0.220005	0.2278052	1339	1113	1472	844	1322	1203
FTH1P15	1	0	0.9313564	1	0	3	2	0	2
GFM2	1.0212895	0.0303919	0.5731522	1110	1026	1152	907	1240	1211
LINC02120	#DIV/0!	#DIV/0!	0.237847	0	0	0	1	0	5
FTH1P16	0.75	-0.415037	0.7108891	7	7	2	2	5	5
LIMD1	0.6548303	-0.610807	0.000168	1838	1928	1979	884	1418	1460
LIMD2	1.3333333	0.4150375	0.4370972	21	15	18	31	32	9
SH3PXD2A	0.9780616	-0.032003	0.9482424	1149	1241	1211	936	1235	1351
SH3PXD2B	0.7948589	-0.331229	0.0362956	8975	10505	10280	6033	8320	9302
AP005230.1	2.0714286	1.0506261	0.2527048	2	2	10	6	8	15
LINC02116	0	#NUM!	0.6385096	0	2	0	0	0	0

LINC02111	0	#NUM!	0.3451105	2	0	3	0	0	0
PSENEN	1.1348315	0.1824781	0.6064898	27	26	36	46	41	14
OR4D1	0	#NUM!	0.824326	0	0	1	0	0	0
FTH1P23	0.5434783	-0.879706	0.3562897	4	21	21	10	13	2
IZUMO4	0.4193548	-1.253757	0.1976336	10	16	5	4	4	5
LINC02112	#DIV/0!	#DIV/0!	0.6554062	0	0	0	0	2	0
IZUMO1	1.875	0.9068906	0.4809097	3	3	2	3	1	11
LIME1	0.9918033	-0.011874	0.8988643	35	34	53	42	58	21
FTH1P20	0.3333333	-1.584963	0.2603142	5	9	4	3	0	3
TLR1	0.5714286	-0.807355	0.3764979	9	17	16	1	6	17
LINC02108	0.1666667	-2.584963	0.3723105	3	0	3	0	1	0
TLR9	0	#NUM!	0.824326	0	1	0	0	0	0
TLR7	1.6402878	0.7139489	0.0336188	55	45	39	61	79	88
LINC02109	0.5	-1	0.3442137	9	11	8	0	11	3
TLR6	2.1666667	1.1154772	0.4528522	2	0	4	1	1	11
LINC02104	#DIV/0!	#DIV/0!	0.4098001	0	0	0	1	3	0
TLR5	0.5324675	-0.909235	0.1951974	28	13	36	16	18	7
LINC02103	0	#NUM!	0.6410557	1	1	0	0	0	0
TLR4	#DIV/0!	#DIV/0!	0.8030358	0	0	0	0	0	1
VGLL1	1.2444323	0.3154878	0.0021922	3313	3597	4091	4553	4950	4187
TLR3	0.9889299	-0.01606	0.9559143	215	126	201	104	247	185
VGLL4	0.8948805	-0.160233	0.3767876	1800	2007	2053	1423	1890	1931
LINC02105	1	0	0.9989755	2	4	0	3	3	0
VGLL3	1.2297214	0.2983315	0.1004484	3277	1915	2883	2007	3823	4100
AC010132.4	0	#NUM!	0.6436235	2	0	0	0	0	0
AC113139.1	0	#NUM!	0.3451105	2	0	3	0	0	0
AC010132.3	0	#NUM!	0.199693	0	4	3	0	0	0
LINC02145	0.5	-1	0.7645326	4	0	0	2	0	0
SIAH2-AS1	1	0	0.9672374	2	1	0	0	3	0
PHYHIP	0.296875	-1.752072	0.0434252	19	19	26	5	14	0
LIMA1	1.0664852	0.092864	0.3259288	5677	3964	5791	4236	6089	6133
IPO5P1	1.1081505	0.1481538	0.2770028	196	188	254	245	234	228
APBB2	0.9249752	-0.112513	0.6223341	2091	1814	2133	1360	2050	2175
AC069213.3	#DIV/0!	#DIV/0!	0.8030358	0	0	0	1	0	0
APBB1	#DIV/0!	#DIV/0!	0.4980481	0	0	0	2	1	0
APBB3	0.91625	-0.126187	0.8503444	234	218	348	271	244	218
LINC02133	1.8125	0.857981	0.1067856	12	16	20	17	30	40
LINC02132	0	#NUM!	0.6840298	0	0	2	0	0	0
SORBS2	2.5440613	1.3471334	6.60E-09	97	66	98	179	244	241
SORBS1	0.7543353	-0.406722	0.0840821	240	214	238	156	179	187
SORBS3	0.8275862	-0.273018	0.3583454	404	493	582	495	400	329
ENO1-AS1	0.125	-3	0.2231104	4	4	0	0	0	1
APBA1	0.885	-0.176251	0.7128042	65	56	79	39	64	74
APBA3	0.914446	-0.12903	0.7612169	235	194	284	213	230	209
APBA2	0.4285714	-1.222392	0.4417995	3	6	12	8	1	0
LINC02127	0	#NUM!	0.824326	0	1	0	0	0	0
AC112503.2	0.3333333	-1.584963	0.669913	3	0	0	0	0	1
TPSB2	#DIV/0!	#DIV/0!	0.4242335	0	0	0	0	3	1
HOXA13	0.6428571	-0.63743	0.6162245	7	5	2	2	6	1
HCFC1R1	1.6122715	0.6890947	0.0008355	221	258	287	522	404	309
COTL1	0.8102454	-0.303569	0.0876757	10004	12419	13476	10210	9905	8972
MIGA2	0.9778737	-0.03228	0.7989193	551	574	728	659	615	538
MIGA1	0.9306883	-0.10363	0.6966257	829	639	624	447	701	799
ANXA11	1.2501325	0.322081	0.0027839	3082	3747	4493	4672	4978	4504

ANXA10	3	1.5849625	0.4299253	0	0	3	0	4	5
PNRC2P1	0.6666667	-0.584963	0.8793857	1	1	1	2	0	0
LZTFL1	2.125	1.0874628	0.4341083	6	0	2	7	0	10
AC046176.1	1.2068966	0.271302	0.4596521	38	17	61	29	58	53
AC009292.1	0.9545455	-0.067114	0.9726144	8	22	14	14	17	11
AC009292.2	0	#NUM!	0.3320541	3	0	2	0	0	0
AL034428.1	0	#NUM!	0.355562	5	0	0	0	0	0
PRKAR2A-AS	1.0634921	0.0888093	0.8547171	22	26	15	15	25	27
AC108134.4	6	2.5849625	0.140013	1	2	0	0	12	6
EFNA5	0.8735043	-0.195113	0.3769624	583	506	666	483	574	476
AC108134.3	1.2727273	0.3479233	0.7850614	7	0	4	2	8	4
EFNA4	0.7218543	-0.47022	0.1514452	113	154	186	125	112	90
EFNB2	1.0530843	0.0746209	0.3588826	1286	1179	1604	1111	1487	1687
EFNB1	0.5674765	-0.817367	1.00E-08	1212	1355	1590	685	817	857
EFNB3	0.5263158	-0.925999	0.4283402	4	12	3	1	7	2
OR4F6	#DIV/0!	#DIV/0!	0.8030358	0	0	0	0	0	1
OR4F3	#DIV/0!	#DIV/0!	0.8030358	0	0	0	1	0	0
EFNA1	0.9496878	-0.074475	0.9623768	2430	2759	3139	2787	2810	2312
AC108134.2	1.2692308	0.3439544	0.5988157	6	7	13	10	14	9
EFNA3	1.275	0.3504972	0.3772871	22	24	34	30	43	29
AC108134.1	0.8	-0.321928	0.8312052	7	15	3	15	5	0
EFNA2	0.6707317	-0.576192	0.4515549	26	13	43	18	9	28
TLX2	0.875	-0.192645	0.8799865	0	9	7	0	12	2
TLX1	#DIV/0!	#DIV/0!	0.1240349	0	0	0	7	1	0
PI4KA	0.7561373	-0.40328	0.004743	3352	3846	3841	2323	2954	3070
DYNLRB2	0.8333333	-0.263034	0.9470936	1	0	5	0	1	4
DYNLRB1	0.9598867	-0.059064	0.9553626	3141	3728	4075	3723	3714	3068
PI4KB	0.826591	-0.274755	0.108728	1319	1383	1525	1168	1185	1141
AC009302.1	1.1551724	0.2081082	0.5996096	13	5	40	27	24	16
LINC02199	#DIV/0!	#DIV/0!	0.5070017	0	0	0	3	0	0
AKTIP	1.0741749	0.1032289	0.2212948	901	934	1104	895	1181	1081
LINC02196	0.3333333	-1.584963	0.4157916	4	3	2	0	0	3
LINC02193	2	1	0.3596004	3	5	2	6	4	10
LINC02192	1	0	0.9890559	0	1	0	0	0	1
OR4K3	0	#NUM!	0.4374838	0	4	0	0	0	0
AC015660.1	#DIV/0!	#DIV/0!	0.5509701	0	0	0	0	3	0
LIMK2	0.7836597	-0.351701	0.0127871	4338	5141	5331	3535	4079	3992
LIMK1	0.9649231	-0.051514	0.9432415	1001	1107	1142	1079	1027	1030
ANXA2R	0.8253012	-0.277007	0.6273319	63	48	55	59	56	22
KISS1	11	3.4594316	0.0916091	0	0	1	8	1	2
AC020915.2	0.3559322	-1.490326	0.047498	21	17	21	3	7	11
AC020915.4	0.7119342	-0.490184	0.2071677	94	60	89	46	51	76
GTF3C2-AS1	0.6666667	-0.584963	0.7748584	1	4	1	2	2	0
AC020915.3	0.5555556	-0.847997	0.52874	5	4	18	9	5	1
AC020915.6	#DIV/0!	#DIV/0!	0.8030358	0	0	0	0	1	0
AC020915.5	1	0	0.9094265	0	0	3	3	0	0
ZNF815P	1.3005464	0.3791179	0.1879213	52	65	66	76	99	63
AC087620.1	0.6666667	-0.584963	0.8234316	0	3	0	0	2	0
AC007384.1	#DIV/0!	#DIV/0!	0.8030358	0	0	0	1	0	0
AC108866.1	6	2.5849625	0.0701522	1	0	2	8	5	5
ZNF826P	0.8552632	-0.22556	0.4021076	612	330	502	281	470	484
AC091153.3	3	1.5849625	0.1108882	5	3	2	10	5	15
AC091153.2	0.7142857	-0.485427	0.797745	2	5	0	0	0	5
XYLB	0.9043779	-0.145002	0.6272503	277	260	331	186	283	316

AC091153.1	0.8846154	-0.176878	0.9661304	11	3	12	14	9	0
SEPT7P2	0.8712446	-0.19885	0.4793368	294	254	384	253	284	275
SEPT7P1	1	0	0.978278	0	0	2	0	2	0
SEPT7P3	#DIV/0!	#DIV/0!	0.5509701	0	0	0	0	3	0
SEPT7P6	0.5333333	-0.906891	0.543945	5	2	8	1	1	6
SEPT7P8	1.1463415	0.1970368	0.6289485	31	28	23	28	39	27
SEPT7P9	0.4615385	-1.115477	0.5056339	4	2	7	4	1	1
AC004840.1	0	#NUM!	0.5337954	0	3	0	0	0	0
TMA7	1.2287105	0.297145	0.1382119	127	117	167	204	160	141
HNRNPA3P9	2.1428571	1.0995357	0.391412	2	0	5	5	1	9
IL22RA1	2.5	1.3219281	0.481483	3	1	0	2	0	8
LYPD6B	1.5960265	0.6744846	0.047022	37	62	52	72	108	61
HNRNPA3P1	0.6	-0.736966	0.662841	4	1	5	1	2	3
NOSTRIN	1.6489945	0.7215866	4.24E-05	419	250	425	607	636	561
HNRNPA3P3	1.1	0.1375035	0.6603187	37	33	40	29	43	49
AC091393.1	0	#NUM!	0.4374838	0	4	0	0	0	0
HNRNPA3P2	0	#NUM!	0.824326	0	1	0	0	0	0
HNRNPA3P5	1.2355769	0.3051848	0.2271123	69	64	75	90	92	75
HNRNPA3P4	0	#NUM!	0.824326	1	0	0	0	0	0
HNRNPA3P6	0.9850267	-0.021765	0.8796959	291	298	346	247	356	318
AC018692.1	0	#NUM!	0.5436524	2	0	1	0	0	0
B3GALT2	0	#NUM!	0.6840298	0	0	2	0	0	0
B3GALT1	0	#NUM!	0.1882176	1	1	5	0	0	0
AL645568.2	#DIV/0!	#DIV/0!	0.5509701	0	0	0	0	3	0
TMC1	0.7923497	-0.335791	0.3350152	149	79	138	68	116	106
AL645568.1	1.2869565	0.3639633	0.2858224	29	39	47	53	58	37
TMC8	0.5789474	-0.788496	0.226277	24	22	30	4	25	15
TMC7	0.6408567	-0.641926	0.0207879	156	235	216	93	181	115
TMC6	0.9267264	-0.109785	0.7887648	575	601	721	607	588	563
TMC5	1.0260417	0.0370893	0.6262281	337	417	398	341	402	439
TMC4	0.4395604	-1.185867	0.109926	15	29	47	21	5	14
TMC3	1.0571429	0.0801703	0.8312711	9	10	16	10	7	20
TMC2	0.7826087	-0.353637	0.6402536	11	21	14	2	18	16
AL583856.1	1.2307692	0.2995603	0.3650959	45	36	49	52	71	37
AC144836.1	1.5666667	0.6476983	0.3365276	13	3	14	24	16	7
AC097662.1	1	0	0.9873936	9	5	8	4	15	3
AL583856.2	#DIV/0!	#DIV/0!	0.4737294	0	0	0	0	4	0
TMF1	1.2824427	0.3588944	0.0474479	711	374	749	568	936	848
AXDND1	1.6428571	0.716207	0.2909589	12	7	9	21	17	8
GGA2	0.7167693	-0.480419	0.000189	4141	4560	5104	2889	3446	3560
GGA1	0.9295526	-0.105392	0.7704631	1395	1709	1992	1522	1609	1606
GGA3	0.835384	-0.259489	0.102375	1657	1854	1853	1370	1647	1464
TCOF1	0.7884963	-0.342824	0.0100973	10424	11996	12960	8445	9856	9596
AC025554.1	#DIV/0!	#DIV/0!	0.8030358	0	0	0	0	0	1
AC112493.1	0.9722222	-0.040642	0.9919257	11	9	16	5	12	18
ATRNL1	#DIV/0!	#DIV/0!	0.4909737	0	0	0	2	0	1
HSD52	#DIV/0!	#DIV/0!	0.8030358	0	0	0	0	1	0
BYSL	0.8115758	-0.301202	0.0766175	1362	1595	1777	1261	1297	1284
AC020911.2	#DIV/0!	#DIV/0!	0.5070017	0	0	0	3	0	0
AC020911.1	0	#NUM!	0.5387425	3	0	0	0	0	0
RPL22L1	0.7173673	-0.479216	0.0079346	530	603	675	412	425	460
LINC02562	0	#NUM!	0.824326	0	1	0	0	0	0
LINC02563	0.2	-2.321928	0.4256458	0	4	1	1	0	0
LINC02560	24	4.5849625	0.0060231	0	1	0	12	4	8

UCKL1-AS1	1.1428571	0.1926451	0.781527	4	15	9	8	7	17
ZC3HAV1L	0.5232877	-0.934324	0.0080323	120	115	130	34	59	98
YY1	1.0324274	0.0460403	0.3983389	5473	5520	6153	4902	6596	6204
YY2	0.625	-0.678072	0.2876777	35	22	15	12	13	20
LINC02557	#DIV/0!	#DIV/0!	0.8030358	0	0	0	0	0	1
LINC02551	3	1.5849625	0.6944949	0	0	1	0	3	0
LINC02550	1.3454545	0.4280937	0.3697219	17	15	23	22	31	21
LINC02553	#DIV/0!	#DIV/0!	0.8030358	0	0	0	0	0	1
AL109797.1	9	3.169925	0.1644993	0	1	0	8	0	1
ZFP69B	1.2420382	0.3127096	0.2919531	48	45	64	47	76	72
AC068768.2	1.2857143	0.3625701	0.7274861	1	1	5	9	0	0
AC068768.1	1.4074074	0.49304	0.5135114	12	9	6	10	18	10
LINC02547	1	0	0.9579379	6	0	14	0	11	9
LINC02544	0.2	-2.321928	0.4521254	0	5	0	0	0	1
LINC02580	0	#NUM!	0.4185478	3	0	1	0	0	0
LINC02581	0.56	-0.836501	0.0306309	55	87	83	30	45	51
TACSTD2	1.2100547	0.2750722	0.0042252	3257	3728	4174	4271	4815	4417
AL391421.1	0.9333333	-0.099536	0.9085337	32	38	35	27	29	42
LINC02577	0.8333333	-0.263034	0.9653543	0	1	5	2	1	2
MLLT10P1	1.3333333	0.4150375	0.7185914	9	1	11	0	22	6
LINC02576	1.1111111	0.1520031	0.9324395	9	0	0	3	4	3
LINC02579	0.3939394	-1.343954	0.3966747	9	1	23	12	0	1
LINC02572	0.25	-2	0.0391622	12	10	18	1	5	4
LINC02575	1.1966912	0.2590509	0.1599759	142	187	215	203	267	181
LINC02574	#DIV/0!	#DIV/0!	0.5509701	0	0	0	0	3	0
LINC02570	1.2857143	0.3625701	0.8088433	4	3	0	5	0	4
B3GALT4	0.5560538	-0.846704	0.0251696	57	84	82	37	46	41
B3GALT6	1.0815331	0.1130778	0.4068198	359	593	483	559	511	482
AC107058.1	#DIV/0!	#DIV/0!	0.5070017	0	0	0	3	0	0
B3GALT5	0	#NUM!	0.5337954	0	3	0	0	0	0
OGFOD1P1	#DIV/0!	#DIV/0!	0.8030358	0	0	0	0	0	1
DBIP2	#DIV/0!	#DIV/0!	0.5070017	0	0	0	3	0	0
GGCT	0.9797297	-0.029544	0.8074314	546	538	692	632	637	471
AP000925.1	0	#NUM!	0.6840298	0	0	2	0	0	0
AL031777.3	0.6571429	-0.605721	0.5335762	11	7	17	5	5	13
TMIE	0	#NUM!	0.5337954	0	3	0	0	0	0
GGCX	1.0365129	0.0517381	0.4453245	1678	2052	1994	1752	2133	2048
LRIT2	#DIV/0!	#DIV/0!	0.6099294	0	0	0	2	0	0
LRIT3	0	#NUM!	0.824326	1	0	0	0	0	0
LINC02569	4.8888889	2.2895066	0.0153603	4	4	1	12	22	10
AL031777.1	0.2459016	-2.023847	0.0149573	16	14	31	6	7	2
LINC02565	0	#NUM!	0.6840298	0	0	2	0	0	0
LINC02568	0.3333333	-1.584963	0.3774648	8	0	4	0	3	1
LINC02520	2	1	0.4805525	4	0	3	5	0	9
EFCAB14	0.949316	-0.07504	0.7977439	2969	2741	3208	2110	3291	3065
EFCAB13	1.5362319	0.619396	0.1739903	18	17	34	22	37	47
EFCAB12	1.2222222	0.2895066	0.7897389	4	11	3	10	12	0
EFCAB11	1.0636872	0.0890739	0.4118869	273	287	335	272	358	322
EFCAB10	0.7407407	-0.432959	0.6440437	13	7	7	1	11	8
ATP7B	1.1336704	0.1810012	0.1190587	2614	2841	3051	2361	3430	3852
LINC02519	#DIV/0!	#DIV/0!	0.5509701	0	0	0	0	3	0
AP005136.2	0.9038462	-0.145851	0.9648363	10	10	32	30	10	7
LINC02518	1.0714286	0.0995357	0.8534493	5	2	7	8	6	1
ATP7A	1.0789229	0.1095918	0.5195004	377	323	377	229	448	485

LINC02511	0	#NUM!	0.6436235	2	0	0	0	0	0
KSR1	0.8687789	-0.202939	0.2524123	4637	5274	5902	3463	4853	5422
OVCH1	1	0	0.9239532	0	0	2	2	0	0
OVCH2	0.8245614	-0.278301	0.7792981	6	22	29	16	24	7
KSR2	0.8804348	-0.183712	0.8517073	27	24	41	32	23	26
AC087276.3	#DIV/0!	#DIV/0!	0.6411933	0	0	0	0	0	2
AC087276.2	0	#NUM!	0.1544204	4	2	1	0	0	0
OR7A5	2	1	0.8324799	1	0	0	0	2	0
LINC02506	1.040404	0.0571439	0.7353318	53	76	69	66	73	67
LINC02505	0.2222222	-2.169925	0.0511159	10	5	30	0	8	2
SEC22B	0.9813372	-0.027179	0.8232254	3143	3050	3559	2724	3409	3437
SEC22A	1.1837238	0.2433325	0.0920565	269	218	324	311	320	329
AC087276.1	0.75	-0.415037	0.7972976	6	5	1	4	0	5
SEC22C	0.8195701	-0.287061	0.114728	559	561	648	386	557	506
DPH6-AS1	0.5	-1	0.7601959	0	0	6	0	0	3
FOCAD	1.0613065	0.0858414	0.3411739	1639	1619	1717	1393	1863	2024
LINC02542	0.6292135	-0.668379	0.2510646	29	21	39	15	24	17
SEC23A	0.8515152	-0.231896	0.2437124	1455	1271	1564	832	1315	1506
LINC02541	#DIV/0!	#DIV/0!	0.8030358	0	0	0	0	0	1
AC103974.1	0	#NUM!	0.3473206	0	5	0	0	0	0
LINC02533	1.5	0.5849625	0.7219617	1	0	3	3	3	0
ATP9B	1.0179775	0.0257057	0.7393991	134	106	205	107	191	155
LINC02532	0.5	-1	0.6615819	5	1	0	0	0	3
SEC23B	0.9289045	-0.106398	0.6160786	2983	3229	3437	2495	3280	3188
ATP9A	1.5909091	0.6698514	0.3774981	10	5	7	10	13	12
LINC02535	#DIV/0!	#DIV/0!	0.8030358	0	0	0	1	0	0
SEC24B	1.1059322	0.1452629	0.2519615	978	752	1102	729	1150	1253
LINC02530	0	#NUM!	0.6436235	2	0	0	0	0	0
SEC24A	0.9793091	-0.030164	0.951681	1959	1472	1982	1221	2080	2000
SMIM12P1	1	0	0.9796677	4	0	3	0	0	7
ATOX1	1.045756	0.0645462	0.3818485	775	989	1252	1099	1077	978
CTCFL	0.721519	-0.470891	0.4792434	31	13	35	7	29	21
AL451061.1	#DIV/0!	#DIV/0!	0.6411933	0	0	0	0	0	2
LINC02525	0.5698925	-0.811238	0.1831506	48	16	29	13	27	13
LINC02528	0.5	-1	0.8188512	2	0	0	0	1	0
LINC02527	1.0100503	0.0144271	0.9235427	56	81	62	44	94	63
LINC02522	0.8194444	-0.287282	0.7455687	24	15	33	25	25	9
SEC24D	0.9616547	-0.056409	0.9389123	1461	1312	1530	1109	1514	1515
LINC02521	1	0	0.9602092	4	0	0	4	0	0
SEC24C	0.8986086	-0.154235	0.4701118	2250	2777	2735	1549	2593	2833
OR7G1	#DIV/0!	#DIV/0!	0.8030358	0	0	0	1	0	0
TRMT112P6	1	0	0.9966043	1	1	3	0	5	0
ZAN	1.6153846	0.6918777	0.4766897	3	1	9	6	11	4
MTND5P15	0	#NUM!	0.6840298	0	0	2	0	0	0
MTND5P14	0.3333333	-1.584963	0.5281956	0	6	0	0	2	0
MTND5P12	0	#NUM!	0.824326	0	0	1	0	0	0
MTND5P11	0.6	-0.736966	0.6925404	0	5	10	5	1	3
MEM56-RWDI	0.4782609	-1.06413	0.3500656	10	7	6	0	5	6
TMPO	0.8445155	-0.243804	0.1283639	5278	4630	5727	3471	4604	5129
A2ML1-AS1	0.2608696	-1.938599	0.0682331	25	1	20	3	3	6
A2ML1-AS2	0	#NUM!	0.4460243	4	0	0	0	0	0
ATP5S	0.7639485	-0.388453	0.118874	294	248	390	242	266	204
PCDH10	1.3157895	0.3959287	0.3943611	37	35	23	26	62	37
PCDH15	1	0	0.9890559	1	0	0	1	0	0

ATP5J	1.1681113	0.2241778	0.0413631	1270	1433	1681	1746	1884	1491
ATP5I	0.8494624	-0.235378	0.7143386	30	34	29	30	33	16
ATP5H	1.035404	0.0501938	0.4329704	1279	1578	1747	1691	1666	1410
PCDH12	1.4	0.4854268	0.6256475	8	4	3	6	10	5
ATP5O	1.4937656	0.5789538	0.0076906	121	130	150	237	181	181
PCDH19	0.4473684	-1.160465	0.2214914	19	12	7	3	2	12
PCDH18	0.8648649	-0.209453	0.7305206	28	25	21	14	25	25
SORD2P	1.6666667	0.7369656	0.6484783	4	1	1	1	6	3
PCDH17	1.1219512	0.16601	0.5293326	52	52	60	48	67	69
ATP5L	0.9257959	-0.111234	0.8346588	1204	1158	1627	1338	1247	1108
AC104073.2	1.3333333	0.4150375	0.7298143	0	0	6	5	0	3
AC104073.1	0	#NUM!	0.1165453	4	2	2	0	0	0
ATP5B	0.8377531	-0.255403	0.0978193	25873	31553	32887	23911	27353	24396
AC104073.4	0.4166667	-1.263034	0.2600537	8	11	17	3	0	12
AL161638.1	0	#NUM!	0.824326	1	0	0	0	0	0
ATP5E	1.4156943	0.5015098	0.000827	1013	1157	1258	1870	1681	1302
ATP5D	1.0943953	0.1301339	0.2709176	981	1139	1270	1513	1259	938
PAG1	2.4705882	1.3048546	0.19954	15	0	2	20	16	6
SEC31B	1.2959641	0.3740258	0.1924036	101	50	72	85	100	104
SEC31A	1.0238027	0.0339377	0.51445	4407	4304	5153	3626	5260	5308
PIK3CD-AS2	1.5084034	0.5930223	0.0285542	78	66	94	156	106	97
MTND5P28	1	0	0.9587748	1	2	3	2	2	2
ATP4B	0	#NUM!	0.824326	1	0	0	0	0	0
TATDN1P1	#DIV/0!	#DIV/0!	0.8030358	0	0	0	1	0	0
ATP4A	0	#NUM!	0.1632507	4	0	4	0	0	0
PAEP	8.6666667	3.1154772	0.0256627	0	0	3	6	14	6
PAF1	0.9627591	-0.054753	0.9158414	1584	1745	2122	1792	1845	1611
OR7C1	1.875	0.9068906	0.2198008	11	7	6	11	12	22
LINC02485	4	2	0.502311	1	0	0	4	0	0
LINC02484	0.5217391	-0.938599	0.3827709	9	7	7	4	5	3
LINC02487	#DIV/0!	#DIV/0!	0.8030358	0	0	0	0	1	0
LINC02486	0.7714286	-0.374396	0.2849774	110	90	115	85	94	64
LINC02482	5.25	2.3923174	0.0850582	3	1	0	15	2	4
MIR4697HG	1.1548718	0.2077327	0.1086369	296	291	388	333	388	405
AL158839.1	2.125	1.0874628	0.0518547	6	27	15	33	36	33
AC092471.1	2.5	1.3219281	0.5647457	1	0	1	2	3	0
AC008619.1	#DIV/0!	#DIV/0!	0.6554062	0	0	0	0	2	0
LINC02477	0	#NUM!	0.6436235	2	0	0	0	0	0
MTND5P42	4	2	0.5760976	1	0	0	0	4	0
LINC02476	1.0769231	0.1069152	0.8796481	3	14	9	9	16	3
LINC02475	15	3.9068906	0.0560656	0	1	0	5	0	10
MTND5P40	0	#NUM!	0.824326	1	0	0	0	0	0
TMEM198B	0.9544199	-0.067304	0.998881	230	190	304	190	219	282
ZFR	0.9496395	-0.074548	0.8501076	3529	2417	3486	2209	3413	3335
SORCS1	0	#NUM!	0.3473206	0	5	0	0	0	0
BUD31P2	#DIV/0!	#DIV/0!	0.2106225	0	0	0	4	0	2
ZFY	1.1580756	0.2117294	0.4440987	129	55	107	73	120	144
SORCS2	0.9565217	-0.06413	0.9867224	6	5	12	5	7	10
ZFX	0.8607383	-0.216354	0.4749774	488	245	459	269	370	387
AP000924.1	0.8245614	-0.278301	0.6914415	16	20	21	6	20	21
AL590682.1	#DIV/0!	#DIV/0!	0.6482162	0	0	0	0	1	1
ATP23	0.892489	-0.164094	0.5842111	219	214	246	189	230	187
INPP1	0	#NUM!	0.3473206	0	5	0	0	0	0
NDUFB4P12	#DIV/0!	#DIV/0!	0.2796611	0	0	0	4	1	0

PAK1	0.7839667	-0.351136	0.0170322	2340	2580	2764	1945	2122	1957
PAK6	0.8692927	-0.202086	0.5860656	301	378	438	386	360	225
PAK3	3.7079646	1.8906275	1.39E-07	23	58	32	102	179	138
mulation;GO:0	#VALUE!	#VALUE!	m;GO:0005	04510|hsa04	ling pathwa	1) activated kinase 3			
PAK2	0.9611442	-0.057175	0.9257447	5347	5368	6065	4239	5860	6029
NDUFB4P11	1.5930233	0.6717673	0.0817374	22	30	34	45	44	48
PAK4	1.1196796	0.163086	0.0804324	3694	4464	4576	4617	5046	4595
LINC02496	0	#NUM!	0.4460243	4	0	0	0	0	0
LINC02492	#DIV/0!	#DIV/0!	0.6099294	0	0	0	2	0	0
RNY3P1	#DIV/0!	#DIV/0!	0.5509701	0	0	0	0	3	0
LINC02488	0	#NUM!	0.824326	0	0	1	0	0	0
RNU5E-10P	0	#NUM!	0.4217812	0	0	5	0	0	0
JADE1	1.0611103	0.0855746	0.2891192	2287	2022	2662	1931	2649	2817
JADE3	0.7831594	-0.352622	0.0409843	2433	2172	2580	1307	2051	2269
KNDC1	12.75	3.6724253	0.0008741	1	1	2	36	5	10
JADE2	0.7643554	-0.387684	0.008516	2786	3278	3671	2114	2549	2778
PALM	0	#NUM!	0.6385096	0	2	0	0	0	0
GGT1	1.5840708	0.6636368	0.0635528	29	41	43	74	46	59
TMX4	0.6801075	-0.556165	0.0042805	350	375	391	214	269	276
FP565324.1	2	1	0.8147319	1	0	0	0	0	2
TMX3	1.0546559	0.0767723	0.4800215	360	281	347	293	368	381
TMX2	0.9138307	-0.130001	0.5972767	2803	2937	3428	2815	2986	2577
TMX1	1.002259	0.0032554	0.7405665	3692	3113	3819	2630	4027	3991
AC008953.1	#DIV/0!	#DIV/0!	0.8030358	0	0	0	0	0	1
AL133520.1	0.8611111	-0.215729	0.8463716	10	11	15	9	8	14
DYNC1I2P1	0.9248826	-0.112658	0.7710684	155	123	148	100	169	125
CYP11A1	1.51796	0.6021337	1.56E-06	1068	1264	1315	1842	1968	1726
GGT5	0.24	-2.058894	0.1453915	3	12	10	0	0	6
AC242843.1	#DIV/0!	#DIV/0!	0.1338503	0	0	0	4	1	2
GGT7	0.8804692	-0.183656	0.3302231	1617	1654	2100	1508	1662	1559
GGT6	1	0	0.971885	1	1	0	0	2	0
AL365440.1	1.1428571	0.1926451	0.7377282	9	1	11	13	4	7
PAN2	0.934217	-0.09817	0.7877606	673	625	739	618	687	598
PAN3	0.9460276	-0.080046	0.849502	868	567	881	499	848	844
CYP11B1	#DIV/0!	#DIV/0!	0.6411933	0	0	0	0	0	2
PICALM	1.0172388	0.0246584	0.587158	7052	5708	7137	5271	7583	7386
GSK3B	0.9787325	-0.031013	0.9172334	3125	2872	3313	2380	3408	3324
;GO:0006468|p	#VALUE!	#VALUE!	:0005739|m	04917|hsa04	1elanogenes	ynthase kinase 3 beta			
GSK3A	0.9862093	-0.020034	0.7534914	838	910	1080	931	1010	848
TMEM191A	0.962406	-0.055282	0.9431798	84	84	98	102	74	80
TMEM191C	1.33687	0.4188592	0.0441347	112	120	145	187	153	164
TMEM191B	1.6294118	0.7043512	0.0213654	41	50	79	100	109	68
AL731533.2	1.5128205	0.5972408	0.1452409	23	29	26	40	37	41
HSDL2	1.1698642	0.226341	0.0631961	2622	1833	2686	2149	3237	2968
HSDL1	0.9108352	-0.134738	0.6096855	799	759	1100	698	919	804
AC012676.5	0.3636364	-1.459432	0.0194894	34	21	88	6	22	24
AC012676.4	1.2	0.2630344	0.897817	0	3	2	0	3	3
AC012676.1	0.5714286	-0.807355	0.3805862	11	12	12	3	4	13
AC012676.3	0.7391304	-0.436099	0.6624828	6	13	4	3	5	9
AC012676.2	3	1.5849625	0.6684577	1	0	0	0	0	3
AC006160.1	0.7272727	-0.459432	0.8133194	4	0	7	2	4	2
ZP1	0.5384615	-0.893085	0.5474696	6	4	3	0	1	6
ZP3	0.8235294	-0.280108	0.6758546	17	35	16	14	14	28
PAOX	0.960452	-0.058215	0.9816819	45	56	76	48	58	64

PARG	1.069395	0.0967949	0.4275399	1051	708	1051	706	1071	1228
AL355607.1	#DIV/0!	#DIV/0!	0.0750283	0	0	0	9	1	0
PARN	0.8869165	-0.17313	0.334784	2096	1726	2262	1479	2067	1850
PARL	0.9144144	-0.12908	0.898913	56	56	110	62	68	73
ATOH8	2.0357143	1.0255351	0.1279528	7	7	14	37	9	11
AP003072.2	1.5	0.5849625	0.732271	0	3	3	0	3	6
AP003072.4	0	#NUM!	0.824326	1	0	0	0	0	0
AP003072.3	#DIV/0!	#DIV/0!	0.8030358	0	0	0	1	0	0
SMNDC1	1.1998633	0.26287	0.01618	1566	1307	1515	1522	1932	1811
ATOH1	#DIV/0!	#DIV/0!	0.3981469	0	0	0	4	0	0
AP003072.5	0	#NUM!	0.824326	1	0	0	0	0	0
AC142381.4	0	#NUM!	0.6840298	0	0	2	0	0	0
AC142381.3	0.5535714	-0.853159	0.1002629	34	34	44	12	23	27
CHURC1	1.0260521	0.037104	0.7331191	440	183	375	259	411	354
AC142381.2	0	#NUM!	0.6840298	0	0	2	0	0	0
REXO1L1P	0	#NUM!	0.5781912	0	0	3	0	0	0
R3HDM4	0.8882194	-0.171012	0.4814757	1395	1577	1841	1506	1471	1298
R3HDM1	1.0183686	0.0262599	0.5938597	1068	934	1210	841	1171	1259
R3HDM2	0.7170362	-0.479882	0.0057413	502	565	647	347	453	429
PATJ	1.1353303	0.183112	0.1627444	2327	1716	2467	1693	2763	2935
AC068279.1	0.4	-1.321928	0.6016774	5	0	0	0	1	1
AC011451.3	0	#NUM!	0.3473206	0	5	0	0	0	0
AC011451.2	0.25	-2	0.2713626	5	3	4	0	0	3
AC011451.1	1.8	0.8479969	0.404763	2	3	5	12	2	4
SYTL1	0.9828375	-0.024975	0.8013837	264	276	334	324	272	263
CD3EAP	0.7975518	-0.32635	0.2266878	316	387	359	329	250	268
LGALS16	4.5625	2.1898246	2.09E-20	64	87	73	380	344	298
PASK	0.7691575	-0.378649	0.0537794	557	671	873	493	538	585
SYTL5	1.0673077	0.0939761	0.7576149	28	16	60	17	54	40
SYTL4	0.6029412	-0.729911	0.1842081	28	57	51	12	22	48
SYTL3	0.4	-1.321928	0.075657	11	32	32	13	5	12
SYTL2	0.6694215	-0.579013	0.2323511	50	34	37	23	32	26
AL354861.3	2.5	1.3219281	0.5635007	2	0	0	3	1	1
LGALS13	0	#NUM!	0.824326	0	1	0	0	0	0
LGALS12	4.25	2.0874628	0.0456847	1	2	5	15	2	17
LETM1P2	0	#NUM!	0.0920998	1	7	1	0	0	0
LINS1	0.9818702	-0.026396	0.8190876	330	290	428	311	370	348
AL137145.2	0.5	-1	0.8143938	2	0	0	1	0	0
CDC42SE1	0.8499595	-0.234534	0.1087161	3369	3647	4101	2600	3544	3305
AC009686.2	3	1.5849625	0.2302918	2	2	1	9	3	3
AC093849.1	#DIV/0!	#DIV/0!	0.6554062	0	0	0	0	2	0
OR5P3	0.9	-0.152003	0.958523	0	4	6	9	0	0
INO80	0.9184263	-0.122764	0.5711016	1504	1358	1637	1064	1519	1549
OR5P2	0	#NUM!	0.5337954	0	3	0	0	0	0
AC021491.2	#DIV/0!	#DIV/0!	0.8030358	0	0	0	0	1	0
LILRB1-AS1	#DIV/0!	#DIV/0!	0.8030358	0	0	0	0	0	1
AC021491.4	1.2	0.2630344	0.7474514	9	3	3	13	1	4
CDC42SE2	0.9301247	-0.104504	0.6391383	2199	1876	2179	1526	2223	2068
CYB561D1	0.6498117	-0.621906	0.0006169	599	585	675	287	497	424
CYB561D2	0.7919321	-0.336551	0.3032081	134	171	166	129	159	85
CYB561D2	0.7919321	-0.336551	0.3032081	134	171	166	129	159	85
PAXX	1.0705379	0.0983359	0.2985522	1607	1924	2083	2332	1968	1710
STPG3-AS1	1.8333333	0.8744691	0.3727342	3	4	5	9	10	3
HSBP1	0.9671472	-0.048193	0.9073633	3636	3892	4404	3892	4150	3498

PAX4	4	2	0.5325759	0	0	1	0	2	2
PAWR	1.1078009	0.1476986	0.1194836	2761	2070	2822	2444	3049	2985
PAX3	#DIV/0!	#DIV/0!	0.2164227	0	0	0	3	0	3
PAX6	0.3870968	-1.369234	0.1873109	10	9	12	6	2	4
PAX5	2	1	0.807847	1	0	0	0	1	1
TTLL7-IT1	#DIV/0!	#DIV/0!	0.6554062	0	0	0	0	2	0
PAX2	#DIV/0!	#DIV/0!	0.2533058	0	0	0	0	7	0
PAX8	0.9086538	-0.138197	0.7379598	102	144	170	85	148	145
ZNF833P	0	#NUM!	0.5387425	3	0	0	0	0	0
PAX9	0.9592668	-0.059996	0.9696012	324	288	370	260	346	336
CTDP1	0.7695625	-0.37789	0.0679169	424	573	626	401	457	391
METTL7B	0.5753425	-0.797507	0.2087807	16	29	28	12	18	12
METTL7A	0.386771	-1.370448	4.36E-21	1179	1370	1412	467	543	522
AC091152.4	1	0	0.9578216	1	0	6	0	4	3
FZD10-AS1	1.7128713	0.7764167	0.0064729	67	77	58	108	135	103
AC091152.2	0.1666667	-2.584963	0.3862605	6	0	0	0	1	0
AC068769.1	0.4444444	-1.169925	0.4223344	1	6	11	2	1	5
AC016642.1	0.1666667	-2.584963	0.0541909	11	7	6	1	3	0
KTN1	1.1991516	0.2620141	0.088631	3400	1818	3740	2885	4537	3320
ZYX	0.8399457	-0.251632	0.1916779	1025	1298	1357	1022	1076	993
GHDC	1.2780749	0.3539723	0.1436899	83	144	147	202	156	120
AC103879.1	#DIV/0!	#DIV/0!	0.8030358	0	0	0	0	0	1
LINC02443	#DIV/0!	#DIV/0!	0.8030358	0	0	0	0	1	0
LINC02442	0	#NUM!	0.824326	0	0	1	0	0	0
BZW2	0.9775091	-0.032818	0.8025798	5117	5523	6478	5280	6054	5399
BZW1	1.1139168	0.1556415	0.0926421	3078	2519	3155	2723	3591	3435
AL391422.1	0.2692308	-1.893085	0.119474	5	9	12	1	1	5
TUBB8	1.4361702	0.5222267	0.2368917	29	9	56	36	60	39
TUBB6	0.9361807	-0.095141	0.7563766	4302	4748	5115	4303	4757	4201
TUBB3	1.59375	0.6724253	0.3020835	15	4	13	14	16	21
TUBB1	1.4166667	0.5025003	0.6943557	3	2	7	0	13	4
LINC02438	0.3	-1.736966	0.3563818	4	4	2	0	0	3
SMARCAD1	1.2870813	0.3641032	0.037881	1182	589	1155	953	1475	1338
LINC02434	1.2272727	0.2954559	0.6914915	4	8	10	9	14	4
AL391422.3	0.8733536	-0.195362	0.4543804	298	316	373	278	328	256
LINC02436	0	#NUM!	0.824326	0	0	1	0	0	0
AL391422.2	0	#NUM!	0.4217812	0	0	5	0	0	0
LINC02435	1.875	0.9068906	0.5320801	5	3	0	4	9	2
AC128657.1	0	#NUM!	0.5172237	1	2	0	0	0	0
CYB561A3	1.0214477	0.0306154	0.6280282	224	245	277	262	222	278
AC109454.2	1.3571429	0.4405726	0.4982296	12	4	12	14	9	15
AC109454.1	0	#NUM!	0.824326	1	0	0	0	0	0
AC124784.1	3	1.5849625	0.5381078	2	0	0	0	2	4
LINC02428	0.9569892	-0.063425	0.9716682	27	40	26	32	30	27
TNIK	1.2491329	0.320927	0.0274821	567	544	619	527	766	868
LINC02463	0.25	-2	0.2848902	7	5	0	2	0	1
METTL2B	0.8877524	-0.171771	0.3797634	1109	1297	1407	987	1168	1230
TNKS	0.8465571	-0.240321	0.2471386	978	816	1067	583	833	1006
METTL2A	0.8960996	-0.158269	0.4531639	911	987	1153	853	967	914
AC109454.4	0	#NUM!	0.6840298	0	0	2	0	0	0
AC109454.3	0.6818182	-0.552541	0.429913	14	19	33	8	23	14
AC104561.3	1.6923077	0.7589919	0.4832309	1	4	8	2	14	6
AC104561.2	5.3333333	2.4150375	0.1528385	0	3	0	5	1	10
AC104561.1	0.7	-0.514573	0.8092803	4	0	6	3	2	2

PITRM1	0.8694433	-0.201836	0.2237875	2187	2540	2764	1899	2299	2315
AL109806.1	0	#NUM!	0.6840298	0	0	2	0	0	0
LINC02458	0.625	-0.678072	0.7644069	0	4	4	2	0	3
LINC02452	0	#NUM!	0.4217812	0	0	5	0	0	0
LINC02454	#DIV/0!	#DIV/0!	0.8030358	0	0	0	0	1	0
LINC02453	0.4	-1.321928	0.6165241	0	3	2	1	0	1
LINC02450	0.8	-0.321928	0.8376027	3	7	0	0	0	8
TNK2	0.8914469	-0.165779	0.5354879	1848	2077	2634	2076	1917	1854
TNK1	0.625	-0.678072	0.6314099	9	4	3	1	0	9
LINC02449	0.6029412	-0.729911	0.2448856	24	24	20	11	19	11
LINC02444	0.6060606	-0.722466	0.4174058	13	9	11	4	7	9
LINC02447	1	0	0.9787773	2	0	1	1	1	1
TUBA8	0.8181818	-0.289507	0.9215374	4	0	7	4	5	0
AC010864.1	0.9818182	-0.026472	0.9683405	33	9	13	23	20	11
METTL12	1.6346154	0.7089512	0.0607075	29	33	42	61	73	36
SCPEP1	0.8518585	-0.231314	0.1687958	1668	1919	1955	1490	1678	1553
METTL14	1.2719011	0.3469865	0.0231263	1071	659	1021	893	1311	1295
AC012186.2	1.5	0.5849625	0.7666222	4	0	0	3	2	1
METTL13	0.8519362	-0.231183	0.1357992	2342	2628	2932	2042	2488	2202
MCM8-AS1	#DIV/0!	#DIV/0!	0.8030358	0	0	0	0	0	1
METTL16	0.8216929	-0.283329	0.0807065	1224	1442	1658	1045	1286	1222
METTL15	1.1335248	0.1808159	0.2493863	491	363	539	346	639	594
UQCRFS1P1	0.8444444	-0.243926	0.6922823	48	67	65	71	42	39
METTL18	1.0837563	0.1160404	0.5147584	144	70	180	132	173	122
METTL17	0.8103914	-0.303309	0.0642252	881	979	1104	726	848	828
AC140725.1	#DIV/0!	#DIV/0!	0.2887809	0	0	0	0	0	6
RPL21P1	0	#NUM!	0.824326	0	1	0	0	0	0
METTL23	1.2926829	0.3703684	0.0451443	168	176	189	189	279	221
METTL22	1.0231959	0.0330824	0.5185444	488	458	606	522	501	565
METTL25	0.6521739	-0.616671	0.0534945	91	96	112	53	77	65
AC073174.1	1.1531532	0.2055841	0.5483849	36	39	36	34	50	44
DMRTA1	0.1428571	-2.807355	0.0680766	3	8	10	2	0	1
TNMD	0	#NUM!	0.6385096	0	2	0	0	0	0
RPL21P3	2	1	0.6315376	3	0	0	3	2	1
RPL21P5	0	#NUM!	0.5781912	0	0	3	0	0	0
METTL27	0.8540146	-0.227667	0.7723982	11	65	61	41	49	27
METTL26	1.0536082	0.0753385	0.4076891	702	827	896	1029	785	741
LINC02421	0	#NUM!	0.6631647	1	0	1	0	0	0
ETFRF1	1.0346457	0.0491368	0.6350441	213	150	272	169	275	213
AC005084.1	#DIV/0!	#DIV/0!	0.2758481	0	0	0	4	0	1
AL035409.1	#DIV/0!	#DIV/0!	0.6099294	0	0	0	2	0	0
HSF2BP	1.972973	0.9803712	0.0905484	14	9	14	26	32	15
AL161637.1	#DIV/0!	#DIV/0!	0.6554062	0	0	0	0	2	0
TNS4	#DIV/0!	#DIV/0!	0.0431236	0	0	0	4	9	0
TNS3	0.8555937	-0.225002	0.1857103	5642	6397	6589	4074	5536	6328
TNS2	0.5081967	-0.976541	0.2336254	20	10	31	16	9	6
TNS1	0.7770563	-0.363909	0.2044368	137	144	181	90	127	142
AC010624.4	0.35	-1.514573	0.3397956	6	8	6	7	0	0
AC010624.3	1	0	0.8837993	3	7	14	13	8	3
LINC02396	#DIV/0!	#DIV/0!	0.8030358	0	0	0	0	1	0
LINC02395	0	#NUM!	0.824326	1	0	0	0	0	0
SACM1L	1.0223752	0.0319248	0.658074	1308	777	1401	834	1362	1368
LINC02391	#DIV/0!	#DIV/0!	0.6482162	0	0	0	0	1	1
LINC02390	#DIV/0!	#DIV/0!	0.242062	0	0	0	6	0	0

RUNX3	0.75	-0.415037	0.8396822	12	2	10	14	0	4
RUNX2	0.6666667	-0.584963	0.826126	0	3	3	2	0	2
RUNX1	1.597397	0.6757229	3.36E-07	784	716	805	970	1351	1361
RNY4P3	0	#NUM!	0.6840298	0	0	2	0	0	0
RNY4P6	0	#NUM!	0.6840298	0	0	2	0	0	0
LINC02389	1.5	0.5849625	0.7974621	0	4	0	1	2	3
AC010624.2	#DIV/0!	#DIV/0!	0.3981469	0	0	0	4	0	0
AC010624.1	0.5839416	-0.776104	0.1671104	48	19	70	16	40	24
UBL7-AS1	0.8492647	-0.235714	0.615708	51	114	107	74	73	84
AC092376.2	0	#NUM!	0.824326	1	0	0	0	0	0
IPO7P2	0	#NUM!	0.824326	0	1	0	0	0	0
AC006557.5	0	#NUM!	0.2106783	2	2	2	0	0	0
AC020663.1	0.3333333	-1.584963	0.6574028	0	2	1	0	0	1
AC020663.3	1.0285714	0.040642	0.8640046	16	7	12	17	9	10
AC073569.1	#DIV/0!	#DIV/0!	0.6099294	0	0	0	2	0	0
AC073569.3	2	1	0.735384	2	0	0	0	2	2
AC006557.1	1.9090909	0.9328858	0.439083	9	0	2	5	8	8
GSTT2B	0	#NUM!	0.6385096	0	2	0	0	0	0
AMMECR1	1.0767134	0.1066343	0.3409102	930	707	931	701	1115	949
TMSB4XP8	1.3409091	0.4232114	0.0489689	96	88	124	129	145	139
TMSB4XP4	1.1935484	0.2552571	0.6644821	8	3	20	9	17	11
TMSB4XP3	0	#NUM!	0.824326	0	1	0	0	0	0
THAP5P1	1	0	0.9961776	2	0	1	0	2	1
TMSB4XP1	0	#NUM!	0.824326	1	0	0	0	0	0
DMRTC1	#DIV/0!	#DIV/0!	0.8030358	0	0	0	0	1	0
NARF-AS1	0	#NUM!	0.824326	0	0	1	0	0	0
OR6A2	#DIV/0!	#DIV/0!	0.8030358	0	0	0	0	1	0
LINC02363	2.5	1.3219281	0.6288365	1	1	0	0	0	5
LINC02362	1.6666667	0.7369656	0.6717781	4	0	2	0	2	8
LINC02361	0.7021277	-0.510195	0.3535567	31	26	37	16	25	25
EPB41L4B	0.9227133	-0.116046	0.6000014	1330	1333	1568	1117	1433	1354
EPB41L4A	1.08	0.1110313	0.8310548	33	23	19	15	40	26
UCHL1	0.3333333	-1.584963	0.6715904	3	0	0	0	1	0
LINC02357	#DIV/0!	#DIV/0!	0.6554062	0	0	0	0	2	0
LINC02356	0	#NUM!	0.1915466	1	0	7	0	0	0
LINC02353	#DIV/0!	#DIV/0!	0.8030358	0	0	0	0	0	1
LINC02352	1	0	0.9726337	2	0	3	0	1	4
IL17RE	1.1990431	0.2618835	0.079154	356	255	434	439	388	426
TUBG2	0.7344398	-0.445284	0.2384542	81	90	70	53	82	42
IL17RD	0.8087005	-0.306323	0.1393782	594	567	632	355	485	610
TUBG1	1.0196705	0.0281031	0.5377072	1048	1411	1608	1440	1480	1227
UCHL3	1.2592593	0.3325753	0.5680229	4	3	20	17	7	10
IL17RC	0.9315068	-0.102362	0.9712181	112	120	206	155	150	103
IL17RB	0.9418345	-0.086455	0.8548268	140	157	150	111	148	162
UCHL5	1	0	0.7088909	1305	871	1465	1005	1401	1235
IL17RA	0.873002	-0.195943	0.3794565	923	1281	1237	966	1152	886
LINC02346	1	0	0.9861498	0	2	0	0	0	2
LINC02345	0	#NUM!	0.824326	0	1	0	0	0	0
LINC02348	0	#NUM!	0.824326	1	0	0	0	0	0
LINC02387	1.2320442	0.301054	0.3157129	67	35	79	105	73	45
TNXB	0.71875	-0.476438	0.6296636	16	8	8	8	14	1
LINC02382	1	0	0.9719164	5	2	4	4	7	0
TNXA	1	0	0.9890559	1	0	0	0	1	0
LINC02381	0.4482759	-1.157541	0.2711107	12	7	10	1	3	9

LINC02384	9	3.169925	0.1403579	0	1	0	5	1	3
TUBD1	1.2268695	0.2949817	0.0499429	251	217	321	277	364	327
PBLD	0.9575163	-0.062631	0.9634278	102	90	114	77	109	107
SUDS3P1	1.3125	0.3923174	0.5369284	9	26	13	17	33	13
RORA-AS1	0.8695652	-0.201634	0.8839416	9	15	22	13	6	21
AC239804.1	3	1.5849625	0.6122545	0	0	1	2	0	1
RANBP2	1.2375529	0.3074902	0.0670404	3664	1896	3659	2664	4598	4147
LINC02374	#DIV/0!	#DIV/0!	0.8030358	0	0	0	0	1	0
RANBP1	0.9332869	-0.099607	0.8191349	8601	10156	11402	9905	9897	8345
LINC02377	0.5714286	-0.807355	0.7137227	0	5	2	0	0	4
USP17L18	0	#NUM!	0.5781912	0	0	3	0	0	0
RANBP3	0.9166461	-0.125563	0.6442428	1144	1381	1518	1210	1354	1142
LINC02371	1.25	0.3219281	0.7961749	0	0	4	3	1	1
LINC02370	3.8	1.9259994	0.1898363	2	1	2	0	10	9
AL358975.1	0.4285714	-1.222392	0.5784642	0	4	3	1	2	0
LINC02373	0	#NUM!	0.6631647	1	0	1	0	0	0
MTSS1L	0.6327345	-0.660328	7.25E-05	716	888	901	432	580	573
RANBP9	0.9628455	-0.054624	0.9935644	1523	1216	1675	1153	1547	1550
TUBE1	0.9398844	-0.089445	0.902951	262	278	325	281	275	257
RANBP6	1.1346328	0.1822255	0.1999617	836	536	834	612	967	924
GHRH	0	#NUM!	0.6385096	0	2	0	0	0	0
R3HCC1	0.9004739	-0.151244	0.5907801	626	703	781	667	664	569
GHRL	0.7727273	-0.371969	0.7981153	7	5	10	6	1	10
LINC02367	1.2	0.2630344	0.8284934	4	2	4	2	8	2
GOLT1B	1.0431779	0.0609852	0.5528281	1027	723	1145	675	1213	1132
GOLT1A	1.3696682	0.4538265	0.0758841	65	67	79	86	100	103
ARHGAP39	1.2146739	0.2805691	0.0725576	214	231	291	239	336	319
ARHGAP35	0.8470588	-0.239466	0.2287056	814	868	953	545	774	913
ARHGAP44	0.8797297	-0.184868	0.5259904	192	271	277	181	233	237
ARHGAP42	0.8363286	-0.257858	0.3065086	641	368	549	293	507	503
ARHGAP40	0.9074074	-0.140178	0.7715557	323	275	428	338	287	306
PRRT3-AS1	0.0666667	-3.906891	0.0515115	4	3	8	0	1	0
PDIA3	0.9901398	-0.014296	0.6694687	31692	34065	40022	33105	38259	33372
GHSR	0	#NUM!	0.824326	1	0	0	0	0	0
PDIA2	0	#NUM!	0.2106783	2	2	2	0	0	0
AC242842.1	1.180602	0.2395227	0.2695694	98	66	135	104	112	137
PDIA6	1.0355146	0.0503479	0.3115594	19947	21392	24127	20022	24497	23272
LGALS9C	#DIV/0!	#DIV/0!	0.8030358	0	0	0	1	0	0
PDIA5	0.9287945	-0.106569	0.6615167	1427	1702	1674	1330	1685	1446
ARHGAP45	0.9622117	-0.055574	0.9228071	1090	1144	1365	1193	1133	1137
LGALS9B	#DIV/0!	#DIV/0!	0.8030358	0	0	0	0	1	0
PDIA4	0.8392288	-0.252864	0.076125	11371	12971	13936	9826	11330	10968
LRIF1	1.5425425	0.6253103	4.06E-05	728	483	787	840	1220	1022
AL359073.1	#DIV/0!	#DIV/0!	0.1513955	0	0	0	0	4	4
ARHGAP19	1.0408421	0.0577512	0.4101656	809	680	886	718	911	843
ARHGAP18	1.2013394	0.2646438	0.0643643	1767	1053	1809	1431	2158	1972
ARHGAP17	0.7627357	-0.390745	0.0042615	2572	2901	3066	1921	2415	2177
ARHGAP15	1	0	0.9430718	7	0	7	3	0	11
ARHGAP12	1.1309192	0.1774959	0.2262221	905	598	1010	640	1117	1085
ARHGAP22	0.9061977	-0.142102	0.6824647	169	223	205	172	211	158
ARHGAP21	1.2248912	0.2926536	0.0403148	4064	2706	4262	3182	4917	5414
ARHGAP20	0.8333333	-0.263034	0.7298126	11	21	28	6	25	19
AC012435.2	1.5	0.5849625	0.7844944	0	1	1	3	0	0
AC012435.1	2.375	1.2479275	0.1568633	4	1	11	6	16	16

ASPSCR1	0.9228636	-0.115811	0.7779301	1053	1471	1443	1338	1206	1117
ARHGAP29	1.3003399	0.3788888	0.0279066	2915	1639	2801	2117	3660	3787
ARHGAP28	1.4805266	0.5661104	0.0011272	706	437	680	670	925	1104
ARHGAP27	1.0496436	0.0698996	0.3644242	1168	1415	1486	1379	1594	1298
ARHGAP26	1.0278497	0.0396294	0.494642	477	428	639	472	573	542
ARHGAP25	0	#NUM!	0.6385096	0	2	0	0	0	0
ARHGAP24	1.6556604	0.7274068	0.0109929	77	61	74	87	107	157
ARHGAP23	0.7705198	-0.376096	0.0128287	3563	4443	4287	2896	3258	3318
ARHGAP33	1.0738462	0.1027873	0.527886	115	91	119	148	96	105
ARHGAP32	0.761396	-0.393281	0.0230317	1459	1210	1543	804	1116	1287
LATS2-AS1	0	#NUM!	0.6840298	0	0	2	0	0	0
ARHGAP31	1.72	0.7824086	0.2802	12	6	7	12	11	20
ARHGAP30	0.7771429	-0.363748	0.4590856	32	64	79	23	69	44
AC020913.3	0.3043478	-1.716207	0.1577779	10	8	5	1	1	5
ULBP2	0.9910714	-0.012939	0.9091602	27	34	51	30	49	32
ULBP1	0.885514	-0.175413	0.6741418	163	97	168	116	129	134
ULBP3	0.7894737	-0.341037	0.5878293	28	24	24	7	20	33
PAN3-AS1	0.6774194	-0.561879	0.4355181	36	17	9	15	11	16
AC009446.1	#DIV/0!	#DIV/0!	0.8030358	0	0	0	0	1	0
AP003469.4	1.0212766	0.0303736	0.7758385	29	10	55	37	30	29
AP003469.3	0	#NUM!	0.6385096	0	2	0	0	0	0
ARHGAP10	1.1787106	0.2372096	0.0094955	1986	2107	2577	2358	2906	2598
AC242988.1	#DIV/0!	#DIV/0!	0.5338029	0	0	0	0	1	2
AL355596.1	0	#NUM!	0.6631647	1	0	1	0	0	0
AP003469.2	1.0714286	0.0995357	0.9777039	9	4	1	0	7	8
AP003469.1	3	1.5849625	0.6546003	0	1	0	0	2	1
AC092720.1	1.0666667	0.0931094	0.9446193	6	3	6	2	14	0
DLEU2L	0.625	-0.678072	0.8106403	0	1	7	2	0	3
TOB2	0.9345896	-0.097595	0.7180844	2914	3487	3735	2602	3284	3587
TOB1	0.8190128	-0.288042	0.0738876	1114	1032	1136	819	973	896
LRIG1	0.8481848	-0.237549	0.4862607	91	120	92	64	102	91
LRIG2	0.823991	-0.279299	0.1842094	587	466	731	450	533	487
SPINT2	1.0103674	0.01488	0.5815724	3889	5163	5320	4755	5154	4612
ALKBH7	0.9332659	-0.09964	0.9302932	503	687	788	735	631	480
ALKBH6	1.0263158	0.0374747	0.7758102	68	33	51	68	41	47
ALKBH8	1.0774194	0.1075799	0.4479559	337	233	360	237	396	369
ALKBH3	5	2.3219281	0.1437879	2	0	1	2	5	8
ALKBH2	1.0487265	0.0686384	0.475323	505	666	635	680	684	530
ALKBH5	1.0431138	0.0608965	0.3828344	1446	1770	1794	1597	1820	1809
ALKBH4	0.9606557	-0.057909	0.9934165	204	343	368	248	338	293
ALKBH1	0.9754355	-0.035882	0.8904721	639	753	847	655	827	702
AC090616.3	0	#NUM!	0.824326	1	0	0	0	0	0
AL391863.1	#DIV/0!	#DIV/0!	0.8030358	0	0	0	0	0	1
AC090616.7	0.5	-1	0.3377017	14	3	15	6	6	4
FAM185BP	0.5454545	-0.874469	0.2572786	21	19	15	14	5	11
SPINT1	0.8996519	-0.152561	0.5064921	8140	9499	10513	8735	8808	7784
AP000873.2	0.7380952	-0.438121	0.3801305	67	28	73	33	42	49
AP000873.4	0.4583333	-1.125531	0.4087819	8	3	13	5	0	6
ATRIP	0.5625	-0.830075	0.5463454	2	14	0	0	5	4
TOE1	0.707649	-0.498894	0.0073611	595	732	804	515	511	482
MIR33B	#DIV/0!	#DIV/0!	0.5070017	0	0	0	3	0	0
PBX3	1.3555556	0.4388842	0.0642945	84	93	93	107	139	120
PBX2	0.9387125	-0.091245	0.7353587	2393	2888	2812	2264	2870	2463
PBX4	0.921875	-0.117357	0.9054767	23	51	54	37	44	37

MIR339	2.6666667	1.4150375	0.5422199	0	3	0	0	3	5
PBX1	0.7559233	-0.403688	0.0186688	853	870	936	490	775	745
SEC24B-AS1	1.0789474	0.1096245	0.7952847	11	9	18	13	19	9
AC011944.2	0	#NUM!	0.5075476	0	0	4	0	0	0
AC011944.1	1.3181818	0.3985494	0.6534213	12	1	9	4	13	12
CRYBB2P1	1.1737892	0.2311733	0.1015857	311	313	429	461	413	362
AC092919.2	0.9457831	-0.080419	0.9655044	42	48	76	43	70	44
AC092919.1	1	0	0.9890559	0	1	0	1	0	0
IL18BP	0.6351351	-0.654865	0.0462678	154	136	154	44	107	131
AC006518.2	#DIV/0!	#DIV/0!	0.6411933	0	0	0	0	0	2
AC108868.2	0	#NUM!	0.824326	1	0	0	0	0	0
PCCA-AS1	3	1.5849625	0.6552898	1	0	0	0	2	1
TTC28-AS1	1.083815	0.1161186	0.6085571	111	122	113	119	179	77
AC006028.1	1	0	0.9890559	0	0	1	0	1	0
GHRLOS	0.7142857	-0.485427	0.4707674	18	21	24	8	15	22
GHRLOS	0.7142857	-0.485427	0.4707674	18	21	24	8	15	22
PSMD12P	1	0	0.8801582	2	5	11	11	4	3
AL049767.1	#DIV/0!	#DIV/0!	0.8030358	0	0	0	0	1	0
MYADML	0	#NUM!	0.824326	1	0	0	0	0	0
AC116025.2	#DIV/0!	#DIV/0!	0.2545643	0	0	0	0	4	2
APEX2	0.9391261	-0.090609	0.8283898	1673	2026	2297	1807	2055	1769
AC231533.2	0.9565217	-0.06413	0.9746892	2	12	9	5	11	6
AC231533.1	0.1428571	-2.807355	0.3870602	0	0	7	0	0	1
GID4	1.0265171	0.0377576	0.5203287	590	619	752	558	705	750
APEX1	0.9643379	-0.052389	0.9493805	9798	11665	12747	10518	11726	10746
SHKBP1	0.8453504	-0.242379	0.2238688	1663	2003	2270	1744	1681	1593
GID8	0.9901497	-0.014281	0.7201426	3896	4306	4488	4163	4368	4034
TMEM167B	0.9698085	-0.044228	0.9444573	869	842	1005	751	952	931
TMEM167A	1.061517	0.0861275	0.2990154	5074	3860	5436	4024	5711	5519
LAPTM4B	1.0498534	0.0701878	0.2651478	2214	2436	2852	2503	2816	2557
LAPTM4A	0.9924276	-0.010966	0.7450169	6012	6890	7171	5852	7591	6478
TOM1	0.8154884	-0.294264	0.0683021	2808	3310	3670	2627	2754	2601
SDCBP2	0.4117647	-1.280108	0.3696203	2	8	7	2	5	0
AC008915.2	0.1666667	-2.584963	0.3351635	1	2	3	0	1	0
AC008915.1	#DIV/0!	#DIV/0!	0.6099294	0	0	0	2	0	0
NONOP2	0.7631579	-0.389947	0.6464962	14	11	13	8	16	5
OR9A4	0	#NUM!	0.824326	0	0	1	0	0	0
AC243571.2	#DIV/0!	#DIV/0!	0.3831122	0	0	0	0	5	0
AP001858.2	#DIV/0!	#DIV/0!	0.4909737	0	0	0	2	0	1
FAM96AP2	0.25	-2	0.5021925	2	1	1	0	0	1
AL445623.2	0.6388889	-0.646363	0.6280887	8	4	24	11	12	0
UCKL1	1.0652621	0.0912084	0.3219311	553	584	809	764	716	593
AC006122.1	5	2.3219281	0.4886016	0	0	1	0	5	0
ZNF891	0.813449	-0.297876	0.3693172	185	103	173	77	155	143
ZNF888	1.011811	0.0169399	0.7728808	625	296	603	372	651	519
ZNF883	1.719697	0.7821544	0.0231431	47	33	52	53	105	69
ZNF880	0.9768177	-0.033839	0.9304247	300	271	378	235	388	304
PCCA	0.9004458	-0.151289	0.5362117	421	411	514	336	477	399
PCCB	0.8894555	-0.169006	0.3938818	1567	1796	2128	1522	1801	1561
ZNF879	0.9465241	-0.079289	0.9431029	213	122	226	155	210	166
ZNF878	1.278481	0.3544307	0.1445661	81	74	82	94	103	106
TOP1	1.1639129	0.2189832	0.039724	6100	4597	6855	5423	7867	7139
DCTPP1	0.8426113	-0.247061	0.3331985	526	695	755	613	564	488
UBQLN1	1.1602784	0.214471	0.0370858	6177	5396	6533	5418	7844	7746

AC018410.2	0.21875	-2.192645	0.0675956	6	12	14	0	1	6
ZNF865	1.0370656	0.0525072	0.5711659	376	484	435	460	499	384
UBQLN4	0.7645927	-0.387237	0.0102232	1799	2154	2283	1282	1777	1709
ZNF862	0.5604396	-0.835369	0.181106	31	20	40	14	11	26
UBQLN2	0.8233083	-0.280495	0.0657667	1799	1990	2063	1362	1710	1746
ZNF860	1.3057325	0.3848594	0.0615419	170	139	162	152	251	212
AL590004.3	#DIV/0!	#DIV/0!	0.6554062	0	0	0	0	2	0
TM6SF1	0.25	-2	0.5512175	2	0	2	0	1	0
EFR3B	0.7087379	-0.496676	0.3609354	31	47	25	13	23	37
TM6SF2	#DIV/0!	#DIV/0!	0.3597364	0	0	0	0	0	5
AL512770.1	1.2727273	0.3479233	0.5065116	14	12	18	20	24	12
UBQLNL	1.0416667	0.0588937	0.8642338	7	2	15	5	6	14
ZNF853	3	1.5849625	0.6684577	1	0	0	0	0	3
VEZF1P1	0	#NUM!	0.6604275	0	1	1	0	0	0
ZNF852	0.8267717	-0.274439	0.4219355	134	100	147	81	102	132
ZNF850	0.6789536	-0.558615	0.0073992	607	454	621	266	403	473
MTFR1L	0.9652729	-0.050991	0.9117898	391	435	585	429	482	451
EFR3A	0.9375704	-0.093001	0.7495181	1678	1204	1555	906	1552	1702
TP63	1.0291731	0.0414856	0.5601538	1414	1417	1728	1112	1740	1840
GIN1	1.3014184	0.3800849	0.0931113	199	113	252	170	326	238
TMEM170B	1.1336207	0.180938	0.4597382	85	54	93	62	97	104
TMEM170A	0.8932884	-0.162802	0.419377	1981	1561	2090	1266	1872	1893
TP53	0.620909	-0.687546	9.94E-07	2725	3420	3602	1865	2214	1973
ıse to DNA dar	#DIV/0!	#DIV/0!							
n cell cycle arr	#VALUE!	#VALUE!	GO:000582	05167|hsa05	eukemia vir	r protein p53			
RN7SL868P	#DIV/0!	#DIV/0!	0.5070017	0	0	0	3	0	0
GIPR	1.462963	0.5488932	0.2744431	15	11	28	27	36	16
AC064853.3	#DIV/0!	#DIV/0!	0.3831122	0	0	0	0	5	0
APELA	0	#NUM!	0.824326	1	0	0	0	0	0
AL139407.1	1.6956522	0.7618403	0.3303965	8	1	14	9	17	13
B4GALNT1	0.6027397	-0.730393	0.2872641	19	19	35	4	23	17
B4GALNT3	1.3108504	0.3905031	0.0291537	161	229	292	255	323	316
B4GALNT4	1.7767857	0.8292697	0.0164687	28	32	52	76	63	60
PCK2	1.3332136	0.414908	0.0023617	752	1015	1018	1268	1232	1213
RTFDC1	0.9457885	-0.08041	0.8949326	1912	2197	2587	2030	2233	2070
HARS2	0.842047	-0.248027	0.1248632	1146	1160	1309	934	1111	999
TP73	1.4621849	0.5481257	0.025689	72	79	87	112	113	123
COX7B	0.9664257	-0.049269	0.8841837	3471	3897	4665	3989	4168	3472
AC073916.1	0	#NUM!	0.5781912	0	0	3	0	0	0
COX7C	1.0391629	0.0554219	0.3821719	5839	6593	7638	7522	7465	5869
PCM1	1.1608466	0.2151773	0.1167479	1392	864	1524	1155	1798	1435
PCLO	0.2363636	-2.08092	2.56E-07	94	84	97	10	29	26
NMRK2	0.9253112	-0.111989	0.8322249	137	182	163	159	144	143
NMRK1	1.2550336	0.3277259	0.0120487	745	625	865	968	1039	798
AC009743.1	3.4	1.7655347	0.2893457	0	2	3	0	15	2
SETP21	#DIV/0!	#DIV/0!	0.8030358	0	0	0	0	0	1
HYDIN2	0.125	-3	0.2143977	4	1	3	0	1	0
SETP20	0.7272727	-0.459432	0.8190017	6	0	5	4	3	1
MCF2L2	1.1666667	0.2223924	0.7796359	18	16	8	6	25	18
GIT1	0.9500873	-0.073868	0.8851234	1169	1366	1472	1145	1346	1316
COX8A	1.0072595	0.0104355	0.723715	132	190	229	242	192	121
COX8C	0.5833333	-0.777608	0.4977814	7	6	11	1	12	1
AC018557.2	#DIV/0!	#DIV/0!	0.3597364	0	0	0	0	0	5
TOX4	0.9327304	-0.100468	0.6675125	3795	4249	4562	3469	4312	3977

TOX3	0	#NUM!	0.4374838	0	4	0	0	0	0
TOX2	#DIV/0!	#DIV/0!	0.8030358	0	0	0	1	0	0
PCNA	0.8850844	-0.176113	0.3032873	8084	8289	10116	7500	8505	7440
COX5B	0.9956827	-0.006242	0.641882	4082	4977	5765	5298	5009	4453
COX5A	0.80694	-0.309467	0.0908173	2573	2984	3492	2530	2686	2086
AC007106.1	2	1	0.8324799	1	0	0	0	2	0
PCNP	1.0678948	0.0947695	0.2616352	5410	4762	6059	4399	6689	6245
PCNT	0.927924	-0.107921	0.6952084	1386	1408	1840	1095	1559	1646
AC002401.1	#DIV/0!	#DIV/0!	0.5509701	0	0	0	0	3	0
ATP2B1-AS1	0.25	-2	0.2292897	13	0	3	2	1	1
GIT2	0.7912525	-0.33779	0.1316652	333	284	389	200	288	308
TMEM176B	0	#NUM!	0.824326	0	1	0	0	0	0
COX6C	0.9397779	-0.089608	0.8513885	1654	1985	2123	1855	2003	1557
IL18R1	1.375	0.4594316	0.3165617	21	16	27	37	35	16
Z98742.1	0	#NUM!	0.355562	5	0	0	0	0	0
NIP7P3	0.6666667	-0.584963	0.9395362	0	0	3	2	0	0
BX255923.2	0.5	-1	0.8652929	0	0	2	0	1	0
AC135731.1	0	#NUM!	0.824326	0	0	1	0	0	0
AC097468.3	0.8867925	-0.173332	0.8721601	15	9	29	7	22	18
AC097468.1	0.6428571	-0.63743	0.7233797	7	1	6	6	3	0
AL591468.1	#DIV/0!	#DIV/0!	0.8030358	0	0	0	1	0	0
SPINK4	5	2.3219281	0.4886016	0	0	1	0	5	0
SPINK2	5.5	2.4594316	0.0164462	7	0	1	16	12	16
SPINK1	0.6	-0.736966	0.8851219	0	0	5	3	0	0
SPINK6	#DIV/0!	#DIV/0!	0.5509701	0	0	0	0	3	0
SPINK5	1.125	0.169925	0.4595595	102	45	101	92	94	93
MIR27B	0.9090909	-0.137504	0.9902785	0	2	9	2	6	2
MIR27A	#DIV/0!	#DIV/0!	0.2088383	0	0	0	5	0	1
PCP2	0.3529412	-1.5025	0.2702446	8	4	5	3	2	1
PCP4	0	#NUM!	0.824326	1	0	0	0	0	0
Z98742.3	0.6666667	-0.584963	0.8754808	0	3	0	2	0	0
COX19	0.8799571	-0.184495	0.5018181	273	288	372	231	277	313
COX16	1.0091743	0.0131754	0.8779576	39	31	39	33	44	33
COX15	1.1504891	0.2022473	0.045062	1316	1240	1431	1261	1712	1614
COX18	0.9989712	-0.001485	0.783449	302	330	340	279	336	356
COX17	1.035868	0.0508402	0.6086416	239	215	243	204	271	247
AC017081.1	#DIV/0!	#DIV/0!	0.8030358	0	0	0	0	0	1
TIMM50	0.8406618	-0.250403	0.2128738	1824	2370	2515	1956	1943	1741
COX11	0.9166329	-0.125584	0.6322906	697	798	976	644	901	720
COX14	0.9225473	-0.116305	0.8237484	134	214	233	168	190	178
COX10	0.9621947	-0.055599	0.9882858	897	1028	1064	919	960	997
SETP17	3	1.5849625	0.4925092	1	0	1	0	2	4
SETP14	1.0680272	0.0949484	0.698874	52	38	57	39	65	53
TIMM44	0.8392857	-0.252766	0.1674761	1243	1678	1615	1162	1371	1274
THRAP3P1	0	#NUM!	0.6385096	0	2	0	0	0	0
SETP12	1.8461538	0.8845228	0.3898124	2	7	4	4	6	14
COX20	0.957265	-0.06301	0.9232003	54	33	30	27	48	37
PCTP	0.962127	-0.055701	0.9946056	1516	1762	1950	1489	1831	1710
MIR23A	0	#NUM!	0.3267077	0	3	2	0	0	0
AL139011.1	#DIV/0!	#DIV/0!	0.8030358	0	0	0	1	0	0
AL359918.1	#DIV/0!	#DIV/0!	0.8030358	0	0	0	0	0	1
RALY-AS1	1.0871795	0.1205901	0.6443553	65	69	61	56	92	64
FDPSP4	0	#NUM!	0.824326	1	0	0	0	0	0
FDPSP3	2	1	0.6090148	0	2	2	0	5	3

FDPSP2	#DIV/0!	#DIV/0!	0.6411933	0	0	0	0	0	2
FDPSP1	0	#NUM!	0.5781912	0	0	3	0	0	0
AC026634.1	#DIV/0!	#DIV/0!	0.5371397	0	0	0	0	0	3
TIMM8B	0.8087405	-0.306251	0.061555	1188	1345	1540	1016	1258	1020
TIMM8A	0.952381	-0.070389	0.8933181	705	627	684	587	685	648
TPBG	0.710257	-0.493587	0.000362	1412	1401	1584	914	1181	1028
AC079305.3	#DIV/0!	#DIV/0!	0.8030358	0	0	0	0	0	1
AC079305.1	0.8571429	-0.222392	0.8968005	1	5	8	1	5	6
AC025161.1	0.5	-1	0.8079011	0	2	0	1	0	0
NPIPB13	0.9354839	-0.096215	0.9400344	12	9	10	6	9	14
NPIPB12	0.8703704	-0.200299	0.823253	27	7	20	13	25	9
NPIPB11	0.6565657	-0.606989	0.2982734	29	21	49	16	29	20
NPIPB15	1.6666667	0.7369656	0.7471303	3	0	0	3	2	0
GJA1	0.7449306	-0.424822	0.0519891	325	290	322	186	297	215
GJA5	1.2367309	0.3065316	0.0186219	610	763	756	751	1018	864
GJA4	0	#NUM!	0.07221	1	0	12	0	0	0
LAGE3P1	3.3333333	1.7369656	0.3441781	0	3	0	6	1	3
AP005431.1	0	#NUM!	0.824326	0	1	0	0	0	0
AC005532.2	#DIV/0!	#DIV/0!	0.6554062	0	0	0	0	2	0
LIPT2	0.9166667	-0.125531	0.8722245	93	66	117	87	87	79
AC005532.1	0.875	-0.192645	0.9299821	9	1	14	3	3	15
LIPT1	1.0637363	0.0891405	0.5323083	169	130	156	167	172	145
AC108471.2	1.1320755	0.1789701	0.6726358	10	16	27	15	18	27
TPH1	4	2	0.5362768	1	0	0	0	3	1
AC108471.1	#DIV/0!	#DIV/0!	0.4098001	0	0	0	1	3	0
AKAP17A	0.9987059	-0.001868	0.6103984	1794	2009	2379	2031	2096	2047
FAM60BP	1	0	0.9443813	1	0	1	2	0	0
GJC1	1.0029838	0.0042983	0.7918217	732	782	832	555	836	962
GJC3	1.2	0.2630344	0.9023107	1	3	1	0	0	6
MIR212	0	#NUM!	0.5337954	0	3	0	0	0	0
GJC2	2	1	0.0594106	8	22	17	36	25	33
MIR210	1.7272727	0.7884959	0.4495553	6	1	4	13	1	5
POM121C	0.7932847	-0.334089	0.0208187	2040	2316	2494	1509	2010	1915
POM121B	0.7878788	-0.343954	0.6874847	9	18	6	8	12	6
MIR215	0	#NUM!	0.4460243	4	0	0	0	0	0
GJB1	0	#NUM!	0.6436235	2	0	0	0	0	0
GJB4	#DIV/0!	#DIV/0!	0.6411933	0	0	0	0	0	2
MIR221	0.8	-0.321928	0.937578	1	0	4	0	0	4
GJB3	#DIV/0!	#DIV/0!	0.3096126	0	0	0	5	0	0
GJB5	0.2674419	-1.902703	0.004569	23	42	21	4	9	10
GJB7	1.5182927	0.6024499	0.058277	40	58	66	66	70	113
AC023310.4	0	#NUM!	0.2605273	0	3	3	0	0	0
TPI1	0.9693932	-0.044846	0.8737798	18611	23348	24987	22739	22566	19592
AP001619.1	1.2307692	0.2995603	0.7232884	0	5	8	8	4	4
FAM60CP	0.862069	-0.214125	0.8034978	25	20	42	24	22	29
AC020910.3	#DIV/0!	#DIV/0!	0.1500119	0	0	0	0	3	5
AC020910.5	0.4166667	-1.263034	0.2431557	8	10	6	3	3	4
AC020910.4	1.3103448	0.3899465	0.5487748	2	13	14	20	10	8
GJD3	0.2631579	-1.925999	0.2321969	4	2	13	0	5	0
AC002059.1	0.5	-1	0.8638172	0	0	2	1	0	0
AL161772.1	1.0120482	0.017278	0.8000978	34	56	76	55	61	52
SPTY2D1	1.0264668	0.037687	0.615753	1105	843	1188	753	1239	1227
AP001619.2	0	#NUM!	0.6385096	0	2	0	0	0	0
TPK1	0.84	-0.251539	0.4865775	195	179	276	213	173	160

SYVN1	0.8338075	-0.262214	0.1056149	1218	1388	1612	1018	1324	1175
ACA64	1.3333333	0.4150375	0.8356718	2	0	1	2	2	0
ACA64	1.3333333	0.4150375	0.8356718	2	0	1	2	2	0
ACA64	1.3333333	0.4150375	0.8356718	2	0	1	2	2	0
ACA64	1.3333333	0.4150375	0.8356718	2	0	1	2	2	0
ACA64	1.3333333	0.4150375	0.8356718	2	0	1	2	2	0
ACA64	1.3333333	0.4150375	0.8356718	2	0	1	2	2	0
AGMAT	0.0555556	-4.169925	0.0538324	4	0	14	0	0	1
ACA59	0.3684211	-1.440573	0.2832152	5	6	8	0	6	1
ACA59	0.3684211	-1.440573	0.2832152	5	6	8	0	6	1
MRE11	1.0213333	0.0304538	0.5897044	1129	655	1216	891	1117	1056
TPMT	1.0796761	0.1105985	0.2254725	1292	1001	1535	1217	1613	1303
TMEM179B	1	0	0.7471083	160	173	194	222	148	157
NPEPPS	1.1001004	0.1376352	0.0746384	5428	5220	6285	5243	6834	6551
TMEM178B	2.1428571	1.0995357	0.4129774	4	1	2	2	2	11
TPM4	1.0144424	0.020687	0.4601178	16515	16290	19610	15266	19441	18465
TMEM178A	#DIV/0!	#DIV/0!	0.8030358	0	0	0	0	1	0
TPM3	0.9701104	-0.043779	0.8960911	18382	19599	22341	17941	20863	19715
TPM2	1.677037	0.7459146	0.0004046	157	201	317	388	457	287
TPM1	0.886639	-0.173581	0.2845071	28161	28124	34831	25480	28537	26770
ZNF799	0.9367681	-0.094236	0.8479721	157	128	142	122	157	121
AC004943.1	1.4186047	0.5044726	0.3656228	11	6	26	28	7	26
ZNF793	0.7619048	-0.392317	0.7350383	6	12	3	8	5	3
ZNF792	0	#NUM!	0.6840298	0	0	2	0	0	0
AC004943.3	0.75	-0.415037	0.8724887	4	0	0	0	0	3
ZNF791	0.9180328	-0.123382	0.7366417	472	323	486	307	373	496
AC004943.2	0.9921722	-0.011338	0.8119276	324	316	382	276	329	409
ZNF790	0.7564103	-0.402759	0.3124496	97	66	71	31	63	83
AKAP17BP	0	#NUM!	0.4117982	0	3	1	0	0	0
AC233289.1	#DIV/0!	#DIV/0!	0.5509701	0	0	0	0	3	0
ZNF789	0.8523132	-0.230544	0.3072511	382	325	417	250	348	360
ZNF788	1.125	0.169925	0.8527216	0	2	6	2	3	4
TPPP	0.7692308	-0.378512	0.5619303	21	30	66	25	31	34
ZNF787	0.9111035	-0.134313	0.7159226	780	1074	1082	996	924	755
ZNF786	0.9834213	-0.024119	0.8312443	375	425	527	371	479	455
PSPC1-AS2	1.5490196	0.6313554	0.2803863	28	6	17	18	33	28
ZNF785	1.7222222	0.7842713	0.3286492	7	3	8	12	12	7
ZNF784	0.8192771	-0.287577	0.437602	99	187	129	125	124	91
ZNF783	0.770751	-0.375663	0.1849626	142	159	205	121	128	141
ZNF782	0.9962264	-0.005454	0.8073855	202	98	230	147	181	200
AKIRIN2	1.0411433	0.0581686	0.3334266	1585	1807	2101	1833	2019	1867
ZNF781	1.4252874	0.5112528	0.1903514	33	20	34	42	46	36
AKIRIN1	1.0831201	0.1151932	0.18406	2870	2907	3210	2590	3654	3490
PDCL	0.8627595	-0.21297	0.191626	1349	1323	1423	1031	1313	1189
AKT3-IT1	4	2	0.5760976	1	0	0	0	4	0
TPP2	1.0396586	0.0561098	0.534999	2930	1766	2919	1958	2980	2979
ZNF778	0.9793713	-0.030072	0.8583627	668	619	749	580	634	780
AL592546.1	0.9375	-0.093109	0.9699142	3	7	6	5	7	3
TPP1	0.9568728	-0.063601	0.917056	3022	3525	3609	2859	3419	3440
ZNF777	0.7377691	-0.438759	0.0339059	412	544	577	382	387	362
ZNF776	1.0026132	0.0037652	0.8357117	431	277	440	249	481	421
ZNF775	1.36	0.4436067	0.257056	27	26	47	66	48	22
ZNF774	0.753861	-0.40763	0.0499221	306	337	393	201	306	274
MIR31HG	1.6666667	0.7369656	0.7274237	2	0	1	1	0	4

ZNF773	2.6666667	1.4150375	0.5692132	2	1	0	0	8	0
ZNF772	0.9283019	-0.107334	0.7424784	277	252	266	163	262	313
ZNF771	0.9376299	-0.092909	0.9774956	116	161	204	176	146	129
ZNF770	0.9601858	-0.058614	0.8950146	592	395	520	254	539	654
AC011453.2	0.7804878	-0.357552	0.7931878	8	5	28	8	24	0
MIR320E	3.6666667	1.8744691	0.3258192	0	0	3	3	8	0
MIR320A	0.375	-1.415037	0.624337	0	0	8	0	0	3
AC011453.1	1.045811	0.0646221	0.2922101	2390	2003	2876	2468	2693	2441
ZNF768	0.8148576	-0.29538	0.1639342	708	967	923	719	787	611
ZNF766	1.1011315	0.1389868	0.3203739	498	323	593	387	645	525
ZNF765	1.1329056	0.1800276	0.2668265	401	213	477	338	491	407
OR8D2	0	#NUM!	0.1888496	6	1	0	0	0	0
ZNF764	0.747006	-0.420808	0.1229417	195	188	285	160	150	189
OR8D1	0.6451613	-0.632268	0.5298901	12	10	40	22	7	11
ZNF763	1.3267974	0.4079481	0.184975	56	43	54	86	58	59
TPRN	1.1730141	0.2302204	0.0483221	1256	1551	1788	1943	1828	1619
ZNF761	1.3071663	0.3864427	0.0242514	1401	713	1542	1290	1958	1531
OR6C76	0	#NUM!	0.824326	1	0	0	0	0	0
ZNF75D	0.7740113	-0.369573	0.0715014	441	354	444	291	374	294
ZNF75A	1.0130354	0.0186846	0.6677809	618	393	600	474	577	581
NPEPL1	1.8	0.8479969	0.3788442	2	0	13	12	12	3
ZNF750	1.4076133	0.4932511	0.0003528	651	862	825	997	1244	1050
PDHB	0.8863485	-0.174054	0.384895	1392	1423	1690	1315	1380	1298
AC018797.3	0	#NUM!	0.6840298	0	0	2	0	0	0
AC018797.2	1.0285714	0.040642	0.9456849	6	17	12	7	21	8
AC022868.1	0	#NUM!	0.5781912	0	0	3	0	0	0
OR8B8	0	#NUM!	0.5643268	1	0	2	0	0	0
ZNF749	0.9209302	-0.118836	0.7577958	140	131	159	99	166	131
ZNF747	1.1451613	0.1955508	0.6364368	11	19	32	26	33	12
ZNF746	0.7288454	-0.456315	0.0039505	834	883	942	521	740	677
OR8B3	0	#NUM!	0.5781912	0	0	3	0	0	0
OR8B2	0.3333333	-1.584963	0.5884702	0	0	6	0	1	1
AC090617.6	1.2903226	0.3677318	0.6289139	19	4	8	9	19	12
ZNF740	0.9679767	-0.046956	0.9397388	443	420	511	410	472	448
AC090617.3	#DIV/0!	#DIV/0!	0.8030358	0	0	0	0	1	0
AC090617.9	0	#NUM!	0.824326	1	0	0	0	0	0
ZNF738	0.888467	-0.17061	0.3691941	1253	1294	1667	1017	1429	1298
ZNF737	1.0142857	0.0204641	0.7866014	128	67	155	81	123	151
ZNF736	0.7820789	-0.354614	0.1398743	503	338	554	238	428	425
OR8A1	1.0918367	0.1267571	0.6833505	42	19	37	32	38	37
ZNF732	1.4634146	0.5493386	0.0098706	191	113	229	201	310	269
ZNF730	0.972028	-0.04093	0.9140282	93	59	134	77	106	95
TPT1	1.037642	0.0533088	0.3471049	20944	23806	27855	26227	26516	22595
UMODL1	#DIV/0!	#DIV/0!	0.3981469	0	0	0	4	0	0
TMEM183A	0.8610587	-0.215817	0.1644091	6832	8040	8440	6151	7284	6638
AL139398.1	0.25	-2	0.5293454	0	3	1	0	0	1
MIR3138	#DIV/0!	#DIV/0!	0.4121728	0	0	0	0	1	3
ZNF846	1.1714286	0.228269	0.4759758	29	27	49	35	39	49
PDK4	#DIV/0!	#DIV/0!	0.6099294	0	0	0	2	0	0
ZNF845	1.2534562	0.3259116	0.0854366	526	272	504	406	690	536
PDK3	1.4753019	0.5610102	0.0005982	260	332	319	389	462	493
ZNF844	#DIV/0!	#DIV/0!	4.15E-06	0	0	0	14	21	18
MIR3136	1.5	0.5849625	0.8175361	2	0	0	3	0	0
ZNF843	2.1111111	1.0780025	0.3458253	1	4	4	10	8	1

ZNF841	0.6315789	-0.662965	0.6356192	13	0	6	0	5	7
AL079342.3	0.5	-1	0.81753	2	0	0	0	0	1
PDK1	1.0951949	0.1311877	0.3283503	824	588	794	567	911	938
OR8H2	0	#NUM!	0.3473206	0	5	0	0	0	0
PDHX	0.9286391	-0.10681	0.6932368	1080	918	1169	774	1038	1129
MIR4458HG	0	#NUM!	0.824326	0	0	1	0	0	0
AC004704.1	0.8	-0.321928	0.8725717	1	4	0	0	0	4
ZNF839	0.8786446	-0.186648	0.5459114	420	294	555	325	406	384
RUNDC3A-AS	1.225	0.2927817	0.5620528	9	9	22	16	18	15
ZNF837	0.8156028	-0.294061	0.5827606	55	37	49	42	32	41
ZNF836	1.0625	0.0874628	0.6637836	126	80	98	81	119	123
AC007347.1	#DIV/0!	#DIV/0!	0.8030358	0	0	0	0	0	1
MIR3128	0	#NUM!	0.824326	1	0	0	0	0	0
ZNF835	0.7651934	-0.386104	0.2721789	81	136	145	64	94	119
ZNF831	0	#NUM!	0.6410557	1	1	0	0	0	0
OR8G5	0.2631579	-1.925999	0.0169259	20	24	13	5	3	7
ZNF830	1.0715909	0.0997542	0.3010467	565	513	682	546	753	587
OR8G1	0.7083333	-0.4975	0.7121407	12	0	12	0	10	7
TMEM185B	0.8569501	-0.222717	0.2109607	1461	1811	1915	1381	1597	1467
TMEM185A	0.8268229	-0.27435	0.2673822	239	234	295	200	237	198
GSKIP	1.2622519	0.3359999	0.0070459	1804	1290	1844	1690	2422	2121
AC007191.1	0.6808511	-0.554589	0.2156158	43	51	47	26	33	37
DDN-AS1	0.2222222	-2.169925	0.26351	3	4	2	0	1	1
ZNF829	1.1457143	0.1962473	0.3647433	135	71	144	108	160	133
ZNF827	1.2727273	0.3479233	0.8196719	8	1	2	0	4	10
ZNF823	1.0107335	0.0154026	0.7194716	450	283	385	345	383	402
ZNF821	1.0717489	0.0999669	0.6069551	88	35	100	91	84	64
TMEM184A	0.631068	-0.664133	0.216154	27	40	36	14	21	30
TMEM184C	1.1484067	0.1996336	0.065126	1113	975	1207	1039	1507	1238
TMEM184B	0.9524715	-0.070252	0.9110572	1913	2205	2194	1942	2052	2018
AL139158.2	0.1948052	-2.359896	0.0064162	21	30	26	12	2	1
TPX2	1.002857	0.0041159	0.577662	12195	12041	13916	11162	13530	13569
OR5BA1P	1	0	0.9861498	0	2	0	0	0	2
ZNF816	1.217695	0.2841528	0.144921	348	165	346	329	418	299
ZNF814	0.896648	-0.157386	0.8215971	136	71	151	124	105	92
ZNF813	1.2042578	0.2681442	0.1210404	663	421	654	459	850	784
AC096733.1	#DIV/0!	#DIV/0!	0.8030358	0	0	0	0	0	1
ZCCHC11	0.9280944	-0.107657	0.7418344	1771	1023	1698	942	1581	1646
ZCCHC10	1.1073874	0.14716	0.1906629	1019	734	1022	868	1207	998
ZCCHC18	1	0	0.9520161	5	4	5	4	1	9
IPO9-AS1	0.4090909	-1.289507	0.3165041	6	8	8	3	0	6
ZCCHC17	1.0518868	0.0729794	0.4642804	609	659	640	721	748	538
ZCCHC14	0.9362832	-0.094983	0.758025	729	736	795	512	713	891
ZNF808	1.0283465	0.0403264	0.7481298	241	129	265	128	260	265
ZNF806	0	#NUM!	0.824326	1	0	0	0	0	0
ZNF805	0.5282258	-0.920773	0.0181303	88	70	90	25	42	64
YWHAQP6	0	#NUM!	0.4460243	4	0	0	0	0	0
MIR3178	0	#NUM!	0.824326	1	0	0	0	0	0
ZNF800	0.9055919	-0.143067	0.6365997	532	326	519	336	476	435
MIR3176	0.3472222	-1.526069	0.0434888	22	31	19	1	8	16
AC096733.2	0.962963	-0.054448	0.9197868	47	35	26	19	43	42
ZCCHC24	1.1712963	0.2281061	0.4427448	65	66	85	44	110	99
IL9RP3	0.2894737	-1.788496	0.0922598	16	12	10	6	0	5
TEKT4P2	1.1351351	0.1828641	0.7492431	17	10	10	13	7	22

AC009502.2	1.3333333	0.4150375	0.782164	0	1	5	0	1	7
AC009502.1	1.1428571	0.1926451	0.9410281	1	6	0	1	4	3
MIR3165	0	#NUM!	0.6840298	0	0	2	0	0	0
MIR3164	0.1666667	-2.584963	0.1075959	8	2	8	1	0	2
PDPR	0.7102207	-0.493661	0.008255	1127	1058	1259	528	922	996
PDPN	1.5	0.5849625	0.6227025	2	5	7	0	12	9
AL365203.2	0.7567568	-0.402098	0.6372252	18	7	12	6	15	7
AL109804.1	#DIV/0!	#DIV/0!	0.8030358	0	0	0	0	1	0
MIR3153	0.7391304	-0.436099	0.705053	12	3	8	4	4	9
AC005042.1	0.1785714	-2.485427	0.046162	11	4	13	2	1	2
OR8J3	1	0	0.9890559	0	0	1	1	0	0
AC018558.1	0	#NUM!	0.6840298	0	0	2	0	0	0
PDP1	0.884427	-0.177185	0.5011696	389	305	327	172	397	334
HIST3H2A	1.206422	0.2707347	0.1723715	171	261	222	256	323	210
PDP2	0.7947513	-0.331425	0.0327389	2525	2431	2703	1536	2210	2341
MIR3145	#DIV/0!	#DIV/0!	0.8030358	0	0	0	0	1	0
MIR3143	#DIV/0!	#DIV/0!	0.6554062	0	0	0	0	2	0
LGALS8-AS1	0.3114754	-1.68281	0.0469851	12	15	34	4	3	12
MIR149	0.9705882	-0.043069	0.9645829	26	24	18	8	13	45
AC092032.1	4	2	0.502311	1	0	0	4	0	0
MKRN2OS	2.1363636	1.0951572	0.1287279	7	4	11	27	11	9
AC007681.1	2.1176471	1.0824622	0.0102346	28	18	22	44	50	50
AL121827.1	#DIV/0!	#DIV/0!	0.5371397	0	0	0	0	0	3
AC093110.1	0.9038462	-0.145851	0.9319284	25	4	23	14	12	21
RNU6-964P	2.1428571	1.0995357	0.5537882	4	0	3	0	15	0
AC087623.3	1.3888889	0.4739312	0.5814898	11	14	11	1	16	33
MIR3192	#DIV/0!	#DIV/0!	0.4501902	0	0	0	0	0	4
AC087623.2	0.7307692	-0.452512	0.4805318	22	19	37	10	27	20
AC087623.1	#DIV/0!	#DIV/0!	0.2106225	0	0	0	4	0	2
MIR3196	0	#NUM!	0.824326	0	1	0	0	0	0
AC087623.4	1	0	0.9890559	1	0	0	0	0	1
SPECC1	0.9176785	-0.123939	0.5183231	2742	2555	2939	2198	2767	2593
TUBG1P	0	#NUM!	0.5075476	0	0	4	0	0	0
TSGA10	1.1072664	0.1470024	0.477603	111	78	100	92	122	106
TSGA13	#DIV/0!	#DIV/0!	0.8030358	0	0	0	1	0	0
MIR186	0.3333333	-1.584963	0.2538661	8	4	6	2	4	0
MIR3189	1.1666667	0.2223924	0.7918964	4	2	6	4	3	7
AL158196.1	0.8571429	-0.222392	0.9266844	2	1	4	1	3	2
AC108472.1	#DIV/0!	#DIV/0!	0.8030358	0	0	0	0	1	0
MRFAP1L1	1.1005314	0.1382003	0.0831221	2178	2117	2668	2482	2677	2504
RNU4-5P	1	0	0.9696357	0	2	0	0	2	0
BRD3OS	1.1725998	0.2297107	0.1420785	260	354	313	344	360	383
AL158206.1	1.1453252	0.1957573	0.0844273	687	523	758	689	801	764
THRAP3	0.9541295	-0.067743	0.8729288	8207	8208	9811	6720	9236	9067
AC090950.1	4	2	0.5067587	0	0	1	4	0	0
PDXK	0.8846154	-0.176878	0.3678301	1859	2244	2319	1843	2014	1824
AC023790.2	7	2.8073549	0.2798517	0	0	1	7	0	0
PDXP	0.9583333	-0.061401	0.9930609	40	31	49	36	45	34
OR8S1	4.5	2.169925	0.3447684	2	0	0	2	7	0
AC020661.1	0.6190476	-0.691878	0.541215	13	9	20	15	11	0
AC020661.4	#DIV/0!	#DIV/0!	0.8030358	0	0	0	0	0	1
UXT-AS1	1.125	0.169925	0.8535427	4	0	4	4	0	5
AC020661.5	0	#NUM!	0.824326	1	0	0	0	0	0
AL158050.1	#DIV/0!	#DIV/0!	0.6251462	0	0	0	1	0	1

PDX1	2	1	0.8186965	0	0	1	0	0	2
AC012531.1	0.6590909	-0.601451	0.4598435	16	16	12	13	9	7
AC008175.1	0	#NUM!	0.824326	0	1	0	0	0	0
RNU4-8P	#DIV/0!	#DIV/0!	0.6251462	0	0	0	1	0	1
AL358933.1	0	#NUM!	0.3958203	2	2	0	0	0	0
HIST3H3	1	0	0.971885	1	1	0	0	2	0
ERVH48-1	1.1093882	0.1497643	0.0709791	15885	17524	18699	16390	20986	20432
AC107884.1	0.36	-1.473931	0.2588375	11	1	13	0	5	4
AC107884.2	1	0	0.9710643	0	0	4	0	4	0
AC239809.3	0.84	-0.251539	0.9621934	4	1	20	11	5	5
MIMT1	0.7795699	-0.35925	0.506447	63	30	93	43	66	36
MIR553	0	#NUM!	0.3100057	4	1	0	0	0	0
NADK2-AS1	1.4782609	0.5639009	0.4728644	12	3	8	8	9	17
AC239809.1	#DIV/0!	#DIV/0!	0.8030358	0	0	0	0	1	0
AL031577.1	0.5	-1	0.7950692	0	0	4	1	0	1
MIR559	1	0	0.9905468	2	0	1	0	0	3
PDYN	#DIV/0!	#DIV/0!	0.0960885	0	0	0	8	1	0
AC006026.1	#DIV/0!	#DIV/0!	0.5509701	0	0	0	0	3	0
MIR563	5	2.3219281	0.4416563	1	0	0	0	4	1
MIR561	#DIV/0!	#DIV/0!	0.6099294	0	0	0	2	0	0
AC006026.3	7	2.8073549	0.2979818	0	0	1	0	5	2
AC025162.2	0.0588235	-4.087463	0.0297318	7	5	5	0	1	0
AC040934.1	2.3076923	1.2064509	0.2220567	8	0	5	10	10	10
MEAF6	0.7657584	-0.385039	0.0161502	791	745	923	578	678	627
MIR567	0	#NUM!	0.5781912	0	0	3	0	0	0
MIR573	4.2857143	2.0995357	0.0485343	2	4	1	10	8	12
MIR570	1.3333333	0.4150375	0.6877742	5	5	5	4	13	3
AC239799.2	0.1428571	-2.807355	0.2042206	4	0	10	2	0	0
MIR588	0	#NUM!	0.824326	0	1	0	0	0	0
MLLT11	0.9424779	-0.085469	0.9112335	65	91	70	66	61	86
ASS1P2	0.4285714	-1.222392	0.3355924	4	4	13	2	5	2
MLLT10	1.1479336	0.1990392	0.0749461	2774	2080	2913	2309	3306	3301
TRPC6P	0	#NUM!	0.6436235	2	0	0	0	0	0
HIST2H2AB	0	#NUM!	0.2883218	0	0	7	0	0	0
LITAF	1.0824781	0.1143378	0.11591	4067	4369	5010	4451	5304	4800
MIR503	13	3.7004397	0.0997845	1	0	0	0	10	3
HIST2H2AC	1.6896552	0.7567288	0.0228186	29	70	75	95	120	79
HIST2H2BC	1.0222222	0.0317089	0.882313	22	8	15	16	5	25
AC016251.1	0.5	-1	0.7689504	0	0	4	0	1	1
HIST2H2BB	#DIV/0!	#DIV/0!	0.6554062	0	0	0	0	2	0
HIST2H2BA	#DIV/0!	#DIV/0!	0.6099294	0	0	0	2	0	0
HIST2H2BF	0.974359	-0.037475	0.9287273	7	7	25	8	10	20
HIST2H2BE	1.0054152	0.0077914	0.7673662	153	172	229	160	234	163
SUMF2	0.9602428	-0.058529	0.9891932	3796	4613	4771	4184	4362	4110
HIST2H2BD	0.5	-1	0.3022026	12	5	17	3	9	5
SUMF1	0.8582888	-0.220465	0.2938174	831	1068	1093	807	823	938
RNU4-1	1.3333333	0.4150375	0.8938282	0	3	0	1	3	0
FSTL1	1.8333333	0.8744691	0.5751443	2	4	0	10	0	1
MIR525	0	#NUM!	0.824326	0	0	1	0	0	0
FSTL4	0	#NUM!	0.4521054	1	0	3	0	0	0
MIR524	4	2	0.5525509	0	1	0	0	0	4
FSTL3	0.4354506	-1.199419	1.40E-12	1264	1540	1557	653	718	528
FSTL5	0.9555556	-0.065588	0.955761	69	71	85	56	86	73
CARNS1	5	2.3219281	0.4840007	1	0	0	0	5	0

AC016397.2	0.8709677	-0.199309	0.8617026	7	7	17	2	15	10
AL592295.2	#DIV/0!	#DIV/0!	0.5070017	0	0	0	3	0	0
AL592295.1	0.5	-1	0.8652929	0	0	2	0	0	1
MIR542	#DIV/0!	#DIV/0!	0.6099294	0	0	0	2	0	0
AL592295.4	0.6296296	-0.667425	0.6054935	6	1	20	2	9	6
AL592295.3	0.5	-1	0.6763245	3	0	3	0	3	0
ZNF699	0.8244275	-0.278535	0.5764772	49	51	31	14	36	58
ZNF697	0.9380816	-0.092215	0.7641244	861	893	1040	680	934	1007
ZNF696	0.8955375	-0.159174	0.5837948	294	320	372	271	286	326
RNU4-2	#DIV/0!	#DIV/0!	0.6554062	0	0	0	0	2	0
ZNF695	1.3897436	0.4748187	0.0372077	143	123	124	143	173	226
PECR	1.2327672	0.3019004	0.0351812	332	307	362	426	418	390
ZNF692	0.7876264	-0.344417	0.0761291	554	456	671	408	473	443
ZNF689	0.7169559	-0.480044	0.0202217	457	582	459	341	384	349
ZNF688	0.906367	-0.141833	0.8280244	61	104	102	90	75	77
ZNF687	0.8221932	-0.282451	0.1028431	1086	1387	1357	924	1118	1107
ZNF684	1.4099379	0.4956316	0.1450566	78	29	54	74	77	76
ZNF683	0.2	-2.321928	0.2449455	2	3	5	1	1	0
ZNF682	0.9898711	-0.014687	0.7750666	371	243	472	317	395	363
ZNF681	1.1592105	0.2131426	0.2499105	280	136	344	247	339	295
ZNF680	1.25039	0.3223782	0.1000284	523	242	517	400	635	568
ACAP3	1.0940083	0.1296236	0.1950695	1094	1281	1497	1571	1399	1266
ACAP2	1.0060397	0.0086872	0.7858872	1394	800	1283	816	1346	1336
ACAP1	1.056	0.0786098	0.6793715	25	37	63	48	38	46
ZNF678	0.8848921	-0.176427	0.691711	111	69	98	34	87	125
ZNF677	0.9334443	-0.099364	0.7822802	369	363	470	276	415	431
ZNF675	1.0956256	0.1317549	0.4246658	393	221	369	257	441	379
ZNF674	1.0155039	0.0221957	0.7650039	101	69	88	103	76	83
LINC02100	1.1052632	0.1443899	0.7934195	4	4	11	7	5	9
ZNF672	0.9005873	-0.151062	0.4650795	1388	1519	1690	1297	1402	1441
AL133230.1	0.48	-1.058894	0.3766672	11	4	10	0	3	9
ZNF671	0.2857143	-1.807355	0.266045	3	9	2	0	4	0
LINC02102	0	#NUM!	0.824326	0	1	0	0	0	0
ZNF670	0.8442623	-0.244237	0.4776746	181	109	198	123	151	138
PEF1	0.9074173	-0.140162	0.6177043	1047	1317	1384	1172	1239	990
THADA	0.9957139	-0.006197	0.7879161	1306	1051	1376	931	1404	1382
ACAT2	0.8250883	-0.27738	0.270016	360	335	437	300	396	238
ACAT1	0.7297211	-0.454583	0.0048976	994	956	1169	764	779	733
CNNM3-DT	1	0	0.9934006	11	13	13	1	19	17
AL365205.1	0.725	-0.463947	0.5718901	18	9	13	6	10	13
ZNF669	0.9252874	-0.112027	0.8344559	173	127	222	118	170	195
ZNF668	0.7597865	-0.396334	0.1291884	164	194	204	137	157	133
ZNF665	0.7829181	-0.353067	0.1937688	174	155	233	117	161	162
ZNF664	0.9217877	-0.117494	0.6119891	2728	2378	3128	1841	2840	2909
ZNF662	0.7176471	-0.478654	0.0809917	190	137	183	118	132	116
AC005775.1	0.5	-1	0.7699579	0	0	6	2	0	1
ZNF658	0.8591065	-0.219091	0.5926106	121	63	107	55	127	68
ZNF655	1.0196438	0.0280652	0.6247663	1457	915	1446	1032	1435	1426
ZNF654	1.0601202	0.0842279	0.5753467	414	170	414	263	392	403
ZNF653	1.2807018	0.3569345	0.2096819	59	50	62	89	61	69
ZNF652	0.9777778	-0.032421	0.9416186	732	567	726	513	782	685
SUMO4	1	0	0.9763481	1	2	3	1	3	2
NRARP	1.125	0.169925	0.819378	7	14	11	5	19	12
SUMO1	1.1547655	0.2075999	0.015087	4237	3853	5214	4531	5750	5082

SUMO3	0.973691	-0.038464	0.8969627	2219	2685	2850	2369	2868	2313
SUMO2	0.9950638	-0.007139	0.6526443	4232	4613	5336	4297	5236	4578
ZNF648	0.4848485	-1.044394	0.2630121	12	14	7	1	7	8
ZNF646	0.7554599	-0.404573	0.0096817	1015	1121	1115	696	891	869
ZNF644	1.3141534	0.3941337	0.0648299	605	297	610	413	861	713
ZNF641	1.0272277	0.038756	0.6701048	221	280	307	205	305	320
OVGP1	1.2634271	0.3373424	0.1221726	139	138	114	152	169	173
PEG3	0.6710007	-0.575614	0.0045111	21618	13658	19946	7595	13741	15718
GKN1	0	#NUM!	0.824326	0	1	0	0	0	0
C2orf92	1.1098901	0.1504168	0.6176871	23	22	46	29	30	42
C2orf91	0	#NUM!	0.5200113	2	1	0	0	0	0
ZNF639	1.1102684	0.1509085	0.2045906	630	480	604	535	677	691
ZNF638	1.4296395	0.5156514	0.0021584	2925	1471	3094	3147	4207	3354
ZNF37BP	1.2434641	0.3143648	0.1183946	218	114	280	219	259	283
NFYAP1	0.5	-1	0.6115323	7	0	1	0	1	3
C2orf81	#DIV/0!	#DIV/0!	0.8030358	0	0	0	0	1	0
C2orf83	#DIV/0!	#DIV/0!	0.1028291	0	0	0	0	0	11
C2orf82	0.88	-0.184425	0.9125347	45	10	45	45	32	11
CKMT2	0.8	-0.321928	0.5689322	27	45	53	30	29	41
C2orf88	1.0588235	0.0824622	0.9428428	9	0	8	0	11	7
ZNF629	0.8988476	-0.153852	0.7139267	223	226	332	223	206	273
ZNF628	0.6835067	-0.548973	0.0768561	215	170	288	190	131	139
ZNF627	0.850967	-0.232825	0.3172203	304	275	300	193	299	256
ZNF626	0.81	-0.304006	0.6617496	18	32	50	25	39	17
CDC37L1	1.1414503	0.1908681	0.1199115	359	329	429	372	446	457
ZNF624	0.8655462	-0.208317	0.6365534	102	52	84	54	76	76
ZNF623	0.8578256	-0.221244	0.2630281	519	550	605	374	542	520
ZNF622	0.9396802	-0.089758	0.8637448	2333	2868	3055	2691	2652	2415
ZNF621	9	3.169925	0.142306	0	0	1	5	1	3
C2orf72	1.5087594	0.5933627	2.51E-06	843	990	964	1296	1479	1445
C2orf78	0.7222222	-0.469485	0.7353278	7	5	6	7	2	4
AC008663.3	#DIV/0!	#DIV/0!	0.4501902	0	0	0	0	0	4
AC008663.2	0.0967742	-3.369234	0.0170493	20	6	5	0	0	3
AC079880.1	1.1052632	0.1443899	0.7726889	13	10	15	10	9	23
AC008663.1	0	#NUM!	0.5436524	2	0	1	0	0	0
ZNF619	6	2.5849625	0.2850678	1	0	0	3	1	2
C2orf74	0.5128205	-0.963474	0.2654468	14	10	15	2	11	7
ZNF618	0.5762712	-0.79518	0.0296537	77	69	90	30	53	53
C2orf76	1.0972569	0.1339013	0.4550323	124	114	163	108	186	146
ZNF616	1.0318665	0.0452563	0.7789268	288	150	221	149	304	227
ZNF615	1.1284585	0.1743534	0.4535458	206	77	223	141	261	169
ZNF614	2	1	0.6543984	1	0	1	2	0	2
ZNF613	0.8125	-0.29956	0.4042527	117	74	113	65	107	75
ZNF611	1.0860633	0.1191082	0.5003511	711	461	629	377	748	831
AC105053.1	#DIV/0!	#DIV/0!	0.242062	0	0	0	6	0	0
ZNF610	0.7037037	-0.50696	0.3368087	27	35	46	17	33	26
LINC02044	#DIV/0!	#DIV/0!	0.6099294	0	0	0	2	0	0
AC141557.2	1.2692308	0.3439544	0.7105285	18	5	3	6	12	15
AC141557.1	1.2727273	0.3479233	0.524905	19	13	12	25	9	22
LINC02041	0.95	-0.074001	0.8917051	33	38	29	13	41	41
LINC02043	0.6666667	-0.584963	0.7710192	4	1	4	0	0	6
SRGAP2D	1.0441176	0.0622843	0.8633006	59	53	24	40	45	57
C2orf66	3.6666667	1.8744691	0.2683803	0	0	3	4	5	2
SRGAP2C	1.0018293	0.0026367	0.7935314	572	475	593	400	656	587

C2orf69	1.3070132	0.3862738	0.0177353	820	510	709	677	1032	956
C2orf68	1.0480452	0.067701	0.3139358	1232	1309	1705	1551	1496	1403
ZNF729	0	#NUM!	0.824326	1	0	0	0	0	0
ZNF727	0.6727273	-0.571906	0.4354074	23	11	21	11	13	13
ZNF726	0.8424069	-0.247411	0.4687652	121	98	130	62	139	93
ZNF724	0.6896552	-0.536053	0.152666	110	71	109	41	64	95
ZNF723	#DIV/0!	#DIV/0!	0.8030358	0	0	0	1	0	0
ZNF721	1.1354467	0.18326	0.3015395	279	152	263	212	302	274
ZNF720	0.7853261	-0.348636	0.1675869	240	196	300	182	204	192
LINC02037	0.4210526	-1.247928	0.3732313	11	1	7	0	7	1
PELO	1.133645	0.180969	0.0769879	1562	1767	1804	1976	1982	1861
SRGAP2B	0.7383721	-0.43758	0.1150156	286	279	295	122	203	310
LINC02036	0.8461538	-0.241008	0.6607732	42	34	28	27	41	20
LINC02035	0.8453977	-0.242298	0.3593756	430	257	432	262	365	319
PHYKPL	1.0041374	0.0059566	0.646407	779	733	905	733	924	770
LINC02031	0	#NUM!	0.824326	0	1	0	0	0	0
C2orf50	1.2631579	0.337035	0.7247349	3	10	6	4	13	7
THAP2	0.9672897	-0.04798	0.9982132	74	62	78	52	76	79
THAP1	1.171223	0.2280158	0.1805391	236	176	283	206	339	269
THAP4	1.0183778	0.0262729	0.5383265	1133	1425	1523	1360	1495	1301
THAP3	1.0872541	0.1206891	0.3585421	282	425	462	462	465	344
THAP6	1.0477419	0.0672834	0.5585579	302	202	271	237	297	278
THAP5	0.8318294	-0.26564	0.3487007	460	251	508	258	423	333
ZNF718	0.7889447	-0.342004	0.2980741	212	123	262	100	220	151
THAP8	0.7760943	-0.365696	0.1790693	170	192	232	100	204	157
ZNF717	1	0	0.9890559	1	0	0	1	0	0
THAP7	0.9236364	-0.114603	0.7532249	582	678	665	635	646	497
ZNF716	1.0967742	0.1332665	0.7998799	13	6	12	10	13	11
C2orf54	0.397351	-1.331514	0.0077544	55	49	47	28	17	15
THAP9	1.4660767	0.5519606	0.0465213	137	56	146	122	207	168
ZNF714	1.0383838	0.0543398	0.6085496	904	633	938	559	937	1074
ZNF713	0.7602041	-0.395541	0.3577287	65	47	84	34	53	62
ZNF711	0.5	-1	0.8638172	0	0	2	1	0	0
ZNF710	0.7506053	-0.413874	0.0701973	334	481	424	211	363	356
LINC02026	0.5	-1	0.3999509	8	10	8	4	0	9
LINC02028	1.2	0.2630344	0.8801943	5	0	0	3	1	2
LINC02068	#DIV/0!	#DIV/0!	0.5509701	0	0	0	0	3	0
AC004947.1	0	#NUM!	0.824326	0	0	1	0	0	0
LINC02063	0	#NUM!	0.3473206	0	5	0	0	0	0
LINC02062	2.3333333	1.2223924	0.4829829	0	6	0	8	6	0
LINC02065	1.3714286	0.4556795	0.4494977	15	8	12	15	13	20
AC004947.2	0	#NUM!	0.6436235	2	0	0	0	0	0
LINC02064	1.1428571	0.1926451	0.958577	2	4	1	0	8	0
LINC02060	0.5	-1	0.8652929	0	0	2	0	1	0
APH1A	0.9444115	-0.082512	0.8616611	2418	2978	3041	2643	2848	2477
ZNF709	0	#NUM!	0.3608108	0	1	4	0	0	0
ZNF708	0.9482201	-0.076706	0.9152183	389	178	360	218	359	302
APH1B	1.1827815	0.2421835	0.0803952	485	515	510	466	655	665
ZNF707	0.8504924	-0.23363	0.353206	315	375	427	323	334	293
CDC37P1	0.5	-1	0.6946839	0	4	0	0	1	1
ZNF706	0.9957256	-0.00618	0.6380618	1411	1515	1987	1545	1796	1551
C2orf40	0	#NUM!	0.824326	0	0	1	0	0	0
ZNF704	1.1196388	0.1630334	0.1730651	746	608	861	628	878	974
C2orf42	1.1594203	0.2134036	0.1511197	194	245	320	293	291	296

ZNF703	0.8079096	-0.307734	0.364384	101	123	130	99	95	92
ZNF701	0.814238	-0.296477	0.2828473	350	205	344	163	287	282
ZNF700	0.997555	-0.003532	0.7511548	270	164	384	241	322	253
CDC37P2	0	#NUM!	0.3052077	1	4	0	0	0	0
C2orf49	1.1845638	0.2443559	0.015203	1180	1024	1372	1239	1561	1436
C2orf48	0.7939394	-0.332899	0.6560797	39	47	79	70	31	30
LINC02059	1.3333333	0.4150375	0.787987	0	0	3	4	0	0
LINC02056	0	#NUM!	0.5172237	1	2	0	0	0	0
LINC02055	0	#NUM!	0.5387425	3	0	0	0	0	0
AC018809.2	0.6880734	-0.539366	0.4085358	38	15	56	11	24	40
INTS1	0.8871075	-0.172819	0.3353945	2216	2578	2753	2065	2439	2191
INTS3	0.8138058	-0.297244	0.068641	1445	1616	1821	1025	1448	1500
LINC02054	#DIV/0!	#DIV/0!	0.6554062	0	0	0	0	2	0
INTS2	0.9490323	-0.075471	0.8909649	653	350	547	366	512	593
AL590002.1	0	#NUM!	0.6385096	0	2	0	0	0	0
INTS5	0.9582801	-0.061481	0.9898497	721	768	860	769	785	697
INTS4	0.7758737	-0.366106	0.0123419	1424	1466	1545	985	1236	1220
PEMT	0.9518828	-0.071144	0.9779303	128	165	185	158	181	116
INTS7	0.9104478	-0.135352	0.5760794	660	519	630	396	642	609
INTS6	1.2571791	0.3301901	0.0276892	883	573	912	750	1182	1045
AC018809.1	1.6538462	0.725825	0.3649692	12	3	11	5	23	15
INTS9	0.8568873	-0.222823	0.3408706	345	365	408	291	311	356
INTS8	1.0620994	0.0869187	0.4611988	946	633	917	632	974	1045
LINC02048	1.3333333	0.4150375	0.787987	0	0	3	4	0	0
MINCR	0.7103825	-0.493332	0.2360072	49	66	68	40	55	35
LINC02001	1.029932	0.0425491	0.5800963	207	239	289	251	274	232
TMEM151A	0	#NUM!	0.6840298	0	0	2	0	0	0
LINC02000	2.875	1.523562	0.2528204	6	0	2	1	10	12
LINC02002	#DIV/0!	#DIV/0!	0.4501902	0	0	0	0	0	4
TMEM151B	#DIV/0!	#DIV/0!	0.8030358	0	0	0	0	0	1
PEPD	0.9324563	-0.100892	0.7538041	1556	1735	1802	1581	1624	1544
ACADL	#DIV/0!	#DIV/0!	0.6554062	0	0	0	0	2	0
AL365204.1	1.25	0.3219281	0.856132	0	3	1	3	2	0
ACADM	1.086545	0.1197479	0.2615213	1107	743	1108	908	1159	1147
ACADS	0.9207792	-0.119073	0.8916752	261	210	299	293	204	212
AC018413.1	1.5909091	0.6698514	0.2618467	5	21	18	17	23	30
ACAD8	0.8801002	-0.18426	0.3857906	805	919	1070	793	859	807
TMEM150B	0.3809524	-1.392317	0.2796449	4	9	8	5	2	1
TMEM150A	1.0438982	0.061981	0.4563377	328	378	433	377	410	402
ACAD9	0.720758	-0.472413	0.00096	1431	1486	1674	1015	1117	1177
MINK1	0.9590111	-0.060381	0.9224121	4468	5532	5492	4262	5358	5237
AL365194.1	#DIV/0!	#DIV/0!	0.6099294	0	0	0	2	0	0
TMEM150C	#DIV/0!	#DIV/0!	0.8030358	0	0	0	1	0	0
KXD1	0.9966059	-0.004905	0.6894721	680	792	885	783	818	748
C2orf16	0.0769231	-3.70044	0.1105693	0	4	9	0	1	0
C2orf15	1.605042	0.6826111	0.0443747	34	44	41	64	70	57
LINC02025	#DIV/0!	#DIV/0!	0.8030358	0	0	0	1	0	0
LINC02021	1.9142857	0.9368062	0.1088731	8	11	16	17	27	23
DOT1L	0.9243173	-0.11354	0.6269981	1972	2357	2555	1855	2309	2199
PERP	1.0463662	0.0653879	0.2525278	10349	10181	11778	9830	12485	11491
PES1	0.8048816	-0.313151	0.0682018	3741	4656	5164	3695	3853	3367
LINC02019	0.4166667	-1.263034	0.4061323	4	6	2	0	2	3
LINC02016	0	#NUM!	0.824326	1	0	0	0	0	0
LINC02018	1.1234568	0.1679446	0.5943011	26	18	37	38	28	25

AC010627.1	3	1.5849625	0.660331	0	0	1	0	2	1
LINC02014	0.6037736	-0.72792	0.3565564	20	18	15	11	4	17
AC044797.1	0.3214286	-1.63743	0.1468444	9	8	11	0	6	3
PER2	1.1146015	0.156528	0.2119021	552	567	600	491	680	745
PER1	0.8841762	-0.177594	0.6148993	167	189	257	169	219	154
GFI1B	1.6666667	0.7369656	0.6746006	1	4	1	0	3	7
PER3	1.516129	0.6003925	0.3731076	8	7	16	8	12	27
AC009501.1	0.4941176	-1.017074	0.2714088	29	1	55	15	17	10
ESYT3	#DIV/0!	#DIV/0!	0.3981469	0	0	0	4	0	0
AC112198.1	#DIV/0!	#DIV/0!	0.8030358	0	0	0	0	0	1
ESYT2	0.8910393	-0.166439	0.3511187	3117	2708	3527	2169	3127	3037
ESYT1	0.8598826	-0.217788	0.1656031	2119	2367	2501	1797	2163	2048
LINC02005	#DIV/0!	#DIV/0!	0.8030358	0	0	0	0	1	0
AC112198.2	0	#NUM!	0.5337954	0	3	0	0	0	0
LINC02004	1.4285714	0.5145732	0.6760421	2	3	2	8	0	2
ACAA2	0.9159869	-0.126601	0.59489	1124	1173	1381	1057	1239	1073
TRAC	#DIV/0!	#DIV/0!	0.8030358	0	0	0	0	0	1
ACAA1	0.9010368	-0.150342	0.6432017	933	1048	1202	1080	964	824
EOMES	0.9667897	-0.048726	0.8597429	73	82	116	127	54	81
AC092427.1	3	1.5849625	0.6253252	0	0	1	3	0	0
AL157713.1	1.8333333	0.8744691	0.3978886	7	11	0	9	12	12
FAM92A	0.9536424	-0.06848	0.999901	198	153	253	179	218	179
AC146944.3	0.8870968	-0.172837	0.8208055	26	16	20	15	21	19
AC146944.2	1	0	0.9837228	2	0	0	0	1	1
AC146944.4	0.6048387	-0.725378	0.1575713	30	40	54	27	26	22
AC146944.1	1.375	0.4594316	0.6907864	2	0	6	5	0	6
AL117381.1	2	1	0.6081043	1	2	0	3	1	2
AC108475.1	0.5348837	-0.902703	0.2660432	20	17	6	7	10	6
ACACB	0.8663239	-0.207022	0.4086865	243	253	282	187	245	242
ACACA	0.899279	-0.153159	0.4029231	4278	4519	4656	3104	4293	4701
ARMC10P1	0.8275862	-0.273018	0.7727493	7	10	12	1	18	5
FAM95C	2.2	1.1375035	0.4221175	1	0	4	4	2	5
AL158207.1	#DIV/0!	#DIV/0!	0.4501902	0	0	0	0	0	4
IL17REL	#DIV/0!	#DIV/0!	0.4737294	0	0	0	0	4	0
MARK2P8	0.8333333	-0.263034	0.8855417	3	3	0	0	0	5
MARK2P9	#DIV/0!	#DIV/0!	0.6554062	0	0	0	0	2	0
LINC02089	#DIV/0!	#DIV/0!	0.8030358	0	0	0	1	0	0
LINC02084	#DIV/0!	#DIV/0!	0.8030358	0	0	0	0	0	1
TREH	0.65625	-0.607683	0.6785577	7	2	23	9	12	0
LINC02086	0.3333333	-1.584963	0.4849187	1	0	11	3	1	0
LINC02081	0.8076923	-0.308122	0.8214956	13	3	10	10	6	5
LINC02080	0	#NUM!	0.5436524	2	0	1	0	0	0
ZNF385C	0.7601351	-0.395672	0.2823629	68	110	118	74	80	71
ZNF385B	1.6	0.6780719	0.475625	5	9	6	2	16	14
HORMAD1	#DIV/0!	#DIV/0!	0.4242335	0	0	0	0	3	1
ZNF385A	0.8815287	-0.181921	0.4197871	1725	2056	2499	1868	1896	1772
BCKDHA	0.3076923	-1.70044	0.3594417	6	3	4	4	0	0
KRT222	0.7272727	-0.459432	0.7970541	10	1	0	3	0	5
LINC02079	0	#NUM!	0.5781912	0	0	3	0	0	0
LINC02073	#DIV/0!	#DIV/0!	0.6099294	0	0	0	2	0	0
LINC02076	0	#NUM!	0.1701088	3	0	5	0	0	0
BCKDHB	0.9471572	-0.078324	0.913015	494	407	594	360	453	603
PEX2	0.8311758	-0.266774	0.1700113	670	531	653	460	540	541
PEX1	0.8597108	-0.218077	0.2534435	880	764	915	562	798	840

GPCPD1	0.8363687	-0.257789	0.177309	1244	942	1383	746	1152	1087
TCTE3	0.5471698	-0.869939	0.0333832	88	86	91	50	72	23
TCTE1	1.56	0.641546	0.4437989	9	3	13	23	16	0
TRDN	7	2.8073549	0.307745	0	0	1	0	6	1
PEX7	0.8281829	-0.271979	0.2615981	290	233	286	199	264	207
HIST4H4	0.8513514	-0.232173	0.6921971	26	28	20	13	27	23
PEX3	0.9656582	-0.050415	0.9310629	431	330	462	379	382	420
PEX6	0.9813176	-0.027208	0.9008506	344	311	362	270	340	388
PEX5	0.6118699	-0.708703	8.70E-06	1094	1401	1532	751	823	890
FAM96B	0.915005	-0.128148	0.7140583	1205	1234	1573	1315	1301	1055
FAM96A	0.8628631	-0.212796	0.1986823	1209	1224	1388	995	1189	1113
AL359764.1	0	#NUM!	0.5781912	0	0	3	0	0	0
MIR498	#DIV/0!	#DIV/0!	0.8030358	0	0	0	0	0	1
MARVELD2	0.8040841	-0.314582	0.0399863	1568	1533	1845	1033	1493	1451
MARVELD1	1.1417843	0.1912902	0.0618074	956	1025	1348	1278	1348	1175
AL512283.1	1.375	0.4594316	0.4917756	5	4	15	24	5	4
MARVELD3	0.674552	-0.567998	0.0003779	825	960	1005	566	637	679
MIR491	#DIV/0!	#DIV/0!	0.8030358	0	0	0	0	0	1
LINC02096	#DIV/0!	#DIV/0!	0.5070017	0	0	0	3	0	0
GOLPH3	0.9805089	-0.028397	0.8775764	8704	8608	9572	7118	9595	9647
GLB1	0.7909786	-0.33829	0.0210854	2383	2840	2891	1914	2321	2183
FAM98C	0.8652668	-0.208783	0.5384414	331	371	441	395	335	259
RANGRF	0.4615385	-1.115477	0.5184321	5	0	8	2	2	2
FAM98B	0.9568706	-0.063604	0.9244096	2653	2243	3080	2009	2937	2686
FAM98A	1.02823	0.040163	0.4057704	3134	2965	3607	2796	3683	3501
TRIR	1.1378645	0.1863287	0.1132779	2674	3402	3767	4149	4038	3013
TRIO	0.9358443	-0.09566	0.7073464	9528	8972	10227	5909	9719	11256
GUSBP11	4	2	0.5532124	1	0	0	0	0	4
GLDC	1.1624225	0.2171345	0.0137471	15862	17375	18677	17310	21778	21258
TRIL	0.7	-0.514573	0.384066	27	39	24	25	17	21
GLDN	1.0472973	0.066671	0.7901453	47	47	54	30	56	69
GLE1	0.9293807	-0.105658	0.6651108	1933	2136	2374	1794	2278	1916
MILR1	#DIV/0!	#DIV/0!	0.0074651	0	0	0	3	3	12
AC008568.1	0.4117647	-1.280108	0.3826333	14	1	2	1	6	0
AC142384.1	1.3636364	0.447459	0.6519204	2	1	8	9	6	0
GLCE	0.966805	-0.048703	0.9227078	257	170	296	214	237	248
PIH1D1	1.2148245	0.2807479	0.015666	1811	1972	2315	2727	2390	2291
AC239798.2	1.1612903	0.2157287	0.7403381	17	6	8	11	12	13
AC239798.1	0.7272727	-0.459432	0.7492338	4	3	4	1	6	1
PIH1D3	0.875	-0.192645	0.9320462	7	0	9	2	4	8
TYRO3	1.2859811	0.3628695	0.0004958	3299	3872	4071	4582	5139	4736
PIH1D2	0.9290323	-0.106199	0.9188143	55	30	70	38	61	45
AC105052.2	1.8421053	0.8813555	0.2557036	4	2	13	15	10	10
GLG1	0.7528188	-0.409625	0.0022779	4882	5415	5756	3468	4190	4427
MIR454	#DIV/0!	#DIV/0!	0.6554062	0	0	0	0	2	0
CCDC74BP1	0	#NUM!	0.5200113	2	1	0	0	0	0
AC140479.4	0	#NUM!	0.6436235	2	0	0	0	0	0
AC140479.3	#DIV/0!	#DIV/0!	0.5371397	0	0	0	0	0	3
TRMU	0.9321464	-0.101372	0.7841271	1596	1546	1913	1588	1672	1452
TRMO	1.2756286	0.3512084	0.0111438	303	327	404	419	462	438
GLI1	1	0	0.9960906	0	5	0	3	2	0
PFAS	0.8968818	-0.15701	0.4101735	3203	3614	3734	2474	3259	3730
GLI3	0.6017675	-0.732722	0.0001966	1163	1248	1323	453	841	953
GLI2	1.5	0.5849625	0.7809202	2	1	1	0	4	2

GLI4	1.1208333	0.1645718	0.4493281	66	88	86	93	80	96
PITPNA-AS1	0.5833333	-0.777608	0.6502524	1	8	3	4	0	3
GOLGA2P10	0.8653846	-0.208587	0.6198259	87	77	96	75	86	64
HSFX1	#DIV/0!	#DIV/0!	0.3597364	0	0	0	0	0	5
HSFX4	#DIV/0!	#DIV/0!	0.6099294	0	0	0	2	0	0
HSFX3	3	1.5849625	0.6944949	0	0	1	0	3	0
AL121574.1	0.9375	-0.093109	0.9867731	10	2	4	6	1	8
ZNF599	1.0346154	0.0490945	0.7006284	92	54	114	75	90	104
ZNF598	0.9338655	-0.098713	0.8001085	990	1192	1326	1053	1087	1136
ZNF597	0.8667366	-0.206334	0.467068	347	246	360	208	271	347
ZNF596	0.7898089	-0.340424	0.5633399	54	27	76	45	45	34
ZNF595	0.9951241	-0.007052	0.7966145	852	555	849	599	875	771
ZNF594	1.0352798	0.0500207	0.7112625	312	183	327	174	372	305
ZNF593	0	#NUM!	0.5622741	0	1	2	0	0	0
ZNF592	0.8545023	-0.226844	0.1698524	1739	1997	2161	1485	1734	1820
AC006517.1	2.3333333	1.2223924	0.5353122	0	0	3	3	4	0
AC091931.1	#DIV/0!	#DIV/0!	0.4155775	0	0	0	0	2	2
ZNF589	0.808542	-0.306605	0.1472518	594	642	801	583	563	501
ZNF587	0.7214022	-0.471124	0.0116102	777	637	754	388	552	624
ZNF586	1.2421227	0.3128077	0.0335841	382	406	418	387	558	553
ZNF584	0.8635171	-0.211703	0.5427408	102	124	155	81	137	111
ZNF581	0.9505495	-0.073166	0.9744855	460	432	564	479	519	386
ZNF580	1.0023502	0.0033866	0.730116	216	289	346	271	321	261
LAPTM5	0.875	-0.192645	0.8433832	16	9	7	10	12	6
OR8B10P	0.6666667	-0.584963	0.8308055	3	0	0	0	2	0
SULF1	1.1111111	0.1520031	0.8605078	3	0	6	2	3	5
SULF2	0.7824773	-0.353879	0.3258399	111	83	137	77	78	104
MIR421	0	#NUM!	0.5075476	0	0	4	0	0	0
AL158209.1	1.3333333	0.4150375	0.8933112	0	2	1	0	4	0
ZNF579	1.2091097	0.2739452	0.1743034	165	175	143	210	191	183
ZNF578	0.9782609	-0.031709	0.9951473	34	20	38	16	55	19
ZNF576	1.0470348	0.0663093	0.5742041	139	168	182	157	181	174
ZNF575	1.0769231	0.1069152	0.906126	4	7	2	8	6	0
ZNF574	0.7240618	-0.465815	0.0046002	692	763	810	503	564	573
ZNF573	0.7711443	-0.374927	0.4041531	71	51	79	39	41	75
PHLDB1	0.7254779	-0.462996	0.009479	847	1139	1205	741	773	801
ZNF571	0.8125	-0.29956	0.6396094	38	28	78	36	41	40
ZNF570	0.8925373	-0.164016	0.7357319	138	61	136	73	114	112
ZNF569	1.1401515	0.1892256	0.4876143	102	54	108	64	144	93
ZNF568	1.0042373	0.0061002	0.8813281	82	74	80	60	96	81
AC119396.1	2.5714286	1.3625701	0.2870752	3	0	4	8	9	1
ZNF567	0.9189189	-0.121991	0.7437615	313	187	314	184	306	258
PHLDA2	1.2763158	0.3519853	0.1537037	71	67	90	100	110	81
ZNF566	0.9308756	-0.10334	0.8763908	240	139	272	171	214	221
PHLDA1	0.6339869	-0.657475	0.1818827	48	34	71	19	32	46
ZNF565	0.6906475	-0.533979	0.3152101	45	28	66	31	43	22
ZNF564	0.5714286	-0.807355	0.5721345	1	8	5	0	4	4
PHLDA3	0.7212083	-0.471512	0.0034796	13259	16038	17279	11924	11488	10179
ZNF563	14	3.8073549	0.0478853	0	0	1	5	6	3
ZNF562	0.7891051	-0.341711	0.1324175	474	320	491	268	357	389
ZNF561	0.8353511	-0.259545	0.2052455	574	551	527	326	563	491
ZNF560	0	#NUM!	0.4005425	3	1	0	0	0	0
AC083855.1	0	#NUM!	0.824326	1	0	0	0	0	0
PRPF38AP1	0	#NUM!	0.6840298	0	0	2	0	0	0

PRPF38AP2	#DIV/0!	#DIV/0!	0.4242335	0	0	0	0	3	1
ZNF559	1.117506	0.1602826	0.4582016	174	72	171	146	198	122
ZNF558	1.2293869	0.297939	0.0624679	337	229	380	334	379	450
ZNF557	0.7614108	-0.393253	0.184269	136	153	193	89	121	157
ZNF556	0.6547085	-0.611075	0.0375321	133	168	145	61	117	114
PHLDB3	1.4662772	0.5521579	0.0003836	483	618	782	1020	930	811
ZNF555	1.1831325	0.2426117	0.2644093	139	131	145	110	173	208
PHLDB2	1.045089	0.0636258	0.5640779	1538	934	1631	945	1544	1799
ZNF554	0.8428246	-0.246696	0.4407543	125	167	147	121	141	108
ZNF552	0.6846767	-0.546505	0.0005895	733	726	799	432	556	558
ZNF551	0.7353659	-0.443466	0.0315763	622	459	559	281	498	427
ZNF550	0.4545455	-1.137504	0.4747381	2	5	4	0	3	2
AC117834.1	1	0	0.9890559	0	0	1	0	0	1
GLO1	1.0868627	0.1201697	0.0964424	11309	10352	13095	10790	14220	12765
AC104463.2	1.4761905	0.5618789	0.4814206	10	5	6	9	13	9
ZNF549	0	#NUM!	0.2347887	0	1	6	0	0	0
ZNF548	0.9546926	-0.066892	0.9617838	82	73	154	79	99	117
ZNF547	0.832	-0.265345	0.6929065	34	31	60	38	32	34
ZNF546	1.7407407	0.7997013	0.2511942	8	9	10	9	15	23
ZNF544	0.9215988	-0.117789	0.6396699	872	752	978	685	896	817
ZNF543	0.7616162	-0.392864	0.1773374	138	183	174	79	173	125
ZNF541	0	#NUM!	0.824326	0	1	0	0	0	0
ZNF540	0.8556701	-0.224873	0.8087846	32	18	47	34	28	21
GLMN	1.0588638	0.082517	0.46377	540	319	602	436	623	488
GLMP	0.7898894	-0.340277	0.0631847	919	1102	1144	852	878	770
SPIRE2	1.3140665	0.3940383	0.0041608	648	497	810	884	893	792
SPIRE1	0.8315412	-0.26614	0.4653212	72	104	103	62	92	78
ZNF536	0.8181818	-0.289507	0.839275	6	9	18	10	4	13
ZNF532	0.8586957	-0.219781	0.6306838	59	73	52	51	73	34
ZNF530	0.7903066	-0.339516	0.1083061	338	331	342	222	279	298
C19orf67	0.6734694	-0.570316	0.4721444	13	12	24	5	19	9
C19orf66	2.195122	1.1343011	0.0295783	11	15	15	34	30	26
INSL6	1.0324074	0.0460124	0.621973	128	116	188	135	139	172
GM2A	0.9075182	-0.140002	0.4420056	22120	25250	26504	20657	23938	22447
ZNF529	0.904814	-0.144307	0.6595748	347	220	347	188	308	331
SLC26A4-AS1	0	#NUM!	0.5387425	3	0	0	0	0	0
ZNF528	0.9454545	-0.08092	0.9646826	14	10	31	17	14	21
ZNF527	0.7478261	-0.419225	0.2422391	119	82	144	85	108	65
ZNF526	0.7696386	-0.377747	0.0327781	598	738	739	465	587	545
ZNF525	1.1774194	0.2356282	0.196167	636	301	675	461	762	675
ZNF524	1.1018364	0.13991	0.4283753	154	250	195	260	186	214
INSL3	#DIV/0!	#DIV/0!	0.4909737	0	0	0	2	0	1
ZNF521	2	1	0.8186965	0	0	1	0	0	2
INSL4	3.3940887	1.7630243	1.27E-09	80	33	90	263	214	212
C19orf73	0.8452381	-0.24257	0.7706268	20	23	41	20	17	34
C19orf70	0.8811122	-0.182602	0.5732706	313	371	359	352	313	254
C19orf71	0.5316456	-0.911463	0.1304849	24	33	22	12	19	11
ZNF519	1.0932642	0.1286422	0.4705333	152	96	138	124	146	152
ZNF517	0.7893175	-0.341322	0.3097427	83	132	122	74	96	96
ZNF516	0.7173638	-0.479223	0.0573786	207	288	294	128	209	229
ZNF514	0.904918	-0.144141	0.6042631	573	412	540	429	486	465
ZNF513	0.9621514	-0.055664	0.9645969	288	336	380	325	347	294
ZNF512	0.5980392	-0.741688	0.1737728	26	37	39	20	26	15
SMPDL3B	1.1500816	0.2017362	0.3299475	136	271	206	239	264	202

ZNF511	1.3461538	0.4288433	0.1448549	35	77	96	103	91	86
SMPDL3A	1.0603144	0.0844921	0.3024772	1077	885	1155	938	1234	1133
ZNF510	1.0867888	0.1200716	0.4921022	406	214	417	236	454	437
C19orf84	1.875	0.9068906	0.3039554	5	8	3	11	10	9
AC005534.1	1	0	0.9767213	3	0	0	0	2	1
C19orf81	1.0754717	0.1049696	0.7481492	37	28	41	26	57	31
GLS2	0.75	-0.415037	0.8160165	7	4	9	11	3	1
TYRP1	0.4705882	-1.087463	0.0887067	32	37	16	4	19	17
GLRX	0.6750552	-0.566923	0.0011458	766	694	805	519	553	457
BACH1-IT2	6.5	2.7004397	0.1150542	0	1	1	9	1	3
BACH1-IT1	0.5833333	-0.777608	0.4536546	25	10	1	6	2	13
ZNF507	1.0186757	0.0266949	0.7603352	1216	690	1039	658	1200	1142
ZNF506	0.8894691	-0.168984	0.5726431	446	262	441	221	424	377
ZNF503	1.3174603	0.3977595	0.4820045	4	24	35	16	43	24
ZNF501	0.8902954	-0.167644	0.7614229	81	48	108	52	87	72
ZNF500	0.7189365	-0.476064	0.0086826	505	627	711	407	469	449
AC009407.1	5.5	2.4594316	0.1784597	0	0	2	9	1	1
AC104619.3	0	#NUM!	0.824326	1	0	0	0	0	0
BX276092.9	#DIV/0!	#DIV/0!	0.8030358	0	0	0	0	0	1
PFKL	1.0771613	0.1072344	0.2015074	9220	11498	11889	11861	12341	10921
GLRB	0.7916667	-0.337035	0.7905671	2	10	12	4	5	10
BX276092.2	0	#NUM!	0.6385096	0	2	0	0	0	0
PFKM	0.9034908	-0.146418	0.4319093	1773	2021	2050	1527	1999	1754
olytic process t	#DIV/0!	#DIV/0!							
):0003872|6-ph	#VALUE!	#VALUE!	ofructokina:	pathways|Car	ructokinase, muscle				
PFKP	0.9838736	-0.023455	0.7673692	15950	19112	20499	17187	19446	18032
AL021937.4	0	#NUM!	0.2515625	5	0	1	0	0	0
GLTP	1.0815752	0.113134	0.3681888	227	203	281	216	265	288
AL021937.2	0	#NUM!	0.1851375	8	0	0	0	0	0
AL021937.1	0.4	-1.321928	0.4558467	3	7	0	0	1	3
C19orf25	0.9822785	-0.025796	0.8126117	303	403	479	395	417	352
C19orf24	0.9356201	-0.096005	0.9636364	515	548	832	661	613	499
ZNF609	0.8646305	-0.209844	0.2771169	1732	1776	1796	1093	1596	1897
ZNF608	0.8	-0.321928	0.1431598	545	483	632	282	505	541
RUSC2	0.7392947	-0.435779	0.0075656	982	1028	1166	592	863	893
ZNF607	0.9768467	-0.033796	0.9448905	351	212	344	216	335	335
RUSC1	0.9630872	-0.054262	0.9666843	1032	1251	1293	1154	1213	1077
ZNF606	0.8838951	-0.178053	0.5622703	291	201	309	212	253	243
ZNF605	0.9986486	-0.001951	0.8122074	267	179	294	196	310	233
AC026877.1	0.5	-1	0.6672322	3	1	8	6	0	0
ZNF600	1.1560137	0.2091586	0.1538273	539	356	560	441	686	555
GLUL	0.8282752	-0.271818	0.0528019	9385	10966	11578	7711	9598	9137
C19orf38	0.84	-0.251539	0.840852	3	8	14	3	11	7
AC010722.1	1.6666667	0.7369656	0.7589961	2	0	1	0	0	5
PFN4	0.2972973	-1.750022	0.1180457	21	1	15	4	5	2
PFN1	1.0296832	0.0422005	0.4207978	10562	11623	13795	13718	12740	10590
AC013267.1	0	#NUM!	0.824326	1	0	0	0	0	0
PFN2	1.0047506	0.0068374	0.6244824	875	906	1166	837	1114	1010
TMEM161B	0.9242726	-0.11361	0.7639961	938	578	993	644	817	858
TMEM161A	0.8002114	-0.321547	0.2108853	498	560	834	558	485	471
C19orf48	0.9445794	-0.082256	0.9096718	3494	3703	4333	3798	3880	3213
C19orf44	0.8164557	-0.292553	0.2748364	196	198	238	139	167	210
C19orf47	0.9813118	-0.027216	0.8021052	707	948	1074	863	916	899
WFDC13	0	#NUM!	0.824326	1	0	0	0	0	0

C19orf54	1.0019142	0.002759	0.6811999	773	910	929	849	933	835
C19orf53	1.0547912	0.0769575	0.3063459	2334	2785	3167	3081	3073	2586
AC144548.2	#DIV/0!	#DIV/0!	0.8030358	0	0	0	0	0	1
C19orf57	0.8032787	-0.316027	0.4385927	53	70	60	32	66	49
CEP57L1P1	1.2307692	0.2995603	0.7662421	3	7	3	5	3	8
IQANK1	0.9859772	-0.020374	0.8294888	679	789	814	662	813	775
KRT24	1.22	0.2868811	0.6356759	26	9	15	10	19	32
KRT23	3.1039604	1.6341102	8.03E-37	491	494	631	1592	1866	1558
AL139396.1	0.6486486	-0.624491	0.2773361	44	32	35	6	26	40
AC004706.1	0.8064516	-0.31034	0.7419304	14	7	10	2	8	15
INSRR	0	#NUM!	0.5387425	3	0	0	0	0	0
KRT34	#DIV/0!	#DIV/0!	0.1436754	0	0	0	2	1	4
AP001462.1	0.8275862	-0.273018	0.8442209	12	0	17	3	15	6
KRT39	0.75	-0.415037	0.8099985	2	3	3	1	2	3
KRT38	5.5	2.4594316	0.187978	0	1	1	1	4	6
AC004706.4	0.7115385	-0.490986	0.5028136	17	16	19	3	19	15
AC004706.3	0.3333333	-1.584963	0.2133616	5	8	17	1	0	9
AC015818.6	0	#NUM!	0.824326	1	0	0	0	0	0
AC015818.2	#DIV/0!	#DIV/0!	0.8030358	0	0	0	0	1	0
AL359273.1	5	2.3219281	0.4606115	1	0	0	0	0	5
CA12	1.3499218	0.4328758	3.53E-05	2137	2222	2674	2600	3433	3461
CA11	1.183908	0.243557	0.5032364	22	30	35	32	40	31
CACNA1C-AS	1	0	0.923879	1	1	2	4	0	0
AL390294.1	#DIV/0!	#DIV/0!	0.5509701	0	0	0	0	3	0
KRT13	0.8333333	-0.263034	0.9048522	1	3	2	0	0	5
C19orf18	0.875	-0.192645	0.9888247	1	1	6	3	1	3
KRT10	0.9535888	-0.068561	0.9599066	493	646	714	570	699	498
C19orf12	0.9568997	-0.06356	0.9765393	821	882	1058	830	1021	791
KRT19	1.081294	0.1127589	0.200833	44784	56112	60555	62033	60918	51625
KRT18	1.0338466	0.0480221	0.4033916	32790	38948	43606	43484	41005	34759
KRT17	1.3431953	0.4256691	0.1402047	47	49	73	94	64	69
KRT16	0	#NUM!	0.0230175	8	5	1	0	0	0
KRT15	0.7285068	-0.456986	0.2546043	56	73	92	54	61	46
KRT14	#DIV/0!	#DIV/0!	0.0794429	0	0	0	5	5	0
AC091588.3	0.4615385	-1.115477	0.3499644	5	11	10	0	2	10
AP006216.1	#DIV/0!	#DIV/0!	0.5509701	0	0	0	0	3	0
AC091588.2	#DIV/0!	#DIV/0!	0.3109758	0	0	0	0	6	0
AC091588.1	1.375	0.4594316	0.7257363	10	0	6	0	11	11
OIP5-AS1	0.9624586	-0.055204	0.9402386	2855	2365	2931	2017	2884	2944
CA14	0.8481013	-0.237692	0.6863066	27	26	26	11	30	26
CA13	1.1443299	0.194503	0.6469368	26	39	32	21	52	38
KRT71	0.8421053	-0.247928	0.8601436	8	3	8	2	3	11
AC022960.2	#DIV/0!	#DIV/0!	0.6554062	0	0	0	0	2	0
AC022960.1	0.1666667	-2.584963	0.3399309	4	2	0	0	1	0
AC022960.3	0	#NUM!	0.824326	0	1	0	0	0	0
KRT79	#DIV/0!	#DIV/0!	0.1623641	0	0	0	4	0	3
TSC2	0.7728614	-0.371718	0.0083132	1553	1654	1878	1186	1417	1327
TSC1	1.0188497	0.0269412	0.4813071	2003	1932	2272	1840	2225	2259
AC080080.1	0.2956522	-1.758027	0.0031497	48	19	48	8	13	13
Z98885.3	0.2	-2.321928	0.1400137	5	7	3	0	2	1
Z98885.2	2.4285714	1.2801079	0.1370105	6	2	6	16	6	12
KRT40	#DIV/0!	#DIV/0!	0.8030358	0	0	0	1	0	0
AL031670.1	0.8130841	-0.298523	0.6063304	47	25	35	26	25	36
COBLL1	1.4436071	0.5296782	0.0028444	1557	849	1575	1337	2300	2110

NF1P11	3.1111111	1.6374299	0.1387928	1	6	2	20	6	2
NF1P10	0.3333333	-1.584963	0.2444606	19	1	7	6	3	0
PSMA3-AS1	1.041769	0.0590355	0.5898446	473	229	519	338	490	444
ACBD7	1.610687	0.6876762	0.0821977	52	37	42	33	90	88
BTNL9	0.745098	-0.424498	0.4175859	35	40	27	19	39	18
ACBD6	0.8857996	-0.174948	0.4310186	1532	1771	2056	1610	1601	1536
BTNL8	0.8333333	-0.263034	0.9246114	0	5	1	3	0	2
ACBD5	1.143853	0.1939016	0.1126968	1112	911	1133	898	1406	1306
ACBD4	1.2268041	0.2949049	0.1208768	158	159	168	218	211	166
BTNL3	0	#NUM!	0.3482063	0	0	6	0	0	0
MRGBP	0.9844072	-0.022673	0.7184271	1254	1355	1752	1459	1481	1353
Z98885.1	0	#NUM!	0.6436235	2	0	0	0	0	0
AC107980.1	0	#NUM!	0.5337954	0	3	0	0	0	0
ACBD3	0.994489	-0.007973	0.7671569	2131	1619	2238	1547	2176	2232
ETV5-AS1	1	0	0.9904495	3	0	0	0	0	3
AC008079.2	1.2564103	0.3293076	0.5171088	11	9	19	21	14	14
SCYL3	0.8177738	-0.290226	0.2097889	401	299	414	215	354	342
MIR34B	17	4.0874628	0.0255158	0	0	1	11	4	2
THA1P	0.2	-2.321928	0.4011739	3	1	1	0	1	0
AC021549.1	#DIV/0!	#DIV/0!	0.8030358	0	0	0	0	0	1
CA5B	0.6296296	-0.667425	0.2251292	50	27	31	9	37	22
CA5A	0	#NUM!	0.824326	0	0	1	0	0	0
KYNU	0.7010526	-0.512405	0.0679032	137	140	198	101	110	122
SCYL2	1.1897356	0.250641	0.0874584	1508	850	1500	1209	1696	1685
SCYL1	0.9257789	-0.11126	0.7382484	1839	2189	2520	2032	2148	1882
TSFM	0.9510269	-0.072442	0.972306	296	446	524	380	463	361
JAGN1	0.9485539	-0.076198	0.9278272	1215	1312	1380	1303	1256	1147
AC093014.1	1.6666667	0.7369656	0.7232255	3	3	0	0	0	10
HSH2D	7.4634146	2.8998358	4.40E-13	12	8	21	93	124	89
KRT81	0.9873418	-0.018379	0.9917415	19	37	23	19	32	27
KRT80	1.2922318	0.3698649	0.0411584	230	307	274	394	321	333
TUFT1	0.8937269	-0.162094	0.3573998	2110	2145	2520	1857	2219	1979
KRT86	0.6860465	-0.543622	0.4432365	20	43	23	32	12	15
KRT83	#DIV/0!	#DIV/0!	0.4501902	0	0	0	0	0	4
AL020997.3	0.25	-2	0.5155155	1	3	0	0	1	0
GMDS	0.845125	-0.242763	0.2330504	864	952	1025	836	838	727
AL020997.2	#DIV/0!	#DIV/0!	0.8030358	0	0	0	0	1	0
AL020997.1	0.7941176	-0.332575	0.7562079	14	4	16	7	9	11
AC010378.1	0	#NUM!	0.005607	7	3	10	0	0	0
AL157714.2	0	#NUM!	0.824326	1	0	0	0	0	0
AL031430.1	0	#NUM!	0.4460243	4	0	0	0	0	0
TSHR	0.8095238	-0.304855	0.7989371	7	12	2	9	5	3
AL139156.2	1.25	0.3219281	0.7782449	2	0	6	2	5	3
TSKS	0.9327943	-0.100369	0.86181	572	626	781	651	637	558
TUBBP2	0	#NUM!	0.824326	0	1	0	0	0	0
TUBBP1	0.5794393	-0.787271	0.077937	54	79	81	49	25	50
GMFB	0.841738	-0.248557	0.2522226	1185	768	1200	634	1085	935
TUBBP5	0.8747475	-0.193062	0.5936066	129	168	198	137	140	156
GMFG	0.5454545	-0.874469	0.710744	1	0	10	3	3	0
TUBBP8	#DIV/0!	#DIV/0!	0.8030358	0	0	0	0	1	0
TSKU	0.7332778	-0.447568	0.0067176	1007	1294	1302	802	910	930
HIST3H2BB	1.8589744	0.8945069	0.0585694	9	22	47	31	56	58
AC068726.1	0.5	-1	0.7177262	0	3	1	0	0	2
CTAGE1	1.0625	0.0874628	0.913119	6	3	7	4	12	1

CTAGE5	0.8926282	-0.163869	0.510649	869	623	1004	624	838	766
CTAGE4	1.025641	0.0365259	0.7520591	63	40	92	61	84	55
AL445471.1	#DIV/0!	#DIV/0!	0.5230806	0	0	0	1	2	0
CTAGE9	1.5714286	0.6520767	0.3692797	5	6	10	13	9	11
TMEM126A	1.0357143	0.0506261	0.4890205	374	292	454	385	392	383
CTAGE8	1.5602837	0.6418084	0.0757612	56	22	63	93	76	51
CTAGE6	1.8	0.8479969	0.4338208	0	4	6	9	5	4
LIVAR	0	#NUM!	0.824326	1	0	0	0	0	0
TMEM126B	0.9302575	-0.104298	0.8269166	965	630	1201	723	1005	873
RHOT2	0.9554523	-0.065744	0.977683	2284	2275	2759	2458	2380	2154
RHOT1	0.893438	-0.16256	0.5873112	772	377	634	401	618	574
SYNDIG1	#DIV/0!	#DIV/0!	0.5371397	0	0	0	0	0	3
AC008567.3	1.1147541	0.1567255	0.7074367	27	10	24	18	24	26
AC008567.2	0	#NUM!	0.824326	0	0	1	0	0	0
AC018552.4	0	#NUM!	0.6631647	1	0	1	0	0	0
AC018552.2	0.4615385	-1.115477	0.1873604	13	18	8	4	8	6
DLG3-AS1	1	0	0.9922023	2	0	0	0	0	2
AL031183.1	0	#NUM!	0.824326	0	0	1	0	0	0
ZNF497	0.5252918	-0.928809	0.0105698	71	103	83	41	43	51
ZNF496	0.8333333	-0.263034	0.1214714	1215	1378	1385	963	1121	1231
AP003031.2	1.875	0.9068906	0.5054567	5	1	2	1	7	7
ANXA8L1	0.9110716	-0.134364	0.5574387	704	690	855	580	778	691
ZNF493	1.2079772	0.2725932	0.1956114	282	119	301	228	310	310
ZNF492	1.5691057	0.6499425	0.011645	85	75	86	93	151	142
AC104462.1	0	#NUM!	0.5378194	0	2	1	0	0	0
ZNF491	0.3333333	-1.584963	0.2890196	5	5	5	0	1	4
ZNF490	1.3863636	0.4713057	0.4575521	19	10	15	8	19	34
AC091053.2	1.2222222	0.2895066	0.8044313	1	0	8	0	2	9
MED19	0.9167914	-0.125335	0.6629225	943	1168	1230	1019	1132	912
MED16	0.9590038	-0.060392	0.9485387	1509	1855	1856	1884	1510	1612
MED15	1.0565496	0.0793605	0.23287	2656	3001	3397	2984	3329	3253
MED18	0.8853238	-0.175723	0.4837658	487	589	607	491	574	425
MED17	0.8712121	-0.198904	0.6540072	70	99	95	63	64	103
MECP2	0.8906091	-0.167136	0.4101406	1181	1294	1465	1114	1188	1207
MED12	0.7611762	-0.393698	0.0113886	2961	3528	3577	2027	2661	2974
MED11	1.260479	0.3339721	0.1363431	86	99	149	143	170	108
MED14	0.8096923	-0.304554	0.1103702	957	811	1059	537	849	903
MED13	1.2757764	0.3513755	0.0289308	1891	1247	1692	1411	2248	2503
MECOM	0.3846154	-1.378512	0.0028112	58	58	79	10	41	24
MED10	0.9107177	-0.134924	0.5457177	1577	1665	2011	1508	1748	1528
TSPO	2.9	1.5360529	0.0820061	6	0	14	41	14	3
ZNF488	2.0625	1.0443941	0.0034402	27	33	36	54	76	68
ZNF487	1.3875	0.4724878	0.1386284	49	44	67	52	104	66
ZNF486	1.0557522	0.0782713	0.4912053	395	300	435	294	470	429
ZNF485	2	1	0.3890374	5	0	4	7	7	4
ZNF484	#DIV/0!	#DIV/0!	0.6482162	0	0	0	0	1	1
ZNF483	#DIV/0!	#DIV/0!	0.8030358	0	0	0	0	0	1
CNOT6L	1.0133448	0.0191252	0.7324703	845	647	831	546	869	939
ZNF480	1.0727131	0.1012642	0.3970507	475	306	498	373	525	474
AL445231.1	1	0	0.9798948	6	0	4	2	6	2
GMIP	1.0670103	0.0935741	0.4013214	313	418	433	361	460	421
MED27	0.9400564	-0.089181	0.8575516	820	1002	1014	911	892	863
MED26	0.8901099	-0.167945	0.5355016	255	306	349	229	313	268
MED29	0.8962455	-0.158034	0.4737311	1442	1767	1745	1470	1552	1418

PGM2L1	1.4060475	0.4916453	0.0494814	182	101	180	132	254	265
MED28	0.9892545	-0.015586	0.7349229	2816	2962	3249	2677	3164	3089
MED23	0.8555838	-0.225019	0.3253514	1046	683	1020	533	909	910
MED22	0.9717008	-0.041416	0.9352223	3132	3677	3792	3012	3608	3681
MED25	0.9417151	-0.086638	0.8413905	1315	1503	1660	1371	1520	1326
MED24	0.9175087	-0.124206	0.5422036	2036	2248	2347	1819	2284	1981
MIR760	0	#NUM!	0.4374838	0	4	0	0	0	0
MED21	0.9066667	-0.141356	0.5150302	913	749	888	634	853	825
MED20	0.963123	-0.054208	0.996807	1035	1137	1299	950	1198	1195
AC025164.2	1.6097561	0.6868421	0.287624	17	15	9	11	21	34
ZNF479	0	#NUM!	0.824326	1	0	0	0	0	0
ZNF474	0	#NUM!	0.824326	1	0	0	0	0	0
ZNF473	0.7979976	-0.325544	0.0805	562	548	588	380	462	513
ZNF470	0.9981982	-0.002602	0.8005005	205	130	220	157	201	196
TSR3	1.0576923	0.08092	0.3619976	972	1196	1316	1382	1273	1030
MIR766	0	#NUM!	0.824326	1	0	0	0	0	0
ZNF469	1	0	0.9707229	3	0	0	0	3	0
ZNF468	1.0545153	0.07658	0.4483951	1547	1059	1613	1083	1750	1616
ZNF467	0.5222222	-0.937264	0.1300228	25	34	31	21	18	8
ZNF462	1.6078431	0.6851267	0.2089028	22	13	16	21	41	20
ZNF461	0.7446809	-0.425306	0.2743423	124	61	97	72	75	63
ZNF460	0.7578475	-0.40002	0.32772	76	83	64	24	64	81
AC138819.1	#DIV/0!	#DIV/0!	0.8030358	0	0	0	0	1	0
NUDT22	0.851971	-0.231124	0.4308882	385	395	463	415	338	306
NUDT21	0.9411624	-0.087484	0.7497869	5801	4877	6097	4399	5781	5608
AC008171.1	#DIV/0!	#DIV/0!	0.6099294	0	0	0	2	0	0
TSR2	0.929636	-0.105262	0.7597859	1547	2018	2205	1737	1993	1634
TSR1	0.8244429	-0.278508	0.0543754	2853	2823	3210	1976	2655	2695
AL137100.2	0	#NUM!	0.2219629	0	7	0	0	0	0
AL137100.1	0	#NUM!	0.824326	1	0	0	0	0	0
ZNF454	#DIV/0!	#DIV/0!	0.5509701	0	0	0	0	3	0
AL606763.1	0.8888889	-0.169925	0.8413466	16	32	42	12	22	46
ZNF451	1.0437743	0.0618098	0.588759	751	462	843	467	866	813
GMNN	0.9744825	-0.037292	0.8016808	2467	2161	3053	2434	2680	2371
NUDT11	0.7481203	-0.418658	0.0476531	331	334	399	198	339	259
NUDT10	0	#NUM!	0.2232483	1	2	3	0	0	0
AL160313.1	3	1.5849625	0.6677907	0	1	0	0	0	3
NUDT19	1.15	0.2016339	0.6882877	16	7	17	13	14	19
ZNF449	0.9936609	-0.009175	0.7982606	234	120	277	179	182	266
NUDT18	0.8244147	-0.278558	0.3448058	185	173	240	164	154	175
HMGB1P6	0.999786	-0.000309	0.6331988	1461	1432	1779	1496	1759	1416
NUDT17	0.9032258	-0.146841	0.8024589	41	39	44	21	37	54
HMGB1P5	0.9210079	-0.118715	0.5893257	2317	2177	2570	2017	2407	2082
NUDT16	0.9341726	-0.098239	0.7308583	1229	1163	1421	992	1238	1332
ZNF446	1.0046729	0.0067259	0.7942497	48	63	103	66	77	72
NUDT15	1.0671386	0.0937476	0.2240746	1422	1343	1614	1319	1704	1650
ZNF445	0.9044139	-0.144945	0.5609046	1094	1077	1250	693	1041	1360
NUDT14	1.005487	0.0078944	0.7219459	188	259	282	272	258	203
ZNF444	0.9366883	-0.094359	0.896889	300	459	473	413	430	311
NUDT13	0.6129032	-0.706269	0.1695954	53	24	47	18	33	25
ZNF443	0.8030691	-0.316404	0.3660223	123	111	157	69	97	148
HMGB1P1	10	3.3219281	0.1389117	1	0	0	6	4	0
NUDT12	#DIV/0!	#DIV/0!	0.203811	0	0	0	1	3	2
ZNF442	0	#NUM!	0.824326	0	1	0	0	0	0

ZNF441	0.6612903	-0.596644	0.1741298	74	38	74	40	43	40
ZNF440	0.8254675	-0.276717	0.2515103	873	489	884	489	702	663
GCSHP5	1.0641483	0.0896992	0.3590885	430	451	522	480	573	440
GCSHP4	#DIV/0!	#DIV/0!	0.3831122	0	0	0	0	5	0
GCSHP2	0	#NUM!	0.824326	0	0	1	0	0	0
ZNF439	1.266436	0.3407742	0.2107062	125	54	110	99	153	114
ZNF438	1.0482375	0.0679656	0.5245901	166	152	221	175	204	186
GMNC	1	0	0.9998043	0	4	5	0	8	1
ZNF436	1.0303448	0.0431272	0.6163377	484	367	599	343	537	614
ZNF433	0.8438228	-0.244988	0.4693209	122	121	186	90	159	113
ZNF432	0.8247012	-0.278057	0.5458698	110	40	101	47	87	73
ZNF431	1.159306	0.2132614	0.1253159	677	452	773	564	848	793
ZNF430	0.8715203	-0.198394	0.4954459	387	214	333	203	347	264
TMEM121B	#DIV/0!	#DIV/0!	0.8030358	0	0	0	0	1	0
GMPS	0.9215467	-0.117871	0.5527302	6088	5670	7043	5082	6267	5977
GMPR	0.9236641	-0.11456	0.945441	34	45	52	51	34	36
RBM5-AS1	2	1	0.4816196	4	1	1	3	5	4
AL137246.1	0	#NUM!	0.824326	1	0	0	0	0	0
MED30	0.922619	-0.116193	0.8542592	207	223	242	248	202	170
ZNF429	1.1048544	0.1438562	0.50758	206	83	226	122	213	234
MED31	0.7701863	-0.376721	0.3895411	66	51	44	20	59	45
ZNF428	0.7893916	-0.341187	0.3387603	191	214	236	221	175	110
AL669831.5	1.0465116	0.0655883	0.7780611	75	38	59	42	69	69
PGK1	0.9638545	-0.053113	0.9961211	28877	35055	36108	29507	34834	32083
AL669831.4	2.3333333	1.2223924	0.1043271	2	11	8	17	14	18
ZNF426	0.9184028	-0.122801	0.7145783	404	310	438	320	383	355
AL669831.3	0.7608696	-0.394279	0.6452371	12	19	15	7	5	23
ZNF425	0.5491803	-0.864648	0.0433373	43	101	100	50	45	39
AL669831.2	#DIV/0!	#DIV/0!	0.1884392	0	0	0	0	0	8
PGK2	0.5	-1	0.7932724	0	0	4	0	0	2
AL669831.1	0.8492872	-0.235676	0.4843956	203	117	171	90	147	180
ZNF423	0.4189189	-1.255257	0.0588347	17	32	25	7	9	15
AL139393.2	1.2844444	0.3611445	0.0528238	152	130	168	176	217	185
AL139393.1	2	1	0.7766092	0	1	0	1	0	1
ZNF420	1.5767442	0.6569486	0.03216	83	33	99	108	131	100
ALG1L2	0.7377049	-0.438884	0.4825009	34	34	54	42	21	27
AC079780.1	3.5	1.8073549	0.328211	4	0	0	3	0	11
TMEM120B	0.7579821	-0.399764	0.0375862	442	489	635	367	411	409
TMEM120A	0.948046	-0.076971	0.9897709	637	676	862	744	704	614
ZNF419	0.9497487	-0.074382	0.9537906	178	174	245	163	208	196
ZNF418	0.774744	-0.368208	0.1686534	208	156	222	136	152	166
ZNF417	0.7928364	-0.334905	0.1536857	359	311	363	184	292	343
ZNF416	0.6957211	-0.523419	0.0363299	222	185	224	103	187	149
ZNF415	1.1796247	0.2383279	0.1752108	440	267	412	328	567	425
ZNF414	0.8924731	-0.164119	0.6939204	159	206	193	193	176	129
ZNF410	0.7752809	-0.367209	0.5329084	30	24	35	16	23	30
AC004690.1	#DIV/0!	#DIV/0!	0.5070017	0	0	0	3	0	0
MIPEP	0.7614933	-0.393097	0.0122585	902	962	1029	671	821	711
AC107398.4	0	#NUM!	0.824326	1	0	0	0	0	0
ZNF408	0.7386478	-0.437041	0.0281895	531	693	758	489	544	431
AC107398.3	#DIV/0!	#DIV/0!	0.304772	0	0	0	1	0	4
ZNF407	0.816047	-0.293276	0.3538226	175	144	192	97	128	192
AC107398.2	1	0	0.971258	1	0	2	0	0	3
PGM3	0.9217764	-0.117511	0.6653786	1354	1047	1562	972	1360	1321

PGM2	0.9305837	-0.103792	0.6738024	1848	1551	2032	1318	1958	1778
ZNF404	1.5571429	0.6389013	0.1453826	16	14	40	35	32	42
PGM5	1.4117647	0.4974997	0.6253523	7	4	6	2	15	7
PGLS	1.0132412	0.0189777	0.6076918	485	581	671	707	565	488
YAF2	0.8434211	-0.245675	0.3418198	261	232	267	136	282	223
PGM1	0.9351254	-0.096768	0.7863051	4080	4651	5219	4328	4574	4143
RN7SL825P	#DIV/0!	#DIV/0!	0.2021377	0	0	0	1	6	0
LAMTOR3P2	0	#NUM!	0.4324683	2	0	2	0	0	0
TFIP11	0.8405722	-0.250556	0.194344	1012	1400	1433	862	1188	1182
AC004846.1	0.9	-0.152003	0.8720661	32	13	25	20	21	22
RNU6-916P	1.5	0.5849625	0.8898407	0	2	0	0	3	0
AL136418.1	1.4545455	0.5405684	0.6901418	3	5	3	0	5	11
AC004846.2	#DIV/0!	#DIV/0!	0.6317974	0	0	0	1	1	0
FOXRED1	0.860217	-0.217227	0.2346751	1612	1815	2103	1564	1647	1546
FOXRED2	0.7458399	-0.423062	0.0090789	2072	2438	2521	1274	1959	2011
AC016257.1	0.75	-0.415037	0.8334391	2	2	0	0	2	1
AC245047.8	0	#NUM!	0.824326	1	0	0	0	0	0
CAHM	0.8467153	-0.240051	0.676415	33	50	54	41	47	28
SNRPD2	1.0294459	0.041868	0.4189836	4484	5556	6329	5760	5938	5153
SNRPD1	1.3175411	0.397848	0.000182	3035	3049	4132	4441	4899	4120
SNRPD3	1.029842	0.042423	0.3863345	3568	4001	4394	3991	4303	4026
FAM76B	0.9528487	-0.069681	0.9082046	545	400	582	368	557	530
FAM76A	1.2174274	0.2838357	0.0338505	374	375	456	395	550	522
GGNBP1	#DIV/0!	#DIV/0!	0.6554062	0	0	0	0	2	0
GGNBP2	1.0094853	0.0136199	0.5936304	2259	1681	2491	1881	2468	2143
RN7SL820P	1	0	0.9922023	2	0	0	0	0	2
AC245140.2	0.7058824	-0.5025	0.5285674	13	27	11	17	12	7
GSPT2	0	#NUM!	0.6436235	2	0	0	0	0	0
GSPT1	1.0467332	0.0658938	0.2513053	7141	6834	7958	6773	8229	7956
PGS1	0.8481435	-0.23762	0.1902886	706	768	923	588	734	711
AP001363.1	#DIV/0!	#DIV/0!	0.8030358	0	0	0	0	0	1
TSPEAR-AS2	1.022779	0.0324945	0.6846191	120	156	163	160	150	139
AP001363.2	0.4166667	-1.263034	0.4346366	7	2	3	2	3	0
FAM78A	1.2733056	0.3485787	0.0009662	1183	1312	1592	1535	1940	1729
TSPEAR-AS1	0.8299712	-0.268867	0.4552389	106	95	146	91	108	89
CALY	7.5	2.9068906	0.094184	0	2	0	10	2	3
CALU	0.911988	-0.132913	0.4497122	11957	11653	13010	8685	12503	12209
CALR	0.9663083	-0.049444	0.9215112	34467	42420	45814	38679	41938	37950
MEDAG	#DIV/0!	#DIV/0!	0.6099294	0	0	0	2	0	0
MAP1LC3B	1.0908745	0.1254851	0.1138429	2519	2455	2905	2808	3059	2728
MAP1LC3A	1.2	0.2630344	0.8258853	3	0	2	6	0	0
MAP1LC3C	0.5933584	-0.753024	1.52E-05	468	534	594	279	356	312
HSPB1P1	#DIV/0!	#DIV/0!	0.6554062	0	0	0	0	2	0
HSPB1P2	2	1	0.565048	2	0	4	0	11	1
HTATIP2	0.8524616	-0.230293	0.1485263	3789	4081	4703	3432	3960	3326
CAP1	1.024394	0.0347707	0.56156	4009	4401	4626	3382	5027	4945
CANX	0.8552826	-0.225527	0.1344465	67773	60967	73233	44478	63748	64518
YAP1	0.7319407	-0.450201	0.0037929	6588	5734	7114	3414	5196	5616
CAPG	0.7505519	-0.413976	0.158526	137	174	142	113	128	99
AC006994.2	2	1	0.790186	0	0	1	1	1	0
RNU6-911P	0.5	-1	0.8652929	0	0	2	0	0	1
AC006994.1	0	#NUM!	0.824326	1	0	0	0	0	0
CAP2	0.7783731	-0.361466	0.0784021	747	599	707	363	580	655
GABBR2	1.0666667	0.0931094	0.7531918	46	65	39	47	61	52

GABBR1	1.6129032	0.6896599	0.2946062	13	4	14	12	18	20
AC074019.1	0	#NUM!	0.824326	0	1	0	0	0	0
TTC1	1.1056672	0.1449172	0.0691064	3354	3137	4061	3484	4438	3745
NAPA-AS1	0.625	-0.678072	0.6838592	5	2	1	0	2	3
TTC9	1.011734	0.0168301	0.7060259	520	502	512	428	570	554
TTC8	0.9330629	-0.099954	0.8299223	338	240	408	227	350	343
TTC6	0.1818182	-2.459432	0.2749219	4	0	7	0	0	2
TTC5	0.948999	-0.075521	0.8646474	663	712	723	599	691	701
TTC4	1.1612903	0.2157287	0.6899342	6	8	17	14	16	6
TTC3	1.2264026	0.2944327	0.0694217	2414	1224	2422	1876	2850	2706
AC027228.2	0.2903226	-1.784271	0.1227296	4	11	16	0	4	5
AC027228.1	0	#NUM!	0.5337954	0	3	0	0	0	0
RTN4IP1	0.8406564	-0.250412	0.2244474	537	571	781	479	564	545
CAPS	1.0530303	0.074547	0.7823071	58	43	31	50	45	44
CARS	1.136408	0.1844809	0.0678904	1285	1288	1525	1637	1533	1487
AC007738.1	0	#NUM!	0.824326	1	0	0	0	0	0
TTF2	0.7335431	-0.447046	0.0077443	1353	1321	1458	716	1134	1181
TTF1	1.1147834	0.1567634	0.2001559	775	482	982	842	833	821
CARF	0.9616279	-0.056449	0.9421773	318	206	336	158	338	331
CAST	1.1800719	0.2388748	0.016962	3685	2898	3985	3554	4730	4187
CASK	0.9879518	-0.017487	0.9453513	391	317	371	233	444	389
AC016747.3	0.4	-1.321928	0.2676334	9	6	5	1	5	2
AC016747.2	0.7	-0.514573	0.6851088	3	5	12	2	9	3
AC016747.1	1.066055	0.0922819	0.4087965	358	328	404	309	463	390
TSNARE1	0.8066971	-0.309901	0.3053455	208	197	252	204	180	146
GNAZ	0.3684211	-1.440573	0.0044055	56	41	55	7	23	26
FAM71D	1	0	0.9890559	0	0	1	0	0	1
RHPN1	1.9807692	0.9860608	0.0739721	14	4	34	35	23	45
RHPN2	0.7720246	-0.373281	0.026312	1611	1852	1906	985	1595	1565
FAM71C	#DIV/0!	#DIV/0!	0.8030358	0	0	0	0	1	0
YARS	1.1077287	0.1476046	0.0822057	4018	4710	5233	4984	5436	5045
CAV3	0	#NUM!	0.4374838	0	4	0	0	0	0
CAV2	1.0915404	0.1263655	0.292926	528	461	595	636	650	443
CAV1	1.3012846	0.3799365	2.46E-05	6540	6015	7529	7836	9517	8782
CNOT10	0.8745268	-0.193426	0.3214843	1823	1471	2253	1396	1796	1659
Z98257.1	0	#NUM!	0.3049182	1	1	3	0	0	0
AC007342.5	1.1515152	0.2035334	0.7129666	28	27	11	19	22	35
AC007342.4	1.1954023	0.2574962	0.4796564	33	20	34	39	38	27
AC007342.3	#DIV/0!	#DIV/0!	0.288876	0	0	0	3	2	0
GNAL	0.3076923	-1.70044	0.0014235	31	47	52	9	19	12
AC007342.9	1.0833333	0.1154772	0.7654327	17	13	18	17	15	20
AP001269.2	0	#NUM!	0.6385096	0	2	0	0	0	0
AC007342.8	0.4857143	-1.04182	0.2601548	6	16	13	3	10	4
AP001269.1	0	#NUM!	0.6436235	2	0	0	0	0	0
CNOT11	1.0010191	0.0014695	0.6031667	3873	4073	4810	3772	4806	4191
AC007342.7	1.0144928	0.0207586	0.8765954	21	12	36	16	35	19
GNAQ	1.2048736	0.2688817	0.0379357	3541	2493	3733	2903	4289	4576
GNB2	0.9479988	-0.077043	0.9938247	3897	4615	5180	4939	4285	3756
AC007342.6	1.5	0.5849625	0.8403947	2	2	0	0	6	0
GNB1	1.0907147	0.1252738	0.1032158	8113	9269	10210	9113	11003	9979
GNAS	1.0550684	0.0773365	0.2301237	30021	33350	38012	35529	37935	33502
GNB4	0.9215288	-0.117899	0.6273897	1679	1599	1641	920	1656	1957
GNB3	0.5416667	-0.884523	0.4090887	6	10	8	5	4	4
GNB5	0.9016667	-0.149334	0.6562071	206	187	207	166	175	200

SLC35E2B	0.81887	-0.288294	0.0917261	1104	1205	1390	749	1188	1092
AL031673.1	0.3333333	-1.584963	0.3242242	5	5	2	0	1	3
IDI2-AS1	0	#NUM!	0.1789363	2	0	6	0	0	0
MYO18B	0.3333333	-1.584963	0.0391897	16	22	16	4	8	6
MYO18A	0.9540062	-0.067929	0.8707122	3612	4211	4483	3083	4169	4488
AL513123.1	0	#NUM!	0.0834281	5	5	0	0	0	0
PDPK1	0.9784127	-0.031485	0.9103309	1497	1477	1751	1167	1649	1807
TTI2	0.7205721	-0.472785	0.0329147	271	299	339	169	224	262
TTI1	0.9596047	-0.059488	0.931531	1746	1907	2115	1508	2027	2000
GS1-594A7.3	1.4285714	0.5145732	0.7141657	0	4	3	3	5	2
FAM72D	0.8460648	-0.24116	0.310458	278	263	323	212	255	264
FAM72C	0.9732541	-0.039112	0.8736099	206	206	261	245	229	181
FAM72B	0.9968828	-0.004504	0.7894857	552	498	554	461	629	509
FAM72A	1.1351616	0.1828977	0.1588693	343	303	375	328	405	426
AC108727.1	1	0	0.994626	0	1	2	0	3	0
GNG3	0.7741935	-0.369234	0.7064194	12	7	12	7	13	4
GNG2	3.3333333	1.7369656	0.3321886	0	0	3	6	0	4
GNG5	1.1546205	0.2074188	0.0620589	2229	2374	3145	3178	3323	2445
GNG4	0.7740586	-0.369485	0.5263103	73	45	121	73	38	74
GNG7	1.2432432	0.3141086	0.6528849	13	19	5	15	10	21
AP001029.2	0	#NUM!	0.4324683	2	0	2	0	0	0
AP001029.1	0.6666667	-0.584963	0.9395362	0	0	3	2	0	0
RHOXF1-AS1	1.5714286	0.6520767	0.1756899	23	13	27	20	41	38
MYO15B	0.45	-1.152003	0.4241595	3	7	10	6	3	0
MYO15A	0.8888889	-0.169925	0.9382858	7	4	7	7	4	5
NRDE2	0.8218138	-0.283116	0.1287216	768	734	1001	666	718	673
AC021151.1	#DIV/0!	#DIV/0!	0.8030358	0	0	0	0	0	1
GPATCH2	1.1992084	0.2620824	0.1115573	272	178	308	256	327	326
GPATCH1	0.9543413	-0.067423	0.9666864	415	358	563	429	468	378
GPATCH4	0.8434579	-0.245612	0.220484	1568	1451	2117	1463	1557	1312
PHAX	0.943852	-0.083367	0.8666449	1585	1231	1779	1313	1620	1404
GPATCH3	0.9903459	-0.013996	0.8131694	332	474	437	422	425	384
PHB2	0.867241	-0.205495	0.2997017	8037	10017	10848	8566	8923	7576
GPATCH8	0.8383531	-0.25437	0.1629391	2055	1909	2278	1203	1932	2098
CMKLR1	1	0	0.9890559	0	0	1	0	0	1
AC084346.2	7	2.8073549	0.2850717	0	1	0	0	2	5
RHNO1	0.8271158	-0.273839	0.1231728	1133	1065	1394	984	1009	978
ZNF398	0.824279	-0.278795	0.1803034	476	512	503	349	398	482
ZNF397	0.9334511	-0.099354	0.8119648	595	427	676	432	570	583
TUBAP2	0.6388889	-0.646363	0.4583913	16	15	5	9	7	7
ZNF396	0.8823529	-0.180572	0.7783782	38	16	31	8	37	30
INTS9-AS1	0.6	-0.736966	0.725778	2	2	1	0	3	0
ZNF395	1.1116152	0.1526575	0.1129624	1226	1506	1676	1605	1709	1586
ZNF394	1.085829	0.1187969	0.1570929	1013	1011	1215	1065	1303	1149
ZNF391	0.7811861	-0.356262	0.2364258	168	126	195	83	173	126
AC019288.1	0.3846154	-1.378512	0.443814	10	0	3	0	5	0
PHYHIPL	0	#NUM!	0.824326	0	0	1	0	0	0
AL353743.1	1.3496933	0.4326316	0.2155649	43	32	88	41	74	105
RPSAP76	0	#NUM!	0.6385096	0	2	0	0	0	0
AL353743.3	0	#NUM!	0.824326	0	0	1	0	0	0
AL353743.2	#DIV/0!	#DIV/0!	0.8030358	0	0	0	0	1	0
NRBF2	1.4492948	0.5353511	1.86E-06	982	928	1068	1284	1554	1478
AC092620.2	#DIV/0!	#DIV/0!	0.6317974	0	0	0	1	1	0
AC092620.3	0.25	-2	0.5705817	1	0	3	0	1	0

AL513122.1	#DIV/0!	#DIV/0!	0.6554062	0	0	0	0	2	0
PHC2	0.9746925	-0.036981	0.8179389	1237	1345	1646	1363	1431	1327
ZNF384	0.807666	-0.308169	0.1168604	813	998	1111	553	865	942
PHC1	0.9768922	-0.033729	0.9561928	954	950	1082	708	1093	1116
ZNF383	0.7947154	-0.33149	0.2805054	178	119	195	123	133	135
ZNF382	1.3101266	0.3897062	0.2066917	37	40	81	52	82	73
AL031432.5	#DIV/0!	#DIV/0!	0.6411933	0	0	0	0	0	2
AL031432.4	0.8333333	-0.263034	0.6854922	34	27	59	31	36	33
AL031432.3	1.2857143	0.3625701	0.856043	7	0	0	1	3	5
AL031432.2	1.1481481	0.1993088	0.7983178	15	6	6	8	15	8
RPSAP72	0	#NUM!	0.6436235	2	0	0	0	0	0
PHC3	0.8448928	-0.24316	0.3623099	635	401	550	263	483	594
RPSAP70	2.3333333	1.2223924	0.5344886	0	2	1	7	0	0
AC105206.2	0.4285714	-1.222392	0.5909805	4	0	3	0	3	0
ZNF37A	1.148273	0.1994656	0.3371267	523	233	431	301	477	585
AL445472.1	0.6769231	-0.562936	0.4009553	33	18	14	5	17	22
AC105206.1	0	#NUM!	0.824326	1	0	0	0	0	0
AC079447.1	0.8235294	-0.280108	0.7717168	14	5	15	3	14	11
PHF3	1.1872195	0.2475867	0.1091839	1858	990	1831	1411	2185	1959
PHF2	0.9324201	-0.100948	0.7194445	709	695	786	586	731	725
PHF1	0.9346405	-0.097517	0.9346708	310	239	369	309	250	299
PHF7	0.7180952	-0.477753	0.0865322	156	181	188	82	135	160
MIR624	#DIV/0!	#DIV/0!	0.8030358	0	0	0	0	1	0
PHF6	1.0875758	0.1211159	0.3806608	1258	898	1144	804	1377	1408
PHEX	0.4285714	-1.222392	0.5744397	1	1	5	1	1	1
PHF8	0.7970211	-0.32731	0.046856	1914	2122	2275	1221	1877	1932
RPSAP54	1.5833333	0.662965	0.4990793	3	5	4	7	6	6
RPSAP51	0	#NUM!	0.3168171	0	4	1	0	0	0
RPSAP58	0.9583668	-0.06135	0.9667084	3204	3894	4119	3896	3756	3098
TCTN3	1.1190426	0.162265	0.1166088	1352	1702	1709	1536	1989	1805
MIR3613	0.375	-1.415037	0.5368174	3	0	5	0	3	0
TCTN2	0.9099237	-0.136183	0.6566526	228	225	202	170	206	220
TCTN1	0.9587426	-0.060785	0.9840682	194	142	173	151	151	186
ZNF367	0.707987	-0.498205	0.0028168	919	757	1091	556	675	728
ZNF366	1.3333333	0.4150375	0.7841756	4	5	0	3	5	4
ZNF365	0	#NUM!	0.6840298	0	0	2	0	0	0
ZNF362	0.8082508	-0.307125	0.1011618	861	982	1187	820	883	746
AC061965.1	#DIV/0!	#DIV/0!	0.8030358	0	0	0	0	1	0
AC001226.2	0.4166667	-1.263034	0.2961518	2	9	13	1	4	5
GNL2	1.1681614	0.2242397	0.0543837	2317	1553	2820	2424	2849	2542
AC001226.1	2.25	1.169925	0.5303649	4	0	0	3	4	2
AL158055.1	1.8333333	0.8744691	0.5985546	2	3	1	1	0	10
GNL1	0.8923048	-0.164392	0.4365048	3015	3600	3924	3144	3293	2967
MIR635	1.25	0.3219281	0.609129	10	9	17	10	26	9
GNL3	0.9540792	-0.067819	0.969742	4945	3813	5767	4152	5158	4548
APBB1IP	1.3201039	0.4006514	0.0005141	802	866	1028	1150	1281	1128
MIR643	#DIV/0!	#DIV/0!	0.062303	0	0	0	0	9	3
RPSAP43	#DIV/0!	#DIV/0!	0.3831122	0	0	0	0	5	0
MIR641	1	0	0.9890559	1	0	0	0	1	0
TUBB2B	0.7058824	-0.5025	0.6026947	9	9	16	5	6	13
AC097713.1	#DIV/0!	#DIV/0!	0.8030358	0	0	0	0	1	0
TUBB2A	0.9168937	-0.125174	0.7795447	199	257	278	233	223	217
RPSAP47	11	3.4594316	0.0914334	0	1	0	7	3	1
RPSAP44	#DIV/0!	#DIV/0!	0.8030358	0	0	0	0	1	0

AL162615.1	0.1111111	-3.169925	0.0241308	4	14	9	1	0	2
AC104461.1	0.5	-1	0.8652929	0	0	2	0	1	0
RPSAP49	1.2424242	0.3131579	0.5179344	27	26	13	38	22	22
ZNF358	0.9826087	-0.025311	0.9356745	32	41	42	35	35	43
ZNF350	1.4615385	0.5474878	0.6024845	3	6	4	3	5	11
WDR45B	1.0080583	0.0115791	0.5249146	2730	2914	3415	2849	3255	3028
MIR647	1.2857143	0.3625701	0.5675578	11	13	11	11	20	14
AP000534.2	2.3333333	1.2223924	0.5691174	3	0	0	3	0	4
AL078612.2	1.0588235	0.0824622	0.8863776	5	7	5	7	4	7
AC005674.2	0.5363636	-0.898717	0.1021554	30	26	54	16	25	18
RPSAP36	1	0	0.9984385	3	0	2	0	3	2
ABI3BP	1.8461538	0.8845228	0.3899325	0	6	7	8	11	5
AC091198.1	0.75	-0.415037	0.8408623	0	4	0	0	2	1
ZNF347	1.0287081	0.0408337	0.6720973	576	341	546	355	569	581
ZNF346	0.9198093	-0.120593	0.6241577	1356	1567	1691	1336	1493	1415
ZNF345	1.0491803	0.0692627	0.7678951	85	64	95	43	115	98
HMGB2P1	#DIV/0!	#DIV/0!	0.5070017	0	0	0	3	0	0
ZNF343	0.7736721	-0.370206	0.215878	136	140	157	110	102	123
ZNF341	1.2356495	0.3052696	0.0395675	340	259	394	419	430	378
MIR659	0.375	-1.415037	0.4703804	1	3	4	0	2	1
TUBB4B	1.1096247	0.1500717	0.1032176	18381	22620	24778	24837	26002	22151
TUBB4A	1.3333333	0.4150375	0.8405645	2	0	1	1	2	1
GNMT	0.4482759	-1.157541	0.2593634	13	7	9	6	4	3
ZNF33B	1.252074	0.3243199	0.0530084	590	312	665	584	736	642
ZNF33A	1.0867964	0.1200817	0.4089788	744	421	736	490	853	723
RPSAP24	0	#NUM!	0.824326	1	0	0	0	0	0
RNU1-103P	1.6666667	0.7369656	0.4826973	8	9	1	4	8	18
AC025165.1	#DIV/0!	#DIV/0!	0.8030358	0	0	0	0	1	0
ZNF337	0.9291339	-0.106042	0.8968735	48	34	45	23	28	67
ZNF335	0.9049577	-0.144078	0.5040862	1224	1469	1442	1153	1286	1303
AC025165.5	0.6923077	-0.530515	0.7517998	2	7	4	6	1	2
ZNF334	1.0548023	0.0769726	0.5223499	695	340	735	499	745	623
ZNF333	0.8494845	-0.23534	0.490239	167	128	190	84	141	187
ZNF331	0.8460607	-0.241167	0.1227743	21663	16703	23600	14859	19379	18189
ZNF330	1.1070414	0.1467091	0.1156269	936	946	1285	1069	1335	1102
AL390208.1	1.2727273	0.3479233	0.7920148	5	2	4	0	2	12
TMEM131L	0.6779303	-0.560791	0.0124232	294	270	383	211	199	232
NRBP2	0.8618267	-0.21453	0.563545	173	108	146	117	110	141
PDCL3P6	#DIV/0!	#DIV/0!	0.1506188	0	0	0	8	0	0
PDCL3P5	0.7307692	-0.452512	0.6596018	8	8	10	6	8	5
PDCL3P4	0.0454545	-4.459432	0.0166539	1	11	10	0	0	1
PDCL3P3	#DIV/0!	#DIV/0!	0.3096126	0	0	0	5	0	0
ZNF329	1.0517241	0.0727563	0.7978781	35	48	33	41	51	30
TUBB7P	0.2142857	-2.222392	0.2523705	7	7	0	3	0	0
MIR3653	9	3.169925	0.1998211	1	0	0	0	1	8
ZNF326	1.0534918	0.0751791	0.3390211	2404	1682	2644	2054	2668	2368
ZNF324	0.5963512	-0.745766	0.0014183	223	338	316	147	187	189
ZNF322	0.9810726	-0.027568	0.9485544	215	161	258	120	244	258
ZNF320	1.0817955	0.1134278	0.3299325	724	463	818	600	867	702
AC093495.1	0.4571429	-1.129283	0.0110155	70	40	65	27	29	24
AC079781.5	2.2	1.1375035	0.5476886	4	1	0	0	5	6
NRBP1	0.831602	-0.266035	0.0917072	4187	5099	5346	3759	4138	4271
MIR3649	0	#NUM!	0.824326	1	0	0	0	0	0
ZNF319	0.8582375	-0.220551	0.5104834	196	223	364	201	233	238

ZNF318	0.8195745	-0.287053	0.195301	780	748	822	383	715	828
ZNF317	0.8305785	-0.267812	0.1191376	821	850	991	616	776	819
ZNF316	0.779853	-0.358726	0.0220644	1867	2368	2297	1470	1939	1685
PHIP	1.2297688	0.2983871	0.1103169	1412	700	1348	926	1718	1611
FAM8A1	1.0061224	0.0088059	0.7332742	861	708	881	617	925	923
ZNF311	0.8275862	-0.273018	0.8026634	14	8	7	11	8	5
CBFB	0.9543152	-0.067462	0.8508584	3318	3181	3570	2620	3624	3365
MIR603	#DIV/0!	#DIV/0!	0.4501902	0	0	0	0	0	4
MIR602	0	#NUM!	0.1527178	2	4	1	0	0	0
MIR601	0	#NUM!	0.276989	0	6	0	0	0	0
AL160314.3	0.6666667	-0.584963	0.9053332	0	0	3	1	1	0
YBEY	1.2571757	0.3301862	0.0450155	253	292	326	395	403	297
AL160314.2	0.3125	-1.678072	0.1040178	10	12	10	4	5	1
AL160314.1	#DIV/0!	#DIV/0!	0.8030358	0	0	0	0	0	1
ZNF304	0.9949239	-0.007342	0.8438645	319	305	361	236	360	384
ZNF302	1.079922	0.1109272	0.5282629	208	87	218	154	211	189
ZNF300	4	2	0.0567855	2	1	4	13	5	10
TMEM132D	3.5	1.8073549	0.4735253	2	0	0	0	5	2
TMEM132E	1.2727273	0.3479233	0.6798081	5	11	6	7	3	18
TMEM132B	1	0	0.9838634	1	0	1	0	0	2
TMEM132A	0.7792958	-0.359757	0.0397707	1892	2485	2723	1845	1878	1810
MIR616	2	1	0.5386162	2	0	3	1	7	2
PHKB	1.0401514	0.0567936	0.4560289	5478	3754	5562	4051	5771	5566
MIR621	0.4829157	-1.050157	0.0002549	133	135	171	67	64	81
ADCY10	0.8879668	-0.171422	0.6794379	116	56	69	38	94	82
AL390198.2	0	#NUM!	0.4217812	0	0	5	0	0	0
AL390198.1	1.6666667	0.7369656	0.388117	6	7	5	7	13	10
CBWD6	1.2531969	0.3256131	0.2014627	160	59	172	173	187	130
CBWD5	1.2191465	0.2858715	0.0210277	575	462	697	681	751	682
TTLL10-AS1	2	1	0.6476092	0	0	2	3	1	0
CBWD3	1.1036585	0.1422939	0.3942993	248	139	269	190	240	294
CBWD2	1.0624751	0.0874291	0.3747672	905	569	1039	771	1005	894
CBWD1	1.2871464	0.3641762	0.0016918	912	776	1105	1056	1383	1156
KARSP2	0	#NUM!	0.4217812	0	0	5	0	0	0
KARSP1	#DIV/0!	#DIV/0!	0.5070017	0	0	0	3	0	0
MIR3690	0	#NUM!	0.1670145	3	1	3	0	0	0
HAUS4	0.9404125	-0.088634	0.851886	346	441	522	349	481	401
AC018553.2	0.0344828	-4.857981	0.0049811	2	21	6	0	1	0
HAUS3	1.2021818	0.2656551	0.0965614	547	309	519	469	619	565
AC018553.1	2.2	1.1375035	0.4712262	3	0	2	3	7	1
HAUS6	1.3191489	0.3996075	0.0140689	1162	675	1171	977	1533	1458
HAUS5	0.7943925	-0.332076	0.0625818	1039	1022	1470	895	1039	871
HAUS2	0.9216329	-0.117736	0.5890304	1144	1037	1175	809	1180	1104
HAUS1	1.0525148	0.0738405	0.382397	426	393	533	454	526	443
MIR3685	0.755102	-0.405256	0.7099185	22	3	24	15	8	14
AL928970.1	0.5	-1	0.7259387	1	0	5	0	0	3
MIR3682	0.84375	-0.245112	0.7732052	13	10	9	3	22	2
C1DP1	0	#NUM!	0.824326	1	0	0	0	0	0
FIP1L1	1.3014996	0.3801749	0.0060136	354	395	518	552	560	537
HAUS8	0.9562586	-0.064527	0.9567884	1061	1245	1329	1096	1239	1141
MIR3677	0.5	-1	0.7913401	0	1	3	2	0	0
HAUS7	0.6153846	-0.70044	0.4092792	11	17	11	1	15	8
C1DP5	0	#NUM!	0.824326	0	0	1	0	0	0
C1DP4	0	#NUM!	0.824326	0	0	1	0	0	0

C1DP3	1.25	0.3219281	0.7503101	2	2	4	6	3	1
C1DP2	8	3	0.0027856	4	0	2	14	22	12
CYP51A1	0.8071429	-0.309104	0.5053473	56	48	36	28	37	48
MIR3668	0	#NUM!	0.6436235	2	0	0	0	0	0
MIR3662	0	#NUM!	0.284725	6	0	0	0	0	0
PI15	2.625	1.3923174	0.2221844	3	0	5	11	7	3
PI16	0.1666667	-2.584963	0.4522891	0	0	6	0	1	0
NECTIN4	1.3443171	0.4268735	4.67E-05	1271	1297	1620	1723	2088	1819
NECTIN3	1.3513514	0.4344028	0.0092796	924	540	793	792	1099	1159
NECTIN2	0.9659831	-0.04993	0.9308726	5686	7363	7676	6538	6894	6588
NECTIN1	0.9678509	-0.047143	0.9775211	2420	2884	2690	2186	2762	2789
ISCA2	1.1042535	0.1430715	0.265684	344	417	438	503	434	387
ISCA1	1.0925064	0.1276417	0.1034508	2050	2136	2473	2159	2581	2535
RN7SL834P	0.5555556	-0.847997	0.6850588	0	9	0	3	0	2
FAM89B	5	2.3219281	0.1442915	1	1	1	10	5	0
FAM89A	0.876536	-0.190115	0.3825133	939	1179	1300	978	1020	998
CBLC	1.1888218	0.2495324	0.0465856	749	935	964	1119	1043	986
CBLB	1.4850969	0.570557	2.17E-06	1870	1635	1863	2132	3104	2736
AC119403.1	1.1538462	0.2064509	0.7965279	2	1	10	4	9	2
AC119403.2	0.5714286	-0.807355	0.7668514	1	0	6	2	2	0
RNU6-925P	0.5555556	-0.847997	0.7345817	0	0	9	0	4	1
AC007249.1	1.8571429	0.8930848	0.3829653	5	8	1	8	10	8
AC007249.2	1.1525424	0.2048198	0.563734	36	48	34	38	55	43
AC105935.1	1	0	0.9897585	0	3	2	0	0	5
TRERNA1	1.5	0.5849625	0.8233276	0	2	0	3	0	0
AL831711.1	0	#NUM!	0.824326	1	0	0	0	0	0
AL354726.1	2.3333333	1.2223924	0.6086496	3	0	0	0	3	4
RN7SL832P	1.3809524	0.4656636	0.318142	18	16	29	24	19	44
AC015522.1	0.375	-1.415037	0.5136197	8	0	0	0	1	2
KRT33B	3.5	1.8073549	0.2427258	1	0	3	1	5	8
KRT33A	2.5	1.3219281	0.5400818	0	0	4	0	10	0
MAATS1	1.7678571	0.8220017	0.0991116	19	7	30	33	38	28
CASP16P	1.6666667	0.7369656	0.6750575	1	4	1	0	2	8
AC004596.1	0.4642857	-1.106915	0.3233648	11	15	2	8	3	2
RAET1K	0.9264706	-0.110183	0.9644145	20	11	37	13	20	30
SH3TC2-DT	0.6666667	-0.584963	0.8272397	3	0	0	0	1	1
AL449106.1	0.9230769	-0.115477	0.9590687	1	10	2	7	5	0
AC064850.1	1.4	0.4854268	0.5173307	4	6	10	11	11	6
XIAPP1	#DIV/0!	#DIV/0!	0.6411933	0	0	0	0	0	2
XIAPP3	2	1	0.807847	1	0	0	0	1	1
XIAPP2	0.5	-1	0.81753	2	0	0	0	0	1
AL133371.3	2	1	0.7812375	0	0	1	1	0	1
AL133371.2	0	#NUM!	0.824326	0	1	0	0	0	0
AL133371.1	0	#NUM!	0.5781912	0	0	3	0	0	0
RNU6-921P	8	3	0.232169	0	0	1	8	0	0
CBR1	#DIV/0!	#DIV/0!	0.3981469	0	0	0	4	0	0
TUBB	0.9092088	-0.137316	0.5272747	36202	44015	47020	37579	41618	36488
AC009404.1	1.1111111	0.1520031	0.7699614	28	15	20	11	31	28
TOPORS	1.5075825	0.592237	0.0008588	852	463	927	856	1271	1253
RNU6-920P	#DIV/0!	#DIV/0!	0.3096126	0	0	0	5	0	0
AC011458.1	#DIV/0!	#DIV/0!	0.1513955	0	0	0	0	4	4
GPBP1L1	1.0569636	0.0799258	0.2612215	2555	2367	2890	2266	3016	2975
CBR4	1.2483829	0.3200605	0.0488717	249	194	330	268	336	361
CBR3	0.875	-0.192645	0.9076509	3	2	3	1	5	1

CBSL	2.7333333	1.4506614	0.000486	17	21	22	51	64	49
LHFPL5	0	#NUM!	0.1381401	3	5	0	0	0	0
FAM86C2P	0.8492462	-0.235745	0.4812394	124	135	139	108	105	125
LHFPL3	1	0	0.9782189	11	6	10	4	14	9
LHFPL2	0.8236617	-0.279876	0.1222532	714	669	840	529	618	684
LHFPL1	0.7857143	-0.347923	0.7334667	11	6	11	1	8	13
LHFPL6	1.2433993	0.3142897	0.0023432	1161	1136	1339	1427	1549	1545
IGSF11-AS1	0	#NUM!	0.824326	0	1	0	0	0	0
PNKDP1	0	#NUM!	0.6410557	1	1	0	0	0	0
TRBV14	#DIV/0!	#DIV/0!	0.6411933	0	0	0	0	0	2
TRBV16	0	#NUM!	0.6385096	0	2	0	0	0	0
RPSAP14	0	#NUM!	0.824326	1	0	0	0	0	0
RPSAP13	0	#NUM!	0.6840298	0	0	2	0	0	0
RPSAP12	1.2068966	0.271302	0.2485674	79	103	108	152	104	94
RPSAP18	3.5	1.8073549	0.2668589	4	0	0	8	1	5
NRCAM	0.2955665	-1.758445	0.0001171	78	54	71	12	17	31
RPSAP16	0	#NUM!	0.824326	1	0	0	0	0	0
RPSAP15	1.0286513	0.040754	0.584295	417	518	496	476	551	445
FAM81B	0	#NUM!	0.3026413	2	3	0	0	0	0
FAM81A	0.8403361	-0.250962	0.535459	116	79	162	90	108	102
RPSAP19	0.8388626	-0.253494	0.4460165	117	143	162	116	138	100
PHYH	1.1040381	0.14279	0.1289544	1149	1067	1350	1323	1434	1180
SFTPD-AS1	#DIV/0!	#DIV/0!	0.2685846	0	0	0	1	2	2
SYCE1L	1	0	0.9671711	5	0	0	0	4	1
TUG1	1.0422097	0.0596456	0.524754	2588	1460	2704	1759	2650	2628
TUFM	0.8475755	-0.238586	0.2213549	5586	6473	6770	5744	5338	4877
AC025166.1	0.1666667	-2.584963	0.3616229	4	0	2	0	0	1
FAM83H	0.9029635	-0.14726	0.4213313	3334	3772	3962	3058	3616	3320
AC087721.1	0	#NUM!	0.824326	0	0	1	0	0	0
FAM83G	1.2640638	0.3380693	0.0018221	1422	1567	1775	1655	2163	2204
TRPM2-AS	#DIV/0!	#DIV/0!	0.8030358	0	0	0	0	1	0
FAM83F	1.8518519	0.8889687	0.0102692	35	42	58	113	82	55
FAM83E	1.75	0.8073549	0.6220796	0	1	3	7	0	0
FAM83D	0.9978629	-0.003087	0.6450547	3490	3925	4283	3535	4130	4008
FAM83B	0.9581967	-0.061606	0.9036993	496	295	429	242	456	471
FAM83A	1.3333333	0.4150375	0.8450489	1	0	2	0	2	2
CBX8	0.8986928	-0.1541	0.6130369	202	200	210	166	208	176
CBX7	0.9640103	-0.05288	0.9232585	219	249	310	254	235	261
CBX6	0.7347543	-0.444666	0.0140451	495	597	597	364	469	408
CBX5	0.7522663	-0.410685	0.0148926	4858	4102	4498	2377	3694	4053
CBX4	0.9917741	-0.011917	0.7388972	1003	1306	1338	1186	1232	1199
CBX3	1.1582566	0.2119549	0.047565	10847	7342	11775	10912	12621	11173
CBX2	1.1564356	0.209685	0.079518	676	850	999	784	1124	1012
CBX1	0.9945959	-0.007818	0.673885	5419	5263	6157	4813	6044	5891
LRP1B	0.125	-3	0.2471647	5	0	3	0	0	1
NMUR2	2	1	0.834705	0	0	1	0	2	0
LRP10	0.8293498	-0.269947	0.0839219	3883	4773	4816	3461	3943	3769
LRP12	0.6840959	-0.54773	0.0681734	143	150	166	59	120	135
LRP11	0.0322581	-4.954196	0.002935	11	5	15	0	0	1
CBY3	0.75	-0.415037	0.8064377	7	0	5	0	3	6
CBY1	1.2205387	0.2875181	0.102285	193	186	215	260	267	198
OSTN-AS1	0	#NUM!	0.1554035	0	1	8	0	0	0
YBX2	1.3406114	0.4228911	0.0948246	68	63	98	103	120	84
YBX1	1.0826617	0.1145826	0.1156745	25573	28931	33348	30128	34313	30673

YBX3	0.5464371	-0.871873	3.85E-13	6137	6699	7611	3524	3998	3651
MIR589	0.754717	-0.405992	0.5974103	16	12	25	6	18	16
SPRED3	0.32	-1.643856	0.0627508	25	19	6	3	11	2
AC139887.4	3.1666667	1.662965	0.240517	2	4	0	2	5	12
SPRED2	1.0632184	0.088438	0.3203447	1517	1543	1638	1313	1870	1812
SPRED1	0.9869138	-0.019004	0.9540201	336	276	305	203	367	335
LRP5L	0.7727273	-0.371969	0.3708536	47	70	81	37	62	54
MIR593	0	#NUM!	0.6436235	2	0	0	0	0	0
AC136616.1	0.7333333	-0.447459	0.59729	15	23	37	28	11	16
FAM85B	1	0	0.9647111	1	2	0	0	3	0
MIR590	0.4153846	-1.26748	0.0848854	18	19	28	2	13	12
AC139887.1	#DIV/0!	#DIV/0!	0.1700664	0	0	0	0	9	0
AC139887.2	0.9566396	-0.063953	0.9918971	130	81	158	91	112	150
SLFNL1-AS1	0.6458333	-0.630766	0.2911629	19	32	45	21	24	17
FAM84A	1.2126963	0.2782183	0.0368161	486	508	534	500	727	626
AC016590.3	0.9487179	-0.075949	0.9633088	26	31	21	31	29	14
AC016590.2	0.2142857	-2.222392	0.2246828	1	6	7	0	0	3
AC016590.1	1.0338983	0.0480943	0.7846508	15	13	31	25	13	23
FAM84B	0.7738884	-0.369803	0.0957966	329	356	372	184	315	319
AC107982.3	0.5	-1	0.680026	3	1	0	0	1	1
THBS2	1.2131148	0.278716	0.5802035	25	11	25	17	39	18
THBS1	1.1923077	0.2537566	0.6961942	2	13	11	13	6	12
THBS4	#DIV/0!	#DIV/0!	0.5415473	0	0	0	0	2	1
THBS3	1.7209302	0.7831886	0.147953	12	14	17	19	22	33
SND1-IT1	0.1666667	-2.584963	0.4002565	0	1	5	0	1	0
FAM87B	0	#NUM!	0.5075476	0	0	4	0	0	0
FAM87A	1.6666667	0.7369656	0.7816572	1	2	0	0	0	5
TCF7L2	1.3659925	0.4499496	0.0001147	2073	2081	2521	2357	3239	3522
TCF7L1	0.8954839	-0.159261	0.5013735	1126	1351	1398	1179	1249	1042
AC139491.2	0	#NUM!	0.824326	1	0	0	0	0	0
ZNF296	1.1491228	0.200533	0.4512632	41	92	95	96	104	62
PID1	2.8333333	1.5025003	0.0191637	9	4	11	27	22	19
OSGEPL1	1.0254083	0.0361985	0.6244036	392	310	400	303	411	416
ZNF292	1.0045814	0.0065944	0.8625292	2520	1150	2660	1468	2476	2415
AL592309.2	0	#NUM!	0.824326	1	0	0	0	0	0
AL592309.1	#DIV/0!	#DIV/0!	0.6554062	0	0	0	0	2	0
A1BG-AS1	0.5918367	-0.756729	0.3230694	17	19	13	5	7	17
MEF2A	0.821645	-0.283413	0.2086101	403	310	442	234	374	341
ZNF287	0.8407407	-0.250267	0.6869989	81	66	123	100	54	73
MEF2C	1.1923077	0.2537566	0.7175435	7	28	17	11	39	12
migration;GO:(	#DIV/0!	#DIV/0!							
n;GO:0035984	#VALUE!	#VALUE!	napse;GO:0'1|hsa04921|hrathyroid h(enhancer factor 2C						
ZNF285	#DIV/0!	#DIV/0!	0.6554062	0	0	0	0	2	0
MEF2B	0.8	-0.321928	0.8695068	2	2	1	1	3	0
ZNF284	1.7755102	0.8282337	0.1310422	19	21	9	31	28	28
ZNF283	0.990676	-0.013515	0.864752	164	94	171	116	165	144
ZNF282	0.8486933	-0.236685	0.1968058	890	986	1032	738	816	914
ZNF281	0.9143252	-0.129221	0.6031795	3183	2320	3181	1685	2988	3267
SETMAR	0.9151426	-0.127931	0.734935	436	405	526	432	399	420
AP000777.2	0	#NUM!	0.0671316	2	7	1	0	0	0
THEGL	1.5	0.5849625	0.8695629	0	2	0	0	0	3
LETMD1	0.7855808	-0.348168	0.0381501	1082	1124	1539	832	1021	1089
ZNF277	1.4808975	0.5664718	0.0005247	633	381	635	770	929	743
ZNF276	0.8810573	-0.182692	0.5300646	211	208	262	181	232	187

PIF1	0.9090171	-0.137621	0.6281459	762	698	1002	707	776	755
ZNF275	0.6393443	-0.645335	0.0010234	424	380	416	204	277	299
ZNF274	0.8073733	-0.308692	0.063263	1016	968	1271	831	903	894
ZNF273	0.959203	-0.060092	0.9271867	358	287	409	221	427	363
GRPEL1	1.0510382	0.0718151	0.3158906	1438	1758	1957	1794	1880	1742
GRPEL2	1.0234417	0.0334289	0.596126	1298	1093	1363	958	1494	1390
ZNF268	0.9707602	-0.042813	0.9010842	429	167	430	314	357	325
ZNF267	1.1117647	0.1528515	0.3038179	672	392	636	484	780	626
ZNF266	0.7898383	-0.340371	0.1843574	495	289	515	307	332	387
PIGUP1	10	3.3219281	0.1608246	0	1	0	0	5	5
ZNF264	0.6808824	-0.554523	0.0556665	224	236	220	78	172	213
AC005771.1	0.5	-1	0.8638172	0	0	2	1	0	0
ZNF263	0.77362	-0.370303	0.0167311	1475	1725	1637	1086	1407	1249
ZNF260	1.1456386	0.196152	0.2460637	518	286	480	383	622	466
FANCD2P2	0.5384615	-0.893085	0.5714919	1	10	2	2	0	5
FANCD2OS	2	1	0.4490927	1	0	6	3	8	3
ZNF257	0.9939247	-0.008792	0.7836549	254	209	360	213	302	303
ZNF256	0.771028	-0.375145	0.089897	376	363	331	241	344	240
PIGS	0.8210348	-0.284485	0.0448492	2169	2202	2703	1702	2174	1932
ZNF254	1.1542192	0.2069172	0.2875156	423	236	372	275	532	383
PIGR	2	1	0.8324799	1	0	0	0	2	0
ZNF253	0.9364676	-0.094699	0.8444316	311	186	290	207	292	238
PIGU	0.8293458	-0.269954	0.0871163	1375	1579	1769	1166	1441	1310
PIGT	0.9381403	-0.092124	0.7912867	3670	4387	4827	3840	4337	3910
ZNF251	0.8924205	-0.164204	0.7497688	141	89	179	116	120	129
PIGO	0.9450491	-0.081539	0.8485726	1120	1264	1383	1102	1256	1202
ZNF250	0.9639344	-0.052993	0.9231681	95	81	129	109	103	82
PIGN	0.9770408	-0.033509	0.9694207	308	187	289	164	267	335
PIGQ	1.1657754	0.2212899	0.1300367	261	266	408	404	371	315
PIGP	1.0130814	0.0187501	0.6854433	214	222	252	235	250	212
LRRC4	0.6666667	-0.584963	0.7970114	0	3	3	1	3	0
LRRC3	1.4735178	0.5592645	5.90E-05	718	951	861	1150	1327	1251
PIGZ	0.3478261	-1.523562	0.273147	0	11	12	3	2	3
LRRC6	4.6153846	2.2064509	0.0039336	5	2	6	27	19	14
PIGW	0.71422	-0.48556	0.0021531	700	685	788	445	572	535
PIGV	0.9194313	-0.121186	0.7726544	293	332	430	319	347	304
LRRC2	0.6666667	-0.584963	0.8287635	3	3	0	4	0	0
LRRC1	0.9528282	-0.069712	0.908111	1363	1570	1752	1342	1677	1445
PIGX	0.832943	-0.26371	0.2964537	363	453	465	385	335	347
CTLA4	1.3333333	0.4150375	0.8730834	0	3	0	2	2	0
NELFCD	0.8922306	-0.164512	0.3706551	2912	3083	3581	2739	3006	2799
ZNF248	1.2234589	0.2909656	0.0611037	441	285	442	427	560	442
AC243965.2	0.2	-2.321928	0.201469	6	1	8	0	0	3
AC243965.1	0	#NUM!	0.6385096	0	2	0	0	0	0
LYPLA2	1.0782234	0.1086561	0.2005454	3369	4143	4556	4529	4421	4062
LYPLA1	0.9173663	-0.12443	0.5658736	2893	2439	3006	1813	2888	2948
PIFO	1.2171254	0.2834778	0.1662387	104	101	122	135	123	140
LRRD1	0	#NUM!	0.824326	0	0	1	0	0	0
GON7	1.2387333	0.3088656	0.0126976	519	495	628	641	773	620
PIGC	0.9066798	-0.141335	0.5641575	856	1021	1177	862	1047	860
PIGB	0.7657005	-0.385148	0.2367505	138	118	158	72	163	82
LRRC7	1.0581395	0.0815299	0.7921366	34	20	32	21	35	35
PIGA	1.0058725	0.0084474	0.7726196	433	276	483	276	449	474
LRRC9	1.2608696	0.334419	0.682164	3	7	13	3	16	10

PIGK	1.164271	0.219427	0.0914857	1083	782	1057	961	1409	1032
TMEM106C	0.9946899	-0.007681	0.6702938	5664	6908	7390	6403	6969	6484
AC018804.1	2.1666667	1.1154772	0.4738486	2	2	2	0	2	11
AL391669.1	0.25	-2	0.5622508	0	1	3	1	0	0
TMEM106B	0.8637979	-0.211234	0.3514414	1960	1194	1912	1034	1720	1622
TUT1	0.8154639	-0.294307	0.2732739	270	310	390	288	255	248
ZNF239	0.8761905	-0.190684	0.6893646	60	60	90	40	70	74
PIGM	0.8950131	-0.160019	0.5626137	426	330	387	331	351	341
TLR10	0	#NUM!	0.5781912	0	0	3	0	0	0
PIGL	1.1388013	0.187516	0.4521567	123	104	90	87	127	147
PIGG	0.9273016	-0.108889	0.7153347	971	947	1232	892	1040	989
ZNF236	0.9403292	-0.088762	0.8153452	153	167	166	89	184	184
PIGF	1	0	0.8293203	137	97	115	117	108	124
ZNF235	0.8310811	-0.266939	0.5754368	96	70	130	66	59	121
TMEM106A	0.9090909	-0.137504	0.8768676	1	10	0	0	8	2
ZNF234	0.9816327	-0.026745	0.9258985	181	135	174	125	161	195
PIGH	1.0117994	0.0169233	0.5994225	487	564	644	556	573	586
ZNF233	1.0983607	0.1353519	0.7636877	27	16	18	17	27	23
ZNF232	1.0789474	0.1096245	0.2589214	452	410	544	476	548	493
ZNF230	0.7419355	-0.430634	0.1681948	150	108	145	91	129	79
BGLAP	0	#NUM!	0.5781912	0	0	3	0	0	0
ZNF229	1.1223022	0.1664611	0.558833	53	37	49	44	56	56
ZNF227	0.8297101	-0.269321	0.320944	199	160	193	114	184	160
ZNF226	0.7685306	-0.379825	0.1752586	249	183	337	198	217	176
ZNF225	0.9871465	-0.018664	0.8515635	151	83	155	121	126	137
MEF2D	0.6533044	-0.614173	0.0001547	813	959	997	576	668	565
ZNF224	0.8225806	-0.281771	0.5116322	143	79	212	112	128	117
AC037459.3	0.625	-0.678072	0.5896932	9	7	0	2	4	4
ZNF223	#DIV/0!	#DIV/0!	0.8030358	0	0	0	0	0	1
AC037459.2	0.3571429	-1.485427	0.2951371	4	7	3	1	2	2
ZNF222	0	#NUM!	0.6436235	2	0	0	0	0	0
ZNF221	0.6413043	-0.640919	0.3601414	27	27	38	11	7	41
OR11M1P	0.5	-1	0.8652929	0	0	2	0	1	0
AL359915.2	0.6905738	-0.534133	0.0457149	165	141	182	103	132	102
ZNF219	0.866129	-0.207346	0.4131931	676	877	927	775	732	641
ZNF217	0.9474801	-0.077832	0.7844192	4366	3839	4514	2816	4544	4691
GOPC	1.1012406	0.1391297	0.2359598	1689	1196	1629	1252	1858	1861
ZNF215	0.9714567	-0.041778	0.9953135	422	224	370	224	398	365
ZNF214	1.0441176	0.0622843	0.8717192	33	17	18	16	19	36
ZNF213	0.7895349	-0.340925	0.1340073	247	289	324	209	252	218
ZNF212	0.962804	-0.054686	0.9062586	422	395	581	452	467	427
ZNF211	0.8508323	-0.233053	0.2792796	517	442	603	327	499	503
F13A1	#DIV/0!	#DIV/0!	0.8030358	0	0	0	1	0	0
BNIP3P10	1.7272727	0.7884959	0.4506933	3	1	7	5	2	12
SAYSD1	0.9913295	-0.012563	0.8202793	238	223	231	270	240	176
PTENP1	0.8	-0.321928	0.9219351	2	0	3	0	0	4
BNIP3P16	0.5	-1	0.3774281	7	8	5	1	5	4
BNIP3P17	1.21875	0.2854022	0.4247992	31	18	47	33	45	39
BNIP3P11	1.5555556	0.6374299	0.3659304	1	16	19	13	24	19
ZNF208	#DIV/0!	#DIV/0!	0.5509701	0	0	0	0	3	0
PIM1	0.8172836	-0.291091	0.0526739	4319	5184	5679	3826	4307	4275
ZNF207	0.9827654	-0.025081	0.7715919	5013	4433	5756	4389	5546	5005
PIM3	1.1723313	0.2293804	0.028107	3460	3728	4522	4872	4821	4035
ZNF205	1.0979432	0.1348034	0.2958052	255	351	415	395	398	328

PIM2	0.7810345	-0.356542	0.1731861	177	204	199	146	170	137
ZNF202	0.7947805	-0.331372	0.0997517	491	497	698	411	447	482
ZNF200	0.894822	-0.160327	0.4905595	603	522	729	471	639	549
AC005041.3	0.4782609	-1.06413	0.1113569	23	22	24	10	17	6
AC005041.2	#DIV/0!	#DIV/0!	0.5070017	0	0	0	3	0	0
AC005041.1	0	#NUM!	0.4117982	0	3	1	0	0	0
BNIP3P20	0	#NUM!	0.824326	0	0	1	0	0	0
AL135925.1	1.0941176	0.1297679	0.668407	31	16	38	29	27	37
BNIP3P27	1	0	0.9872082	3	0	4	0	5	2
BNIP3P26	1.6666667	0.7369656	0.7035687	2	4	0	0	2	8
BNIP3P29	0.6666667	-0.584963	0.8496791	5	0	1	4	0	0
BNIP3P22	2.6666667	1.4150375	0.596136	3	0	0	0	8	0
BNIP3P25	0.6666667	-0.584963	0.8234316	0	3	0	0	2	0
BNIP3P24	2.2941176	1.1979394	0.1717387	6	2	9	5	22	12
RAD54L2	0.7744302	-0.368793	0.0277612	1153	1288	1376	745	1063	1148
AL357518.1	0	#NUM!	0.824326	0	0	1	0	0	0
ATXN3	1.1273243	0.1729026	0.1168578	1088	790	1295	1063	1375	1139
ATXN2	0.9362477	-0.095038	0.7212592	1690	1704	2096	1301	1962	1877
ATXN1	0.2998084	-1.737887	1.53E-13	346	307	391	69	107	137
ATXN7	0.8453515	-0.242377	0.2744313	822	613	770	407	701	756
THEM5	9.7222222	3.2812861	3.19E-09	5	6	7	64	74	37
THEM4	1.2939124	0.3717399	0.0011495	838	721	1020	1033	1149	1155
THEM6	0.9601329	-0.058694	0.9219727	317	441	446	473	337	346
C9orf85	1.2756489	0.3512314	0.0118107	589	452	577	650	793	621
C9orf84	0.7777778	-0.36257	0.7910572	9	3	6	6	4	4
RARRES2P2	0	#NUM!	0.5387425	3	0	0	0	0	0
ALG3P1	#DIV/0!	#DIV/0!	0.8030358	0	0	0	0	0	1
GOT1	1.4799091	0.5655086	1.56E-06	2811	3298	3572	4870	5023	4434
GOT2	0.8493605	-0.235551	0.1134822	6825	7689	8395	6078	7039	6341
PIN1	0.9920958	-0.011449	0.7187372	1117	1467	1591	1431	1460	1251
PIN4	1.123348	0.1678049	0.0894044	1057	1045	1530	1393	1386	1301
CATIP-AS2	1.3333333	0.4150375	0.6864986	1	7	7	2	6	12
AC004692.1	#DIV/0!	#DIV/0!	0.8030358	0	0	0	1	0	0
HSP90AB1	0.8971172	-0.156632	0.3290984	65100	70124	80468	58422	70626	64453
AC002056.1	0.9816514	-0.026717	0.9566649	32	50	27	45	31	31
AC005281.1	0.75	-0.415037	0.8724887	4	0	0	0	0	3
LRRK2	1.3333333	0.4150375	0.857748	0	3	0	2	1	1
RASGRF2	1.1900711	0.2510478	0.0213483	2259	1837	2370	2039	2892	2764
LRRK1	0.7063622	-0.50152	0.007871	564	565	710	301	519	479
RASGRF1	0.7580398	-0.399654	0.0103174	1267	1245	1406	758	1110	1102
SIRPAP1	#DIV/0!	#DIV/0!	0.6099294	0	0	0	2	0	0
HMG20B	1.1137956	0.1554846	0.121566	1651	1723	2048	2155	2160	1724
C9orf66	1.0307692	0.0437214	0.7393836	85	35	75	80	62	59
CCIN	0.7657143	-0.385122	0.4105861	45	60	70	38	35	61
HMG20A	0.9226566	-0.116134	0.6469698	760	811	808	470	807	918
C9orf64	1.1785888	0.2370605	0.0497921	637	706	712	687	852	883
AC004702.1	#DIV/0!	#DIV/0!	0.6554062	0	0	0	0	2	0
HSP90AA1	1.1723384	0.2293891	0.0897196	55399	30012	58254	47888	65312	55224
NDUFC2	0.7560976	-0.403356	0.2155624	103	103	163	69	115	95
NDUFC1	1.2767645	0.3524924	0.0250292	361	414	486	607	571	432
PARD3B	0.9296435	-0.10525	0.723142	395	308	363	219	399	373
C9orf78	1.0735746	0.1024224	0.1900071	1690	1972	2196	1928	2206	2155
C9orf72	1.1066499	0.1461989	0.312197	266	187	344	232	321	329
NDUFB9	1.0410765	0.0580761	0.45612	591	733	794	845	685	675

NDUFB8	2.1176471	1.0824622	0.0266705	19	15	17	26	45	37
NDUFB7	0.9580741	-0.061791	0.9304697	2104	2531	2759	2769	2329	1986
NDUFB6	1.125753	0.1708903	0.1439174	687	910	1059	1011	1109	870
RBMY2EP	2.8	1.4854268	0.296181	1	0	4	3	2	9
NDUFB5	0.9650134	-0.051379	0.9046942	2306	2296	2858	2364	2513	2322
KHDC1L	0.2857143	-1.807355	0.4181372	5	2	0	1	1	0
NDUFB4	1.011518	0.0165219	0.5428616	1677	1940	2200	2014	2054	1816
NDUFB3	1.1859074	0.2459913	0.0204912	1270	1401	1842	1799	1879	1674
CXXC1	0.8976378	-0.155795	0.5003157	1293	1446	1706	1318	1468	1204
NDUFB2	0.8589744	-0.219313	0.5489668	193	248	261	238	221	144
NDUFB1	1.0373533	0.0529073	0.4397772	570	565	739	629	738	577
PARD6G	0.7657993	-0.384962	0.3165978	77	78	114	61	60	85
CXXC5	1.1342863	0.1817849	0.044003	1387	1490	1740	1599	1905	1733
CXXC4	0.4265734	-1.229134	0.0153123	57	36	50	24	14	23
BNIP3P38	#DIV/0!	#DIV/0!	0.6411933	0	0	0	0	0	2
BNIP3P37	3	1.5849625	0.5002013	0	0	2	0	2	4
PARD6B	0.8684901	-0.203419	0.3205272	988	736	945	592	858	868
BNIP3P39	1.3125	0.3923174	0.7887788	3	11	2	0	10	11
PARD6A	0.6632653	-0.592342	0.3409633	14	65	19	16	22	27
BNIP3P34	1.5	0.5849625	0.7447237	3	0	3	0	7	2
CCL5	7.1038961	2.8286105	3.29E-22	26	22	29	173	199	175
CCL2	0.5	-1	0.6571007	2	4	2	4	0	0
IARS2	0.9507365	-0.072883	0.8379332	4777	4209	5203	3689	5032	4769
C9orf43	1.1621622	0.2168114	0.6998541	19	8	10	21	15	7
C9orf40	1.0842057	0.1166385	0.1532887	1405	1392	1597	1541	1628	1595
NDUFA9	0.781362	-0.355937	0.3448277	86	72	121	68	85	65
NDUFA8	1.0168397	0.0240923	0.4895997	2373	2600	2925	2595	2916	2520
NDUFA7	0.6923077	-0.530515	0.5675868	5	28	6	12	7	8
NDUFA6	0.9665535	-0.049079	0.8567473	2734	3042	3941	3299	3362	2731
NDUFA5	1.0112958	0.0162051	0.6288281	1610	1276	1806	1263	1897	1585
NDUFA4	1.0184253	0.0263402	0.4743812	7207	8389	9641	8670	9291	7741
NDUFA3	1.0392321	0.0555178	0.4360858	716	791	889	865	899	726
NDUFA2	1.0057661	0.0082948	0.6636123	340	401	473	476	406	339
NDUFA1	1.0625798	0.0875712	0.2895665	1108	1284	1523	1449	1492	1219
AC018554.1	2.3333333	1.2223924	0.6407536	0	3	0	0	0	7
BNIP3P40	0.5	-1	0.8652929	0	0	2	0	1	0
BNIP3P42	1	0	0.9890559	0	0	1	0	0	1
CCM2	1.038961	0.0551416	0.4624503	711	940	967	1001	853	866
PRC1-AS1	0.2666667	-1.906891	0.2626934	12	0	3	0	3	1
AC243725.1	0	#NUM!	0.6385096	0	2	0	0	0	0
C9orf50	0.5714286	-0.807355	0.6564263	2	5	0	0	2	2
CCNK	0.7043151	-0.505707	0.0023142	1038	1207	1208	617	883	932
CCNI	1.042446	0.0599727	0.2817022	3990	3916	4604	3933	4820	4288
CCNH	1.1898507	0.2507806	0.0349533	1210	838	1302	1242	1429	1315
CCNF	0.9007926	-0.150733	0.4206142	2202	2497	2871	2012	2508	2299
AC107983.2	1.0297619	0.0423108	0.9121359	74	39	55	15	54	104
AC107983.1	0.6666667	-0.584963	0.8050508	2	1	3	2	2	0
CCNC	1.0387981	0.0549152	0.3645975	3041	2417	3228	2635	3313	3075
C9orf24	0	#NUM!	0.824326	0	0	1	0	0	0
FAM53C	0.8623284	-0.213691	0.2018705	1691	1676	1950	1188	1679	1718
FAM53B	1.0644639	0.090127	0.2829547	843	1060	1184	1041	1125	1120
FAM53A	0.8181818	-0.289507	0.937983	2	2	7	6	3	0
AC006512.1	#DIV/0!	#DIV/0!	0.242062	0	0	0	6	0	0
AC004453.1	1.0571429	0.0801703	0.7543341	33	65	42	55	53	40

RN7SL845P	9	3.169925	0.2317136	0	1	0	0	0	9
MIR9-3	0	#NUM!	0.6840298	0	0	2	0	0	0
AC004453.2	0	#NUM!	0.824326	1	0	0	0	0	0
PISD	0.8857234	-0.175072	0.3952869	957	1007	1160	894	938	935
AC119150.1	1.5	0.5849625	0.6620483	1	0	5	4	0	5
CCNY	1.1966285	0.2589754	0.0063404	2476	2703	2948	2843	3584	3298
LACTB2	0.8311688	-0.266787	0.3485388	245	165	283	155	231	190
CCNQ	0.9399494	-0.089345	0.9406365	443	505	634	541	501	445
CCNO	1.3333333	0.4150375	0.7728949	7	1	4	1	14	1
AL606760.3	#DIV/0!	#DIV/0!	0.2327526	0	0	0	0	0	7
AL606760.2	0	#NUM!	0.3052077	1	4	0	0	0	0
AL606760.1	3.0714286	1.6189098	0.1109232	11	2	1	5	22	16
FAM86B3P	0.9340659	-0.098404	0.9497683	93	59	121	85	94	76
AC096638.2	0	#NUM!	0.1996036	0	0	9	0	0	0
CD19	0.2875	-1.798366	0.01728	11	31	38	4	15	4
SLAMF9	1	0	0.9890559	1	0	0	1	0	0
SLAMF7	6	2.5849625	0.0207836	2	4	0	8	10	18
SURF6	0.9569213	-0.063528	0.9735828	1482	1780	1961	1742	1651	1605
CD14	1.125	0.169925	0.8539354	1	4	3	5	0	4
SLAMF1	2	1	0.8186965	0	0	1	0	0	2
CCR1	1.1587302	0.2125446	0.3986831	61	33	95	77	70	72
SURF1	1.0480387	0.0676919	0.3408	1011	1085	1422	1342	1170	1175
IQSEC2	0.8571429	-0.222392	0.6092467	117	131	200	137	110	137
LRRN3	#DIV/0!	#DIV/0!	0.356754	0	0	0	1	1	2
C9orf16	1.132123	0.1790308	0.181102	496	738	749	833	716	696
IQSEC1	0.9798937	-0.029303	0.912638	2124	2326	2513	1740	2474	2609
LRRN2	0.15	-2.736966	0.0003499	31	17	32	2	6	4
SURF2	1.1521197	0.2042906	0.2003146	382	419	402	565	465	356
ADHFE1	1.8181818	0.8624965	0.063101	17	14	24	36	29	35
IQSEC3	0.3333333	-1.584963	0.6538103	0	0	6	2	0	0
LRRN4	0	#NUM!	0.0121386	7	5	4	0	0	0
SURF4	0.9374866	-0.09313	0.7317495	10728	13261	13251	10578	12670	11664
ACER2	0.8962888	-0.157964	0.5352088	624	687	656	358	613	792
ACER3	1.0671203	0.0937229	0.3603012	1198	868	1167	898	1281	1271
CD28	0.2	-2.321928	0.1816032	5	3	7	0	0	3
AC108479.2	0	#NUM!	0.5337954	0	3	0	0	0	0
LRRN1	#DIV/0!	#DIV/0!	0.8030358	0	0	0	0	0	1
AC108479.1	0.9444444	-0.082462	0.9950603	2	8	8	7	5	5
CD24	0.6949979	-0.52492	6.35E-06	10043	9480	11244	6082	7867	7434
CD22	3.6363636	1.8624965	0.0370996	4	4	3	8	15	17
CD40	1.1428571	0.1926451	0.5373193	28	43	41	51	43	34
FAM57B	2.5	1.3219281	0.6749786	2	0	0	0	5	0
FAM57A	0.9656965	-0.050358	0.9587495	508	687	729	581	677	600
APOC4-APOC2	0	#NUM!	0.824326	0	0	1	0	0	0
CD3E	0.5	-1	0.812391	0	2	0	0	1	0
GRID1-AS1	#DIV/0!	#DIV/0!	0.3831122	0	0	0	0	5	0
AC006262.4	#DIV/0!	#DIV/0!	0.0158433	0	0	0	2	5	8
CCR9	2	1	0.7736272	0	1	0	2	0	0
CD38	0.5	-1	0.6695648	0	6	0	1	0	2
AC135048.4	#DIV/0!	#DIV/0!	0.8030358	0	0	0	0	1	0
CCR7	4.5791139	2.1950685	2.69E-26	76	117	123	459	563	425
CD37	0.6291209	-0.668591	0.0579737	144	113	107	47	66	116
AC135048.3	0	#NUM!	0.5378194	0	2	1	0	0	0
CD36	1.1470588	0.1979394	0.7125832	13	2	19	11	14	14

OR6D1P	0	#NUM!	0.824326	0	0	1	0	0	0
CCR5	1	0	0.9890559	0	0	1	0	0	1
CCR4	2	1	0.807847	1	0	0	0	1	1
CD34	0	#NUM!	0.824326	1	0	0	0	0	0
CCR3	1.0555556	0.0780025	0.8598875	19	14	21	8	23	26
CD33	3	1.5849625	0.6067982	0	1	0	2	0	1
CCT3	0.8908503	-0.166745	0.3885665	12770	16054	16948	13053	14674	13049
CD53	1.6944024	0.7607766	1.28E-05	176	227	258	316	414	390
CCT2	0.9439126	-0.083275	0.7845035	9337	8977	10623	8240	10049	9025
CD52	0.6	-0.736966	0.6730608	3	4	3	4	1	1
CICP27	0.1111111	-3.169925	0.1612038	5	3	1	0	0	1
IGLL4P	#DIV/0!	#DIV/0!	0.4501902	0	0	0	0	0	4
CICP22	0.9354839	-0.096215	0.9550416	9	11	11	9	11	9
AC006262.1	3.8	1.9259994	0.2103175	2	0	3	0	12	7
CICP24	0	#NUM!	0.824326	0	0	1	0	0	0
AC002545.1	0	#NUM!	0.824326	0	1	0	0	0	0
CICP26	#DIV/0!	#DIV/0!	0.6554062	0	0	0	0	2	0
PDRG1	1.106338	0.1457923	0.1617228	856	921	1063	1129	1091	922
AC093840.1	0.5714286	-0.807355	0.6646619	3	3	1	2	1	1
AL390195.2	#DIV/0!	#DIV/0!	0.263214	0	0	0	0	5	1
AL390195.1	0	#NUM!	0.5781912	0	0	3	0	0	0
CD47	1.16578	0.2212955	0.0130555	3694	3418	4542	3790	4985	4811
CD46	0.8884317	-0.170667	0.3955584	3029	2299	3178	1945	2710	2902
CICP17	0	#NUM!	0.824326	0	0	1	0	0	0
CD63	0.9449653	-0.081667	0.9223357	13704	15882	17984	15787	15611	13554
CICP16	0.8275862	-0.273018	0.8115299	5	8	16	1	11	12
CICP19	#DIV/0!	#DIV/0!	0.8030358	0	0	0	0	0	1
CICP18	0.1111111	-3.169925	0.0949179	0	15	3	0	0	2
MIS12	0.8305275	-0.2679	0.1406878	915	726	1032	639	822	759
CICP13	0.5	-1	0.81753	2	0	0	0	0	1
CICP14	0.64	-0.643856	0.0668805	145	138	167	37	114	137
AL133372.2	1.5	0.5849625	0.7500193	2	2	0	6	0	0
GPC1	0.8740902	-0.194146	0.3698301	3692	4636	4999	4071	3902	3676
GPC3	0.6215139	-0.686141	0.0556132	88	71	92	33	68	55
GPC2	1.3979592	0.4833222	0.0663335	59	53	84	89	92	93
RN7SL840P	0	#NUM!	0.824326	0	0	1	0	0	0
CD59	0.857868	-0.221172	0.2351009	1142	1120	1284	1015	1074	953
GPC5	0.1034483	-3.273018	0.0190296	13	10	6	0	0	3
CCT8	1.1927863	0.2543356	0.0068971	8347	6902	9260	8506	11173	9555
CD58	0.7078652	-0.498454	0.4064995	59	19	100	39	42	45
GPC4	1.9122231	0.9352509	1.43E-14	1378	1702	1796	2644	3371	3309
CCT7	0.9544218	-0.067301	0.9744549	14913	18299	19730	16849	17654	16026
CCT5	0.959789	-0.059211	0.9877988	17328	17802	21372	16289	19734	18207
CD55	1.3168006	0.3970369	2.81E-05	4101	3705	4634	4946	6070	5365
CCT4	0.9831457	-0.024523	0.7655047	12998	13410	15421	12438	15139	13547
CD74	4.0625	2.0223678	6.42E-19	76	81	83	345	368	262
CD72	2.2857143	1.1926451	0.3747892	0	0	7	3	4	9
CD70	1.32	0.4005379	0.5527228	8	5	12	10	13	10
DKKL1	1	0	0.909486	3	0	10	4	4	5
AC009403.1	0.6428571	-0.63743	0.2110491	89	26	67	22	38	57
AC009403.2	0	#NUM!	0.6385096	0	2	0	0	0	0
BAALC-AS1	0.4915254	-1.024662	0.1528786	17	15	27	7	11	11
GPAM	1.1421736	0.191782	0.1578487	998	881	1047	779	1187	1376
CD69	#DIV/0!	#DIV/0!	0.8030358	0	0	0	1	0	0

AL163192.1	#DIV/0!	#DIV/0!	0.2887809	0	0	0	0	0	6
CD86	0.0909091	-3.459432	0.2174974	0	0	11	0	0	1
CD83	0.6909091	-0.533432	0.5660969	7	22	26	19	7	12
CD82	0.6470588	-0.628031	0.6703572	5	2	10	0	3	8
CD81	0.8755204	-0.191787	0.4133444	3480	3804	4485	3773	3578	2953
CD80	#DIV/0!	#DIV/0!	0.2905913	0	0	0	2	0	3
GSN-AS1	2.25	1.169925	0.5141228	0	1	3	1	0	8
AC069218.1	#DIV/0!	#DIV/0!	0.6411933	0	0	0	0	0	2
AL731577.2	1	0	0.9447558	13	12	14	10	14	15
PPFIA1	0.9143739	-0.129144	0.5157202	2615	2411	2752	1771	2657	2684
EP300	0.818482	-0.288977	0.1384674	5482	5609	5563	2838	4906	5887
PPFIA4	0.9518717	-0.071161	0.9655212	528	389	579	443	501	480
PPFIA3	0.9114181	-0.133815	0.5902274	707	876	878	700	839	704
PPFIA2	0	#NUM!	0.6436235	2	0	0	0	0	0
CD96	1.25	0.3219281	0.7539655	2	4	6	2	6	7
RUNDC3B	1.245283	0.3164737	0.2863802	57	47	55	64	80	54
RUNDC3A	5	2.3219281	0.2104728	1	1	0	5	1	4
GPD2	1.2121212	0.277534	0.6858237	7	7	19	3	18	19
GPD1	2.5	1.3219281	0.5660017	1	0	1	1	0	4
SPTY2D1OS	0.9777778	-0.032421	0.9177307	22	7	16	23	7	14
TOR1AIP2	1.026464	0.037683	0.4397278	5108	4134	5495	4270	5562	5295
PDS5B	0.9798483	-0.02937	0.9221552	1622	1014	1582	1022	1638	1473
PDS5A	1.0761356	0.1058599	0.3746536	2977	1801	3037	2015	3304	3091
RASGRP2	0.9745405	-0.037206	0.8156923	2139	2483	2723	2720	2340	2098
RASGRP1	0.5862069	-0.770518	0.4630893	17	5	7	0	7	10
RASGRP4	0.6666667	-0.584963	0.8124442	2	1	0	0	1	1
TOR1AIP1	1.000451	0.0006504	0.7089823	1576	1242	1617	1212	1649	1576
MIRLET7A1	1	0	0.9890559	1	0	0	0	0	1
RASGRP3	5.2142857	2.3824696	0.0035538	10	1	3	14	38	21
H1FX	1.0264317	0.0376377	0.4855701	1171	1459	1683	1587	1557	1283
NDUFV3	0.9608476	-0.05762	0.9806905	1486	1596	1873	1539	1791	1431
NDUFV2	1.1595745	0.2135955	0.6174107	41	25	28	21	40	48
NDUFV1	0.8628838	-0.212762	0.3119179	5047	5622	6725	5379	5188	4442
CD99	1.0121859	0.0174743	0.5608745	4065	5157	5385	5149	5159	4477
CCZ1	0.926753	-0.109743	0.7480991	705	645	889	645	744	686
ISG15	5.1990834	2.3782573	1.04E-58	1992	2420	2570	13461	12368	10471
ISG20	0.9238095	-0.114333	0.9558473	29	33	43	43	34	20
H1F0	1.3128805	0.3927356	7.60E-05	8620	9885	10896	12261	13791	12548
RNU6-930P	#DIV/0!	#DIV/0!	0.3200852	0	0	0	0	2	3
AL513365.1	0.3333333	-1.584963	0.6622061	0	3	0	0	0	1
AL513365.2	0.3333333	-1.584963	0.2428531	4	7	7	0	4	2
BX255925.1	#DIV/0!	#DIV/0!	0.4980481	0	0	0	2	1	0
GPHN	0.782547	-0.353751	0.0235961	891	853	1075	630	810	766
KCTD9P6	2	1	0.807847	1	0	0	0	1	1
KCTD9P5	0	#NUM!	0.6436235	2	0	0	0	0	0
KCTD9P4	0.6363636	-0.652077	0.7080529	6	0	5	2	4	1
HSP90B2P	0.9375	-0.093109	0.9089605	23	2	7	3	16	11
KCTD9P2	1.2727273	0.3479233	0.7061297	3	4	4	8	3	3
AL109945.1	2.5	1.3219281	0.591979	0	0	2	0	3	2
A4GALT	1.4745405	0.5602655	1.37E-05	887	1142	1290	1604	1721	1569
PJA2	1.0181387	0.0259341	0.716683	3760	2325	3618	2167	3872	3840
PJA1	0.785498	-0.34832	0.0184496	1813	2082	2380	1512	1730	1687
NDUFS8	0.8741608	-0.194029	0.4887649	1609	1932	2268	1939	1760	1379
NDUFS7	0.8791946	-0.185746	0.8365757	31	55	63	53	26	52

NDUFS6	0.9946426	-0.00775	0.6751757	2121	2853	3239	3042	2772	2355
NDUFS5	1.0942629	0.1299594	0.1494889	4246	4677	5335	5514	5554	4534
NDUFS4	1.0145669	0.020864	0.5200373	1534	1569	1977	1665	1956	1533
NDUFS3	0.9064521	-0.141697	0.5860173	1722	2023	2284	1842	2065	1558
NDUFS2	0.7828796	-0.353138	0.0323717	1995	2576	2812	1841	2108	1831
NDUFS1	0.9724889	-0.040246	0.9604503	3685	3158	3989	2719	3970	3845
NIM1K	2.1333333	1.0931094	0.3143615	0	13	2	20	8	4
SPACA6P-AS	0.5909091	-0.758992	0.4734769	11	6	5	2	8	3
MIRLET7F1	0.4285714	-1.222392	0.5581142	5	2	0	0	3	0
ZNF29P	0.75	-0.415037	0.9117941	1	0	3	0	0	3
HSP90B3P	4.5	2.169925	0.3315903	1	1	0	0	7	2
AC021054.1	0.7537879	-0.407769	0.2572809	87	75	102	63	64	72
FAM50B	0.7960784	-0.329018	0.3318683	229	211	325	249	166	194
FAM50A	0.9296883	-0.105181	0.8187579	2639	3507	3895	3294	3284	2757
AL132640.2	4.2	2.0703893	0.1211232	4	0	1	15	4	2
ZNF177	0	#NUM!	0.824326	0	0	1	0	0	0
AL132640.1	1.9642857	0.9740048	0.1882743	13	1	14	19	26	10
ZNF175	2.5454545	1.3479233	0.2392067	8	0	3	4	6	18
ZNF174	0.9683406	-0.046413	0.9838995	234	375	307	290	316	281
ZNF169	0.6995516	-0.515498	0.0924785	138	143	165	74	93	145
UBE2R2-AS1	1.6666667	0.7369656	0.7792639	2	1	0	0	0	5
ZNF165	1.146328	0.1970199	0.1514559	647	470	694	741	791	544
AC133965.1	0	#NUM!	0.824326	0	0	1	0	0	0
SPATA8-AS1	#DIV/0!	#DIV/0!	0.6411933	0	0	0	0	0	2
ZNF160	1.0249138	0.0355025	0.6043358	992	569	1048	733	1031	910
SPIN2B	0.6930693	-0.528928	0.0827768	109	121	174	89	96	95
SPIN2A	0.4	-1.321928	0.5975473	1	4	0	0	0	2
ZNF157	#DIV/0!	#DIV/0!	0.8030358	0	0	0	0	0	1
ZNF155	0.5837321	-0.776622	0.0546776	73	69	67	22	43	57
ZNF154	0.9803922	-0.028569	0.9276373	47	20	35	37	26	37
AC008074.3	0.4	-1.321928	0.435518	1	7	2	0	2	2
AC008074.2	0	#NUM!	0.6840298	0	0	2	0	0	0
AC008074.1	#DIV/0!	#DIV/0!	0.6317974	0	0	0	1	1	0
AC106760.2	0	#NUM!	0.3912131	1	1	2	0	0	0
ZNF148	1.0031368	0.0045183	0.8299956	669	333	592	380	664	555
ZNF146	1.2575433	0.330608	0.0323351	2671	1407	2915	2484	3449	2861
RBMY1A1	2.1428571	1.0995357	0.2785873	8	4	2	4	15	11
ZNF143	1.2060932	0.2703414	0.0538551	565	381	728	605	739	675
ZNF142	0.8528104	-0.229703	0.2174795	809	772	892	566	731	812
ZNF141	0.9833148	-0.024275	0.9200301	368	168	363	202	364	318
ZNF140	0.7153614	-0.483256	0.0549728	241	170	253	138	189	148
CDC7	1.0722087	0.1005858	0.3445657	610	394	644	536	605	626
CDC6	0.8684432	-0.203497	0.1625788	3762	3698	4398	2936	3862	3500
AL355802.1	1.5555556	0.6374299	0.6145956	5	2	2	3	1	10
GPN2	0.9043739	-0.145009	0.5493484	703	757	872	679	743	687
GPN1	0.9537345	-0.06834	0.9584671	999	1058	1250	996	1092	1066
AL355802.2	#DIV/0!	#DIV/0!	0.1930205	0	0	0	0	3	4
GPN3	1.1500168	0.201655	0.0522647	949	794	1230	1072	1186	1161
MESTIT1	#DIV/0!	#DIV/0!	0.5509701	0	0	0	0	3	0
ZNF138	1.2458732	0.3171572	0.1238166	465	187	499	405	571	458
ZNF136	0.6918239	-0.531523	0.2516298	54	34	71	27	44	39
ZNF134	0.9006515	-0.150959	0.5502589	422	389	417	331	377	398
ZNF133	1.2841994	0.3608692	0.0122349	319	274	350	385	417	409
ZNF132	0.5057471	-0.983512	0.1274008	32	23	32	3	19	22

ZNF131	1.0577833	0.0810441	0.4024342	1488	934	1593	1134	1586	1527
AL355312.2	0.7	-0.514573	0.6203626	14	3	13	1	10	10
HAVCR1	1.1677444	0.2237245	0.2523788	967	806	856	567	1136	1367
ZNF124	0.879558	-0.185149	0.5583279	326	208	371	190	292	314
HAVCR2	0.2592593	-1.947533	0.111855	3	18	6	0	3	4
ZNF121	0.9068689	-0.141034	0.6448696	953	526	894	574	797	781
TIMM29	0.7975859	-0.326288	0.2062516	279	334	464	296	291	272
TIMM23	1.0232283	0.0331281	0.4119841	3624	3844	4371	3902	4254	3958
TIMM22	0.827265	-0.273578	0.1847788	620	851	891	626	698	630
TIMM21	0.9249191	-0.112601	0.6980959	464	493	588	406	551	472
OR5BH1P	#DIV/0!	#DIV/0!	0.2796611	0	0	0	4	1	0
ZNF117	0.9279176	-0.107931	0.8105118	362	165	347	182	367	262
ZNF114	1.0666231	0.0930505	0.3086087	494	418	619	539	583	511
ZNF112	0.6941748	-0.526629	0.1758239	54	70	82	37	53	53
GPS1	0.8681891	-0.203919	0.3057036	2814	3250	3351	2834	2665	2675
TIMM13	0.988568	-0.016588	0.7315784	881	1101	1342	1237	1185	864
GPS2	0.7419355	-0.430634	0.6883976	8	6	17	6	8	9
AC017083.1	3.1538462	1.6571123	0.06694	9	2	2	19	8	14
TIMM10	0.9609918	-0.057404	0.9145812	944	1099	1264	1204	1070	904
AC017083.3	#DIV/0!	#DIV/0!	0.288876	0	0	0	3	2	0
AC004941.1	0.25	-2	0.5155155	1	3	0	0	1	0
ATP6AP1L	0.9322493	-0.101212	0.8500445	124	103	142	91	128	125
ZNF107	1.2350427	0.304561	0.12295	268	161	273	207	364	296
AC243964.1	#DIV/0!	#DIV/0!	0.4737294	0	0	0	0	4	0
ALKAL2	1.125	0.169925	0.787312	0	4	12	7	6	5
ZNF106	0.8548857	-0.226196	0.2902512	3806	2934	3893	1921	3250	3919
AC243964.3	0.2307692	-2.115477	0.0827972	6	10	10	0	3	3
AC243964.2	6	2.5849625	0.3599688	0	0	1	0	4	2
ZNF101	0.8070557	-0.30926	0.0774714	700	795	801	570	676	607
ZNF100	1.0400829	0.0566986	0.5851906	559	268	620	416	597	492
GPR1	1.6311475	0.7058873	0.0052573	88	73	83	118	124	156
GPR4	0.125	-3	0.0933336	7	6	3	2	0	0
TYW1B	1.0535714	0.0752881	0.6663823	88	56	80	79	62	95
GPR3	0.6304348	-0.665581	0.4197363	6	30	10	7	8	14
AC090953.1	0	#NUM!	0.6840298	0	0	2	0	0	0
AC093019.1	1.5	0.5849625	0.7328778	3	0	1	6	0	0
RNU1-1	#DIV/0!	#DIV/0!	0.8030358	0	0	0	1	0	0
CTNND2	5	2.3219281	0.385839	0	0	1	4	0	1
CTNND1	0.9205264	-0.119469	0.5553259	18277	19385	20546	13392	19152	21038
CDH9	0	#NUM!	0.824326	0	0	1	0	0	0
CDH8	0.8774446	-0.18862	0.5758229	285	175	307	150	235	288
AL133373.2	0	#NUM!	0.5781912	0	0	3	0	0	0
CDH6	1.5	0.5849625	0.8175361	2	0	0	3	0	0
CDH5	1.181805	0.240992	0.0276067	1907	2480	2494	2550	2789	2793
CDH4	0.7407407	-0.432959	0.4534885	46	40	76	36	21	63
CDH3	0.4594241	-1.122102	1.26E-12	1091	1309	1420	597	594	564
AL732414.1	0.6756757	-0.565597	0.5079865	20	9	8	3	8	14
APMAP	0.798913	-0.32389	0.02514	3012	3520	3772	2480	3095	2657
CDH2	1.2222222	0.2895066	0.8535868	4	2	3	0	7	4
CDH1	0.9299483	-0.104778	0.6109678	31453	34123	36620	25709	34423	34905
AC073130.3	0.6	-0.736966	0.7466503	2	3	0	0	0	3
GPT2	1.2451404	0.3163084	0.0476245	225	296	405	391	365	397
AC073130.2	0.12	-3.058894	0.0680953	2	1	22	1	2	0
AC073130.1	1	0	0.9974913	5	1	5	0	9	2

OR11N1P	0	#NUM!	0.4374838	0	4	0	0	0	0
AC021785.1	0	#NUM!	0.824326	1	0	0	0	0	0
AC134312.1	0.1111111	-3.169925	0.0693661	5	4	9	0	0	2
LSMEM2	0	#NUM!	0.824326	0	1	0	0	0	0
LSMEM1	0.8125	-0.29956	0.8159792	12	2	18	7	11	8
AC134312.4	0.5	-1	0.7145709	4	0	0	0	2	0
JMJD1C-AS1	0.7777778	-0.36257	0.7558707	1	12	5	0	8	6
RBMY1HP	1	0	0.9400153	0	2	4	2	0	4
AL121820.2	1.1807229	0.2396704	0.5513762	17	38	28	40	35	23
AL121820.1	1.3571429	0.4405726	0.7140145	2	2	10	2	17	0
CDK9	0.8978671	-0.155426	0.5086886	1494	1598	1784	1494	1624	1260
CDK8	0.9960403	-0.005724	0.7885186	938	821	1019	695	1049	1023
AC006023.2	0	#NUM!	0.6840298	0	0	2	0	0	0
CDK7	1.1558541	0.2089593	0.0350432	2603	2067	3145	2829	3363	2841
AC006023.1	#DIV/0!	#DIV/0!	0.8030358	0	0	0	0	0	1
CDK6	0.9068687	-0.141034	0.5752429	1469	1058	1564	790	1492	1428
CDK5	1.0461165	0.0650435	0.4829869	200	254	370	304	281	277
CDK4	0.8453597	-0.242363	0.1704119	2511	3128	3369	2479	2731	2405
CDK3	1.125	0.169925	0.8494094	1	3	4	4	0	5
CDK2	1.0627741	0.087835	0.1932604	2491	2499	2991	2667	3089	2726
CDK1	1.3837458	0.468579	5.18E-05	2984	2254	3166	3264	4421	3944
TCAM1P	2.2666667	1.1805722	0.1575111	4	6	5	13	12	9
WRAP53	0.9422794	-0.085773	0.8980477	730	1016	974	913	871	779
AC073370.1	0.25	-2	0.3955922	1	3	4	2	0	0
AC009299.2	1.296875	0.3750394	0.4002691	15	25	24	28	30	25
AC009299.1	0.6	-0.736966	0.7824522	0	3	2	2	1	0
GPX2	1	0	0.9490467	9	16	16	10	18	13
RFX3-AS1	0.3333333	-1.584963	0.3029446	14	0	4	0	3	3
GPX1	0.8284901	-0.271444	0.1759305	6708	8471	9047	7307	6833	5931
GPX4	1.0646475	0.0903759	0.3069088	3631	4688	5185	5403	4956	4018
GPX3	1.1493289	0.2007917	0.1765557	306	430	456	497	498	375
GPX6	0	#NUM!	0.5781912	0	0	3	0	0	0
GPX5	1.1666667	0.2223924	0.963458	6	0	0	0	7	0
LIX1L	1.164557	0.2197812	0.5682138	38	13	28	44	20	28
GPX8	1.1374869	0.18585	0.0661351	1981	1656	2109	1755	2401	2380
HK2P1	0	#NUM!	0.6410557	1	1	0	0	0	0
YDJC	0.8532532	-0.228954	0.4104484	767	935	1126	934	791	688
AC009299.3	1.3636364	0.447459	0.6243839	10	4	8	3	3	24
WRAP73	1.2728304	0.3480402	0.0075389	522	558	683	790	756	698
AC093591.3	1.5	0.5849625	0.7023348	2	0	2	4	1	1
AC004691.1	0.4285714	-1.222392	0.6201542	3	0	4	0	0	3
AC093591.2	1	0	0.9398037	0	0	3	1	1	1
CDNF	2.0377358	1.026967	0.0293288	15	19	19	31	42	35
AC009309.1	0.8	-0.321928	0.8351609	15	6	9	13	0	11
AC087473.1	2	1	0.4957532	1	2	2	3	5	2
FOLR3	0	#NUM!	0.2904576	0	1	5	0	0	0
CDO1	1.1657143	0.2212142	0.2851093	111	82	157	125	171	112
FOLR2	0.4	-1.321928	0.7347189	0	0	5	2	0	0
FOLR1	1.0386096	0.0546535	0.3733393	4749	6015	6641	6065	6428	5584
PJVK	0.56	-0.836501	0.4321357	31	0	19	2	8	18
AC096637.2	0	#NUM!	0.4521054	1	0	3	0	0	0
AC096637.1	0	#NUM!	0.4185478	3	0	1	0	0	0
SMARCE1P5	0.5714286	-0.807355	0.573633	2	12	0	0	7	1
SMARCE1P6	0	#NUM!	0.5378194	0	2	1	0	0	0

SMARCE1P1	#DIV/0!	#DIV/0!	0.8030358	0	0	0	0	0	1
SMARCE1P3	0	#NUM!	0.824326	0	0	1	0	0	0
SMARCE1P2	#DIV/0!	#DIV/0!	0.8030358	0	0	0	0	0	1
CDON	0.5943775	-0.750549	0.0437078	74	104	71	38	48	62
MRPS18B	0.9071196	-0.140635	0.5234586	2295	2692	2991	2325	2621	2291
MRPS18A	0.8462389	-0.240863	0.2106465	530	585	693	443	562	525
MRPS18C	1.4615385	0.5474878	0.0491602	46	58	78	91	84	91
CDS1	1.0984582	0.13548	0.172591	1285	1068	1344	1091	1487	1483
TWF2	0.8391557	-0.252989	0.2915746	361	511	502	368	376	409
TWF1	0.960255	-0.058511	0.9473586	2995	1911	3095	1907	3179	2597
OLMALINC	1.2588235	0.3320761	0.1137718	117	84	139	148	143	137
AL671986.1	0.6666667	-0.584963	0.856923	1	0	2	0	2	0
AC009152.1	5	2.3219281	0.1222223	1	0	2	7	2	6
CDR2	0.9934354	-0.009502	0.7079473	829	841	1072	786	997	941
CDT1	0.9155701	-0.127258	0.7244643	3768	4956	5335	4733	4449	3690
FAM66A	9	3.169925	0.1597598	1	0	0	1	4	4
GADD45B	1.5263158	0.6100535	0.0101794	93	74	118	162	137	136
GADD45A	0.8507211	-0.233242	0.1748887	2006	1953	2559	1809	1947	1789
GADD45G	1.3046743	0.3836896	0.0064002	1085	1335	1709	2023	1872	1492
FAM66E	0	#NUM!	0.824326	0	1	0	0	0	0
FAM66D	1	0	0.9756864	2	0	0	0	2	0
FAM66C	2.2	1.1375035	0.475924	0	0	5	1	5	5
FAM66B	0.5405405	-0.887525	0.3894261	9	8	20	11	4	5
AC139493.2	2	1	0.814195	0	1	0	0	0	2
CDS2	1.0091989	0.0132106	0.6577708	1523	1769	1926	1373	1938	1955
FAM69C	6	2.5849625	0.0110969	1	4	2	11	6	25
FAM69B	1.1416667	0.1911415	0.3650948	66	67	107	99	93	82
FAM69A	0.9377014	-0.0928	0.8616442	332	222	377	223	325	325
VLDLR	1.2542583	0.3268344	0.000941	3498	3164	3847	3634	4829	4718
RNU6-945P	0.36	-1.473931	0.1927305	10	9	6	1	7	1
CDV3	0.973357	-0.038959	0.9029131	9687	8692	10972	7962	10639	9968
MIR25	0.9264706	-0.110183	0.8779253	50	33	53	23	43	60
VN1R110P	2.5	1.3219281	0.5912358	0	1	1	0	3	2
MGAT3-AS1	1.173913	0.2313255	0.741234	7	12	4	18	5	4
AC005186.1	0.3333333	-1.584963	0.3913005	1	6	2	0	1	2
Z83838.1	0	#NUM!	0.6436235	2	0	0	0	0	0
CTNNA1	0.8769605	-0.189416	0.1875194	28640	30342	35126	23771	29928	28830
EBLN2	1.1827957	0.2422009	0.5453008	32	16	45	20	47	43
EBLN1	3	1.5849625	0.3796978	2	0	1	2	5	2
CTNNA3	0.75	-0.415037	0.8862687	2	1	13	8	2	2
OR6E1P	#DIV/0!	#DIV/0!	0.2905913	0	0	0	2	0	3
CTNNA2	0	#NUM!	0.824326	0	0	1	0	0	0
CDX2	0.6887417	-0.537965	0.1926818	75	108	119	82	89	37
CDX1	2.5	1.3219281	0.3848764	1	2	1	5	3	2
AC002546.2	11	3.4594316	0.1042248	1	0	0	2	7	2
AC002546.1	1.4324324	0.5184671	0.2595065	29	22	23	29	51	26
POU6F2-AS2	0.5689655	-0.813587	0.2483735	14	24	20	11	10	12
AL356776.1	0.5	-1	0.7778439	0	0	4	0	2	0
CTNNB1	1.1497086	0.2012683	0.0227635	27298	27341	31328	26731	36516	35590
chromatid coh	#DIV/0!	#DIV/0!							
egulation of he	#VALUE!	#VALUE!	4|nucleoplas	05165|hsa05	papillomav	tenin beta 1			
RN7SL851P	0.5	-1	0.7988788	1	1	0	0	0	1
ATP6V0CP2	0	#NUM!	0.4460243	4	0	0	0	0	0
AC072052.1	3.5	1.8073549	0.1628073	2	0	4	2	6	13

AC002400.1	1	0	0.9905468	2	0	1	0	0	3
SLX1B	0.5	-1	0.812391	0	2	0	0	1	0
APLP2	0.8246697	-0.278112	0.0331842	30263	32456	36431	22909	29795	29062
APLP1	0.5427683	-0.881592	0.0002547	178	218	247	103	133	113
CDYL	0.9686122	-0.046009	0.9983164	1752	1849	2070	1464	2091	1938
DHX9P1	1.0714286	0.0995357	0.9583275	6	5	3	0	7	8
FOLH1	3.7083333	1.8907709	0.0019754	4	7	13	30	36	23
SLX1A	#DIV/0!	#DIV/0!	0.6554062	0	0	0	0	2	0
AC090952.1	#DIV/0!	#DIV/0!	0.8030358	0	0	0	0	1	0
MIR32	0	#NUM!	0.6840298	0	0	2	0	0	0
ZFP36L2	0.9957761	-0.006107	0.7815326	255	327	365	278	350	315
ZFP36L1	1.0279241	0.0397338	0.3757827	9265	9396	11134	8654	11299	10674
AL080276.2	10	3.3219281	0.133309	1	0	0	1	6	3
AL080276.1	6	2.5849625	0.3849176	0	1	0	0	0	6
EP400	0.807356	-0.308723	0.0745844	3163	3385	3403	1902	2857	3275
RNU6-943P	0	#NUM!	0.4460243	4	0	0	0	0	0
RNU6-942P	0	#NUM!	0.824326	1	0	0	0	0	0
MIR98	0.75	-0.415037	0.9161536	0	0	4	0	3	0
PKD2	0.8238866	-0.279482	0.1996471	550	411	521	289	464	468
PKD1	0.7163022	-0.48136	0.0030252	1072	1254	1287	742	857	989
CKS1B	0.8990756	-0.153486	0.478009	3829	4359	4901	3943	4209	3616
MIR93	5	2.3219281	0.4322862	1	0	0	0	3	2
AL512380.2	#DIV/0!	#DIV/0!	0.4737294	0	0	0	0	4	0
TWNK	0.9489226	-0.075638	0.7994396	787	893	826	588	892	898
SSR4P1	0.8676471	-0.20482	0.8301323	17	21	30	23	21	15
ZNF197	5.4285714	2.4405726	0.0221249	0	5	2	8	16	14
TM2D2	1.0349827	0.0496066	0.4032766	929	1029	1215	1028	1208	1048
ZNF195	1.0645395	0.0902295	0.4462792	1044	568	1146	795	1225	916
TM2D1	0.9835355	-0.023951	0.8158903	377	330	447	343	364	428
TM2D3	0.9808887	-0.027839	0.8059531	617	635	841	641	783	629
AL513124.1	#DIV/0!	#DIV/0!	0.6554062	0	0	0	0	2	0
LEKR1	1.2173913	0.283793	0.6520004	7	6	10	13	7	8
ZNF189	1.1430096	0.1928375	0.1910223	336	282	319	295	424	352
AL035401.1	1.875	0.9068906	0.5257244	3	5	0	3	7	5
ZNF185	0.6137223	-0.704342	7.05E-05	567	619	665	288	411	437
ZNF184	1.185	0.2448871	0.283242	131	78	191	106	189	179
ZNF182	1.1509434	0.2028169	0.3117498	149	76	199	167	180	141
ZNF181	0.8536585	-0.228269	0.6356998	76	52	118	44	102	64
ZNF180	0.8440367	-0.244622	0.325221	263	218	282	174	236	234
PIK3IP1	0.6206262	-0.688204	0.0113621	150	190	203	73	136	128
AC008741.2	0.5654952	-0.822413	0.0502818	148	49	116	65	65	47
KRT16P1	#DIV/0!	#DIV/0!	0.8030358	0	0	0	0	0	1
LURAP1L	1.4642857	0.5501971	0.452142	16	6	6	14	8	19
RNU1-134P	0.4285714	-1.222392	0.6304391	0	2	5	0	0	3
OR8Q1P	1.875	0.9068906	0.4749948	2	0	6	3	9	3
FAM19A2	0.7682927	-0.380272	0.5079504	24	34	24	12	23	28
FAM19A4	0.96	-0.058894	0.9962256	3	11	11	6	7	11
MARK2P11	#DIV/0!	#DIV/0!	0.6099294	0	0	0	2	0	0
FAM19A3	0.8	-0.321928	0.8757968	1	2	2	0	2	2
GSTK1	0.9275984	-0.108428	0.835485	1160	1204	1600	1324	1351	1002
AC025752.1	2.3333333	1.2223924	0.6677692	0	3	0	0	7	0
AL161457.2	0.5714286	-0.807355	0.6660987	4	3	0	0	1	3
AL161457.1	0	#NUM!	0.824326	1	0	0	0	0	0
AL136115.2	1.2	0.2630344	0.8098846	1	0	9	0	2	10

AL136115.1	#DIV/0!	#DIV/0!	0.8030358	0	0	0	0	1	0
Z95152.1	2.75	1.4594316	0.2933062	4	0	4	0	8	14
ORAI3	1	0	0.9839161	3	4	2	0	4	5
ORAI2	0.6083333	-0.717066	0.0735569	63	58	119	44	53	49
AP001324.1	1.2323944	0.301464	0.1999203	72	93	119	148	108	94
ORAI1	0.9072883	-0.140367	0.6976289	531	617	718	623	573	497
RNU6-520P	0	#NUM!	0.6840298	0	0	2	0	0	0
AP003721.1	1.2857143	0.3625701	0.7620273	2	1	4	3	0	6
SHMT2	1.0708288	0.0987278	0.2321595	3134	3828	3994	3964	4139	3629
AP003721.3	3.5	1.8073549	0.2066617	1	2	1	9	1	4
SHMT1	0.8045882	-0.313677	0.0282049	1896	1899	2264	1517	1740	1618
B3GLCT	1.2283105	0.2966753	0.1273896	140	113	185	140	212	186
AP003721.4	3.875	1.9541963	0.0534367	3	3	2	10	14	7
AL035563.1	1.3424658	0.4248853	0.3147443	25	13	35	30	38	30
RDH12	10	3.3219281	0.1479262	0	0	1	1	8	1
RDH11	1.0547009	0.0768339	0.2724858	1220	1142	1733	1313	1571	1435
RDH10	1.073904	0.102865	0.2616704	763	752	880	703	973	896
RDH16	2.3043478	1.2043585	0.1026555	11	2	10	13	24	16
RDH14	1.01853	0.0264884	0.5985073	555	452	612	485	643	521
RDH13	0.7357689	-0.442675	0.0138781	714	691	949	585	591	556
GSTM4	0.9821138	-0.026038	0.8182718	144	203	268	219	214	171
GSTM3	0.8975	-0.156016	0.6698739	117	155	128	109	122	128
GSTM2	0.7959184	-0.329308	0.5607866	35	29	34	18	23	37
GSTM1	0	#NUM!	0.824326	0	1	0	0	0	0
AC092447.2	0.4285714	-1.222392	0.6199002	1	0	6	0	1	2
AC092447.5	0.6666667	-0.584963	0.8119618	3	0	6	2	0	4
AC092447.7	2	1	0.4657154	4	0	2	6	3	3
AC137767.1	0.8227848	-0.281413	0.6299685	21	36	22	15	26	24
AC109809.1	#DIV/0!	#DIV/0!	0.8030358	0	0	0	0	0	1
CYP2G1P	0	#NUM!	0.6604275	0	1	1	0	0	0
PKIB	1.1581889	0.2118706	0.0229912	1750	1590	2027	1835	2362	2019
PKIA	1.0747664	0.1040231	0.5132714	206	189	247	149	279	262
PKIG	0.7150259	-0.483933	0.2677546	44	99	50	41	50	47
GSTM5	1.5	0.5849625	0.7734914	0	0	2	3	0	0
GTF2H2C	0.9117548	-0.133282	0.5850716	1078	788	1103	741	1017	949
RNU1-138P	0	#NUM!	0.1172014	0	7	2	0	0	0
GSTO2	0.7592191	-0.397412	0.1438963	154	139	168	103	129	118
GSTO1	0.7903226	-0.339486	0.5375812	36	29	59	24	39	35
AL590764.1	#DIV/0!	#DIV/0!	0.8030358	0	0	0	0	1	0
BSNDP2	0	#NUM!	0.824326	1	0	0	0	0	0
GTF2H2B	1.0303867	0.0431859	0.6159725	388	319	379	303	368	448
FABP5P11	1.75	0.8073549	0.6802753	1	2	1	0	3	4
PKN3	1.1761186	0.2340335	0.0256629	994	1129	1386	1344	1468	1315
GSTP1	0.8417995	-0.248451	0.2547933	8588	10615	11606	9702	8852	7381
SYNPO2	0.9864865	-0.019629	0.937744	35	11	28	26	33	14
ADAM1B	0.3703704	-1.432959	0.207071	12	3	12	1	4	5
ADAM22	1.5862884	0.6656551	0.0020307	124	113	186	173	220	278
ADAM21	1.125	0.169925	0.7012539	24	22	26	16	39	26
AC023355.1	0.8019802	-0.318361	0.6090228	34	21	46	24	34	23
ADAM20	2	1	0.807847	1	0	0	0	1	1
SDAD1	1.1821884	0.24146	0.05684	1350	891	1442	1189	1621	1544
ADAM29	0	#NUM!	0.1626159	2	2	3	0	0	0
AC073529.1	1.1666667	0.2223924	0.7461847	18	11	19	3	27	26
ADAM28	0.9141104	-0.12956	0.8636188	41	43	79	25	56	68

ADAM23	1.0909091	0.1255309	0.8207146	1	2	8	6	1	5
AF064858.3	3.75	1.9068906	0.1074804	2	6	0	8	4	18
AC243732.1	0.875	-0.192645	0.9775499	0	0	8	0	4	3
AF064858.2	0.8762887	-0.190522	0.803536	30	24	43	19	23	43
AF064858.1	0.25	-2	0.1880167	6	4	6	0	1	3
AC115837.1	1	0	0.9983591	1	3	2	1	4	1
AC092687.3	0.5182927	-0.948161	0.0363845	53	41	70	31	27	27
AC092687.2	0	#NUM!	0.5200113	2	1	0	0	0	0
PKLR	#DIV/0!	#DIV/0!	0.6411933	0	0	0	0	0	2
ADAM33	0	#NUM!	0.824326	0	0	1	0	0	0
KIAA2012	0.5	-1	0.8652929	0	0	2	0	1	0
ADAM32	1.1851852	0.2451125	0.7396741	3	1	23	5	24	3
KIAA2013	1.0796443	0.1105561	0.2991191	1489	2040	1757	1965	1890	1852
[truncated: 454,887 more chars]
